# Universality of the DNA methylation codes in Eucaryotes

**DOI:** 10.1038/s41598-018-37407-8

**Published:** 2019-01-17

**Authors:** Benoît Aliaga, Ingo Bulla, Gabriel Mouahid, David Duval, Christoph Grunau

**Affiliations:** 1 University Perpignan Via Domitia, IHPE UMR 5244, CNRS, IFREMER, University Montpellier, F-66860 Perpignan, France; 2 Institute for Mathematics and Informatics, University of Greifswald, Greifswald, Germany; 3 Department of Computer Science, ETH Zürich, Zürich, Switzerland

## Abstract

Genetics and epigenetics are tightly linked heritable information classes. Question arises if epigenetics provides just a set of environment dependent instructions, or whether it is integral part of an inheritance system. We argued that in the latter case the epigenetic code should share the universality quality of the genetic code. We focused on DNA methylation. Since availability of DNA methylation data is biased towards model organisms we developed a method that uses kernel density estimations of CpG observed/expected ratios to infer DNA methylation types in any genome. We show here that our method allows for robust prediction of mosaic and full gene body methylation with a PPV of 1 and 0.87, respectively. We used this prediction to complement experimental data, and applied hierarchical clustering to identify methylation types in ~150 eucaryotic species covering different body plans, reproduction types and living conditions. Our analysis indicates that there are only four gene body methylation types. These types do not follow phylogeny (i.e. phylogenetically distant clades can have identical methylation types) but they are consistent within clades. We conclude that the gene body DNA methylation codes have universality similar to the universality of the genetic code and should consequently be considered as part of the inheritance system.

## Introduction

Living organisms are biological systems in which the complex interaction between different elements such as the nuclear genotype and epigenotype factors and the environment brings about a phenotype that develops and evolves over time^1,2^. For a complete understanding and potential control of biological processes such as development and evolution, it is therefore necessary to understand as many elements of biological systems as possible. In the present work, we focus on the epigenotype unit that we operationally define as any modification of the chromatin-DNA complex that has an impact on the expression and function of genes^3^. Epigenetic information can be stored in a multitude of bearers such as histone modifications, non-coding RNA, the topology of the nucleus, and methylation of DNA. DNA methylation has been one of the most studied epigenetic marks since its discovery in 1948^4^. Methylation occurs at positions 4 and 5 of the pyrimidine ring of cytosine forming either 4-methyl-cytosine (4mC) or 5-methyl-cytosine (5mC), or at position 6 of the purine ring in 6-methyl-adenine (6mA). 6mA and 4mC were believed to occur only in bacteria but recent advances in sequencing technology made it possible to detect them also in eukaryotic species. A specific database (MethSMRT) was dedicated to these modifications^5^, and the available experimental data were used to train an algorithm to predict the occurrence of 4mC^6^ in DNA based on sequence features. We will focus here on 5mC and to facilitate the readability use the term *DNA methylation* for this purpose.

In most eukaryotes, 5mC is overrepresented or even restricted to the dinucleotide CpG context, where ‘p’ stands for the phosphodiester linkage between the cytosine (C) and the guanine (G). In plants, the 5mC can occur in other contexts such as CpHpG or CpHpH, where ‘H’ stands for A, C or T (reviewed in Vanyushin^7^). In contrast, in certain molds, methylation occurs preferentially (>60%) in CpAs^8^. DNA methylation is catalyzed by a family of enzymes called DNA methyltransferase (DNMT) composed of 3 canonicals members (DNMT 1, 2 and 3)^9^. After replication, 5mC will be maintained by the activity of DNMT1, which has a high affinity to hemi-methylated DNA, and that methylates immediately after replication the newly synthesized strand, reproducing methylation patterns in CpG dinucleotide with a fidelity of roughly 99.9%^10^ thus allowing for mitotic heritability of DNA methylation patterns. The role of DNMT2 is controversial because it has little DNA methylation activity^11^ but is able to methylate cytosine 38 in the anticodon loop of aspartic acid transfer RNA^12^ and some authors propose therefore to replace DNMT2 by tRNA (Cytosine(38)-C(5))-Methyltransferase TRDMT1^13^. There are species, such as the model organism *Drosophila melanogaster*, that have only DNMT2 and do not possess 5-methyl-cytosine in their genome, or DNA methylations is so low that it is very difficult to detect^11,14,15^. These enzymes have distinct roles due to the presence of different domain structures. DNA methylation is established by DNMT3 that can methylate the two strands of the DNA *i*.*e*. has a *de novo* methylation function^16^. Various DNA methylation contexts are found across the plant and animal kingdoms. There are species where 5mC is present all over the genome (global methylation) while others can be entirely devoid of methylation. In species with global DNA methylation, only small regions, among them promoters and other regulatory elements, are methylation-free^17^. If 5mC occurs in the promoters of vertebrates, it has a repressive action on the gene transcription^18^. Invertebrates often have a mosaic-type methylation pattern with high methylation in almost all CpG in large blocks of genomic DNA interspersed with almost entirely unmethylated blocks. Changes in DNA methylation occur during organ regeneration^19^, aging^20,21^, in response to bacterial infection^22^, as well as flowering time and root length in *Arabidopsis thaliana*^23^, just to name a few examples. DNA methylation was therefore proposed as a language in which environment and genome talk to each other. Other authors have seen DNA methylation primarily as a genomic defense system against parasitic genomic elements^17^.

Given the apparent heterogeneity of DNA methylation patterns and the multitude of biological processes involved it was suggested that DNA methylation evolved in every phylogenetic clade towards a specific role in controlling gene expression. Essentially, the question is whether or not there is universality in the DNA methylation epigenetic code, conceptually similar to the universality of the genetic code, or if DNA methylation is non-universal and specific to every evolutionary unit. We wished to address this question through the analysis of the evolution of DNA methylation. Here, an obstacle is that a comprehensive analysis of DNA methylation patterns in a wide range of different species is missing. There are currently methods available that allow, in principle, for determining genome-wide DNA methylation patterns (“methylomes”) at a single base resolution. Since DNA methylation patterns can be different in different organisms of a species or even tissues of an individual, for a given species several methylomes can exist. A review of the available data in different databases and in the literature showed that there is a strong bias towards model organisms: there are at least 300 methylomes available for human, mouse and the model plant *A*. *thaliana*, but only 63 for a total of 16 other species (http://smithlabresearch.org/software/methbase/ and Céline Cosseau, pers. communication). As a consequence, global conclusions about the function and importance of DNA methylation are actually based on a very limited and biased amount of data. For this reason, it remains challenging to derive the general rules (if any) that govern DNA methylation in the different branches of the “tree of life”. A potential solution to the caveat that experimental “wet bench” data is missing is to infer DNA methylation indirectly with computational method^24,25^. The basis for this is that methylated CpG sites mutate relatively frequently compared to the other dinucleotides over evolutionary time^26^. If a cytosine is deamined, a deoxy-uracil will form, which is not stable in DNA: it will rapidly be excised by uracil glycosylase and replaced by cytosine. In contrast, 5mC deamination generates thymine, which is less efficiently processed by the DNA repair machinery. Despite the existence of a specific repair mechanism that restores G/C mismatch, the mutation rate from 5mC to T is therefore 10-fold to 50-fold higher than other transitions, depending on local GC content^27^. For Humans, it was estimated that within 20 years, 0.17% of 5mC in the genome were converted into thymine^28^. If 5mC occurs predominantly in CpG pairs, the above-mentioned mechanism will increase the mutation rate from CpG to TpG or CpA and induce an underrepresentation of CpG^29^. Therefore, CpG observed/expected ratio (CpG o/e) in gene bodies can be used to predict if a species’ DNA is methylated in gene bodies or not^26,30,31^. In other words, gene bodies of a species are not methylated when the CpG o/e ratio is in average close to 1, and methylated for an average ratio far below 1. Low CpG o/e is not a condition for methylation but a consequence of it. It is important to note that this is a species-level prediction that uses methylation signatures that pass through the germ-line and need several generations of mutation accumulation to be detectable. It cannot be used to predict methylation changes of individual genes within shorter periods.

These predictions were tested in at least 13 studies comparing CpGo/e to methylation levels obtained with various methods (Table 1 and Supplementary File 1). All came to the conclusion that CpGo/e ratios correlate well (inversely) with methylation levels when species were compared. Nevertheless, there remain technical challenges. For instant, in the past, prediction of *in silico* DNA methylation based on Gaussian distributions, that are relatively straightforward to implement, were used to describe the frequency distribution of CpGo/e ratios^32–35^. But in many species, frequency distributions of CpGo/e ratios are complex or skewed and Gaussian distribution is not suitable. In our hands, only for 58 out of 83 cases (65%) Gaussian mixtures allowed for description of the distribution^36^. These values are comparable to what was found by Bewick and colleagues who used CpG o/e ratios in transcriptomes of 124 species of which only 50 (40.32%) were described correctly with Gaussian mixtures^37^. We have also tested non-Gaussian distributions and the results were even less conclusive than Gaussian distributions: out of 83 only 41% delivered an exploitable result. Therefore, we have developed a new tool, called Notos, to identify DNA methylation signatures within CpGo/e ratios based on kernel density estimations^36^. This novel algorithm delivers robust descriptions of frequency distributions of CpGo/e ratios for up to 172,000 input sequences.

**Table 1:** List of publications in which the authors investigated DNA methylation by a wet bench method and compared the results to CpGo/e ratios.

Species	Formula	Sequences	Validation	References
*Acropora millepora*	Unknown	CDS	MBD-eq	^57^
*Apis melifera*	Unknown	CDS	BS-seq	^78^
*Biomphalaria glabrata*	Matsuo^72^	RNAseq	Restriction enzyme, BS-seq (Nimbus retrotransposon), LC-MS	^79^
*Crassostrea gigas*	Matsuo^72^	EST	Methylation sensitive PCR, BS-Seq	^32^
*Solenopsis invicta*	Unknown	Genome	MeDIP, BS-Seq (9 genes)	^80^
	Gardiner-Garden and Frommer^52^	Promoteur and Genes	BS-Seq	^81^
*Nasonia vitripennis*	Matsuo^72^	Refseq	Cloning and sequencing 18 genes at selected CpG sites, BS-seq	^33^
	Unknown	Genome and coding sequences	Whole genome bisulfite sequencing	^82^
*Locusta migratoria*	Unknown	cDNA, Unigene	Methylation-specific restriction enzyme assays	^83^
*Acyrthosiphon pisum*	Unknown	CDS and predicted genes	MeDIP, BS-seq, restriction enzyme	^35^
*Bombyx mori*	Unknown	Genes	MethylC-seq	^84^
*Nicrophorus vespilloides*	Unknown	Genes	Whole genome bisulfite sequencing	^85^
*Ciona intestinalis*	Unknown	Genes	BS-Seq	^86^
	Unknown	EST	BS-Seq, Methylation-sensitive PCR	^31^
*Arabidopsis thaliana*	Gardiner-Garden and Frommer^52^	CDS	BS-seq	^87^

Here, we have applied this software to predict DNA methylation with CpGo/e ratios in a total of 634 species and to use the results in combination with publicly available experimental data to infer evolution of DNA methylation over the eukaryotic tree of life. We applied Notos on coding sequences coming from three databases (dbEST, CleanEST, and CDS/cDNA). Our results show clearly (i) that DNA methylation prediction by CpGo/e ratio is robust, (ii) that only four types of DNA methylation can be identified in all species despite their wide range of genome sizes, environments, body plans, reproduction types etc., and (iii) that DNA methylation types does not follow phylogeny but is consistent within clades suggesting evolutionary constraints. Taken together our analysis delivers arguments for the idea of the universality of the role of DNA methylation that is preserved through evolution.

## Results

### CDS and cDNAs are the less biased data and thus the best choice for a pan-species study

We focused in this study on gene body DNA methylation. Annotated genomes are now available for many species, but messenger RNA sequence data is even more abundant and mRNA is representative of gene body DNA sequences. They could therefore be used instead of DNA sequence, but mRNA data is for historical reasons stored in different forms and in different databases. We reasoned that data quality will be critical for providing unbiased estimation of DNA methylation in gene bodies and therefore conducted a comparative pilot study to identify the best possible data source for the subsequent pan-species study. We used coding/transcript sequences from full genome annotations (CDS), dbEST, and cleanEST (details in Supplementary File 2). A total of 127 species are in common between CDS and dbEST, and 92 species were in common between dbEST and cleanEST. Only 29 species were common to all three databases (Supplementary File 3). We produced Notos CpGo/e profiles for all intersecting datasets and proceeded to visual inspection. In 11 out of 29 cases (38%) we identified discrepancies in at least one out of the three profiles and decided to clarify their origins by a detailed analysis of the sequences under the differential peaks. An in-depth analysis revealed that these discrepancies were either due to contaminations during the sequencing process, reflect co-occurrence of other species, or are due to bias in data acquisition. For instance, for *Trichoplax adhaerens*, *Anolis carolinensis* (green anole lizard) and *Cordyceps militaris* one or two additional shoulder peaks in dbEST and CleanEST datasets. We isolated the sequences contained in these peaks (dbEST and Clean EST) and performed a Blast2GO analysis with the aim to know their origins and functions. For the anole lizard (Supplementary Fig. 1), two peaks were isolated (peak 1: 0.92–1.08 and peak 2: 1.14–1.22), representing 7,030 and 4,922 sequences, respectively. The majority of sequences under peak 1 in the dbEST profile correspond to chloramphenicol O-acetytransferase used in bacterial cloning vectors. It is therefore likely that these sequences represent contaminations from the EST library generation procedure. Sequences under peak 2 present homologies with sequences from apicomplexans (plasmodium), and platyhelminths suggesting presence of such parasites in the initial biological sample. For *T*. *adhaerens*, a peak was isolated (1.22–1.35), which represents 1,609 ESTs. Most of the sequences under the dbEST peak were identified by Blast2GO as ‘other’. Since *T*. *adherens* is known to contain intracellular bacteria^38^ we believe that these sequences originate from them (Supplementary Fig. 2). For the mold *C*. *militaris*, two peaks were isolated (1.14–1.22, and 1.26–1.32). For the sequences under these peaks, homologies with other fungi sequences were found. We conclude that, in all three species, the additional modes occurred due to presence of sequences from other species, either through contamination during RNA extraction and library preparation, or as co-purification from naturally occurring symbionts or parasites. In other species, we identified other sources of bias in the transcript data. For instance, in *Bombyx mori* an ovarian library cleanEST showed an additional weak shoulder peak. We isolated the 769 sequences under this peak (0.40–0.60). The gene ontology showed that all sequences coded for the ribosomal protein SA (RPSA). In human, RPSA genes are indeed highly expressed in the ovary but no data are available for other species. Nevertheless, it seems unlikely that the high abundance of RPSA ESTs reflects an expression bias. We speculate that the research interest of the submitters focused on this particular gene and that therefore many individual EST were submitted (Supplementary Fig. 3). Also in the duck (*Anas platyrhynchos*), we found a shoulder peak at 0.57 and 0.59 in data from dbEST and Clean EST. Sequences under this peak corresponded to an EST library exclusively composed of immunoglobulins (146 sequences), reflecting probably a bias introduced by specific research interests (Supplementary Fig. 4). Interestingly, these sequences had a CpG*o/e* ratio between 0.5 and 0.8 suggesting hypomethylation, and in human, immunoglobulin genes in lymphoid cells are indeed undermethylated during differentiation^39^.

Finally, when we compared profiles with two peaks (bimodality), where we had noticed differences between CDS (derived from genomes) and dbEST/CleanEST (mRNA) for three invertebrate and one plant species (*Crassostrea gigas*, *Nasonia vitripennis*, *Nematostella vectensis* and *Oryza sativa*) (Supplementary Figs 5–8): mRNA derived profiles showed a higher peak in genes predicted to be methylated. Gene body methylation is suspected to increase transcription^40,41^. The principal differences between CDS and EST data is that for the former only one FASTA sequence per gene is considered while for the later potentially several FASTA sequences for a gene could be present. We therefore hypothesized that RNA abundance induced the bias in EST data. To test this hypothesis, we performed a RNA-seq analysis in these four species. We found that genes under the low CpGo/e peak (presumably hypermethylated) show higher median RNA amounts than genes under the high CpGo/e peak (this presumably hypomethylated). mRNA FPKM medians are 1.95 to 5.45 higher in presumably hypermethylated gene bodies (Supplementary Figs 5–8). We conclude that this expression difference is probably the origin of the bias in EST datasets.

In summary, dbEST and cleanEST have the advantage of being large repositories with data for many species, but for the purpose of our study we considered them too noisy. A complete list of species for each dataset is in Supplementary File 4.

### CpGo/e clustering identifies four types of gene body DNA methylation

After having firmly established that cDNA provides an unbiased data basis, CpG o/e clustering was carried out on the 142 species for which CDS or cDNA were available. Parameters for mode number (n), mode positions (Mo), skewness (sk), and standard deviation (SD) of CpG*o/e* values were iteratively changed using species with known gene body DNA methylation. For further analysis, we used the following features that produced four clusters of CpG*o/e*: (cluster 1) species with one mode Mo ≥ 0.69 and SD an0.12, (cluster 2) species with one mode Mo ≥ 0.69 and SD ≥ 0.12, (cluster 3) species with one mode Mo  s0.69, and (cluster 4) species with (a) two modes or (b) one mode and a skewness smaller than −0.04. Results are in Supplementary File 5 and Supplementary Fig. 9. We then associated the four clusters with known methylation types.

Fourteen species (9.72%), from different phylogenetic groups (*e*.*g*. Ascomycota, Apicomplexa, Basidiomycota, Plathyhelminthes and Arthropoda) constitute the cluster 1. All the species have a CpG*o/e* mode position mode above 0.69 (the mean CpG*o/e* peak position is 1.00), a weak negative skewness (mean_absolute Q50 skewness_ = −0.0019) and a narrow standard deviation (mean_SD_ = Da0.11). For 4 species (29%), experimental data on DNA methylation was available in the literature. All these species showed either absence of DNA methylation in the gene bodies or extremely low levels (Supplementary File 5). The latter was found in only one species (*Chlamydomonas reinhardtii*) where WGBS revealed methylation in exons but still it was 20–30 times weaker compared to other plant species in the same study. We qualify cluster 1 as “ultra-low gene body methylation” (type 1).

It could be argued that absence of gene body methylation is simply a consequence of absence of enzymatic methylation activity. We therefore performed a metanalysis of existing literature data concerning DNMT presence. In cluster 1, 6 out of 14 species have DNMT2 or TRDMT1, and one specie has DNMT1 and DNMT2. Only the *de-novo* methylase DNMT3 is absent in this cluster. Absence of methylation does therefore not indicate necessarily absence of DNMT genes (Supplementary File 5).

Cluster 2 is constituted by 60 species (41.67%), also from different phylogenetic groups (apicomplexa, oobionta, rhodonbionta, ascomycota, basidiomycota, nematoda, platyhelminthes, annelida, arthropoda, ctenophora, chordata, embryophyta). As in the cluster 1, species present in the second cluster have a mode position > 0.69 with a mean mode position very close to the first cluster (mean CpGo/e position is 0.92) and a mean absolute Q50 skewness of 0.0012. However, in contrast to cluster 1, a wide standard deviation (mean_SD_ = Da0.18) has been observed. Literature data were available for 18 species (30%). For 6 species (*Schizosaccharomyces pombe*, *Aspergillus flavus*, *Brugia malayi*, *Meloidogyne incognita*, *Tribolium castaneum*, *Drosophila melanogaster*) no methylation was reported. Methylation in 6 species (*Schistosoma mansoni*, *Schistosoma japonicum*, *Fasciola hepatica*, *Petromyzon marinus*, *Caenorhabditis elegans* and *Saccharomyces cerevisiae*) is controversial since different authors come to different conclusions. Nevertheless, methylation seems to be very low. Only three species (*Trichinella spiralis*, *Solenopsis invicta*, *Physcomitrella patens*) showed DNA methylation in gene bodies. DNMTs were studied in 37 species. In this cluster, 16 species have just DNMT2. One species has DNMT1 or TRDMT1 only, and 6 species have all the DNMT (DNMT1, 2 and 3). Interestingly, two species (*Selaginella moellendorffii*, *Physcomitrella patens*) has just DNMT1 and 3, and two just DNMT1 and 2 (*Tribolium castaneum*, *Gasterosteus aculeatus*). Also, for *Strigamia martima* DNMT1 and DNMT3 were found, but DNMT2 was not searched for (Supplementary File 5). We consider cluster 2 as “low gene body methylation” (type 2).

Species with a mode position ≤ 0.69 form the cluster 3 (mean CpG*o/e* position is 0.45). The skewness is positive and larger than in the two first clusters (mean_absolute Q50 skewness_ = 0.0483). The standard deviation (mean_SD_ = Da0.19) is wider than the cluster 1 and 2. Forty-three species (29.86%) are present in this cluster, belonging to various phylogenetic groups (apicomplexa, sponges and nematodes, arthropoda, a large panel of chordata, and embryophytes). Many of them are important model organisms. For 26 (60%) literature data on DNA methylation was found. Gene bodies are methylated. In 19 species DNMTs were analyzed. Twelve species have all three DNMTs. One species (*Naegleria gruberi*) has DNMT1 and 2, but methylation of DNA was not yet studied. Four species have only DNMT2. Two species has DNMT1 and 3, and one only DNMT1 (Supplementary File 5). We qualify this cluster as with “gene body methylation” (type 3).

Finally, cluster 4 contains species that show bimodality or are strongly negatively skewed CpGo/e distributions (mean_absolute Q50 skewness_ = −0.0424, mean CpG*o/e* position of mode 1 is 0.54, of mode 2 0.85). Twenty-seven species (18.75%) from different phylogenetic groups compose this cluster (apicomplexa, cnidaria, nematoda, arthropoda, mollusca, tunicata, embryophyta). Five species are strongly negatively skewed and 15 species are bimodal. We found information on DNA methylation for 10 species (50%). All species show a mosaic type of methylation with DNA regions of ultra-low methylation interspersed with regions of strong methylation. Eleven species out of 15 that were studied have the three DNMT (1, 2 and 3), two had DNMT1 and 2, and two DNMT1 and 3 with uncertainty about DNMT2 (Supplementary File 5). Species in this cluster were considered as “mosaic type DNA methylation” (type 4).

**Figure 1:**
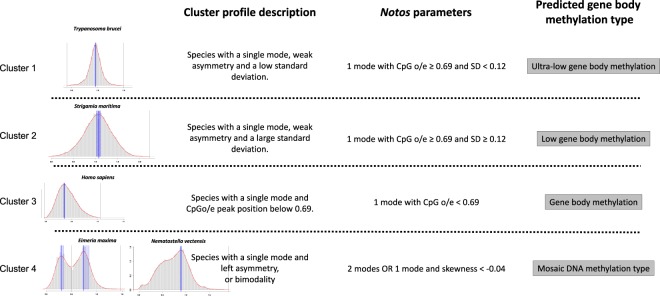
Summary of decision grid for clustering of CpG o/e ratio distributions on species level.

Decision criteria are summarized in Fig. 1.

### DNA methylation types do not follow the tree of life but are consistent within major clades

**Figure 2:**
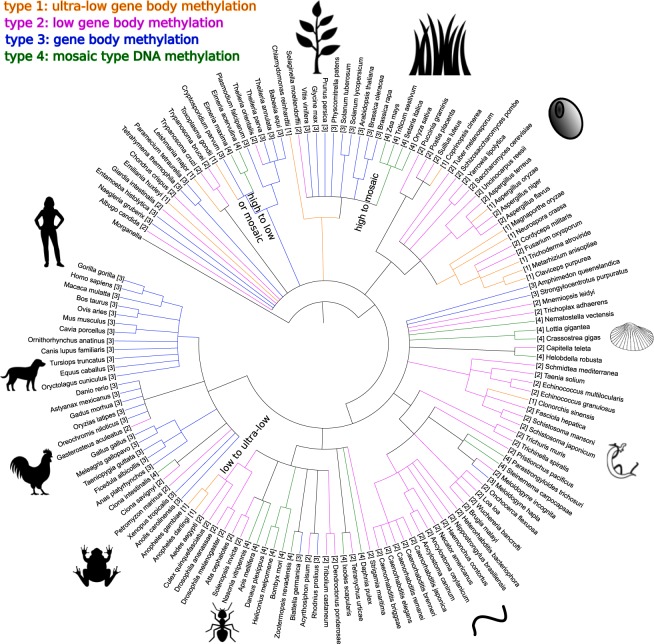
Schematic representation of the “Tree of Life” for 147 species, associated with the four different types of DNA methylation that were identified in this work. Numbers in brackets indicate DNA methylation types (“clusters”) for each species. Line colors correspond to methylation types.

Among unicellular organisms, kinetoplastids are unmethylated, while alveolate protists are generally methylated (Fig. 2) with secondary loss of methylation that can lead to weaker methylation level or mosaic methylation. In flowering plant species, we differentiate either high (probably global) methylation in dicotyledons, and mosaic methylation in poaceae and potentially all monocotyledons. Fungi process in general ultra-low to weak methylation in the gene bodies. Also, platyhelminthes are characterized by low methylation in the coding regions. Gene bodies of deuterostomes are in general strongly methylated. There are, however, peculiar cases, *e*.*g*. two tunicate species (*Ciona intestinalis* and *C*. *savignyi*) that diverged from each other 184 (±15) Mya are in two different clusters (types 4 and 2, respectively)^42^ with mosaic and weak methylation, probably due to secondary loss of methylation. Within lophotrochozoa, annelids show low gene body methylation, and all studied mollusks are of the mosaic type. Nematodes have in general weak gene body methylation. A particular interesting and heterogenic clade concerning methylation types are arthropods. All tested diptera (and potentially all antliophora) belong to the low methylation clusters 1 and 2, certainly due to secondary loss of methylation after splitting from its insect sister clades. All other orders show weak to high methylation with occasionally mosaic type, probably through secondary gain of local methylation.

## Discussion

Evolution is based on the selection of phenotypic variants that must (i) confer a reproductive advantage to the individual, and (ii) are heritable, *i*.*e*. information how to generate the phenotypic variants in response to an environment are passed from parents to offspring. Heritability has traditionally been thought to be exclusively genetic, *i*.*e*. based on variations in the DNA sequence. In this view, genetic information is then expressed under influence of environmental cues to bring about the phenotype, a process known as G × E→ P^43^. During the last 30 years it became however clear, that a substantial amount of heritable phenotypic variance can be coded by non-genetic means^44^. We had earlier conceptualized this view as a systems approach to inheritance, that includes genetic, epigenetic, cytoplasmic and microbial elements that are interrelated by forward and reverse interaction^2^. These elements interact mutually, and with the environment, to give raise to the phenotype. In this concept, genetic information (the genotype) is only one of many elements that as part of an inheritance system providing heritable information, that the environment will shape into a phenotype. We define here ‘inheritance system’ as a system that is able to write and store, transmit, and receive hereditary information^45^. The concept implies also that genotype and epigenotype cannot exist independent of each other, and are interrelated by forward action and feed-back. This is different from the idea that sees the genome as hard-wired information that is controlled by the epigenome^46^. In the latter, the epigenome is conceptually closer to the (molecular) phenotype (*i*.*e*. product of the genotype) than to an element of the inheritance system itself.

The introduction of the epigenotype notion did not really solve the question, since theoretically each phenotype could just be the visible expression of its underlying epigenotype. Given the multiple facets phenotypes can acquire in living organisms, it is remarkable that, with very few exceptions, the genetic material and the genetic code remains extremely constant and thus universal^47^. In other words, there exists a single ‘type’ of genome. The origin of this universality of the genetic code remains enigmatic and controversial but whatever the origin is, it allows to transmit coded information from one generation to the next. These generations can understand the code since it uses a universal and constant key.

Given the presumably close relation between genotype and epigenotype we and others reasoned that the epigenotype and the epigenetic code should equally possess universality. The high conservation of histones and histone marks, and the conservation of methylation of cytosines suggests indeed this. Nevertheless, one could argue that the epigenetic code is simply entirely genetically determined. If this were true, we would expect that different DNA methylation types would correspond to the clades in taxonomical tree that are based on DNA sequence similarity. Our results do not support this view. Alternatively, DNA methylation types could entirely be determined by environmental conditions. In this case, similar environments should impose similar DNA methylation types. Neither our results, nor recent analyses of DNA methylation in invertebrates provide evidence of this. *E*.*g*. a very comprehensive study of DNA methylation in insects^37^ did not find relations of methylation types to social behavior and the authors concluded that DNA methylation must have “more ubiquitous function”. However, compared to the tremendous amount of genomic data that is available, epigenomic data is relatively sparse and biased, which is an obstacle to answer the question conclusively. In the present study, we coped with this caveat by using a hybrid approach in which we combined available experimental data on DNA methylation with results coming from a newly developed software that predicts gene body DNA methylation types with CpG o/e ratios. Our algorithm (based on the number of species positive predicted and true positives based on the literature) allowed for including species for which no experimental DNA methylation data existed. The PPV of the algorithm is excellent for mosaic methylation (PPV = 1), and methylated gene bodies (PPV = 0.875), but decreases then to 0.75 (low methylated) and 0.5 for ultra-low gene body methylation. This is due to the fact that our algorithm does not differentiate well between low and ultra-low methylation. If we consider the dataset as a whole, out of the 54 species with known DNA methylation types, 41 were predicted correctly (total PPV = 0.76).

There are some particularly interesting cases of “wrong” prediction. *Cryptosporium parvum* is a monoxenic unicellular parasite of vertebrates. It belongs to the apicomplexan its exact phylogenetic position is controversial. Exysted oocysts are the only stage that can be used to produce host DNA free genomic DNA preparations. Notos predicts clearly high gene body methylation but LC-ESI-MS did not detect 5mC in exysted oocysts purified from infected cattle^48^. Genome analysis of *C*. *hominis* to which *C*. *parvum* has only 3–5% sequence divergence^49^, showed that the number of genes is reduced (3,952 genes) compared to other apicomplexan, relying heavily on host gene activity. The genome shows also traces of integration of genes by lateral transfer. We hypothesize that either the progenitor DNA was methylated, or that cryptosporidium methylates DNA in the intracellular stages using the vertebrate host DNMTs.

Another peculiar case is the ciliate *Tetrahymena thermophile*. Also for this species Notos predicted high methylation while radioisotope labeling showed that *Tetrahymena* contains only N6-methyl-adenine but not 5mC^50^. *T*. *thermophila* and other ciliates use DNA elimination to remove approximately one-third of the genome, when the somatic macronucleus differentiates from the germline micronucleus. Histone 3 lysine 9 trimethylation (H3K9me3) is deposited on DNA destined for this elimination (reviewed in Bracht^51^). Interestingly, in other ciliates, DNA methylation is used for the tagging of DNA to be eliminated. It might therefore be that *Tetrahymena* had used DNA methylation in the past and has lost this capacity relatively recently, so that we still see traces in the CpG o/e ratio.

In summary, Notos predicts very reliable mosaic and high gene body methylation without being entirely error free. We had earlier^36^ used only mode number (1 or 2, *i*.*e*. non-mosaic and mosaic methylation) and peak position of 0.75 to differentiate species with presumably methylated (<0.75) and non-methylated (≥0.75) gene bodies. For the present work we added skewness −0.04, and SD 0.12, and changed peak position threshold to 0.69 for better prediction.

Conceptually, our approach is based on the classical observation that CpN dinucleotides are observed in statistically expected frequency in low methylated regions or genomes. It was initially used to identify unmethylated CpG island in vertebrate promoters, and two major algorithms exist (Gardiner-Garden and Frommer^52^ and Takai and Jones^53^). These two algorithms use the CpG o/e ratios with a score above 0.60 and 0.65, respectively. ‘Score’ (here ‘mode position’ Mo) is one parameter of our clustering algorithm. We used a decision tree to iteratively adjust this score and reached 0.69. This value is close to what was used in previous studies (*e*.*g*. for *C*. *intestinalis*: 0.70^54^ and 0.80^31^, and *Nematostella vectensis*, 0.70^54^, or *Apis mellifera*, 1.0^54^). It is conceivable that Mo could be slightly different for each major phylogenetical clade, and using more sophisticated clustering algorithms such as support vector machine clustering that can use multiple thresholds could still improve the PPV of our method. In addition, more experimental data on a wide range of organisms is urgently needed.

We find that there are four types of gene body methylation. Despite a much wider data basis in terms of phylogenetic clades, our results confirm earlier findings that concluded on three to four DNA methylation types^17,24^. This could be the result of a “frozen accident” situation in which methylation (e.g. type 1 and type 3) occurred randomly in early ancestors (since 5mC is coding neutral that would not have had an impact on translation), but with the establishment of a chromatin structure 5mC was recruited as epigenetic information carrier, and any change in DNA methylation type would have had a strong impact on genome function and thus fitness and was therefore maintained. Nevertheless, switching of methylation type has occurred in evolutionary time scales. Our findings indicate that there were at least three large events of secondary loss of DNA methylation: in archaeplastida (the “true” plants) where we find one branch with high methylation and another with mosaic methylation (in monocotyledons), ultra-low or mosaic methylation in the apicomplexa branch of “protists”, and one transition to ultra-low gene body methylation in Diptera (Fig. 2). For *D*. *melanogaster* in the dipteran branch it was shown experimentally that only the ‘writing’ capacity of the epigenetic inheritance element was lost, not the receiving (‘reading’) capacity^55^. The reason for evolutionary switching between methylation types is not clear and arguments are controversial.

It has been proposed that secondary loss of DNA methylation occurs because its mutational costs outweighed its adaptive value^56^. Indeed, in mosaic type methylation it is the evolutionary stable “old” genes that are in the methylated compartments meaning that there must be stabilizing mechanisms that prevent mutations there. Therefore, it might not be the mutational costs but the costs of maintaining such mechanisms that becomes an evolutionary burden. It was an early observation that CpG containing codons are used much less in coding sequences of vertebrates, and mutations due to CpG methylation was considered a major cause for such codon bias^57^ and therein. Codon bias was observed also recently in the reef-building coral *Acropora millepora*^57^, and linked to mosaic methylation in this species. Again, phylogenetically old genes which are constitutively expressed are methylated and CpG depleted. The authors conclude that CpG methylation leads to mutations that establish a set of preferred codons in constitutively expressed genes. Once such codon bias is fixed, then alleles that control the abundance of appropriate tRNAs could have stronger effects more amenable to natural selection. The authors hypothesize that an advantage of mutation-driven codon bias that it would be beneficial for organisms with small population size or otherwise inefficient selection. Still another explanation for mosaic methylation was advanced by Gavery and Roberts^32^ who speculated that hypo-methylated regions (here in the pacific oyster *Crassostrea gigas*) might have greater epigenetic flexibility and higher regulatory control than hyper-methylated ones. Mosaic methylation could also be the result of whole genome duplication (WGD) events as suggested for *Oryza sativa*^56^. In addition, we have shown that environmental conditions can influence on germ-line methylation in *C*. *gigas* that possess mosaic methylation, and that blocks of CpG methylation are added or removed preferentially in or around genes^58^. One should keep in mind that DNA methylation is only one of many bearers of epigenetic information. Another one, and probably the most difficult to capture is the topology of the interphase nucleus. Using Hi-C data, Lieberman-Aiden *et al*.^59^. established that the human genome is divided into two compartments (A-B) with pairs of loci in compartment B showing higher interaction frequency at a given genomic distance than pairs of loci in compartment A. They concluded that compartment B is more densely packed (heterochromatic) than compartment A. Higher average DNA methylation was later found to be a good predictor for the open compartment A in human cell lines^60^ but that link could be broken in cancer cells. This cannot be interpreted as DNA methylation being decisive for topologically associated domains (TAD) establishment since DNA methylation free organisms such as *D*. *melanogaster* also presents canonical A-B domains^61^. But in drosophila, such TAD organization is not driven by long-lived interactions but rather relies on the formation of transient, low-frequency contacts^62^. We hypothesize therefore that DNA methylation actually impacts on the relative dynamics of formation of contacts in A and B compartments, possibly stabilizing them. It is tempting to speculate that one consequence of compartmentation of genomes dynamics by methylation is that this might create additional units of selection. Results from tunicates support this idea: *Ciona* CpG*o/e* ratios have different profiles (bimodal for *C*. *intestinalis* and unimodal for *C*. *savignyi*). The *C*. *intestinalis* methylome is predicted to be mosaic that corresponds to experimental observations^63^. Our prediction for *C*. *savignyi* is low methylation (cluster 2). Both species diverged from each other 184 (±15) Mya^42^ and their genomes are very different^64,65^. For instance, analysis of 18S rRNA sequences shows that the pairwise divergence of the two *ciona* species is slightly greater than that between human and *e*.*g*. birds^66^. This is puzzling since developmental features, body plan, effective population size and environment are very similar, and even hybrids can be generated to the tadpole stage^67^. However, *C*. *savignyi* shows a genome wide average Single Nucleotide Polymorphism (SNP) heterozygosity of 4.5% while *C*. *intestinalis*, that has mosaic methylation, is genetically less polymorphic (1.5%) (reviewed in Veeman *et al*.^68^). It is conceivable that the methylated *C*. *intestinalis* genome can generate sufficiently stable TADs so that genome x epigenome interactions can serve as heritable unit of selection, while in *C*. *savignyi* TADs are more dynamic because the relative weight of DNA methylation in the generation of stable heritable phenotypic variants is less important. Our prediction concords with very recent results showing that stress-induced DNA methylation changes in *C*. *savignyi* can occur but are highly ephemeral (<48–120h), and thus not maintained through germline^69^.

In conclusion, our findings indicate that initially there were three types of gene body DNA methylation: ‘primary no methylation’, ‘primary whole genome methylation’, and ‘primary mosaic methylation’ that produced by secondary loss ‘weak methylation’, or ‘secondary no methylation’. These findings are in concordance with the idea that DNA methylation in gene bodies (i) uses three types of universal codes (low, high and mosaic)), and (ii) that it is an element of the inheritance system and not a molecular phenotype that results from genotype × environment interaction. This has immediate practical consequences: e.g. since there are three types of methylation codes, pan-species conclusions about the potential function of DNA methylation can only be drawn within the type (*e*.*g*. functional tests in vertebrates with high gene body methylation cannot be used to conclude on methylation function in mosaic type mollusks). In addition, if DNA methylation is part of the inheritance system then heritable phenotypic diversity can be produced by DNA methylation changes without changes in the DNA sequence. The notion that everything that is heritable is necessarily genetic should be abandoned.

## Methods

### Origin of sequences, data cleaning and Notos parameters

In this study, coding sequences (CDS) or cDNA sequences of 147 species were downloaded from Ensembl and VEGA databases. Expressed sequence tags (ESTs) were downloaded from two different databases: dbEST^70^ (605 species) and CleanEST^71^ (110 species) (Supplementary File 3). We used Notos^36^ for the calculation and modelling of CpG*o/e* distribution with the three datasets, with a minimal length L = mi200 bp and formula 1^72^:

1 $$CpGo/e=\frac{number\,of\,CpG}{number\,of\,C\,\times number\,of\,G}\times \frac{{L}^{2}}{L-1}$$

All the values outside the interval, and all the values with a score of 0 were removed. For each species, the number of mode, the position of mode(s), the Q50 skewness coefficient and the standard deviation (SD) were calculated.

### Blast searches and gene ontology analysis

Database searches were done by Blastx searches against a local instance (ncbi-blast-2.2.30+) of non-redundant ‘nr’ with 20 maximum hits, an E-value of 0.001, and other parameters as default values. Gene ontology searches were performed with blast2go^73^.

### RNA seq analysis

RNAseq datasets for *Nematostella vectensis*, *Nasonia vitripennis*, *Crassostrea gigas* and *Oryza sativa japonica* were downloaded as fastq files from the European Nucleotide Archive and NCBI (details in Supplementary File 3). For each dataset, the reads were filtered with a Fred quality score ≥26. Filtered reads were mapped on their reference genomes (downloaded from Ensembl, details in Supplementary File 3) with RNA STAR^74^ on a local Galaxy instance (v2.4.0d-2). Resulting BAM files and the gff files (downloaded from Ensembl, details in Supplementary File 3) with the coding sequences were used for FPKM estimations with Cufflinks^75^. Annotation gff-files were used to extract CDS in fasta format from their genomes and we calculated the CpG*o/e* ratios with Notos^36^ and detected modes (peaks). To compare FPKM for the genes under the peaks, a bandwidth of 0.2 (±0.1 around mode maximum) was arbitrarily chosen for the CpG o/e ratio. FPKMs were extracted and used for statistical analysis of expression level in gene bodies with low and high predicted methylation. Mood’s median test was used with R^76^.

### Meta-analysis of DNA methylation using literature data

For each species for which data was available in the above-mentioned databases, we searched the literature on Google scholar (as of April–June 2016) with the following keywords: DNA methylation, 5-methyl-cytosine, gene body, mosaic methylation, global methylation, DNA methylation pattern. Articles were obtained from Bib CNRS (https://bib.cnrs.fr/) and manually curated to obtain gene body methylation, and presence of DNMTs.

### Clustering

To identify distinct subgroups within the 147 analyzed species, we generated descriptive analyses, considering both the KDE of the CpGo/e ratios and aggregating statistics based on it. The statistics we used were (1) the number of modes of the KDE, (2) the position of the modes, (3) the standard deviation SD of the CpGo/e ratios, (4) absolute Q50 mode skewness of the CpGo/e ratios, *i*.*e*.,

2 $$\frac{{Q}_{3}+{Q}_{1}}{2}-Mo$$

with Q_1_ and Q_3_ the 25% and 75% quantile of the CpGo/e ratios, respectively, and Mo the global mode of the KDE. We investigated several formulas for the skewness, deeming the absolute Q50 mode skewness the most informative for our analysis^36^. For the sake of readability, we refer to the absolute Q50 mode skewness as “skewness” in what follows.

The four clusters into which we classify the species are specified in the result section. The values of the three thresholds used in the definition of the clusters were determined by evaluating the prediction performance of our approach depending on these three values, using 54 species for which the true methylation type had been determined experimentally (given in Supplementary File 5). Hereby, the clusters correspond to the patterns used in that file like follows: Cluster 1 - not methylated; Cluster 2 - low methylated; Cluster 3 - (high) methylated/global methylation; Cluster 4 - mosaic methylation.

To determine the optimal threshold values, we employed a two-step approach. First, we searched the whole parameter space (*i*.*e*. the space of all possible values the thresholds can assume) using a Metropolis-Hastings algorithm to ensure that strongly deviating from the threshold values we chose manually always leads to a poor prediction performance. Second, we systematically searched the parameter space around the manually chosen values. That is, we evaluated the prediction performance on a grid of size 21³ on9261, covering the following threshold values: skewness from −0.08 to −0.04 in steps of 0.002; peak position from 0.69 to 0.79 in steps of 0.005; SD from 0.11 to 0.21 in steps of 0.005. For the present work we used: skewness −0.04, peak position 0.69, and SD 0.12.

Due to the scarcity of the data, the optimal prediction (76%, 41 out of 54 true) is achieved for a rather large set of threshold values. To judge the performance of our algorithm, it should be noted that for 7 out the 13 misclassified species the true and the predicted classifications are “not methylated” and “low methylated”, or *vice versa* respectively. That is, the mistake made by the algorithm is rather small in these cases. The remaining four wrong predictions are actually peculiar cases that were discussed above.

The clustering was implemented using R version 3.4.0 (Supplementary Files 6 and 7). For visualizing the clustering, the R package dendextend has been used. Parameters were Rscript cluster.r −0.04 0.69 0.12 *input_file_notos input_file_notos_bootstrap* with first parameter [skewness], second [Mo], and third [SD], and Notos outputfiles as input. Further details on our method can be found in^25^.

### Tree of life

We recovered the taxonomic IDs of all investigated species from the NCBI taxonomy database (https://www.ncbi.nlm.nih.gov/taxonomy) and created a tabular file (.txt). We used this file to generate a Phylip tree file based on the classification in the NCBI taxonomy database with the NCBI common taxonomy tree online tool (https://www.ncbi.nlm.nih.gov/Taxonomy/CommonTree/wwwcmt.cgi) and designed a tree with the interactive Tree of life (version 4.0.2) (https://itol.embl.de)^76^.

## Supplementary information

Supplementary figures 1–9

Supplementary file 1

Supplementary file 2

Supplementary file 3

Supplementary file 4

Supplementary file 5

Supplementary file 6

Supplementary file 7

## References

[1] Levine AJ (2017). The Future of Systems Biology. Curr. Opin. Syst. Biol..

[2] Cosseau C (2017). Epi)genetic Inheritance in Schistosoma mansoni: A Systems Approach. Trends Parasitol..

[3] Nicoglou, A. & Merlin, F. Epigenetics: A way to bridge the gap between biological fields. *Stud*. *Hist*. *Philos*. *Sci*. *Part C Stud*. *Hist*. *Philos*. *Biol*. *Biomed*. *Sci*. 1–10 10.1016/j.shpsc.2017.10.002 (2017). 10.1016/j.shpsc.2017.10.002 29033228

[4] Hotchkiss RD (1948). The quantitative separation of purines, pyrimidines and nucleosides by paper chromatography. J. Biol. Chem..

[5] Ye P (2017). MethSMRT: an integrative database for DNA N6-methyladenine and N4-methylcytosine generated by single-molecular real-time sequencing. Nucleic Acids Res..

[6] Chen W, Yang H, Feng P, Ding H, Lin H (2017). iDNA4mC: identifying DNA N4-methylcytosine sites based on nucleotide chemical properties. Bioinformatics.

[7] Vanyushin, B. F. In DNA Methylation: Basic Mechanisms 67–122, 10.1007/3-540-31390-7_4 (Springer-Verlag, 2006).

[8] Cambareri E, Jensen B, Schabtach E, Selker E (1989). Repeat-induced G-C to A-T mutations in Neurospora. Science (80-.)..

[9] Lyko, F. The DNA methyltransferase family: a versatile toolkit for epigenetic regulation. *Nat*. *Rev*. *Genet*. 10.1038/nrg.2017.80 (2017). 10.1038/nrg.2017.80 29033456

[10] Riggs AD, Xiong Z, Wang L, LeBon JM (2008). Methylation dynamics, epigenetic fidelity and X chromosome structure. Epigenetics.

[11] Hermann A, Schmitt S, Jeltsch A (2003). The human Dnmt2 has residual DNA-(Cytosine-C5) methyltransferase activity. J. Biol. Chem..

[12] Goll MG (2006). Methylation of tRNAAsp by the DNA Methyltransferase Homolog Dnmt2. Science (80−.)..

[13] Albalat R (2008). Evolution of DNA-methylation machinery: DNA methyltransferases and methyl-DNA binding proteins in the amphioxus Branchiostoma floridae. Dev. Genes Evol..

[14] Schaefer M, Lyko F (2010). Solving the Dnmt2 enigma. Chromosoma.

[15] Raddatz G (2013). Dnmt2-dependent methylomes lack defined DNA methylation patterns. Proc. Natl. Acad. Sci..

[16] Okano M, Xie S, Li E (1998). Cloning and characterization of a family of novel mammalianDNA (cytosine-5) methyltransferases. Nat. Am. Inc..

[17] Suzuki MM, Bird A (2008). DNA methylation landscapes: provocative insights from epigenomics. Nat. Rev. Genet..

[18] Rivenbark AG (2012). Epigenetic reprogramming of cancer cells via targeted DNA methylation. Epigenetics.

[19] Casimir CM, Gates PB, Patient RK, Brockes JP (1988). Evidence for dedifferentiation and metaplasia in amphibian limb regeneration from inheritance of DNA methylation. Development.

[20] Mugatroyd C, Wu Y, Bockmühl Y, Spengler D (2010). The janus face of DNA methylation in aging. Aging (Albany. NY)..

[21] Zampieri M (2015). Reconfiguration of DNA methylation in aging. Mech. Ageing Dev..

[22] Dowen RH (2012). Widespread dynamic DNA methylation in response to biotic stress. Proc. Natl. Acad. Sci. USA.

[23] Cortijo S (2014). Mapping the Epigenetic Basis of Complex Traits. Science (80-.)..

[24] Yi SV, Goodisman MAD (2009). Computational approaches for understanding the evolution of DNA methylation in animals. Epigenetics.

[25] Bulla I (2018). Notos - a galaxy tool to analyze CpN observed expected ratios for inferring DNA methylation types. BMC Bioinformatics.

[26] Bird AP (1980). DNA methylation and the frequency of CpG in animal DNA. Nucleic Acids Res..

[27] Fryxell KJ, Moon WJ (2005). CpG mutation rates in the human genome are highly dependent on local GC content. Mol. Biol. Evol..

[28] Cooper DN, Krawczak M (1989). Cytosine methylation and the fate of CpG dinucleotides in vertebrate genomes. Hum. Genet..

[29] Jabbari K, Bernardi G (2004). Cytosine methylation and CpG, TpG (CpA) and TpA frequencies. Gene.

[30] Razin A, Cedar H (1977). Distribution of 5-methylcytosine in chromatin. Proc. Natl. Acad. Sci. USA.

[31] Suzuki MM, Kerr ARW, De Sousa D, Bird A (2007). CpG methylation is targeted to transcription units in an invertebrate genome. Genome Res..

[32] Gavery MR, Roberts SB (2010). DNA methylation patterns provide insight into epigenetic regulation in the Pacific oyster (Crassostrea gigas). BMC Genomics.

[33] Park J (2011). Comparative analyses of DNA methylation and sequence evolution using Nasonia genomes. Mol. Biol. Evol..

[34] Dixon GB, Bay LK, Matz MV (2014). Bimodal signatures of germline methylation are linked with gene expression plasticity in the coral Acropora millepora. BMC Genomics.

[35] Walsh TK (2010). A functional DNA methylation system in the pea aphid, Acyrthosiphon pisum. Insect Mol. Biol..

[36] Bulla, I. *et al*. Notos - a Galaxy tool to analyze CpN observed expected ratios for inferring DNA methylation types. bioRxiv 10.1101/180463 (2017). 10.1186/s12859-018-2115-4 PMC5870242 29587630

[37] Bewick AJ, Vogel KJ, Moore AJ, Schmitz RJ (2016). Evolution of DNA Methylation across Insects. Mol. Biol. Evol..

[38] Driscoll T, Gillespie JJ, Nordberg EK, Azad AF, Sobral BW (2013). Bacterial DNA sifted from the Trichoplax adhaerens (Animalia: Placozoa) genome project reveals a putative rickettsial endosymbiont. Genome Biol. Evol..

[39] Storb U, Arp B (1983). Methylation patterns of immunoglobulin genes in lymphoid cells: correlation of expression and differentiation with undermethylation. Proc. Natl. Acad. Sci. USA.

[40] Bewick AJ, Schmitz RJ (2017). Gene body DNA methylation in plants. Curr. Opin. Plant Biol..

[41] He X-J, Chen T, Zhu J-K (2011). Regulation and function of DNA methylation in plants and animals. Cell Res..

[42] D’Onofrio, G., Berná, L. & Alvarez-Valin, F. How fast is the sessile Ciona? *Comp*. *Funct*. *Genomics* **2009** (2009). 10.1155/2009/875901 PMC2801007 20052388

[43] Bowman J (1972). Genotype×environment interactions. Genet. Sel. Evol..

[44] Danchin E (2011). Beyond DNA: integrating inclusive inheritance into an extended theory of evolution. Nat. Rev. Genet..

[45] Lamm, E. In The Standford Encyclopedia of Philosophy (ed. Zalta, E. N.) (Metaphysics Research Lab, Stanford University). at https://plato.stanford.edu/archives/win2014/entries/inheritance-systems (2014).

[46] Laland K (2014). Does evolutionary theory need a rethink?. Nature.

[47] Koonin EV, Novozhilov AS (2009). Origin and evolution of the genetic code: The universal enigma. IUBMB Life.

[48] Gissot M, Choi SW, Thompson RF, Greally JM, Kim K (2008). Toxoplasma gondii and Cryptosporidium parvum lack detectable DNA cytosine methylation. Eukaryot. Cell.

[49] Xu P (2004). The genome of Cryptosporidium hominis. Nature.

[50] Hattman S, Kenny C, Berger L, Pratt K (1978). Comparative study of DNA methylation in three unicellular eucaryotes. J. Bacteriol..

[51] Bracht JR (2014). Beyond transcriptional silencing: Is methylcytosine a widely conserved eukaryotic DNA elimination mechanism?. BioEssays.

[52] Gardiner-Garden M, Frommer M (1987). CpG Islands in vertebrate genomes. J. Mol. Biol..

[53] Takai D, Jones PA (2002). Comprehensive analysis of CpG islands in human chromosomes 21 and 22. Proc. Natl. Acad. Sci. USA.

[54] Nanty L (2011). Comparative methylomics reveals gene-body H3K36me3 in Drosophila predicts DNA methylation and CpG landscapes in other invertebrates. Genome Res..

[55] Lyko F (1999). Mammalian (cytosine-5) methyltransferases cause genomic DNA methylation and lethality in Drosophila. Nat. Genet..

[56] Zemach, A., Mcdaniel, I., Silva, P. & Zilberman, D. Genome-Wide Evolutionary Analysis of Eukaryotic DNA Methylation. *Sci*. *(New York*, *NY)* **11928**, science.1186366v1 (2010). 10.1126/science.1186366 20395474

[57] Dixon GB, Bay LK, Matz MV (2016). Evolutionary Consequences of DNA Methylation in a Basal Metazoan. Mol. Biol. Evol..

[58] Rondon R (2017). Effects of a parental exposure to diuron on Pacific oyster spat methylome. Environ. Epigenetics.

[59] Lieberman-Aiden E (2009). Comprehensive Mapping of Long-Range Interactions Reveals Folding Principles of the Human Genome. Science (80-.)..

[60] Fortin J-P, Hansen KD (2015). Reconstructing A/B compartments as revealed by Hi-C using long-range correlations in epigenetic data. Genome Biol..

[61] Sexton T (2012). Three-dimensional folding and functional organization principles of the Drosophila genome. Cell.

[62] Cattoni, D. I. I. *et al*. Single-cell absolute contact probability detection reveals that chromosomes are organized by multiple, low-frequency yet specific interactions. *Doi*.*Org* 159814 10.1101/159814 (2017). 10.1038/s41467-017-01962-x PMC5700980 29170434

[63] Suzuki MM (2013). Identical sets of methylated and nonmethylated genes in Ciona intestinalis sperm and muscle cells. Epigenetics Chromatin.

[64] Small KS, Brudno M, Hill MM, Sidow A (2007). Extreme genomic variation in a natural population. Proc. Natl. Acad. Sci. USA.

[65] Kourakis, M. J. & Smith, W. C. An organismal perspective on C. intestinalis development, origins and diversification. *Elife* **317** (2015). 10.7554/eLife.06024 PMC4373457 25807088

[66] Johnson DS, Davidson B, Brown CD, Smith WC, Sidow A (2004). Noncoding regulatory sequences of Ciona exhibit strong correspondence between evolutionary constraint and functional importance. Genome Res..

[67] Byrd J, Lambert CC (2000). Mechanism of the block to hybridization and selfing between the sympatric ascidiansCiona intestinalis andCiona savignyi. Mol. Reprod. Dev..

[68] Veeman, M. T., Chiba, S. & Smith, W. C. In Vertebrate Embryogenesis: Embryological, Cellular, and Genetic Methods (ed. Pelegri, F. J.) 401–422 (Humana Press,). 10.1007/978-1-61779-210-6_15 (2011).

[69] Huang X (2017). Rapid response to changing environments during biological invasions: DNA methylation perspectives. Mol. Ecol..

[70] Boguski MS, Tolstoshev TMJLCM (1993). dbEST-database for ‘expressed sequence tags’. Nat. Genet..

[71] Lee B, Shin G (2009). CleanEST: A database of cleansed EST libraries. Nucleic Acids Res..

[72] Matsuo K, Clay O, Takahashi T, Silke J, Schaffner W (1993). Evidence for erosion of mouse CpG islands during mammalian evolution. Somat. Cell Mol. Genet..

[73] Conesa A (2005). Blast2GO: A universal tool for annotation, visualization and analysis in functional genomics research. Bioinformatics.

[74] Dobin A (2013). STAR: Ultrafast universal RNA-seq aligner. Bioinformatics.

[75] Trapnell C (2010). Transcript assembly and quantification by RNA-Seq reveals unannotated transcripts and isoform switching during cell differentiation. Nat. Biotechnol..

[76] R Development Core Team. R: A Language and Environment for Statistical Computing. At http://www.r-project.org (2008).

[77] Letunic I, Bork P (2016). Interactive tree of life (iTOL)v3: an online tool for the display and annotation of phylogenetic and other trees. Nucleic Acids Res..

[78] Lyko, F. *et al*. The honey bee epigenomes: Differential methylation of brain DNA in queens and workers. *PLoS Biol*. **8** (2010). 10.1371/journal.pbio.1000506 PMC2970541 21072239

[79] Fneich S (2013). 5-methyl-cytosine and 5-hydroxy-methyl-cytosine in the genome of Biomphalaria glabrata, a snail intermediate host of Schistosoma mansoni. Parasit. Vectors.

[80] Wurm Y (2011). The genome of the fire ant Solenopsis invicta. Proc Natl Acad Sci USA.

[81] Simola DF (2013). Social insect genomes exhibit dramatic evolution in gene composition and regulation while preserving regulatory features linked to sociality. Genome Res..

[82] Wang, X. *et al*. Function and Evolution of DNA Methylation in Nasonia vitripennis. *PLoS Genet*. **9** (2013). 10.1371/journal.pgen.1003872 PMC3794928 24130511

[83] Robinson, K. L., Tohidi-Esfahani, D., Lo, N., Simpson, S. J. & Sword, G. A. Evidence for widespread genomic methylation in the migratory locust, Locusta migratoria (orthoptera: Acrididae). *PLoS One* **6** (2011). 10.1371/journal.pone.0028167 PMC3230617 22163001

[84] Xiang H (2010). Single base-resolution methylome of the silkworm reveals a sparse epigenomic map. Nat. Biotechnol..

[85] Cunningham CB (2015). The Genome and Methylome of a Beetle with Complex Social Behavior, Nicrophorus vespilloides (Coleoptera: Silphidae). Genome Biol. Evol..

[86] Simmen MW (1999). Nonmethylated transposable elements and methylated genes in a chordate genome. Science.

[87] Chen F-C, Chuang T-J, Lin H-Y, Hsu M-K (2014). The evolution of the coding exome of the Arabidopsis species - the influences of DNA methylation, relative exon position, and exon length. BMC Evol. Biol..

