# Supplementary material for: Universality of the DNA methylation codes in Eucaryotes
Source: Sci Rep. 2019 Jan 17;9:173. doi: 10.1038/s41598-018-37407-8 (PMC6336885; doi:10.1038/s41598-018-37407-8)

Universality of the DNA methylation codes in Eucaryotes

Benoît Aliaga[1], Ingo Bulla[1,2,3], Gabriel Mouahid[1], David Duval[1] and Christoph Grunau[1]*

(1) Univ. Perpignan Via Domitia, IHPE UMR 5244, CNRS, IFREMER, Univ. Montpellier, F-66860 Perpignan, France
(2) Institute for Mathematics and Informatics, University of Greifswald, Greifswald, Germany
(3) Department of Computer Science, ETH Zürich, Zürich, Switzerland

Supplementary figures

Supplementary figure 1 : DNA contamination in *Anolis carolinensis*

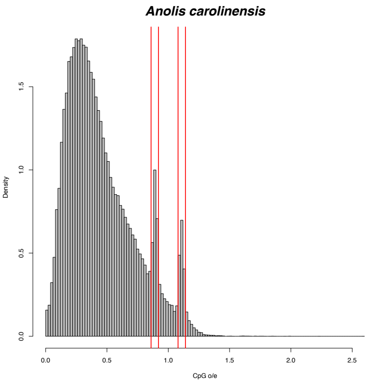

Supplementary figure 1.1: the CpGo/e profile of *Anolis carolinensis* from dbEST and its two additional peaks (peak 01-left and peak 02-right)

When we compared the dbEST profile with CleanEST and CDS profiles, two additional peaks occurred in the dbEST profile. We reasoned that the sequences present in the two peaks were not from *Anolis carolinensis* but were contaminant. In order to verify this hypothesis, we did a gene ontology research in these two additional peaks. We isolated and extracted DNA sequences from the dbEST fasta files. We used Blast2go for gene ontology search[1].

The first peak (7,030 sequences with a CpGo/e ratio between 0.92 and 1.08) contains a chloramphenicol O-acetytransferase used in bacterial cloning vectors. The second peak (4,922 sequences with a CpGo/e ratio between 1.14 and 1.22) present homologies with sequences from apicomplexans (plasmodium), and platyhelminths suggesting presence of such parasites in the initial biological sample.

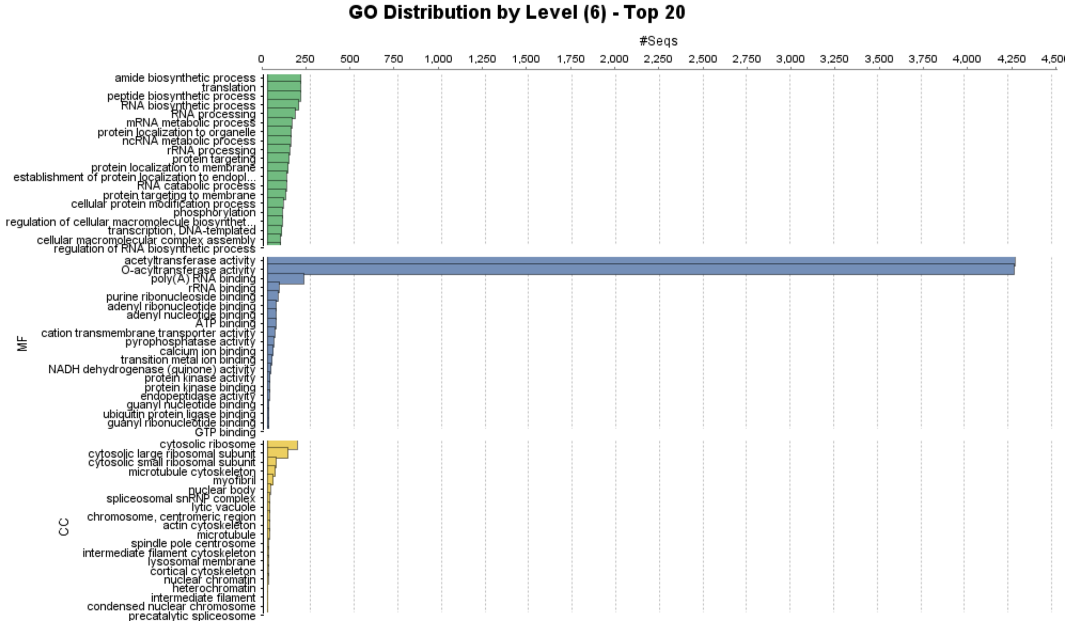

Supplementary figure 1.2: Gene ontology distribution in the Peak 01 (0.92-1.08).

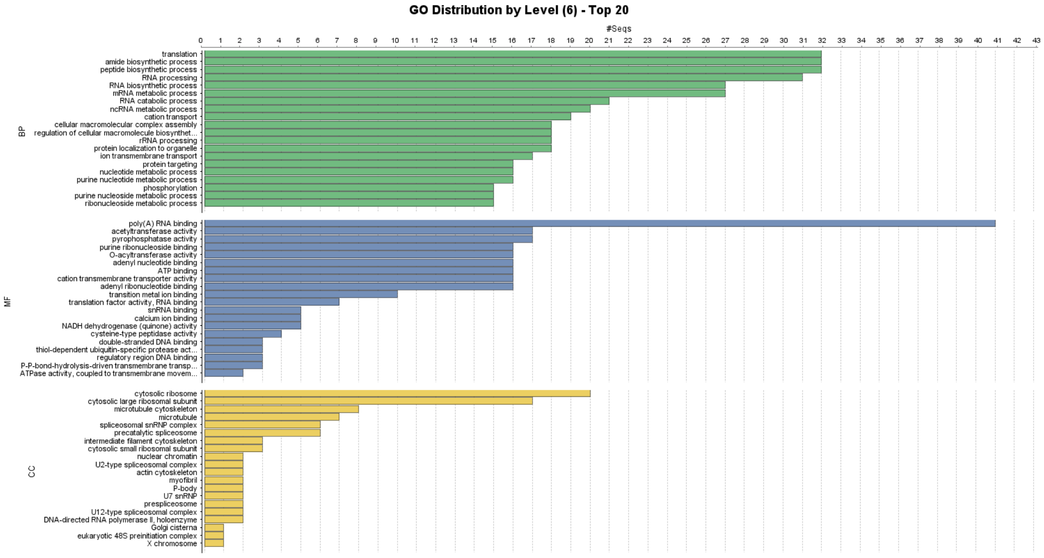

Supplementary figure 1.2: Gene ontology distribution for the peak 02 (1.14-1.22)

Supplementary figure 2: symbiont detection in *Trichoplax adhearens*

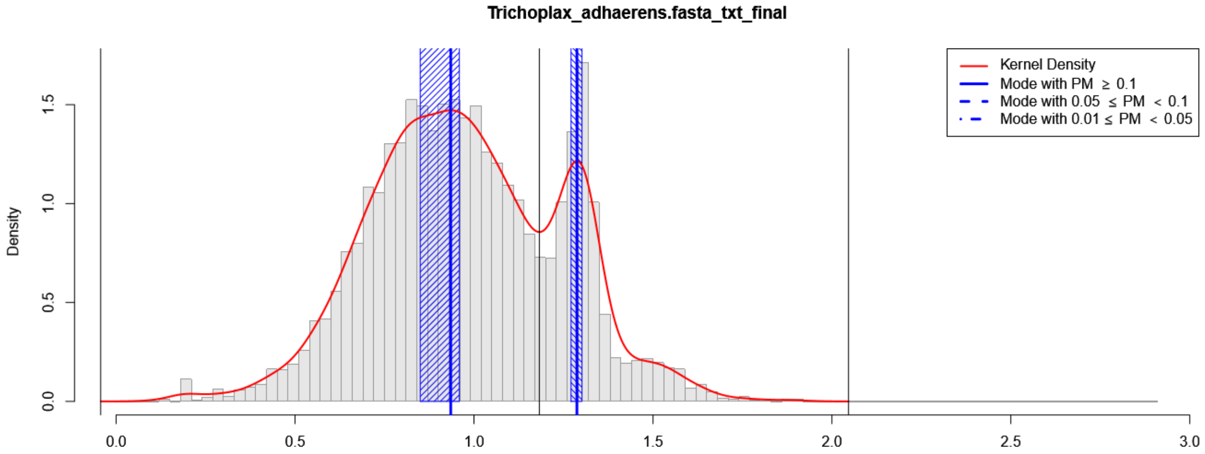

Supplementary figure 2.1: the CpGo/e profile of *Trichoplax adhearens* from dbEST and its additional peak (CpGo/e 1.20 - 1.40)

When we compared the dbEST profile with CDS profile, one additional peak appeared in the dbEST profile (1,609 sequences with a CpGo/e ratio between 1.20 and 1.40). We suggested that sequences present in this peak were not from *Trichoplax adhearens* but were contaminants from intracellular bacteria[2]. In order to verify this hypothesis, we did a gene ontology research in this additional peak. We isolated and extracted DNA sequences from the dbEST fasta files and confirmed our hypothesis.

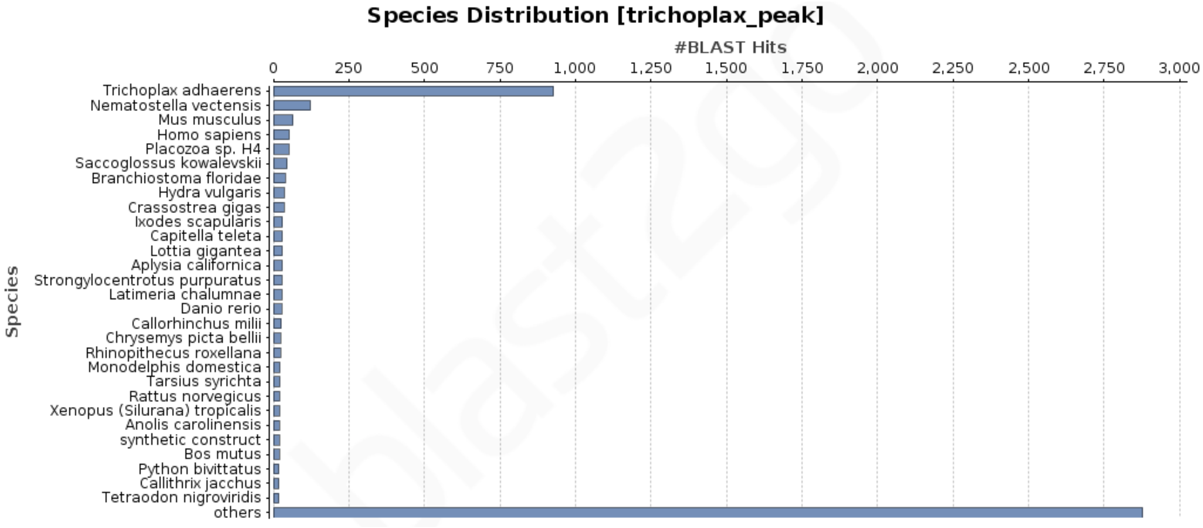

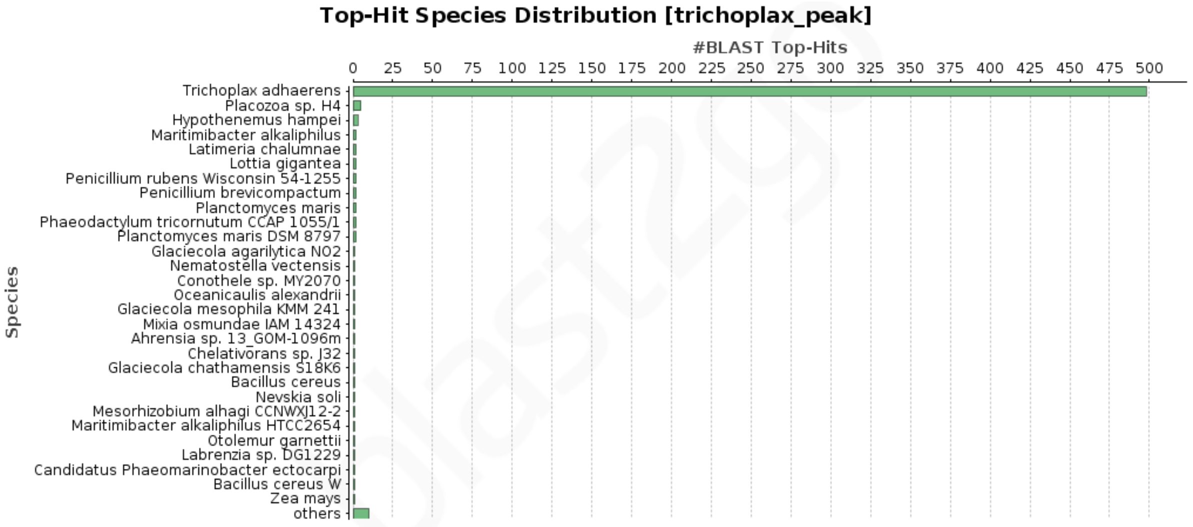

Supplementary figure 2.2: distribution of species distribution in the additional peak in the ESTs of *Trichoplax adhaerens* at a CpGo/e ratio of 1.14 - 1.22

Supplementary figure 3: transcript detection in a *Bombyx mori* ovary library

When we compare the cleanEST library #19823 (*Bombyx mori* overy tissue) profile with the CDS profile, one additional peak was detected with a CpGo/e ratio between 0.40 and 0.60. We suspected the sequences present in this additional peak were contaminants. In order to verify this hypothesis, we isolated and extracted 769 sequences incorporated in this peak and did a gene ontology research with Blast2go[1].

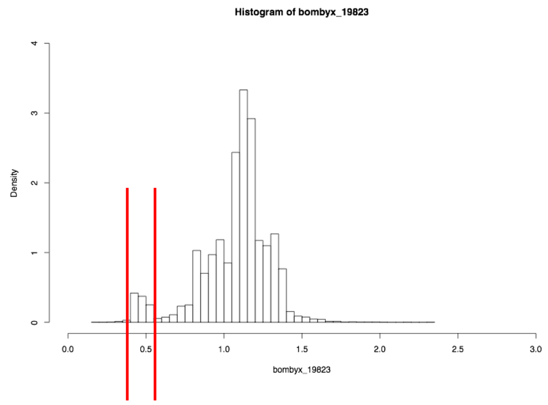

Supplementary figure 3.1: the CpGo/e profile of *Bombyx mori* from cleanEST library n°19823 and its additional peak (included between 0.40 and 0.60)

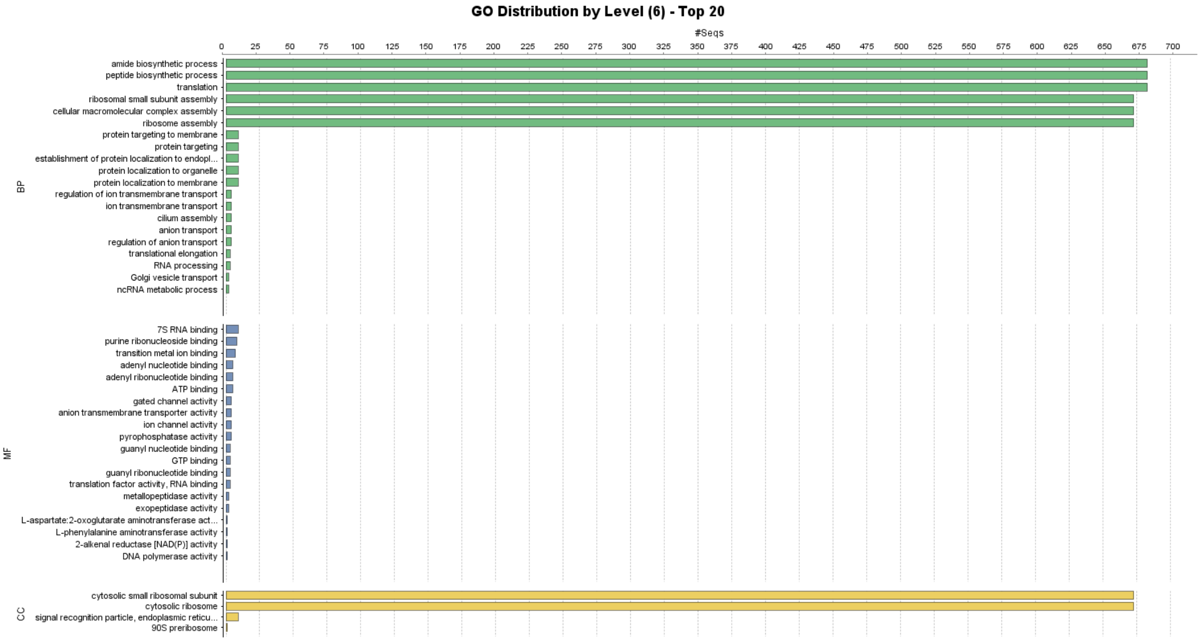

Supplementary figure 3.2: Gene ontology distribution for the additional peak (incorpored between a CpGo/e ratio between 0.40 and 0.60) in *Bombyx mori* ovary library (cleanEST n°19823)

Supplementary figure 4: transcript from a tissue in *Anas platyrhynchos* (spleen)

This dataset is from CleanEST library #17002 and represent 146 sequences. We applied notos to this dataset and obtained a CpGo/e profile. We performed a gene ontology analysis with blast2go[1]. Principal GO term was "antigen binding" suggesting a bias toward immunoglobulins in the EST data set.

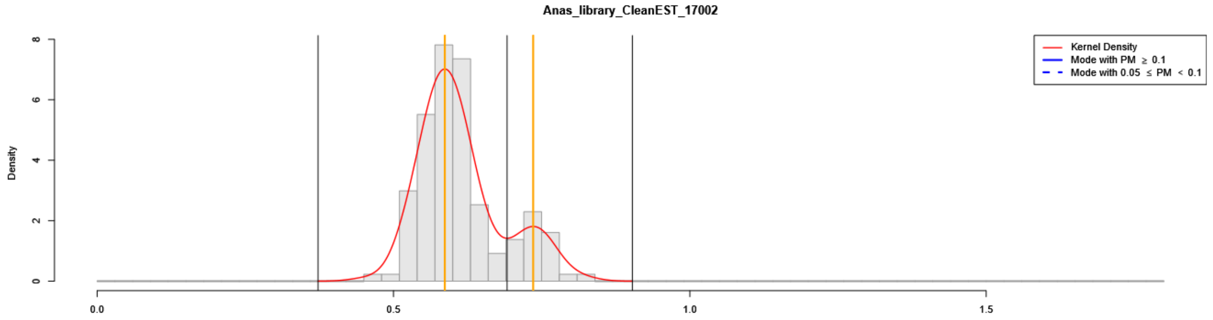

Supplementary figure 4.1: the CpGo/e profile of cleanEST library #17002 (146 sequences) from a tissue in *Anas platyrhynchos* (spleen)

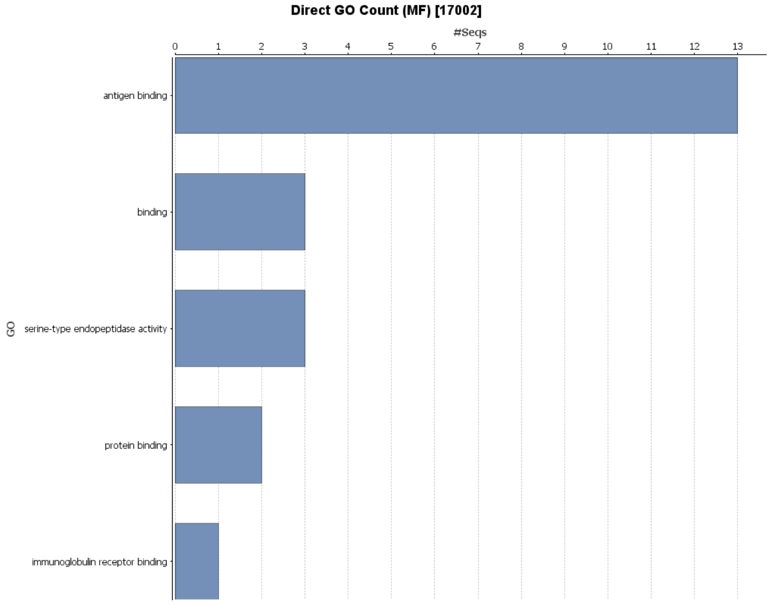

Supplementary figure 4.2: gene ontology of cleanEST library n°17002 (146 sequences) from a tissue in *Anas plastyrhynchos* (spleen)

References

1. Conesa, A. *et al.* Blast2GO: A universal tool for annotation, visualization and analysis in functional genomics research. *Bioinformatics* **21,** 3674–3676 (2005).
2. Driscoll, T., Gillespie, J. J., Nordberg, E. K., Azad, A. F. & Sobral, B. W. Bacterial DNA sifted from the Trichoplax adhaerens (Animalia: Placozoa) genome project reveals a putative rickettsial endosymbiont. *Genome Biol. Evol.* **5,** 621–645 (2013).

Supplementary figure 05: link between CpGo/e and gene expression level: a statistical analysis

When we compared profiles with two peaks (bimodality), we observed differences for three invertebrate and one plant species (*Crassostrea gigas*, *Nasonia vitripennis*, *Nematostella vectensis* and *Oryza sativa*) depending on the origin of the data: cDNA and dbEST/cleanEST. Since in the cDNA data set each gene is represented only once by its genomic coding sequence while in dbEST/cleanest potentially multiple sequence can exist for the same gene, we hypothesized that this could introduce a bias in the dataset by RNA abundance.
To test this hypothesis, we downloaded RNA-seq raw data from European Nucleotide Archive (http://www.ebi.ac.uk/ena/ and/or NCBI, details in supplementary file 3). We filtered the reads with a Phred quality score ≥26. The filtered reads were mapped on their references genomes with RNA STAR[4]. We estimated the FPKM (Fragments Per Kilobase Million) with Cufflinks[5]. We then compared RNA-Seq FPKM for the genes under the two modes. Our results (presented below) show that there are significant differences in FPKM for the two mode positions for *Nasonia vitripennis*, *Crassostrea gigas* and *Oryza sativa*. Genes predicted methylated with *Notos*[1] are more transcribed. This goes in line with earlier observations in other species that gene body methylation is associated with higher transcription[2,3]. We concluded that gene expression difference is probably the origin of the bias in the EST datasets.

Supplementary figure 05. 1: ***Statistical analysis between CpGo/e in CDS extracted from genome and RNA seq expression level in Nematostella vectensis***

cDNA

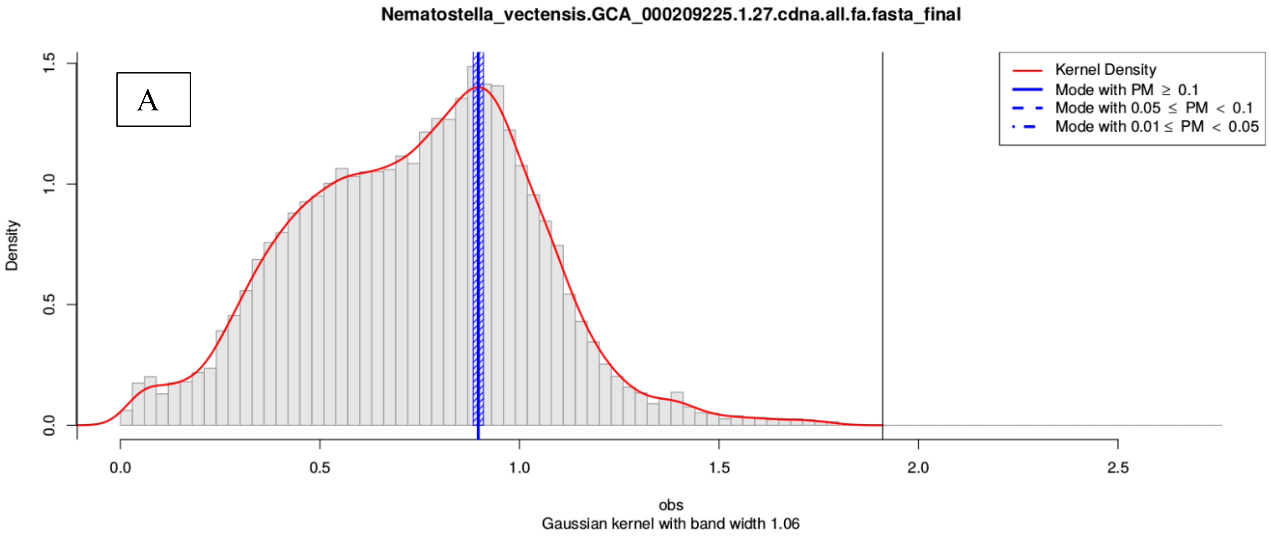

dbEST

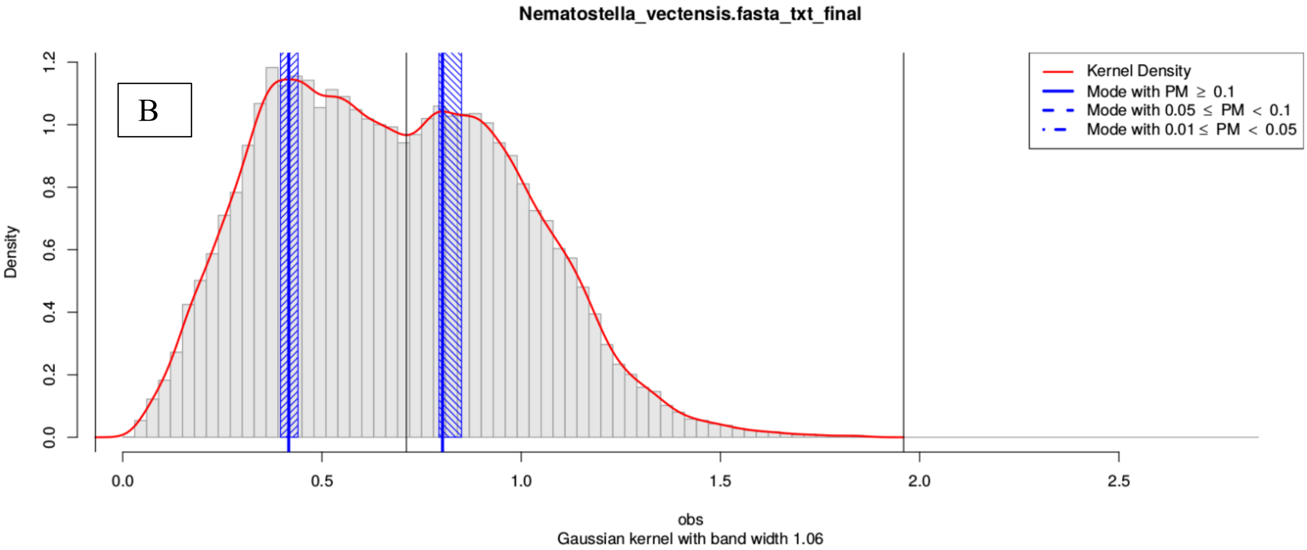

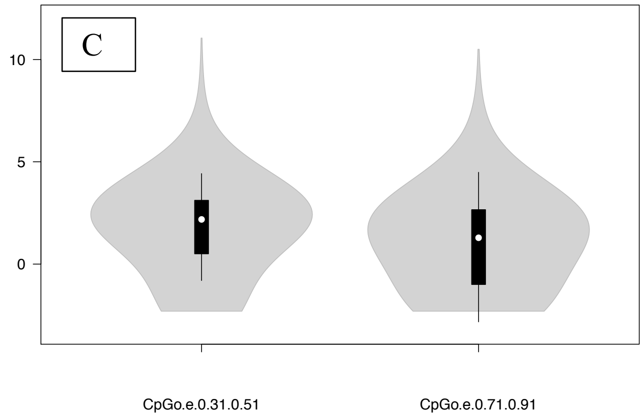

CpGo/e
dbEST peak 1: 0.416
dbEST peak 2: 0.802

cDNA peak: 0.897

CpG o/e 0.31≤ x≤ 0.51: 3,838 genes
CpG o/e 0.71 ≤ x ≤ 0.91: 5,789 genes

RNA-Seq FPKM
median CpG o/e 0.31≤ x ≤ 0.51: 8.78
median CpG o/e 0.71 ≤ x ≤ 0.91: 3.52
Mood's median test: p-value = 0.4677

y axis: log(FPKM)

Supplementary figure 06: RNA-seq analysis *Nasonia vitripennis*

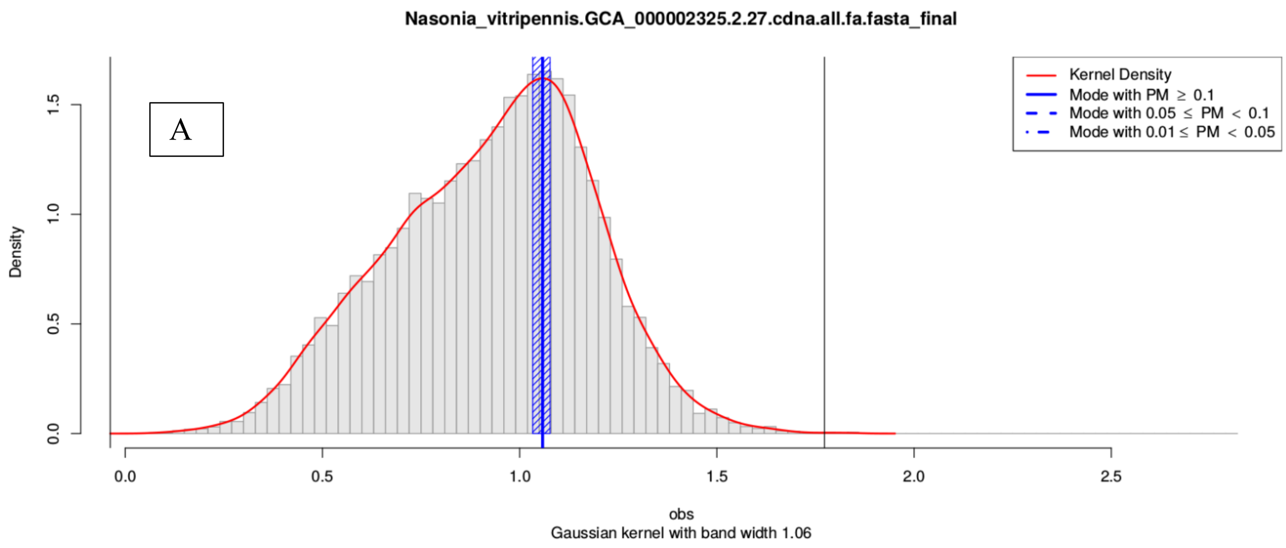

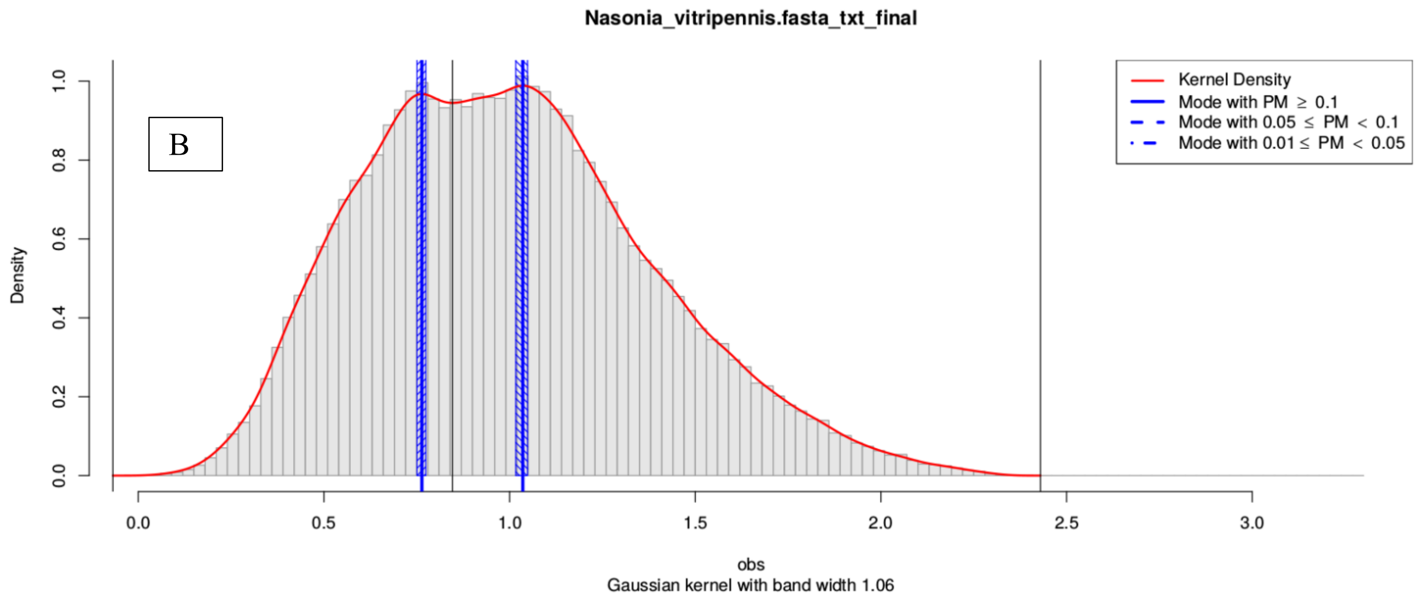

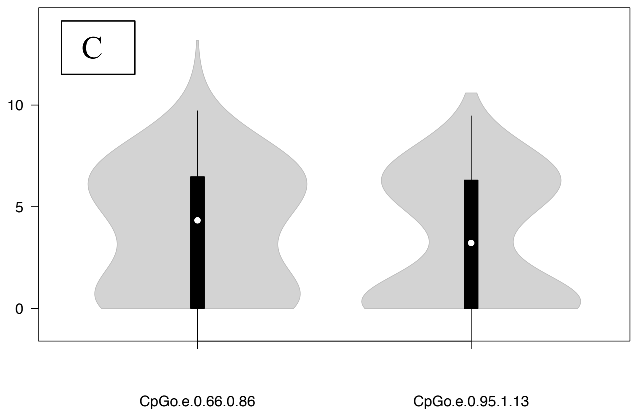

CpGo/e
dbEST peak 1: 0.764
dbEST peak 2: 1.035

cDNA peak: 1.058

CpG o/e $0.66 \leq x \leq 0.86$: 3,571 genes
CpG o/e $0.95 \leq x \leq 1.13$: 4,799 genes

RNA-Seq FPKM
Median CpG o/e $0.66 \leq x \leq 0.86$: 75
Median CpG o/e $0.95 \leq x \leq 1.13$: 24
Mood’s median test: p-value = 1.789e-05

y axis: log(FPKM)

Supplementary figure 07: RNA-seq analysis *Crassostrea gigas*

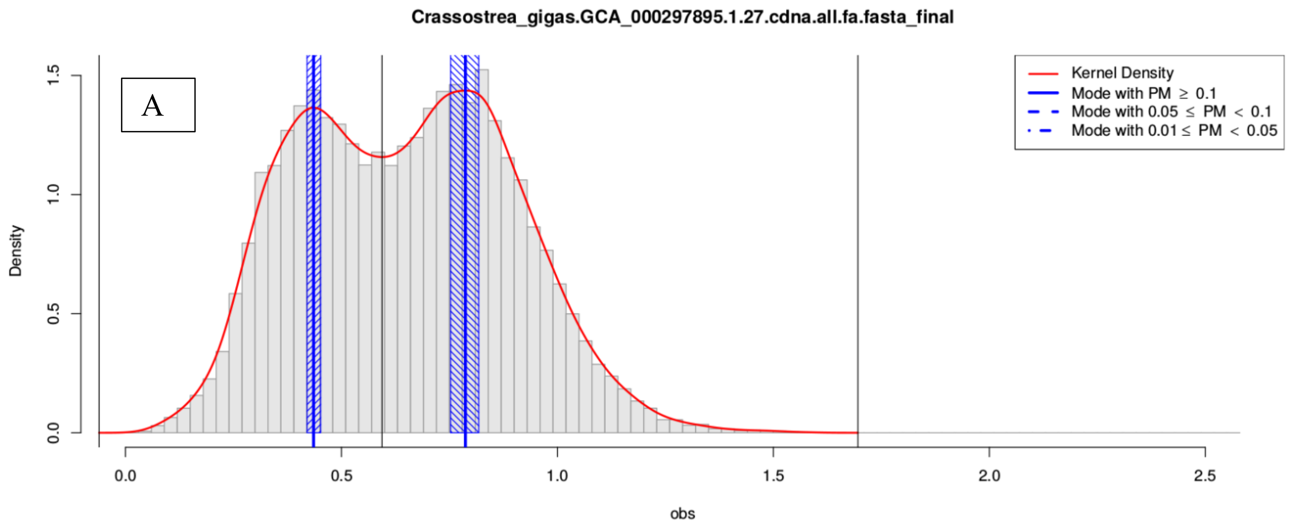

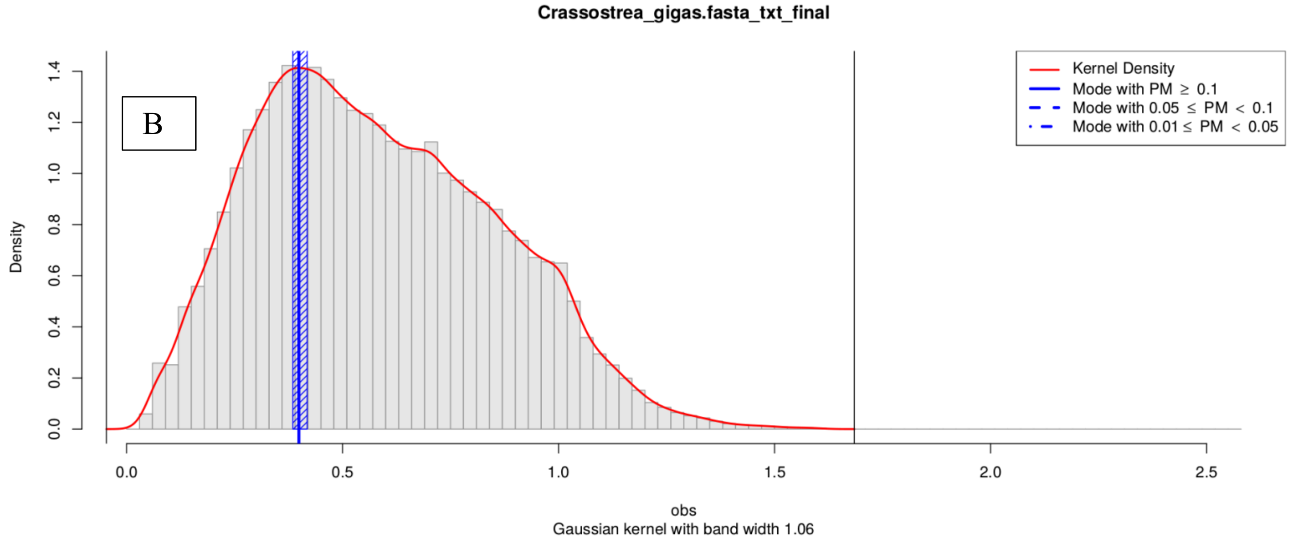

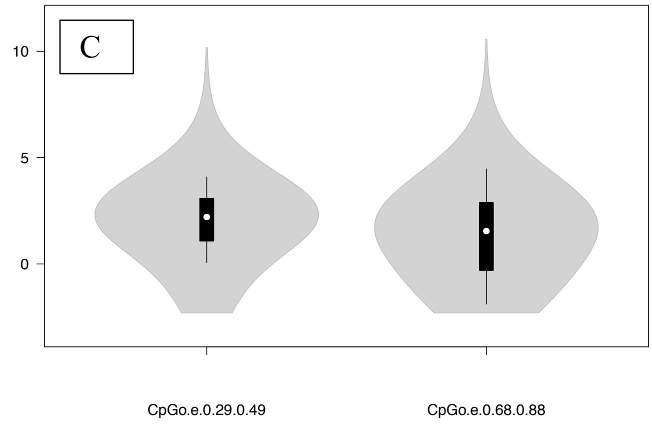

y axis: log(FPKM)

CpGo/e
dbEST peak 1: 0.399

cDNA peak 1: 0.435
cDNA peak 2: 0.787

CpG o/e $0.299 \leq x \leq 0.499$: 6,481 genes
CpG o/e $0.687 \leq x \leq 0.887$: 7,085 genes

RNA-Seq FPKM
median CpG o/e $0.299 \leq x \leq 0.499$: 9.022
median CpG o/e $0.687 \leq x \leq 0.887$: 4.6

Mood’s median test: p-value < 2.2e-16

Supplementary figure 08: RNA-seq analysis *Oryza sativa*

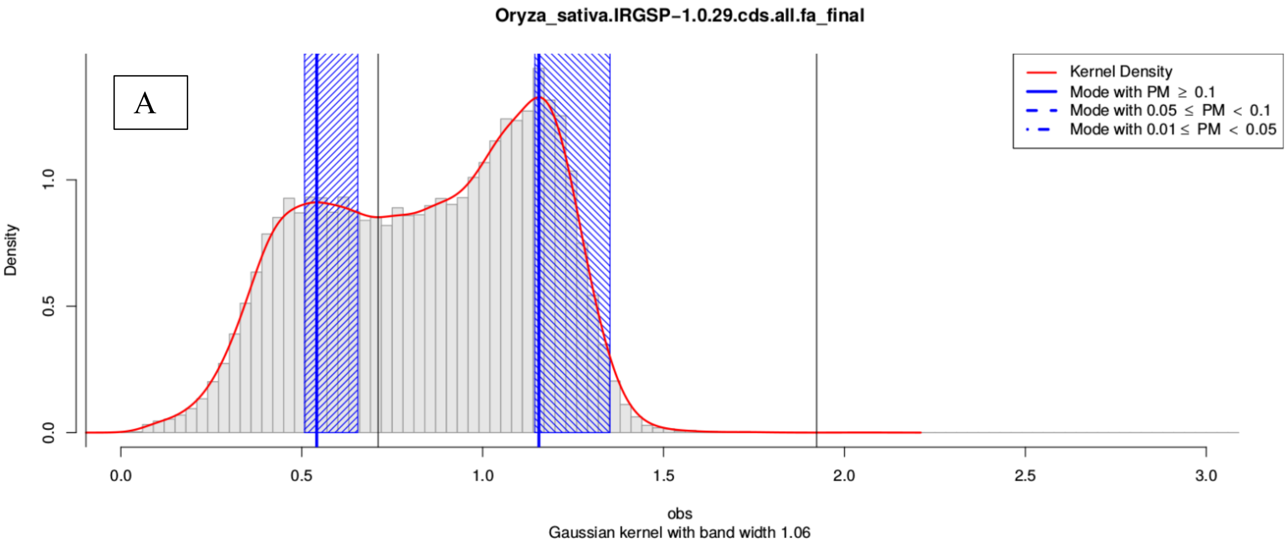

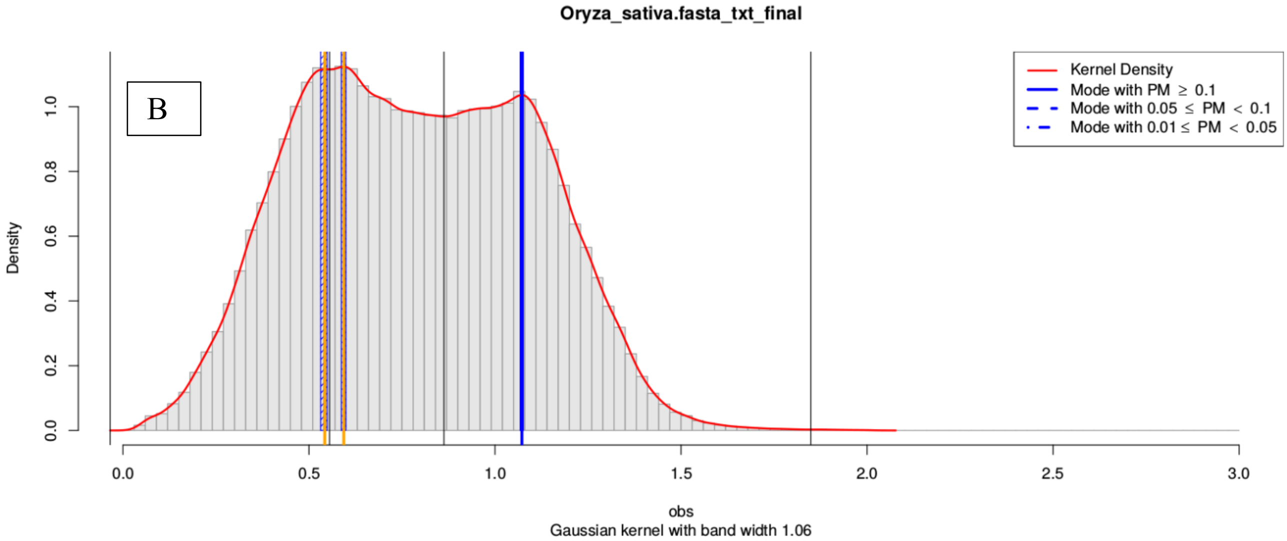

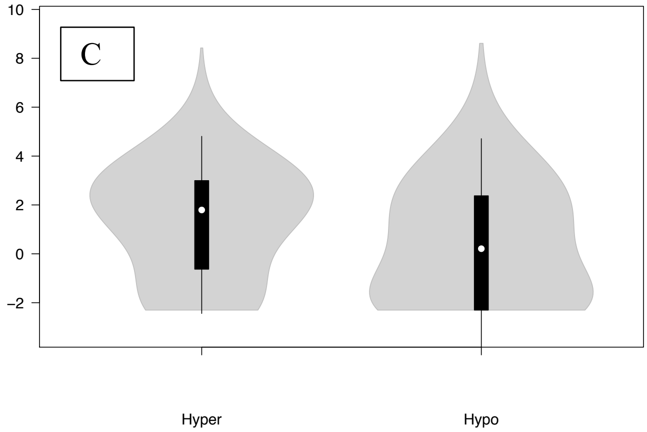

CpGo/e
dbEST peak 1: 0.54
dbEST peak 2: 1.07

cDNA peak 1: 0.541
cDNA peak 2: 1.155

CpG o/e 0.44 ≤ x ≤ 0.64: 6,481 gènes
CpG o/e 0.97 ≤ x ≤ 1.17: 7,085 gènes

RNA-Seq FPKM
CpG o/e 0.44 ≤ x ≤ 0.64: 5.916
CpG o/e 0.97 ≤ x ≤ 1.17: 1.133

Mood's median test: p-value < 2.92e-12

y axis : log(FPKM)

References:

1. Bulla, I. *et al.* Notos - a galaxy tool to analyze CpN observed expected ratios for inferring DNA methylation types. *BMC Bioinformatics* **19,** 105 (2018).
2. Bewick, A. J. & Schmitz, R. J. Gene body DNA methylation in plants. *Curr. Opin. Plant Biol.* **36,** 103–110 (2017).
3. He, X.-J., Chen, T. & Zhu, J.-K. Regulation and function of DNA methylation in plants and animals. *Cell Res.* **21,** 442–465 (2011).
4. Dobin, A. & Gingeras, T. R. Mapping RNA-seq Reads with STAR. in *Current Protocols in Bioinformatics* 11.14.1-11.14.19 (John Wiley & Sons, Inc., 2015). doi:10.1002/0471250953.bi1114s51
5. Trapnell, C. *et al.* Transcript assembly and quantification by RNA-Seq reveals unannotated transcripts and isoform switching during cell differentiation. *Nat. Biotechnol.* **28,** 511–515 (2010).

Supplementary figure 9 : clustering of species according to CpG o/e ratio distributions and methylation types

the numbers given after the species names are number of modes, mode position, absolute Q50 mode skewness, and standard deviation

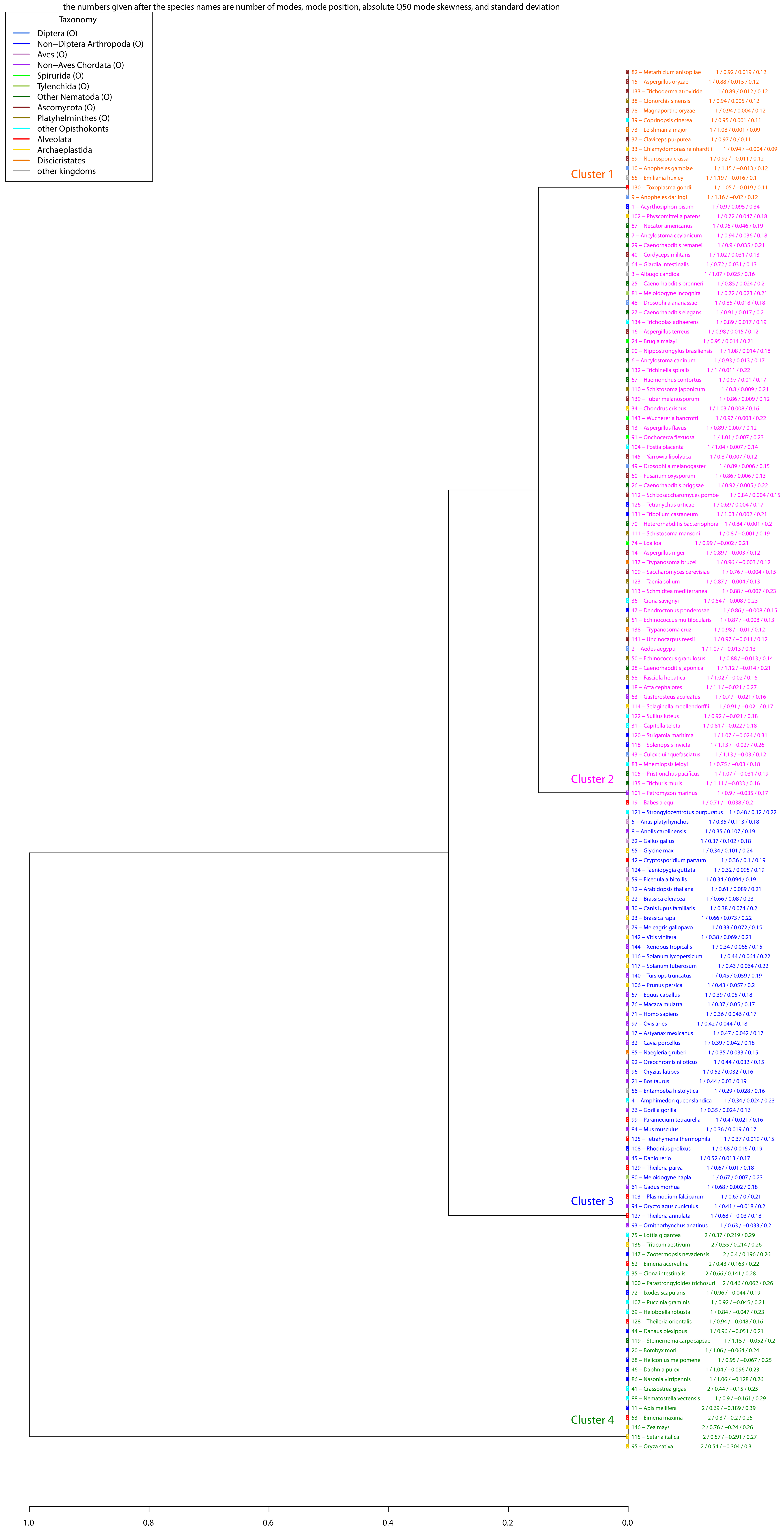

Supplement: Supplementary file 1 — Supplementary figures 1–9 [file 41598_2018_37407_MOESM1_ESM.pdf]
