# Supplementary material for: Universality of the DNA methylation codes in Eucaryotes
Source: Sci Rep. 2019 Jan 17;9:173. doi: 10.1038/s41598-018-37407-8 (PMC6336885; doi:10.1038/s41598-018-37407-8)
Supplement: Supplementary file 3 — Supplementary file 2 [file 41598_2018_37407_MOESM3_ESM.zip › Supplementary_files_02_Histogram_databases_KDE/histogram_kde_cleanEST.pdf]

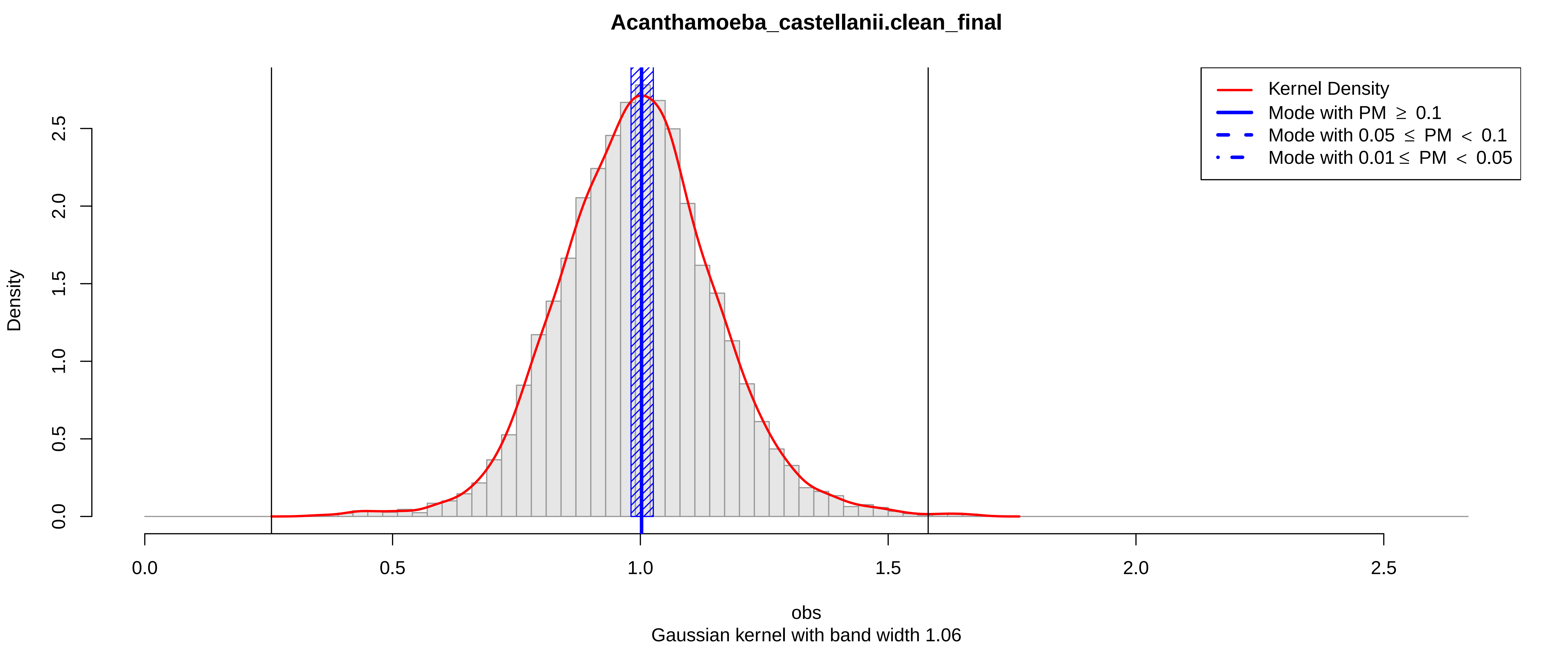

Acanthamoeba_castellanii.clean_final
Kernel Density
Mode with PM ≥ 0.1
Mode with 0.05 ≤ PM < 0.1
Mode with 0.01 ≤ PM < 0.05
Density
0.0
0.5
1.0
1.5
2.0
2.5
obs
Gaussian kernel with band width 1.06

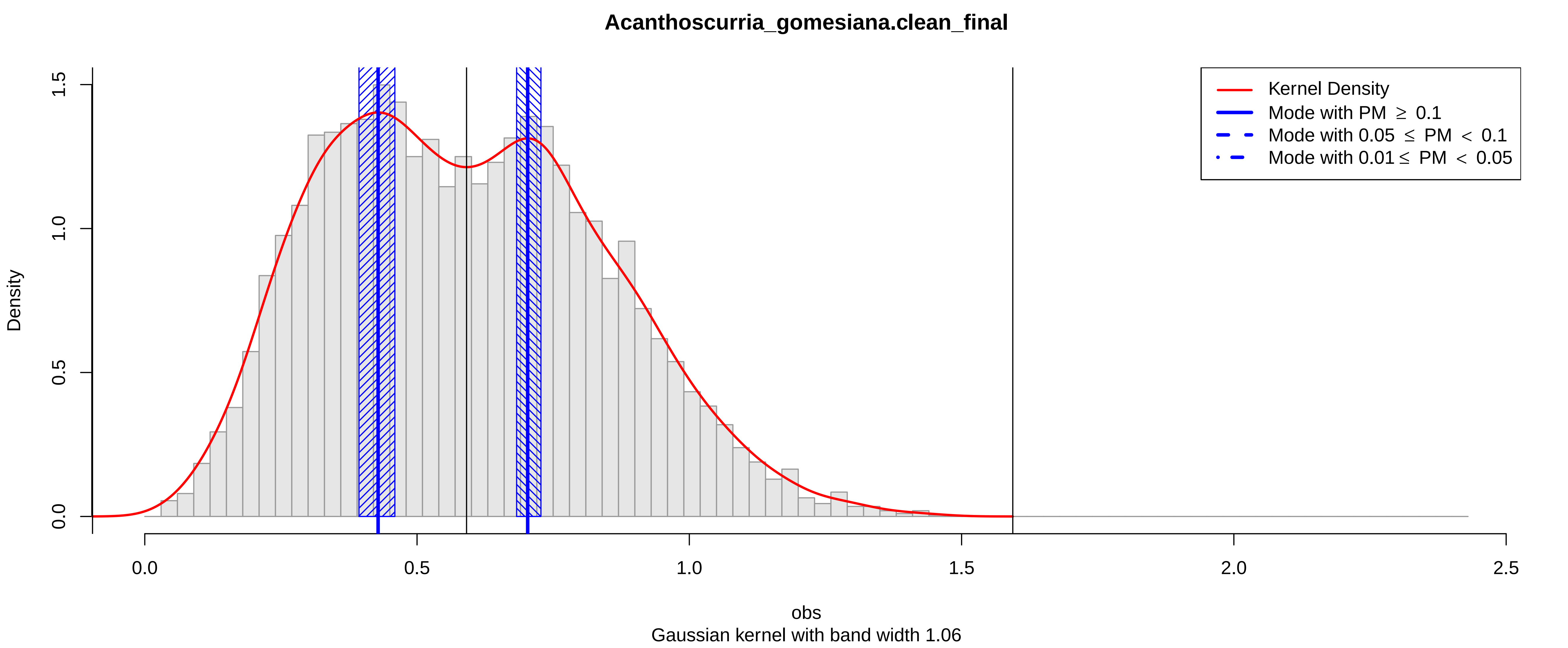

Acanthoscurria_gomesiana.clean_final
Kernel Density
Mode with PM ≥ 0.1
Mode with 0.05 ≤ PM < 0.1
Mode with 0.01 ≤ PM < 0.05
Density
obs
Gaussian kernel with band width 1.06
0.0
0.5
1.0
1.5
2.0
2.5

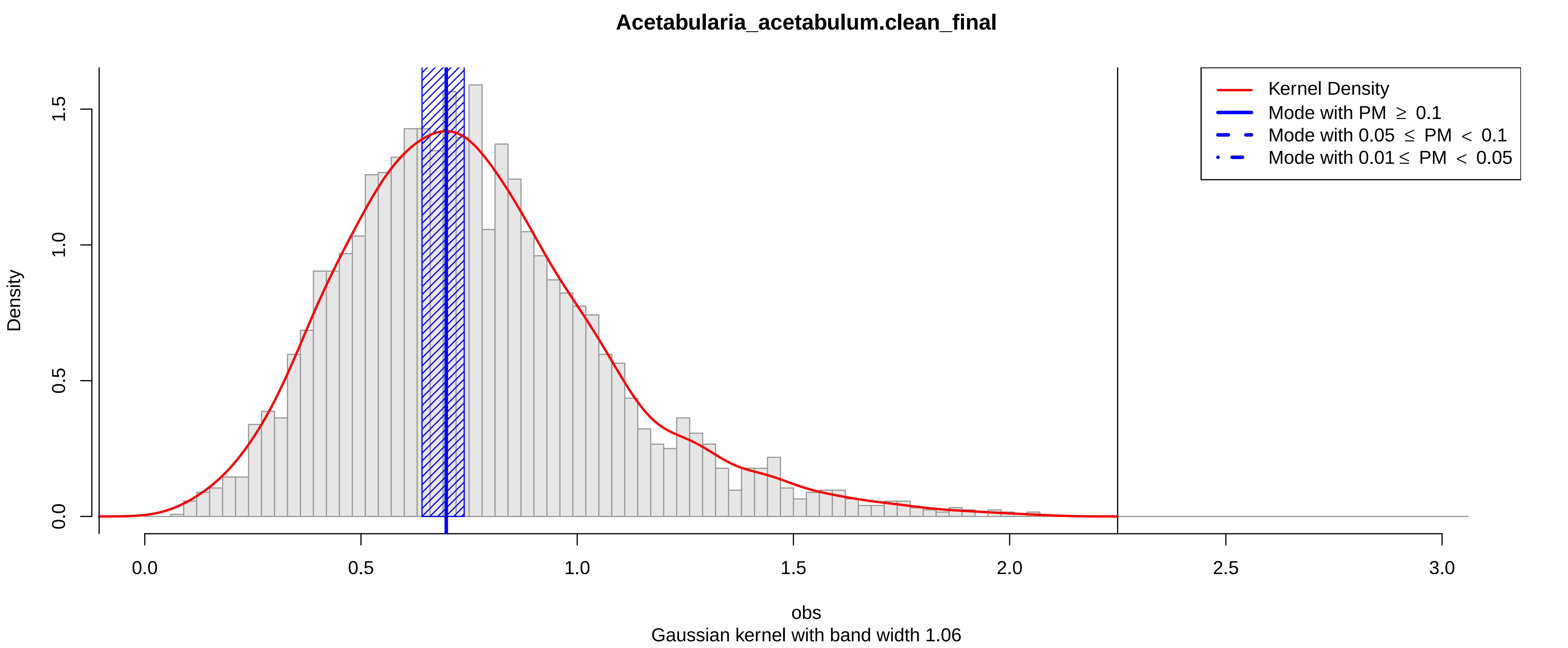

Acetabularia_acetabulum.clean_final
Kernel Density
Mode with PM ≥ 0.1
Mode with 0.05 ≤ PM < 0.1
Mode with 0.01 ≤ PM < 0.05
Density
0.0
0.5
1.0
1.5
2.0
2.5
3.0
obs
Gaussian kernel with band width 1.06

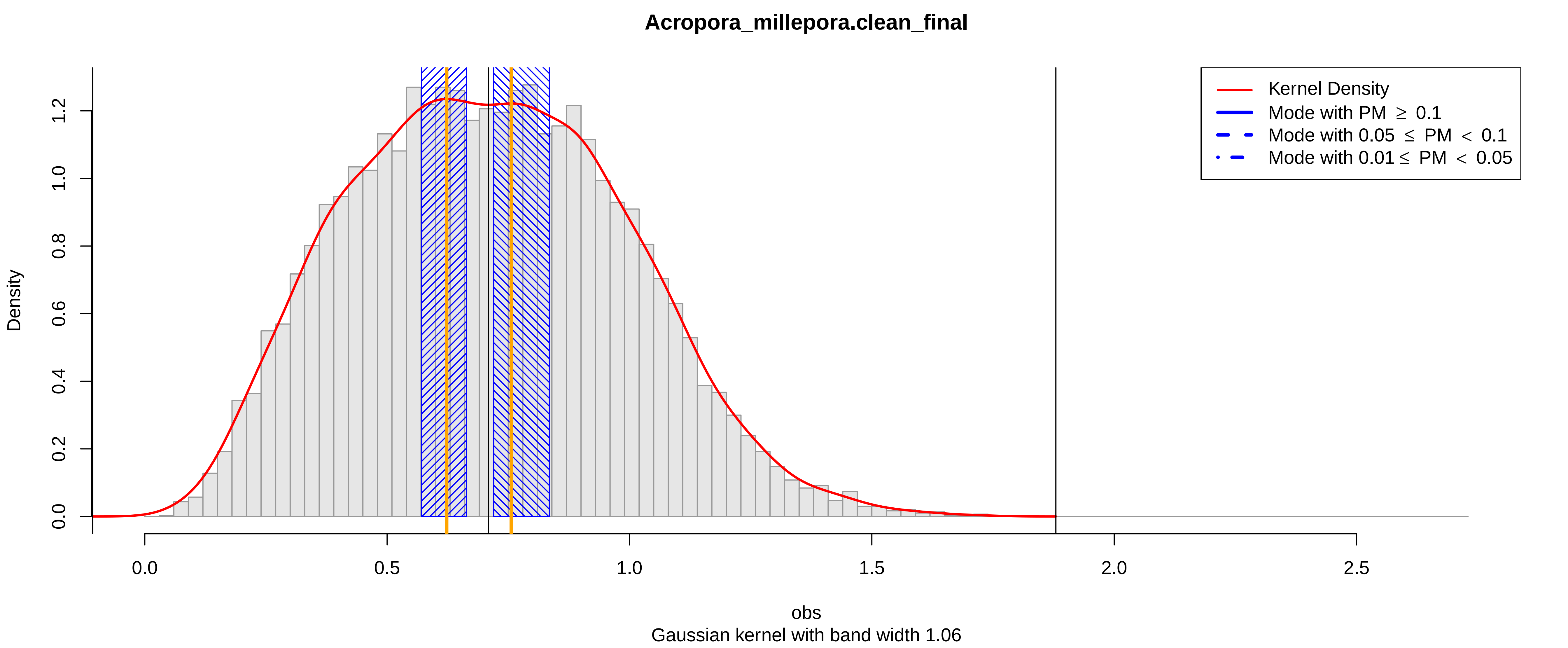

Acropora_millepora.clean_final
Kernel Density
Mode with PM ≥ 0.1
Mode with 0.05 ≤ PM < 0.1
Mode with 0.01 ≤ PM < 0.05
Density
0.0
0.2
0.4
0.6
0.8
1.0
1.2
0.0
0.5
1.0
1.5
2.0
2.5
obs
Gaussian kernel with band width 1.06

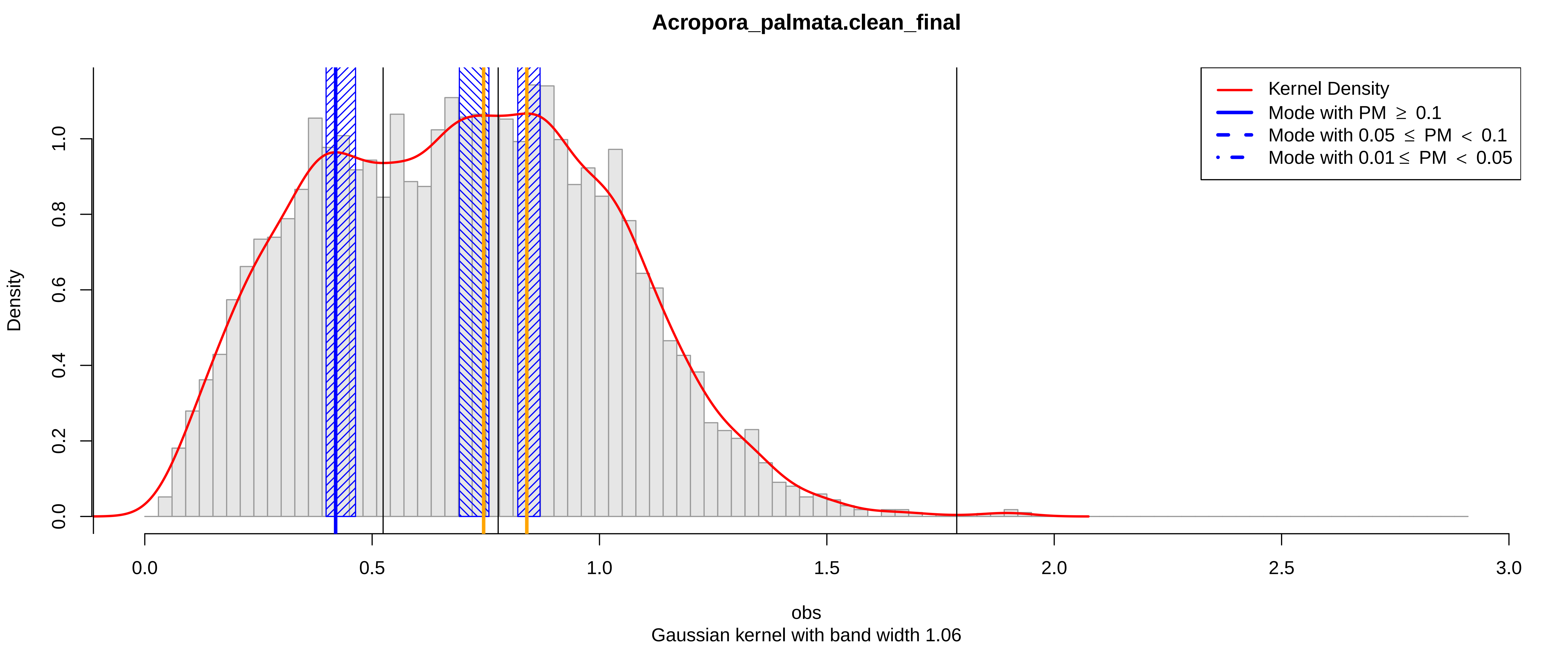

Acropora_palmata.clean_final
Kernel Density
Mode with PM ≥ 0.1
Mode with 0.05 ≤ PM < 0.1
Mode with 0.01 ≤ PM < 0.05
Density
0.0
0.2
0.4
0.6
0.8
1.0
0.0
0.5
1.0
1.5
2.0
2.5
3.0
obs
Gaussian kernel with band width 1.06

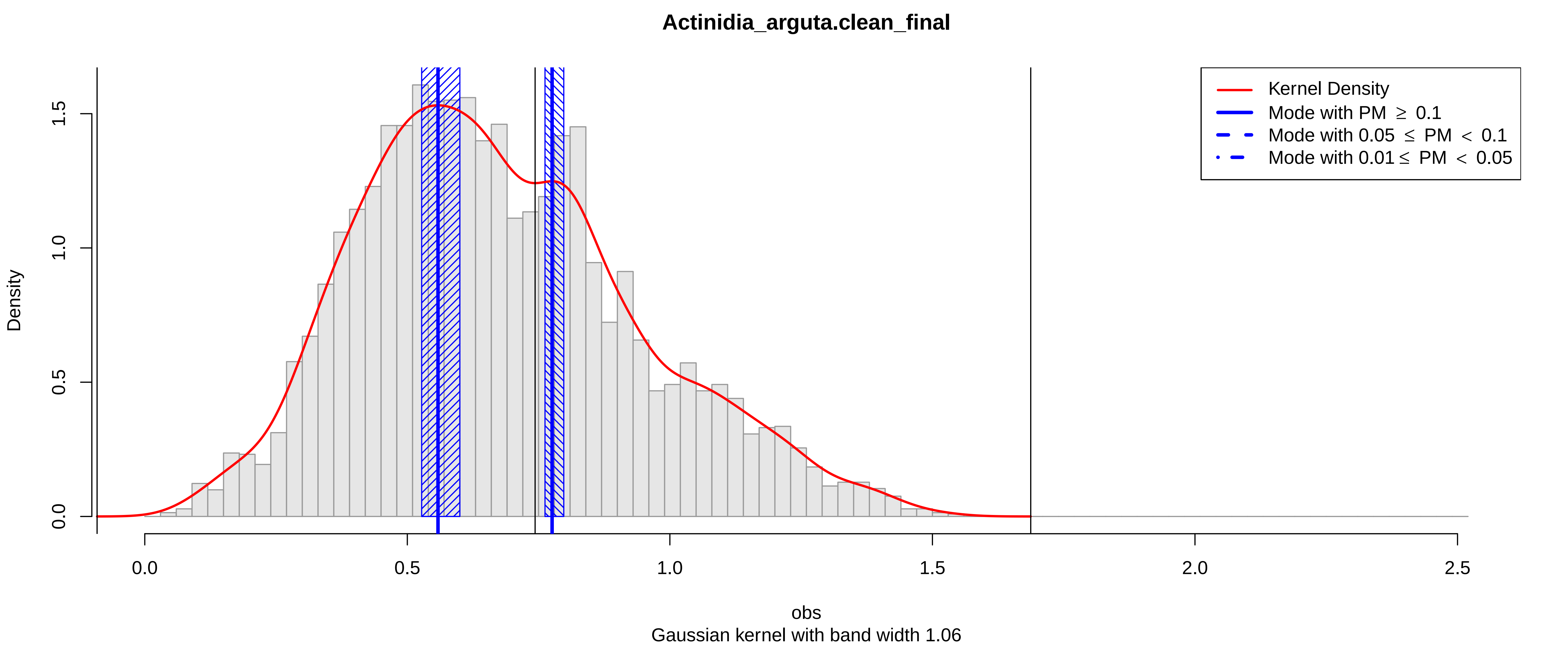

Actinidia_arguta.clean_final
Kernel Density
Mode with PM ≥ 0.1
Mode with 0.05 ≤ PM < 0.1
Mode with 0.01 ≤ PM < 0.05
Density
0.0
0.5
1.0
1.5
2.0
2.5
obs
Gaussian kernel with band width 1.06

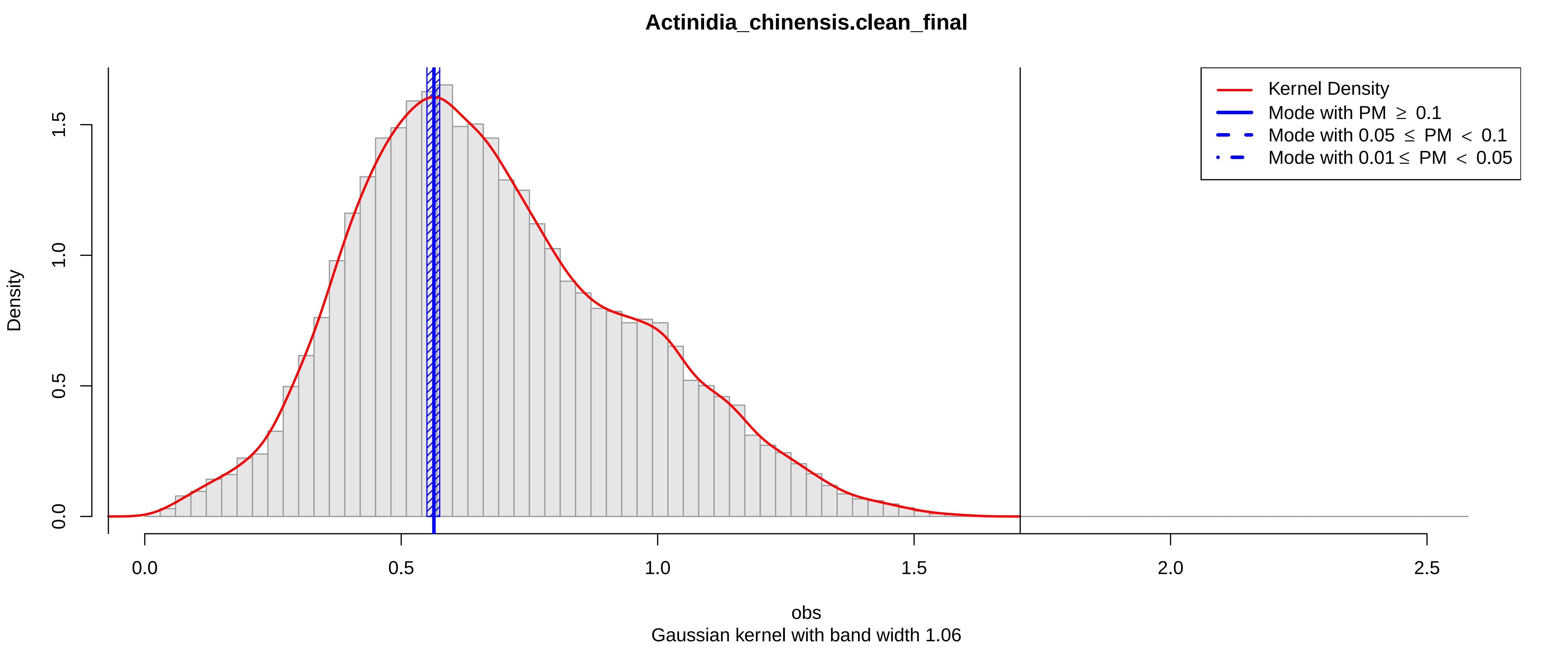
Actinidia_chinensis.clean_final
Kernel Density
Mode with PM ≥ 0.1
Mode with 0.05 ≤ PM < 0.1
Mode with 0.01 ≤ PM < 0.05
Density
0.0
0.5
1.0
1.5
2.0
2.5
obs
Gaussian kernel with band width 1.06

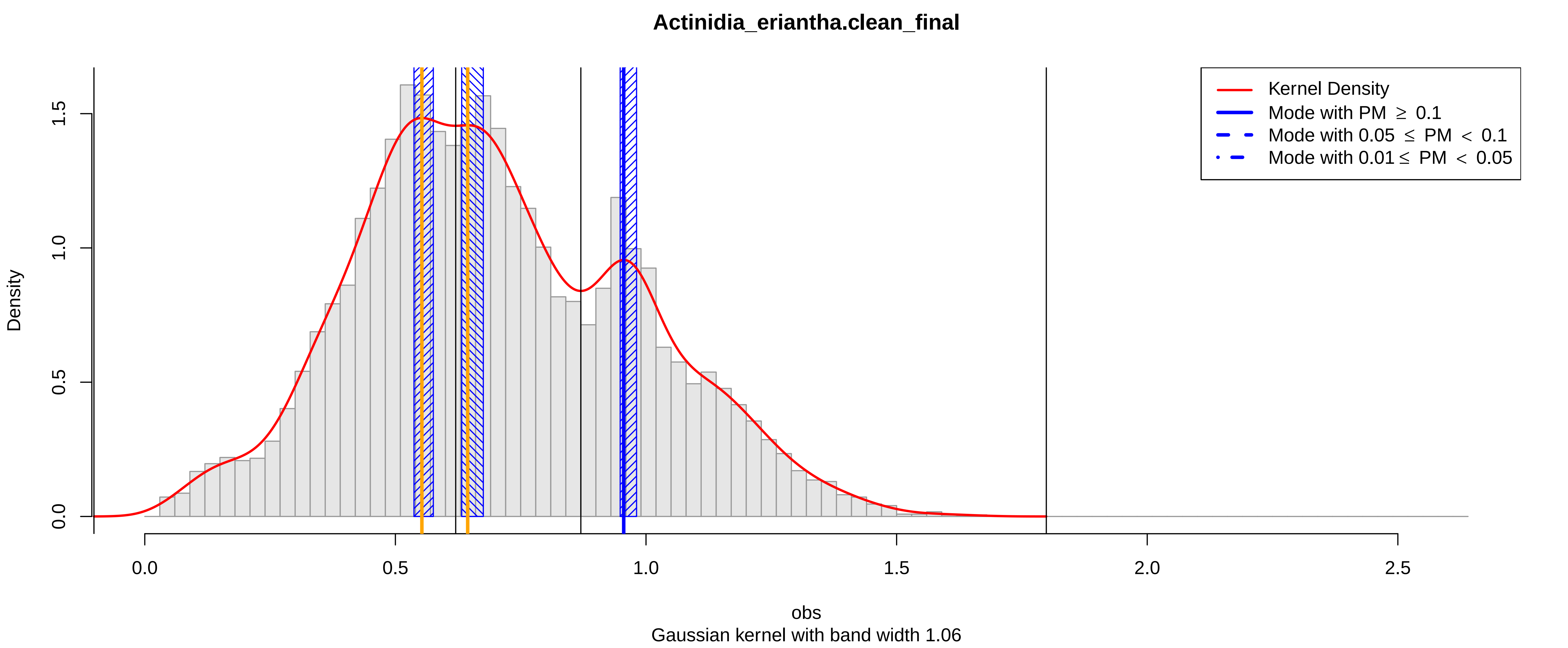
Actinidia_eriantha.clean_final
Kernel Density
Mode with PM ≥ 0.1
Mode with 0.05 ≤ PM < 0.1
Mode with 0.01 ≤ PM < 0.05
Density
0.0
0.5
1.0
1.5
2.0
2.5
obs
Gaussian kernel with band width 1.06

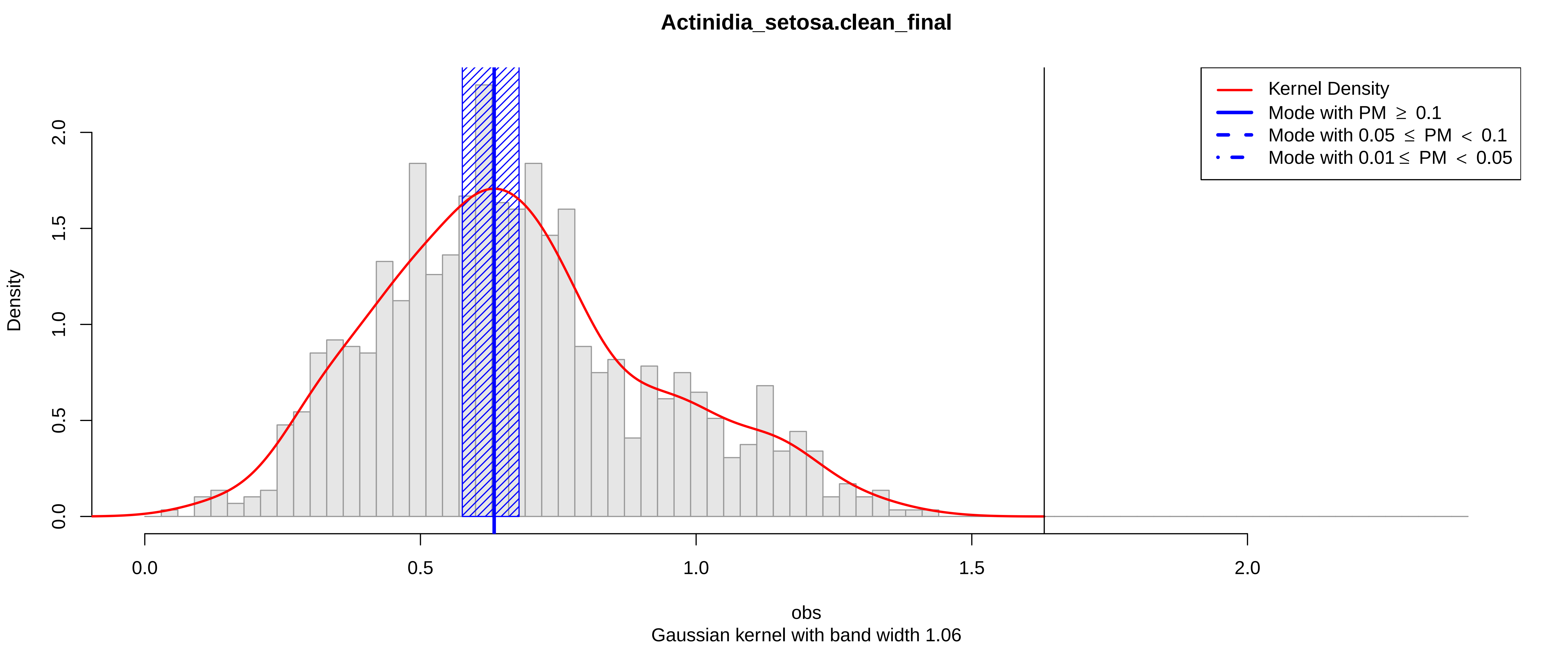
Actinidia_setosa.clean_final
Kernel Density
Mode with PM ≥ 0.1
Mode with 0.05 ≤ PM < 0.1
Mode with 0.01 ≤ PM < 0.05
Density
0.0
0.5
1.0
1.5
2.0
0.0
0.5
1.0
1.5
2.0
obs
Gaussian kernel with band width 1.06

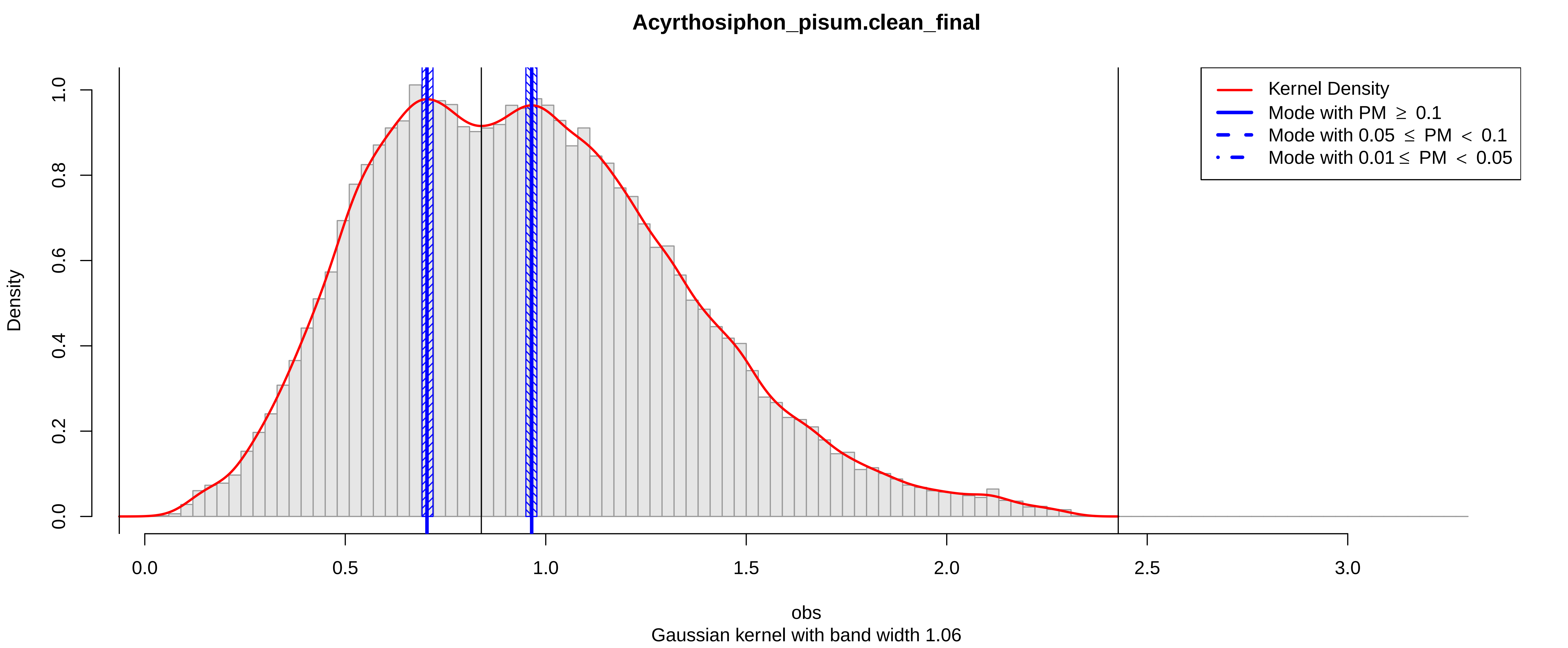

Acyrthosiphon_pisum.clean_final
Kernel Density
Mode with PM ≥ 0.1
Mode with 0.05 ≤ PM < 0.1
Mode with 0.01 ≤ PM < 0.05
Density
0.0
0.2
0.4
0.6
0.8
1.0
0.0
0.5
1.0
1.5
2.0
2.5
3.0
obs
Gaussian kernel with band width 1.06

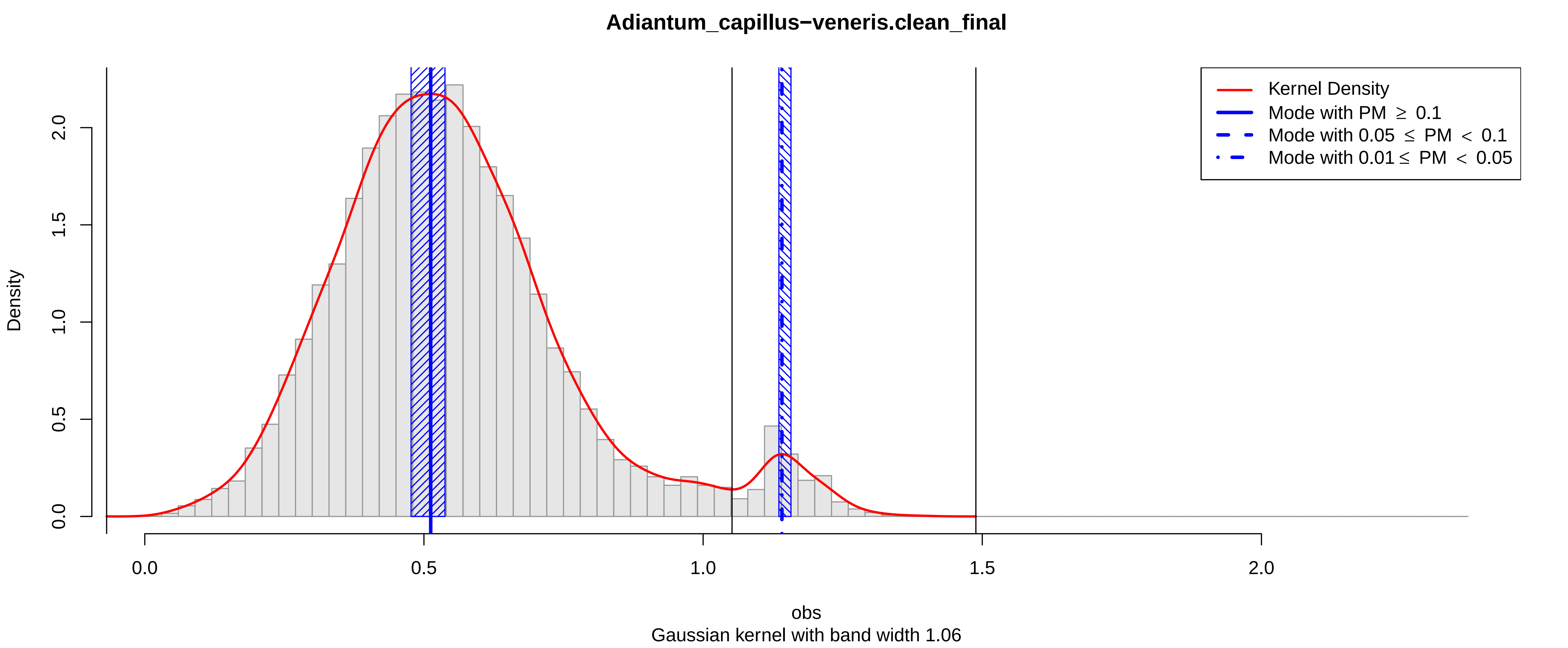

Adiantum_capillus−veneris.clean_final
Kernel Density
Mode with PM ≥ 0.1
Mode with 0.05 ≤ PM < 0.1
Mode with 0.01 ≤ PM < 0.05
Density
0.0
0.5
1.0
1.5
2.0
0.0
0.5
1.0
1.5
2.0
obs
Gaussian kernel with band width 1.06

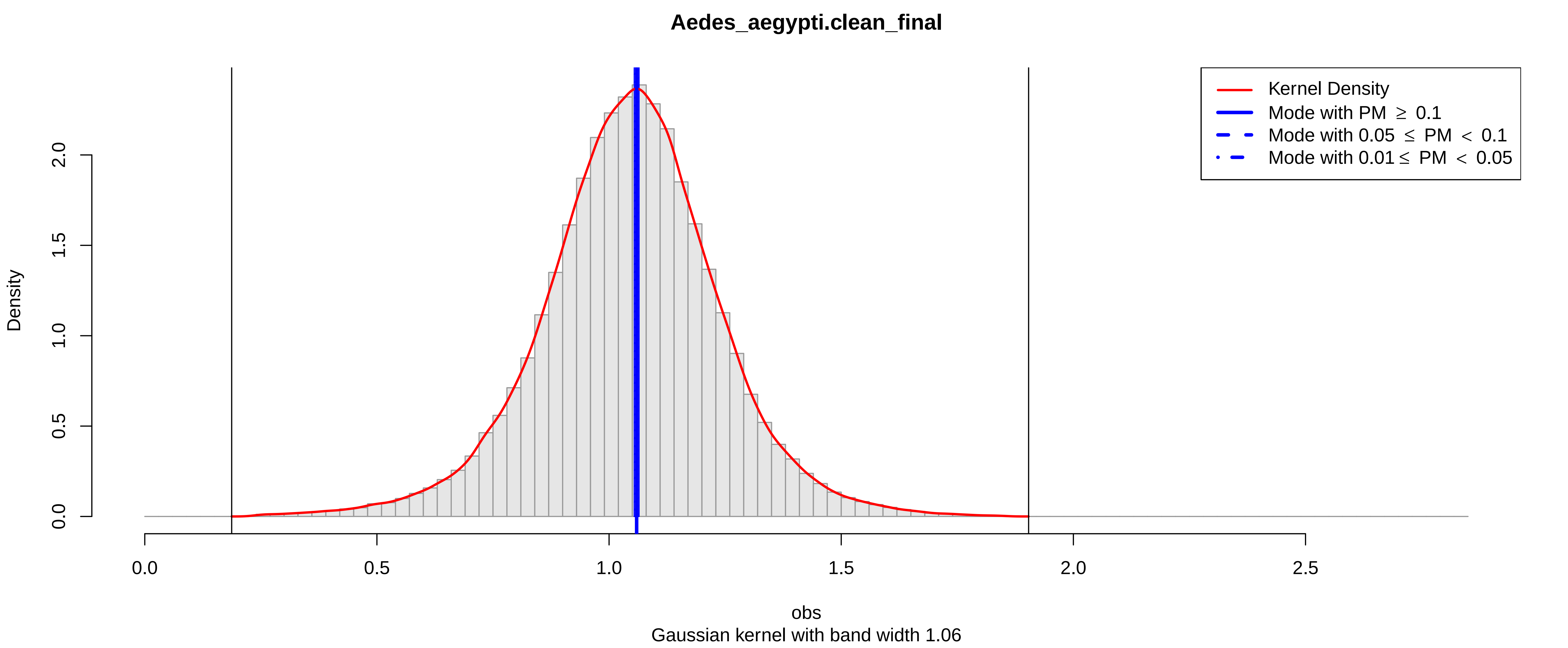
Aedes_aegypti.clean_final
Kernel Density
Mode with PM ≥ 0.1
Mode with 0.05 ≤ PM < 0.1
Mode with 0.01 ≤ PM < 0.05
Density
0.0
0.5
1.0
1.5
2.0
0.0
0.5
1.0
1.5
2.0
2.5
obs
Gaussian kernel with band width 1.06

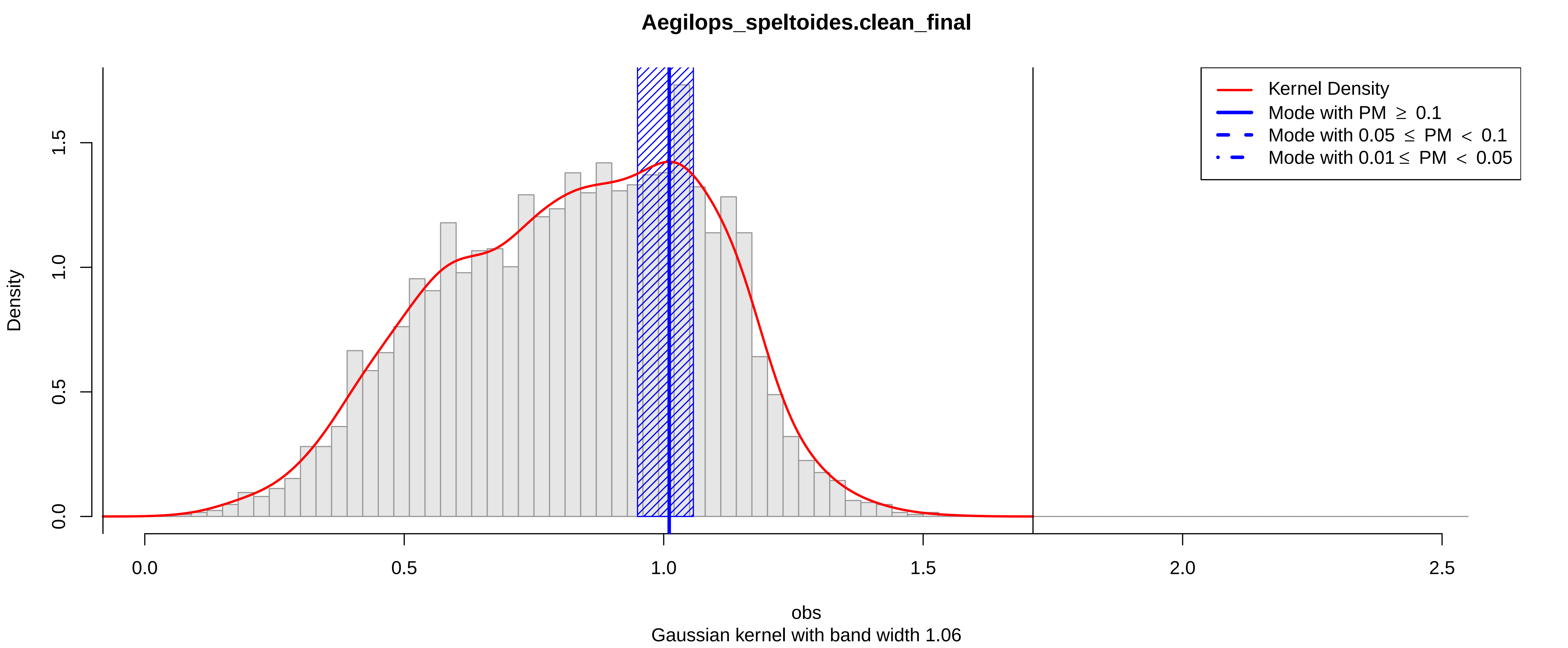

Aegilops_speltoides.clean_final
Kernel Density
Mode with PM ≥ 0.1
Mode with 0.05 ≤ PM < 0.1
Mode with 0.01 ≤ PM < 0.05
Density
0.0
0.5
1.0
1.5
0.0
0.5
1.0
1.5
2.0
2.5
obs
Gaussian kernel with band width 1.06

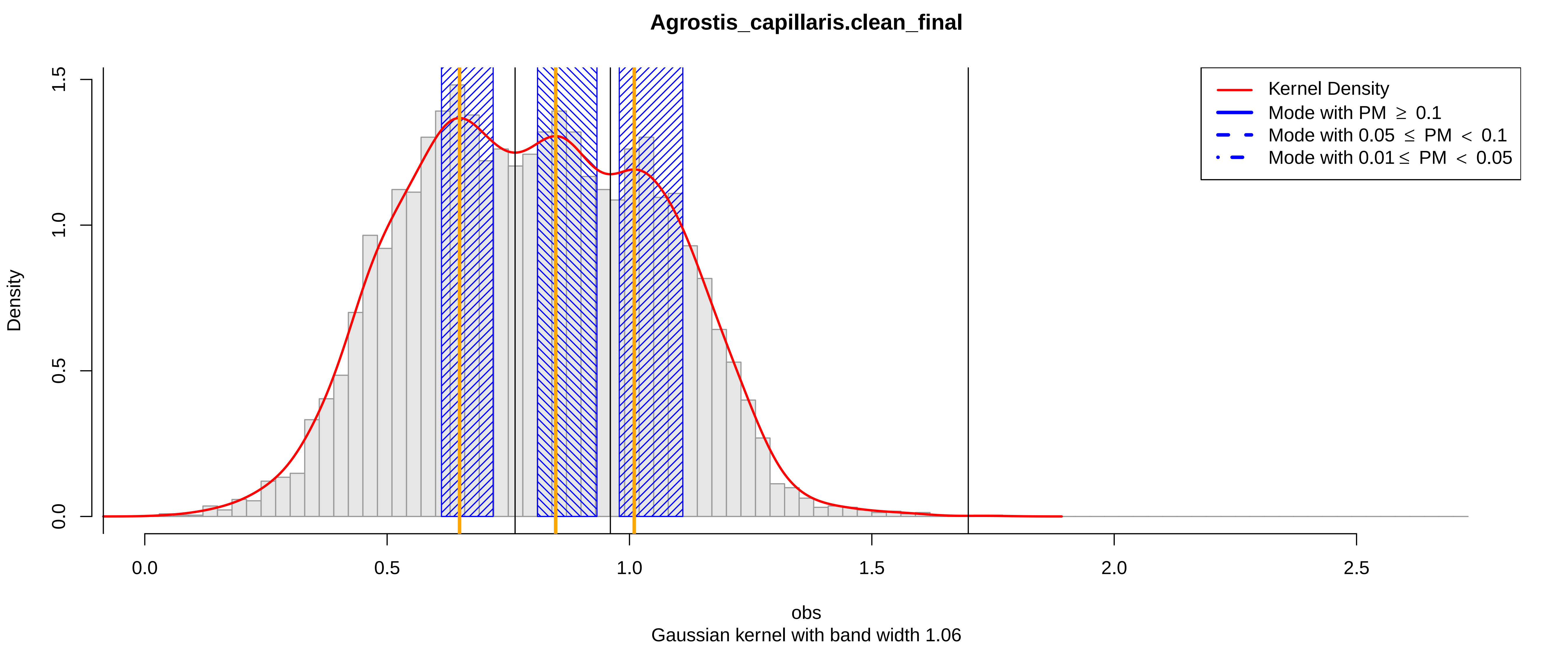
Agrostis_capillaris.clean_final
Kernel Density
Mode with PM ≥ 0.1
Mode with 0.05 ≤ PM < 0.1
Mode with 0.01 ≤ PM < 0.05
Density
0.0
0.5
1.0
1.5
0.0
0.5
1.0
1.5
2.0
2.5
obs
Gaussian kernel with band width 1.06

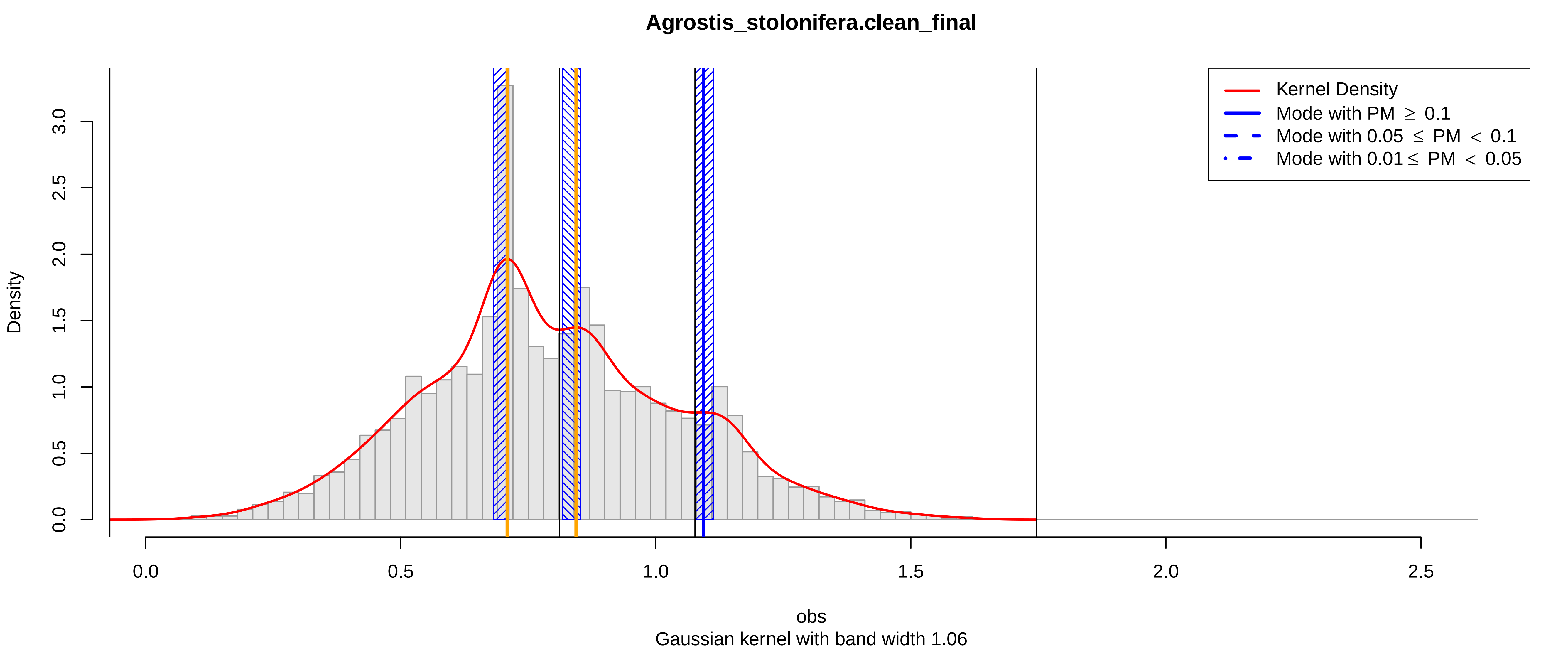

Agrostis_stolonifera.clean_final
Kernel Density
Mode with PM ≥ 0.1
Mode with 0.05 ≤ PM < 0.1
Mode with 0.01 ≤ PM < 0.05
Density
0.0
0.5
1.0
1.5
2.0
2.5
3.0
obs
Gaussian kernel with band width 1.06

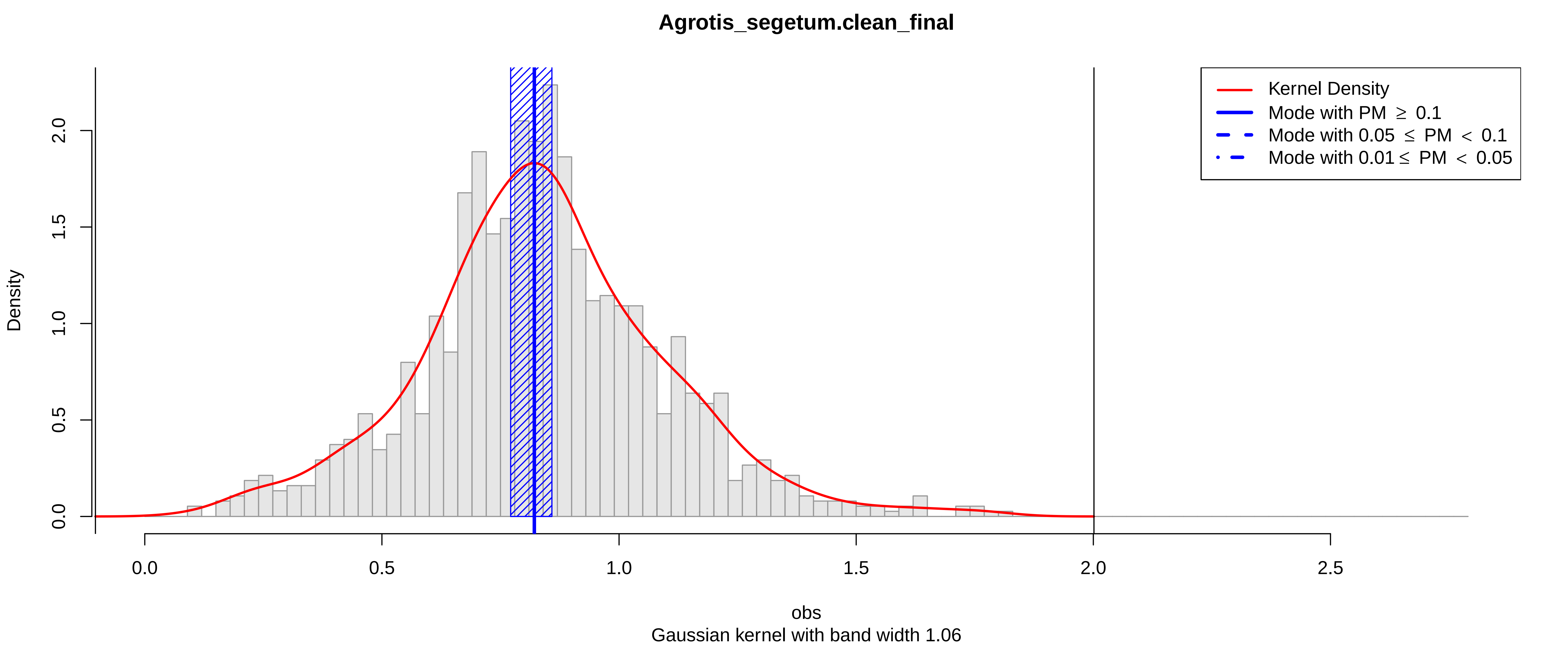

Agrotis_segetum.clean_final
Kernel Density
Mode with PM ≥ 0.1
Mode with 0.05 ≤ PM < 0.1
Mode with 0.01 ≤ PM < 0.05
Density
0.0
0.5
1.0
1.5
2.0
2.5
obs
Gaussian kernel with band width 1.06

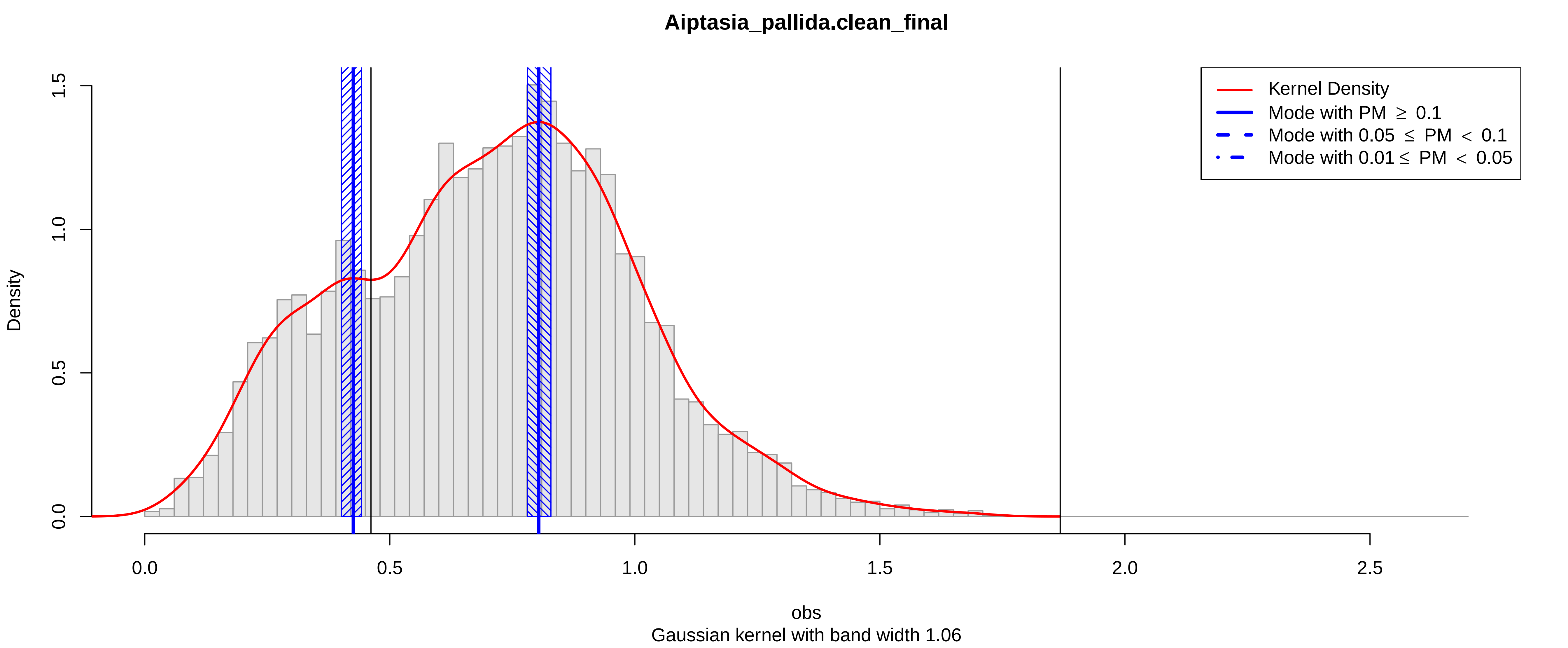

Aiptasia_pallida.clean_final
Kernel Density
Mode with PM ≥ 0.1
Mode with 0.05 ≤ PM < 0.1
Mode with 0.01 ≤ PM < 0.05
Density
0.0
0.5
1.0
1.5
2.0
2.5
obs
Gaussian kernel with band width 1.06

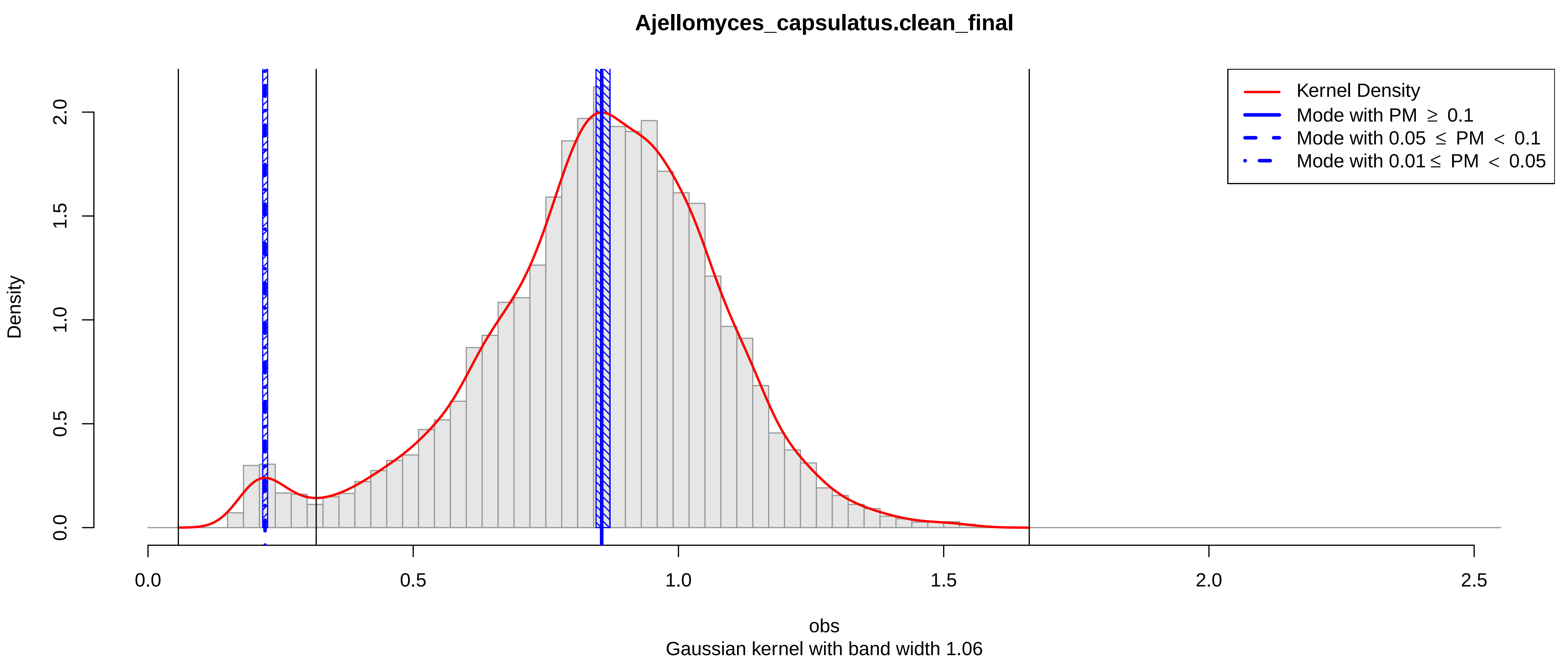
Ajellomyces_capsulatus.clean_final
Kernel Density
Mode with PM ≥ 0.1
Mode with 0.05 ≤ PM < 0.1
Mode with 0.01 ≤ PM < 0.05
Density
0.0
0.5
1.0
1.5
2.0
0.0
0.5
1.0
1.5
2.0
2.5
obs
Gaussian kernel with band width 1.06

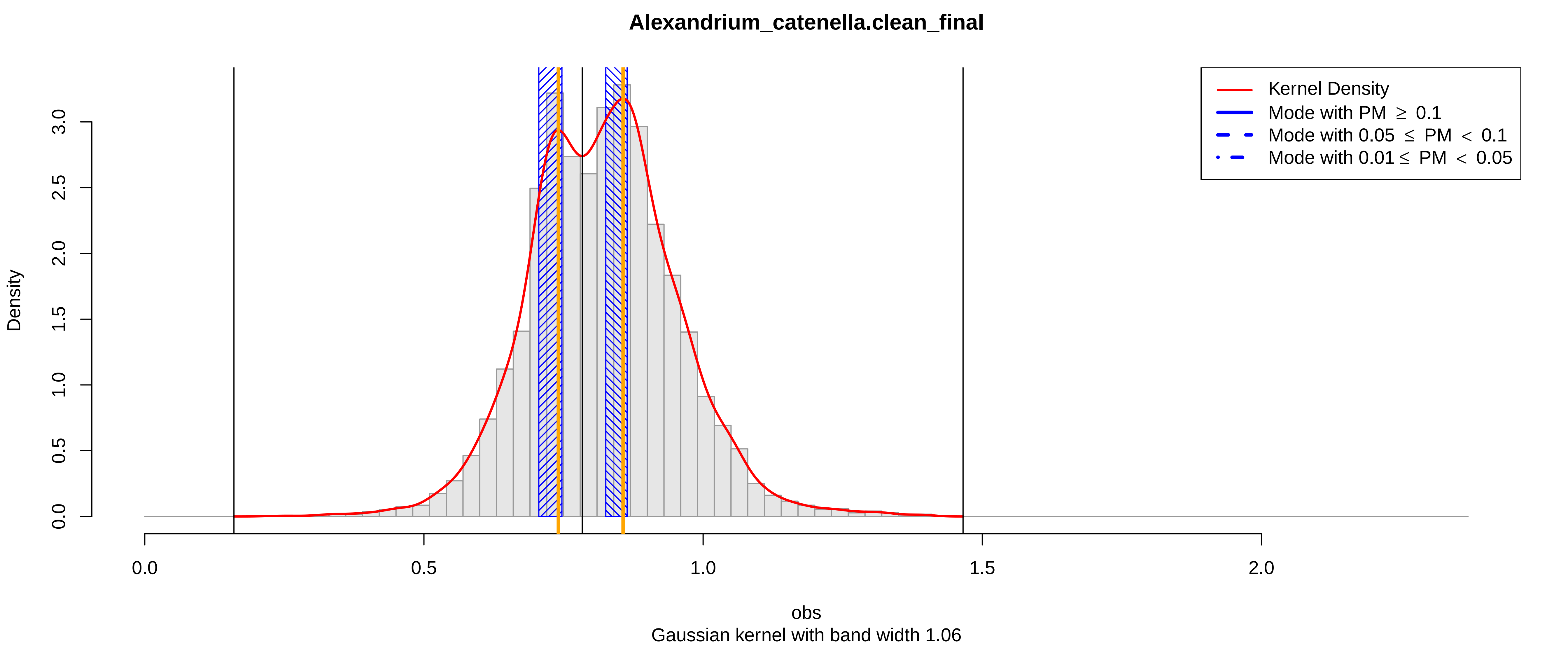

Alexandrium_catenella.clean_final
Kernel Density
Mode with PM ≥ 0.1
Mode with 0.05 ≤ PM < 0.1
Mode with 0.01 ≤ PM < 0.05
Density
0.0
0.5
1.0
1.5
2.0
2.5
3.0
0.0
0.5
1.0
1.5
2.0
obs
Gaussian kernel with band width 1.06

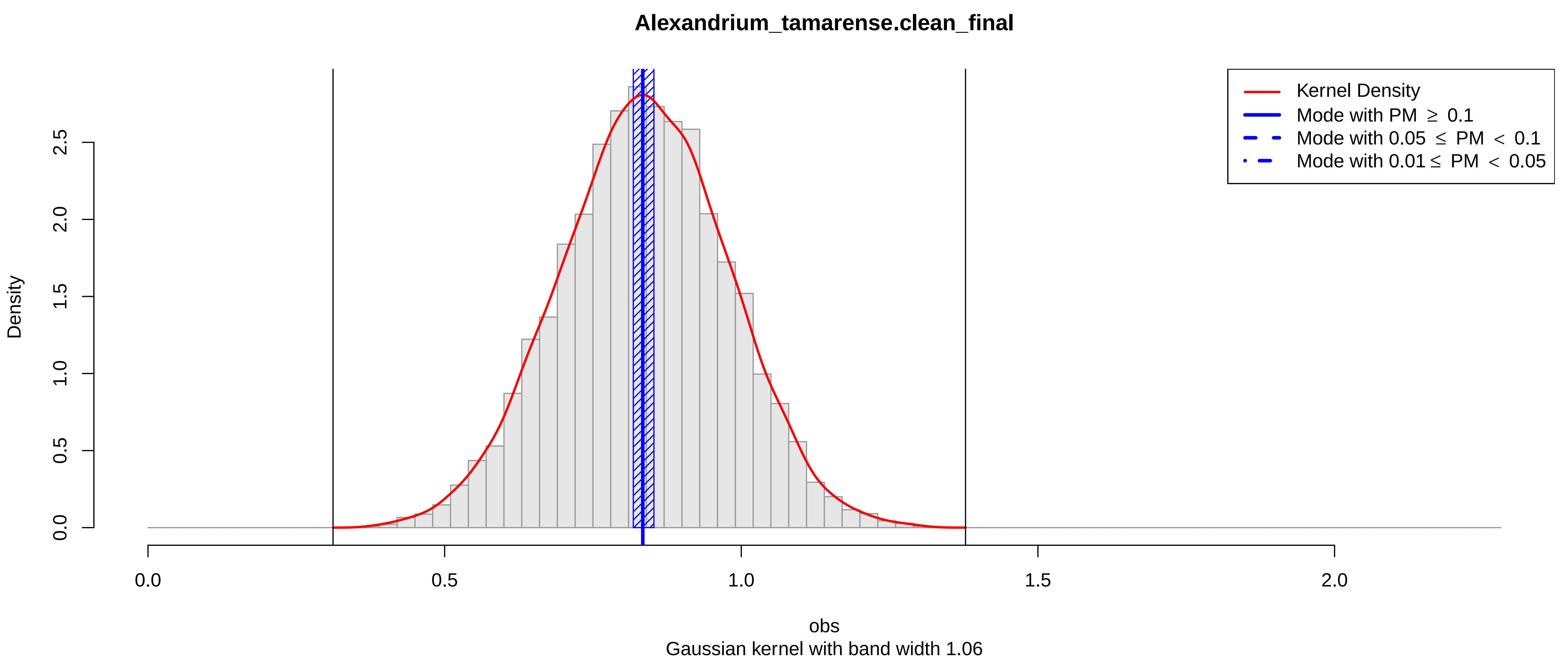

Alexandrium_tamarense.clean_final
Kernel Density
Mode with PM ≥ 0.1
Mode with 0.05 ≤ PM < 0.1
Mode with 0.01 ≤ PM < 0.05
Density
0.0
0.5
1.0
1.5
2.0
2.5
0.0
0.5
1.0
1.5
2.0
obs
Gaussian kernel with band width 1.06

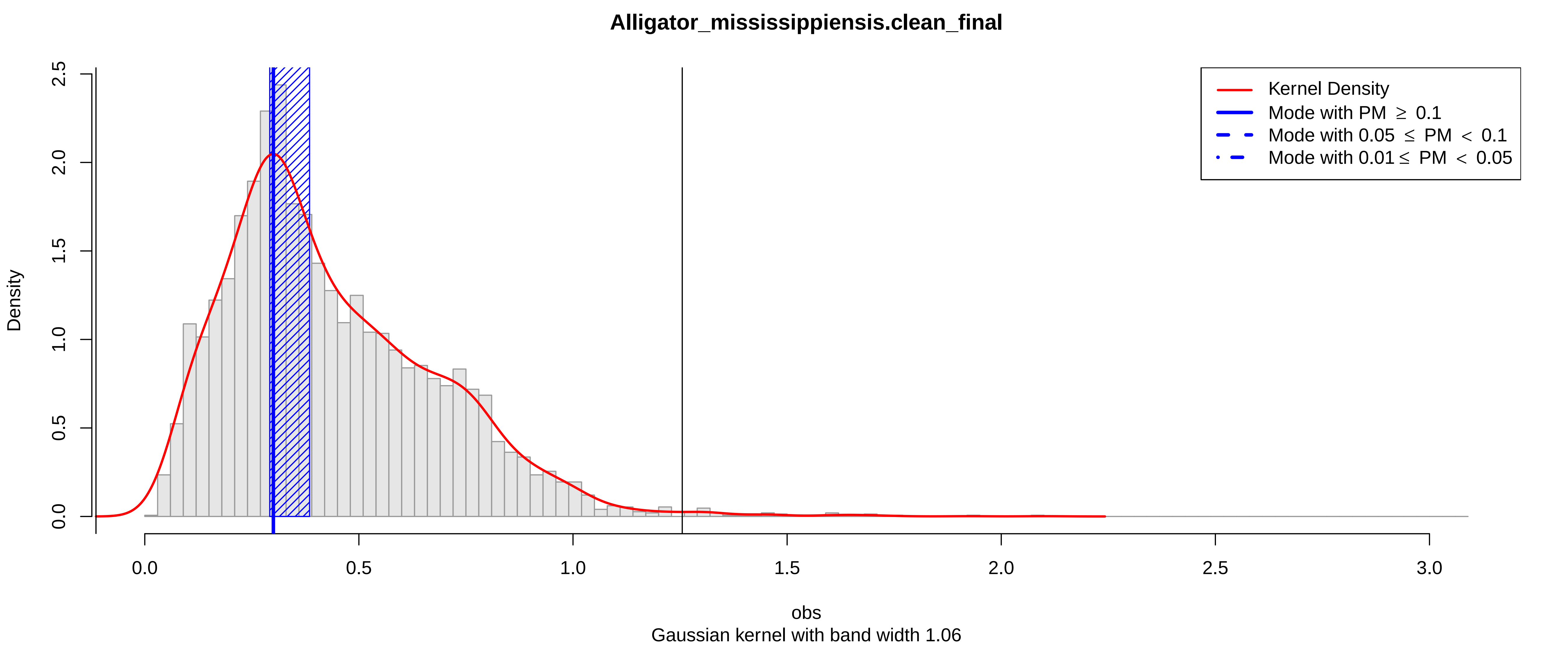
Alligator_mississippiensis.clean_final
Kernel Density
Mode with PM ≥ 0.1
Mode with 0.05 ≤ PM < 0.1
Mode with 0.01 ≤ PM < 0.05
Density
0.0
0.5
1.0
1.5
2.0
2.5
3.0
obs
Gaussian kernel with band width 1.06

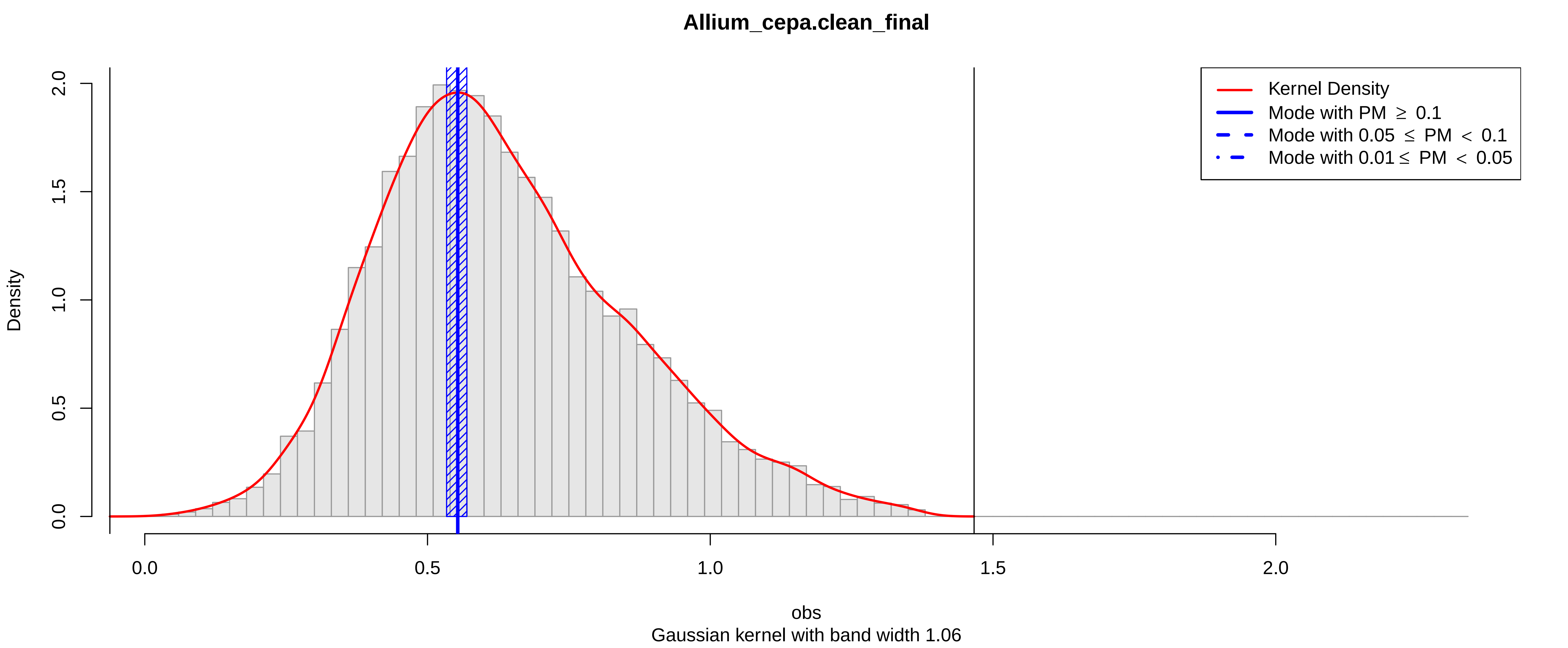

Allium_cepa.clean_final
Kernel Density
Mode with PM ≥ 0.1
Mode with 0.05 ≤ PM < 0.1
Mode with 0.01 ≤ PM < 0.05
Density
0.0
0.5
1.0
1.5
2.0
obs
Gaussian kernel with band width 1.06

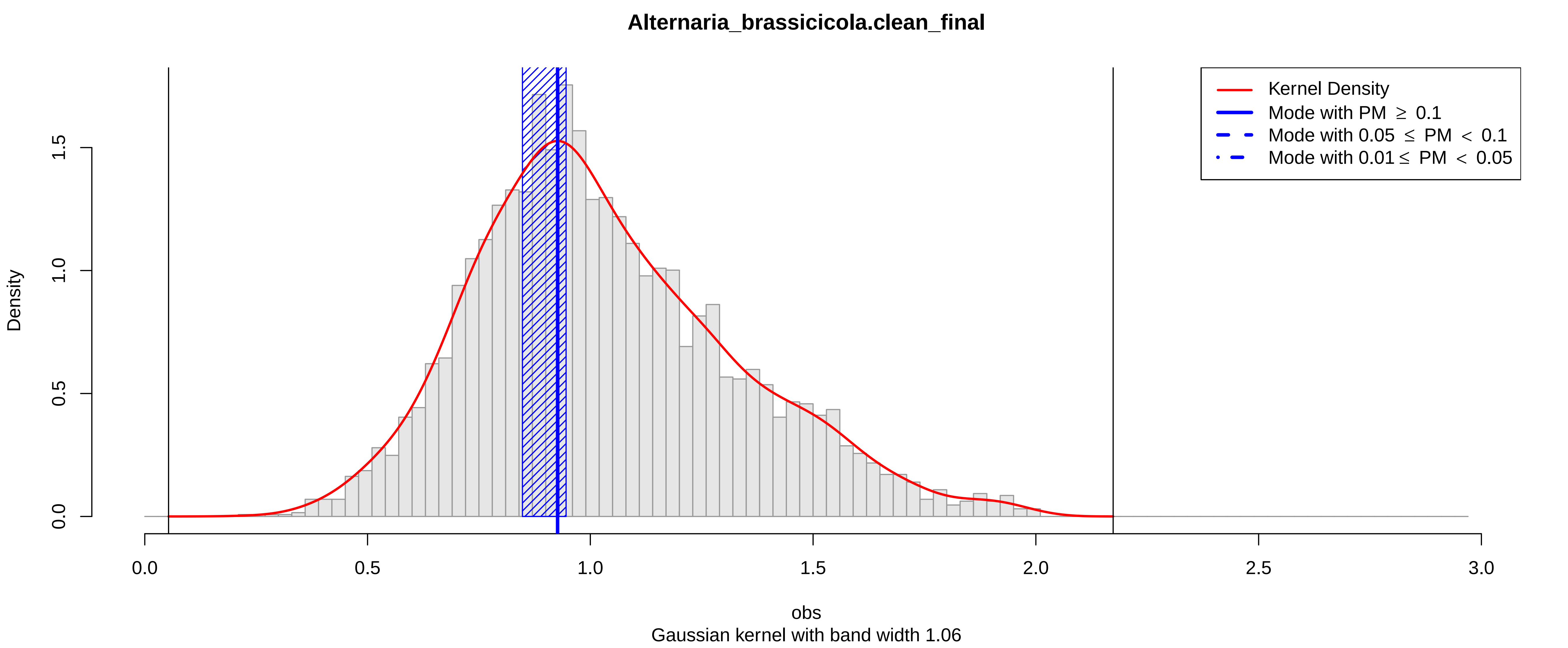
Alternaria_brassicicola.clean_final
Kernel Density
Mode with PM ≥ 0.1
Mode with 0.05 ≤ PM < 0.1
Mode with 0.01 ≤ PM < 0.05
Density
0.0
0.5
1.0
1.5
0.0
0.5
1.0
1.5
2.0
2.5
3.0
obs
Gaussian kernel with band width 1.06

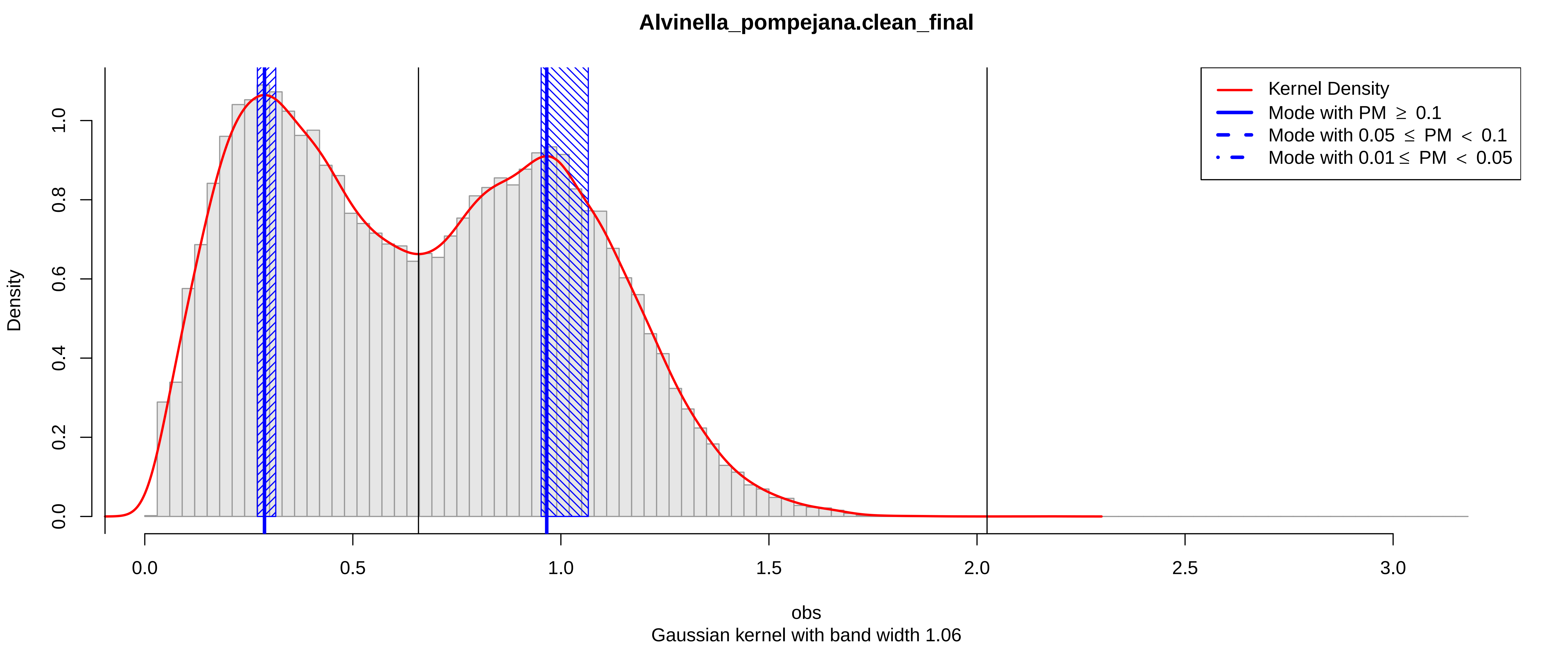

Alvinella_pompejana.clean_final
Kernel Density
Mode with PM ≥ 0.1
Mode with 0.05 ≤ PM < 0.1
Mode with 0.01 ≤ PM < 0.05
Density
0.0
0.2
0.4
0.6
0.8
1.0
0.0
0.5
1.0
1.5
2.0
2.5
3.0
obs
Gaussian kernel with band width 1.06

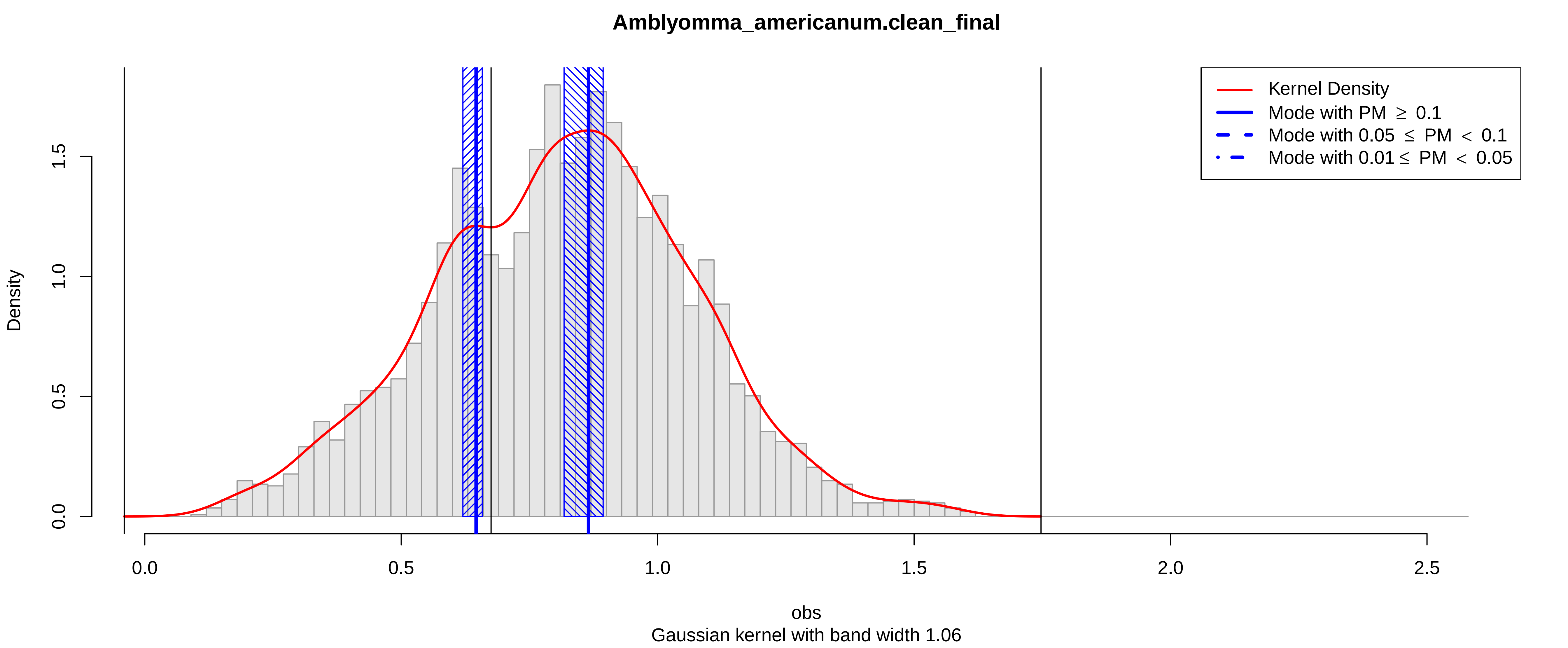

Amblyomma_americanum.clean_final
Kernel Density
Mode with PM ≥ 0.1
Mode with 0.05 ≤ PM < 0.1
Mode with 0.01 ≤ PM < 0.05
Density
0.0
0.5
1.0
1.5
0.0
0.5
1.0
1.5
2.0
2.5
obs
Gaussian kernel with band width 1.06

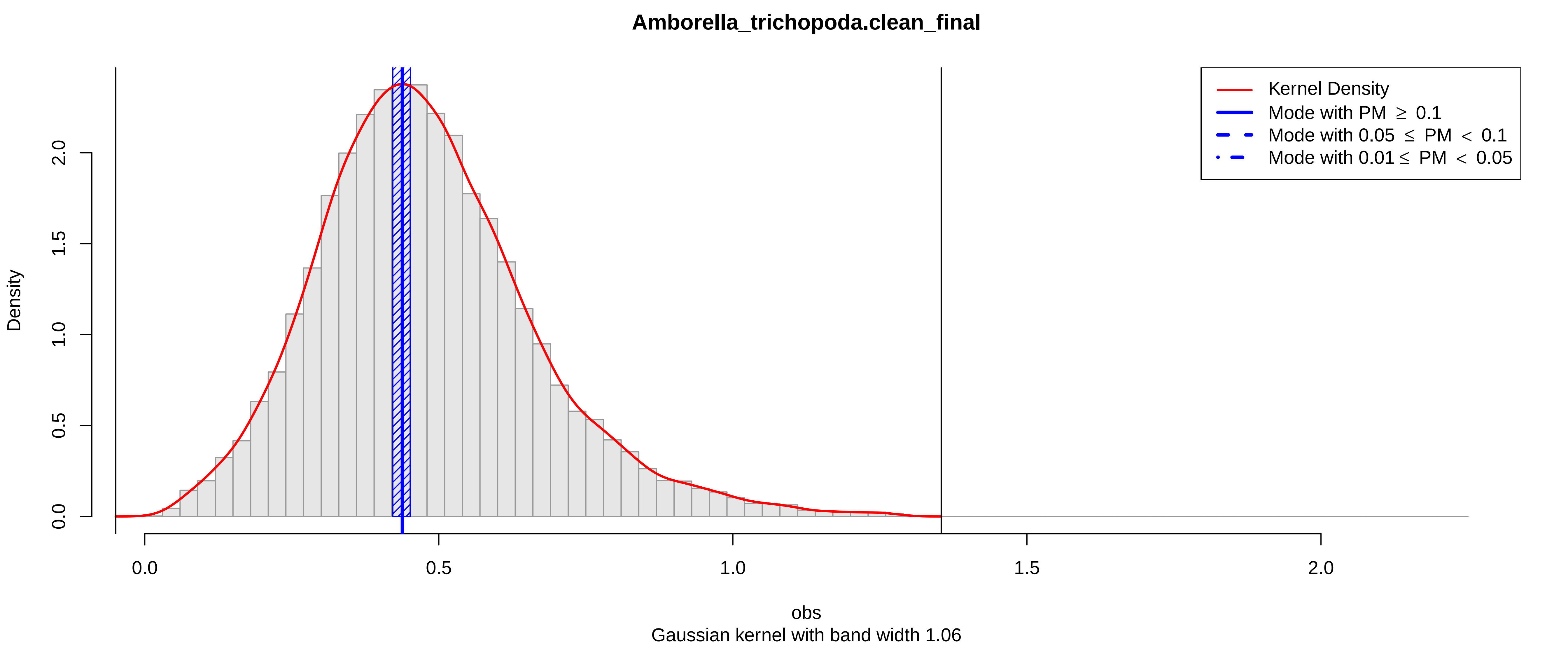

Amborella_trichopoda.clean_final
Kernel Density
Mode with PM ≥ 0.1
Mode with 0.05 ≤ PM < 0.1
Mode with 0.01 ≤ PM < 0.05
Density
0.0
0.5
1.0
1.5
2.0
obs
Gaussian kernel with band width 1.06

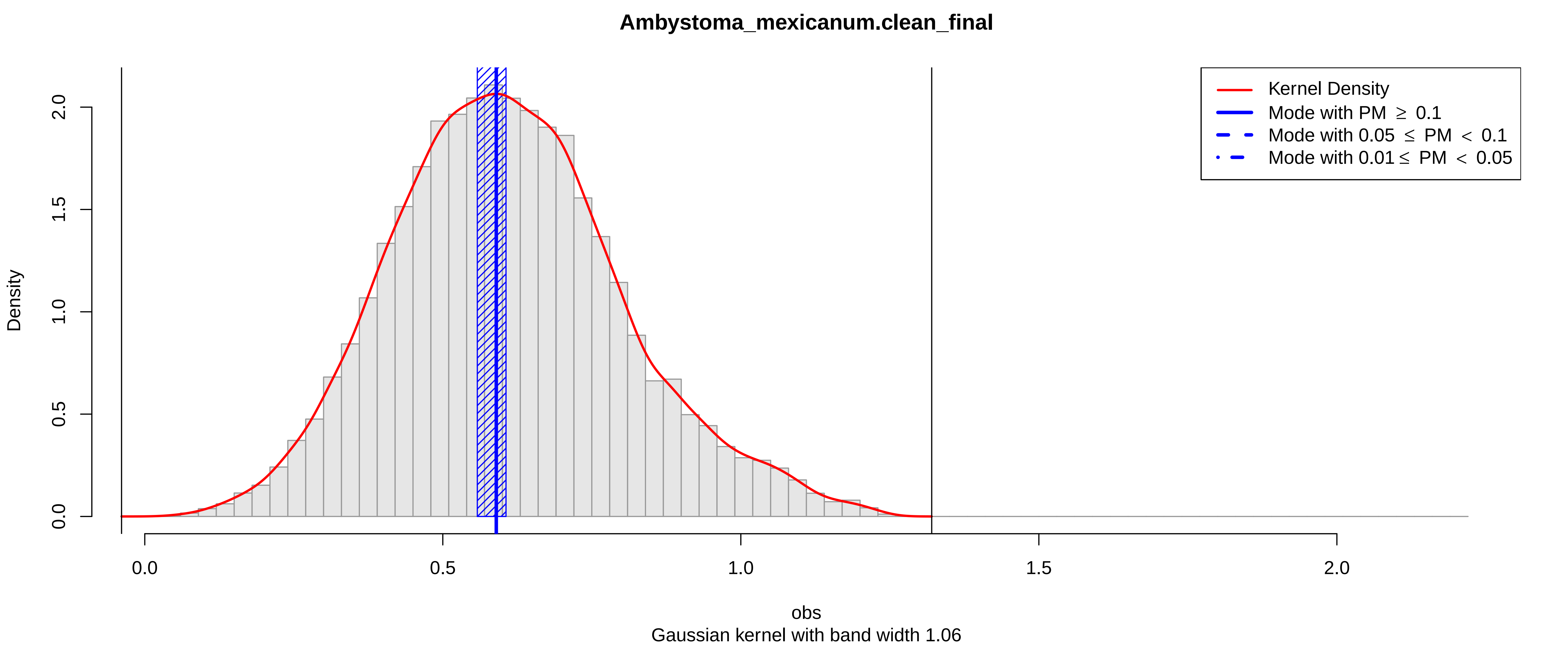
Ambystoma_mexicanum.clean_final
Kernel Density
Mode with PM ≥ 0.1
Mode with 0.05 ≤ PM < 0.1
Mode with 0.01 ≤ PM < 0.05
Density
0.0
0.5
1.0
1.5
2.0
obs
Gaussian kernel with band width 1.06

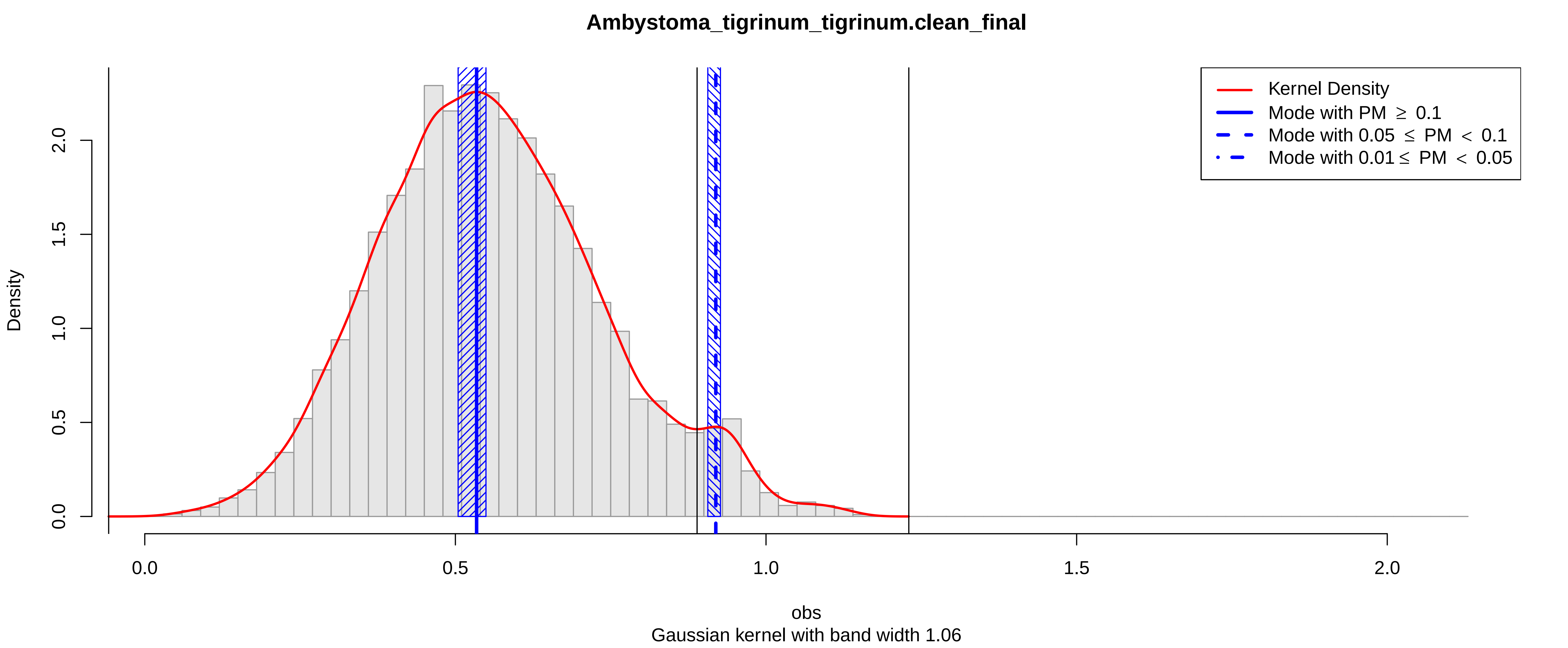
Ambystoma_tigrinum_tigrinum.clean_final
Kernel Density
Mode with PM ≥ 0.1
Mode with 0.05 ≤ PM < 0.1
Mode with 0.01 ≤ PM < 0.05
Density
0.0
0.5
1.0
1.5
2.0
0.0
0.5
1.0
1.5
2.0
obs
Gaussian kernel with band width 1.06

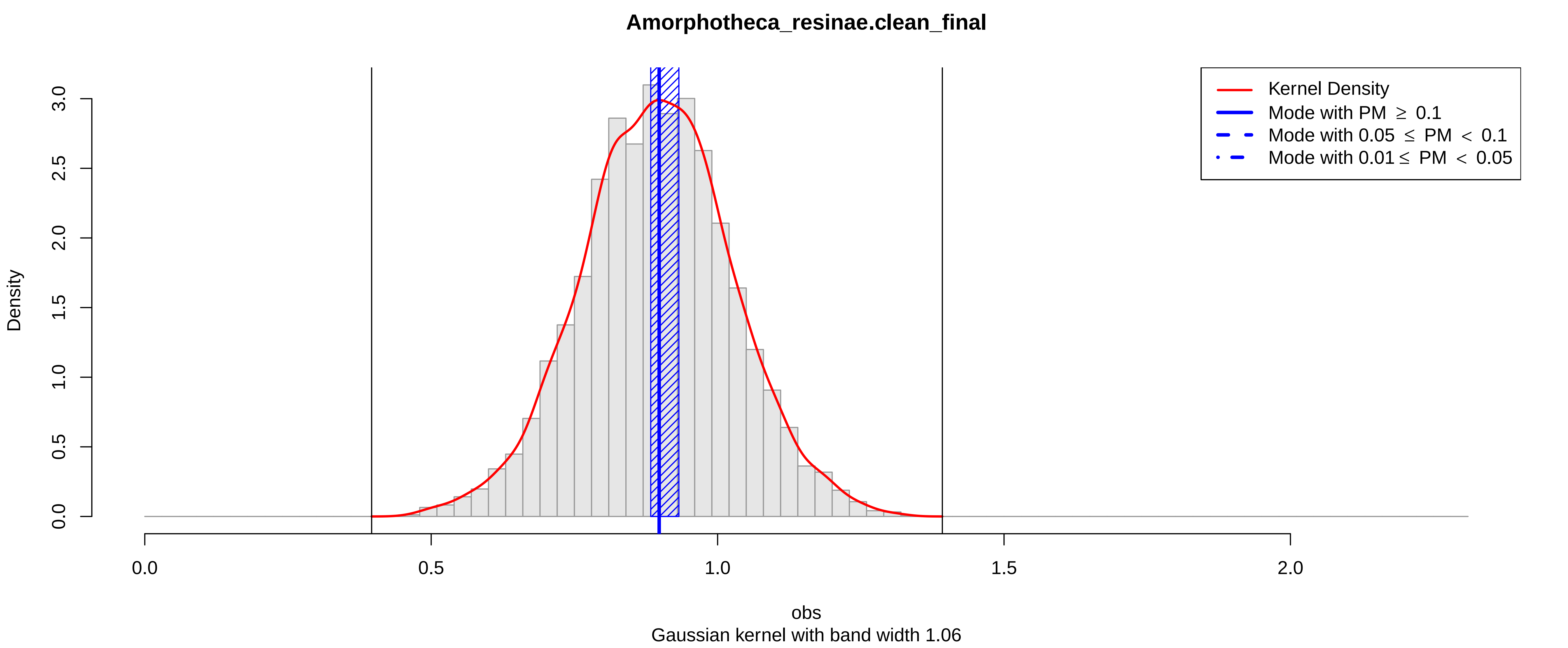

Amorphotheca_resinae.clean_final
Kernel Density
Mode with PM ≥ 0.1
Mode with 0.05 ≤ PM < 0.1
Mode with 0.01 ≤ PM < 0.05
Density
0.0
0.5
1.0
1.5
2.0
2.5
3.0
0.0
0.5
1.0
1.5
2.0
obs
Gaussian kernel with band width 1.06

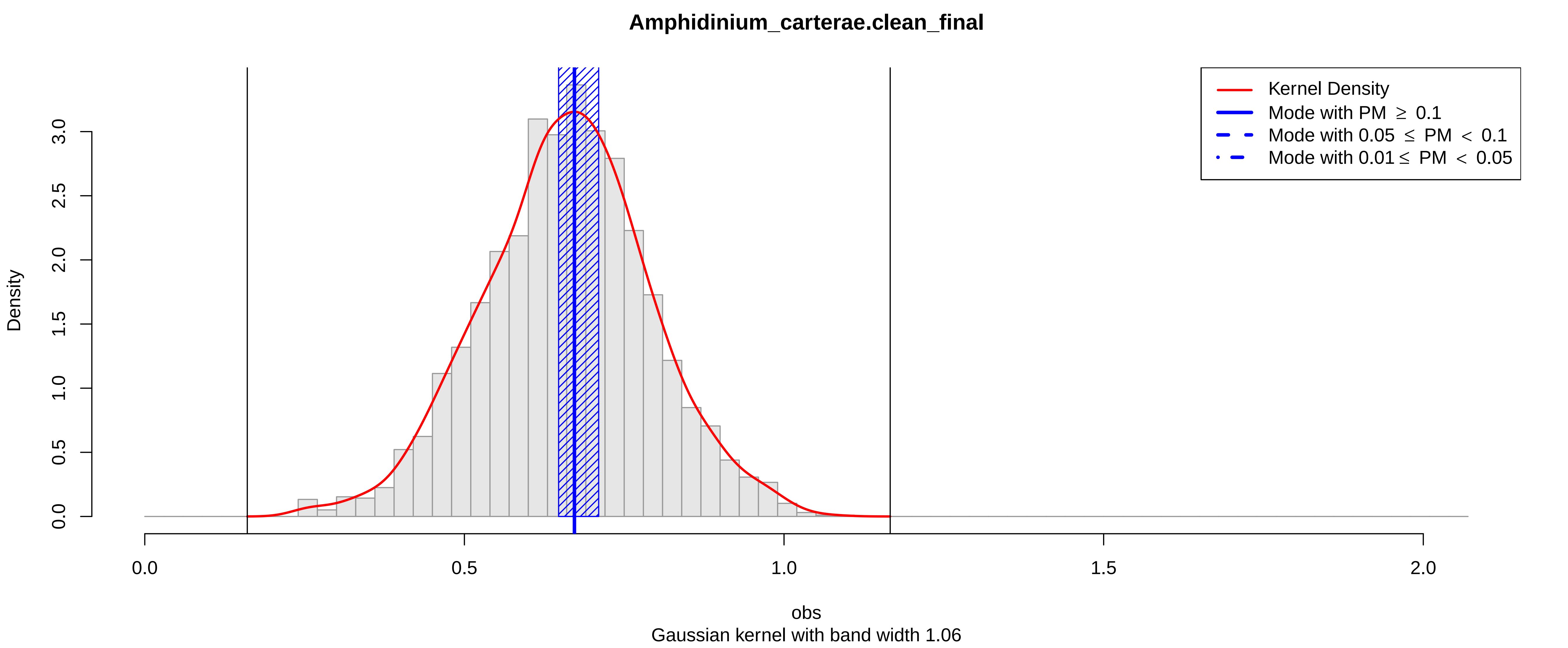
Amphidinium_carterae.clean_final
Kernel Density
Mode with PM ≥ 0.1
Mode with 0.05 ≤ PM < 0.1
Mode with 0.01 ≤ PM < 0.05
Density
0.0
0.5
1.0
1.5
2.0
2.5
3.0
obs
Gaussian kernel with band width 1.06

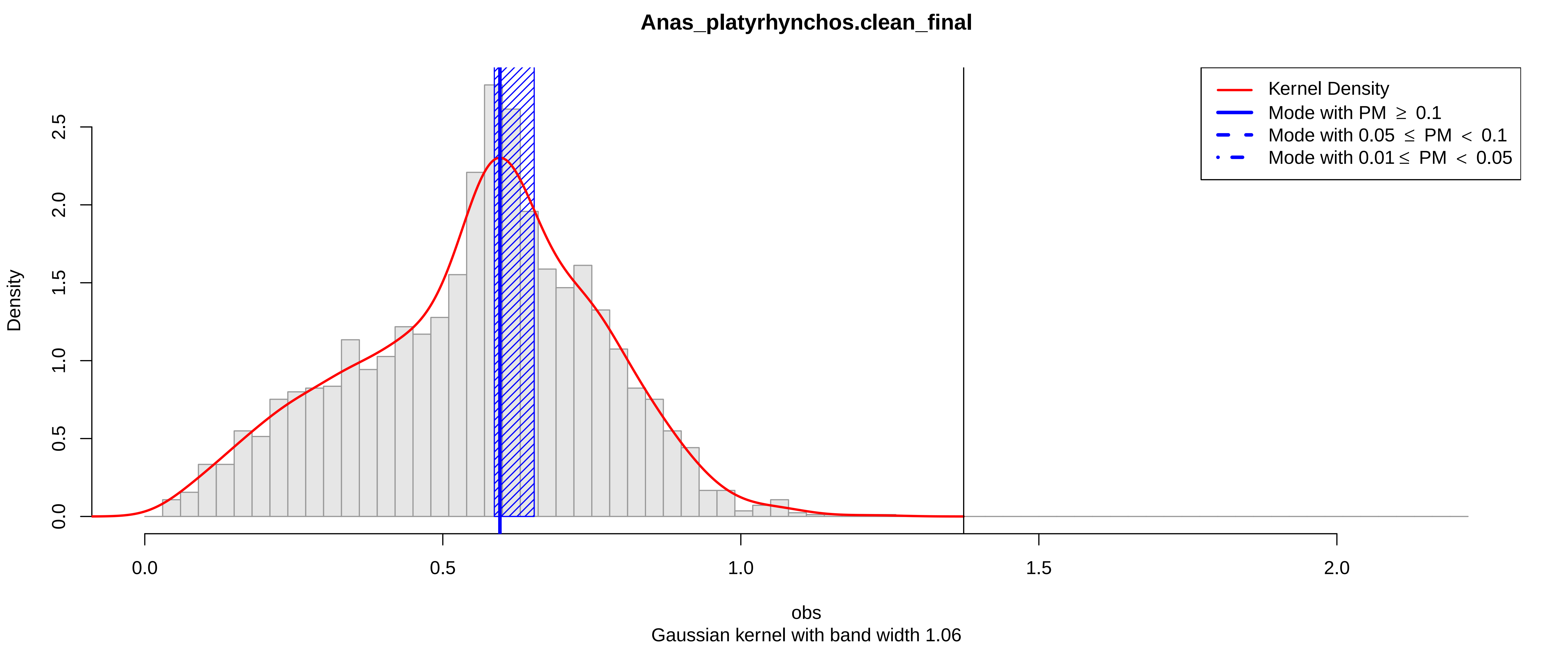
Anas_platyrhynchos.clean_final
Kernel Density
Mode with PM ≥ 0.1
Mode with 0.05 ≤ PM < 0.1
Mode with 0.01 ≤ PM < 0.05
Density
0.0
0.5
1.0
1.5
2.0
2.5
obs
Gaussian kernel with band width 1.06

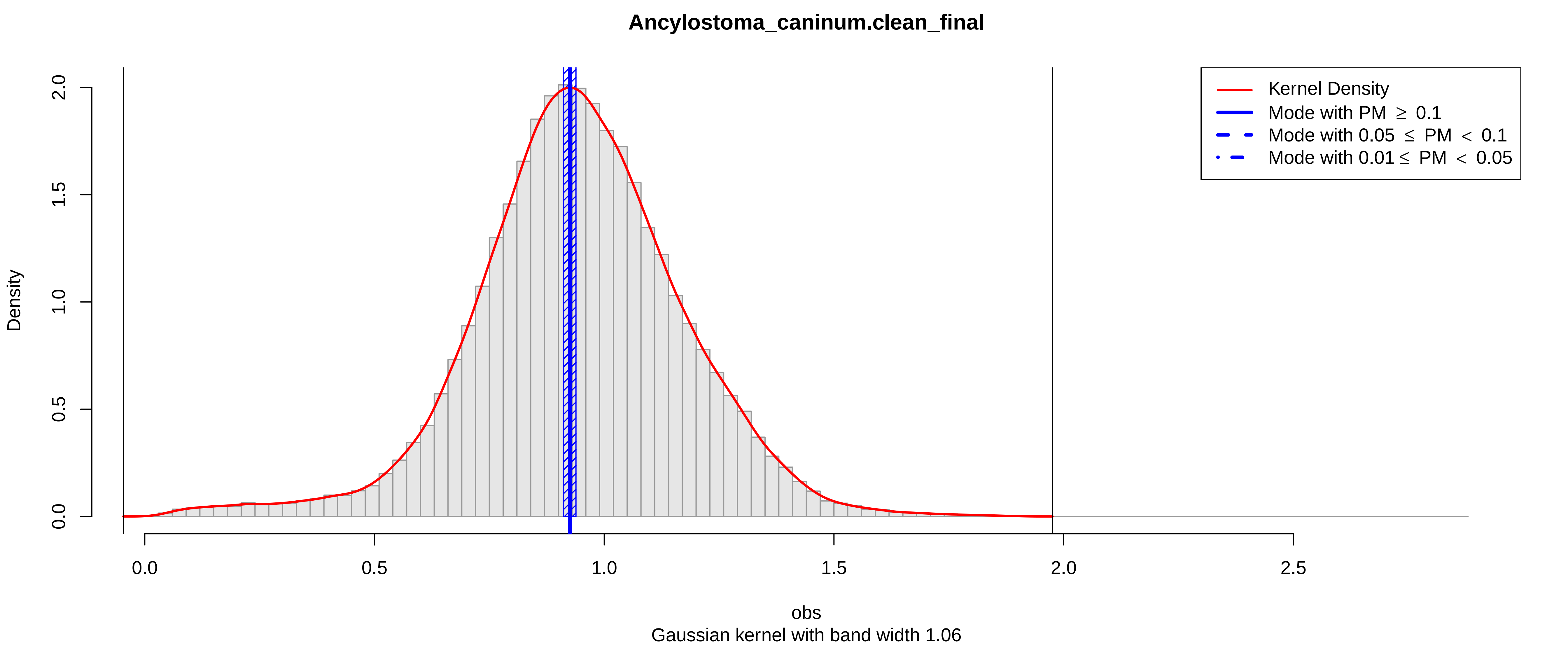

Ancylostoma_caninum.clean_final
Kernel Density
Mode with PM ≥ 0.1
Mode with 0.05 ≤ PM < 0.1
Mode with 0.01 ≤ PM < 0.05
Density
0.0
0.5
1.0
1.5
2.0
0.0
0.5
1.0
1.5
2.0
2.5
obs
Gaussian kernel with band width 1.06

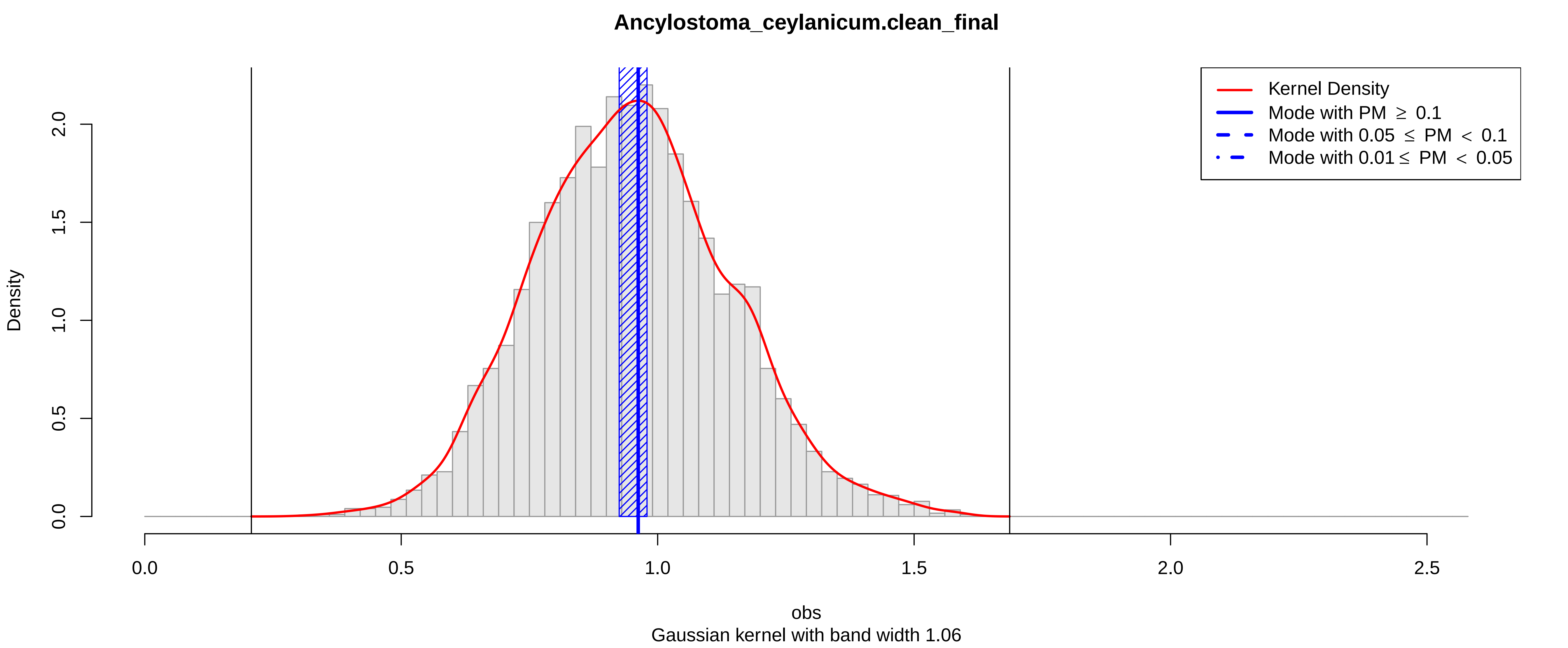

Ancylostoma_ceylanicum.clean_final
Kernel Density
Mode with PM ≥ 0.1
Mode with 0.05 ≤ PM < 0.1
Mode with 0.01 ≤ PM < 0.05
Density
0.0
0.5
1.0
1.5
2.0
0.0
0.5
1.0
1.5
2.0
2.5
obs
Gaussian kernel with band width 1.06

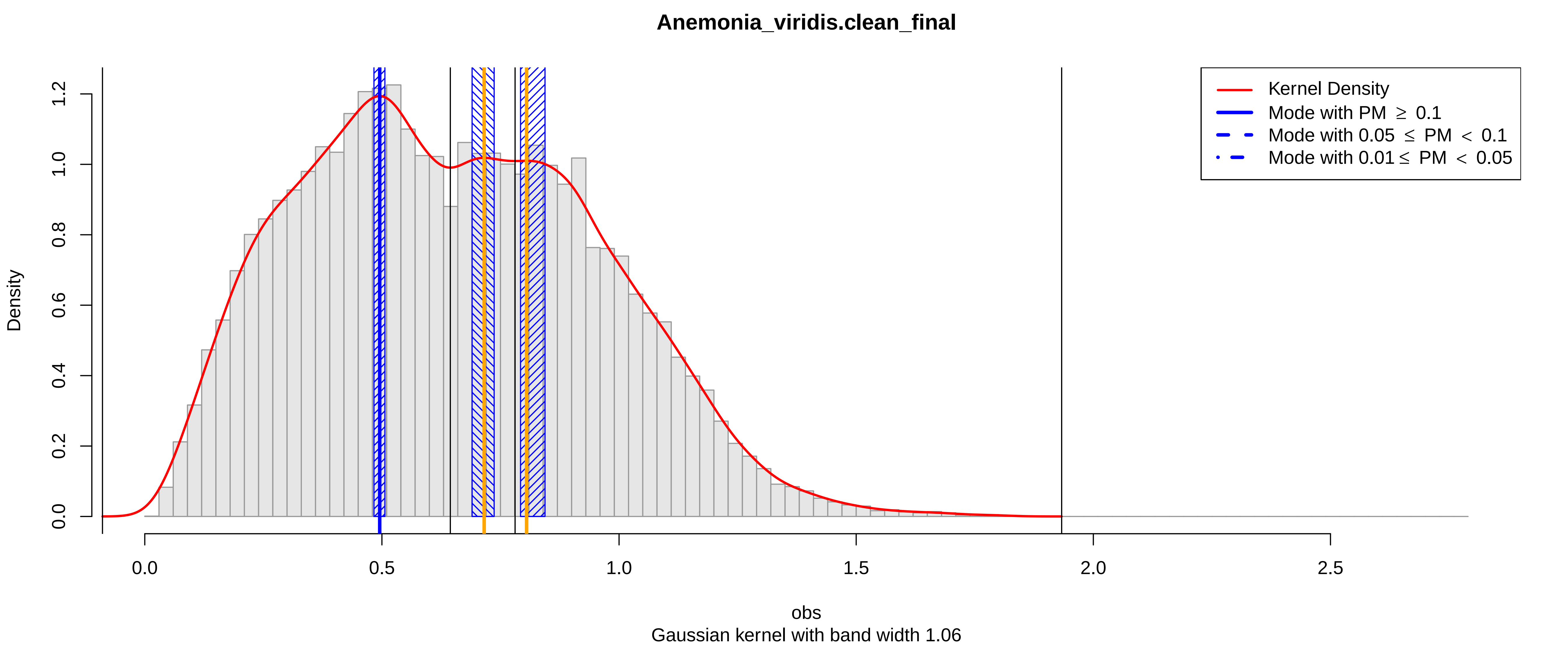

Anemonia_viridis.clean_final
Kernel Density
Mode with PM ≥ 0.1
Mode with 0.05 ≤ PM < 0.1
Mode with 0.01 ≤ PM < 0.05
Density
0.0
0.2
0.4
0.6
0.8
1.0
1.2
0.0
0.5
1.0
1.5
2.0
2.5
obs
Gaussian kernel with band width 1.06

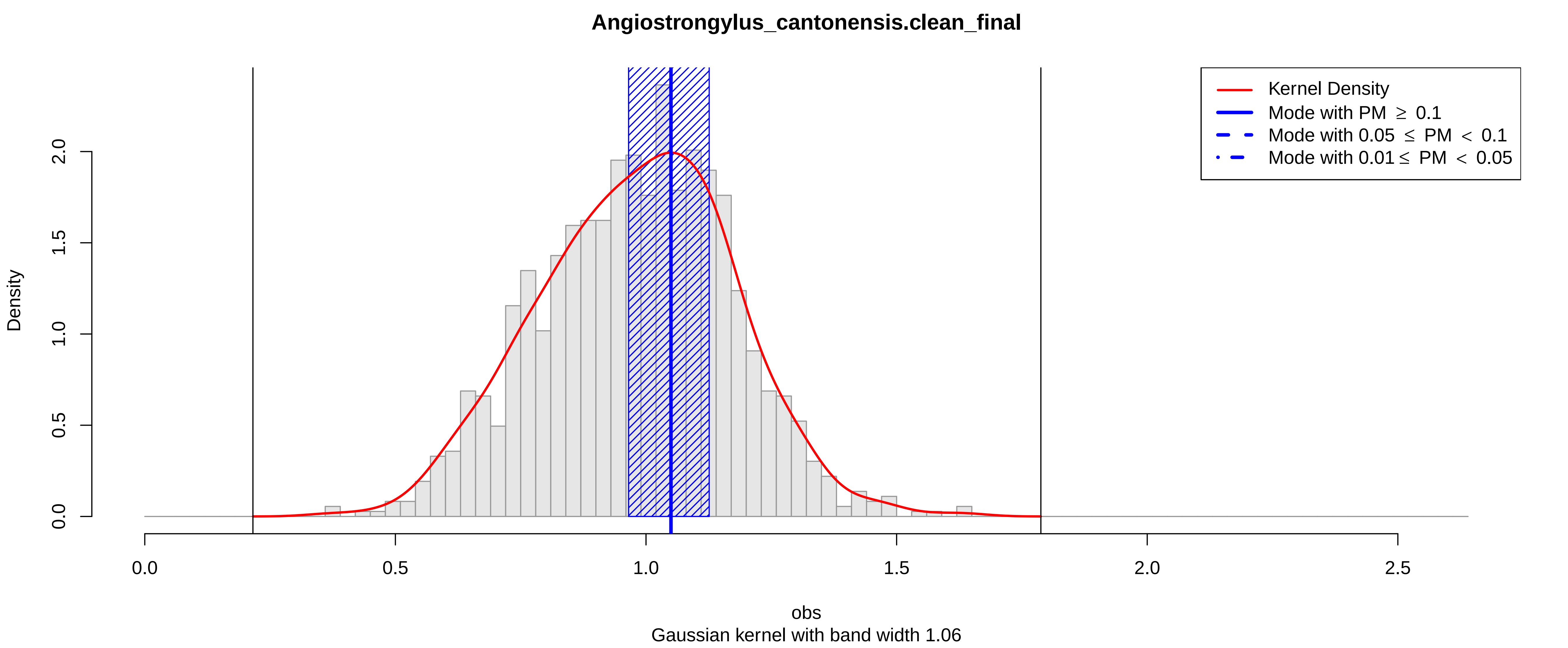
Angiostrongylus_cantonensis.clean_final
Kernel Density
Mode with PM ≥ 0.1
Mode with 0.05 ≤ PM < 0.1
Mode with 0.01 ≤ PM < 0.05
Density
0.0
0.5
1.0
1.5
2.0
2.5
obs
Gaussian kernel with band width 1.06

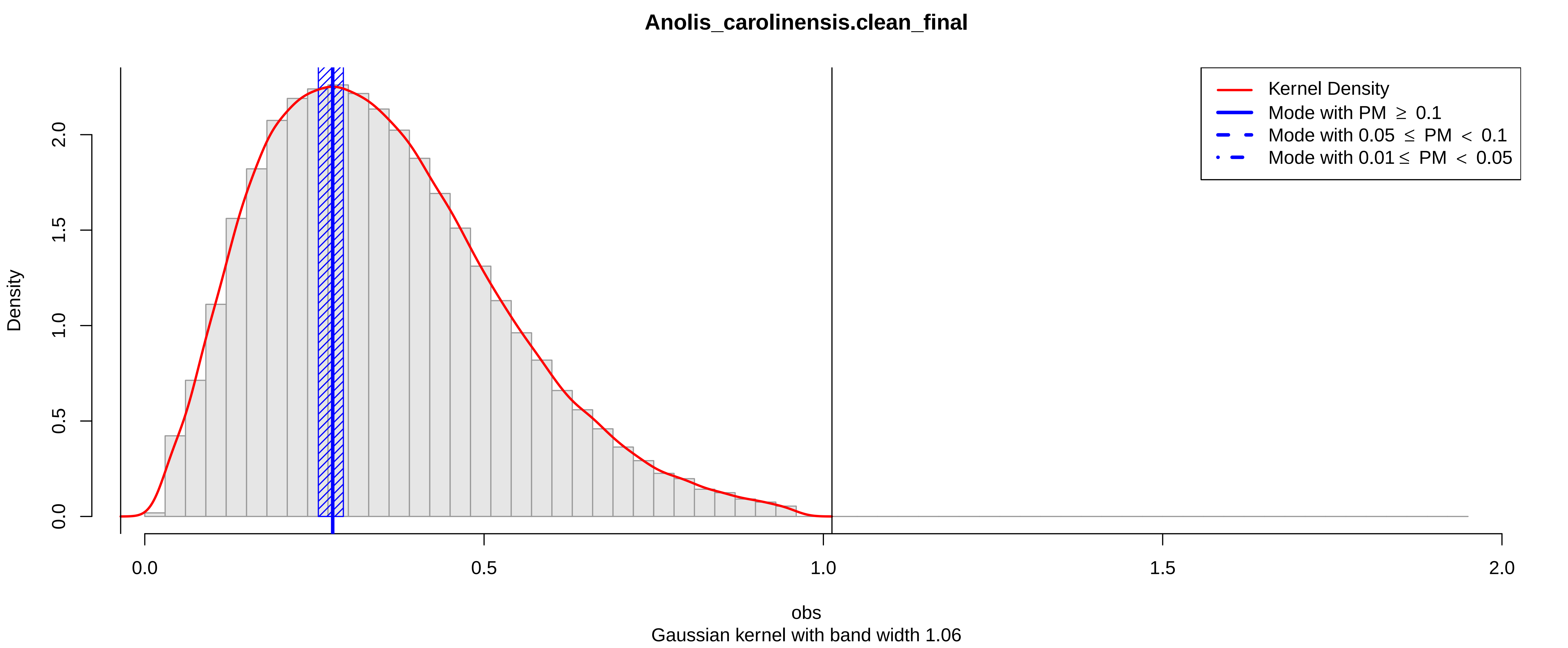

Anolis_carolinensis.clean_final
Kernel Density
Mode with PM ≥ 0.1
Mode with 0.05 ≤ PM < 0.1
Mode with 0.01 ≤ PM < 0.05
Density
0.0
0.5
1.0
1.5
2.0
0.0
0.5
1.0
1.5
2.0
obs
Gaussian kernel with band width 1.06

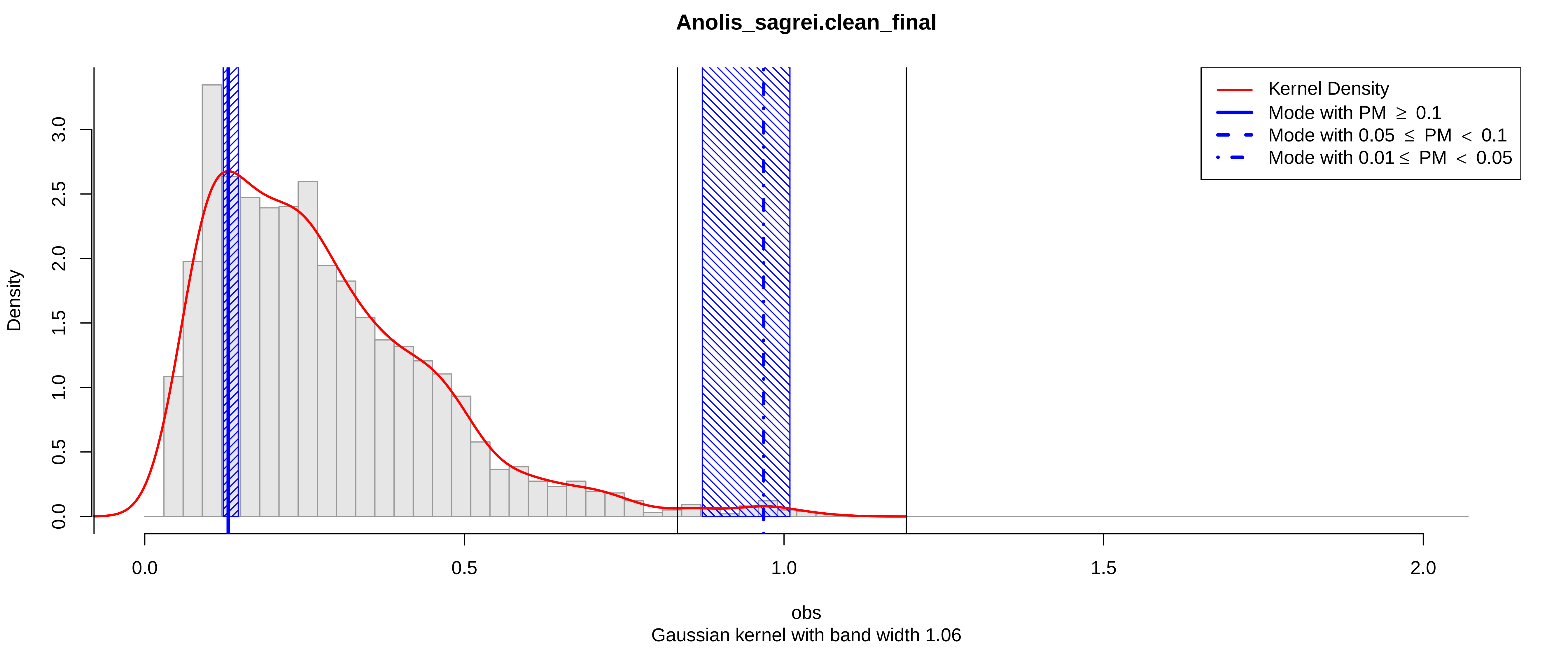

Anolis_sagrei.clean_final
Kernel Density
Mode with PM ≥ 0.1
Mode with 0.05 ≤ PM < 0.1
Mode with 0.01 ≤ PM < 0.05
Density
0.0
0.5
1.0
1.5
2.0
2.5
3.0
0.0
0.5
1.0
1.5
2.0
obs
Gaussian kernel with band width 1.06

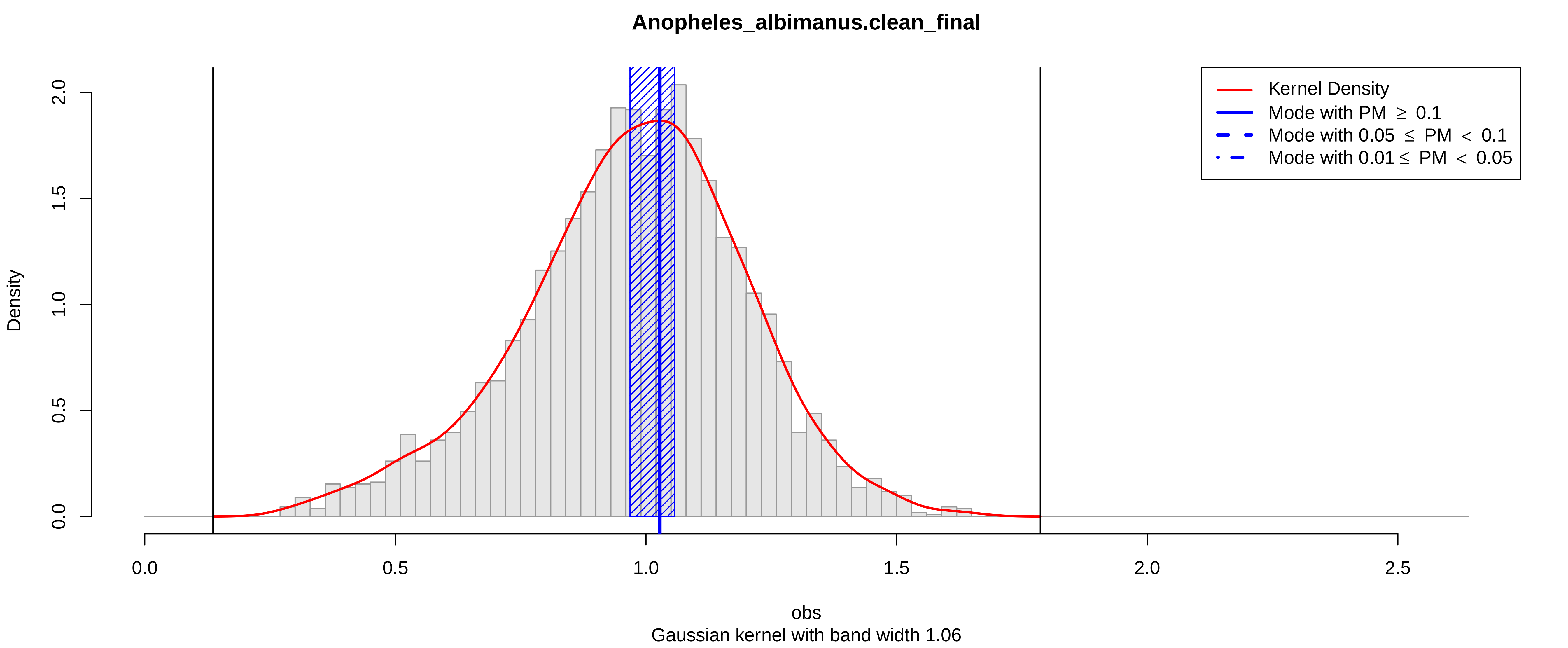

Anopheles_albimanus.clean_final
Kernel Density
Mode with PM ≥ 0.1
Mode with 0.05 ≤ PM < 0.1
Mode with 0.01 ≤ PM < 0.05
Density
0.0
0.5
1.0
1.5
2.0
2.5
obs
Gaussian kernel with band width 1.06

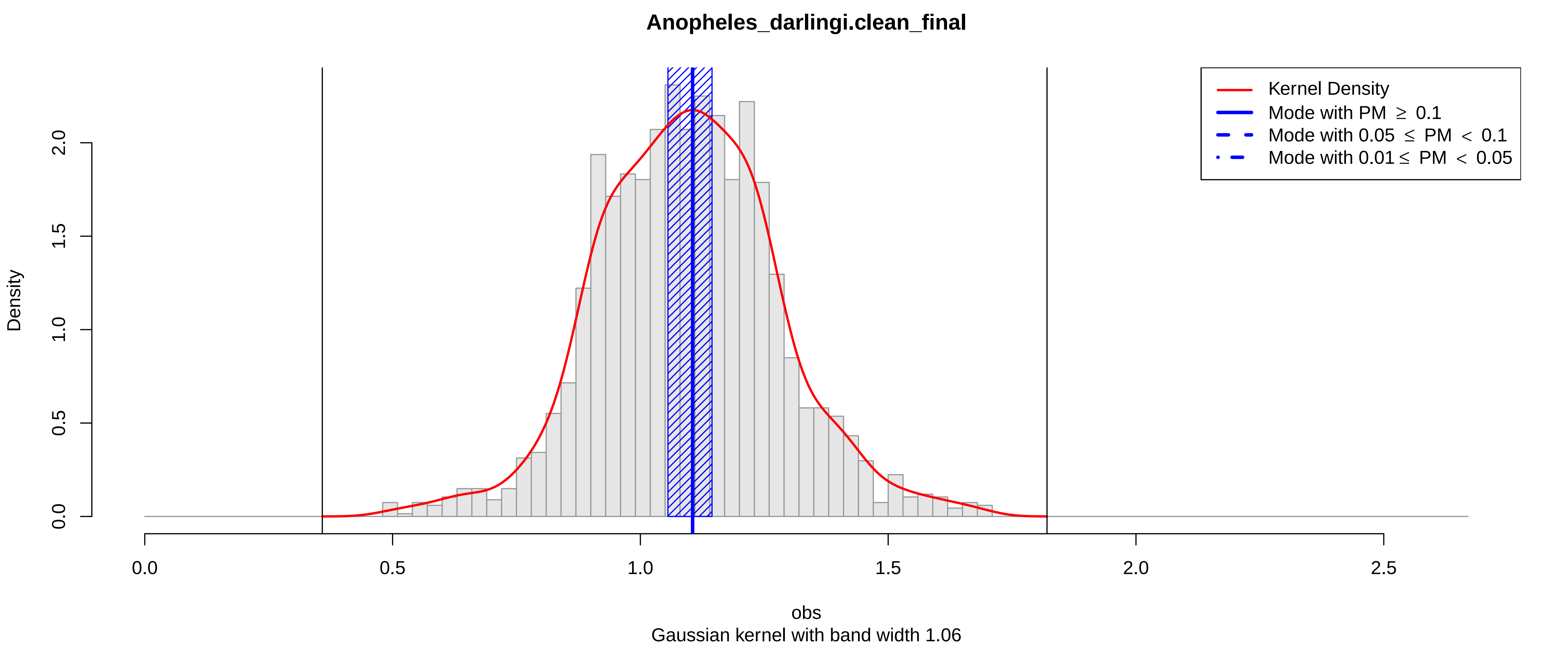
Anopheles_darlingi.clean_final
Kernel Density
Mode with PM ≥ 0.1
Mode with 0.05 ≤ PM < 0.1
Mode with 0.01 ≤ PM < 0.05
Density
0.0
0.5
1.0
1.5
2.0
0.0
0.5
1.0
1.5
2.0
2.5
obs
Gaussian kernel with band width 1.06

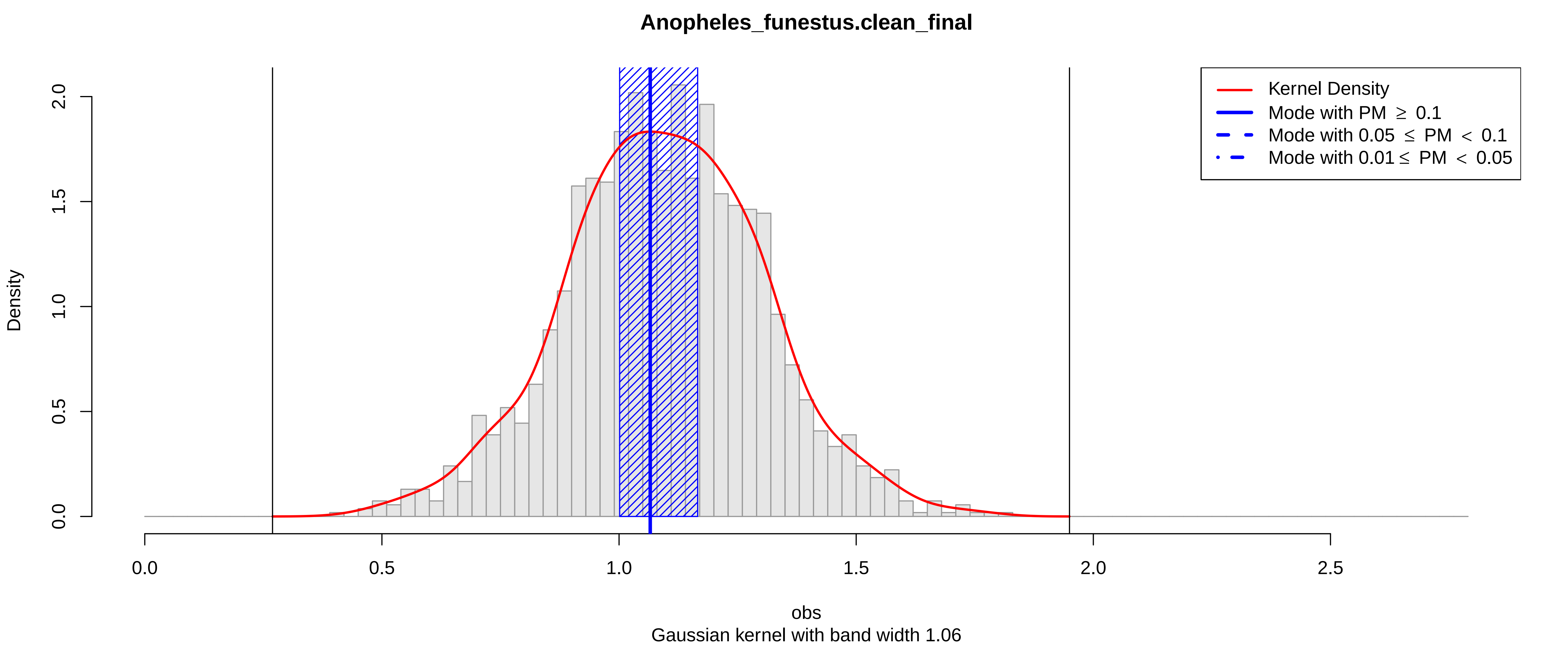

Anopheles_funestus.clean_final
Kernel Density
Mode with PM ≥ 0.1
Mode with 0.05 ≤ PM < 0.1
Mode with 0.01 ≤ PM < 0.05
Density
0.0
0.5
1.0
1.5
2.0
2.5
obs
Gaussian kernel with band width 1.06

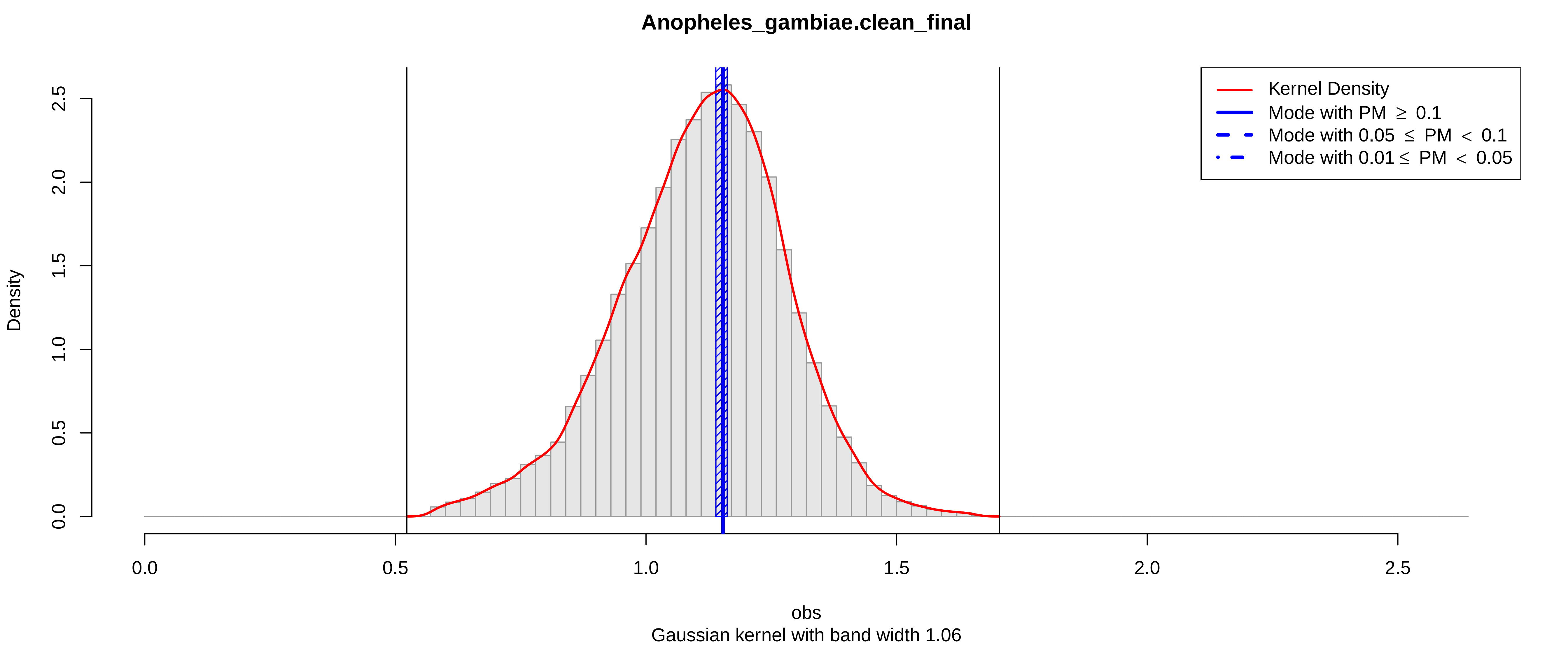

Anopheles_gambiae.clean_final
Density
0.0
0.5
1.0
1.5
2.0
2.5
obs
Gaussian kernel with band width 1.06
Kernel Density
Mode with PM ≥ 0.1
Mode with 0.05 ≤ PM < 0.1
Mode with 0.01 ≤ PM < 0.05

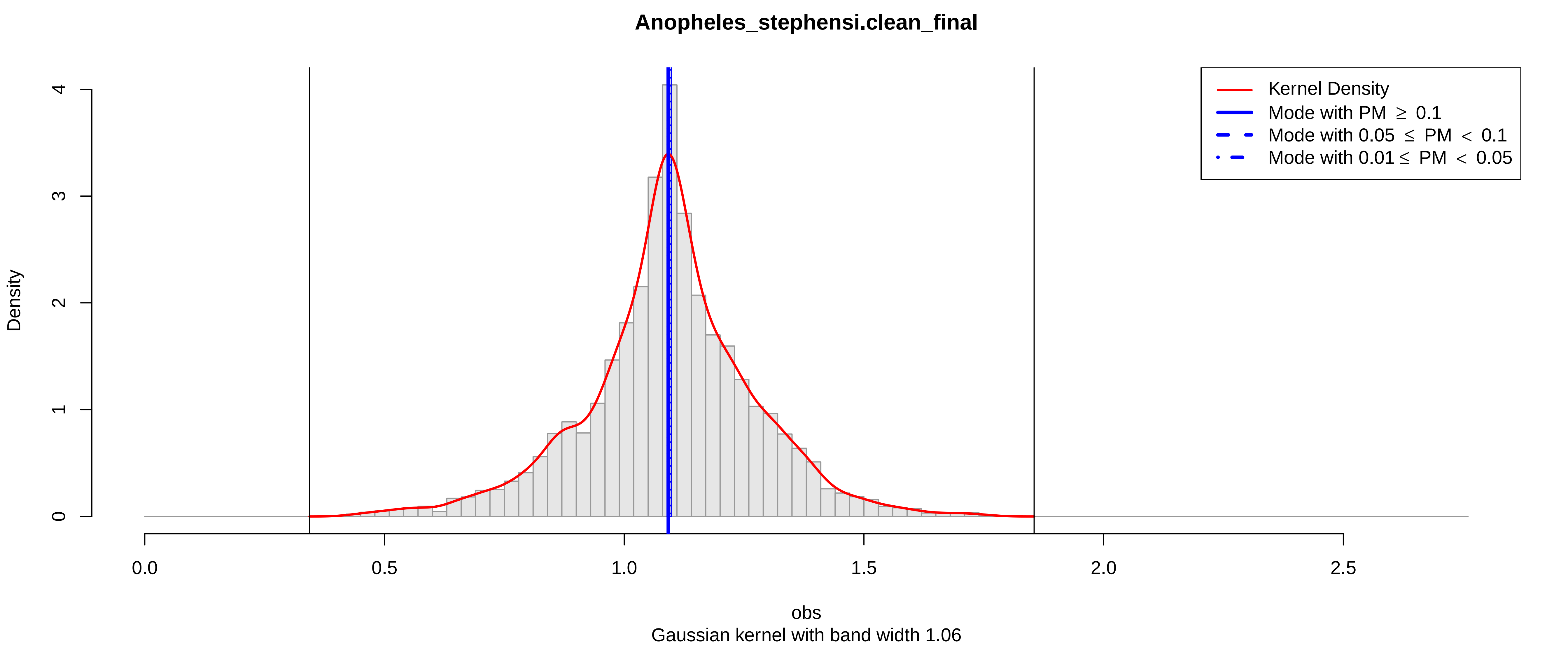

Anopheles_stephensi.clean_final
Kernel Density
Mode with PM ≥ 0.1
Mode with 0.05 ≤ PM < 0.1
Mode with 0.01 ≤ PM < 0.05
Density
0
1
2
3
4
0.0
0.5
1.0
1.5
2.0
2.5
obs
Gaussian kernel with band width 1.06

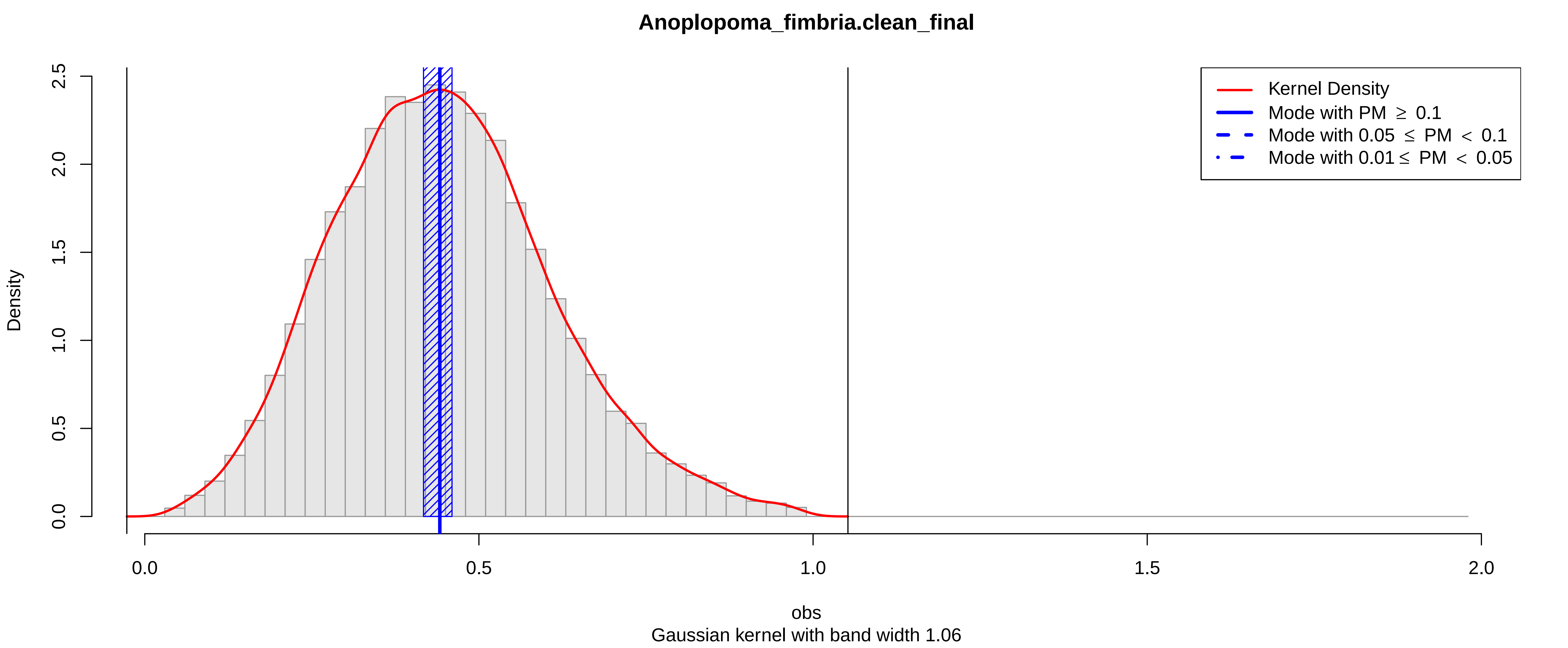
Anoplopoma_fimbria.clean_final
Kernel Density
Mode with PM ≥ 0.1
Mode with 0.05 ≤ PM < 0.1
Mode with 0.01 ≤ PM < 0.05
Density
0.0
0.5
1.0
1.5
2.0
2.5
0.0
0.5
1.0
1.5
2.0
obs
Gaussian kernel with band width 1.06

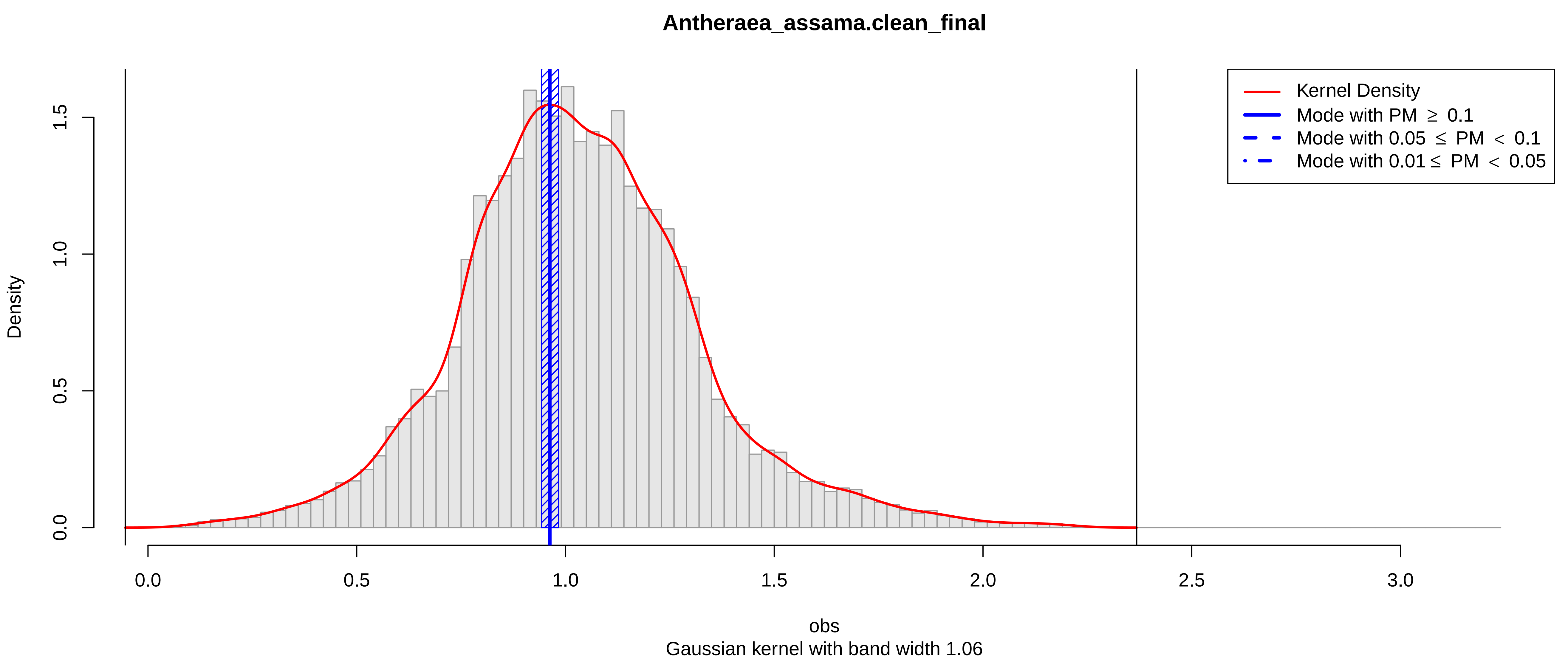

Antheraea_assama.clean_final
Density
1.5
1.0
0.5
0.0
0.0
0.5
1.0
1.5
2.0
2.5
3.0
obs
Gaussian kernel with band width 1.06
Kernel Density
Mode with PM ≥ 0.1
Mode with 0.05 ≤ PM < 0.1
Mode with 0.01 ≤ PM < 0.05

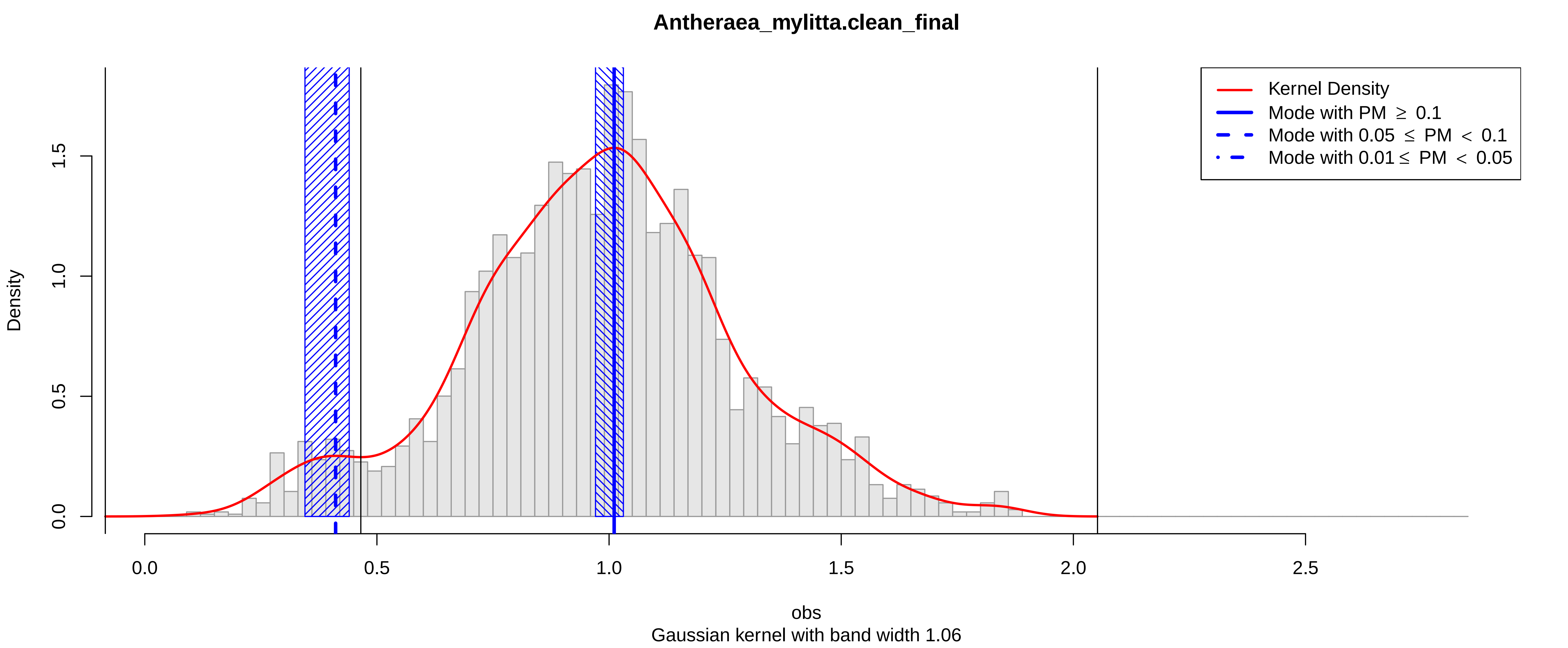
Antheraea_mylitta.clean_final
Kernel Density
Mode with PM ≥ 0.1
Mode with 0.05 ≤ PM < 0.1
Mode with 0.01 ≤ PM < 0.05
Density
0.0
0.5
1.0
1.5
2.0
2.5
obs
Gaussian kernel with band width 1.06

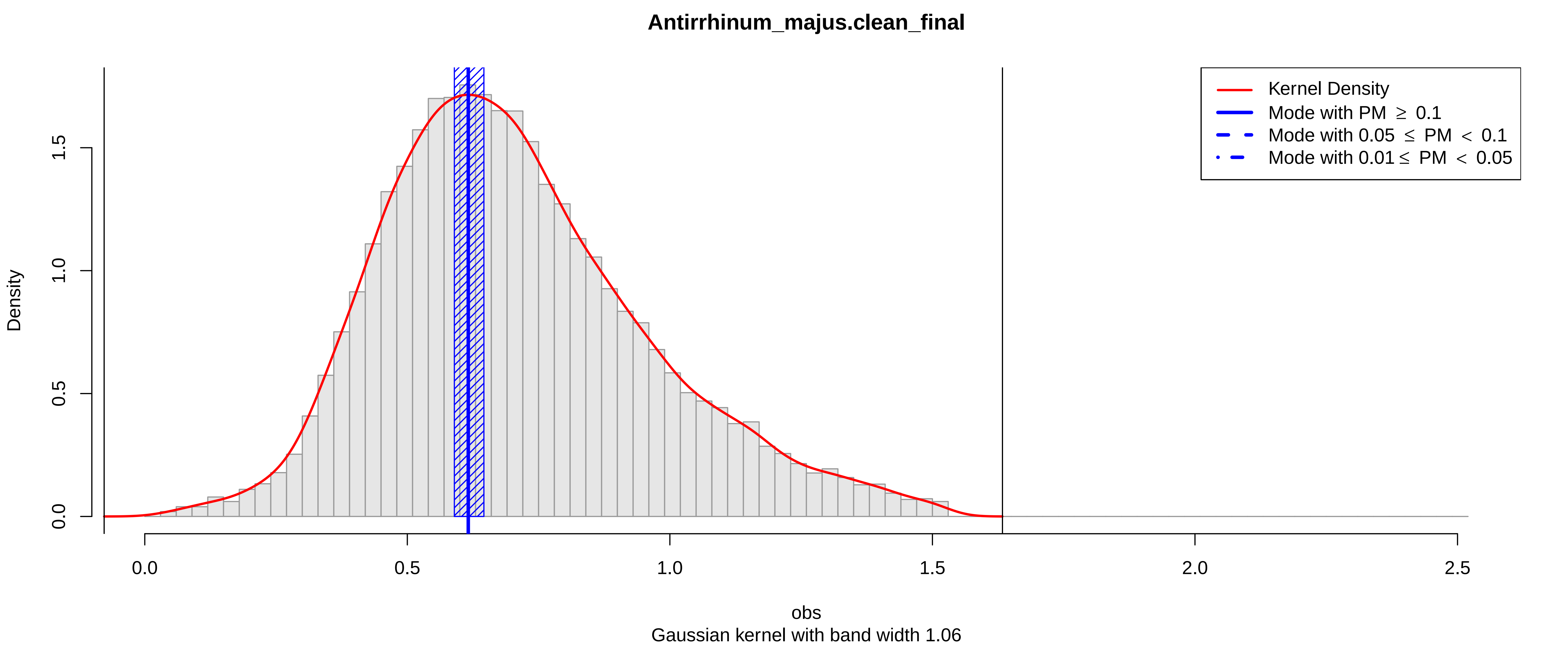

Antirrhinum_majus.clean_final
Kernel Density
Mode with PM ≥ 0.1
Mode with 0.05 ≤ PM < 0.1
Mode with 0.01 ≤ PM < 0.05
Density
0.0
0.5
1.0
1.5
0.0
0.5
1.0
1.5
2.0
2.5
obs
Gaussian kernel with band width 1.06

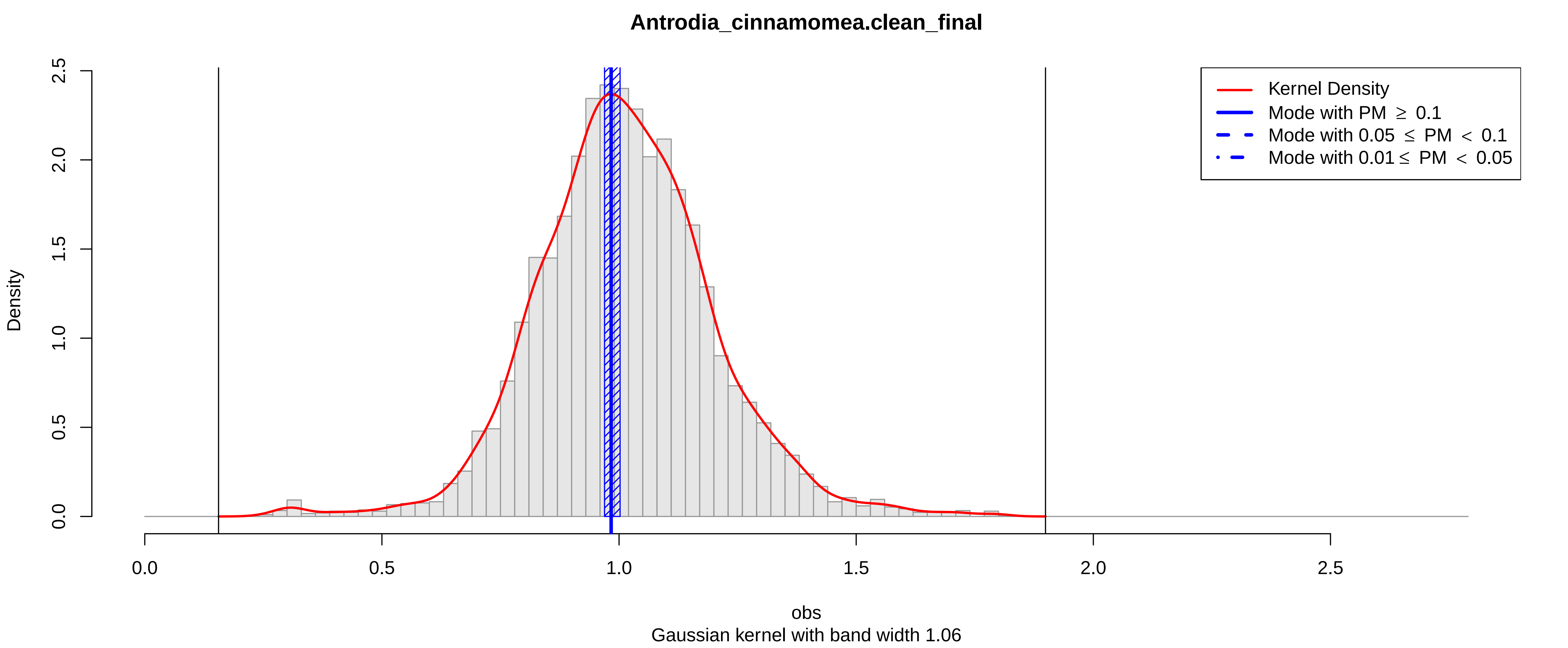

Antrodia_cinnamomea.clean_final
Density
obs
Gaussian kernel with band width 1.06
Kernel Density
Mode with PM ≥ 0.1
Mode with 0.05 ≤ PM < 0.1
Mode with 0.01 ≤ PM < 0.05
0.0
0.5
1.0
1.5
2.0
2.5

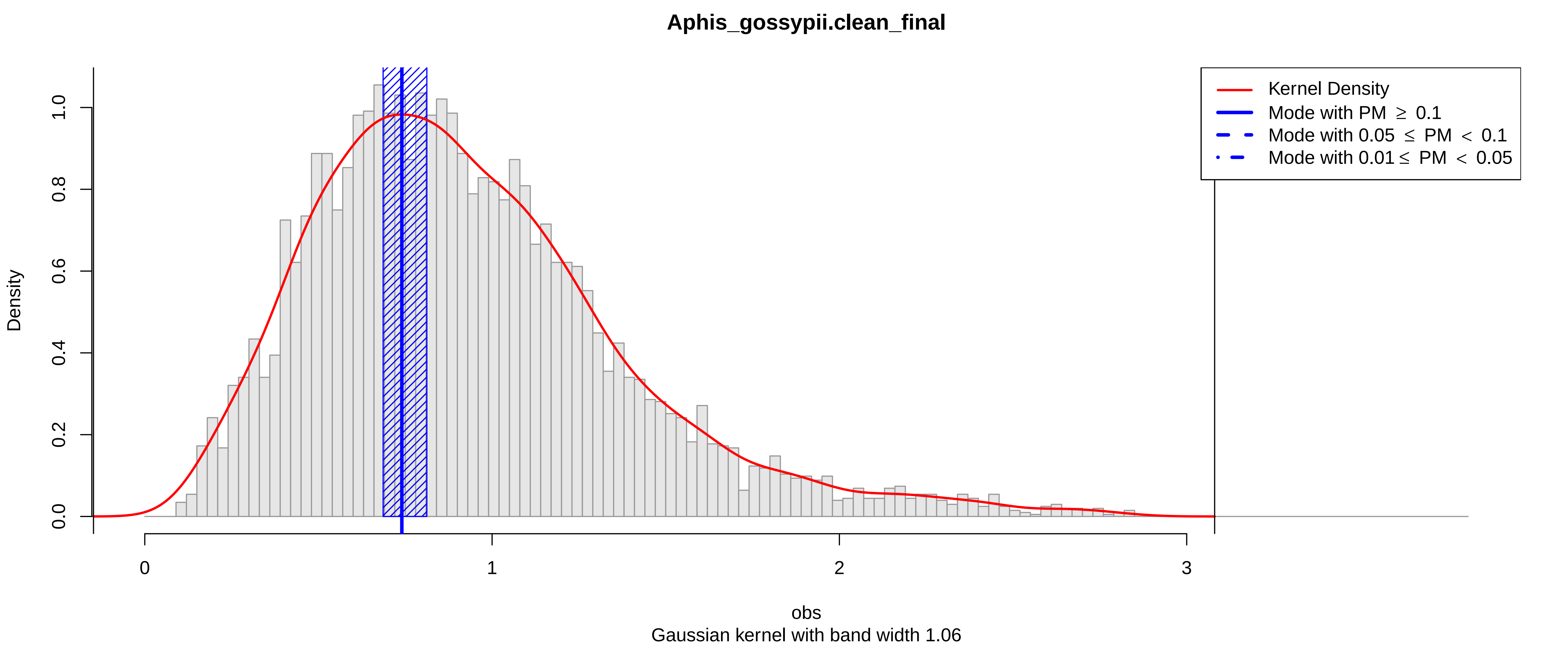

Aphis_gossypii.clean_final
Kernel Density
Mode with PM ≥ 0.1
Mode with 0.05 ≤ PM < 0.1
Mode with 0.01 ≤ PM < 0.05
Density
0.0
0.2
0.4
0.6
0.8
1.0
0
1
2
3
obs
Gaussian kernel with band width 1.06

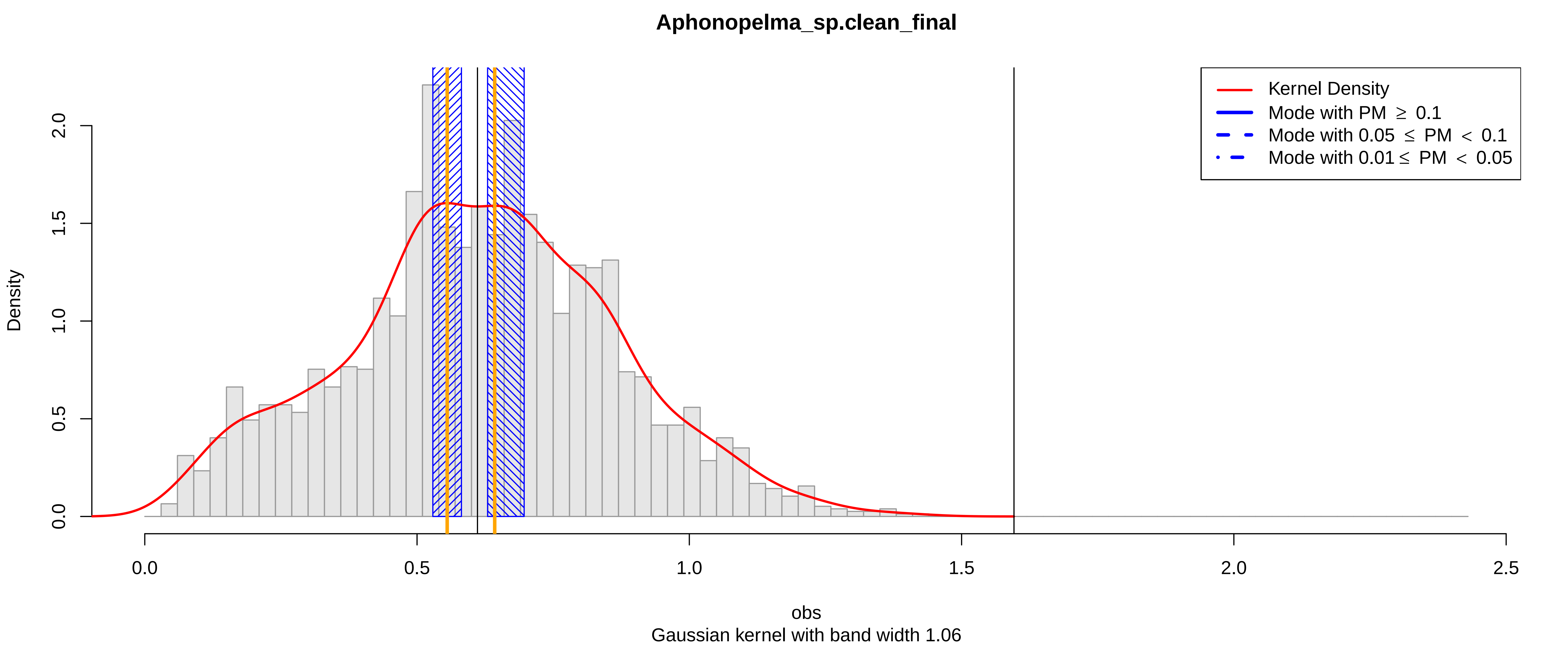
Aphonopelma_sp.clean_final
Kernel Density
Mode with PM ≥ 0.1
Mode with 0.05 ≤ PM < 0.1
Mode with 0.01 ≤ PM < 0.05
Density
0.0
0.5
1.0
1.5
2.0
0.0
0.5
1.0
1.5
2.0
2.5
obs
Gaussian kernel with band width 1.06

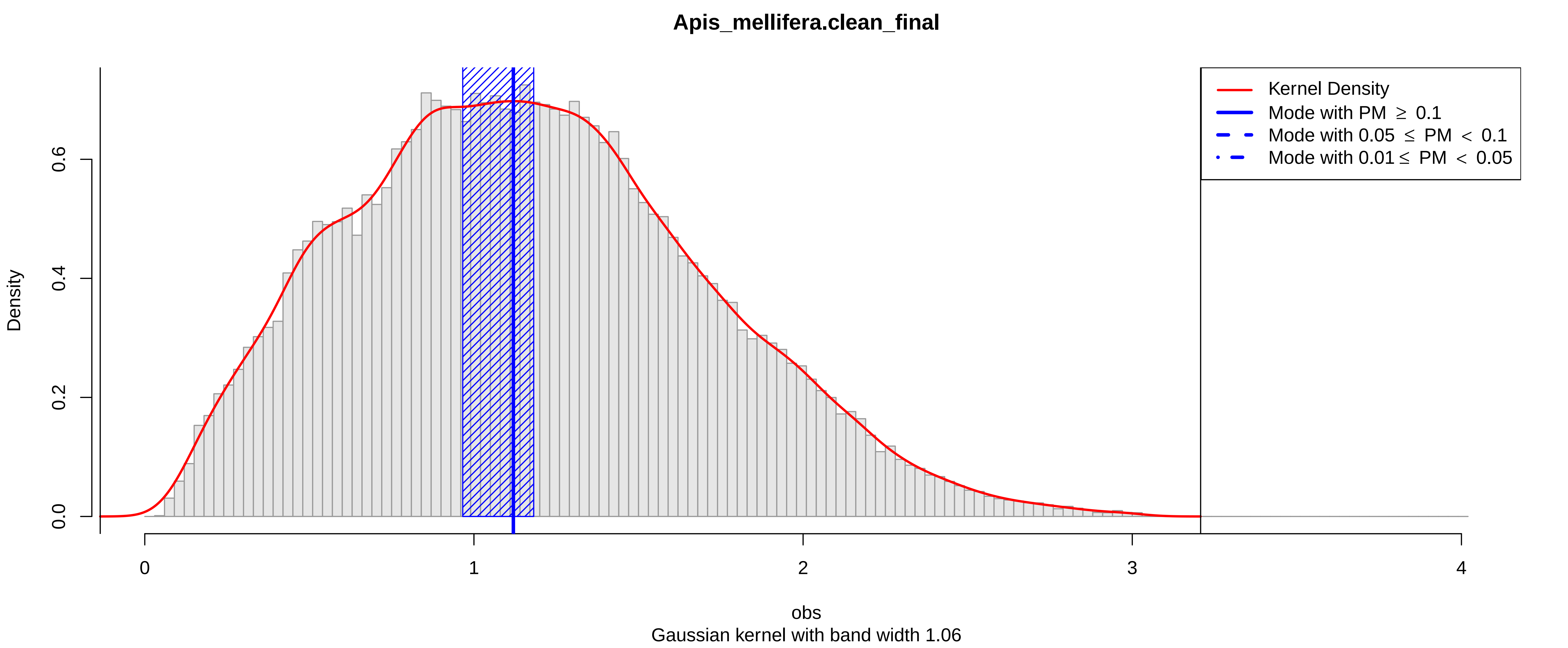
Apis_mellifera.clean_final
Density
0.0
0.2
0.4
0.6
0
1
2
3
4
obs
Gaussian kernel with band width 1.06
Kernel Density
Mode with PM ≥ 0.1
Mode with 0.05 ≤ PM < 0.1
Mode with 0.01 ≤ PM < 0.05

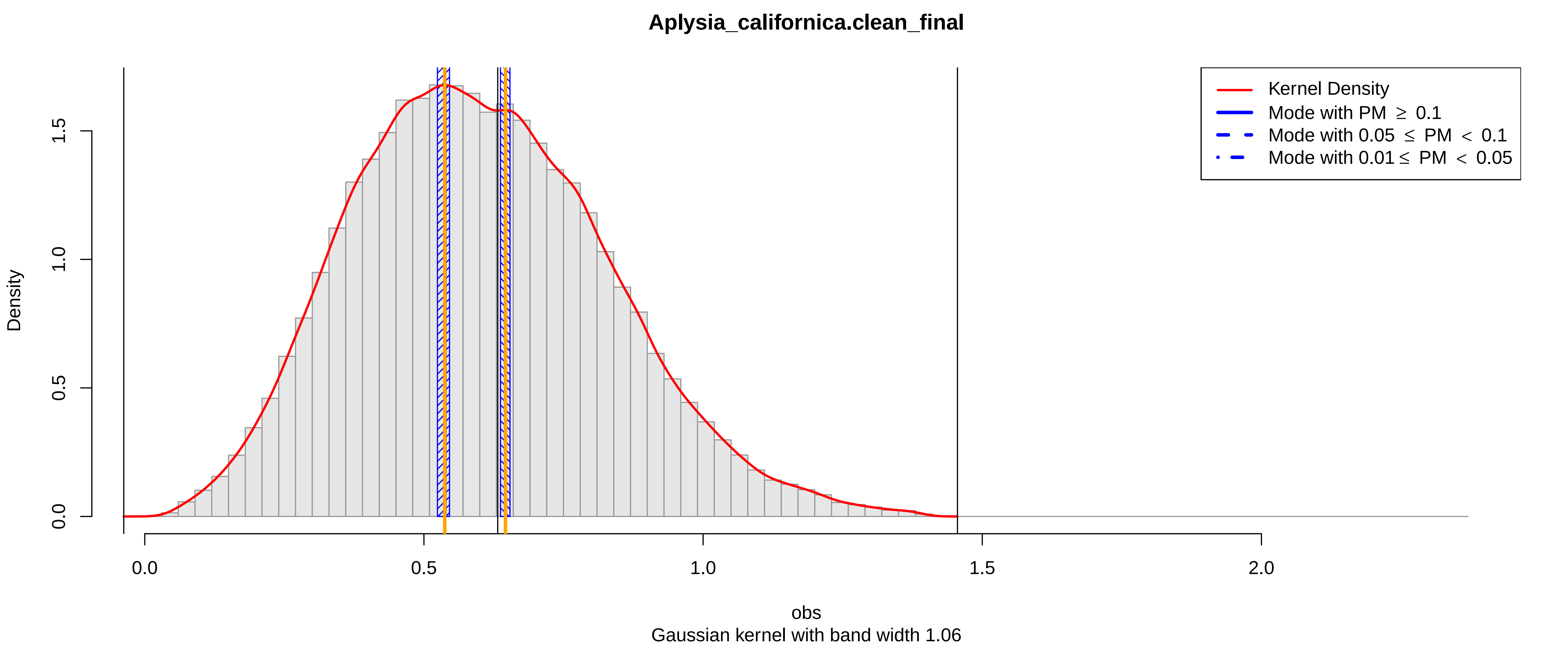

Aplysia_californica.clean_final
Kernel Density
Mode with PM ≥ 0.1
Mode with 0.05 ≤ PM < 0.1
Mode with 0.01 ≤ PM < 0.05
Density
0.0
0.5
1.0
1.5
0.0
0.5
1.0
1.5
2.0
obs
Gaussian kernel with band width 1.06

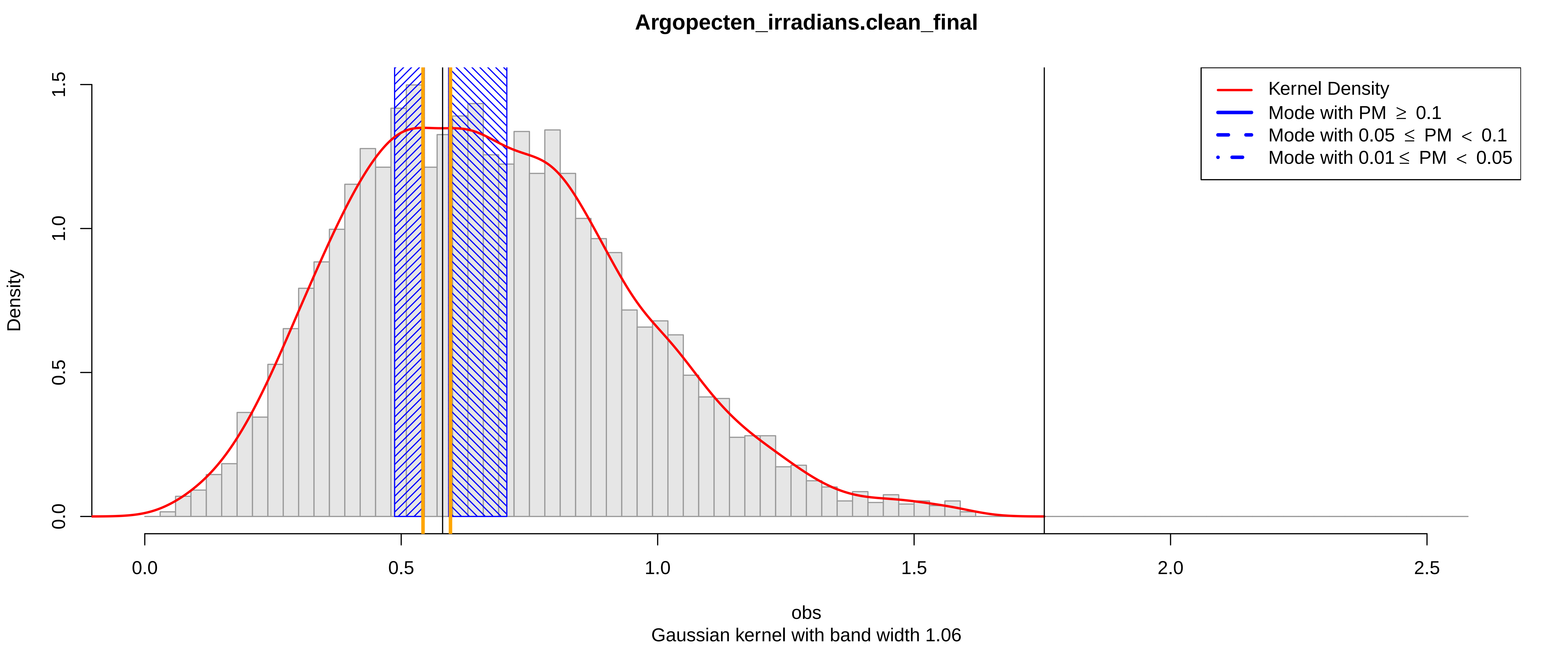

Argopecten_irradians.clean_final
Kernel Density
Mode with PM ≥ 0.1
Mode with 0.05 ≤ PM < 0.1
Mode with 0.01 ≤ PM < 0.05
Density
0.0
0.5
1.0
1.5
0.0
0.5
1.0
1.5
2.0
2.5
obs
Gaussian kernel with band width 1.06

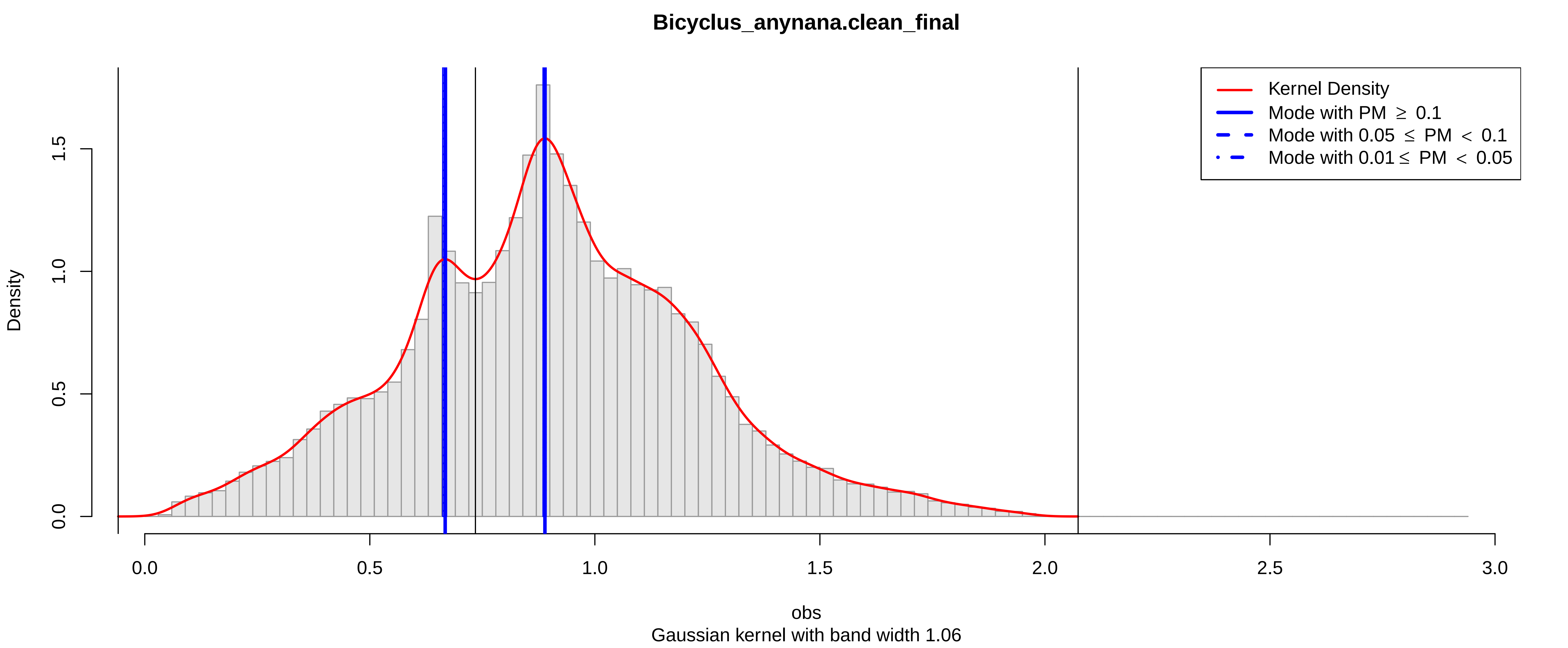

Bicyclus_anynana.clean_final
Kernel Density
Mode with PM ≥ 0.1
Mode with 0.05 ≤ PM < 0.1
Mode with 0.01 ≤ PM < 0.05
Density
0.0
0.5
1.0
1.5
0.0
0.5
1.0
1.5
2.0
2.5
3.0
obs
Gaussian kernel with band width 1.06

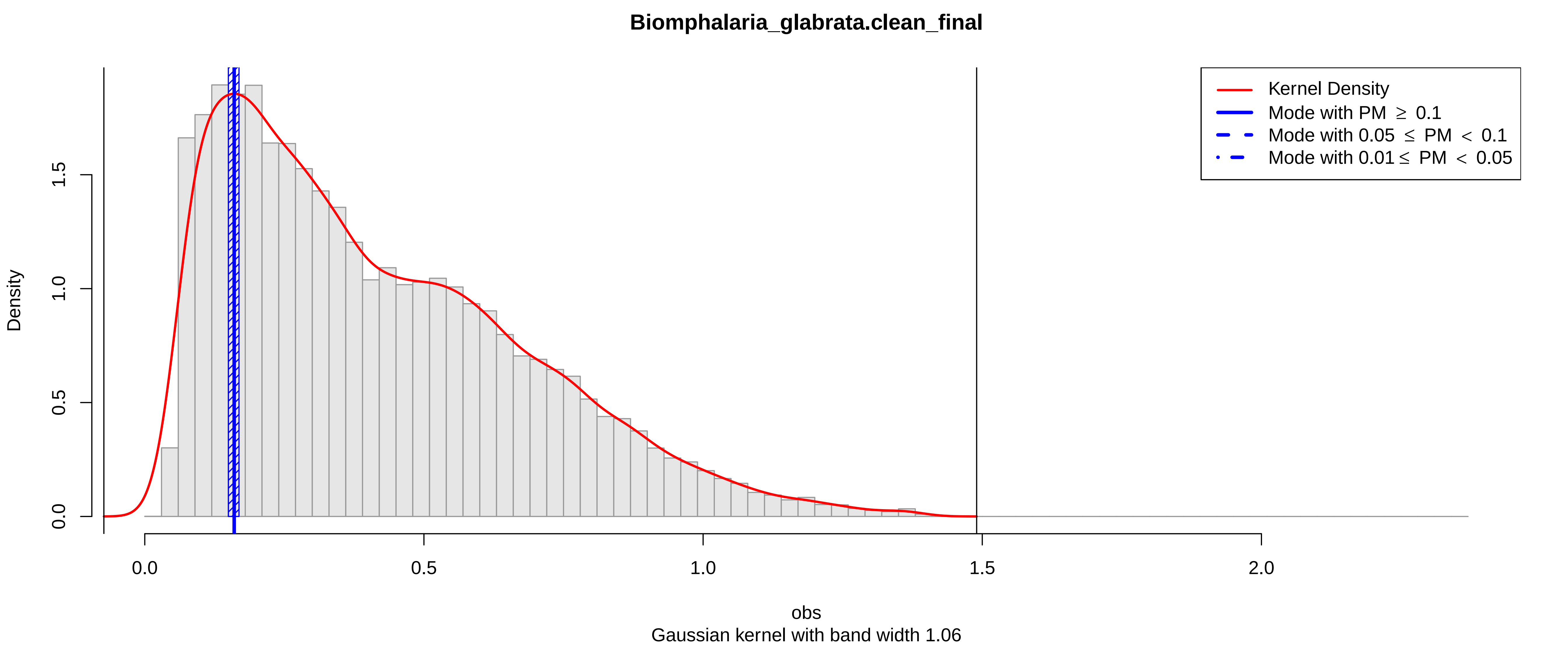

Biomphalaria_glabrata.clean_final
Kernel Density
Mode with PM ≥ 0.1
Mode with 0.05 ≤ PM < 0.1
Mode with 0.01 ≤ PM < 0.05
Density
0.0
0.5
1.0
1.5
2.0
obs
Gaussian kernel with band width 1.06

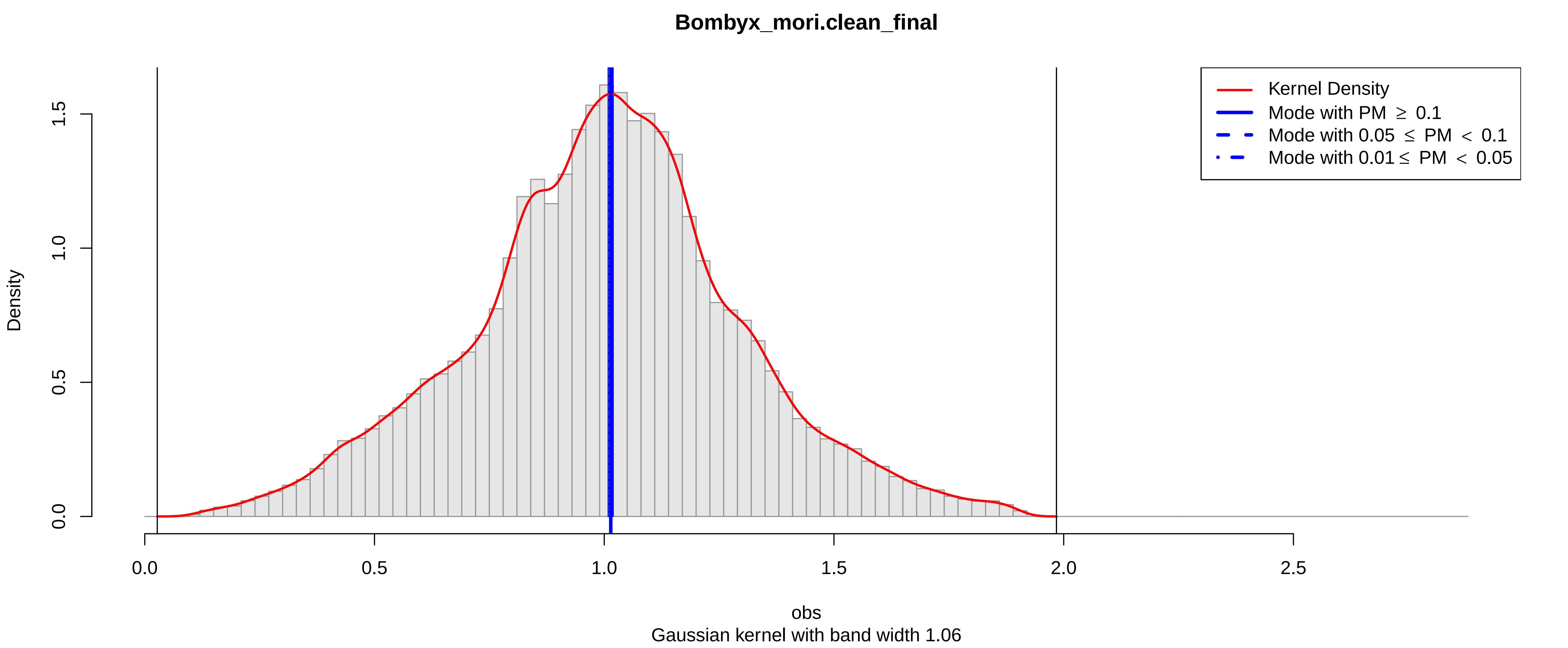
Bombyx_mori.clean_final
Kernel Density
Mode with PM ≥ 0.1
Mode with 0.05 ≤ PM < 0.1
Mode with 0.01 ≤ PM < 0.05
Density
0.0
0.5
1.0
1.5
0.0
0.5
1.0
1.5
2.0
2.5
obs
Gaussian kernel with band width 1.06

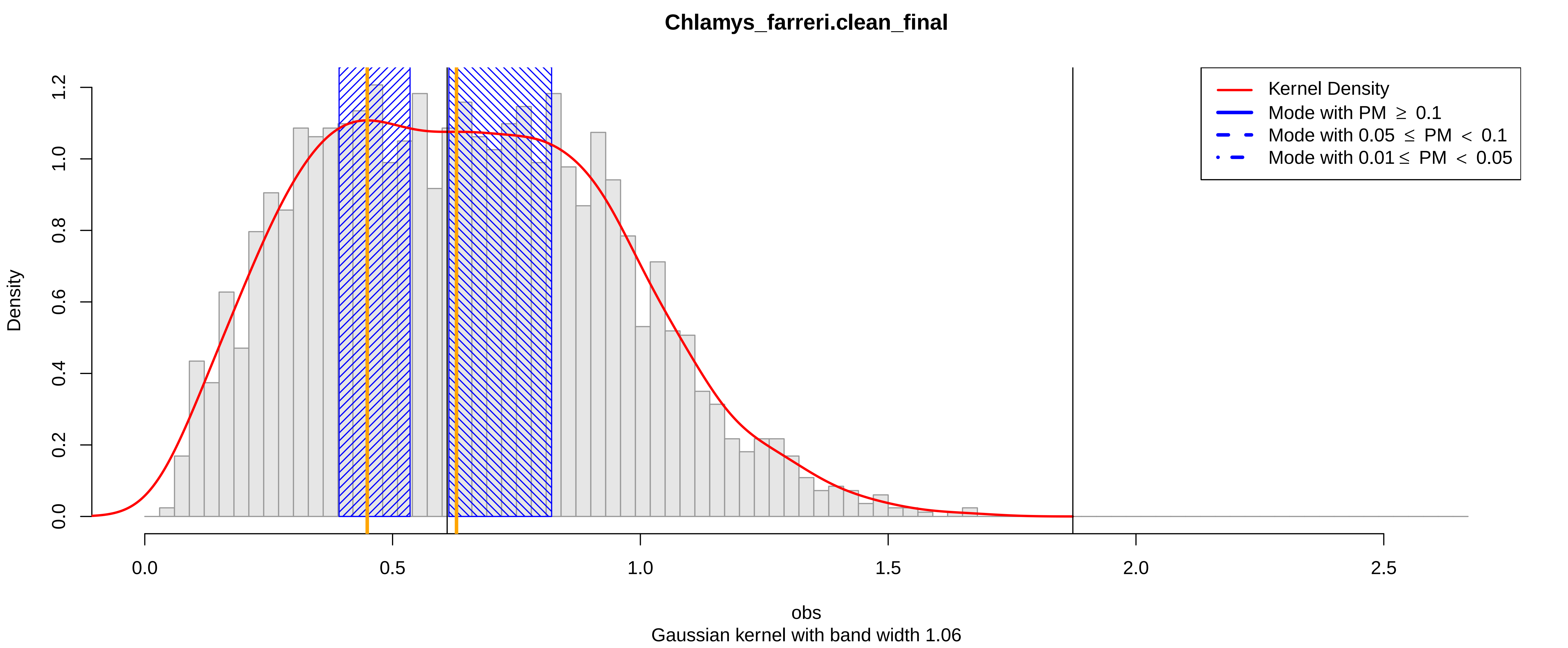
Chlamys_farreri.clean_final
Kernel Density
Mode with PM ≥ 0.1
Mode with 0.05 ≤ PM < 0.1
Mode with 0.01 ≤ PM < 0.05
Density
0.0
0.2
0.4
0.6
0.8
1.0
1.2
0.0
0.5
1.0
1.5
2.0
2.5
obs
Gaussian kernel with band width 1.06

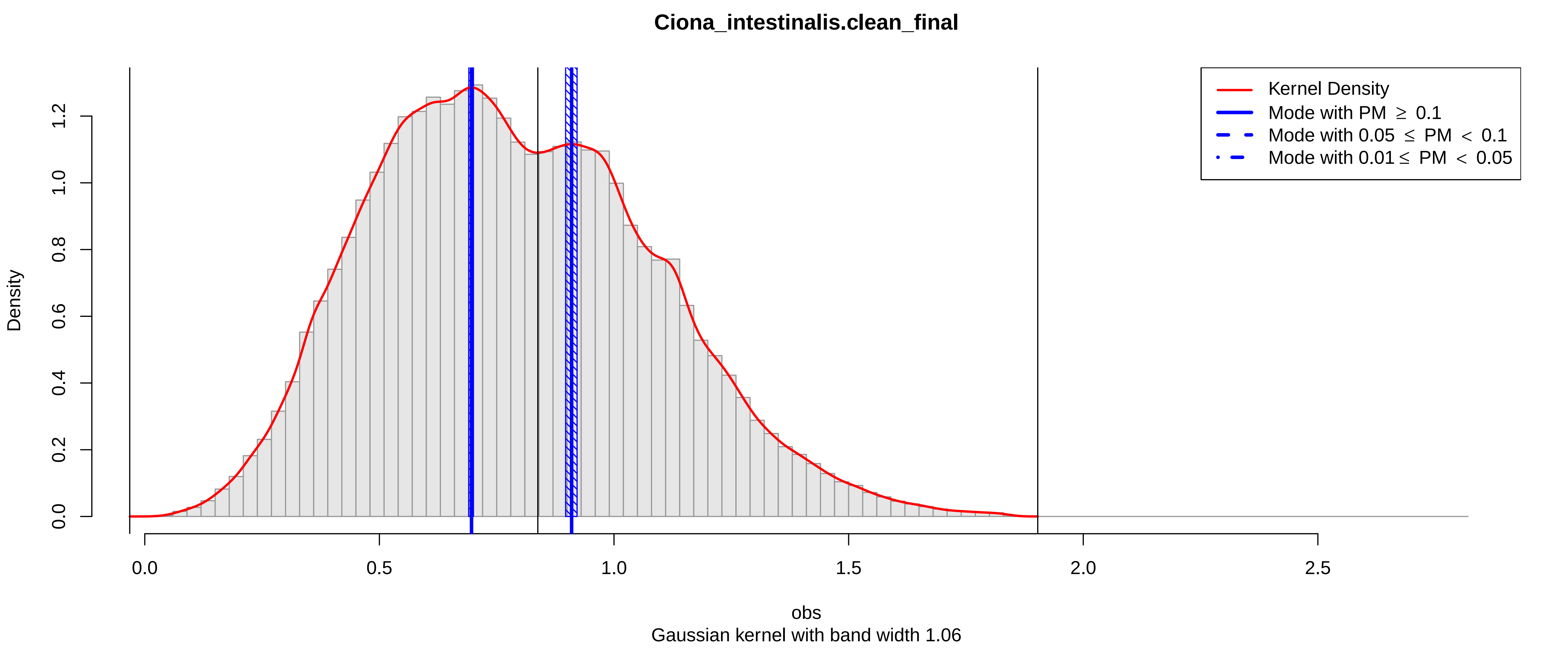

Ciona_intestinalis.clean_final
Density
0.0
0.2
0.4
0.6
0.8
1.0
1.2
0.0
0.5
1.0
1.5
2.0
2.5
obs
Gaussian kernel with band width 1.06
Kernel Density
Mode with PM ≥ 0.1
Mode with 0.05 ≤ PM < 0.1
Mode with 0.01 ≤ PM < 0.05

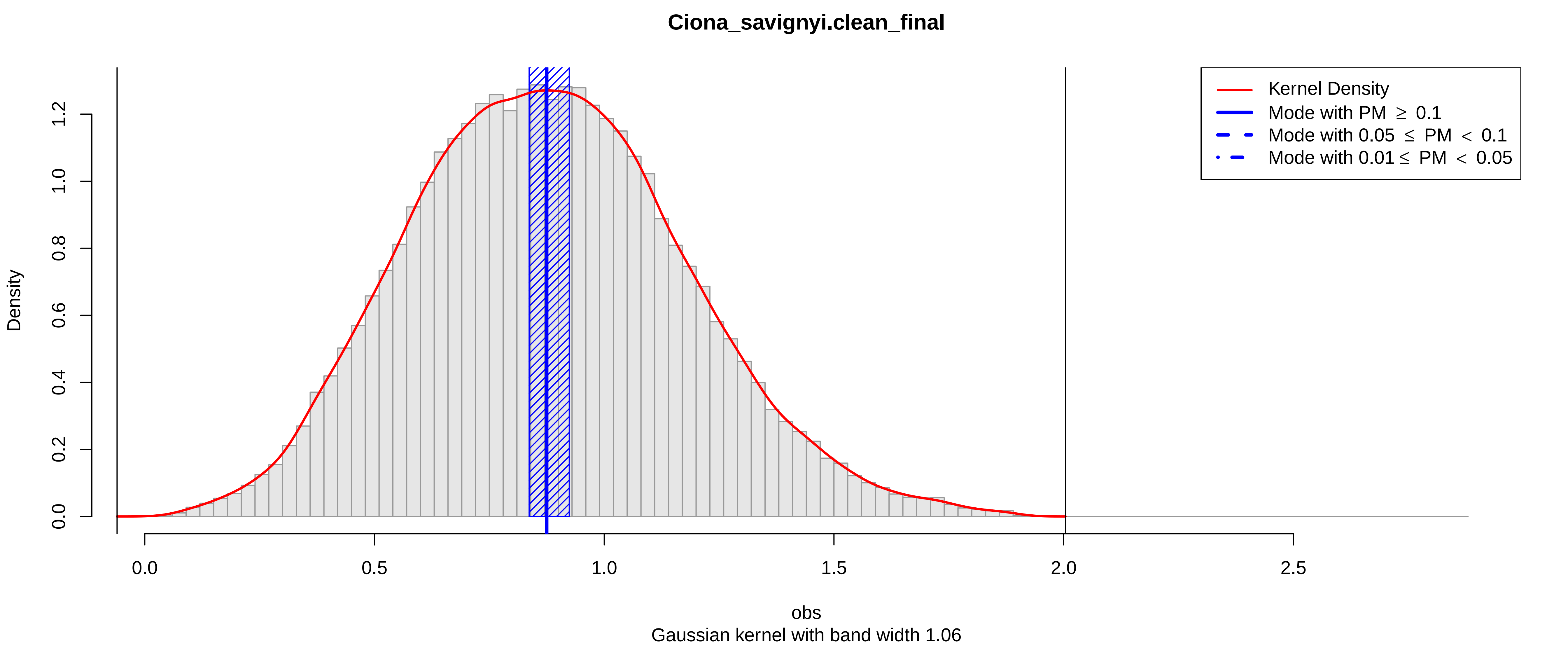

Ciona_savignyi.clean_final
Kernel Density
Mode with PM ≥ 0.1
Mode with 0.05 ≤ PM < 0.1
Mode with 0.01 ≤ PM < 0.05
Density
0.0
0.2
0.4
0.6
0.8
1.0
1.2
0.0
0.5
1.0
1.5
2.0
2.5
obs
Gaussian kernel with band width 1.06

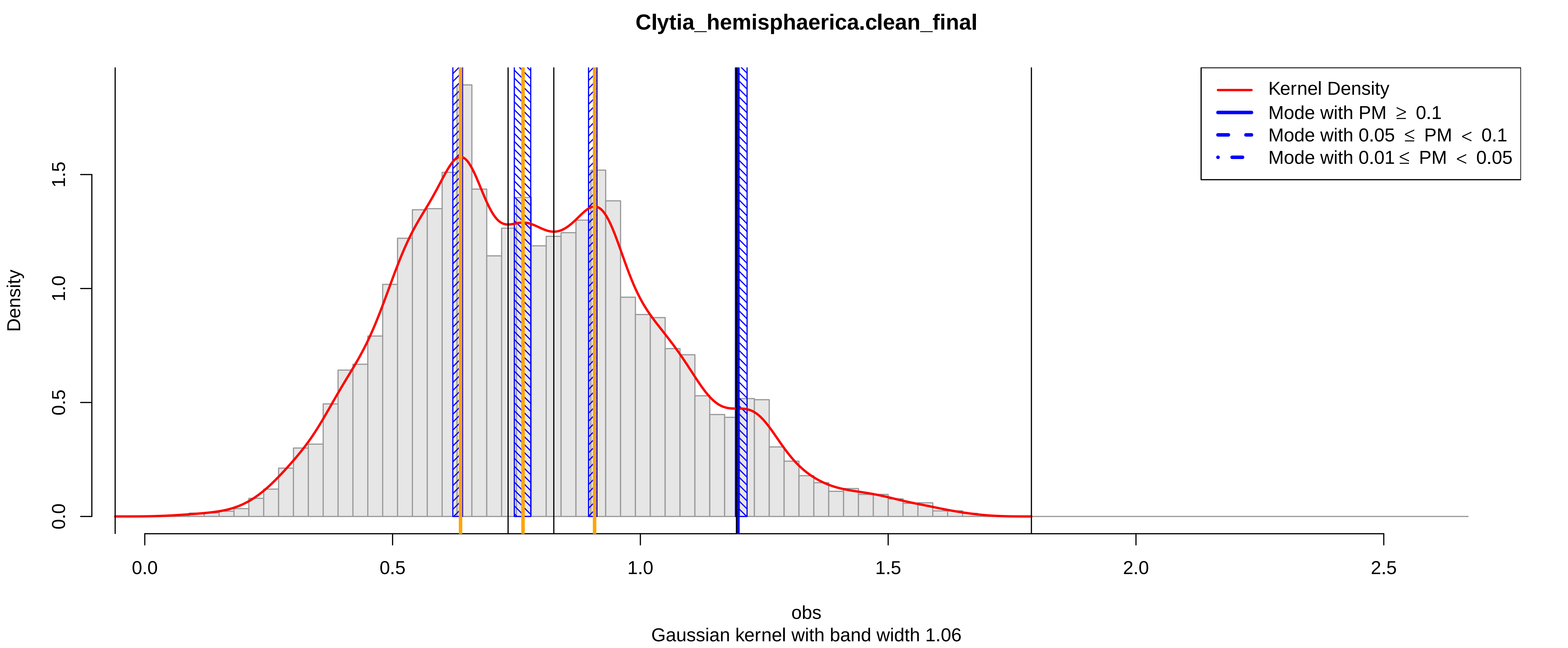
Clytia_hemisphaerica.clean_final
Density
0.0
0.5
1.0
1.5
0.0
0.5
1.0
1.5
2.0
2.5
obs
Gaussian kernel with band width 1.06
Kernel Density
Mode with PM ≥ 0.1
Mode with 0.05 ≤ PM < 0.1
Mode with 0.01 ≤ PM < 0.05

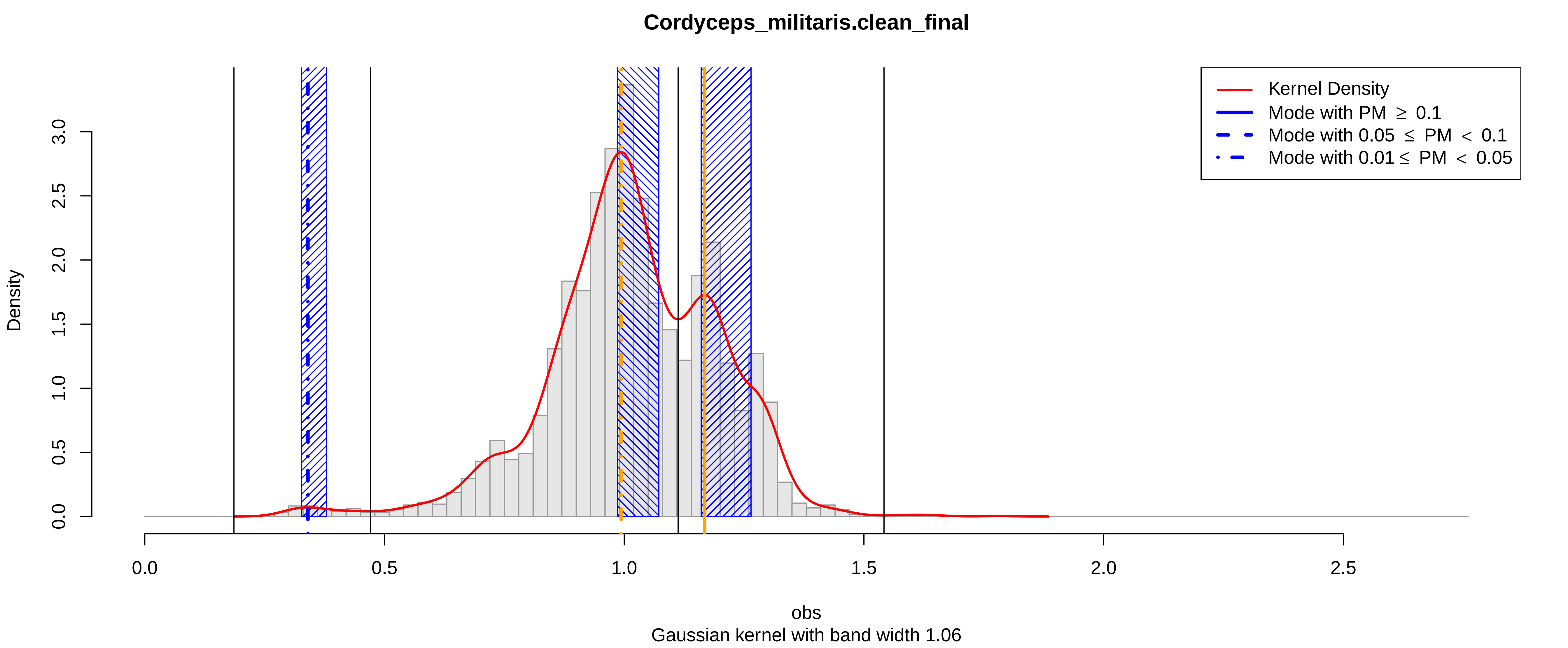
Cordyceps_militaris.clean_final
Kernel Density
Mode with PM ≥ 0.1
Mode with 0.05 ≤ PM < 0.1
Mode with 0.01 ≤ PM < 0.05
Density
0.0
0.5
1.0
1.5
2.0
2.5
3.0
obs
Gaussian kernel with band width 1.06

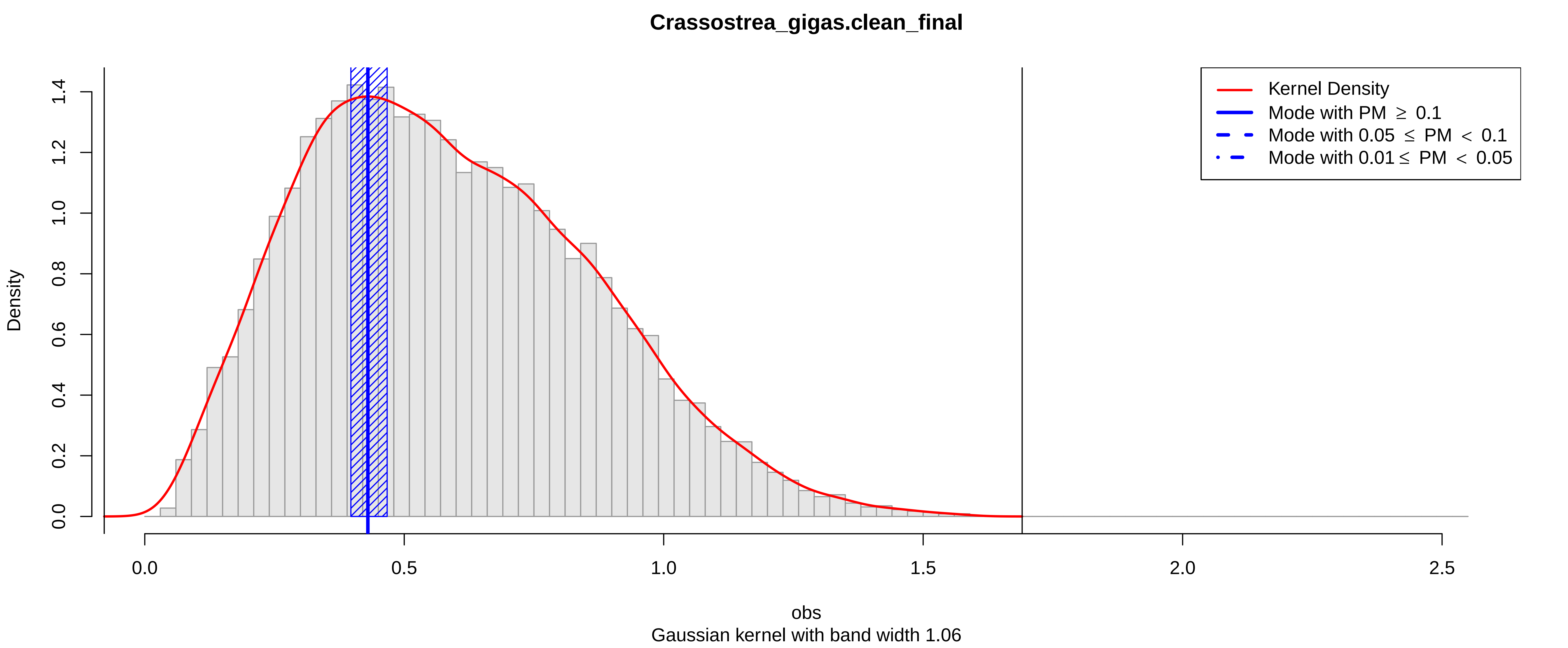

Crassostrea_gigas.clean_final
Kernel Density
Mode with PM ≥ 0.1
Mode with 0.05 ≤ PM < 0.1
Mode with 0.01 ≤ PM < 0.05
Density
0.0
0.2
0.4
0.6
0.8
1.0
1.2
1.4
0.0
0.5
1.0
1.5
2.0
2.5
obs
Gaussian kernel with band width 1.06

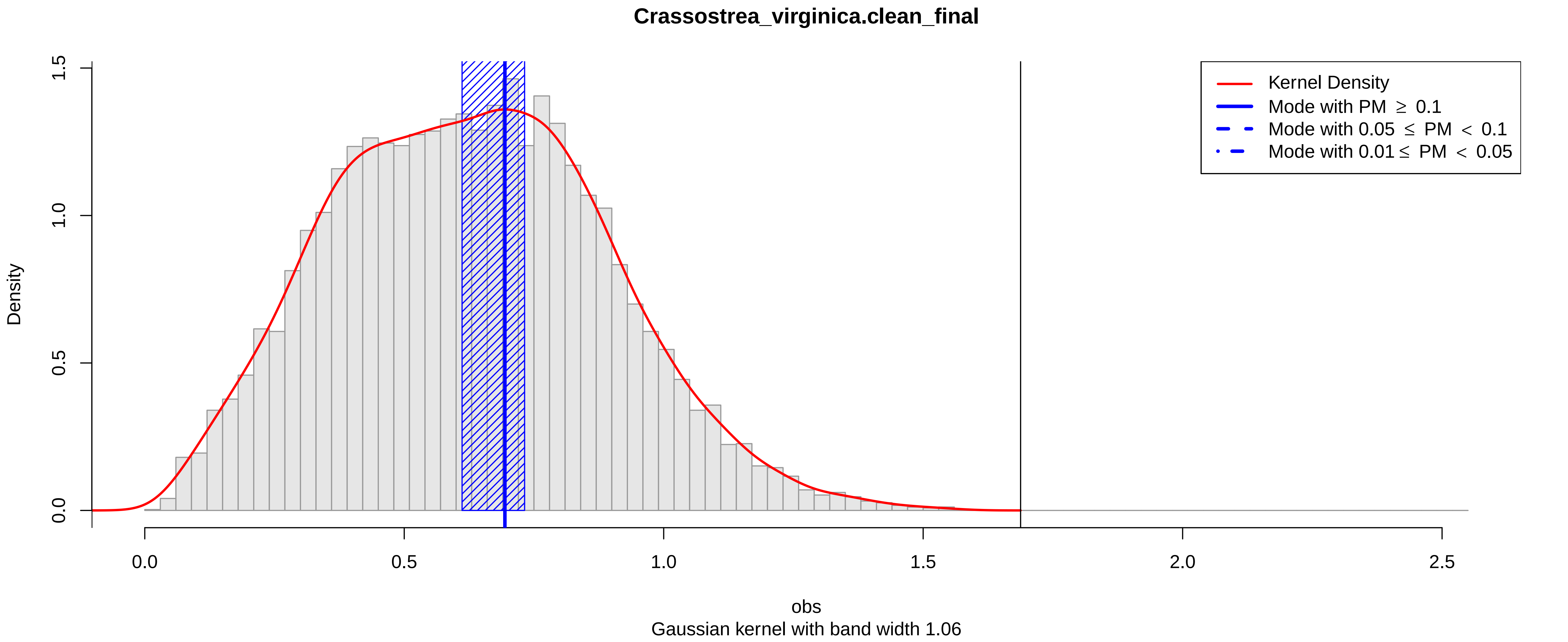
Crassostrea_virginica.clean_final
Kernel Density
Mode with PM ≥ 0.1
Mode with 0.05 ≤ PM < 0.1
Mode with 0.01 ≤ PM < 0.05
Density
0.0
0.5
1.0
1.5
0.0
0.5
1.0
1.5
2.0
2.5
obs
Gaussian kernel with band width 1.06

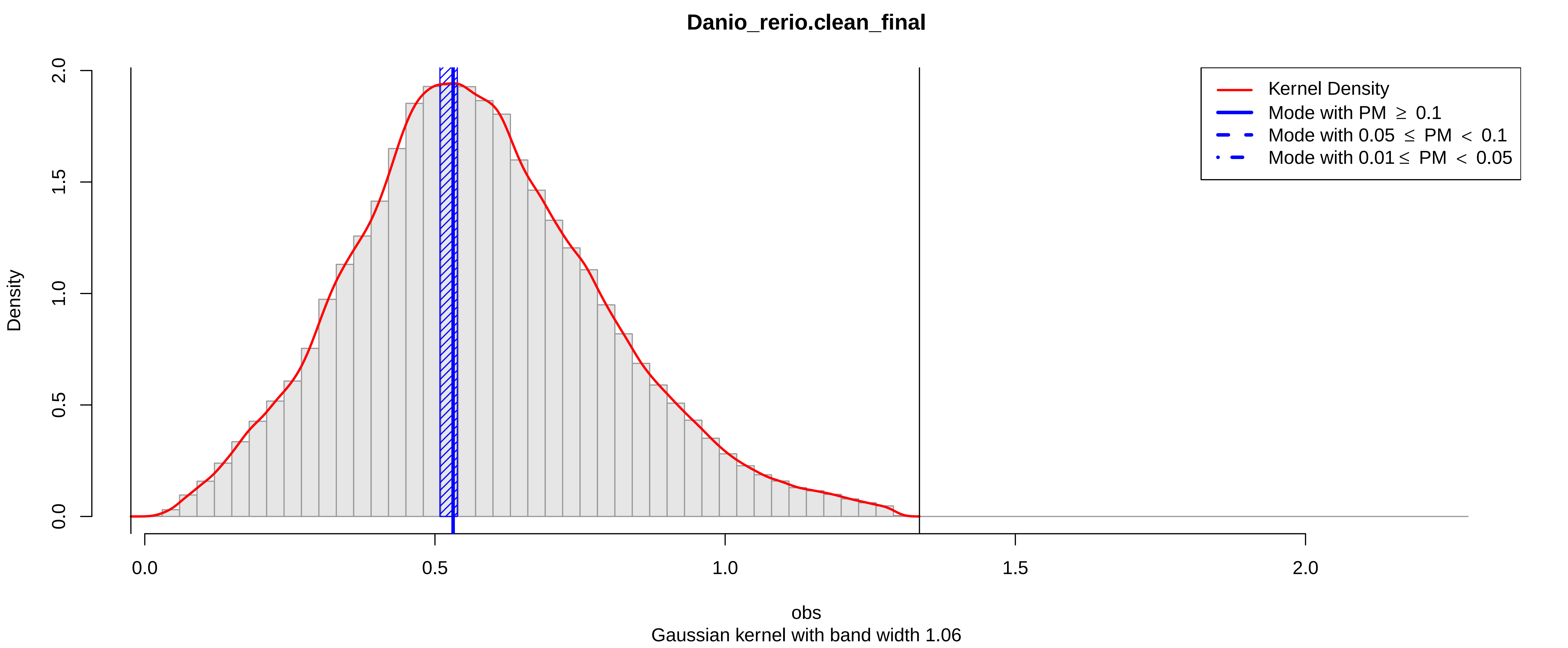
Danio_rerio.clean_final
Density
0.0
0.5
1.0
1.5
2.0
obs
Gaussian kernel with band width 1.06
Kernel Density
Mode with PM ≥ 0.1
Mode with 0.05 ≤ PM < 0.1
Mode with 0.01 ≤ PM < 0.05

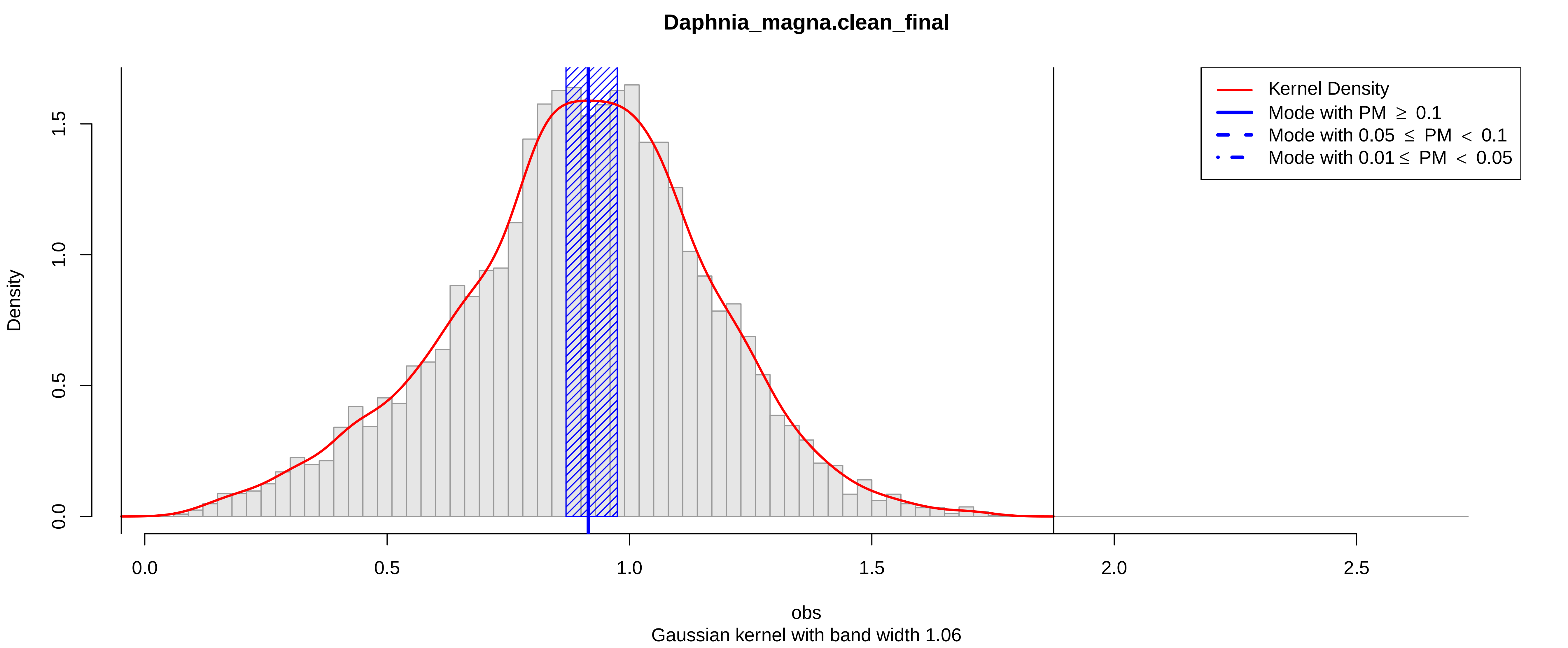
Daphnia_magna.clean_final
Kernel Density
Mode with PM ≥ 0.1
Mode with 0.05 ≤ PM < 0.1
Mode with 0.01 ≤ PM < 0.05
Density
0.0
0.5
1.0
1.5
0.0
0.5
1.0
1.5
2.0
2.5
obs
Gaussian kernel with band width 1.06

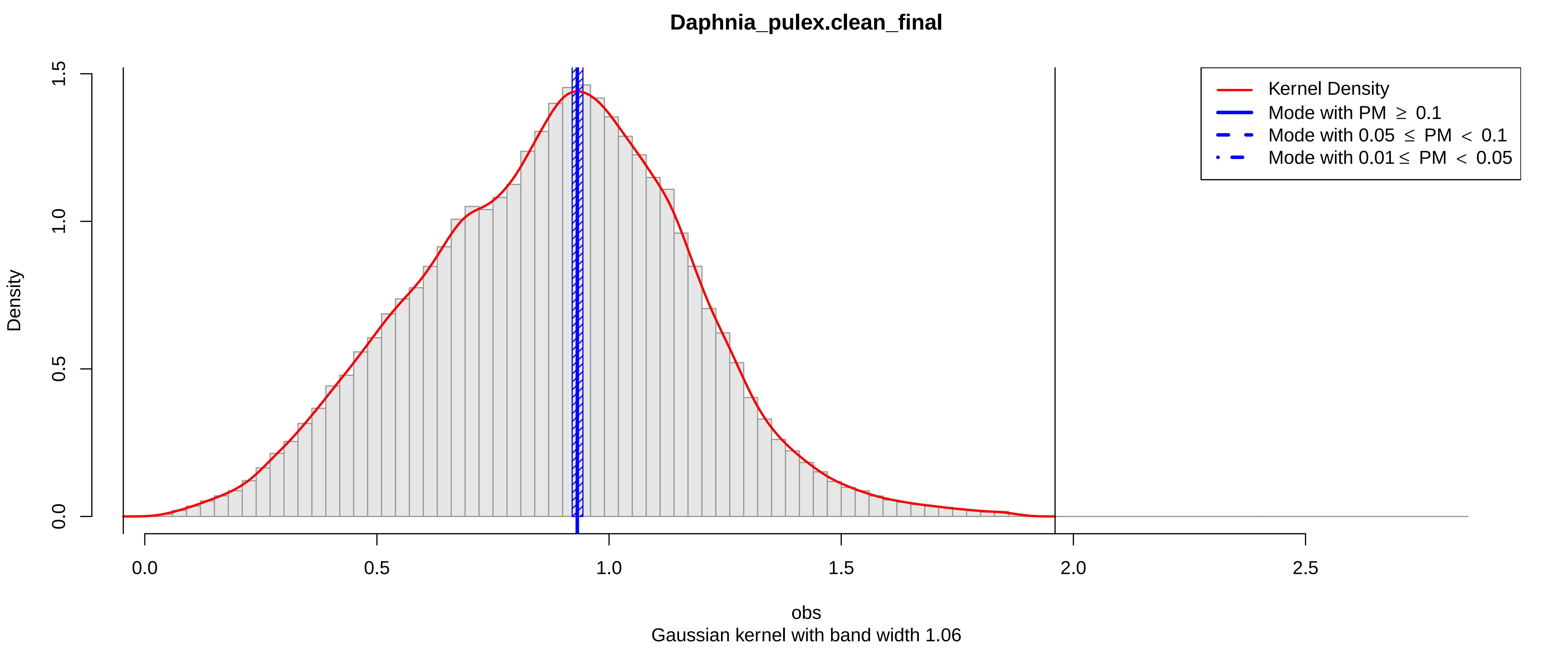
Daphnia_pulex.clean_final
Kernel Density
Mode with PM ≥ 0.1
Mode with 0.05 ≤ PM < 0.1
Mode with 0.01 ≤ PM < 0.05
Density
0.0
0.5
1.0
1.5
0.0
0.5
1.0
1.5
2.0
2.5
obs
Gaussian kernel with band width 1.06

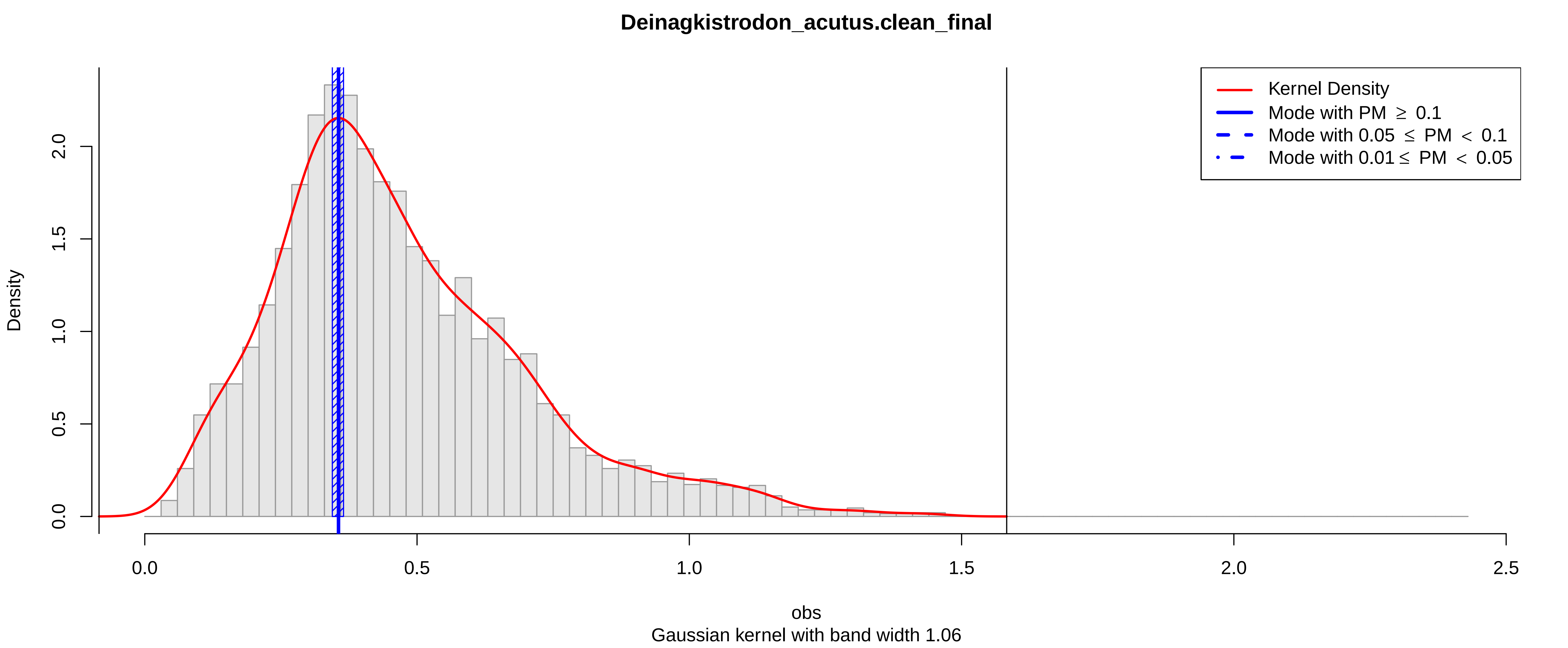

Deinagkistrodon_acutus.clean_final
Kernel Density
Mode with PM ≥ 0.1
Mode with 0.05 ≤ PM < 0.1
Mode with 0.01 ≤ PM < 0.05
Density
0.0
0.5
1.0
1.5
2.0
0.0
0.5
1.0
1.5
2.0
2.5
obs
Gaussian kernel with band width 1.06

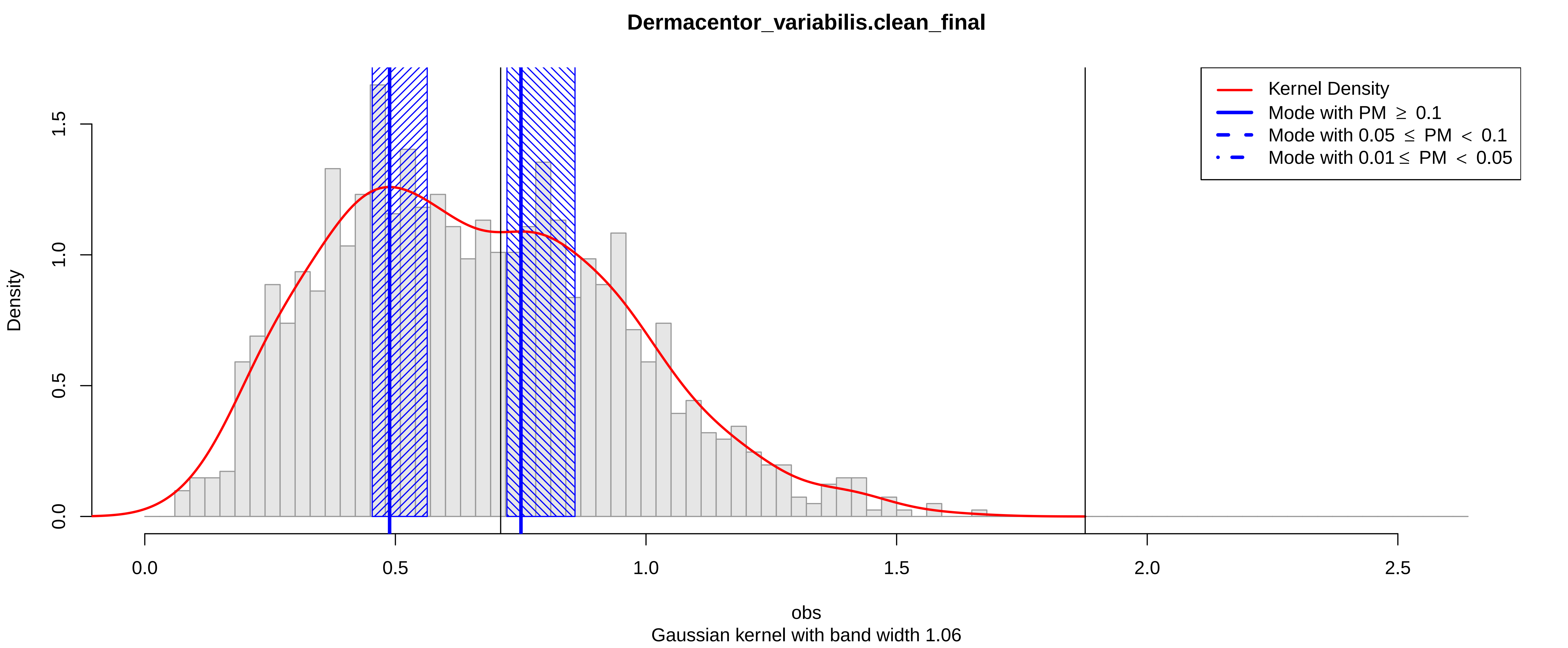
Dermacentor_variabilis.clean_final
Kernel Density
Mode with PM ≥ 0.1
Mode with 0.05 ≤ PM < 0.1
Mode with 0.01 ≤ PM < 0.05
Density
0.0
0.5
1.0
1.5
0.0
0.5
1.0
1.5
2.0
2.5
obs
Gaussian kernel with band width 1.06

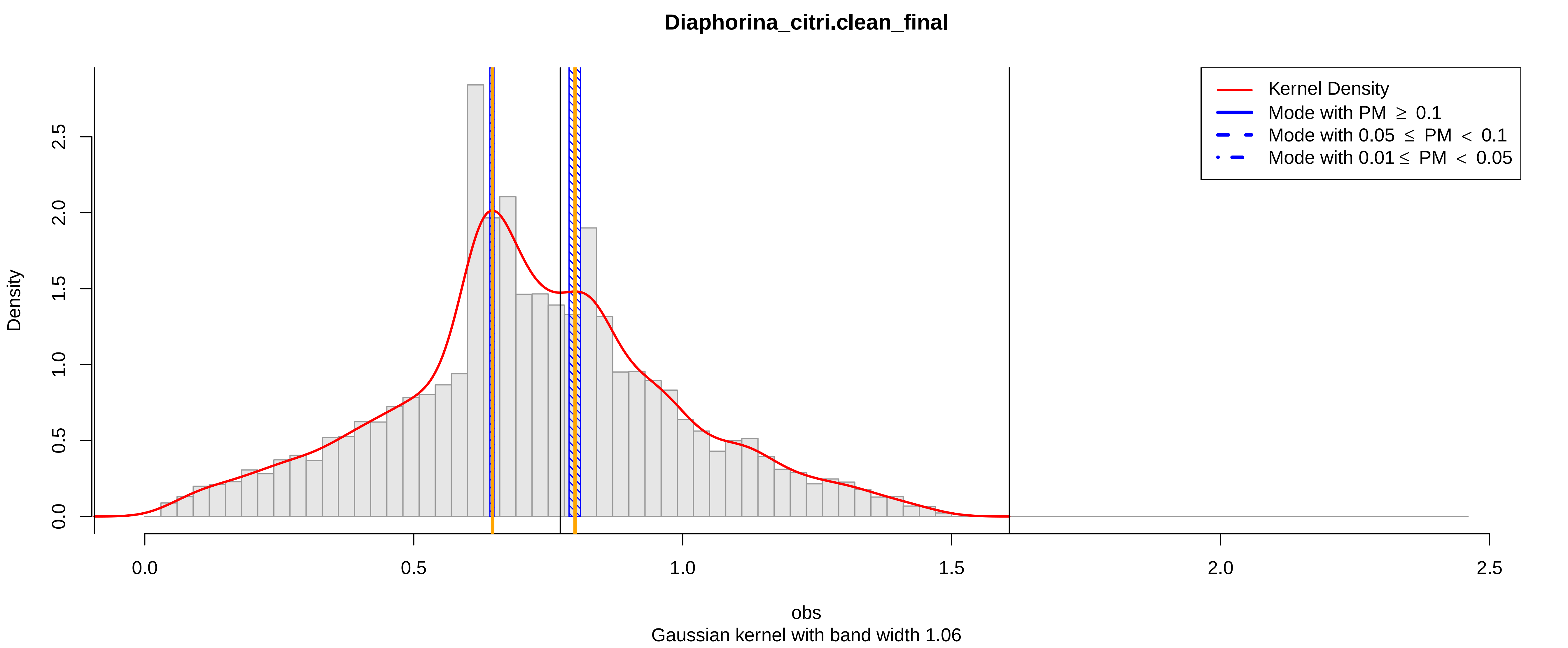
Diaphorina_citri.clean_final
Kernel Density
Mode with PM ≥ 0.1
Mode with 0.05 ≤ PM < 0.1
Mode with 0.01 ≤ PM < 0.05
Density
0.0
0.5
1.0
1.5
2.0
2.5
obs
Gaussian kernel with band width 1.06

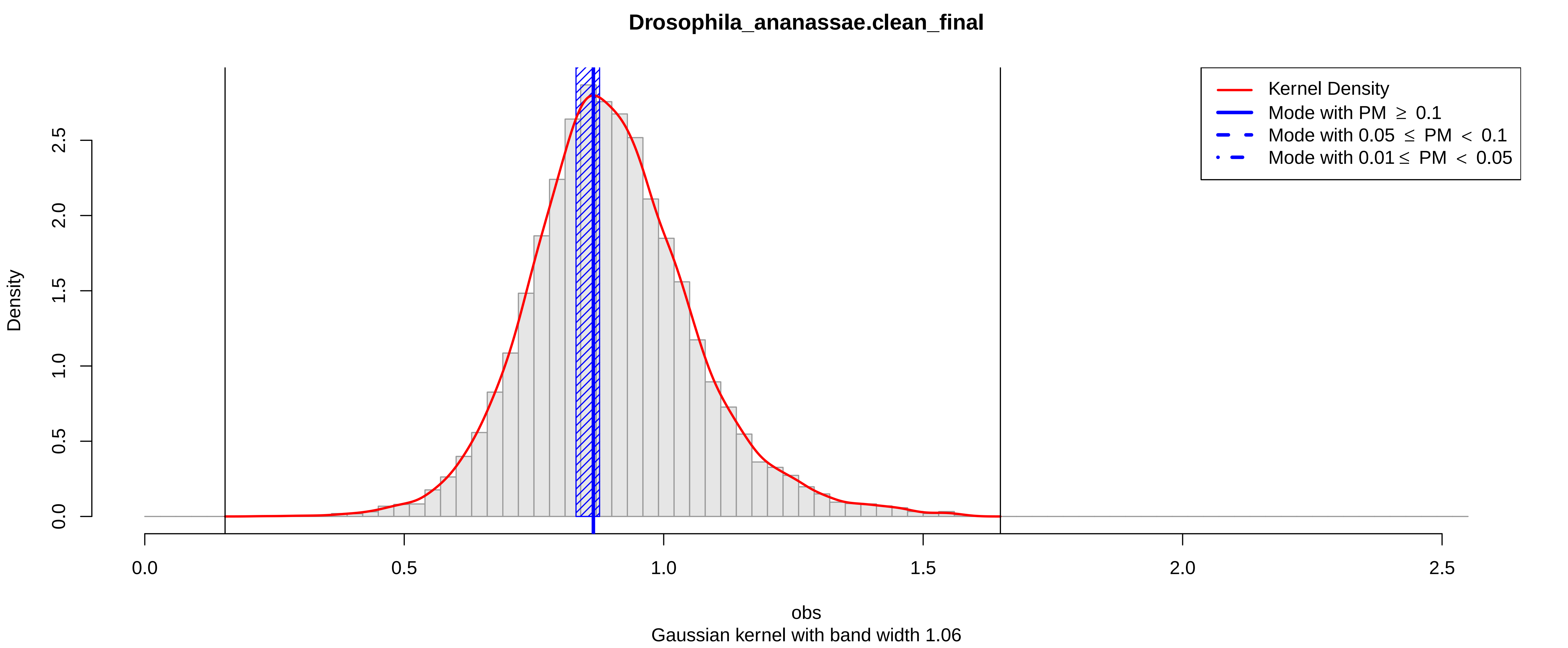

Drosophila_ananassae.clean_final
Kernel Density
Mode with PM ≥ 0.1
Mode with 0.05 ≤ PM < 0.1
Mode with 0.01 ≤ PM < 0.05
Density
0.0
0.5
1.0
1.5
2.0
2.5
obs
Gaussian kernel with band width 1.06

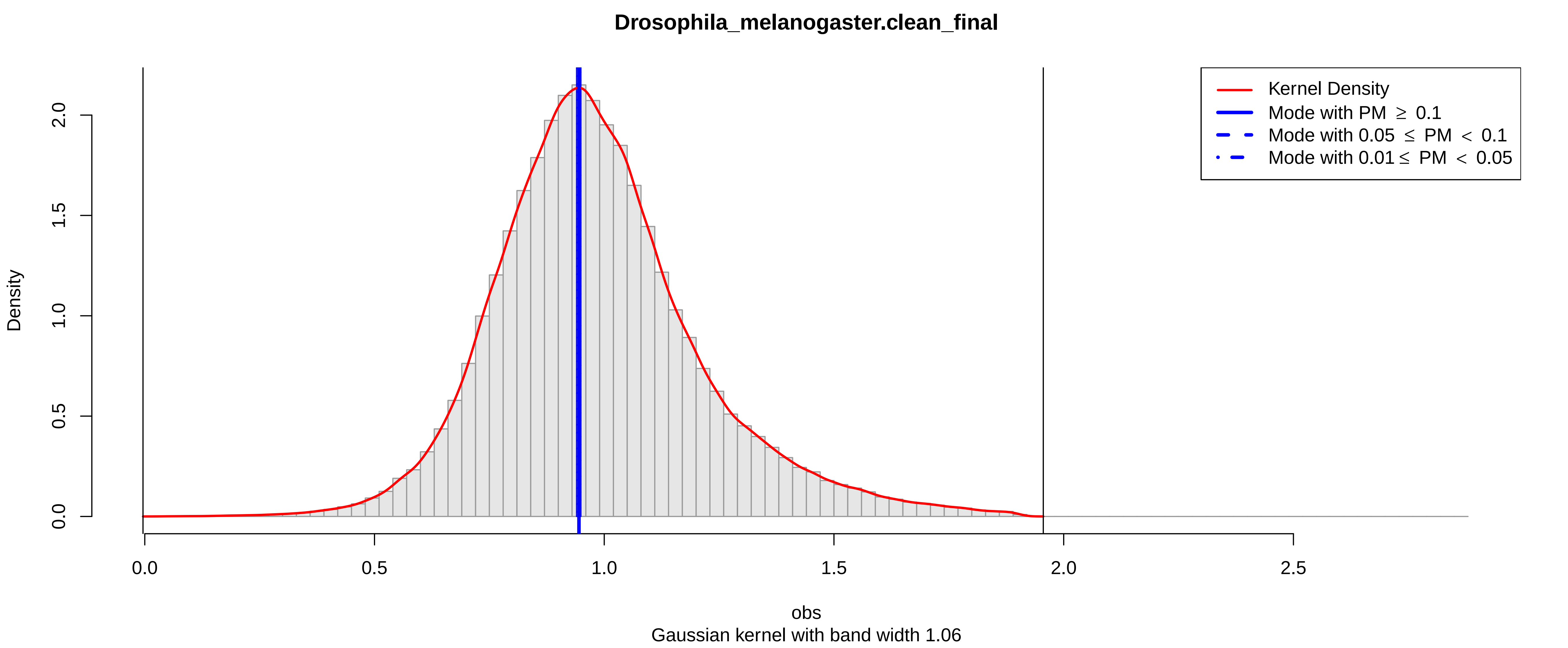
Drosophila_melanogaster.clean_final
Kernel Density
Mode with PM ≥ 0.1
Mode with 0.05 ≤ PM < 0.1
Mode with 0.01 ≤ PM < 0.05
Density
0.0
0.5
1.0
1.5
2.0
0.0
0.5
1.0
1.5
2.0
2.5
obs
Gaussian kernel with band width 1.06

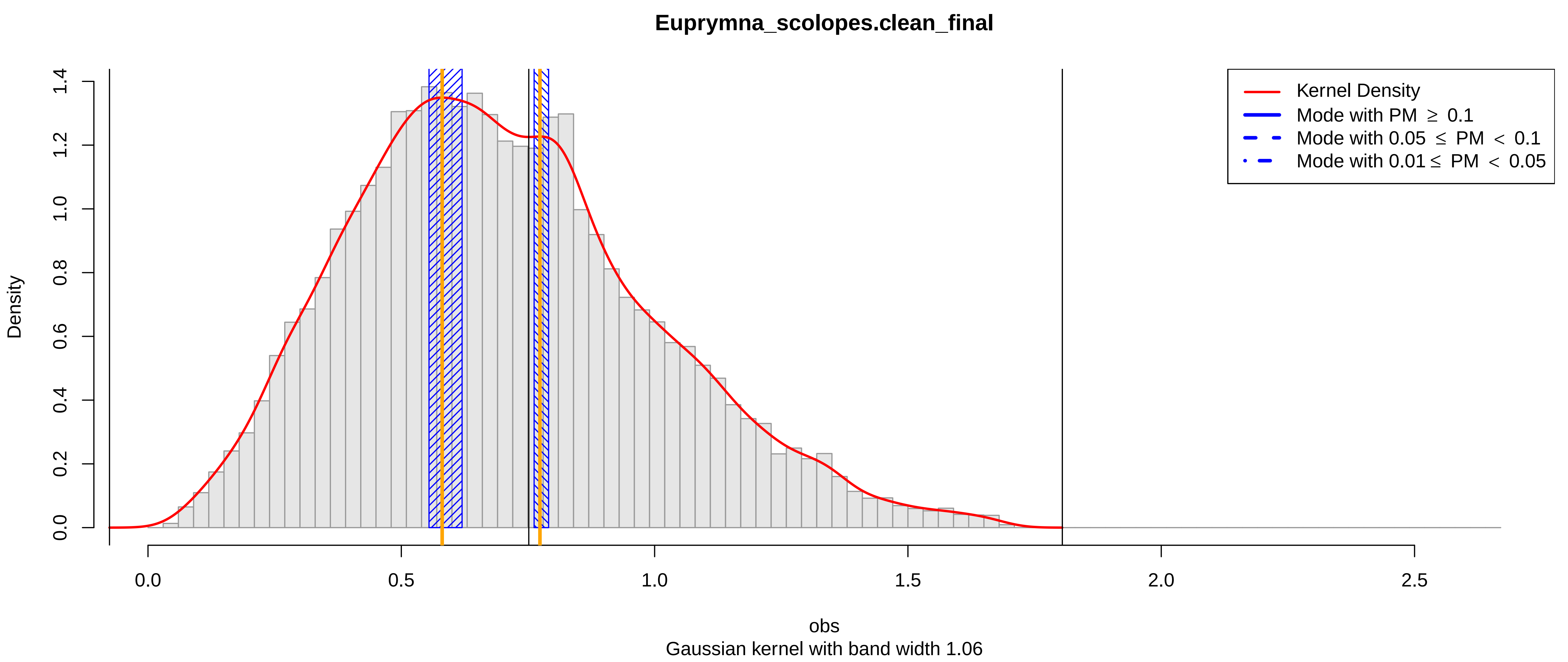
Euprymna_scolopes.clean_final
Kernel Density
Mode with PM ≥ 0.1
Mode with 0.05 ≤ PM < 0.1
Mode with 0.01 ≤ PM < 0.05
Density
0.0
0.2
0.4
0.6
0.8
1.0
1.2
1.4
0.0
0.5
1.0
1.5
2.0
2.5
obs
Gaussian kernel with band width 1.06

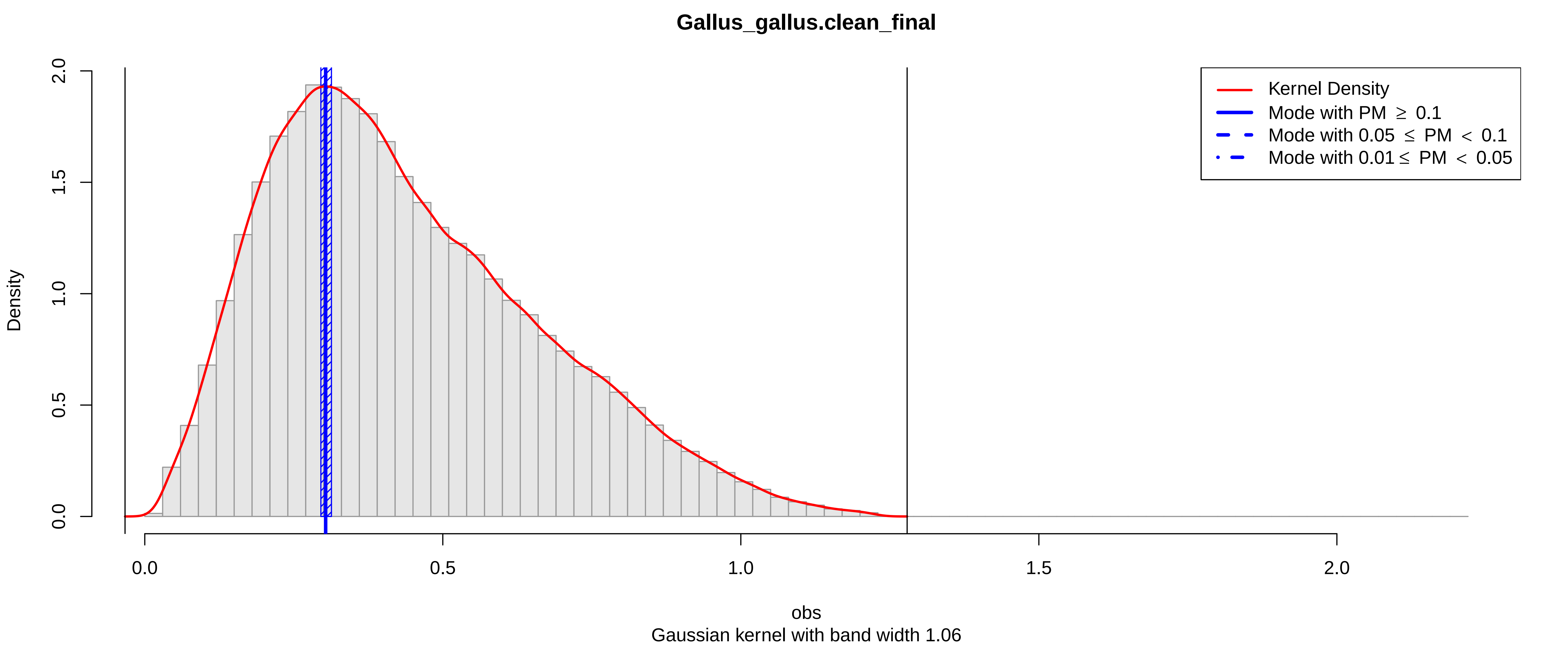
Gallus_gallus.clean_final
Density
0.0
0.5
1.0
1.5
2.0
Kernel Density
Mode with PM ≥ 0.1
Mode with 0.05 ≤ PM < 0.1
Mode with 0.01 ≤ PM < 0.05
0.0
0.5
1.0
1.5
2.0
obs
Gaussian kernel with band width 1.06

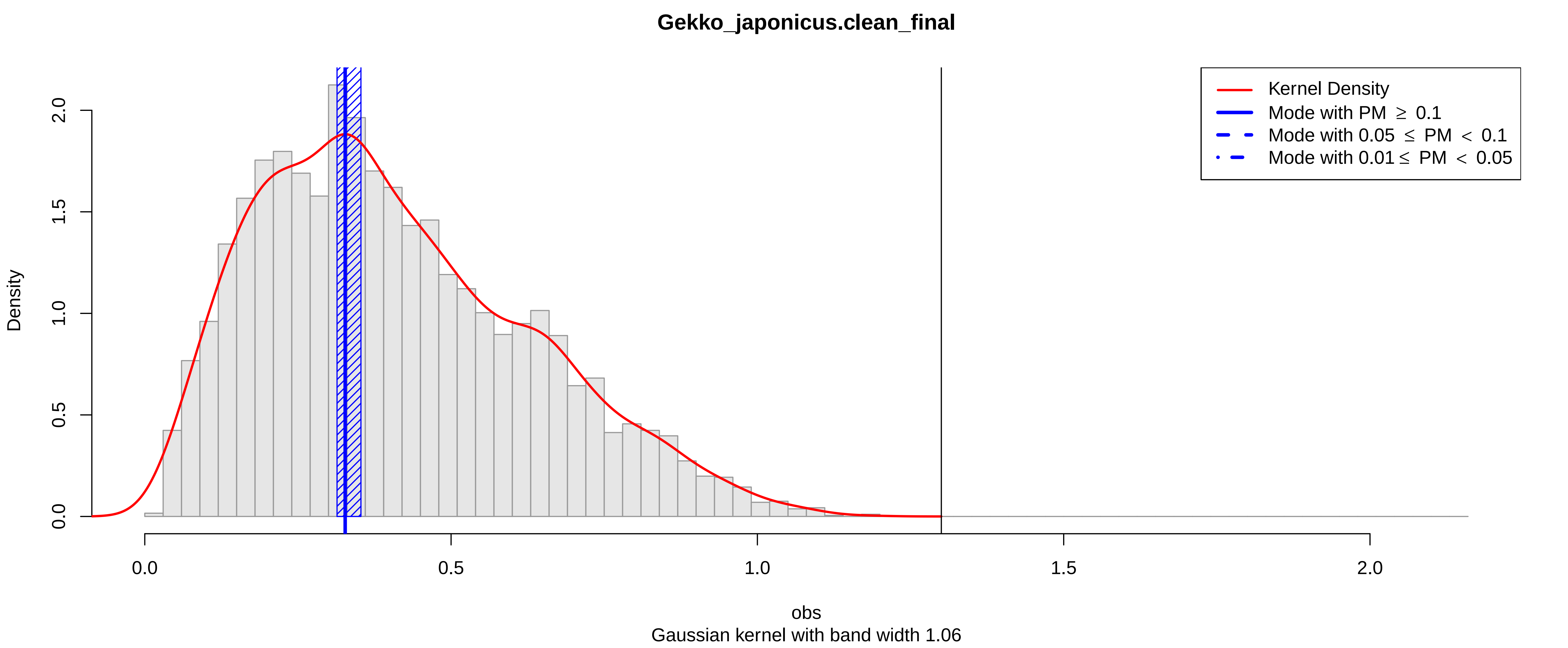
Gekko_japonicus.clean_final
Kernel Density
Mode with PM ≥ 0.1
Mode with 0.05 ≤ PM < 0.1
Mode with 0.01 ≤ PM < 0.05
Density
0.0
0.5
1.0
1.5
2.0
0.0
0.5
1.0
1.5
2.0
obs
Gaussian kernel with band width 1.06

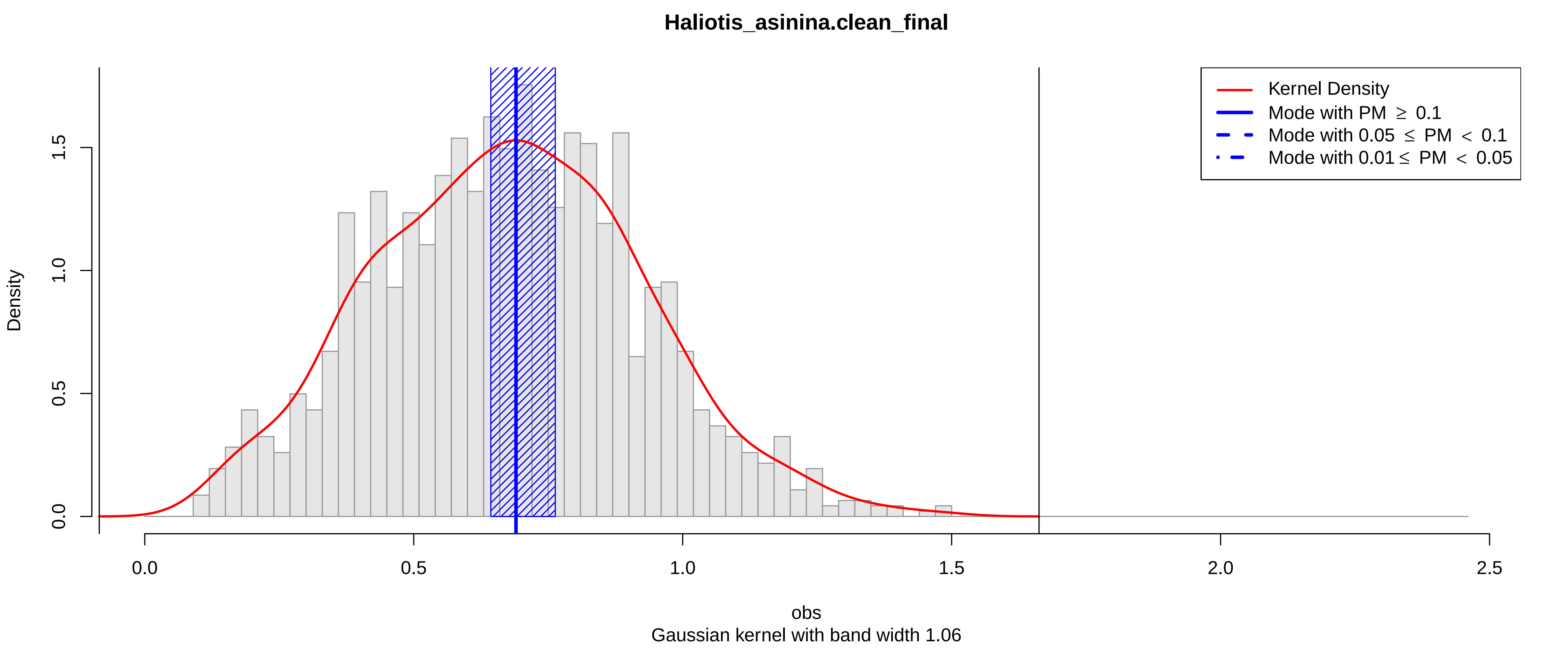

Haliotis_asinina.clean_final
Kernel Density
Mode with PM ≥ 0.1
Mode with 0.05 ≤ PM < 0.1
Mode with 0.01 ≤ PM < 0.05
Density
0.0
0.5
1.0
1.5
0.0
0.5
1.0
1.5
2.0
2.5
obs
Gaussian kernel with band width 1.06

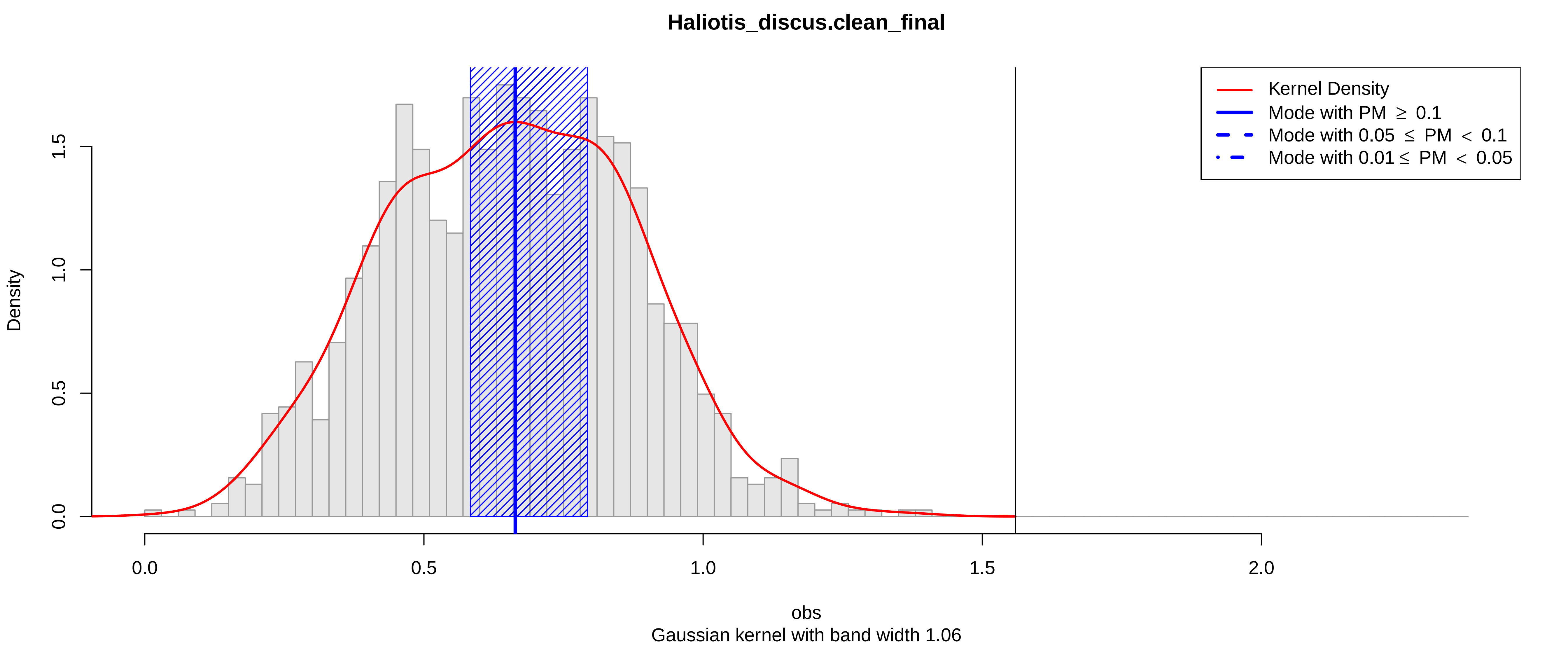
Haliotis_discus.clean_final
Kernel Density
Mode with PM ≥ 0.1
Mode with 0.05 ≤ PM < 0.1
Mode with 0.01 ≤ PM < 0.05
Density
0.0
0.5
1.0
1.5
0.0
0.5
1.0
1.5
2.0
obs
Gaussian kernel with band width 1.06

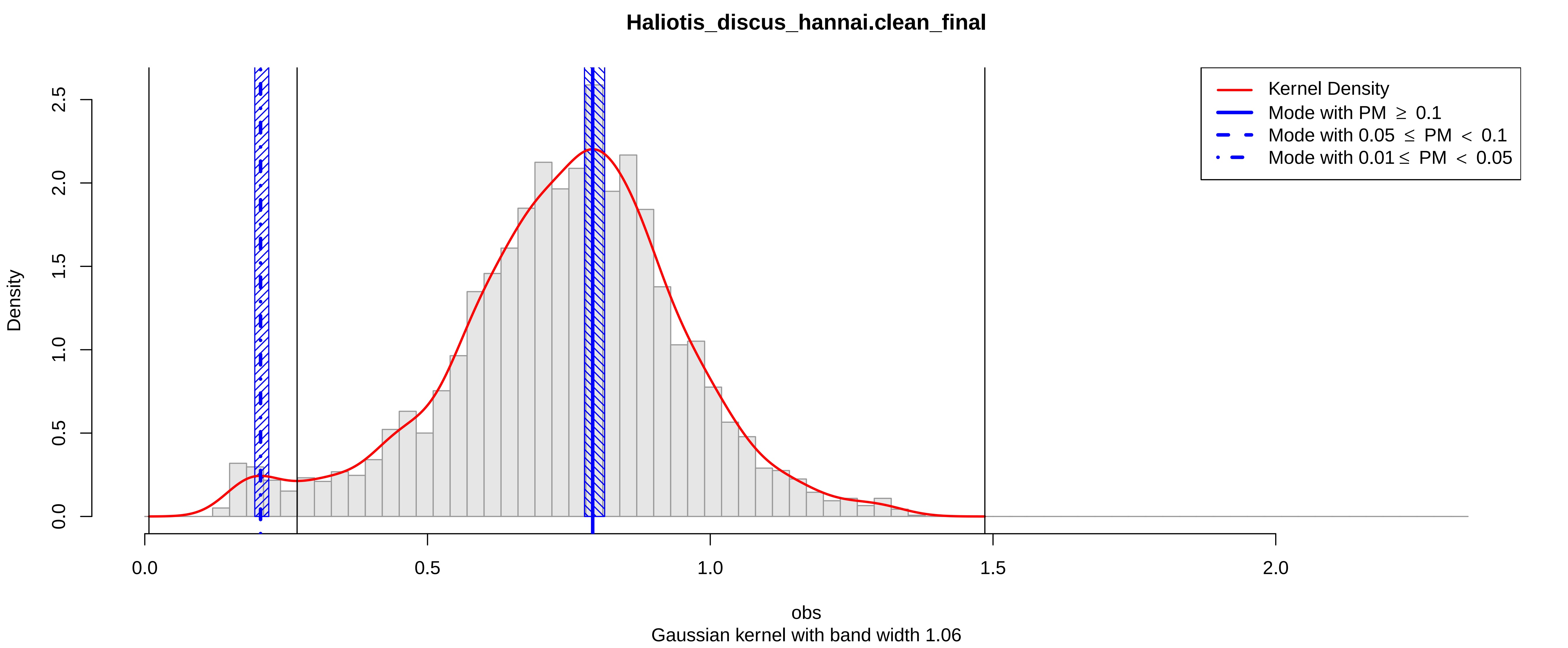
Haliotis_discus_hannai.clean_final
Kernel Density
Mode with PM ≥ 0.1
Mode with 0.05 ≤ PM < 0.1
Mode with 0.01 ≤ PM < 0.05
Density
0.0
0.5
1.0
1.5
2.0
2.5
obs
Gaussian kernel with band width 1.06

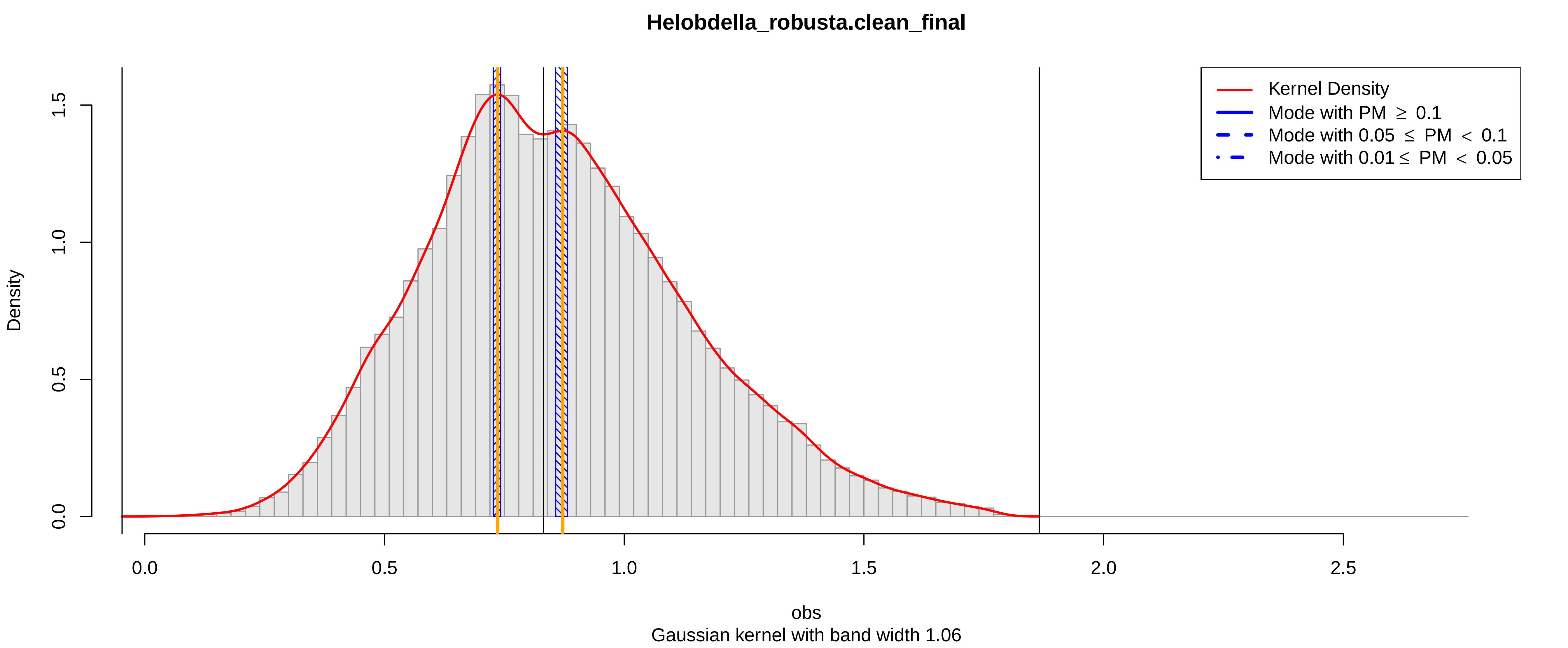
Helobdella_robusta.clean_final
Kernel Density
Mode with PM ≥ 0.1
Mode with 0.05 ≤ PM < 0.1
Mode with 0.01 ≤ PM < 0.05
Density
0.0
0.5
1.0
1.5
0.0
0.5
1.0
1.5
2.0
2.5
obs
Gaussian kernel with band width 1.06

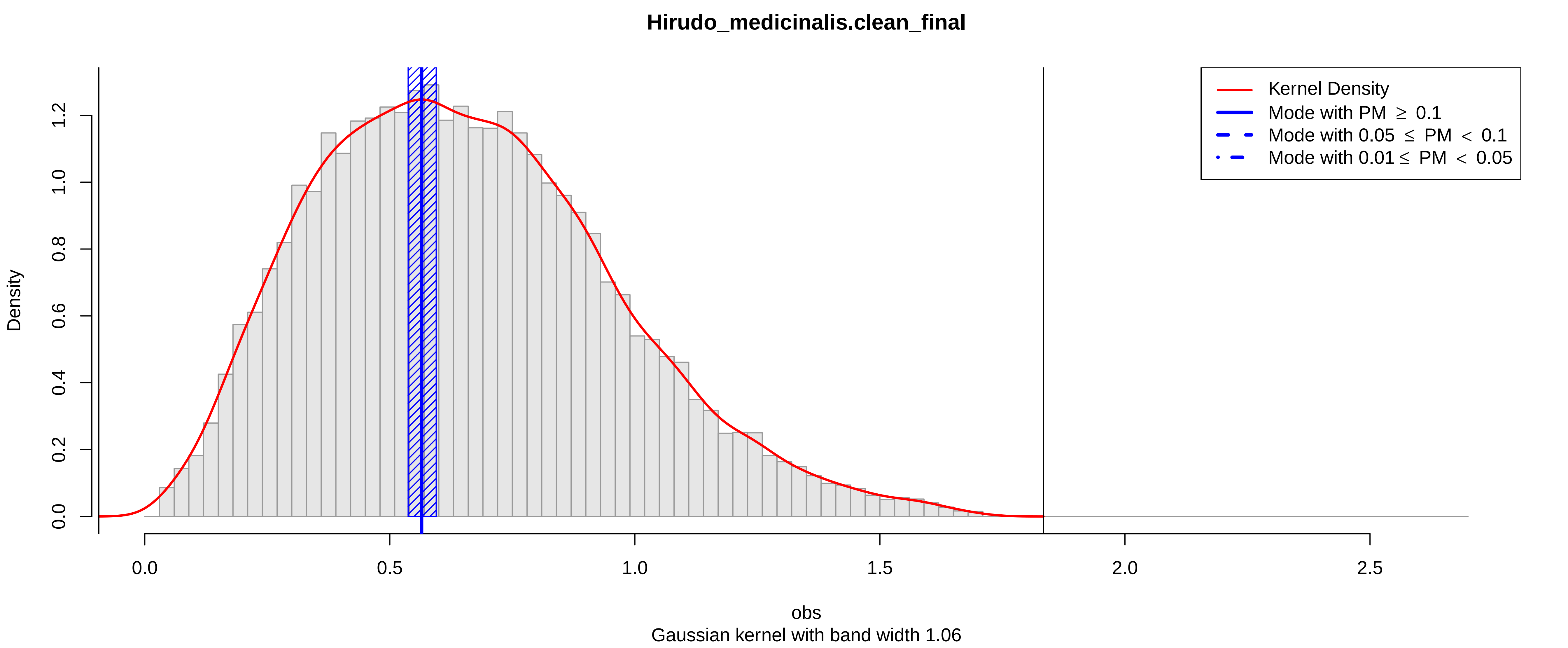

Hirudo_medicinalis.clean_final
Kernel Density
Mode with PM ≥ 0.1
Mode with 0.05 ≤ PM < 0.1
Mode with 0.01 ≤ PM < 0.05
Density
0.0
0.2
0.4
0.6
0.8
1.0
1.2
0.0
0.5
1.0
1.5
2.0
2.5
obs
Gaussian kernel with band width 1.06

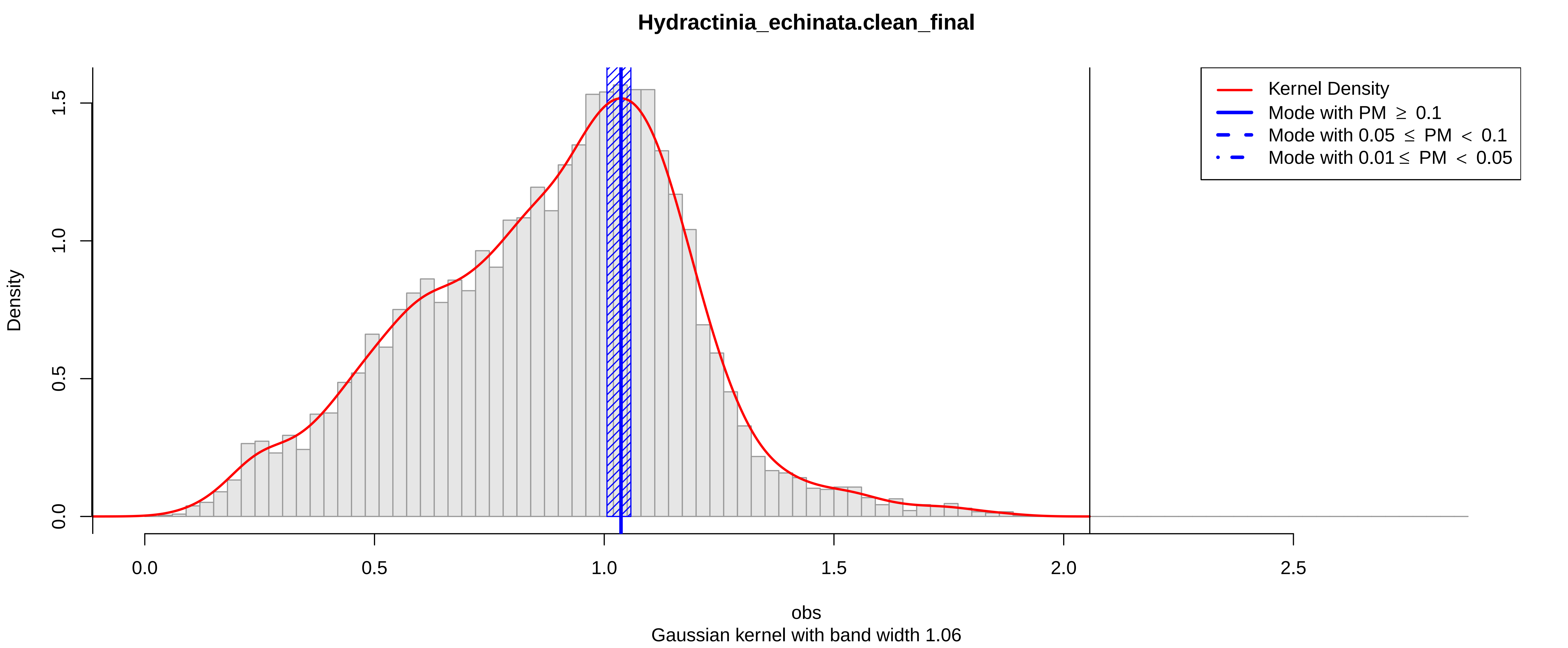
Hydractinia_echinata.clean_final
Kernel Density
Mode with PM ≥ 0.1
Mode with 0.05 ≤ PM < 0.1
Mode with 0.01 ≤ PM < 0.05
Density
0.0
0.5
1.0
1.5
0.0
0.5
1.0
1.5
2.0
2.5
obs
Gaussian kernel with band width 1.06

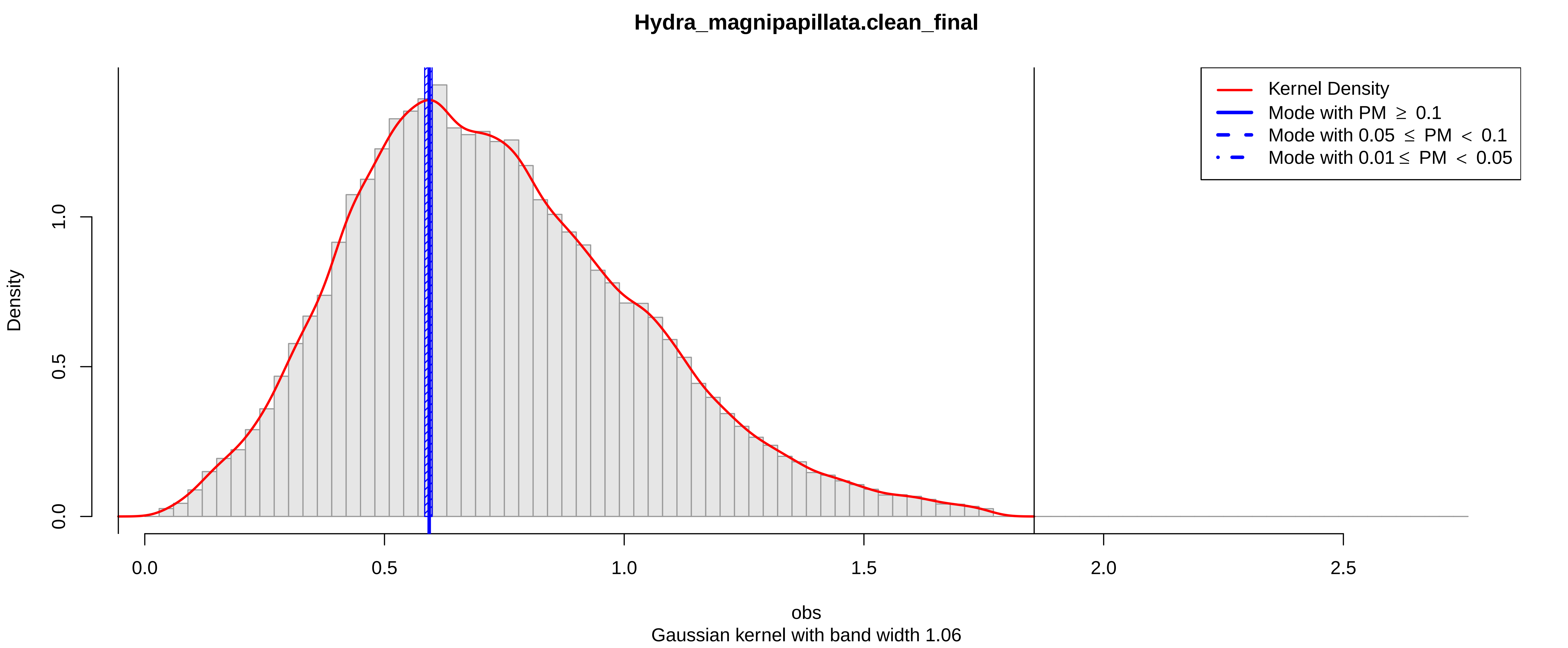

Hydra_magnipapillata.clean_final
Kernel Density
Mode with PM ≥ 0.1
Mode with 0.05 ≤ PM < 0.1
Mode with 0.01 ≤ PM < 0.05
Density
0.0
0.5
1.0
0.0
0.5
1.0
1.5
2.0
2.5
obs
Gaussian kernel with band width 1.06

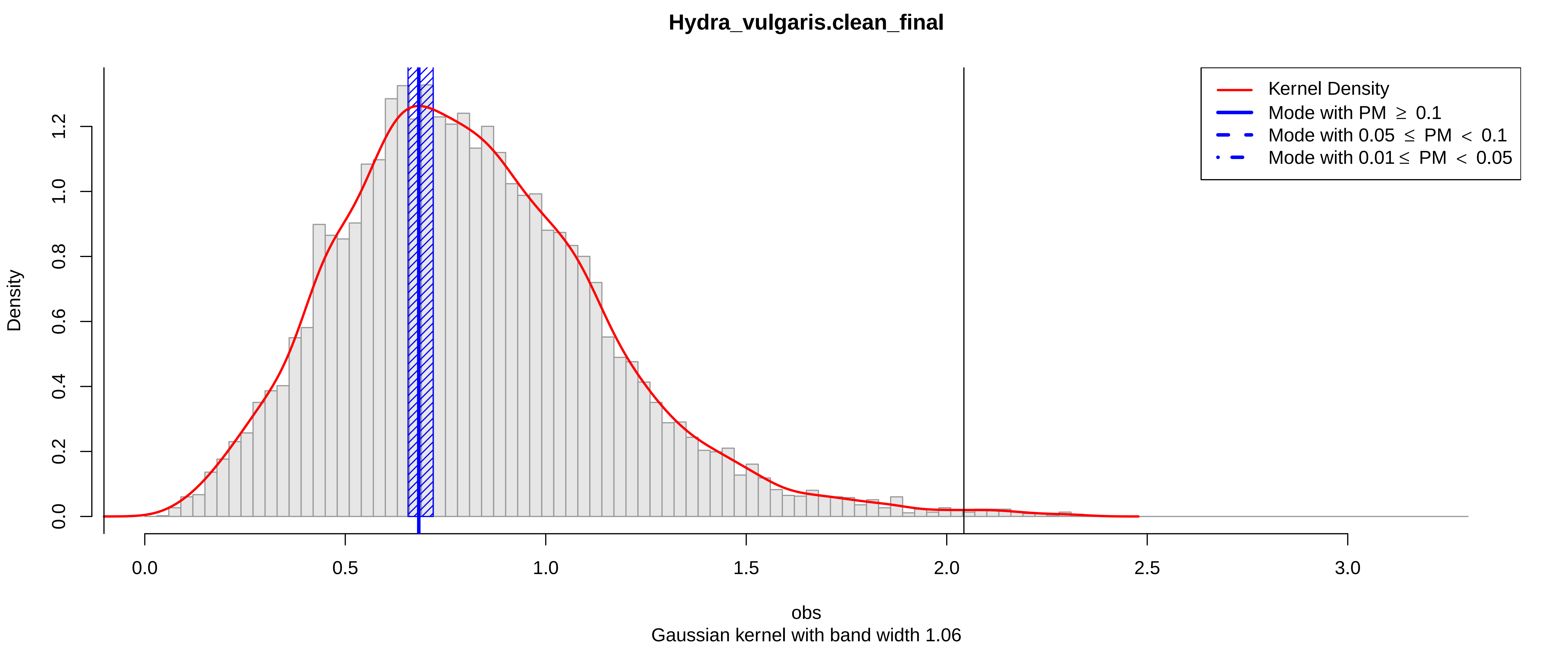

Hydra_vulgaris.clean_final
Kernel Density
Mode with PM ≥ 0.1
Mode with 0.05 ≤ PM < 0.1
Mode with 0.01 ≤ PM < 0.05
Density
0.0
0.2
0.4
0.6
0.8
1.0
1.2
0.0
0.5
1.0
1.5
2.0
2.5
3.0
obs
Gaussian kernel with band width 1.06

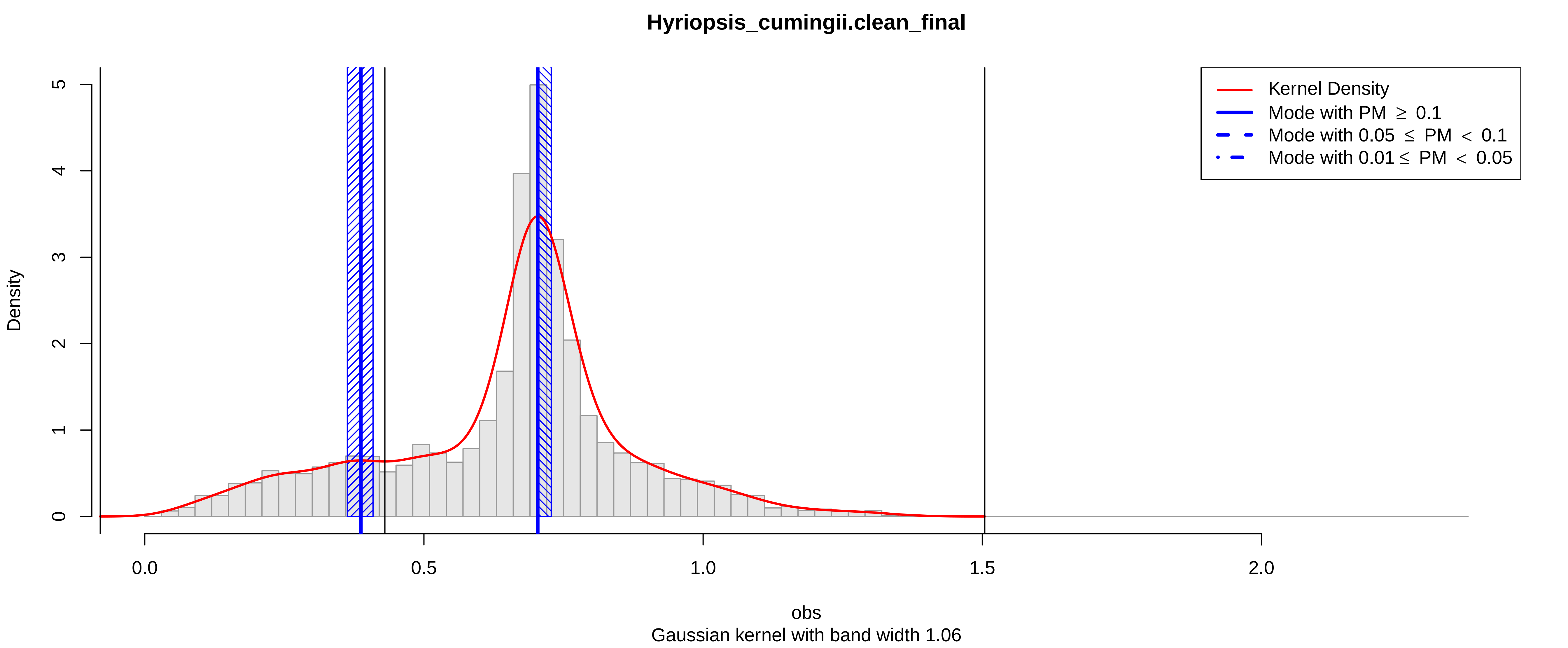

Hyriopsis_cumingii.clean_final
Kernel Density
Mode with PM ≥ 0.1
Mode with 0.05 ≤ PM < 0.1
Mode with 0.01 ≤ PM < 0.05
Density
0
1
2
3
4
5
0.0
0.5
1.0
1.5
2.0
obs
Gaussian kernel with band width 1.06

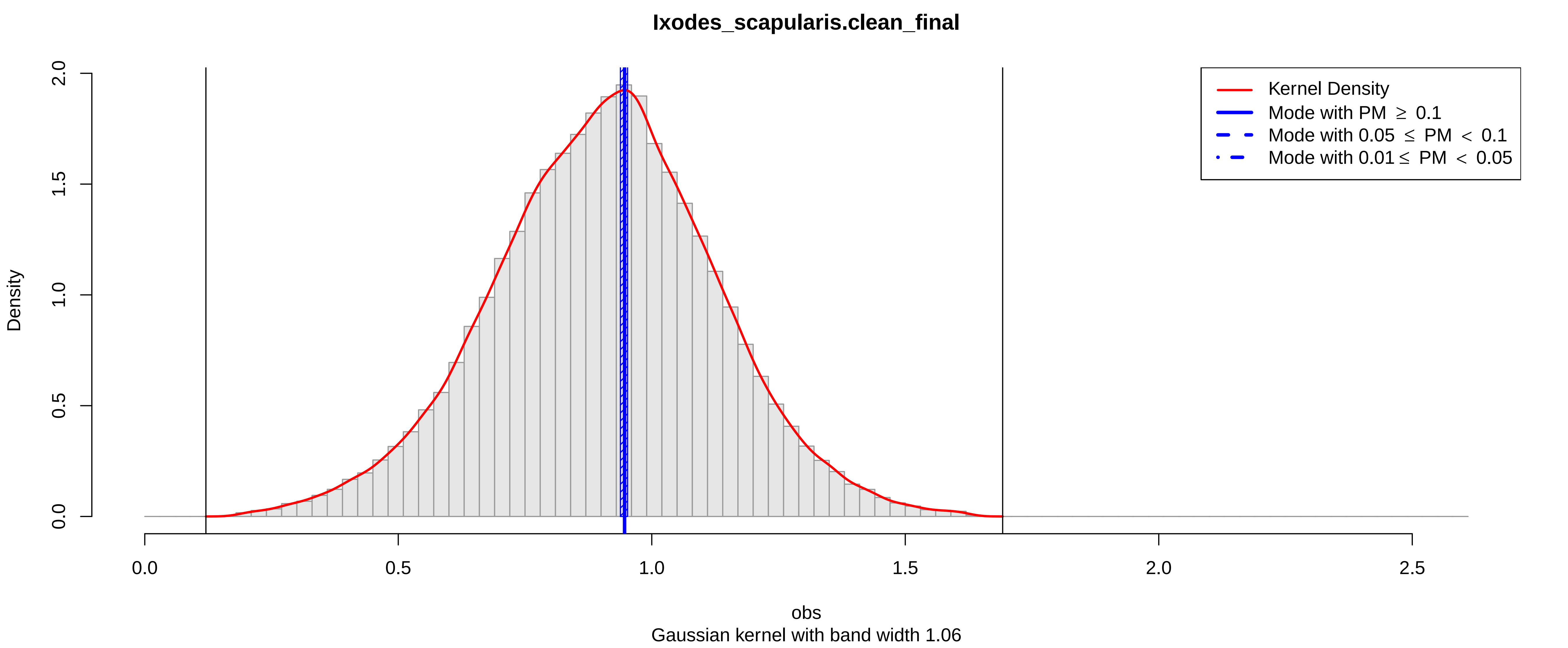
Ixodes_scapularis.clean_final
Density
obs
Gaussian kernel with band width 1.06
Kernel Density
Mode with PM ≥ 0.1
Mode with 0.05 ≤ PM < 0.1
Mode with 0.01 ≤ PM < 0.05
0.0
0.5
1.0
1.5
2.0
2.5

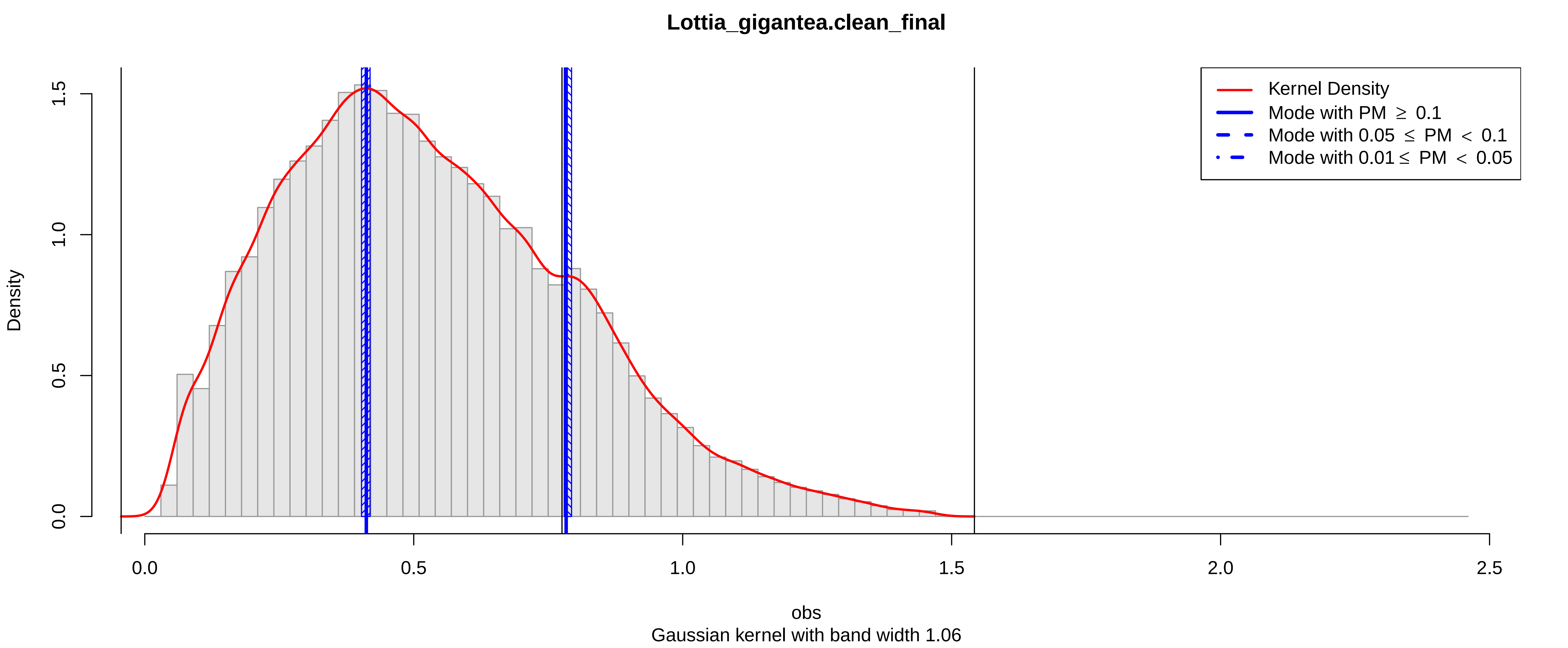

Lottia_gigantea.clean_final
Kernel Density
Mode with PM ≥ 0.1
Mode with 0.05 ≤ PM < 0.1
Mode with 0.01 ≤ PM < 0.05
Density
0.0
0.5
1.0
1.5
2.0
2.5
obs
Gaussian kernel with band width 1.06

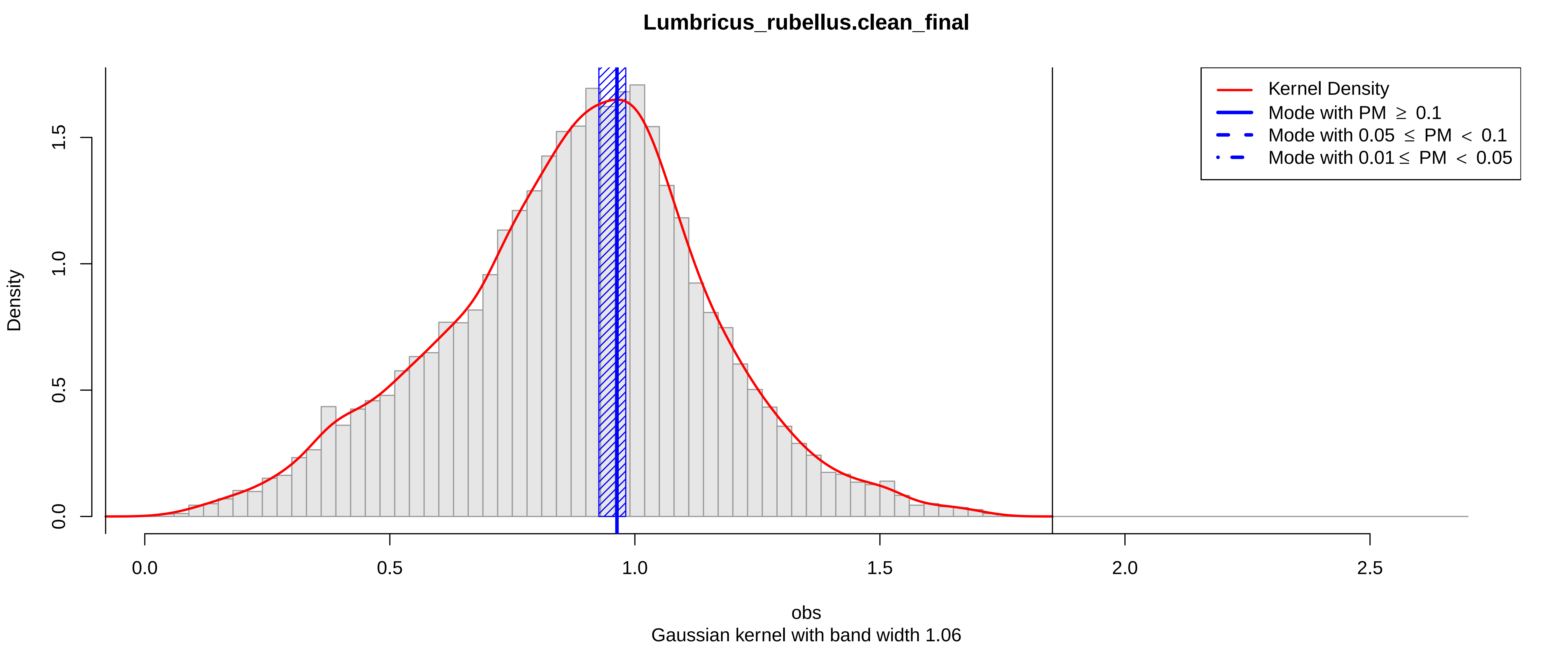

Lumbricus_rubellus.clean_final
Kernel Density
Mode with PM ≥ 0.1
Mode with 0.05 ≤ PM < 0.1
Mode with 0.01 ≤ PM < 0.05
Density
0.0
0.5
1.0
1.5
0.0
0.5
1.0
1.5
2.0
2.5
obs
Gaussian kernel with band width 1.06

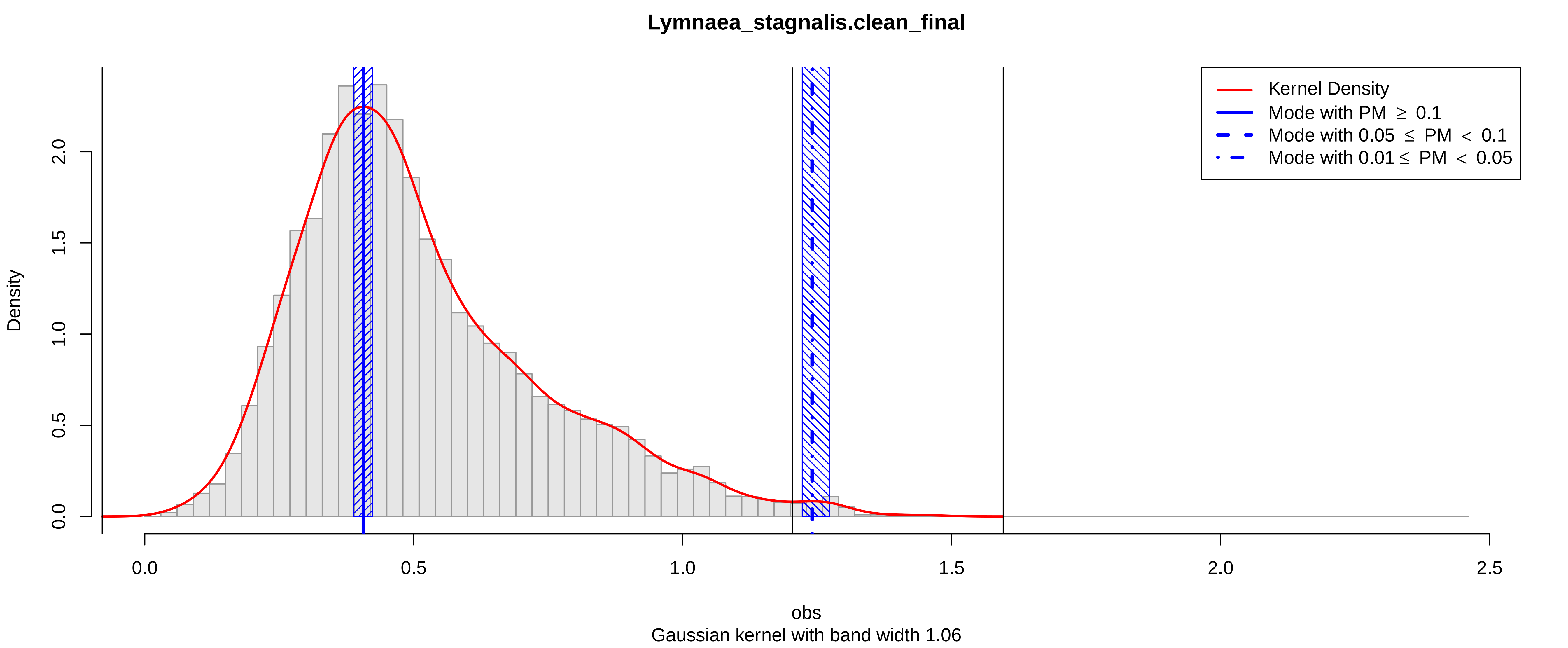
Lymnaea_stagnalis.clean_final
Kernel Density
Mode with PM ≥ 0.1
Mode with 0.05 ≤ PM < 0.1
Mode with 0.01 ≤ PM < 0.05
Density
0.0
0.5
1.0
1.5
2.0
0.0
0.5
1.0
1.5
2.0
2.5
obs
Gaussian kernel with band width 1.06

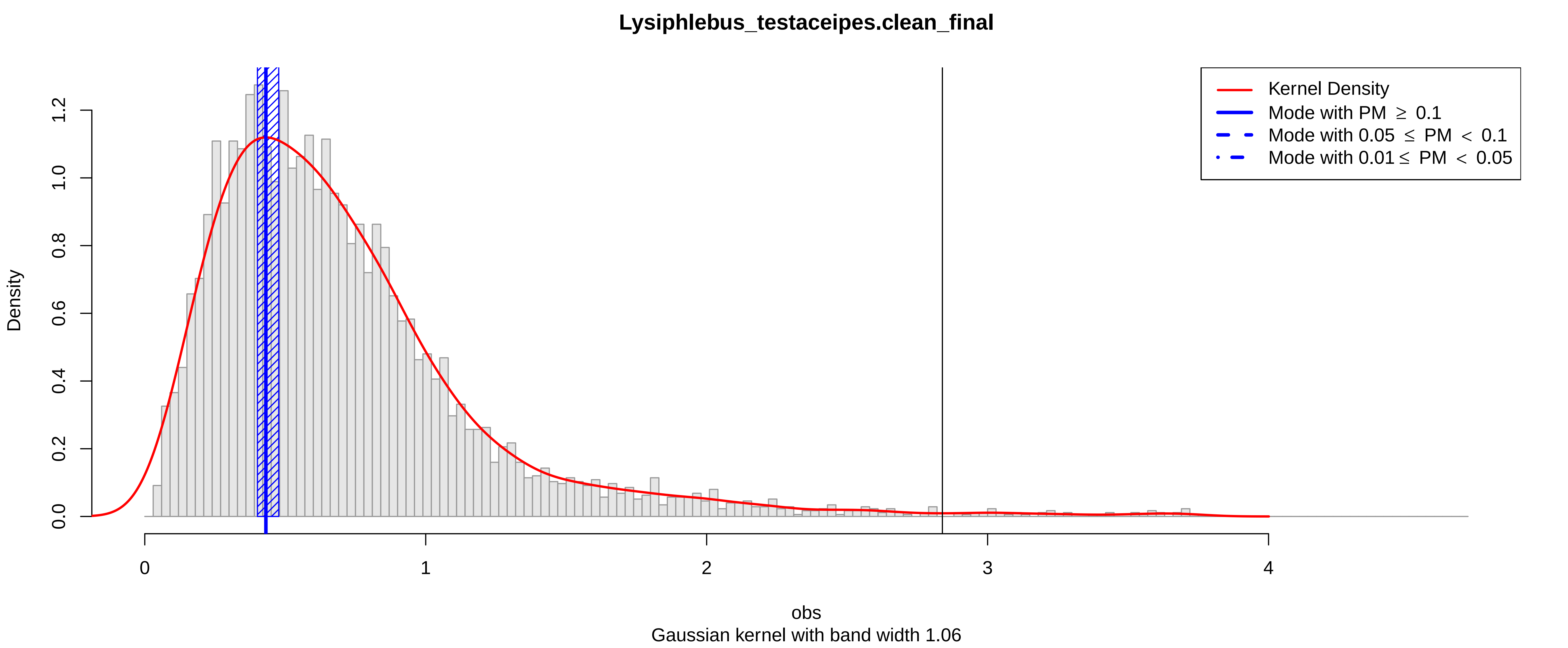

Lysiphlebus_testaceipes.clean_final
Kernel Density
Mode with PM ≥ 0.1
Mode with 0.05 ≤ PM < 0.1
Mode with 0.01 ≤ PM < 0.05
Density
0.0
0.2
0.4
0.6
0.8
1.0
1.2
0
1
2
3
4
obs
Gaussian kernel with band width 1.06

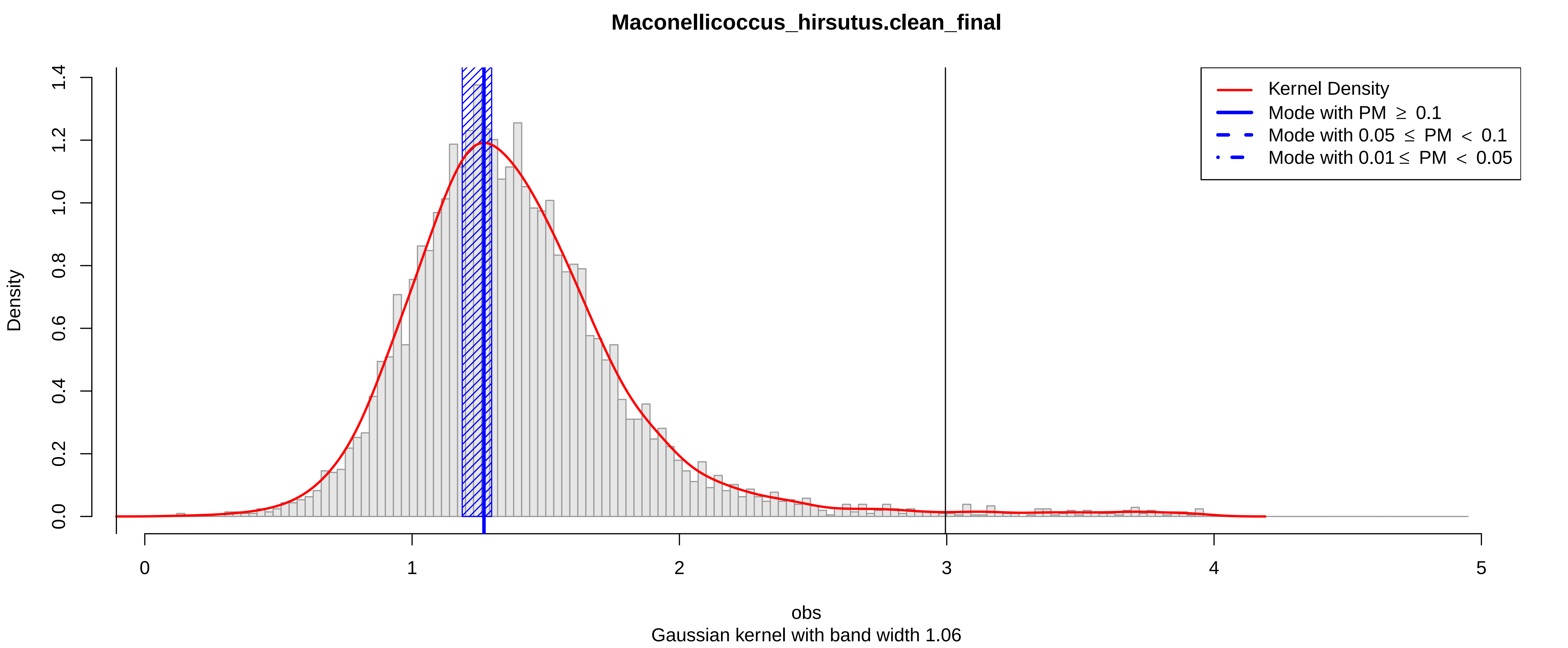
Maconellicoccus_hirsutus.clean_final
Density
0.0
0.2
0.4
0.6
0.8
1.0
1.2
1.4
0
1
2
3
4
5
obs
Gaussian kernel with band width 1.06
Kernel Density
Mode with PM ≥ 0.1
Mode with 0.05 ≤ PM < 0.1
Mode with 0.01 ≤ PM < 0.05

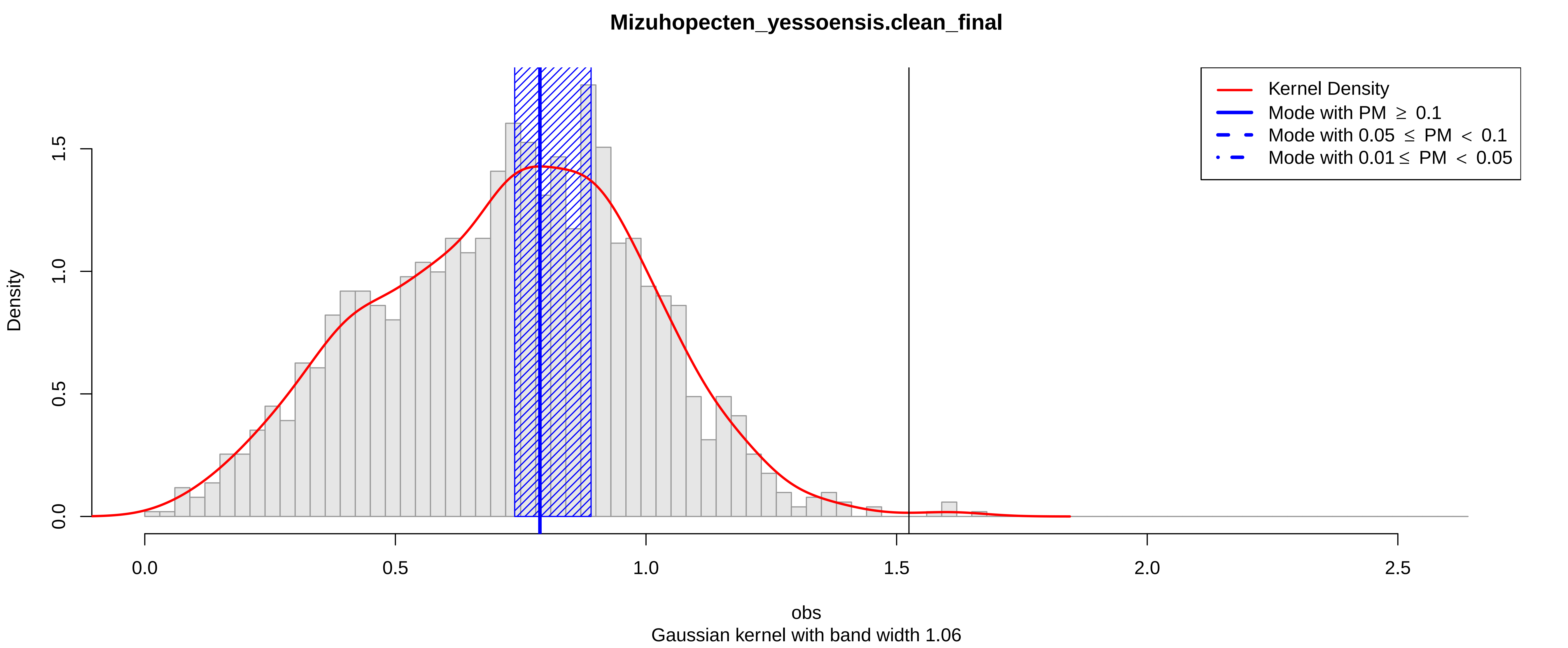
Mizuhopecten_yessoensis.clean_final
Kernel Density
Mode with PM ≥ 0.1
Mode with 0.05 ≤ PM < 0.1
Mode with 0.01 ≤ PM < 0.05
Density
0.0
0.5
1.0
1.5
0.0
0.5
1.0
1.5
2.0
2.5
obs
Gaussian kernel with band width 1.06

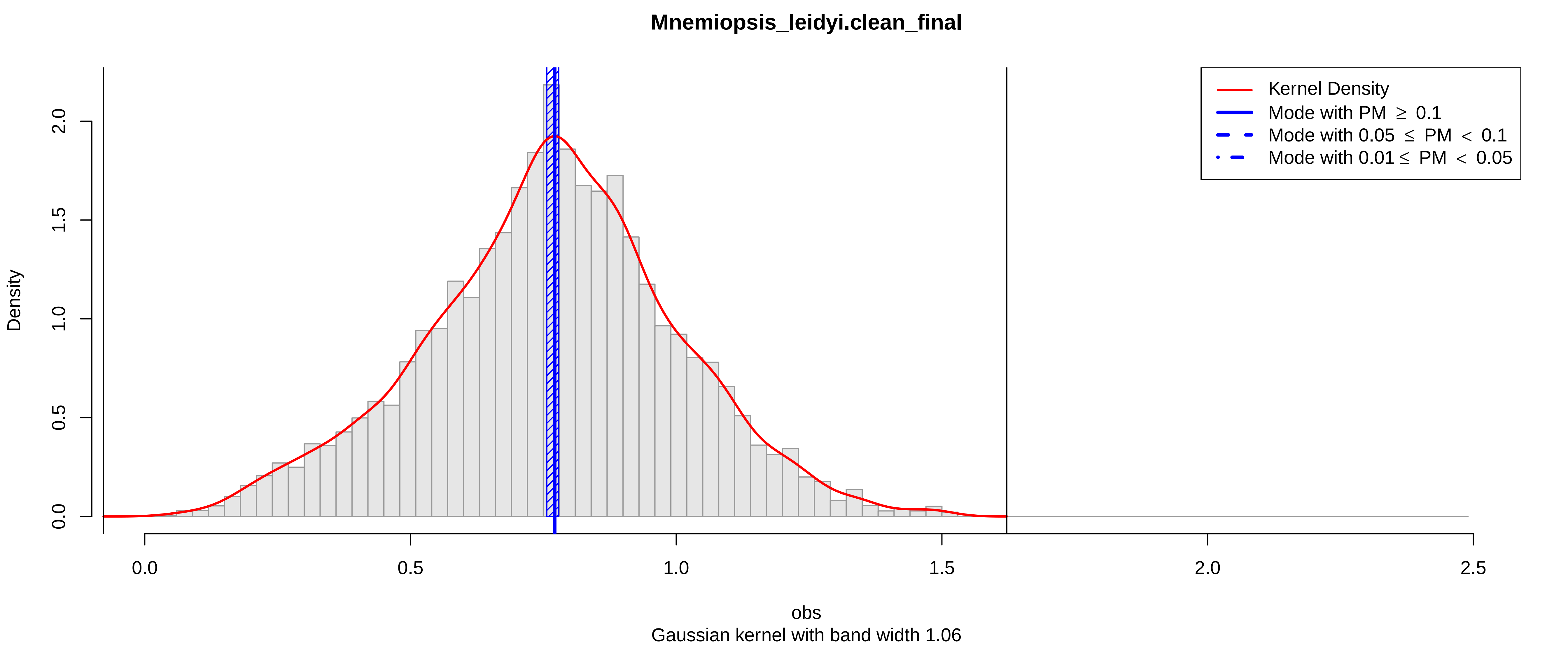
Mnemiopsis_leidyi.clean_final
Kernel Density
Mode with PM ≥ 0.1
Mode with 0.05 ≤ PM < 0.1
Mode with 0.01 ≤ PM < 0.05
Density
0.0
0.5
1.0
1.5
2.0
0.0
0.5
1.0
1.5
2.0
2.5
obs
Gaussian kernel with band width 1.06

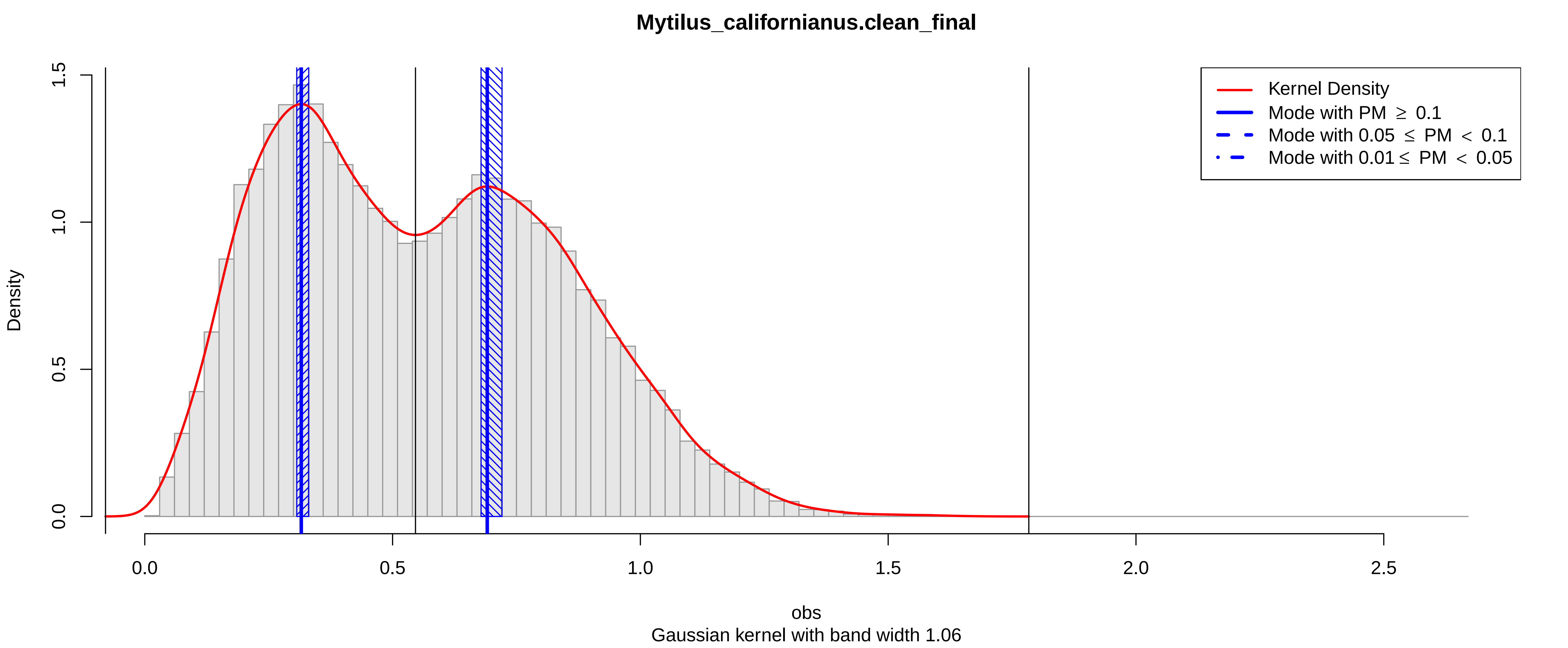

Mytilus_californianus.clean_final
Kernel Density
Mode with PM ≥ 0.1
Mode with 0.05 ≤ PM < 0.1
Mode with 0.01 ≤ PM < 0.05
Density
0.0
0.5
1.0
1.5
0.0
0.5
1.0
1.5
2.0
2.5
obs
Gaussian kernel with band width 1.06

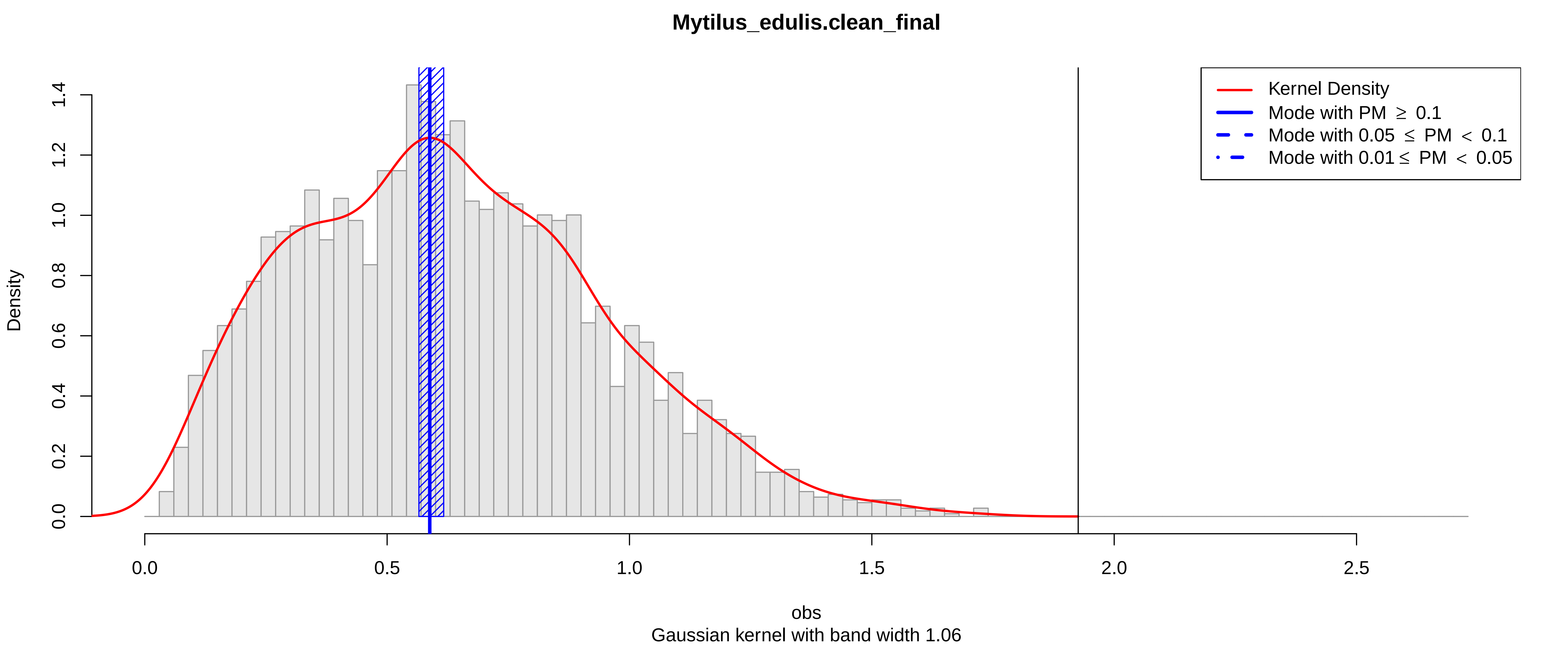

Mytilus_edulis.clean_final
Kernel Density
Mode with PM ≥ 0.1
Mode with 0.05 ≤ PM < 0.1
Mode with 0.01 ≤ PM < 0.05
Density
0.0
0.2
0.4
0.6
0.8
1.0
1.2
1.4
0.0
0.5
1.0
1.5
2.0
2.5
obs
Gaussian kernel with band width 1.06

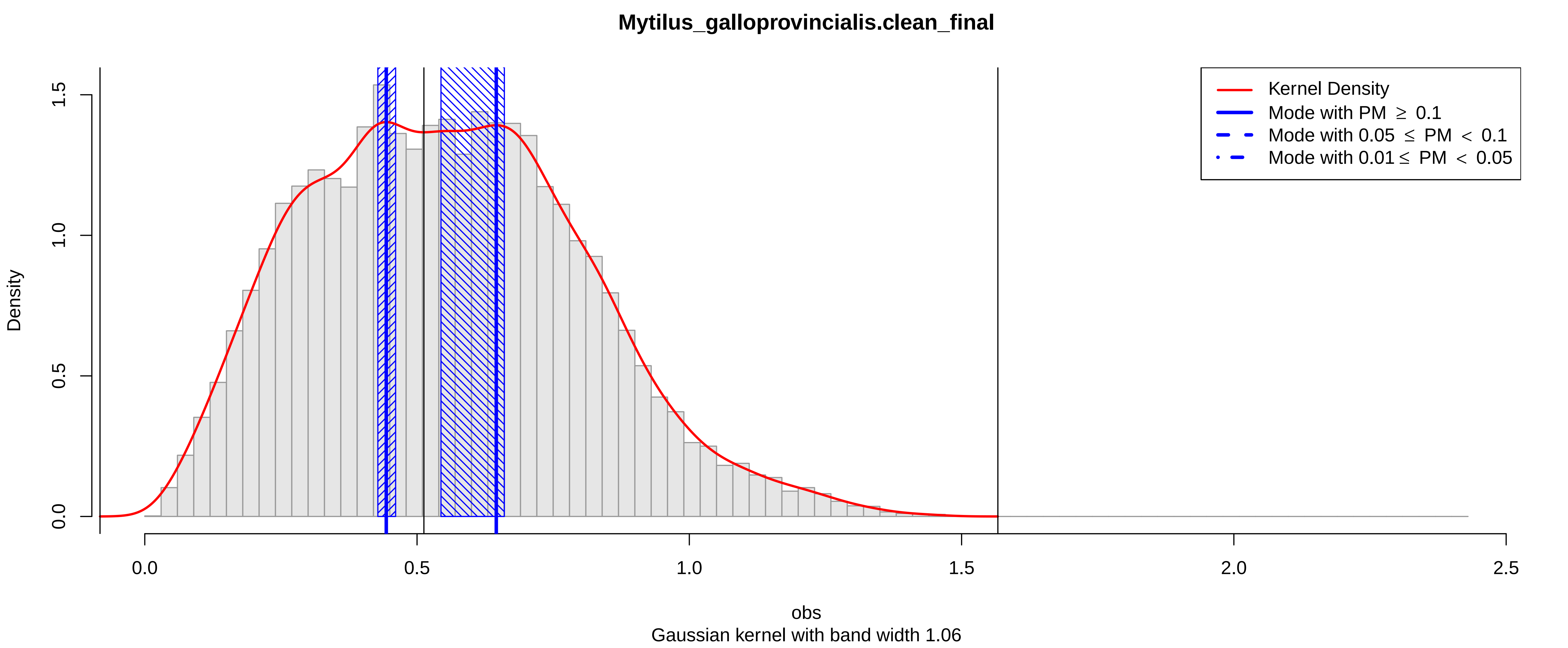
Mytilus_galloprovincialis.clean_final
Density
obs
Gaussian kernel with band width 1.06
Kernel Density
Mode with PM ≥ 0.1
Mode with 0.05 ≤ PM < 0.1
Mode with 0.01 ≤ PM < 0.05
0.0
0.5
1.0
1.5
2.0
2.5

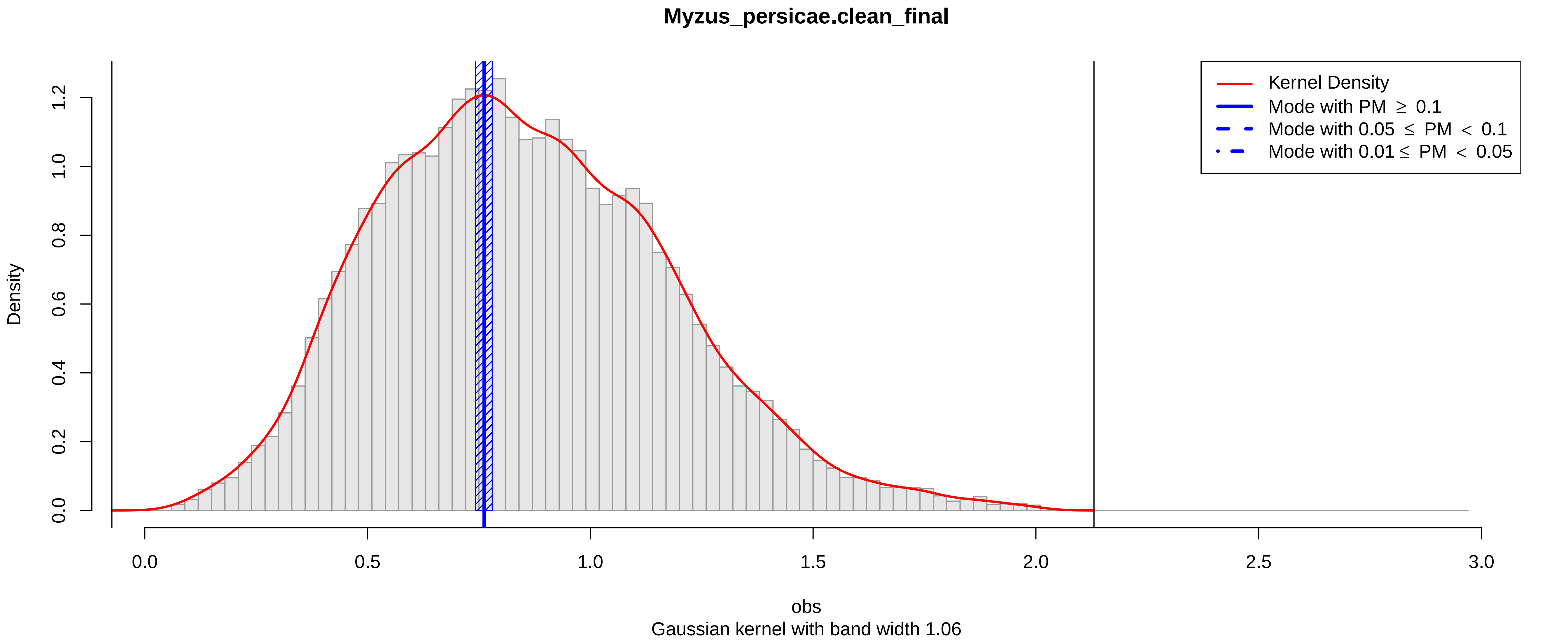
Myzus_persicae.clean_final
Kernel Density
Mode with PM ≥ 0.1
Mode with 0.05 ≤ PM < 0.1
Mode with 0.01 ≤ PM < 0.05
Density
0.0
0.2
0.4
0.6
0.8
1.0
1.2
0.0
0.5
1.0
1.5
2.0
2.5
3.0
obs
Gaussian kernel with band width 1.06

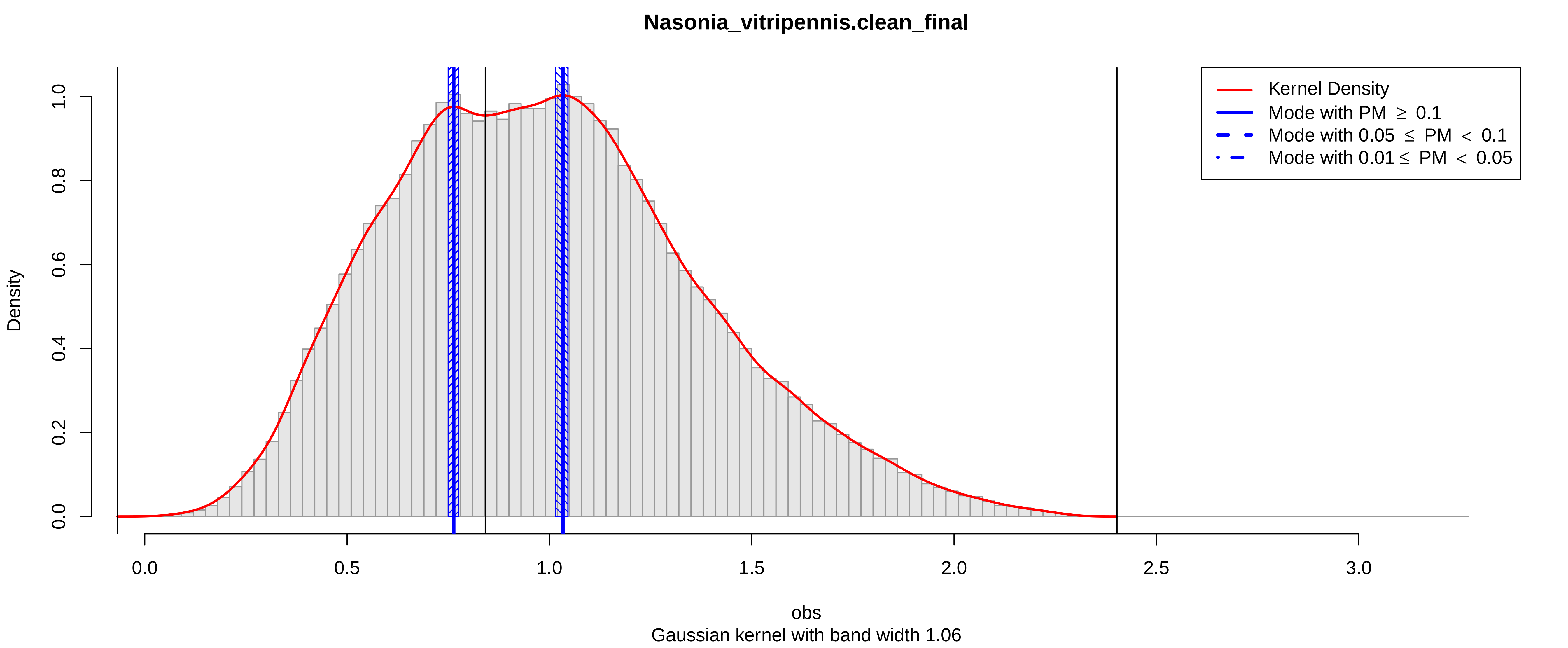

Nasonia_vitripennis.clean_final
Kernel Density
Mode with PM ≥ 0.1
Mode with 0.05 ≤ PM < 0.1
Mode with 0.01 ≤ PM < 0.05
Density
0.0
0.2
0.4
0.6
0.8
1.0
0.0
0.5
1.0
1.5
2.0
2.5
3.0
obs
Gaussian kernel with band width 1.06

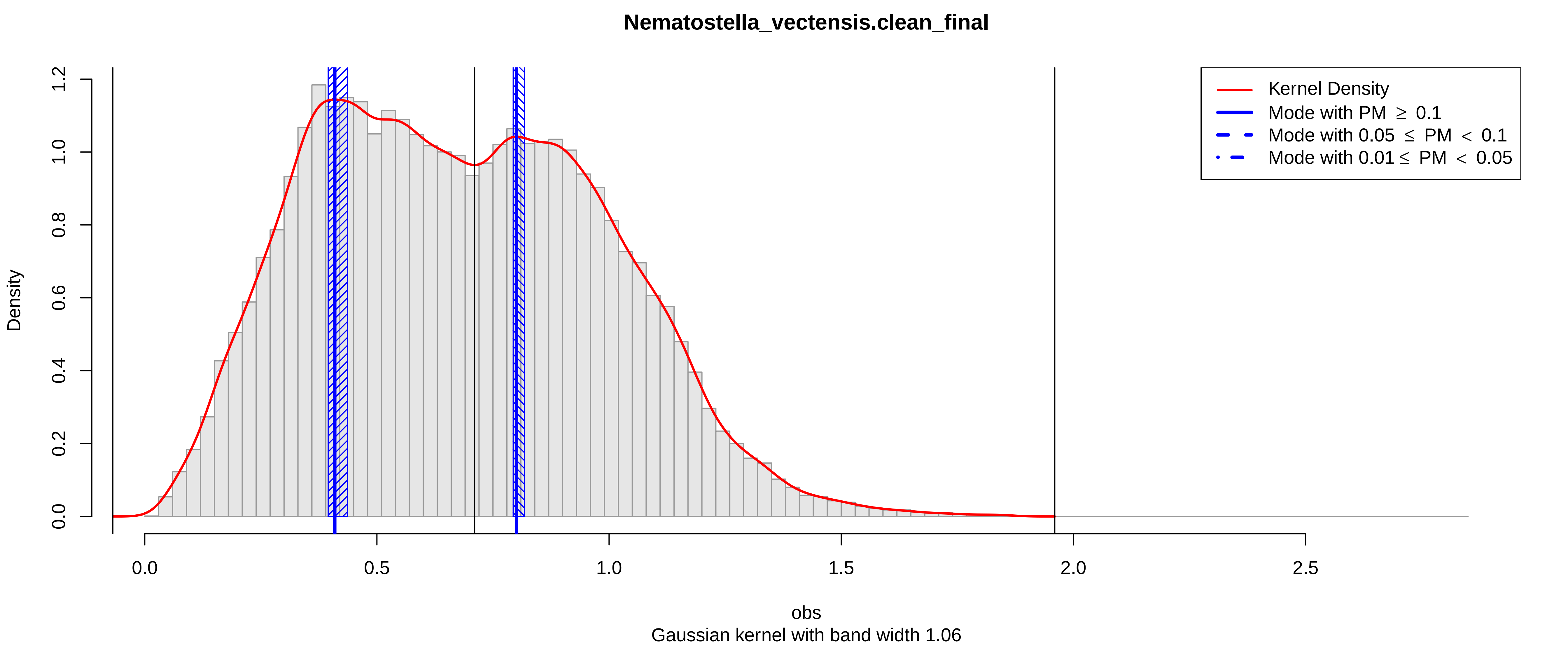

Nematostella_vectensis.clean_final
Kernel Density
Mode with PM ≥ 0.1
Mode with 0.05 ≤ PM < 0.1
Mode with 0.01 ≤ PM < 0.05
Density
0.0
0.2
0.4
0.6
0.8
1.0
1.2
0.0
0.5
1.0
1.5
2.0
2.5
obs
Gaussian kernel with band width 1.06

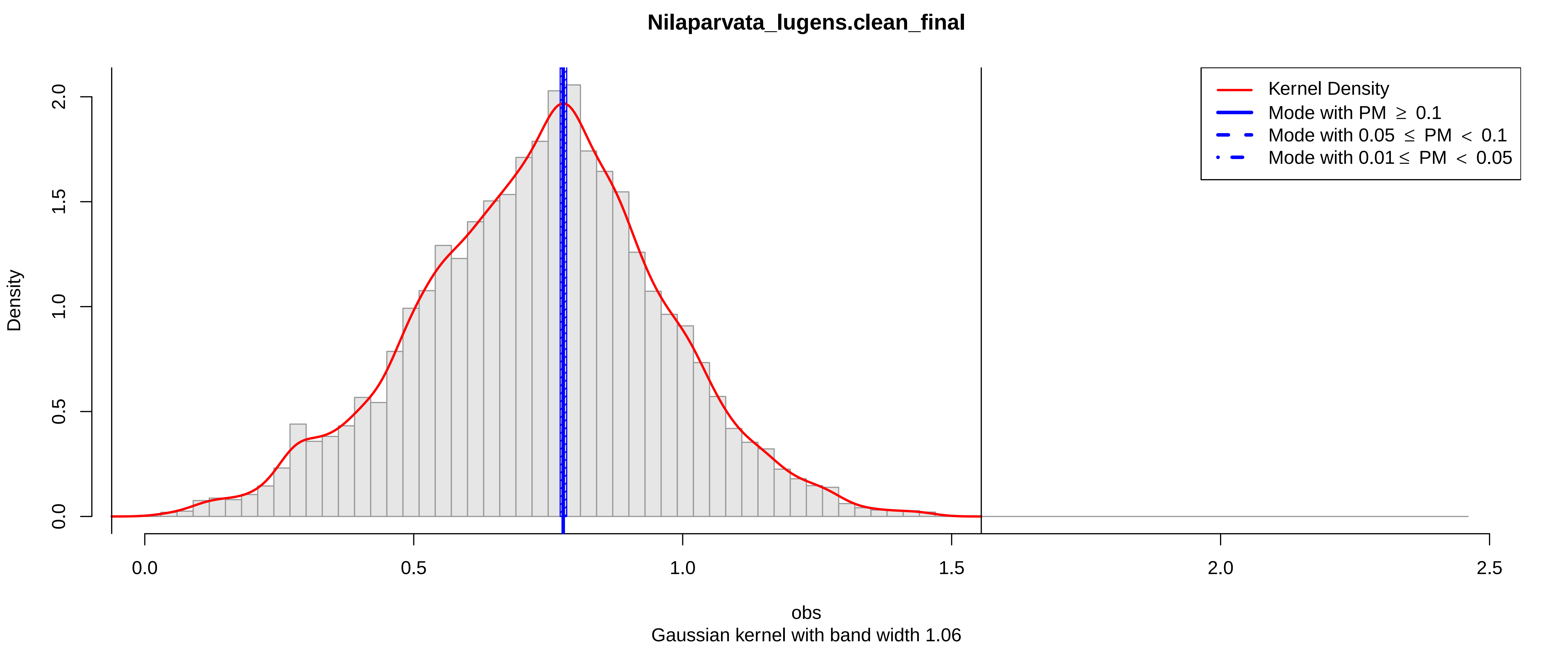

Nilaparvata_lugens.clean_final
Density
0.0
0.5
1.0
1.5
2.0
2.5
obs
Gaussian kernel with band width 1.06
Kernel Density
Mode with PM ≥ 0.1
Mode with 0.05 ≤ PM < 0.1
Mode with 0.01 ≤ PM < 0.05

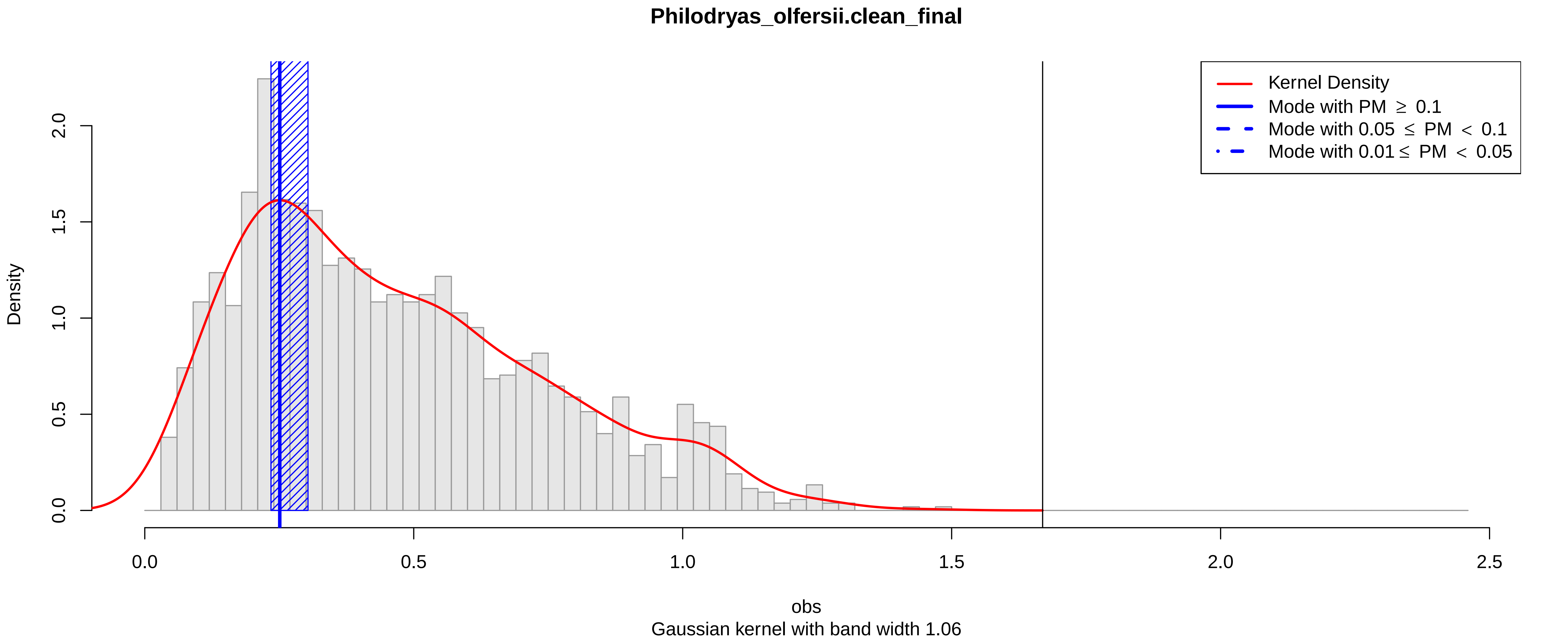
Philodryas_olfersii.clean_final
Kernel Density
Mode with PM ≥ 0.1
Mode with 0.05 ≤ PM < 0.1
Mode with 0.01 ≤ PM < 0.05
Density
0.0
0.5
1.0
1.5
2.0
2.5
obs
Gaussian kernel with band width 1.06

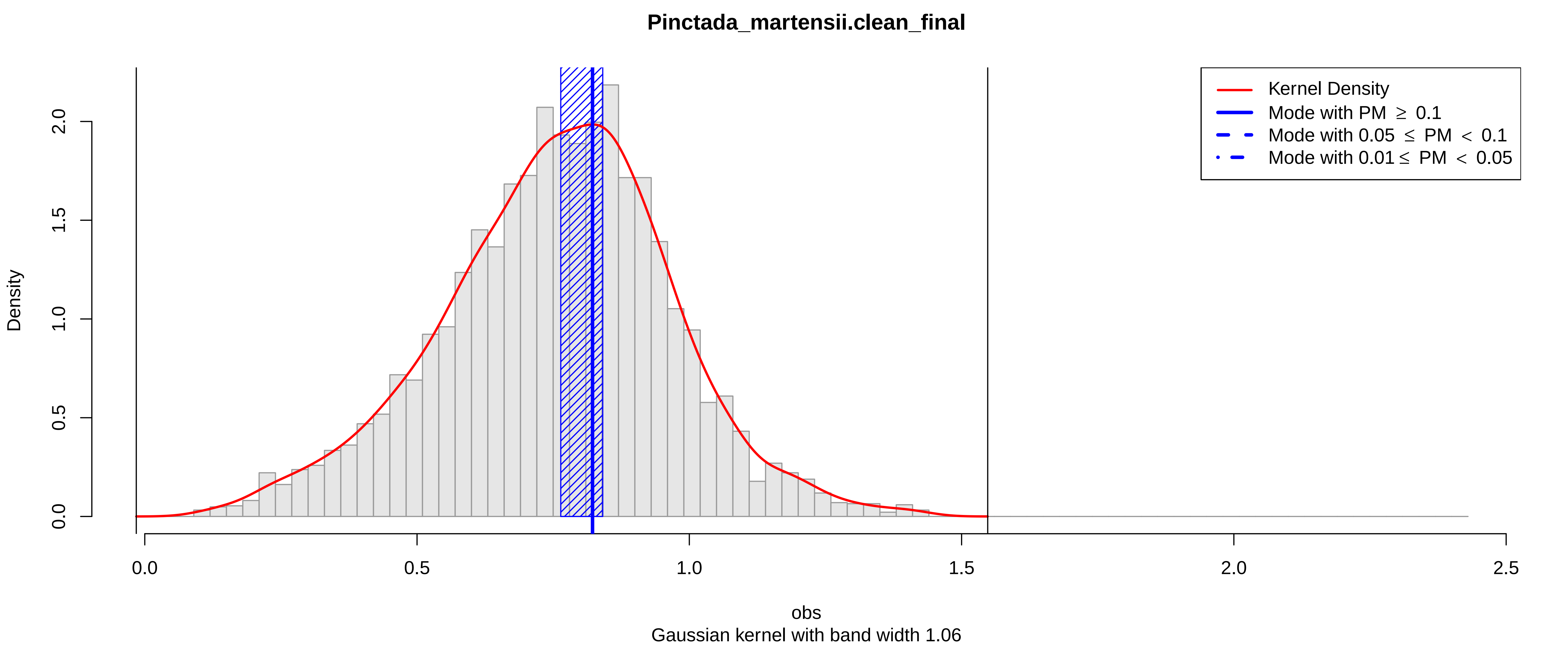
Pinctada_martensii.clean_final
Kernel Density
Mode with PM ≥ 0.1
Mode with 0.05 ≤ PM < 0.1
Mode with 0.01 ≤ PM < 0.05
Density
0.0
0.5
1.0
1.5
2.0
2.5
obs
Gaussian kernel with band width 1.06

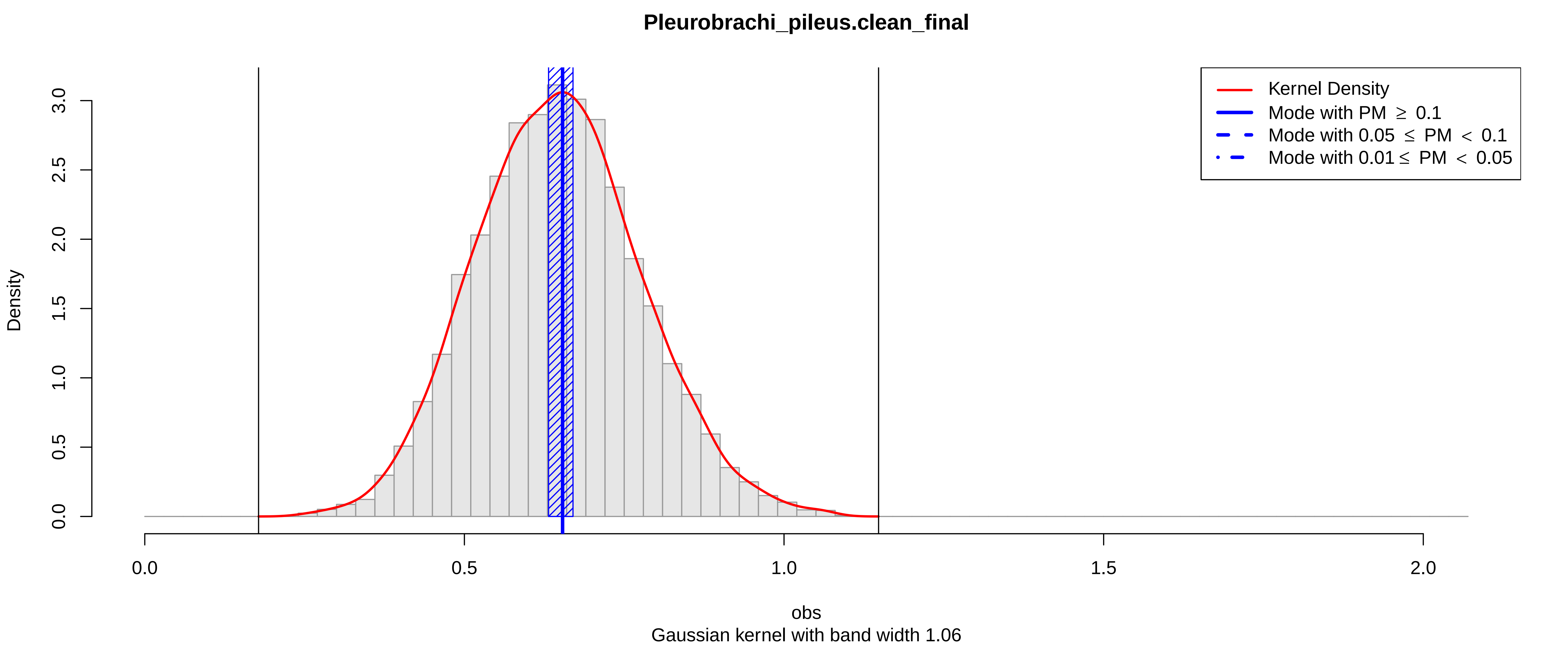

Pleurobrachi_pileus.clean_final
Kernel Density
Mode with PM ≥ 0.1
Mode with 0.05 ≤ PM < 0.1
Mode with 0.01 ≤ PM < 0.05
Density
0.0
0.5
1.0
1.5
2.0
2.5
3.0
0.0
0.5
1.0
1.5
2.0
obs
Gaussian kernel with band width 1.06

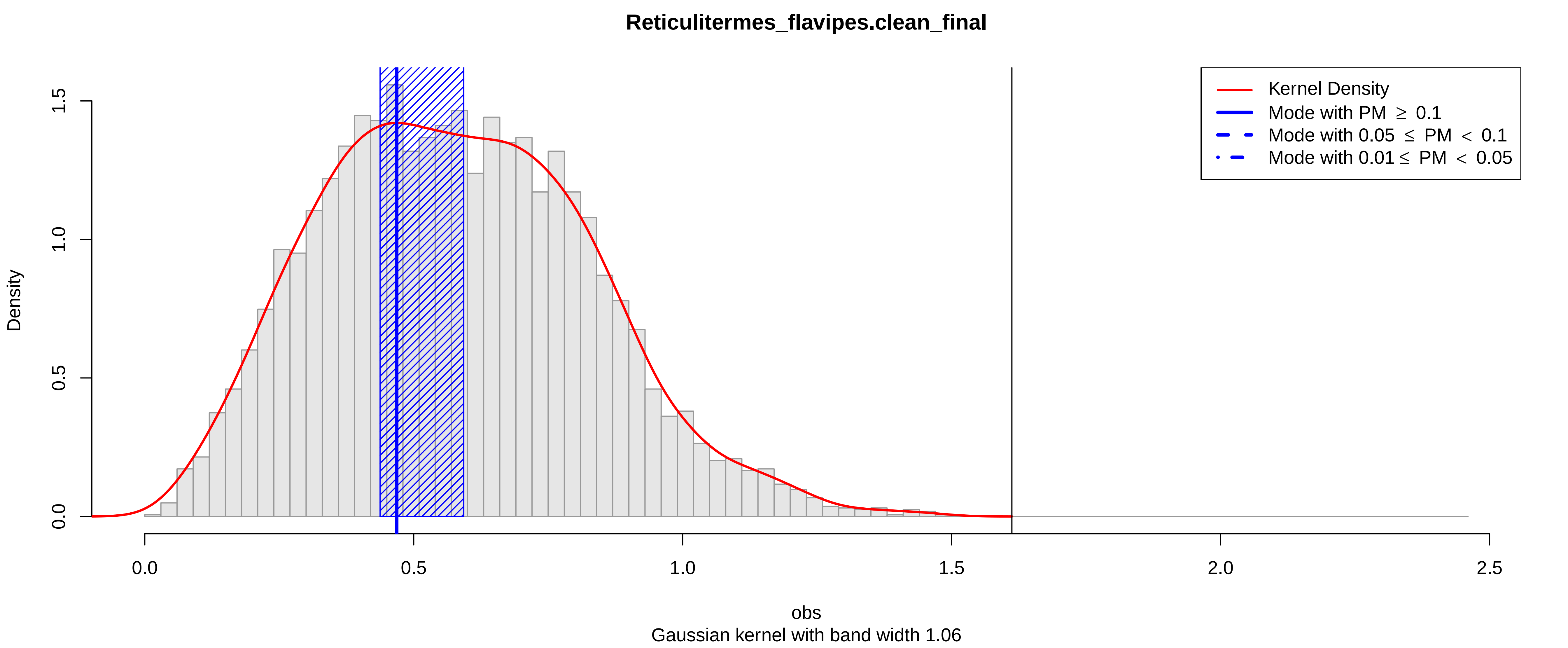

Reticulitermes_flavipes.clean_final
Kernel Density
Mode with PM ≥ 0.1
Mode with 0.05 ≤ PM < 0.1
Mode with 0.01 ≤ PM < 0.05
Density
0.0
0.5
1.0
1.5
0.0
0.5
1.0
1.5
2.0
2.5
obs
Gaussian kernel with band width 1.06

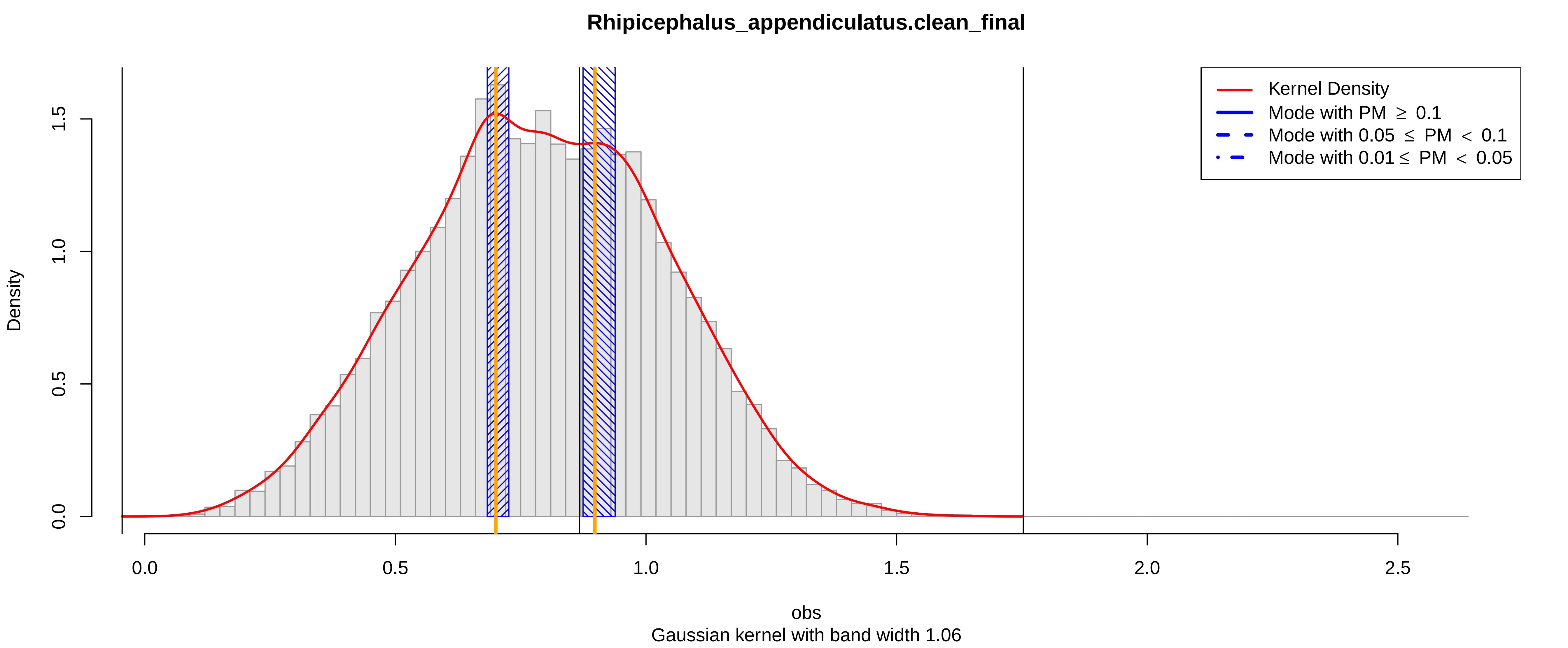

Rhipicephalus_appendiculatus.clean_final
Kernel Density
Mode with PM ≥ 0.1
Mode with 0.05 ≤ PM < 0.1
Mode with 0.01 ≤ PM < 0.05
Density
0.0
0.5
1.0
1.5
2.0
2.5
obs
Gaussian kernel with band width 1.06

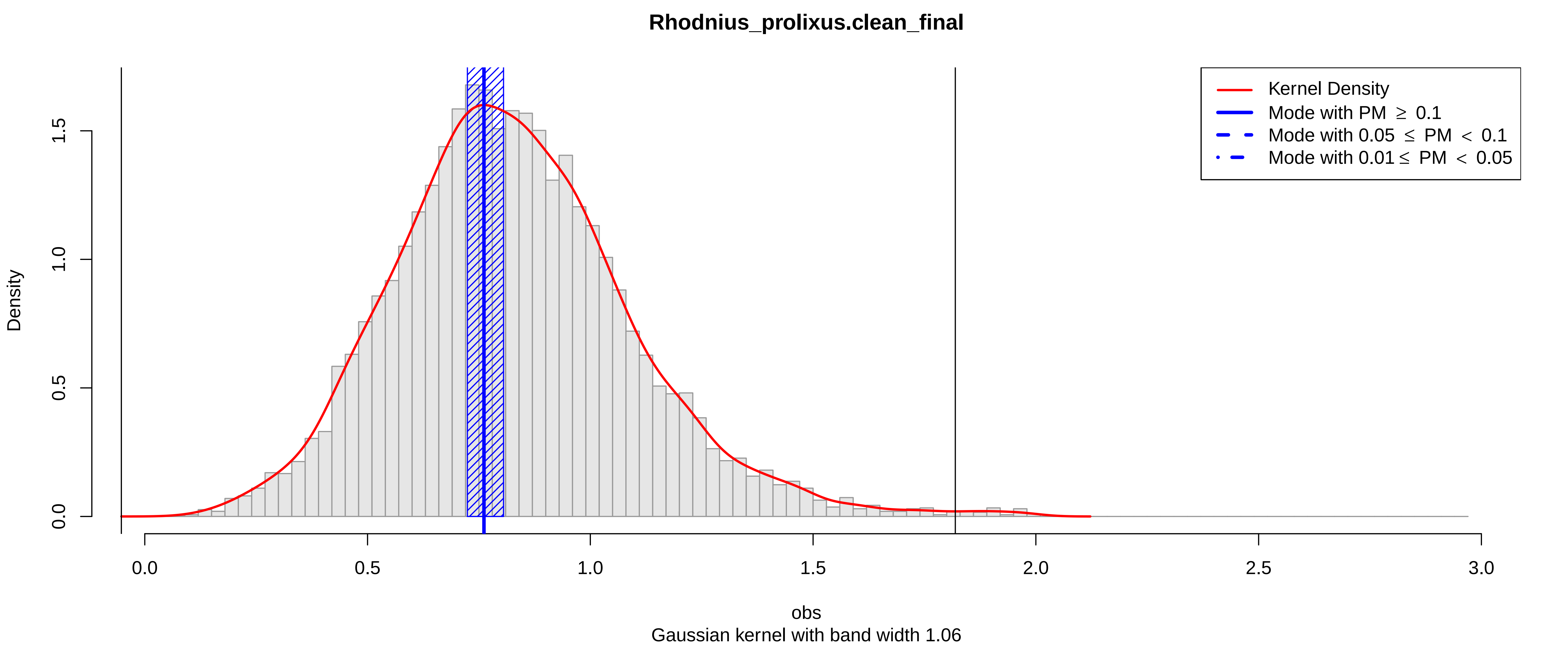
Rhodnius_prolixus.clean_final
Density
0.0
0.5
1.0
1.5
0.0
0.5
1.0
1.5
2.0
2.5
3.0
obs
Gaussian kernel with band width 1.06
Kernel Density
Mode with PM ≥ 0.1
Mode with 0.05 ≤ PM < 0.1
Mode with 0.01 ≤ PM < 0.05

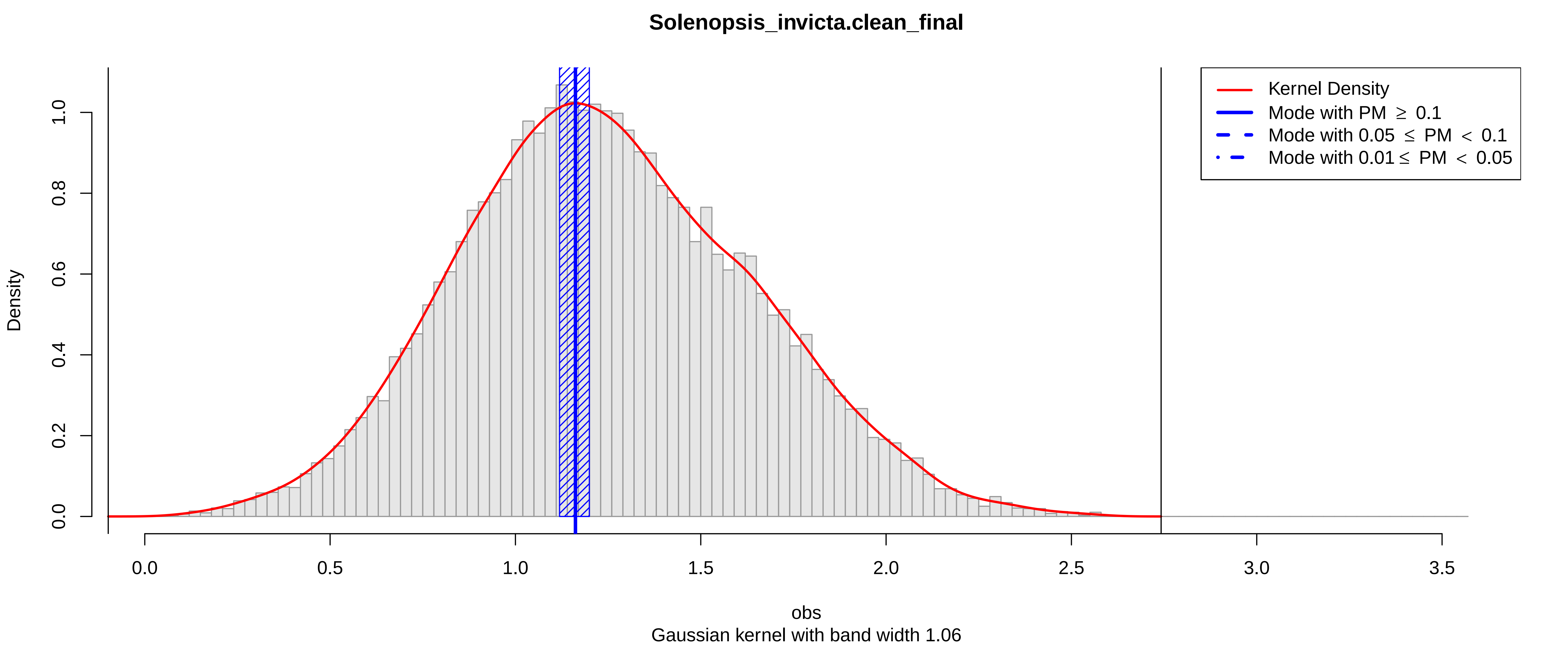

Solenopsis_invicta.clean_final
Kernel Density
Mode with PM ≥ 0.1
Mode with 0.05 ≤ PM < 0.1
Mode with 0.01 ≤ PM < 0.05
Density
0.0
0.2
0.4
0.6
0.8
1.0
0.0
0.5
1.0
1.5
2.0
2.5
3.0
3.5
obs
Gaussian kernel with band width 1.06

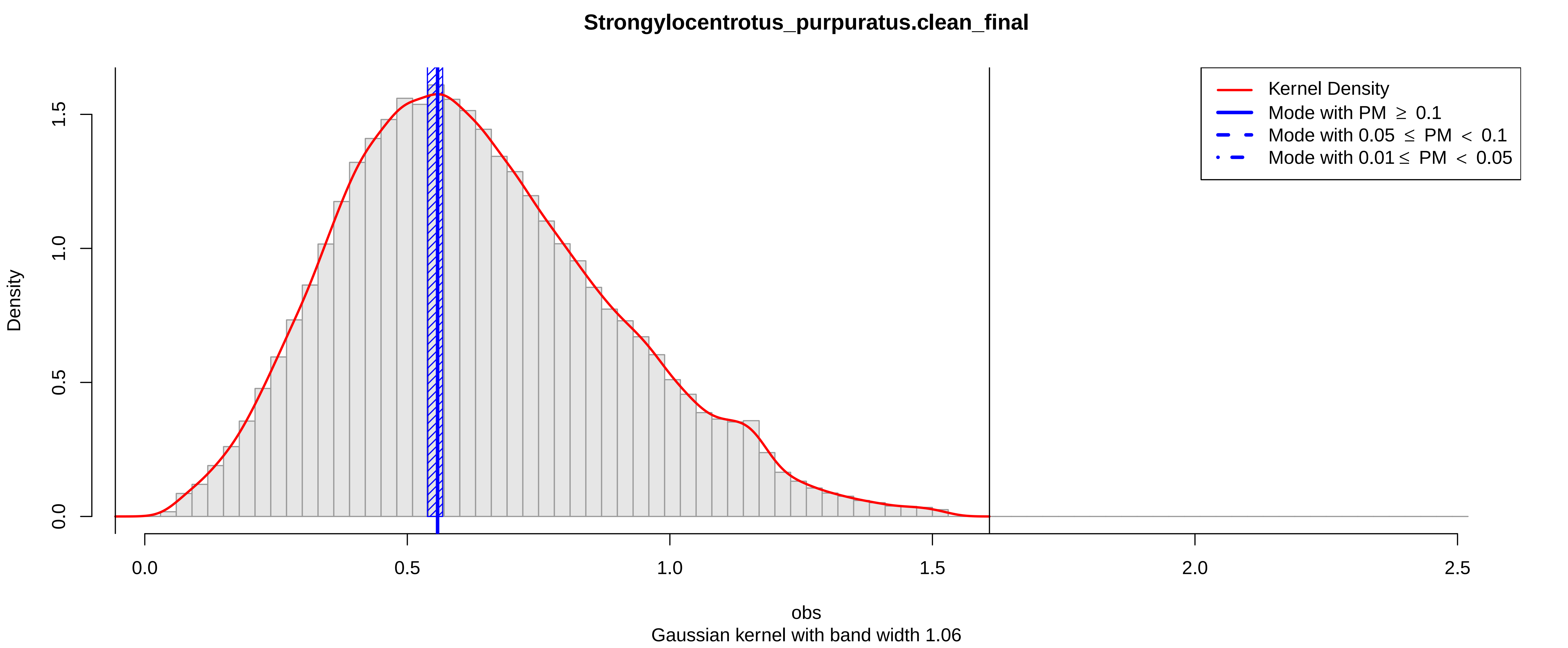

Strongylocentrotus_purpuratus.clean_final
Kernel Density
Mode with PM ≥ 0.1
Mode with 0.05 ≤ PM < 0.1
Mode with 0.01 ≤ PM < 0.05
Density
0.0
0.5
1.0
1.5
0.0
0.5
1.0
1.5
2.0
2.5
obs
Gaussian kernel with band width 1.06

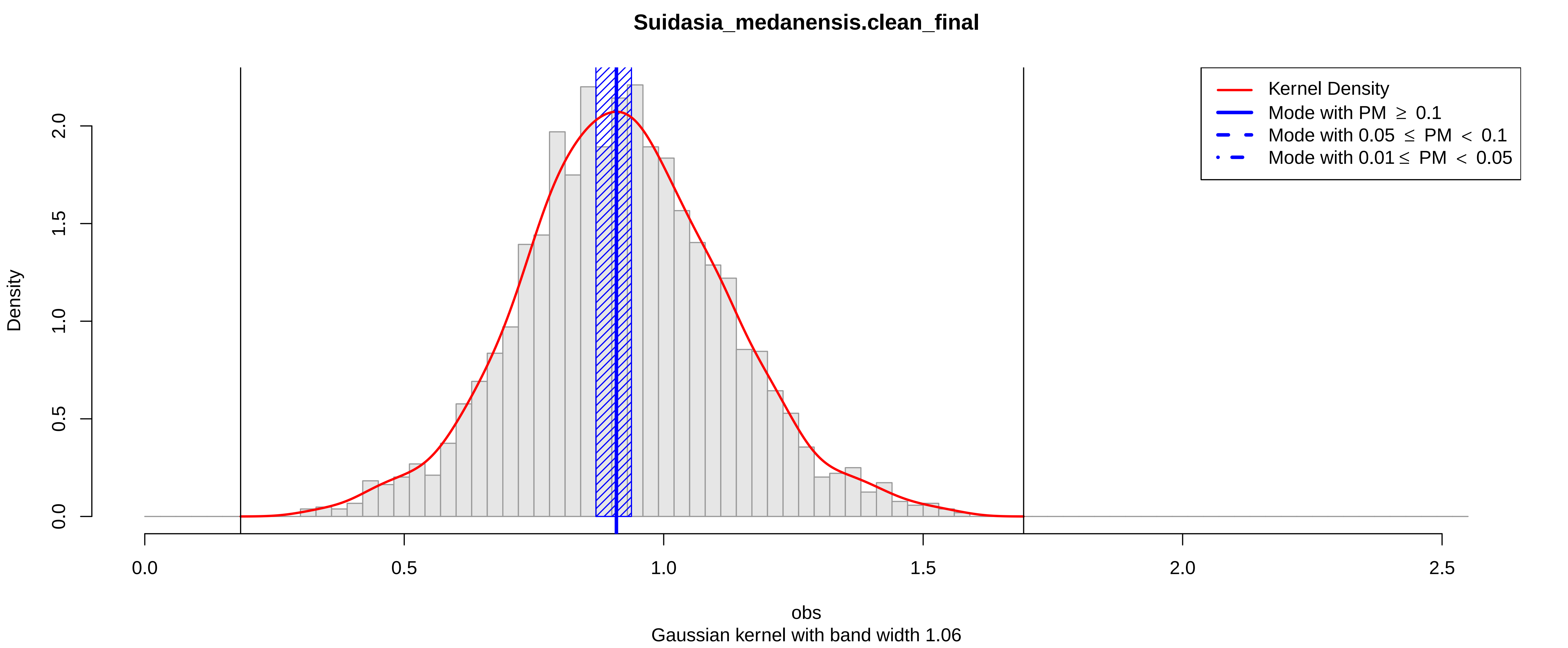
Suidasia_medanensis.clean_final
Kernel Density
Mode with PM ≥ 0.1
Mode with 0.05 ≤ PM < 0.1
Mode with 0.01 ≤ PM < 0.05
Density
0.0
0.5
1.0
1.5
2.0
0.0
0.5
1.0
1.5
2.0
2.5
obs
Gaussian kernel with band width 1.06

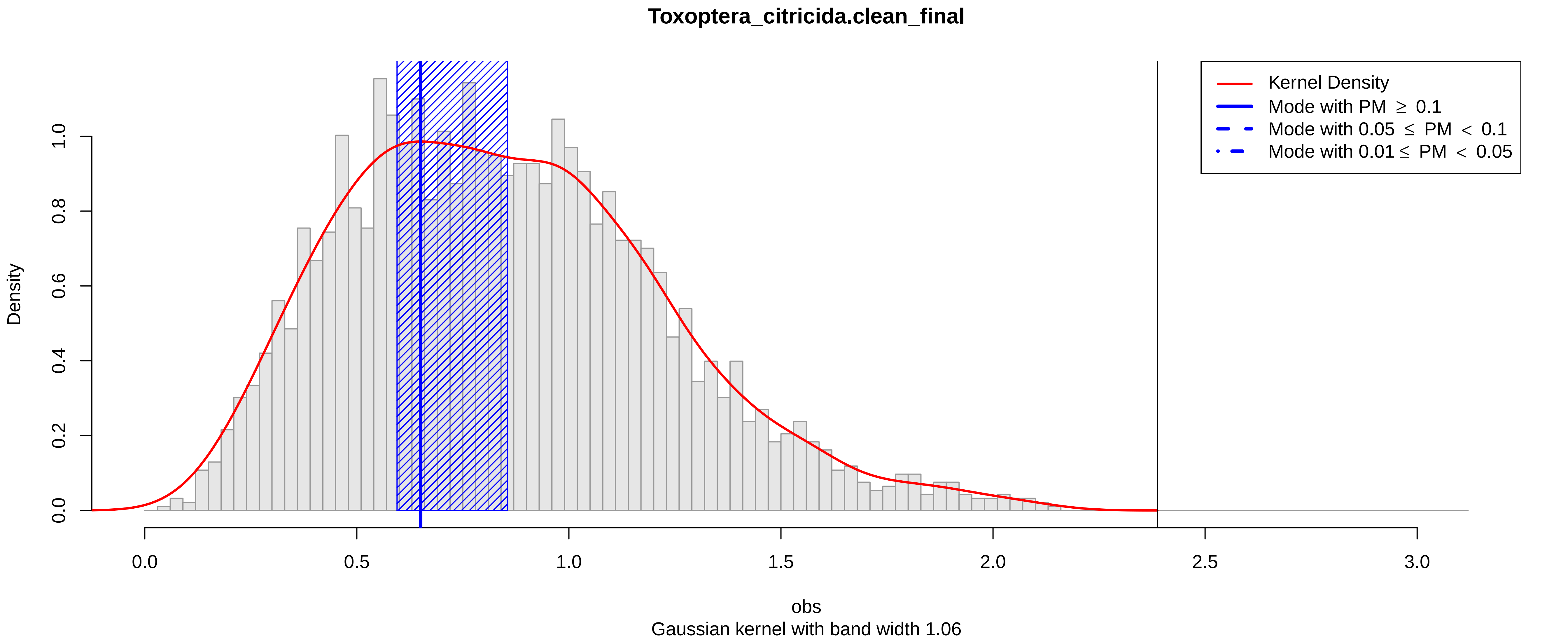

Toxoptera_citricida.clean_final
Kernel Density
Mode with PM ≥ 0.1
Mode with 0.05 ≤ PM < 0.1
Mode with 0.01 ≤ PM < 0.05
Density
0.0
0.2
0.4
0.6
0.8
1.0
0.0
0.5
1.0
1.5
2.0
2.5
3.0
obs
Gaussian kernel with band width 1.06

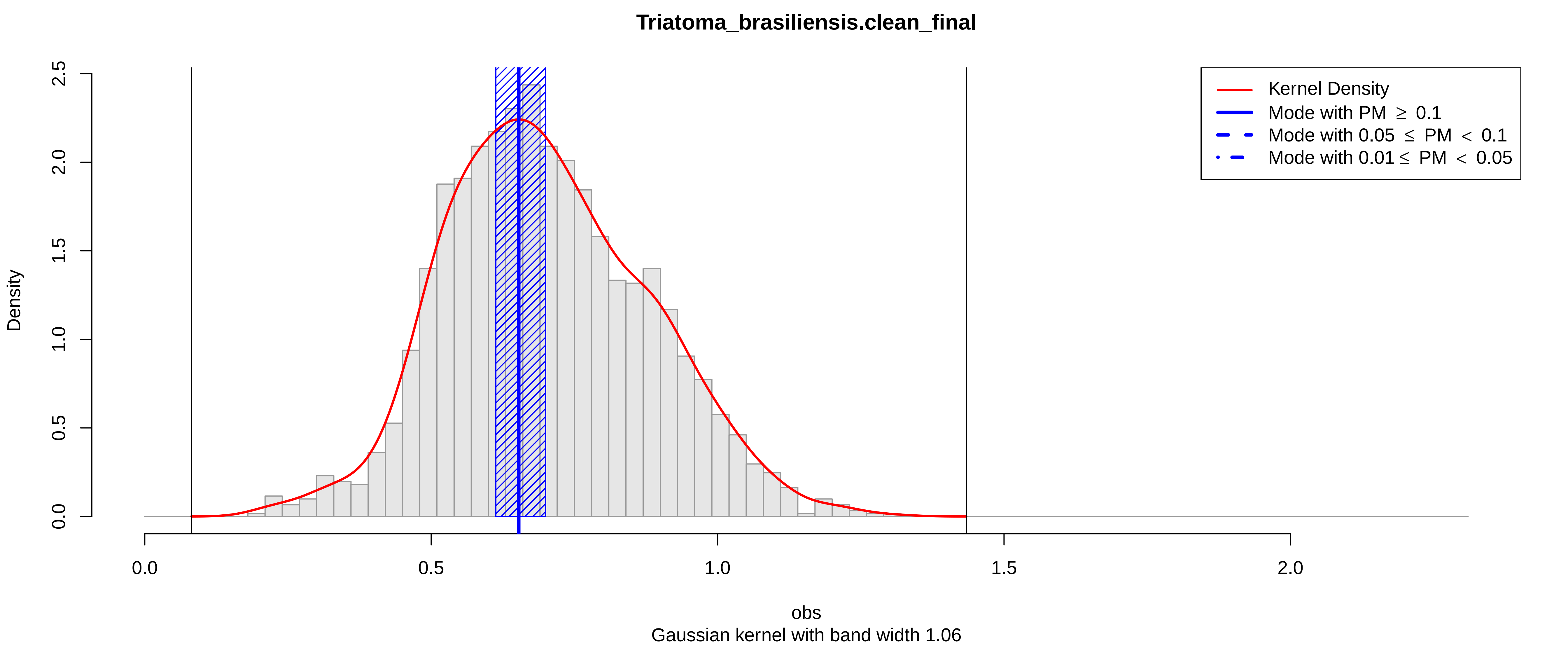
Triatoma_brasiliensis.clean_final
Density
0.0
0.5
1.0
1.5
2.0
2.5
Kernel Density
Mode with PM ≥ 0.1
Mode with 0.05 ≤ PM < 0.1
Mode with 0.01 ≤ PM < 0.05
0.0
0.5
1.0
1.5
2.0
obs
Gaussian kernel with band width 1.06

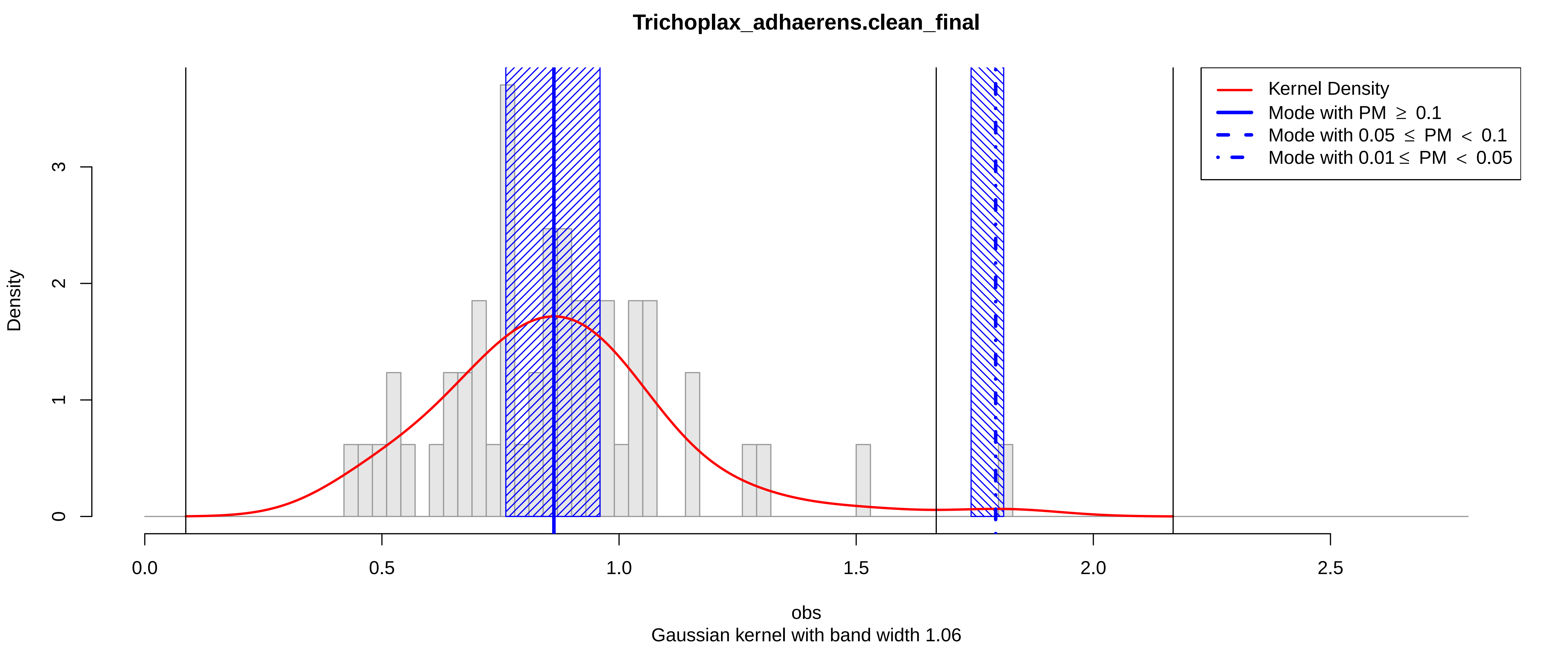

Trichoplax_adhaerens.clean_final
Kernel Density
Mode with PM ≥ 0.1
Mode with 0.05 ≤ PM < 0.1
Mode with 0.01 ≤ PM < 0.05
Density
0
1
2
3
0.0
0.5
1.0
1.5
2.0
2.5
obs
Gaussian kernel with band width 1.06

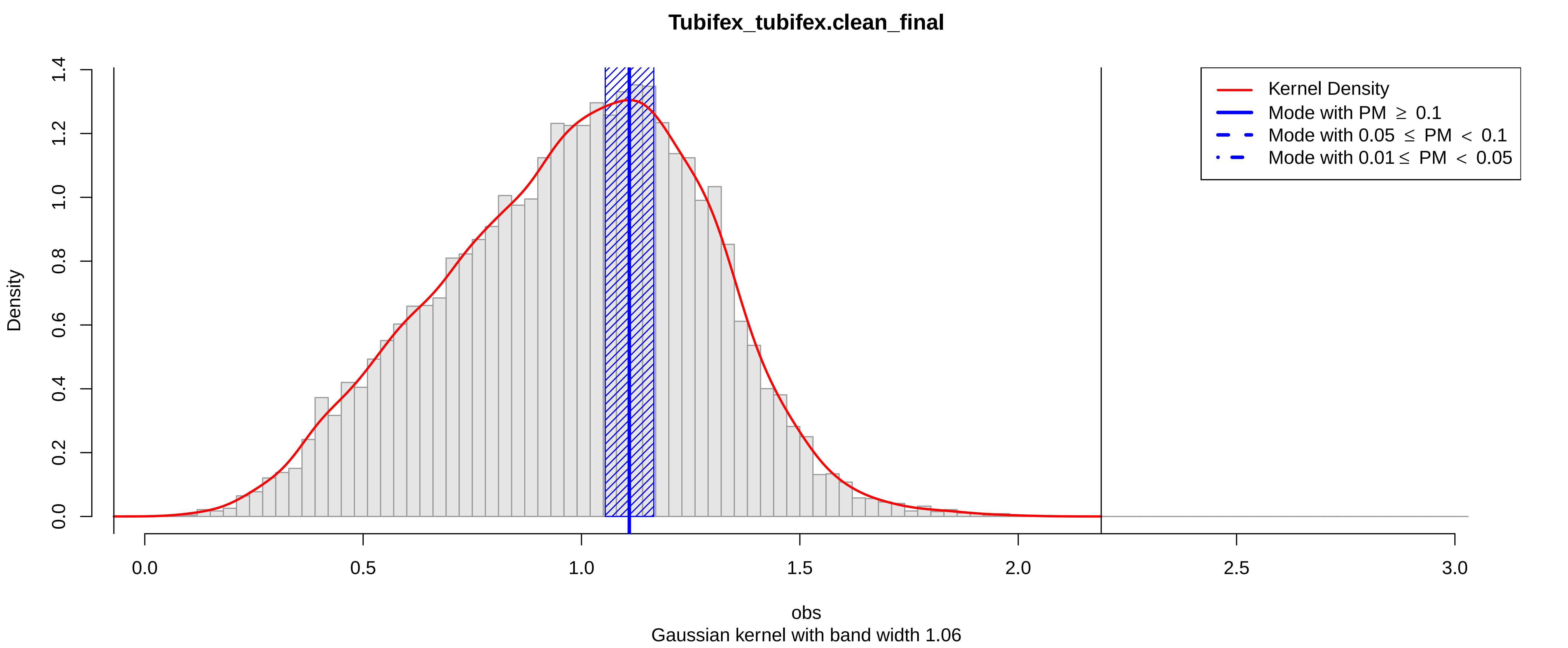

Tubifex_tubifex.clean_final
Density
0.0
0.2
0.4
0.6
0.8
1.0
1.2
1.4
0.0
0.5
1.0
1.5
2.0
2.5
3.0
obs
Gaussian kernel with band width 1.06
Kernel Density
Mode with PM ≥ 0.1
Mode with 0.05 ≤ PM < 0.1
Mode with 0.01 ≤ PM < 0.05
