# Supplementary material for: Universality of the DNA methylation codes in Eucaryotes
Source: Sci Rep. 2019 Jan 17;9:173. doi: 10.1038/s41598-018-37407-8 (PMC6336885; doi:10.1038/s41598-018-37407-8)
Supplement: Supplementary file 3 — Supplementary file 2 [file 41598_2018_37407_MOESM3_ESM.zip › Supplementary_files_02_Histogram_databases_KDE/histogram_kde_Genome.pdf]

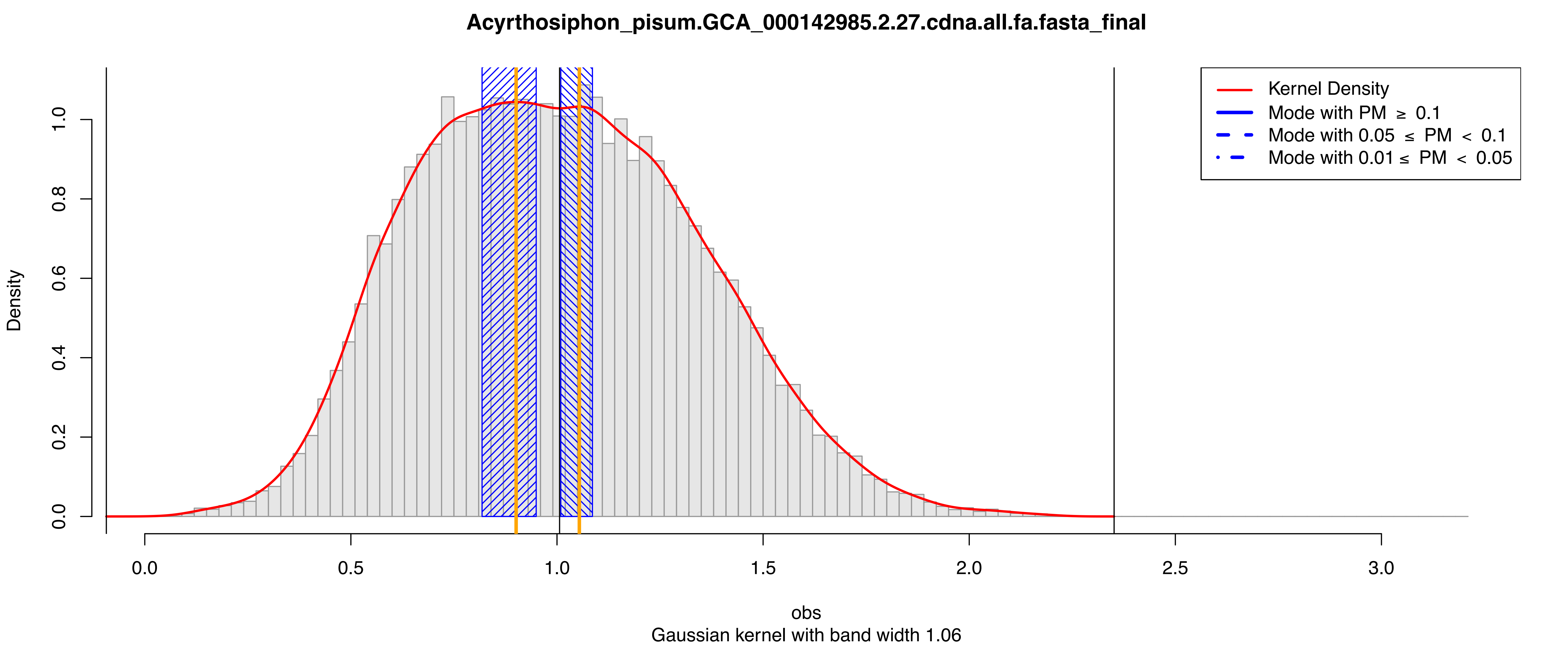

Acyrthosiphon_pisum.GCA_000142985.2.27.cdna.all.fa.fasta_final
Kernel Density
Mode with PM ≥ 0.1
Mode with 0.05 ≤ PM < 0.1
Mode with 0.01 ≤ PM < 0.05
Density
0.0
0.2
0.4
0.6
0.8
1.0
0.0
0.5
1.0
1.5
2.0
2.5
3.0
obs
Gaussian kernel with band width 1.06

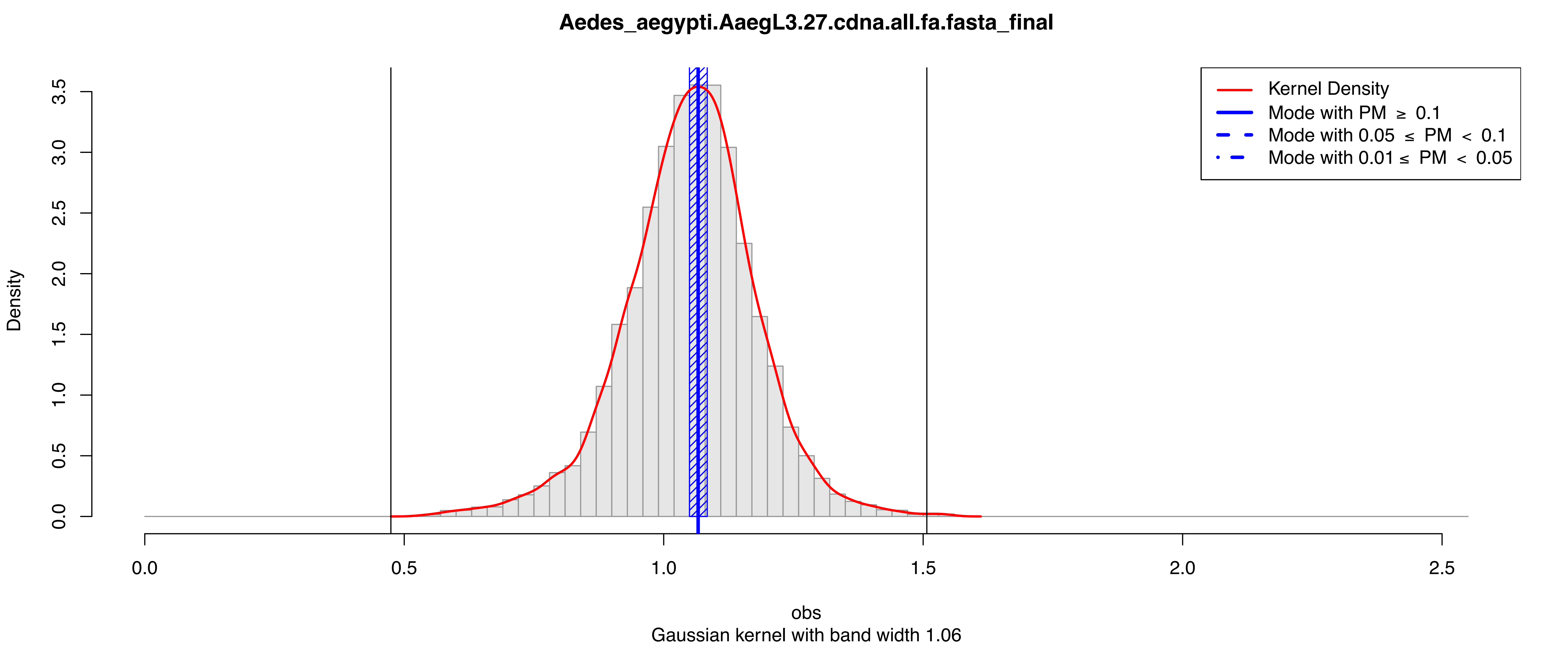

Aedes_aegypti.AaegL3.27.cdna.all.fa.fasta_final
Kernel Density
Mode with PM ≥ 0.1
Mode with 0.05 ≤ PM < 0.1
Mode with 0.01 ≤ PM < 0.05
Density
0.0
0.5
1.0
1.5
2.0
2.5
3.0
3.5
obs
Gaussian kernel with band width 1.06

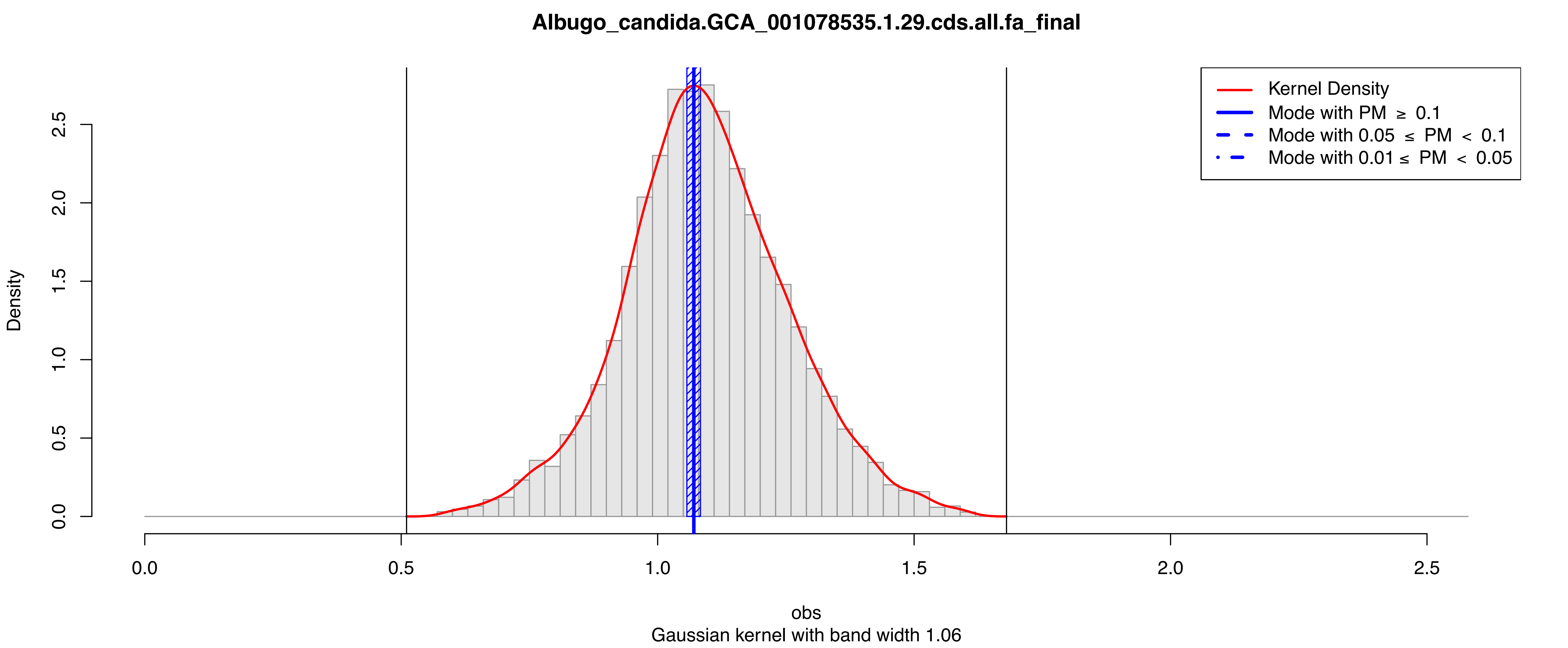

Albugo_candida.GCA_001078535.1.29.cds.all.fa_final
Kernel Density
Mode with PM ≥ 0.1
Mode with 0.05 ≤ PM < 0.1
Mode with 0.01 ≤ PM < 0.05
Density
0.0
0.5
1.0
1.5
2.0
2.5
obs
Gaussian kernel with band width 1.06

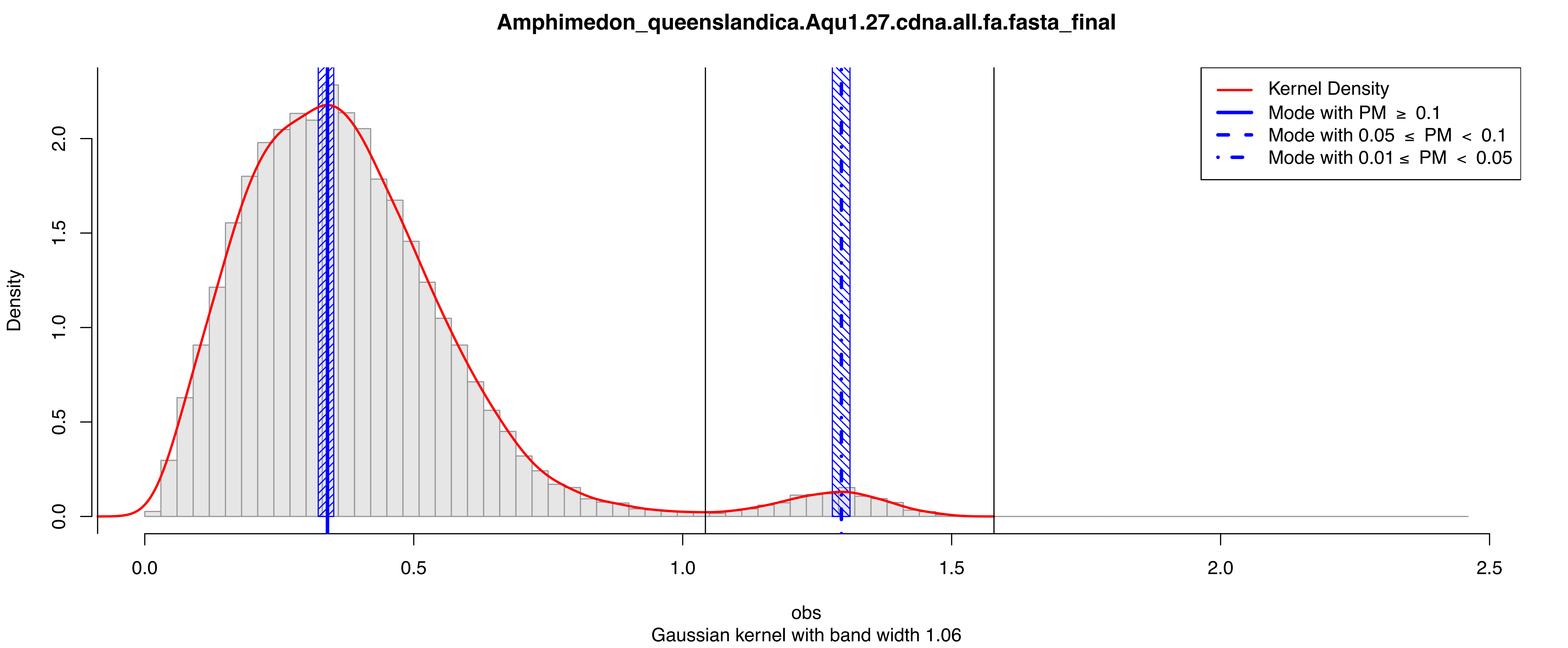

Amphimedon_queenslandica.Aqu1.27.cdna.all.fa.fasta_final
Kernel Density
Mode with PM ≥ 0.1
Mode with 0.05 ≤ PM < 0.1
Mode with 0.01 ≤ PM < 0.05
Density
0.0
0.5
1.0
1.5
2.0
2.5
obs
Gaussian kernel with band width 1.06

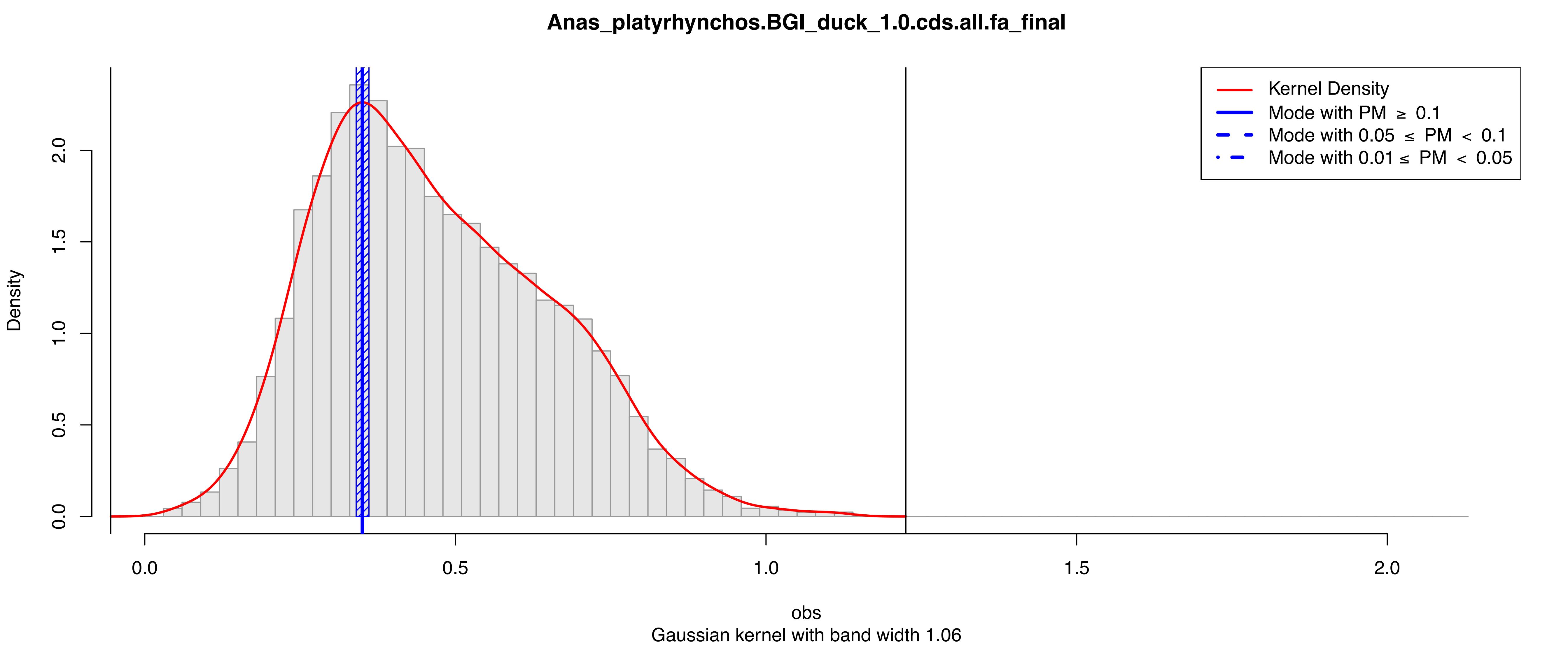
Anas_platyrhynchos.BGI_duck_1.0.cds.all.fa_final
Kernel Density
Mode with PM ≥ 0.1
Mode with 0.05 ≤ PM < 0.1
Mode with 0.01 ≤ PM < 0.05
Density
0.0
0.5
1.0
1.5
2.0
0.0
0.5
1.0
1.5
2.0
obs
Gaussian kernel with band width 1.06

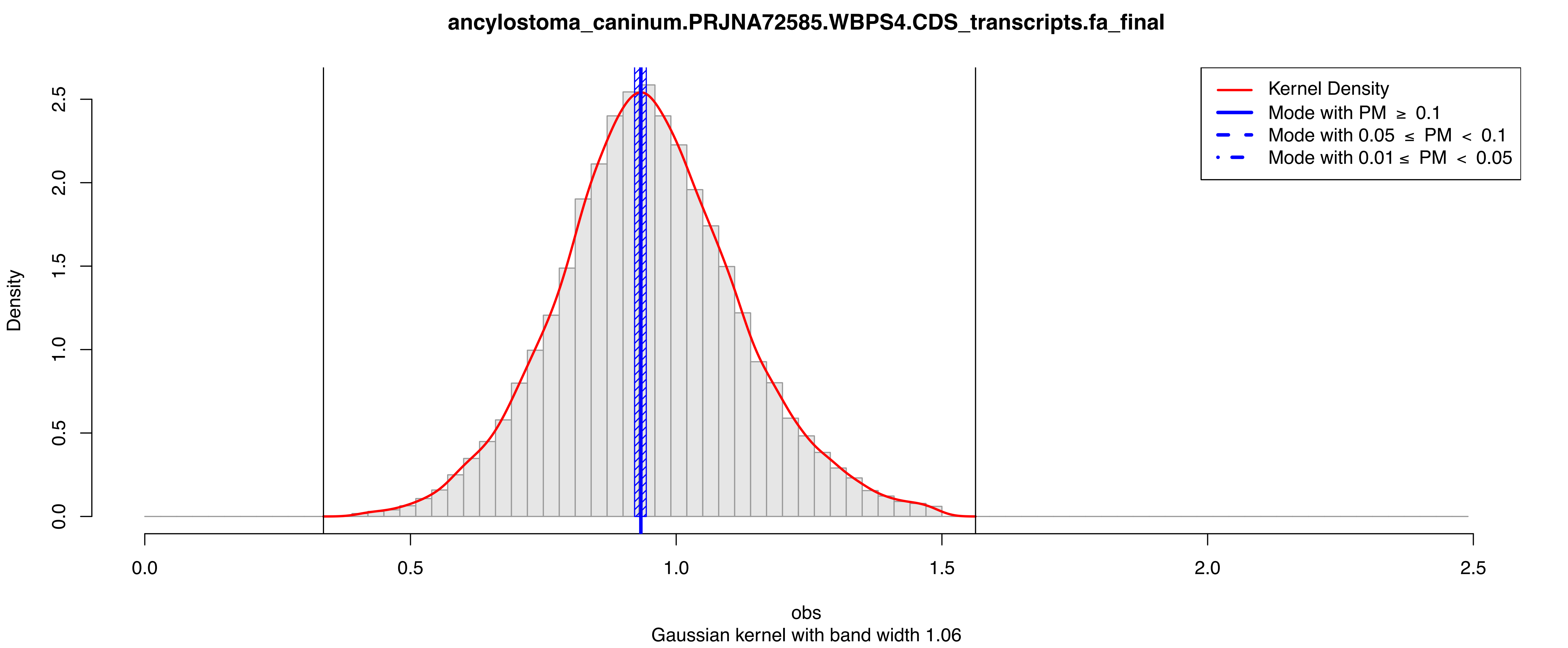
ancylostoma_caninum.PRJNA72585.WBPS4.CDS_transcripts.fa_final
Kernel Density
Mode with PM ≥ 0.1
Mode with 0.05 ≤ PM < 0.1
Mode with 0.01 ≤ PM < 0.05
Density
0.0
0.5
1.0
1.5
2.0
2.5
obs
Gaussian kernel with band width 1.06

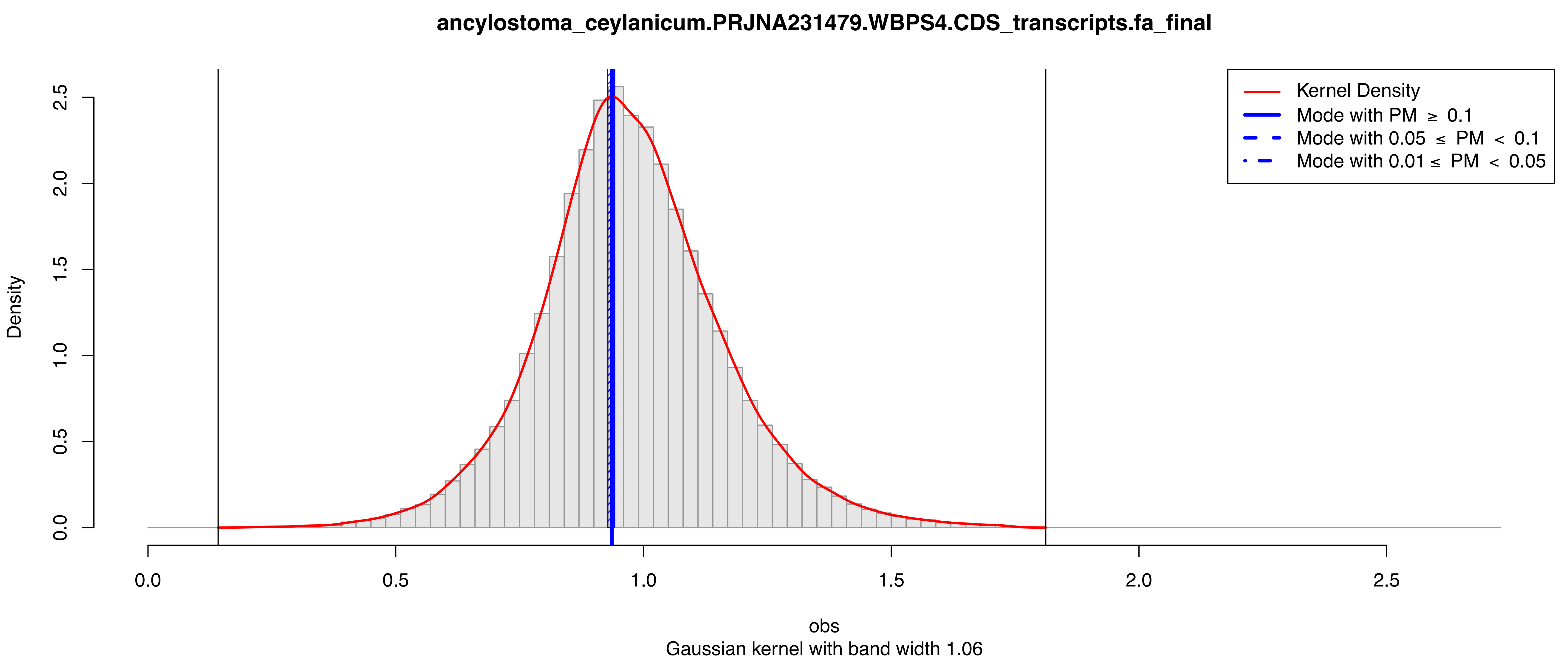
ancylostoma_ceylanicum.PRJNA231479.WBPS4.CDS_transcripts.fa_final
Kernel Density
Mode with PM ≥ 0.1
Mode with 0.05 ≤ PM < 0.1
Mode with 0.01 ≤ PM < 0.05
Density
0.0
0.5
1.0
1.5
2.0
2.5
0.0
0.5
1.0
1.5
2.0
2.5
obs
Gaussian kernel with band width 1.06

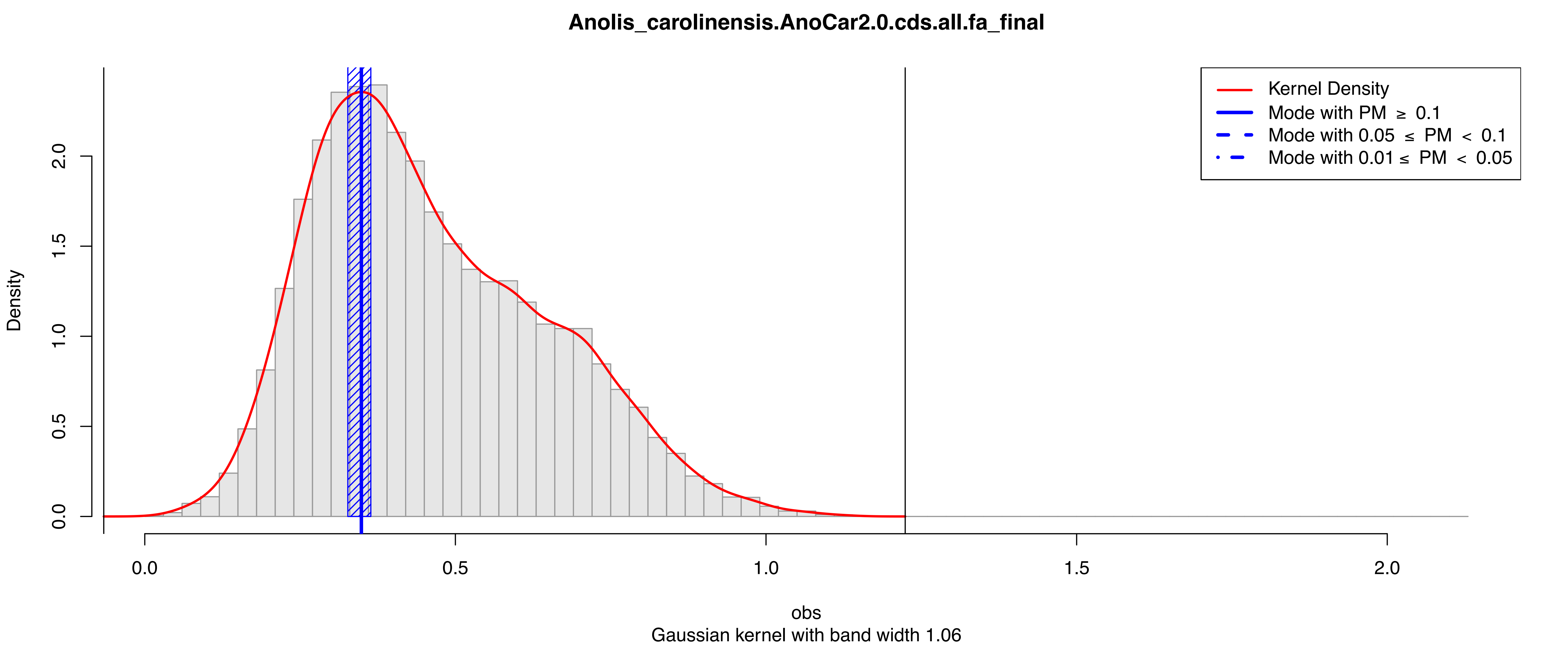

Anolis_carolinensis.AnoCar2.0.cds.all.fa_final
Kernel Density
Mode with PM ≥ 0.1
Mode with 0.05 ≤ PM < 0.1
Mode with 0.01 ≤ PM < 0.05
Density
0.0
0.5
1.0
1.5
2.0
obs
Gaussian kernel with band width 1.06

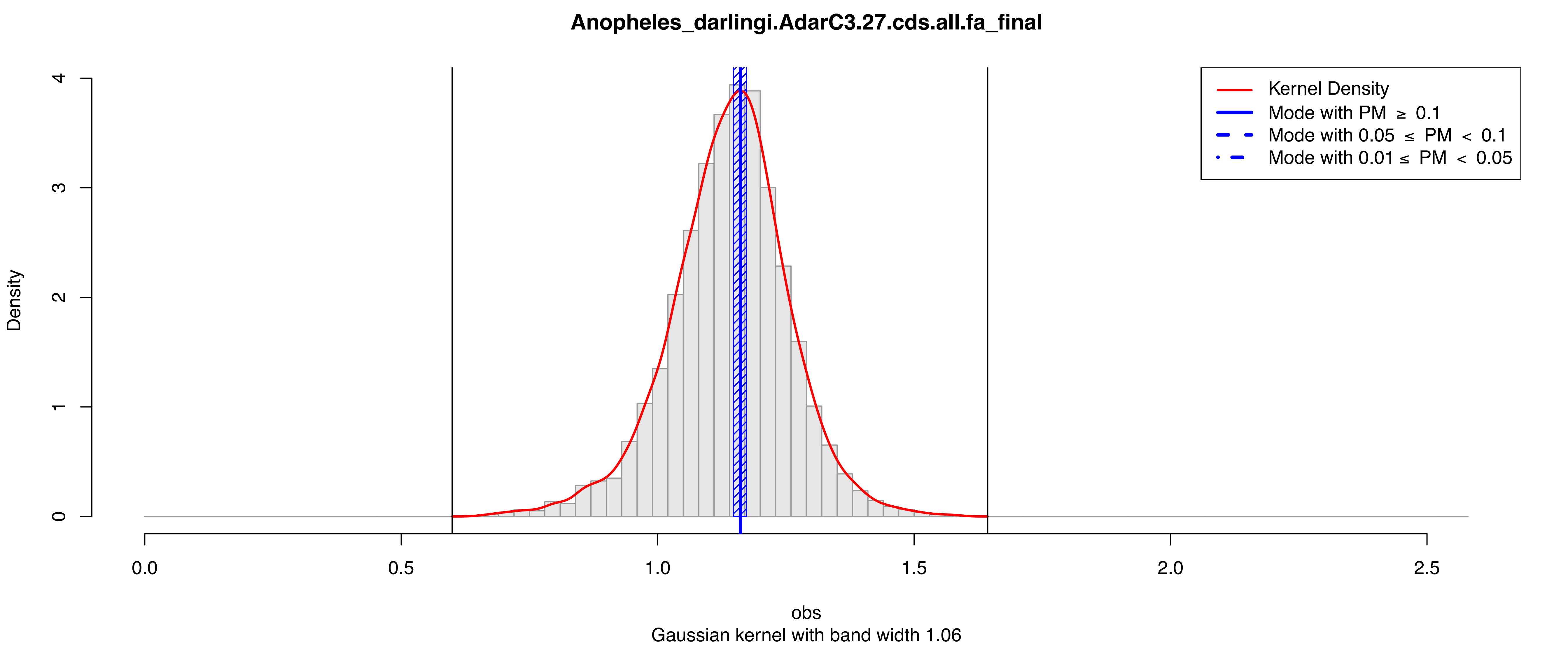

Anopheles_darlingi.AdarC3.27.cds.all.fa_final
Density
0
1
2
3
4
0.0
0.5
1.0
1.5
2.0
2.5
obs
Gaussian kernel with band width 1.06
Kernel Density
Mode with PM ≥ 0.1
Mode with 0.05 ≤ PM < 0.1
Mode with 0.01 ≤ PM < 0.05

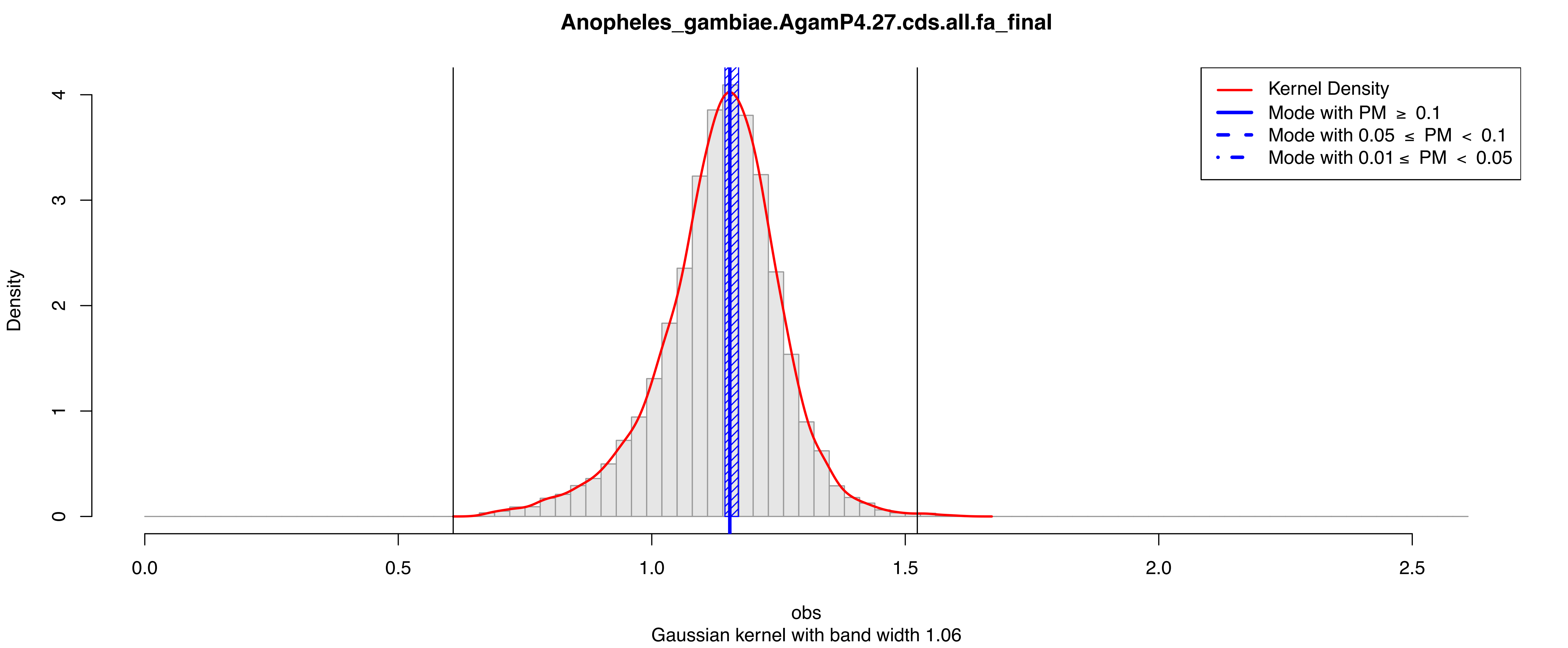

Anopheles_gambiae.AgamP4.27.cds.all.fa_final
Density
0
1
2
3
4
0.0
0.5
1.0
1.5
2.0
2.5
obs
Gaussian kernel with band width 1.06
Kernel Density
Mode with PM ≥ 0.1
Mode with 0.05 ≤ PM < 0.1
Mode with 0.01 ≤ PM < 0.05

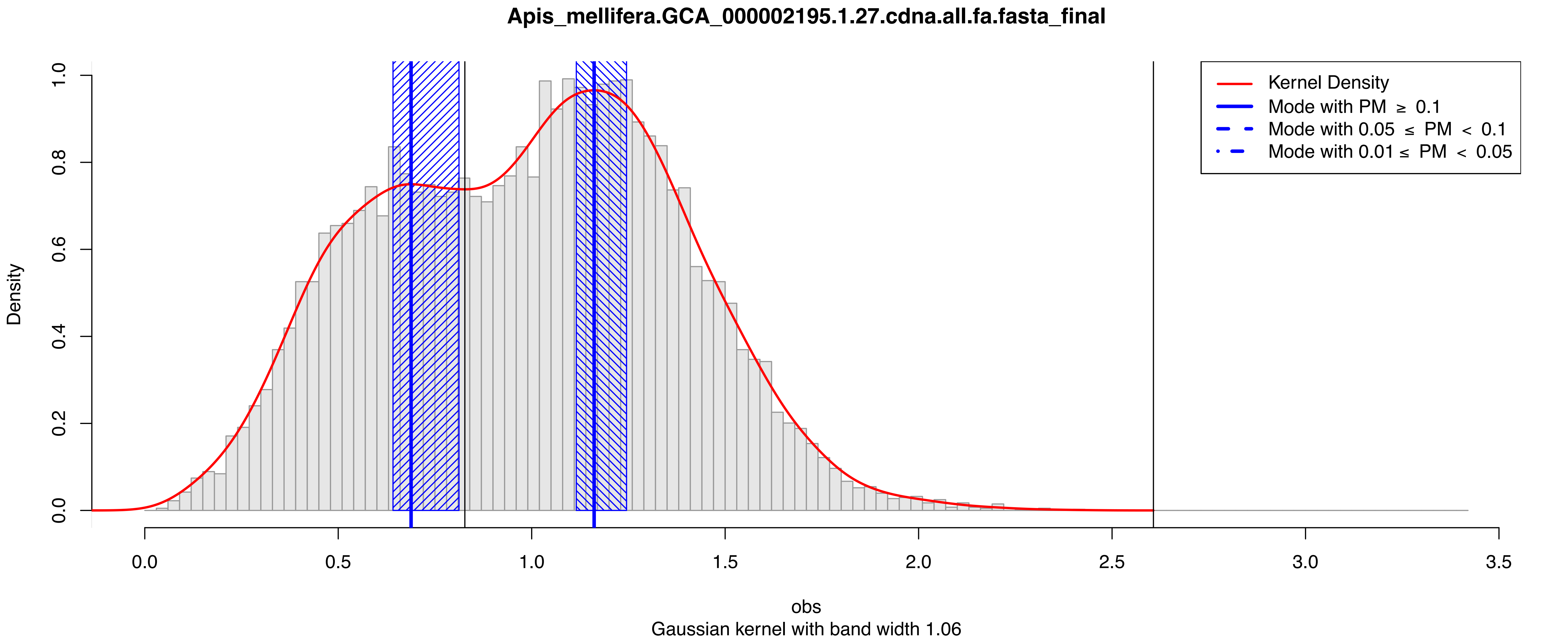
Apis_mellifera.GCA_000002195.1.27.cdna.all.fa.fasta_final
Kernel Density
Mode with PM ≥ 0.1
Mode with 0.05 ≤ PM < 0.1
Mode with 0.01 ≤ PM < 0.05
Density
0.0
0.2
0.4
0.6
0.8
1.0
0.0
0.5
1.0
1.5
2.0
2.5
3.0
3.5
obs
Gaussian kernel with band width 1.06

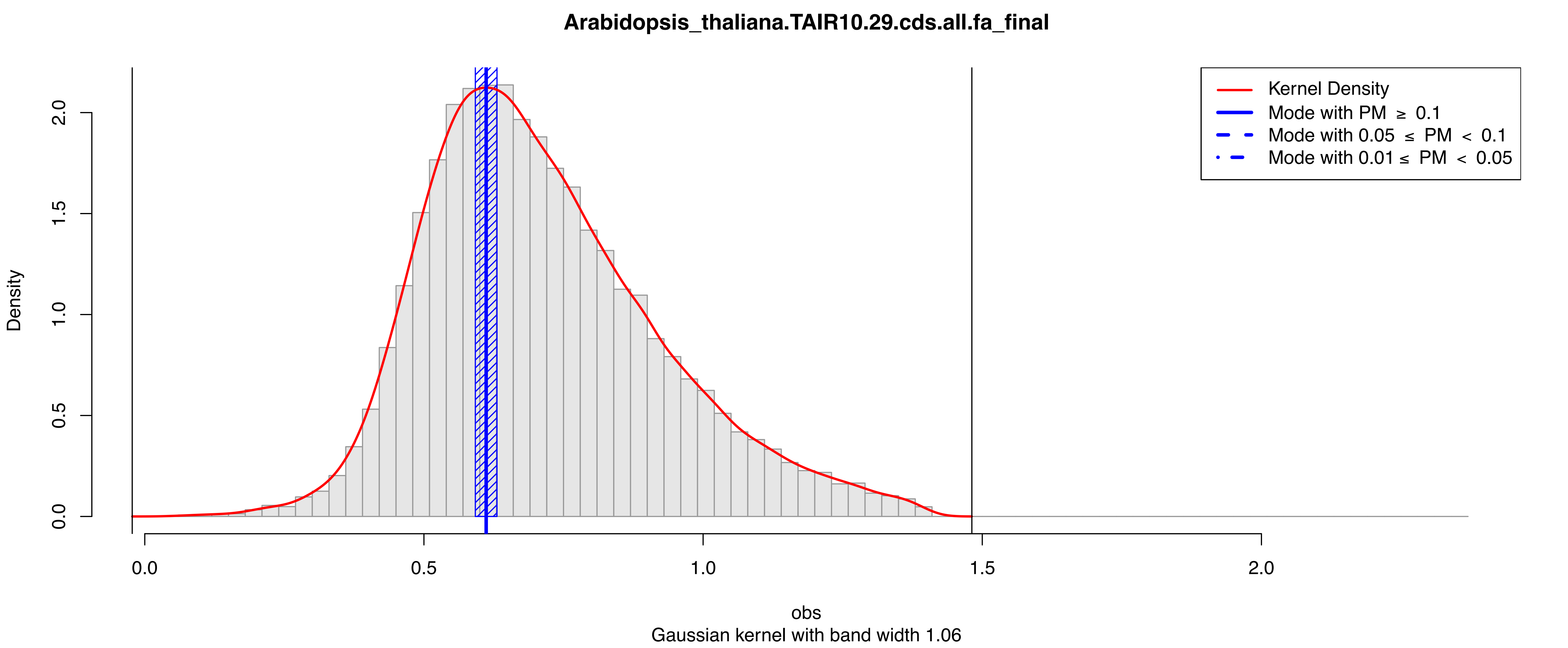
Arabidopsis_thaliana.TAIR10.29.cds.all.fa_final
Kernel Density
Mode with PM ≥ 0.1
Mode with 0.05 ≤ PM < 0.1
Mode with 0.01 ≤ PM < 0.05
Density
0.0
0.5
1.0
1.5
2.0
obs
Gaussian kernel with band width 1.06

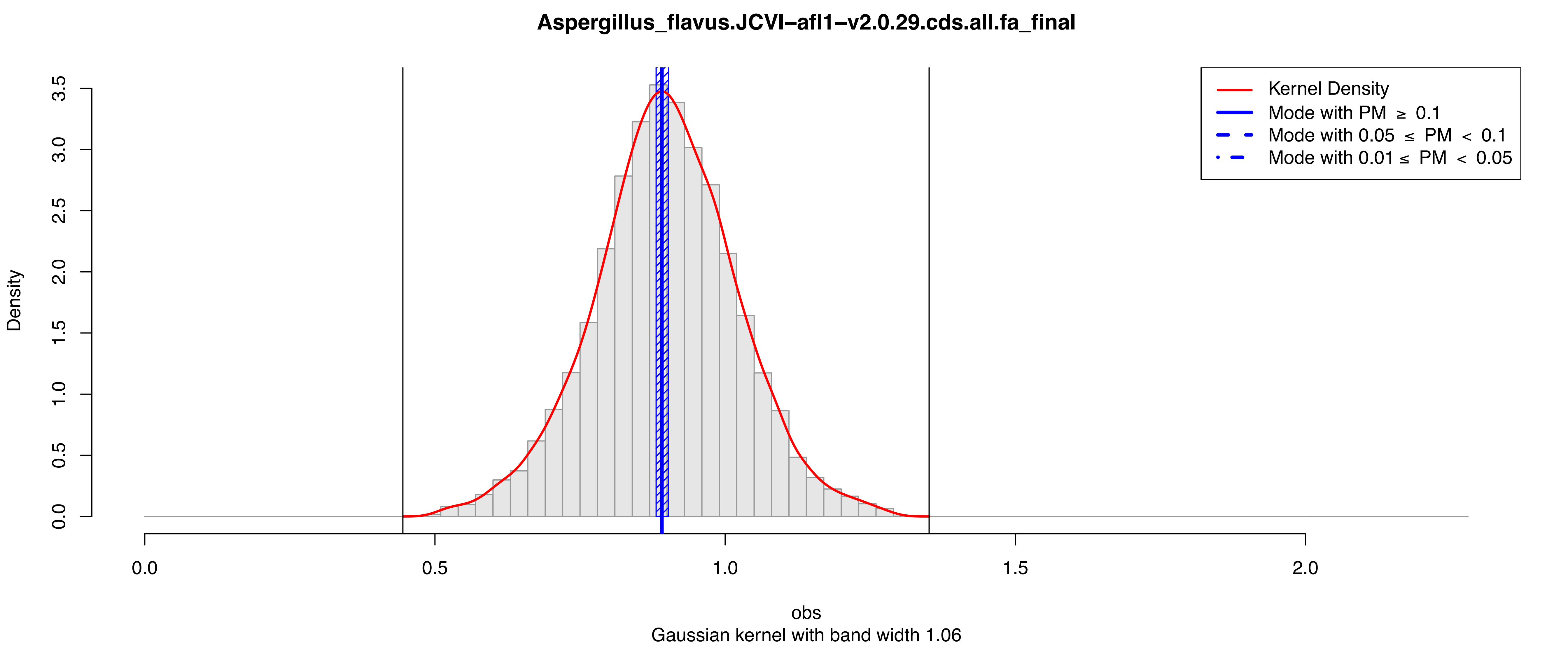
Aspergillus_flavus.JCVI–afl1–v2.0.29.cds.all.fa_final
Density
0.0
0.5
1.0
1.5
2.0
2.5
3.0
3.5
Kernel Density
Mode with PM ≥ 0.1
Mode with 0.05 ≤ PM < 0.1
Mode with 0.01 ≤ PM < 0.05
0.0
0.5
1.0
1.5
2.0
obs
Gaussian kernel with band width 1.06

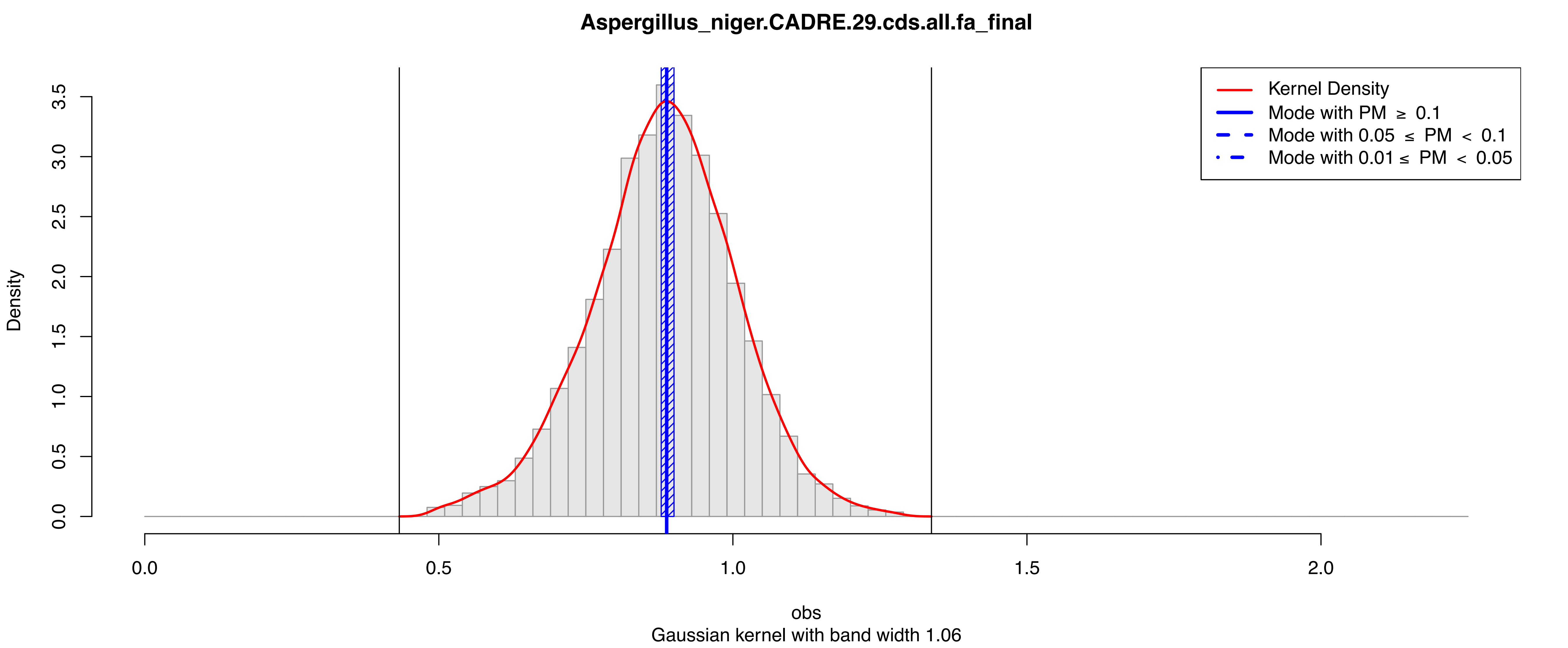

Aspergillus_niger.CADRE.29.cds.all.fa_final
Kernel Density
Mode with PM ≥ 0.1
Mode with 0.05 ≤ PM < 0.1
Mode with 0.01 ≤ PM < 0.05
Density
0.0
0.5
1.0
1.5
2.0
2.5
3.0
3.5
0.0
0.5
1.0
1.5
2.0
obs
Gaussian kernel with band width 1.06

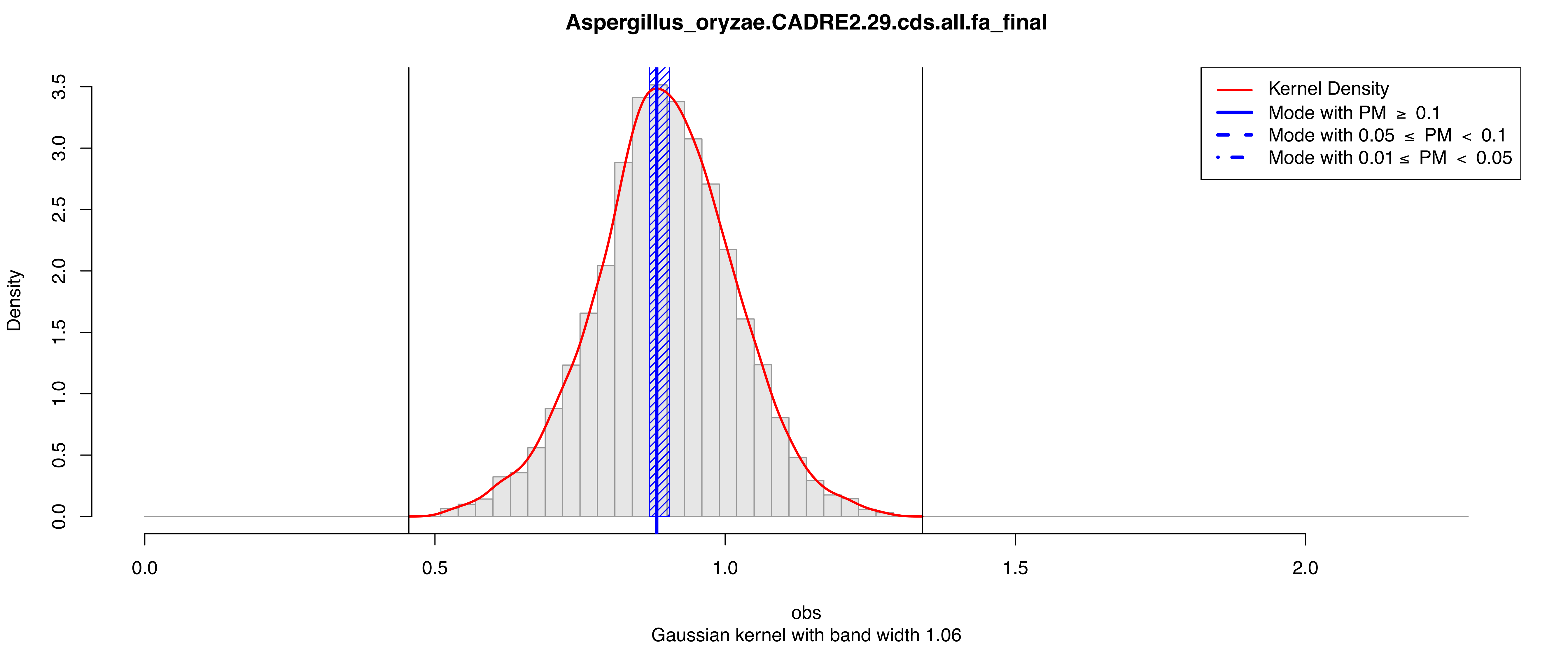

Aspergillus_oryzae.CADRE2.29.cds.all.fa_final
Kernel Density
Mode with PM ≥ 0.1
Mode with 0.05 ≤ PM < 0.1
Mode with 0.01 ≤ PM < 0.05
Density
0.0
0.5
1.0
1.5
2.0
2.5
3.0
3.5
0.0
0.5
1.0
1.5
2.0
obs
Gaussian kernel with band width 1.06

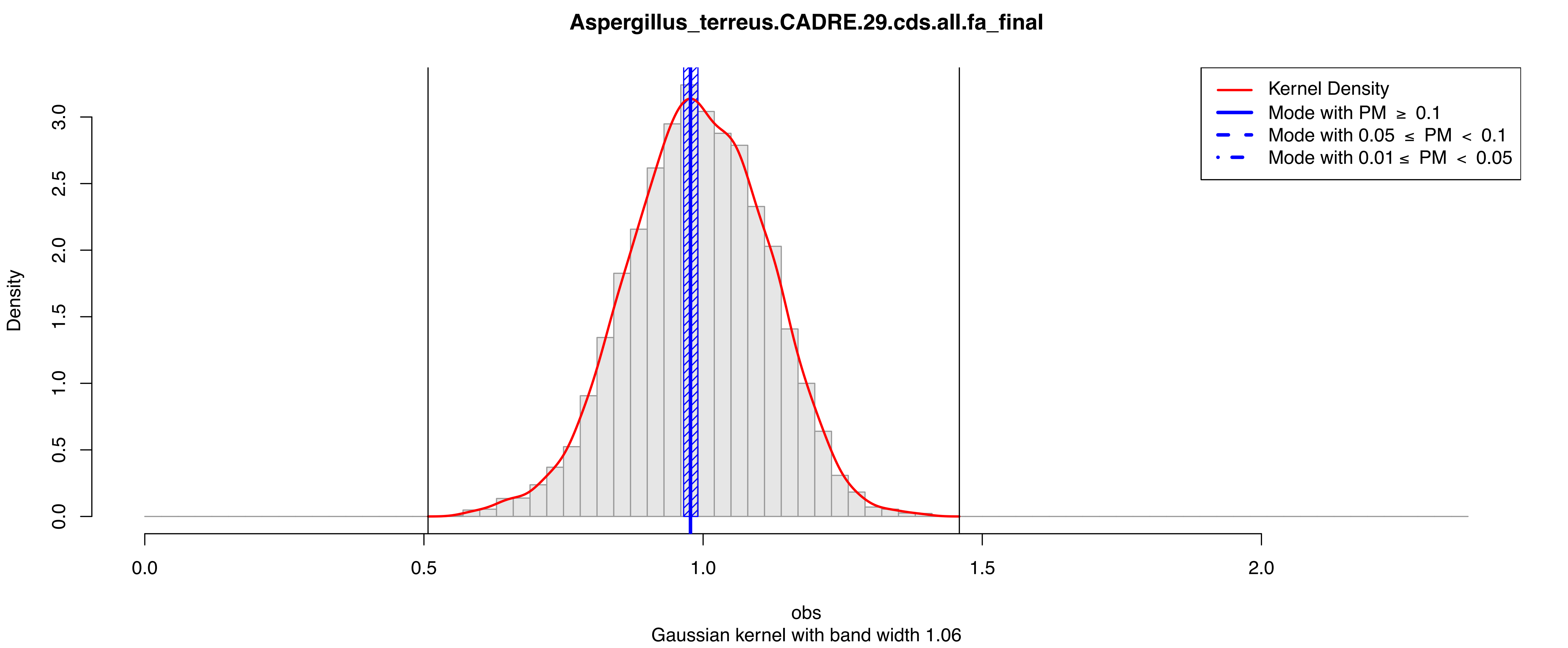
Aspergillus_terreus.CADRE.29.cds.all.fa_final
Kernel Density
Mode with PM ≥ 0.1
Mode with 0.05 ≤ PM < 0.1
Mode with 0.01 ≤ PM < 0.05
Density
0.0
0.5
1.0
1.5
2.0
2.5
3.0
0.0
0.5
1.0
1.5
2.0
obs
Gaussian kernel with band width 1.06

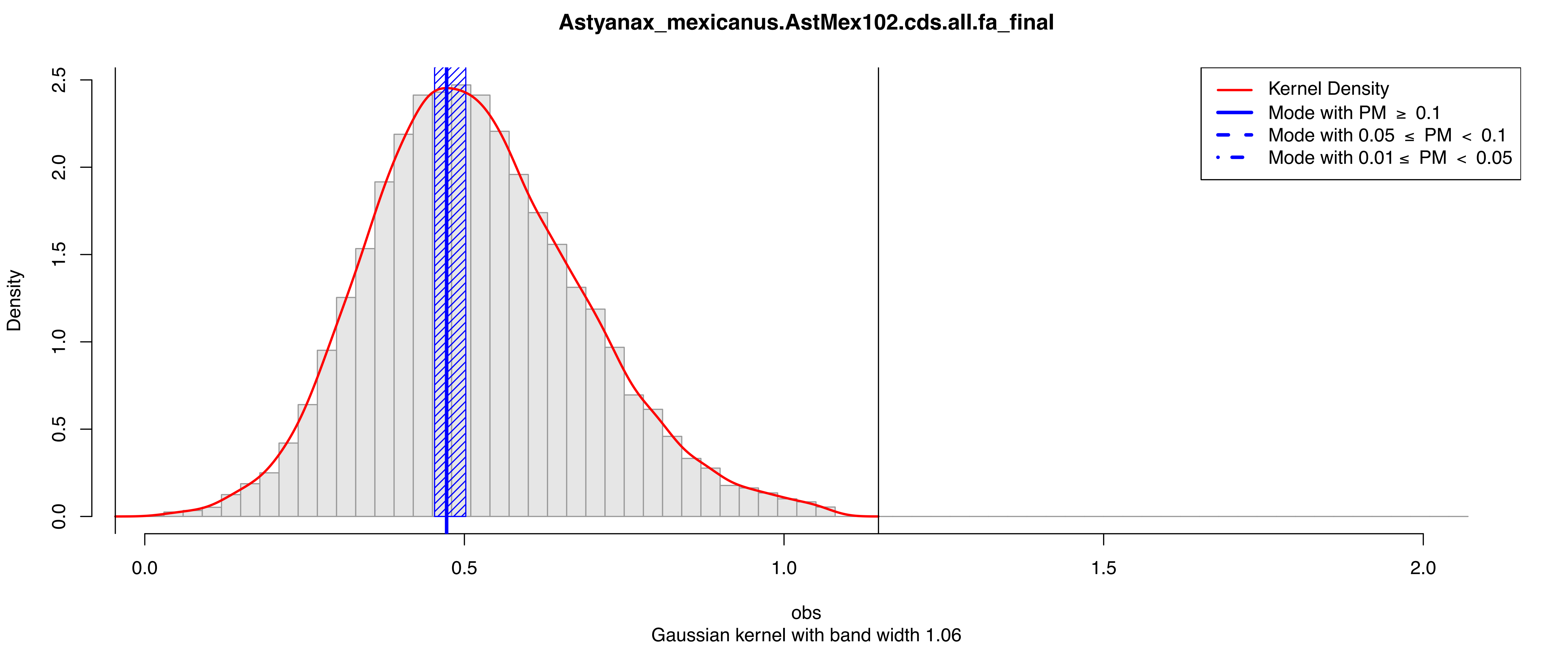

Astyanax_mexicanus.AstMex102.cds.all.fa_final
Kernel Density
Mode with PM ≥ 0.1
Mode with 0.05 ≤ PM < 0.1
Mode with 0.01 ≤ PM < 0.05
Density
0.0
0.5
1.0
1.5
2.0
2.5
obs
Gaussian kernel with band width 1.06

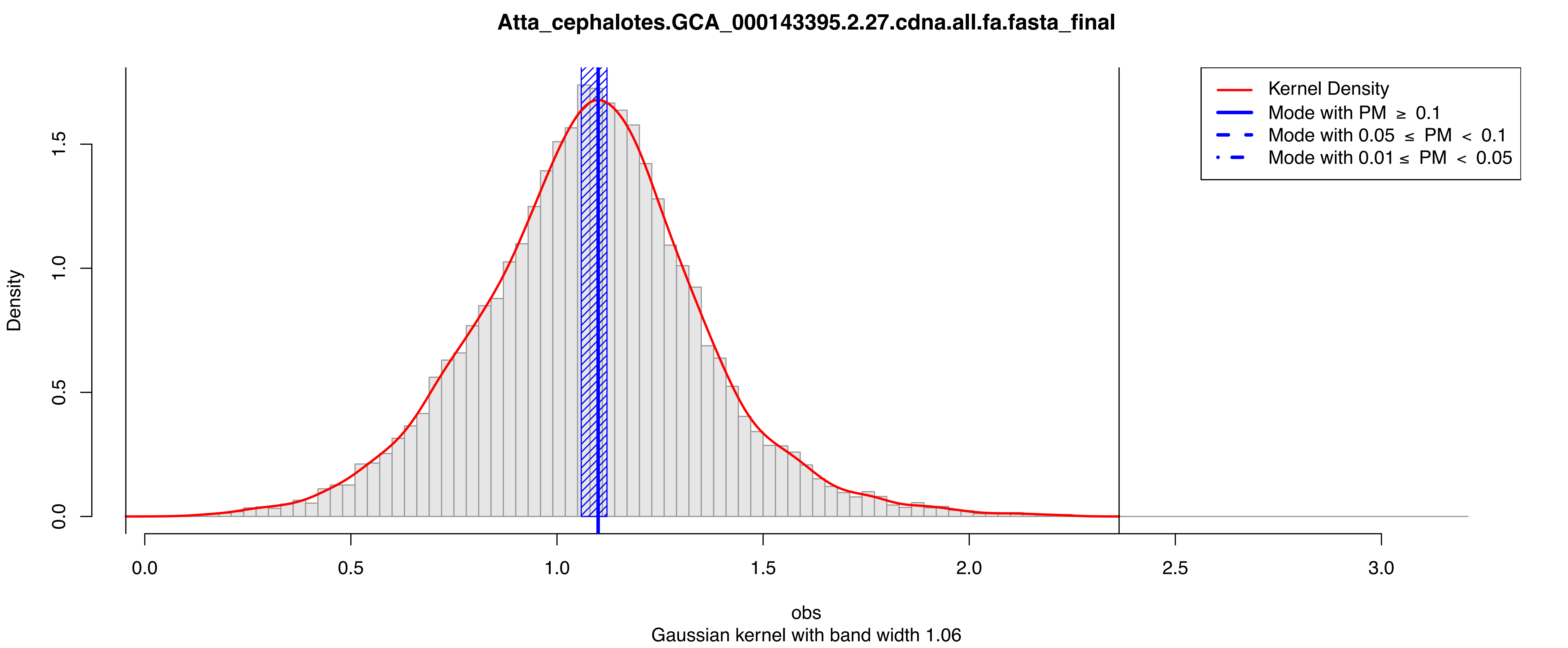

Atta_cephalotes.GCA_000143395.2.27.cdna.all.fa.fasta_final
Kernel Density
Mode with PM ≥ 0.1
Mode with 0.05 ≤ PM < 0.1
Mode with 0.01 ≤ PM < 0.05
Density
0.0
0.5
1.0
1.5
0.0
0.5
1.0
1.5
2.0
2.5
3.0
obs
Gaussian kernel with band width 1.06

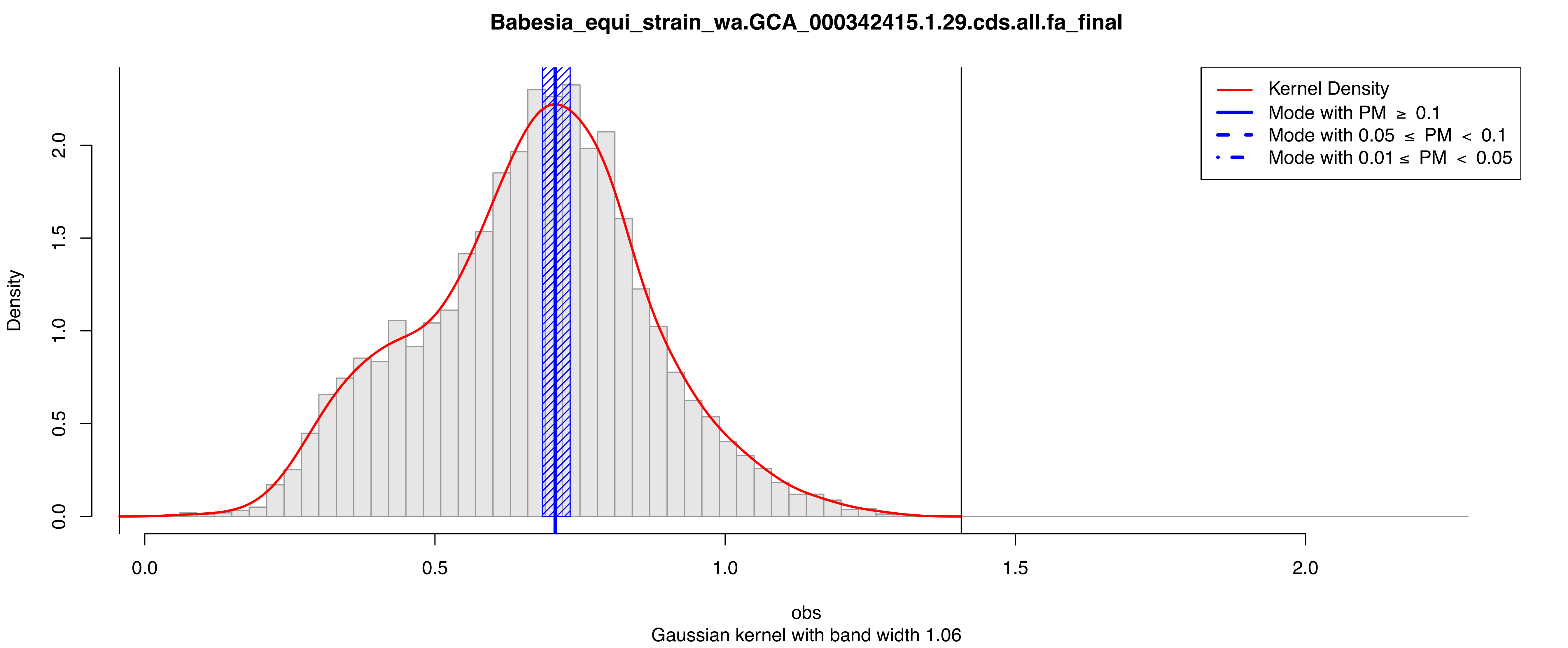

Babesia_equi_strain_wa.GCA_000342415.1.29.cds.all.fa_final
Kernel Density
Mode with PM ≥ 0.1
Mode with 0.05 ≤ PM < 0.1
Mode with 0.01 ≤ PM < 0.05
Density
0.0
0.5
1.0
1.5
2.0
0.0
0.5
1.0
1.5
2.0
obs
Gaussian kernel with band width 1.06

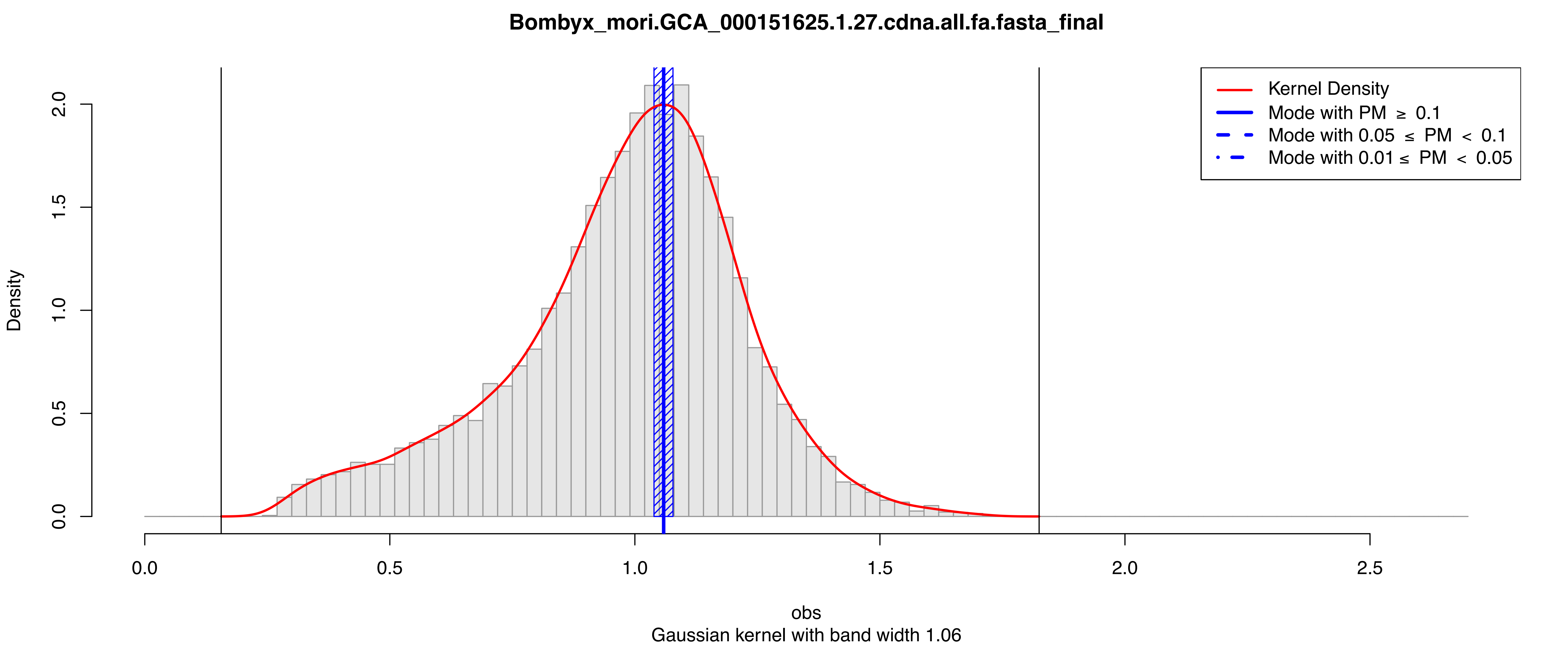

Bombyx_mori.GCA_000151625.1.27.cdna.all.fa.fasta_final
Kernel Density
Mode with PM ≥ 0.1
Mode with 0.05 ≤ PM < 0.1
Mode with 0.01 ≤ PM < 0.05
Density
0.0
0.5
1.0
1.5
2.0
0.0
0.5
1.0
1.5
2.0
2.5
obs
Gaussian kernel with band width 1.06

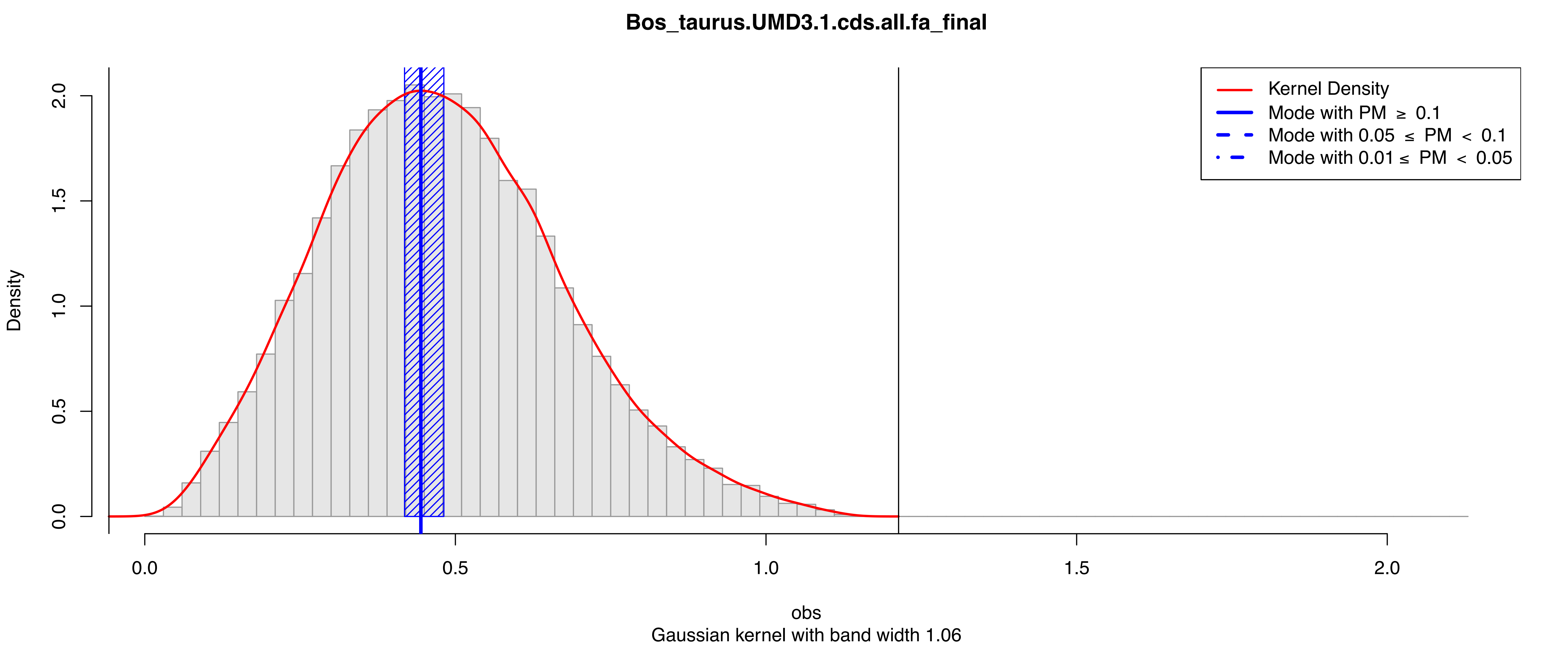

Bos_taurus.UMD3.1.cds.all.fa_final
Kernel Density
Mode with PM ≥ 0.1
Mode with 0.05 ≤ PM < 0.1
Mode with 0.01 ≤ PM < 0.05
Density
0.0
0.5
1.0
1.5
2.0
obs
Gaussian kernel with band width 1.06

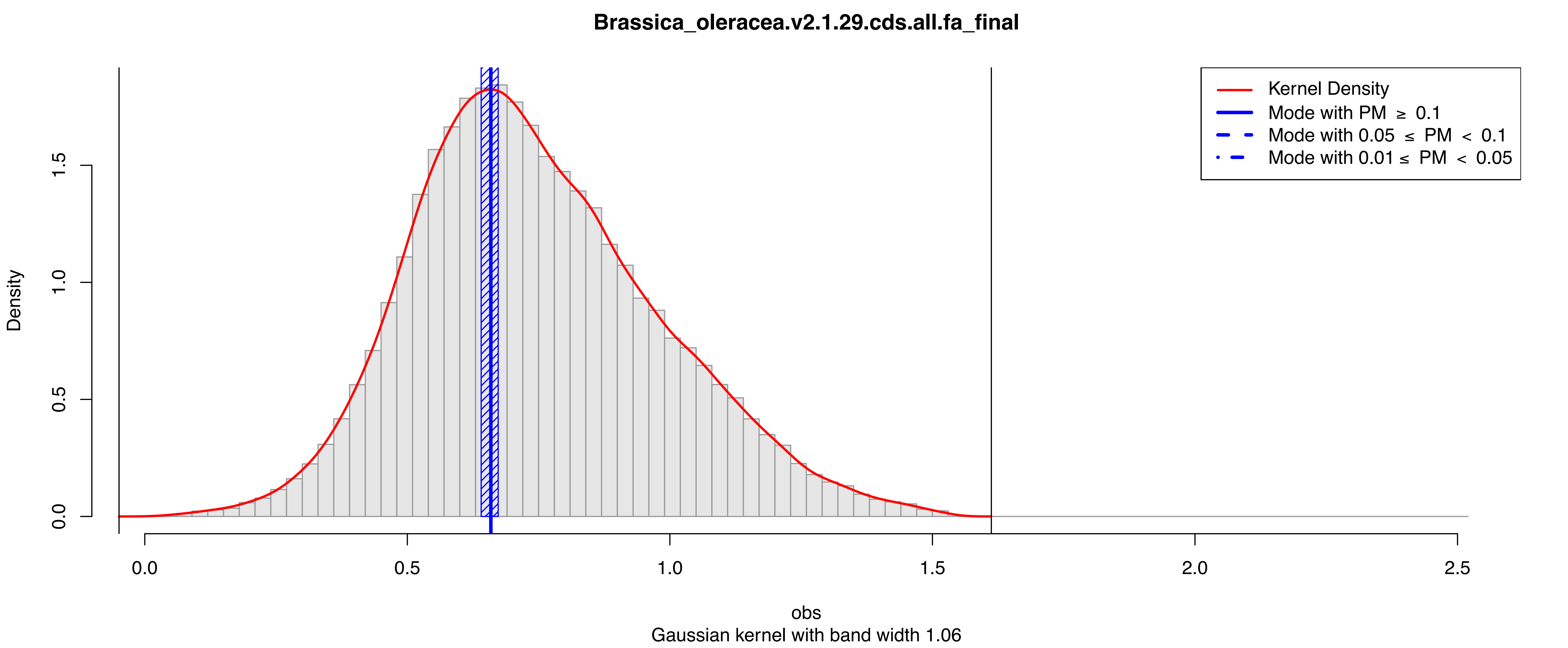

Brassica_oleracea.v2.1.29.cds.all.fa_final
Kernel Density
Mode with PM ≥ 0.1
Mode with 0.05 ≤ PM < 0.1
Mode with 0.01 ≤ PM < 0.05
Density
0.0
0.5
1.0
1.5
0.0
0.5
1.0
1.5
2.0
2.5
obs
Gaussian kernel with band width 1.06

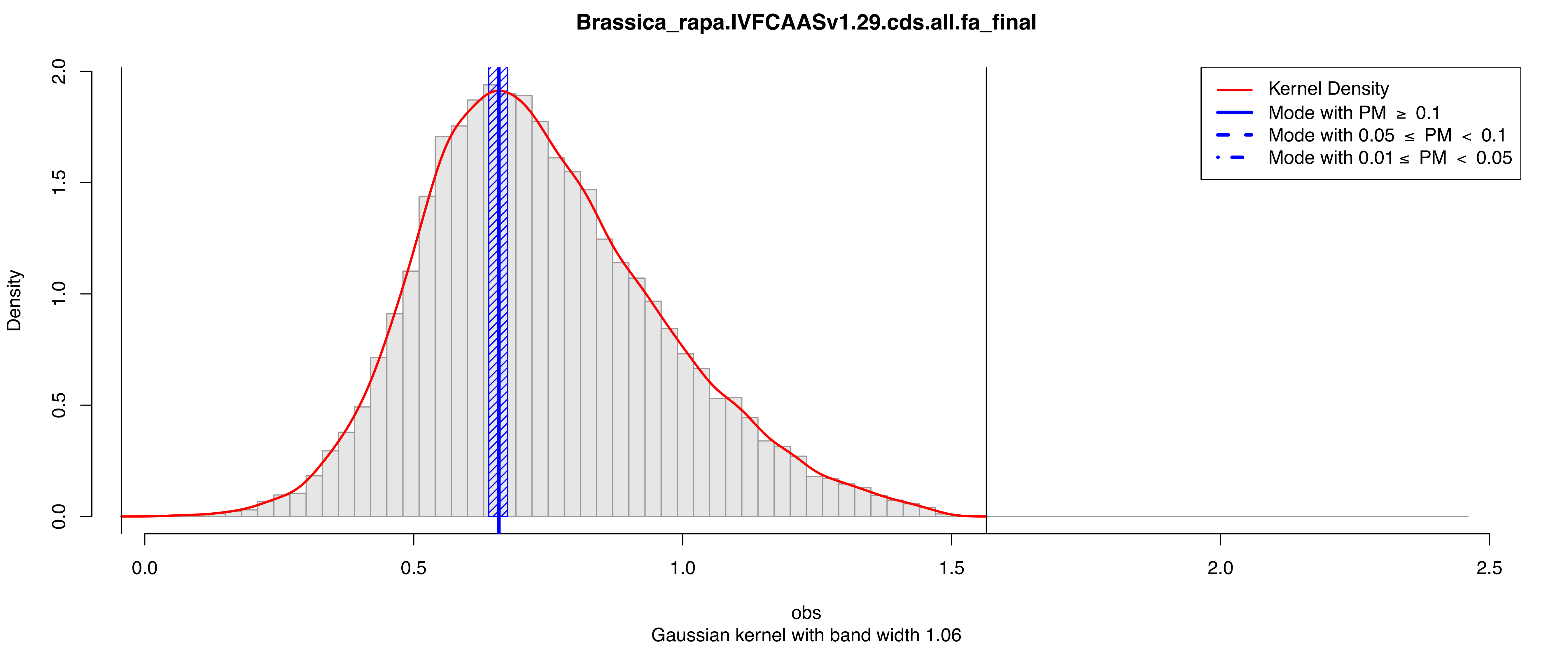
Brassica_rapa.IVFCAASv1.29.cds.all.fa_final
Kernel Density
Mode with PM ≥ 0.1
Mode with 0.05 ≤ PM < 0.1
Mode with 0.01 ≤ PM < 0.05
Density
0.0
0.5
1.0
1.5
2.0
2.5
obs
Gaussian kernel with band width 1.06

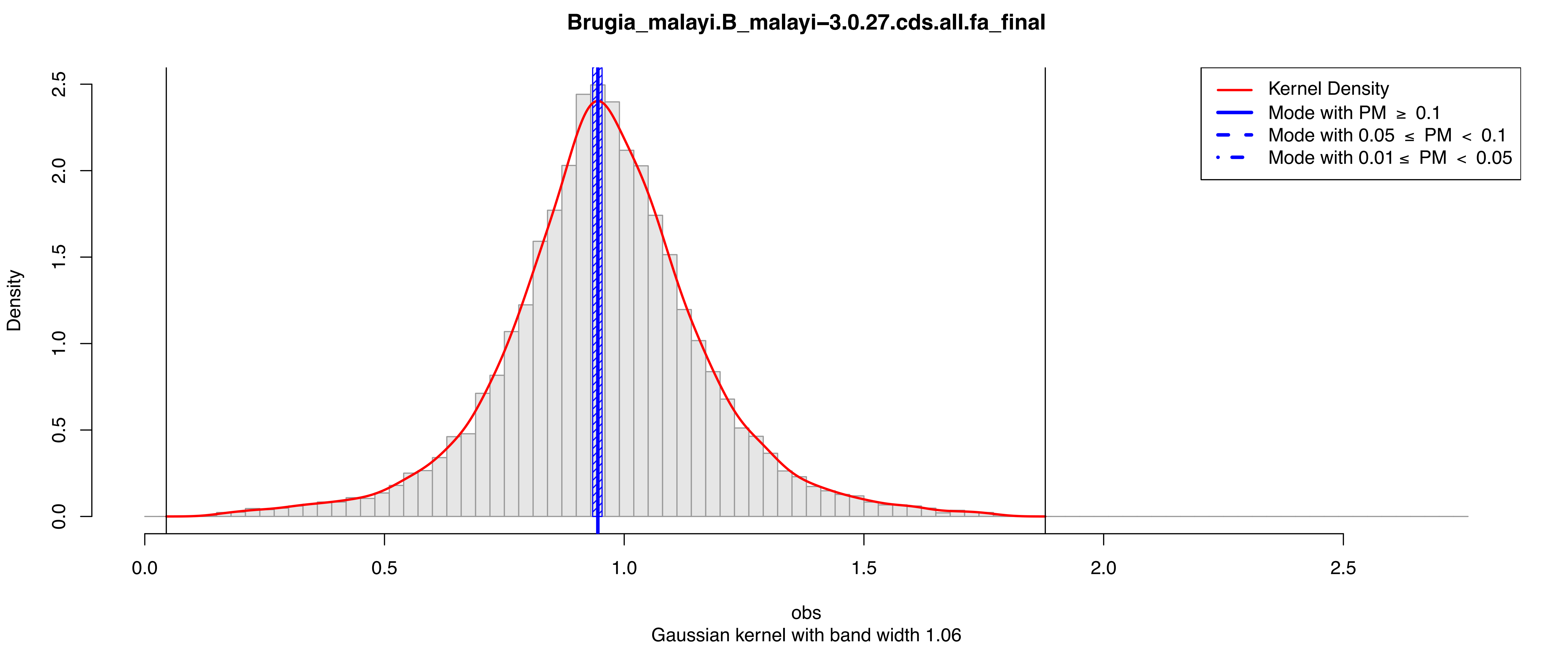

Brugia_malayi.B_malayi–3.0.27.cds.all.fa_final
Kernel Density
Mode with PM ≥ 0.1
Mode with 0.05 ≤ PM < 0.1
Mode with 0.01 ≤ PM < 0.05
Density
0.0
0.5
1.0
1.5
2.0
2.5
0.0
0.5
1.0
1.5
2.0
2.5
obs
Gaussian kernel with band width 1.06

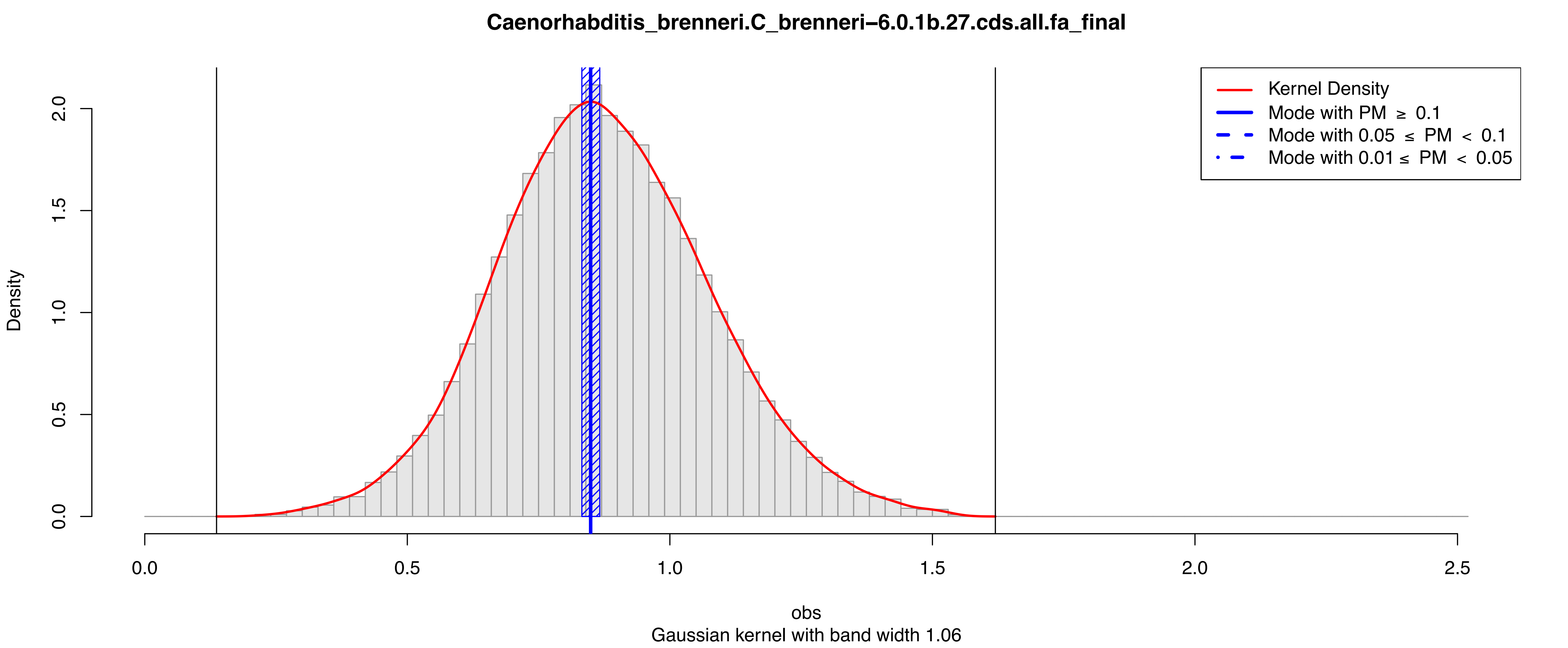
Caenorhabditis_brenneri.C_brenneri–6.0.1b.27.cds.all.fa_final
Kernel Density
Mode with PM ≥ 0.1
Mode with 0.05 ≤ PM < 0.1
Mode with 0.01 ≤ PM < 0.05
Density
0.0
0.5
1.0
1.5
2.0
0.0
0.5
1.0
1.5
2.0
2.5
obs
Gaussian kernel with band width 1.06

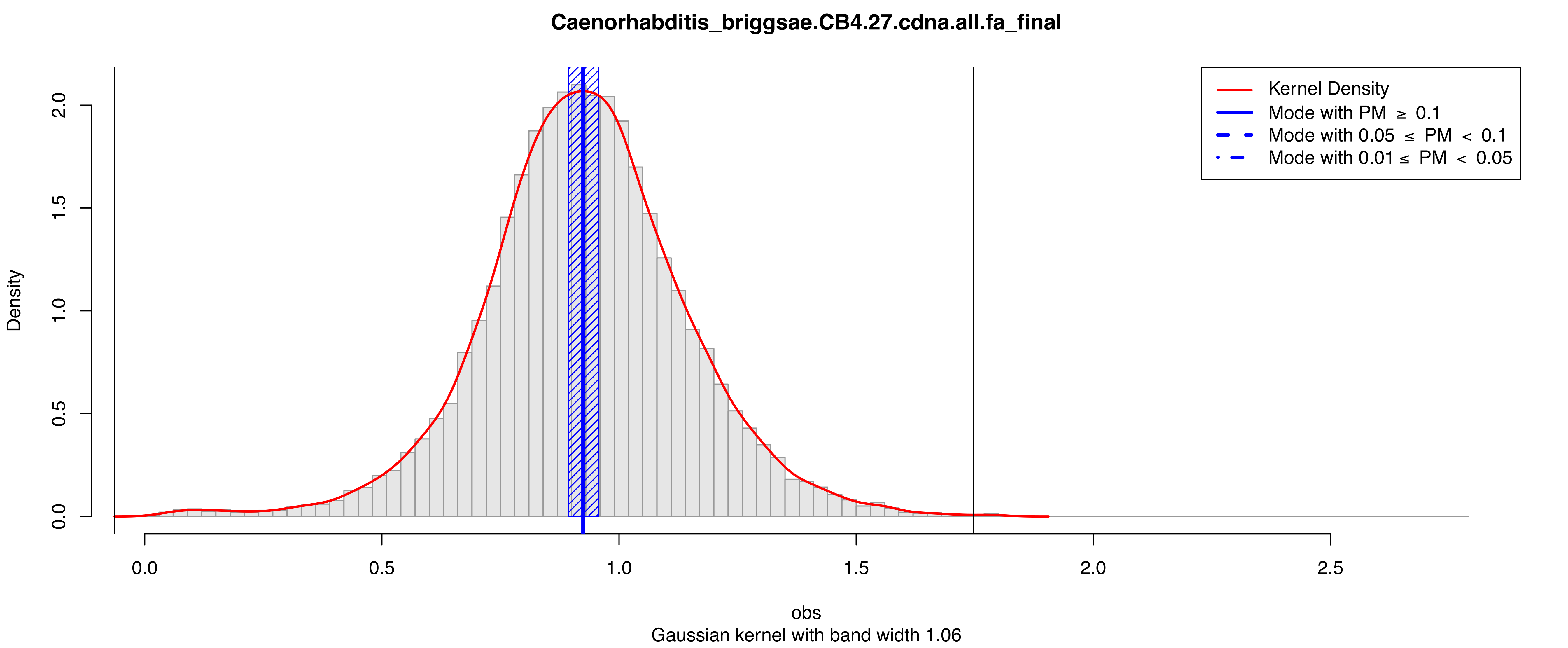

Caenorhabditis_briggsae.CB4.27.cdna.all.fa_final
Kernel Density
Mode with PM ≥ 0.1
Mode with 0.05 ≤ PM < 0.1
Mode with 0.01 ≤ PM < 0.05
Density
0.0
0.5
1.0
1.5
2.0
2.5
obs
Gaussian kernel with band width 1.06

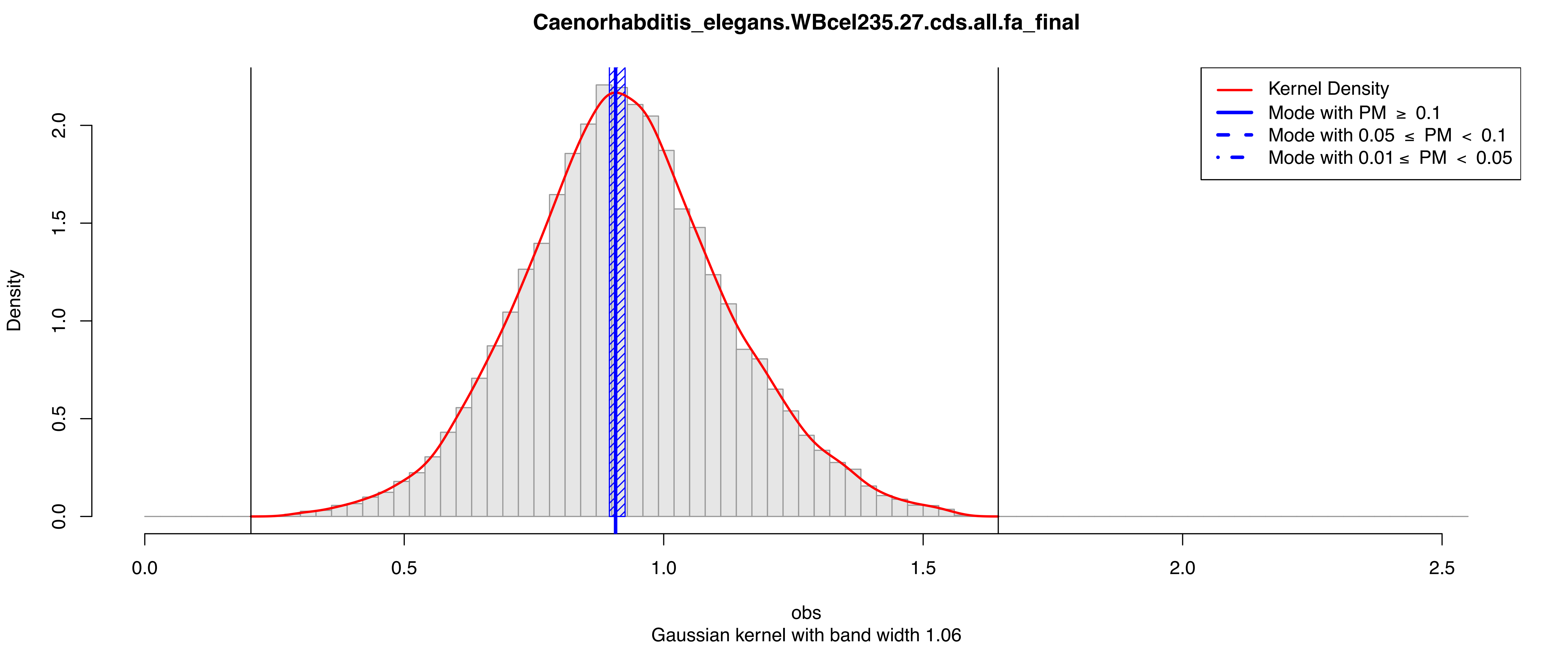
Caenorhabditis_elegans.WBcel235.27.cds.all.fa_final
Kernel Density
Mode with PM ≥ 0.1
Mode with 0.05 ≤ PM < 0.1
Mode with 0.01 ≤ PM < 0.05
Density
0.0
0.5
1.0
1.5
2.0
0.0
0.5
1.0
1.5
2.0
2.5
obs
Gaussian kernel with band width 1.06

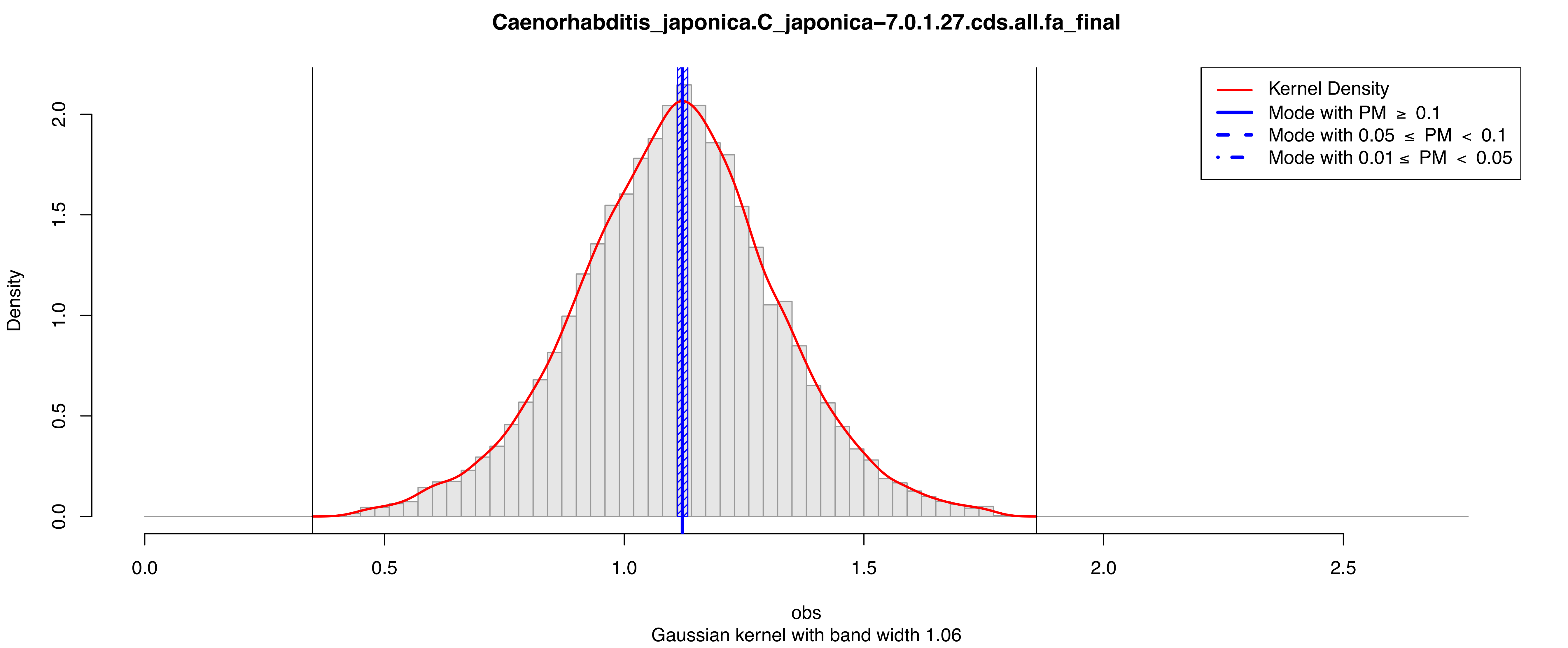
Caenorhabditis_japonica.C_japonica−7.0.1.27.cds.all.fa_final
Kernel Density
Mode with PM ≥ 0.1
Mode with 0.05 ≤ PM < 0.1
Mode with 0.01 ≤ PM < 0.05
Density
0.0
0.5
1.0
1.5
2.0
2.5
obs
Gaussian kernel with band width 1.06

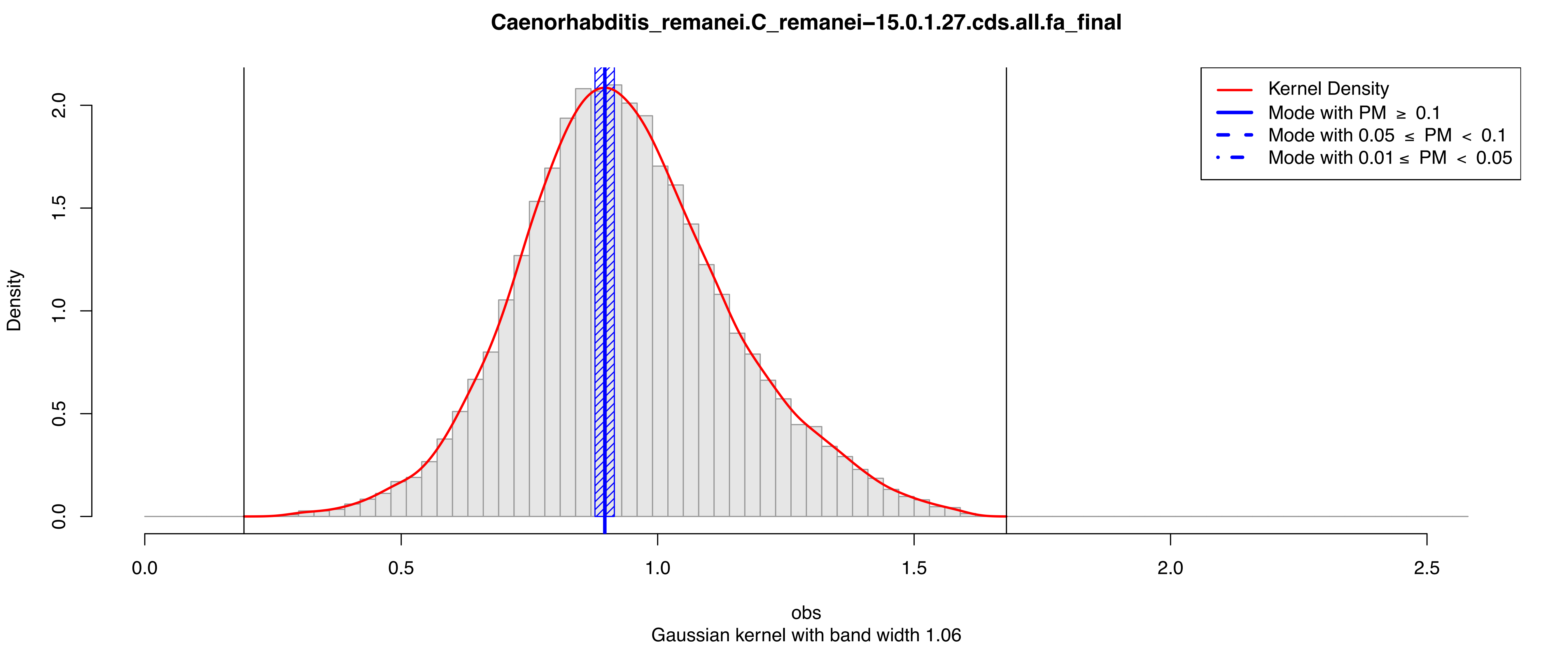
Caenorhabditis_remanei.C_remanei−15.0.1.27.cds.all.fa_final
Kernel Density
Mode with PM ≥ 0.1
Mode with 0.05 ≤ PM < 0.1
Mode with 0.01 ≤ PM < 0.05
Density
0.0
0.5
1.0
1.5
2.0
2.5
obs
Gaussian kernel with band width 1.06

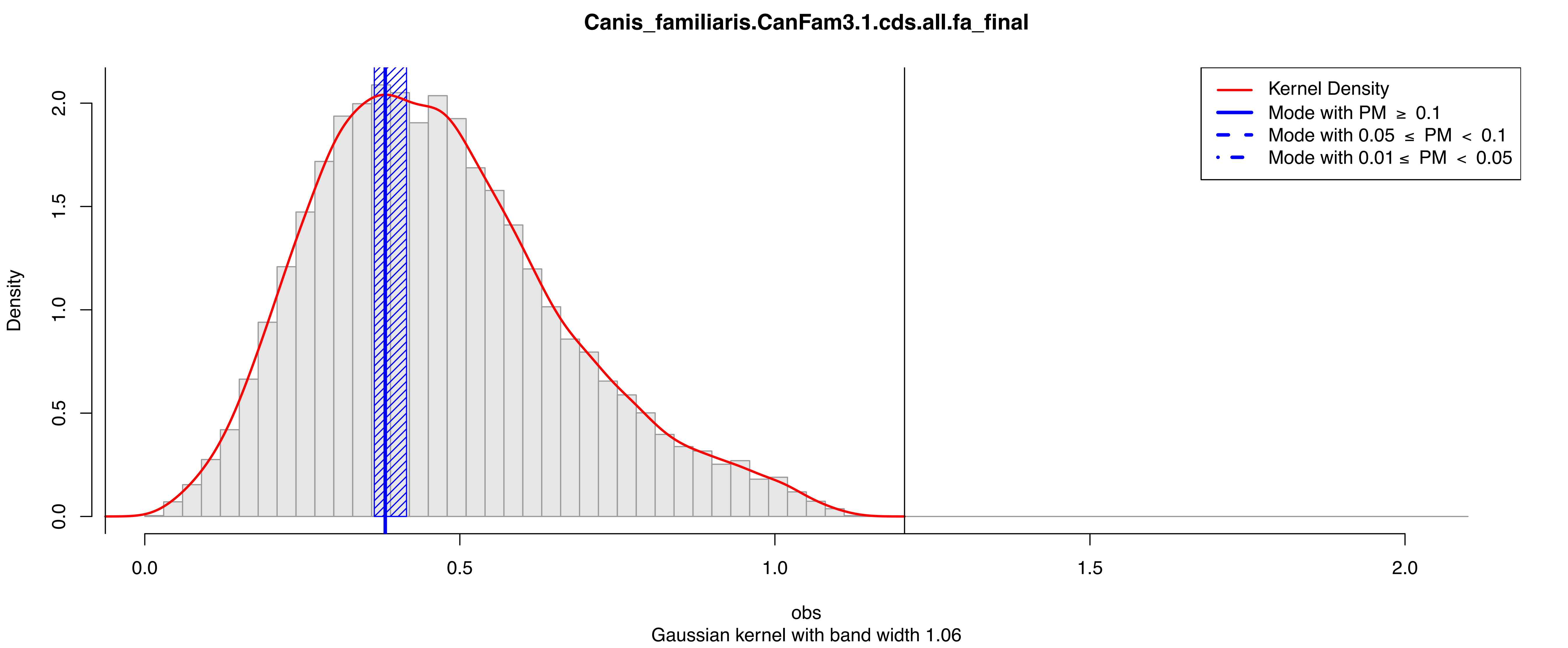

Canis_familiaris.CanFam3.1.cds.all.fa_final
Kernel Density
Mode with PM ≥ 0.1
Mode with 0.05 ≤ PM < 0.1
Mode with 0.01 ≤ PM < 0.05
Density
0.0
0.5
1.0
1.5
2.0
obs
Gaussian kernel with band width 1.06

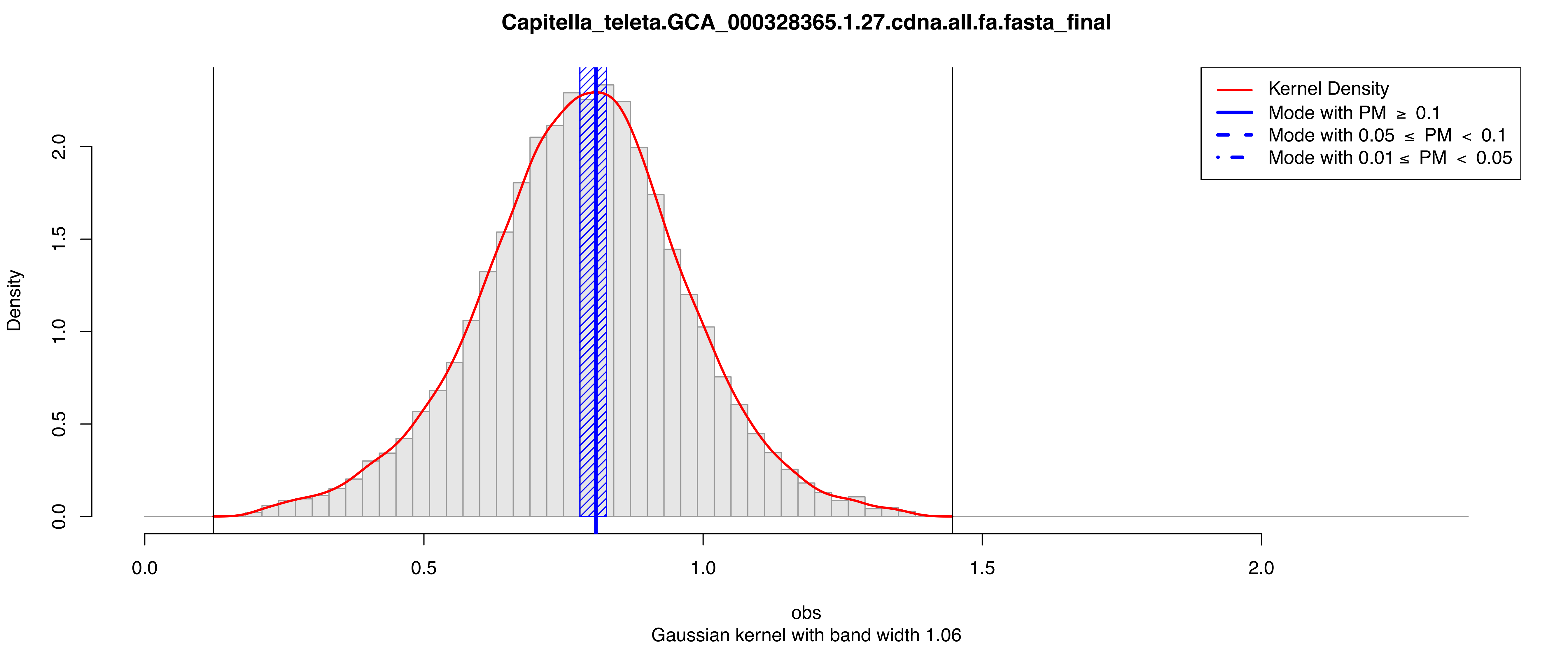
Capitella_teleta.GCA_000328365.1.27.cdna.all.fa.fasta_final
Kernel Density
Mode with PM ≥ 0.1
Mode with 0.05 ≤ PM < 0.1
Mode with 0.01 ≤ PM < 0.05
Density
0.0
0.5
1.0
1.5
2.0
0.0
0.5
1.0
1.5
2.0
obs
Gaussian kernel with band width 1.06

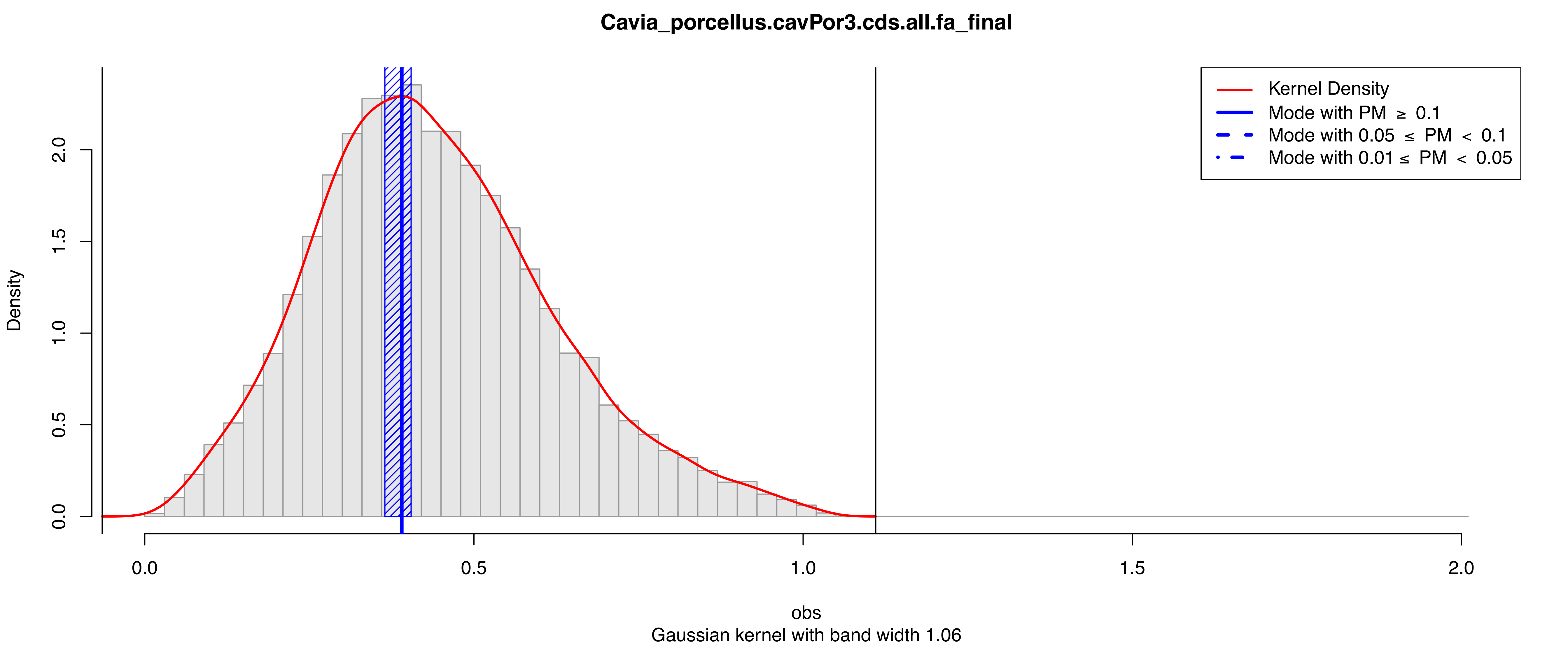
Cavia_porcellus.cavPor3.cds.all.fa_final
Kernel Density
Mode with PM ≥ 0.1
Mode with 0.05 ≤ PM < 0.1
Mode with 0.01 ≤ PM < 0.05
Density
0.0
0.5
1.0
1.5
2.0
obs
Gaussian kernel with band width 1.06

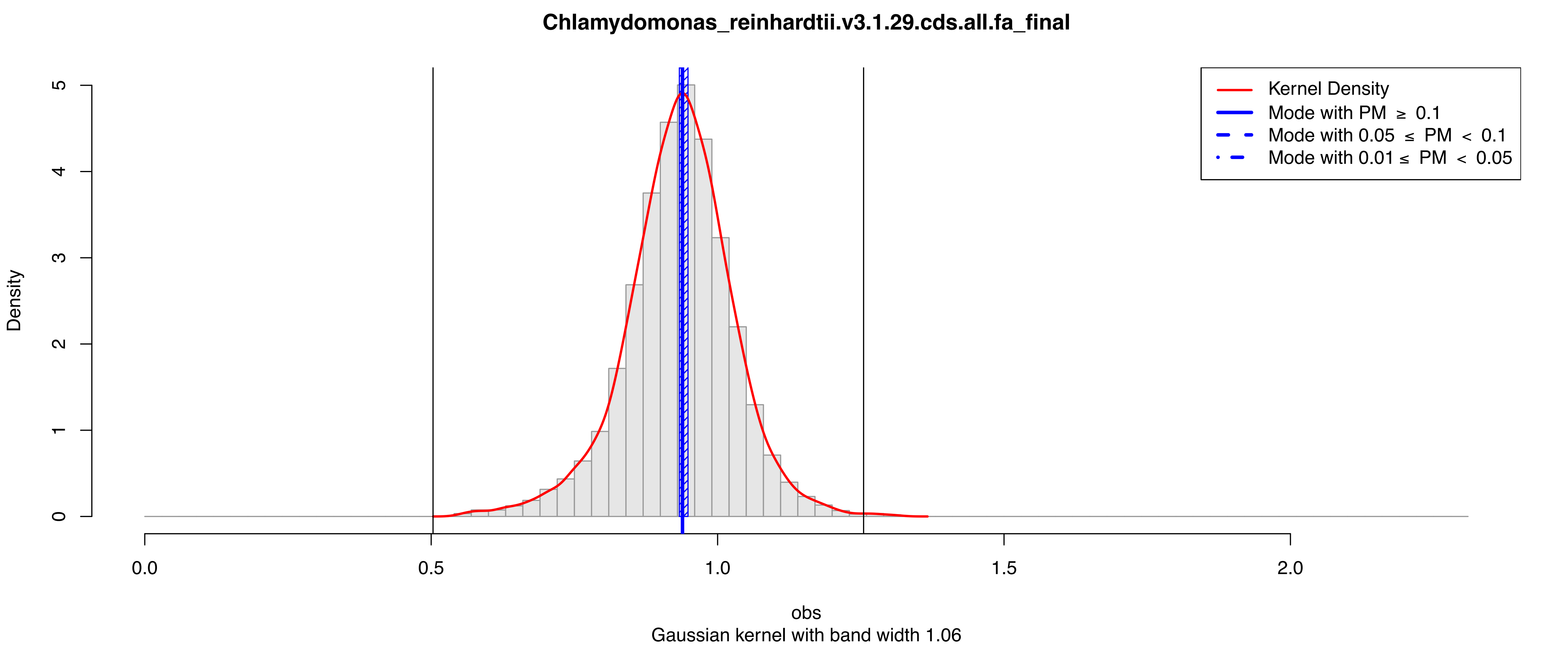

Chlamydomonas_reinhardtii.v3.1.29.cds.all.fa_final
Kernel Density
Mode with PM ≥ 0.1
Mode with 0.05 ≤ PM < 0.1
Mode with 0.01 ≤ PM < 0.05
Density
0
1
2
3
4
5
0.0
0.5
1.0
1.5
2.0
obs
Gaussian kernel with band width 1.06

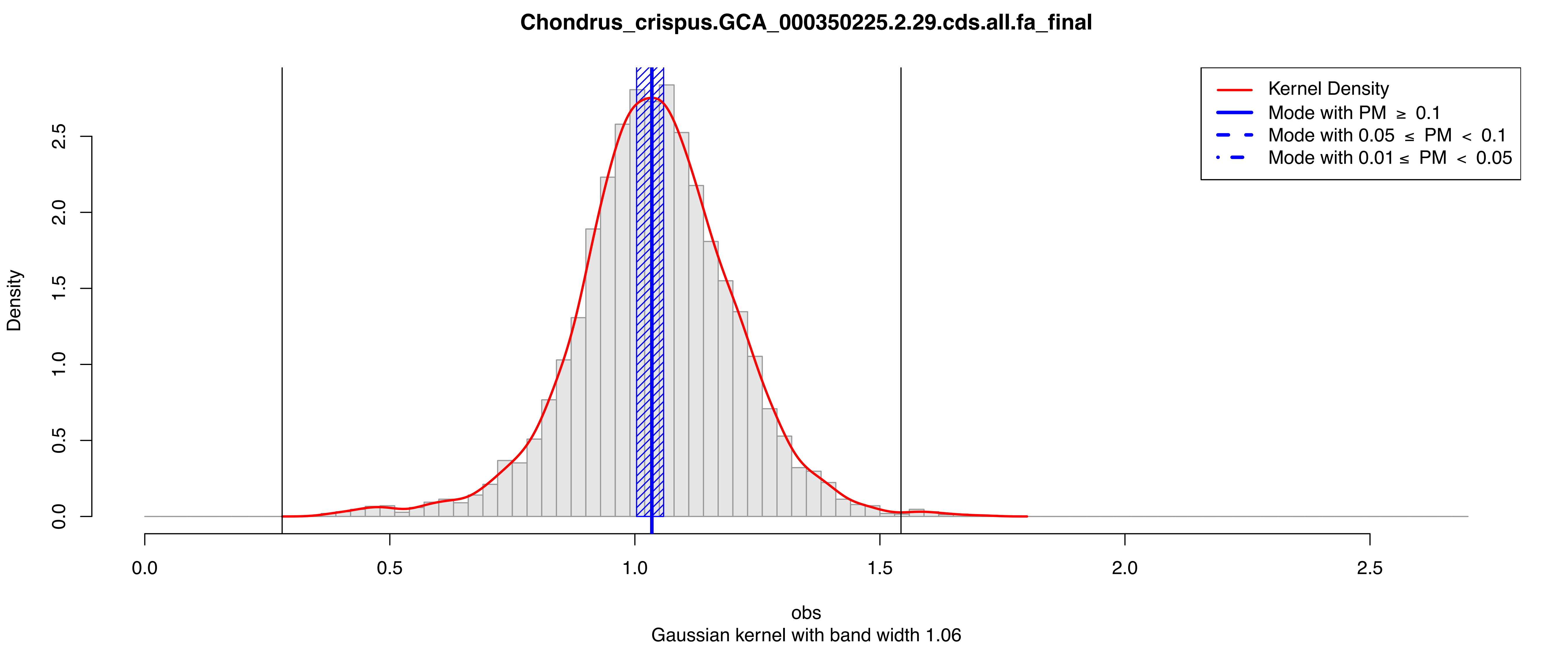

Chondrus_crispus.GCA_000350225.2.29.cds.all.fa_final
Kernel Density
Mode with PM ≥ 0.1
Mode with 0.05 ≤ PM < 0.1
Mode with 0.01 ≤ PM < 0.05
Density
0.0
0.5
1.0
1.5
2.0
2.5
obs
Gaussian kernel with band width 1.06

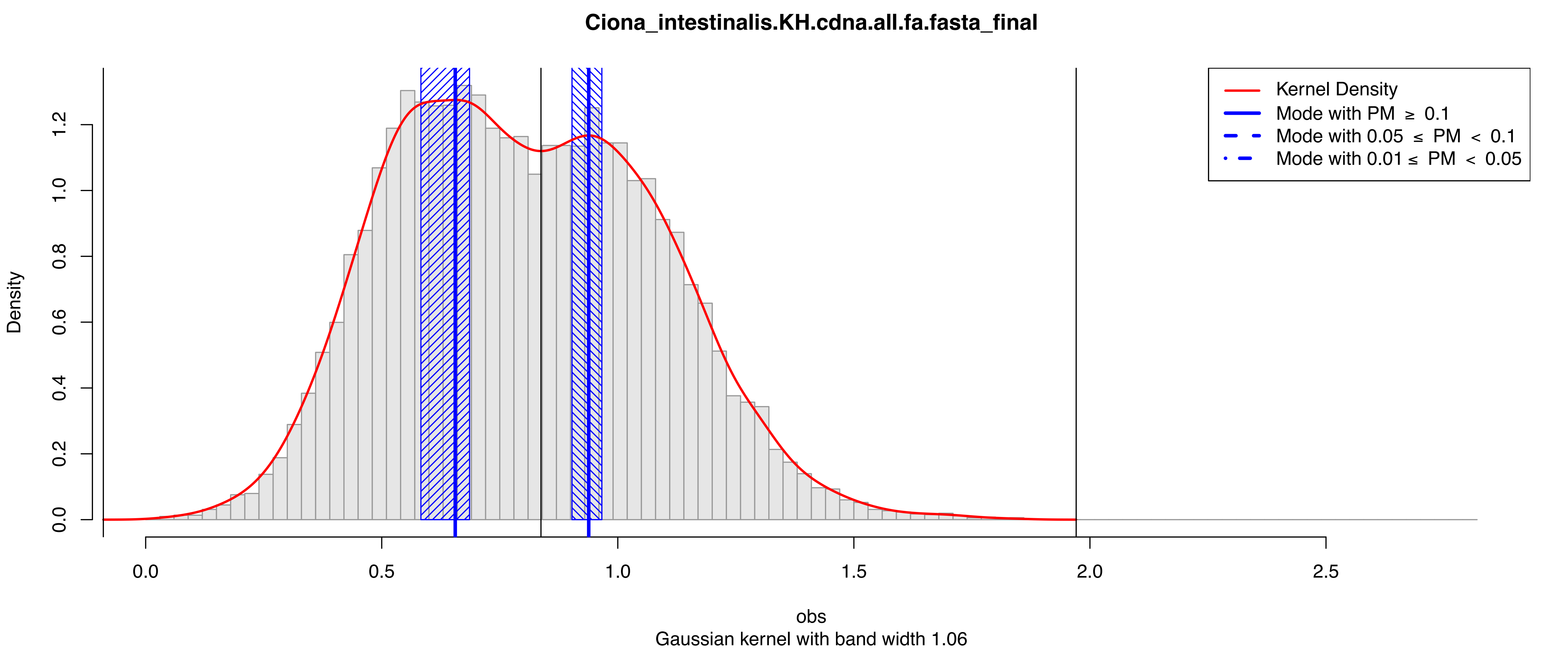

Ciona_intestinalis.KH.cdna.all.fa.fasta_final
Kernel Density
Mode with PM ≥ 0.1
Mode with 0.05 ≤ PM < 0.1
Mode with 0.01 ≤ PM < 0.05
Density
0.0
0.2
0.4
0.6
0.8
1.0
1.2
0.0
0.5
1.0
1.5
2.0
2.5
obs
Gaussian kernel with band width 1.06

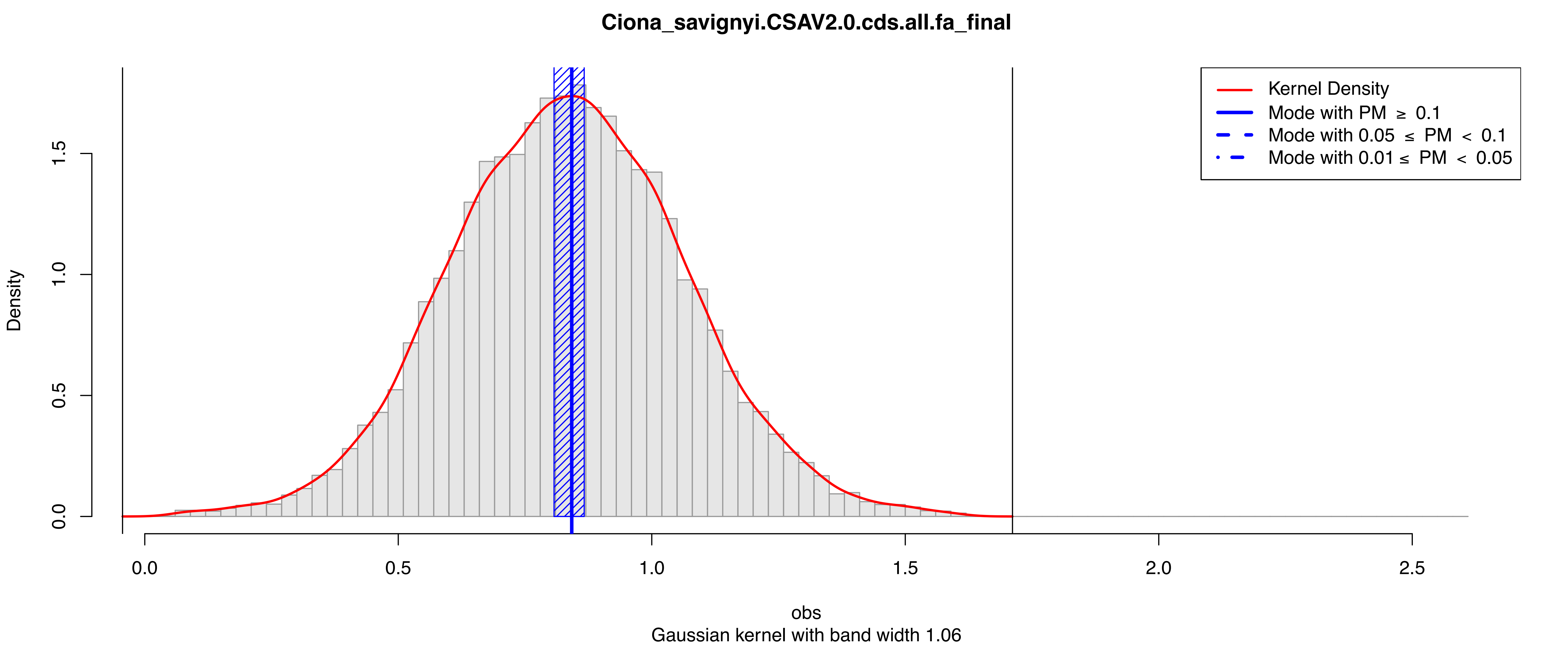
Ciona_savignyi.CSAV2.0.cds.all.fa_final
Kernel Density
Mode with PM ≥ 0.1
Mode with 0.05 ≤ PM < 0.1
Mode with 0.01 ≤ PM < 0.05
Density
0.0
0.5
1.0
1.5
0.0
0.5
1.0
1.5
2.0
2.5
obs
Gaussian kernel with band width 1.06

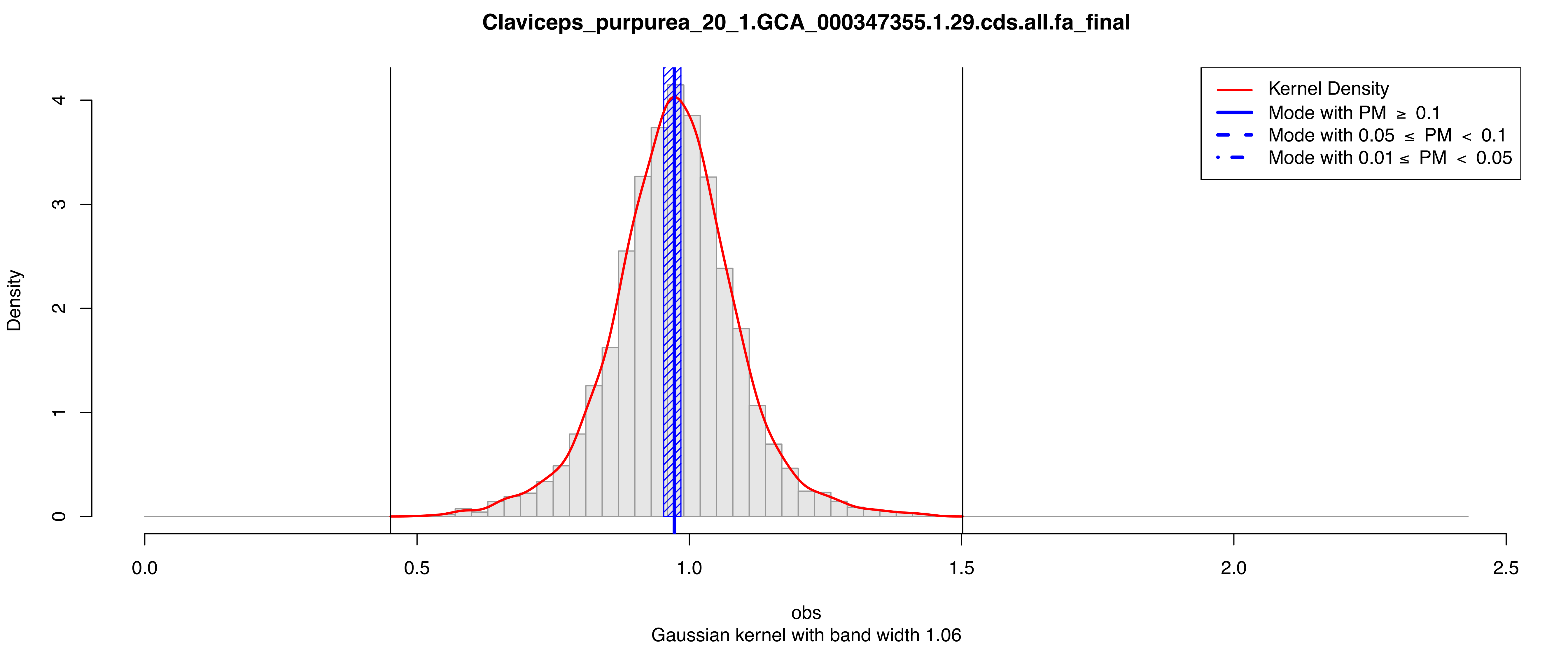

Claviceps_purpurea_20_1.GCA_000347355.1.29.cds.all.fa_final
Kernel Density
Mode with PM ≥ 0.1
Mode with 0.05 ≤ PM < 0.1
Mode with 0.01 ≤ PM < 0.05
Density
0
1
2
3
4
0.0
0.5
1.0
1.5
2.0
2.5
obs
Gaussian kernel with band width 1.06

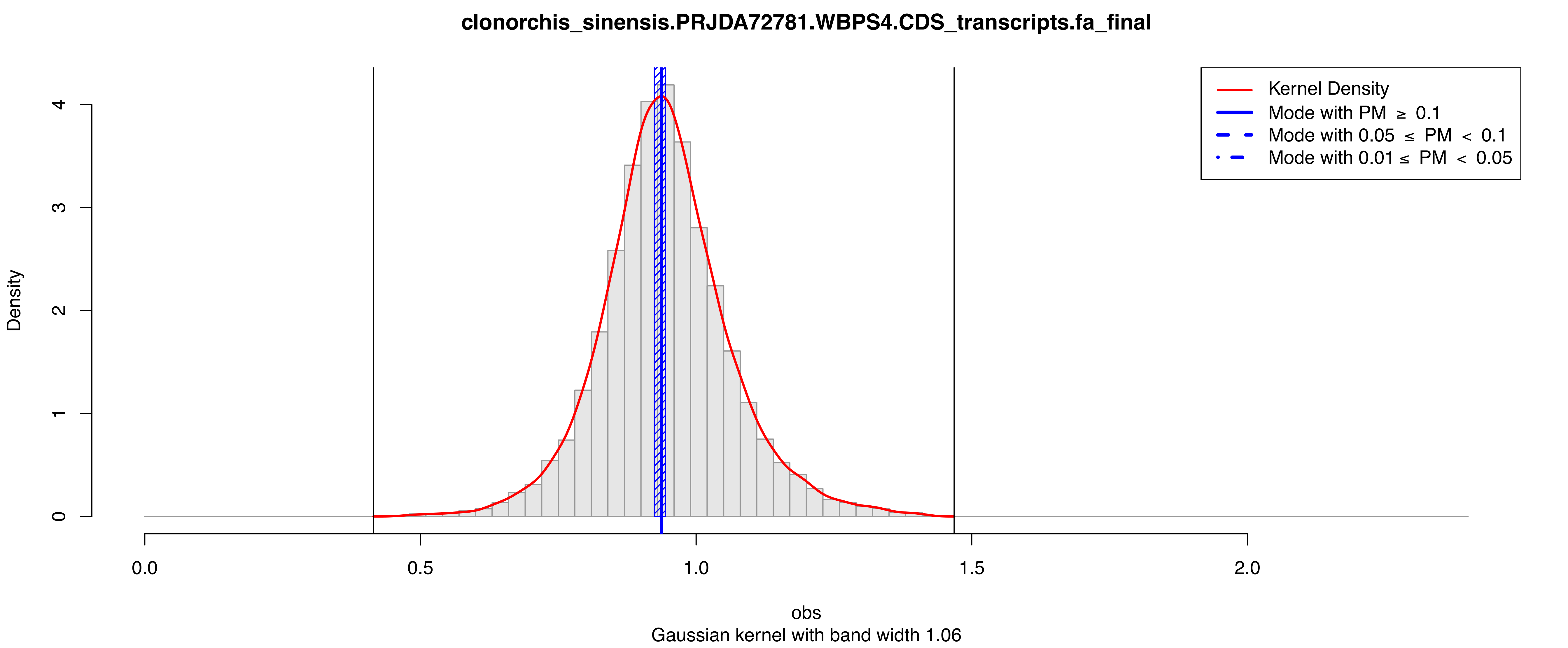

clonorchis_sinensis.PRJDA72781.WBPS4.CDS_transcripts.fa_final
Kernel Density
Mode with PM ≥ 0.1
Mode with 0.05 ≤ PM < 0.1
Mode with 0.01 ≤ PM < 0.05
Density
0
1
2
3
4
0.0
0.5
1.0
1.5
2.0
obs
Gaussian kernel with band width 1.06

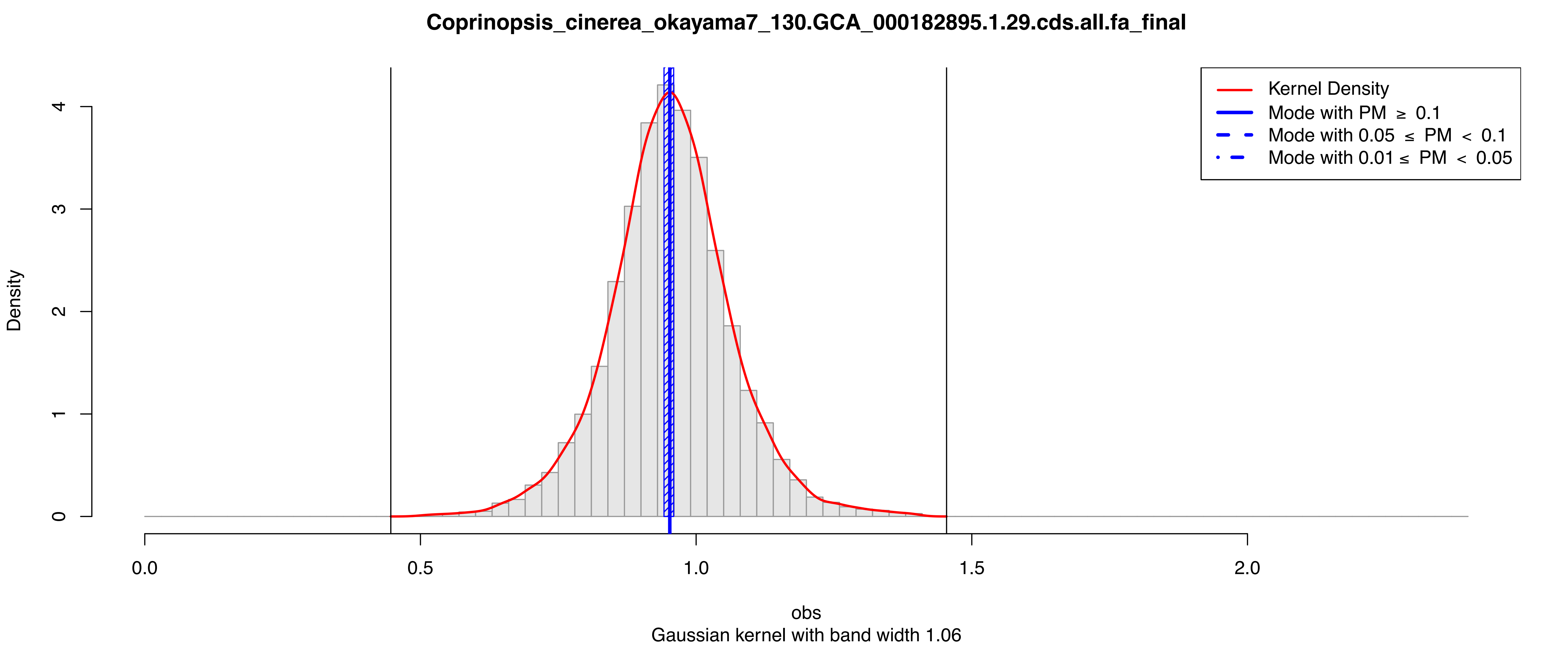
Coprinopsis_cinerea_okayama7_130.GCA_000182895.1.29.cds.all.fa_final
Kernel Density
Mode with PM ≥ 0.1
Mode with 0.05 ≤ PM < 0.1
Mode with 0.01 ≤ PM < 0.05
Density
0
1
2
3
4
0.0
0.5
1.0
1.5
2.0
obs
Gaussian kernel with band width 1.06

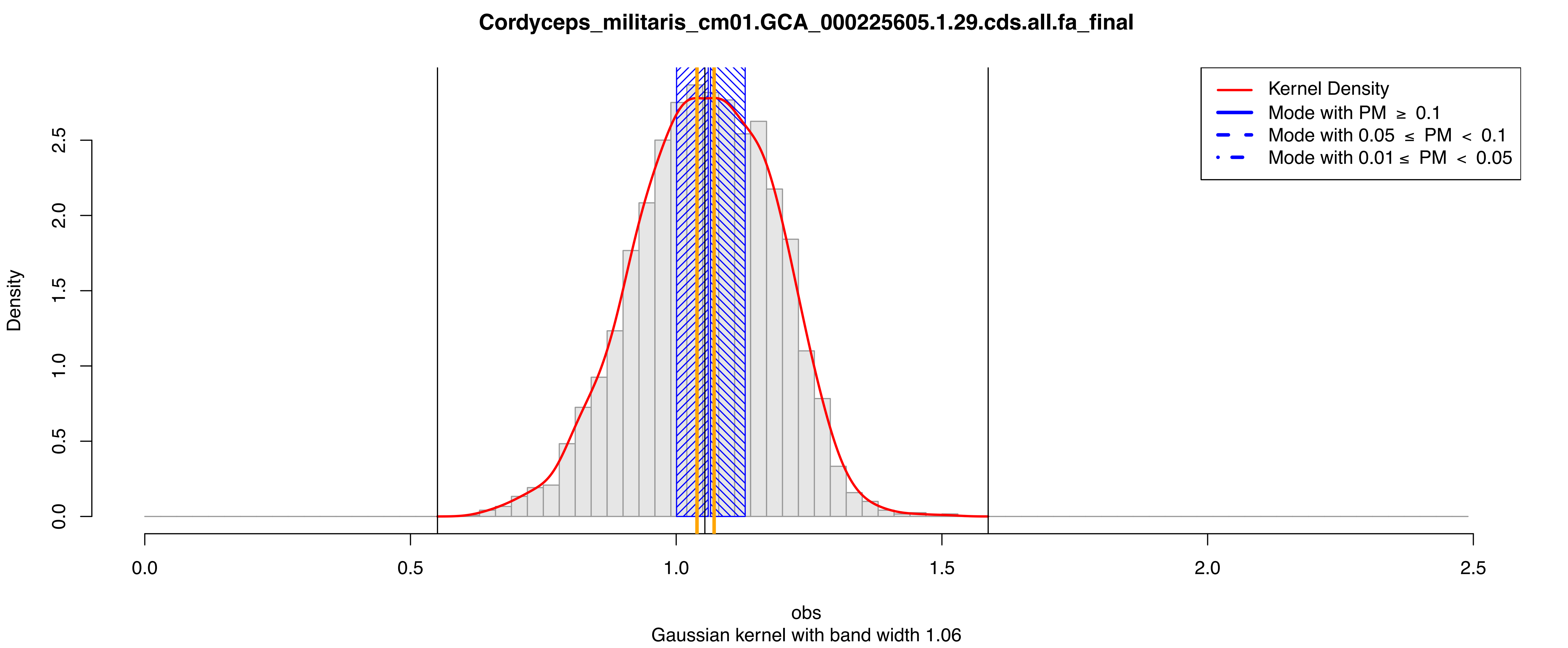
Cordyceps_militaris_cm01.GCA_000225605.1.29.cds.all.fa_final
Kernel Density
Mode with PM ≥ 0.1
Mode with 0.05 ≤ PM < 0.1
Mode with 0.01 ≤ PM < 0.05
Density
0.0
0.5
1.0
1.5
2.0
2.5
obs
Gaussian kernel with band width 1.06

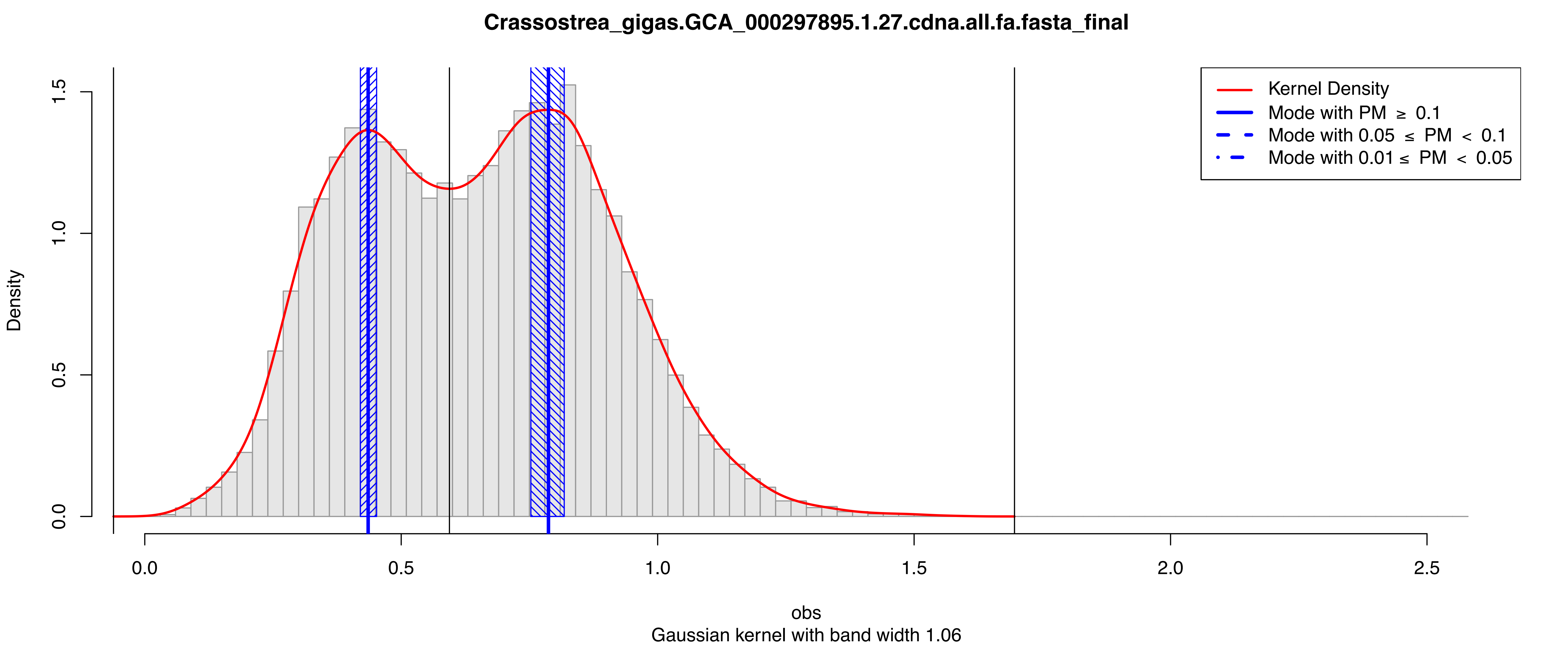

Crassostrea_gigas.GCA_000297895.1.27.cdna.all.fa.fasta_final
Kernel Density
Mode with PM ≥ 0.1
Mode with 0.05 ≤ PM < 0.1
Mode with 0.01 ≤ PM < 0.05
Density
0.0
0.5
1.0
1.5
0.0
0.5
1.0
1.5
2.0
2.5
obs
Gaussian kernel with band width 1.06

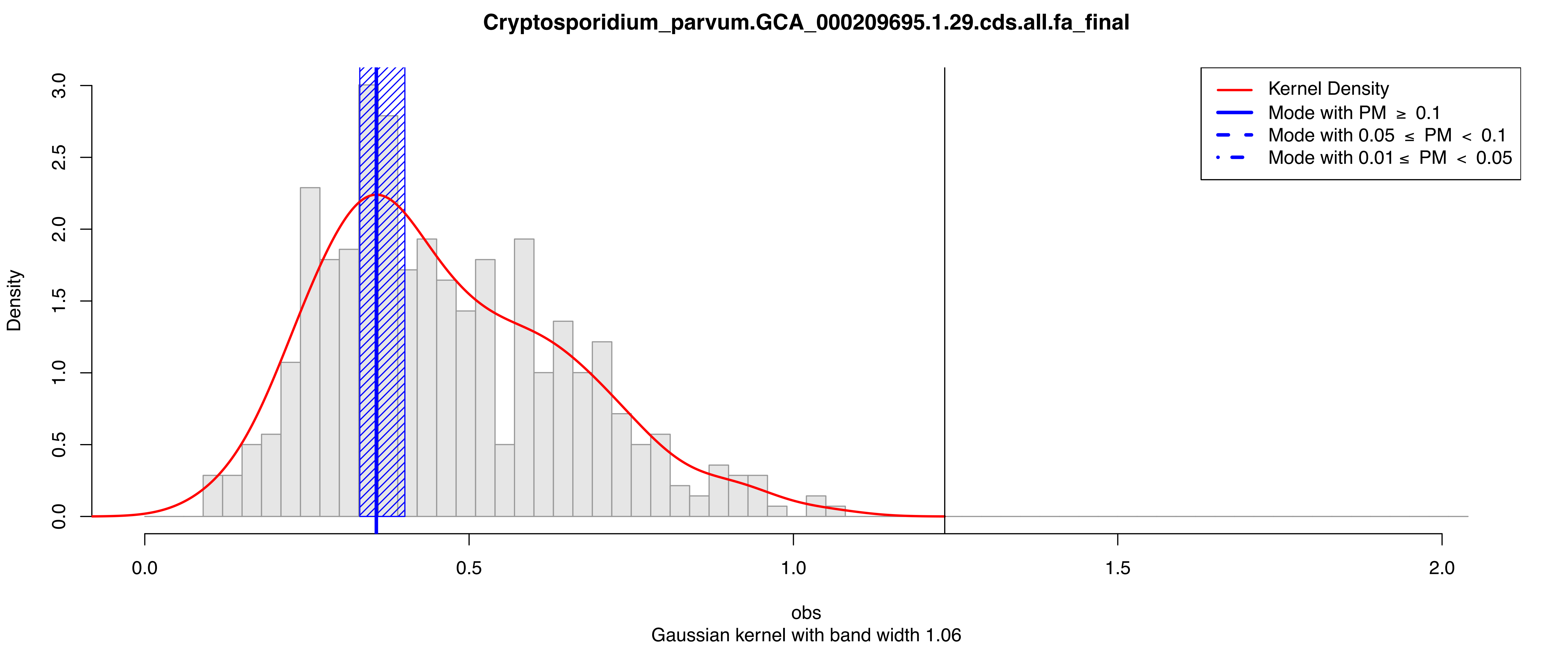

Cryptosporidium_parvum.GCA_000209695.1.29.cds.all.fa_final
Kernel Density
Mode with PM ≥ 0.1
Mode with 0.05 ≤ PM < 0.1
Mode with 0.01 ≤ PM < 0.05
Density
0.0
0.5
1.0
1.5
2.0
2.5
3.0
obs
Gaussian kernel with band width 1.06

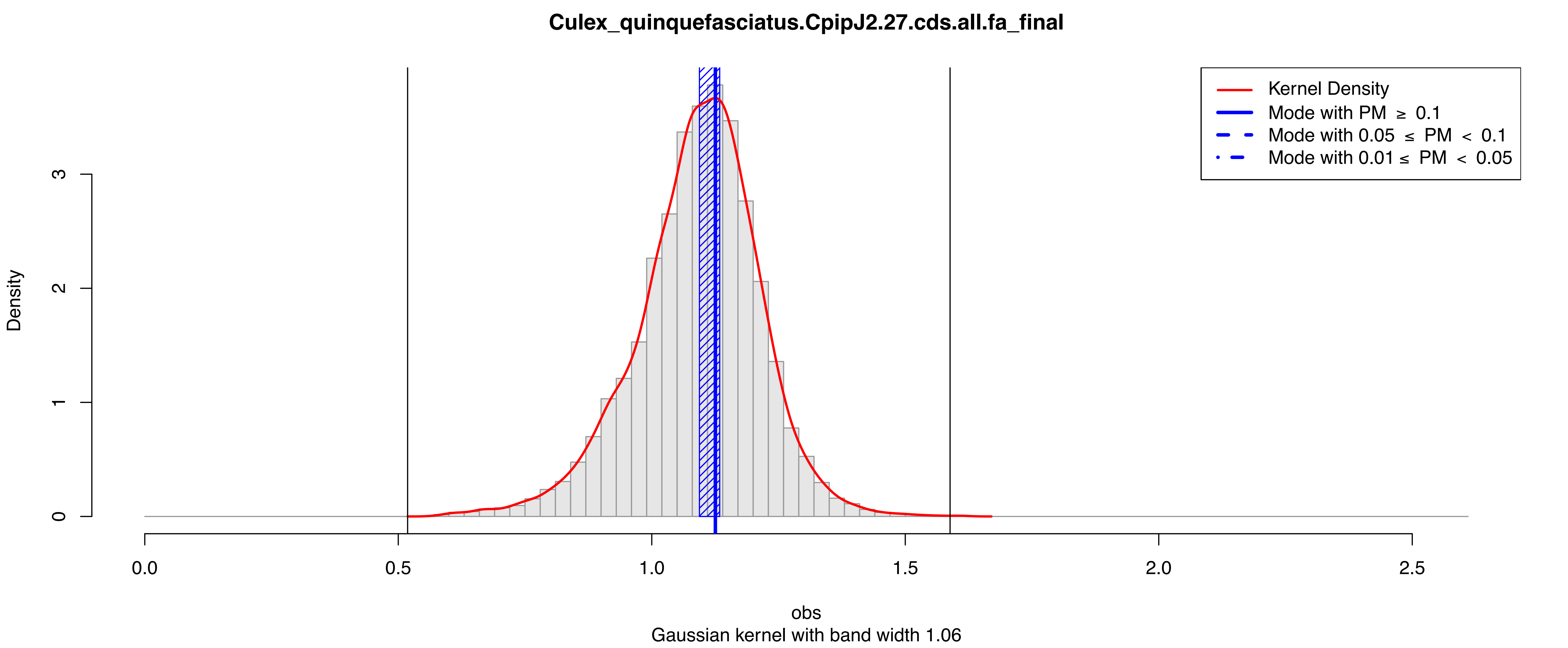
Culex_quinquefasciatus.CpipJ2.27.cds.all.fa_final
Kernel Density
Mode with PM ≥ 0.1
Mode with 0.05 ≤ PM < 0.1
Mode with 0.01 ≤ PM < 0.05
Density
0
1
2
3
0.0
0.5
1.0
1.5
2.0
2.5
obs
Gaussian kernel with band width 1.06

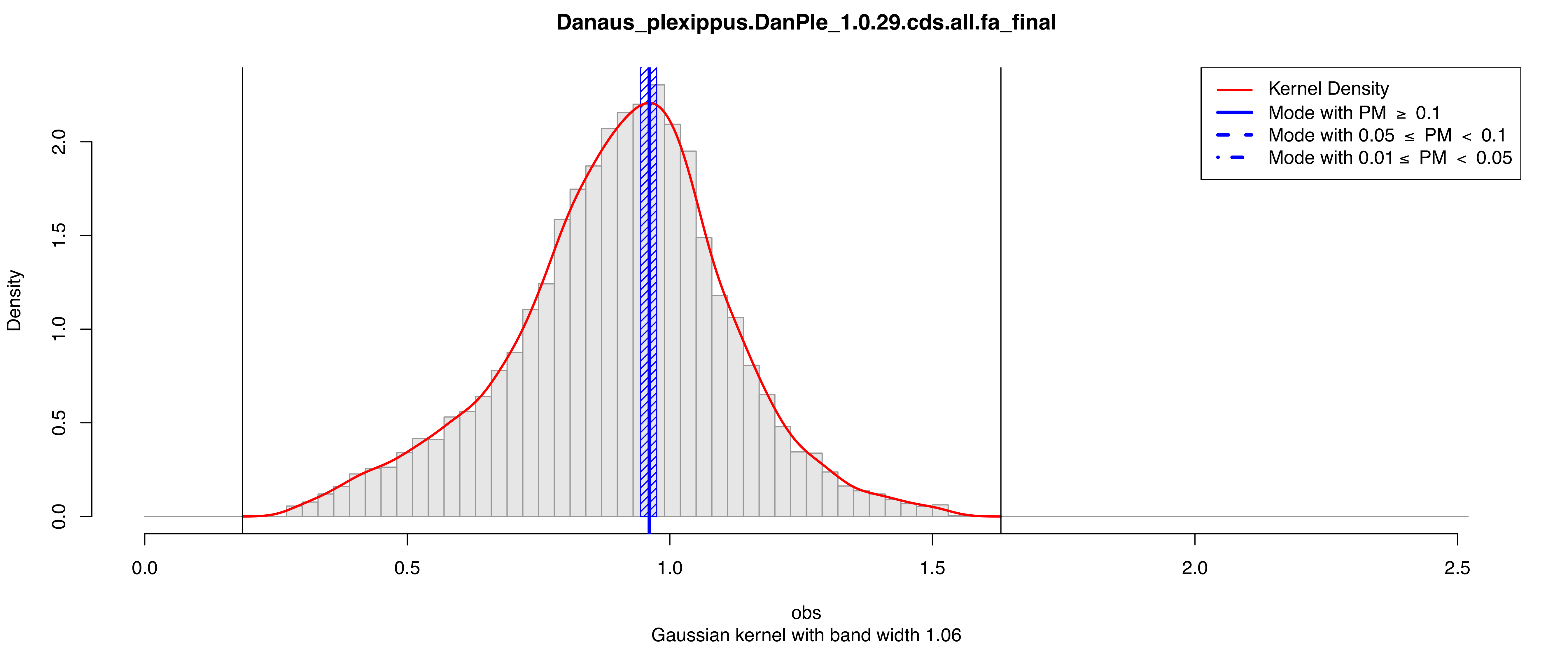

Danaus_plexippus.DanPle_1.0.29.cds.all.fa_final
Kernel Density
Mode with PM ≥ 0.1
Mode with 0.05 ≤ PM < 0.1
Mode with 0.01 ≤ PM < 0.05
Density
0.0
0.5
1.0
1.5
2.0
0.0
0.5
1.0
1.5
2.0
2.5
obs
Gaussian kernel with band width 1.06

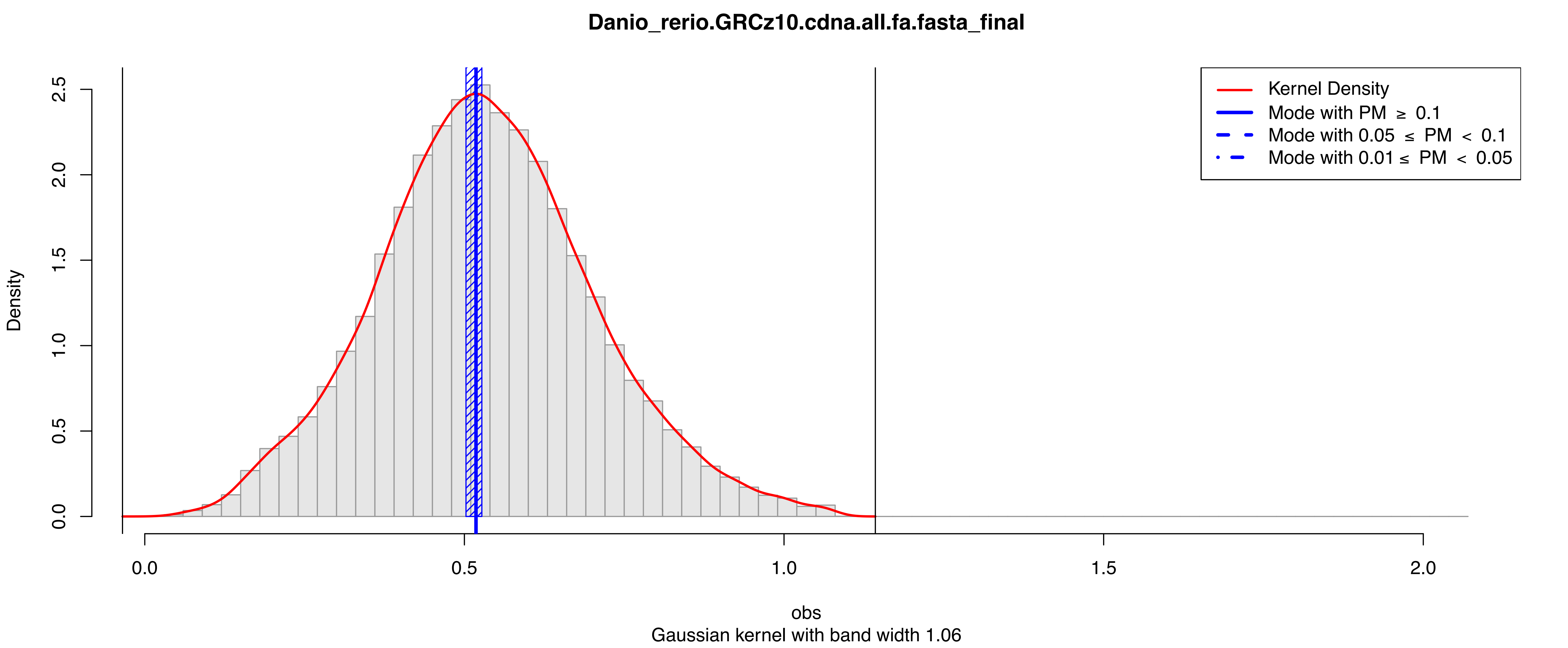

Danio_rerio.GRCz10.cdna.all.fa.fasta_final
Kernel Density
Mode with PM ≥ 0.1
Mode with 0.05 ≤ PM < 0.1
Mode with 0.01 ≤ PM < 0.05
Density
0.0
0.5
1.0
1.5
2.0
2.5
0.0
0.5
1.0
1.5
2.0
obs
Gaussian kernel with band width 1.06

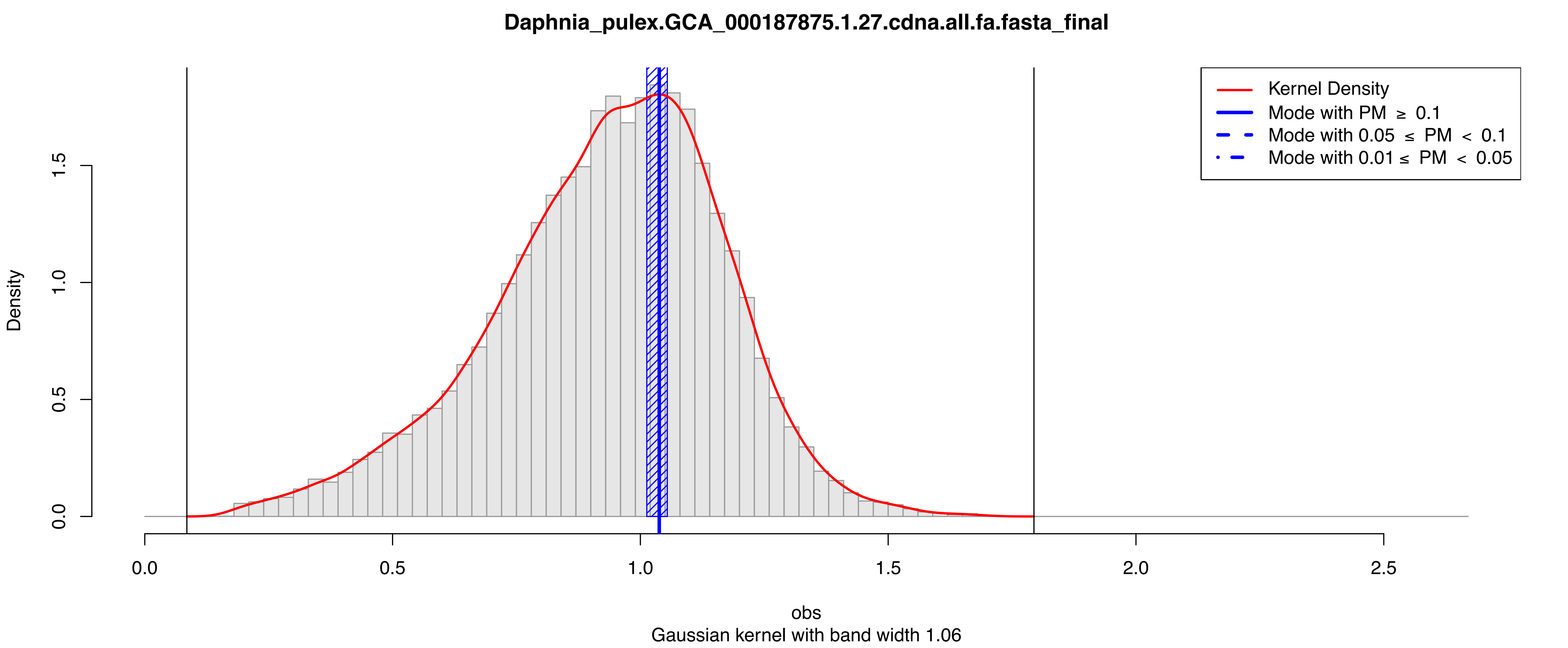

Daphnia_pulex.GCA_000187875.1.27.cdna.all.fa.fasta_final
Kernel Density
Mode with PM ≥ 0.1
Mode with 0.05 ≤ PM < 0.1
Mode with 0.01 ≤ PM < 0.05
Density
0.0
0.5
1.0
1.5
0.0
0.5
1.0
1.5
2.0
2.5
obs
Gaussian kernel with band width 1.06

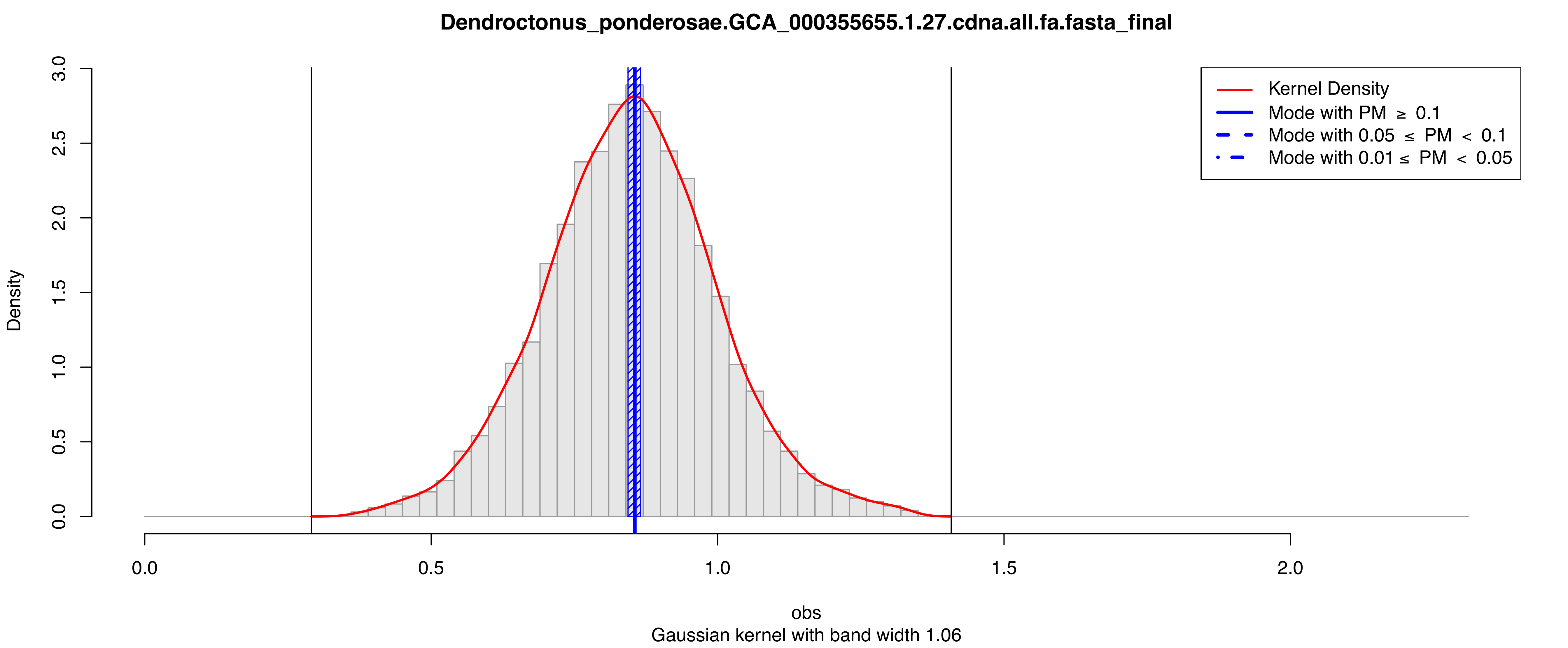

Dendroctonus_ponderosae.GCA_000355655.1.27.cdna.all.fa.fasta_final
Kernel Density
Mode with PM ≥ 0.1
Mode with 0.05 ≤ PM < 0.1
Mode with 0.01 ≤ PM < 0.05
Density
0.0
0.5
1.0
1.5
2.0
2.5
3.0
0.0
0.5
1.0
1.5
2.0
obs
Gaussian kernel with band width 1.06

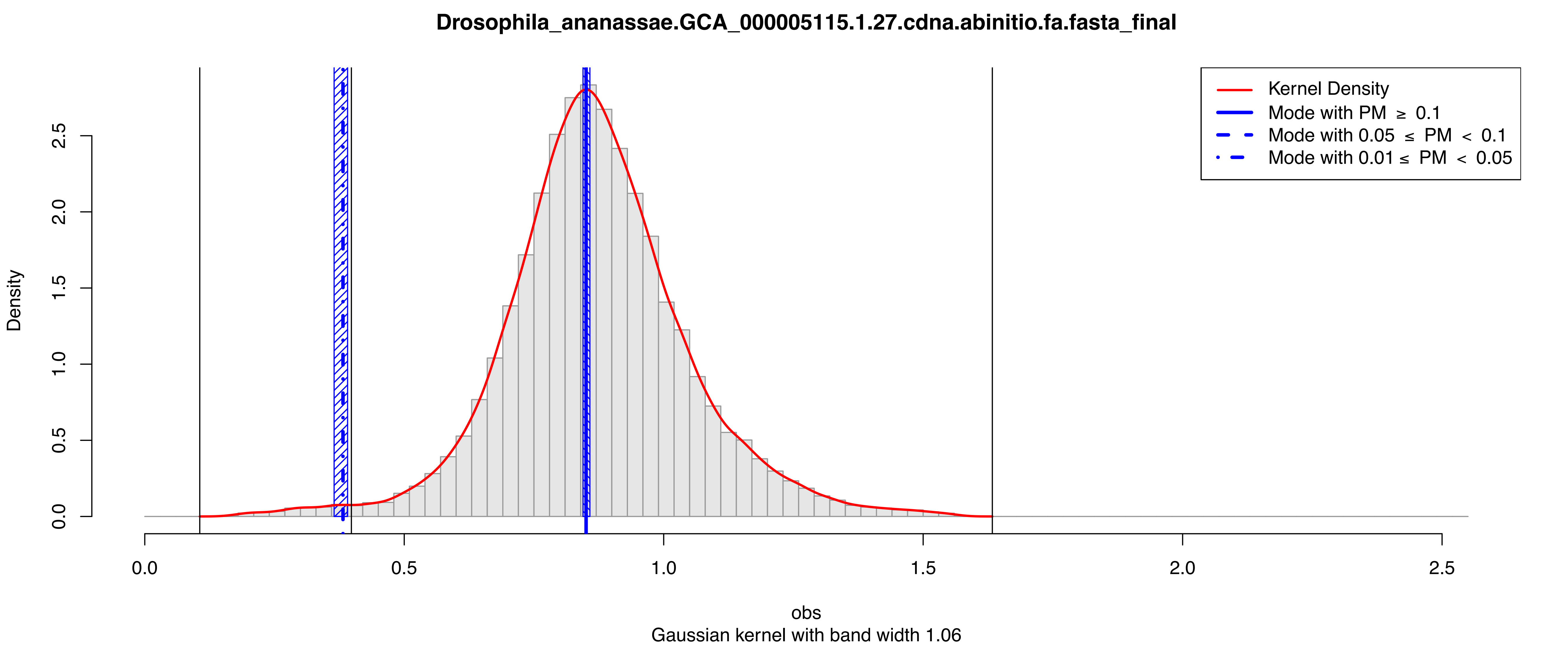
Drosophila_ananassae.GCA_000005115.1.27.cdna.abinitio.fa.fasta_final
Kernel Density
Mode with PM ≥ 0.1
Mode with 0.05 ≤ PM < 0.1
Mode with 0.01 ≤ PM < 0.05
Density
0.0
0.5
1.0
1.5
2.0
2.5
obs
Gaussian kernel with band width 1.06

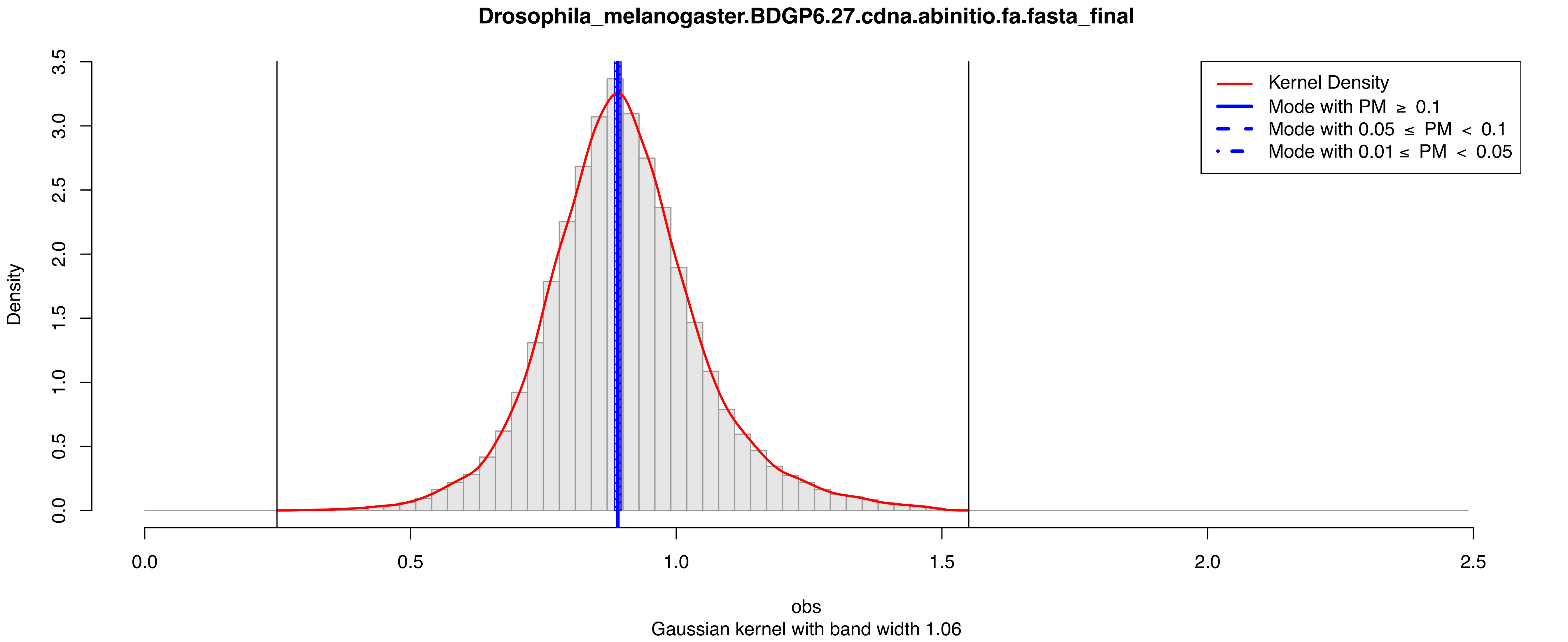

Drosophila_melanogaster.BDGP6.27.cdna.abinitio.fa.fasta_final
Density
obs
Gaussian kernel with band width 1.06
Kernel Density
Mode with PM ≥ 0.1
Mode with 0.05 ≤ PM < 0.1
Mode with 0.01 ≤ PM < 0.05
0.0
0.5
1.0
1.5
2.0
2.5
3.0
3.5

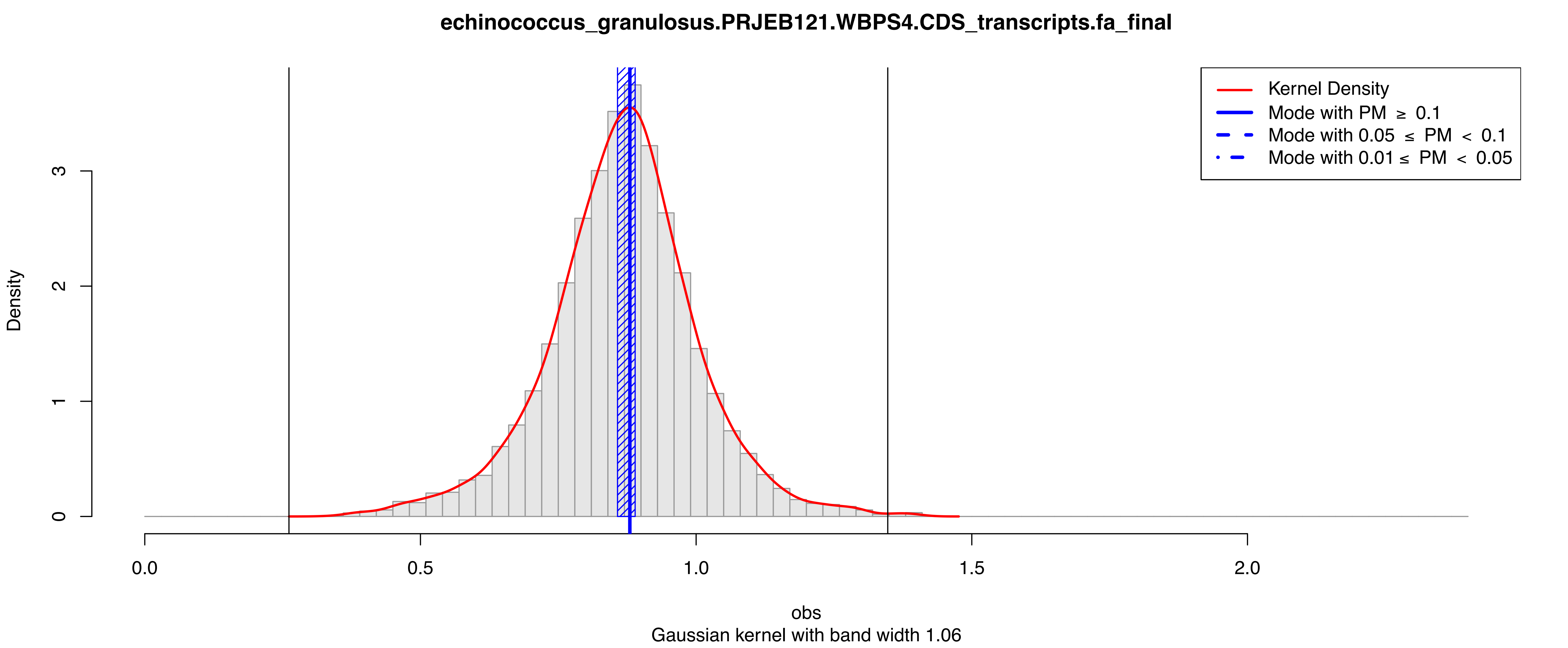

echinococcus_granulosus.PRJEB121.WBPS4.CDS_transcripts.fa_final
Kernel Density
Mode with PM ≥ 0.1
Mode with 0.05 ≤ PM < 0.1
Mode with 0.01 ≤ PM < 0.05
Density
0
1
2
3
0.0
0.5
1.0
1.5
2.0
obs
Gaussian kernel with band width 1.06

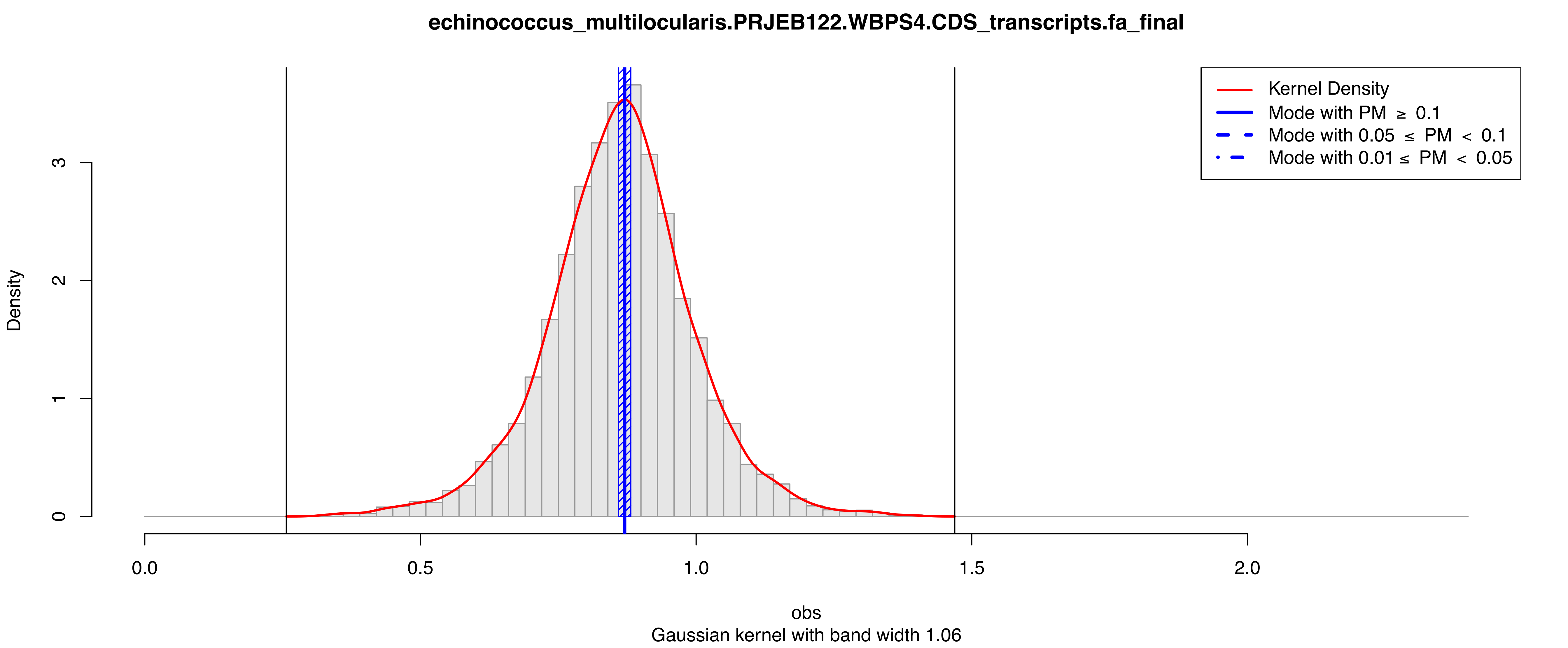

echinococcus_multilocularis.PRJEB122.WBPS4.CDS_transcripts.fa_final
Kernel Density
Mode with PM ≥ 0.1
Mode with 0.05 ≤ PM < 0.1
Mode with 0.01 ≤ PM < 0.05
Density
0
1
2
3
0.0
0.5
1.0
1.5
2.0
obs
Gaussian kernel with band width 1.06

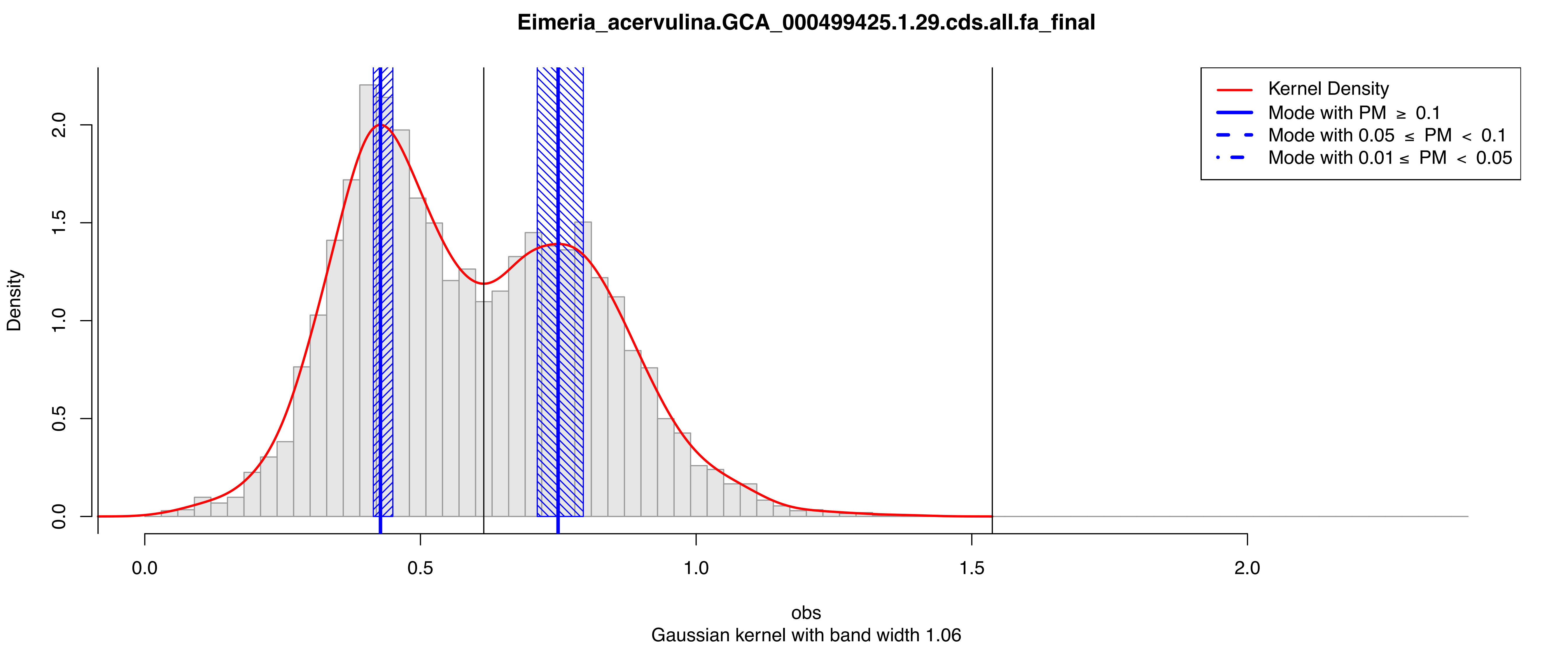

Eimeria_acervulina.GCA_000499425.1.29.cds.all.fa_final
Kernel Density
Mode with PM ≥ 0.1
Mode with 0.05 ≤ PM < 0.1
Mode with 0.01 ≤ PM < 0.05
Density
0.0
0.5
1.0
1.5
2.0
obs
Gaussian kernel with band width 1.06

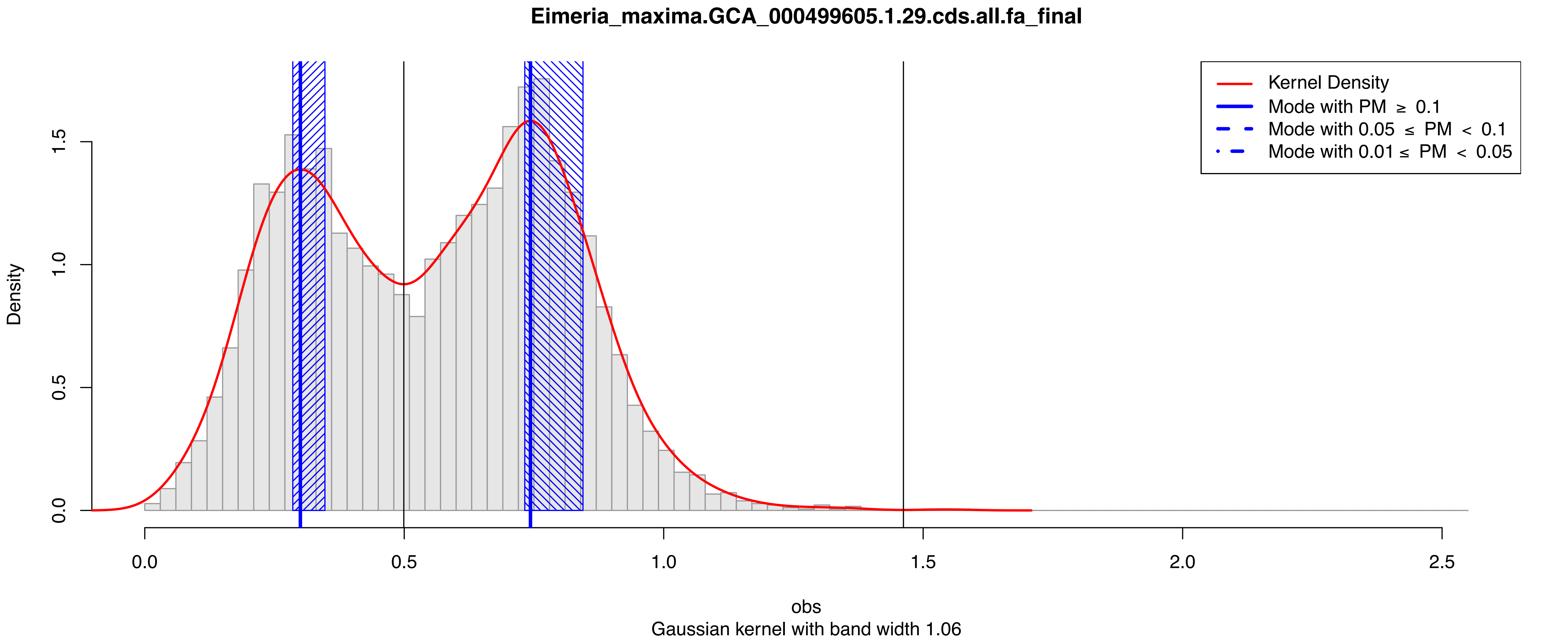

Eimeria_maxima.GCA_000499605.1.29.cds.all.fa_final
Kernel Density
Mode with PM ≥ 0.1
Mode with 0.05 ≤ PM < 0.1
Mode with 0.01 ≤ PM < 0.05
Density
0.0
0.5
1.0
1.5
0.0
0.5
1.0
1.5
2.0
2.5
obs
Gaussian kernel with band width 1.06

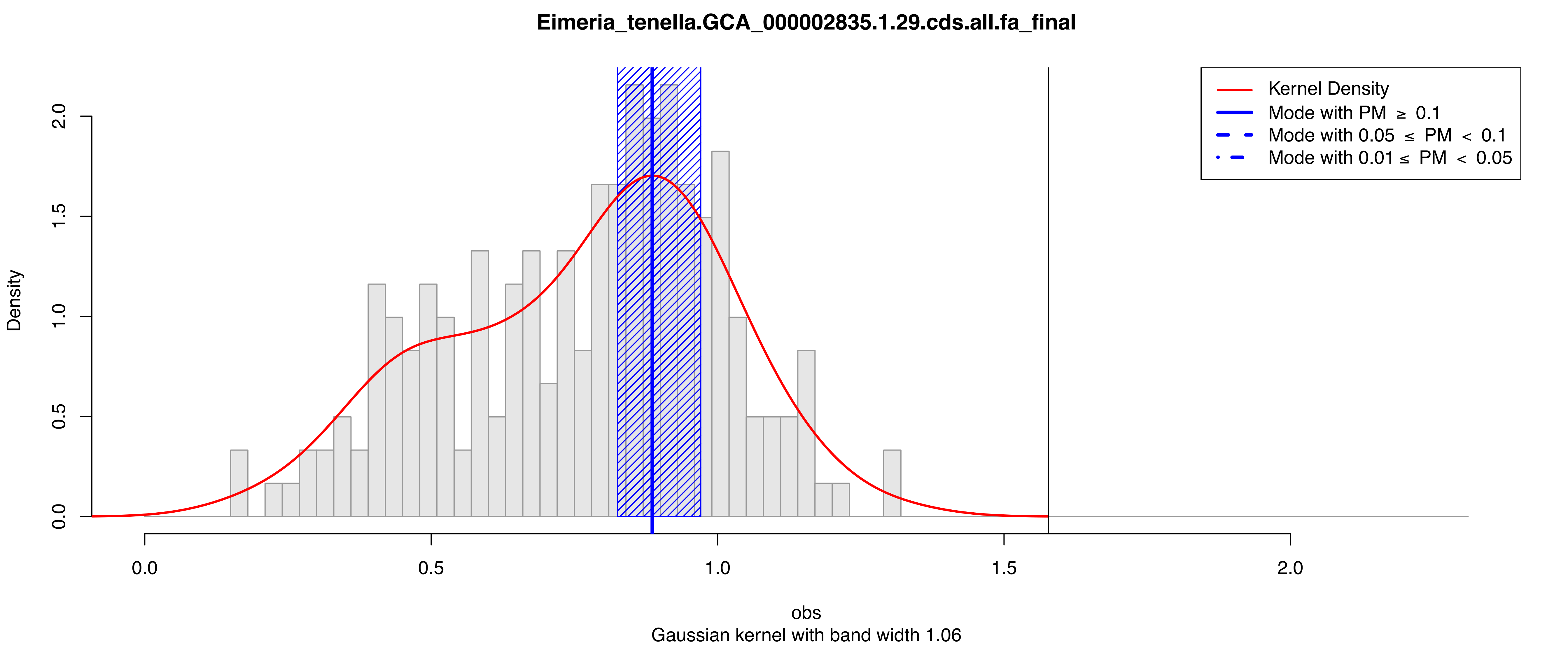
Eimeria_tenella.GCA_000002835.1.29.cds.all.fa_final
Kernel Density
Mode with PM ≥ 0.1
Mode with 0.05 ≤ PM < 0.1
Mode with 0.01 ≤ PM < 0.05
Density
0.0
0.5
1.0
1.5
2.0
obs
Gaussian kernel with band width 1.06

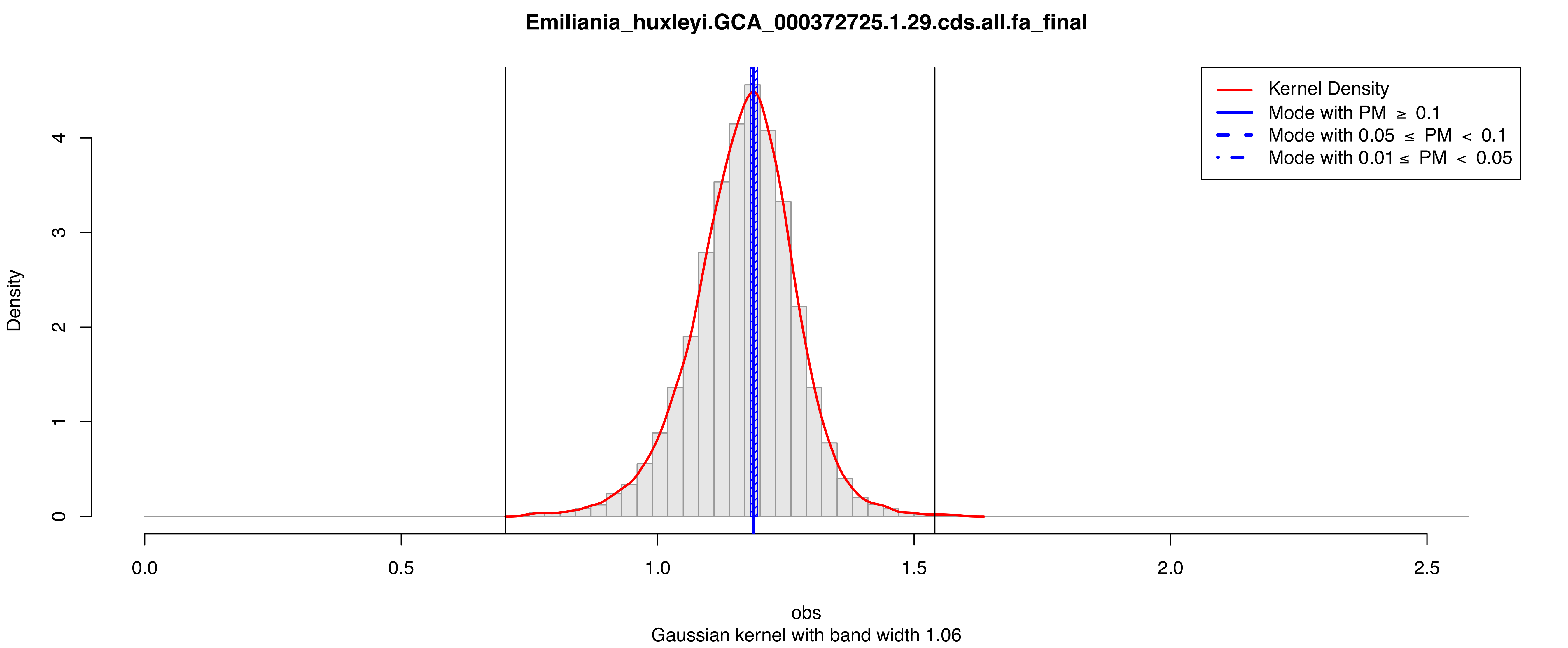

Emiliania_huxleyi.GCA_000372725.1.29.cds.all.fa_final
Kernel Density
Mode with PM ≥ 0.1
Mode with 0.05 ≤ PM < 0.1
Mode with 0.01 ≤ PM < 0.05
Density
0
1
2
3
4
0.0
0.5
1.0
1.5
2.0
2.5
obs
Gaussian kernel with band width 1.06

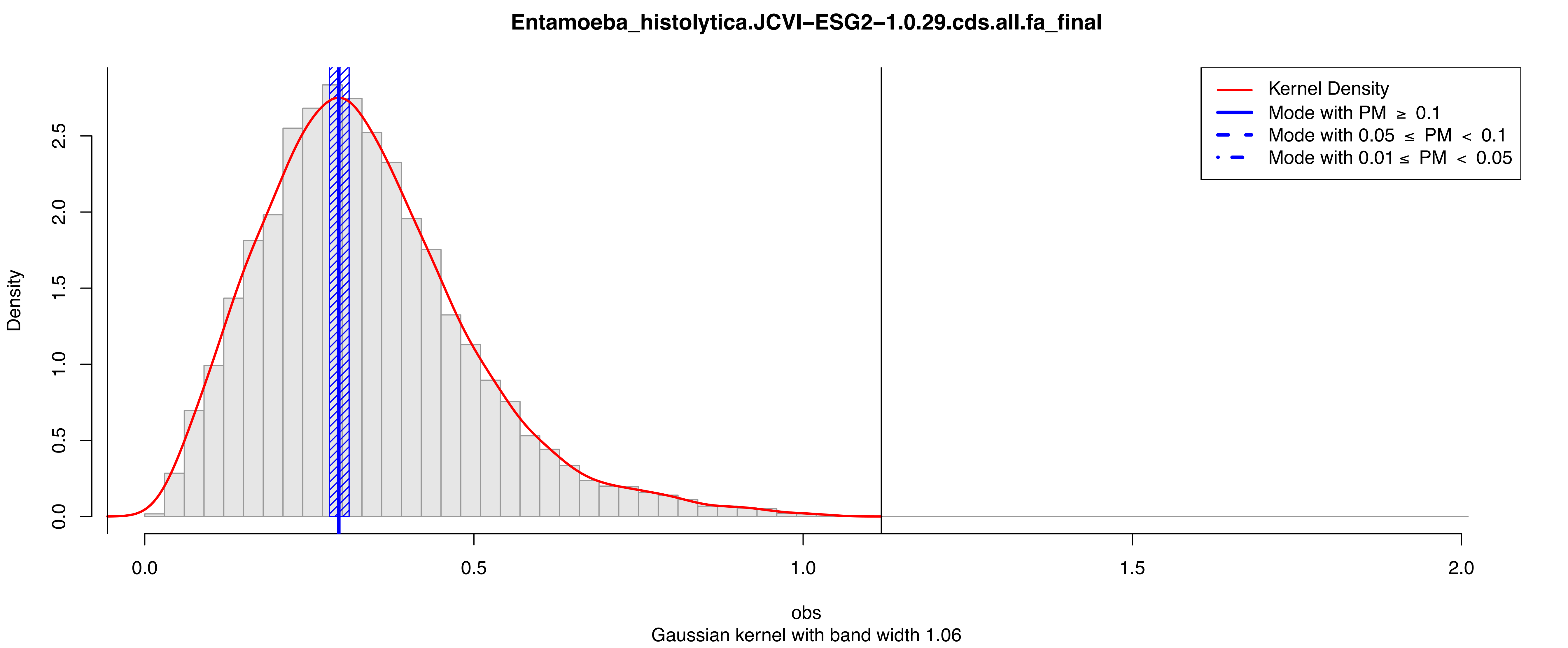

Entamoeba_histolytica.JCVI−ESG2−1.0.29.cds.all.fa_final
Kernel Density
Mode with PM ≥ 0.1
Mode with 0.05 ≤ PM < 0.1
Mode with 0.01 ≤ PM < 0.05
Density
0.0
0.5
1.0
1.5
2.0
2.5
0.0
0.5
1.0
1.5
2.0
obs
Gaussian kernel with band width 1.06

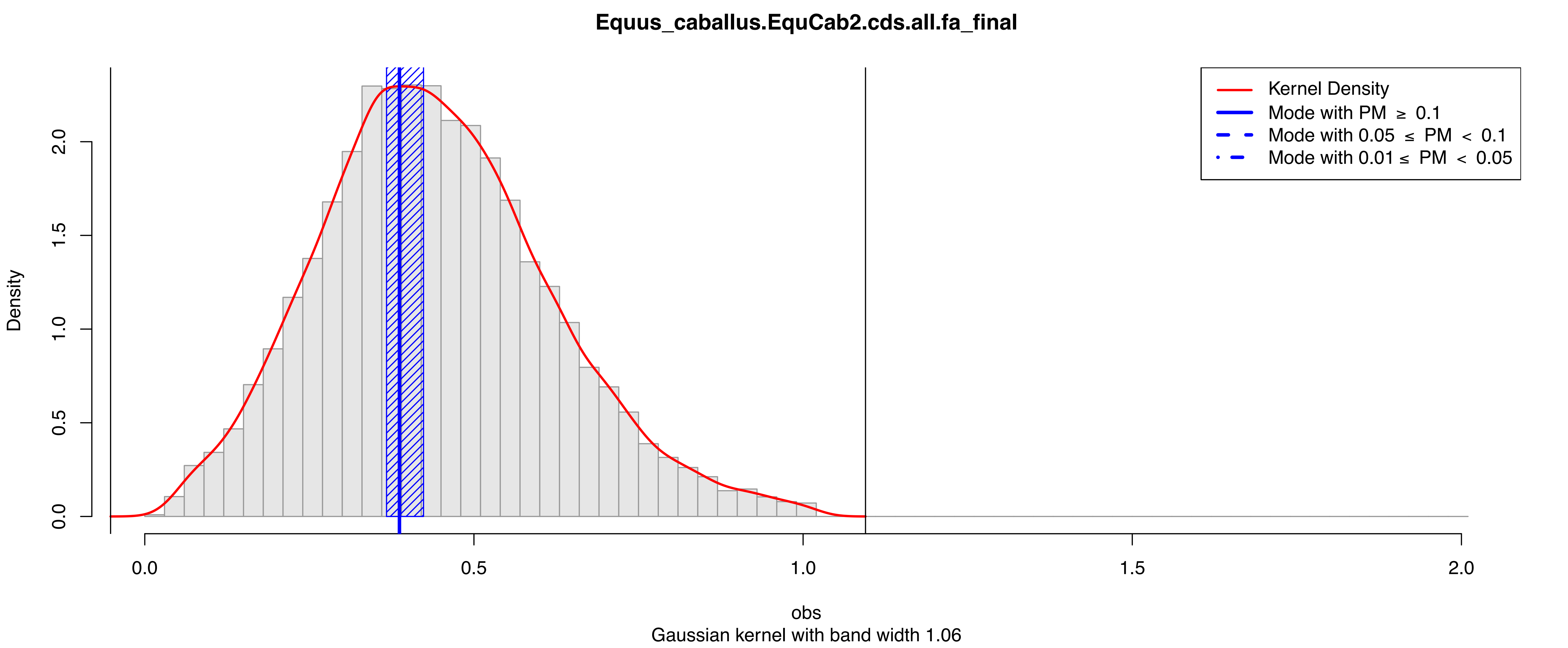

Equus_caballus.EquCab2.cds.all.fa_final
Kernel Density
Mode with PM ≥ 0.1
Mode with 0.05 ≤ PM < 0.1
Mode with 0.01 ≤ PM < 0.05
Density
0.0
0.5
1.0
1.5
2.0
0.0
0.5
1.0
1.5
2.0
obs
Gaussian kernel with band width 1.06

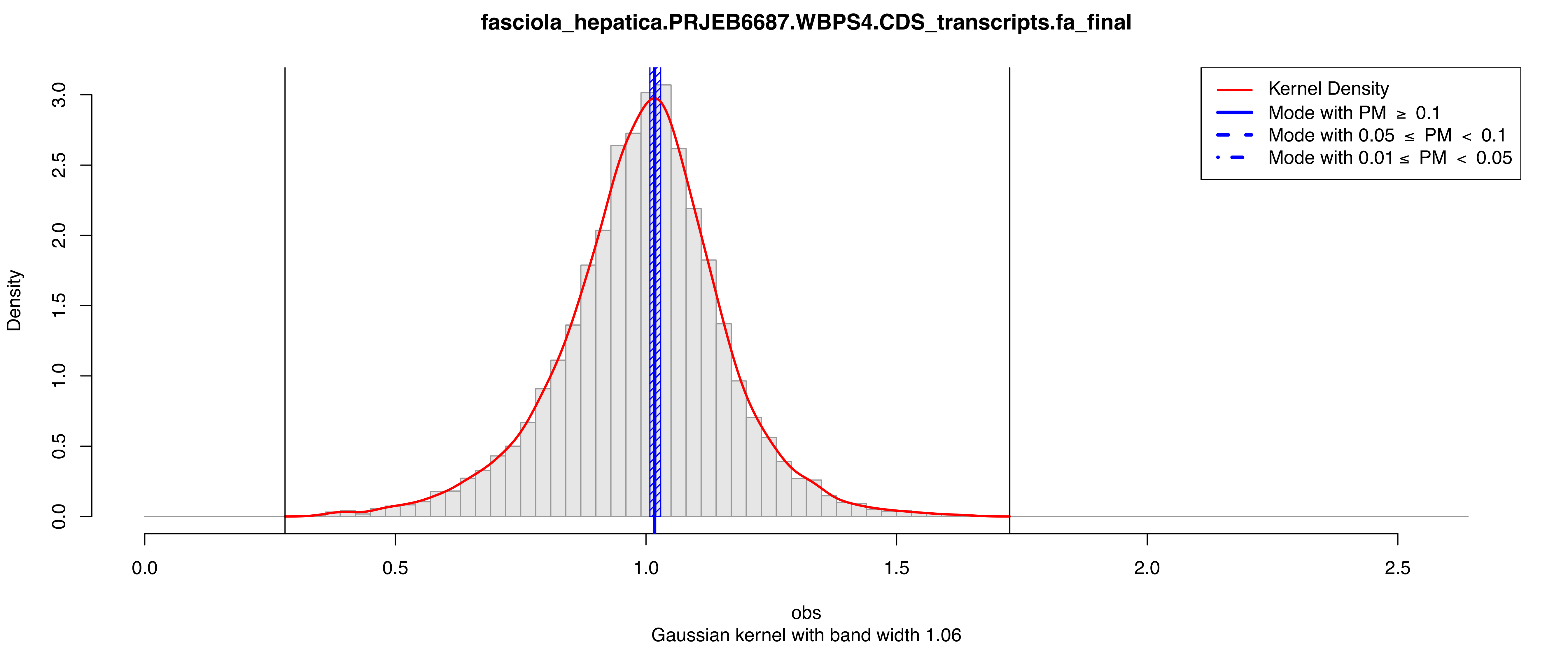
fasciola_hepatica.PRJEB6687.WBPS4.CDS_transcripts.fa_final
Kernel Density
Mode with PM ≥ 0.1
Mode with 0.05 ≤ PM < 0.1
Mode with 0.01 ≤ PM < 0.05
Density
0.0
0.5
1.0
1.5
2.0
2.5
3.0
obs
Gaussian kernel with band width 1.06

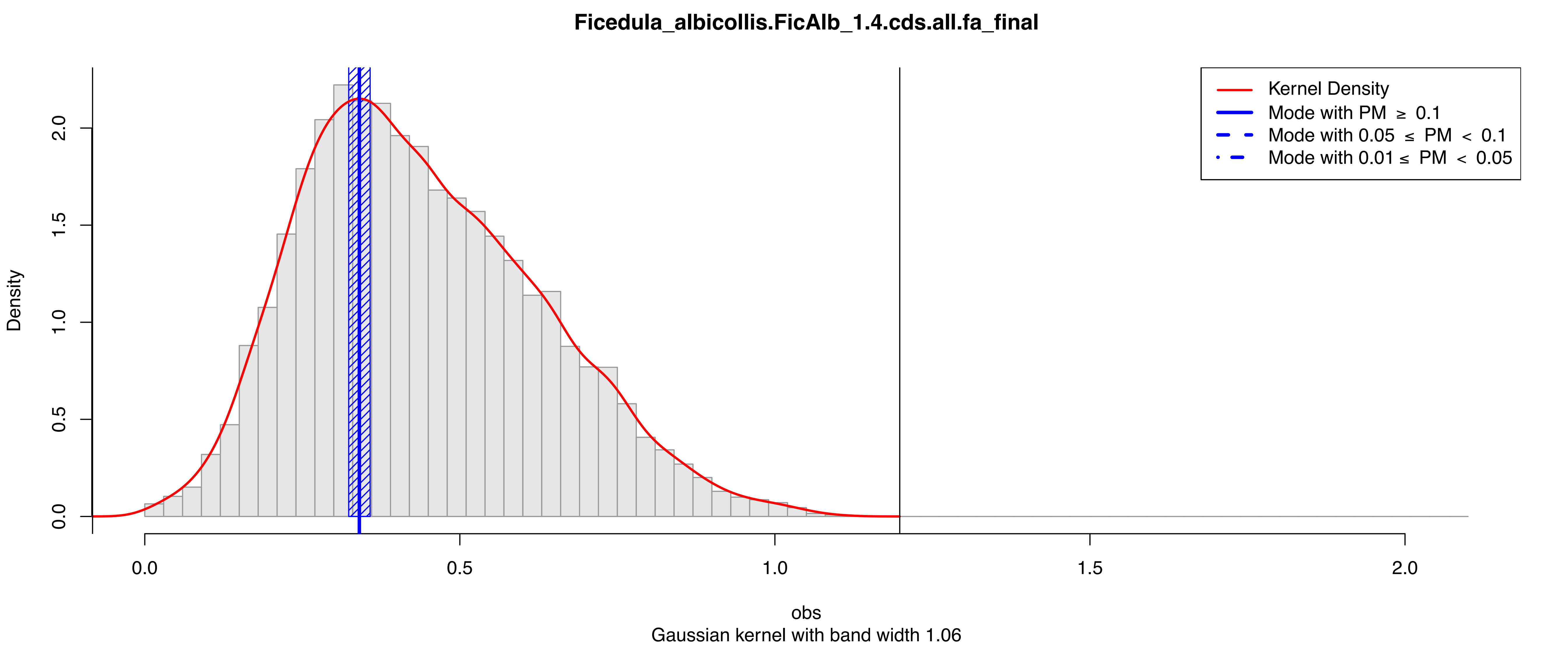

Ficedula_albicollis.FicAlb_1.4.cds.all.fa_final
Kernel Density
Mode with PM ≥ 0.1
Mode with 0.05 ≤ PM < 0.1
Mode with 0.01 ≤ PM < 0.05
Density
0.0
0.5
1.0
1.5
2.0
obs
Gaussian kernel with band width 1.06

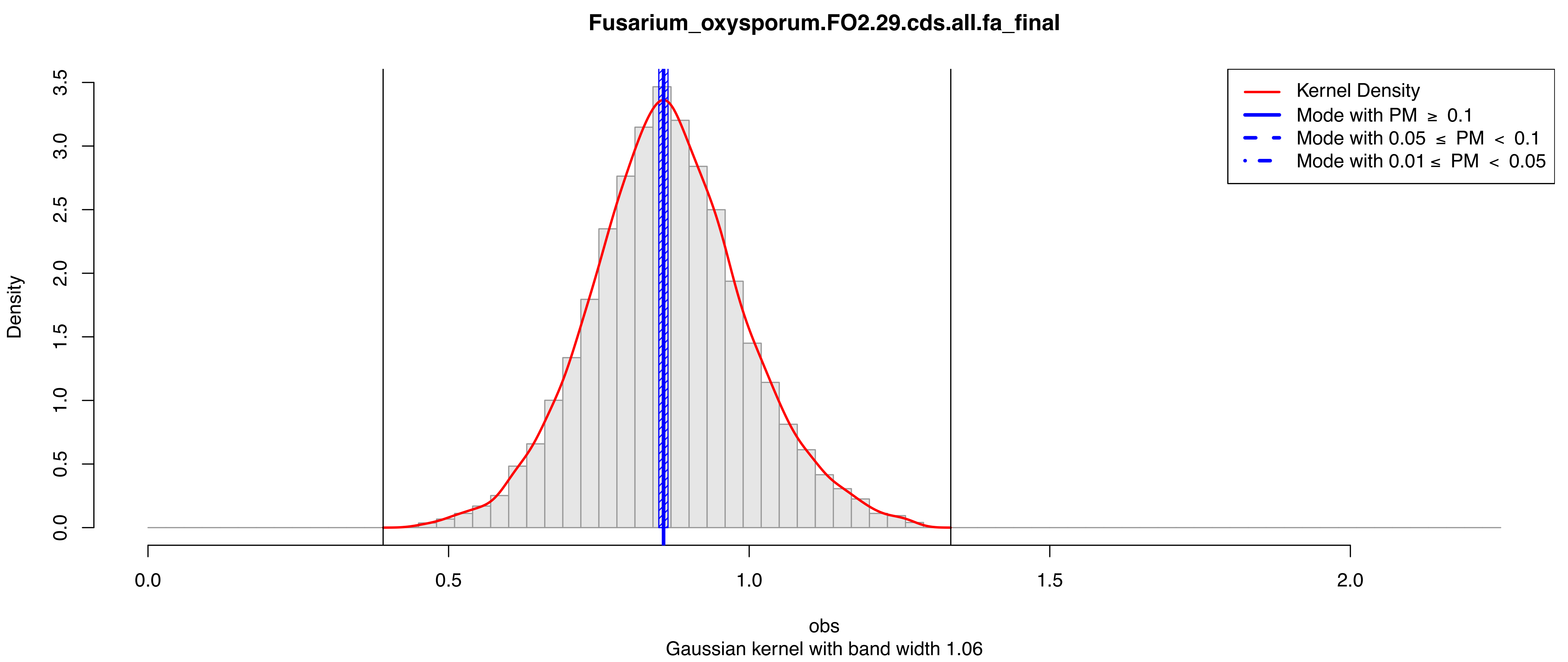
Fusarium_oxysporum.FO2.29.cds.all.fa_final
Density
0.0
0.5
1.0
1.5
2.0
2.5
3.0
3.5
Kernel Density
Mode with PM ≥ 0.1
Mode with 0.05 ≤ PM < 0.1
Mode with 0.01 ≤ PM < 0.05
0.0
0.5
1.0
1.5
2.0
obs
Gaussian kernel with band width 1.06

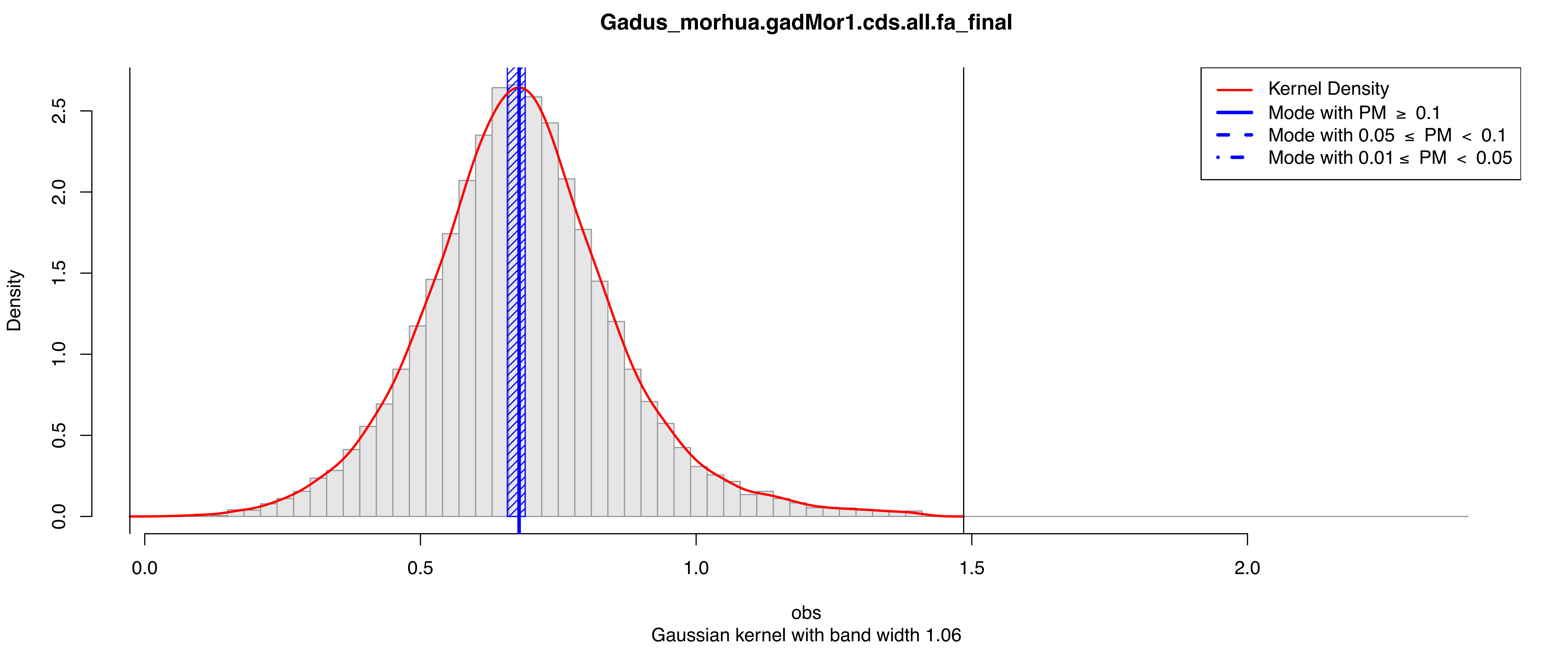

Gadus_morhua.gadMor1.cds.all.fa_final
Kernel Density
Mode with PM ≥ 0.1
Mode with 0.05 ≤ PM < 0.1
Mode with 0.01 ≤ PM < 0.05
Density
0.0
0.5
1.0
1.5
2.0
2.5
0.0
0.5
1.0
1.5
2.0
obs
Gaussian kernel with band width 1.06

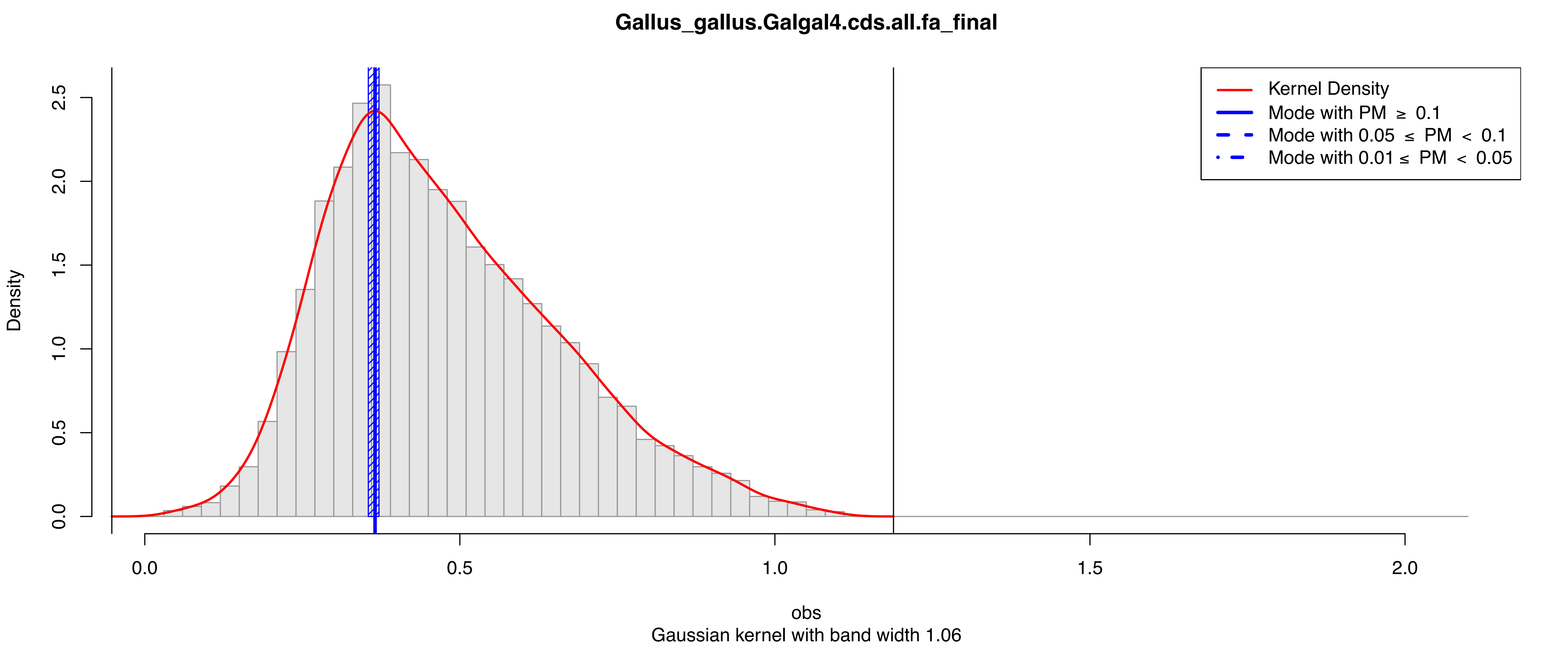
Gallus_gallus.Galgal4.cds.all.fa_final
Kernel Density
Mode with PM ≥ 0.1
Mode with 0.05 ≤ PM < 0.1
Mode with 0.01 ≤ PM < 0.05
Density
0.0
0.5
1.0
1.5
2.0
2.5
0.0
0.5
1.0
1.5
2.0
obs
Gaussian kernel with band width 1.06

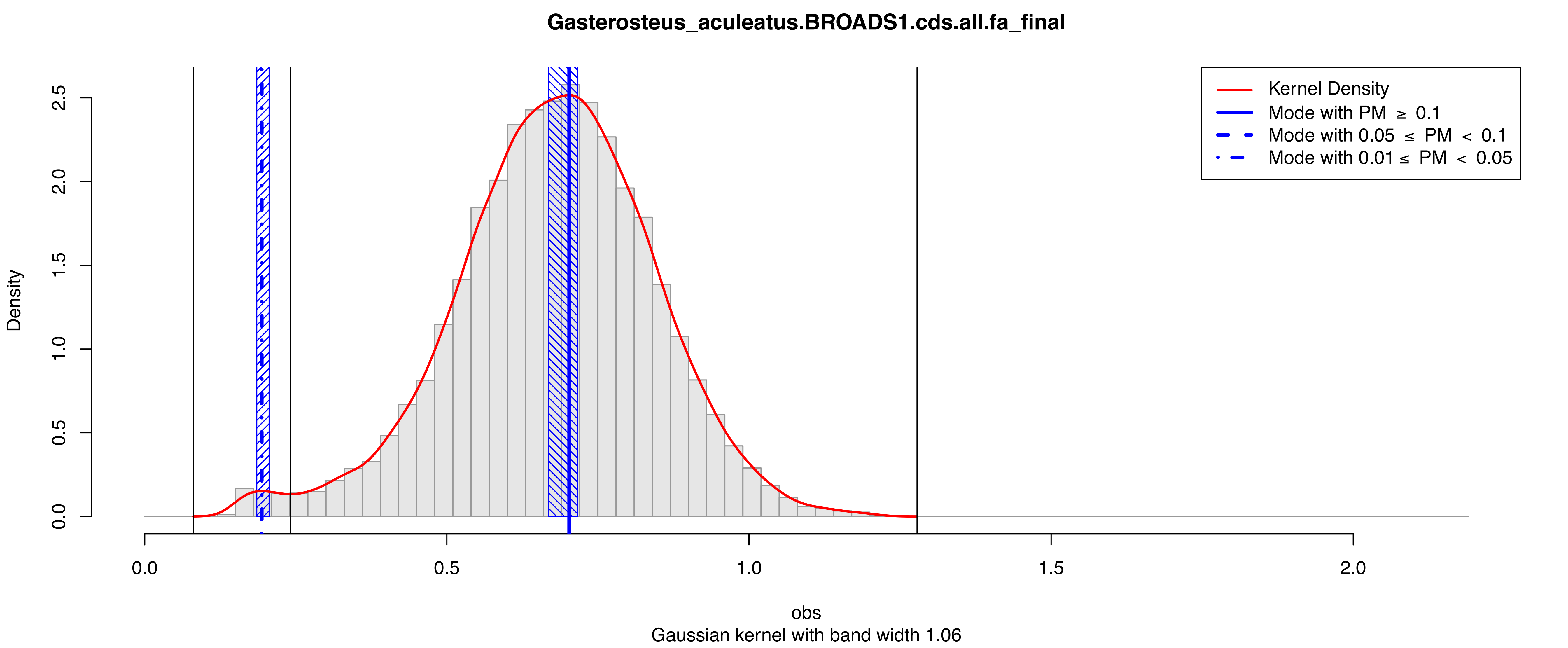

Gasterosteus_aculeatus.BROADS1.cds.all.fa_final
Kernel Density
Mode with PM ≥ 0.1
Mode with 0.05 ≤ PM < 0.1
Mode with 0.01 ≤ PM < 0.05
Density
0.0
0.5
1.0
1.5
2.0
2.5
0.0
0.5
1.0
1.5
2.0
obs
Gaussian kernel with band width 1.06

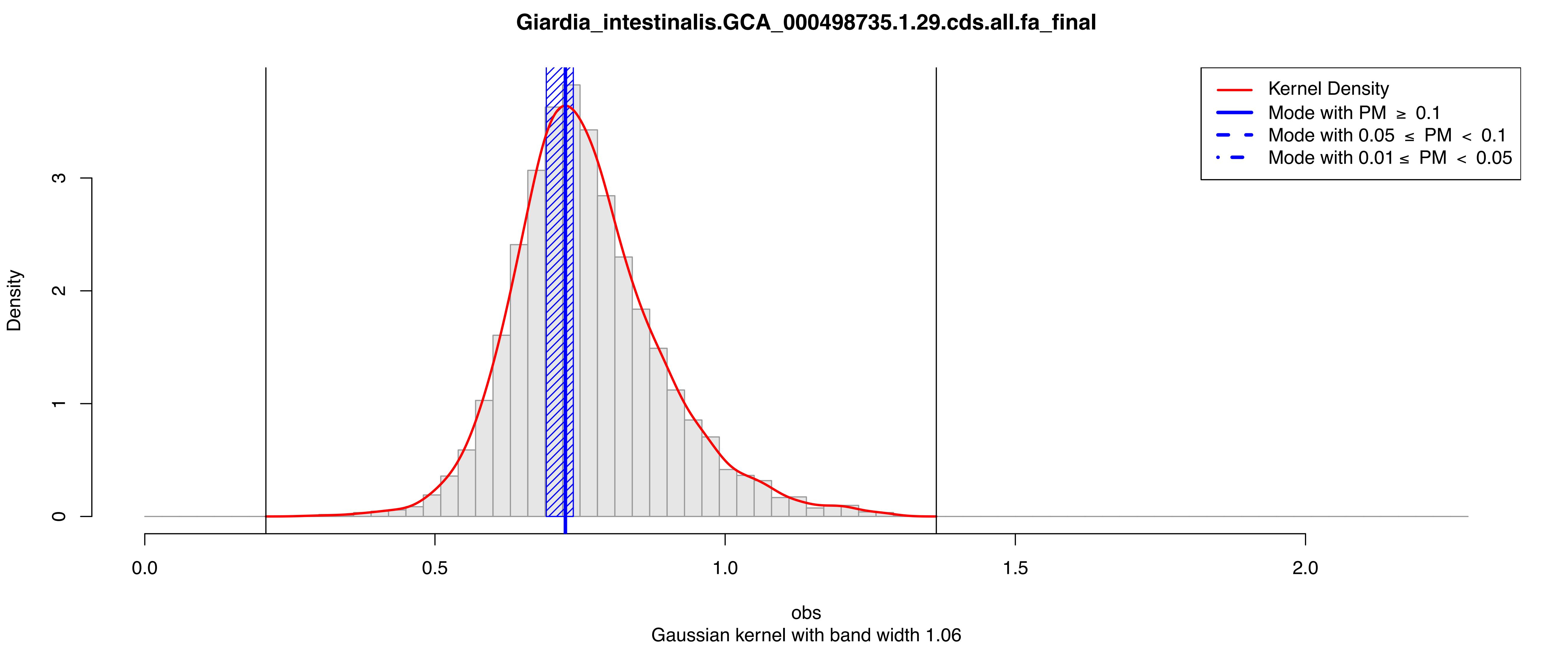

Giardia_intestinalis.GCA_000498735.1.29.cds.all.fa_final
Kernel Density
Mode with PM ≥ 0.1
Mode with 0.05 ≤ PM < 0.1
Mode with 0.01 ≤ PM < 0.05
Density
0
1
2
3
0.0
0.5
1.0
1.5
2.0
obs
Gaussian kernel with band width 1.06

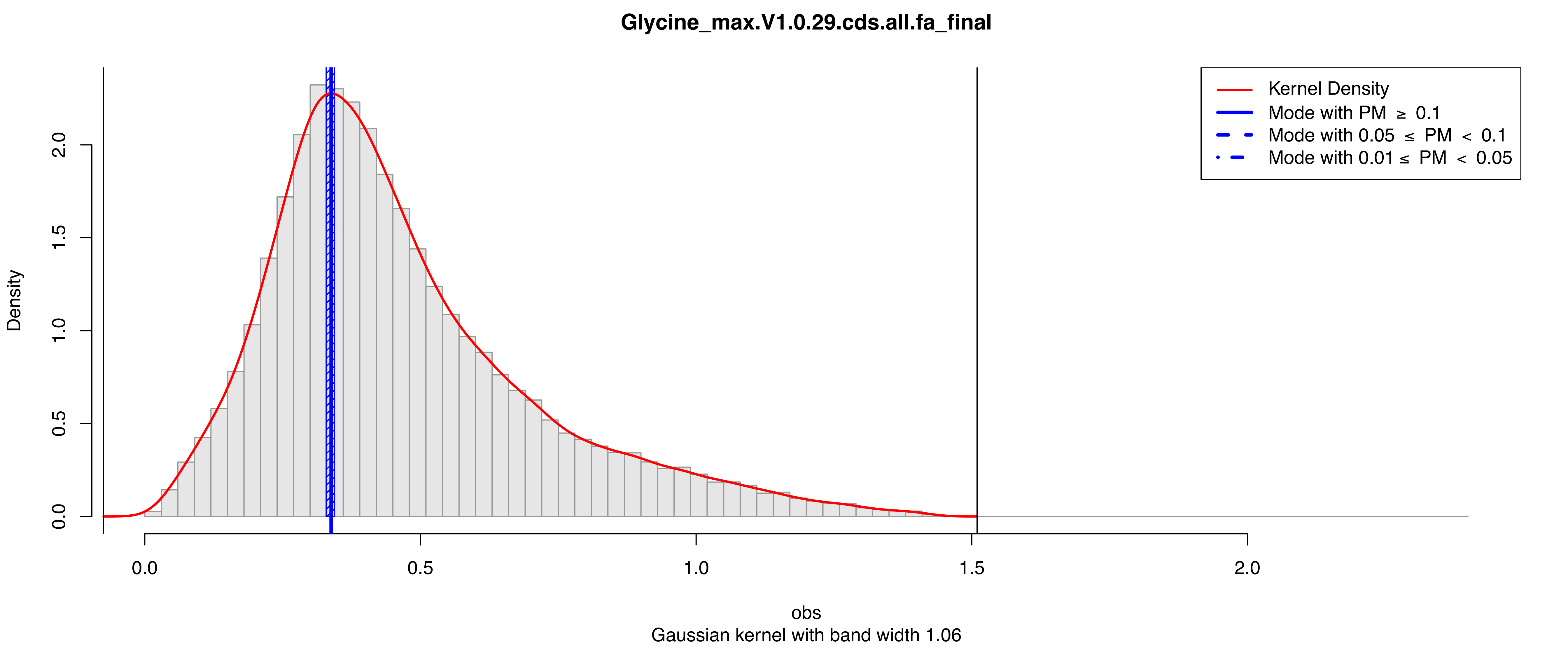

Glycine_max.V1.0.29.cds.all.fa_final
Kernel Density
Mode with PM ≥ 0.1
Mode with 0.05 ≤ PM < 0.1
Mode with 0.01 ≤ PM < 0.05
Density
0.0
0.5
1.0
1.5
2.0
obs
Gaussian kernel with band width 1.06

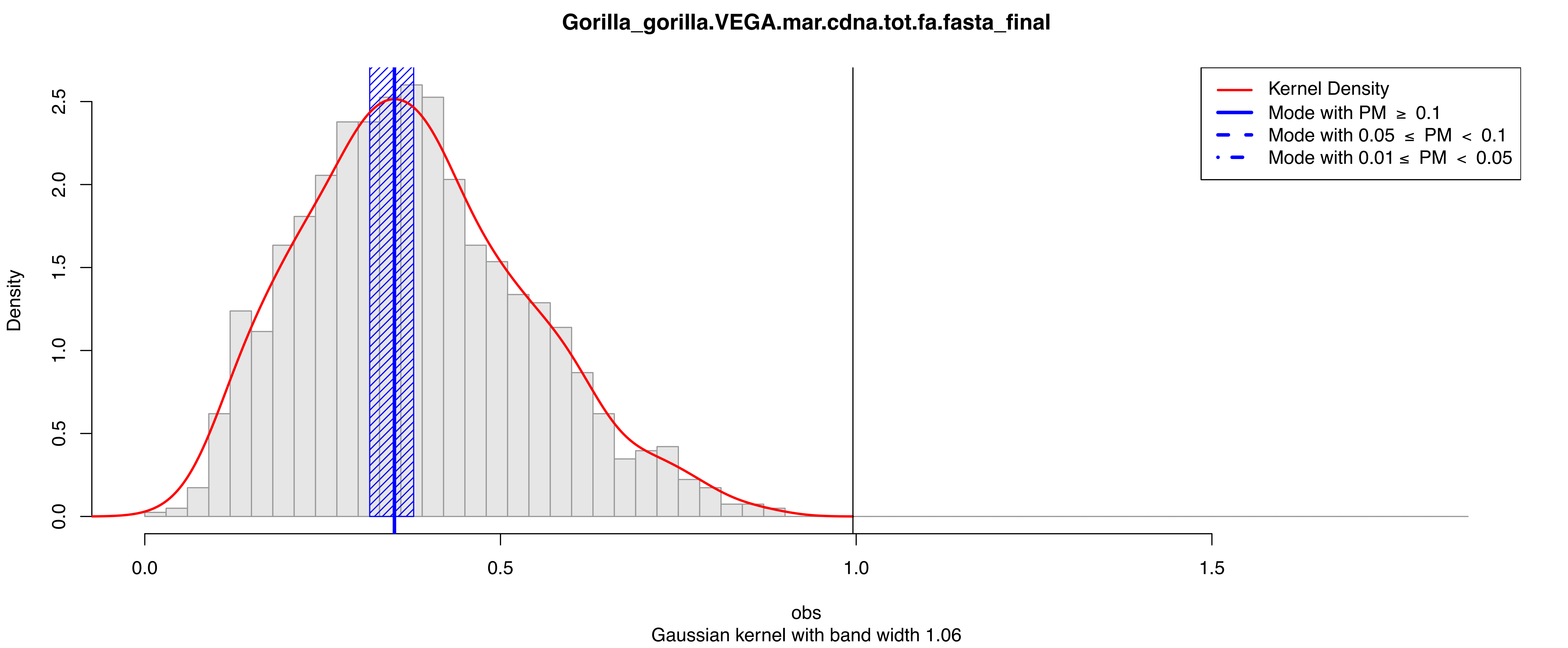

Gorilla_gorilla.VEGA.mar.cdna.tot.fa.fasta_final
Kernel Density
Mode with PM ≥ 0.1
Mode with 0.05 ≤ PM < 0.1
Mode with 0.01 ≤ PM < 0.05
Density
0.0
0.5
1.0
1.5
2.0
2.5
0.0
0.5
1.0
1.5
obs
Gaussian kernel with band width 1.06

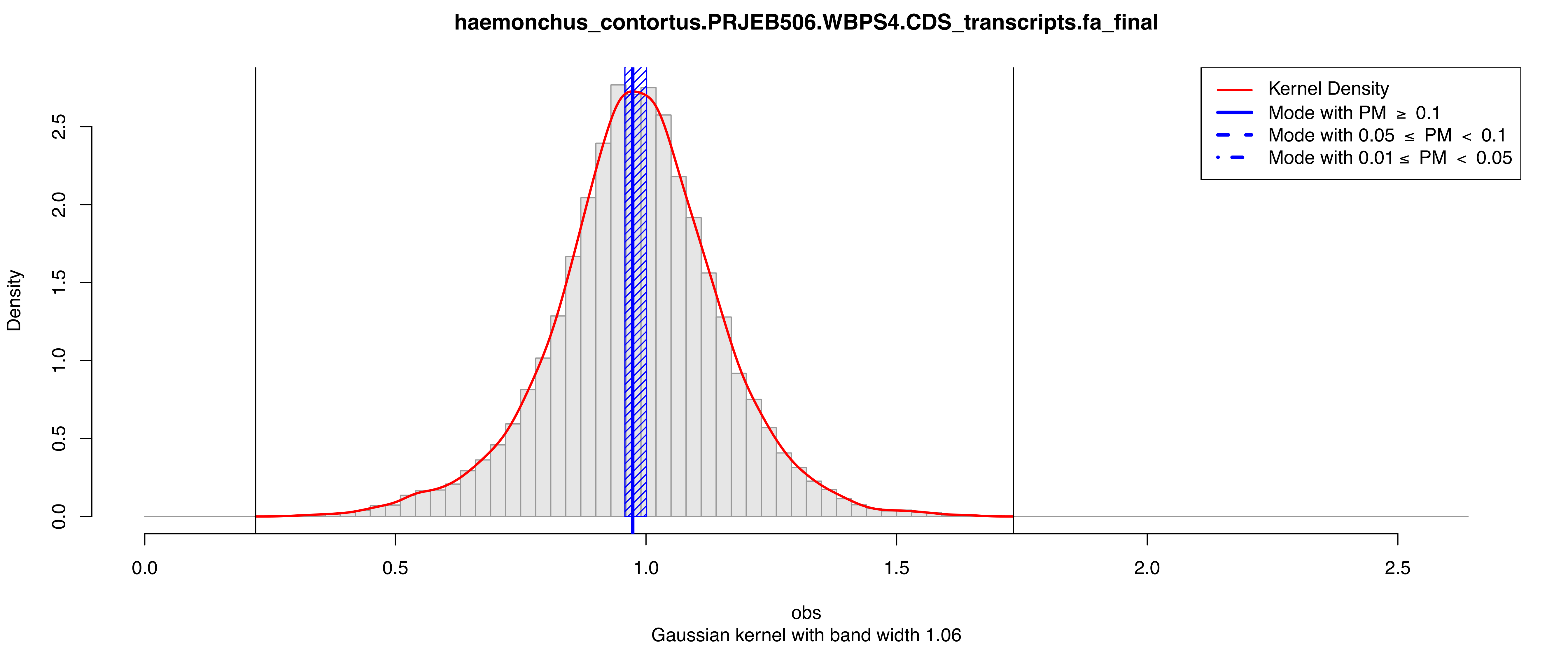
haemonchus_contortus.PRJEB506.WBPS4.CDS_transcripts.fa_final
Kernel Density
Mode with PM ≥ 0.1
Mode with 0.05 ≤ PM < 0.1
Mode with 0.01 ≤ PM < 0.05
Density
0.0
0.5
1.0
1.5
2.0
2.5
obs
Gaussian kernel with band width 1.06

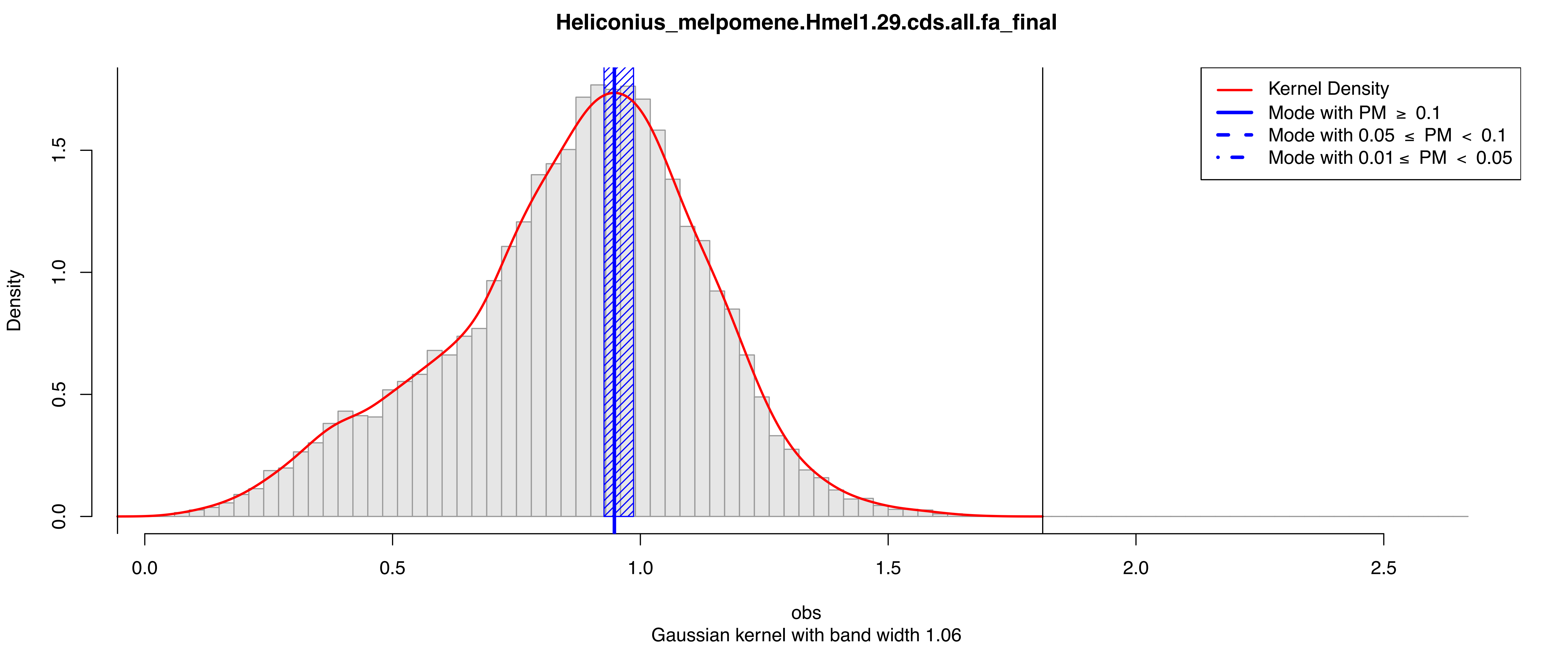

Heliconius_melpomene.Hmel1.29.cds.all.fa_final
Kernel Density
Mode with PM ≥ 0.1
Mode with 0.05 ≤ PM < 0.1
Mode with 0.01 ≤ PM < 0.05
Density
0.0
0.5
1.0
1.5
0.0
0.5
1.0
1.5
2.0
2.5
obs
Gaussian kernel with band width 1.06

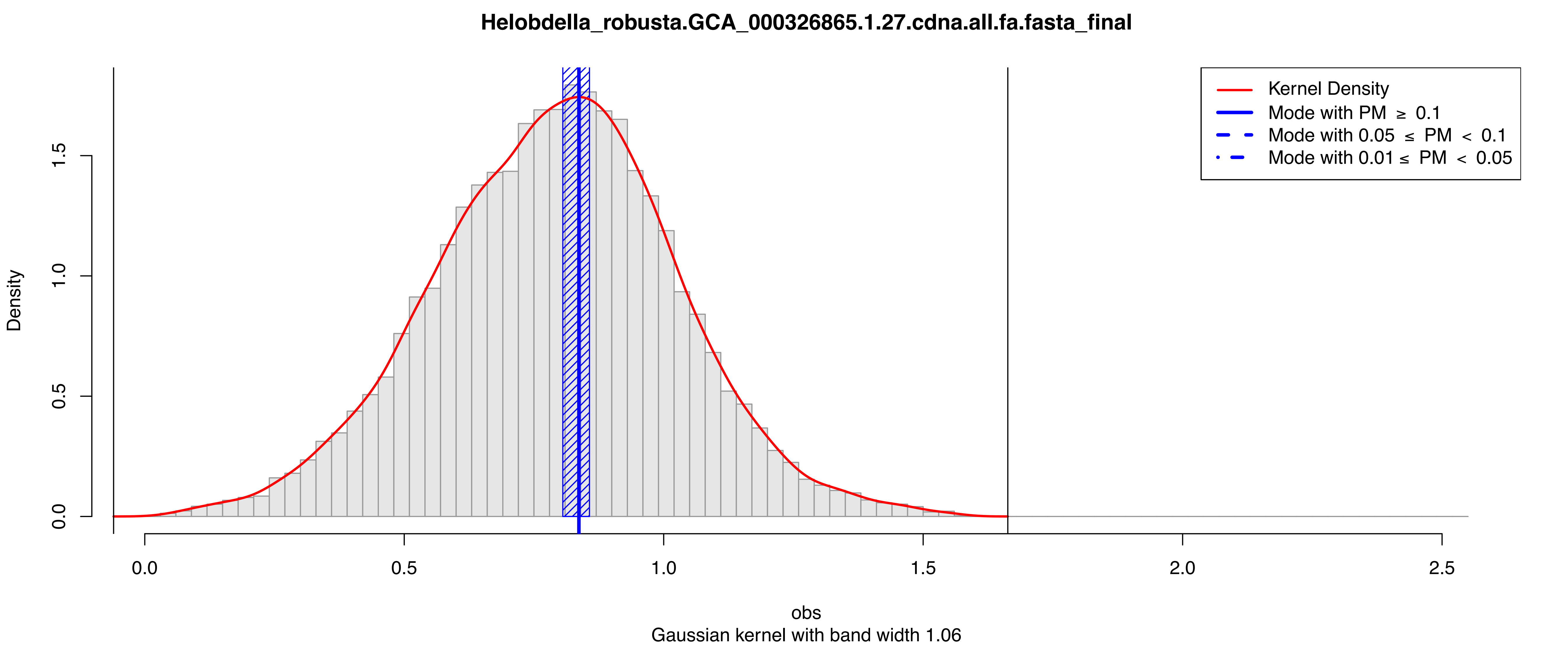

Helobdella_robusta.GCA_000326865.1.27.cdna.all.fa.fasta_final
Kernel Density
Mode with PM ≥ 0.1
Mode with 0.05 ≤ PM < 0.1
Mode with 0.01 ≤ PM < 0.05
Density
0.0
0.5
1.0
1.5
0.0
0.5
1.0
1.5
2.0
2.5
obs
Gaussian kernel with band width 1.06

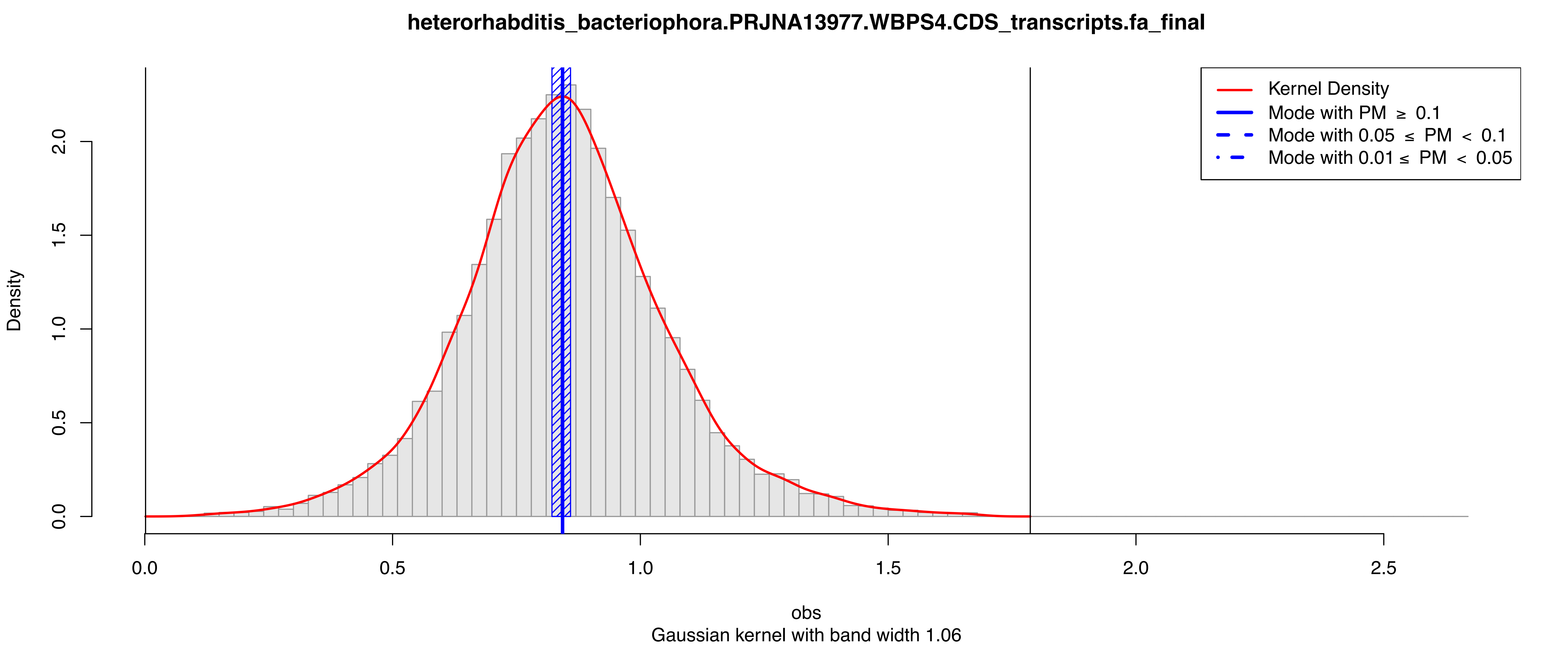
heterorhabditis_bacteriophora.PRJNA13977.WBPS4.CDS_transcripts.fa_final
Kernel Density
Mode with PM ≥ 0.1
Mode with 0.05 ≤ PM < 0.1
Mode with 0.01 ≤ PM < 0.05
Density
0.0
0.5
1.0
1.5
2.0
2.5
obs
Gaussian kernel with band width 1.06

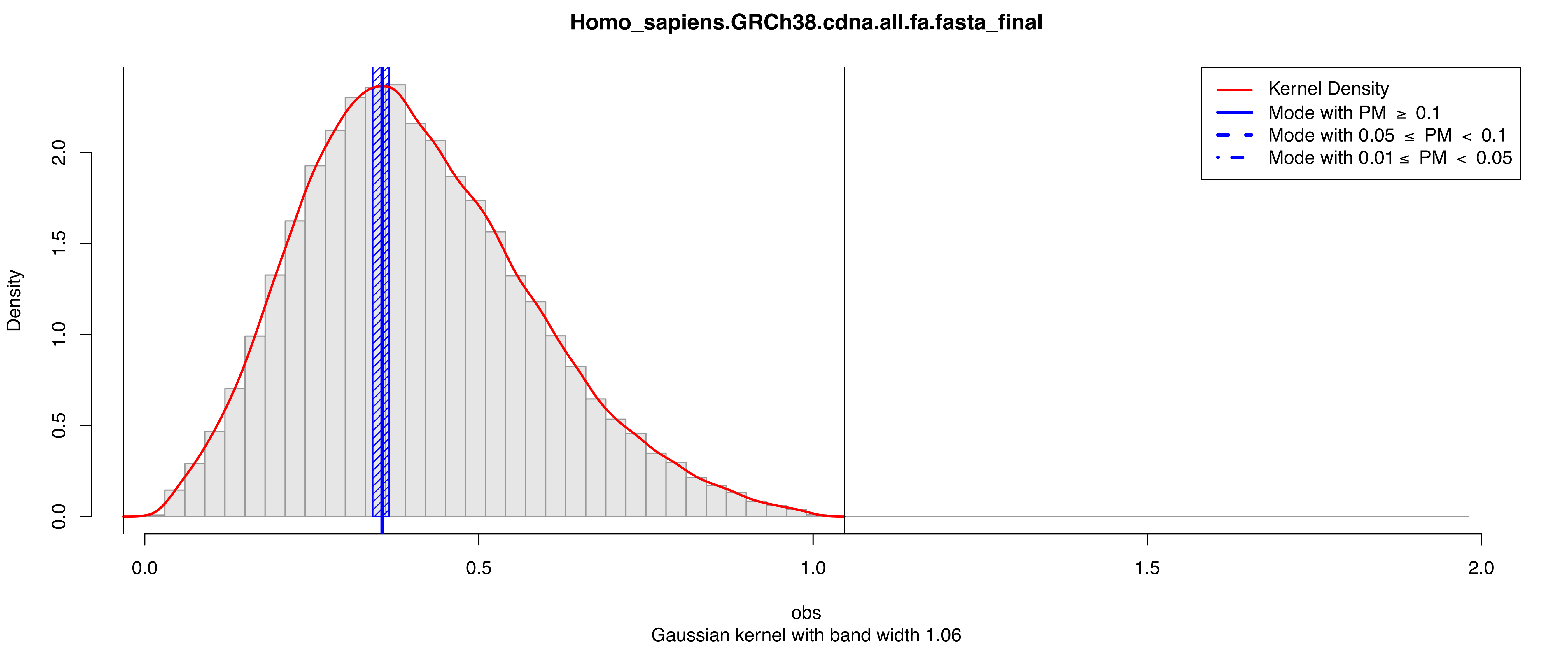
Homo_sapiens.GRCh38.cdna.all.fa.fasta_final
Kernel Density
Mode with PM ≥ 0.1
Mode with 0.05 ≤ PM < 0.1
Mode with 0.01 ≤ PM < 0.05
Density
0.0
0.5
1.0
1.5
2.0
0.0
0.5
1.0
1.5
2.0
obs
Gaussian kernel with band width 1.06

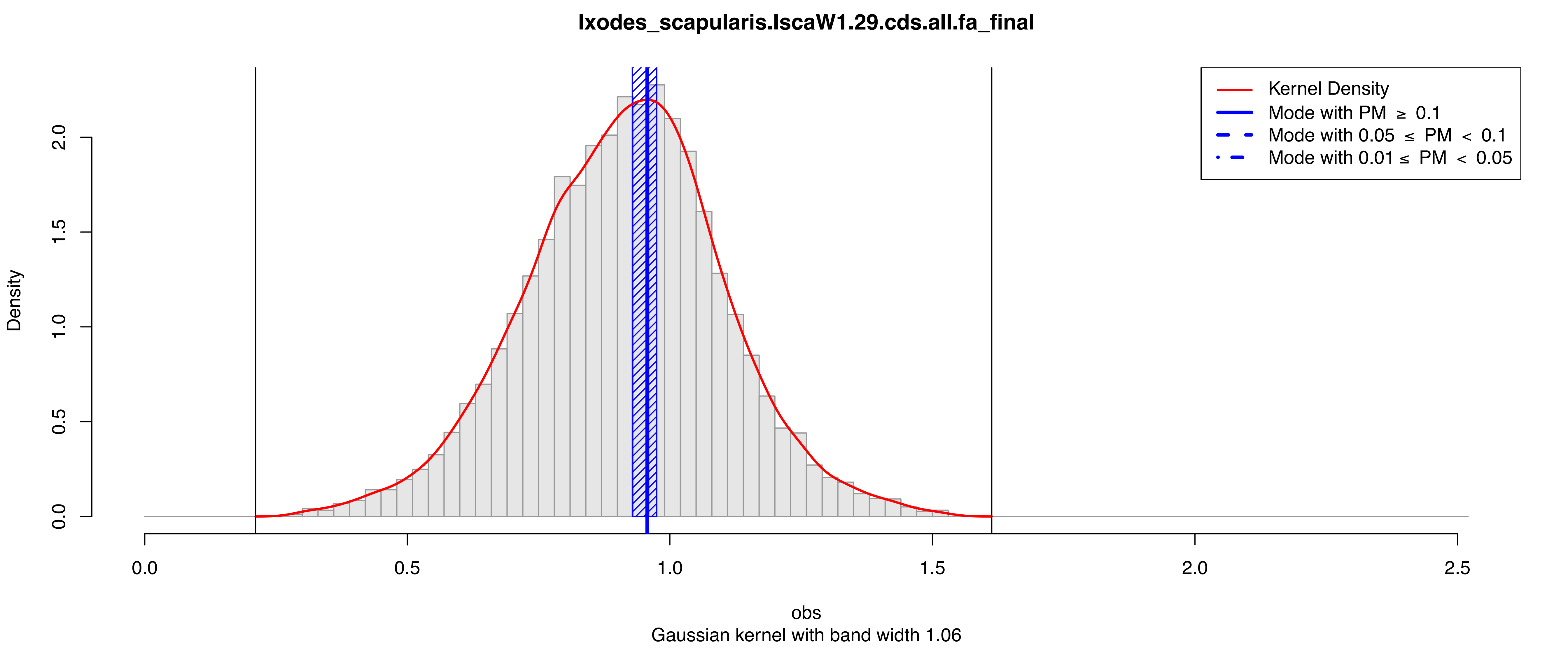

Ixodes_scapularis.IscaW1.29.cds.all.fa_final
Kernel Density
Mode with PM ≥ 0.1
Mode with 0.05 ≤ PM < 0.1
Mode with 0.01 ≤ PM < 0.05
Density
0.0
0.5
1.0
1.5
2.0
0.0
0.5
1.0
1.5
2.0
2.5
obs
Gaussian kernel with band width 1.06

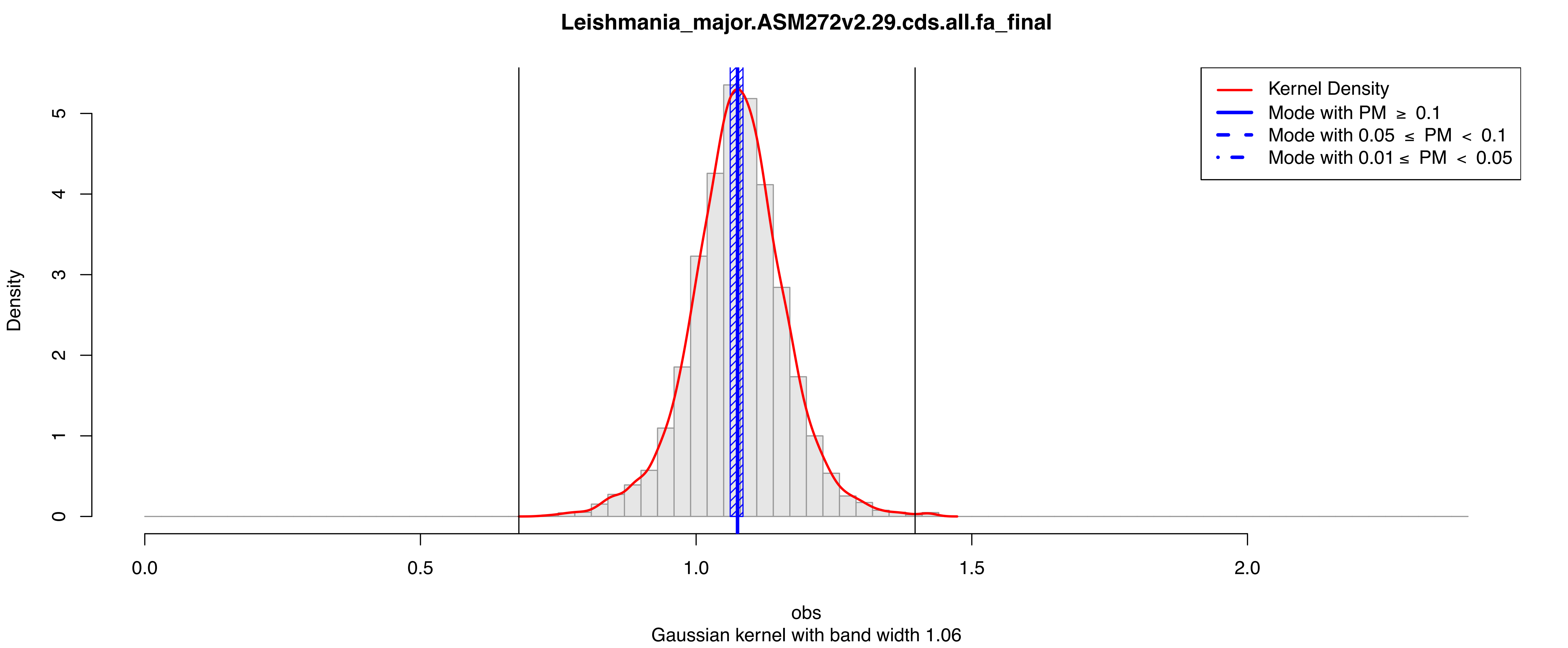
Leishmania_major.ASM272v2.29.cds.all.fa_final
Kernel Density
Mode with PM ≥ 0.1
Mode with 0.05 ≤ PM < 0.1
Mode with 0.01 ≤ PM < 0.05
Density
0
1
2
3
4
5
0.0
0.5
1.0
1.5
2.0
obs
Gaussian kernel with band width 1.06

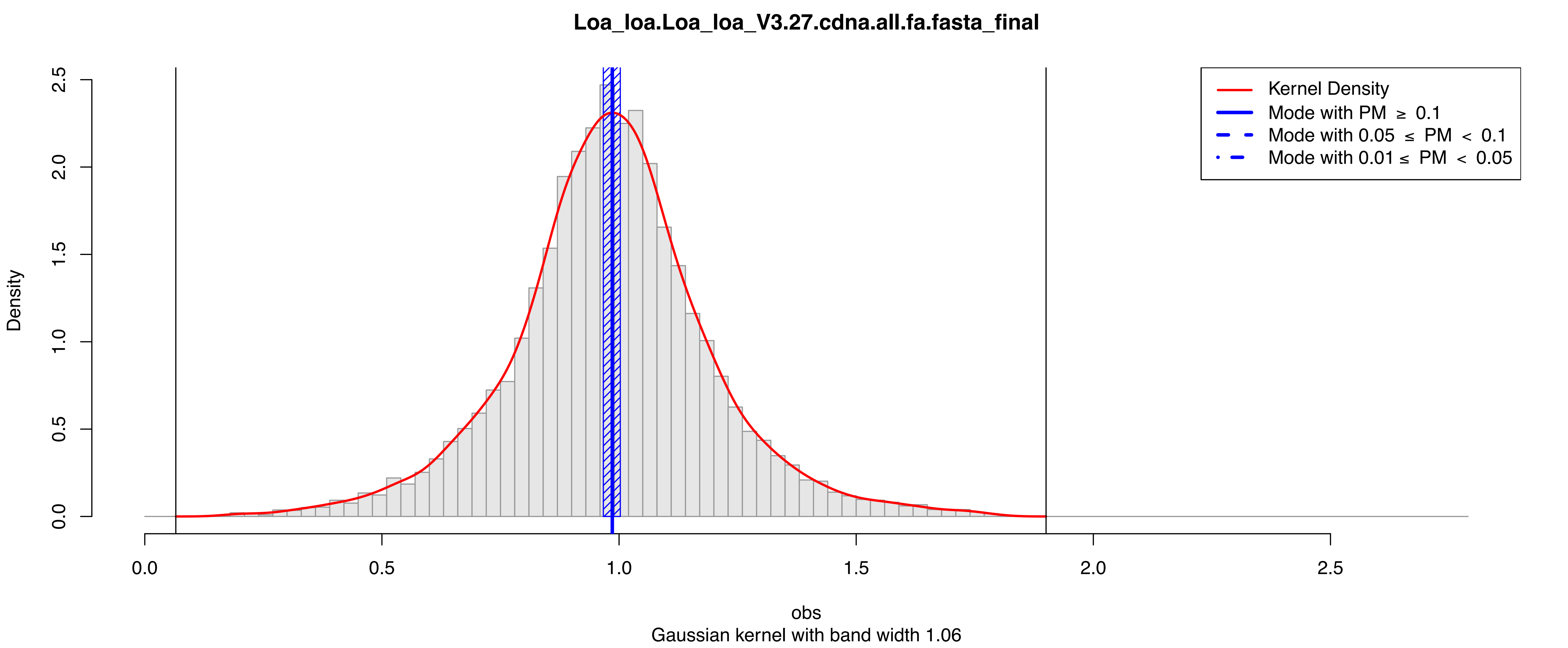

Loa_loa.Loa_loa_V3.27.cdna.all.fa.fasta_final
Kernel Density
Mode with PM ≥ 0.1
Mode with 0.05 ≤ PM < 0.1
Mode with 0.01 ≤ PM < 0.05
Density
0.0
0.5
1.0
1.5
2.0
2.5
obs
Gaussian kernel with band width 1.06

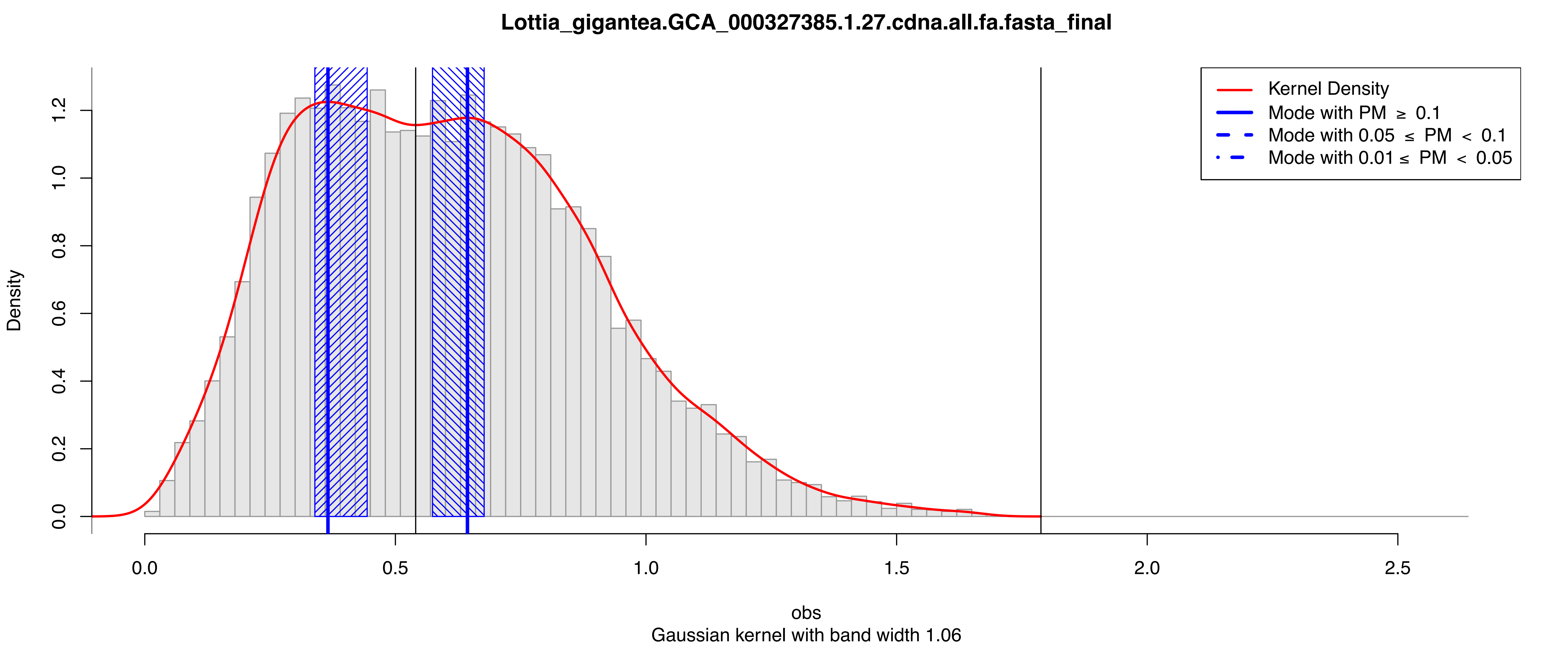

Lottia_gigantea.GCA_000327385.1.27.cdna.all.fa.fasta_final
Kernel Density
Mode with PM ≥ 0.1
Mode with 0.05 ≤ PM < 0.1
Mode with 0.01 ≤ PM < 0.05
Density
0.0
0.2
0.4
0.6
0.8
1.0
1.2
0.0
0.5
1.0
1.5
2.0
2.5
obs
Gaussian kernel with band width 1.06

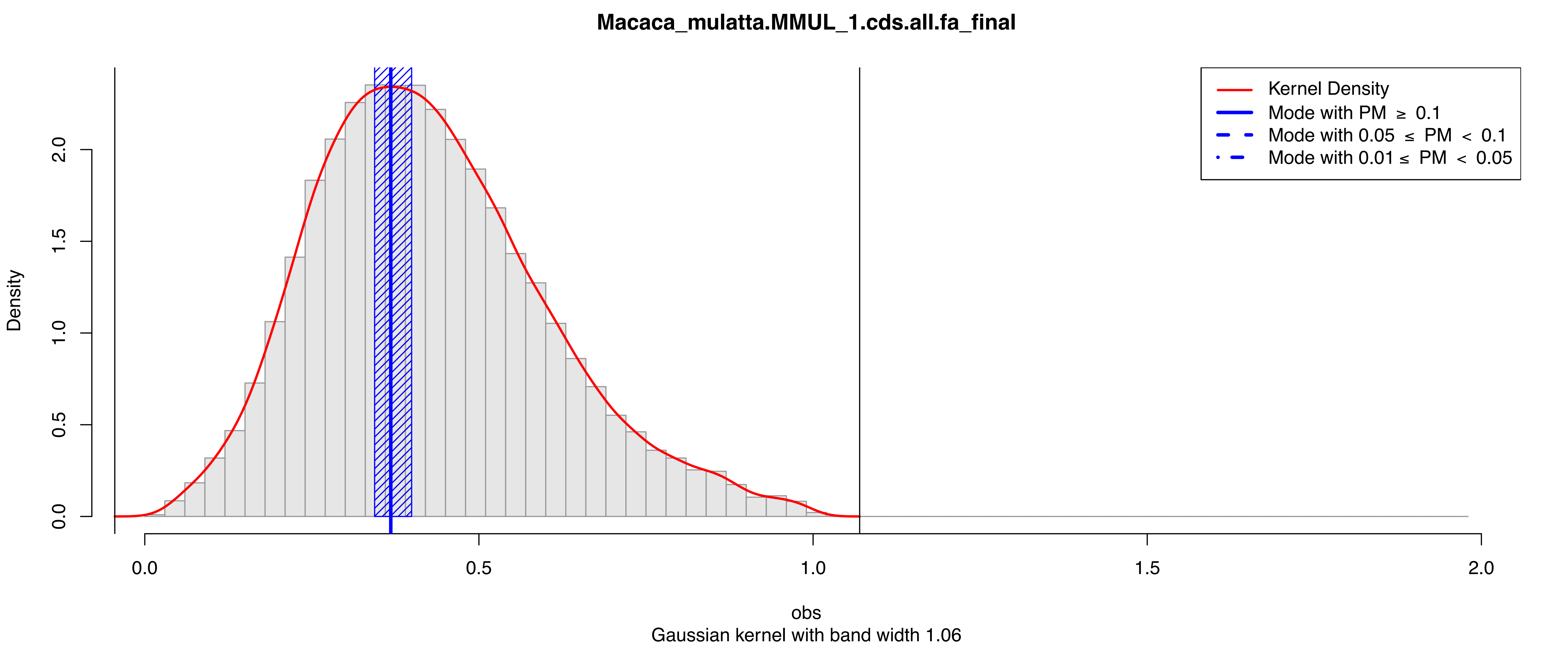

Macaca_mulatta.MMUL_1.cds.all.fa_final
Kernel Density
Mode with PM ≥ 0.1
Mode with 0.05 ≤ PM < 0.1
Mode with 0.01 ≤ PM < 0.05
Density
0.0
0.5
1.0
1.5
2.0
0.0
0.5
1.0
1.5
2.0
obs
Gaussian kernel with band width 1.06

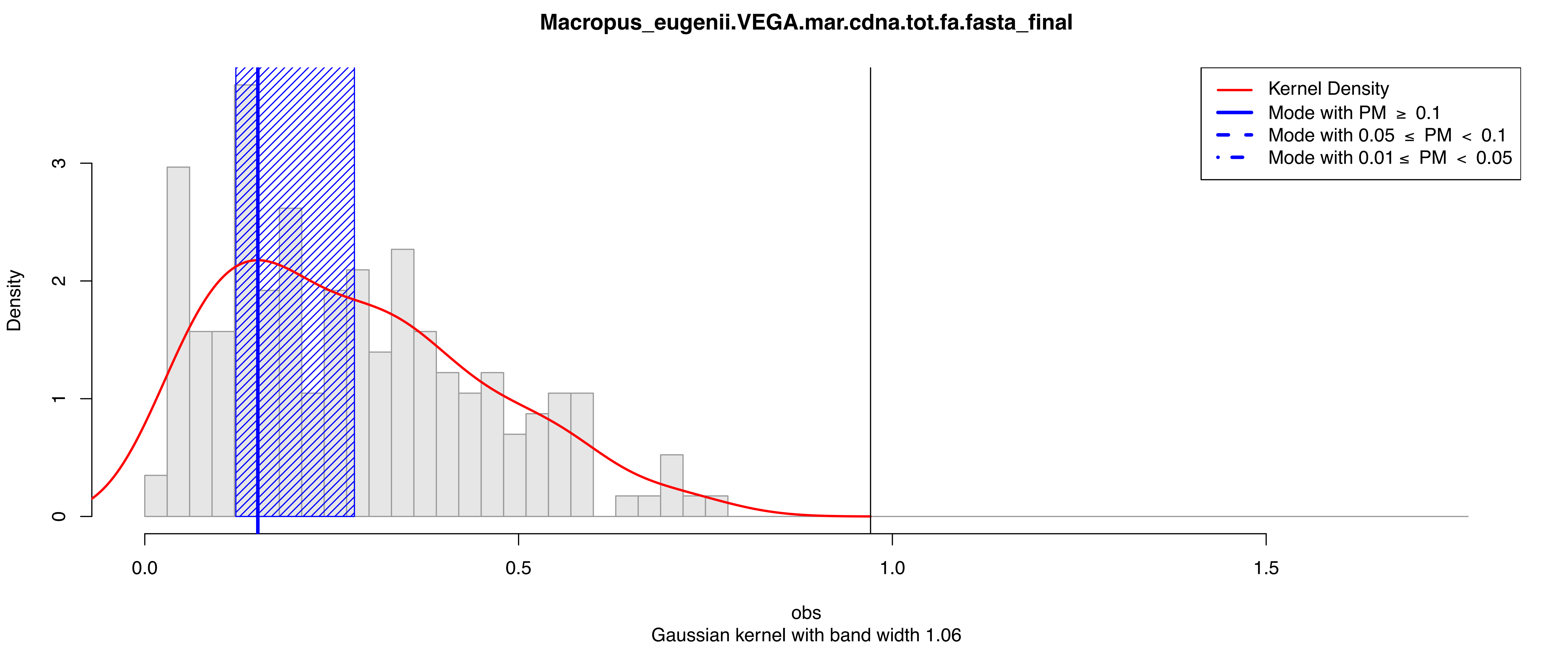

Macropus_eugenii.VEGA.mar.cdna.tot.fa.fasta_final
Kernel Density
Mode with PM ≥ 0.1
Mode with 0.05 ≤ PM < 0.1
Mode with 0.01 ≤ PM < 0.05
Density
0
1
2
3
0.0
0.5
1.0
1.5
obs
Gaussian kernel with band width 1.06

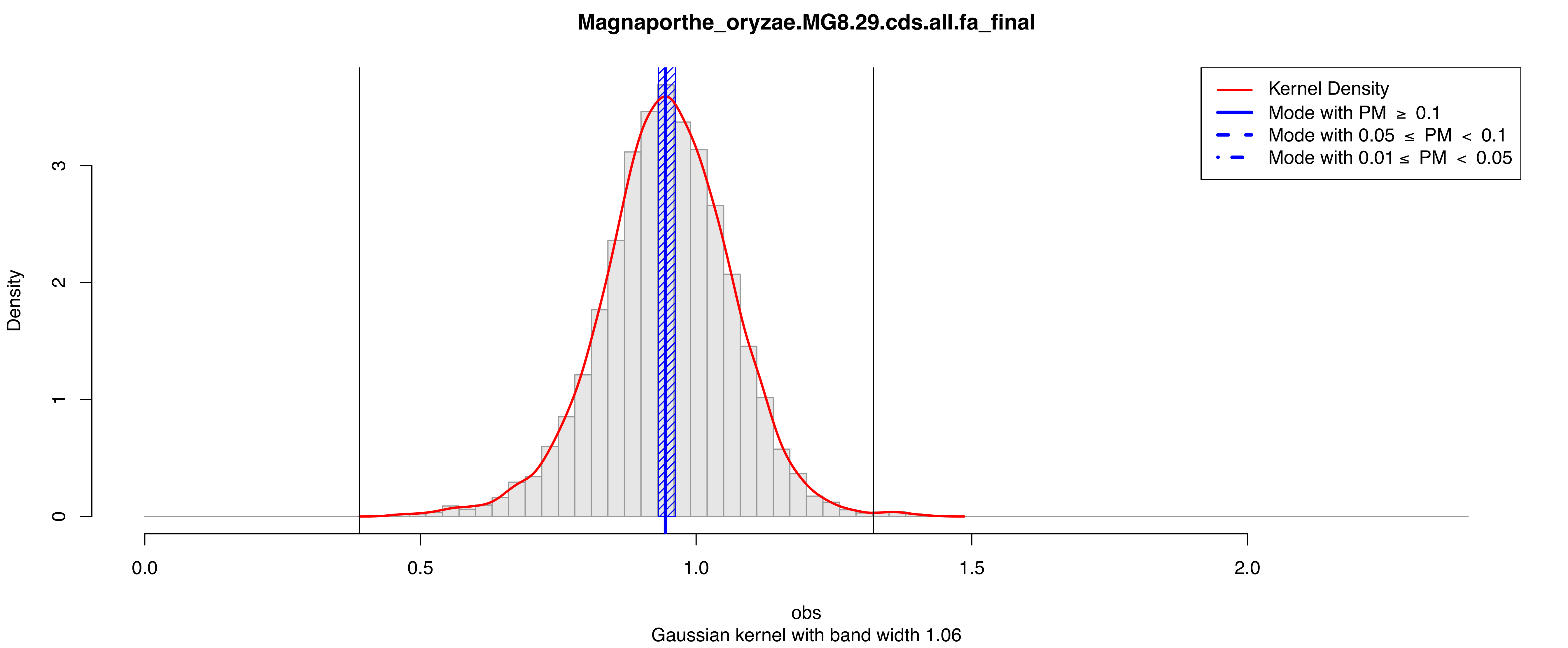
Magnaporthe_oryzae.MG8.29.cds.all.fa_final
Kernel Density
Mode with PM ≥ 0.1
Mode with 0.05 ≤ PM < 0.1
Mode with 0.01 ≤ PM < 0.05
Density
0
1
2
3
0.0
0.5
1.0
1.5
2.0
obs
Gaussian kernel with band width 1.06

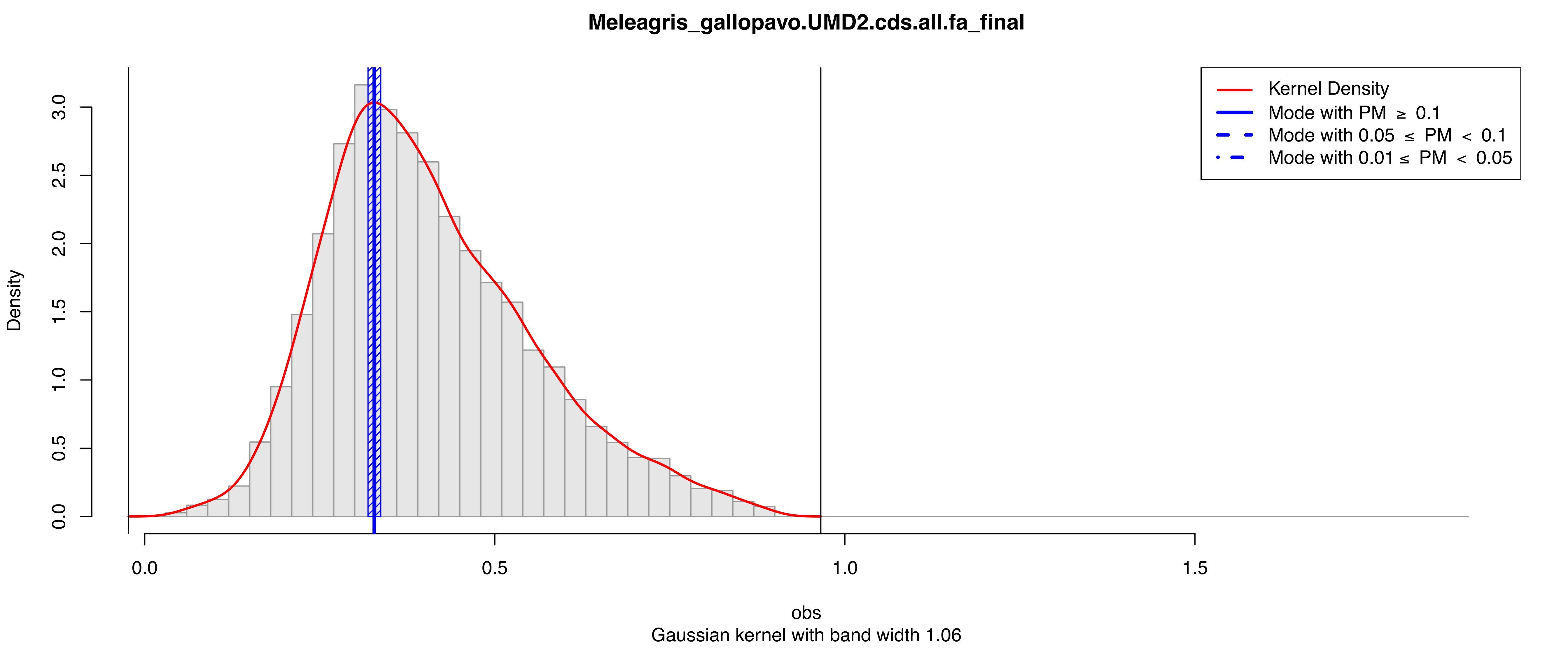
Meleagris_gallopavo.UMD2.cds.all.fa_final
Kernel Density
Mode with PM ≥ 0.1
Mode with 0.05 ≤ PM < 0.1
Mode with 0.01 ≤ PM < 0.05
Density
0.0
0.5
1.0
1.5
2.0
2.5
3.0
0.0
0.5
1.0
1.5
obs
Gaussian kernel with band width 1.06

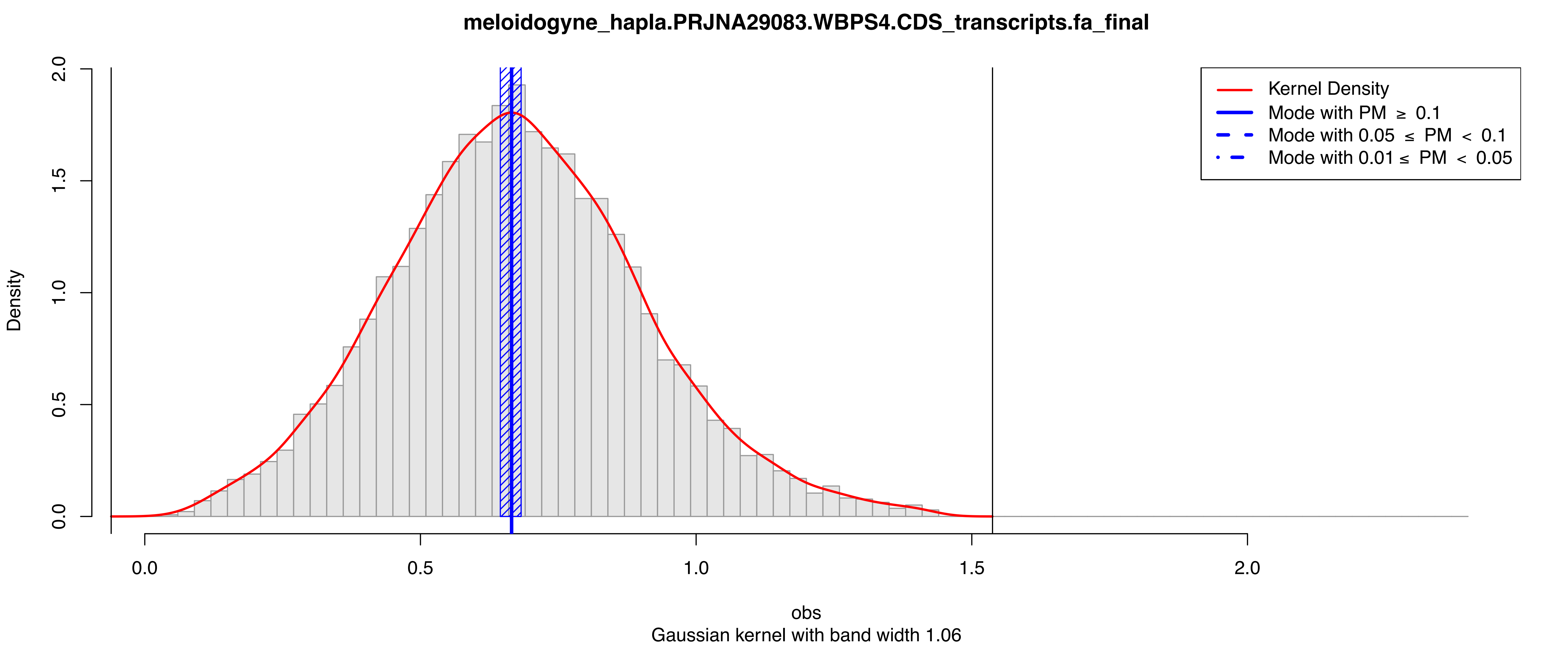
meloidogyne_hapla.PRJNA29083.WBPS4.CDS_transcripts.fa_final
Kernel Density
Mode with PM ≥ 0.1
Mode with 0.05 ≤ PM < 0.1
Mode with 0.01 ≤ PM < 0.05
Density
0.0
0.5
1.0
1.5
2.0
0.0
0.5
1.0
1.5
2.0
obs
Gaussian kernel with band width 1.06

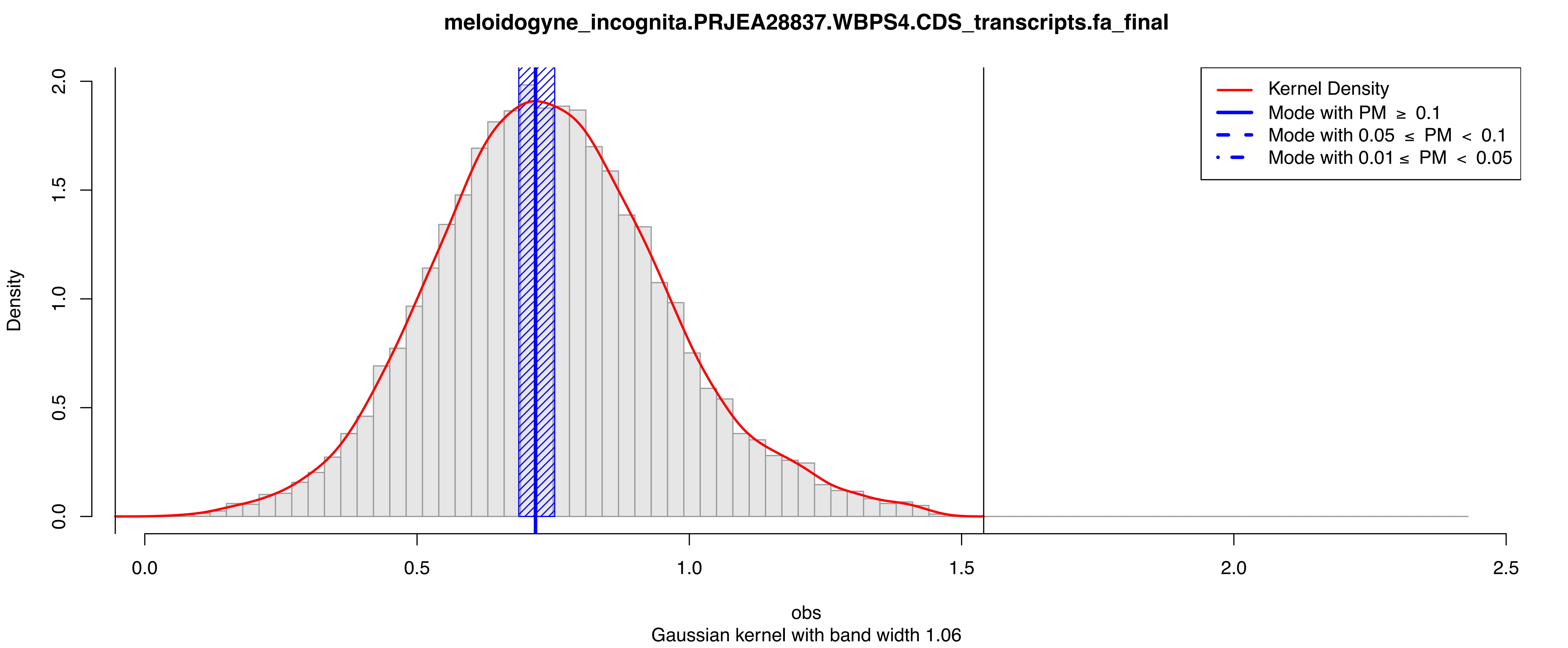

meloidogyne_incognita.PRJEA28837.WBPS4.CDS_transcripts.fa_final
Kernel Density
Mode with PM ≥ 0.1
Mode with 0.05 ≤ PM < 0.1
Mode with 0.01 ≤ PM < 0.05
Density
0.0
0.5
1.0
1.5
2.0
2.5
obs
Gaussian kernel with band width 1.06

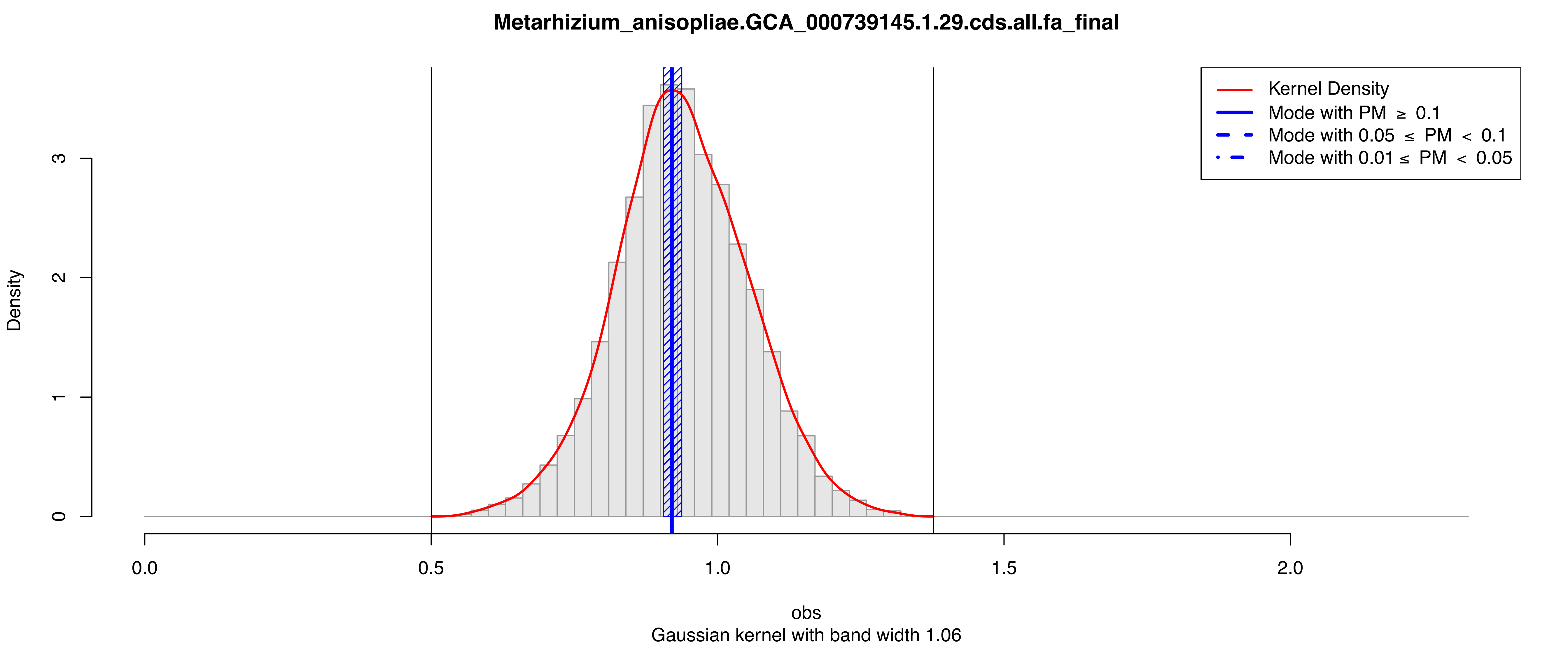

Metarhizium_anisopliae.GCA_000739145.1.29.cds.all.fa_final
Kernel Density
Mode with PM ≥ 0.1
Mode with 0.05 ≤ PM < 0.1
Mode with 0.01 ≤ PM < 0.05
Density
0
1
2
3
0.0
0.5
1.0
1.5
2.0
obs
Gaussian kernel with band width 1.06

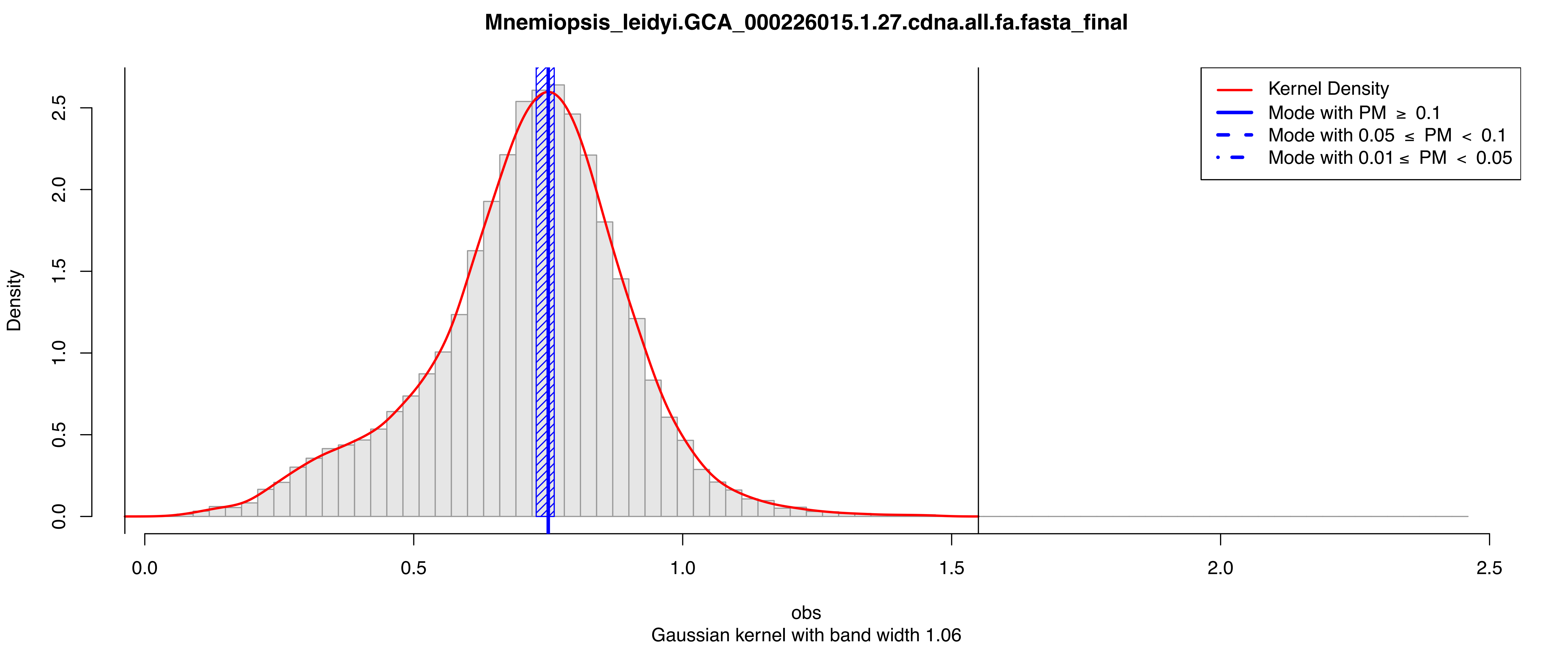
Mnemiopsis_leidyi.GCA_000226015.1.27.cdna.all.fa.fasta_final
Kernel Density
Mode with PM ≥ 0.1
Mode with 0.05 ≤ PM < 0.1
Mode with 0.01 ≤ PM < 0.05
Density
0.0
0.5
1.0
1.5
2.0
2.5
obs
Gaussian kernel with band width 1.06

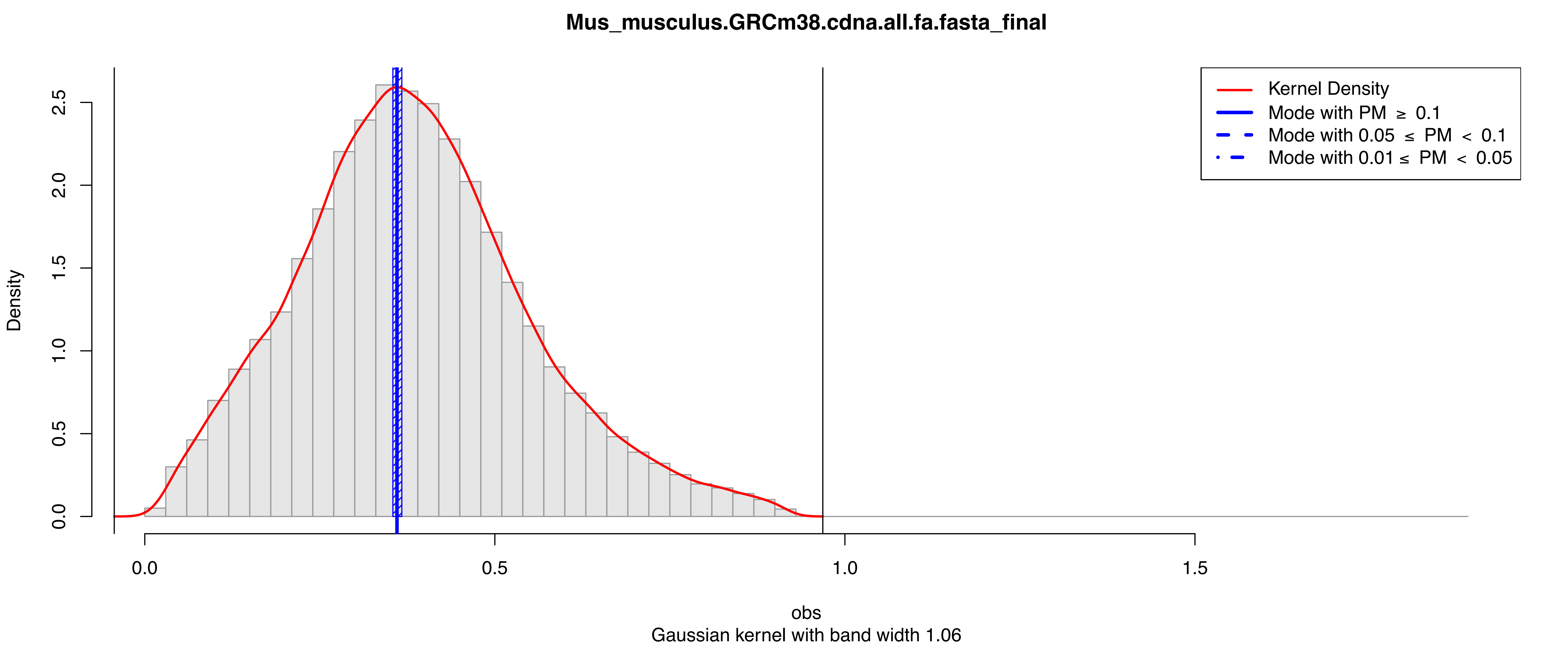
Mus_musculus.GRCm38.cdna.all.fa.fasta_final
Kernel Density
Mode with PM ≥ 0.1
Mode with 0.05 ≤ PM < 0.1
Mode with 0.01 ≤ PM < 0.05
Density
0.0
0.5
1.0
1.5
2.0
2.5
0.0
0.5
1.0
1.5
obs
Gaussian kernel with band width 1.06

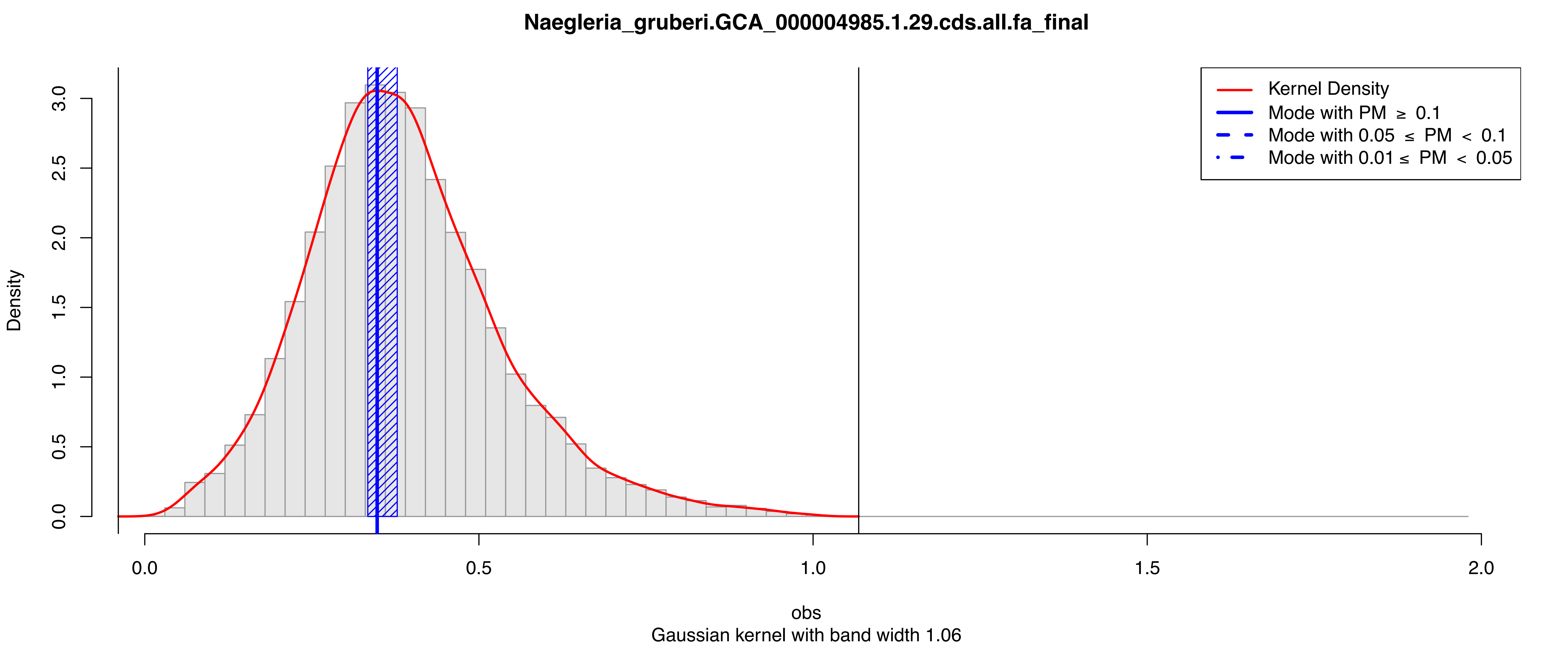

Naegleria_gruberi.GCA_000004985.1.29.cds.all.fa_final
Kernel Density
Mode with PM ≥ 0.1
Mode with 0.05 ≤ PM < 0.1
Mode with 0.01 ≤ PM < 0.05
Density
0.0
0.5
1.0
1.5
2.0
2.5
3.0
0.0
0.5
1.0
1.5
2.0
obs
Gaussian kernel with band width 1.06

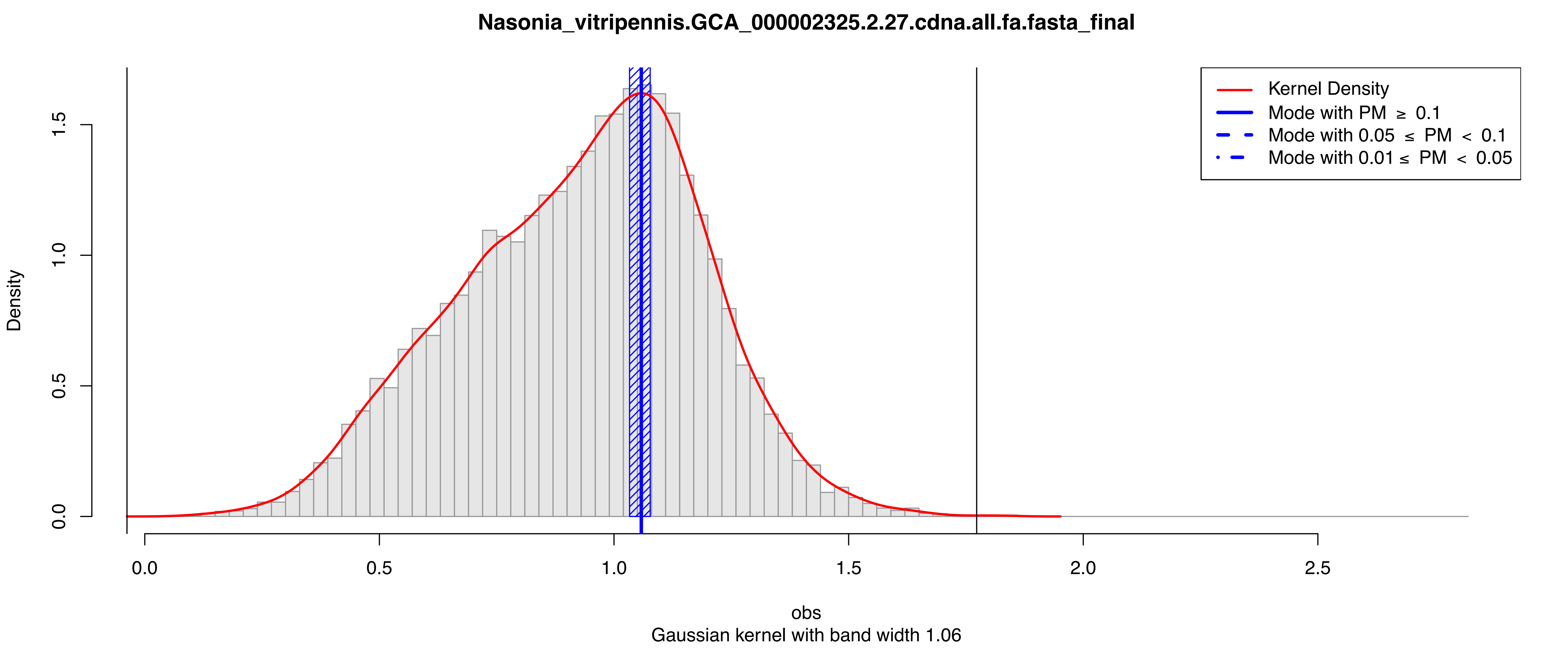
Nasonia_vitripennis.GCA_000002325.2.27.cdna.all.fa.fasta_final
Kernel Density
Mode with PM ≥ 0.1
Mode with 0.05 ≤ PM < 0.1
Mode with 0.01 ≤ PM < 0.05
Density
0.0
0.5
1.0
1.5
0.0
0.5
1.0
1.5
2.0
2.5
obs
Gaussian kernel with band width 1.06

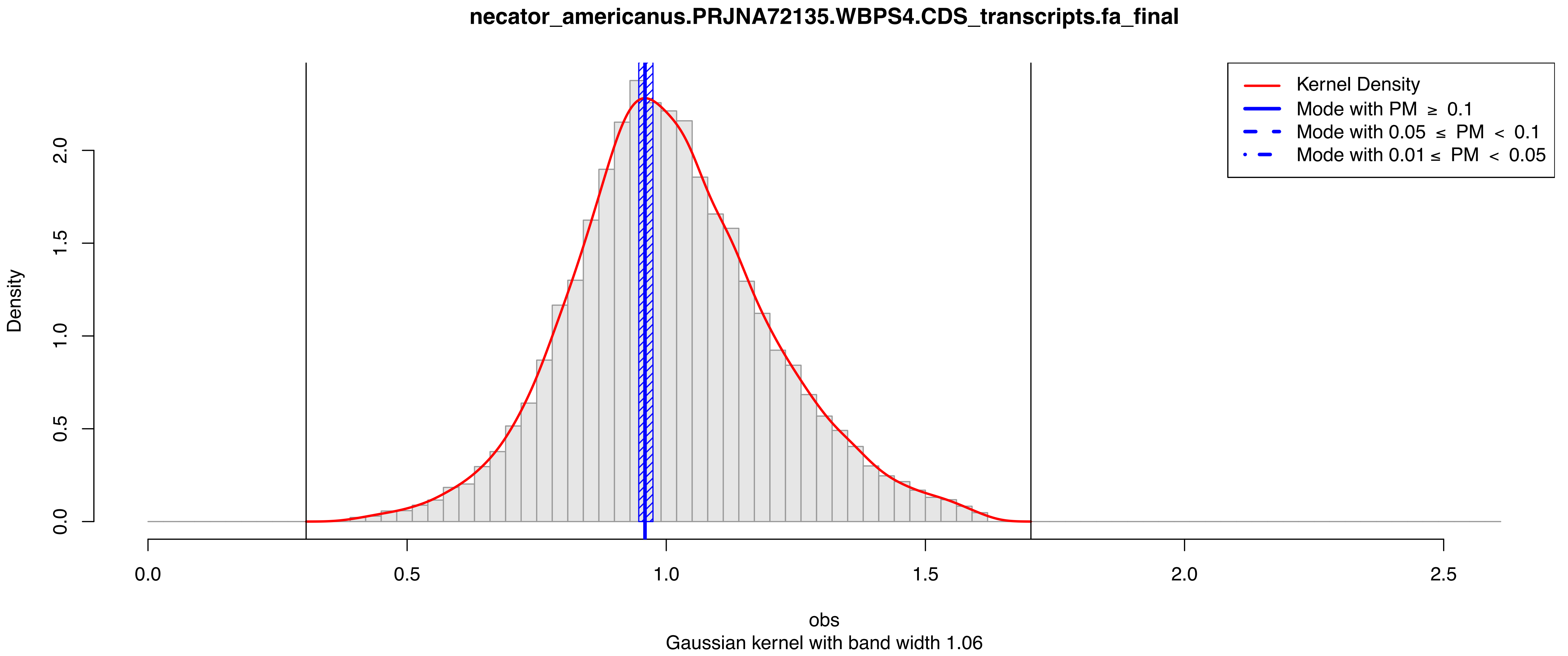

necator_americanus.PRJNA72135.WBPS4.CDS_transcripts.fa_final
Kernel Density
Mode with PM ≥ 0.1
Mode with 0.05 ≤ PM < 0.1
Mode with 0.01 ≤ PM < 0.05
Density
0.0
0.5
1.0
1.5
2.0
2.5
obs
Gaussian kernel with band width 1.06

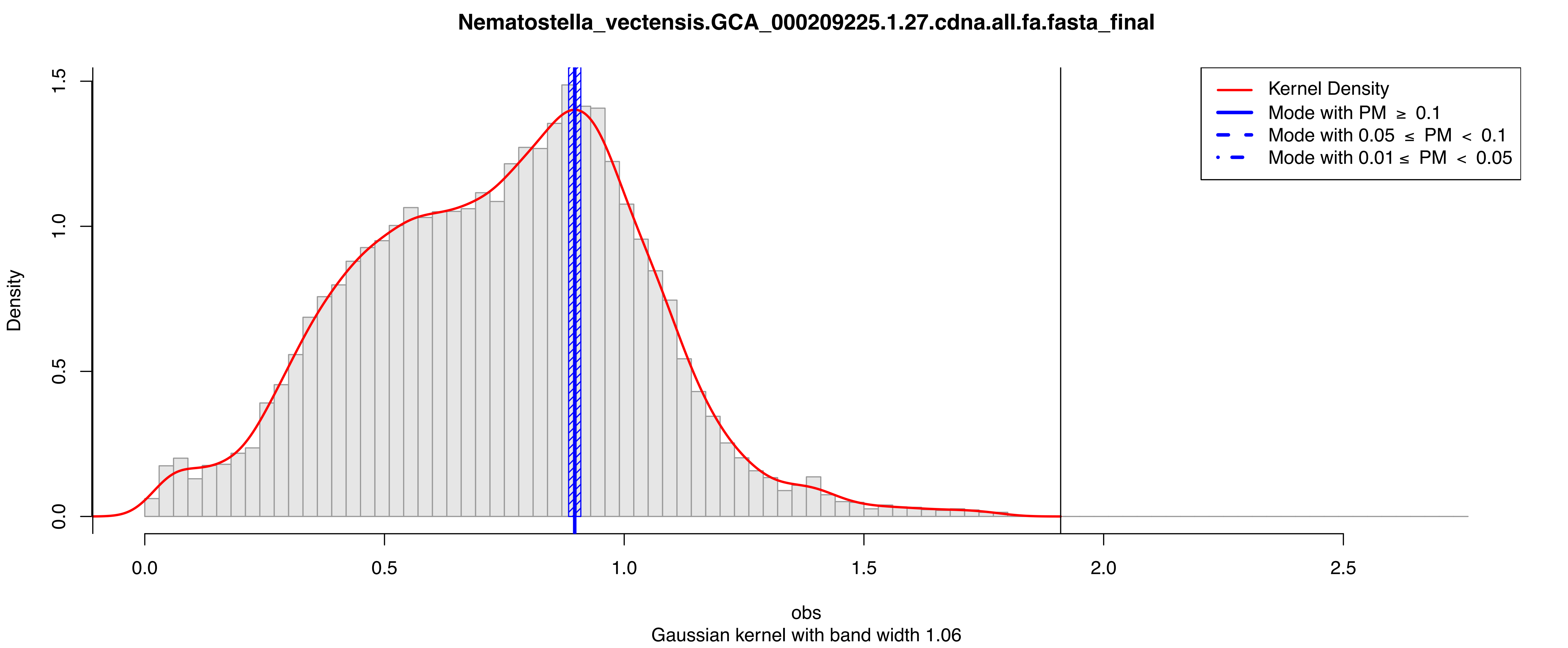

Nematostella_vectensis.GCA_000209225.1.27.cdna.all.fa.fasta_final
Kernel Density
Mode with PM ≥ 0.1
Mode with 0.05 ≤ PM < 0.1
Mode with 0.01 ≤ PM < 0.05
Density
0.0
0.5
1.0
1.5
0.0
0.5
1.0
1.5
2.0
2.5
obs
Gaussian kernel with band width 1.06

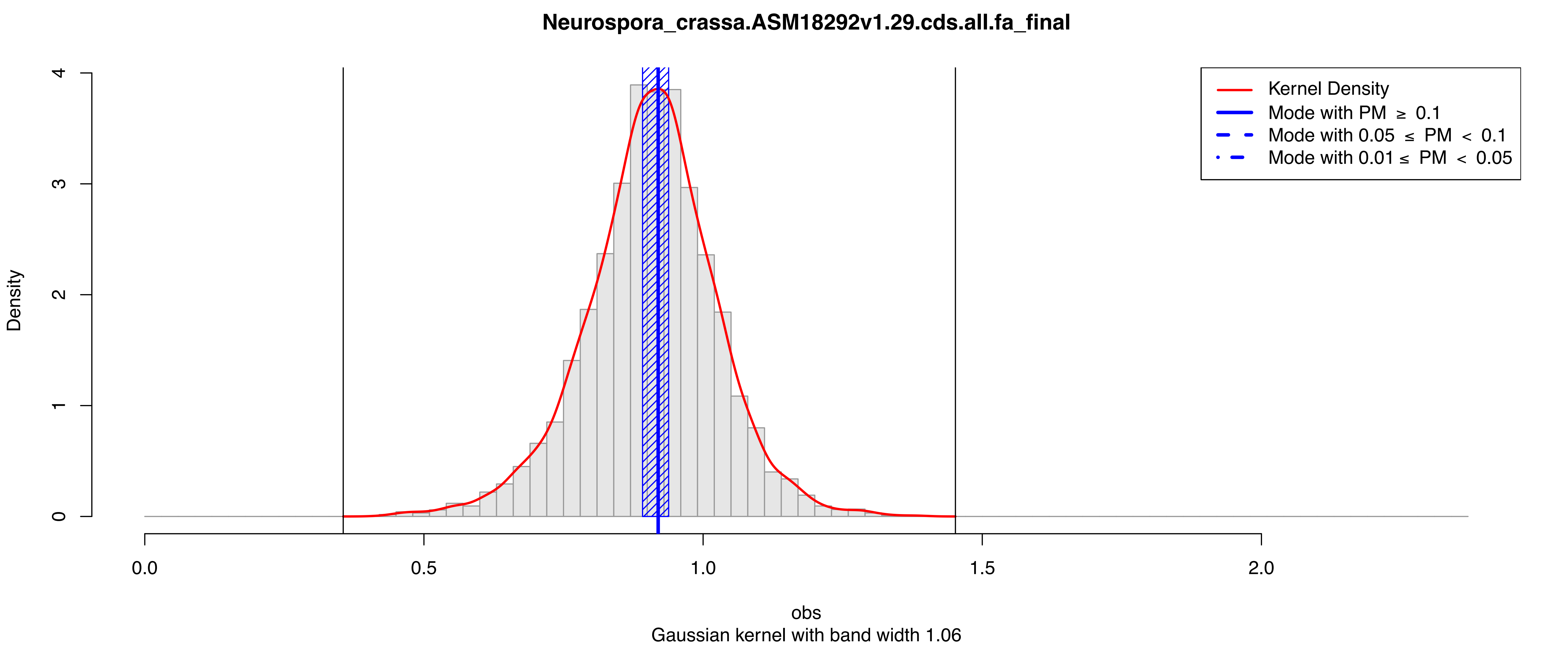

Neurospora_crassa.ASM18292v1.29.cds.all.fa_final
Kernel Density
Mode with PM ≥ 0.1
Mode with 0.05 ≤ PM < 0.1
Mode with 0.01 ≤ PM < 0.05
Density
0
1
2
3
4
0.0
0.5
1.0
1.5
2.0
obs
Gaussian kernel with band width 1.06

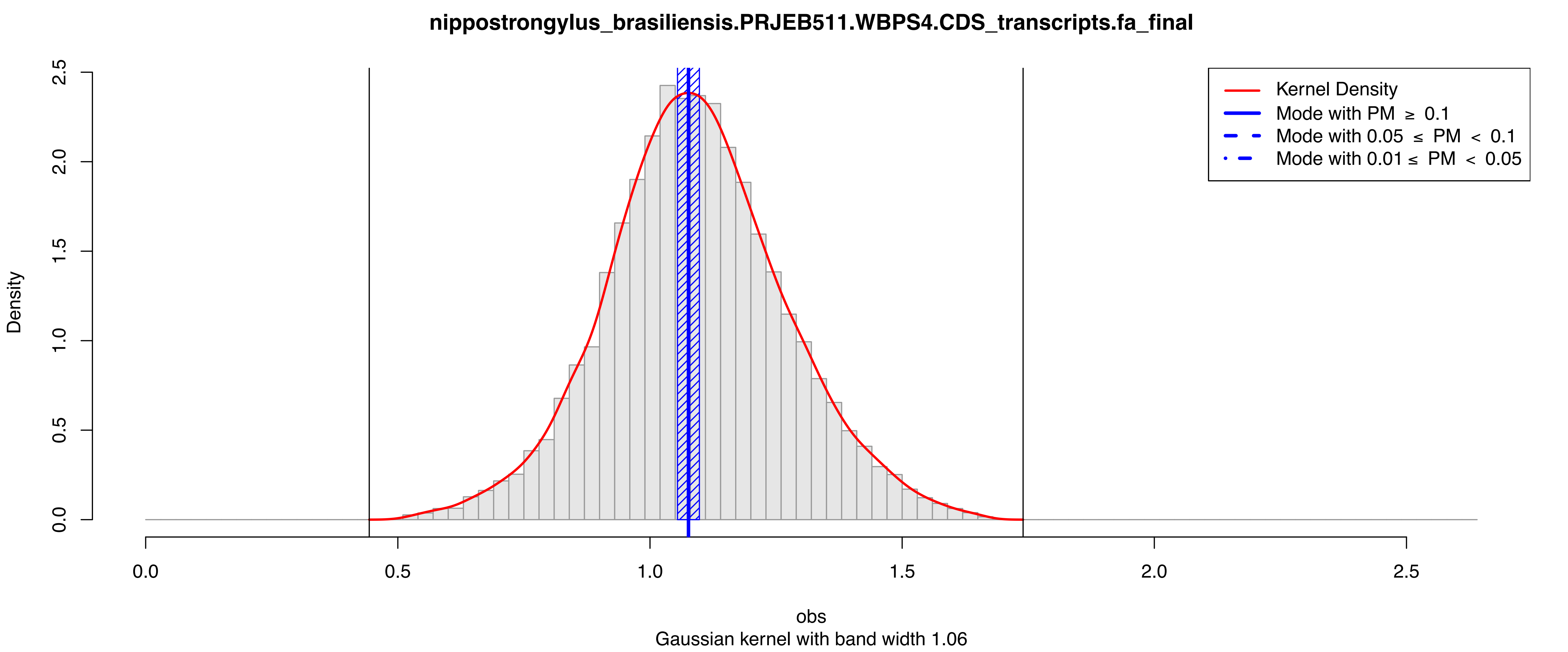

nippostrongylus_brasiliensis.PRJEB511.WBPS4.CDS_transcripts.fa_final
Kernel Density
Mode with PM ≥ 0.1
Mode with 0.05 ≤ PM < 0.1
Mode with 0.01 ≤ PM < 0.05
Density
0.0
0.5
1.0
1.5
2.0
2.5
obs
Gaussian kernel with band width 1.06

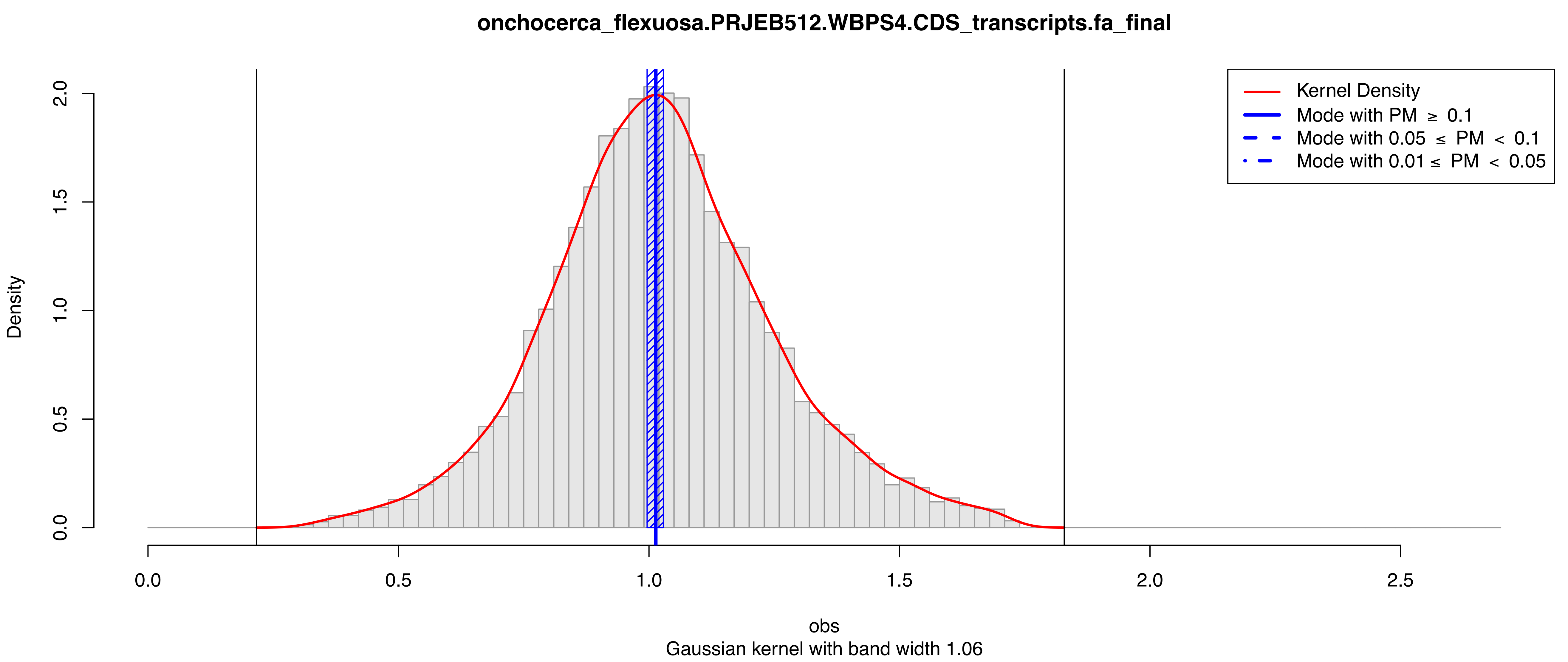

onchocerca_flexuosa.PRJEB512.WBPS4.CDS_transcripts.fa_final
Kernel Density
Mode with PM ≥ 0.1
Mode with 0.05 ≤ PM < 0.1
Mode with 0.01 ≤ PM < 0.05
Density
0.0
0.5
1.0
1.5
2.0
2.5
obs
Gaussian kernel with band width 1.06

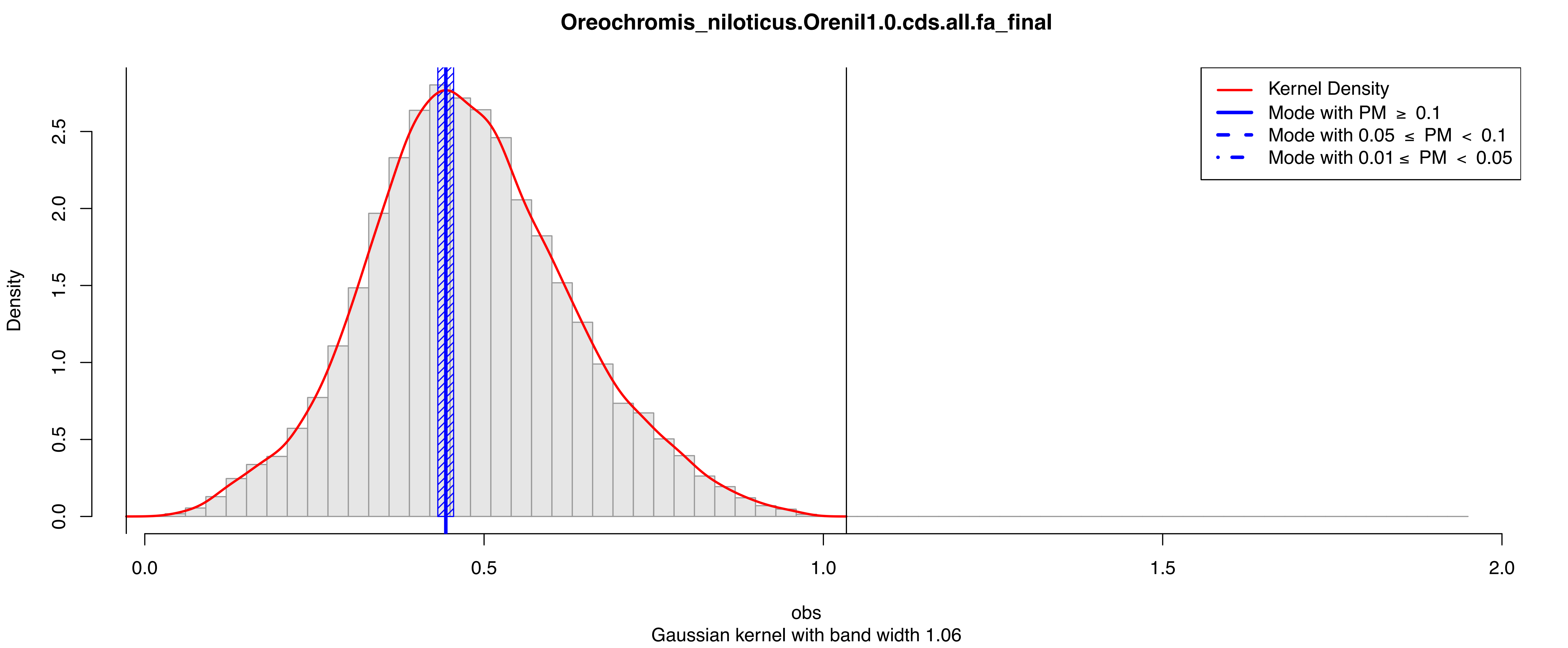

Oreochromis_niloticus.Orenil1.0.cds.all.fa_final
Kernel Density
Mode with PM ≥ 0.1
Mode with 0.05 ≤ PM < 0.1
Mode with 0.01 ≤ PM < 0.05
Density
0.0
0.5
1.0
1.5
2.0
2.5
0.0
0.5
1.0
1.5
2.0
obs
Gaussian kernel with band width 1.06

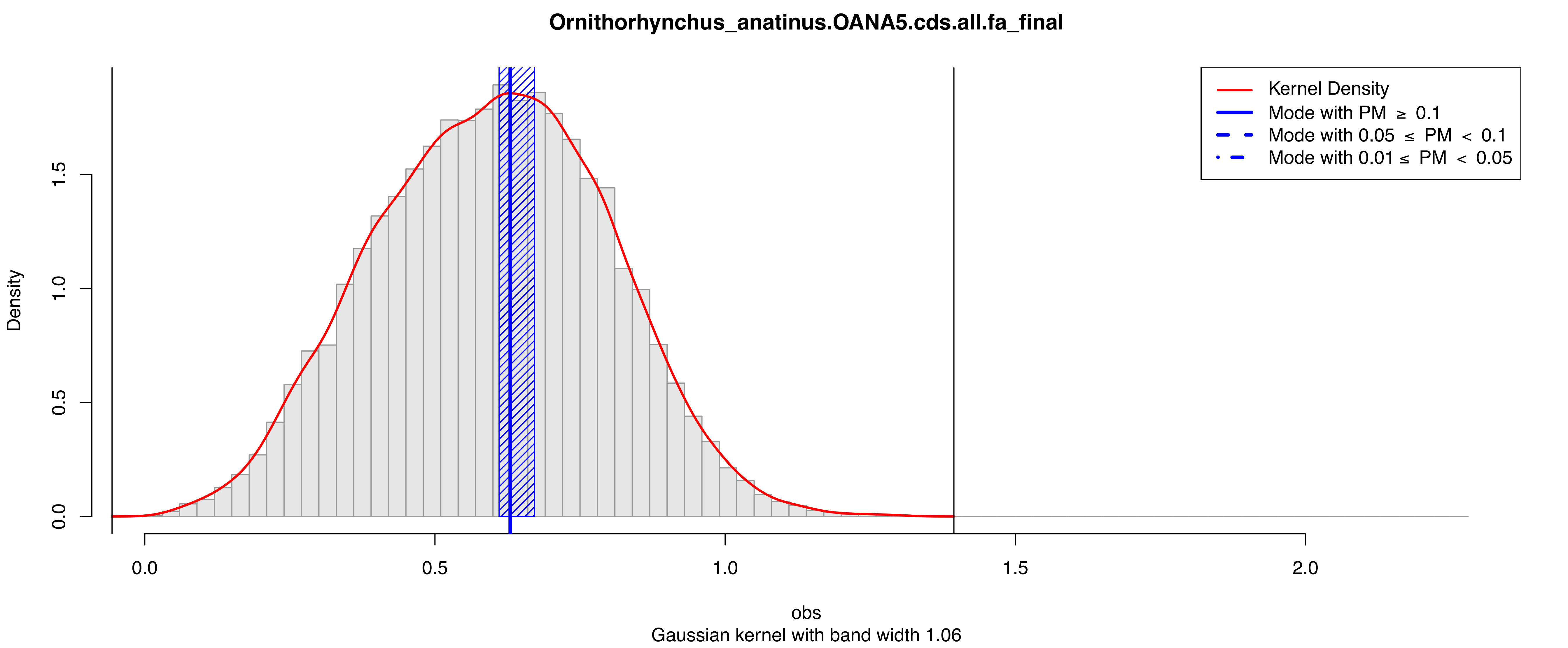

Ornithorhynchus_anatinus.OANA5.cds.all.fa_final
Kernel Density
Mode with PM ≥ 0.1
Mode with 0.05 ≤ PM < 0.1
Mode with 0.01 ≤ PM < 0.05
Density
0.0
0.5
1.0
1.5
0.0
0.5
1.0
1.5
2.0
obs
Gaussian kernel with band width 1.06

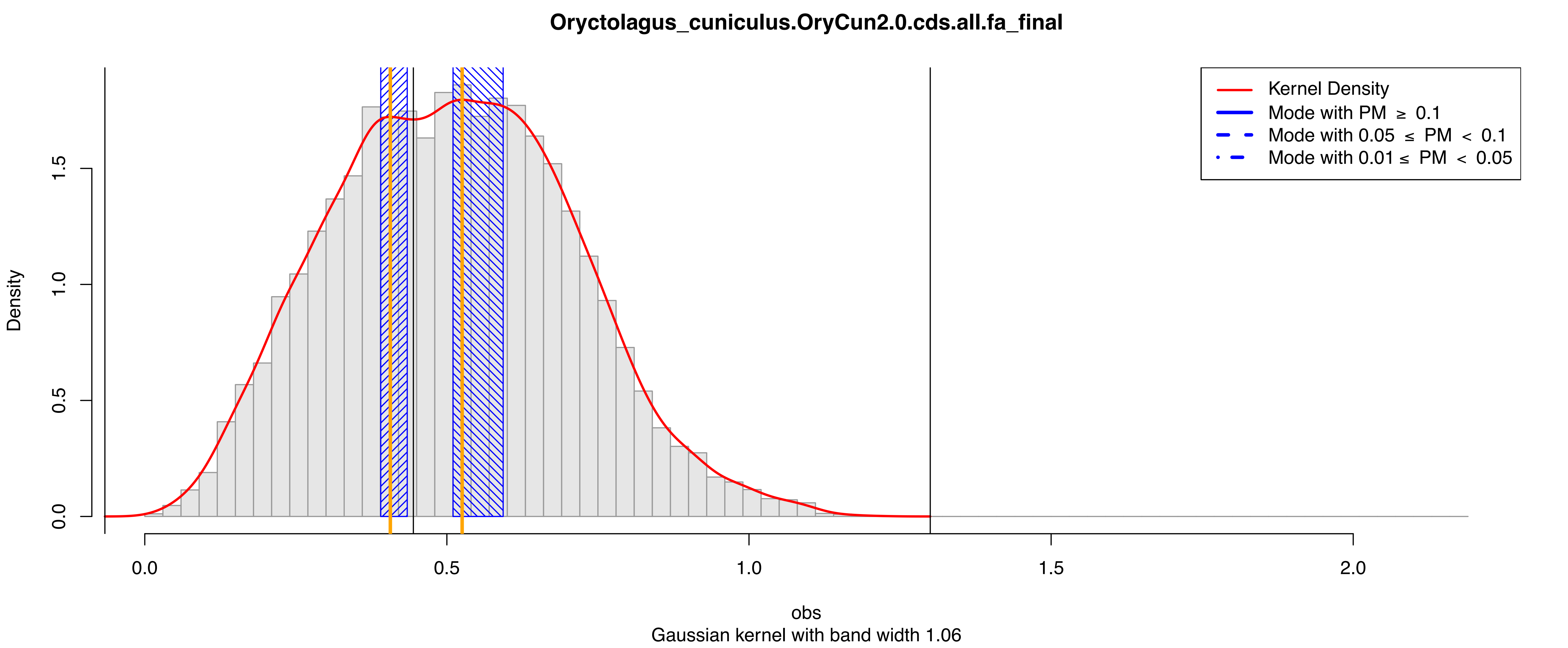

Oryctolagus_cuniculus.OryCun2.0.cds.all.fa_final
Kernel Density
Mode with PM ≥ 0.1
Mode with 0.05 ≤ PM < 0.1
Mode with 0.01 ≤ PM < 0.05
Density
0.0
0.5
1.0
1.5
2.0
obs
Gaussian kernel with band width 1.06

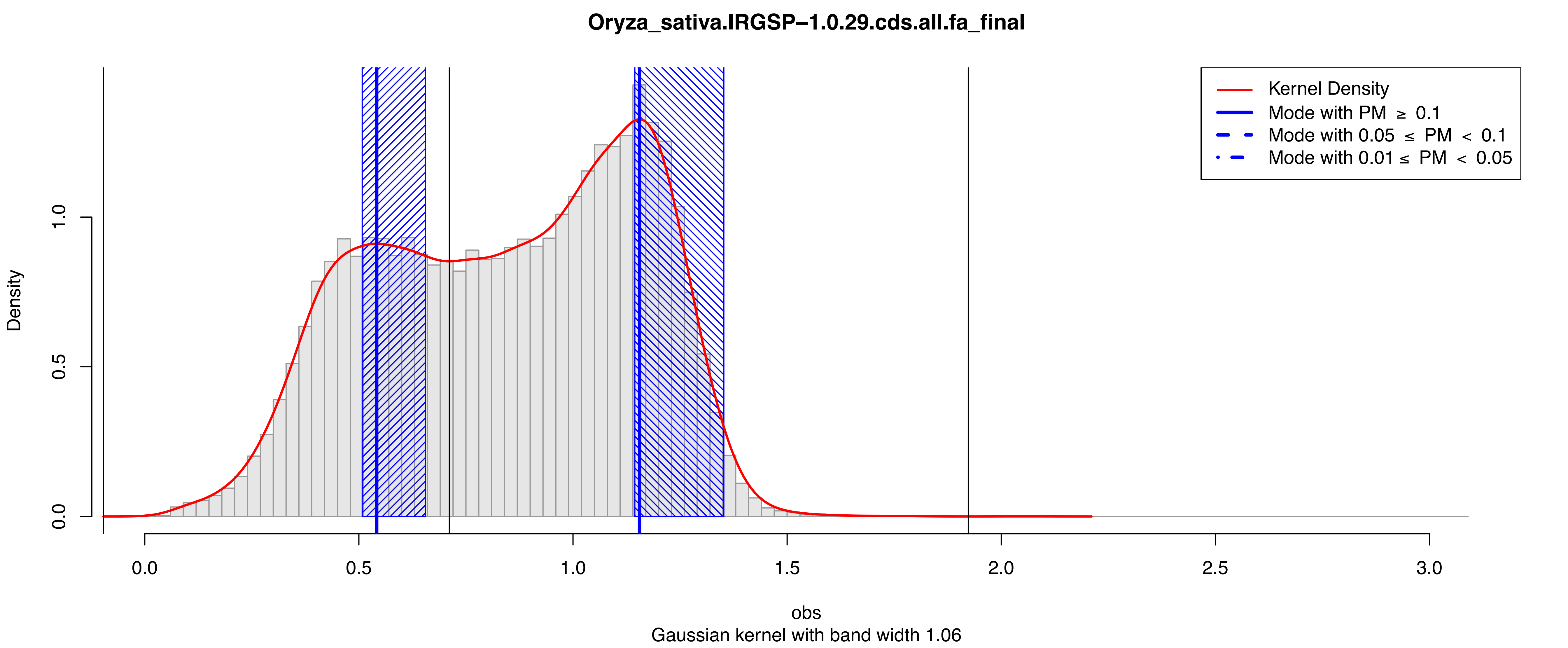

Oryza_sativa.IRGSP-1.0.29.cds.all.fa_final
Kernel Density
Mode with PM ≥ 0.1
Mode with 0.05 ≤ PM < 0.1
Mode with 0.01 ≤ PM < 0.05
Density
0.0
0.5
1.0
0.0
0.5
1.0
1.5
2.0
2.5
3.0
obs
Gaussian kernel with band width 1.06

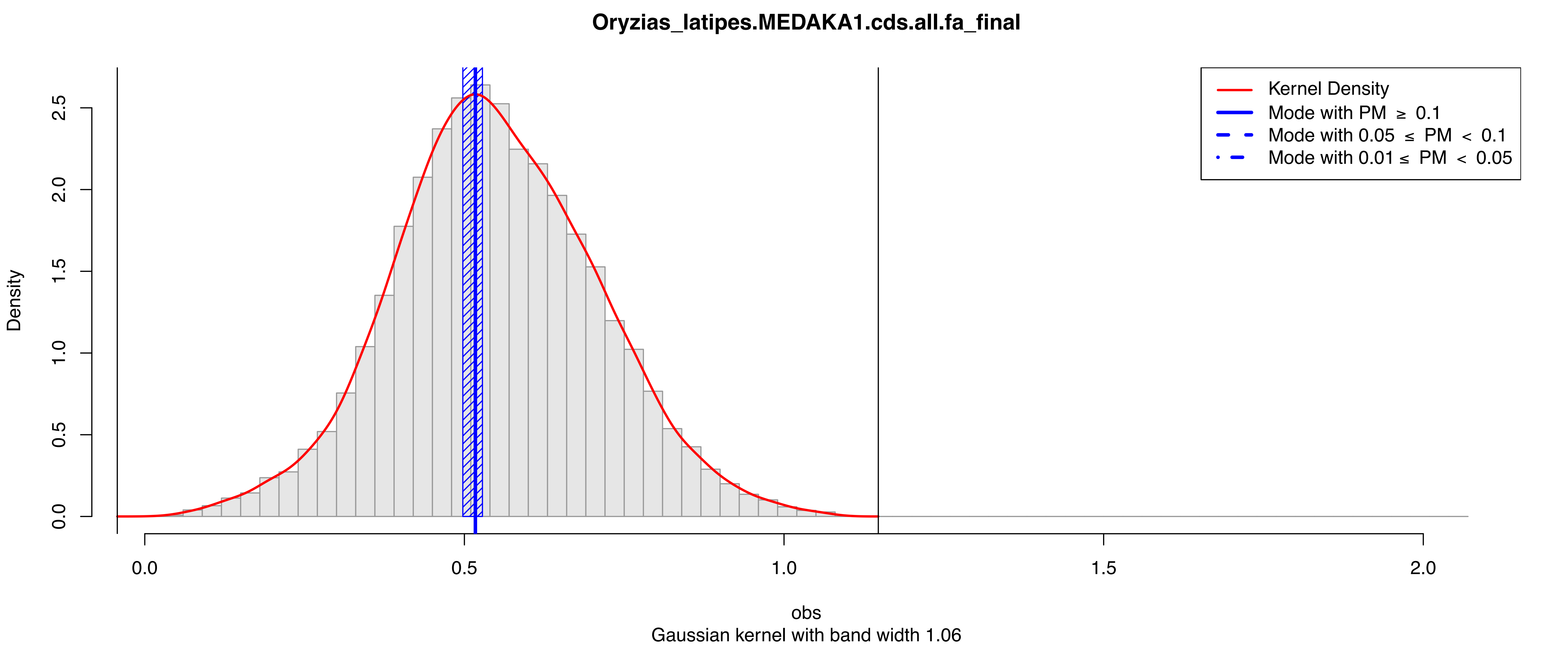
Oryzias_latipes.MEDAKA1.cds.all.fa_final
Kernel Density
Mode with PM ≥ 0.1
Mode with 0.05 ≤ PM < 0.1
Mode with 0.01 ≤ PM < 0.05
Density
0.0
0.5
1.0
1.5
2.0
2.5
0.0
0.5
1.0
1.5
2.0
obs
Gaussian kernel with band width 1.06

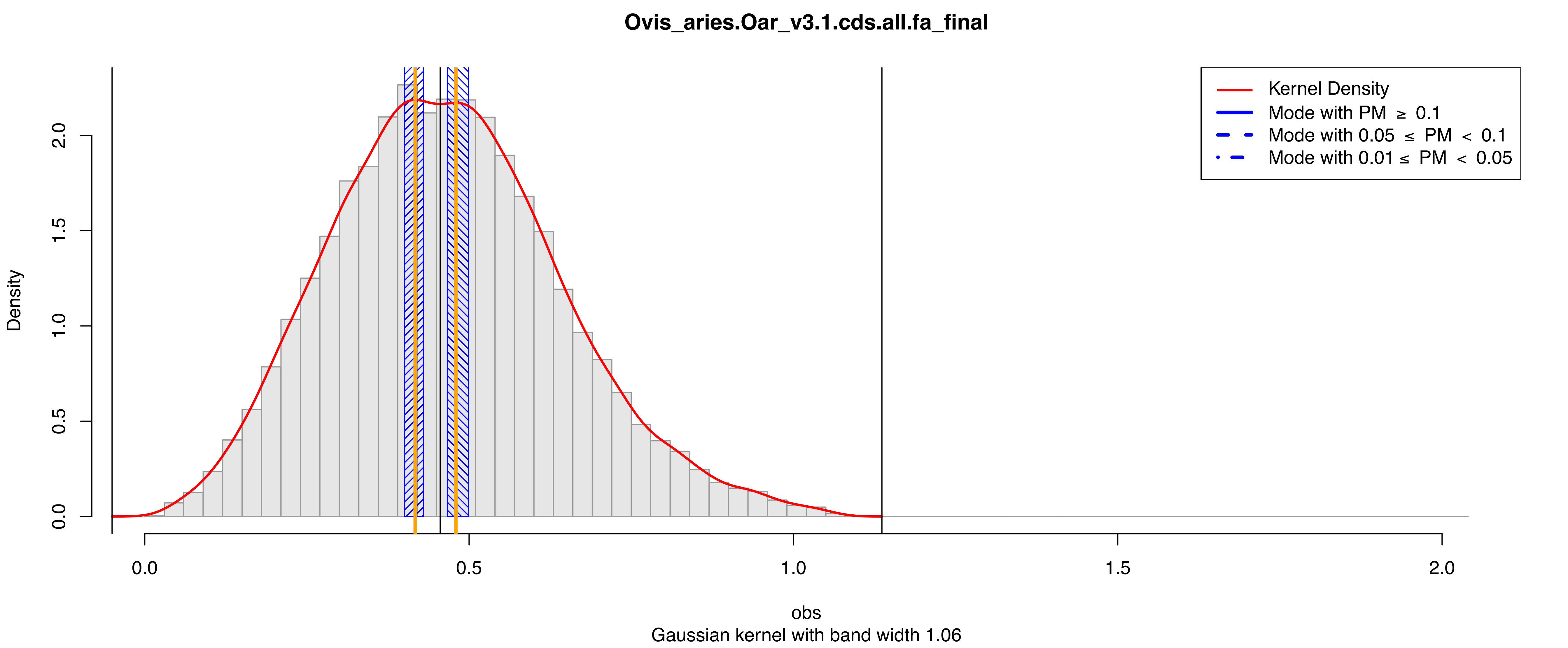

Ovis_aries.Oar_v3.1.cds.all.fa_final
Kernel Density
Mode with PM ≥ 0.1
Mode with 0.05 ≤ PM < 0.1
Mode with 0.01 ≤ PM < 0.05
Density
0.0
0.5
1.0
1.5
2.0
0.0
0.5
1.0
1.5
2.0
obs
Gaussian kernel with band width 1.06

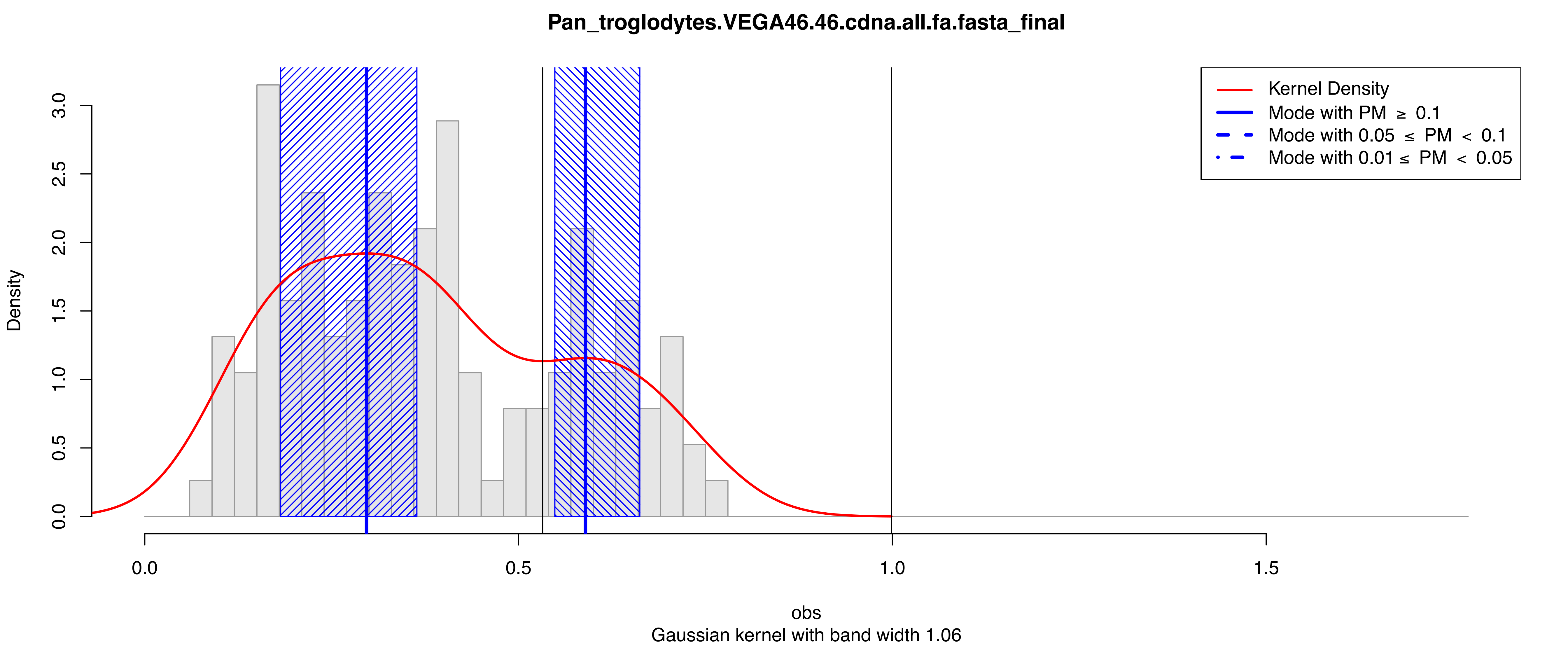

Pan_troglodytes.VEGA46.46.cdna.all.fa.fasta_final
Density
0.0
0.5
1.0
1.5
2.0
2.5
3.0
Kernel Density
Mode with PM ≥ 0.1
Mode with 0.05 ≤ PM < 0.1
Mode with 0.01 ≤ PM < 0.05
0.0
0.5
1.0
1.5
obs
Gaussian kernel with band width 1.06

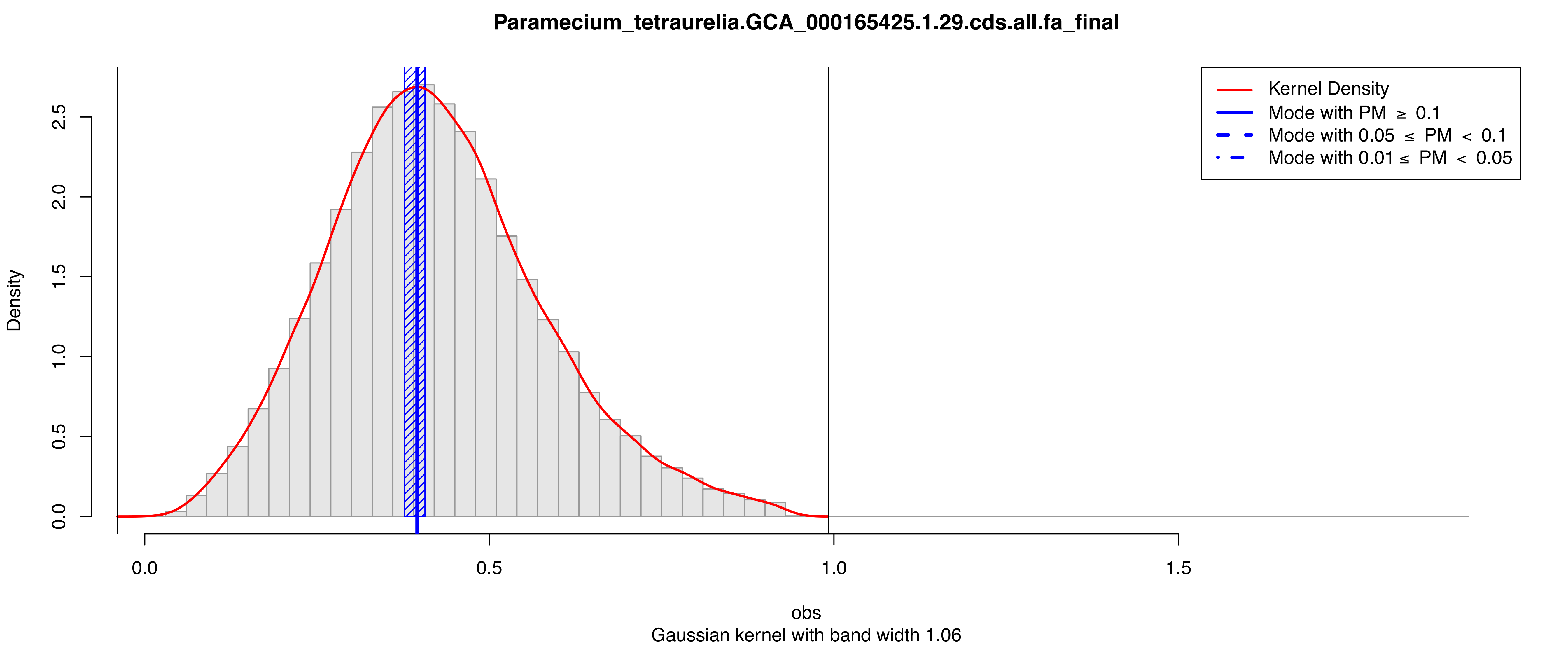

Paramecium_tetraurelia.GCA_000165425.1.29.cds.all.fa_final
Kernel Density
Mode with PM ≥ 0.1
Mode with 0.05 ≤ PM < 0.1
Mode with 0.01 ≤ PM < 0.05
Density
0.0
0.5
1.0
1.5
2.0
2.5
0.0
0.5
1.0
1.5
obs
Gaussian kernel with band width 1.06

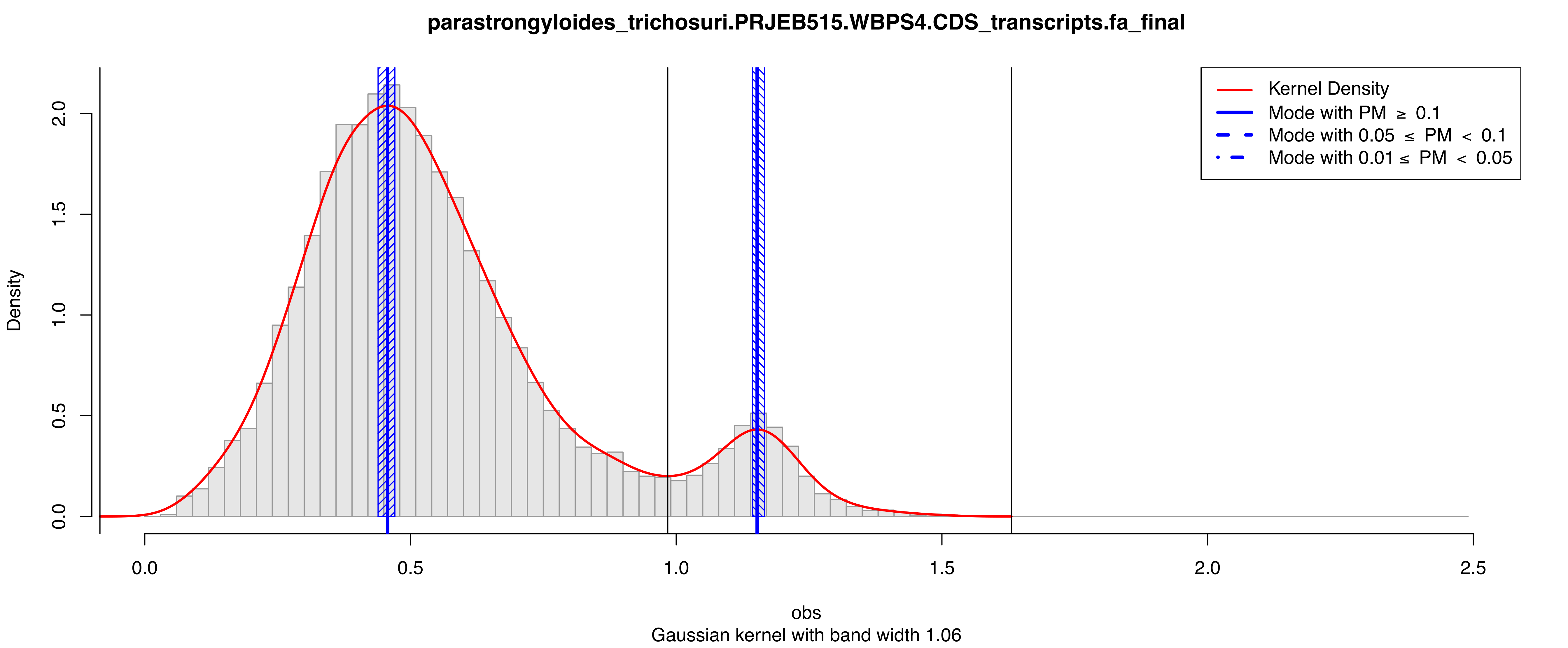
parastrongyloides_trichosuri.PRJEB515.WBPS4.CDS_transcripts.fa_final
Kernel Density
Mode with PM ≥ 0.1
Mode with 0.05 ≤ PM < 0.1
Mode with 0.01 ≤ PM < 0.05
Density
0.0
0.5
1.0
1.5
2.0
2.5
obs
Gaussian kernel with band width 1.06

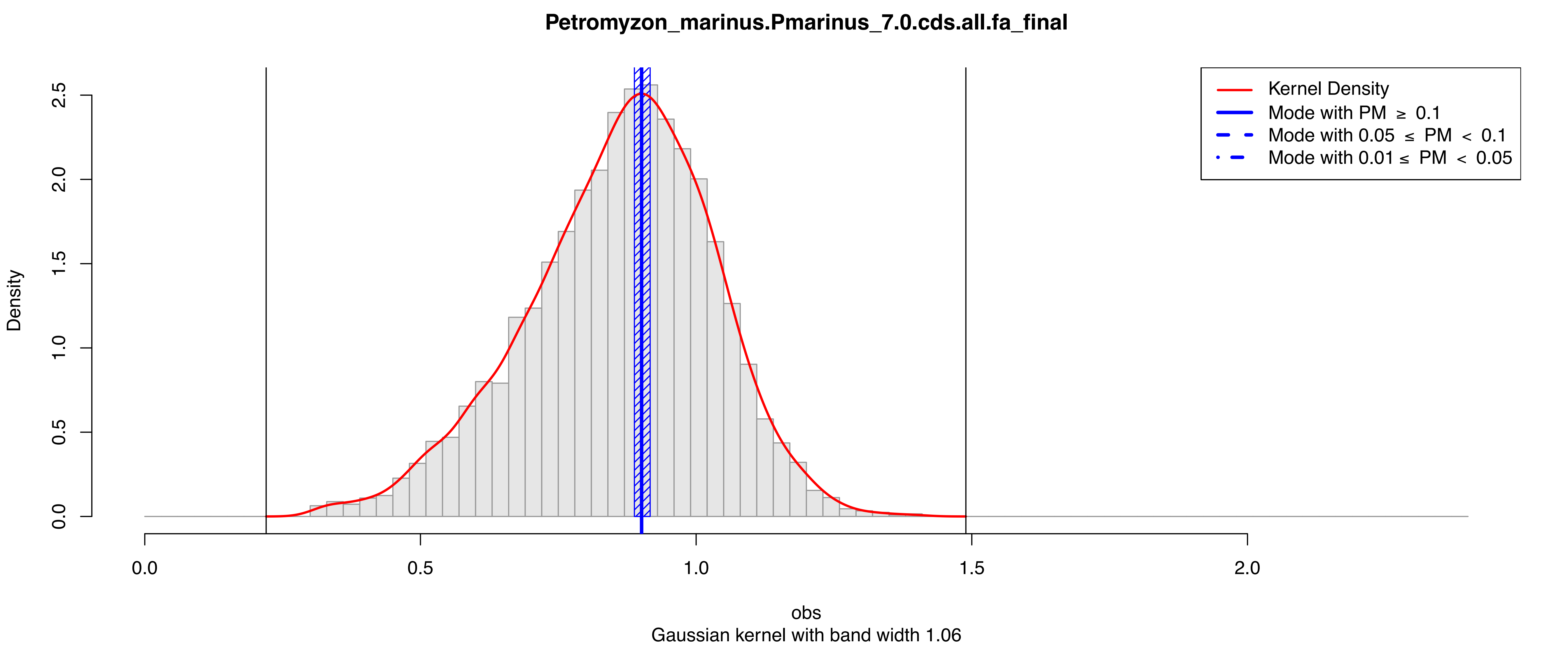

Petromyzon_marinus.Pmarinus_7.0.cds.all.fa_final
Kernel Density
Mode with PM ≥ 0.1
Mode with 0.05 ≤ PM < 0.1
Mode with 0.01 ≤ PM < 0.05
Density
0.0
0.5
1.0
1.5
2.0
2.5
obs
Gaussian kernel with band width 1.06

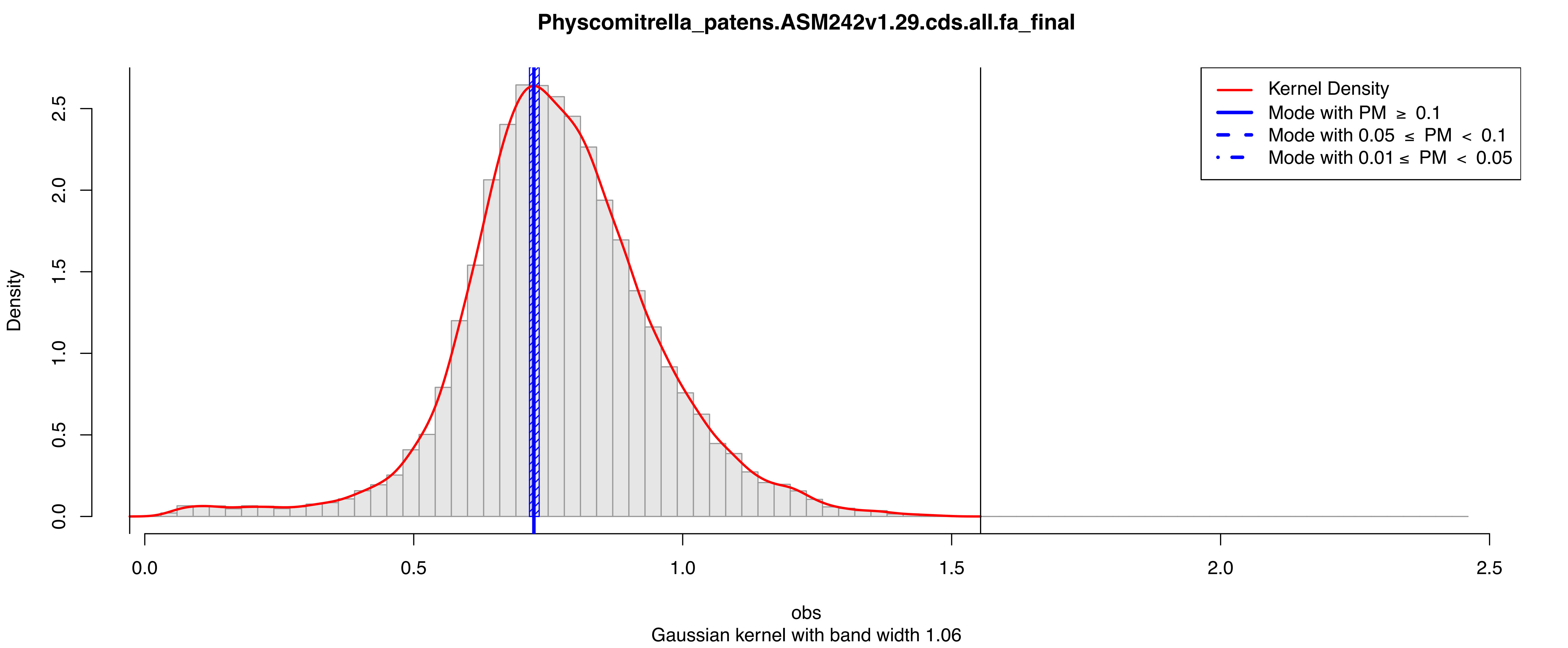
Physcomitrella_patens.ASM242v1.29.cds.all.fa_final
Kernel Density
Mode with PM ≥ 0.1
Mode with 0.05 ≤ PM < 0.1
Mode with 0.01 ≤ PM < 0.05
Density
0.0
0.5
1.0
1.5
2.0
2.5
obs
Gaussian kernel with band width 1.06

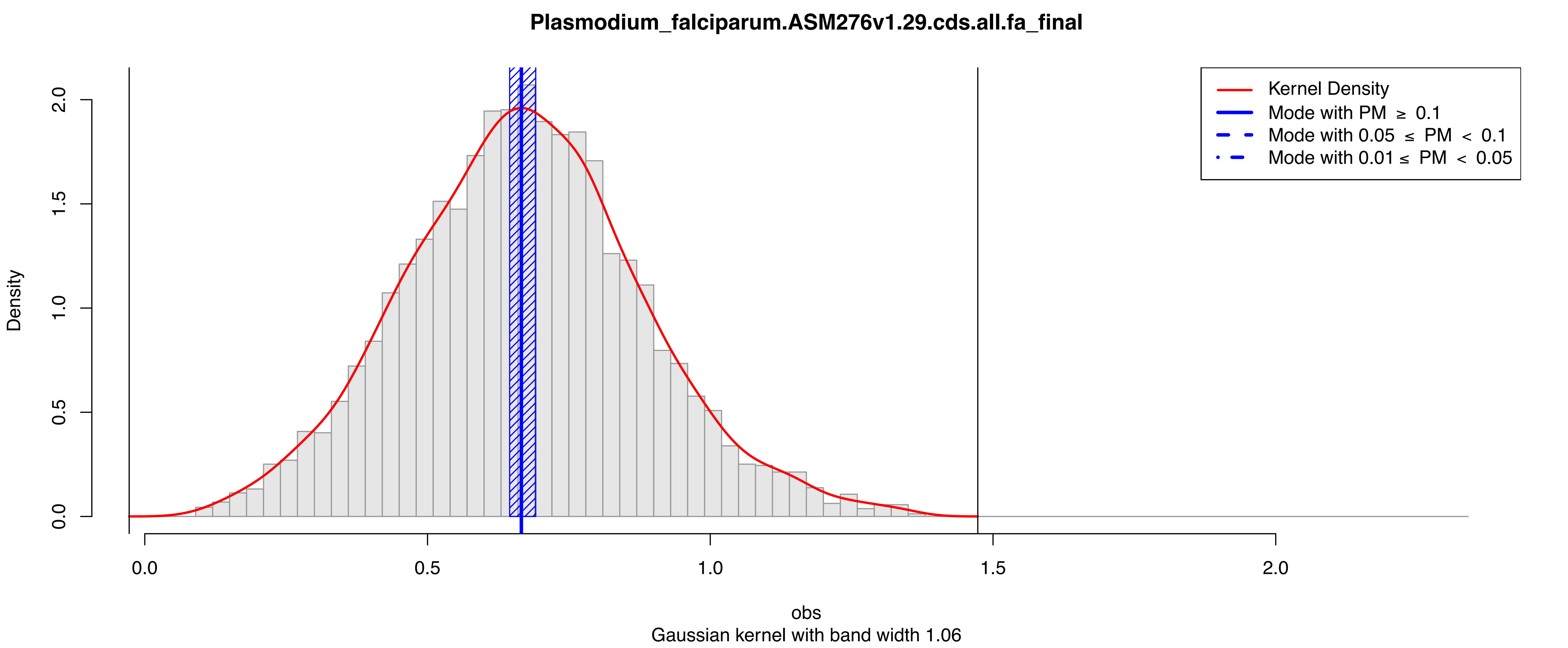

Plasmodium_falciparum.ASM276v1.29.cds.all.fa_final
Density
0.0
0.5
1.0
1.5
2.0
Kernel Density
Mode with PM ≥ 0.1
Mode with 0.05 ≤ PM < 0.1
Mode with 0.01 ≤ PM < 0.05
obs
Gaussian kernel with band width 1.06

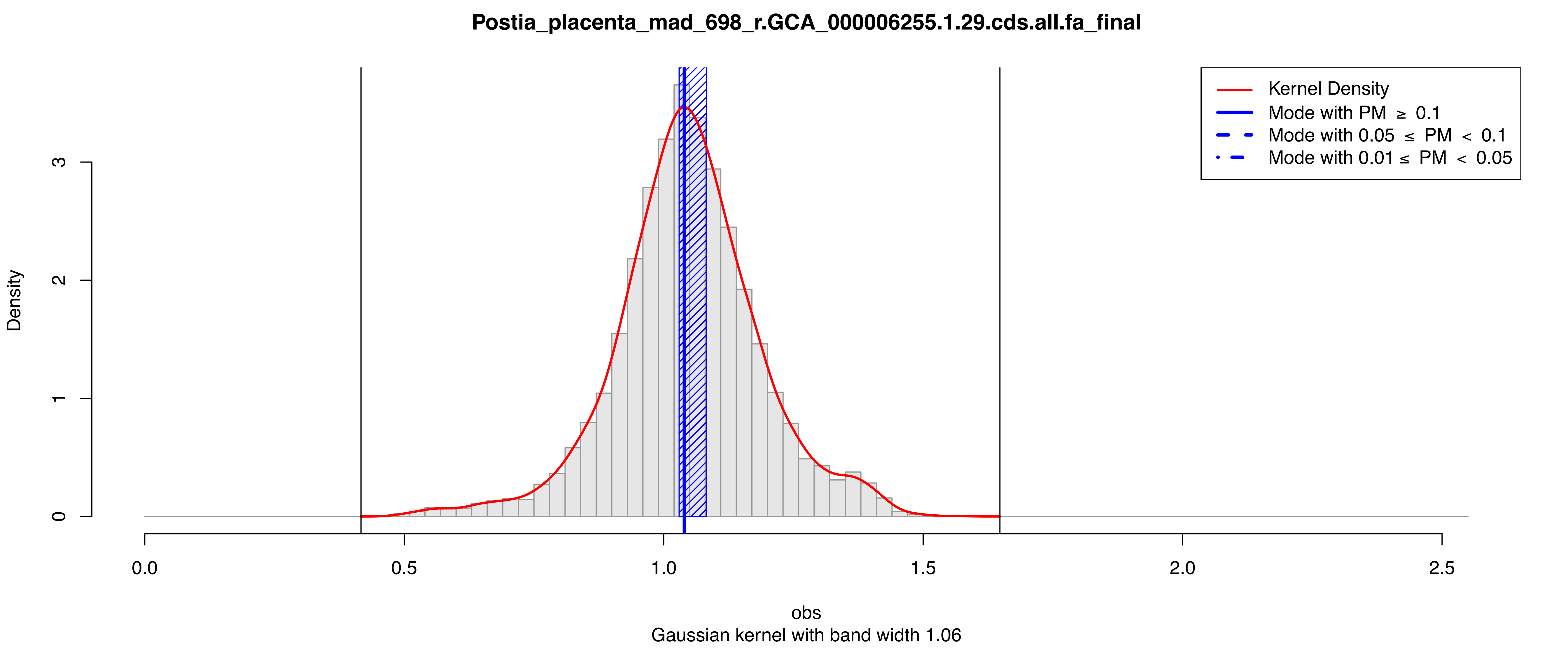

Postia_placenta_mad_698_r.GCA_000006255.1.29.cds.all.fa_final
Kernel Density
Mode with PM ≥ 0.1
Mode with 0.05 ≤ PM < 0.1
Mode with 0.01 ≤ PM < 0.05
Density
0
1
2
3
0.0
0.5
1.0
1.5
2.0
2.5
obs
Gaussian kernel with band width 1.06

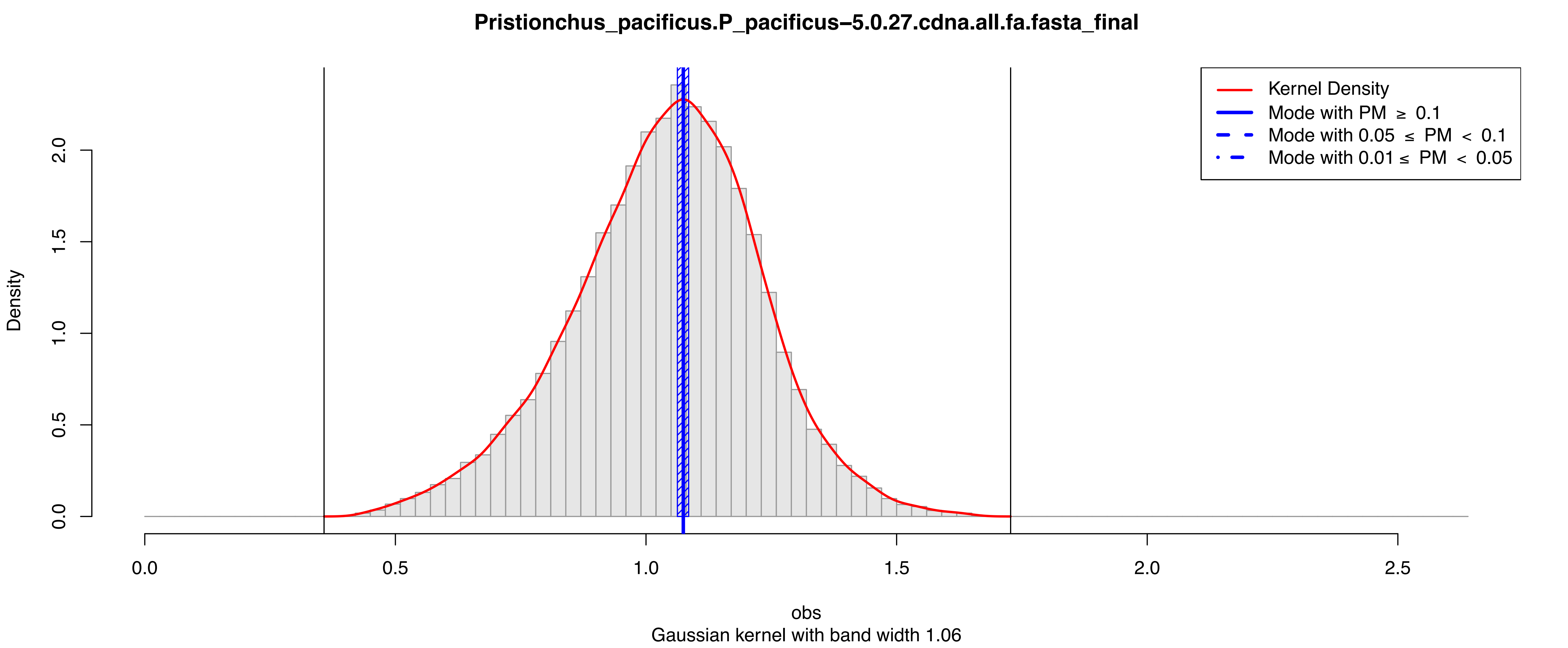

Pristionchus_pacificus.P_pacificus−5.0.27.cdna.all.fa.fasta_final
Kernel Density
Mode with PM ≥ 0.1
Mode with 0.05 ≤ PM < 0.1
Mode with 0.01 ≤ PM < 0.05
Density
0.0
0.5
1.0
1.5
2.0
0.0
0.5
1.0
1.5
2.0
2.5
obs
Gaussian kernel with band width 1.06

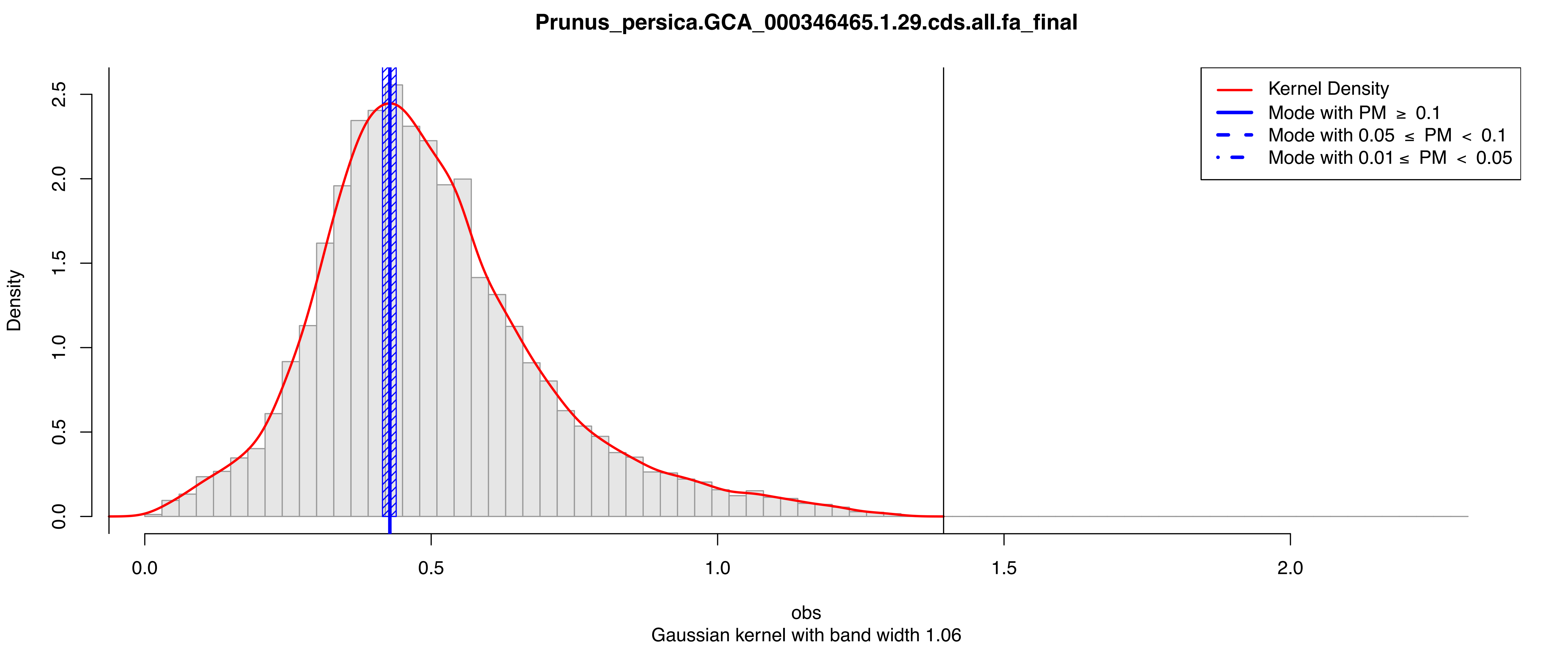

Prunus_persica.GCA_000346465.1.29.cds.all.fa_final
Kernel Density
Mode with PM ≥ 0.1
Mode with 0.05 ≤ PM < 0.1
Mode with 0.01 ≤ PM < 0.05
Density
0.0
0.5
1.0
1.5
2.0
2.5
0.0
0.5
1.0
1.5
2.0
obs
Gaussian kernel with band width 1.06

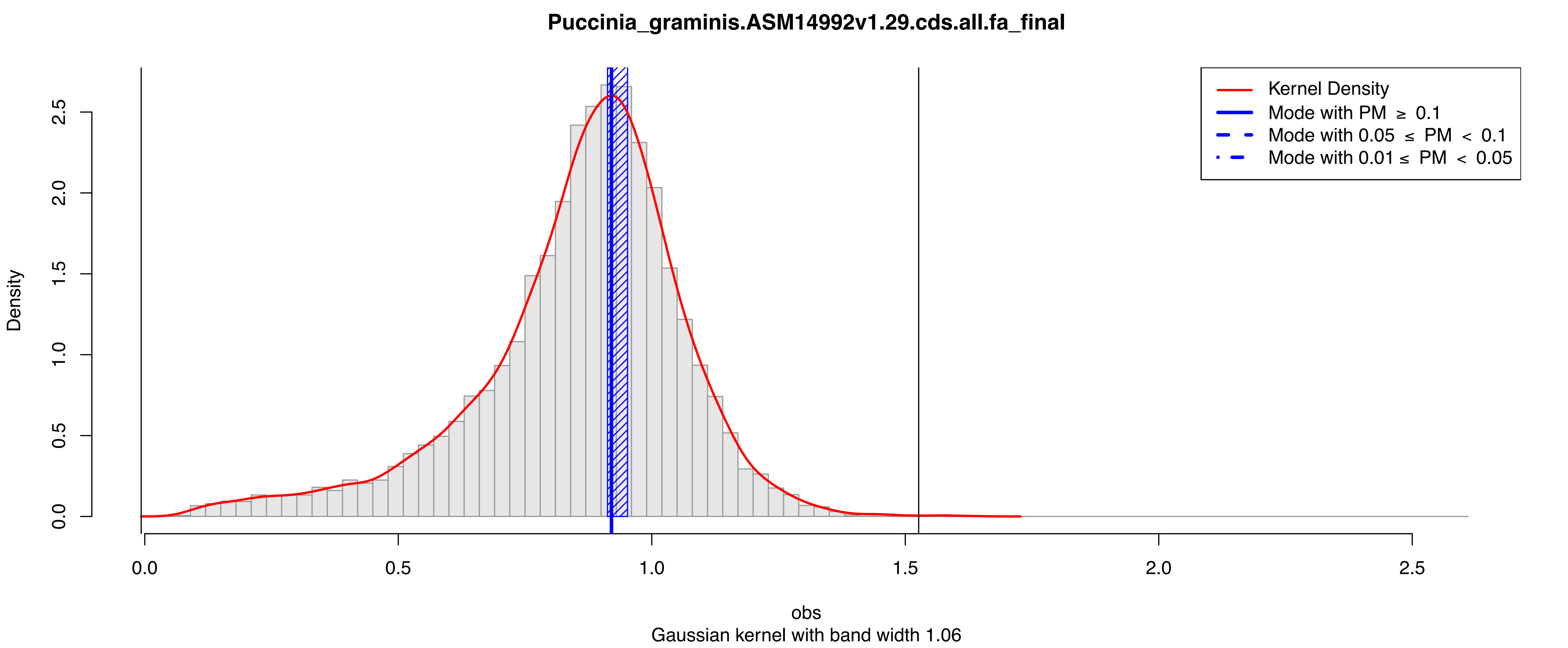

Puccinia_graminis.ASM14992v1.29.cds.all.fa_final
Kernel Density
Mode with PM ≥ 0.1
Mode with 0.05 ≤ PM < 0.1
Mode with 0.01 ≤ PM < 0.05
Density
0.0
0.5
1.0
1.5
2.0
2.5
obs
Gaussian kernel with band width 1.06

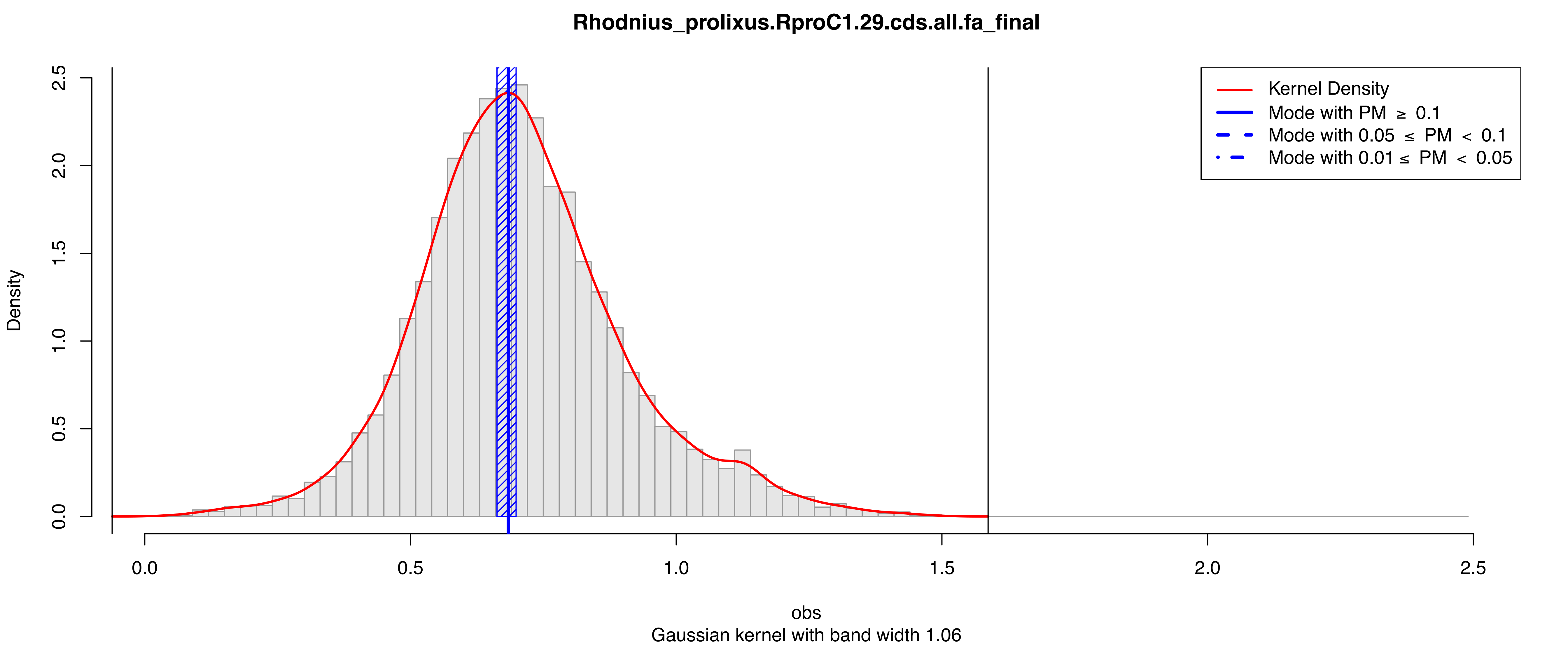

Rhodnius_prolixus.RproC1.29.cds.all.fa_final
Kernel Density
Mode with PM ≥ 0.1
Mode with 0.05 ≤ PM < 0.1
Mode with 0.01 ≤ PM < 0.05
Density
0.0
0.5
1.0
1.5
2.0
2.5
0.0
0.5
1.0
1.5
2.0
2.5
obs
Gaussian kernel with band width 1.06

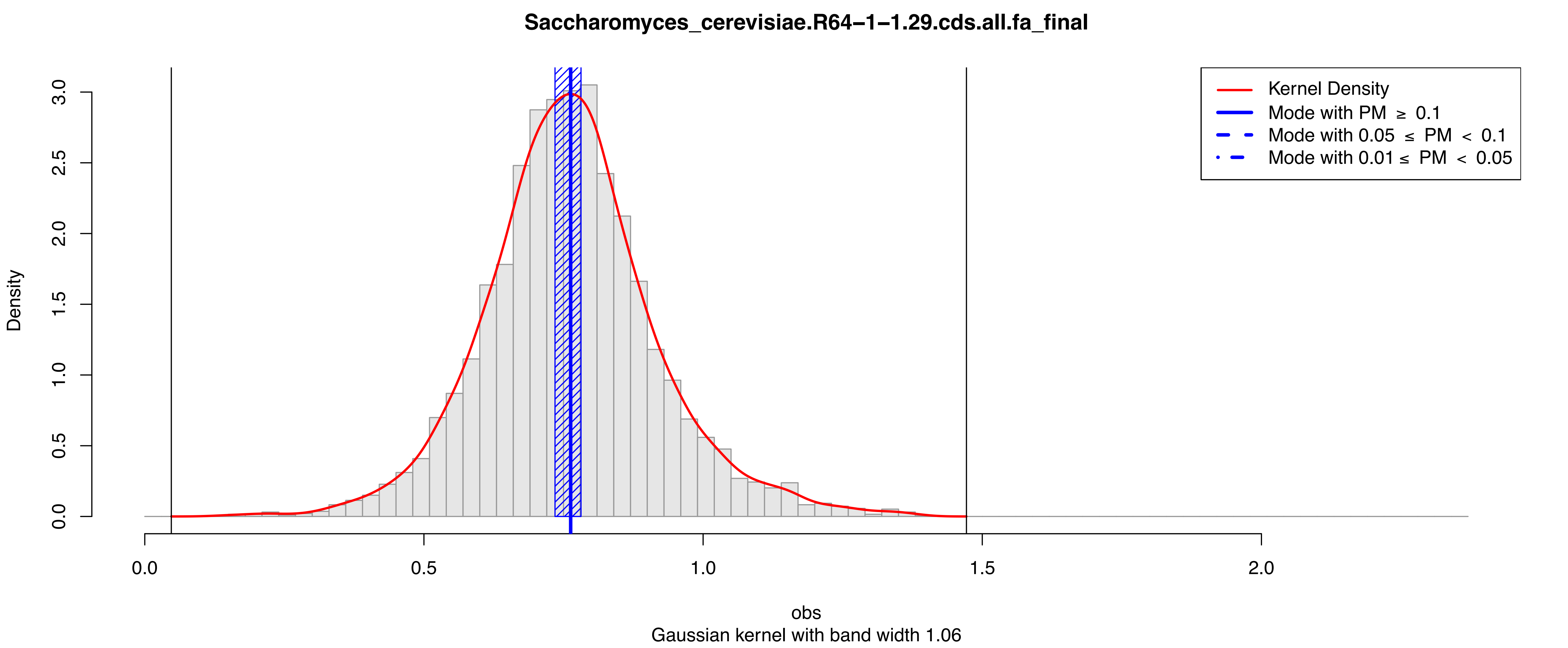
Saccharomyces_cerevisiae.R64−1−1.29.cds.all.fa_final
Kernel Density
Mode with PM ≥ 0.1
Mode with 0.05 ≤ PM < 0.1
Mode with 0.01 ≤ PM < 0.05
Density
0.0
0.5
1.0
1.5
2.0
2.5
3.0
obs
Gaussian kernel with band width 1.06

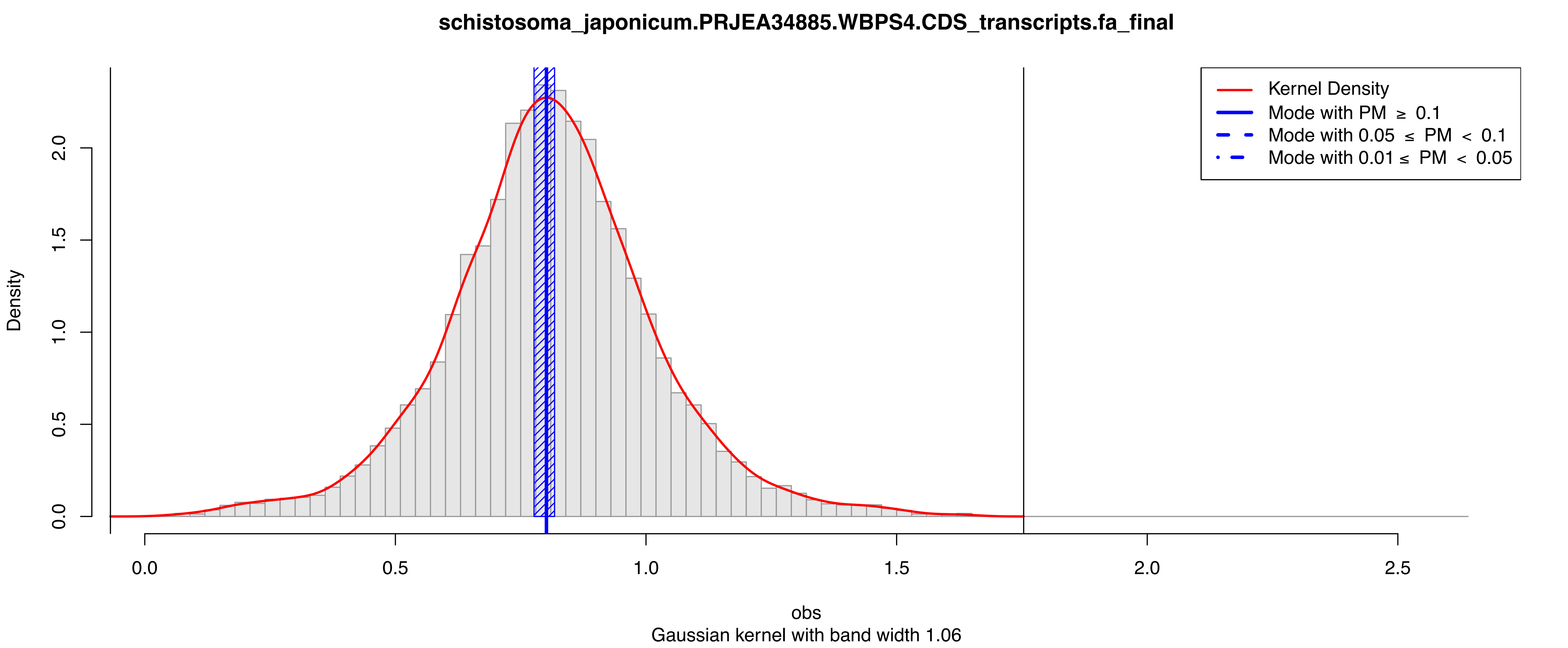

schistosoma_japonicum.PRJEA34885.WBPS4.CDS_transcripts.fa_final
Kernel Density
Mode with PM ≥ 0.1
Mode with 0.05 ≤ PM < 0.1
Mode with 0.01 ≤ PM < 0.05
Density
0.0
0.5
1.0
1.5
2.0
0.0
0.5
1.0
1.5
2.0
2.5
obs
Gaussian kernel with band width 1.06

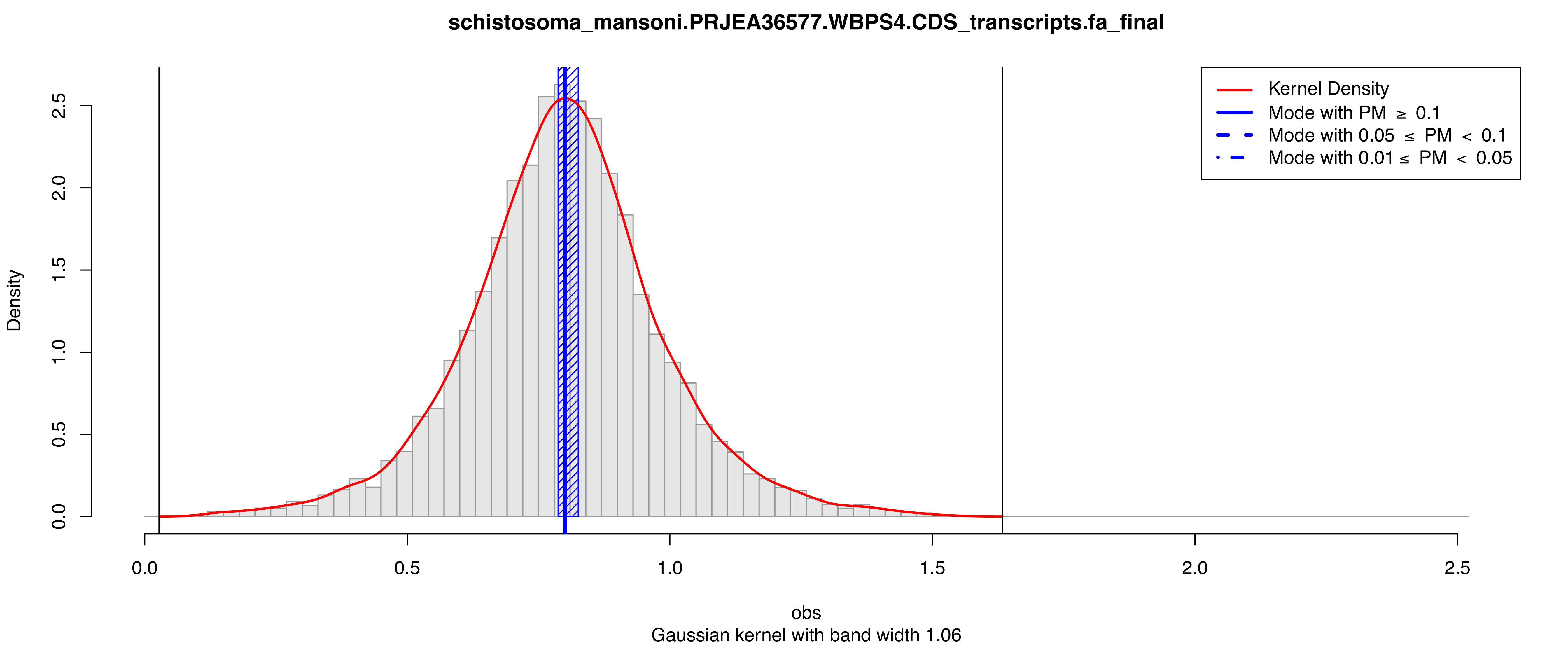
schistosoma_mansoni.PRJEA36577.WBPS4.CDS_transcripts.fa_final
Kernel Density
Mode with PM ≥ 0.1
Mode with 0.05 ≤ PM < 0.1
Mode with 0.01 ≤ PM < 0.05
Density
0.0
0.5
1.0
1.5
2.0
2.5
obs
Gaussian kernel with band width 1.06

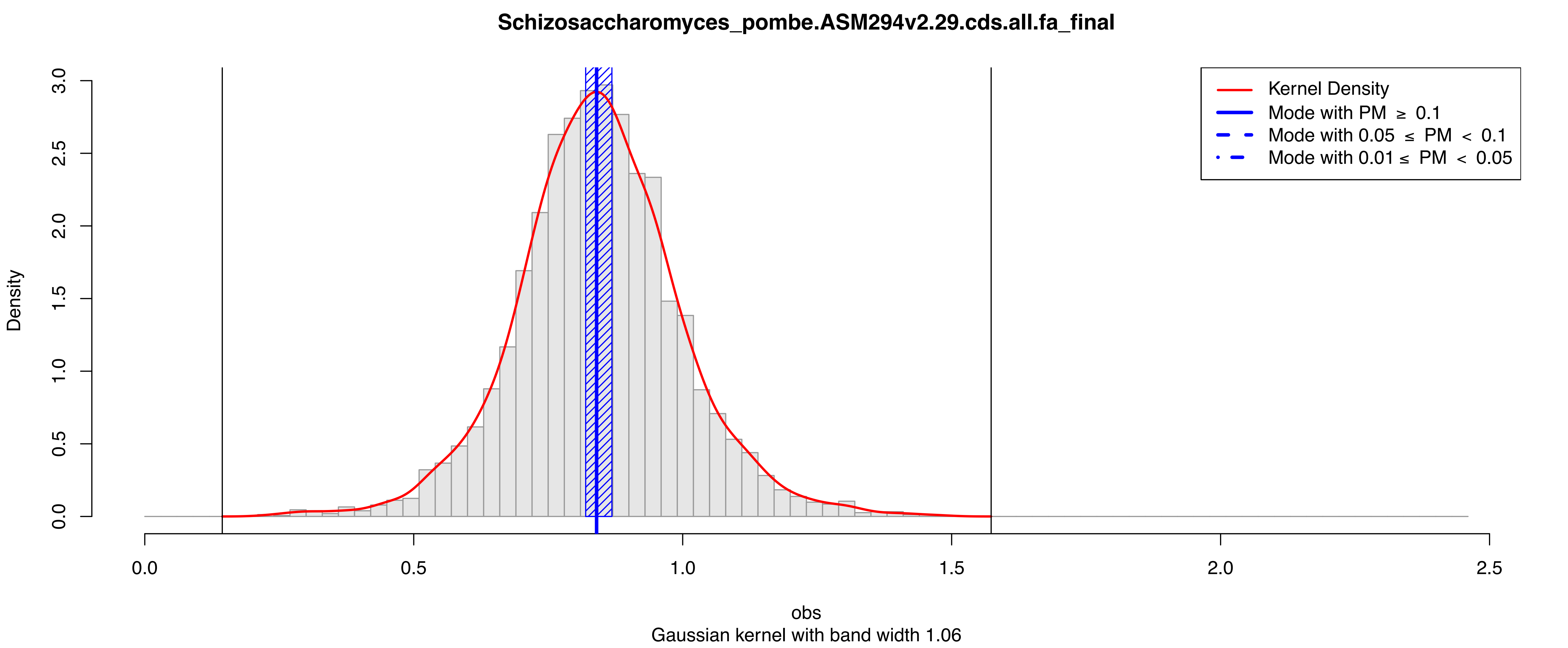

Schizosaccharomyces_pombe.ASM294v2.29.cds.all.fa_final
Kernel Density
Mode with PM ≥ 0.1
Mode with 0.05 ≤ PM < 0.1
Mode with 0.01 ≤ PM < 0.05
Density
0.0
0.5
1.0
1.5
2.0
2.5
3.0
obs
Gaussian kernel with band width 1.06

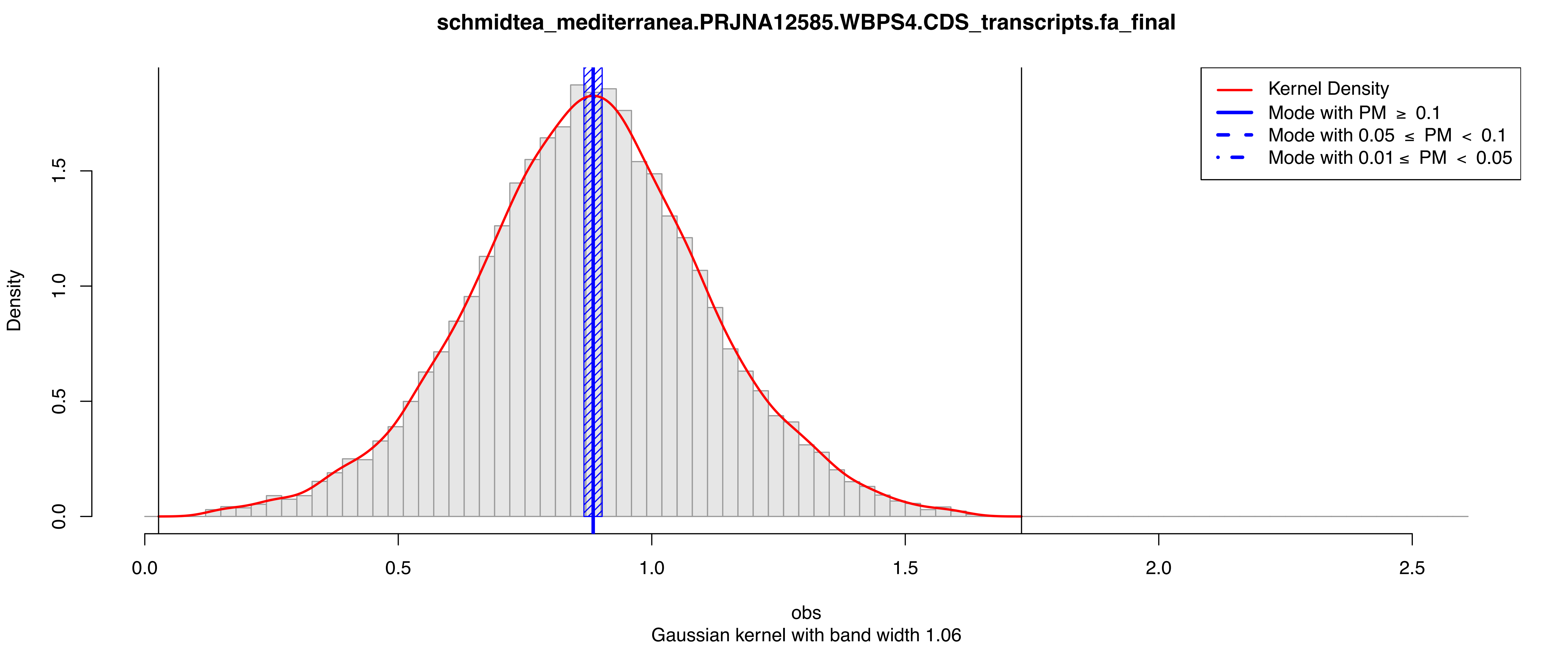

schmidtea_mediterranea.PRJNA12585.WBPS4.CDS_transcripts.fa_final
Kernel Density
Mode with PM ≥ 0.1
Mode with 0.05 ≤ PM < 0.1
Mode with 0.01 ≤ PM < 0.05
Density
0.0
0.5
1.0
1.5
2.0
2.5
obs
Gaussian kernel with band width 1.06

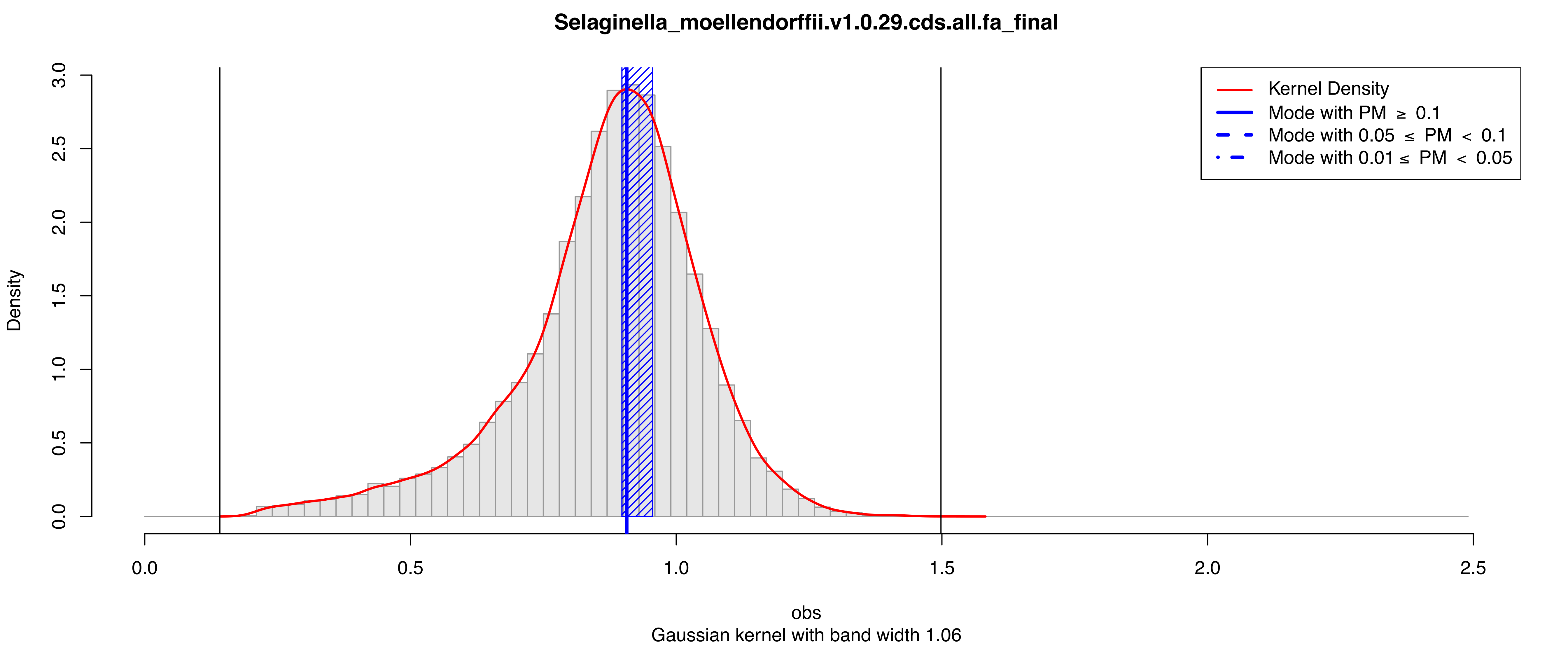
Selaginella_moellendorffii.v1.0.29.cds.all.fa_final
Kernel Density
Mode with PM ≥ 0.1
Mode with 0.05 ≤ PM < 0.1
Mode with 0.01 ≤ PM < 0.05
Density
0.0
0.5
1.0
1.5
2.0
2.5
3.0
0.0
0.5
1.0
1.5
2.0
2.5
obs
Gaussian kernel with band width 1.06

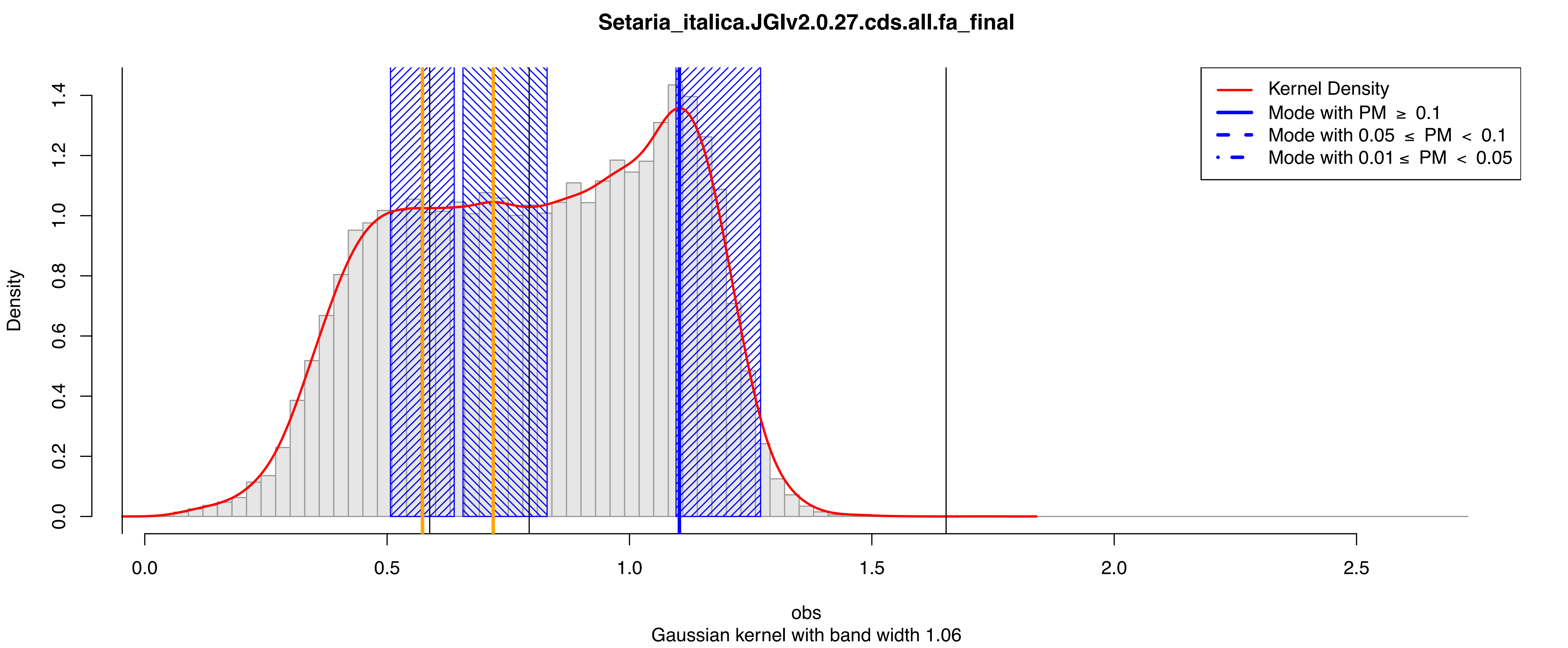

Setaria_italica.JGIv2.0.27.cds.all.fa_final
Kernel Density
Mode with PM ≥ 0.1
Mode with 0.05 ≤ PM < 0.1
Mode with 0.01 ≤ PM < 0.05
Density
0.0
0.2
0.4
0.6
0.8
1.0
1.2
1.4
0.0
0.5
1.0
1.5
2.0
2.5
obs
Gaussian kernel with band width 1.06

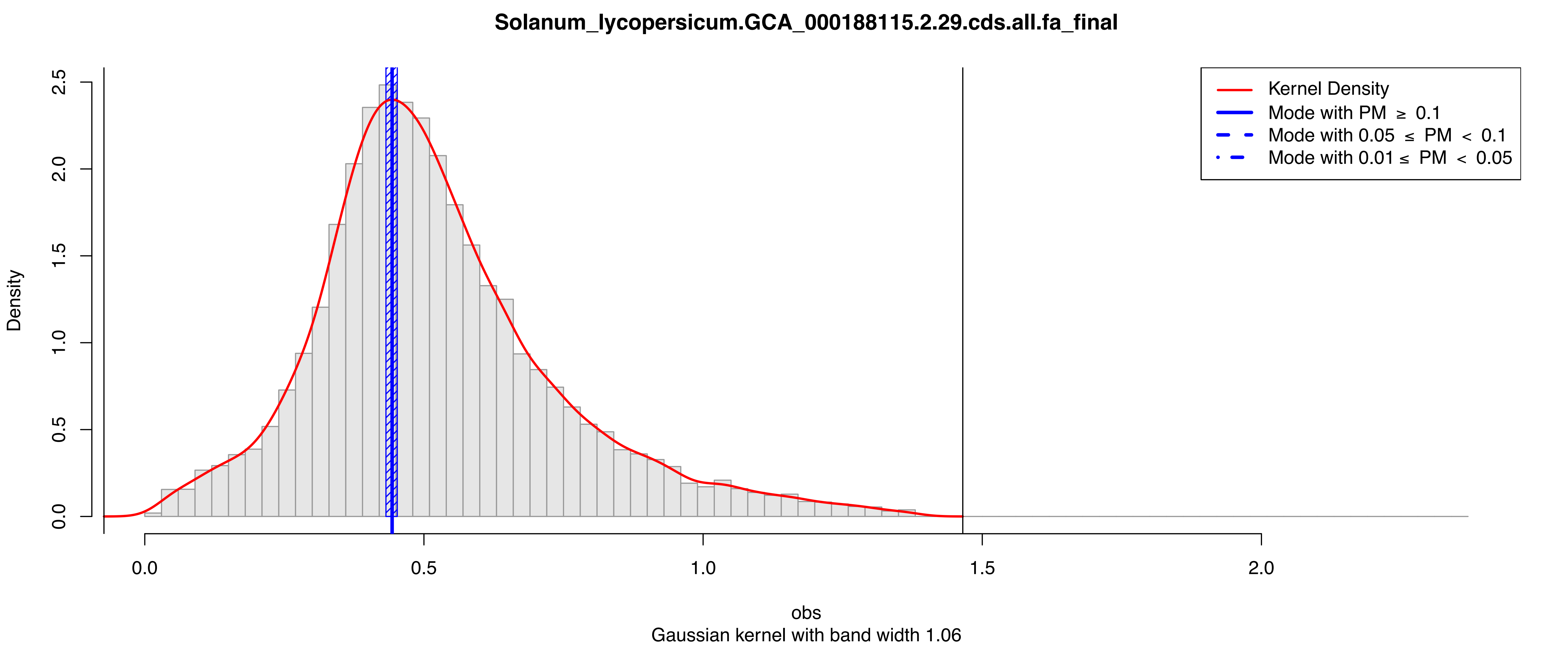
Solanum_lycopersicum.GCA_000188115.2.29.cds.all.fa_final
Kernel Density
Mode with PM ≥ 0.1
Mode with 0.05 ≤ PM < 0.1
Mode with 0.01 ≤ PM < 0.05
Density
0.0
0.5
1.0
1.5
2.0
2.5
obs
Gaussian kernel with band width 1.06

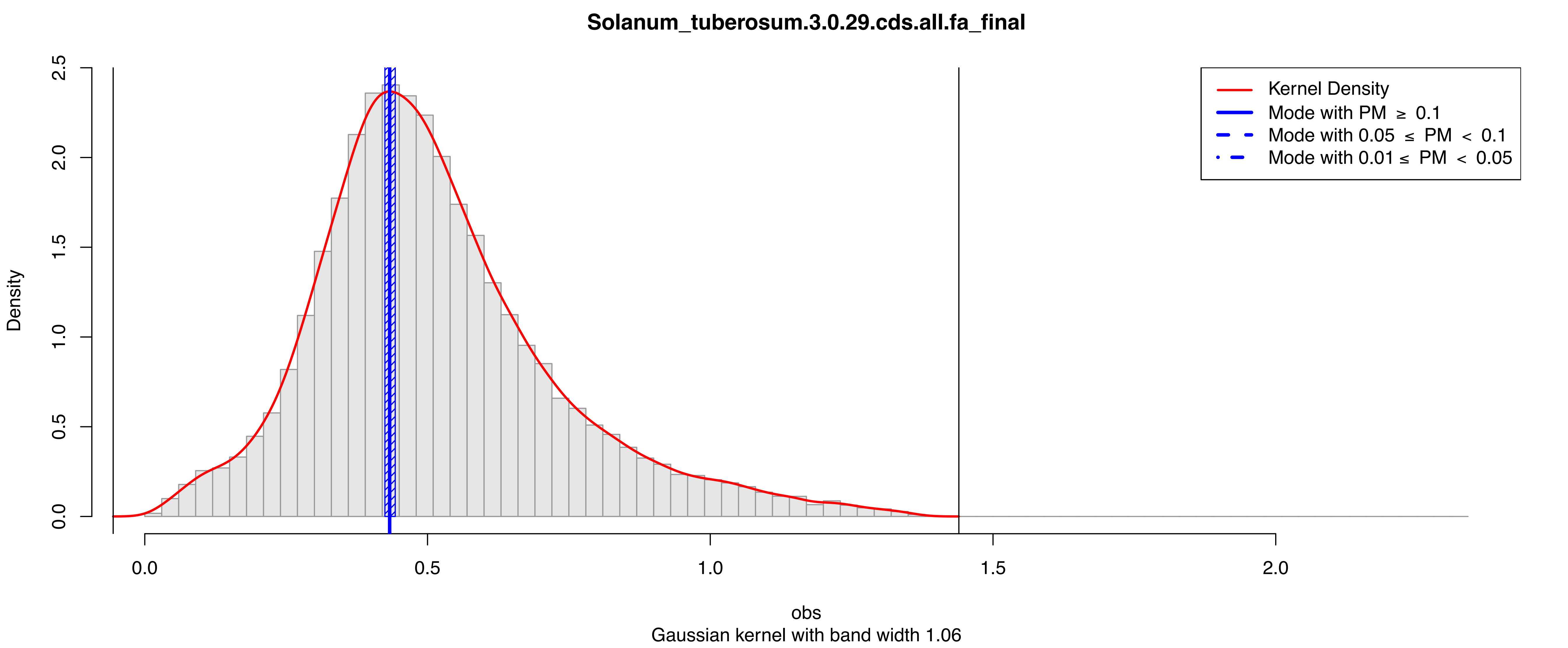

Solanum_tuberosum.3.0.29.cds.all.fa_final
Kernel Density
Mode with PM ≥ 0.1
Mode with 0.05 ≤ PM < 0.1
Mode with 0.01 ≤ PM < 0.05
Density
0.0
0.5
1.0
1.5
2.0
2.5
obs
Gaussian kernel with band width 1.06

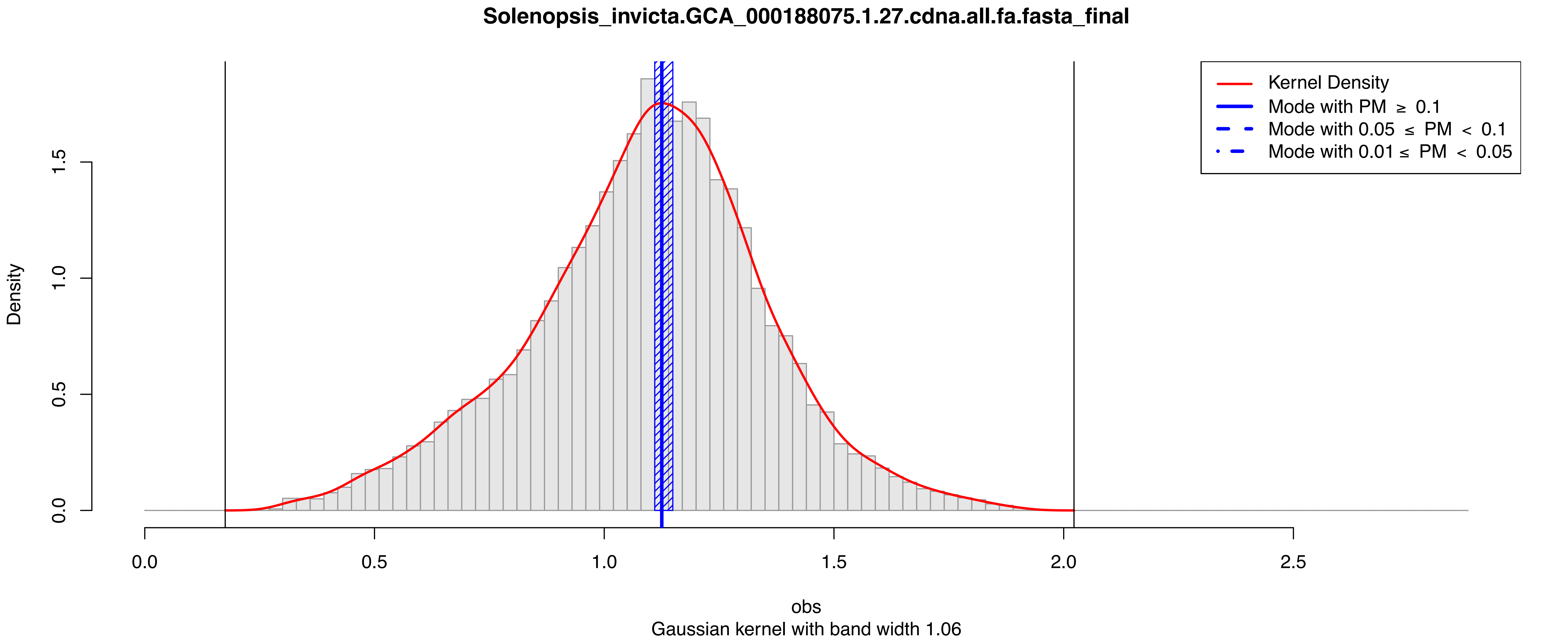
Solenopsis_invicta.GCA_000188075.1.27.cdna.all.fa.fasta_final
Kernel Density
Mode with PM ≥ 0.1
Mode with 0.05 ≤ PM < 0.1
Mode with 0.01 ≤ PM < 0.05
Density
0.0
0.5
1.0
1.5
0.0
0.5
1.0
1.5
2.0
2.5
obs
Gaussian kernel with band width 1.06

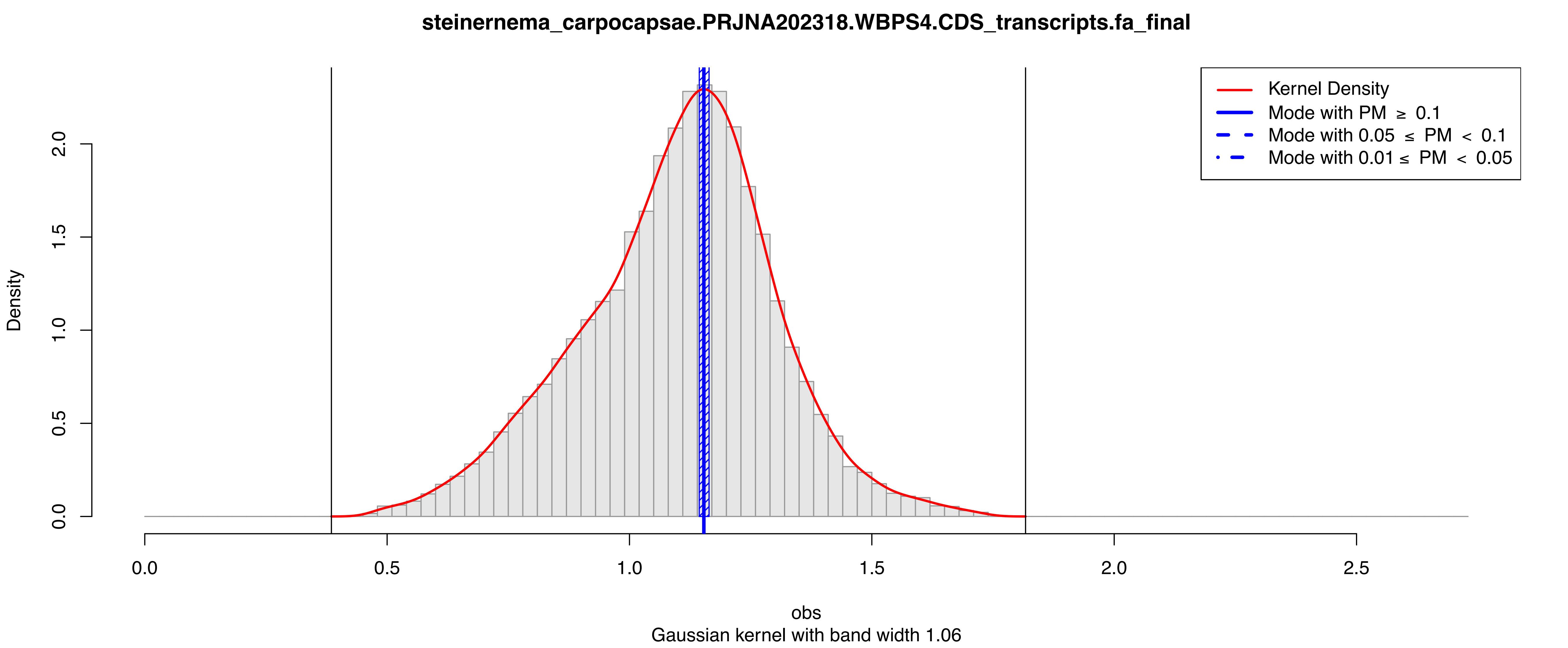

steinernema_carpocapsae.PRJNA202318.WBPS4.CDS_transcripts.fa_final
Kernel Density
Mode with PM ≥ 0.1
Mode with 0.05 ≤ PM < 0.1
Mode with 0.01 ≤ PM < 0.05
Density
0.0
0.5
1.0
1.5
2.0
0.0
0.5
1.0
1.5
2.0
2.5
obs
Gaussian kernel with band width 1.06

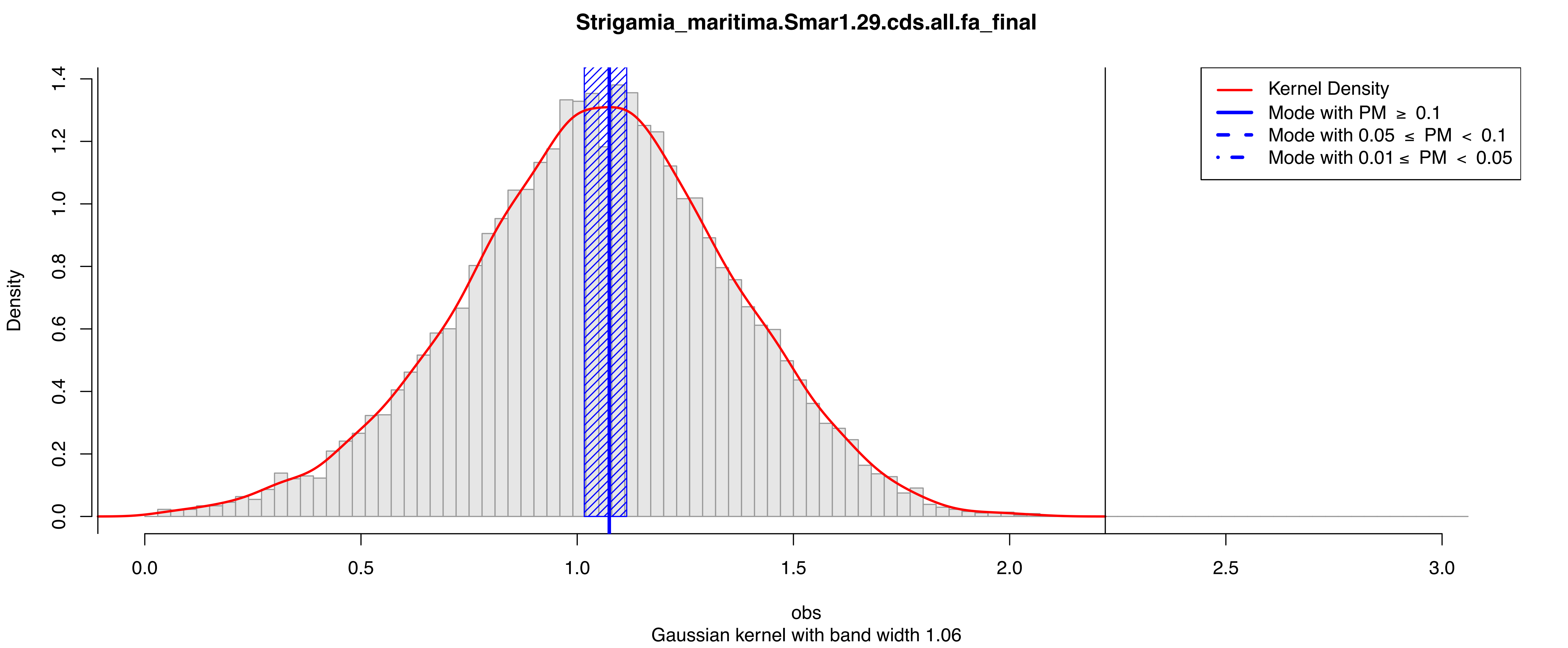

Strigamia_maritima.Smar1.29.cds.all.fa_final
Density
0.0
0.2
0.4
0.6
0.8
1.0
1.2
1.4
0.0
0.5
1.0
1.5
2.0
2.5
3.0
obs
Gaussian kernel with band width 1.06
Kernel Density
Mode with PM ≥ 0.1
Mode with 0.05 ≤ PM < 0.1
Mode with 0.01 ≤ PM < 0.05

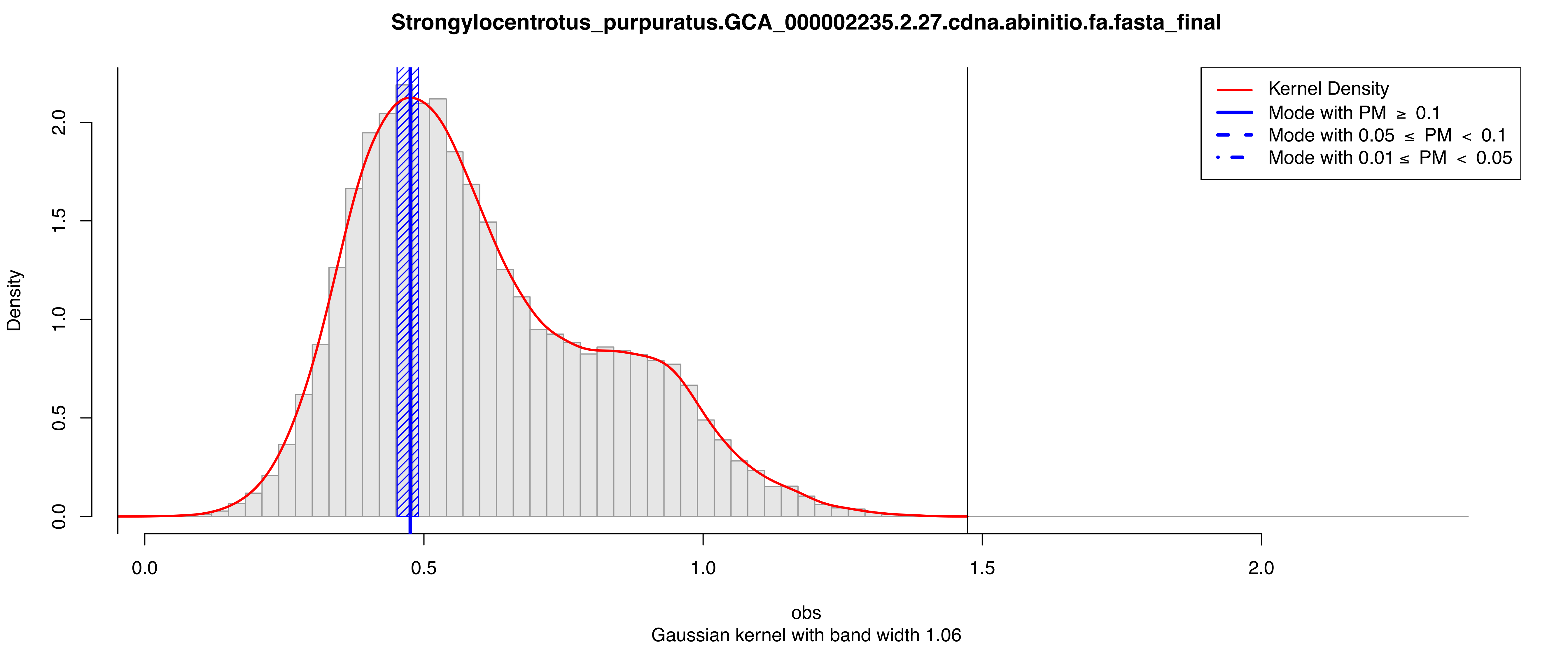

Strongylocentrotus_purpuratus.GCA_000002235.2.27.cdna.abinitio.fa.fasta_final
Kernel Density
Mode with PM ≥ 0.1
Mode with 0.05 ≤ PM < 0.1
Mode with 0.01 ≤ PM < 0.05
Density
0.0
0.5
1.0
1.5
2.0
0.0
0.5
1.0
1.5
2.0
obs
Gaussian kernel with band width 1.06

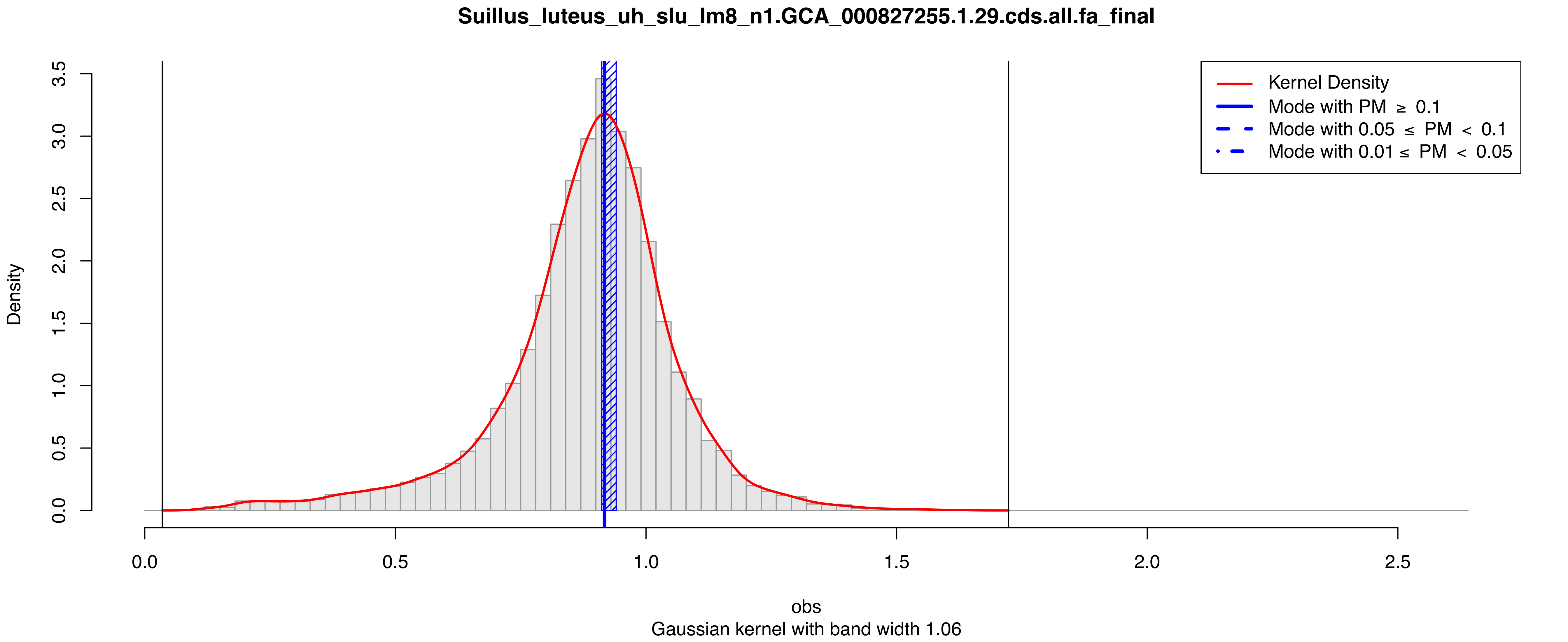
Suillus_luteus_uh_slu_lm8_n1.GCA_000827255.1.29.cds.all.fa_final
Density
obs
Gaussian kernel with band width 1.06
Kernel Density
Mode with PM ≥ 0.1
Mode with 0.05 ≤ PM < 0.1
Mode with 0.01 ≤ PM < 0.05
0.0
0.5
1.0
1.5
2.0
2.5
3.0
3.5

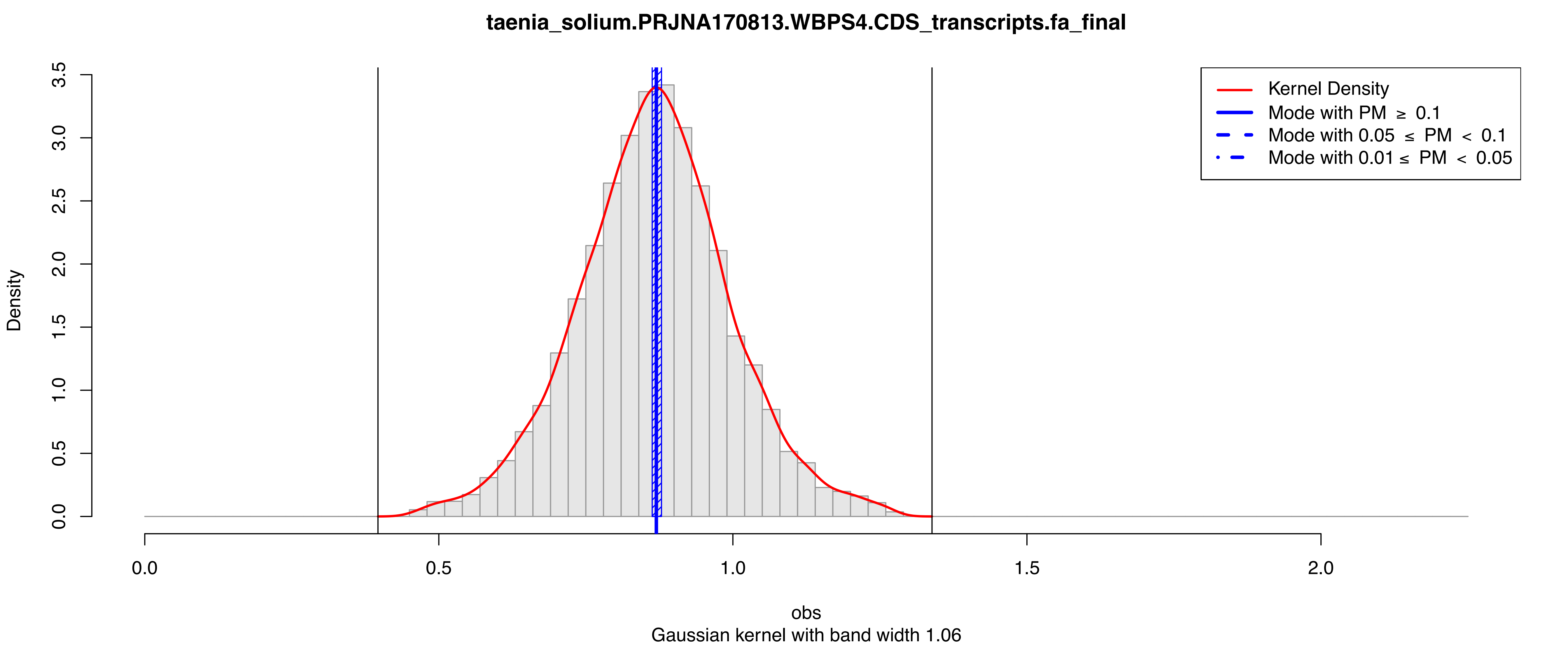

taenia_solium.PRJNA170813.WBPS4.CDS_transcripts.fa_final
Density
0.0
0.5
1.0
1.5
2.0
2.5
3.0
3.5
Kernel Density
Mode with PM ≥ 0.1
Mode with 0.05 ≤ PM < 0.1
Mode with 0.01 ≤ PM < 0.05
0.0
0.5
1.0
1.5
2.0
obs
Gaussian kernel with band width 1.06

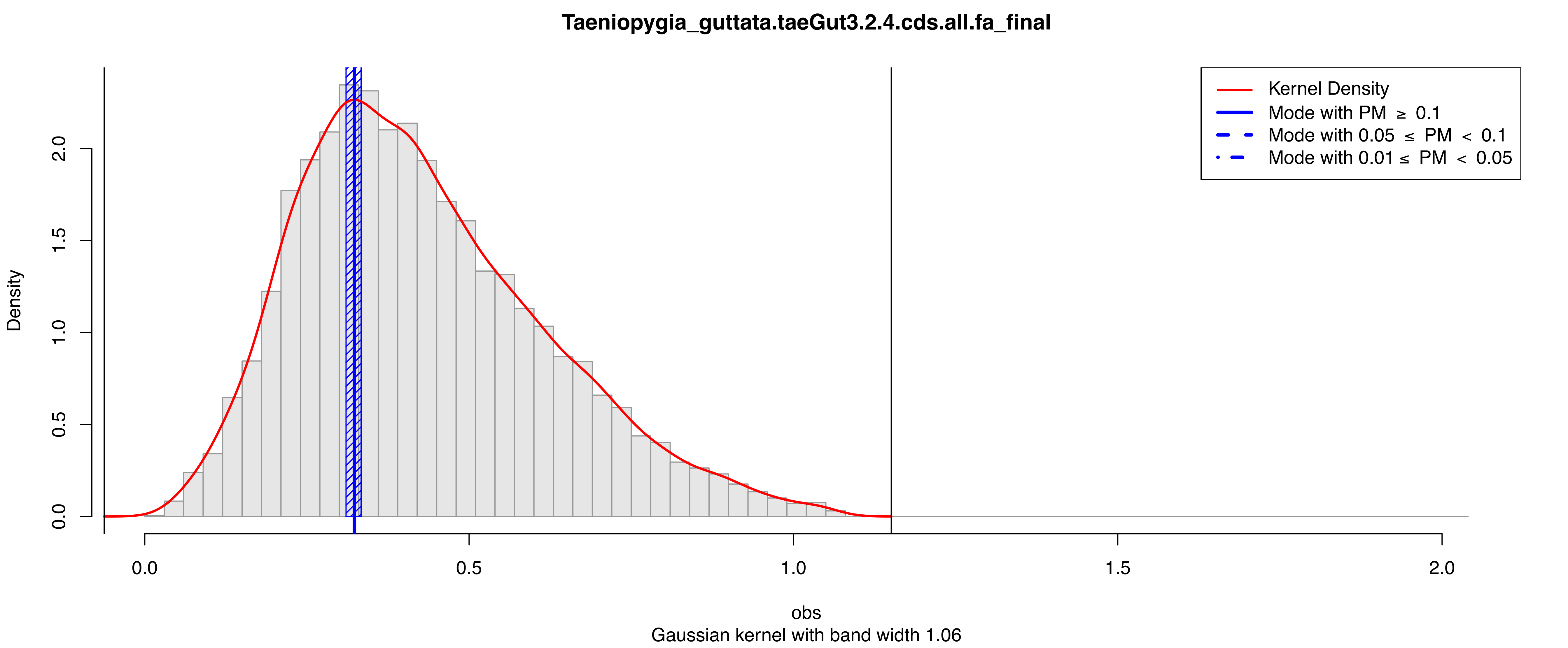

Taeniopygia_guttata.taeGut3.2.4.cds.all.fa_final
Kernel Density
Mode with PM ≥ 0.1
Mode with 0.05 ≤ PM < 0.1
Mode with 0.01 ≤ PM < 0.05
Density
0.0
0.5
1.0
1.5
2.0
0.0
0.5
1.0
1.5
2.0
obs
Gaussian kernel with band width 1.06

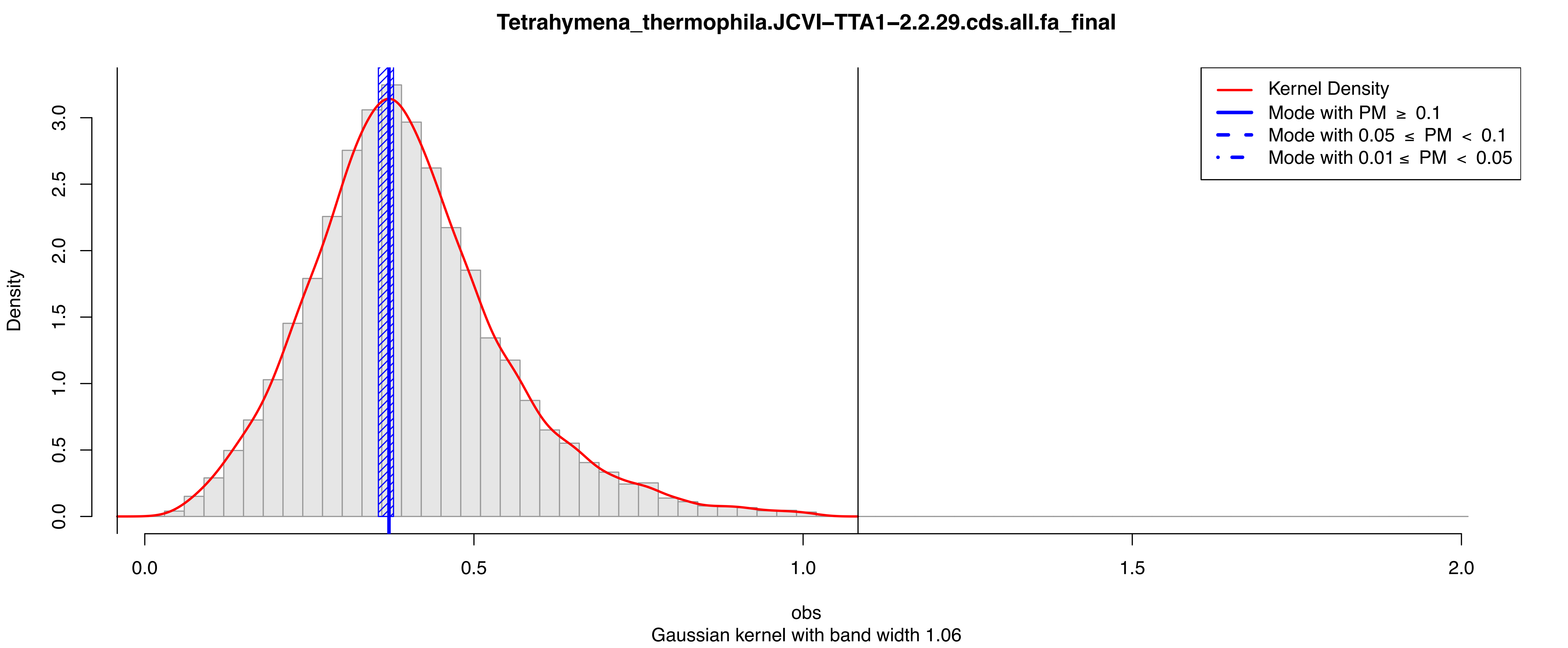
Tetrahymena_thermophila.JCVI–TTA1–2.2.29.cds.all.fa_final
Kernel Density
Mode with PM ≥ 0.1
Mode with 0.05 ≤ PM < 0.1
Mode with 0.01 ≤ PM < 0.05
Density
0.0
0.5
1.0
1.5
2.0
2.5
3.0
0.0
0.5
1.0
1.5
2.0
obs
Gaussian kernel with band width 1.06

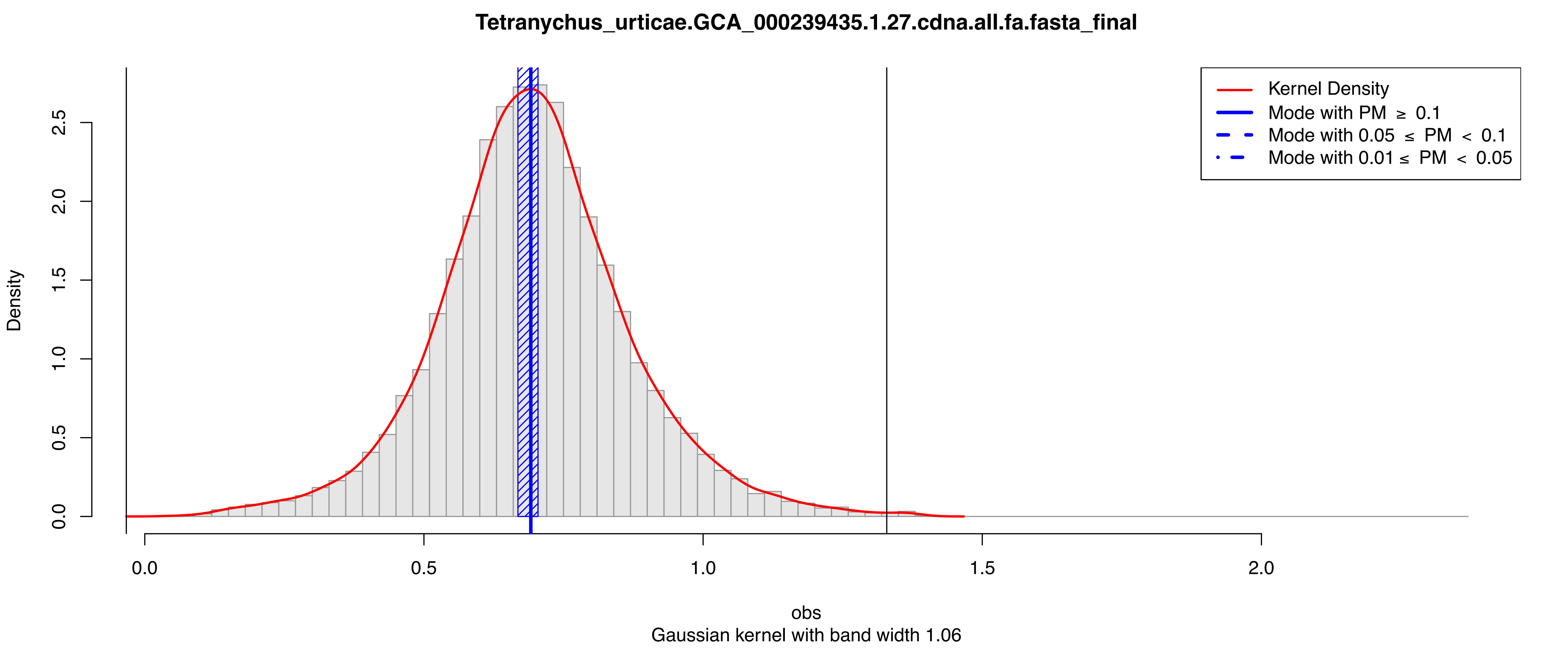

Tetranychus_urticae.GCA_000239435.1.27.cdna.all.fa.fasta_final
Kernel Density
Mode with PM ≥ 0.1
Mode with 0.05 ≤ PM < 0.1
Mode with 0.01 ≤ PM < 0.05
Density
0.0
0.5
1.0
1.5
2.0
2.5
obs
Gaussian kernel with band width 1.06

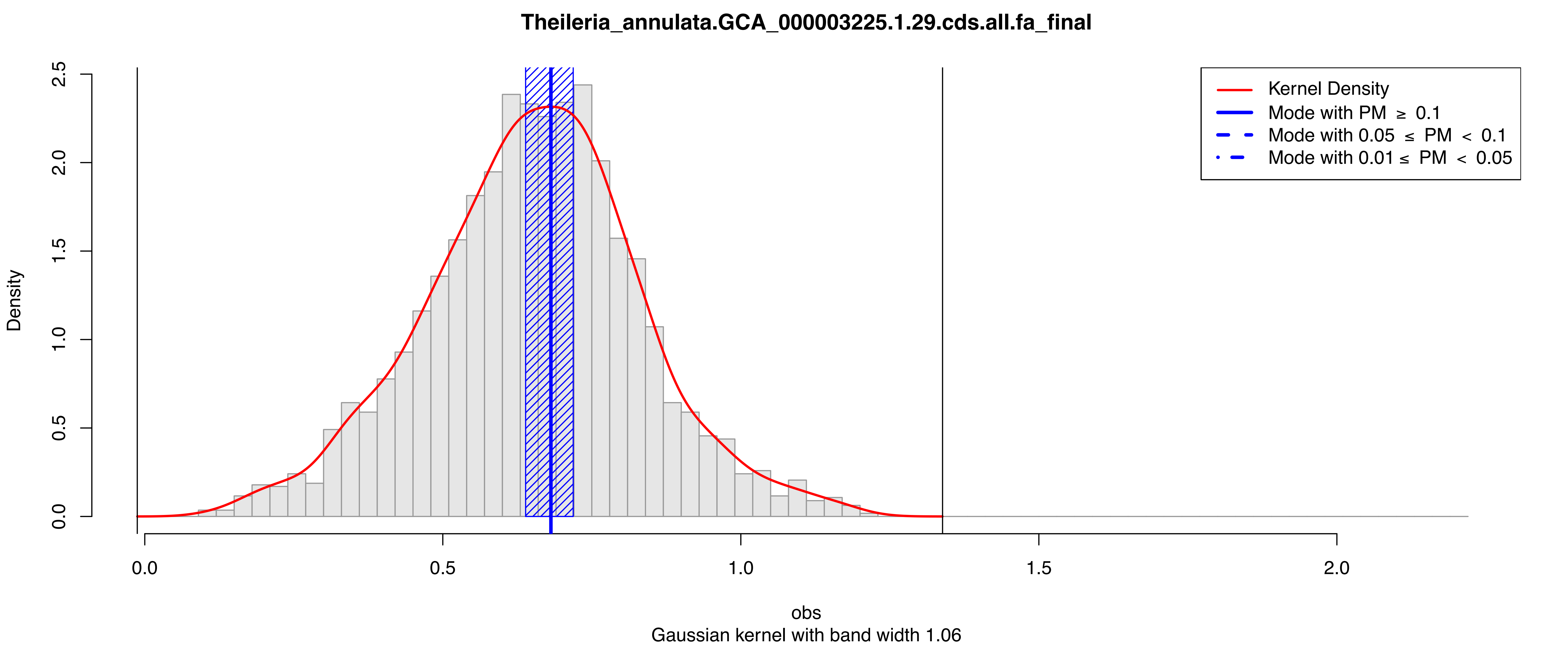

Theileria_annulata.GCA_000003225.1.29.cds.all.fa_final
Kernel Density
Mode with PM ≥ 0.1
Mode with 0.05 ≤ PM < 0.1
Mode with 0.01 ≤ PM < 0.05
Density
0.0
0.5
1.0
1.5
2.0
2.5
obs
Gaussian kernel with band width 1.06

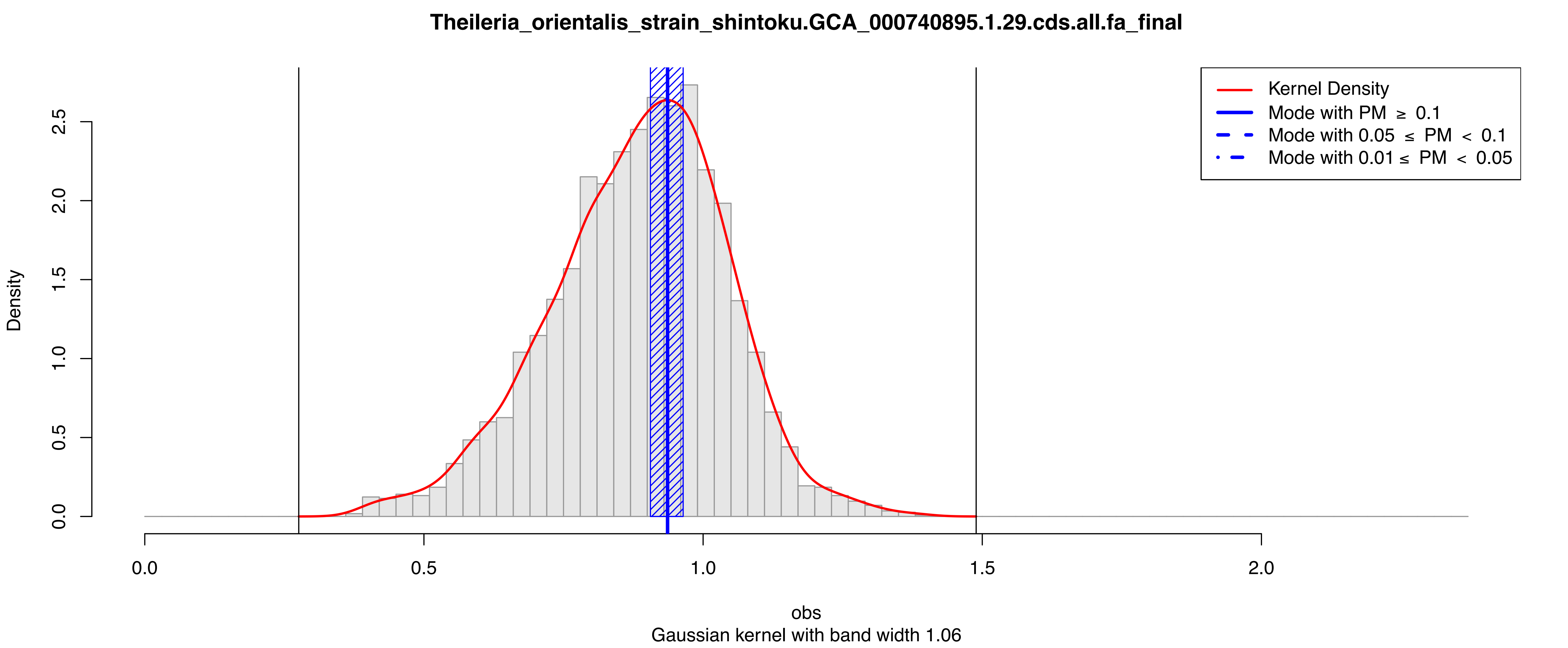
Theileria_orientalis_strain_shintoku.GCA_000740895.1.29.cds.all.fa_final
Kernel Density
Mode with PM ≥ 0.1
Mode with 0.05 ≤ PM < 0.1
Mode with 0.01 ≤ PM < 0.05
Density
0.0
0.5
1.0
1.5
2.0
2.5
0.0
0.5
1.0
1.5
2.0
obs
Gaussian kernel with band width 1.06

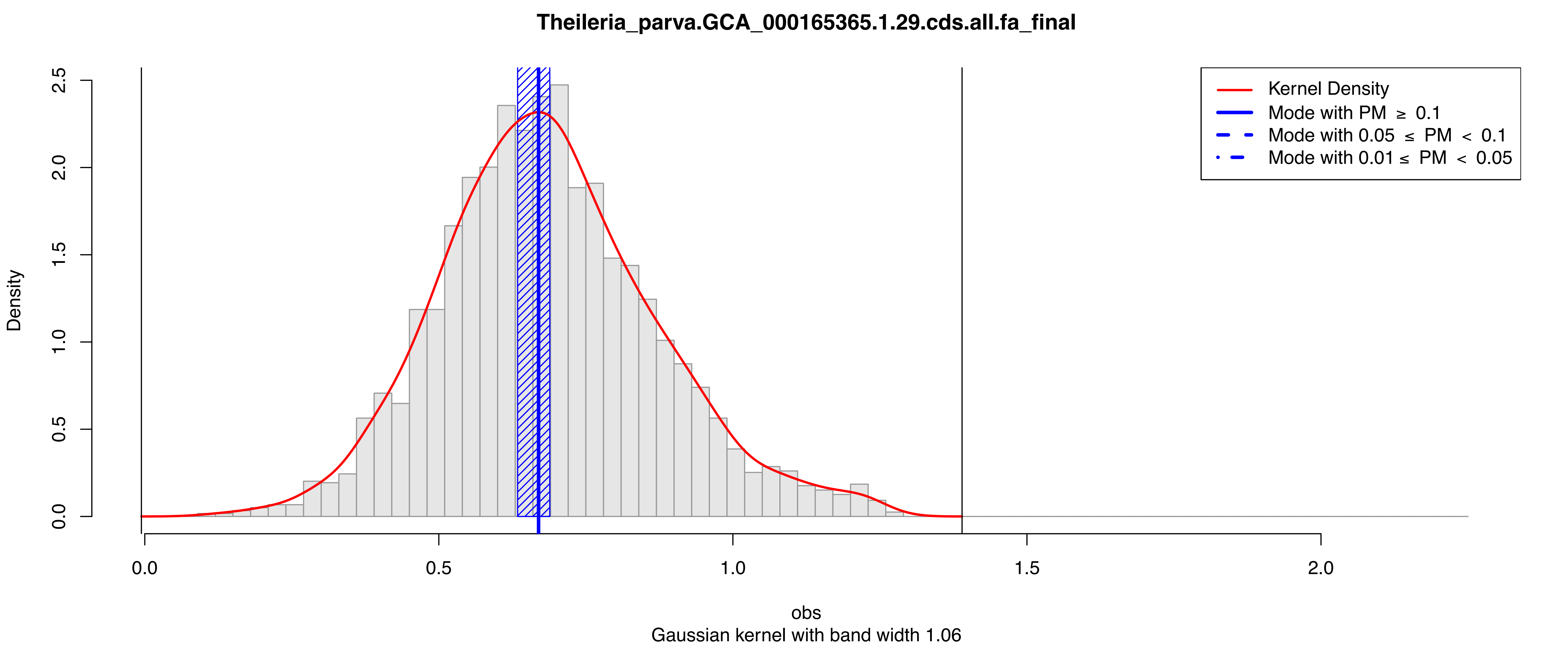

Theileria_parva.GCA_000165365.1.29.cds.all.fa_final
Kernel Density
Mode with PM ≥ 0.1
Mode with 0.05 ≤ PM < 0.1
Mode with 0.01 ≤ PM < 0.05
Density
0.0
0.5
1.0
1.5
2.0
2.5
obs
Gaussian kernel with band width 1.06

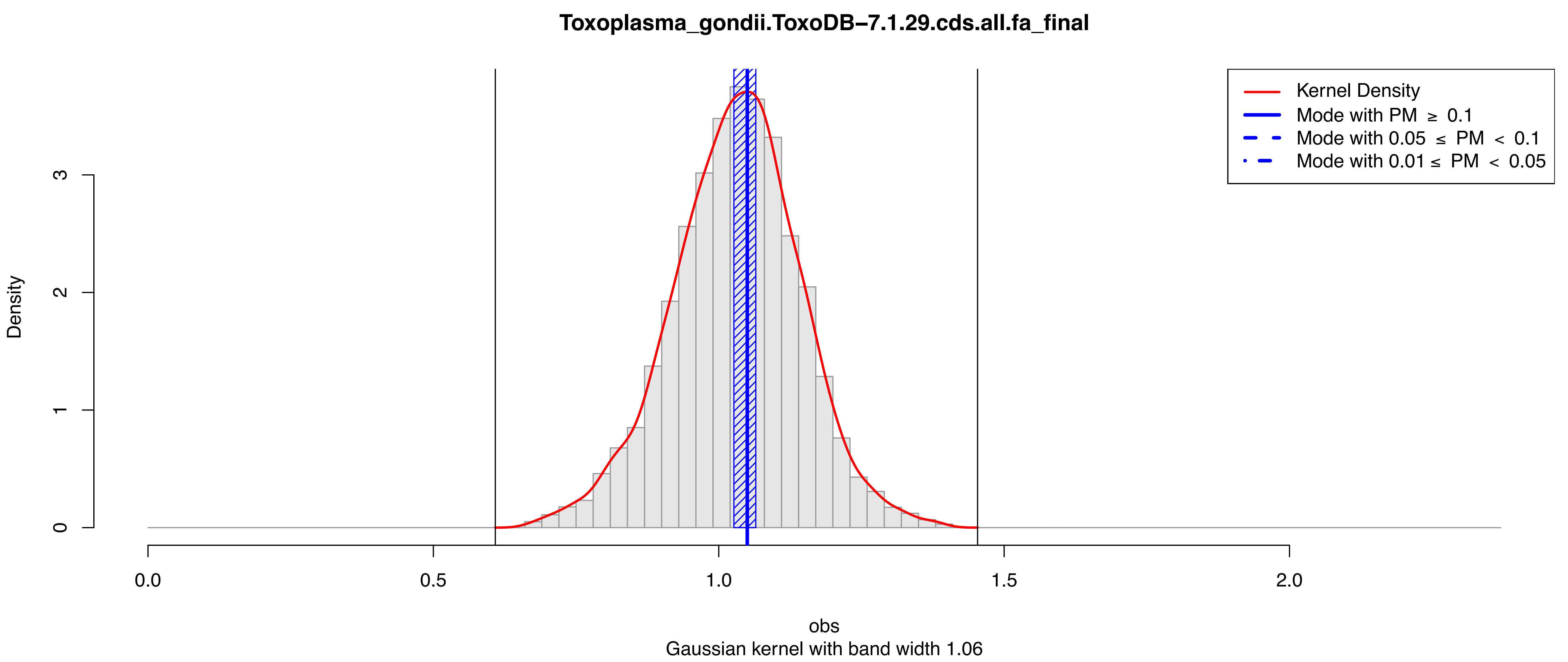

Toxoplasma_gondii.ToxoDB−7.1.29.cds.all.fa_final
Kernel Density
Mode with PM ≥ 0.1
Mode with 0.05 ≤ PM < 0.1
Mode with 0.01 ≤ PM < 0.05
Density
0
1
2
3
0.0
0.5
1.0
1.5
2.0
obs
Gaussian kernel with band width 1.06

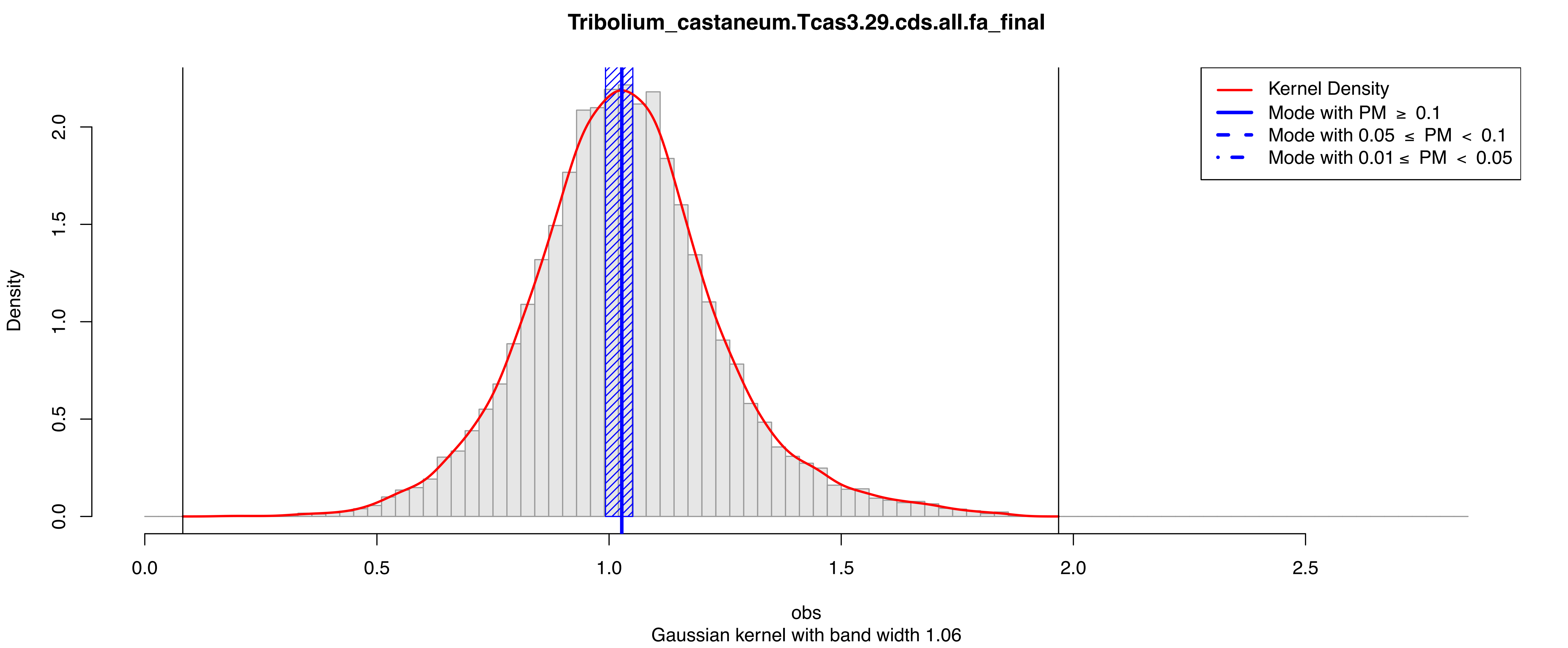
Tribolium_castaneum.Tcas3.29.cds.all.fa_final
Kernel Density
Mode with PM ≥ 0.1
Mode with 0.05 ≤ PM < 0.1
Mode with 0.01 ≤ PM < 0.05
Density
0.0
0.5
1.0
1.5
2.0
0.0
0.5
1.0
1.5
2.0
2.5
obs
Gaussian kernel with band width 1.06

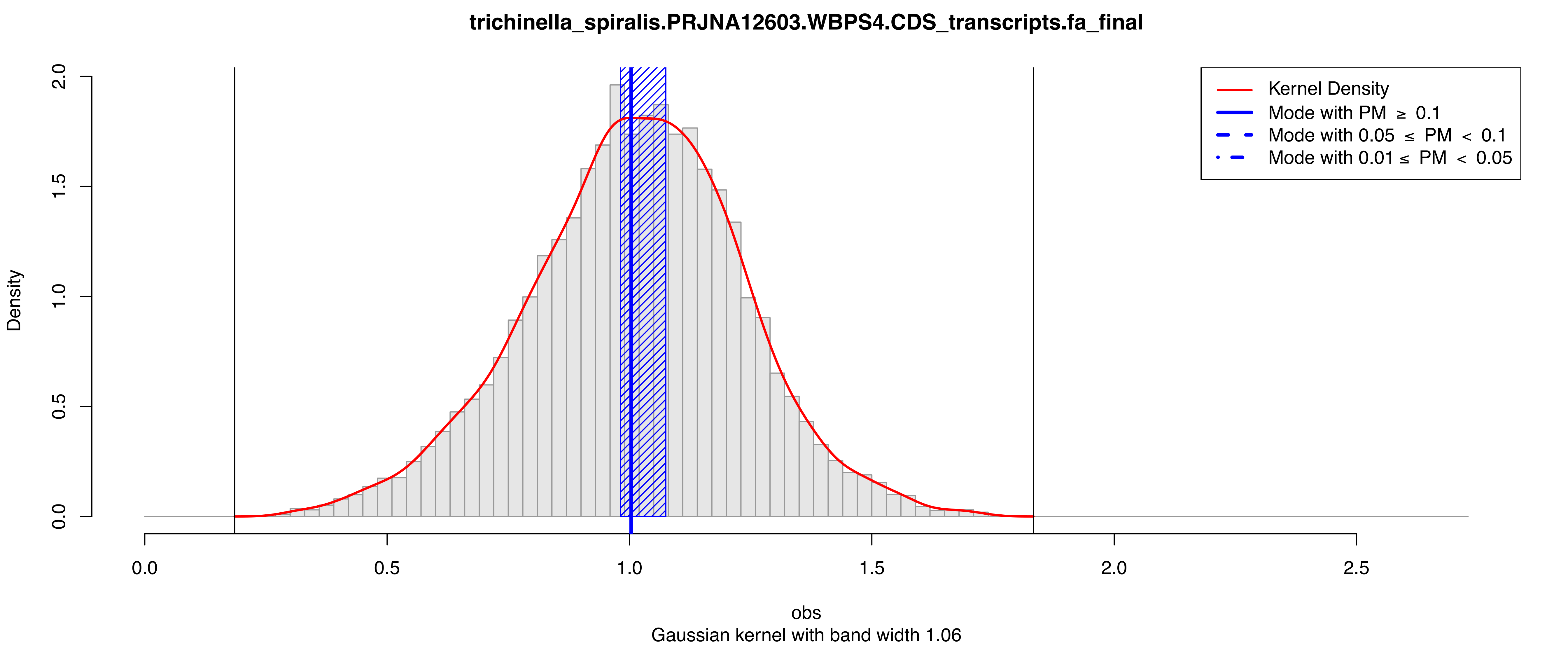
trichinella_spiralis.PRJNA12603.WBPS4.CDS_transcripts.fa_final
Kernel Density
Mode with PM ≥ 0.1
Mode with 0.05 ≤ PM < 0.1
Mode with 0.01 ≤ PM < 0.05
Density
0.0
0.5
1.0
1.5
2.0
0.0
0.5
1.0
1.5
2.0
2.5
obs
Gaussian kernel with band width 1.06

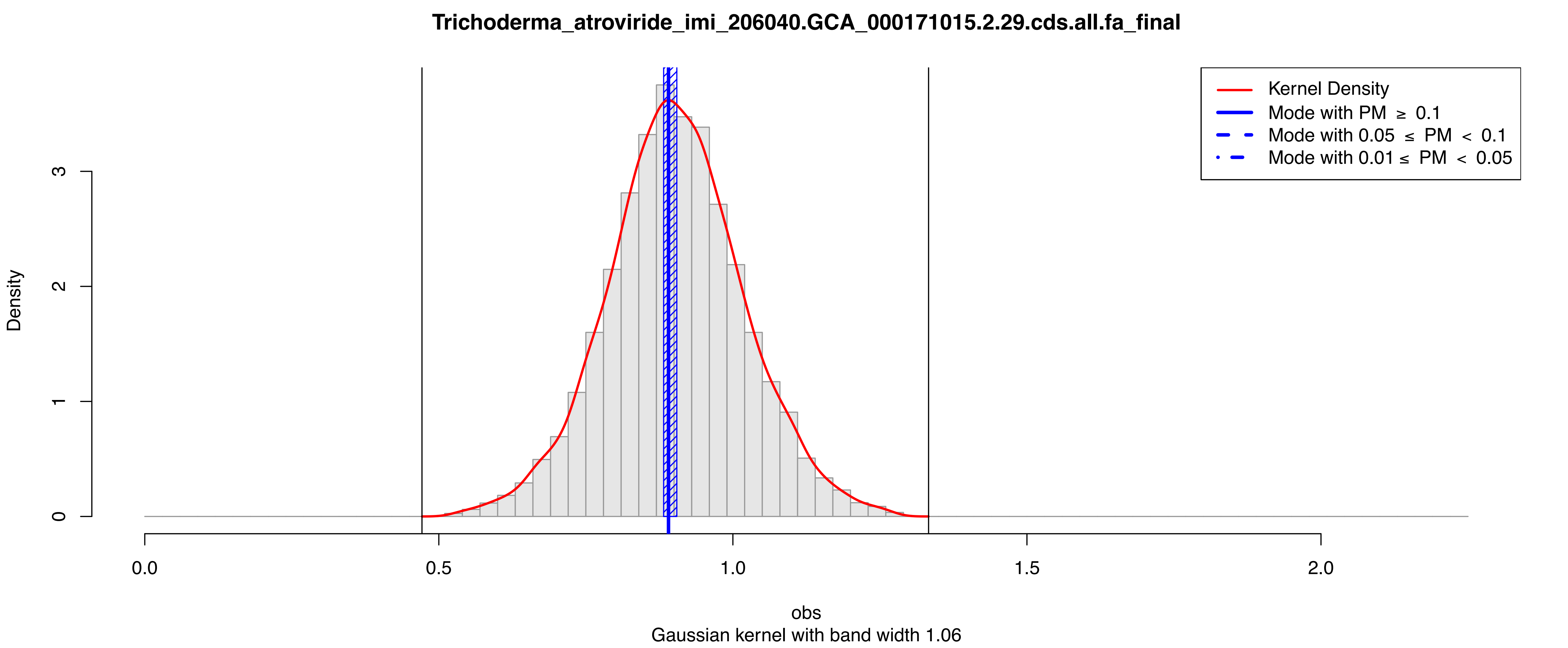

Trichoderma_atroviride_imi_206040.GCA_000171015.2.29.cds.all.fa_final
Kernel Density
Mode with PM ≥ 0.1
Mode with 0.05 ≤ PM < 0.1
Mode with 0.01 ≤ PM < 0.05
Density
0
1
2
3
0.0
0.5
1.0
1.5
2.0
obs
Gaussian kernel with band width 1.06

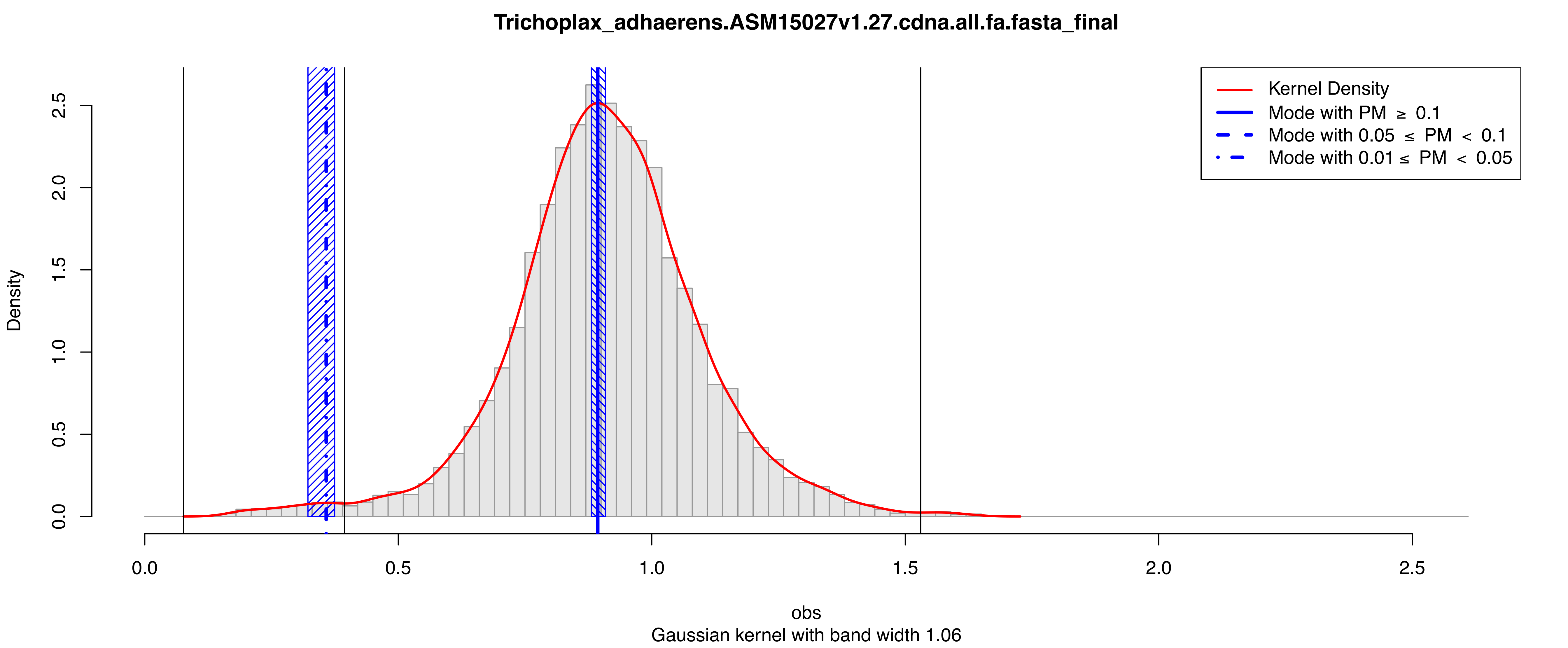
Trichoplax_adhaerens.ASM15027v1.27.cdna.all.fa.fasta_final
Kernel Density
Mode with PM ≥ 0.1
Mode with 0.05 ≤ PM < 0.1
Mode with 0.01 ≤ PM < 0.05
Density
0.0
0.5
1.0
1.5
2.0
2.5
0.0
0.5
1.0
1.5
2.0
2.5
obs
Gaussian kernel with band width 1.06

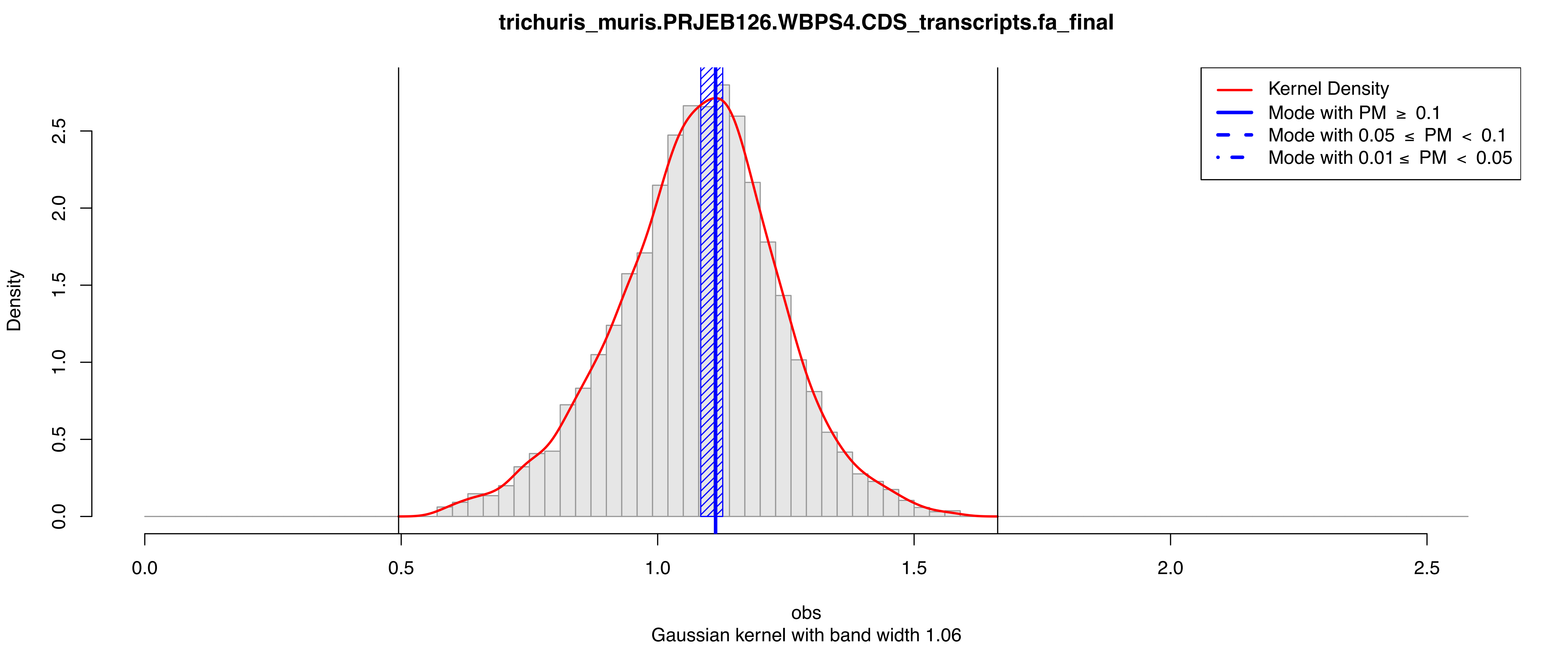
trichuris_muris.PRJEB126.WBPS4.CDS_transcripts.fa_final
Kernel Density
Mode with PM ≥ 0.1
Mode with 0.05 ≤ PM < 0.1
Mode with 0.01 ≤ PM < 0.05
Density
0.0
0.5
1.0
1.5
2.0
2.5
0.0
0.5
1.0
1.5
2.0
2.5
obs
Gaussian kernel with band width 1.06

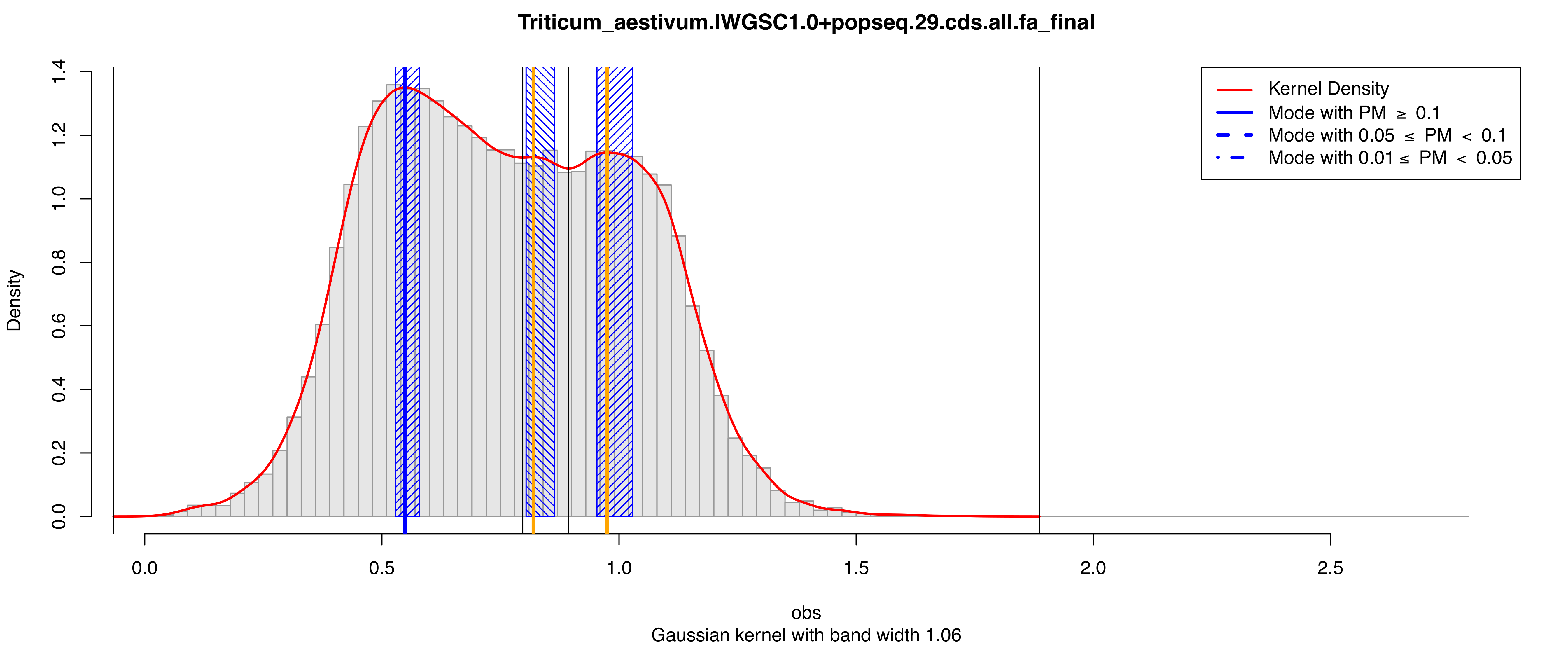

Triticum_aestivum.IWGSC1.0+popseq.29.cds.all.fa_final
Density
0.0
0.2
0.4
0.6
0.8
1.0
1.2
1.4
0.0
0.5
1.0
1.5
2.0
2.5
obs
Gaussian kernel with band width 1.06
Kernel Density
Mode with PM ≥ 0.1
Mode with 0.05 ≤ PM < 0.1
Mode with 0.01 ≤ PM < 0.05

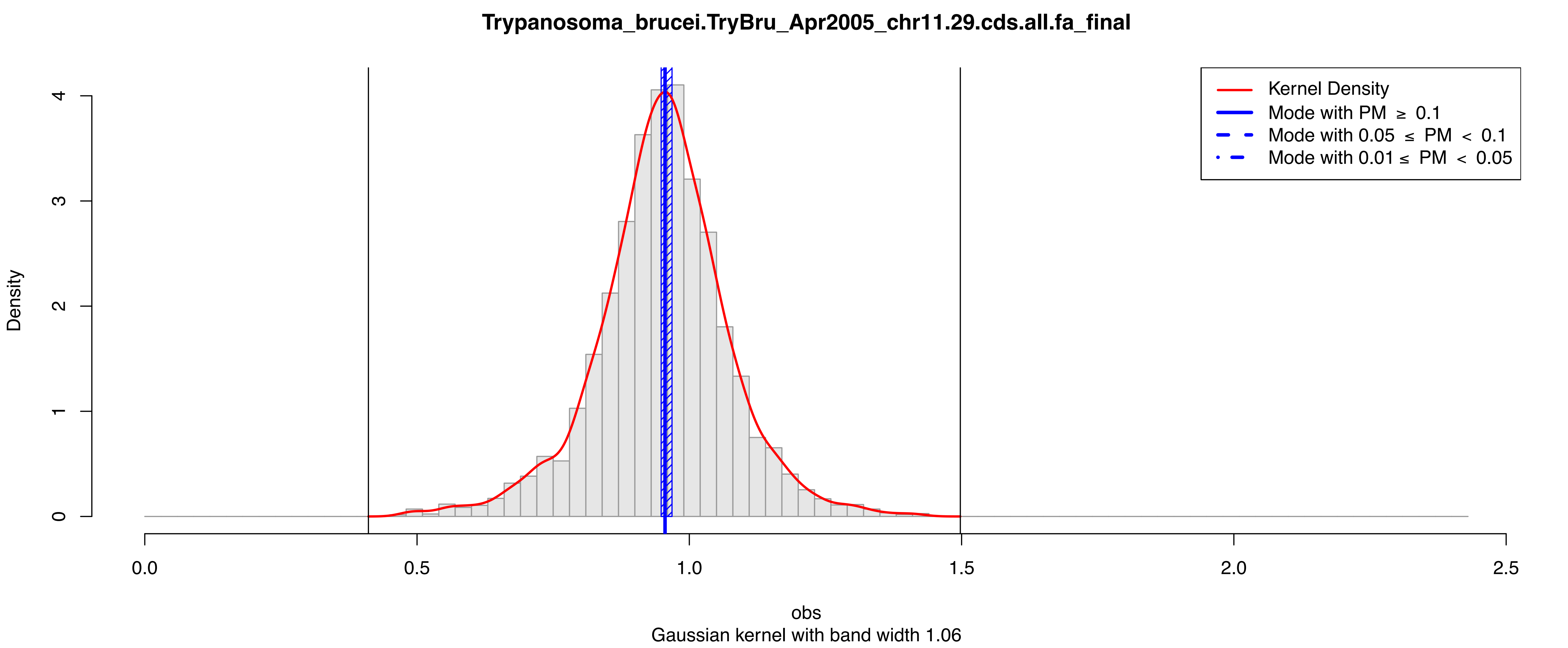

Trypanosoma_brucei.TryBru_Apr2005_chr11.29.cds.all.fa_final
Kernel Density
Mode with PM ≥ 0.1
Mode with 0.05 ≤ PM < 0.1
Mode with 0.01 ≤ PM < 0.05
Density
0
1
2
3
4
0.0
0.5
1.0
1.5
2.0
2.5
obs
Gaussian kernel with band width 1.06

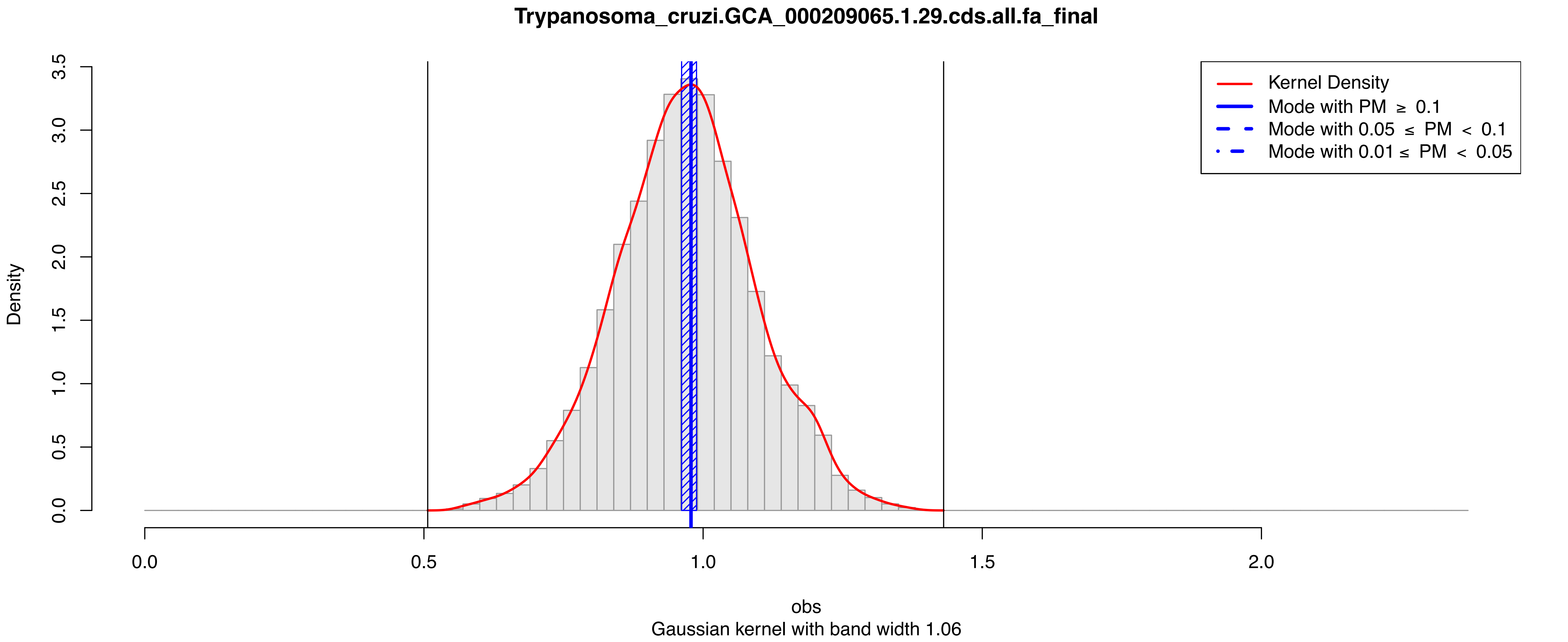
Trypanosoma_cruzi.GCA_000209065.1.29.cds.all.fa_final
Density
0.0
0.5
1.0
1.5
2.0
2.5
3.0
3.5
0.0
0.5
1.0
1.5
2.0
obs
Gaussian kernel with band width 1.06
Kernel Density
Mode with PM ≥ 0.1
Mode with 0.05 ≤ PM < 0.1
Mode with 0.01 ≤ PM < 0.05

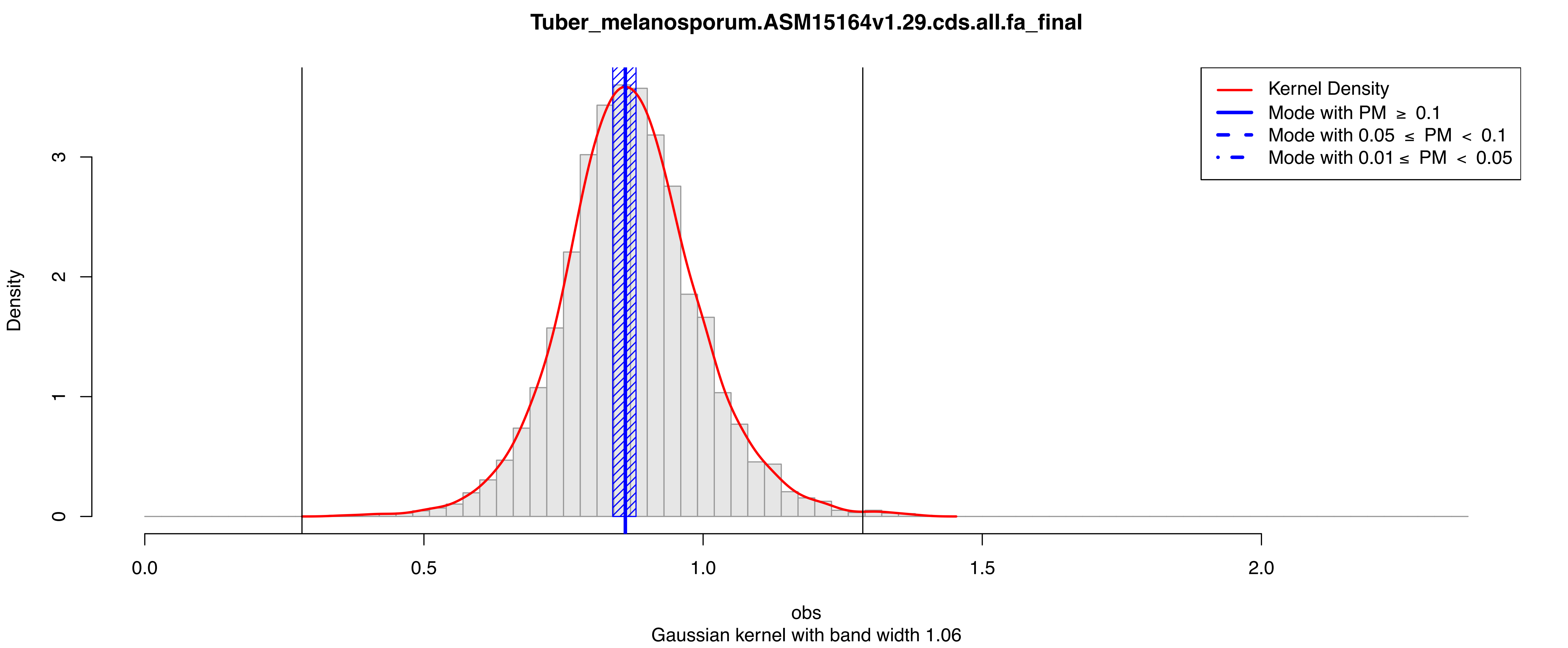
Tuber_melanosporum.ASM15164v1.29.cds.all.fa_final
Kernel Density
Mode with PM ≥ 0.1
Mode with 0.05 ≤ PM < 0.1
Mode with 0.01 ≤ PM < 0.05
Density
0
1
2
3
0.0
0.5
1.0
1.5
2.0
obs
Gaussian kernel with band width 1.06

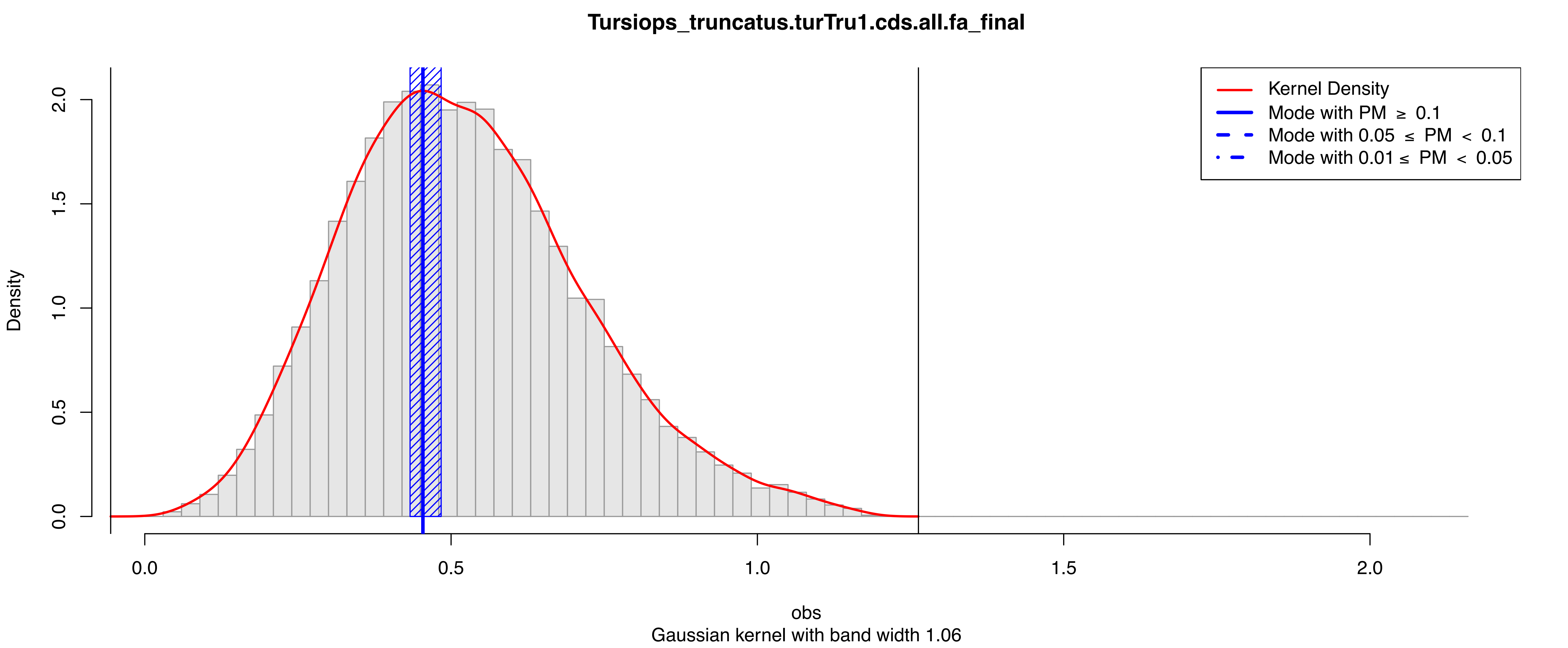

Tursiops_truncatus.turTru1.cds.all.fa_final
Kernel Density
Mode with PM ≥ 0.1
Mode with 0.05 ≤ PM < 0.1
Mode with 0.01 ≤ PM < 0.05
Density
0.0
0.5
1.0
1.5
2.0
obs
Gaussian kernel with band width 1.06

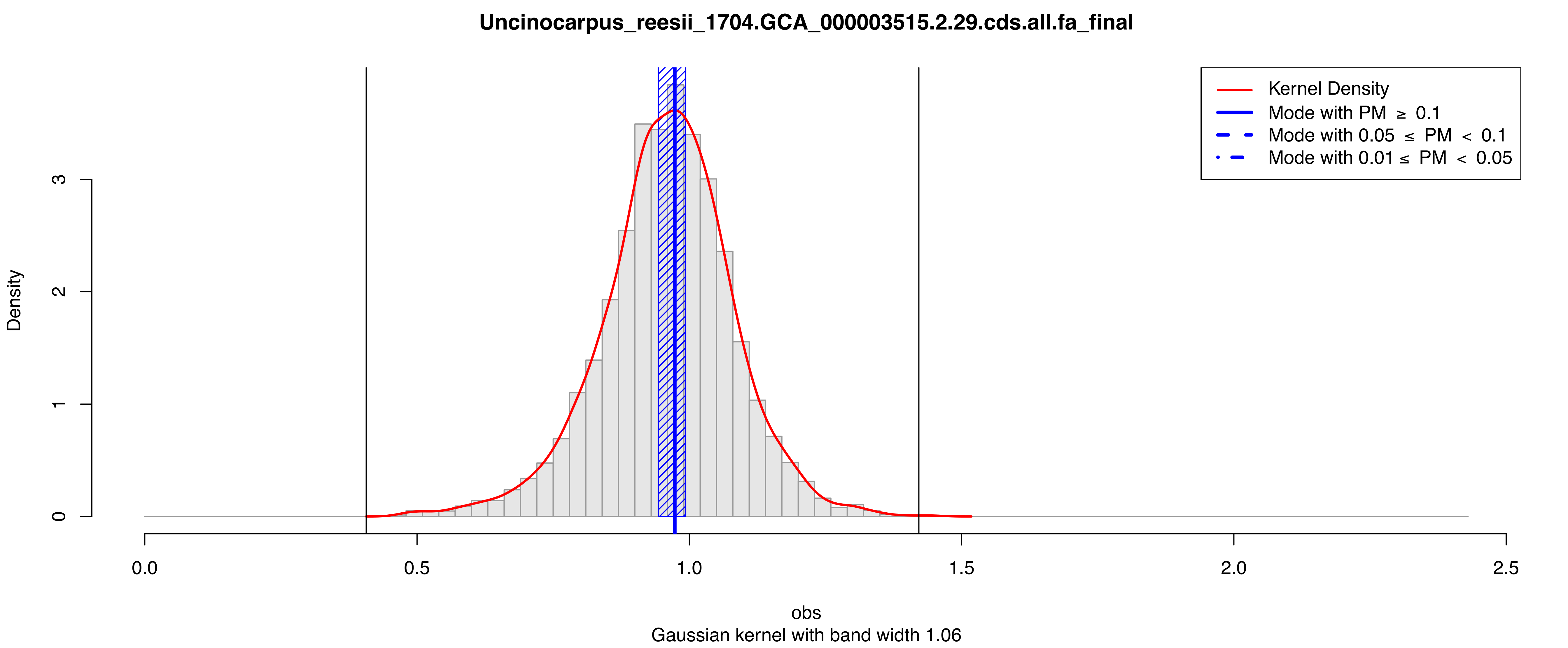

Uncinocarpus_reesii_1704.GCA_000003515.2.29.cds.all.fa_final
Kernel Density
Mode with PM ≥ 0.1
Mode with 0.05 ≤ PM < 0.1
Mode with 0.01 ≤ PM < 0.05
Density
0
1
2
3
0.0
0.5
1.0
1.5
2.0
2.5
obs
Gaussian kernel with band width 1.06

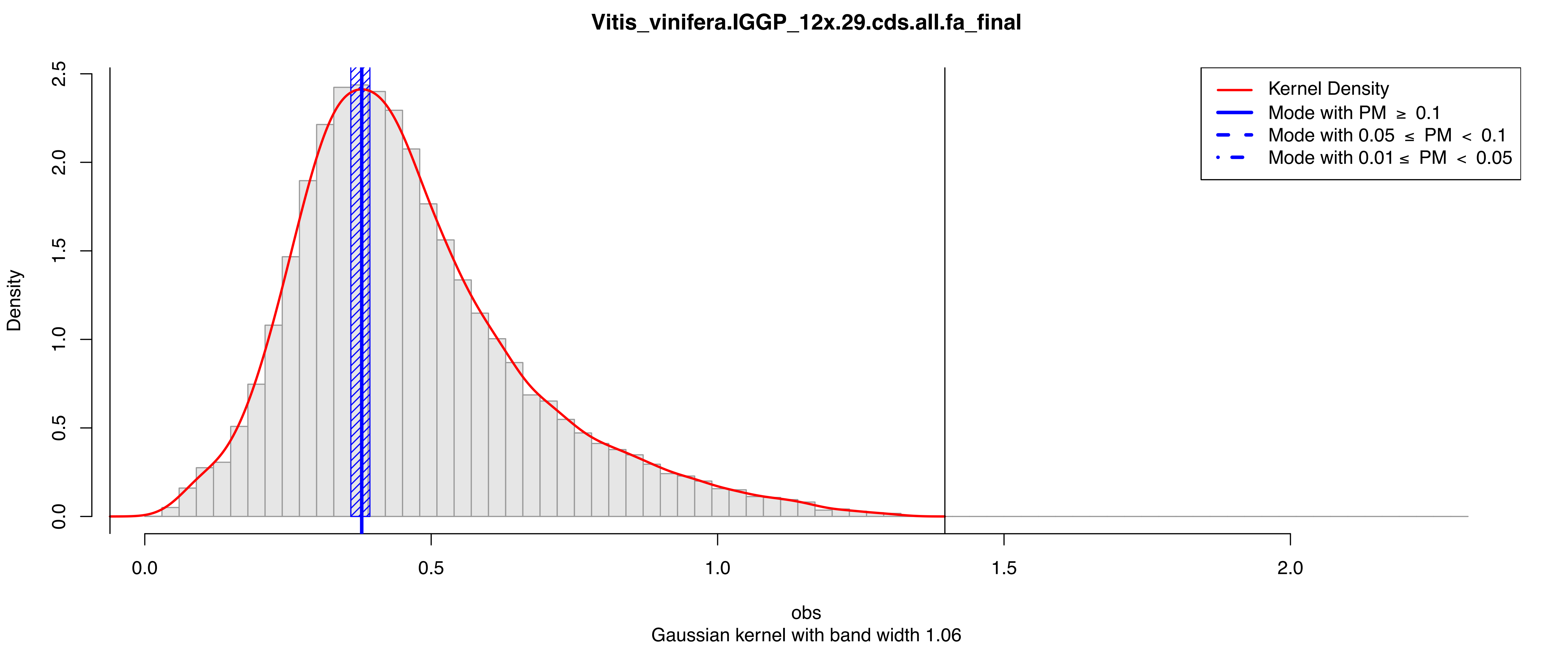
Vitis_vinifera.IGGP_12x.29.cds.all.fa_final
Density
0.0
0.5
1.0
1.5
2.0
2.5
Kernel Density
Mode with PM ≥ 0.1
Mode with 0.05 ≤ PM < 0.1
Mode with 0.01 ≤ PM < 0.05
0.0
0.5
1.0
1.5
2.0
obs
Gaussian kernel with band width 1.06

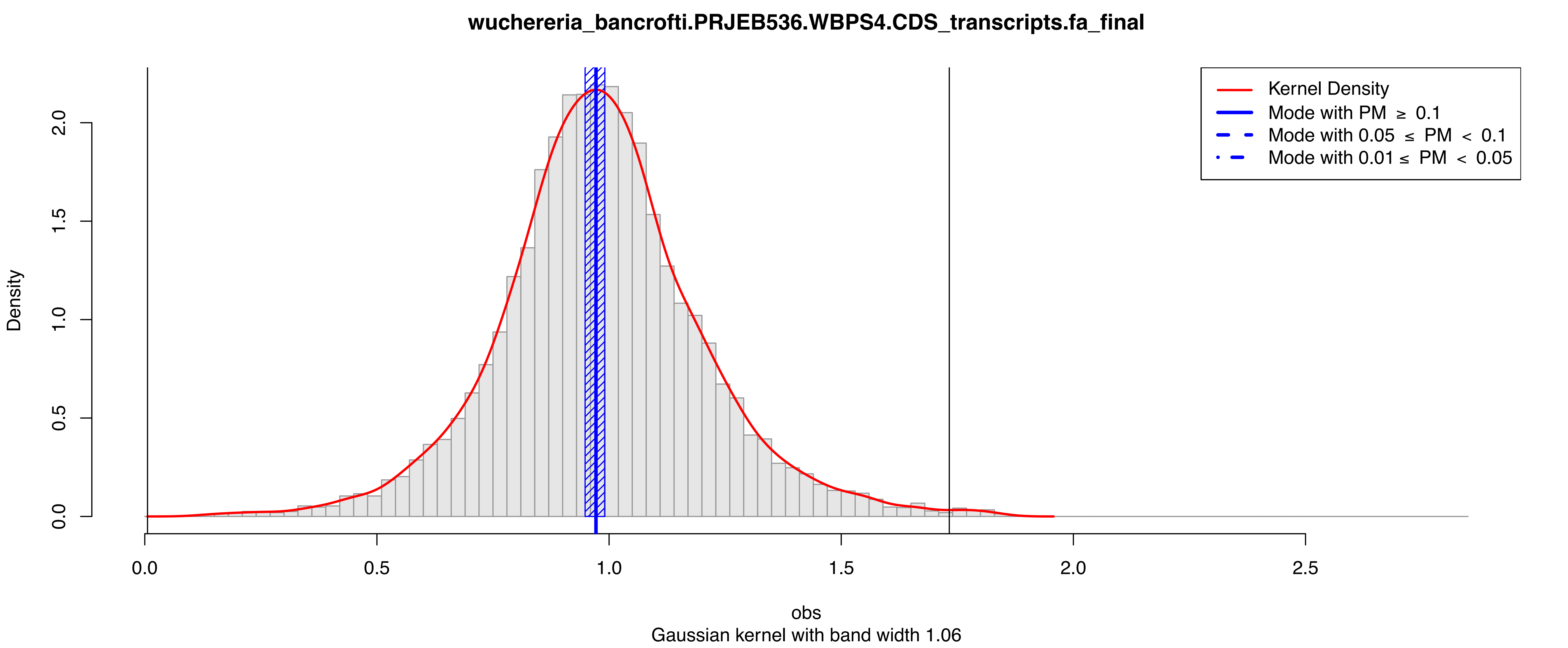

wuchereria_bancrofti.PRJEB536.WBPS4.CDS_transcripts.fa_final
Kernel Density
Mode with PM ≥ 0.1
Mode with 0.05 ≤ PM < 0.1
Mode with 0.01 ≤ PM < 0.05
Density
0.0
0.5
1.0
1.5
2.0
2.5
obs
Gaussian kernel with band width 1.06

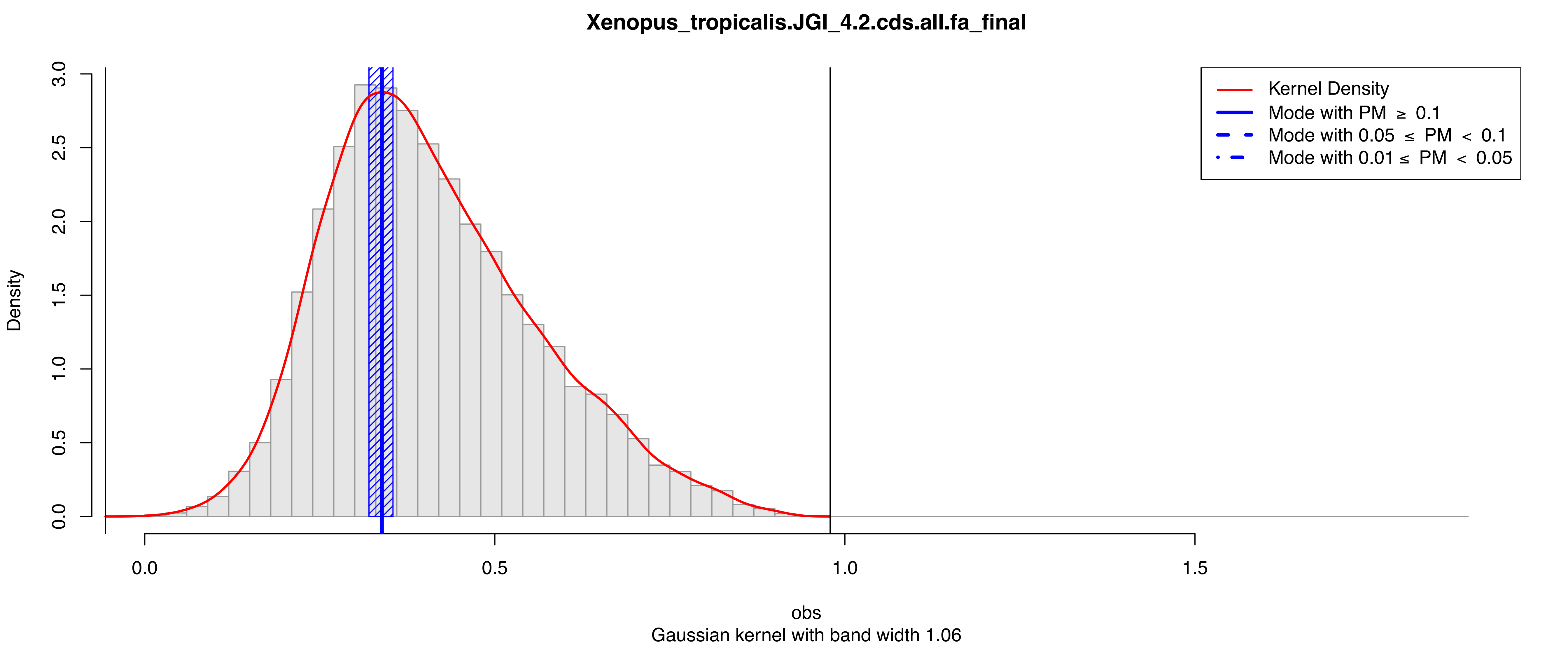

Xenopus_tropicalis.JGI_4.2.cds.all.fa_final
Kernel Density
Mode with PM ≥ 0.1
Mode with 0.05 ≤ PM < 0.1
Mode with 0.01 ≤ PM < 0.05
Density
0.0
0.5
1.0
1.5
2.0
2.5
3.0
0.0
0.5
1.0
1.5
obs
Gaussian kernel with band width 1.06

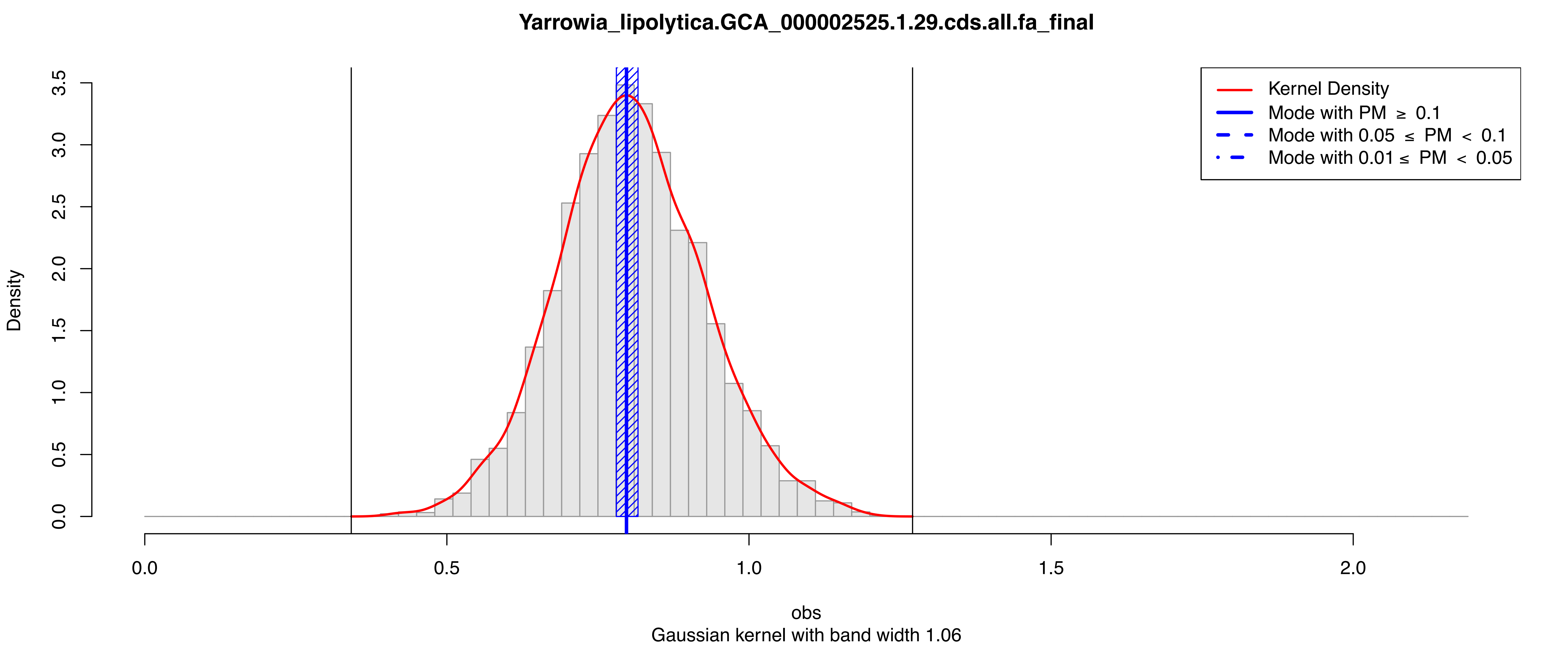
Yarrowia_lipolytica.GCA_000002525.1.29.cds.all.fa_final
Kernel Density
Mode with PM ≥ 0.1
Mode with 0.05 ≤ PM < 0.1
Mode with 0.01 ≤ PM < 0.05
Density
3.5
3.0
2.5
2.0
1.5
1.0
0.5
0.0
0.0
0.5
1.0
1.5
2.0
obs
Gaussian kernel with band width 1.06

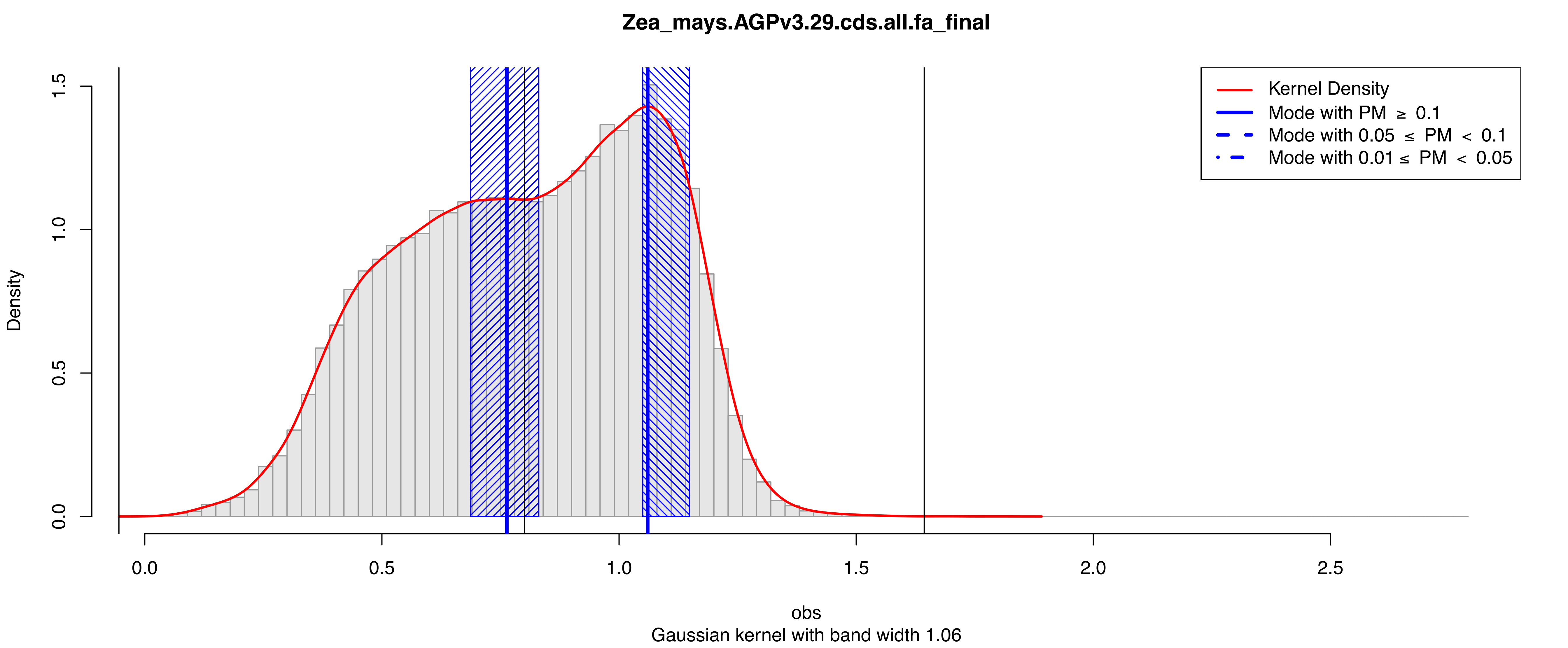
Zea_mays.AGPv3.29.cds.all.fa_final
Kernel Density
Mode with PM ≥ 0.1
Mode with 0.05 ≤ PM < 0.1
Mode with 0.01 ≤ PM < 0.05
Density
0.0
0.5
1.0
1.5
0.0
0.5
1.0
1.5
2.0
2.5
obs
Gaussian kernel with band width 1.06

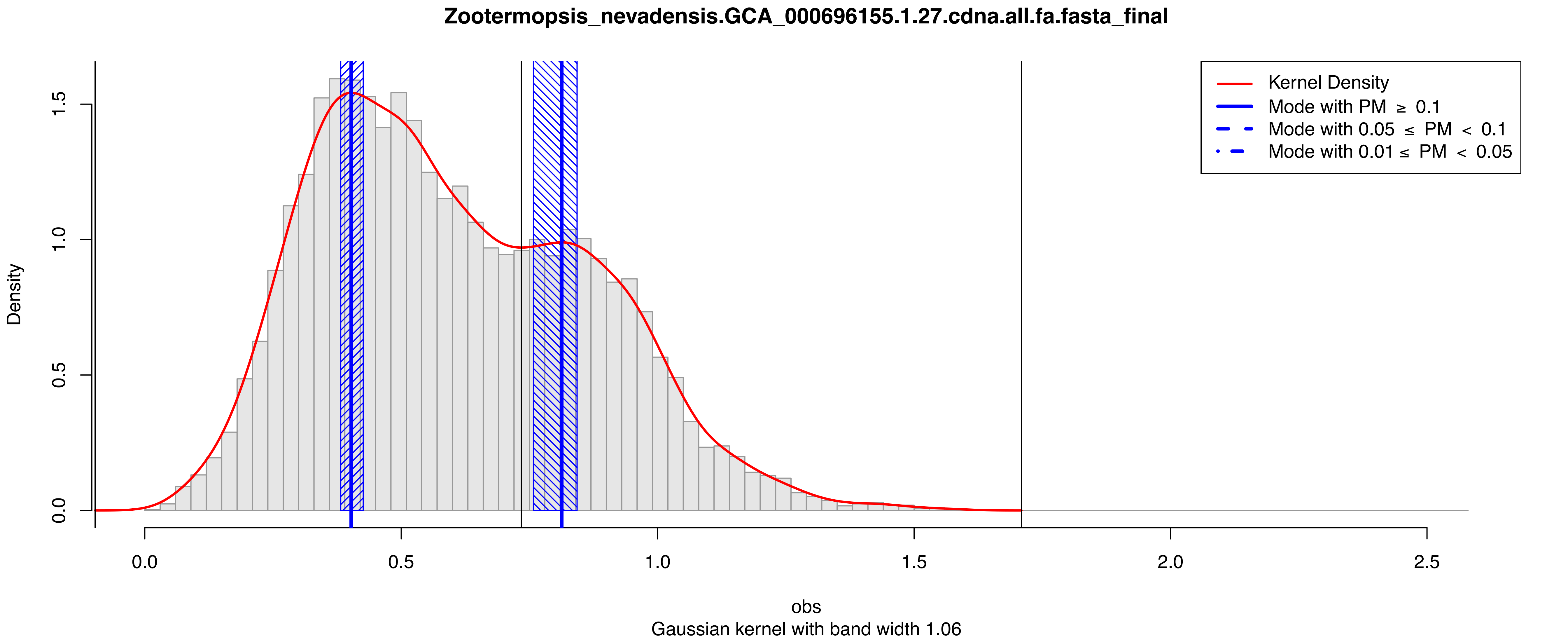
Zootermopsis_nevadensis.GCA_000696155.1.27.cdna.all.fa.fasta_final
Kernel Density
Mode with PM ≥ 0.1
Mode with 0.05 ≤ PM < 0.1
Mode with 0.01 ≤ PM < 0.05
Density
0.0
0.5
1.0
1.5
2.0
2.5
obs
Gaussian kernel with band width 1.06

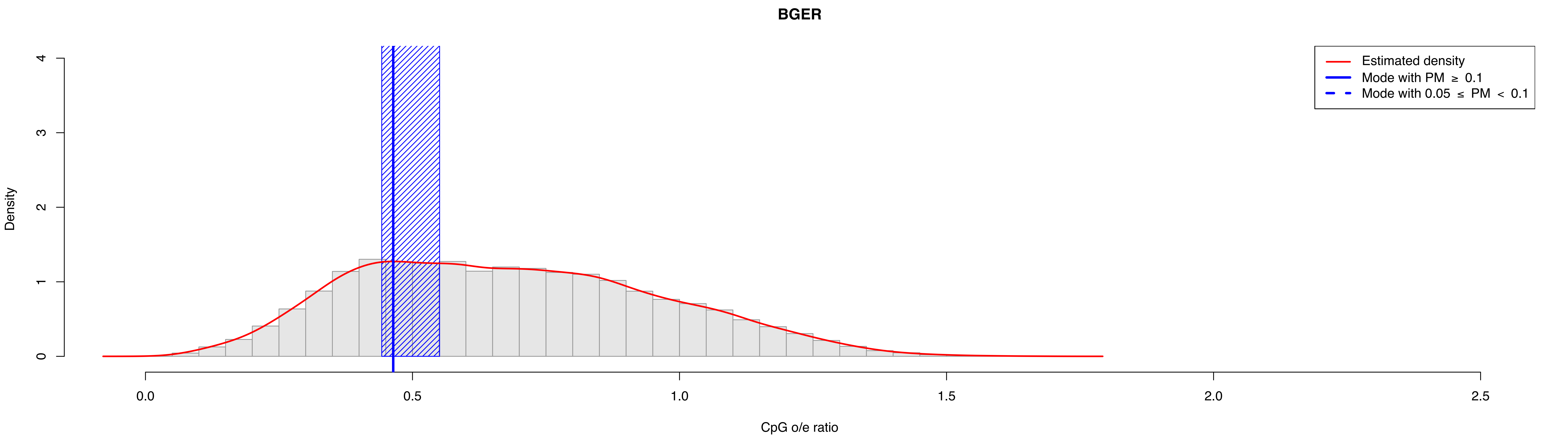
BGER
Density
0
1
2
3
4
0.0
0.5
1.0
1.5
2.0
2.5
CpG o/e ratio
Estimated density
Mode with PM ≥ 0.1
Mode with 0.05 ≤ PM < 0.1
