# Supplementary material for: Universality of the DNA methylation codes in Eucaryotes
Source: Sci Rep. 2019 Jan 17;9:173. doi: 10.1038/s41598-018-37407-8 (PMC6336885; doi:10.1038/s41598-018-37407-8)

Comparison between databases

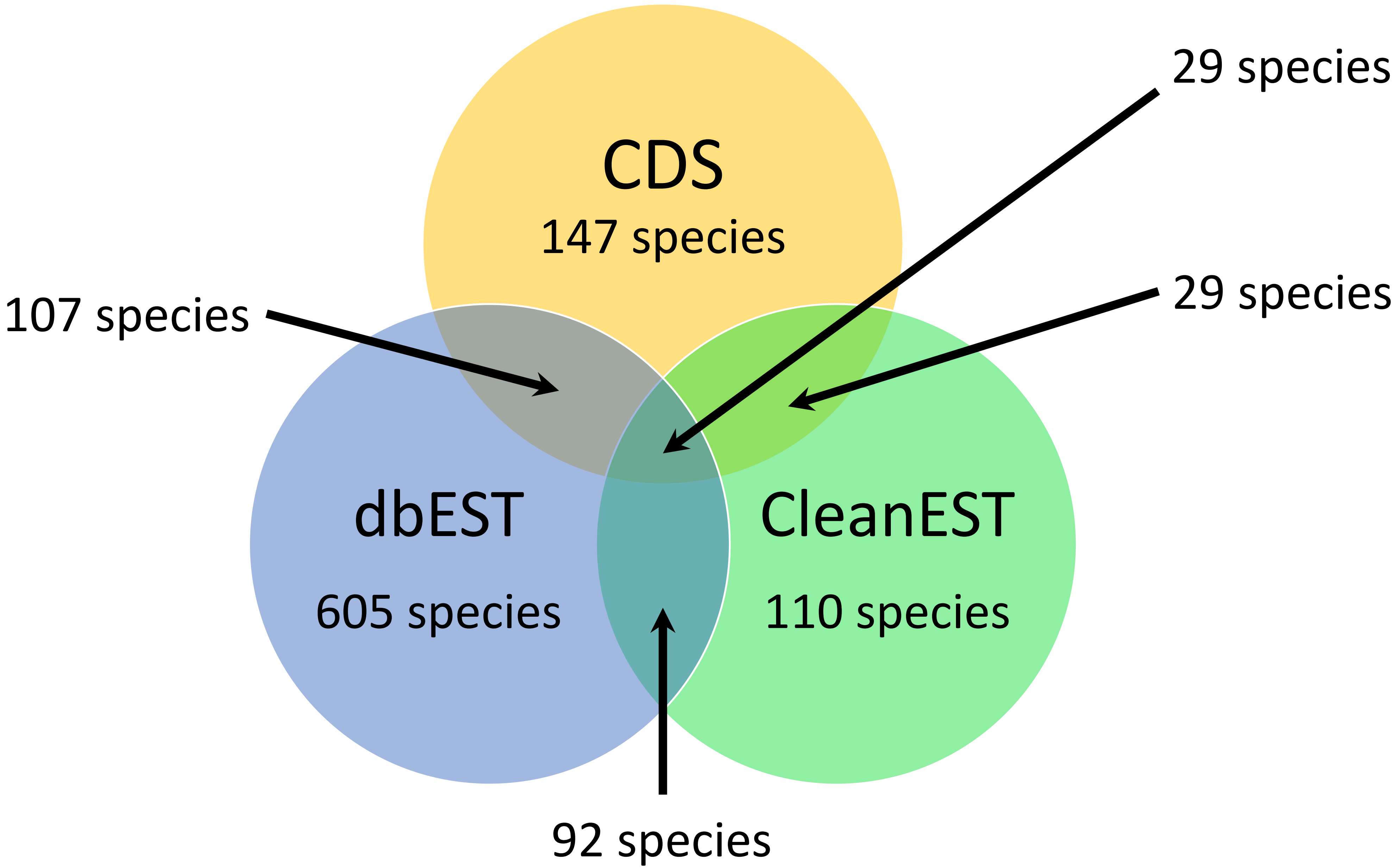

1

Trichoplax adhaerens

CDS

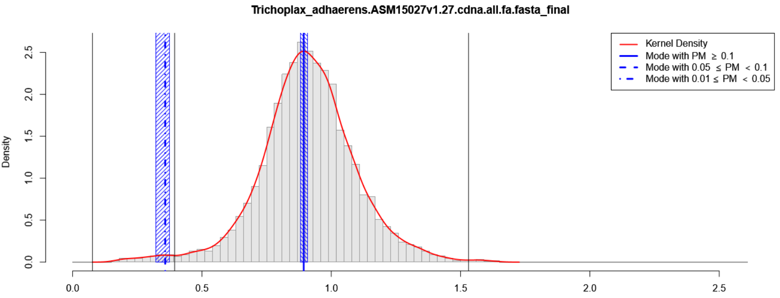

dbEST

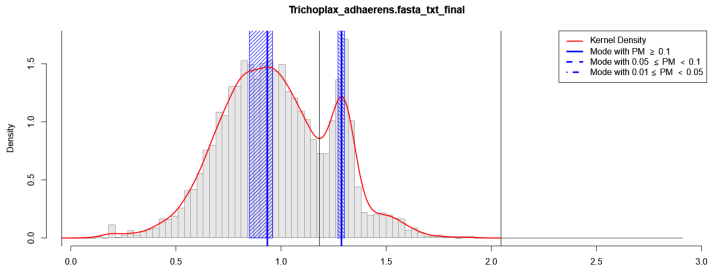

CleanEST

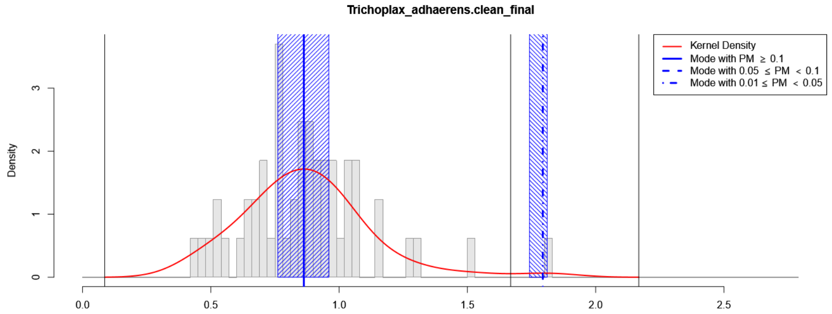

2

Daphnia pulex

CDS

dbEST

CleanEST

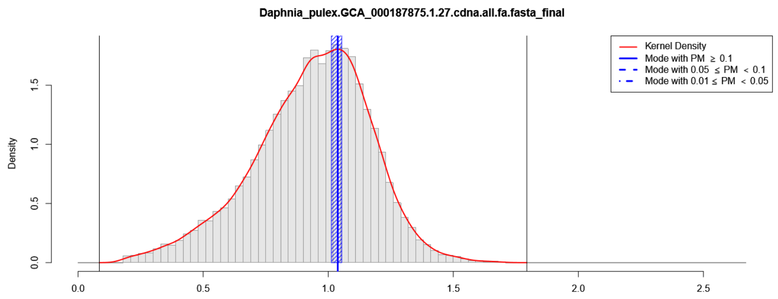

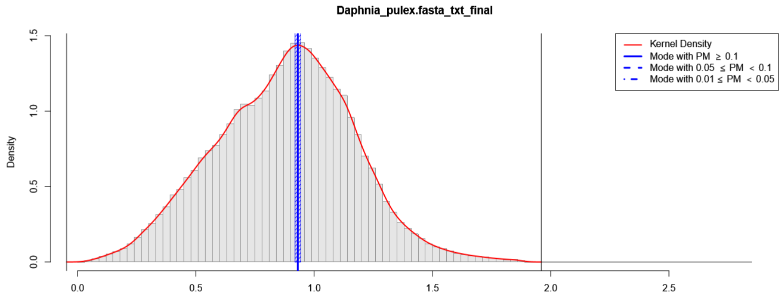

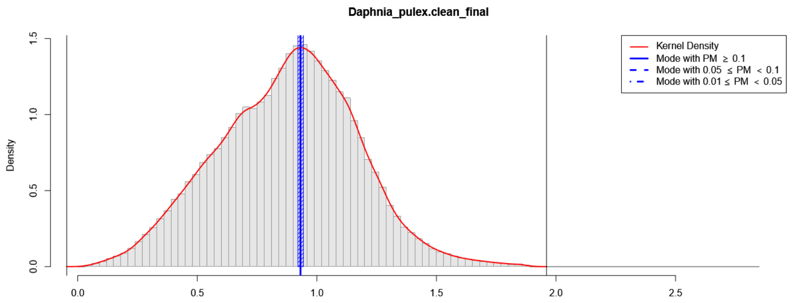

3

Mnemiopsis leidyi

CDS

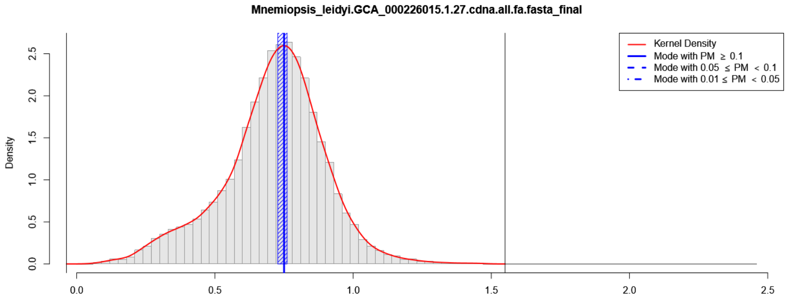

dbEST

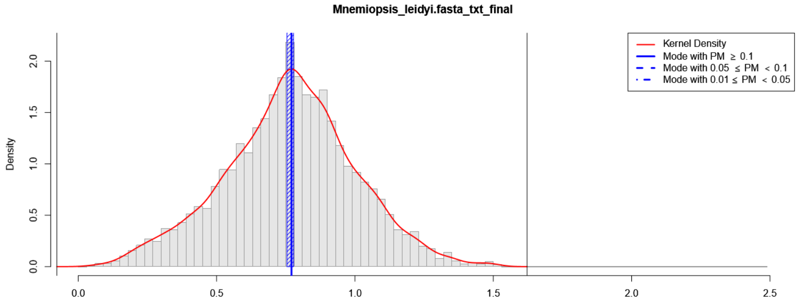

CleanEST

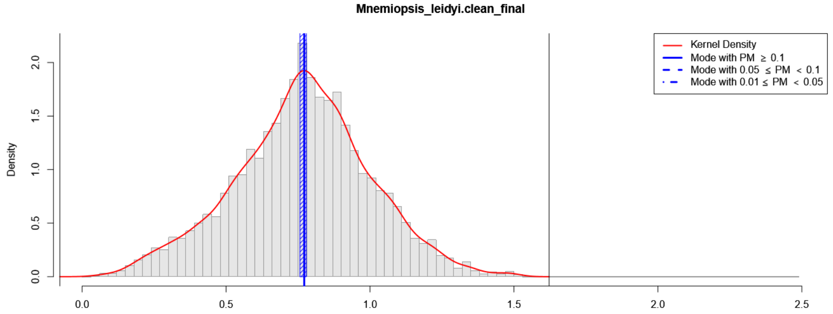

4

Nematostella vectensis

CDS

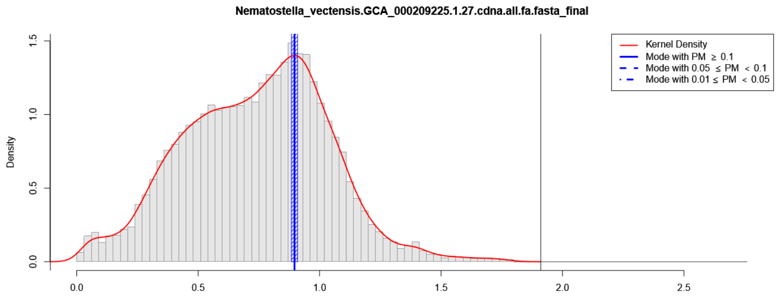

dbEST

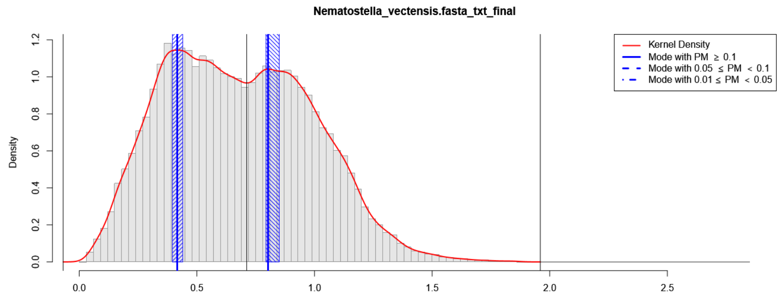

CleanEST

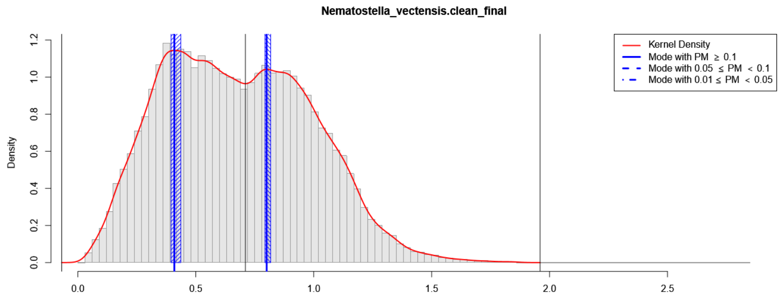

5

Helobdella robusta

CDS

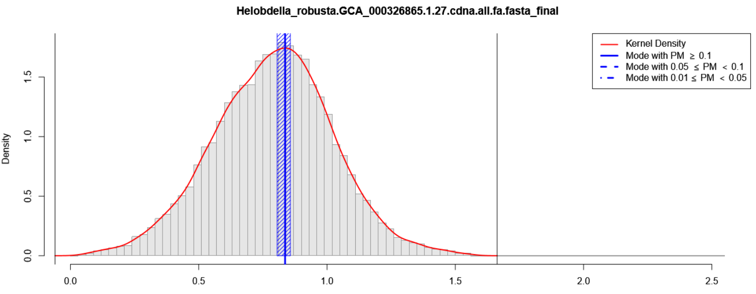

dbEST

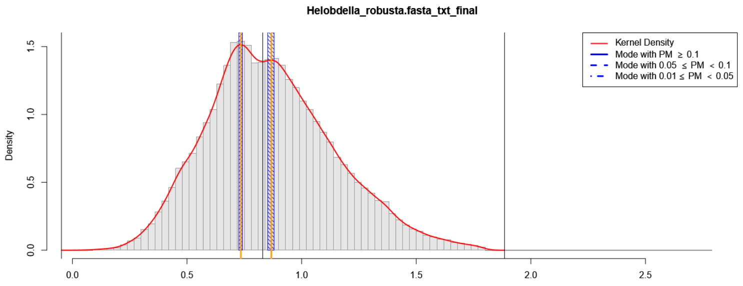

CleanEST

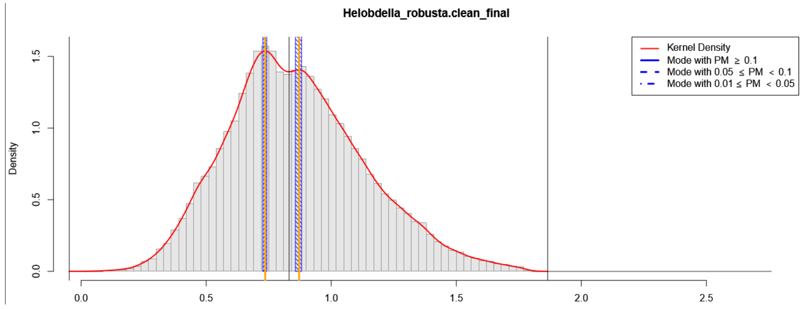

6

Crassostrea gigas

CDS

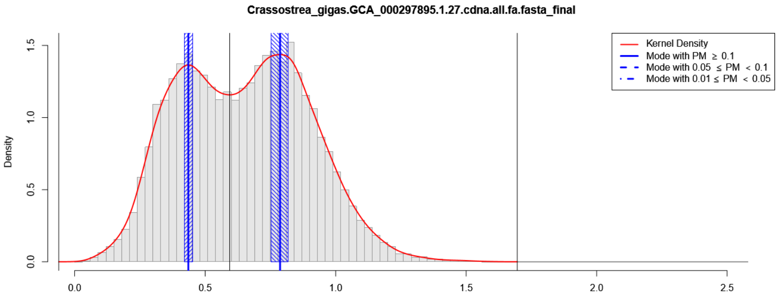

dbEST

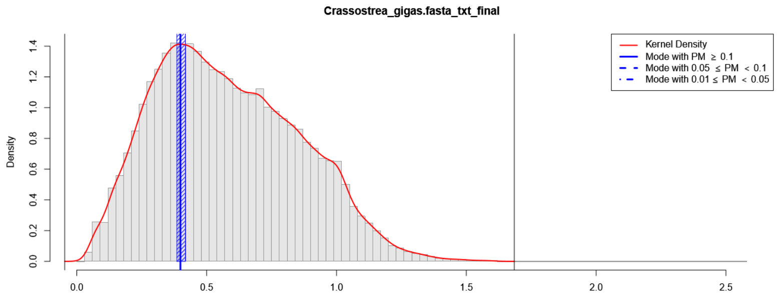

CleanEST

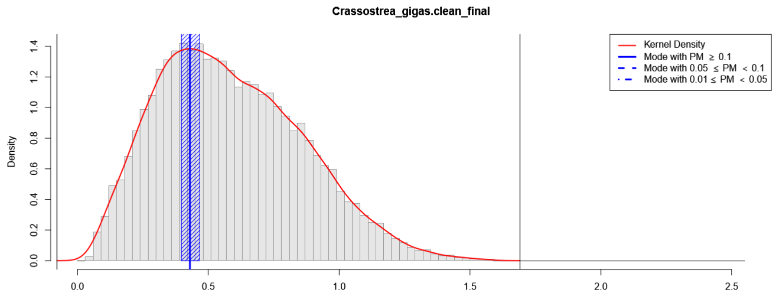

7

Lottia gigantea

CDS

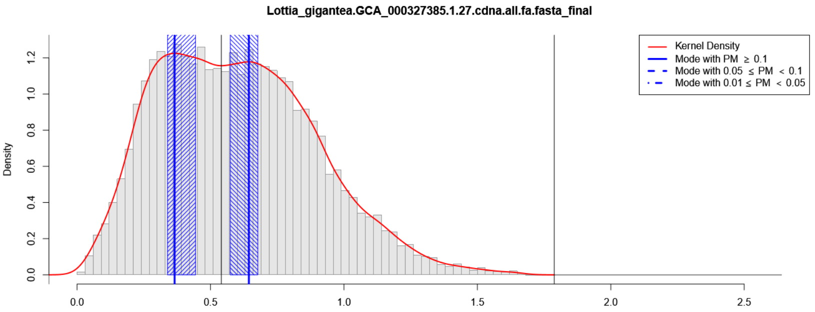

dbEST

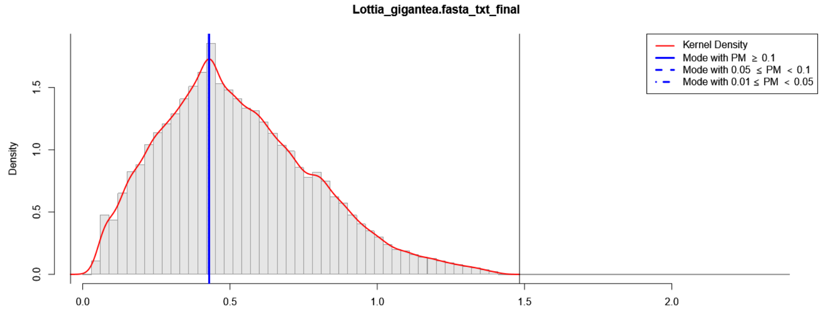

CleanEST

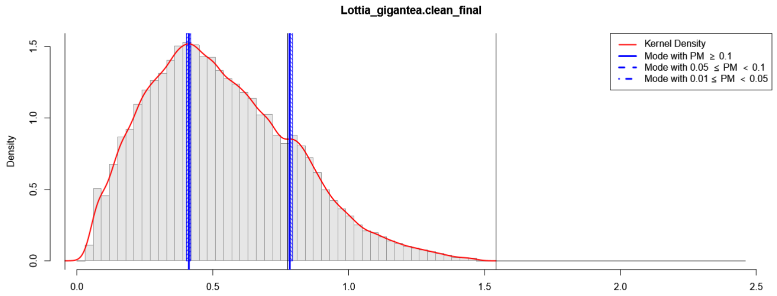

8

Aedes aegypti

CDS

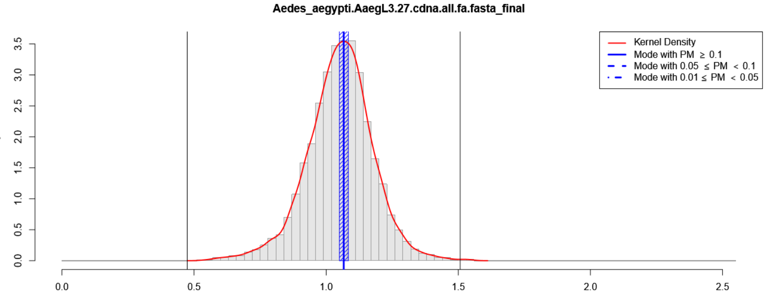

dbEST

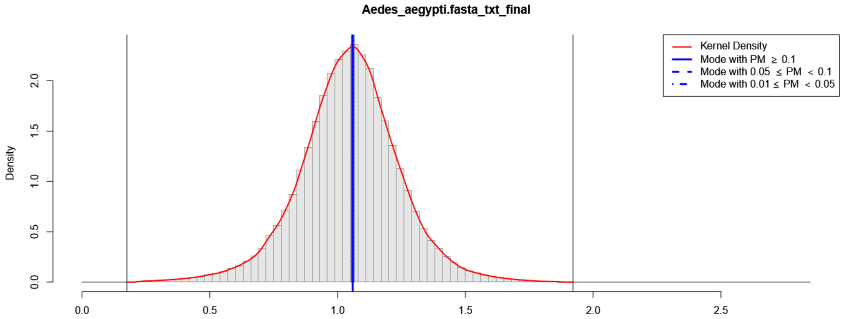

CleanEST

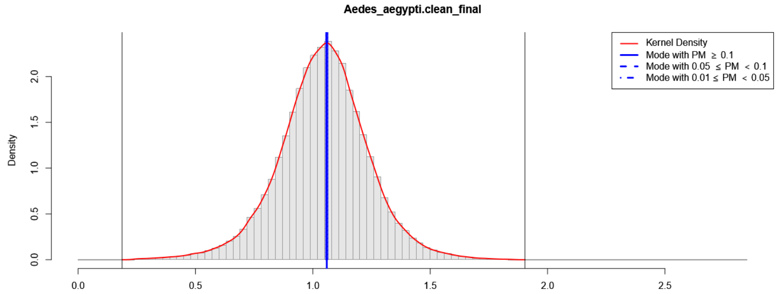

9

Drosophila ananassae

CDS

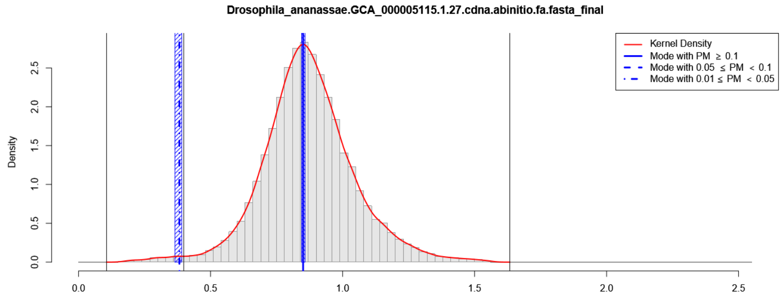

dbEST

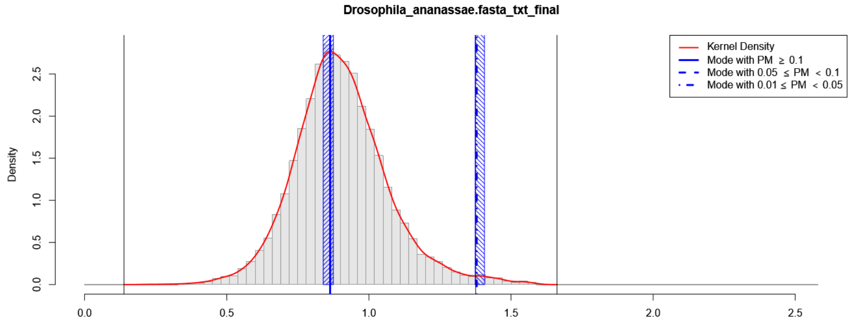

CleanEST

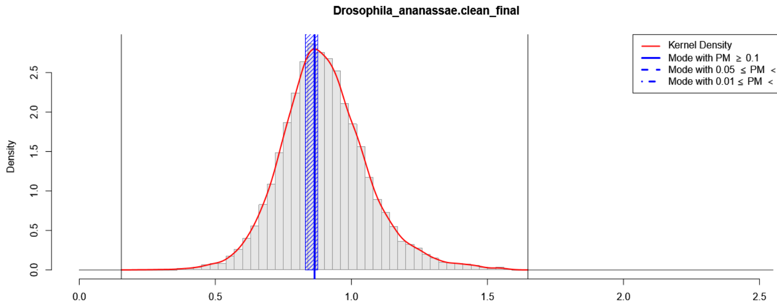

10

Drosophila melanogaster

CDS

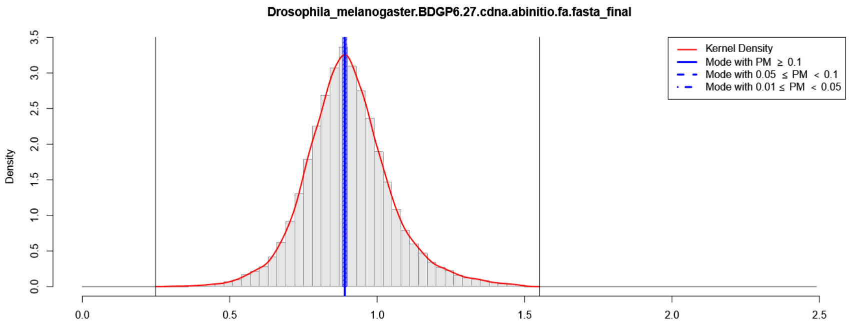

dbEST

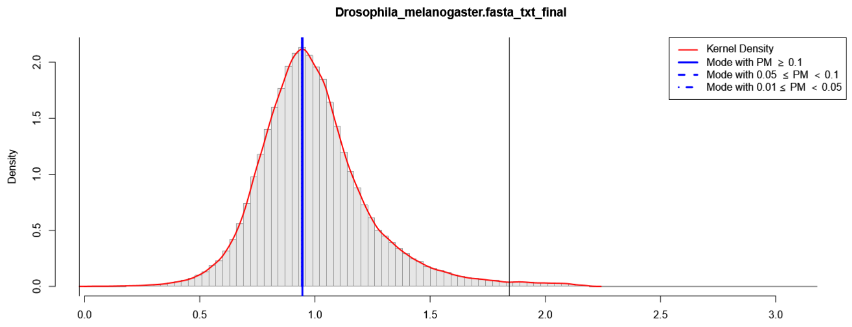

CleanEST

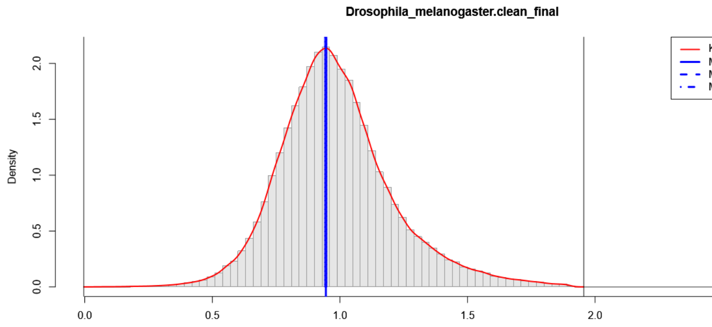

11

Anopheles darlingi

CDS

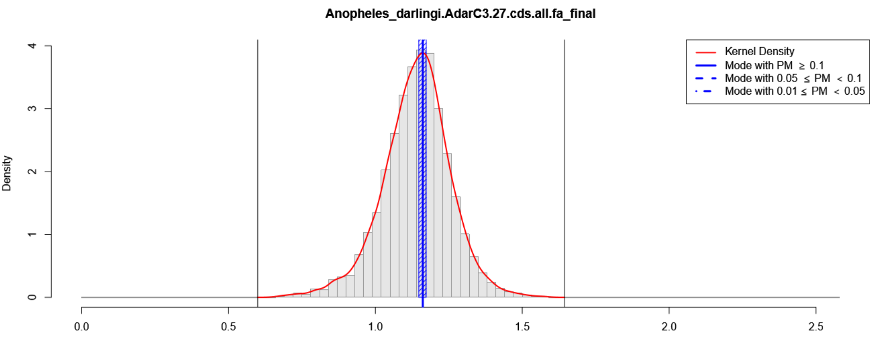

dbEST

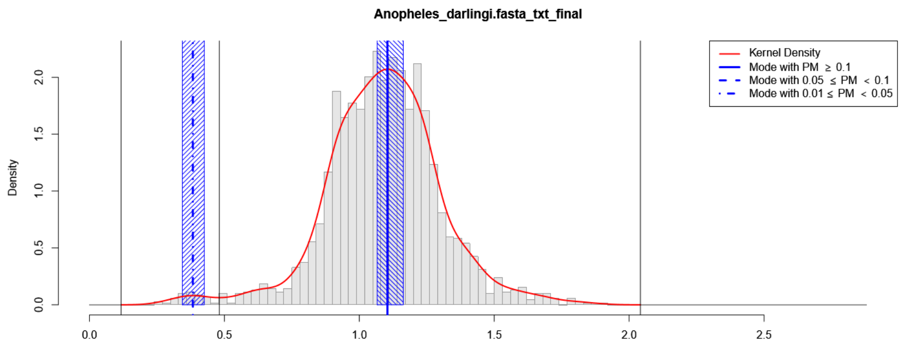

CleanEST

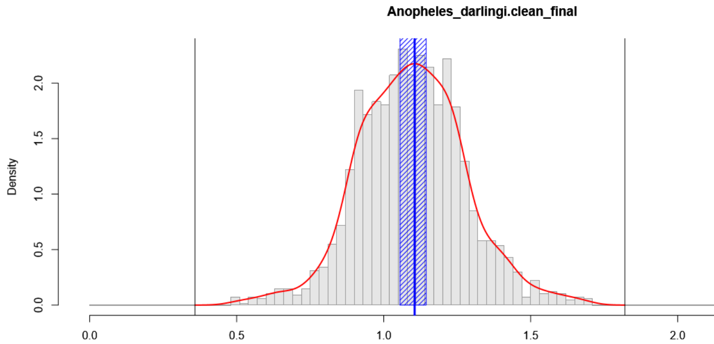

12

Anopheles gambiae

CDS

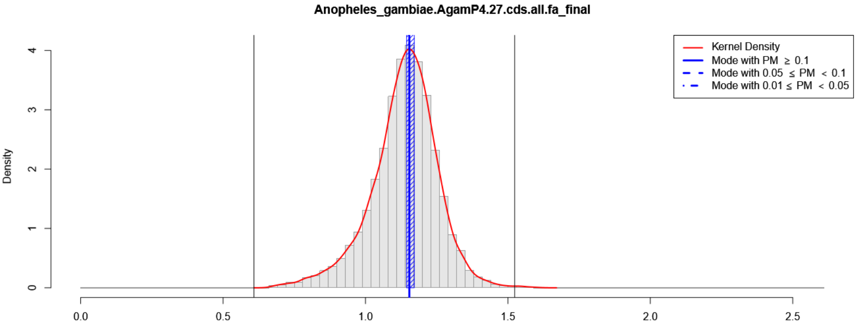

dbEST

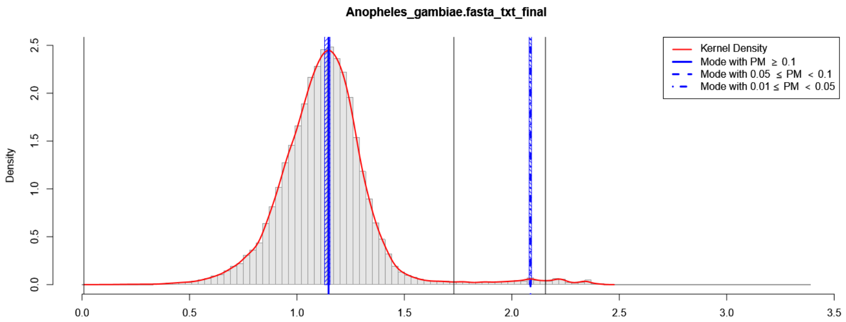

CleanEST

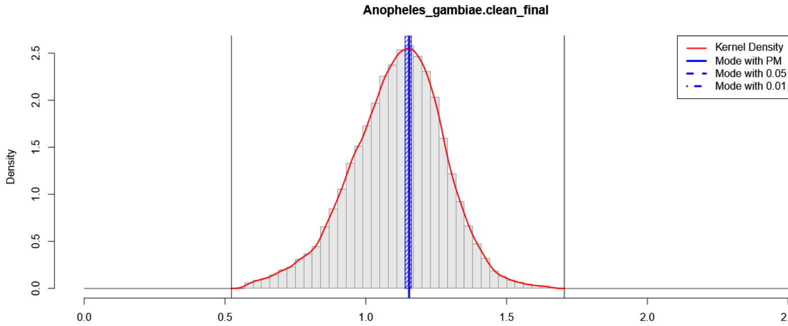

13

Ixodes scapularis

CDS

dbEST

CleanEST

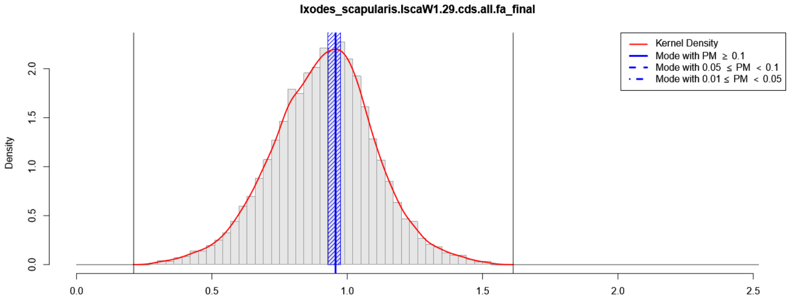

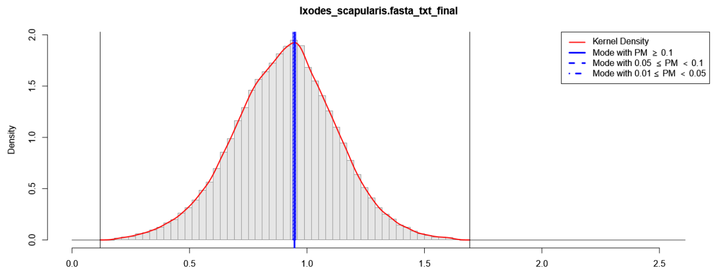

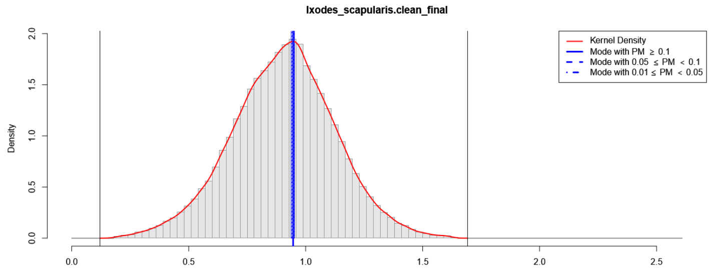

14

Nasonia vitripennis

CDS

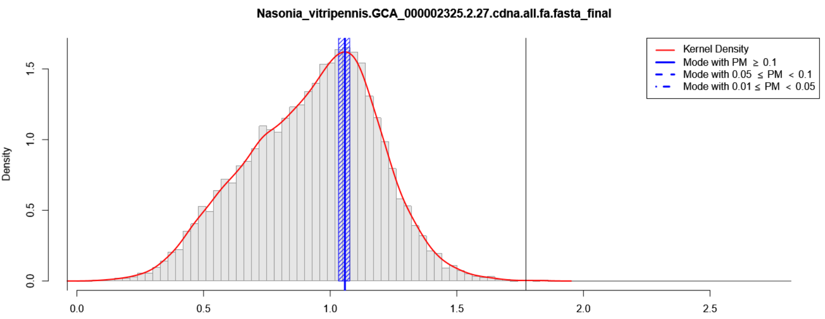

dbEST

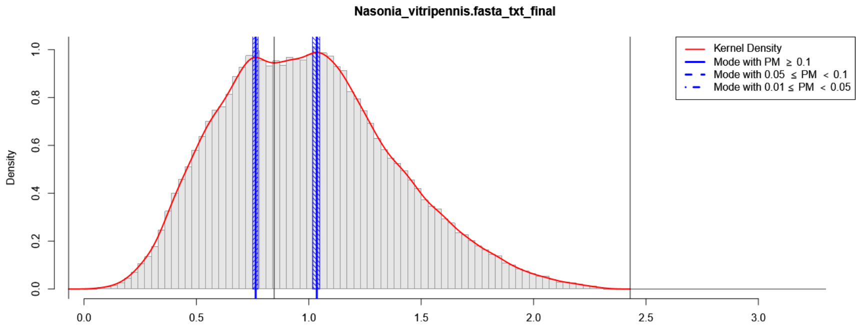

CleanEST

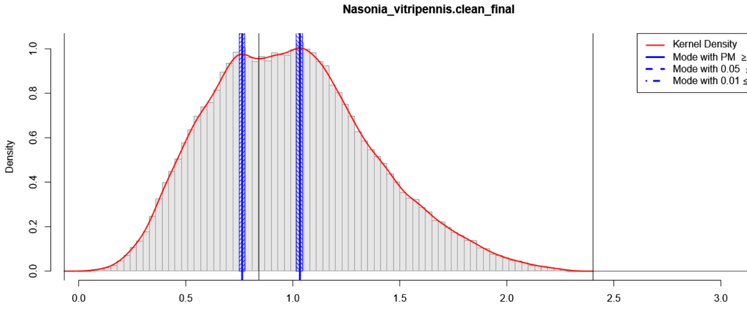

Apis mellifera

15

CDS

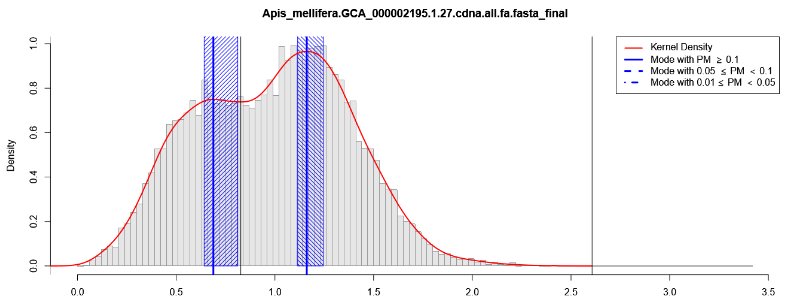

dbEST

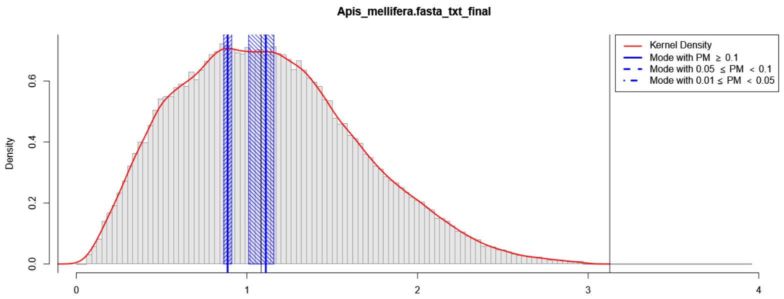

CleanEST

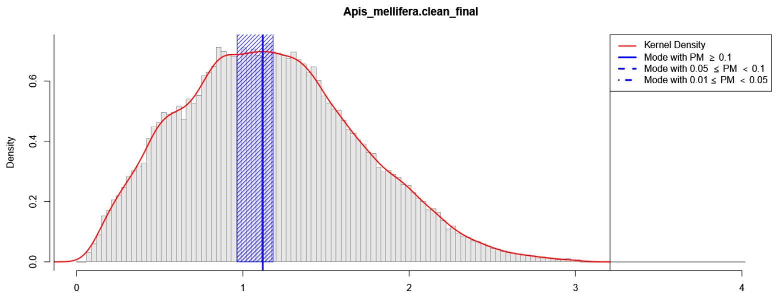

Solenopsis invicta

CDS

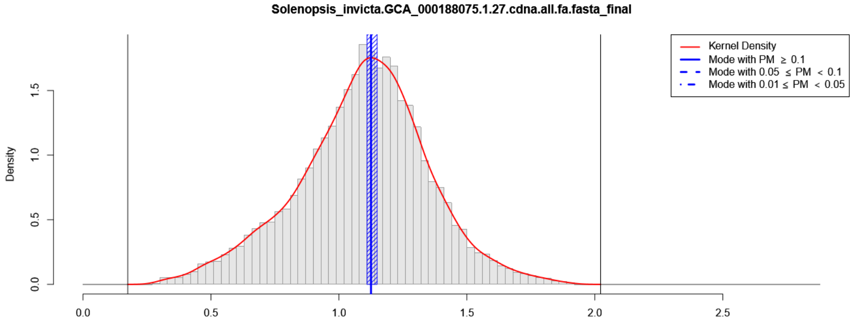

dbEST

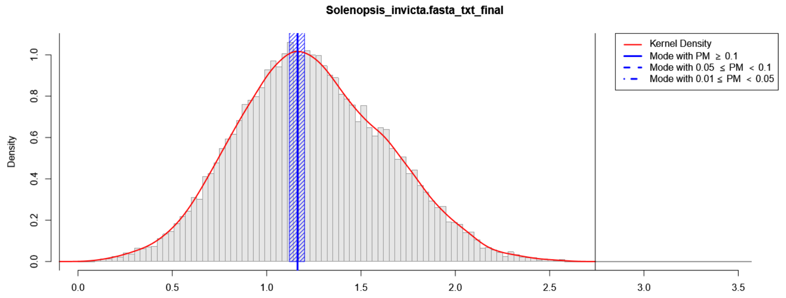

CleanEST

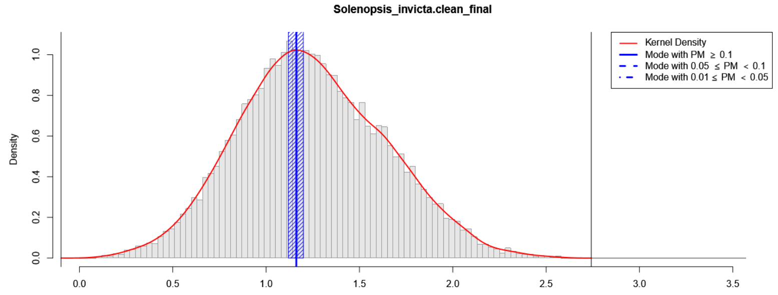

17

Acyrthosiphon pisum

CDS

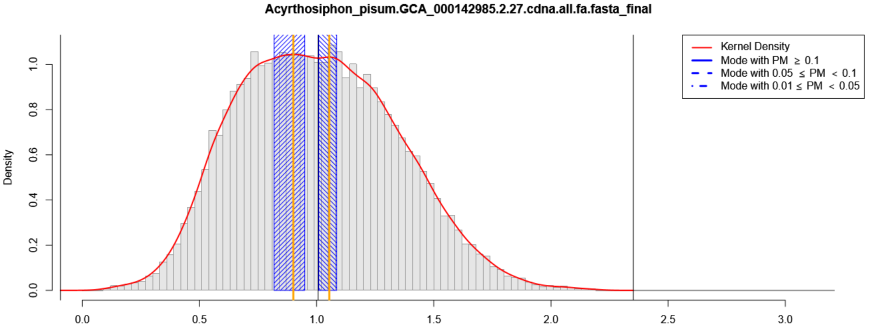

dbEST

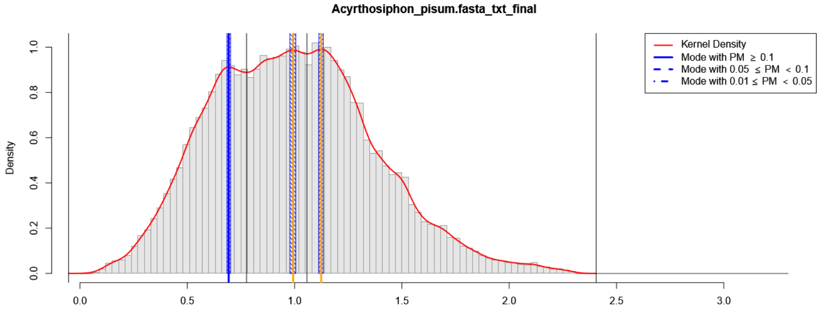

CleanEST

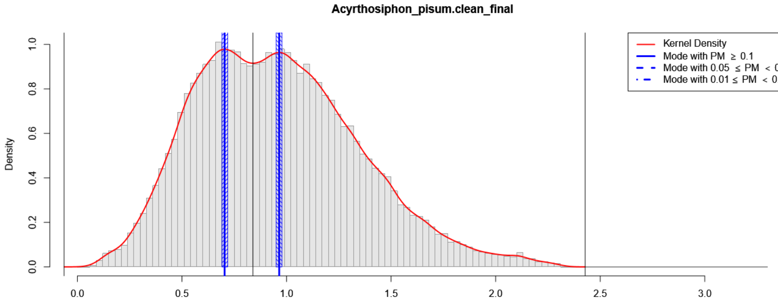

18

Rhodnius prolixus

CDS

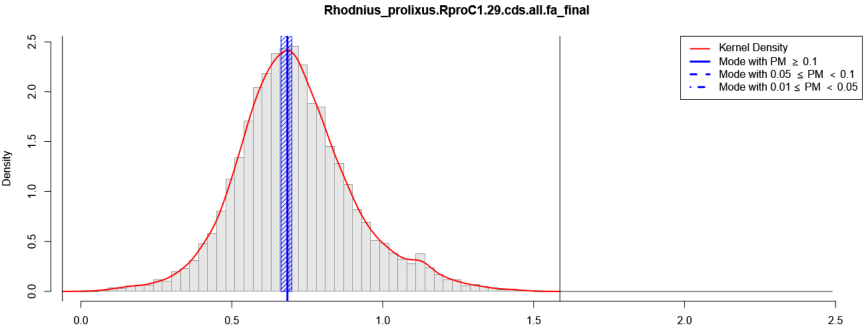

dbEST

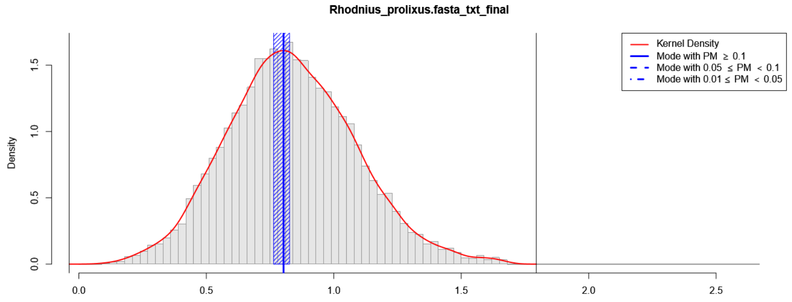

CleanEST

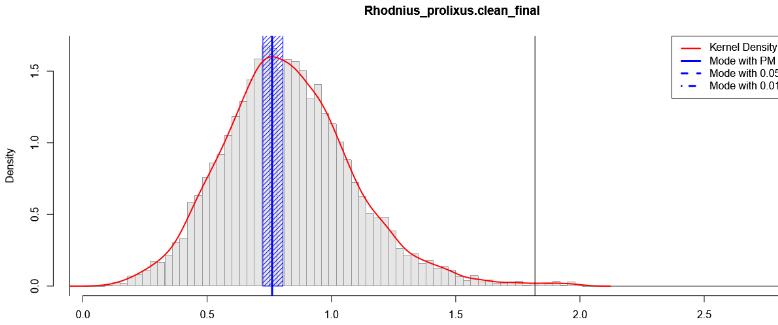

19

Bombyx mori

CDS

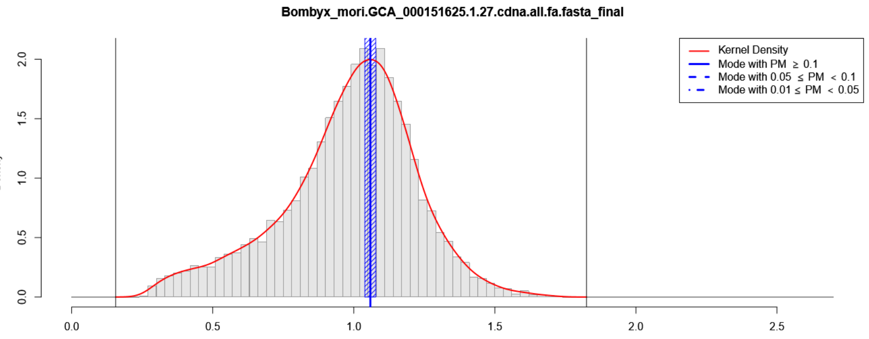

dbEST

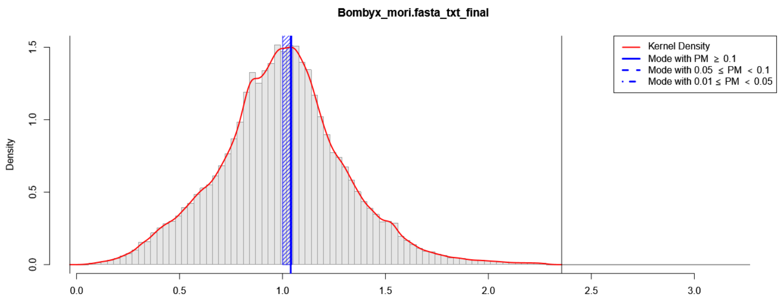

CleanEST

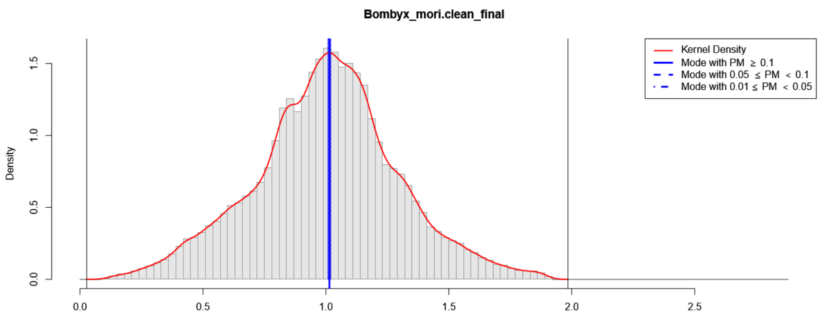

Ancylostoma caninum

CDS

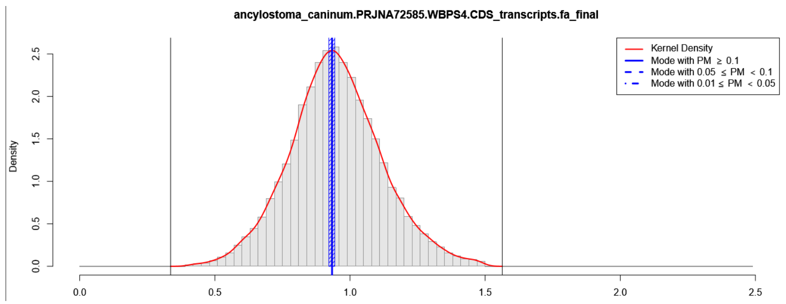

dbEST

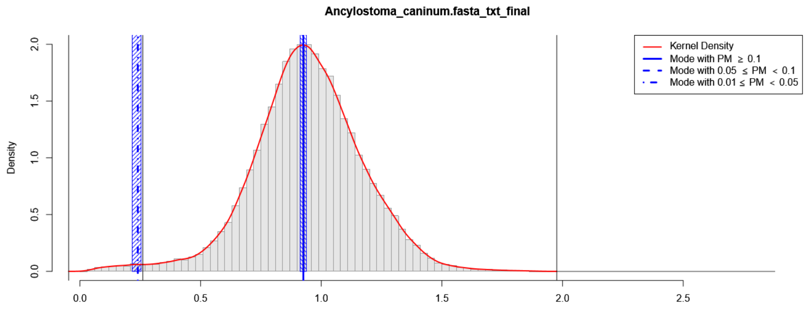

CleanEST

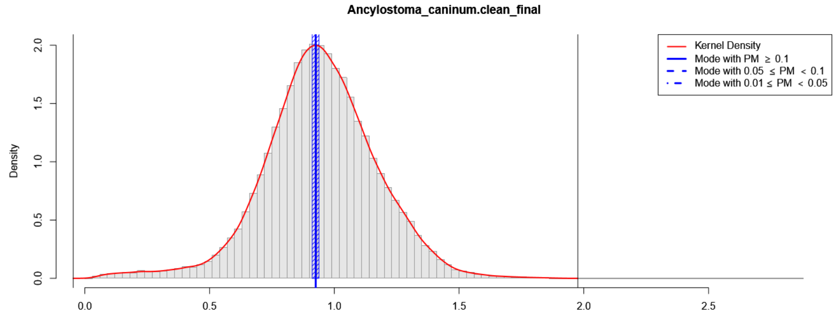

21

Ancylostoma ceylanicum

CDS

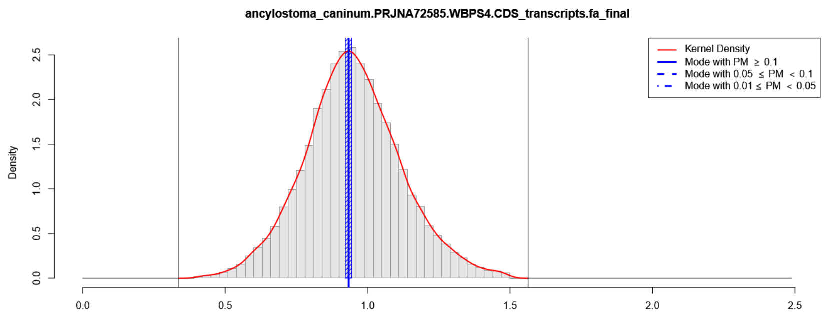

dbEST

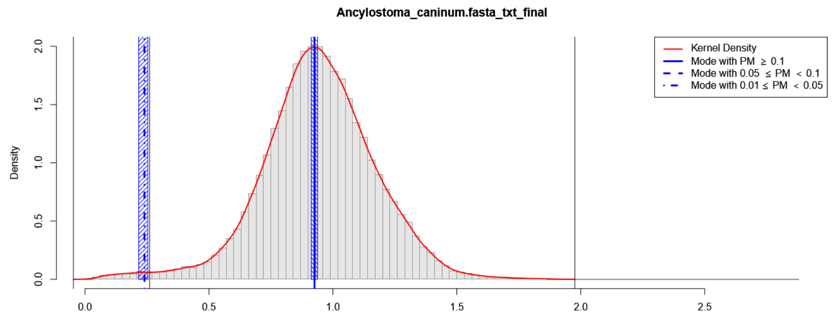

CleanEST

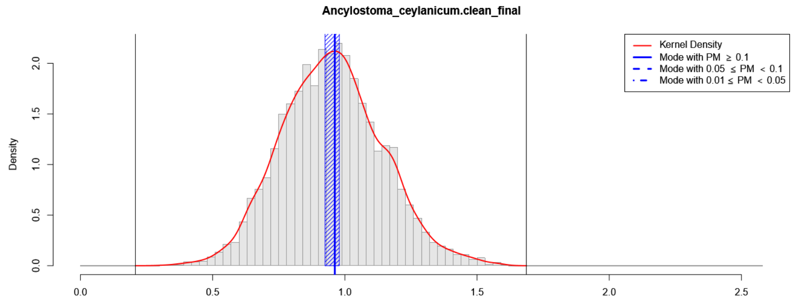

22

Cordyceps militaris

CDS

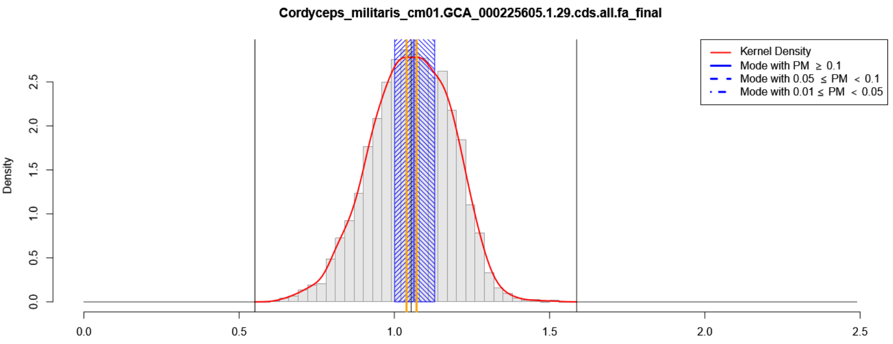

dbEST

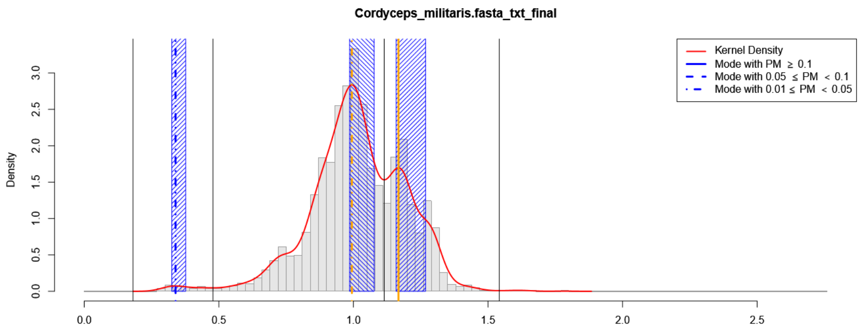

CleanEST

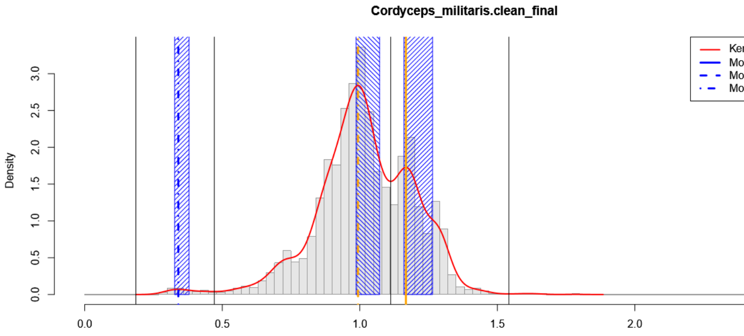

Strongylocentrotus purpuratus

23

CDS

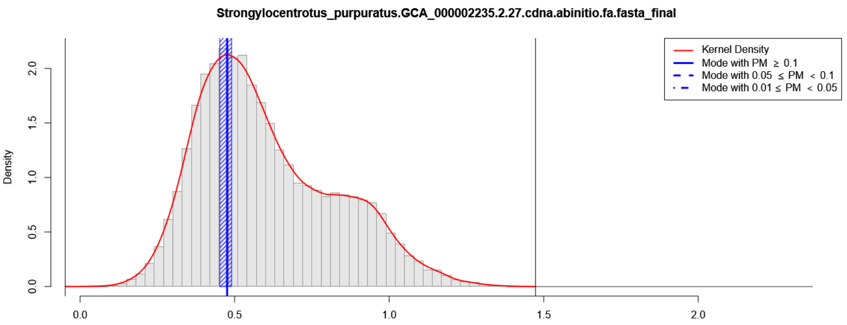

dbEST

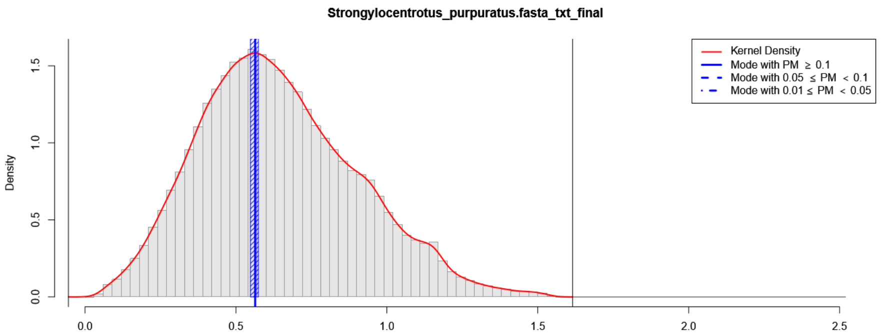

CleanEST

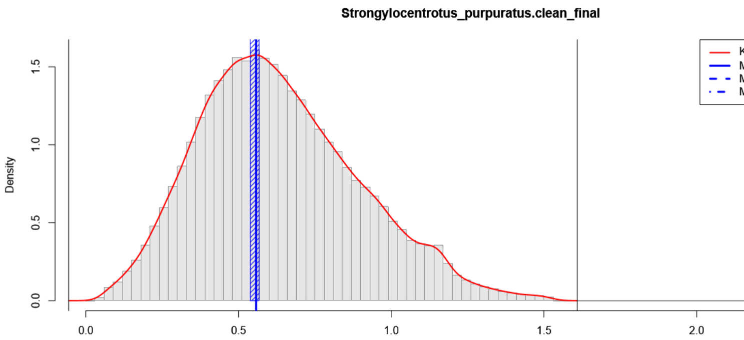

24

Ciona savignyi

CDS

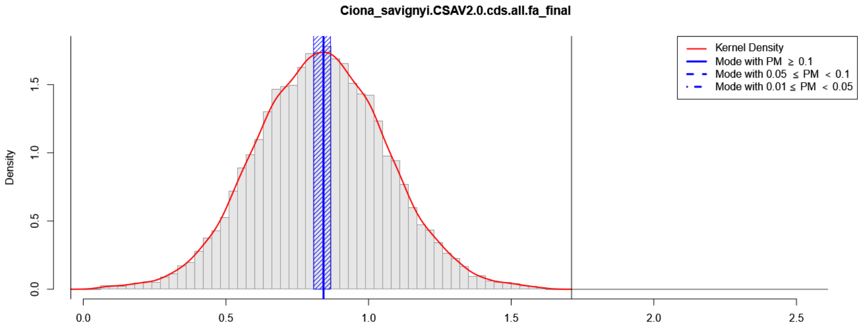

dbEST

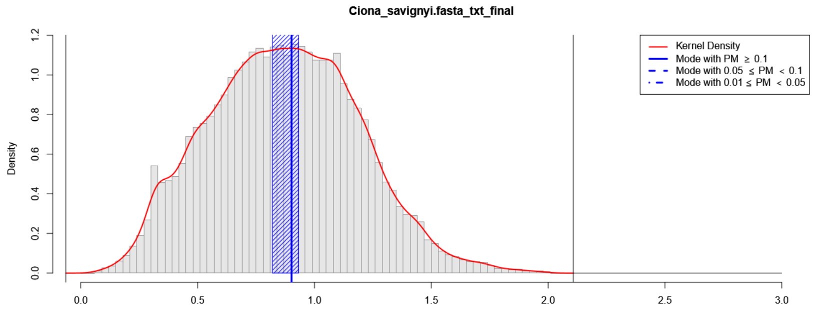

CleanEST

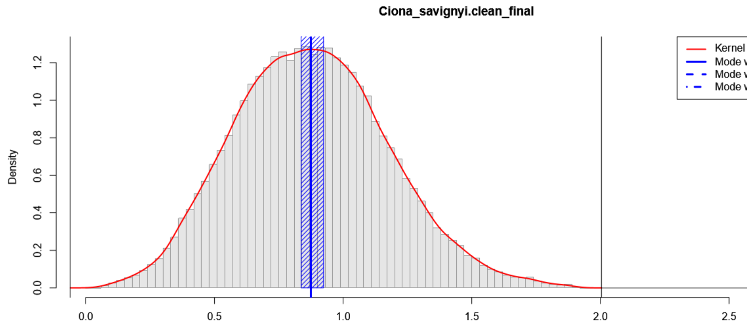

25

Ciona intestinalis

CDS

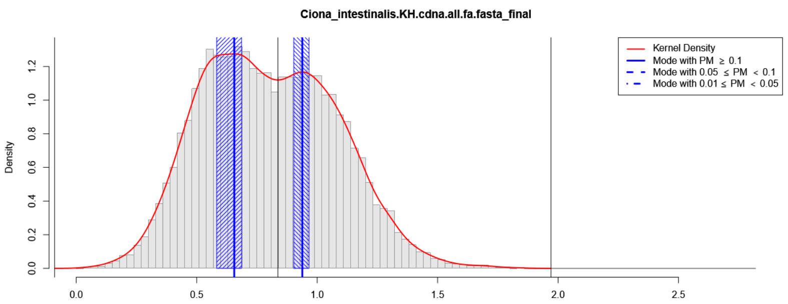

dbEST

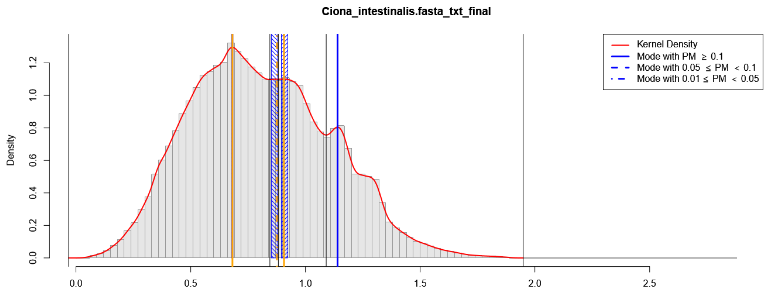

CleanEST

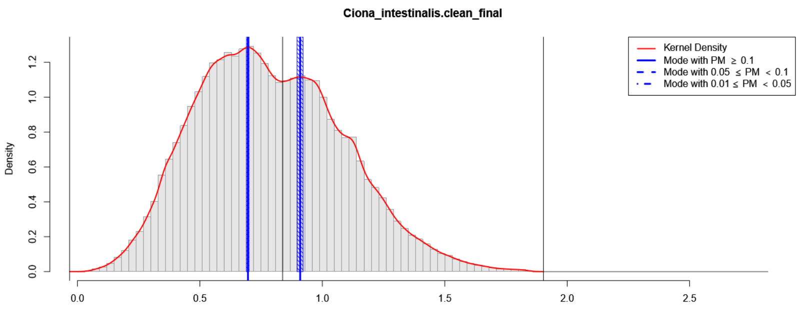

26

Anolis carolinensis

CDS

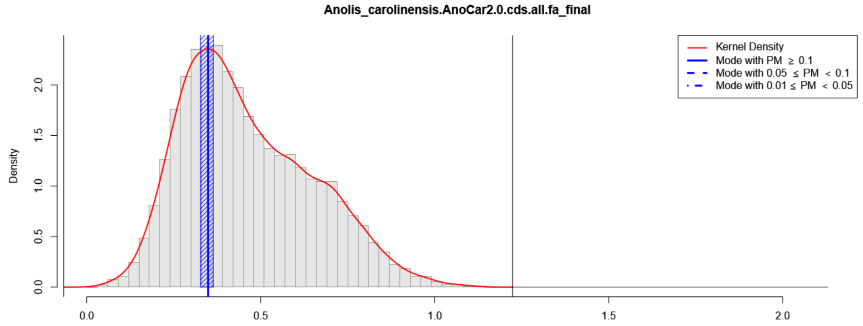

dbEST

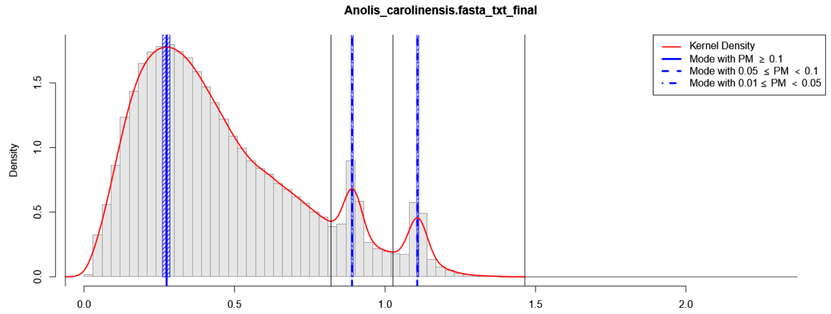

CleanEST

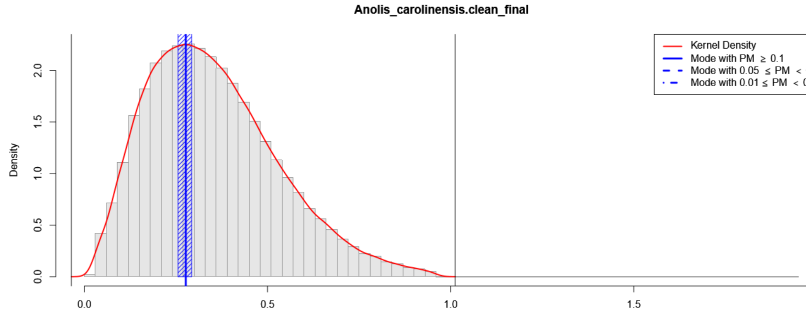

27

Danio rerio

CDS

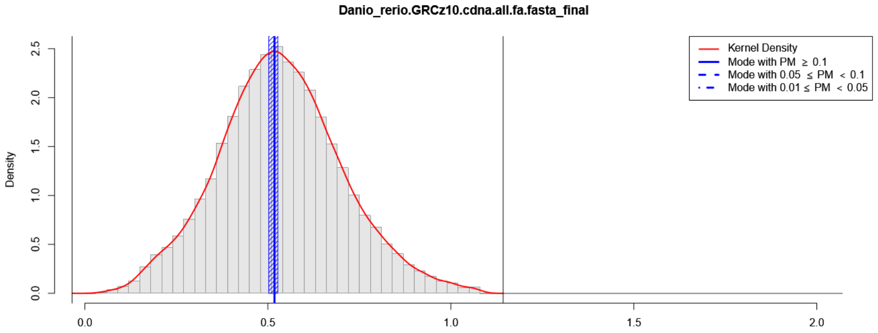

dbEST

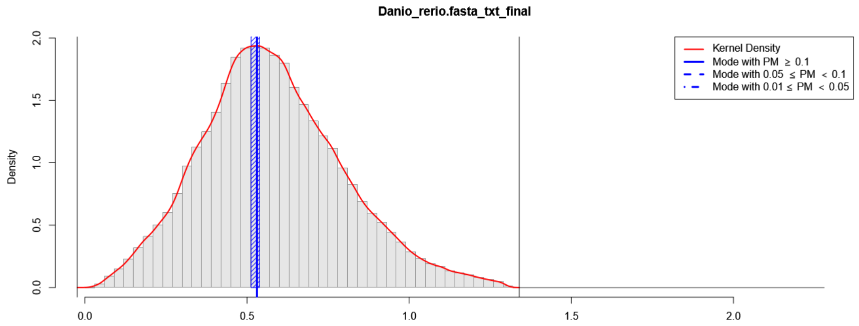

CleanEST

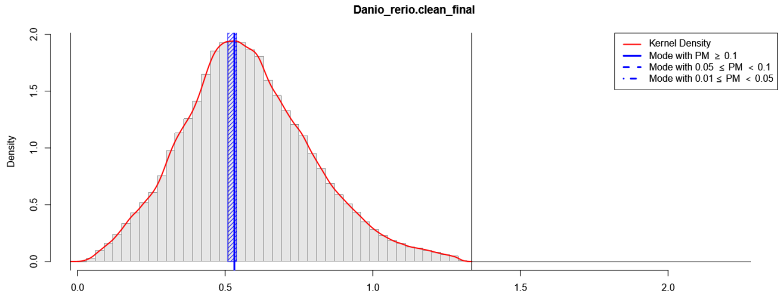

28

Gallus gallus

CDS

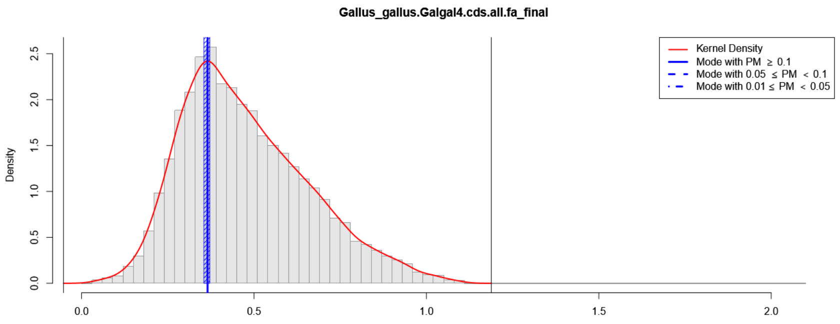

dbEST

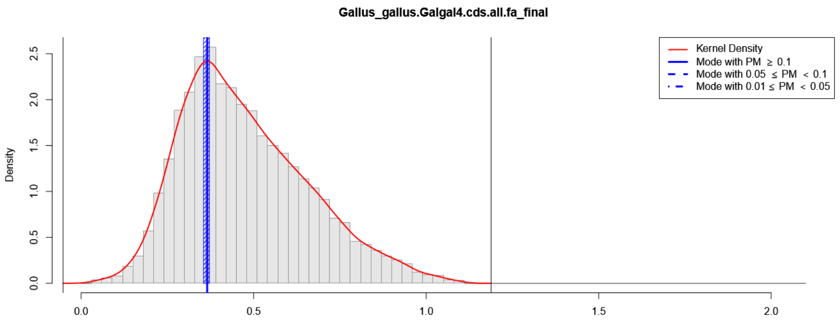

CleanEST

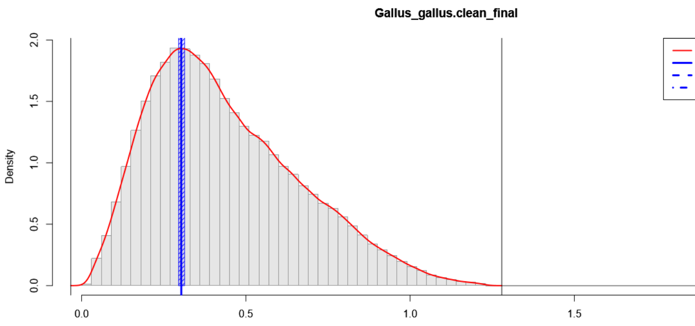

Anas platyrhynchos

CDS

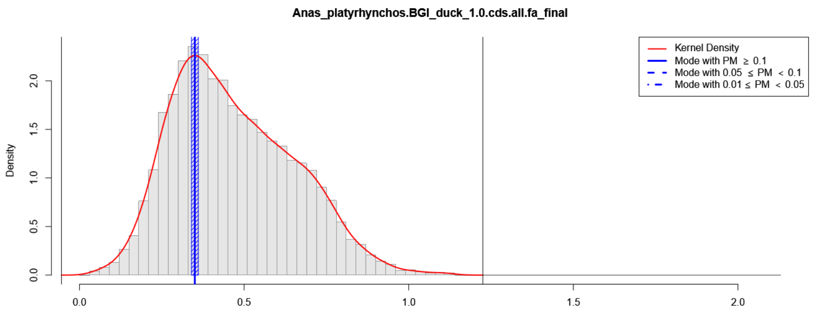

dbEST

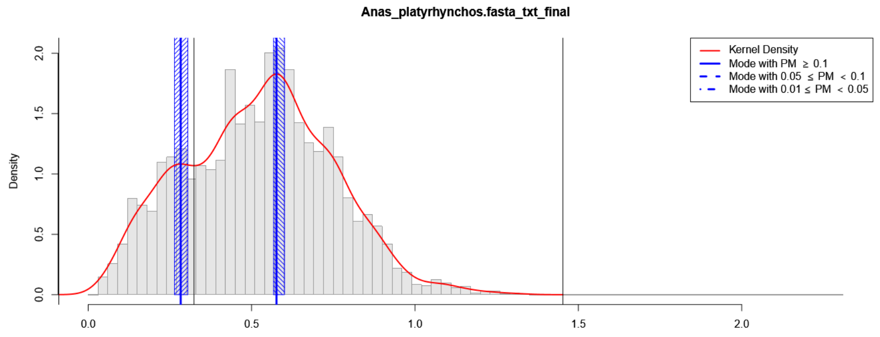

CleanEST

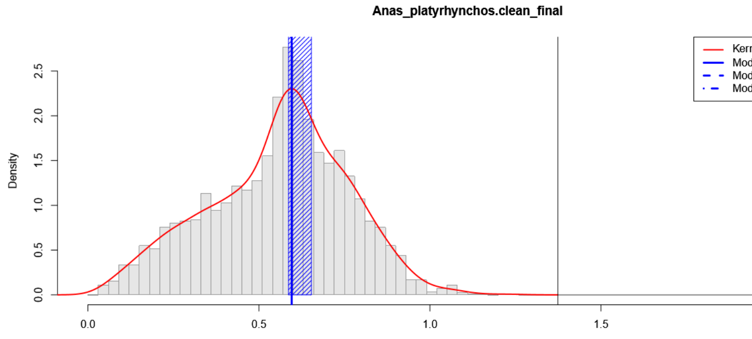

Supplement: Supplementary file 4 — Supplementary file 3 [file 41598_2018_37407_MOESM4_ESM.zip › Supplementary_files_03_database_comparison.pdf]
