# Supplementary material for: Universality of the DNA methylation codes in Eucaryotes
Source: Sci Rep. 2019 Jan 17;9:173. doi: 10.1038/s41598-018-37407-8 (PMC6336885; doi:10.1038/s41598-018-37407-8)
Supplement: Supplementary file 8 — Supplementary file 7 [file 41598_2018_37407_MOESM8_ESM.docx]

Universality of the DNA methylation codes in Eucaryotes

Benoît Aliaga, Ingo Bulla, Gabriel Mouahid, David Duval and Christoph Grunau

# Supp. File 7: How to install packages for cluster.r

For Ubuntu 16.04 and R (version 3.2.3 – “Wooden Christmas Tree”), you need to install some dependencies and R packages.

1. Open the terminal
2. Install libnlopt-dev library with this command line:

sudo apt-get install libnlopt-dev

1. Launch R and install nloptr and lme4 library

install.packages("nloptr")

install.packages("lme4")

1. Install the dendextend library and unlock it:

install.packages("dendextend", dependencies=TRUE, INSTALL_opts = c('--no-lock'))

1. Now you need to install the xlsx and plotrix packages

install.packages("xlsx")

install.packages("plotrix")

1. Quit R and launch the cluster.r in the terminal where the cluster.r is located with this command line:

Rscript cluster.r -0.1 0.75 0.15 Data_descriptives.csv KDE_Bootstrap_results.csv NCBI_TAXON_IDs.csv output.pdf
