# Supplementary material for: Universality of the DNA methylation codes in Eucaryotes
Source: Sci Rep. 2019 Jan 17;9:173. doi: 10.1038/s41598-018-37407-8 (PMC6336885; doi:10.1038/s41598-018-37407-8)
Supplement: Supplementary file 3 — Supplementary file 2 [file histogram_kde_dbEST.pdf › Supplementary_files_02_Histogram_databases_KDE/histogram_kde_dbEST.pdf]

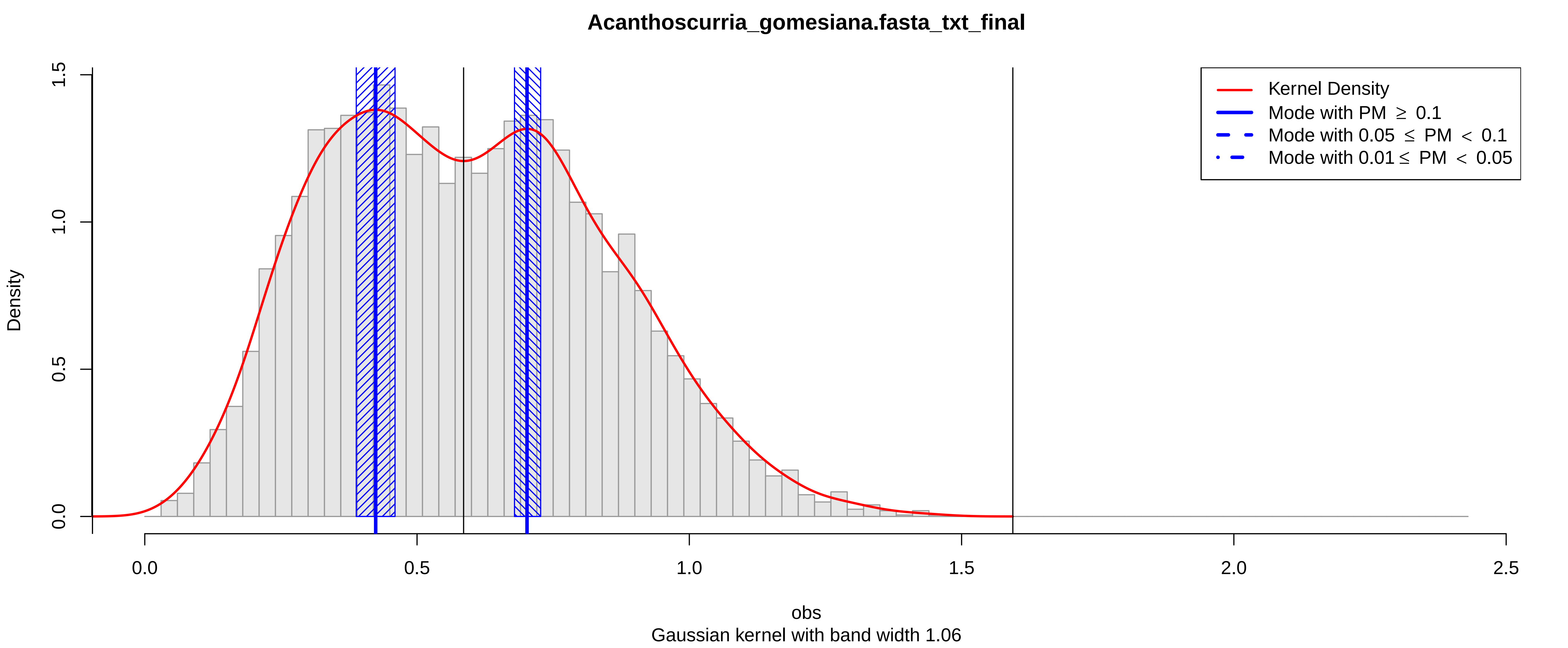

Acanthoscurria_gomesiana.fasta_txt_final
Kernel Density
Mode with PM ≥ 0.1
Mode with 0.05 ≤ PM < 0.1
Mode with 0.01 ≤ PM < 0.05
Density
0.0
0.5
1.0
1.5
0.0
0.5
1.0
1.5
2.0
2.5
obs
Gaussian kernel with band width 1.06

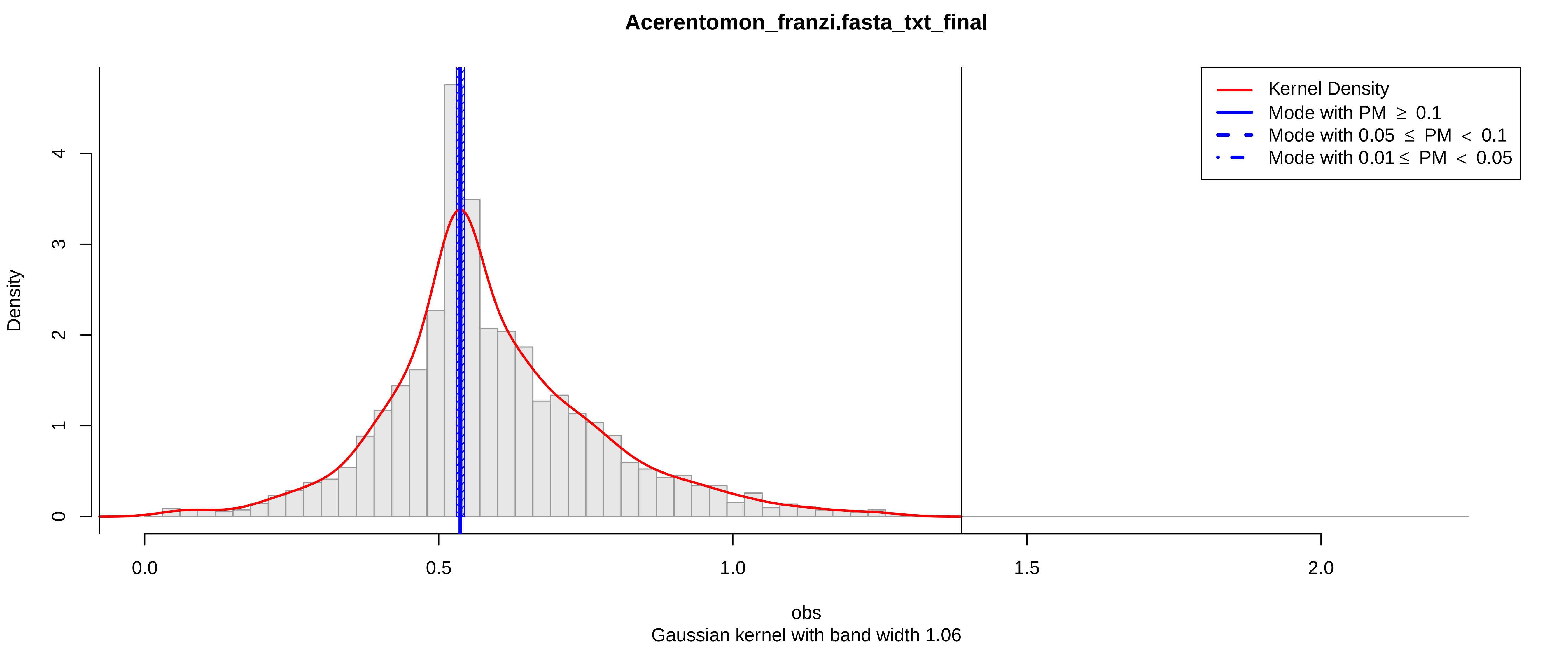
Acerentomon_franzi.fasta_txt_final
Kernel Density
Mode with PM ≥ 0.1
Mode with 0.05 ≤ PM < 0.1
Mode with 0.01 ≤ PM < 0.05
Density
0
1
2
3
4
0.0
0.5
1.0
1.5
2.0
obs
Gaussian kernel with band width 1.06

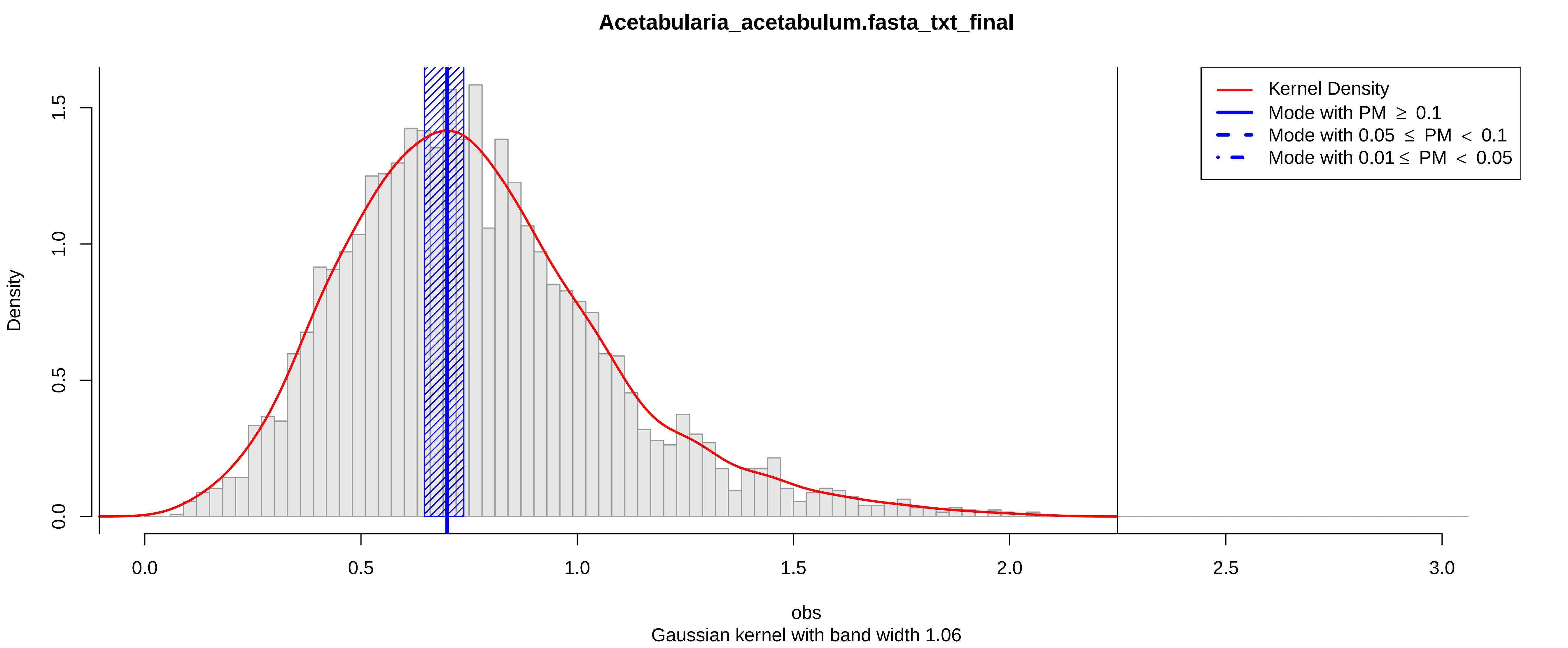

Acetabularia_acetabulum.fasta_txt_final
Kernel Density
Mode with PM ≥ 0.1
Mode with 0.05 ≤ PM < 0.1
Mode with 0.01 ≤ PM < 0.05
Density
0.0
0.5
1.0
1.5
0.0
0.5
1.0
1.5
2.0
2.5
3.0
obs
Gaussian kernel with band width 1.06

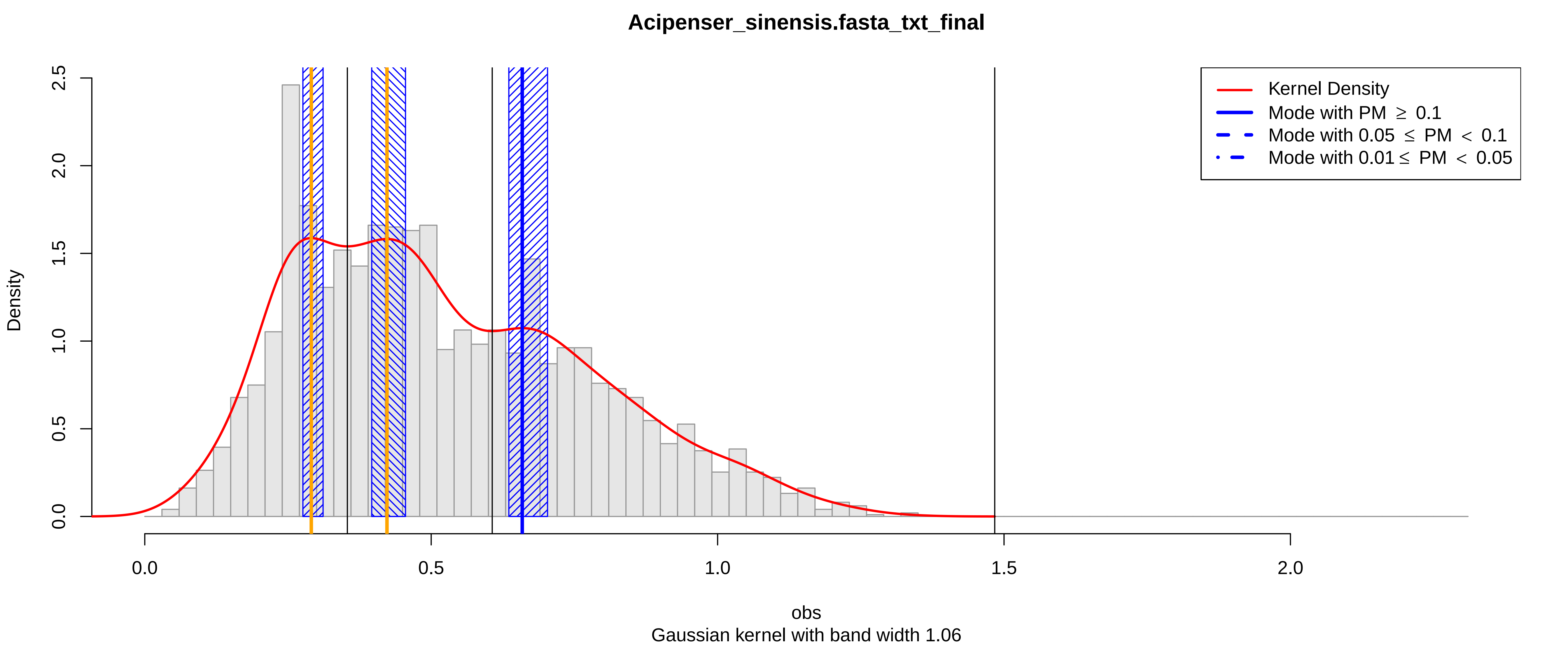

Acipenser_sinensis.fasta_txt_final
Density
0.0
0.5
1.0
1.5
2.0
2.5
0.0
0.5
1.0
1.5
2.0
Kernel Density
Mode with PM ≥ 0.1
Mode with 0.05 ≤ PM < 0.1
Mode with 0.01 ≤ PM < 0.05
obs
Gaussian kernel with band width 1.06

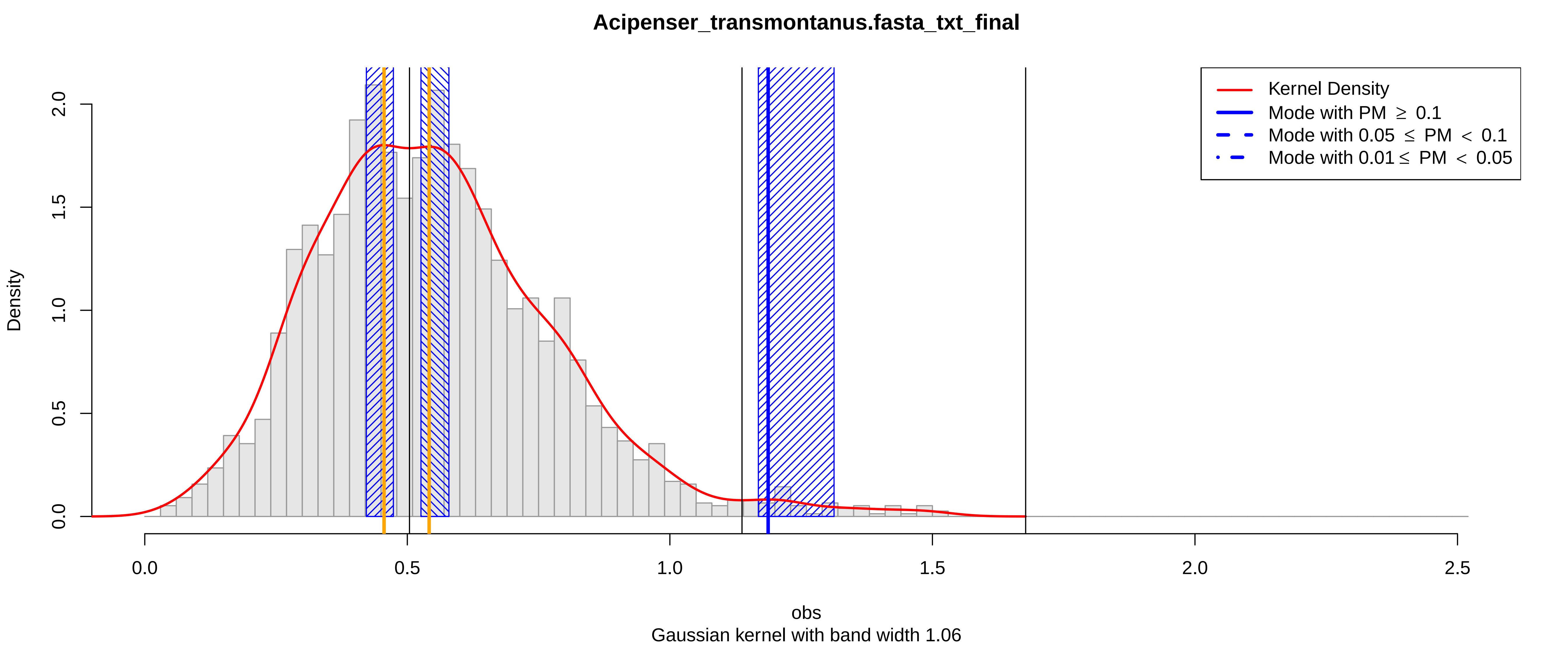
Acipenser_transmontanus.fasta_txt_final
Kernel Density
Mode with PM ≥ 0.1
Mode with 0.05 ≤ PM < 0.1
Mode with 0.01 ≤ PM < 0.05
Density
0.0
0.5
1.0
1.5
2.0
0.0
0.5
1.0
1.5
2.0
2.5
obs
Gaussian kernel with band width 1.06

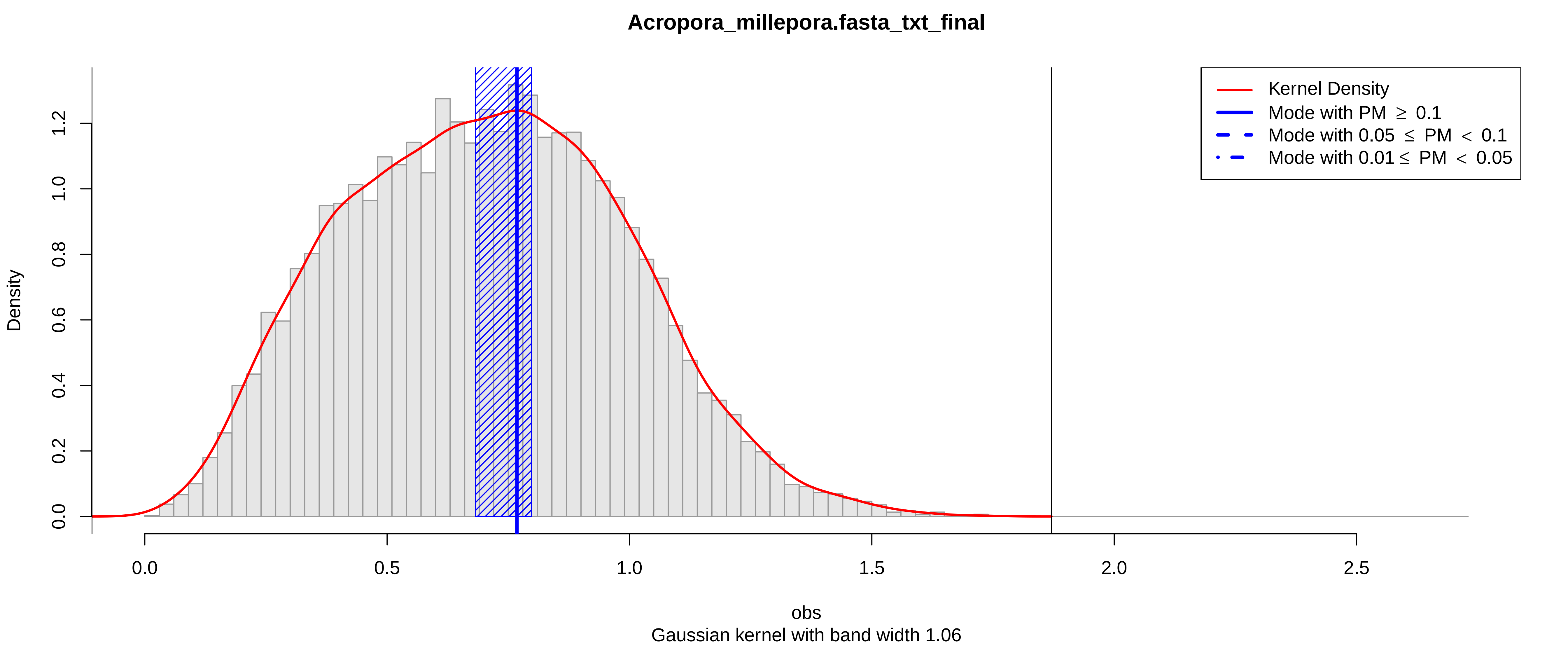

Acropora_millepora.fasta_txt_final
Kernel Density
Mode with PM ≥ 0.1
Mode with 0.05 ≤ PM < 0.1
Mode with 0.01 ≤ PM < 0.05
Density
0.0
0.2
0.4
0.6
0.8
1.0
1.2
0.0
0.5
1.0
1.5
2.0
2.5
obs
Gaussian kernel with band width 1.06

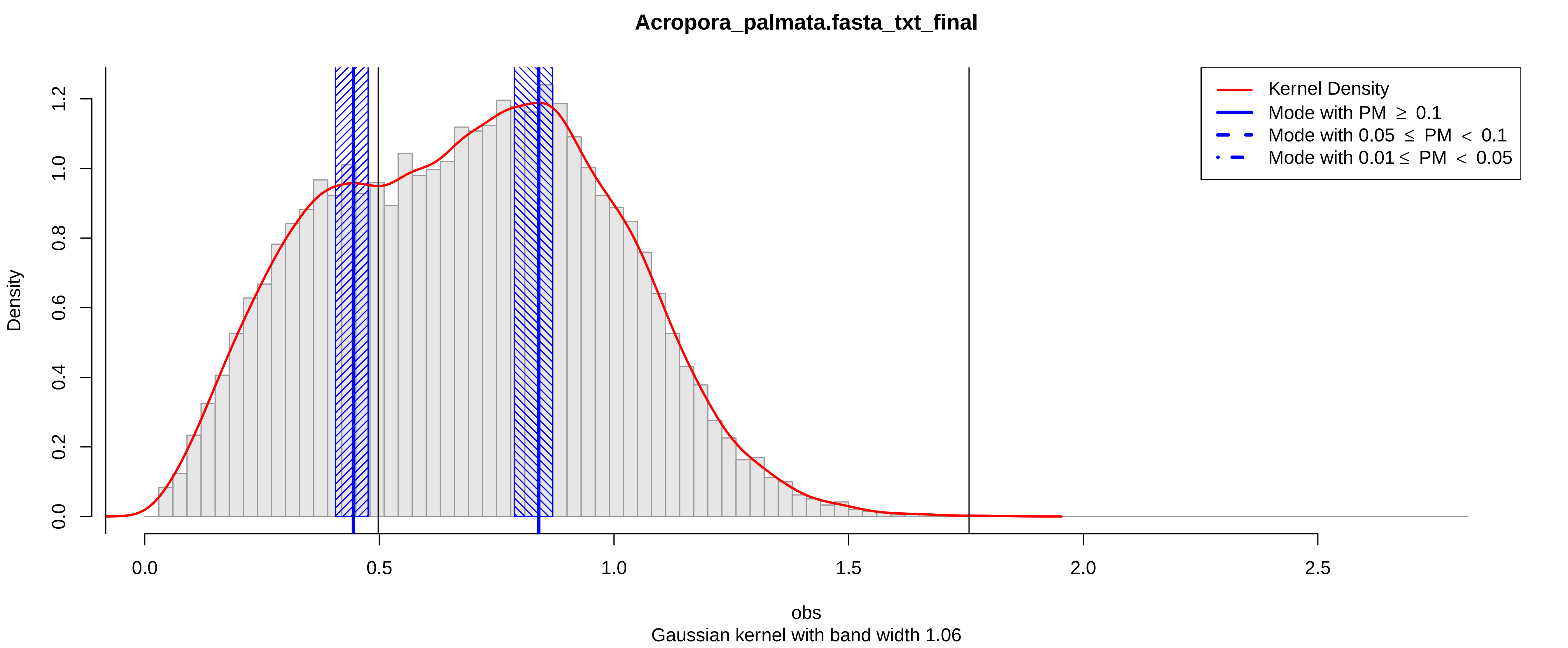
Acropora_palmata.fasta_txt_final
Density
0.0
0.2
0.4
0.6
0.8
1.0
1.2
0.0
0.5
1.0
1.5
2.0
2.5
obs
Gaussian kernel with band width 1.06
Kernel Density
Mode with PM ≥ 0.1
Mode with 0.05 ≤ PM < 0.1
Mode with 0.01 ≤ PM < 0.05

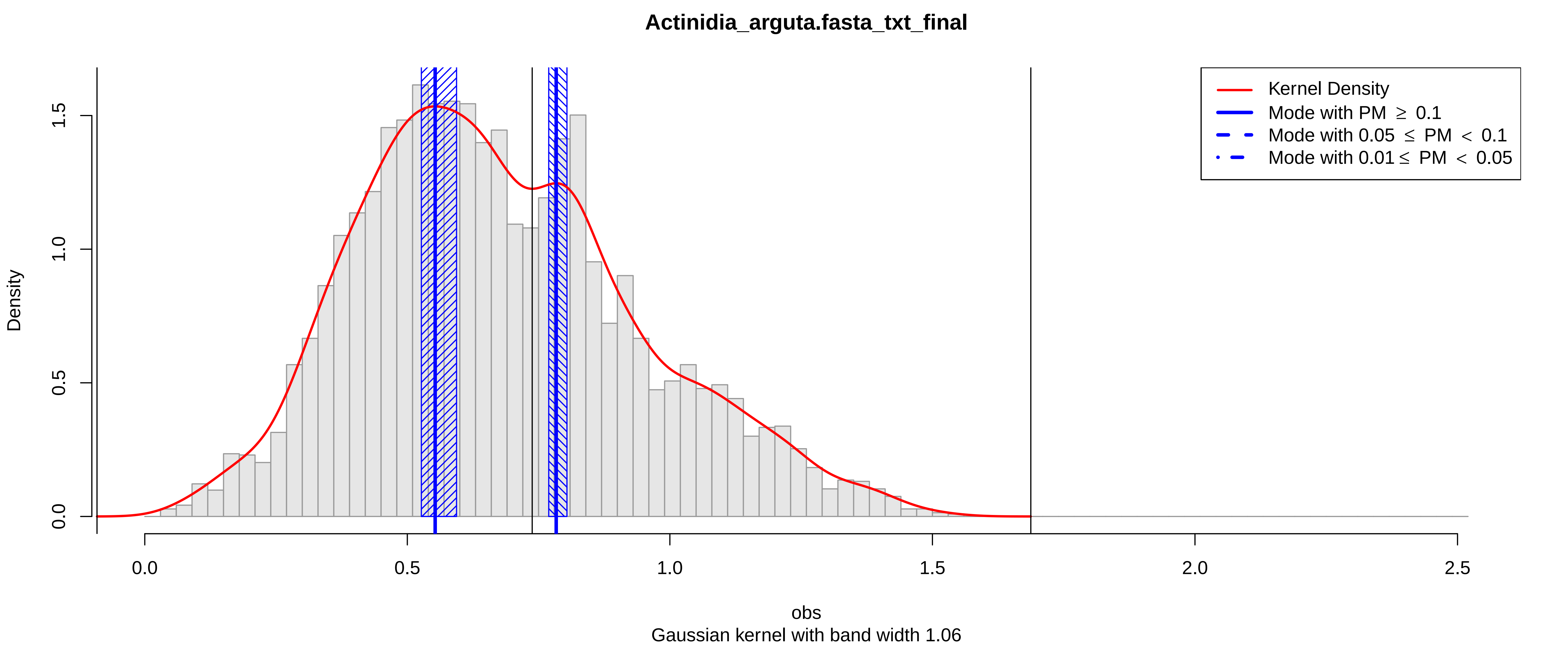

Actinidia_arguta.fasta_txt_final
Kernel Density
Mode with PM ≥ 0.1
Mode with 0.05 ≤ PM < 0.1
Mode with 0.01 ≤ PM < 0.05
Density
0.0
0.5
1.0
1.5
0.0
0.5
1.0
1.5
2.0
2.5
obs
Gaussian kernel with band width 1.06

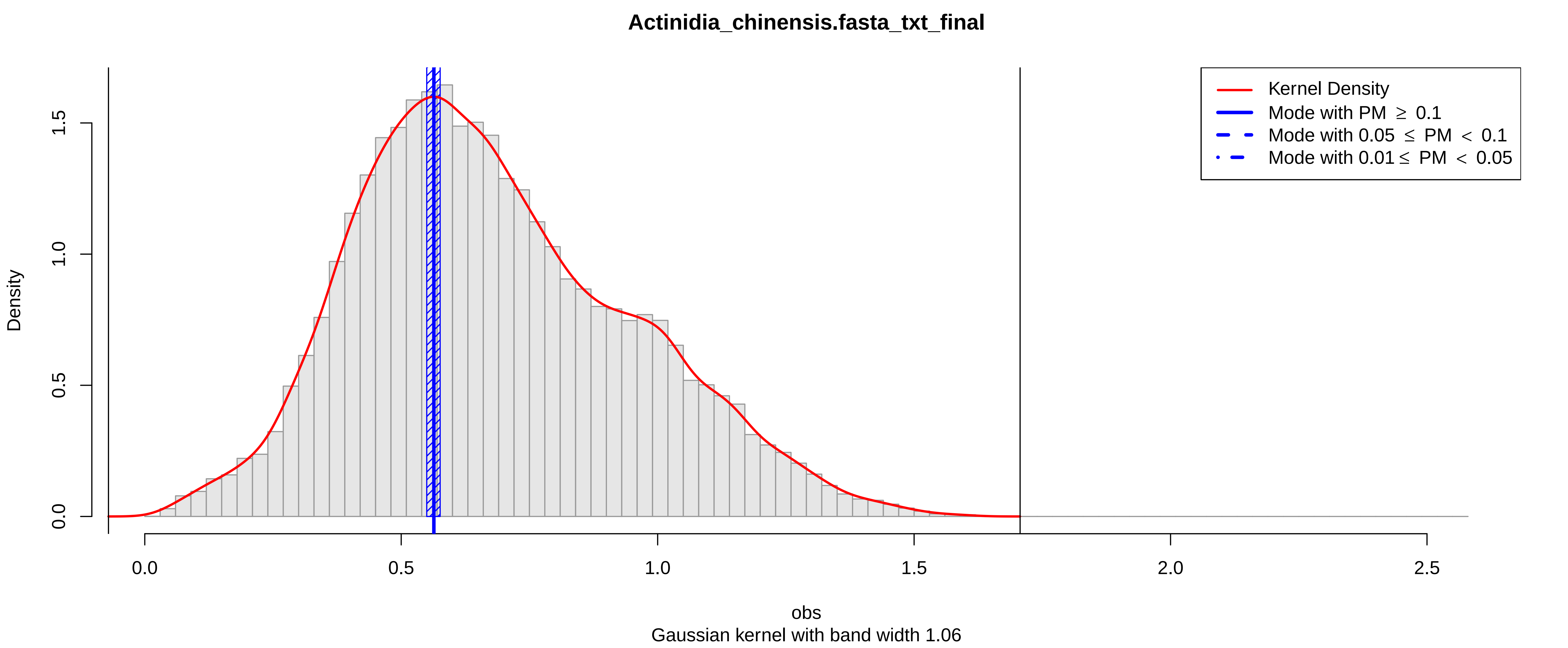
Actinidia_chinensis.fasta_txt_final
Kernel Density
Mode with PM ≥ 0.1
Mode with 0.05 ≤ PM < 0.1
Mode with 0.01 ≤ PM < 0.05
Density
0.0
0.5
1.0
1.5
0.0
0.5
1.0
1.5
2.0
2.5
obs
Gaussian kernel with band width 1.06

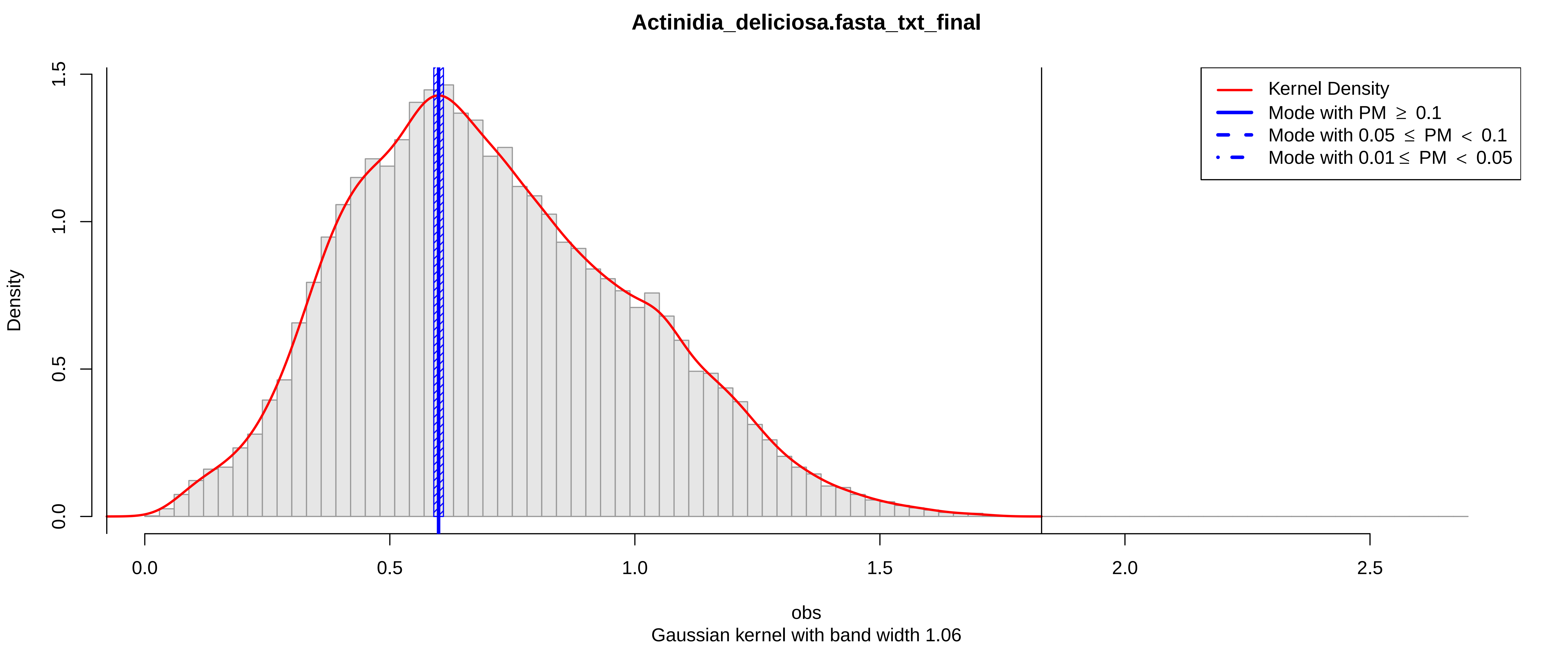
Actinidia_deliciosa.fasta_txt_final
Kernel Density
Mode with PM ≥ 0.1
Mode with 0.05 ≤ PM < 0.1
Mode with 0.01 ≤ PM < 0.05
Density
0.0
0.5
1.0
1.5
2.0
2.5
obs
Gaussian kernel with band width 1.06

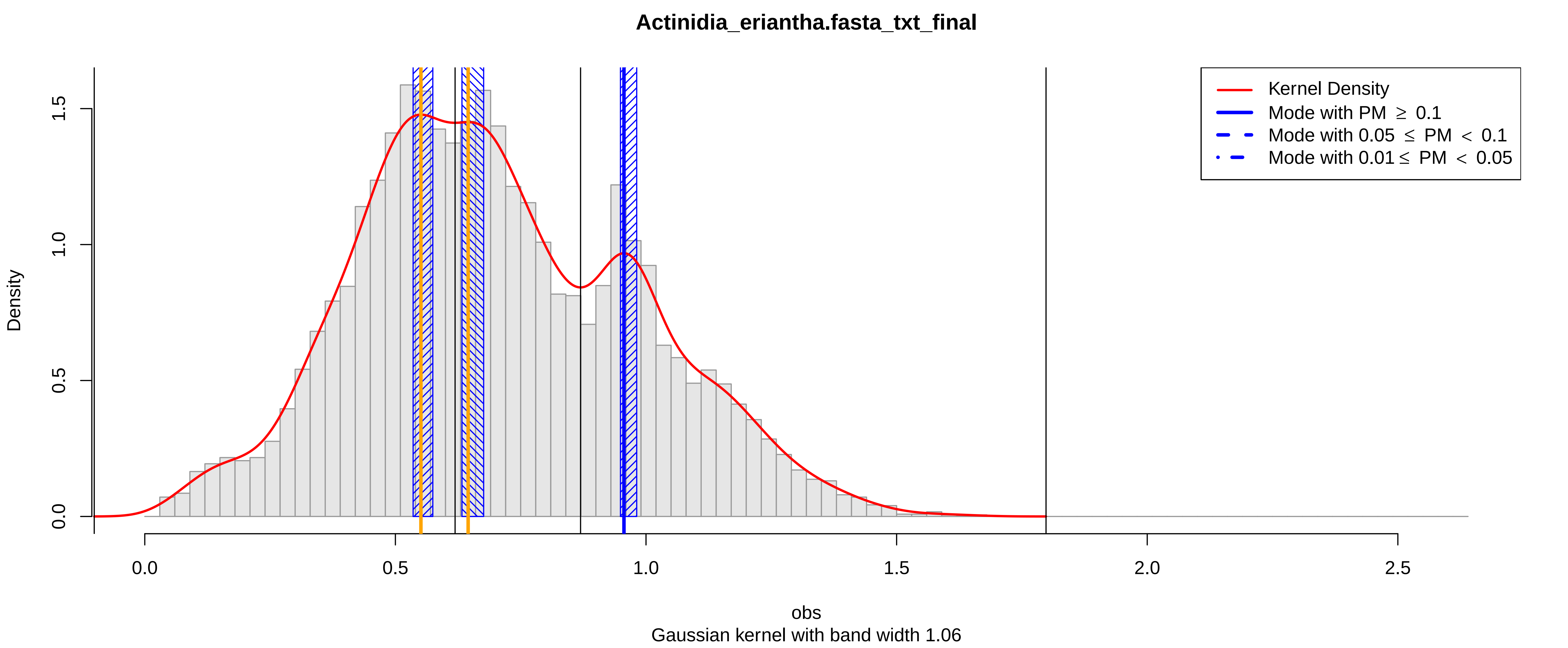

Actinidia_eriantha.fasta_txt_final
Kernel Density
Mode with PM ≥ 0.1
Mode with 0.05 ≤ PM < 0.1
Mode with 0.01 ≤ PM < 0.05
Density
0.0
0.5
1.0
1.5
0.0
0.5
1.0
1.5
2.0
2.5
obs
Gaussian kernel with band width 1.06

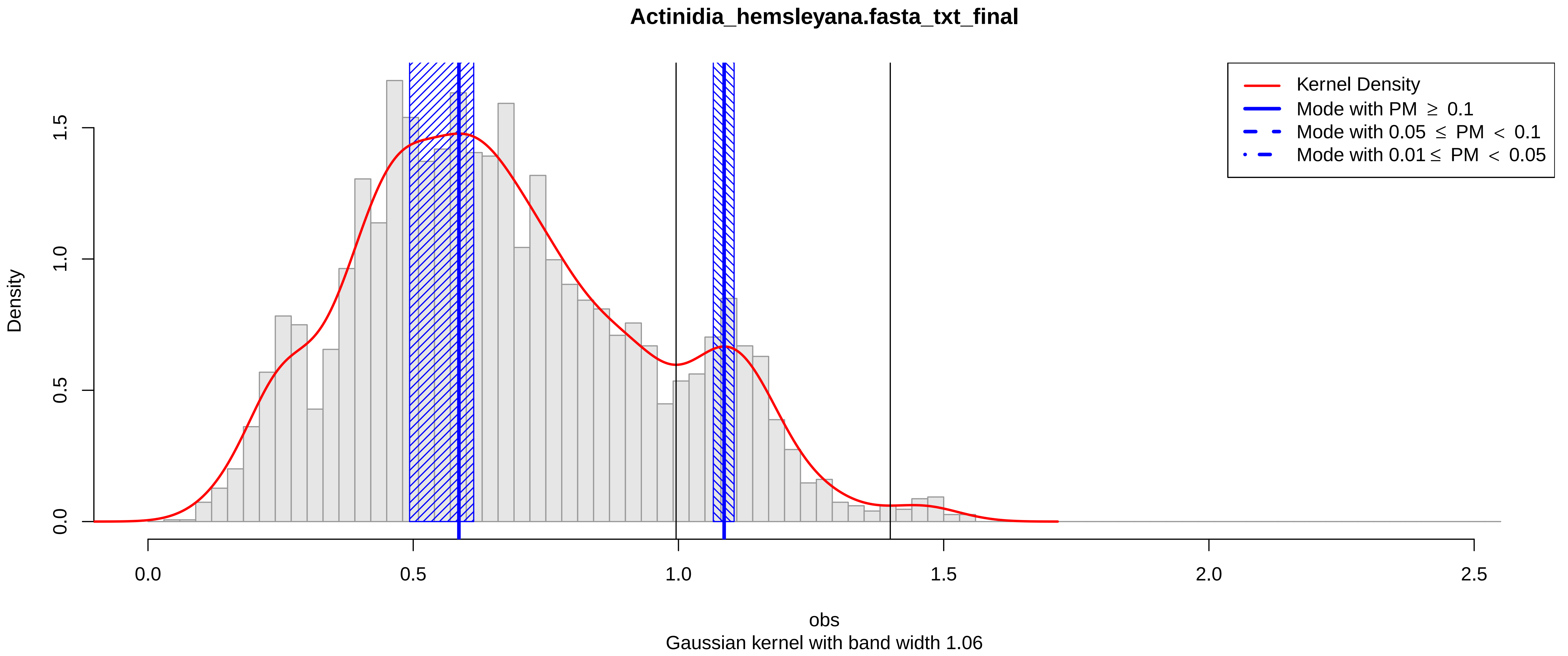

Actinidia_hemsleyana.fasta_txt_final
Density
0.0
0.5
1.0
1.5
0.0
0.5
1.0
1.5
2.0
2.5
obs
Gaussian kernel with band width 1.06
Kernel Density
Mode with PM ≥ 0.1
Mode with 0.05 ≤ PM < 0.1
Mode with 0.01 ≤ PM < 0.05

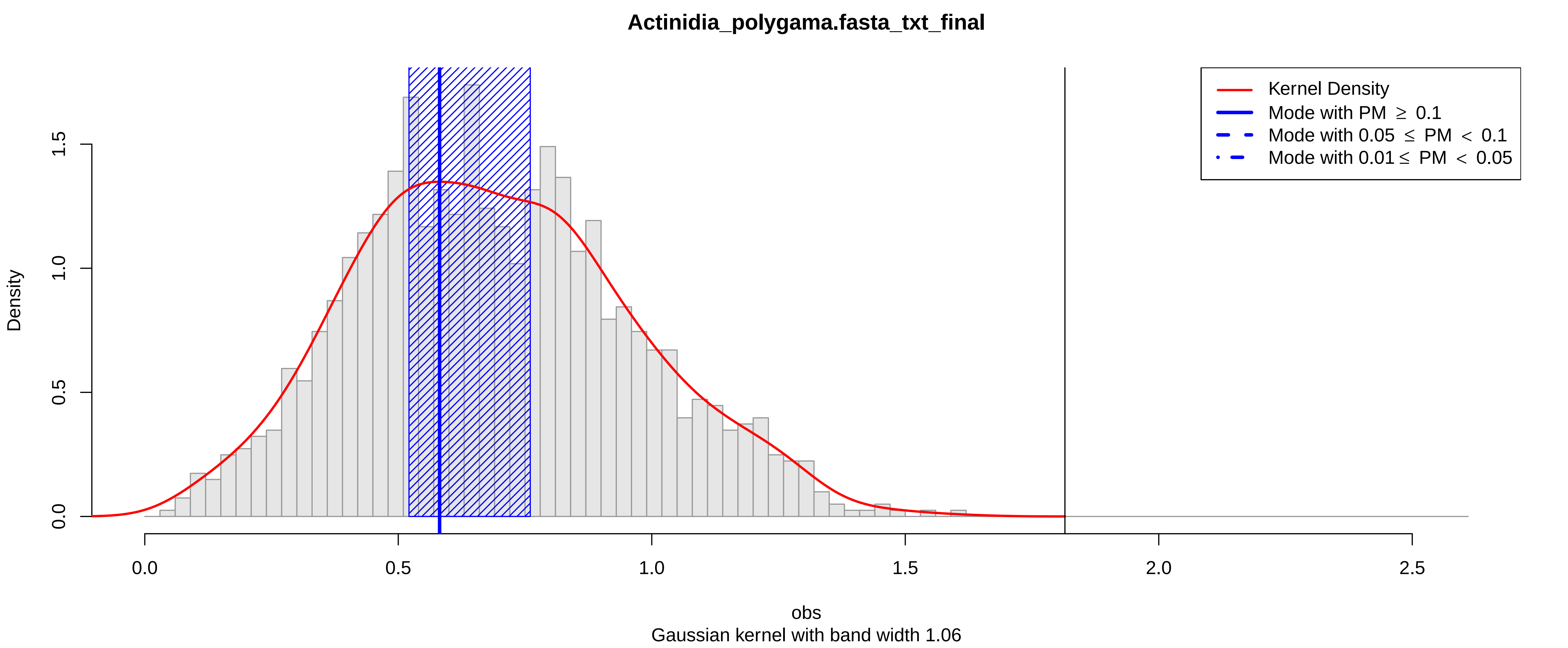

Actinidia_polygama.fasta_txt_final
Kernel Density
Mode with PM ≥ 0.1
Mode with 0.05 ≤ PM < 0.1
Mode with 0.01 ≤ PM < 0.05
Density
0.0
0.5
1.0
1.5
0.0
0.5
1.0
1.5
2.0
2.5
obs
Gaussian kernel with band width 1.06

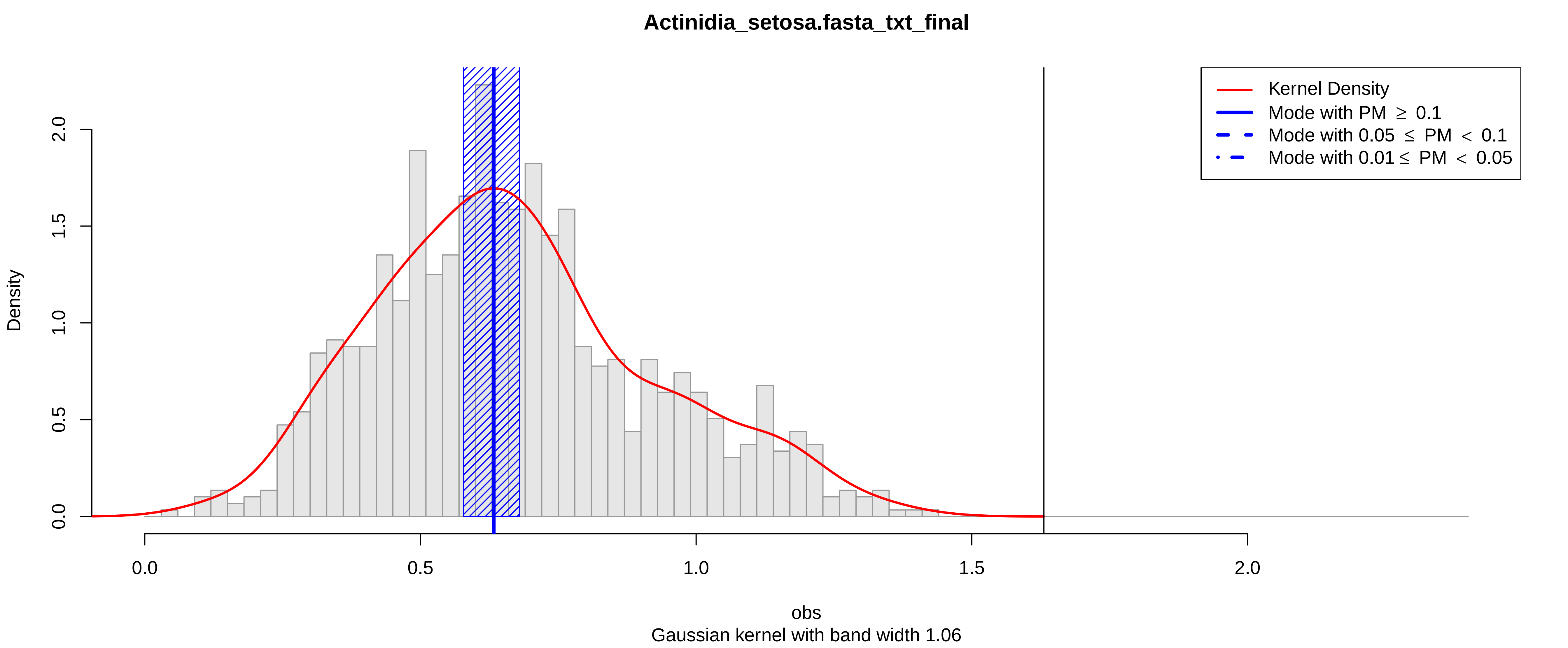

Actinidia_setosa.fasta_txt_final
Kernel Density
Mode with PM ≥ 0.1
Mode with 0.05 ≤ PM < 0.1
Mode with 0.01 ≤ PM < 0.05
Density
0.0
0.5
1.0
1.5
2.0
0.0
0.5
1.0
1.5
2.0
obs
Gaussian kernel with band width 1.06

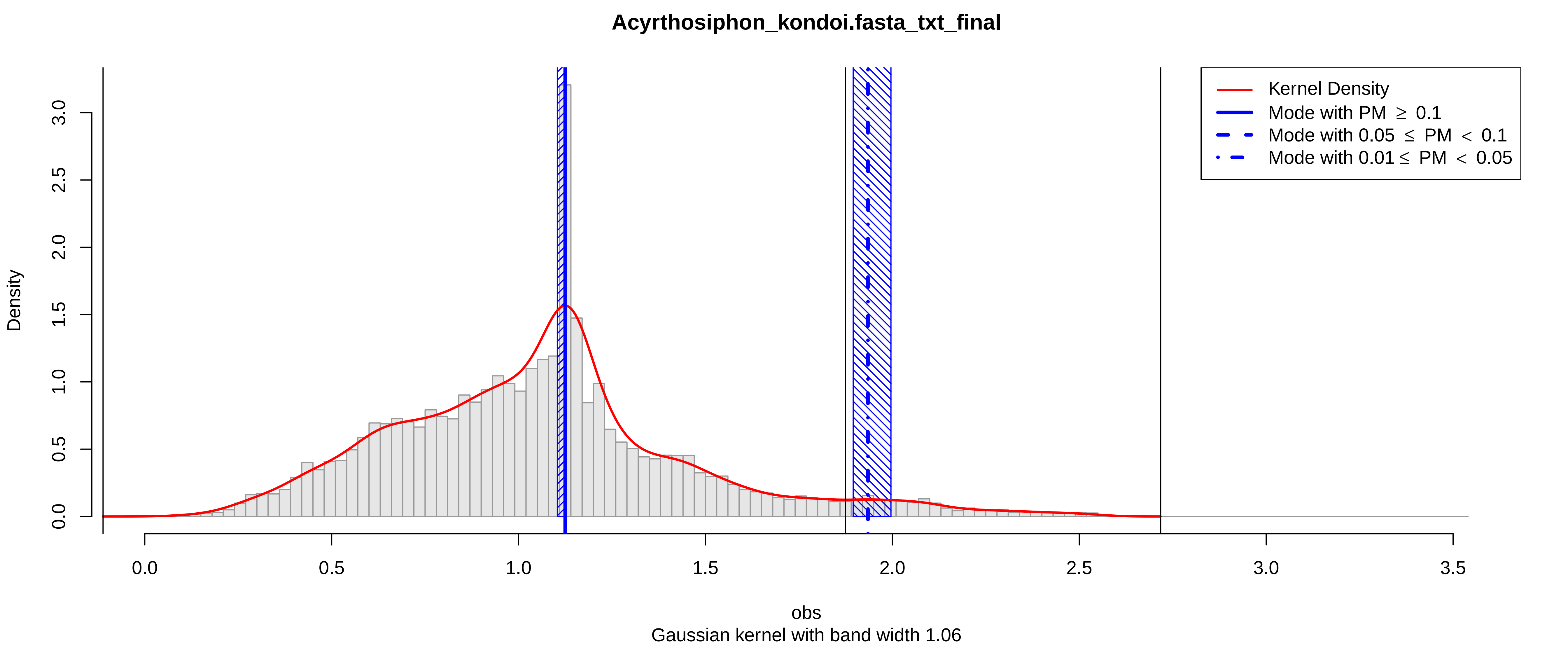
Acyrthosiphon_kondoi.fasta_txt_final
Kernel Density
Mode with PM ≥ 0.1
Mode with 0.05 ≤ PM < 0.1
Mode with 0.01 ≤ PM < 0.05
Density
0.0
0.5
1.0
1.5
2.0
2.5
3.0
3.5
obs
Gaussian kernel with band width 1.06

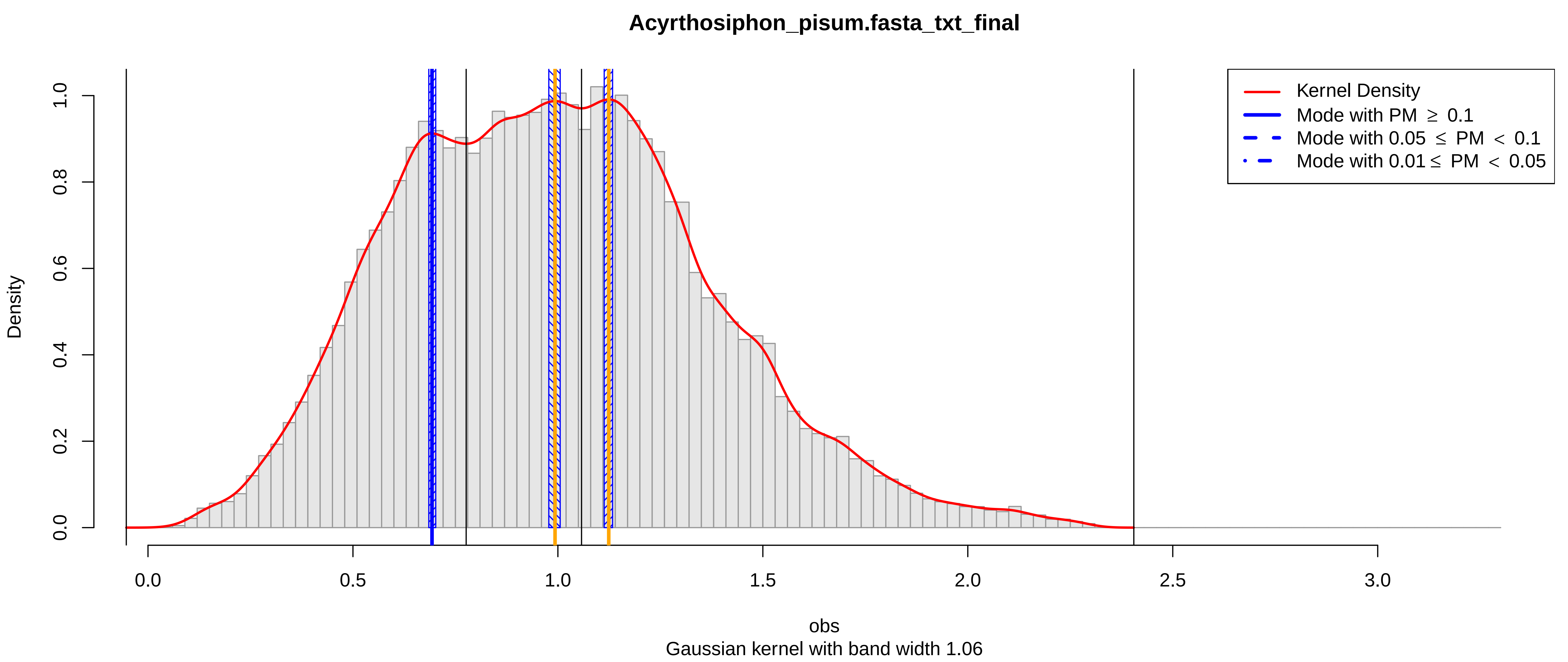

Acyrthosiphon_pisum.fasta_txt_final
Kernel Density
Mode with PM ≥ 0.1
Mode with 0.05 ≤ PM < 0.1
Mode with 0.01 ≤ PM < 0.05
Density
0.0
0.2
0.4
0.6
0.8
1.0
0.0
0.5
1.0
1.5
2.0
2.5
3.0
obs
Gaussian kernel with band width 1.06

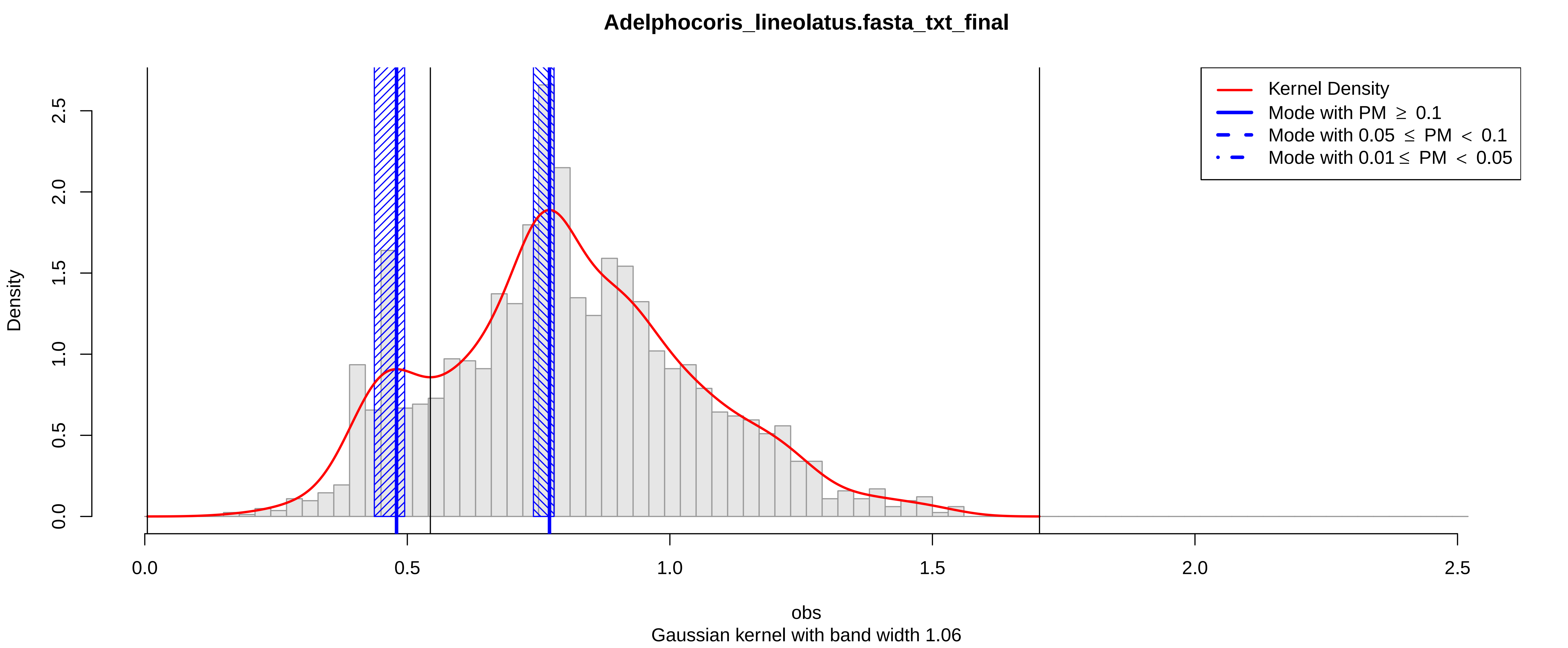
Adelphocoris_lineolatus.fasta_txt_final
Kernel Density
Mode with PM ≥ 0.1
Mode with 0.05 ≤ PM < 0.1
Mode with 0.01 ≤ PM < 0.05
Density
0.0
0.5
1.0
1.5
2.0
2.5
obs
Gaussian kernel with band width 1.06

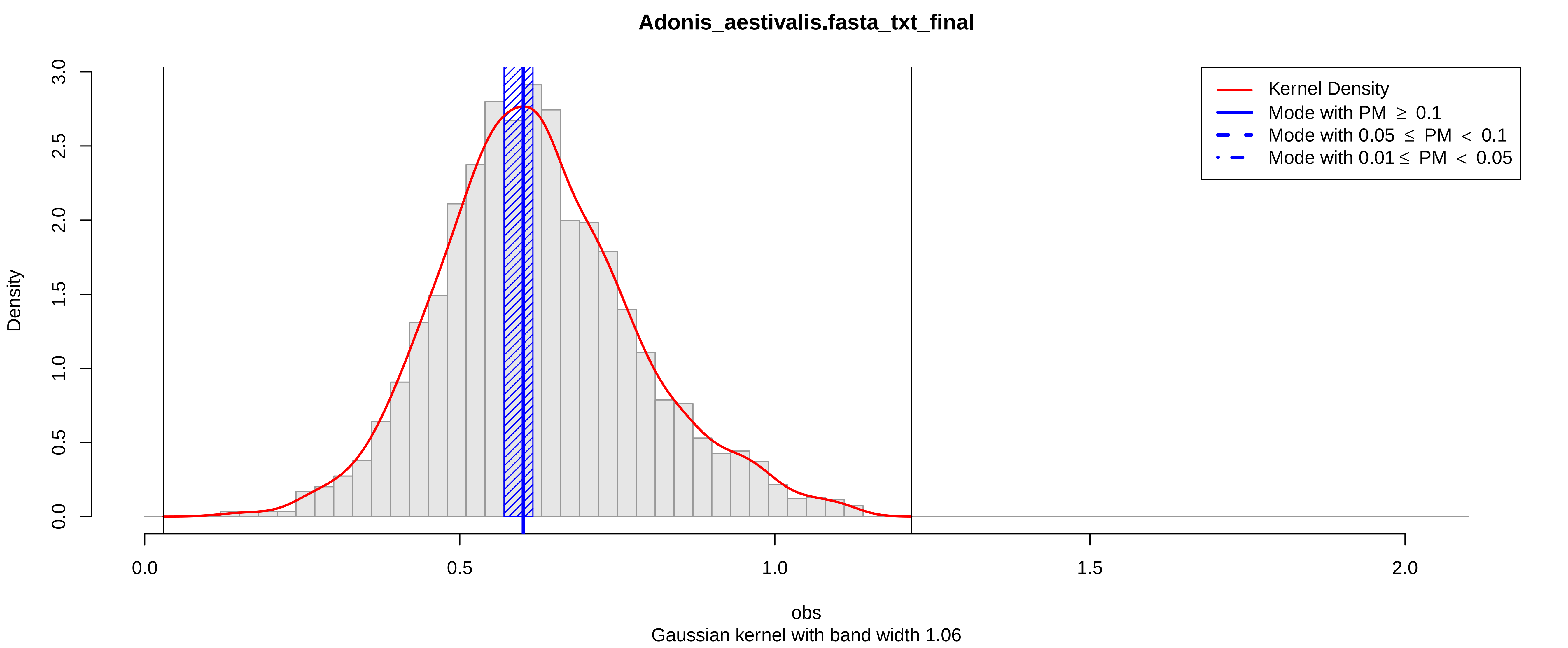

Adonis_aestivalis.fasta_txt_final
Density
0.0
0.5
1.0
1.5
2.0
2.5
3.0
Kernel Density
Mode with PM ≥ 0.1
Mode with 0.05 ≤ PM < 0.1
Mode with 0.01 ≤ PM < 0.05
0.0
0.5
1.0
1.5
2.0
obs
Gaussian kernel with band width 1.06

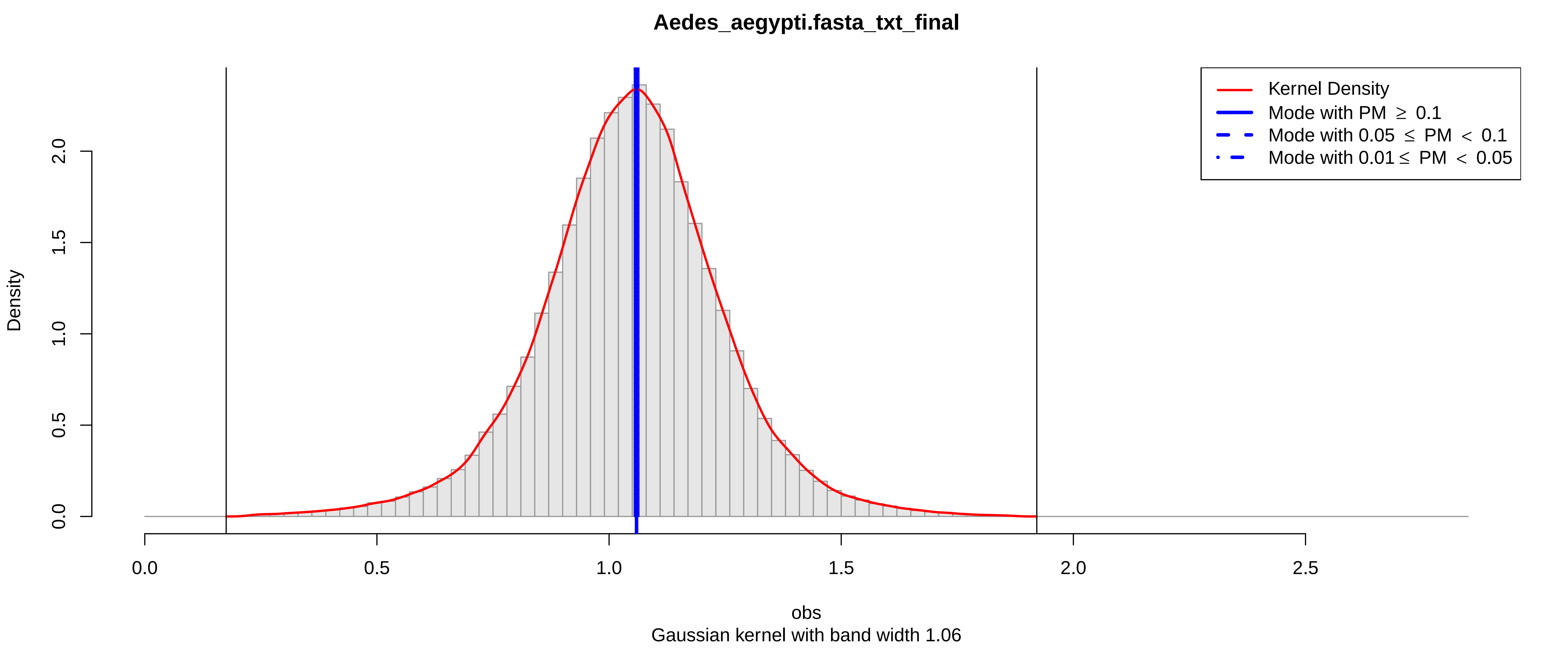

Aedes_aegypti.fasta_txt_final
Kernel Density
Mode with PM ≥ 0.1
Mode with 0.05 ≤ PM < 0.1
Mode with 0.01 ≤ PM < 0.05
Density
0.0
0.5
1.0
1.5
2.0
0.0
0.5
1.0
1.5
2.0
2.5
obs
Gaussian kernel with band width 1.06

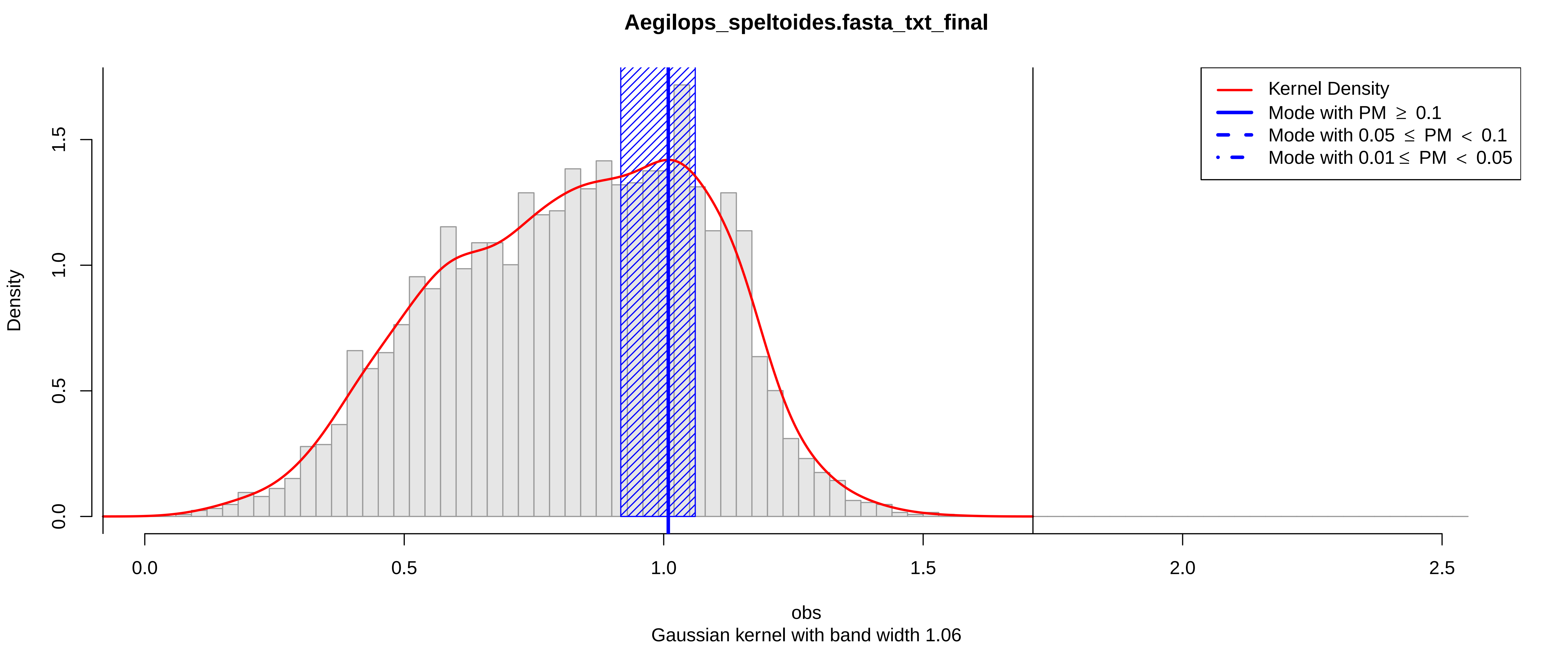

Aegilops_speltoides.fasta_txt_final
Kernel Density
Mode with PM ≥ 0.1
Mode with 0.05 ≤ PM < 0.1
Mode with 0.01 ≤ PM < 0.05
Density
0.0
0.5
1.0
1.5
0.0
0.5
1.0
1.5
2.0
2.5
obs
Gaussian kernel with band width 1.06

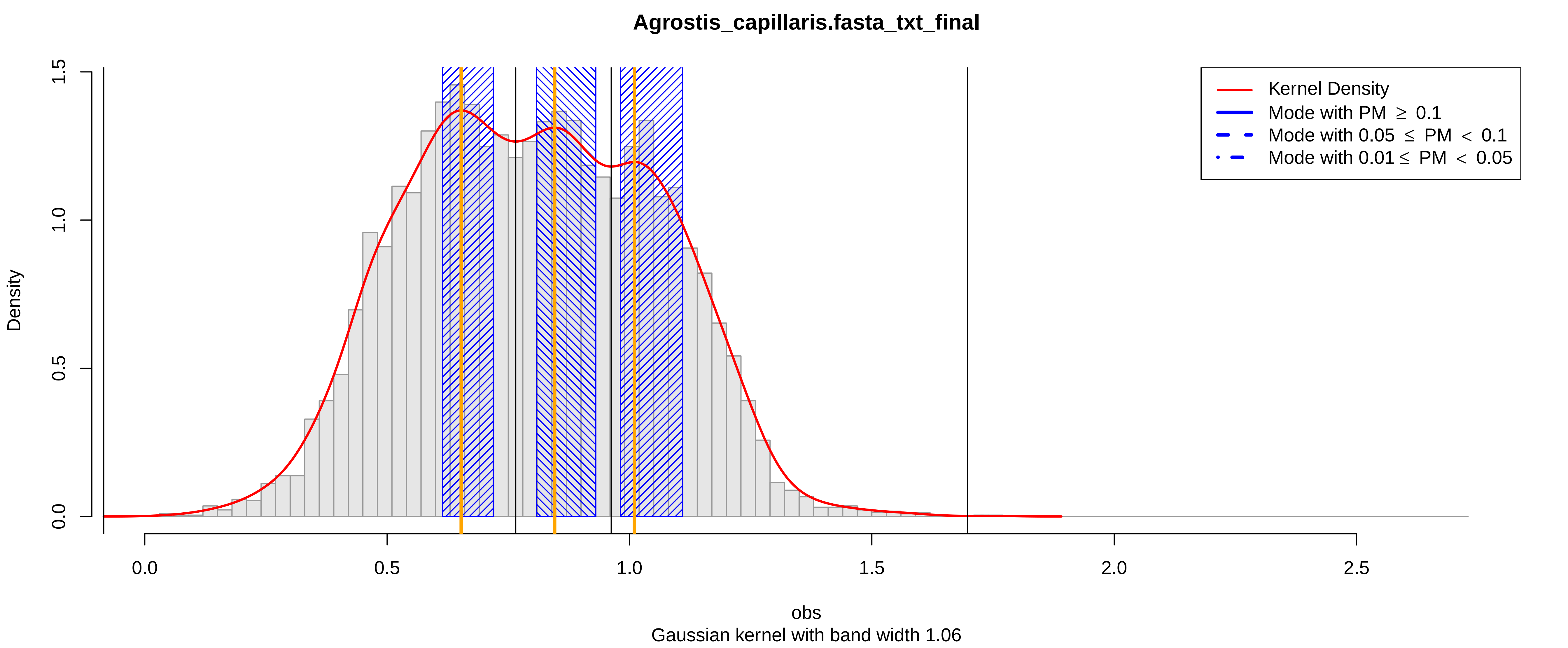

Agrostis_capillaris.fasta_txt_final
Kernel Density
Mode with PM ≥ 0.1
Mode with 0.05 ≤ PM < 0.1
Mode with 0.01 ≤ PM < 0.05
Density
0.0
0.5
1.0
1.5
0.0
0.5
1.0
1.5
2.0
2.5
obs
Gaussian kernel with band width 1.06

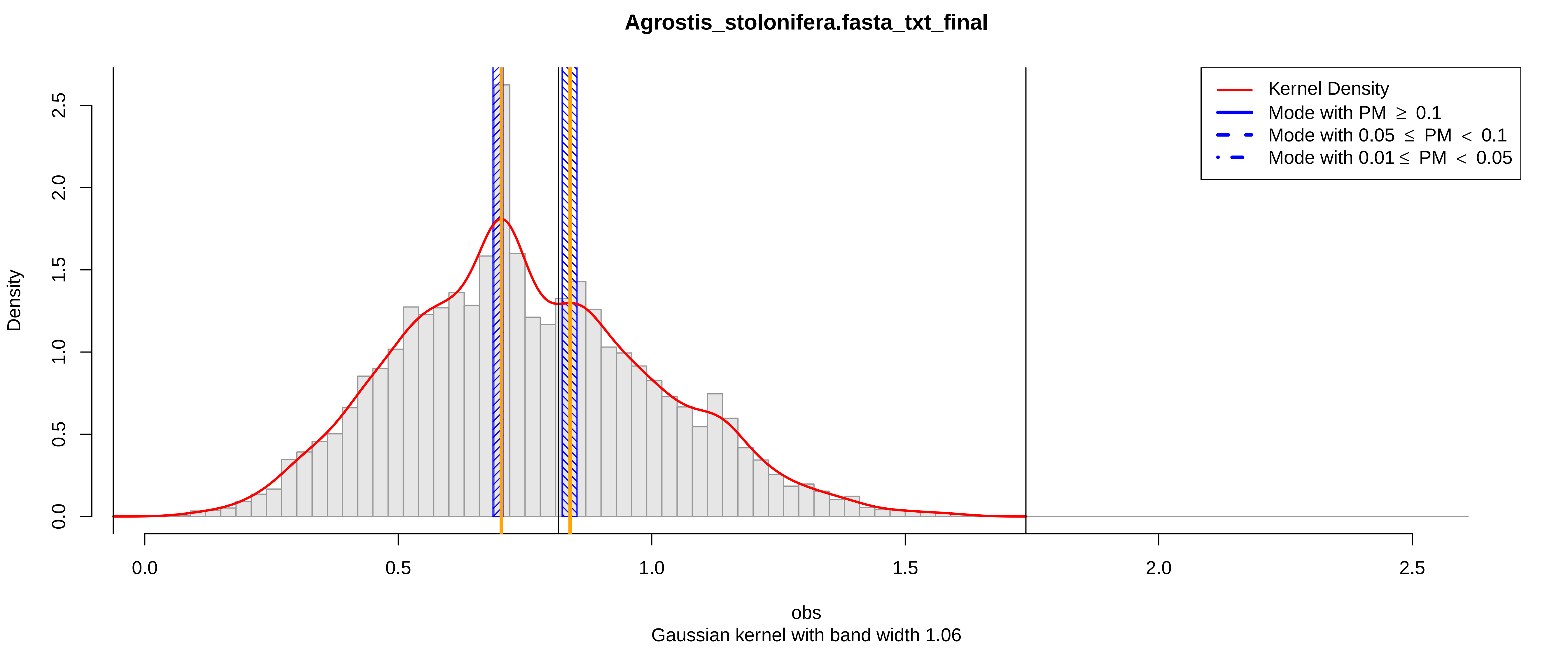
Agrostis_stolonifera.fasta_txt_final
Kernel Density
Mode with PM ≥ 0.1
Mode with 0.05 ≤ PM < 0.1
Mode with 0.01 ≤ PM < 0.05
Density
0.0
0.5
1.0
1.5
2.0
2.5
obs
Gaussian kernel with band width 1.06

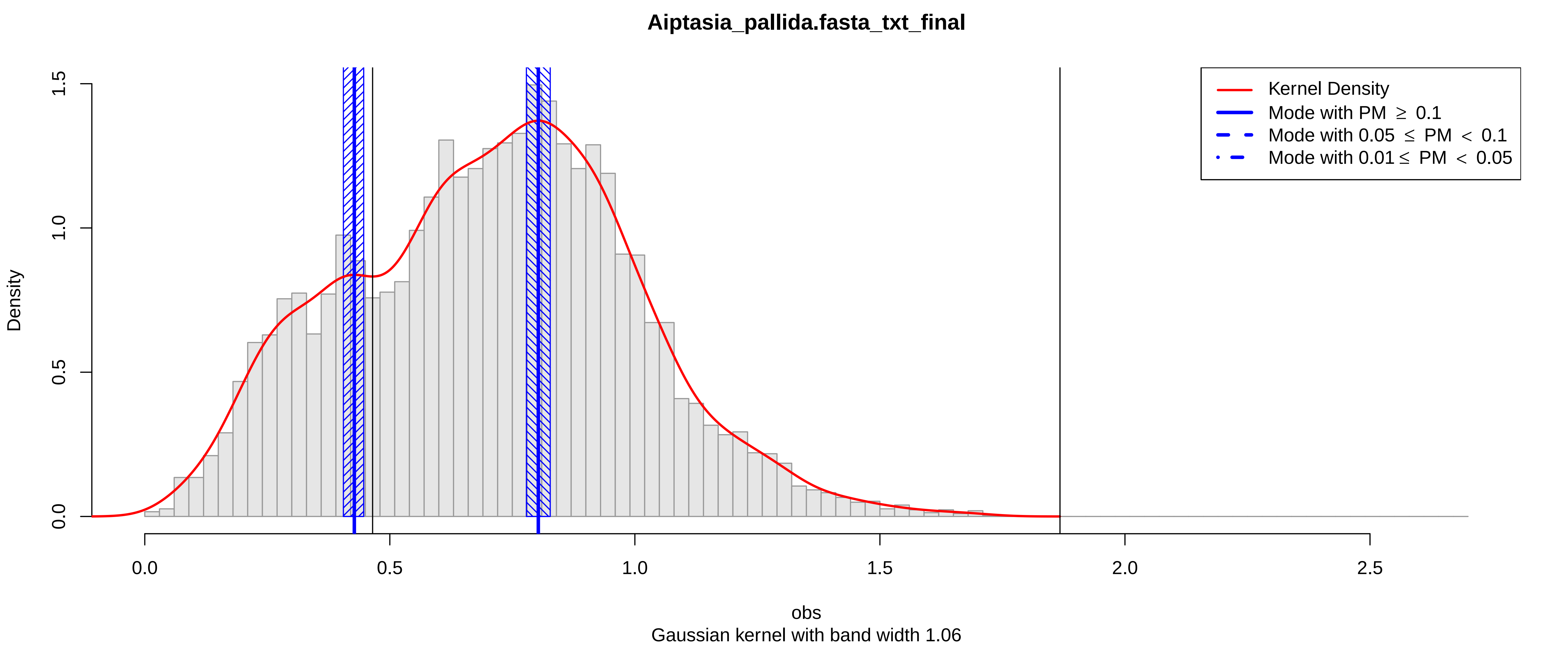
Aiptasia_pallida.fasta_txt_final
Kernel Density
Mode with PM ≥ 0.1
Mode with 0.05 ≤ PM < 0.1
Mode with 0.01 ≤ PM < 0.05
Density
0.0
0.5
1.0
1.5
0.0
0.5
1.0
1.5
2.0
2.5
obs
Gaussian kernel with band width 1.06

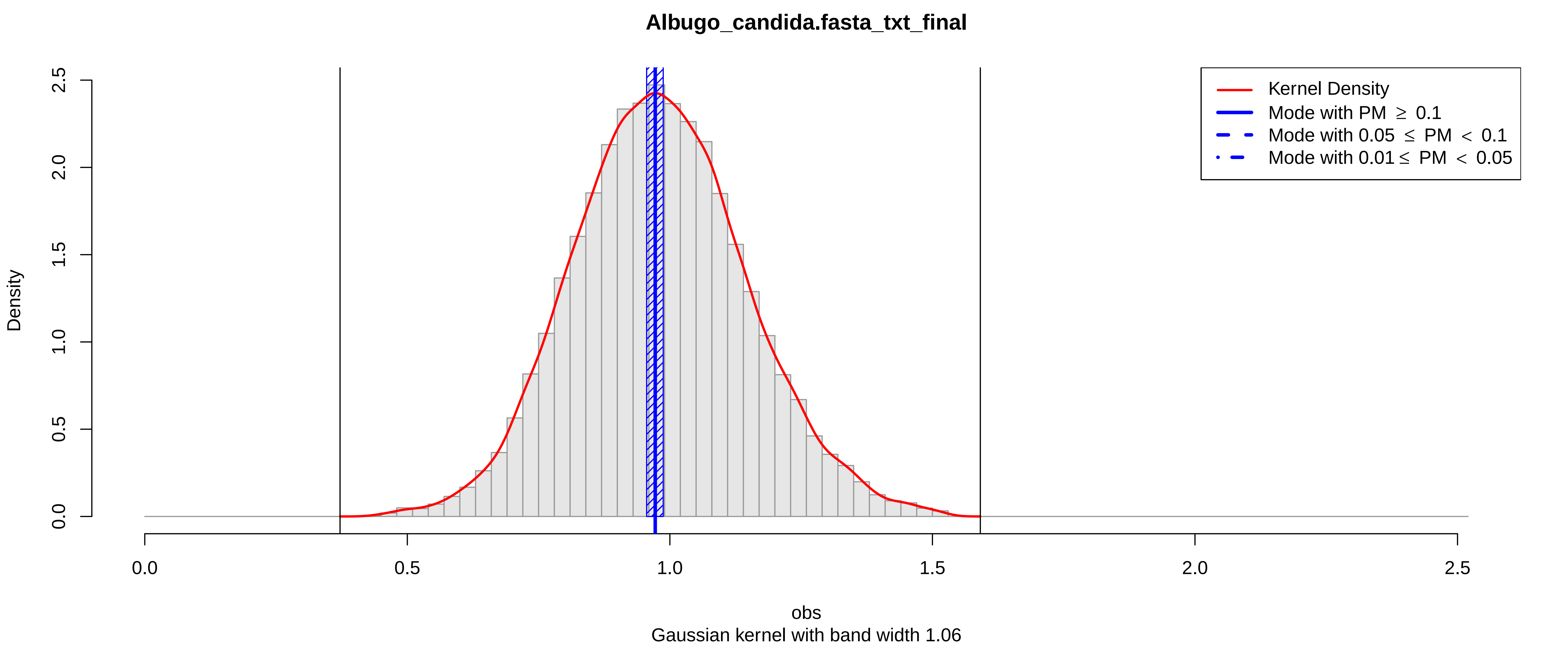

Albugo_candida.fasta_txt_final
Kernel Density
Mode with PM ≥ 0.1
Mode with 0.05 ≤ PM < 0.1
Mode with 0.01 ≤ PM < 0.05
Density
0.0
0.5
1.0
1.5
2.0
2.5
obs
Gaussian kernel with band width 1.06

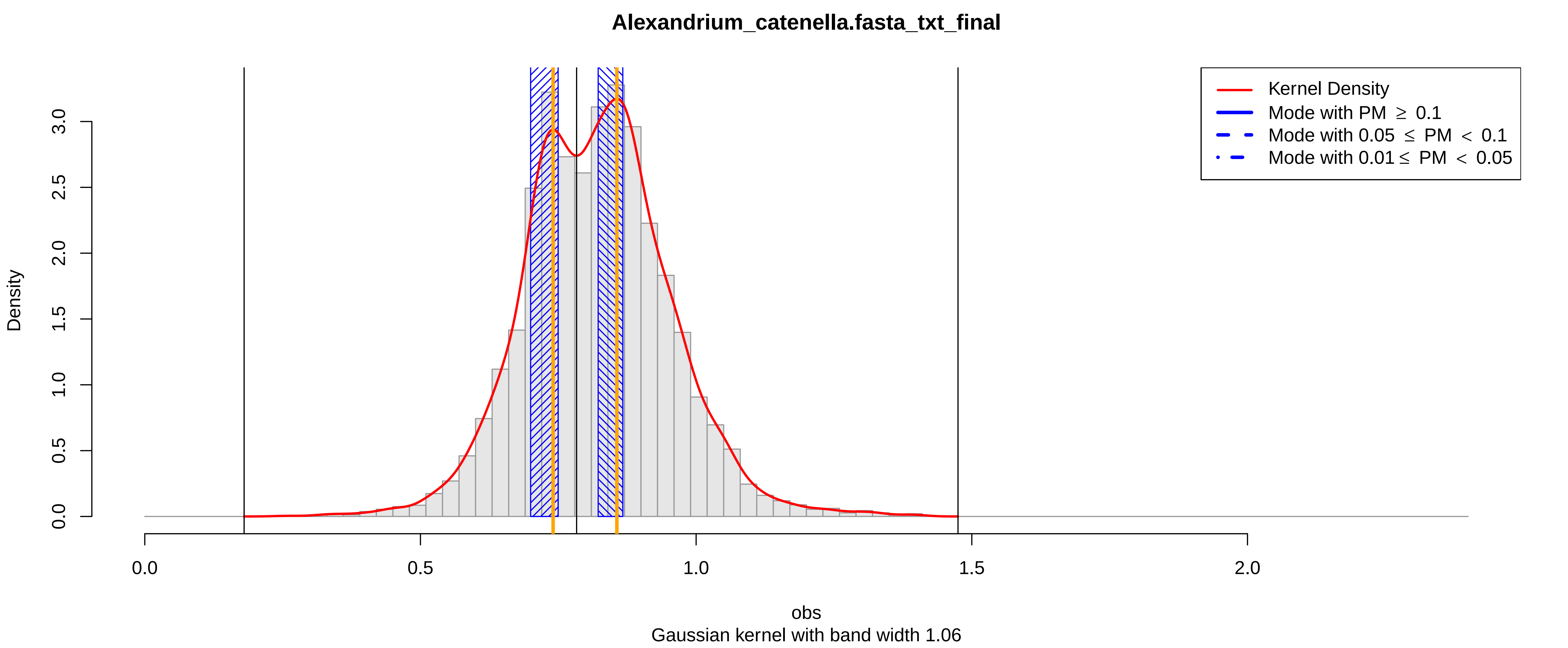
Alexandrium_catenella.fasta_txt_final
Kernel Density
Mode with PM ≥ 0.1
Mode with 0.05 ≤ PM < 0.1
Mode with 0.01 ≤ PM < 0.05
Density
0.0
0.5
1.0
1.5
2.0
2.5
3.0
0.0
0.5
1.0
1.5
2.0
obs
Gaussian kernel with band width 1.06

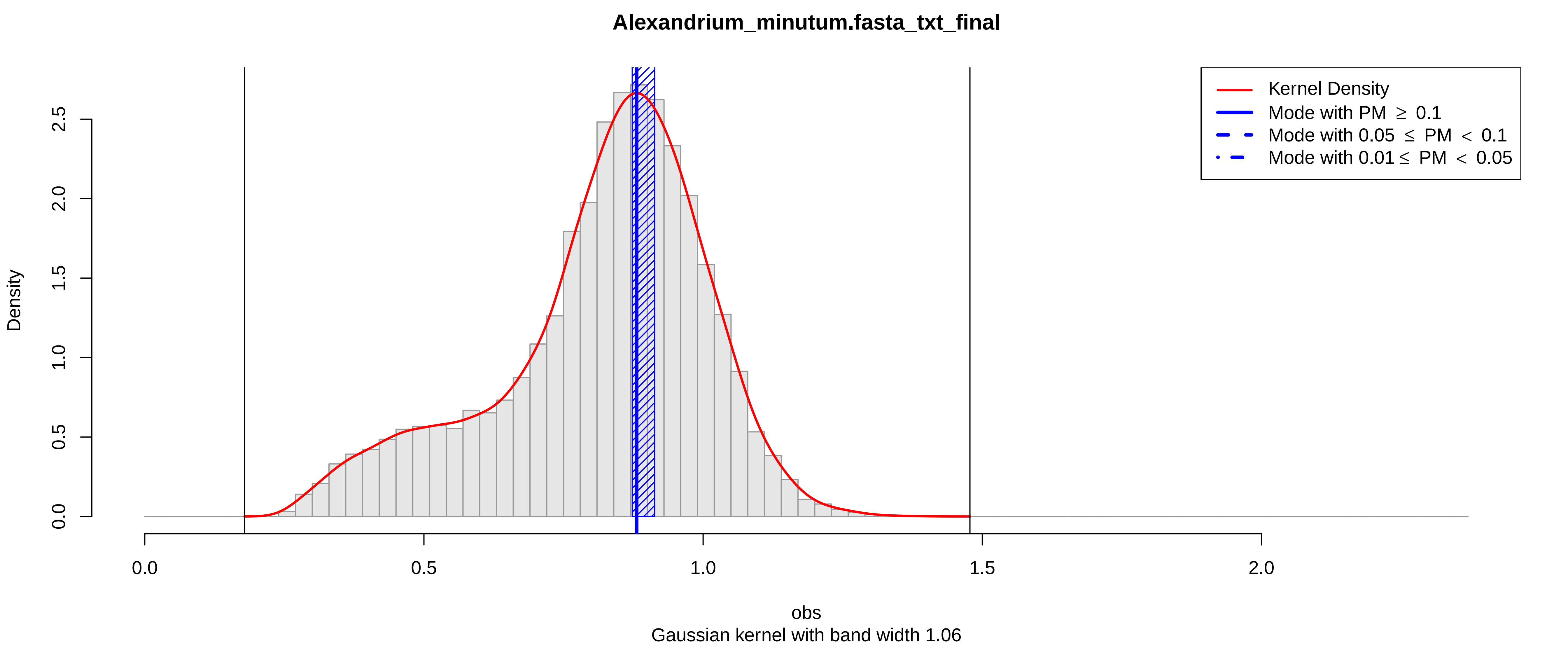
Alexandrium_minutum.fasta_txt_final
Kernel Density
Mode with PM ≥ 0.1
Mode with 0.05 ≤ PM < 0.1
Mode with 0.01 ≤ PM < 0.05
Density
0.0
0.5
1.0
1.5
2.0
2.5
obs
Gaussian kernel with band width 1.06

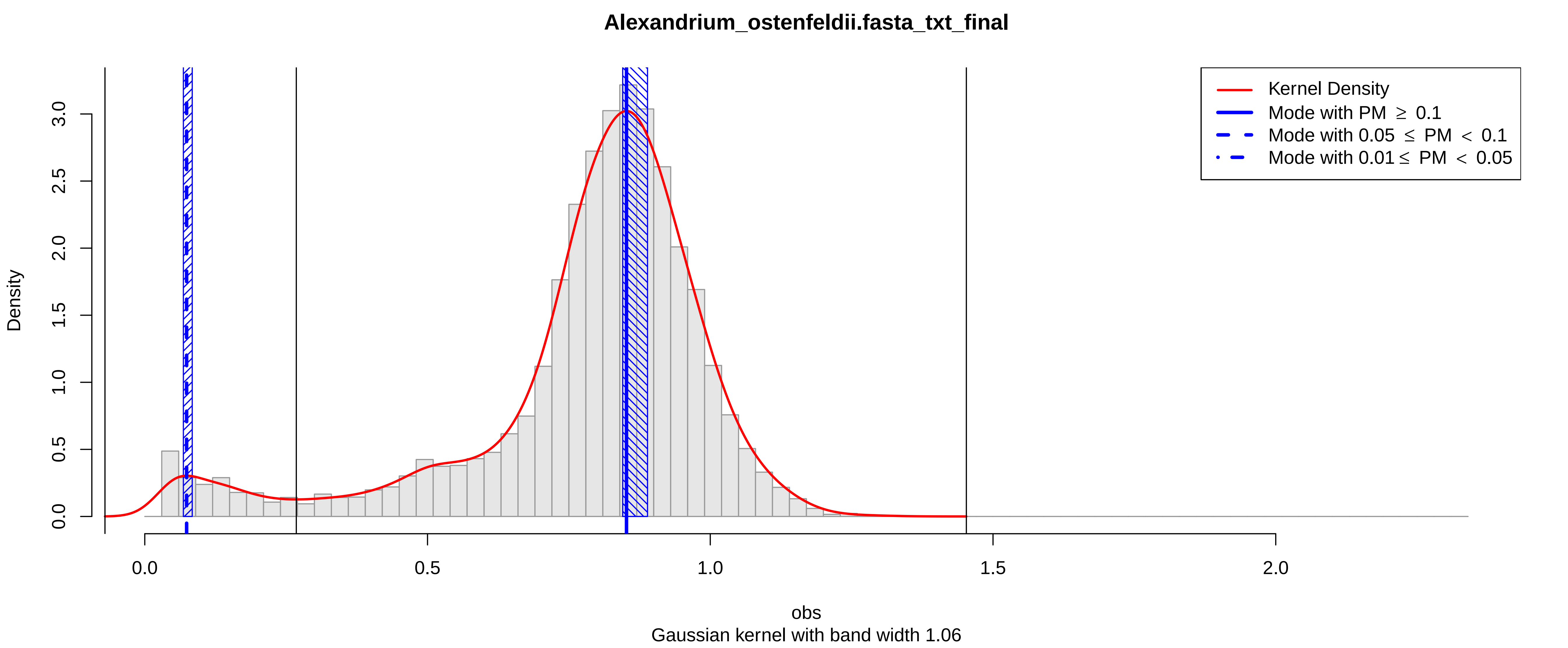

Alexandrium_ostenfeldii.fasta_txt_final
Kernel Density
Mode with PM ≥ 0.1
Mode with 0.05 ≤ PM < 0.1
Mode with 0.01 ≤ PM < 0.05
Density
0.0
0.5
1.0
1.5
2.0
2.5
3.0
obs
Gaussian kernel with band width 1.06

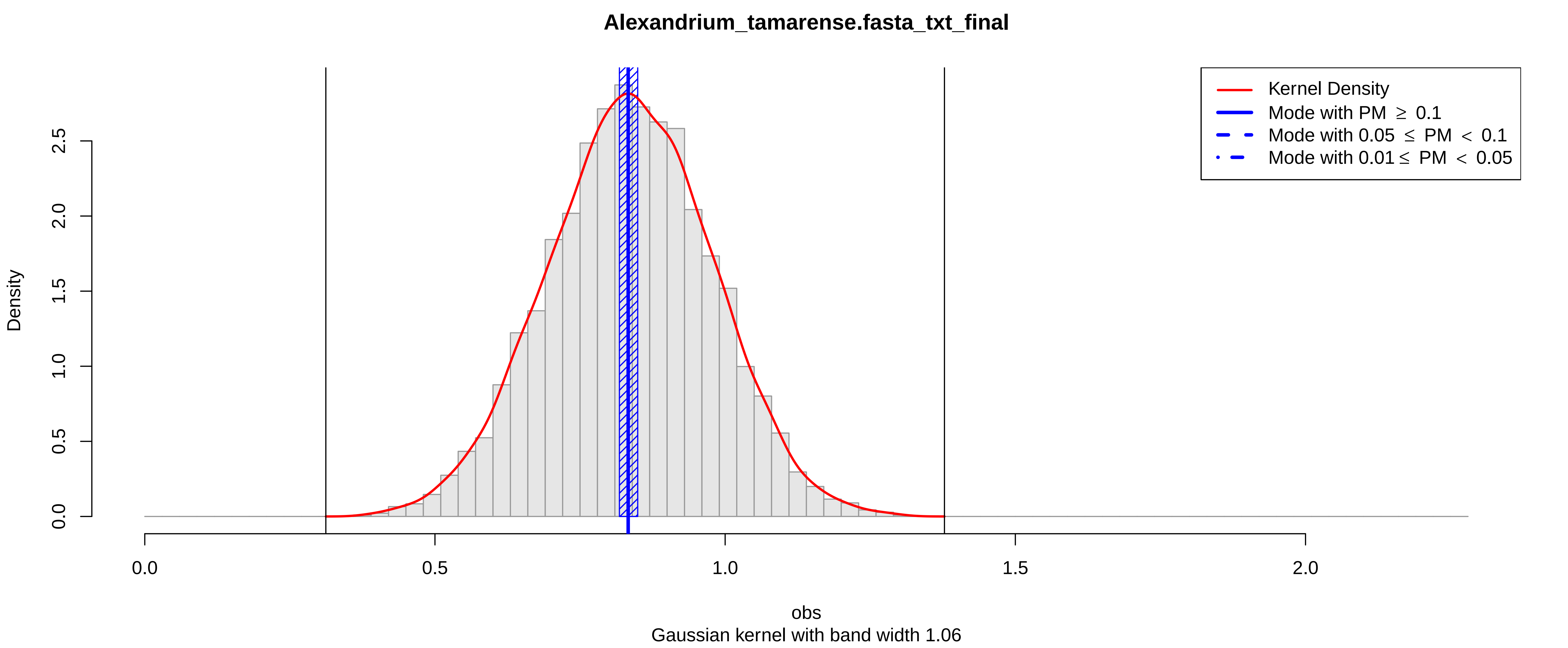
Alexandrium_tamarense.fasta_txt_final
Kernel Density
Mode with PM ≥ 0.1
Mode with 0.05 ≤ PM < 0.1
Mode with 0.01 ≤ PM < 0.05
Density
0.0
0.5
1.0
1.5
2.0
2.5
0.0
0.5
1.0
1.5
2.0
obs
Gaussian kernel with band width 1.06

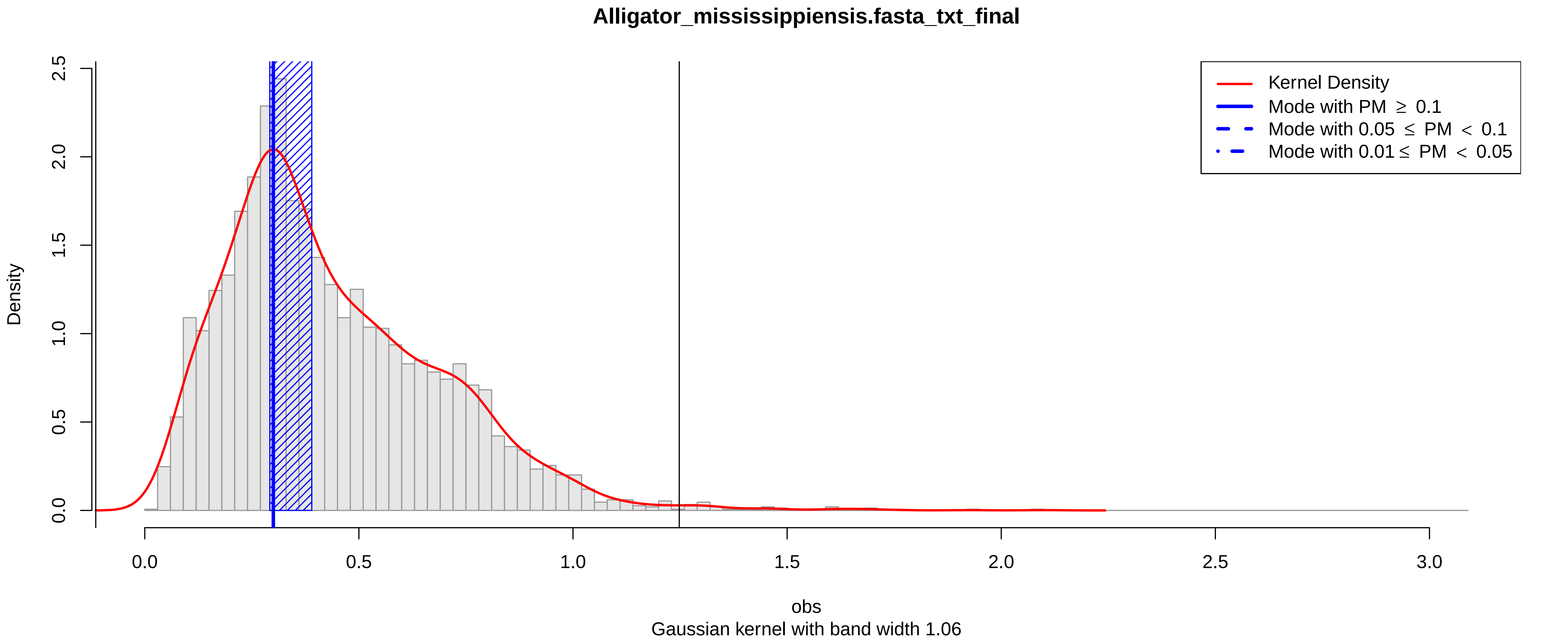
Alligator_mississippiensis.fasta_txt_final
Density
obs
Gaussian kernel with band width 1.06
Kernel Density
Mode with PM ≥ 0.1
Mode with 0.05 ≤ PM < 0.1
Mode with 0.01 ≤ PM < 0.05
0.0
0.5
1.0
1.5
2.0
2.5
3.0

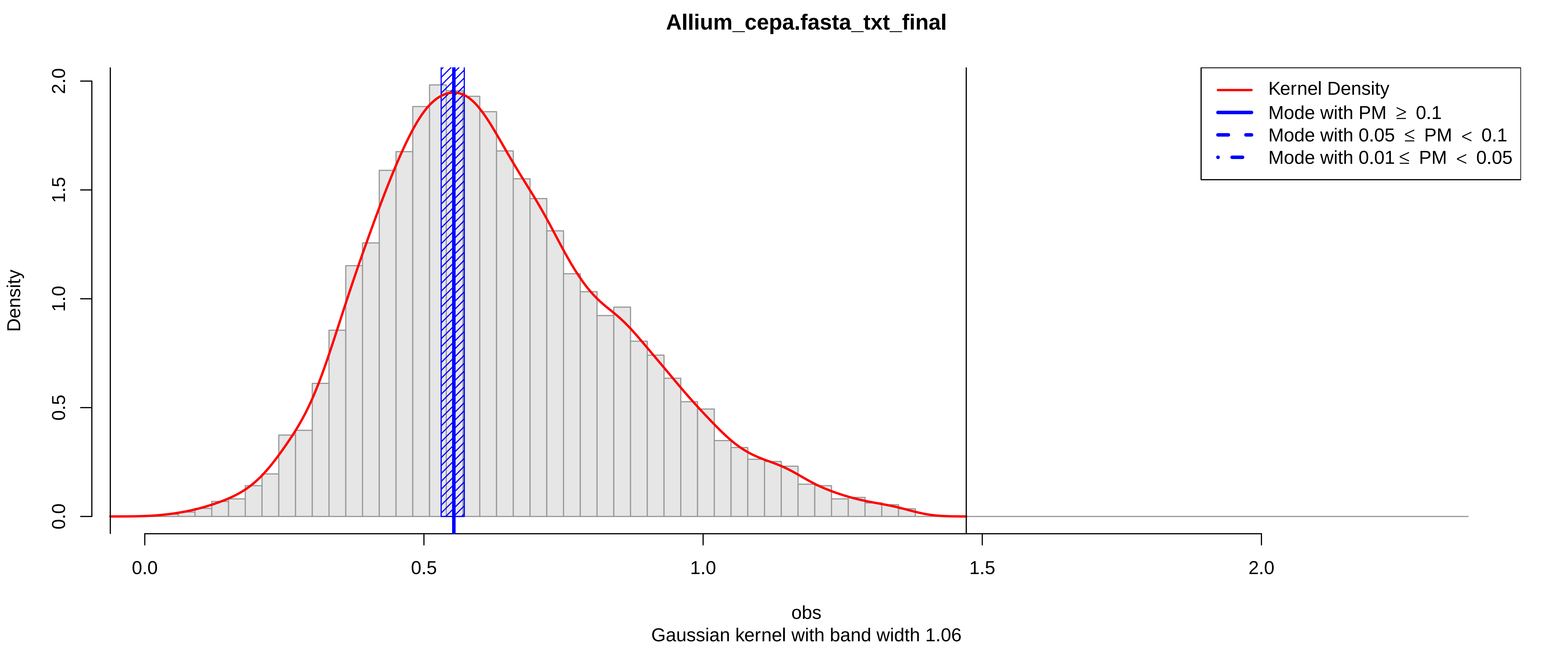

Allium_cepa.fasta_txt_final
Kernel Density
Mode with PM ≥ 0.1
Mode with 0.05 ≤ PM < 0.1
Mode with 0.01 ≤ PM < 0.05
Density
0.0
0.5
1.0
1.5
2.0
0.0
0.5
1.0
1.5
2.0
obs
Gaussian kernel with band width 1.06

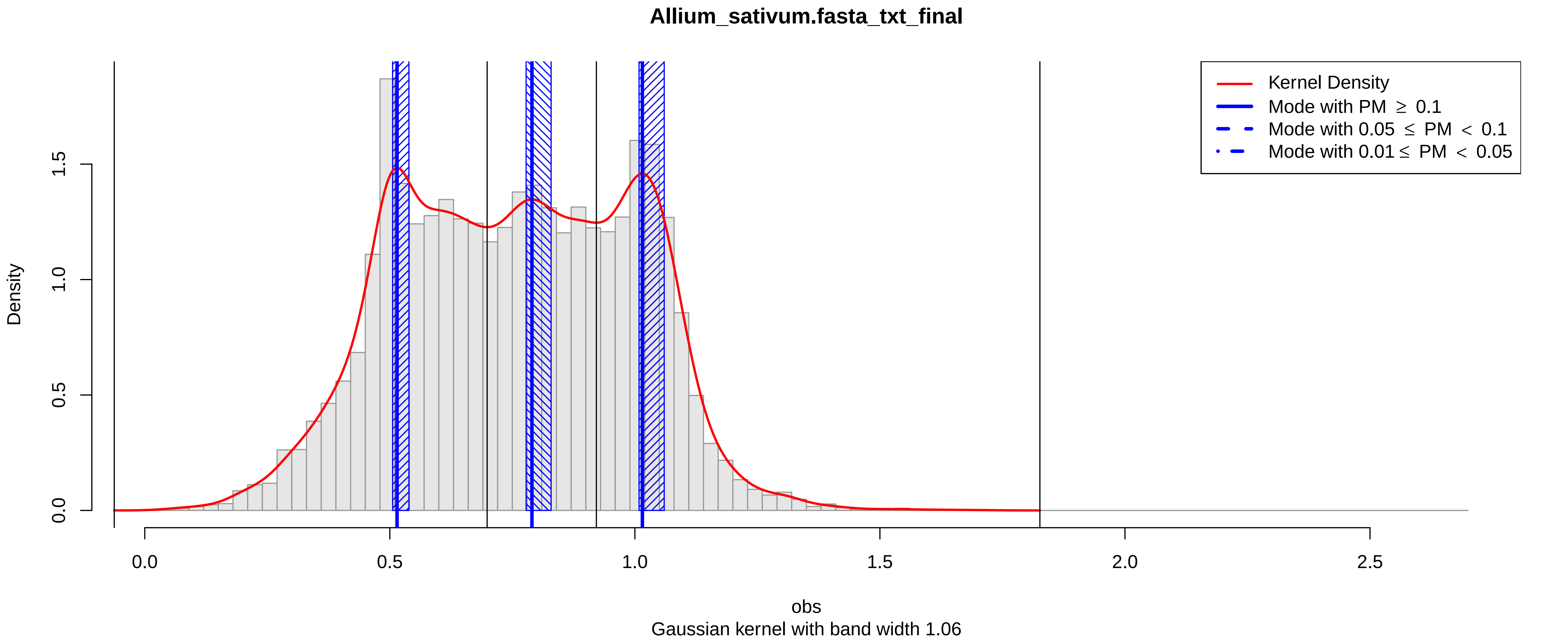
Allium_sativum.fasta_txt_final
Kernel Density
Mode with PM ≥ 0.1
Mode with 0.05 ≤ PM < 0.1
Mode with 0.01 ≤ PM < 0.05
Density
0.0
0.5
1.0
1.5
0.0
0.5
1.0
1.5
2.0
2.5
obs
Gaussian kernel with band width 1.06

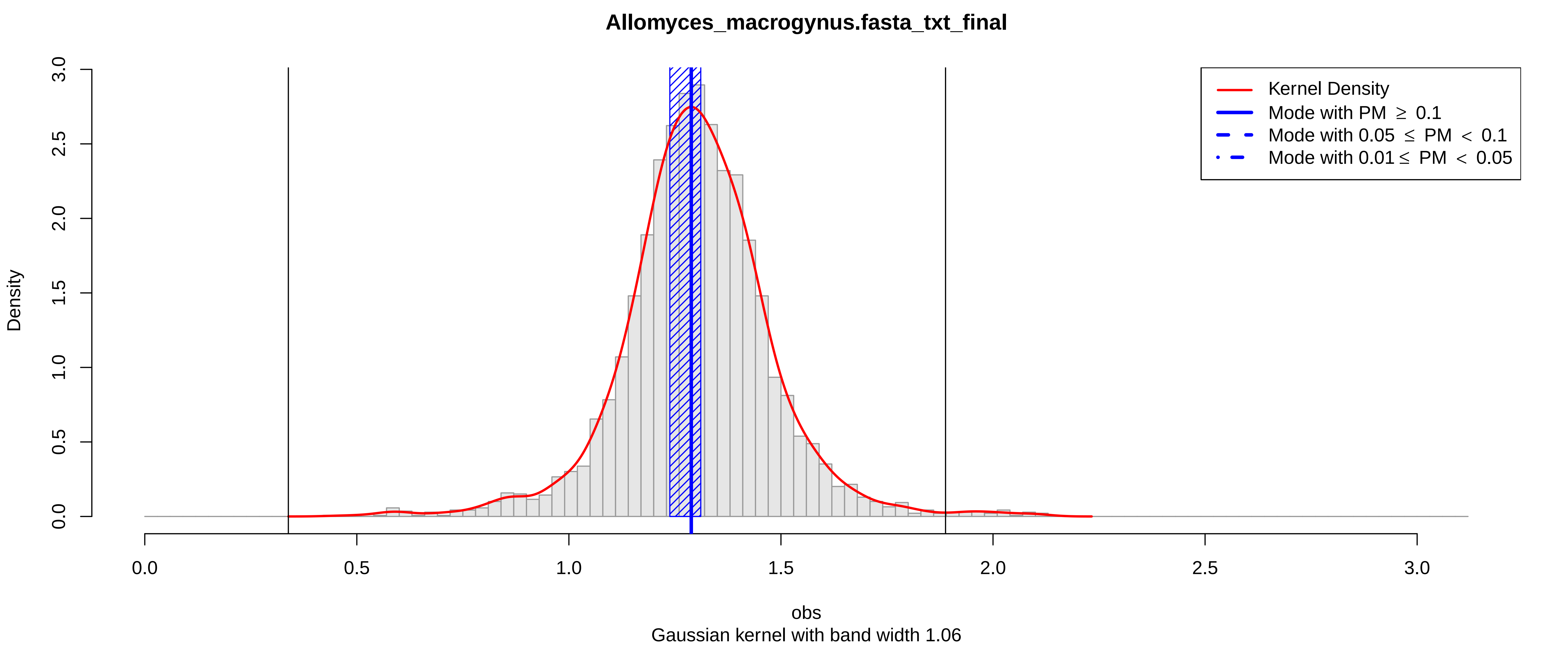
Allomyces_macrogynus.fasta_txt_final
Kernel Density
Mode with PM ≥ 0.1
Mode with 0.05 ≤ PM < 0.1
Mode with 0.01 ≤ PM < 0.05
Density
0.0
0.5
1.0
1.5
2.0
2.5
3.0
obs
Gaussian kernel with band width 1.06

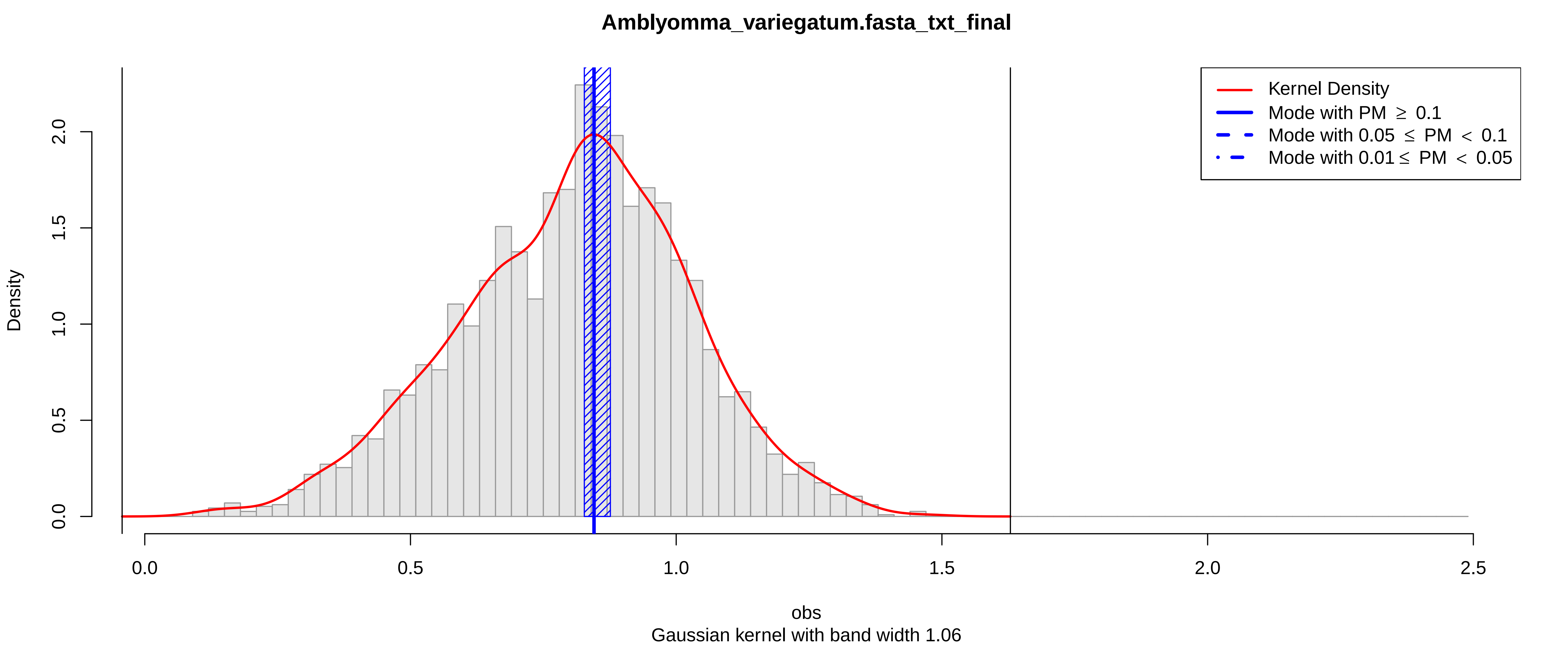
Amblyomma_variegatum.fasta_txt_final
Kernel Density
Mode with PM ≥ 0.1
Mode with 0.05 ≤ PM < 0.1
Mode with 0.01 ≤ PM < 0.05
Density
0.0
0.5
1.0
1.5
2.0
0.0
0.5
1.0
1.5
2.0
2.5
obs
Gaussian kernel with band width 1.06

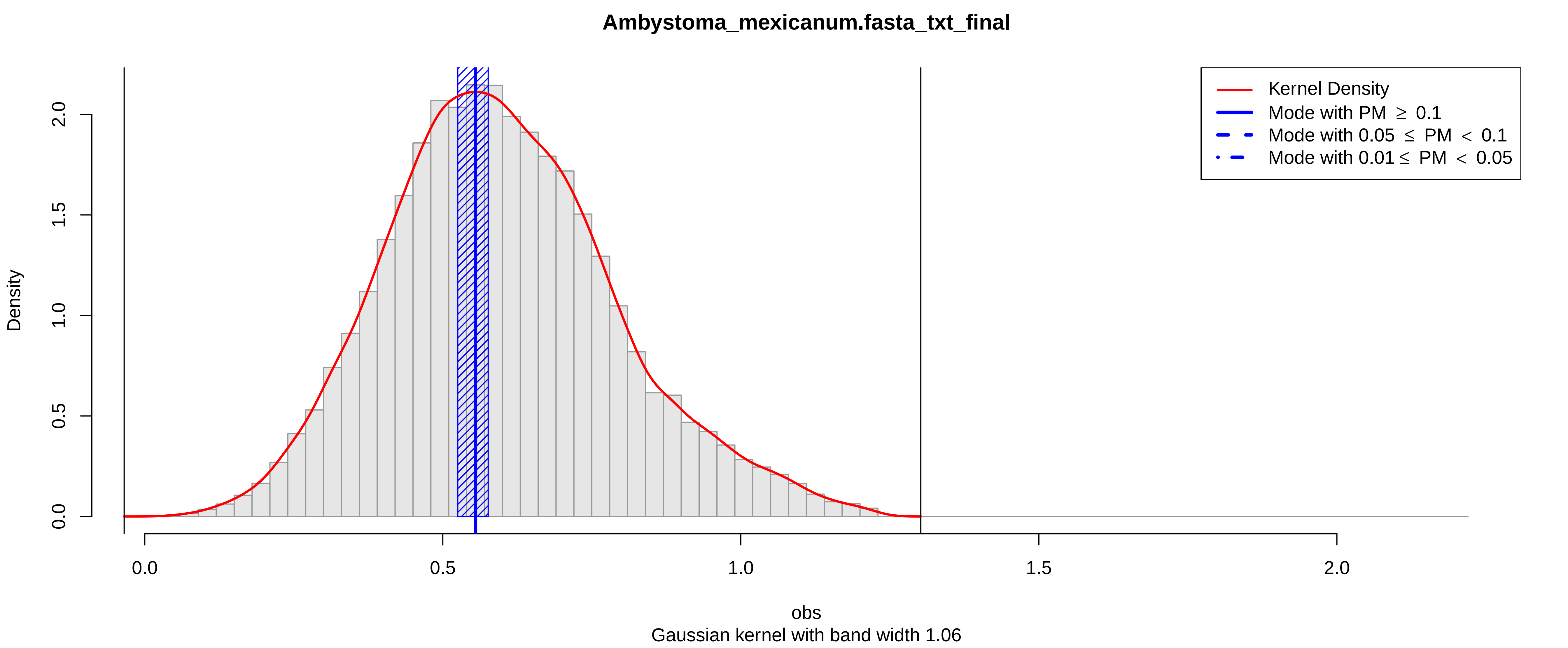

Ambystoma_mexicanum.fasta_txt_final
Kernel Density
Mode with PM ≥ 0.1
Mode with 0.05 ≤ PM < 0.1
Mode with 0.01 ≤ PM < 0.05
Density
0.0
0.5
1.0
1.5
2.0
0.0
0.5
1.0
1.5
2.0
obs
Gaussian kernel with band width 1.06

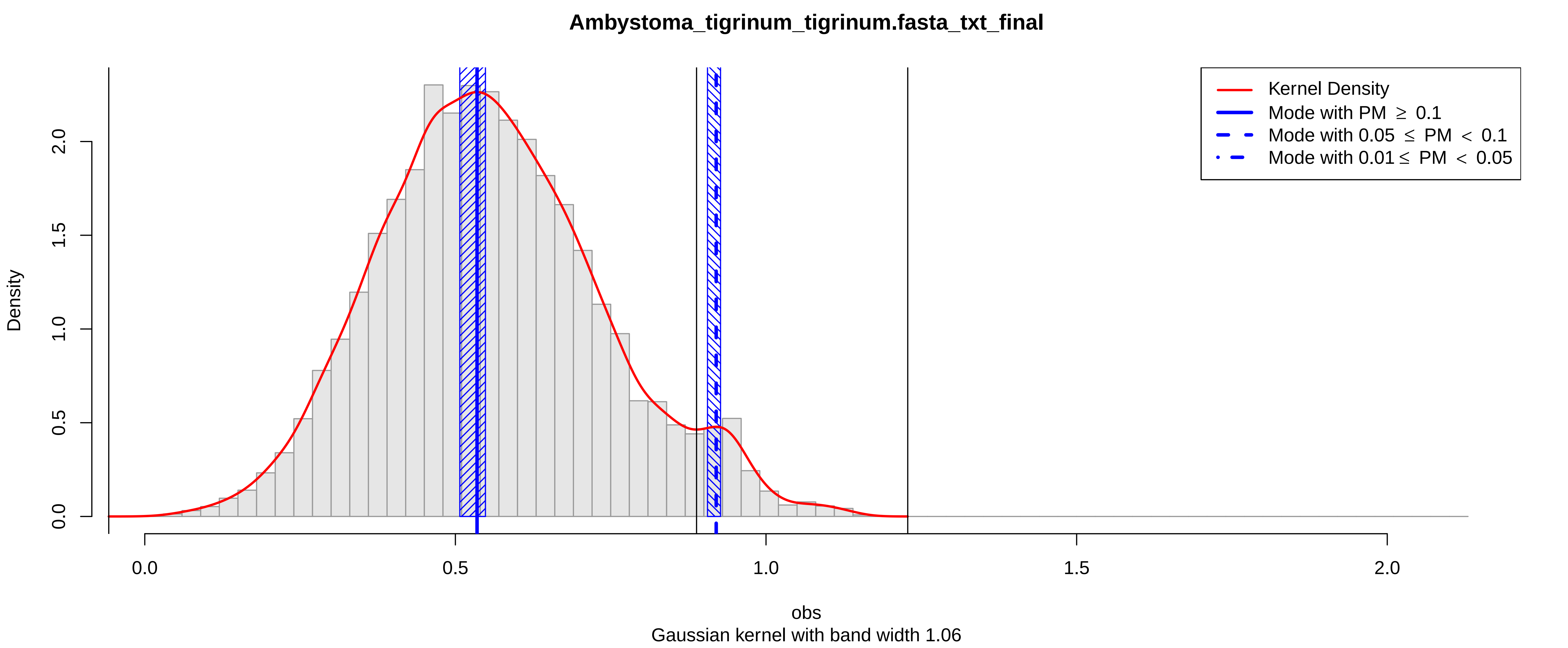

Ambystoma_tigrinum_tigrinum.fasta_txt_final
Kernel Density
Mode with PM ≥ 0.1
Mode with 0.05 ≤ PM < 0.1
Mode with 0.01 ≤ PM < 0.05
Density
0.0
0.5
1.0
1.5
2.0
obs
Gaussian kernel with band width 1.06

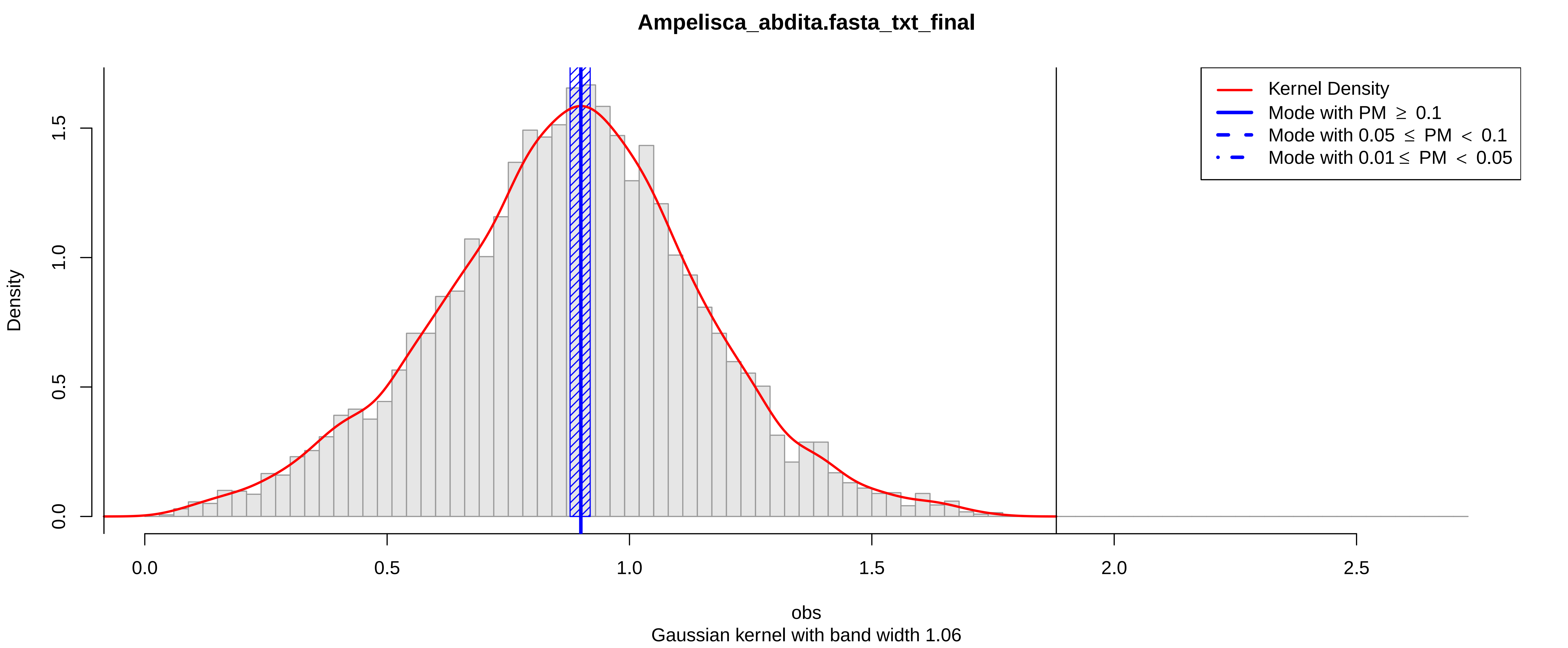

Ampelisca_abdita.fasta_txt_final
Density
0.0
0.5
1.0
1.5
0.0
0.5
1.0
1.5
2.0
2.5
obs
Gaussian kernel with band width 1.06
Kernel Density
Mode with PM ≥ 0.1
Mode with 0.05 ≤ PM < 0.1
Mode with 0.01 ≤ PM < 0.05

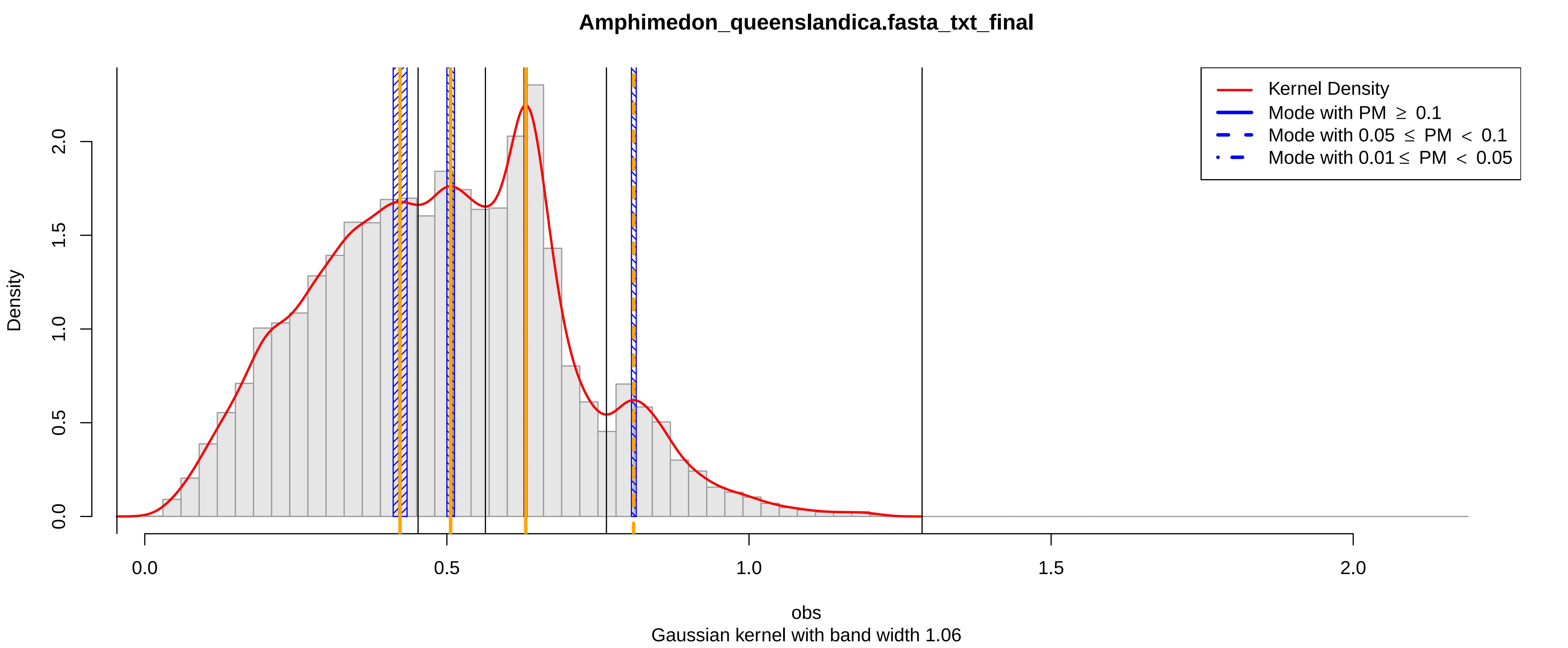
Amphimedon_queenslandica.fasta_txt_final
Kernel Density
Mode with PM ≥ 0.1
Mode with 0.05 ≤ PM < 0.1
Mode with 0.01 ≤ PM < 0.05
Density
0.0
0.5
1.0
1.5
2.0
obs
Gaussian kernel with band width 1.06

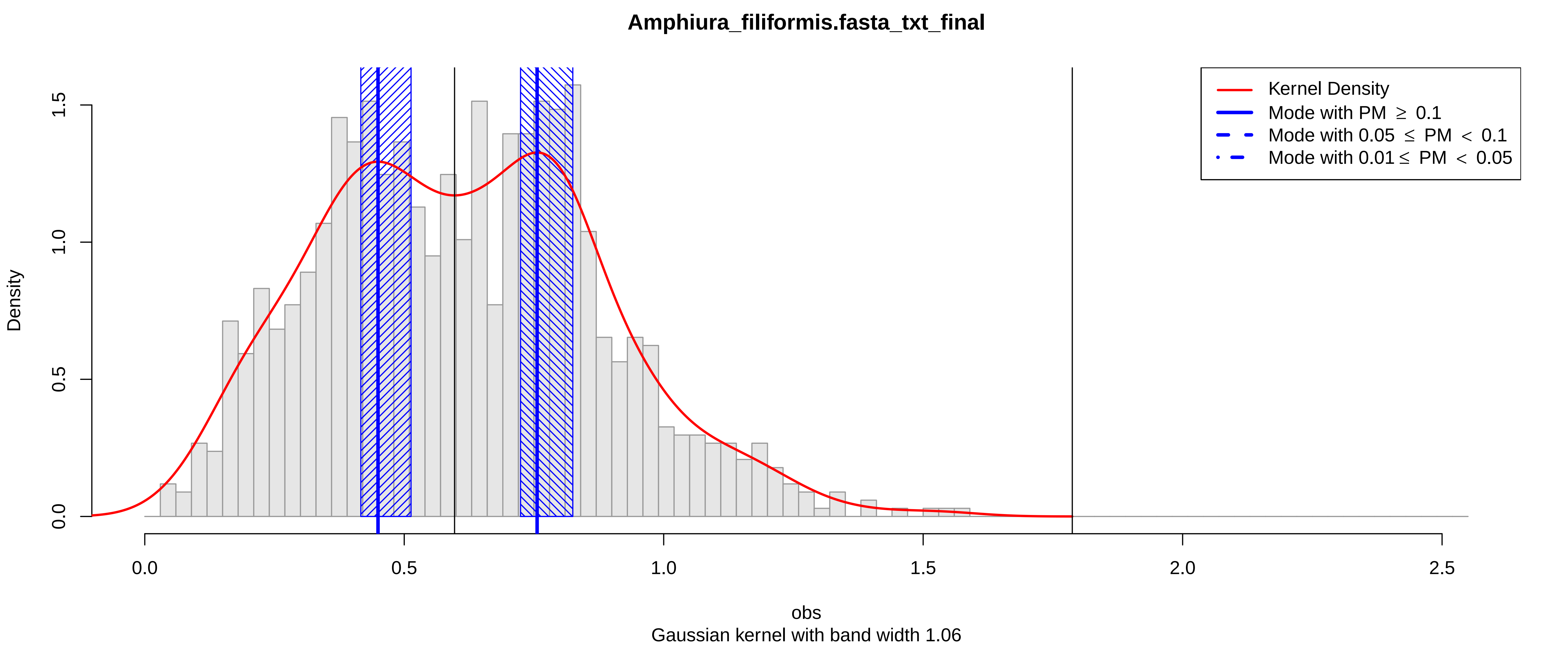

Amphiura_filiformis.fasta_txt_final
Kernel Density
Mode with PM ≥ 0.1
Mode with 0.05 ≤ PM < 0.1
Mode with 0.01 ≤ PM < 0.05
Density
0.0
0.5
1.0
1.5
0.0
0.5
1.0
1.5
2.0
2.5
obs
Gaussian kernel with band width 1.06

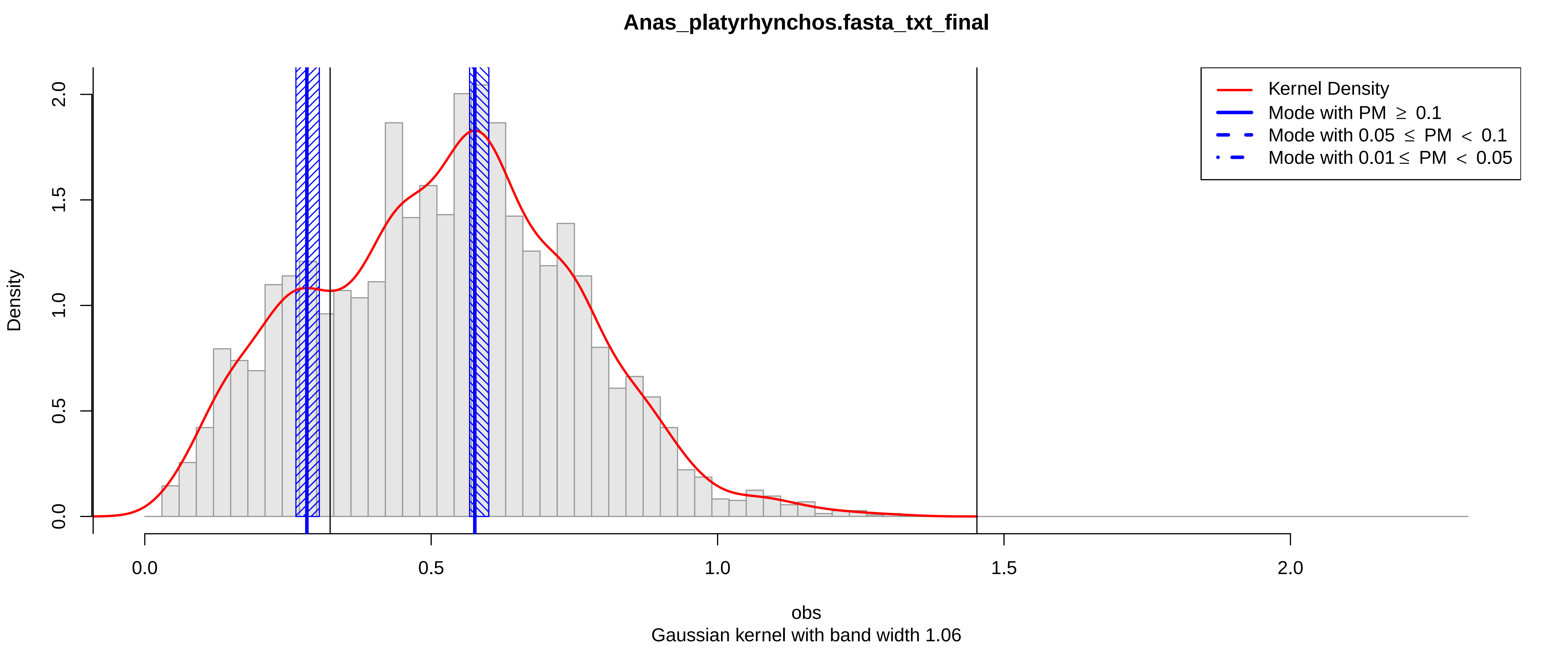

Anas_platyrhynchos.fasta_txt_final
Kernel Density
Mode with PM ≥ 0.1
Mode with 0.05 ≤ PM < 0.1
Mode with 0.01 ≤ PM < 0.05
Density
0.0
0.5
1.0
1.5
2.0
obs
Gaussian kernel with band width 1.06

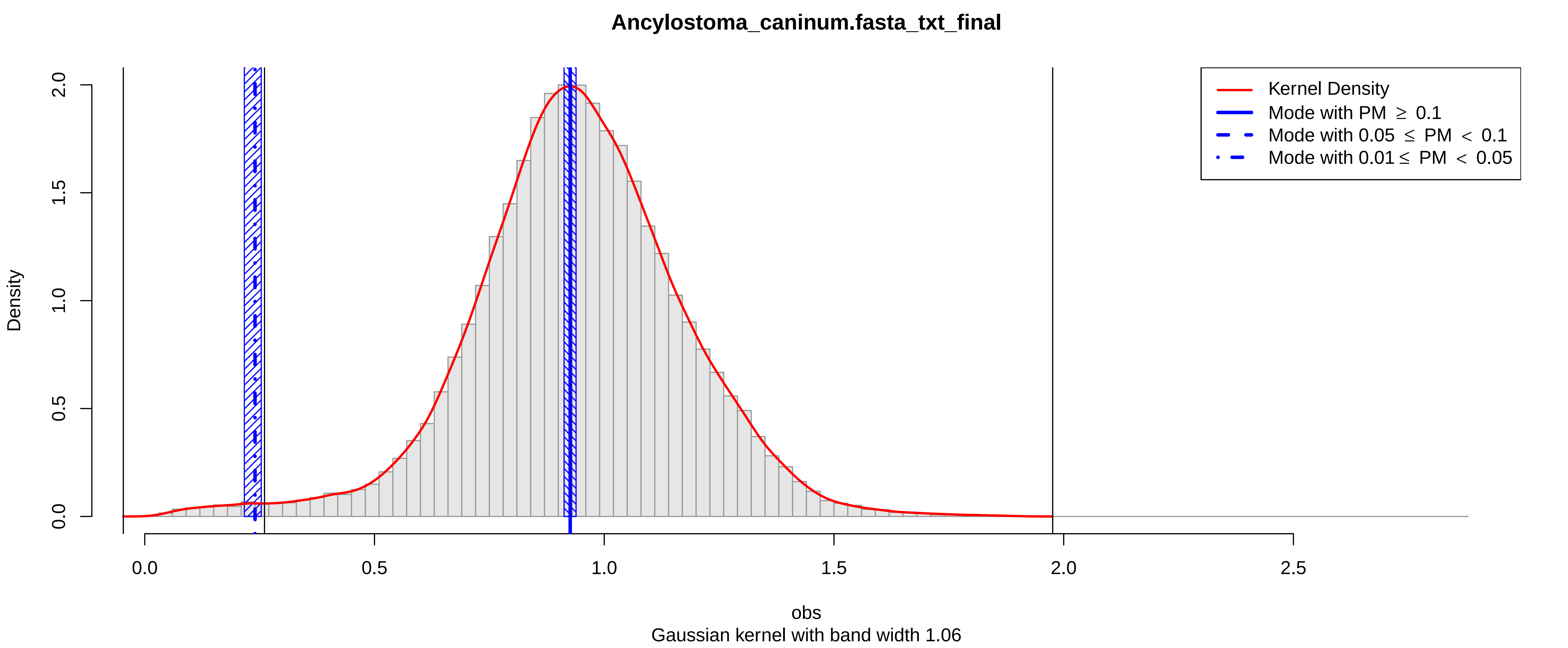
Ancylostoma_caninum.fasta_txt_final
Kernel Density
Mode with PM ≥ 0.1
Mode with 0.05 ≤ PM < 0.1
Mode with 0.01 ≤ PM < 0.05
Density
0.0
0.5
1.0
1.5
2.0
2.5
obs
Gaussian kernel with band width 1.06

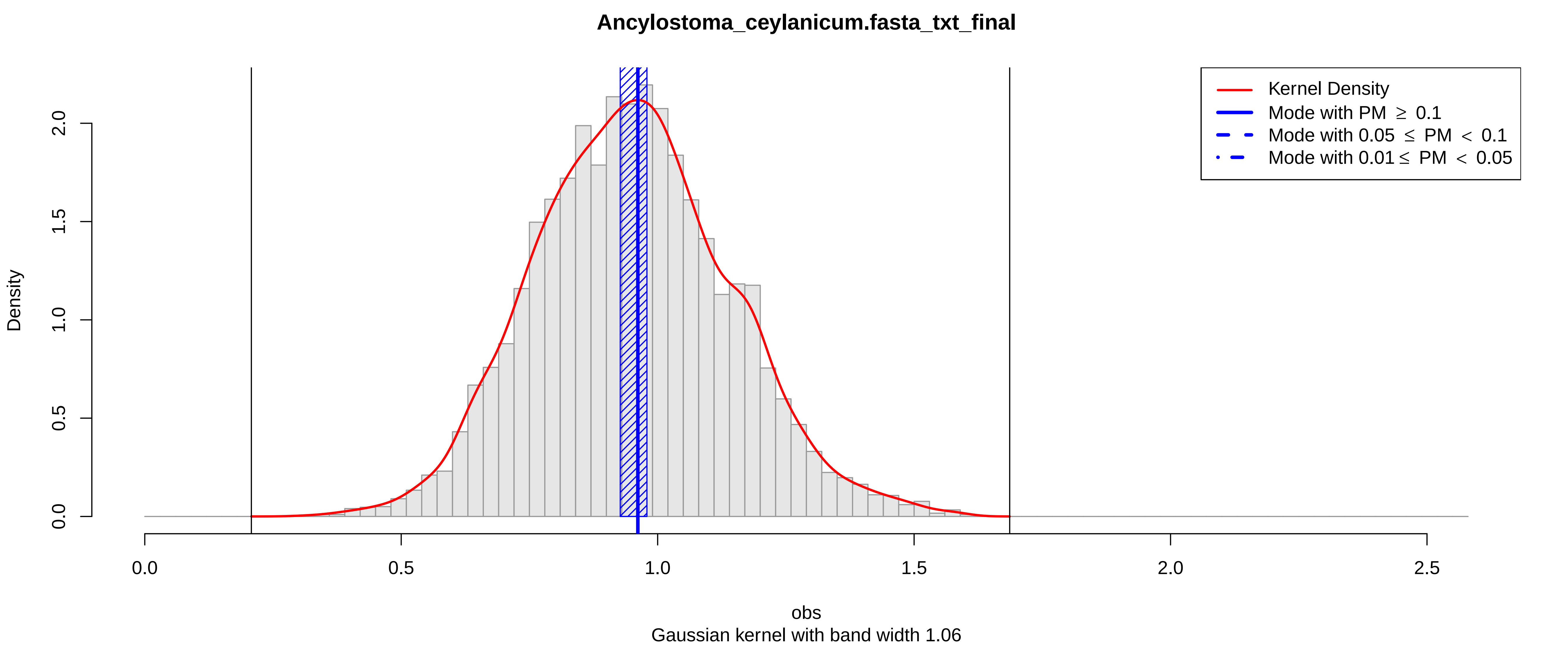
Ancylostoma_ceylanicum.fasta_txt_final
Kernel Density
Mode with PM ≥ 0.1
Mode with 0.05 ≤ PM < 0.1
Mode with 0.01 ≤ PM < 0.05
Density
0.0
0.5
1.0
1.5
2.0
0.0
0.5
1.0
1.5
2.0
2.5
obs
Gaussian kernel with band width 1.06

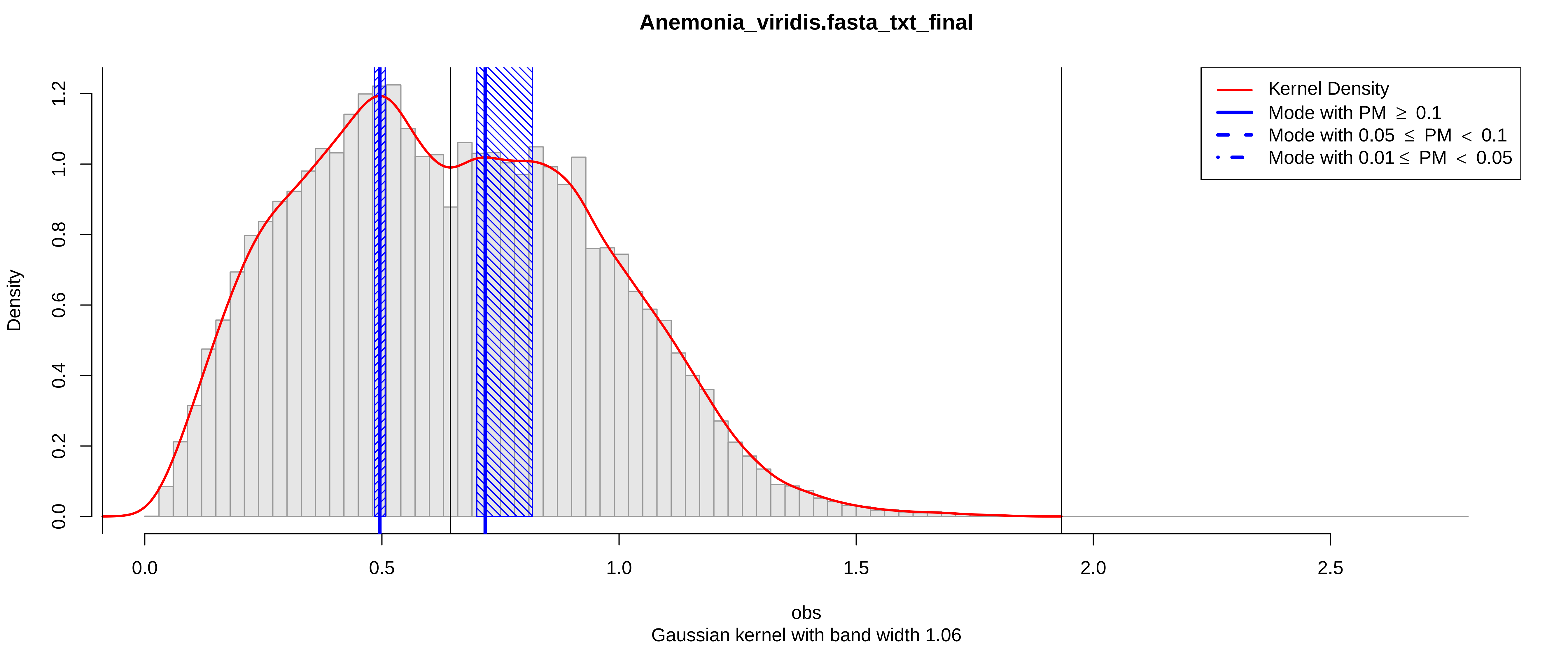
Anemonia_viridis.fasta_txt_final
Kernel Density
Mode with PM ≥ 0.1
Mode with 0.05 ≤ PM < 0.1
Mode with 0.01 ≤ PM < 0.05
Density
0.0
0.2
0.4
0.6
0.8
1.0
1.2
0.0
0.5
1.0
1.5
2.0
2.5
obs
Gaussian kernel with band width 1.06

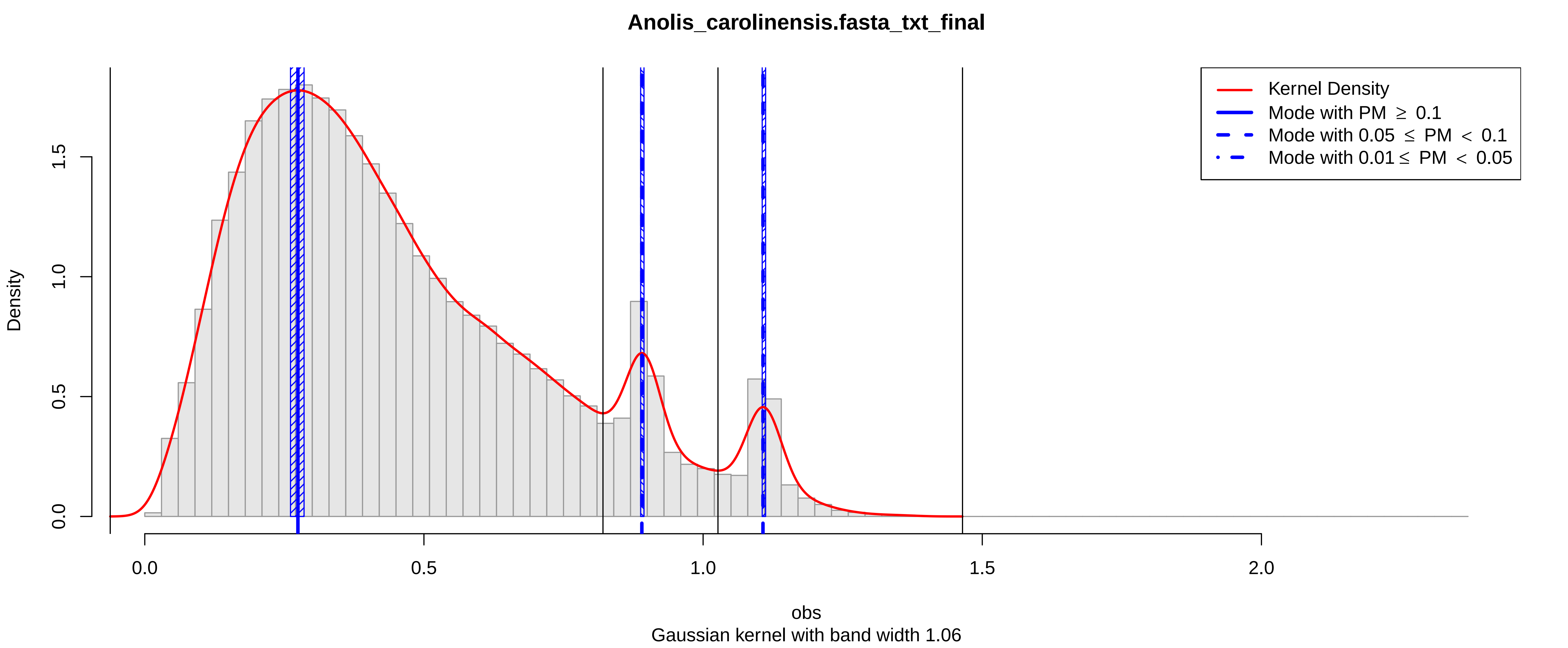

Anolis_carolinensis.fasta_txt_final
Kernel Density
Mode with PM ≥ 0.1
Mode with 0.05 ≤ PM < 0.1
Mode with 0.01 ≤ PM < 0.05
Density
0.0
0.5
1.0
1.5
2.0
obs
Gaussian kernel with band width 1.06

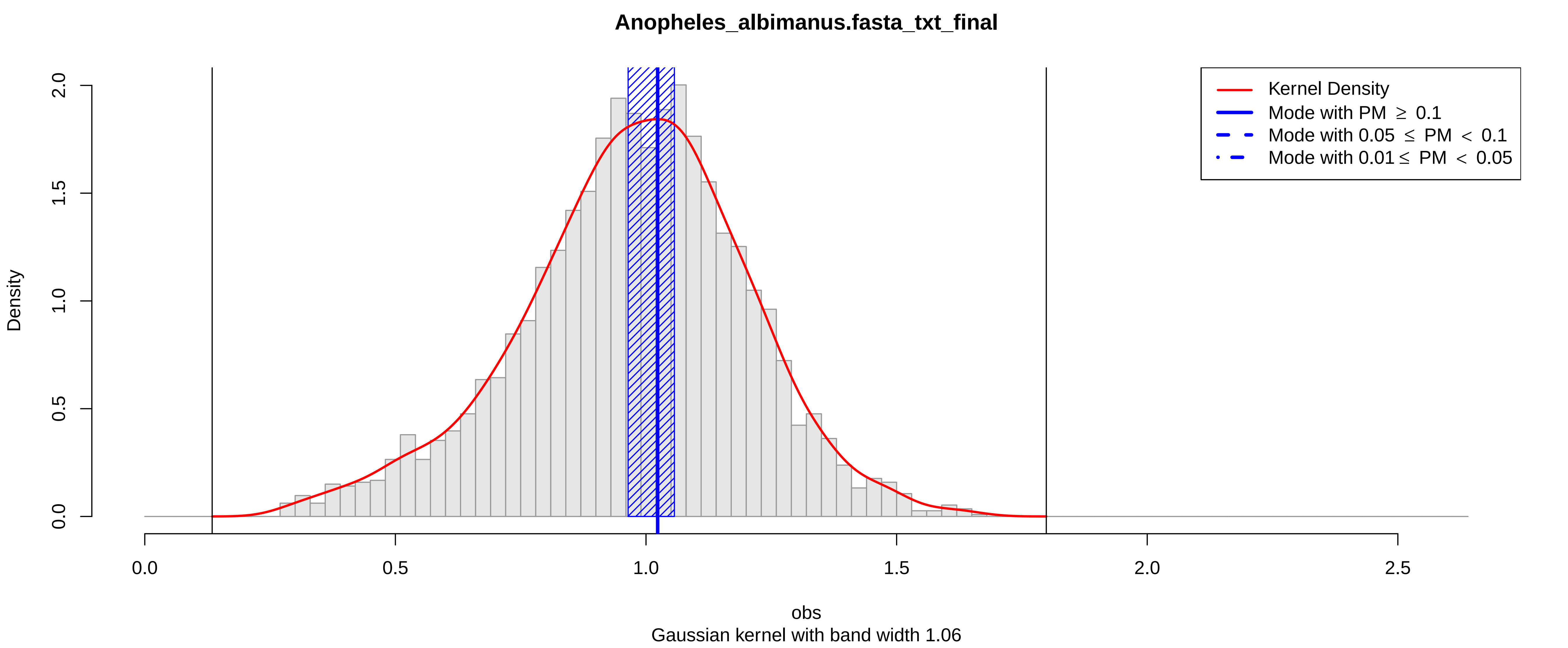

Anopheles_albimanus.fasta_txt_final
Kernel Density
Mode with PM ≥ 0.1
Mode with 0.05 ≤ PM < 0.1
Mode with 0.01 ≤ PM < 0.05
Density
0.0
0.5
1.0
1.5
2.0
2.5
obs
Gaussian kernel with band width 1.06

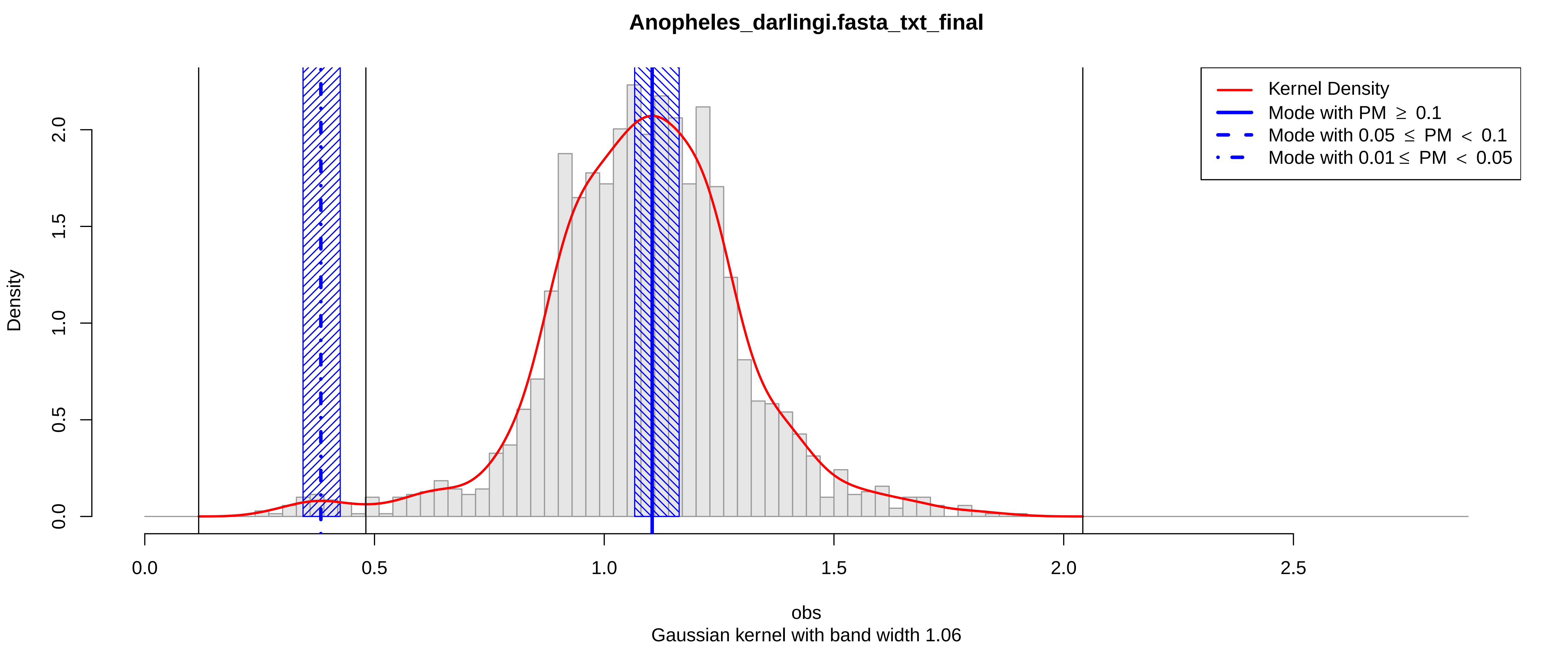

Anopheles_darlingi.fasta_txt_final
Kernel Density
Mode with PM ≥ 0.1
Mode with 0.05 ≤ PM < 0.1
Mode with 0.01 ≤ PM < 0.05
Density
0.0
0.5
1.0
1.5
2.0
0.0
0.5
1.0
1.5
2.0
2.5
obs
Gaussian kernel with band width 1.06

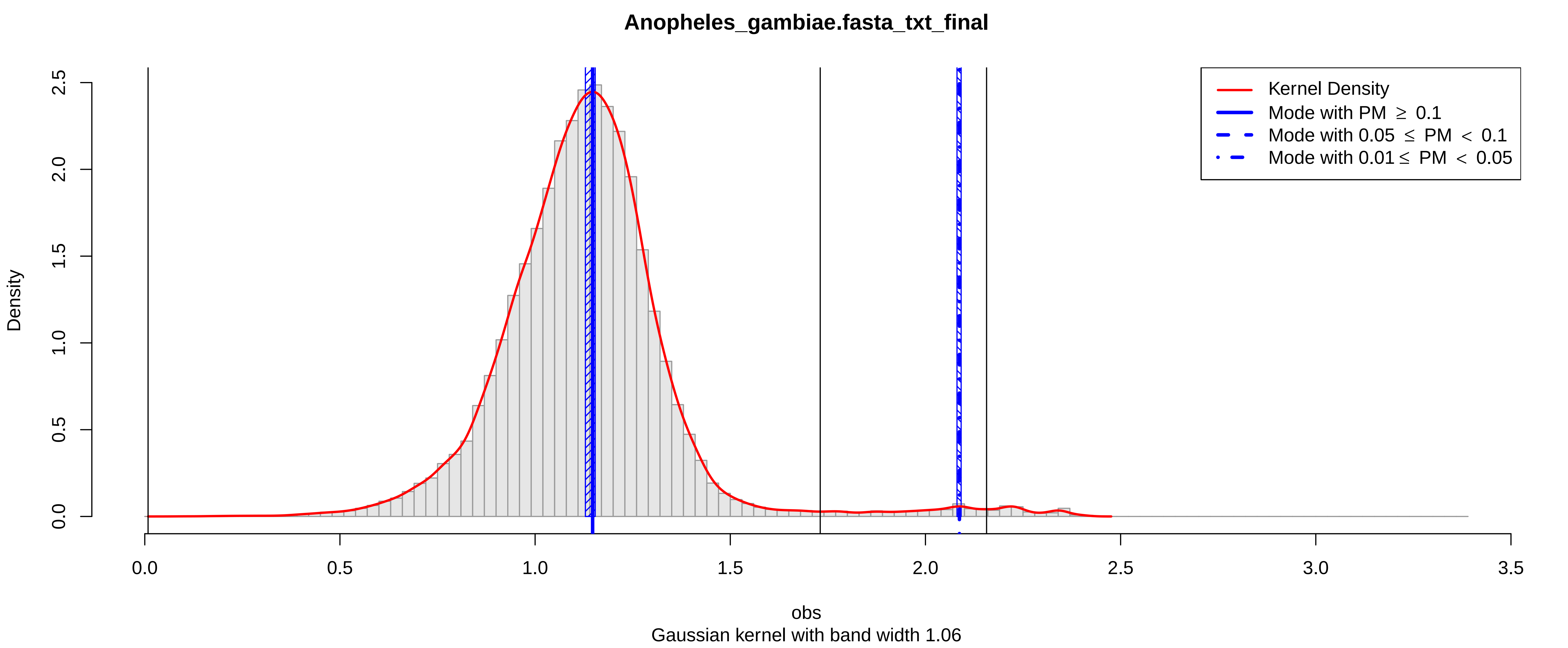

Anopheles_gambiae.fasta_txt_final
Kernel Density
Mode with PM ≥ 0.1
Mode with 0.05 ≤ PM < 0.1
Mode with 0.01 ≤ PM < 0.05
Density
0.0
0.5
1.0
1.5
2.0
2.5
3.0
3.5
obs
Gaussian kernel with band width 1.06

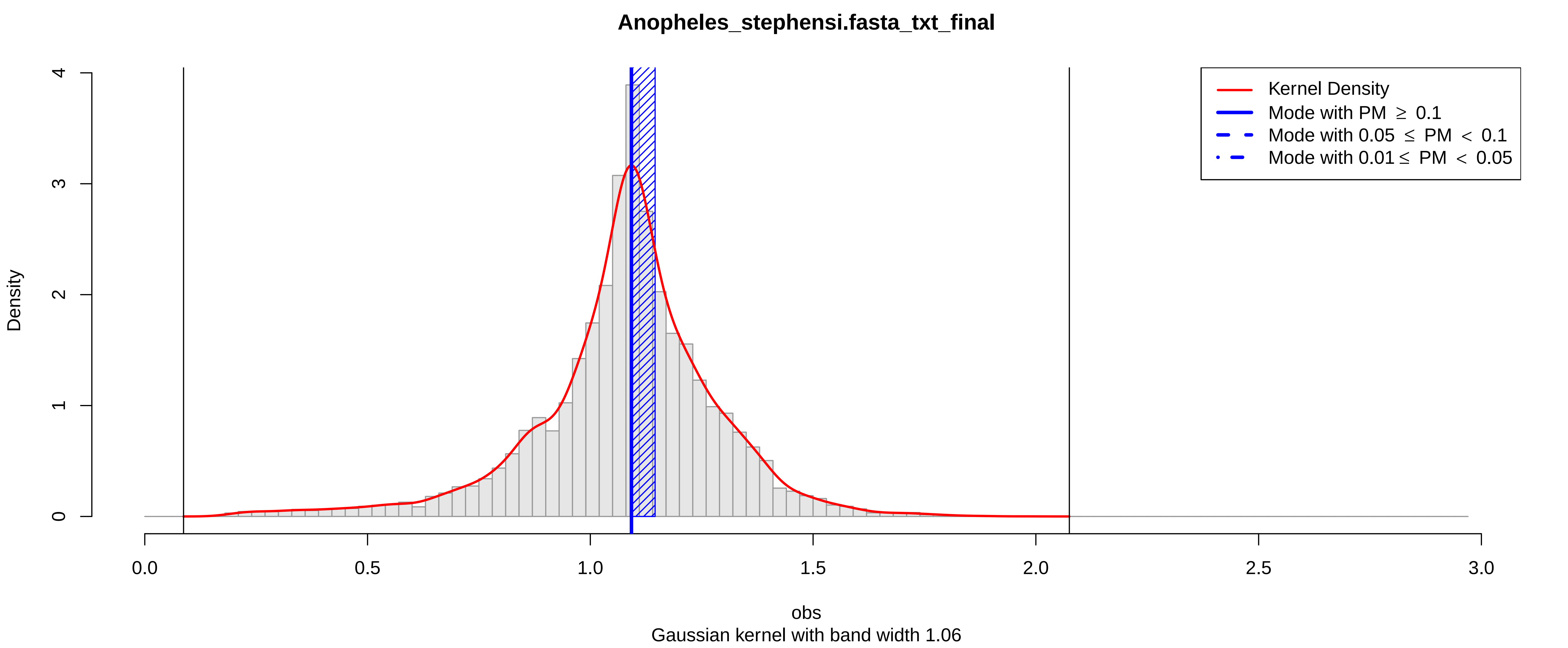

Anopheles_stephensi.fasta_txt_final
Kernel Density
Mode with PM ≥ 0.1
Mode with 0.05 ≤ PM < 0.1
Mode with 0.01 ≤ PM < 0.05
Density
0
1
2
3
4
0.0
0.5
1.0
1.5
2.0
2.5
3.0
obs
Gaussian kernel with band width 1.06

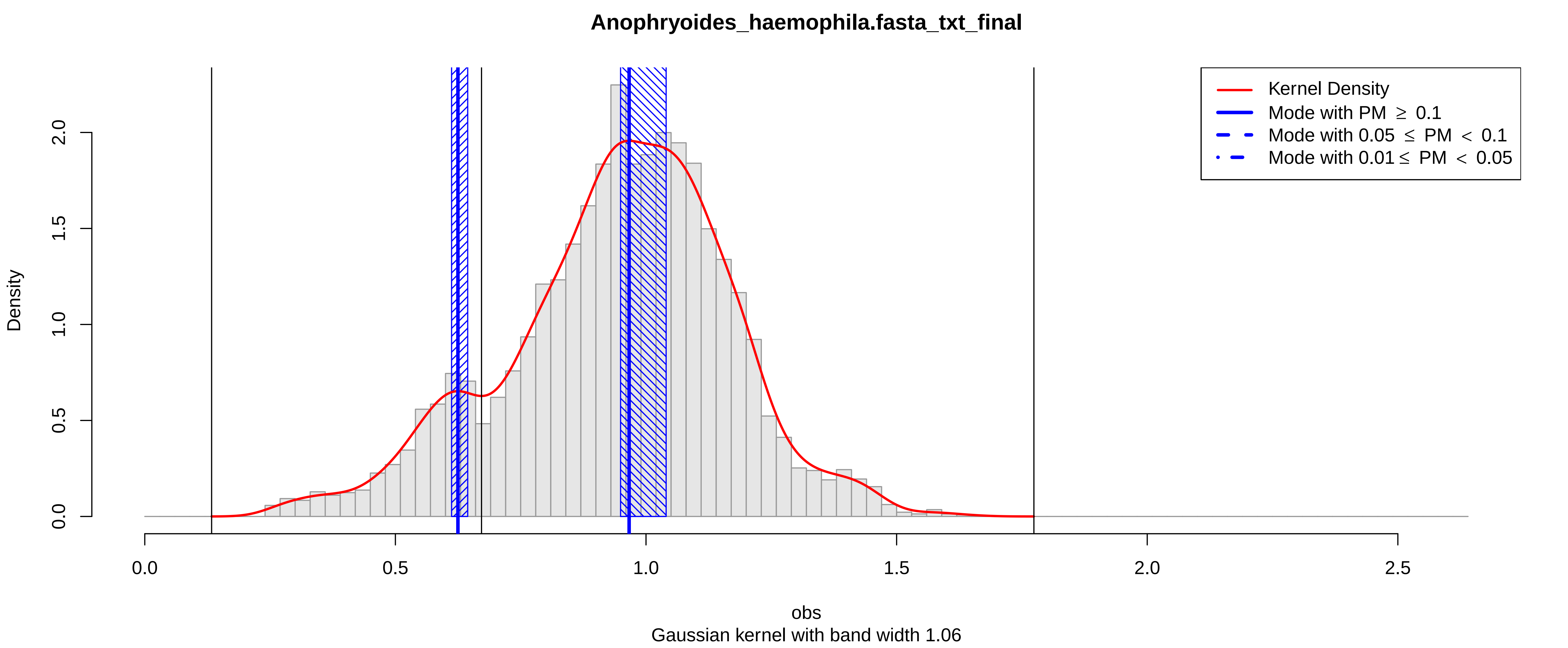
Anophryoides_haemophila.fasta_txt_final
Kernel Density
Mode with PM ≥ 0.1
Mode with 0.05 ≤ PM < 0.1
Mode with 0.01 ≤ PM < 0.05
Density
0.0
0.5
1.0
1.5
2.0
0.0
0.5
1.0
1.5
2.0
2.5
obs
Gaussian kernel with band width 1.06

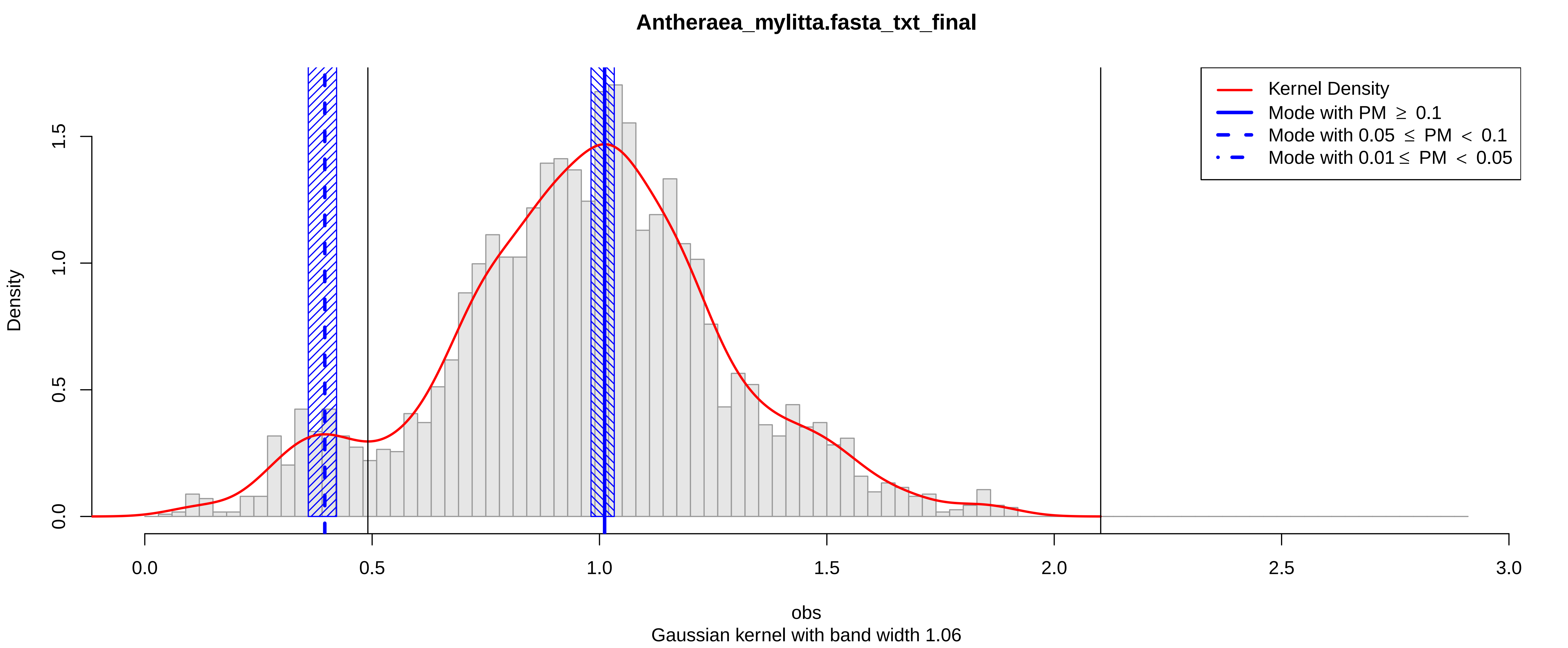

Antheraea_mylitta.fasta_txt_final
Kernel Density
Mode with PM ≥ 0.1
Mode with 0.05 ≤ PM < 0.1
Mode with 0.01 ≤ PM < 0.05
Density
0.0
0.5
1.0
1.5
0.0
0.5
1.0
1.5
2.0
2.5
3.0
obs
Gaussian kernel with band width 1.06

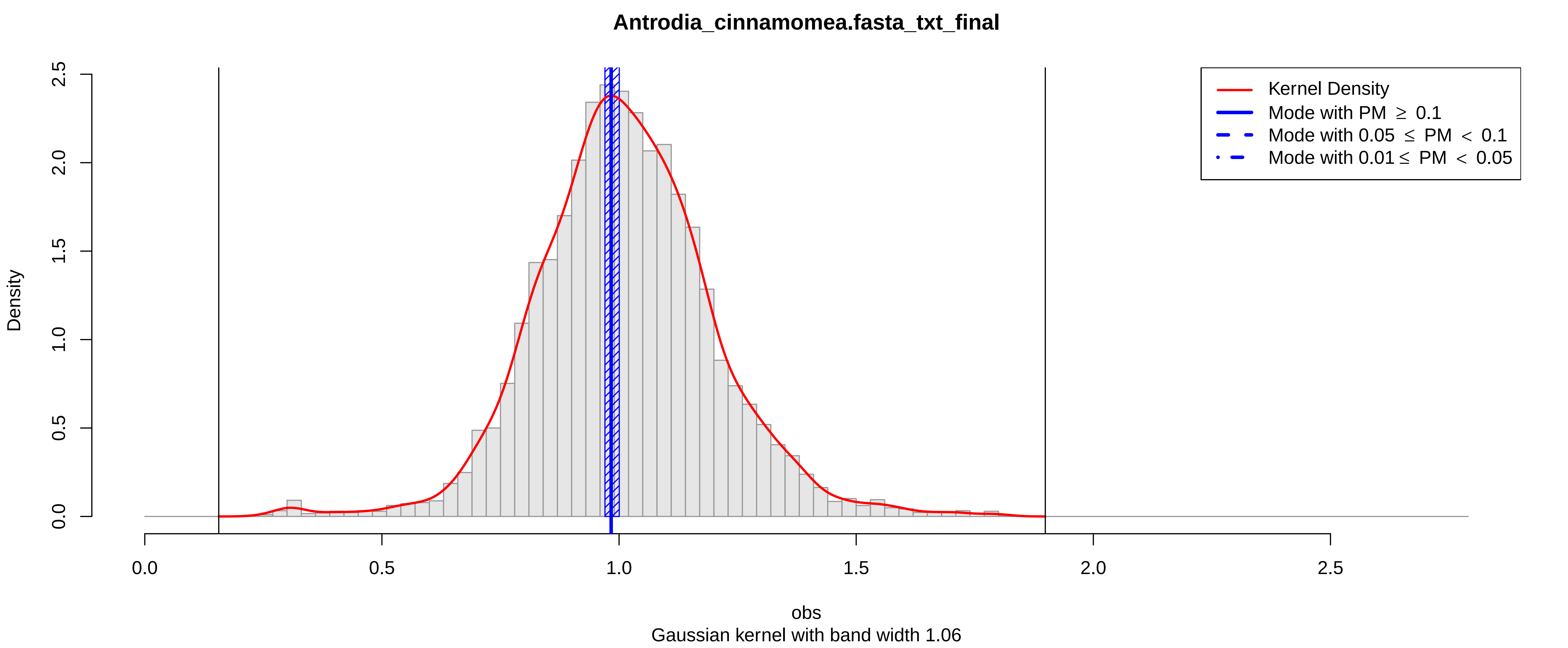

Antrodia_cinnamomea.fasta_txt_final
Kernel Density
Mode with PM ≥ 0.1
Mode with 0.05 ≤ PM < 0.1
Mode with 0.01 ≤ PM < 0.05
Density
0.0
0.5
1.0
1.5
2.0
2.5
obs
Gaussian kernel with band width 1.06

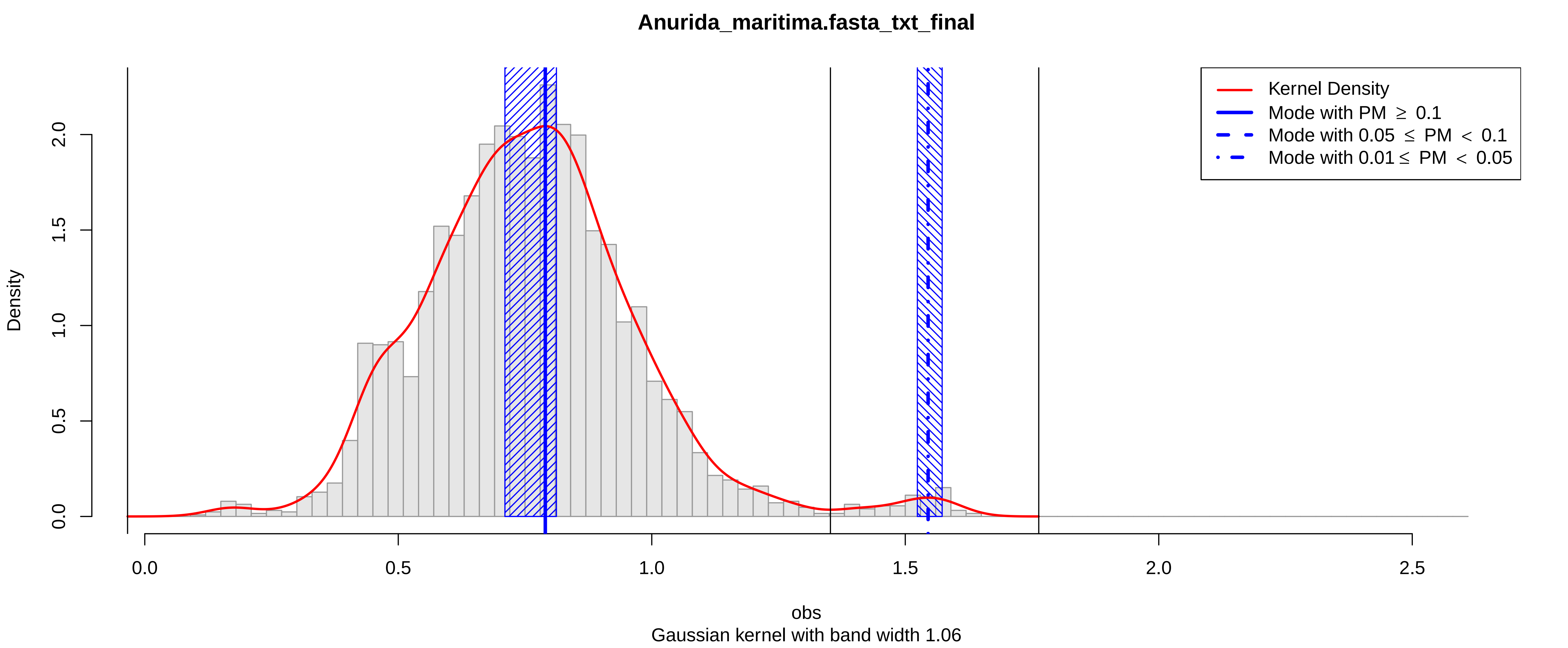

Anurida_maritima.fasta_txt_final
Kernel Density
Mode with PM ≥ 0.1
Mode with 0.05 ≤ PM < 0.1
Mode with 0.01 ≤ PM < 0.05
Density
0.0
0.5
1.0
1.5
2.0
0.0
0.5
1.0
1.5
2.0
2.5
obs
Gaussian kernel with band width 1.06

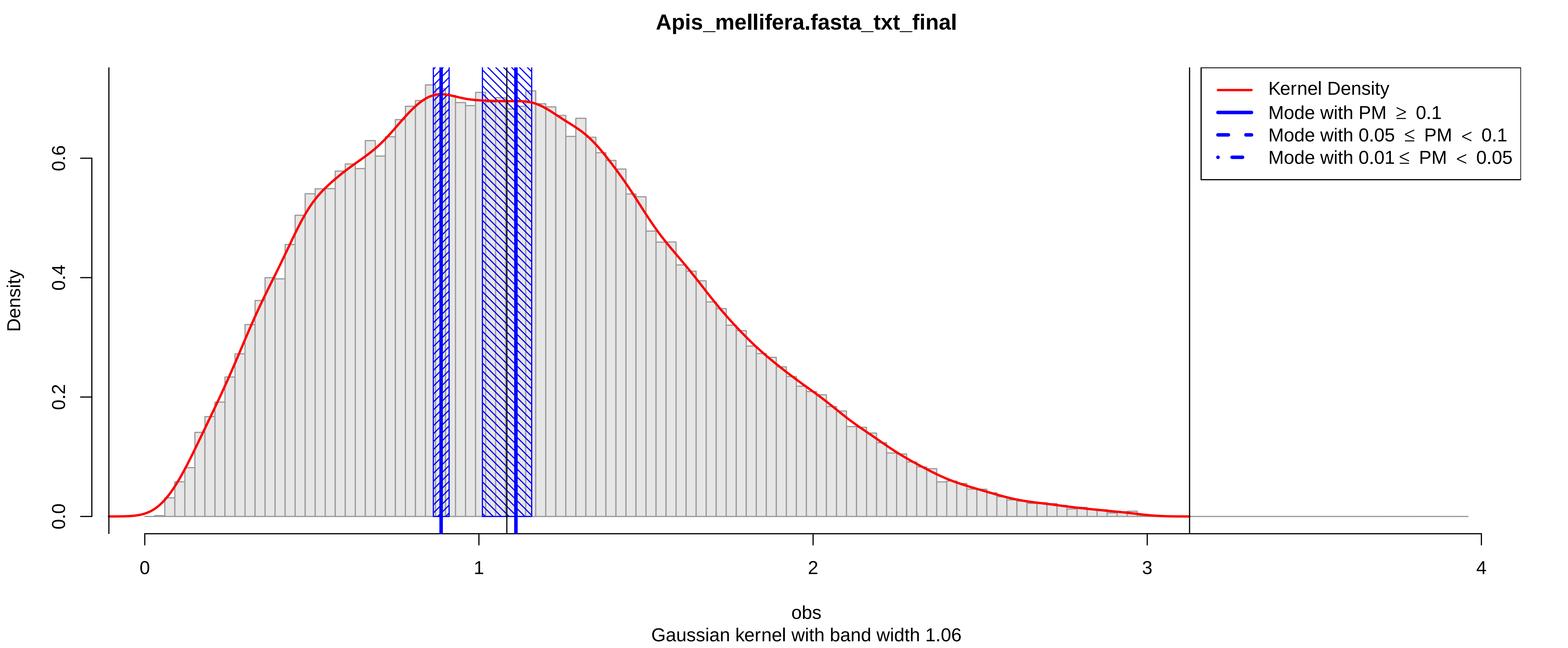
Apis_mellifera.fasta_txt_final
Kernel Density
Mode with PM ≥ 0.1
Mode with 0.05 ≤ PM < 0.1
Mode with 0.01 ≤ PM < 0.05
Density
0.0
0.2
0.4
0.6
0
1
2
3
4
obs
Gaussian kernel with band width 1.06

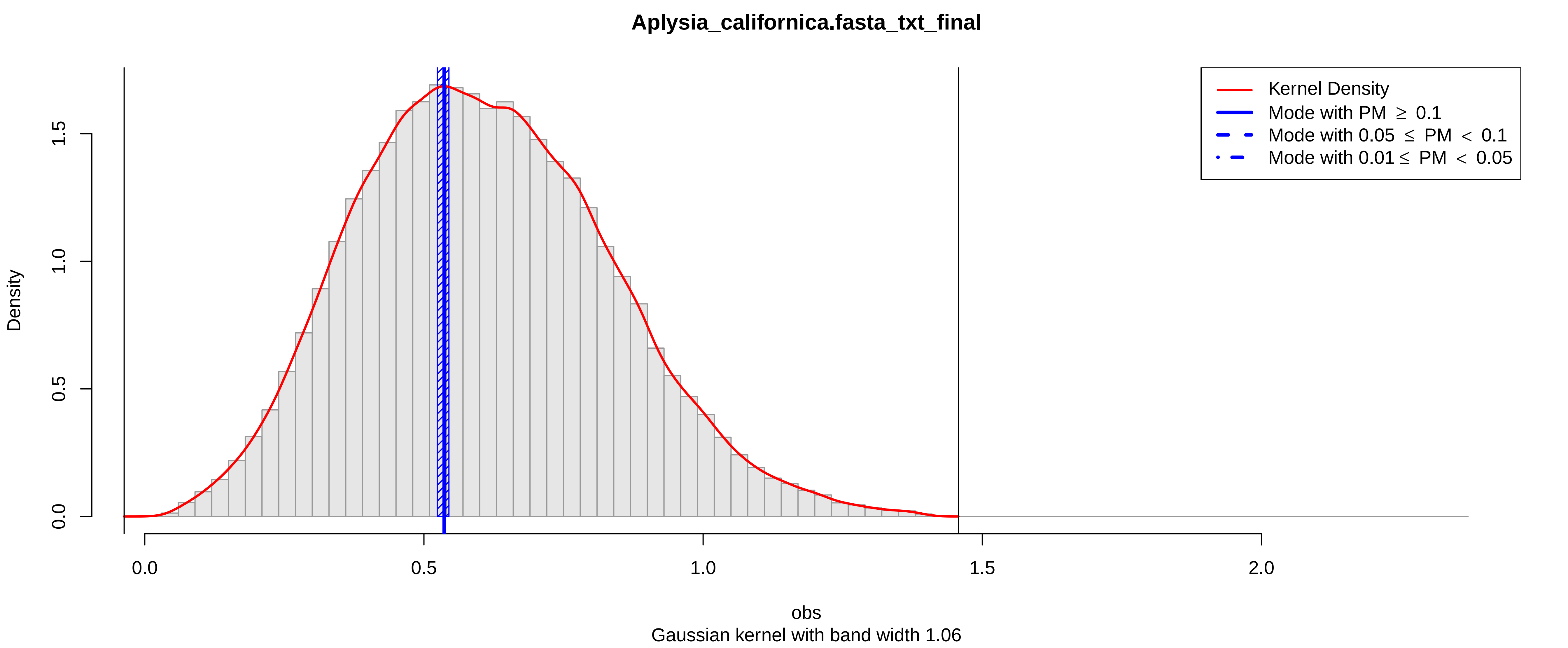

Aplysia_californica.fasta_txt_final
Kernel Density
Mode with PM ≥ 0.1
Mode with 0.05 ≤ PM < 0.1
Mode with 0.01 ≤ PM < 0.05
Density
0.0
0.5
1.0
1.5
0.0
0.5
1.0
1.5
2.0
obs
Gaussian kernel with band width 1.06

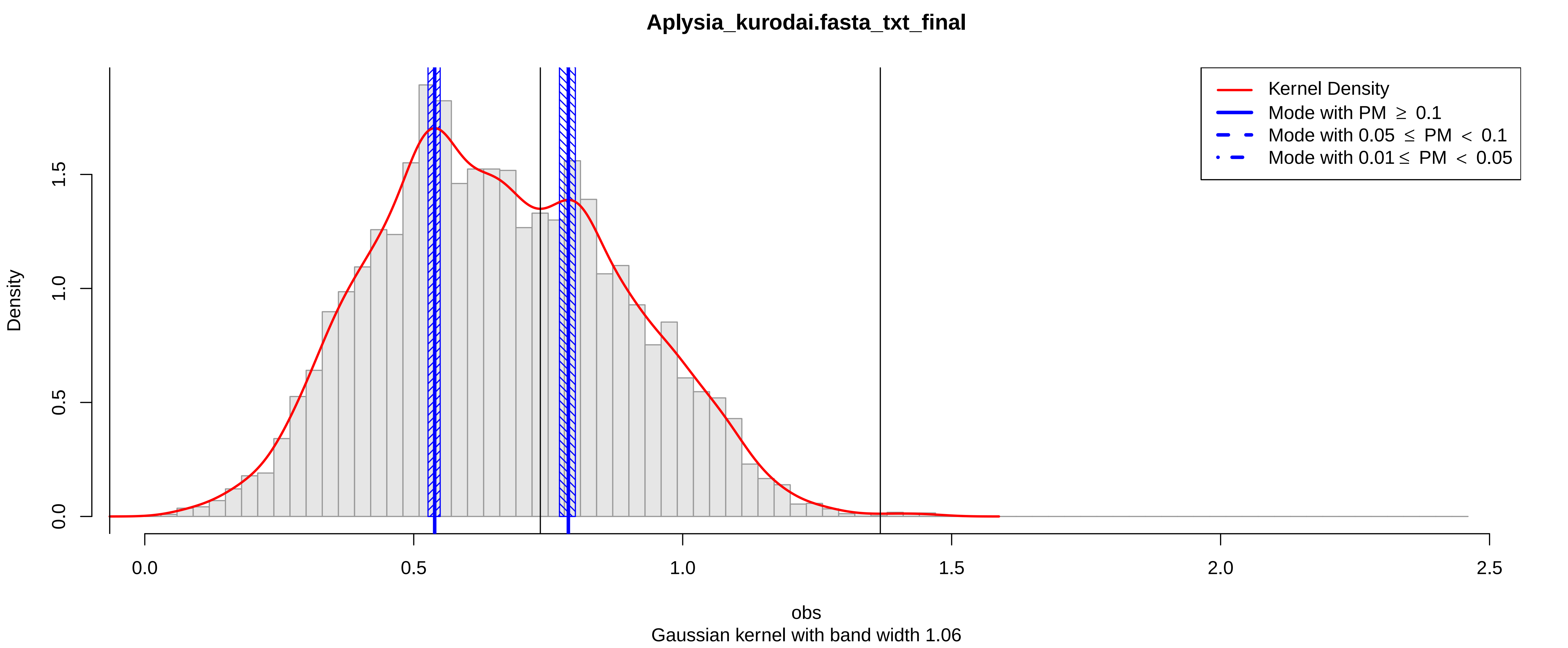
Aplysia_kurodai.fasta_txt_final
Kernel Density
Mode with PM ≥ 0.1
Mode with 0.05 ≤ PM < 0.1
Mode with 0.01 ≤ PM < 0.05
Density
0.0
0.5
1.0
1.5
0.0
0.5
1.0
1.5
2.0
2.5
obs
Gaussian kernel with band width 1.06

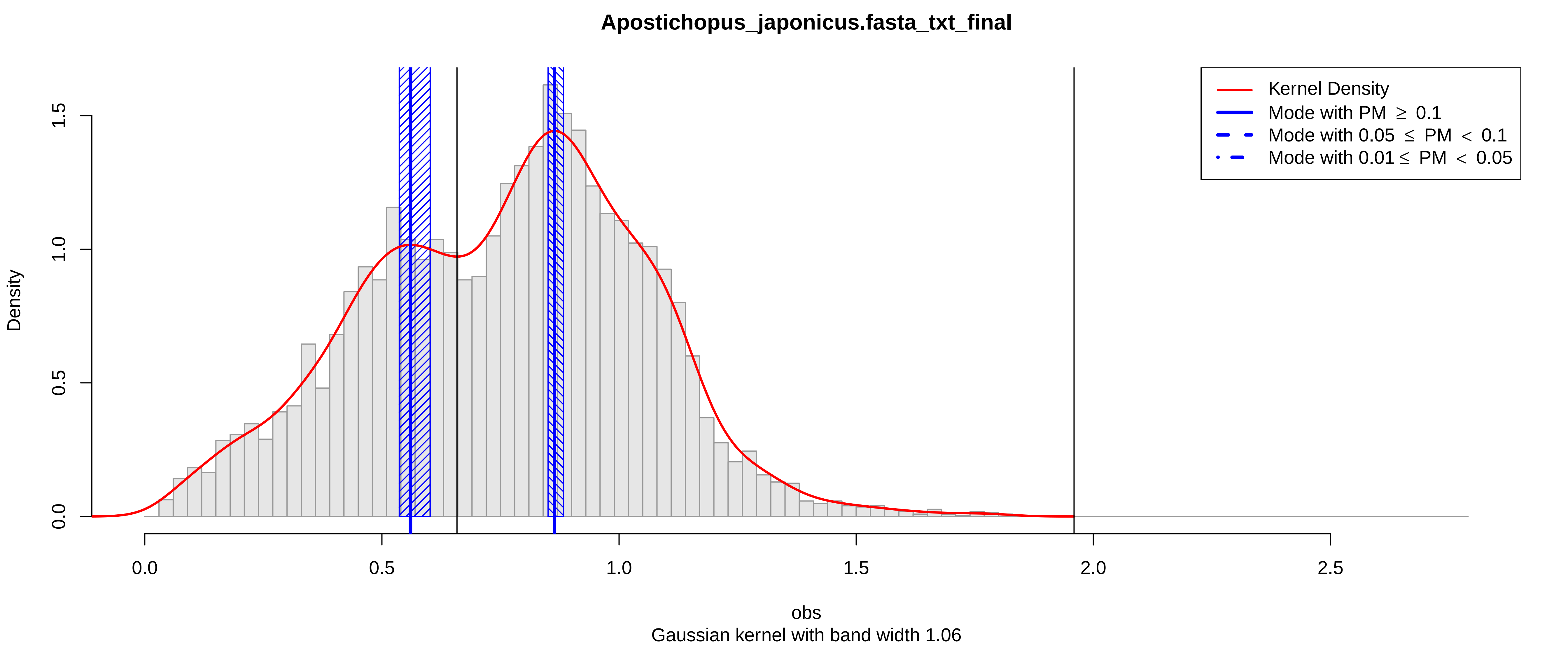
Apostichopus_japonicus.fasta_txt_final
Kernel Density
Mode with PM ≥ 0.1
Mode with 0.05 ≤ PM < 0.1
Mode with 0.01 ≤ PM < 0.05
Density
0.0
0.5
1.0
1.5
0.0
0.5
1.0
1.5
2.0
2.5
obs
Gaussian kernel with band width 1.06

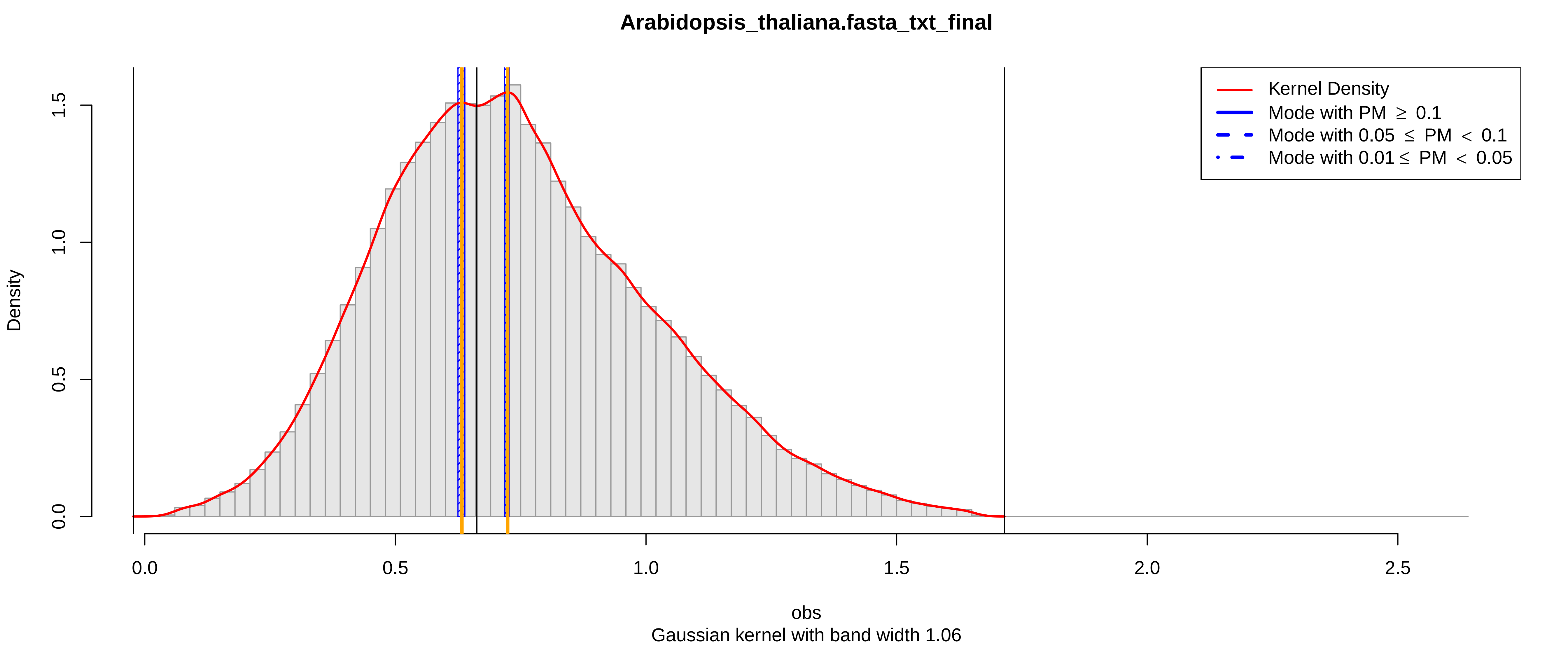
Arabidopsis_thaliana.fasta_txt_final
Kernel Density
Mode with PM ≥ 0.1
Mode with 0.05 ≤ PM < 0.1
Mode with 0.01 ≤ PM < 0.05
Density
0.0
0.5
1.0
1.5
0.0
0.5
1.0
1.5
2.0
2.5
obs
Gaussian kernel with band width 1.06

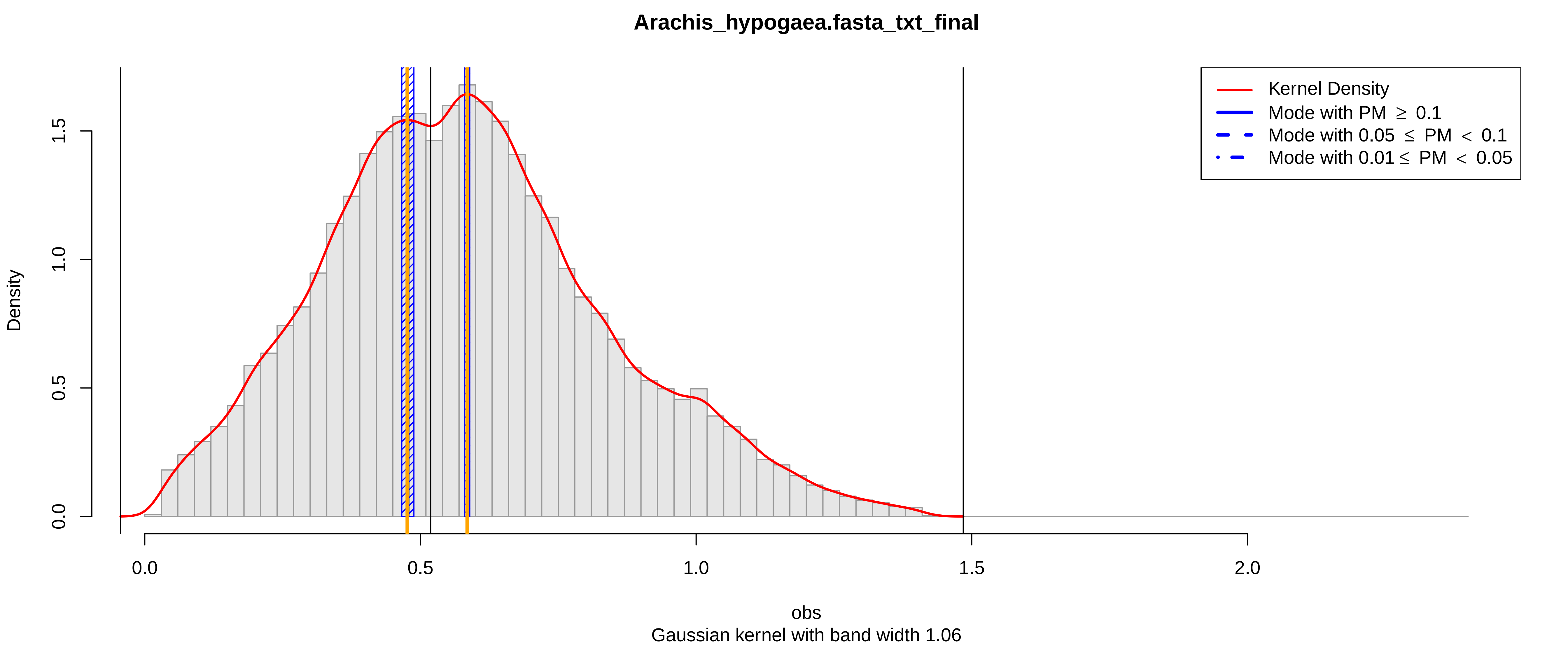
Arachis_hypogaea.fasta_txt_final
Kernel Density
Mode with PM ≥ 0.1
Mode with 0.05 ≤ PM < 0.1
Mode with 0.01 ≤ PM < 0.05
Density
0.0
0.5
1.0
1.5
0.0
0.5
1.0
1.5
2.0
obs
Gaussian kernel with band width 1.06

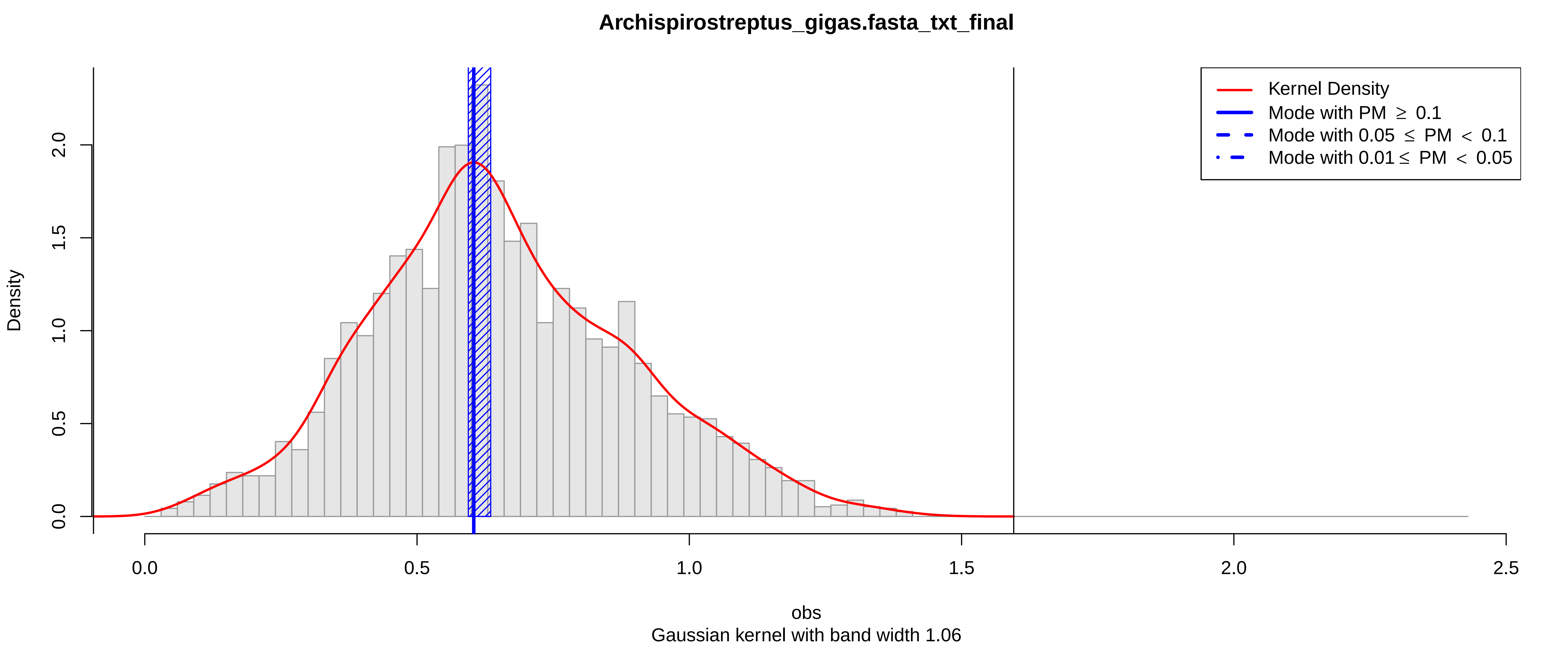

Archispirostreptus_gigas.fasta_txt_final
Kernel Density
Mode with PM ≥ 0.1
Mode with 0.05 ≤ PM < 0.1
Mode with 0.01 ≤ PM < 0.05
Density
0.0
0.5
1.0
1.5
2.0
0.0
0.5
1.0
1.5
2.0
2.5
obs
Gaussian kernel with band width 1.06

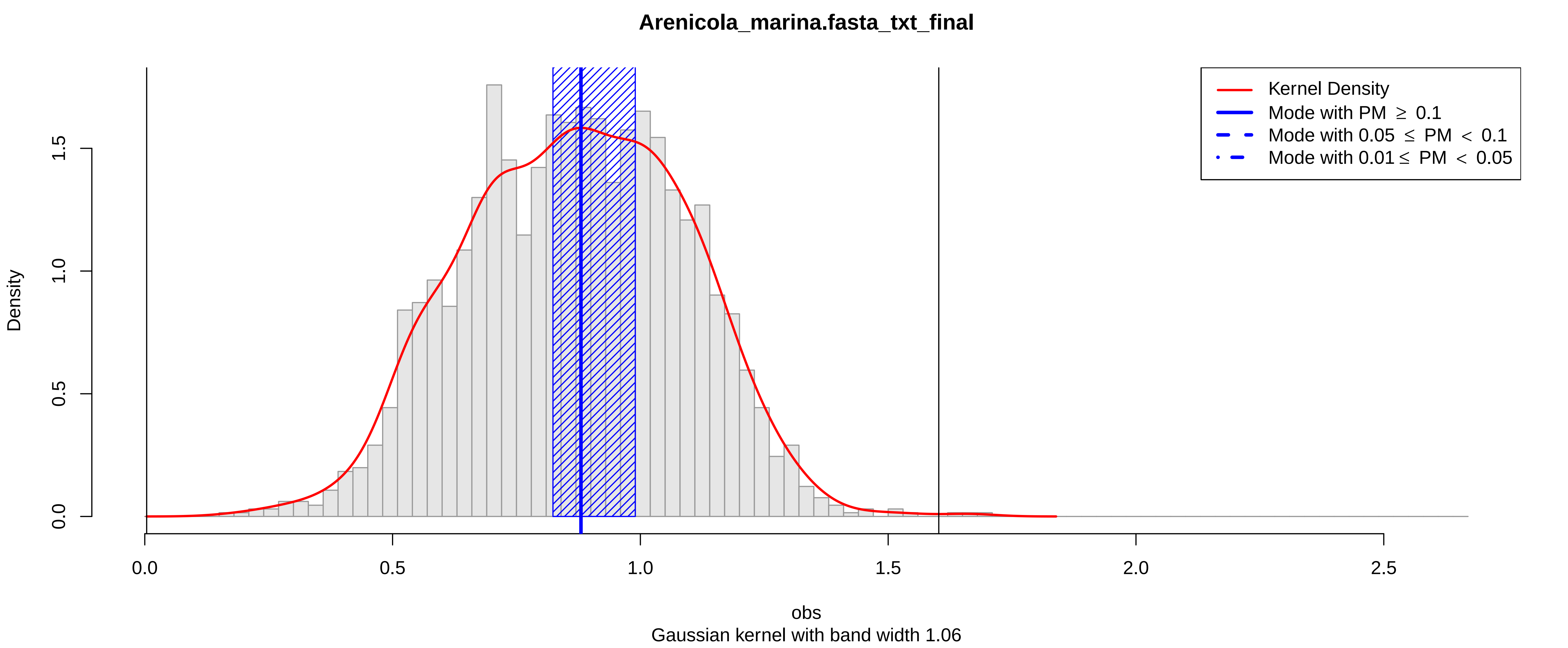

Arenicola_marina.fasta_txt_final
Kernel Density
Mode with PM ≥ 0.1
Mode with 0.05 ≤ PM < 0.1
Mode with 0.01 ≤ PM < 0.05
Density
0.0
0.5
1.0
1.5
2.0
2.5
obs
Gaussian kernel with band width 1.06

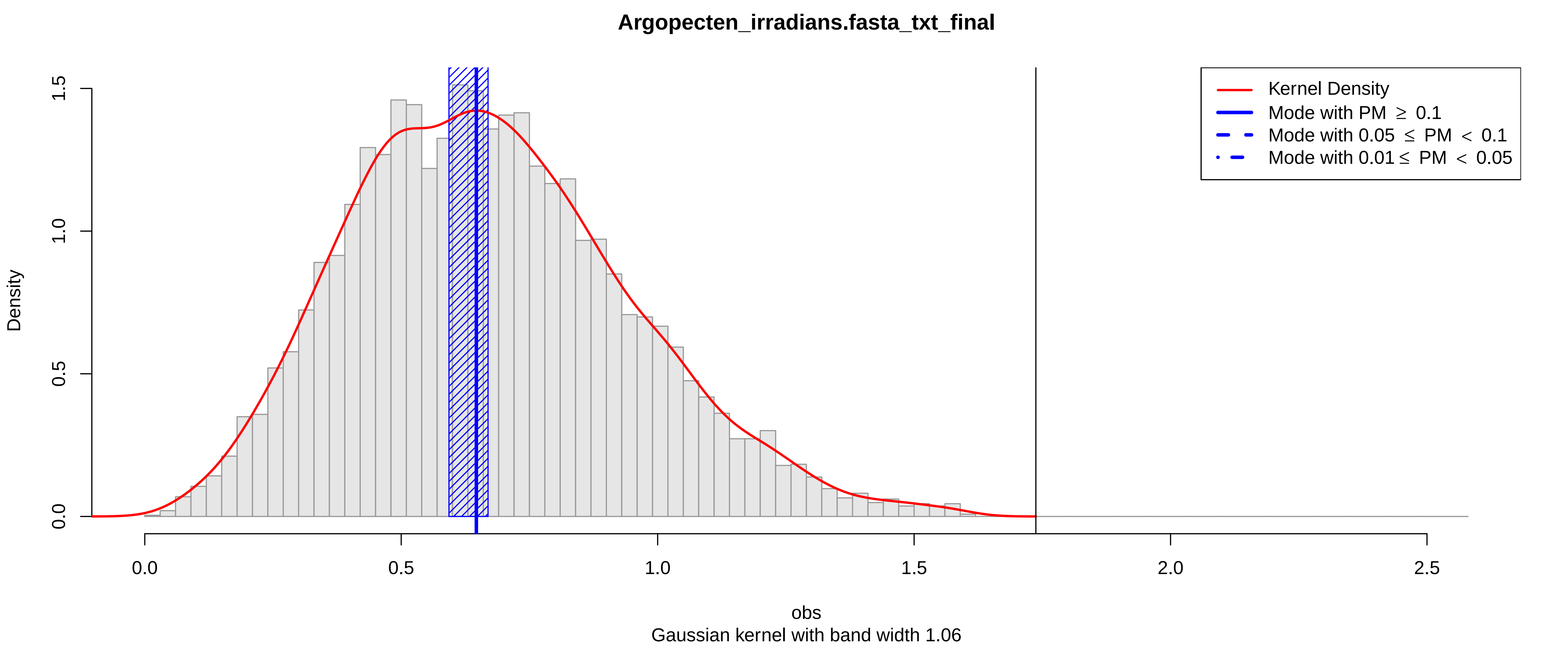

Argopecten_irradians.fasta_txt_final
Density
0.0
0.5
1.0
1.5
0.0
0.5
1.0
1.5
2.0
2.5
obs
Gaussian kernel with band width 1.06
Kernel Density
Mode with PM ≥ 0.1
Mode with 0.05 ≤ PM < 0.1
Mode with 0.01 ≤ PM < 0.05

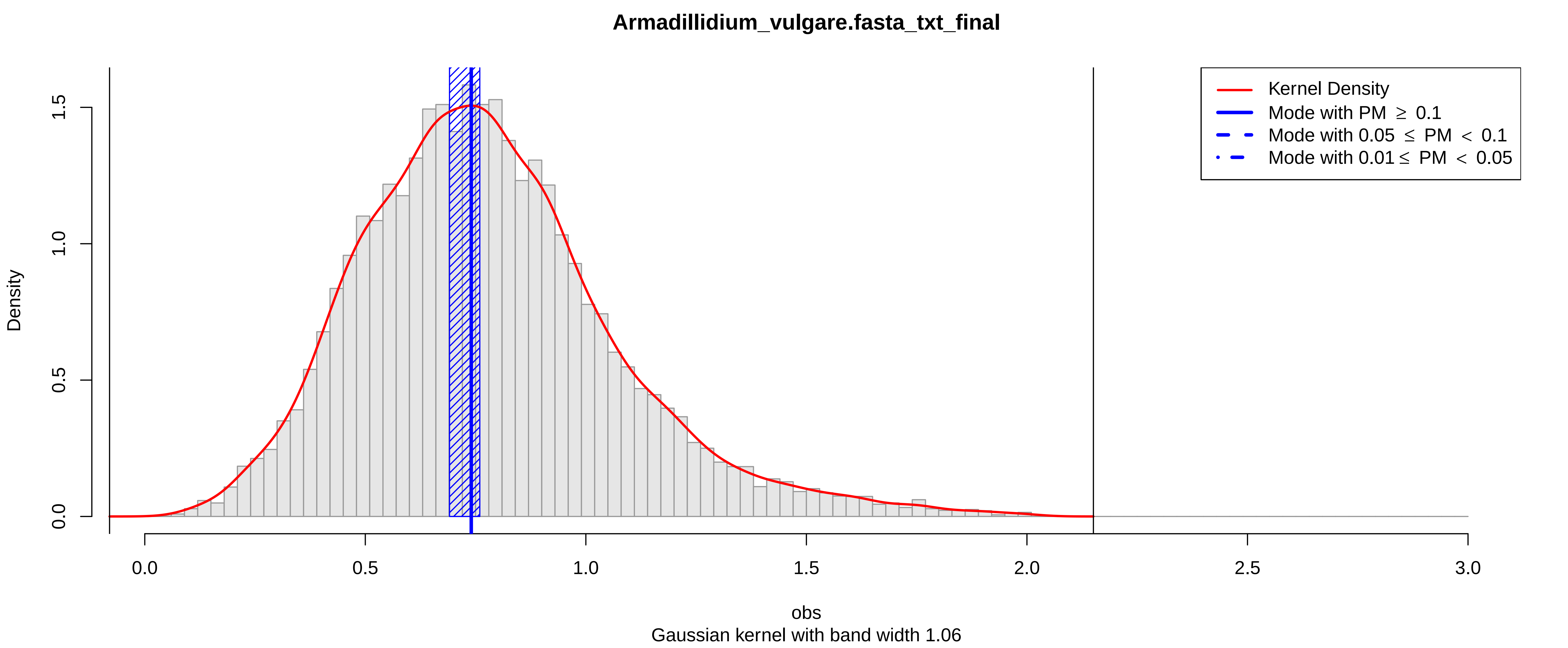
Armadillidium_vulgare.fasta_txt_final
Kernel Density
Mode with PM ≥ 0.1
Mode with 0.05 ≤ PM < 0.1
Mode with 0.01 ≤ PM < 0.05
Density
0.0
0.5
1.0
1.5
2.0
2.5
3.0
obs
Gaussian kernel with band width 1.06

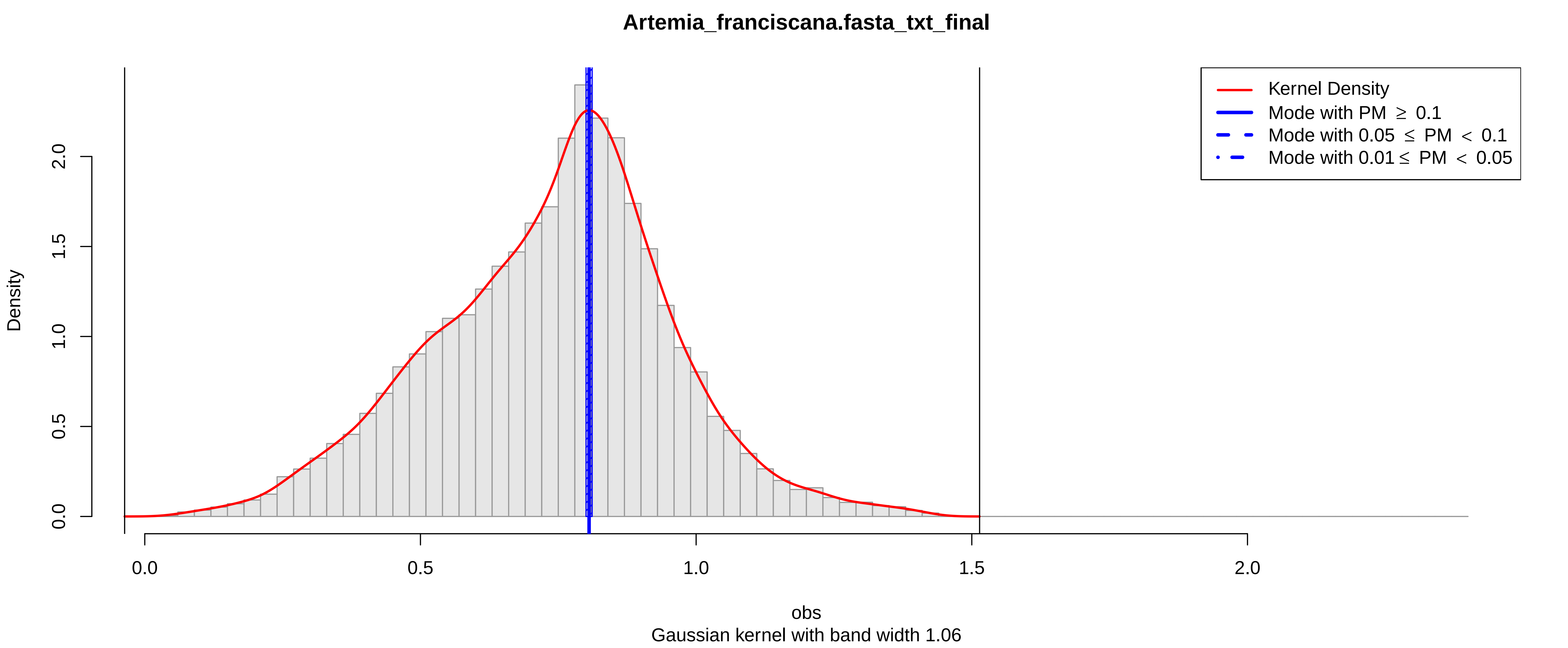

Artemia_franciscana.fasta_txt_final
Kernel Density
Mode with PM ≥ 0.1
Mode with 0.05 ≤ PM < 0.1
Mode with 0.01 ≤ PM < 0.05
Density
0.0
0.5
1.0
1.5
2.0
0.0
0.5
1.0
1.5
2.0
obs
Gaussian kernel with band width 1.06

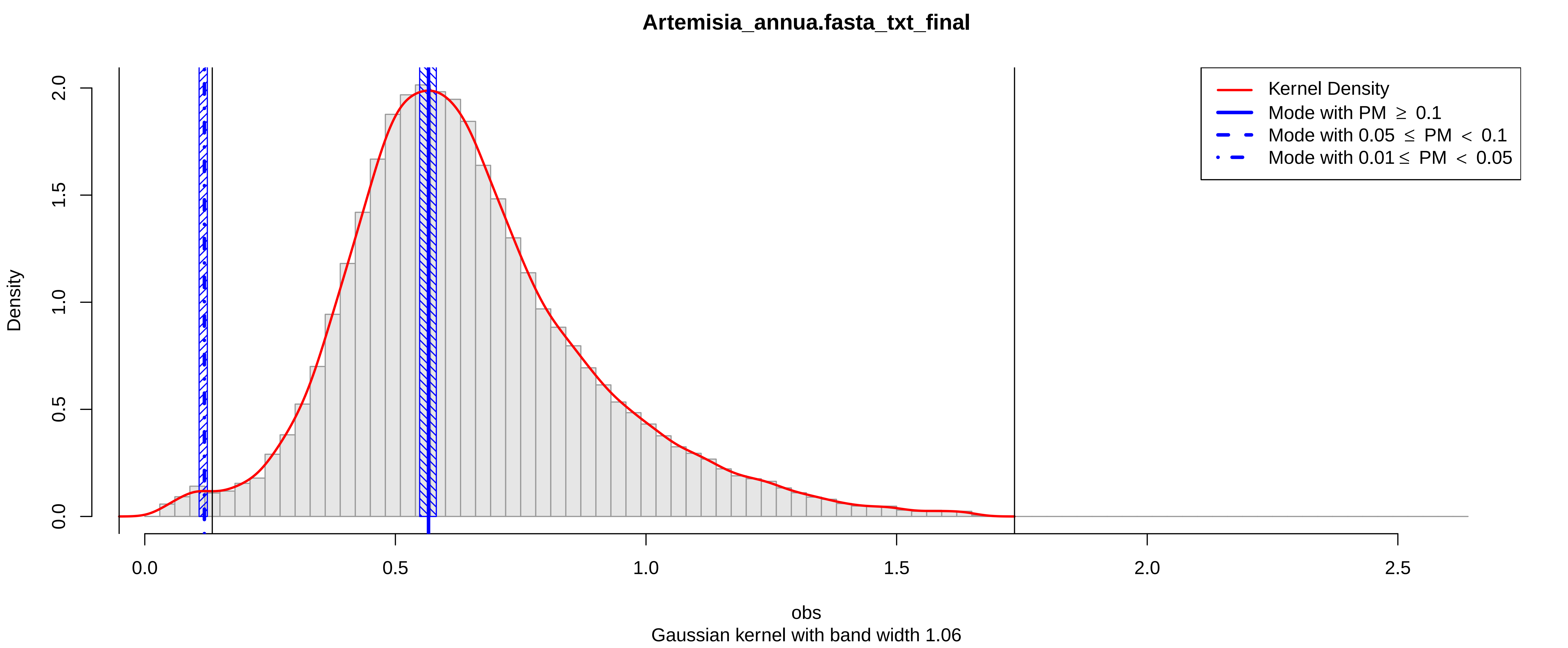

Artemisia_annua.fasta_txt_final
Kernel Density
Mode with PM ≥ 0.1
Mode with 0.05 ≤ PM < 0.1
Mode with 0.01 ≤ PM < 0.05
Density
0.0
0.5
1.0
1.5
2.0
2.5
obs
Gaussian kernel with band width 1.06

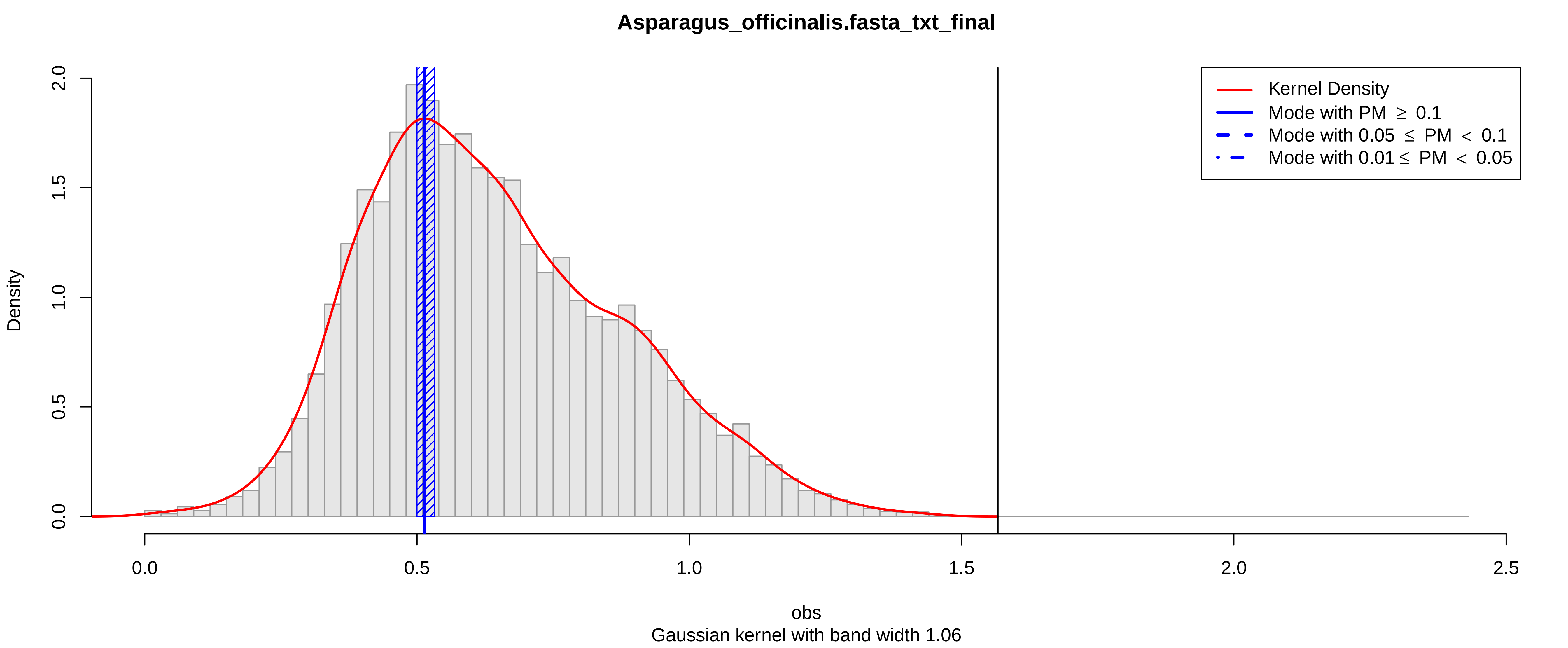

Asparagus_officinalis.fasta_txt_final
Density
0.0
0.5
1.0
1.5
2.0
0.0
0.5
1.0
1.5
2.0
2.5
obs
Gaussian kernel with band width 1.06
Kernel Density
Mode with PM ≥ 0.1
Mode with 0.05 ≤ PM < 0.1
Mode with 0.01 ≤ PM < 0.05

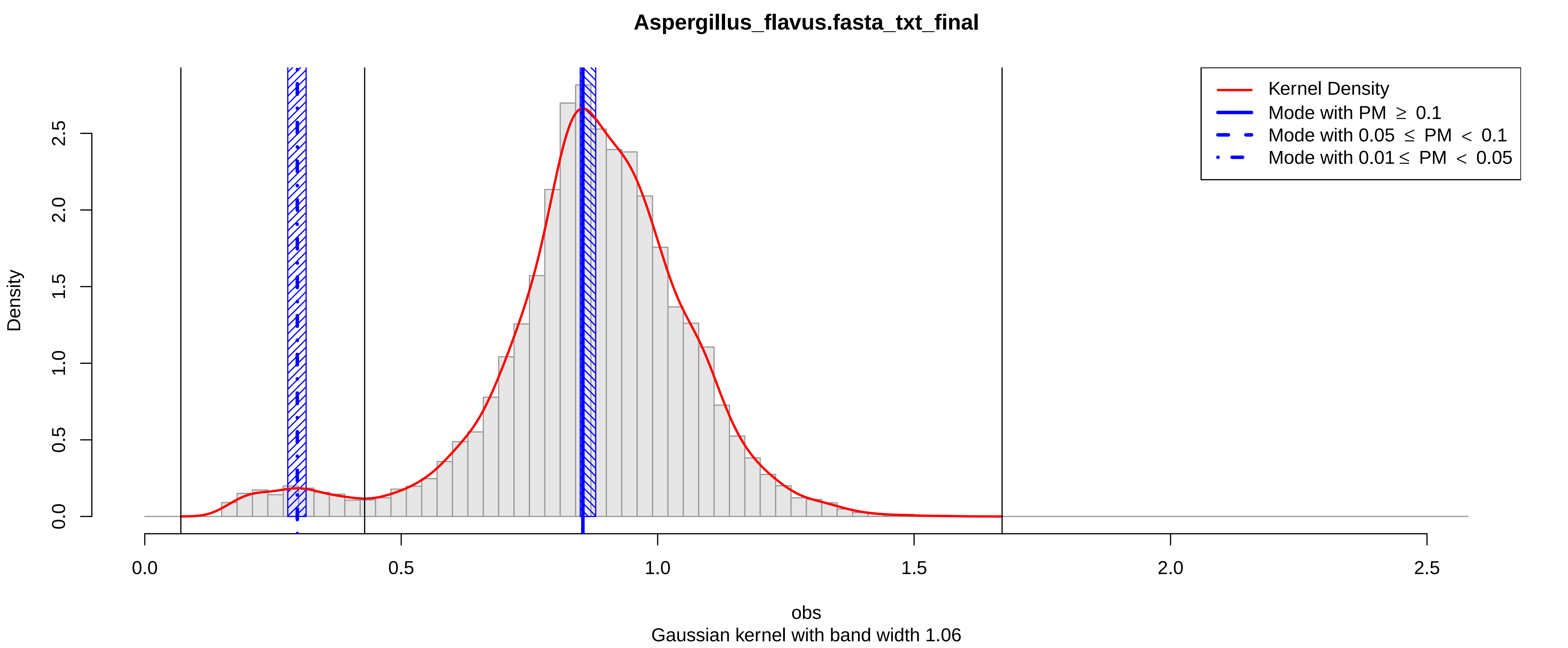
Aspergillus_flavus.fasta_txt_final
Kernel Density
Mode with PM ≥ 0.1
Mode with 0.05 ≤ PM < 0.1
Mode with 0.01 ≤ PM < 0.05
Density
0.0
0.5
1.0
1.5
2.0
2.5
obs
Gaussian kernel with band width 1.06

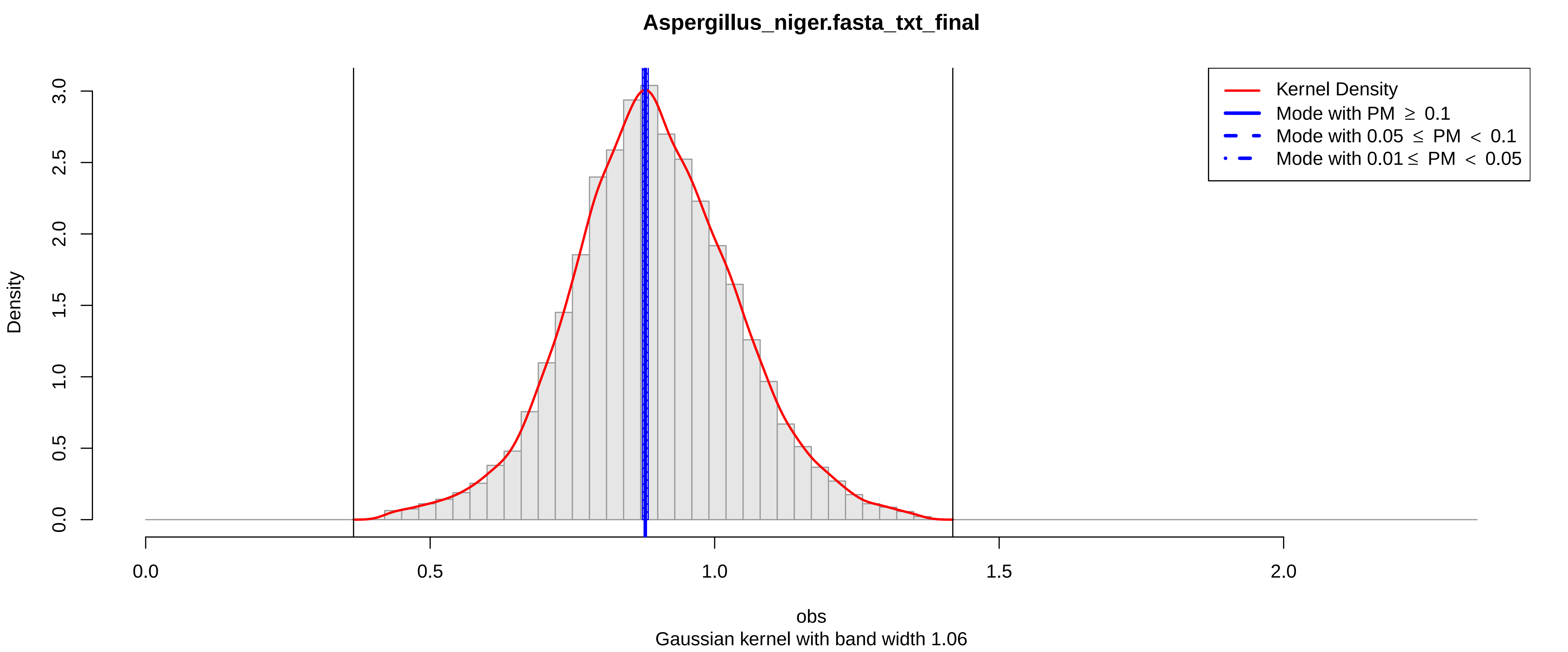
Aspergillus_niger.fasta_txt_final
Kernel Density
Mode with PM ≥ 0.1
Mode with 0.05 ≤ PM < 0.1
Mode with 0.01 ≤ PM < 0.05
Density
3.0
2.5
2.0
1.5
1.0
0.5
0.0
0.0
0.5
1.0
1.5
2.0
obs
Gaussian kernel with band width 1.06

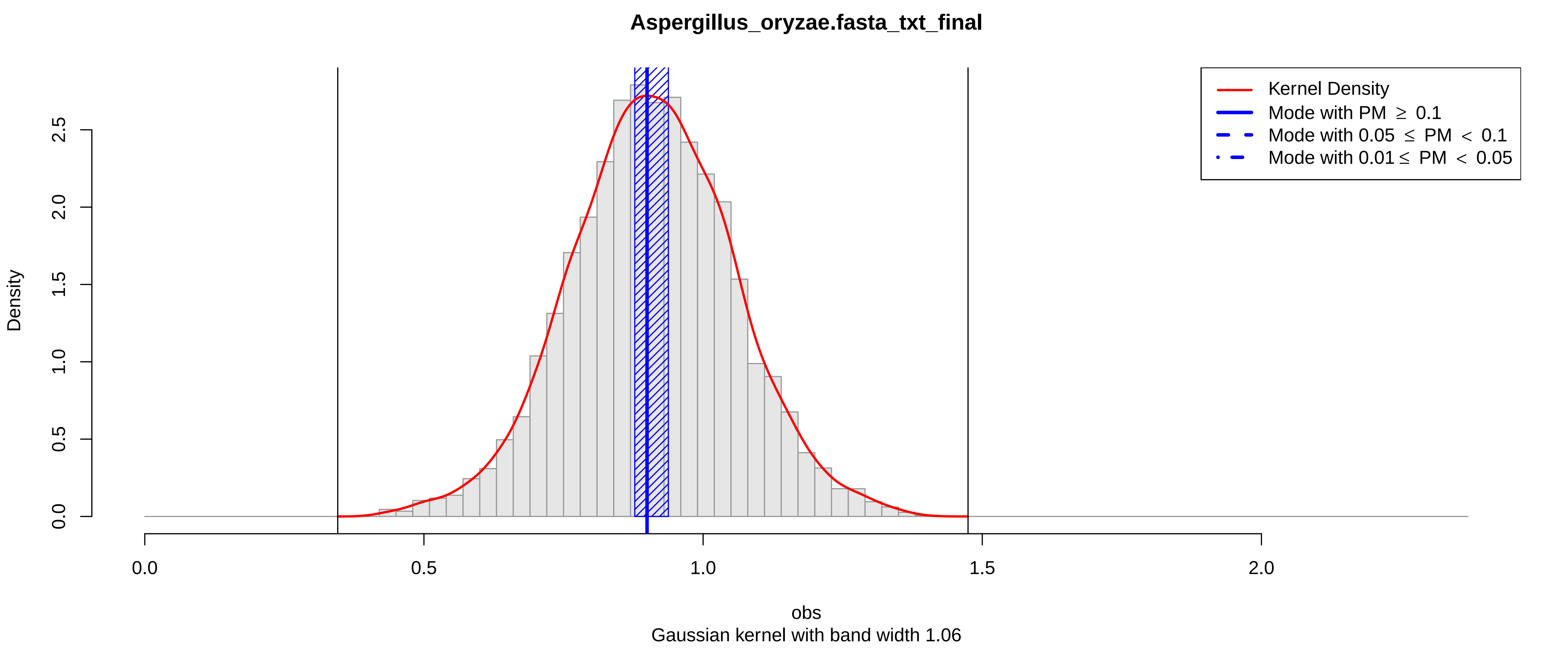

Aspergillus_oryzae.fasta_txt_final
Kernel Density
Mode with PM ≥ 0.1
Mode with 0.05 ≤ PM < 0.1
Mode with 0.01 ≤ PM < 0.05
Density
0.0
0.5
1.0
1.5
2.0
2.5
obs
Gaussian kernel with band width 1.06

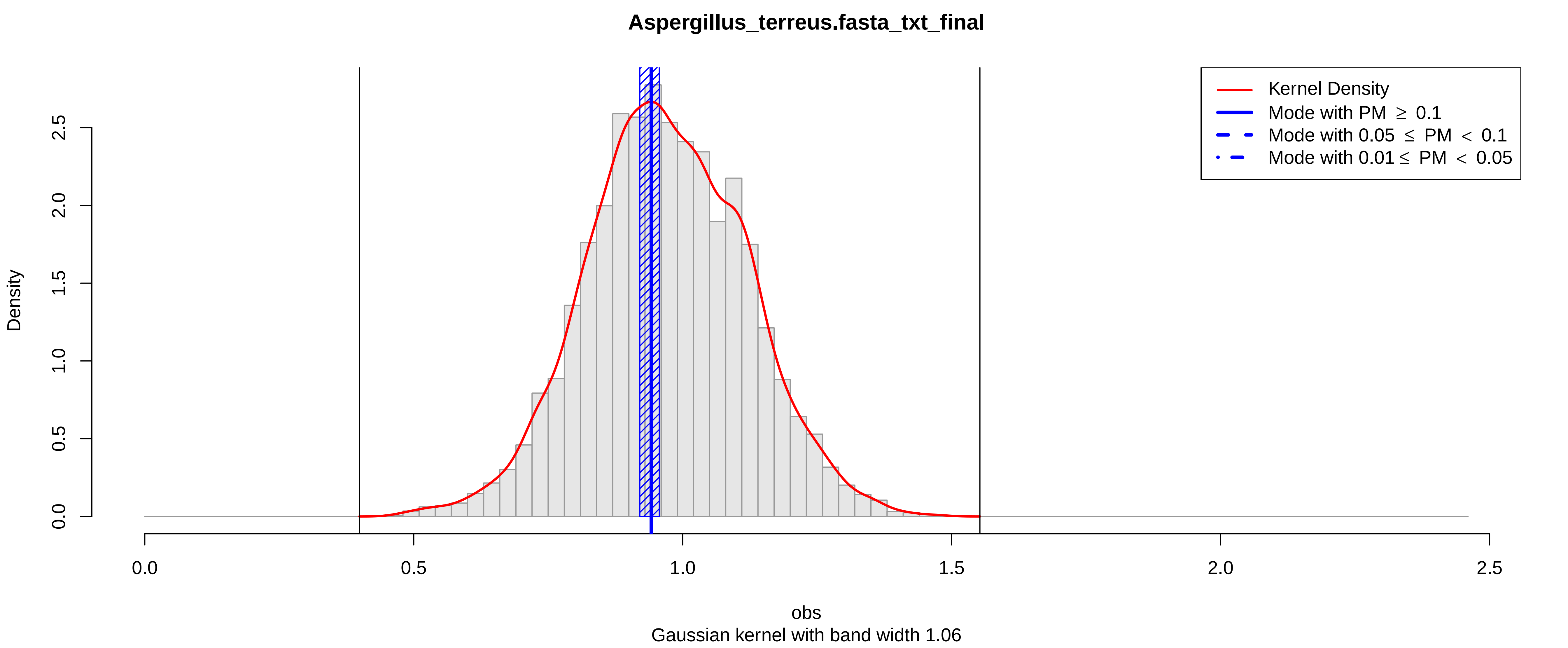

Aspergillus_terreus.fasta_txt_final
Kernel Density
Mode with PM ≥ 0.1
Mode with 0.05 ≤ PM < 0.1
Mode with 0.01 ≤ PM < 0.05
Density
0.0
0.5
1.0
1.5
2.0
2.5
obs
Gaussian kernel with band width 1.06

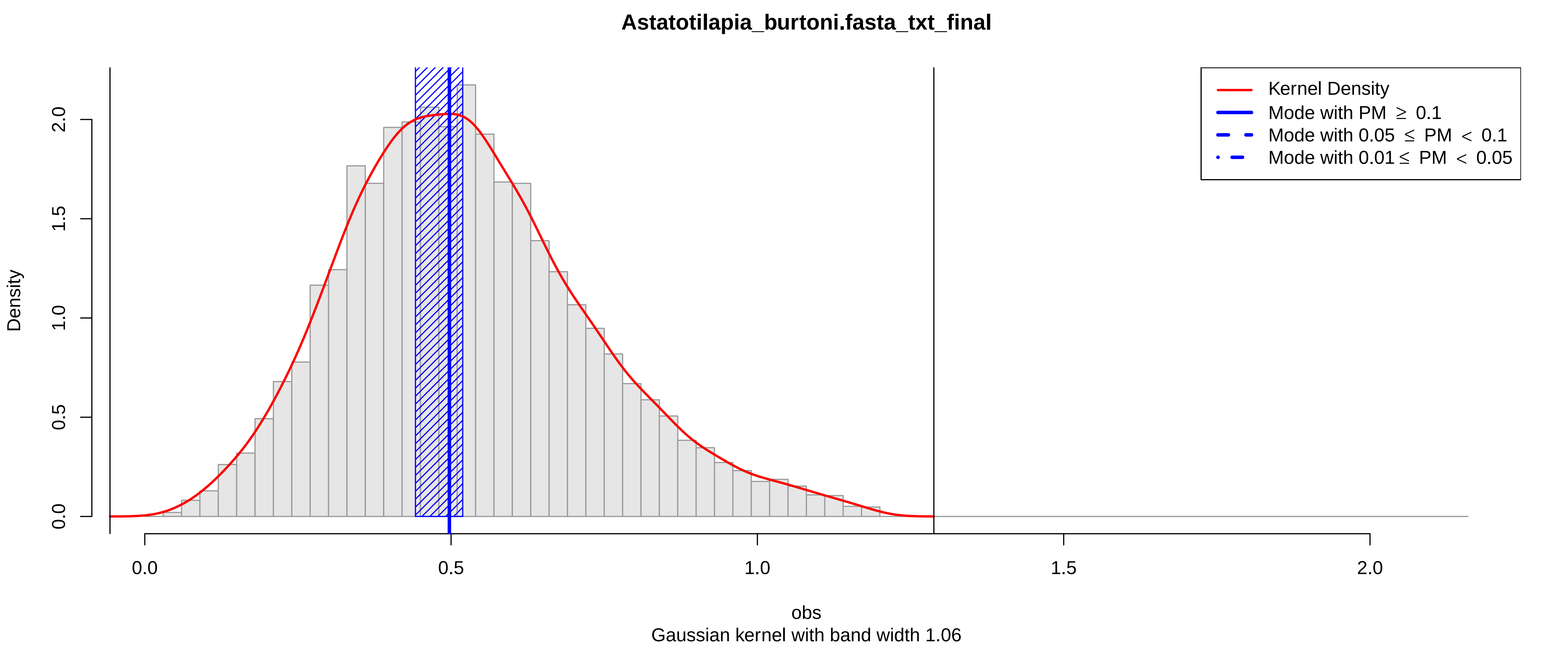
Astatotilapia_burtoni.fasta_txt_final
Kernel Density
Mode with PM ≥ 0.1
Mode with 0.05 ≤ PM < 0.1
Mode with 0.01 ≤ PM < 0.05
Density
0.0
0.5
1.0
1.5
2.0
0.0
0.5
1.0
1.5
2.0
obs
Gaussian kernel with band width 1.06

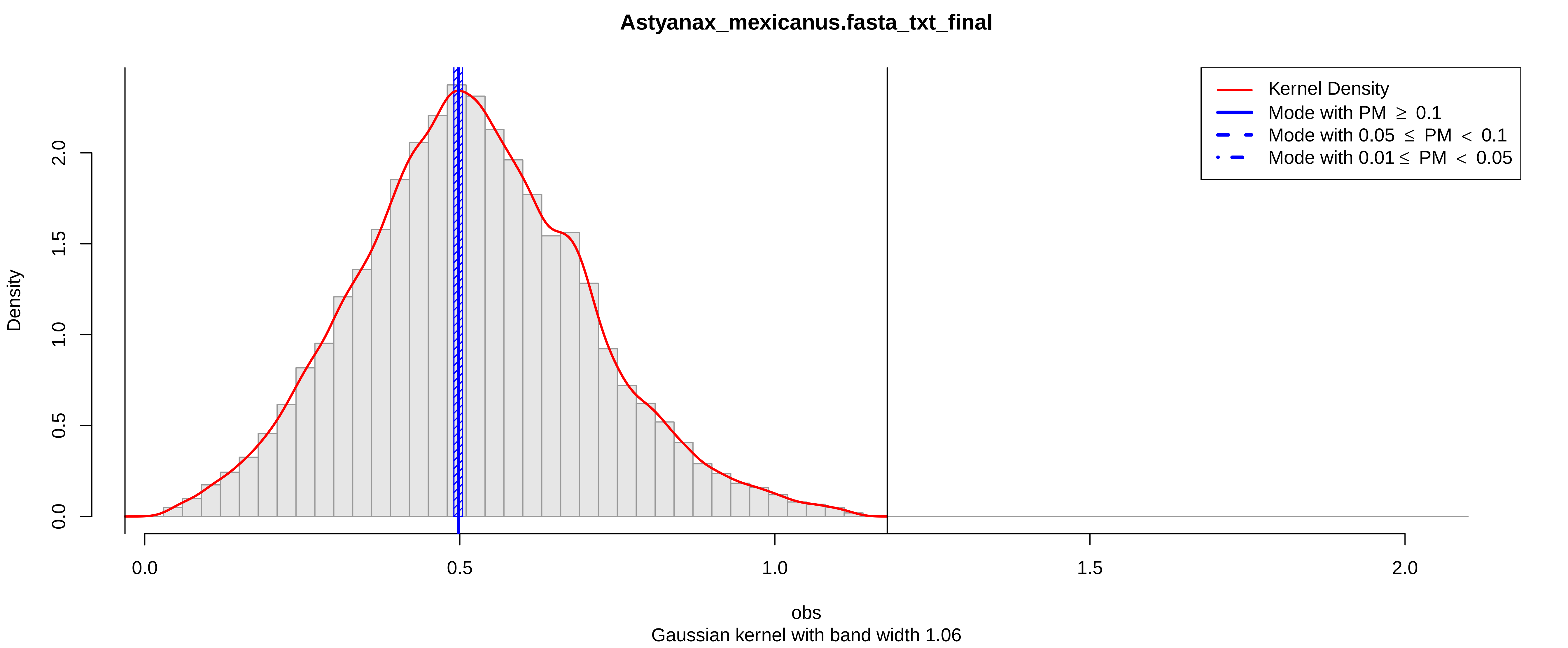

Astyanax_mexicanus.fasta_txt_final
Kernel Density
Mode with PM ≥ 0.1
Mode with 0.05 ≤ PM < 0.1
Mode with 0.01 ≤ PM < 0.05
Density
0.0
0.5
1.0
1.5
2.0
obs
Gaussian kernel with band width 1.06

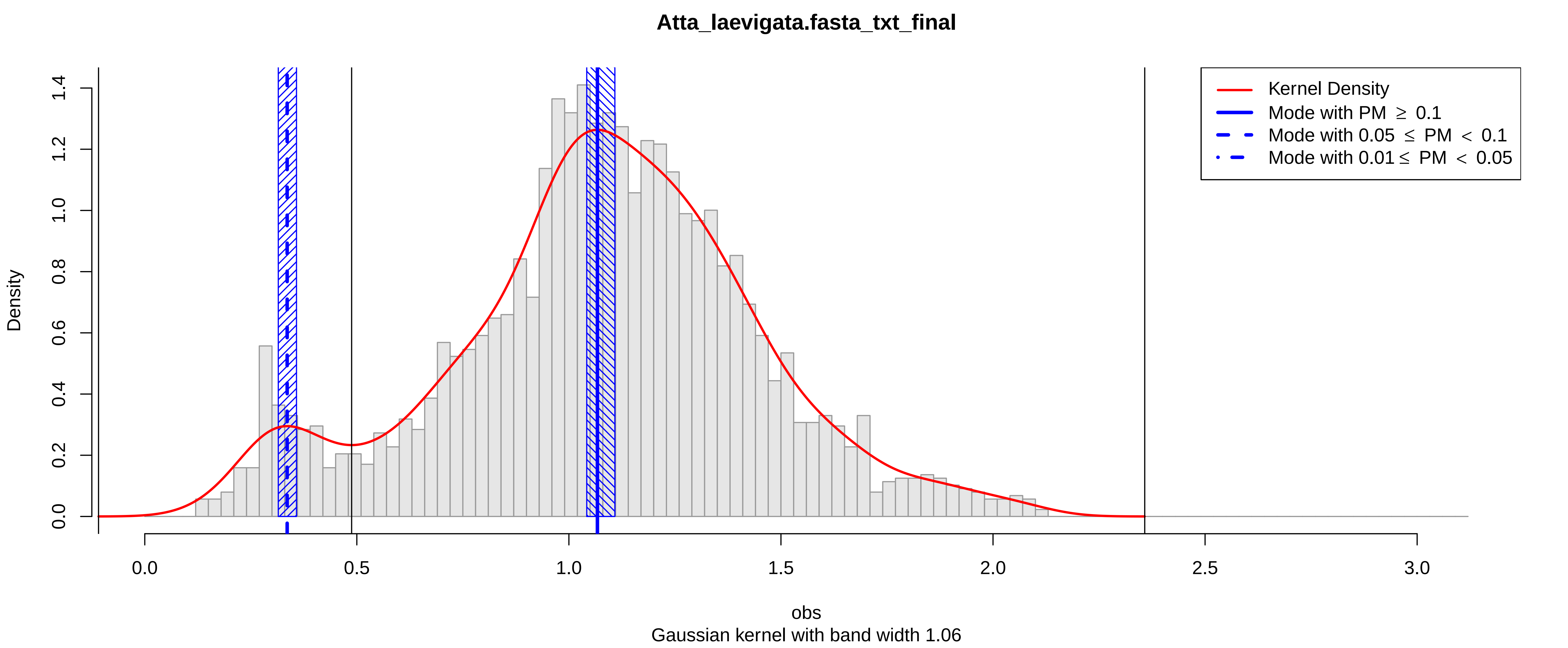

Atta_laevigata.fasta_txt_final
Kernel Density
Mode with PM ≥ 0.1
Mode with 0.05 ≤ PM < 0.1
Mode with 0.01 ≤ PM < 0.05
Density
0.0
0.2
0.4
0.6
0.8
1.0
1.2
1.4
0.0
0.5
1.0
1.5
2.0
2.5
3.0
obs
Gaussian kernel with band width 1.06

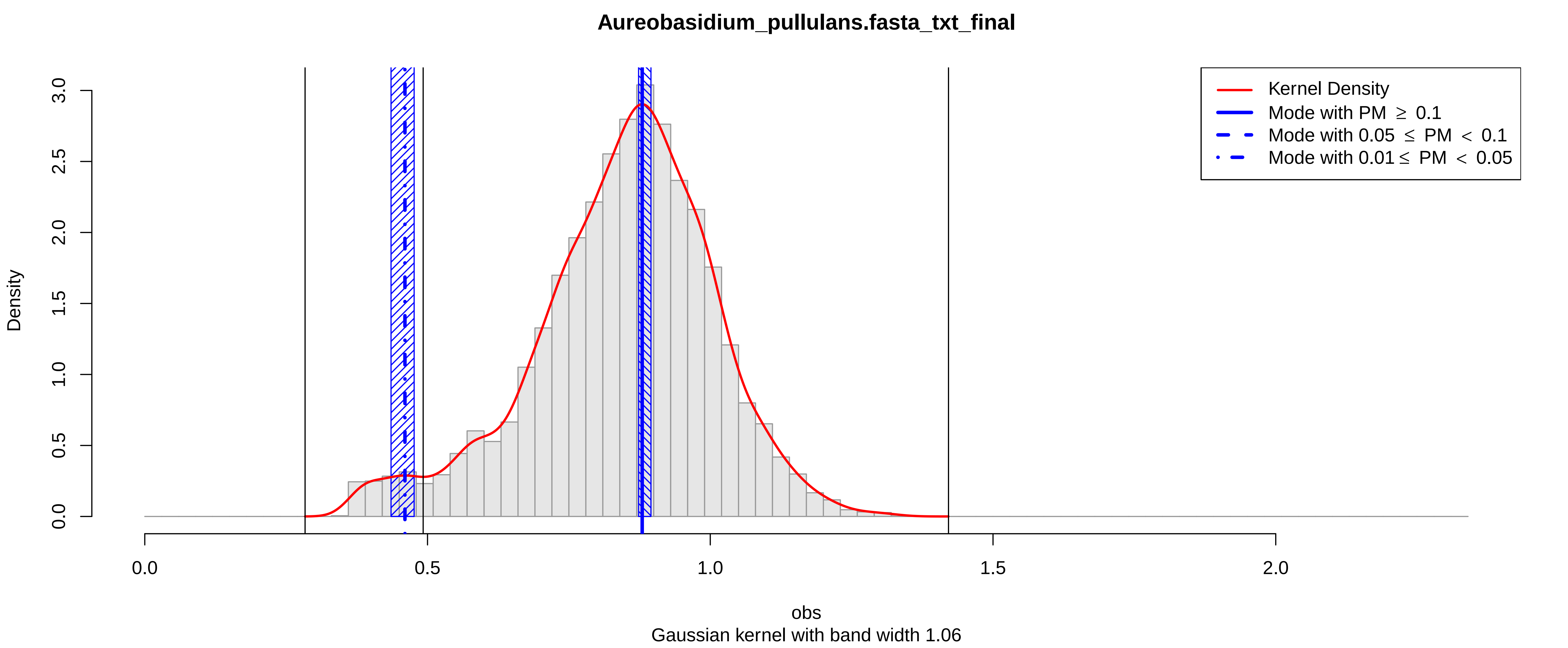
Aureobasidium_pullulans.fasta_txt_final
Kernel Density
Mode with PM ≥ 0.1
Mode with 0.05 ≤ PM < 0.1
Mode with 0.01 ≤ PM < 0.05
Density
0.0
0.5
1.0
1.5
2.0
2.5
3.0
0.0
0.5
1.0
1.5
2.0
obs
Gaussian kernel with band width 1.06

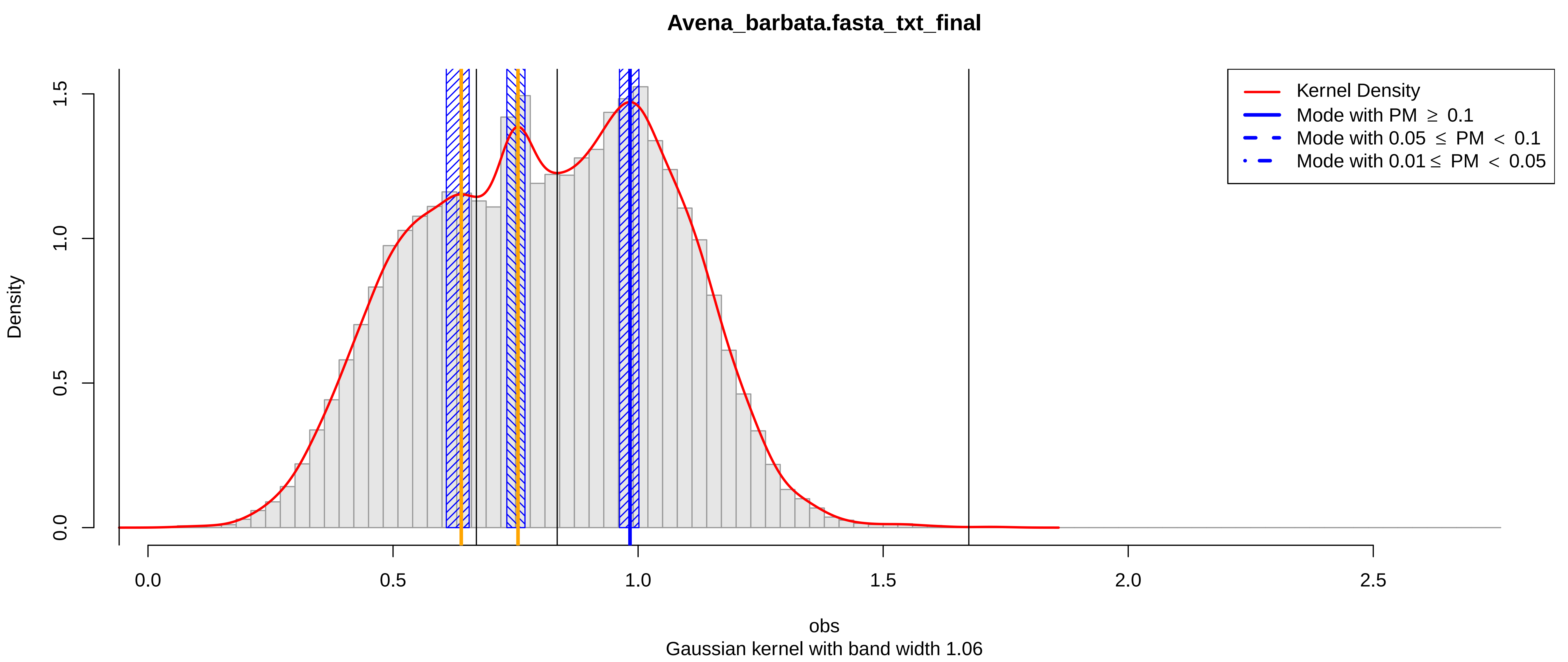

Avena_barbata.fasta_txt_final
Kernel Density
Mode with PM ≥ 0.1
Mode with 0.05 ≤ PM < 0.1
Mode with 0.01 ≤ PM < 0.05
Density
0.0
0.5
1.0
1.5
2.0
2.5
obs
Gaussian kernel with band width 1.06

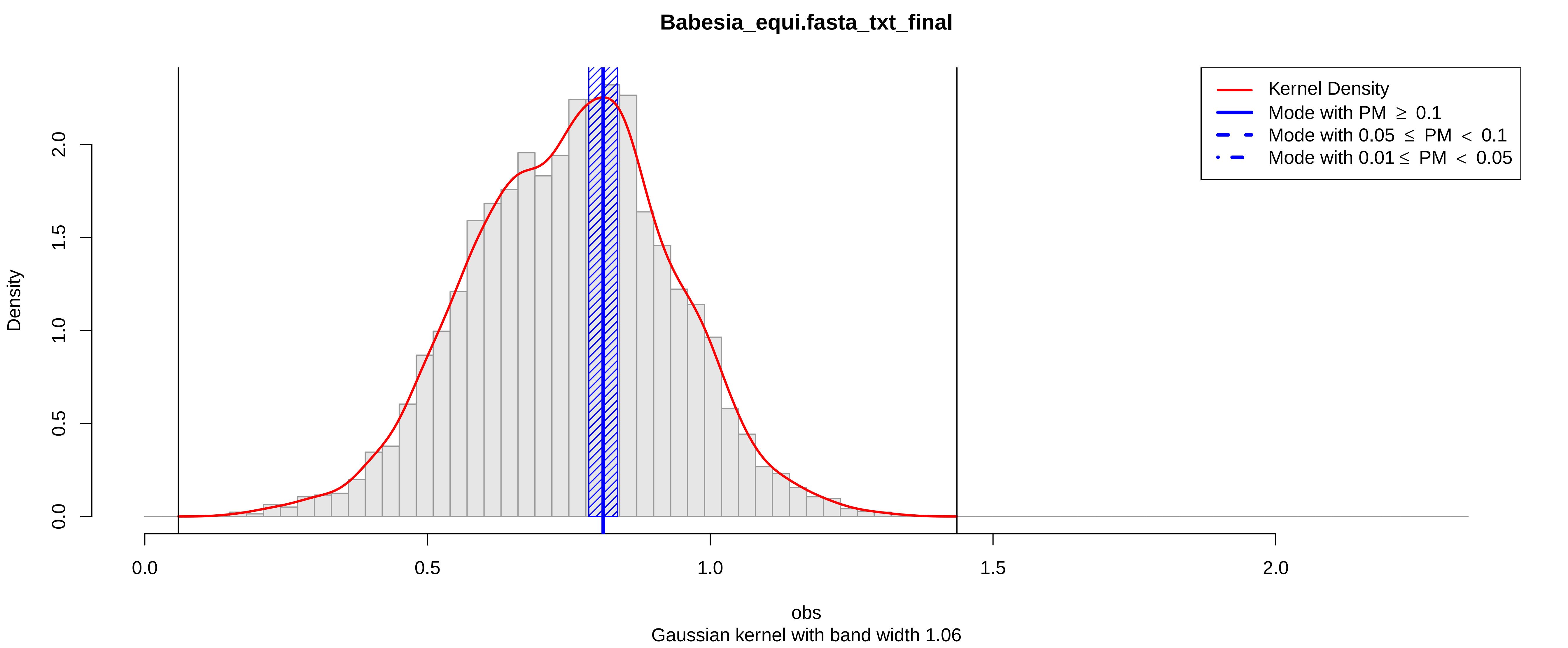
Babesia_equi.fasta_txt_final
Kernel Density
Mode with PM ≥ 0.1
Mode with 0.05 ≤ PM < 0.1
Mode with 0.01 ≤ PM < 0.05
Density
0.0
0.5
1.0
1.5
2.0
0.0
0.5
1.0
1.5
2.0
obs
Gaussian kernel with band width 1.06

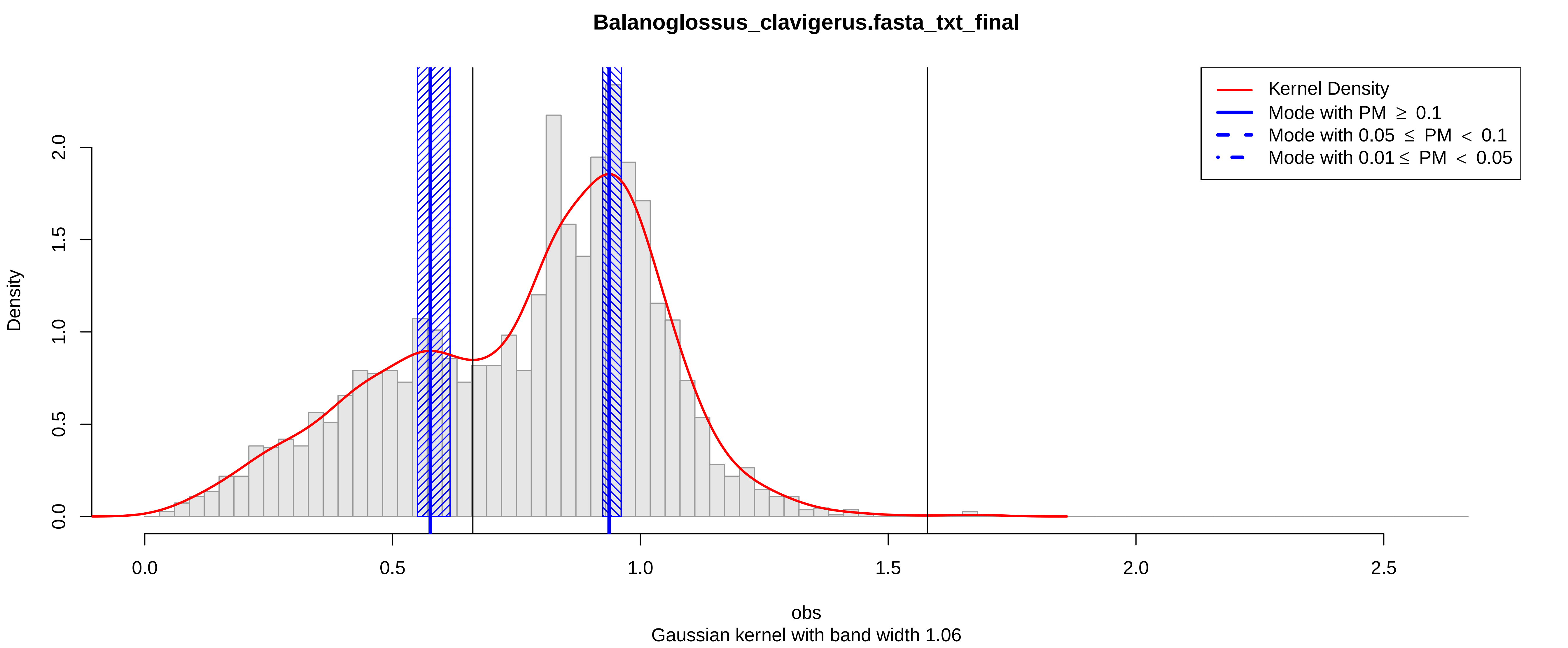

Balanoglossus_clavigerus.fasta_txt_final
Kernel Density
Mode with PM ≥ 0.1
Mode with 0.05 ≤ PM < 0.1
Mode with 0.01 ≤ PM < 0.05
Density
0.0
0.5
1.0
1.5
2.0
2.5
obs
Gaussian kernel with band width 1.06

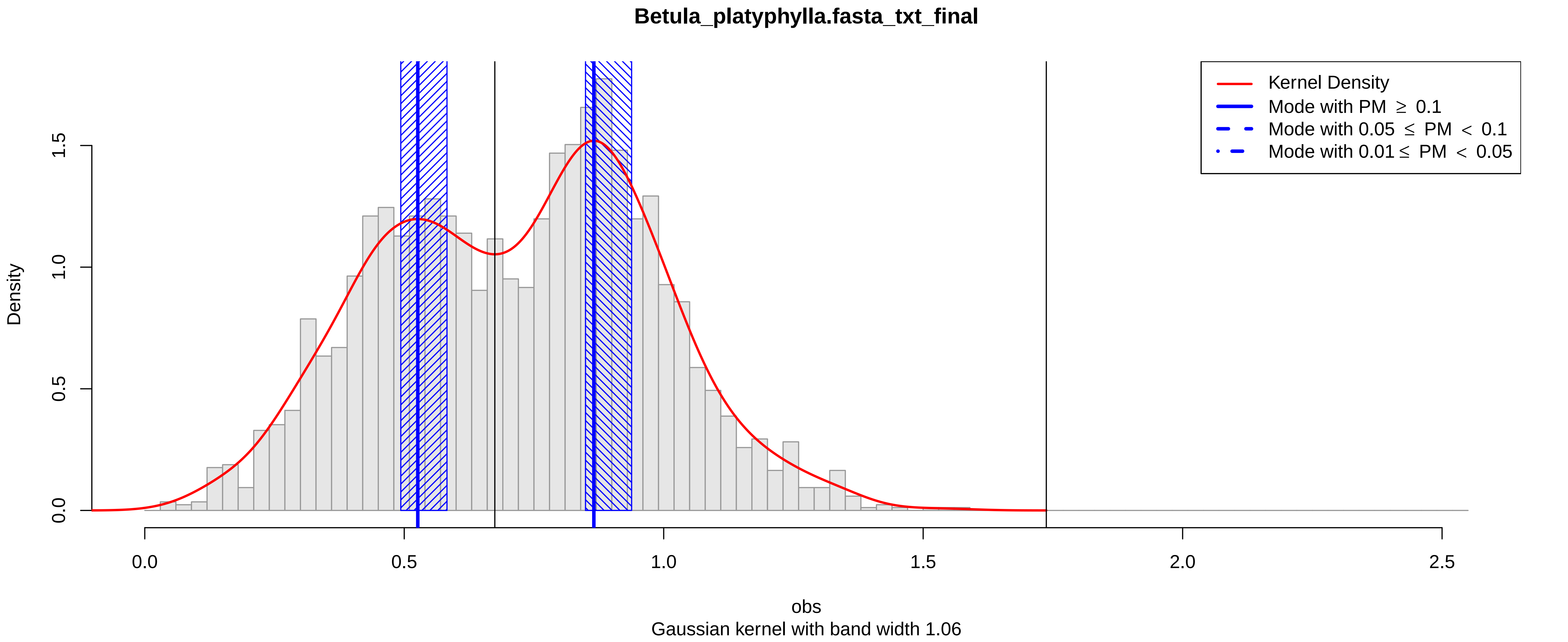

Betula_platyphylla.fasta_txt_final
Kernel Density
Mode with PM ≥ 0.1
Mode with 0.05 ≤ PM < 0.1
Mode with 0.01 ≤ PM < 0.05
Density
0.0
0.5
1.0
1.5
2.0
2.5
obs
Gaussian kernel with band width 1.06

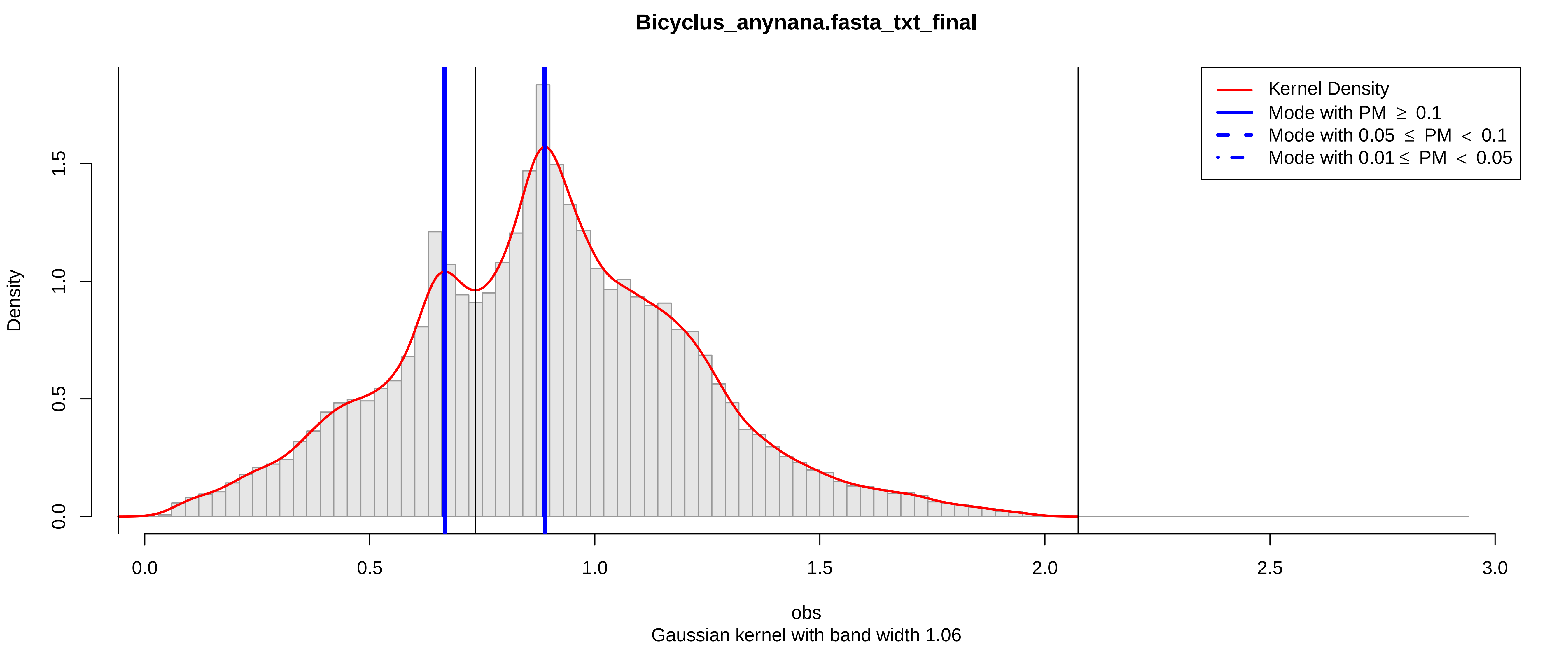
Bicyclus_anynana.fasta_txt_final
Kernel Density
Mode with PM ≥ 0.1
Mode with 0.05 ≤ PM < 0.1
Mode with 0.01 ≤ PM < 0.05
Density
0.0
0.5
1.0
1.5
0.0
0.5
1.0
1.5
2.0
2.5
3.0
obs
Gaussian kernel with band width 1.06

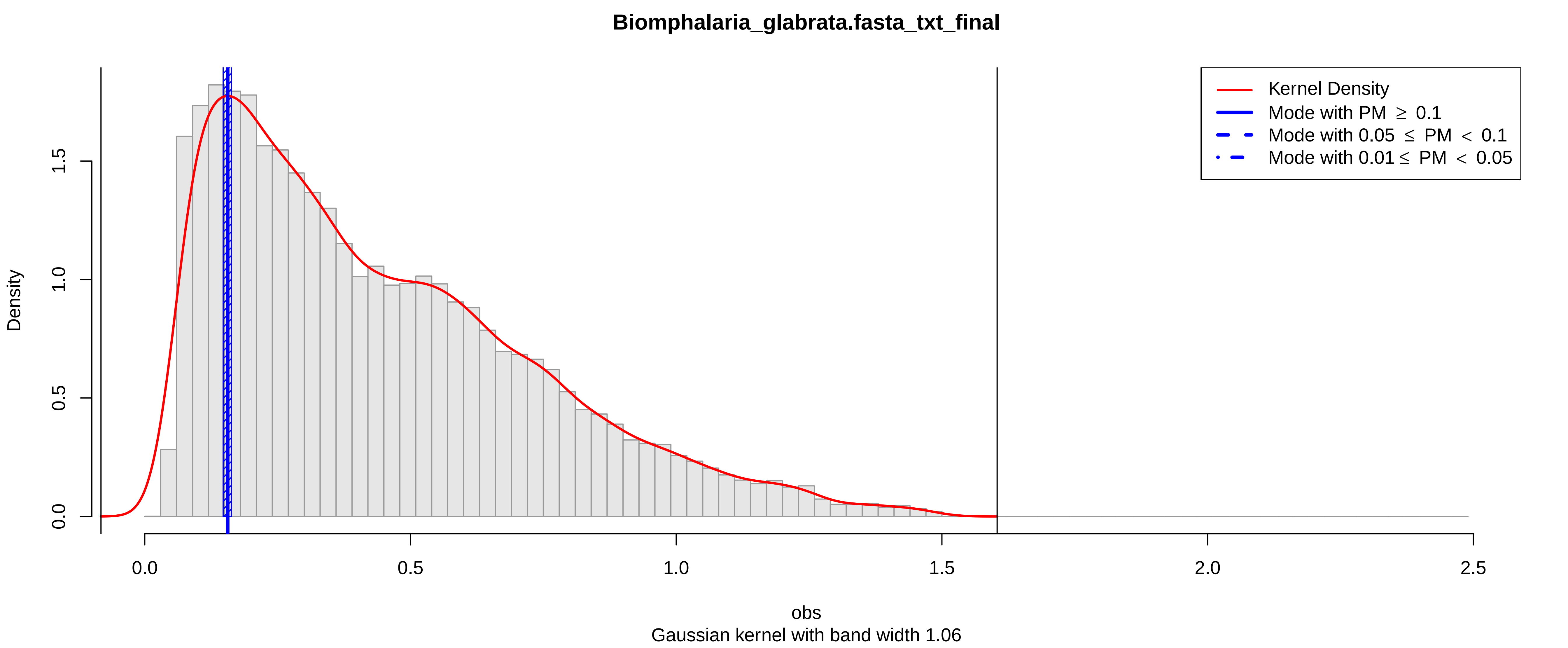
Biomphalaria_glabrata.fasta_txt_final
Density
0.0
0.5
1.0
1.5
0.0
0.5
1.0
1.5
2.0
2.5
obs
Gaussian kernel with band width 1.06
Kernel Density
Mode with PM ≥ 0.1
Mode with 0.05 ≤ PM < 0.1
Mode with 0.01 ≤ PM < 0.05

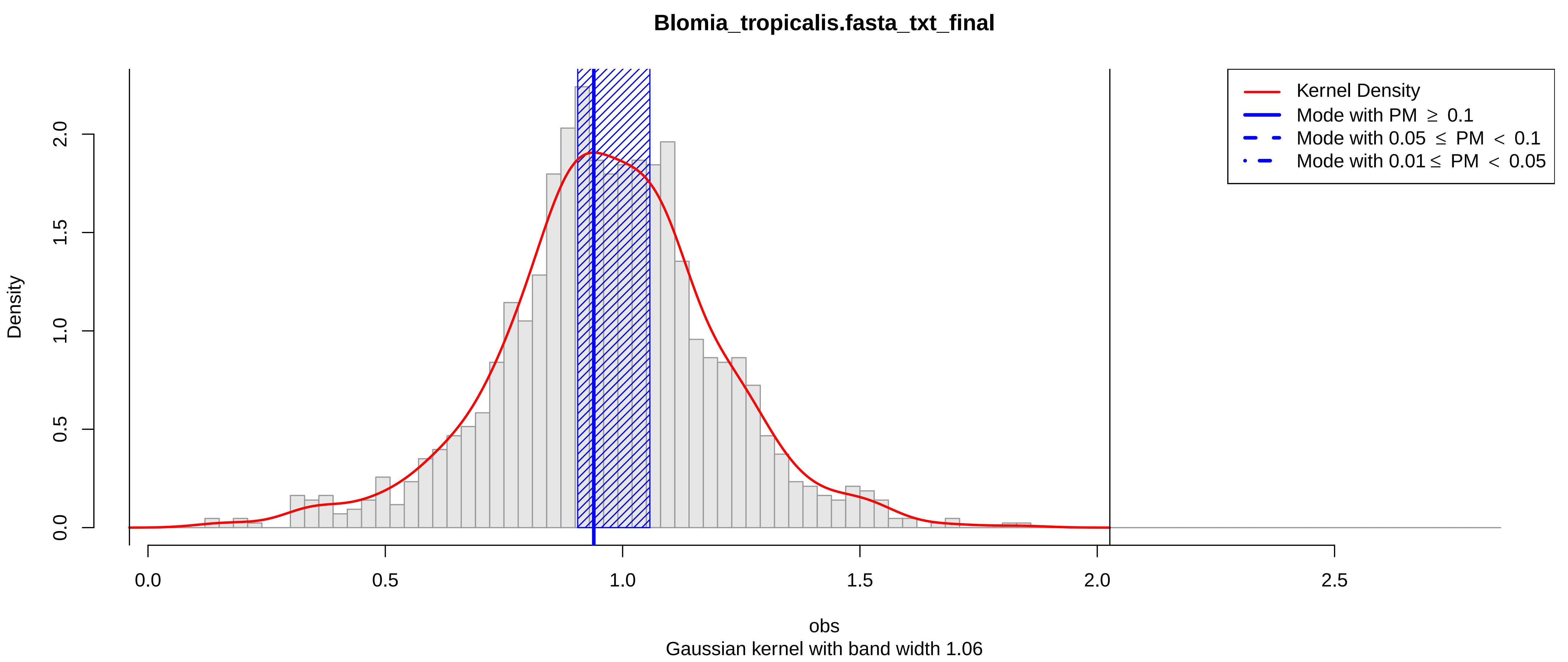
Blomia_tropicalis.fasta_txt_final
Density
0.0
0.5
1.0
1.5
2.0
2.5
obs
Gaussian kernel with band width 1.06
Kernel Density
Mode with PM ≥ 0.1
Mode with 0.05 ≤ PM < 0.1
Mode with 0.01 ≤ PM < 0.05

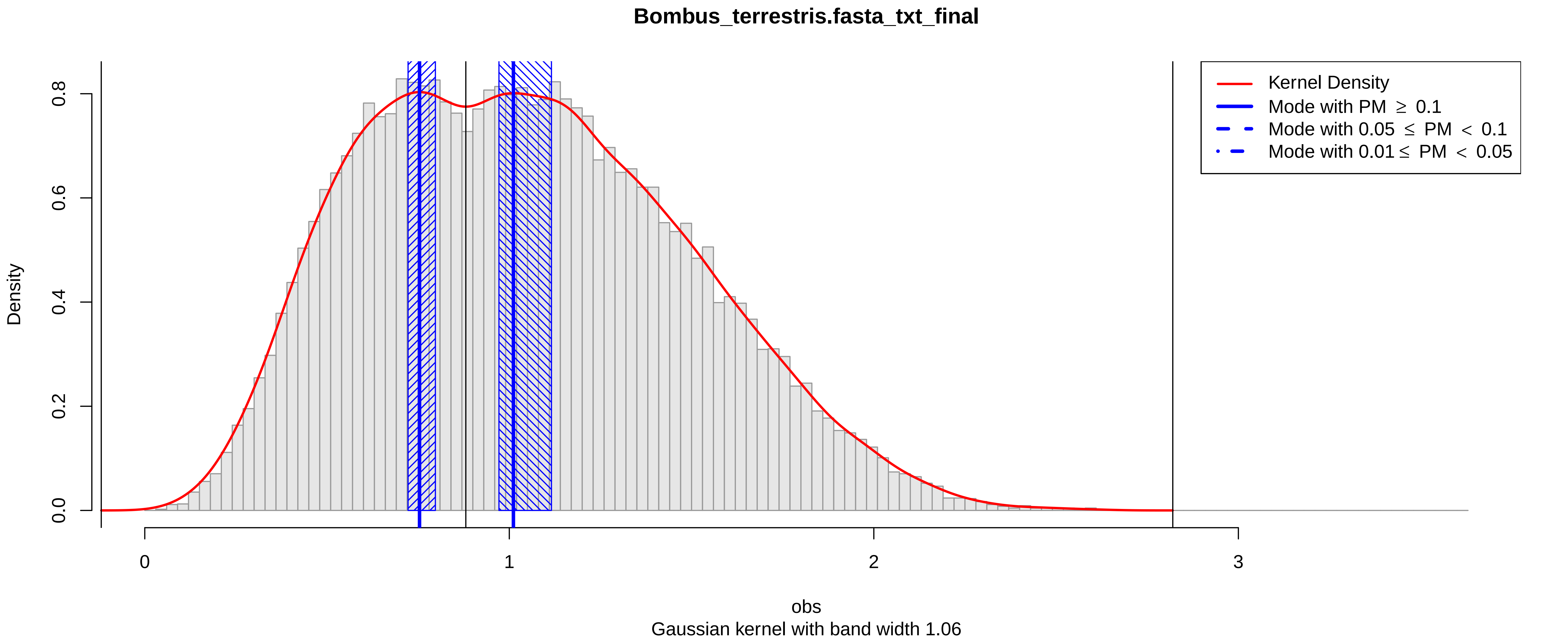

Bombus_terrestris.fasta_txt_final
Kernel Density
Mode with PM ≥ 0.1
Mode with 0.05 ≤ PM < 0.1
Mode with 0.01 ≤ PM < 0.05
Density
0.0
0.2
0.4
0.6
0.8
0
1
2
3
obs
Gaussian kernel with band width 1.06

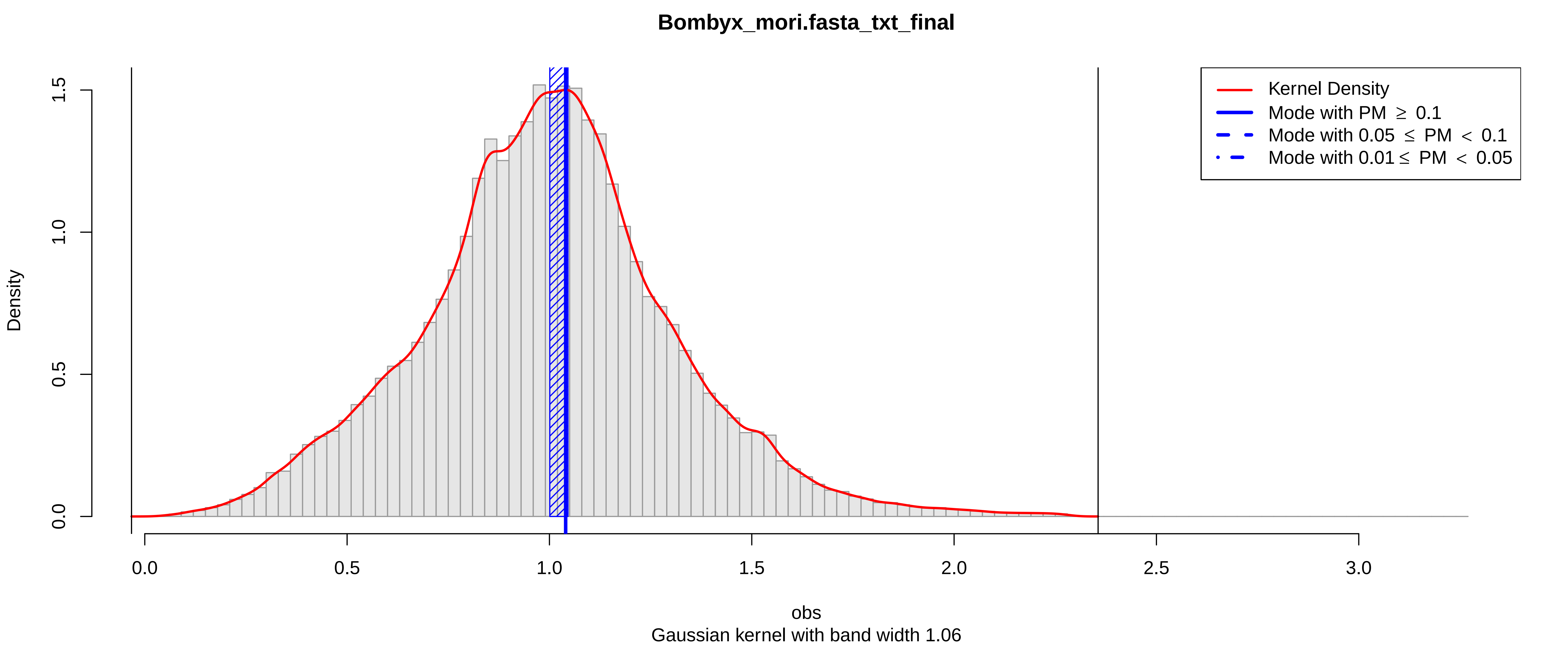

Bombyx_mori.fasta_txt_final
Density
0.0
0.5
1.0
1.5
0.0
0.5
1.0
1.5
2.0
2.5
3.0
Kernel Density
Mode with PM ≥ 0.1
Mode with 0.05 ≤ PM < 0.1
Mode with 0.01 ≤ PM < 0.05
obs
Gaussian kernel with band width 1.06

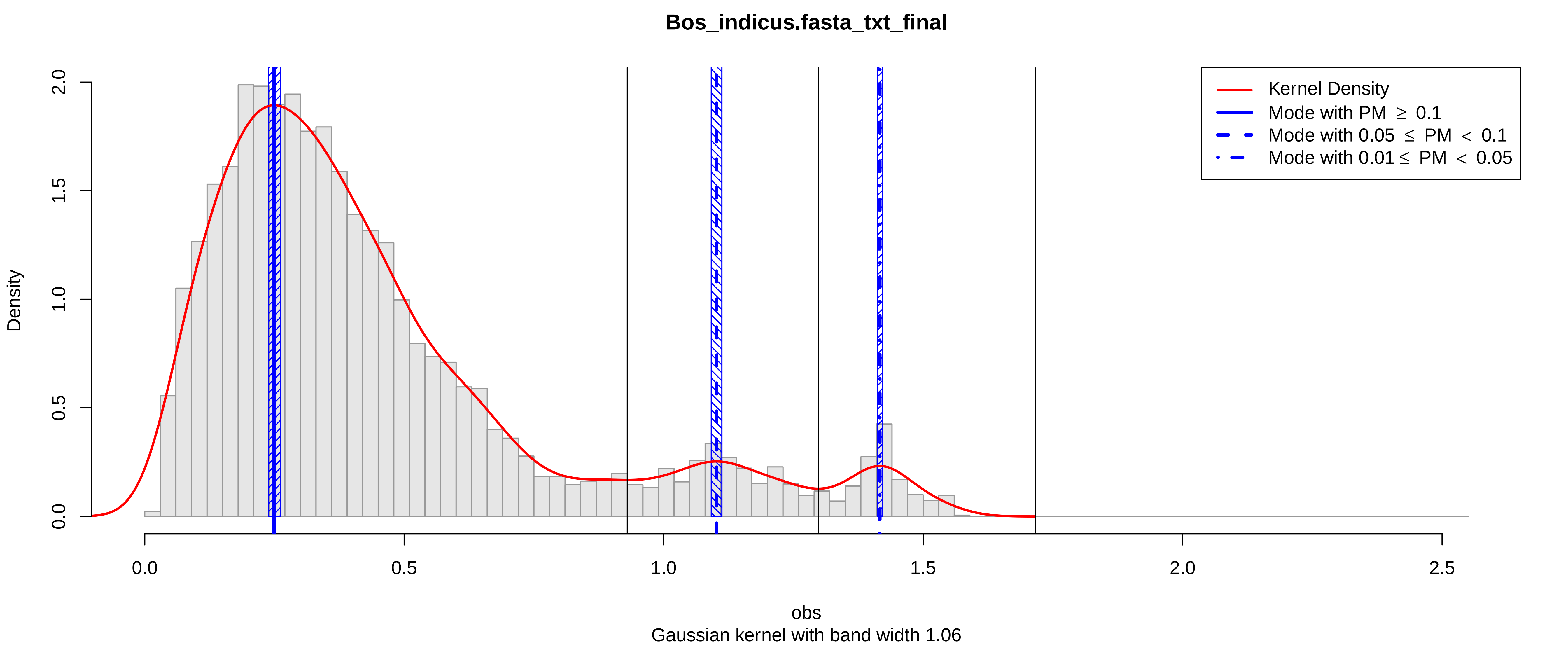

Bos_indicus.fasta_txt_final
Density
0.0
0.5
1.0
1.5
2.0
2.5
Kernel Density
Mode with PM ≥ 0.1
Mode with 0.05 ≤ PM < 0.1
Mode with 0.01 ≤ PM < 0.05
obs
Gaussian kernel with band width 1.06

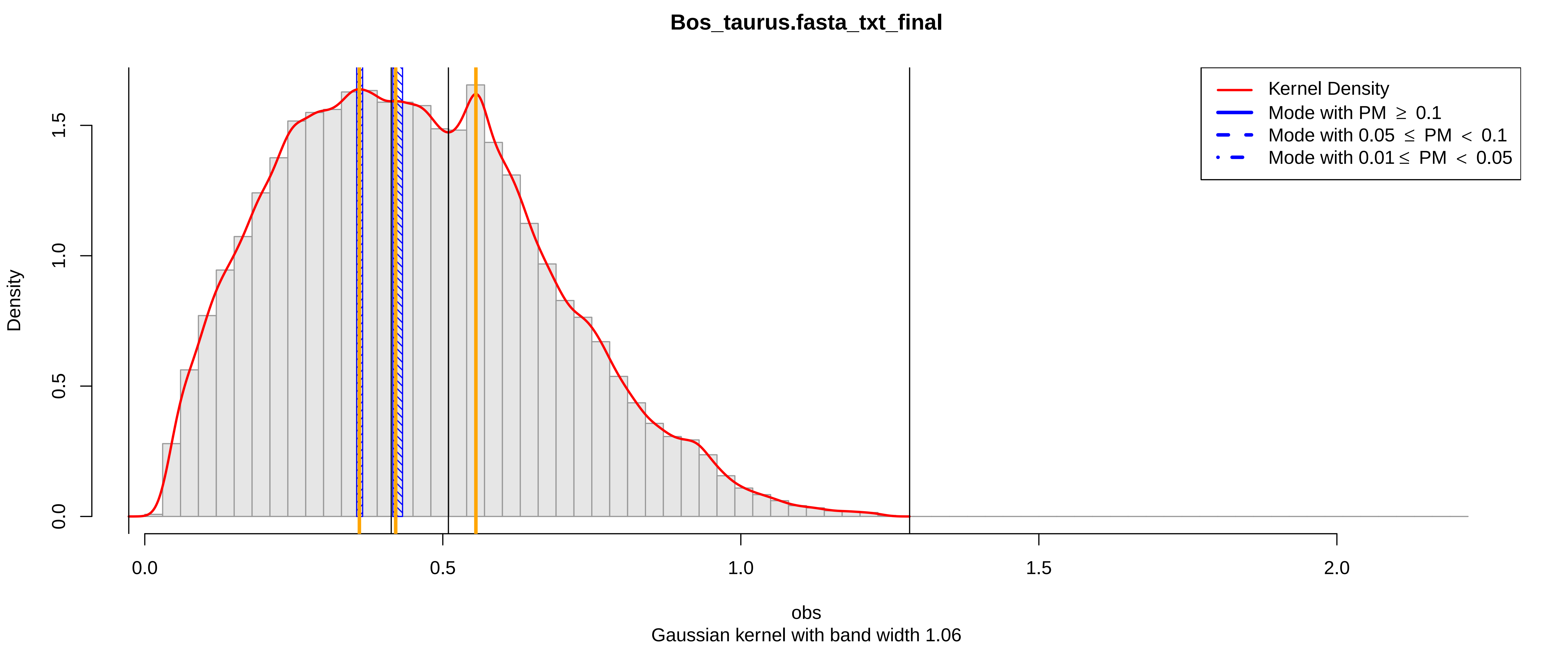

Bos_taurus.fasta_txt_final
Density
0.0
0.5
1.0
1.5
0.0
0.5
1.0
1.5
2.0
obs
Gaussian kernel with band width 1.06
Kernel Density
Mode with PM ≥ 0.1
Mode with 0.05 ≤ PM < 0.1
Mode with 0.01 ≤ PM < 0.05

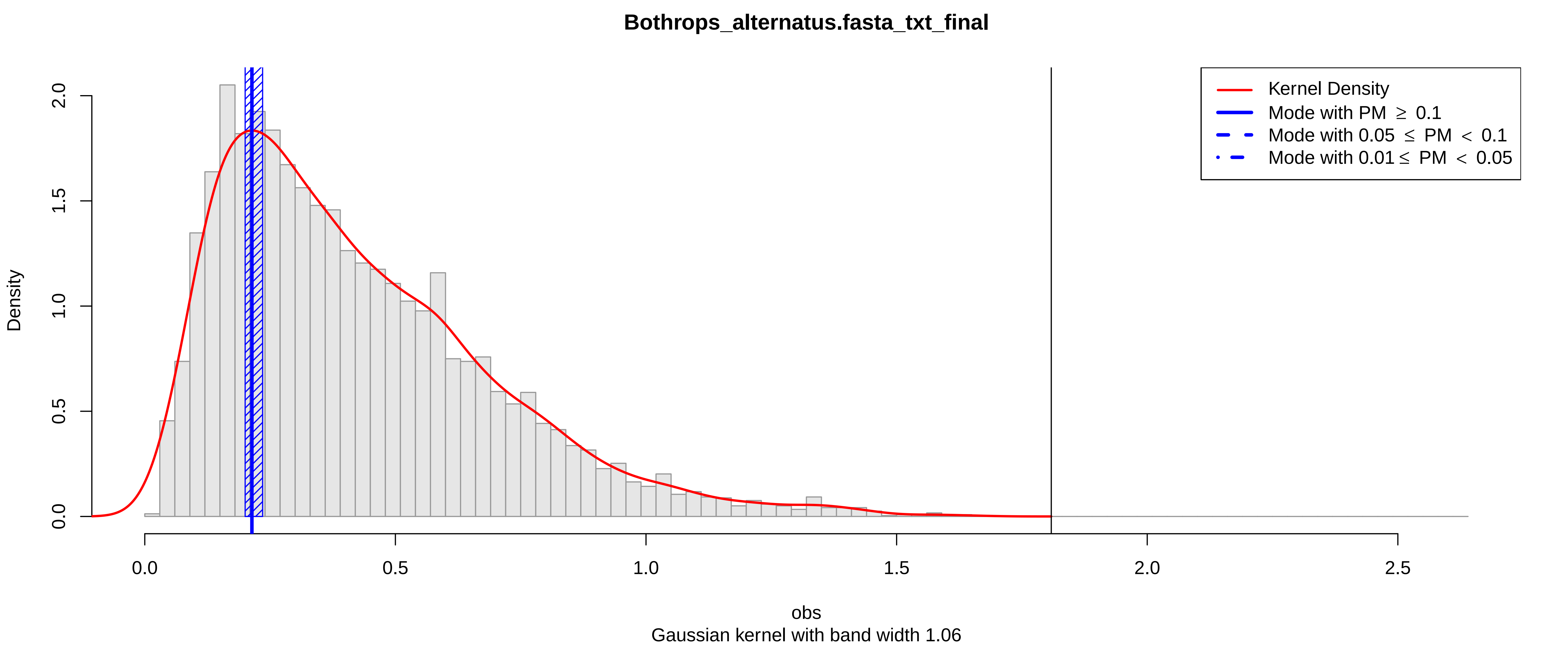
Bothrops_alternatus.fasta_txt_final
Kernel Density
Mode with PM ≥ 0.1
Mode with 0.05 ≤ PM < 0.1
Mode with 0.01 ≤ PM < 0.05
Density
0.0
0.5
1.0
1.5
2.0
0.0
0.5
1.0
1.5
2.0
2.5
obs
Gaussian kernel with band width 1.06

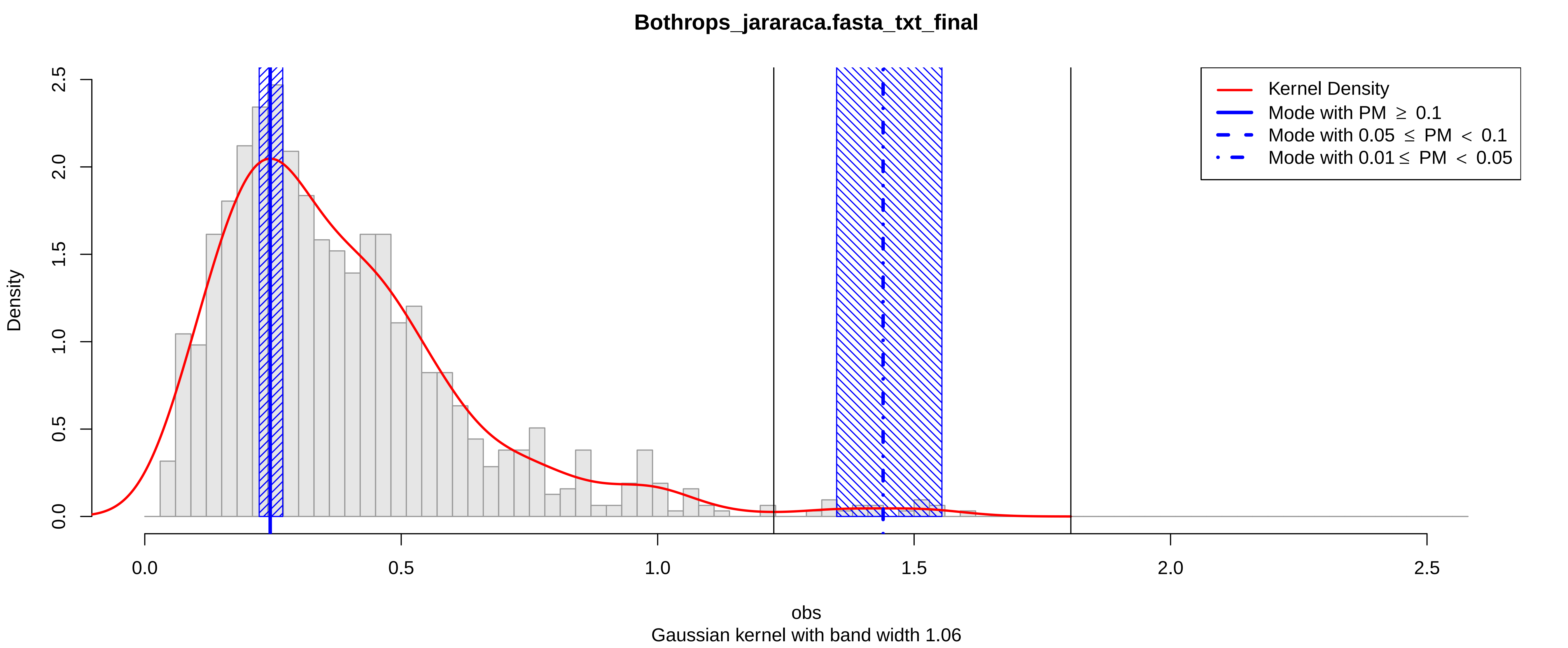

Bothrops_jararaca.fasta_txt_final
Kernel Density
Mode with PM ≥ 0.1
Mode with 0.05 ≤ PM < 0.1
Mode with 0.01 ≤ PM < 0.05
Density
0.0
0.5
1.0
1.5
2.0
2.5
obs
Gaussian kernel with band width 1.06

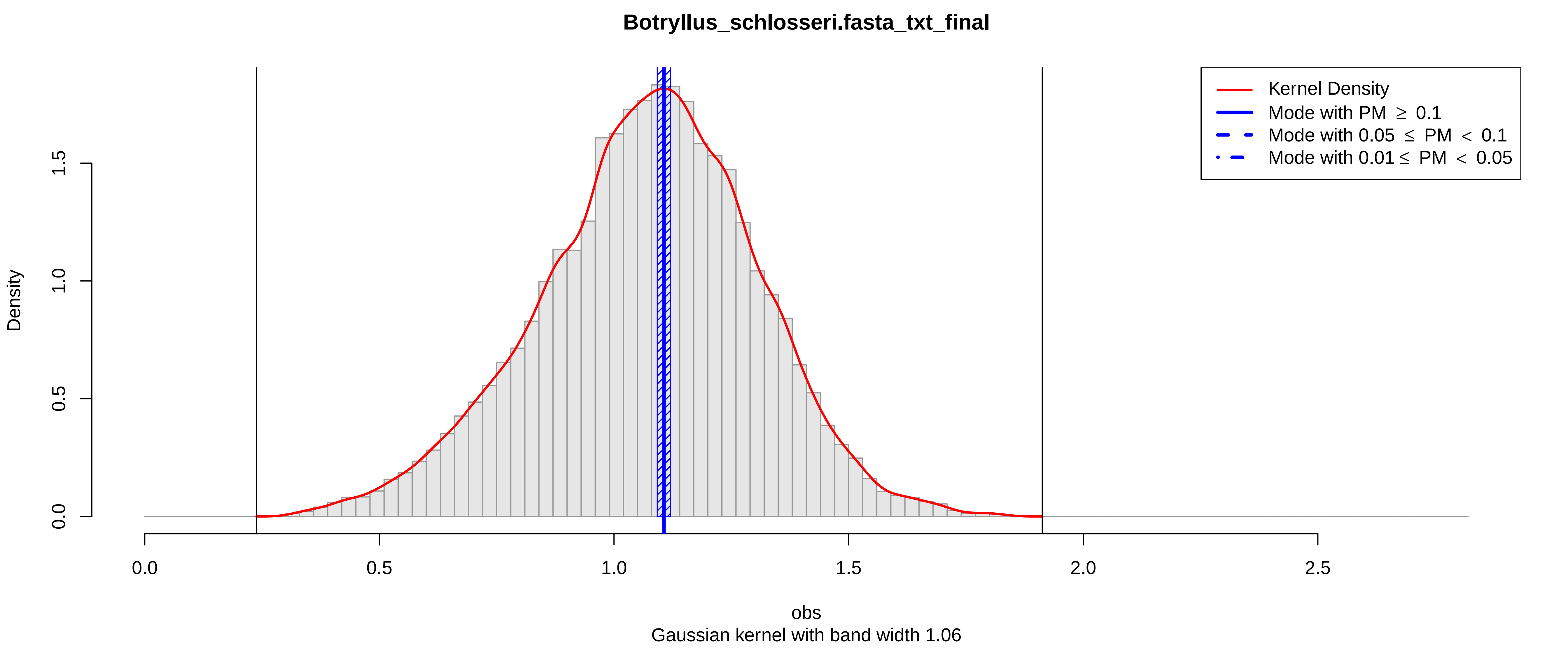

Botryllus_schlosseri.fasta_txt_final
Kernel Density
Mode with PM ≥ 0.1
Mode with 0.05 ≤ PM < 0.1
Mode with 0.01 ≤ PM < 0.05
Density
0.0
0.5
1.0
1.5
0.0
0.5
1.0
1.5
2.0
2.5
obs
Gaussian kernel with band width 1.06

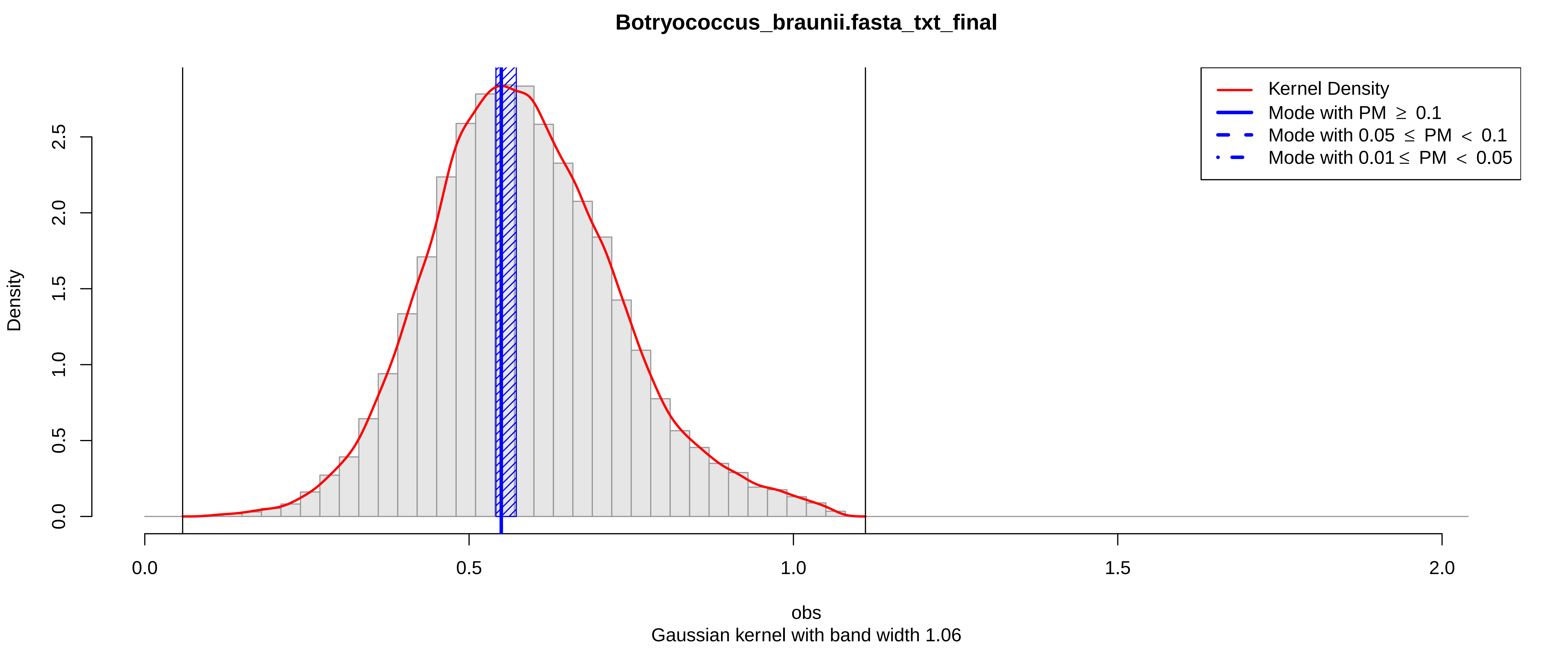

Botryococcus_braunii.fasta_txt_final
Kernel Density
Mode with PM ≥ 0.1
Mode with 0.05 ≤ PM < 0.1
Mode with 0.01 ≤ PM < 0.05
Density
0.0
0.5
1.0
1.5
2.0
2.5
0.0
0.5
1.0
1.5
2.0
obs
Gaussian kernel with band width 1.06

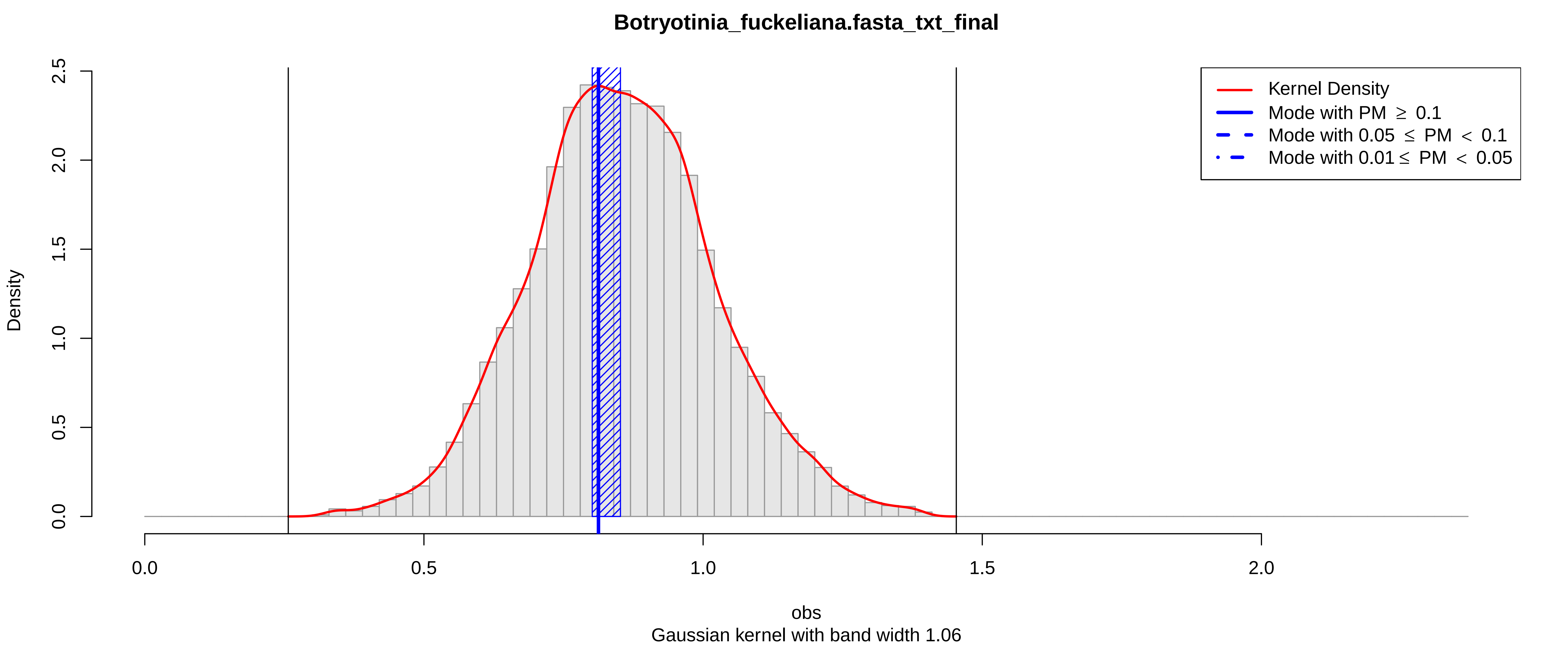

Botryotinia_fuckeliana.fasta_txt_final
Density
2.5
2.0
1.5
1.0
0.5
0.0
0.0
0.5
1.0
1.5
2.0
obs
Gaussian kernel with band width 1.06
Kernel Density
Mode with PM ≥ 0.1
Mode with 0.05 ≤ PM < 0.1
Mode with 0.01 ≤ PM < 0.05

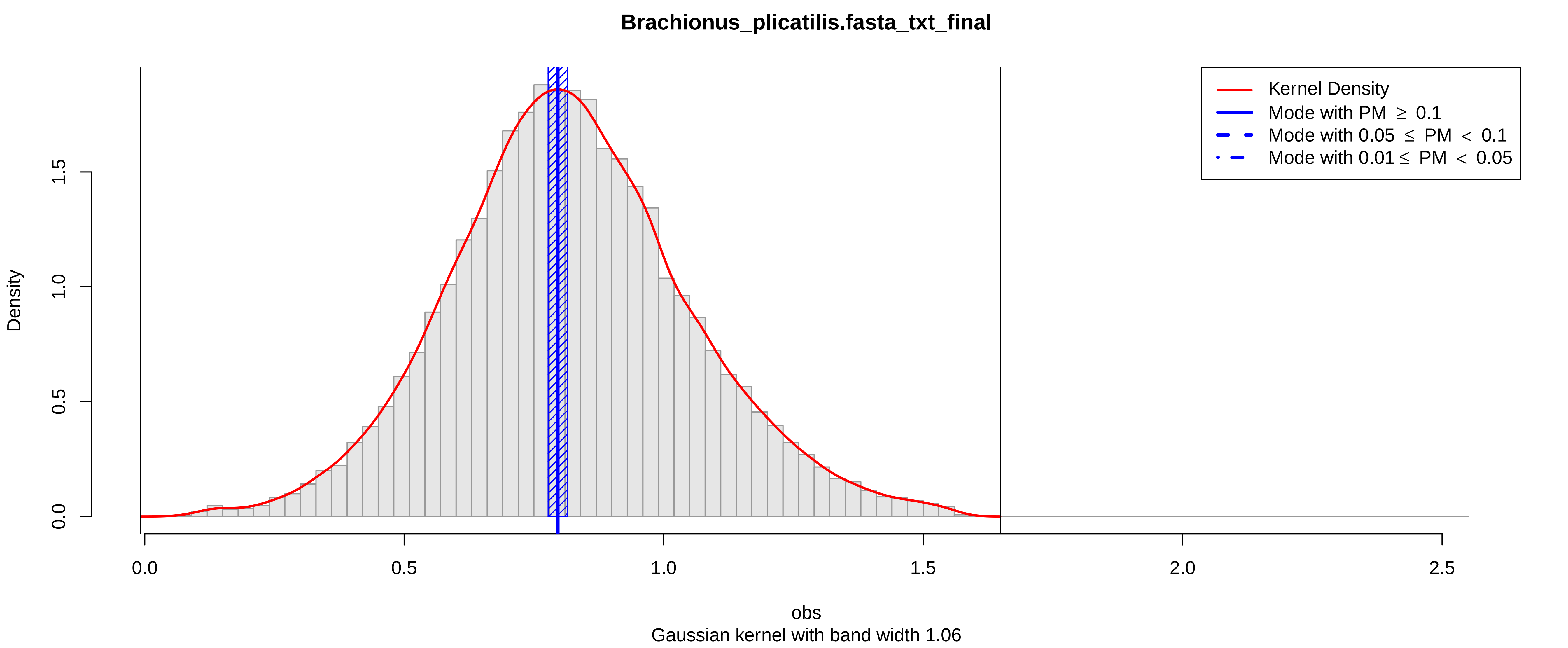

Brachionus_plicatilis.fasta_txt_final
Kernel Density
Mode with PM ≥ 0.1
Mode with 0.05 ≤ PM < 0.1
Mode with 0.01 ≤ PM < 0.05
Density
0.0
0.5
1.0
1.5
0.0
0.5
1.0
1.5
2.0
2.5
obs
Gaussian kernel with band width 1.06

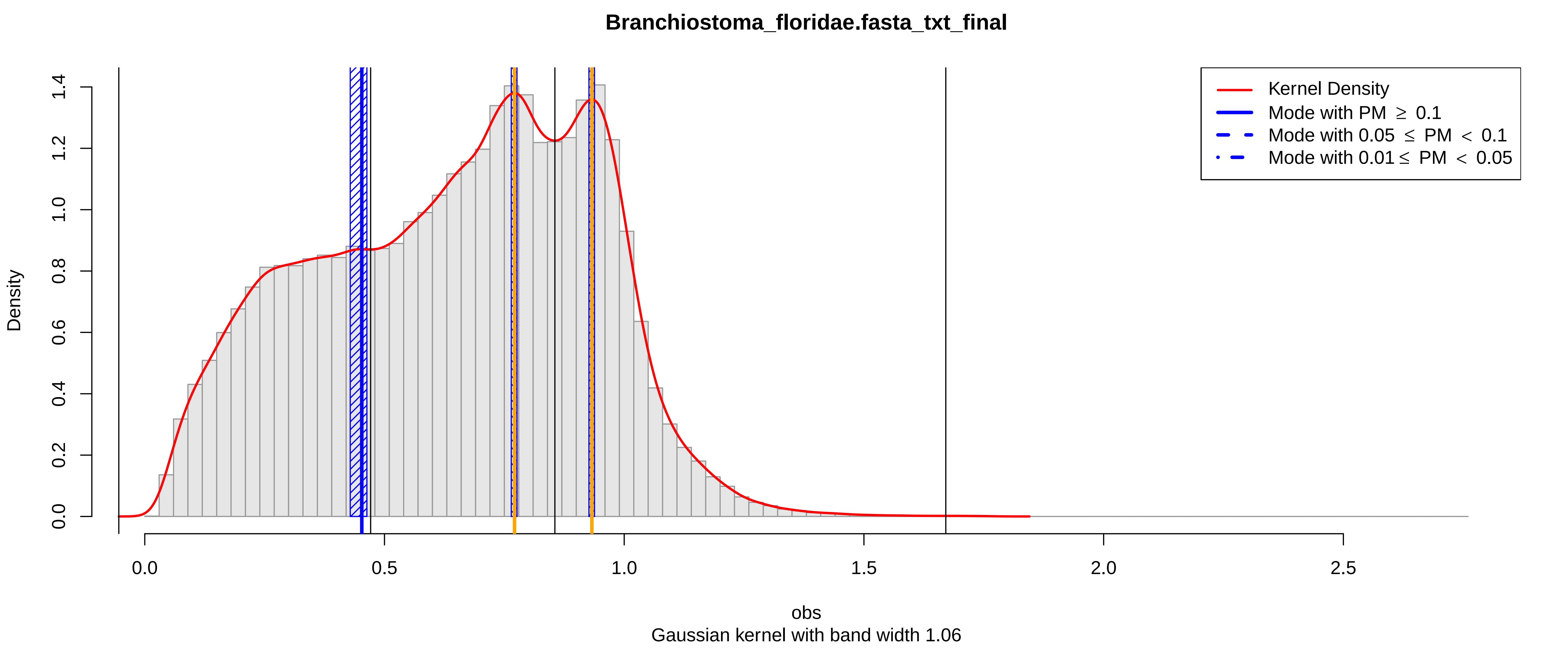

Branchiostoma_floridae.fasta_txt_final
Kernel Density
Mode with PM ≥ 0.1
Mode with 0.05 ≤ PM < 0.1
Mode with 0.01 ≤ PM < 0.05
Density
0.0
0.2
0.4
0.6
0.8
1.0
1.2
1.4
0.0
0.5
1.0
1.5
2.0
2.5
obs
Gaussian kernel with band width 1.06

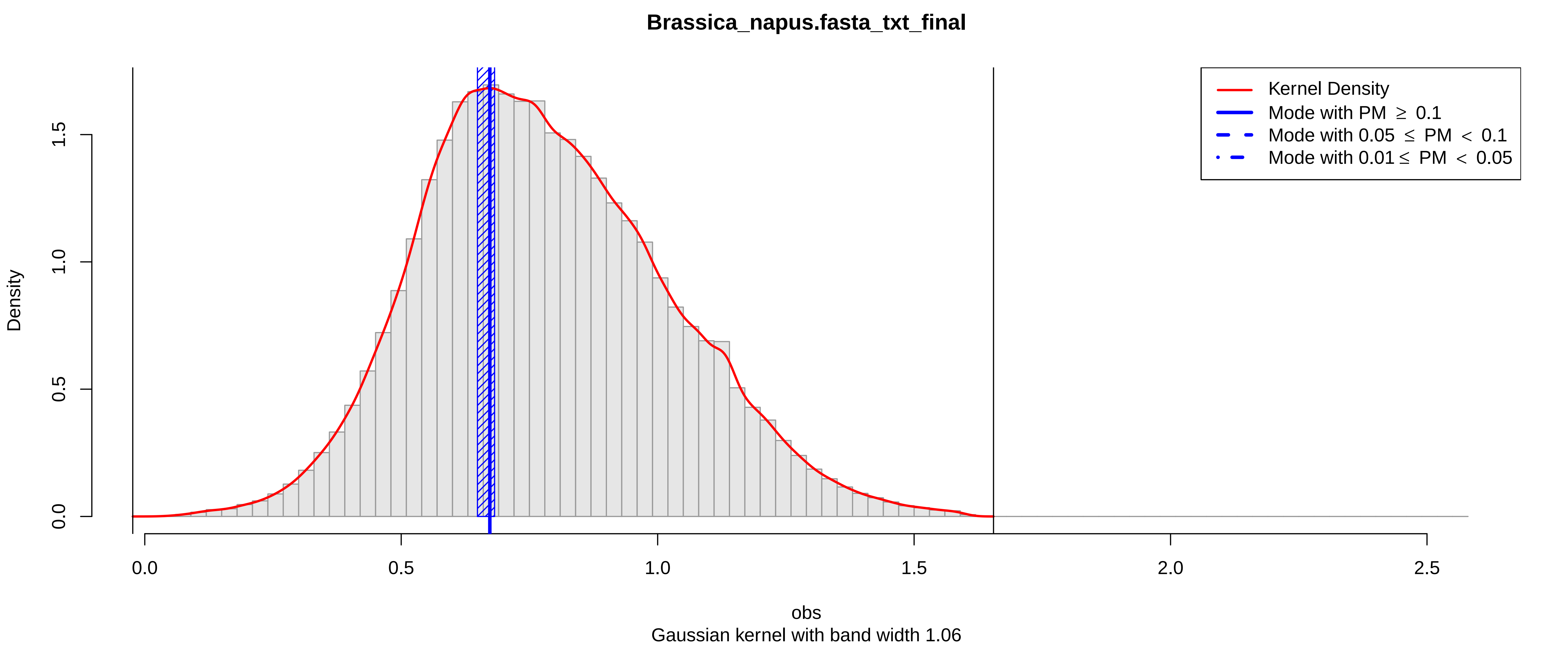

Brassica_napus.fasta_txt_final
Kernel Density
Mode with PM ≥ 0.1
Mode with 0.05 ≤ PM < 0.1
Mode with 0.01 ≤ PM < 0.05
Density
0.0
0.5
1.0
1.5
2.0
2.5
obs
Gaussian kernel with band width 1.06

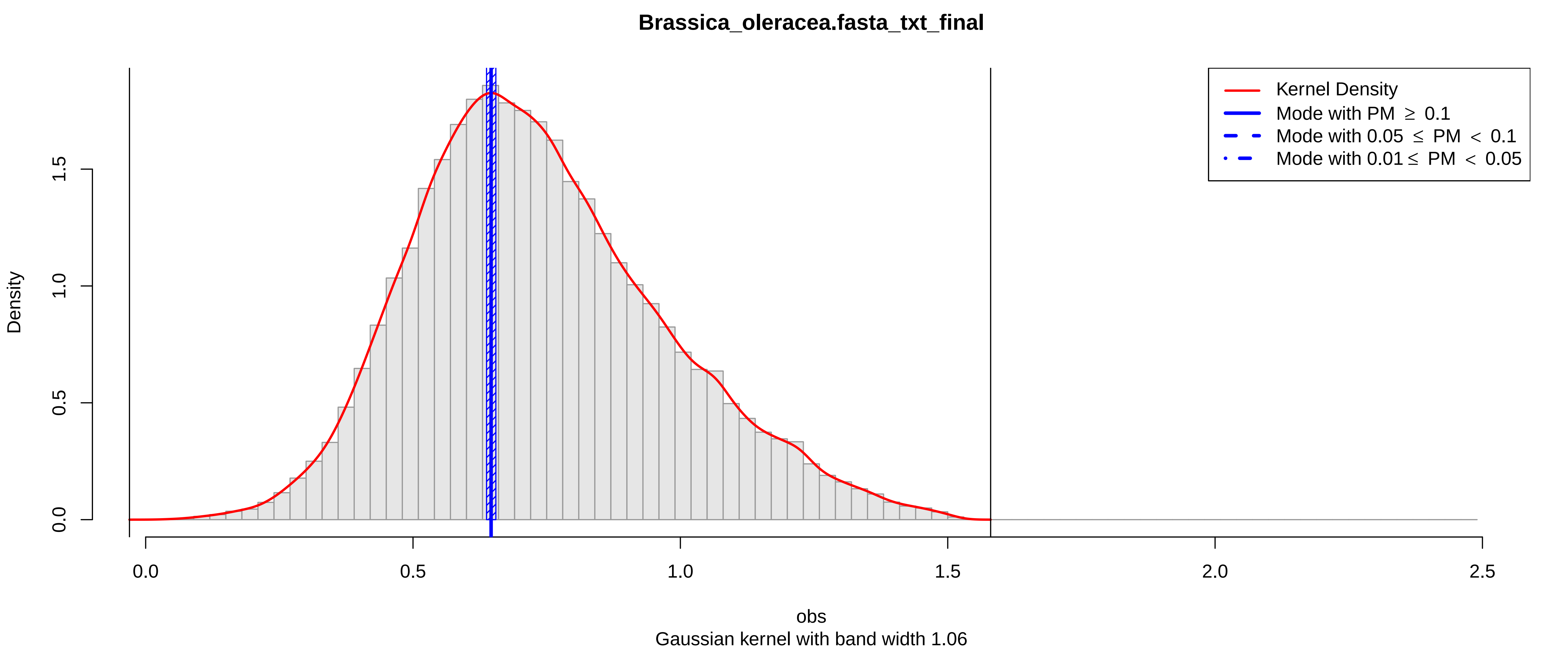

Brassica_oleracea.fasta_txt_final
Kernel Density
Mode with PM ≥ 0.1
Mode with 0.05 ≤ PM < 0.1
Mode with 0.01 ≤ PM < 0.05
Density
0.0
0.5
1.0
1.5
2.0
2.5
obs
Gaussian kernel with band width 1.06

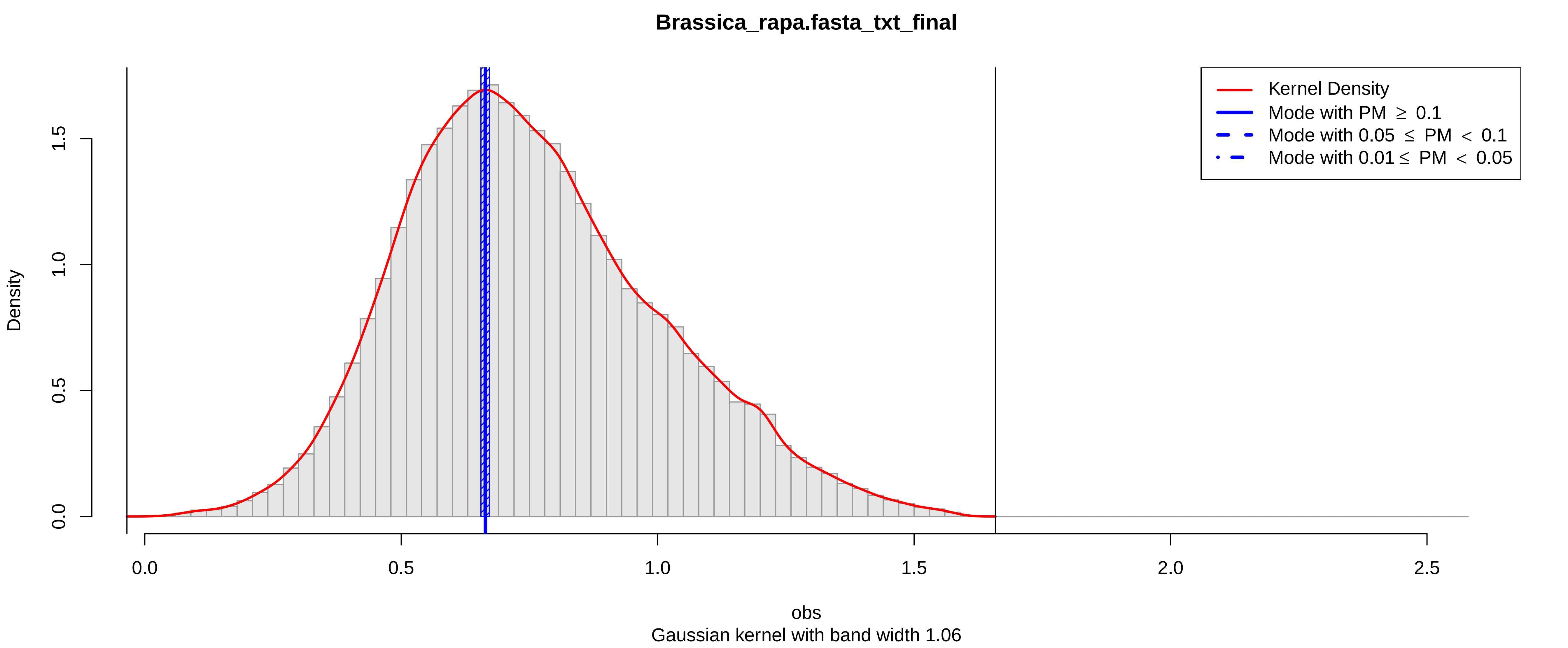

Brassica_rapa.fasta_txt_final
Kernel Density
Mode with PM ≥ 0.1
Mode with 0.05 ≤ PM < 0.1
Mode with 0.01 ≤ PM < 0.05
Density
0.0
0.5
1.0
1.5
0.0
0.5
1.0
1.5
2.0
2.5
obs
Gaussian kernel with band width 1.06

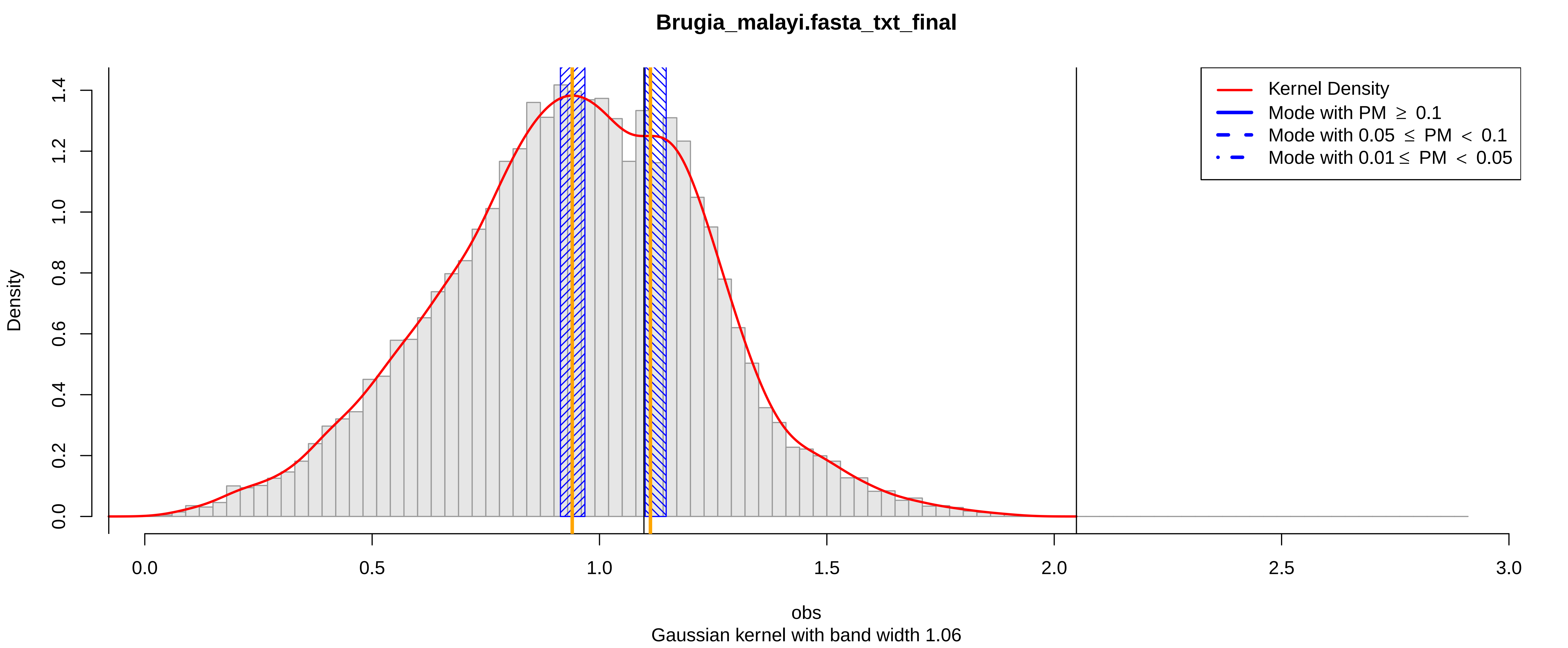
Brugia_malayi.fasta_txt_final
Kernel Density
Mode with PM ≥ 0.1
Mode with 0.05 ≤ PM < 0.1
Mode with 0.01 ≤ PM < 0.05
Density
0.0
0.2
0.4
0.6
0.8
1.0
1.2
1.4
0.0
0.5
1.0
1.5
2.0
2.5
3.0
obs
Gaussian kernel with band width 1.06

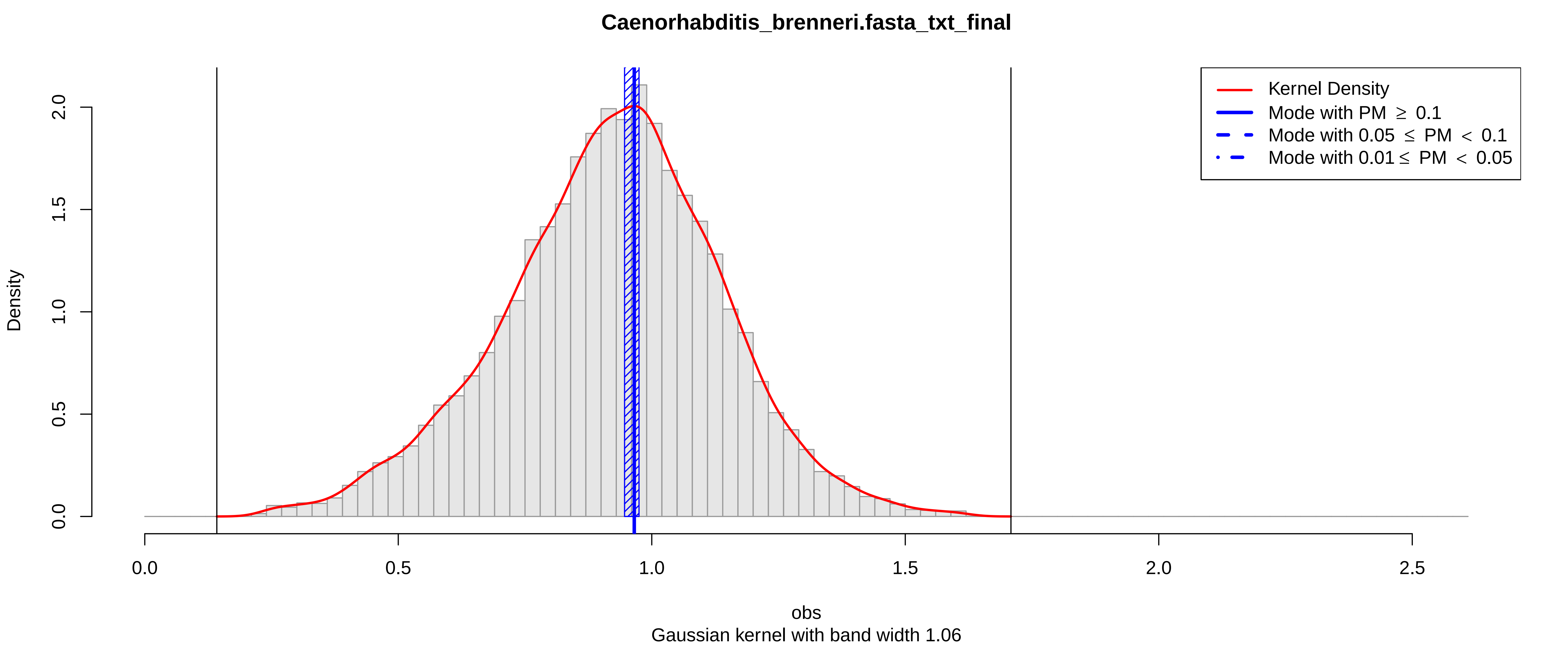

Caenorhabditis_brenneri.fasta_txt_final
Kernel Density
Mode with PM ≥ 0.1
Mode with 0.05 ≤ PM < 0.1
Mode with 0.01 ≤ PM < 0.05
Density
0.0
0.5
1.0
1.5
2.0
0.0
0.5
1.0
1.5
2.0
2.5
obs
Gaussian kernel with band width 1.06

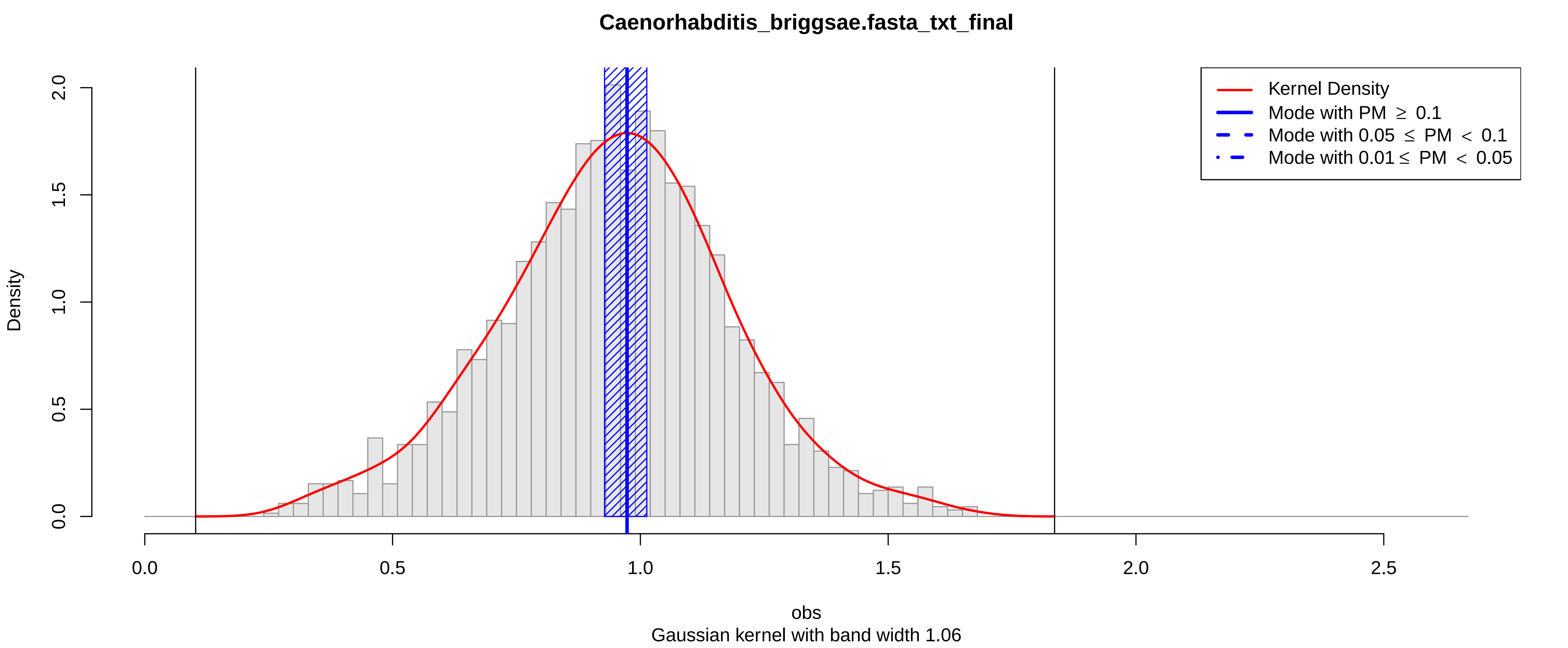
Caenorhabditis_briggsae.fasta_txt_final
Kernel Density
Mode with PM ≥ 0.1
Mode with 0.05 ≤ PM < 0.1
Mode with 0.01 ≤ PM < 0.05
Density
0.0
0.5
1.0
1.5
2.0
0.0
0.5
1.0
1.5
2.0
2.5
obs
Gaussian kernel with band width 1.06

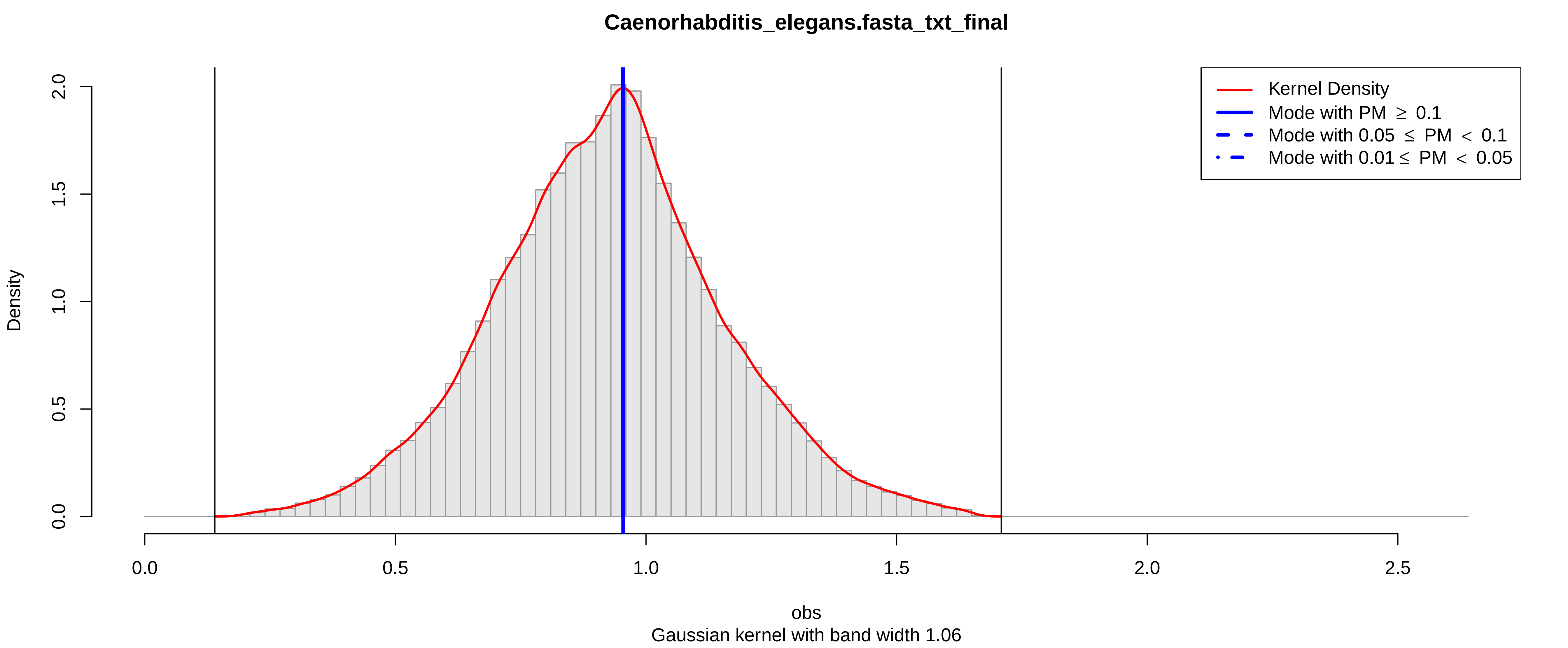

Caenorhabditis_elegans.fasta_txt_final
Kernel Density
Mode with PM ≥ 0.1
Mode with 0.05 ≤ PM < 0.1
Mode with 0.01 ≤ PM < 0.05
Density
0.0
0.5
1.0
1.5
2.0
2.5
obs
Gaussian kernel with band width 1.06

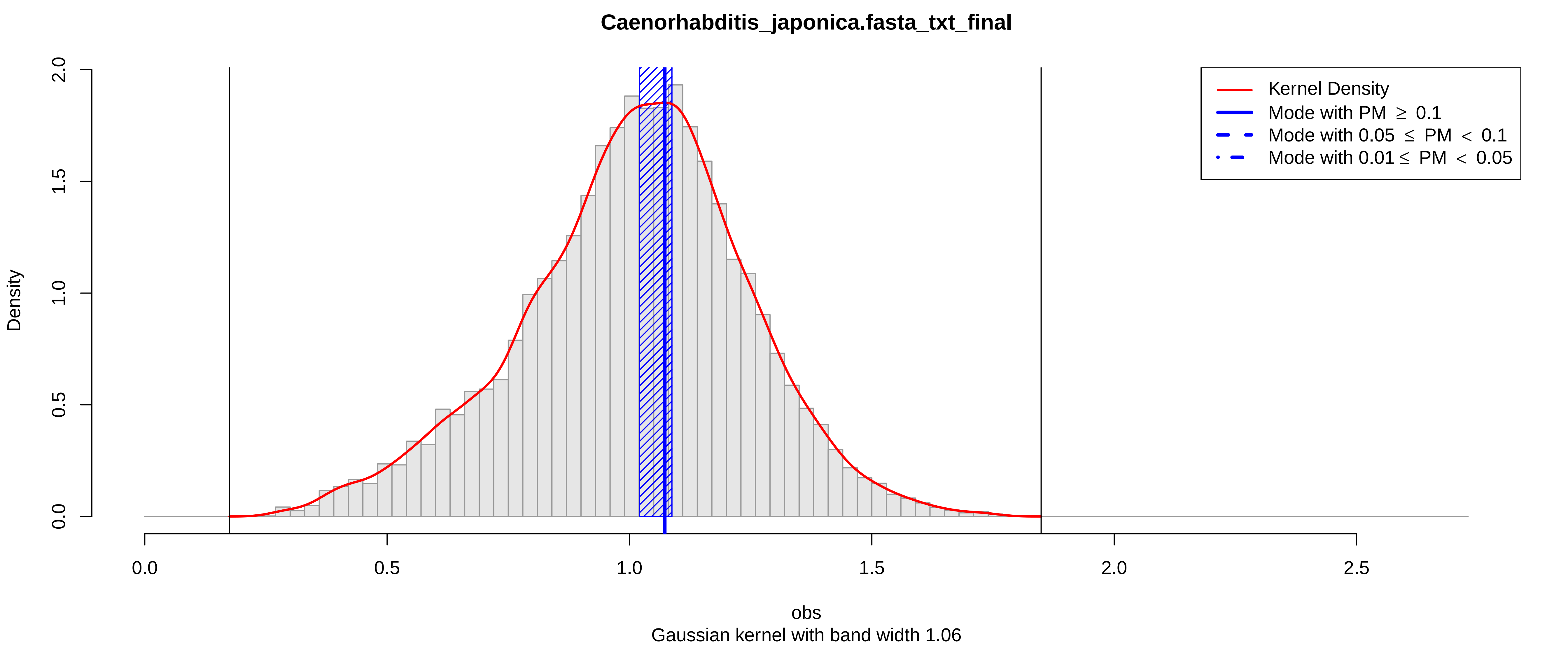

Caenorhabditis_japonica.fasta_txt_final
Density
2.0
1.5
1.0
0.5
0.0
0.0
0.5
1.0
1.5
2.0
2.5
Kernel Density
Mode with PM ≥ 0.1
Mode with 0.05 ≤ PM < 0.1
Mode with 0.01 ≤ PM < 0.05
obs
Gaussian kernel with band width 1.06

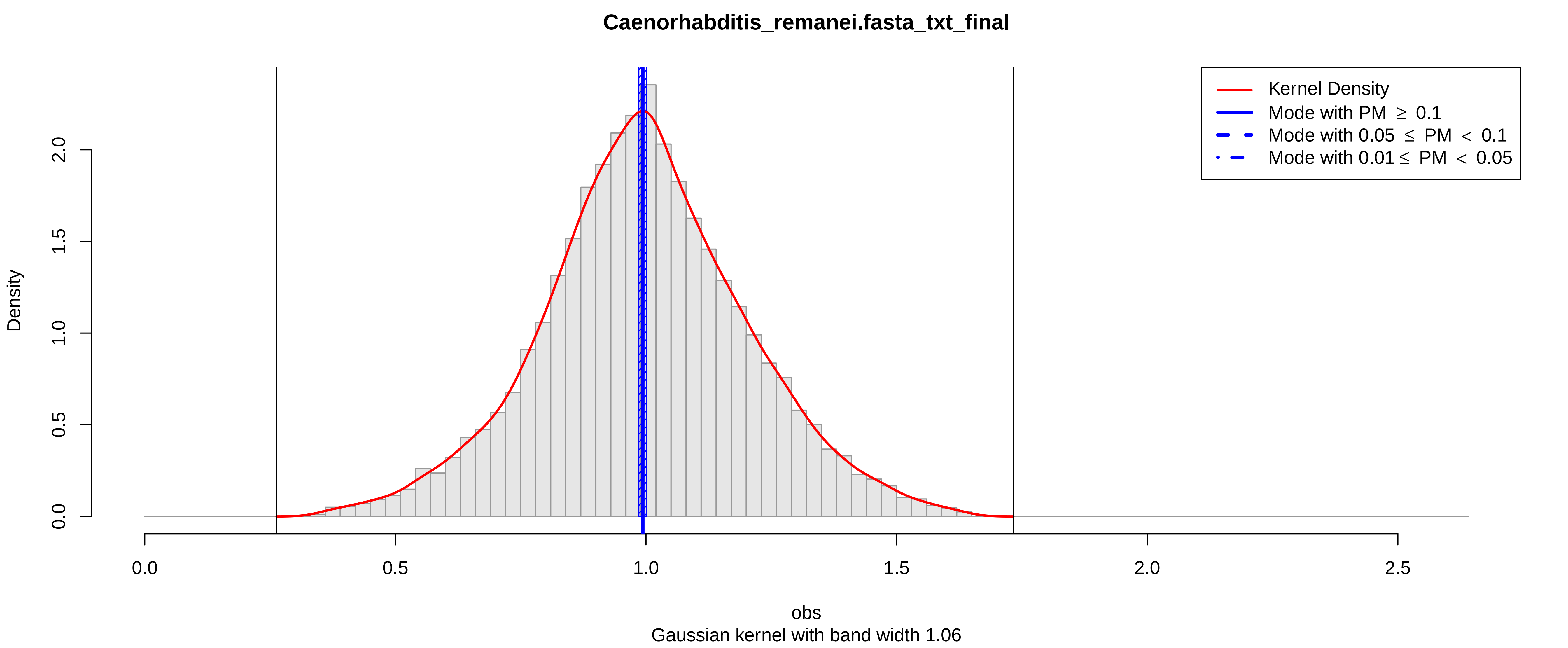

Caenorhabditis_remanei.fasta_txt_final
Kernel Density
Mode with PM ≥ 0.1
Mode with 0.05 ≤ PM < 0.1
Mode with 0.01 ≤ PM < 0.05
Density
0.0
0.5
1.0
1.5
2.0
0.0
0.5
1.0
1.5
2.0
2.5
obs
Gaussian kernel with band width 1.06

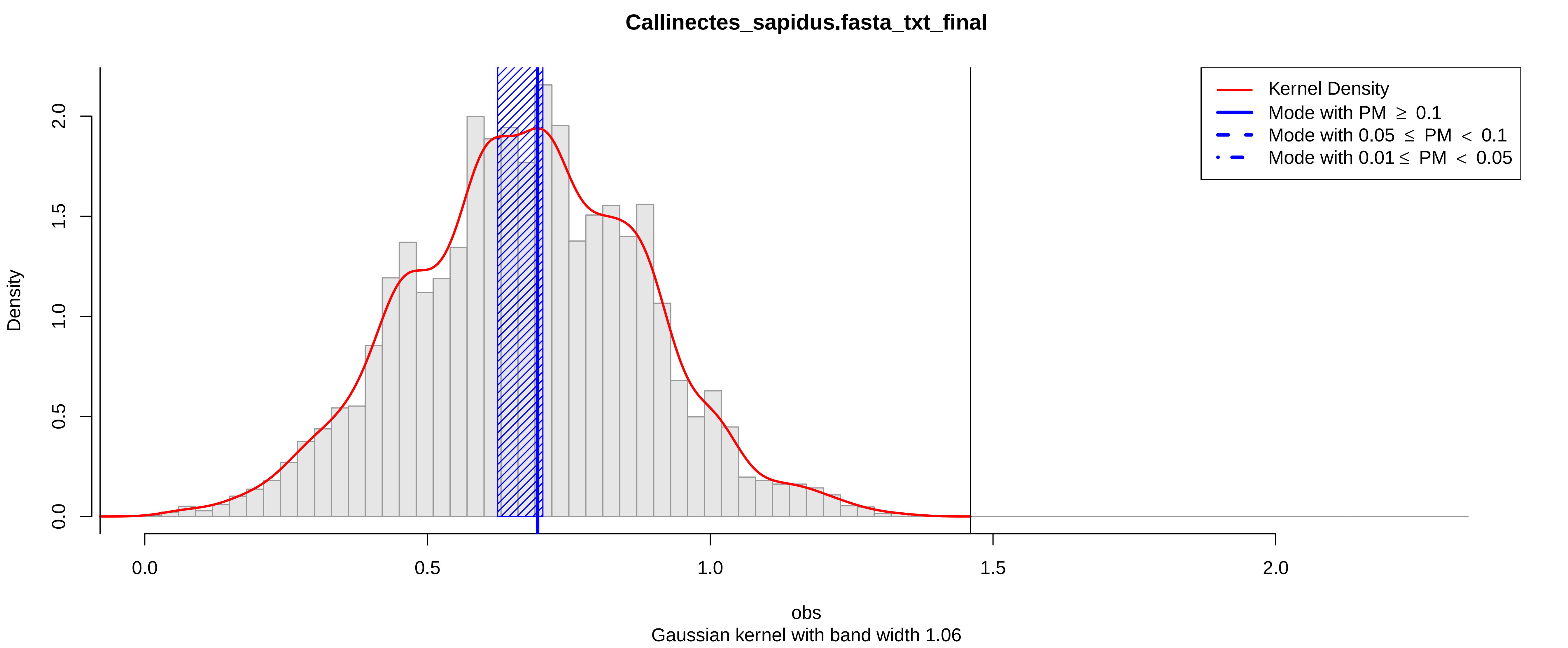

Callinectes_sapidus.fasta_txt_final
Kernel Density
Mode with PM ≥ 0.1
Mode with 0.05 ≤ PM < 0.1
Mode with 0.01 ≤ PM < 0.05
Density
0.0
0.5
1.0
1.5
2.0
0.0
0.5
1.0
1.5
2.0
obs
Gaussian kernel with band width 1.06

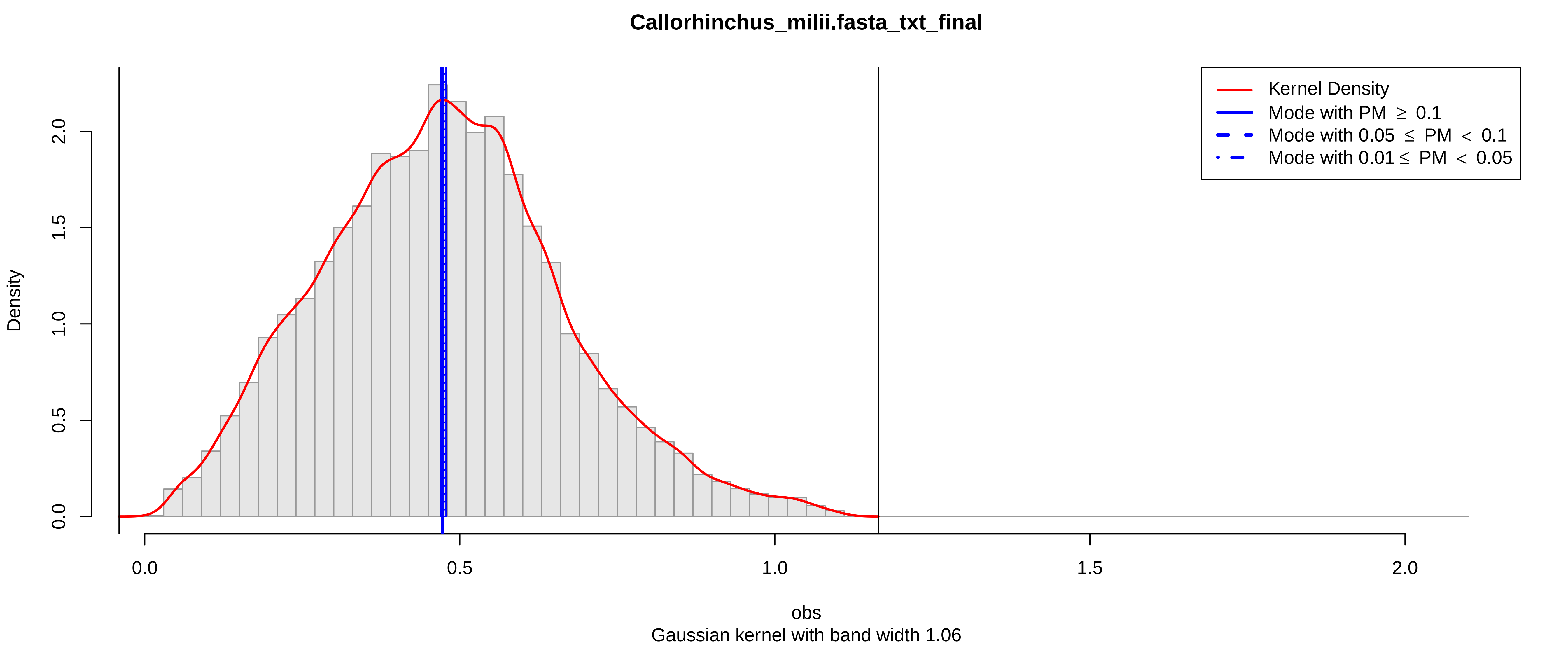

Callorhinchus_milii.fasta_txt_final
Kernel Density
Mode with PM ≥ 0.1
Mode with 0.05 ≤ PM < 0.1
Mode with 0.01 ≤ PM < 0.05
Density
0.0
0.5
1.0
1.5
2.0
obs
Gaussian kernel with band width 1.06

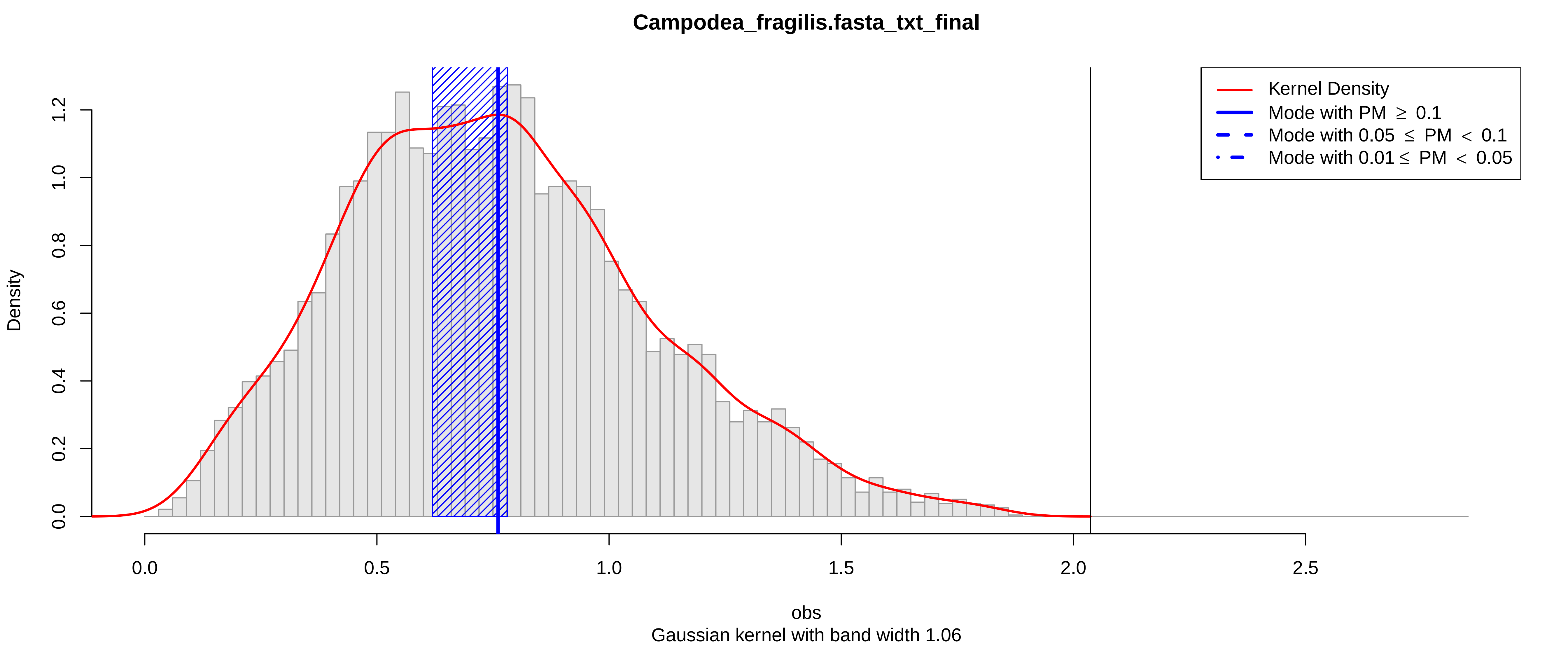

Campodea_fragilis.fasta_txt_final
Kernel Density
Mode with PM ≥ 0.1
Mode with 0.05 ≤ PM < 0.1
Mode with 0.01 ≤ PM < 0.05
Density
0.0
0.2
0.4
0.6
0.8
1.0
1.2
0.0
0.5
1.0
1.5
2.0
2.5
obs
Gaussian kernel with band width 1.06

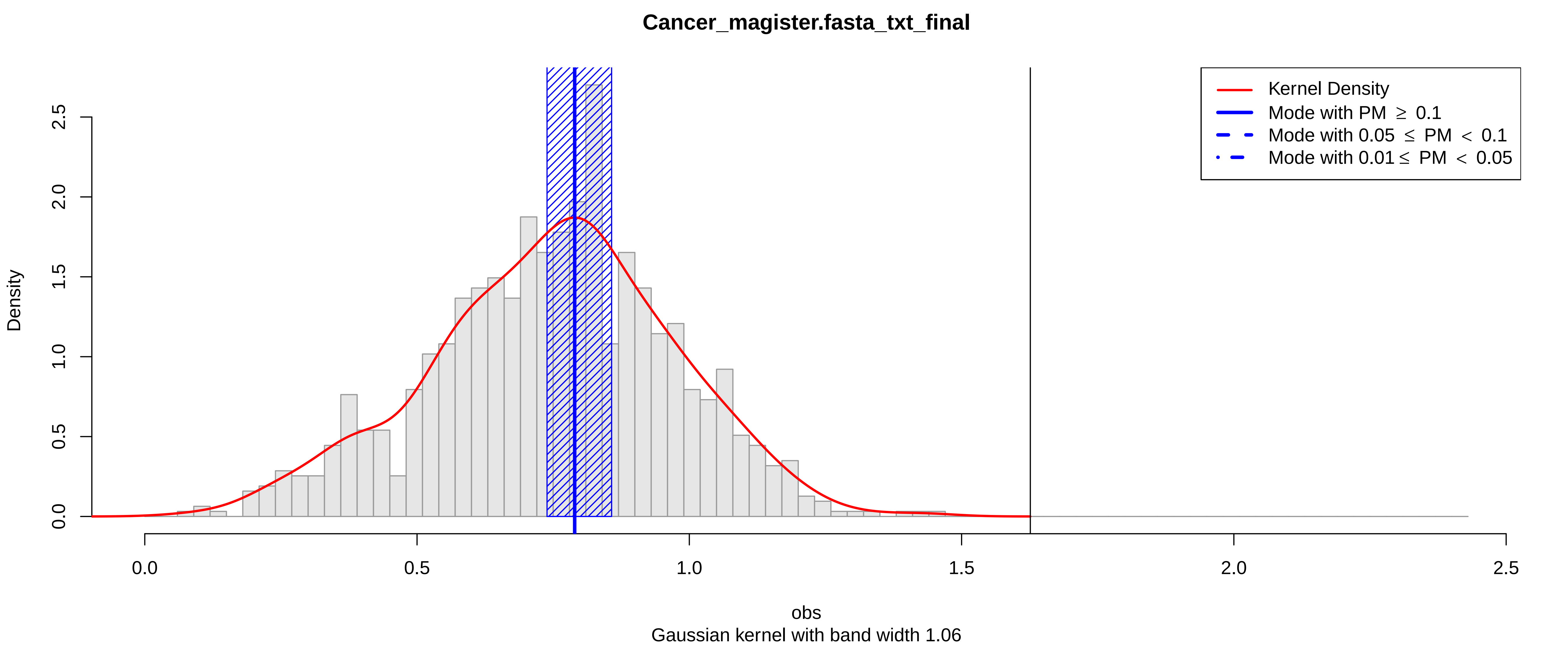

Cancer_magister.fasta_txt_final
Kernel Density
Mode with PM ≥ 0.1
Mode with 0.05 ≤ PM < 0.1
Mode with 0.01 ≤ PM < 0.05
Density
0.0
0.5
1.0
1.5
2.0
2.5
0.0
0.5
1.0
1.5
2.0
2.5
obs
Gaussian kernel with band width 1.06

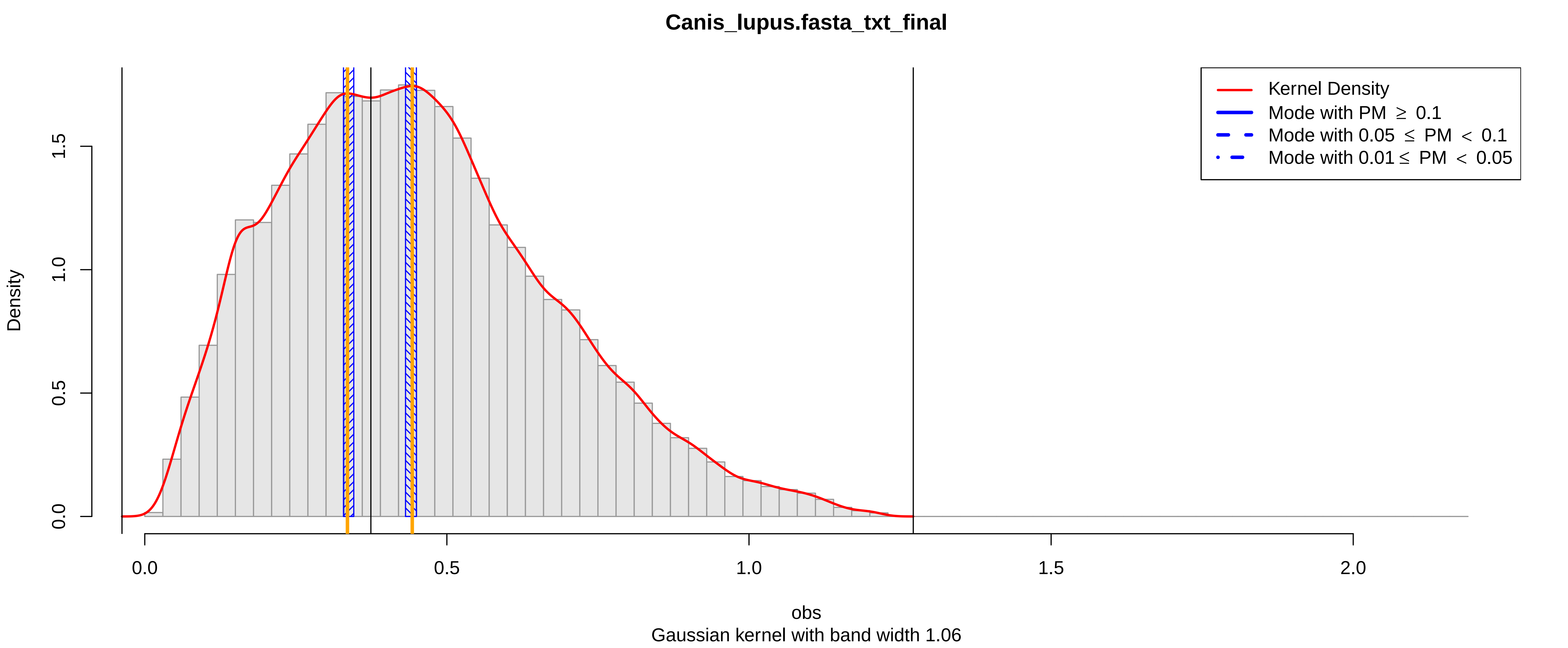

Canis_lupus.fasta_txt_final
Kernel Density
Mode with PM ≥ 0.1
Mode with 0.05 ≤ PM < 0.1
Mode with 0.01 ≤ PM < 0.05
Density
0.0
0.5
1.0
1.5
2.0
obs
Gaussian kernel with band width 1.06

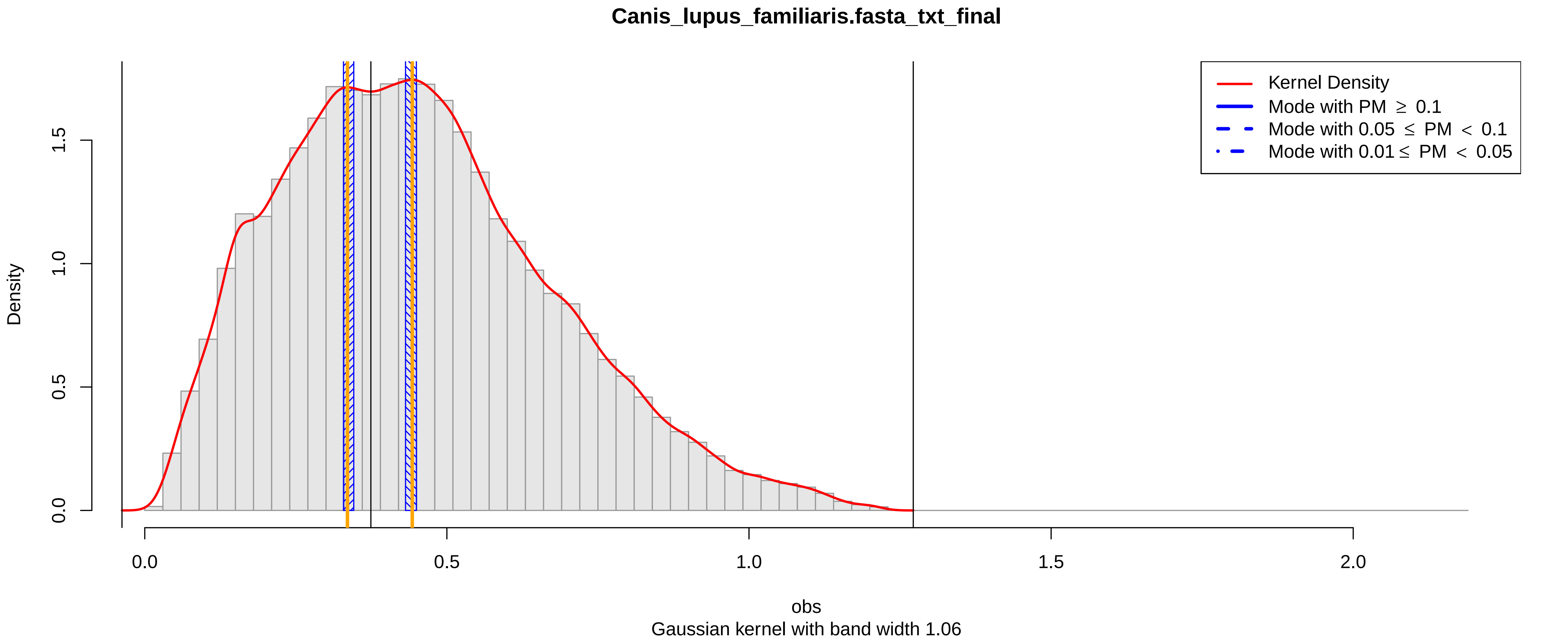
Canis_lupus_familiaris.fasta_txt_final
Kernel Density
Mode with PM ≥ 0.1
Mode with 0.05 ≤ PM < 0.1
Mode with 0.01 ≤ PM < 0.05
Density
0.0
0.5
1.0
1.5
0.0
0.5
1.0
1.5
2.0
obs
Gaussian kernel with band width 1.06

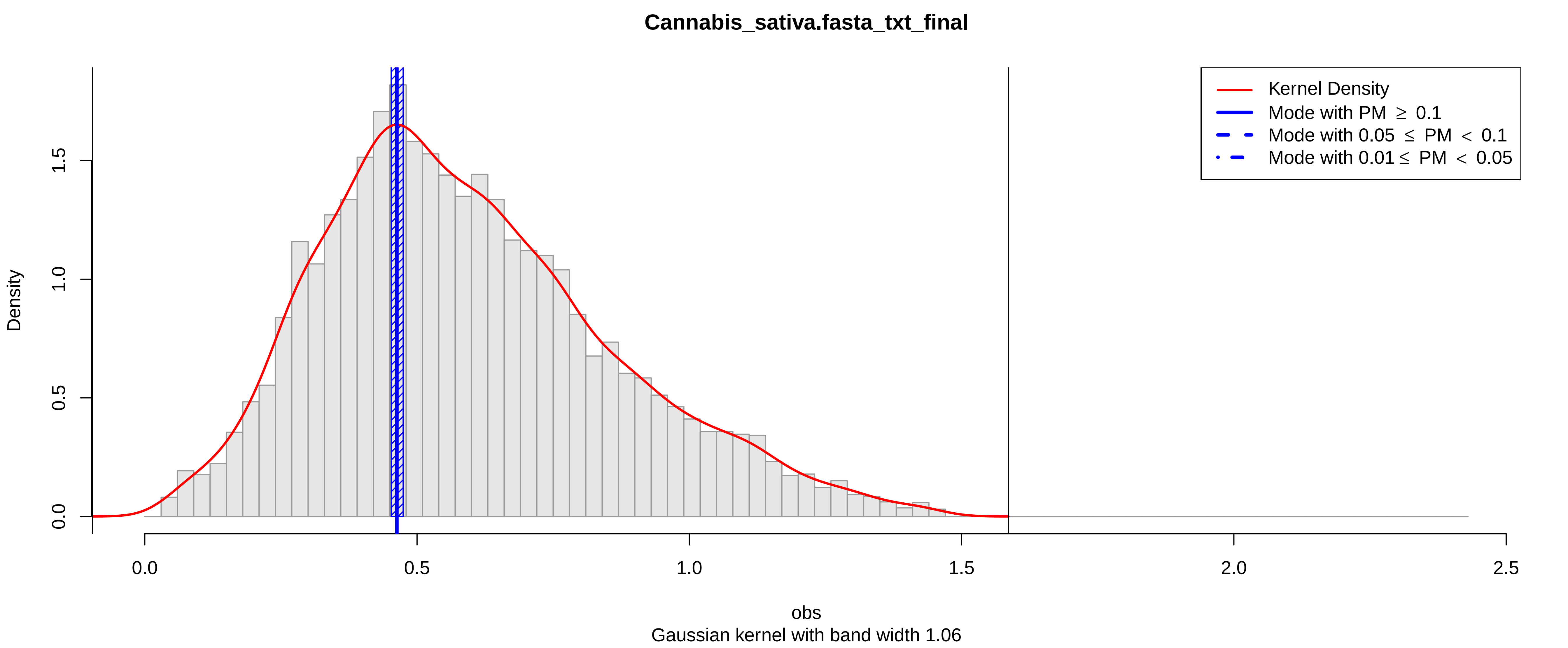
Cannabis_sativa.fasta_txt_final
Kernel Density
Mode with PM ≥ 0.1
Mode with 0.05 ≤ PM < 0.1
Mode with 0.01 ≤ PM < 0.05
Density
0.0
0.5
1.0
1.5
0.0
0.5
1.0
1.5
2.0
2.5
obs
Gaussian kernel with band width 1.06

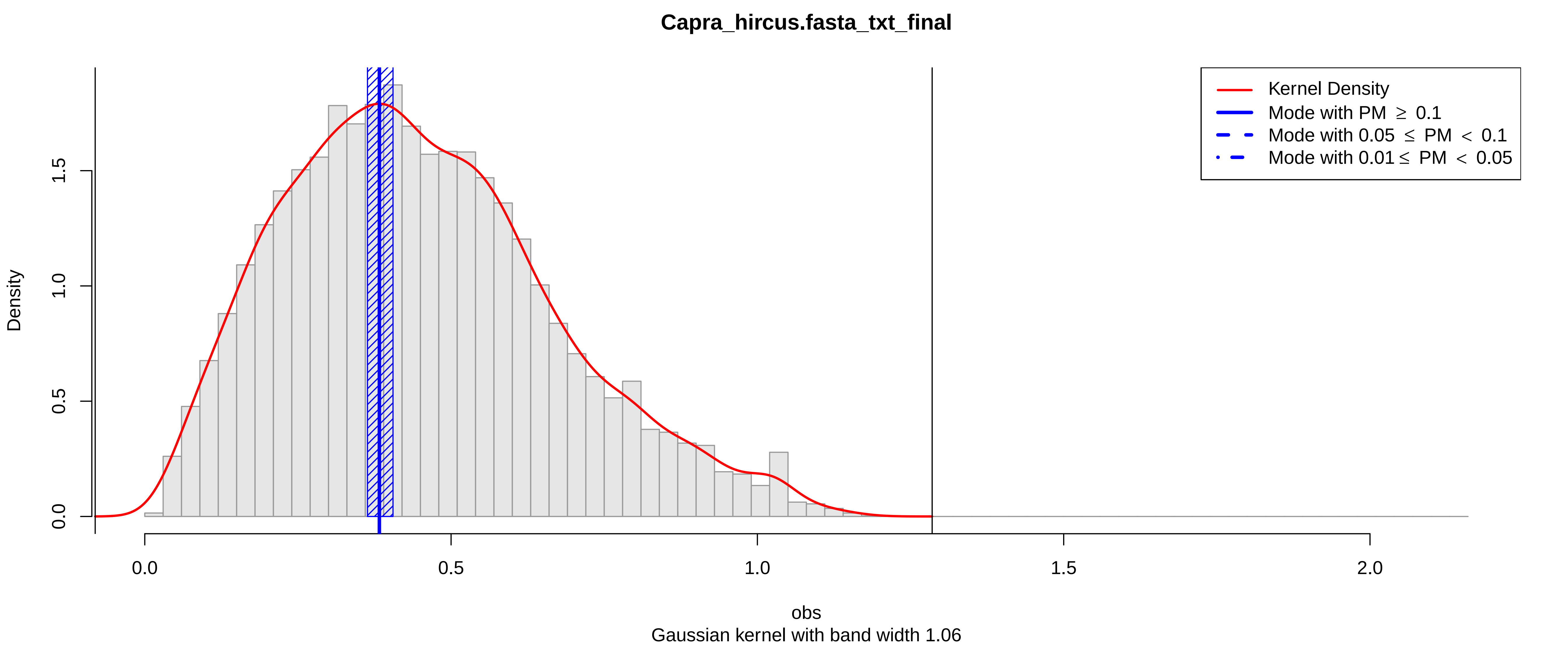

Capra_hircus.fasta_txt_final
Kernel Density
Mode with PM ≥ 0.1
Mode with 0.05 ≤ PM < 0.1
Mode with 0.01 ≤ PM < 0.05
Density
0.0
0.5
1.0
1.5
0.0
0.5
1.0
1.5
2.0
obs
Gaussian kernel with band width 1.06

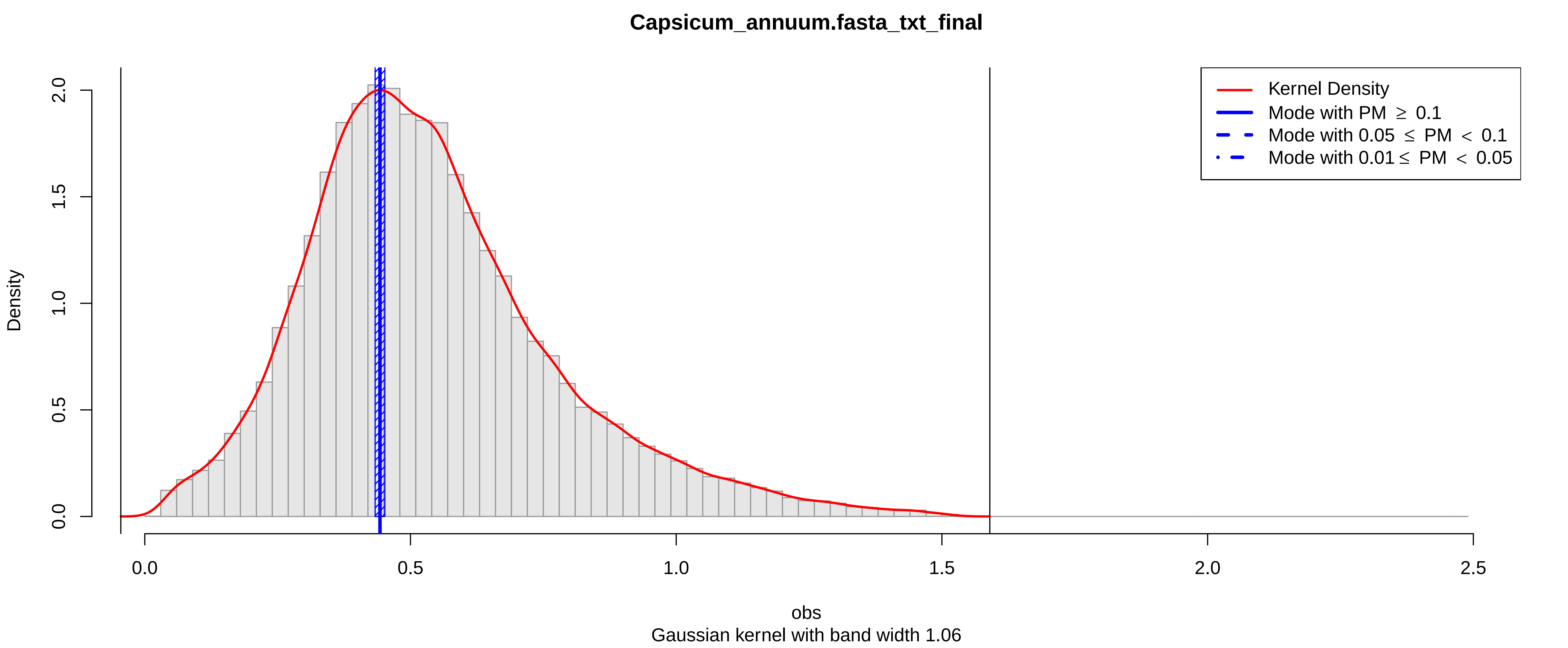
Capsicum_annuum.fasta_txt_final
Kernel Density
Mode with PM ≥ 0.1
Mode with 0.05 ≤ PM < 0.1
Mode with 0.01 ≤ PM < 0.05
Density
0.0
0.5
1.0
1.5
2.0
2.5
obs
Gaussian kernel with band width 1.06

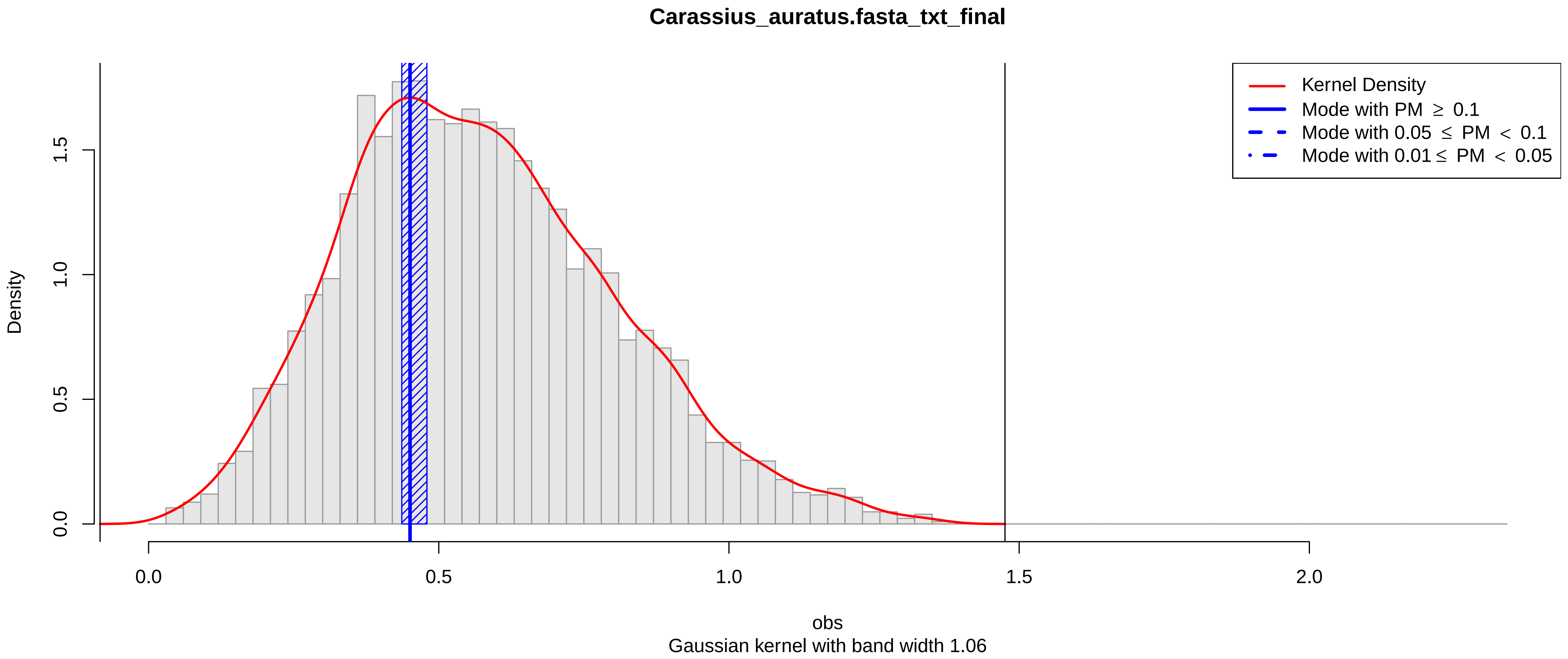
Carassius_auratus.fasta_txt_final
Kernel Density
Mode with PM ≥ 0.1
Mode with 0.05 ≤ PM < 0.1
Mode with 0.01 ≤ PM < 0.05
Density
0.0
0.5
1.0
1.5
0.0
0.5
1.0
1.5
2.0
obs
Gaussian kernel with band width 1.06

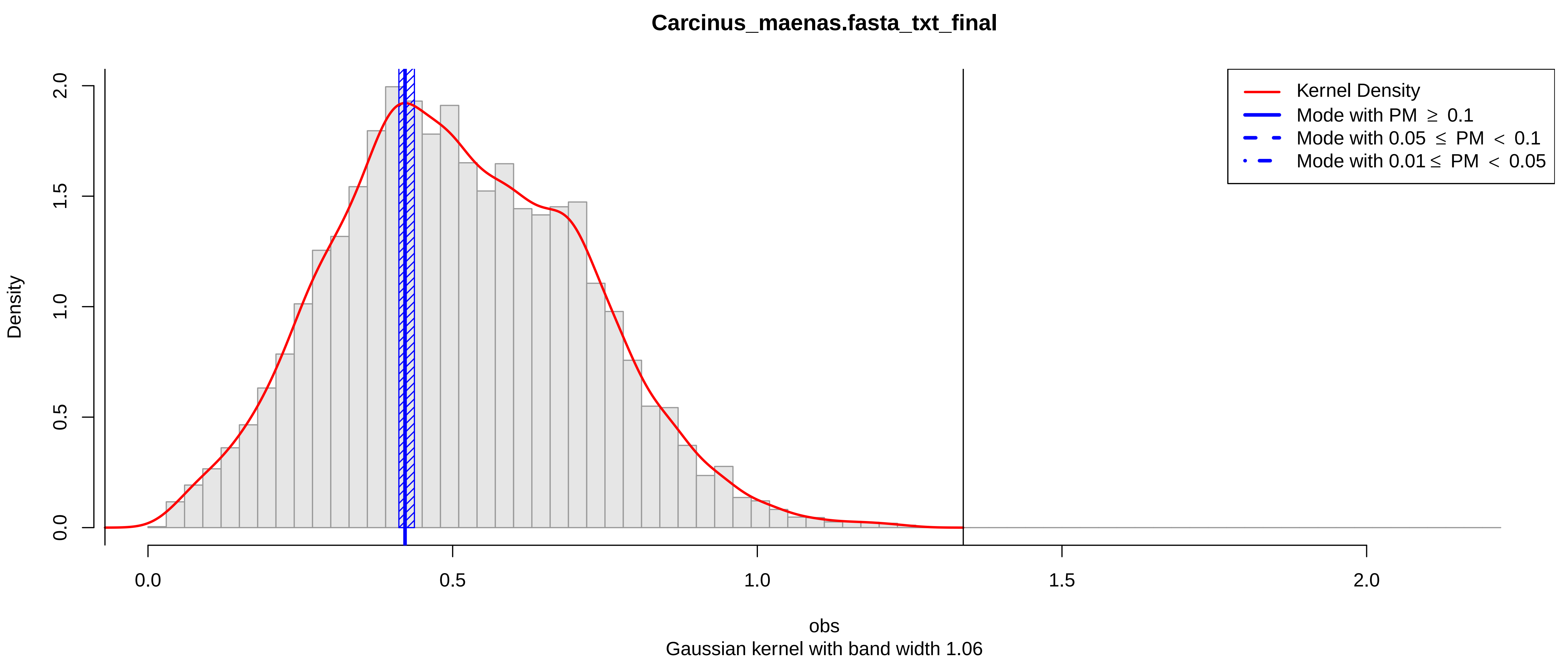

Carcinus_maenas.fasta_txt_final
Kernel Density
Mode with PM ≥ 0.1
Mode with 0.05 ≤ PM < 0.1
Mode with 0.01 ≤ PM < 0.05
Density
0.0
0.5
1.0
1.5
2.0
obs
Gaussian kernel with band width 1.06

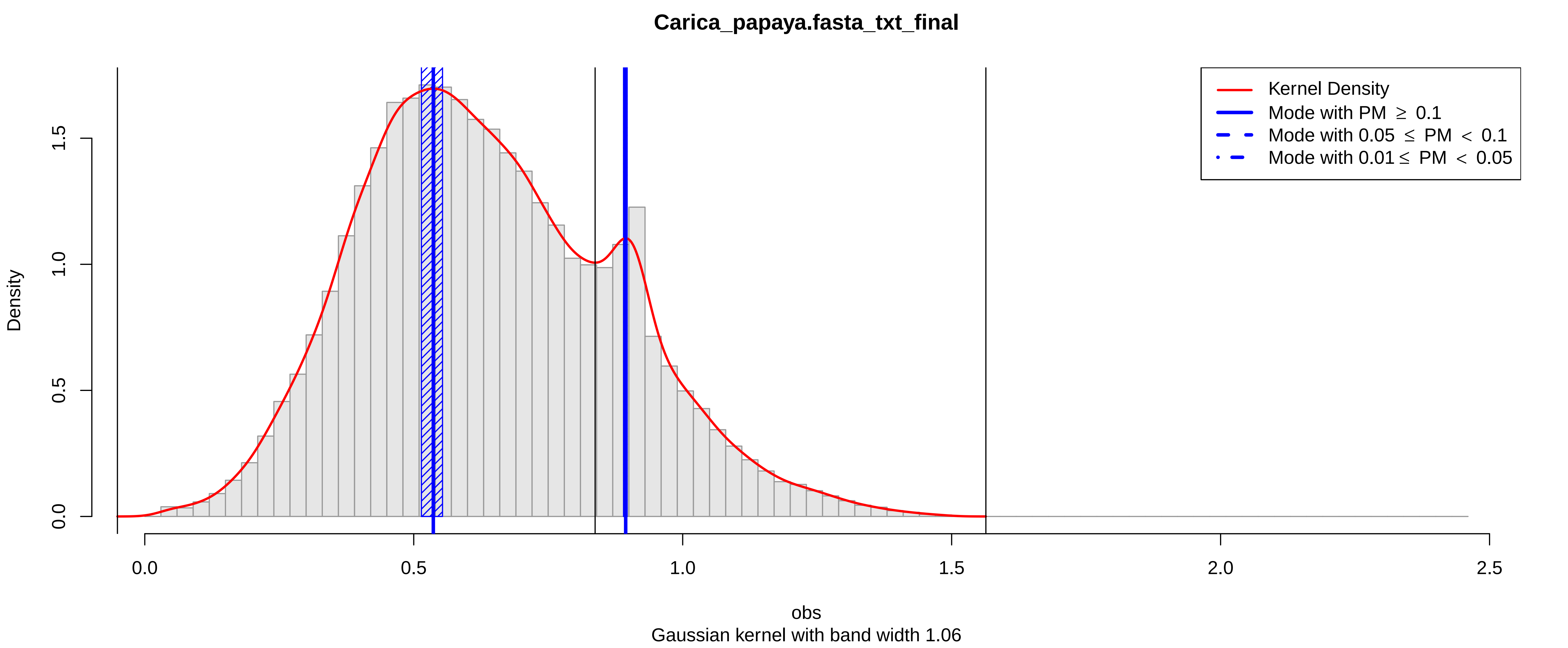
Carica_papaya.fasta_txt_final
Density
0.0
0.5
1.0
1.5
0.0
0.5
1.0
1.5
2.0
2.5
obs
Gaussian kernel with band width 1.06
Kernel Density
Mode with PM ≥ 0.1
Mode with 0.05 ≤ PM < 0.1
Mode with 0.01 ≤ PM < 0.05

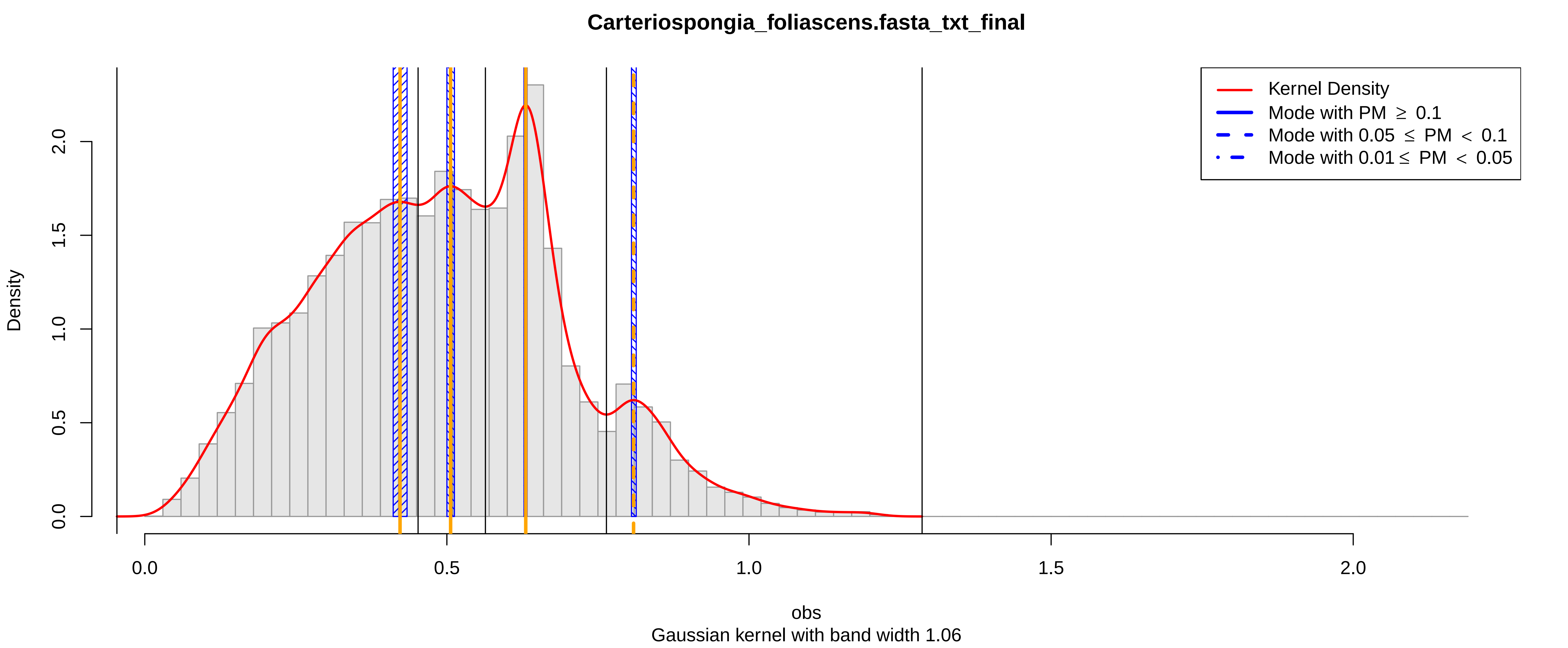

Carteriospongia_foliascens.fasta_txt_final
Kernel Density
Mode with PM ≥ 0.1
Mode with 0.05 ≤ PM < 0.1
Mode with 0.01 ≤ PM < 0.05
Density
0.0
0.5
1.0
1.5
2.0
0.0
0.5
1.0
1.5
2.0
obs
Gaussian kernel with band width 1.06

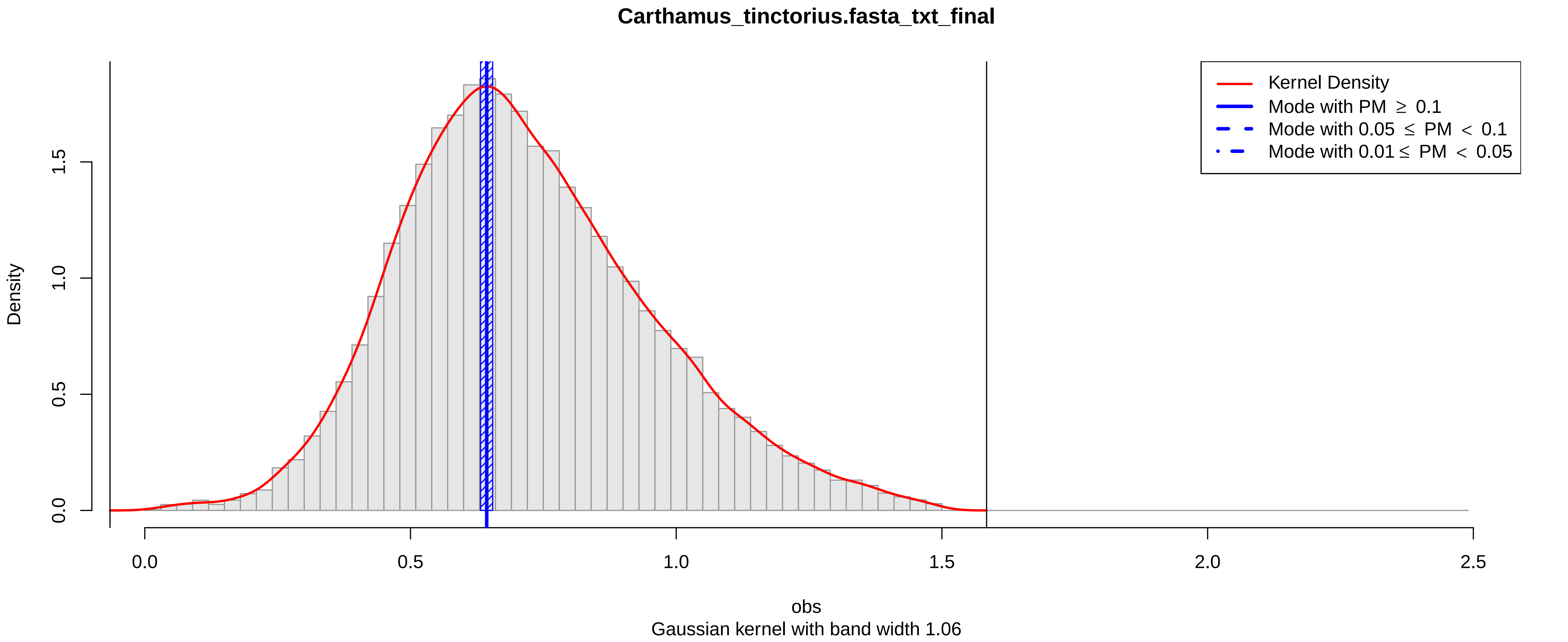
Carthamus_tinctorius.fasta_txt_final
Kernel Density
Mode with PM ≥ 0.1
Mode with 0.05 ≤ PM < 0.1
Mode with 0.01 ≤ PM < 0.05
Density
0.0
0.5
1.0
1.5
0.0
0.5
1.0
1.5
2.0
2.5
obs
Gaussian kernel with band width 1.06

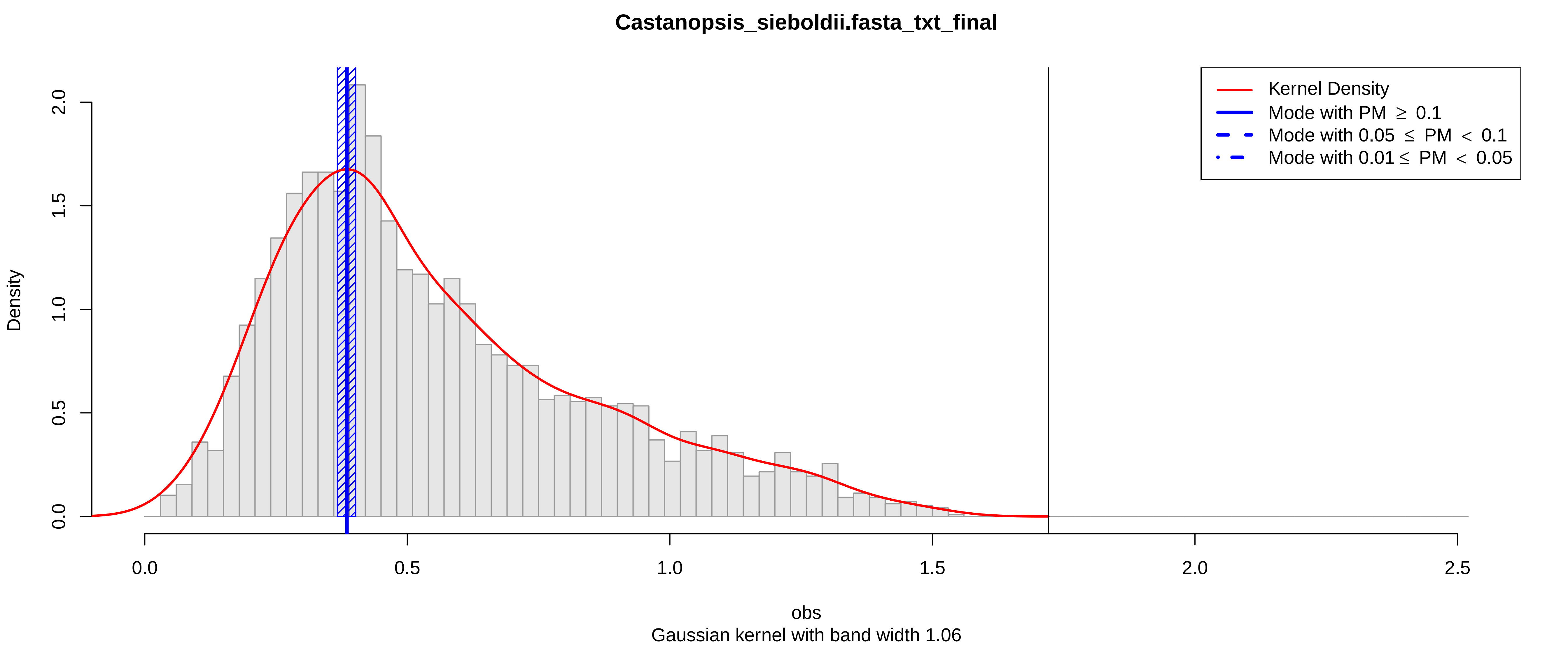

Castanopsis_sieboldii.fasta_txt_final
Kernel Density
Mode with PM ≥ 0.1
Mode with 0.05 ≤ PM < 0.1
Mode with 0.01 ≤ PM < 0.05
Density
0.0
0.5
1.0
1.5
2.0
2.5
obs
Gaussian kernel with band width 1.06

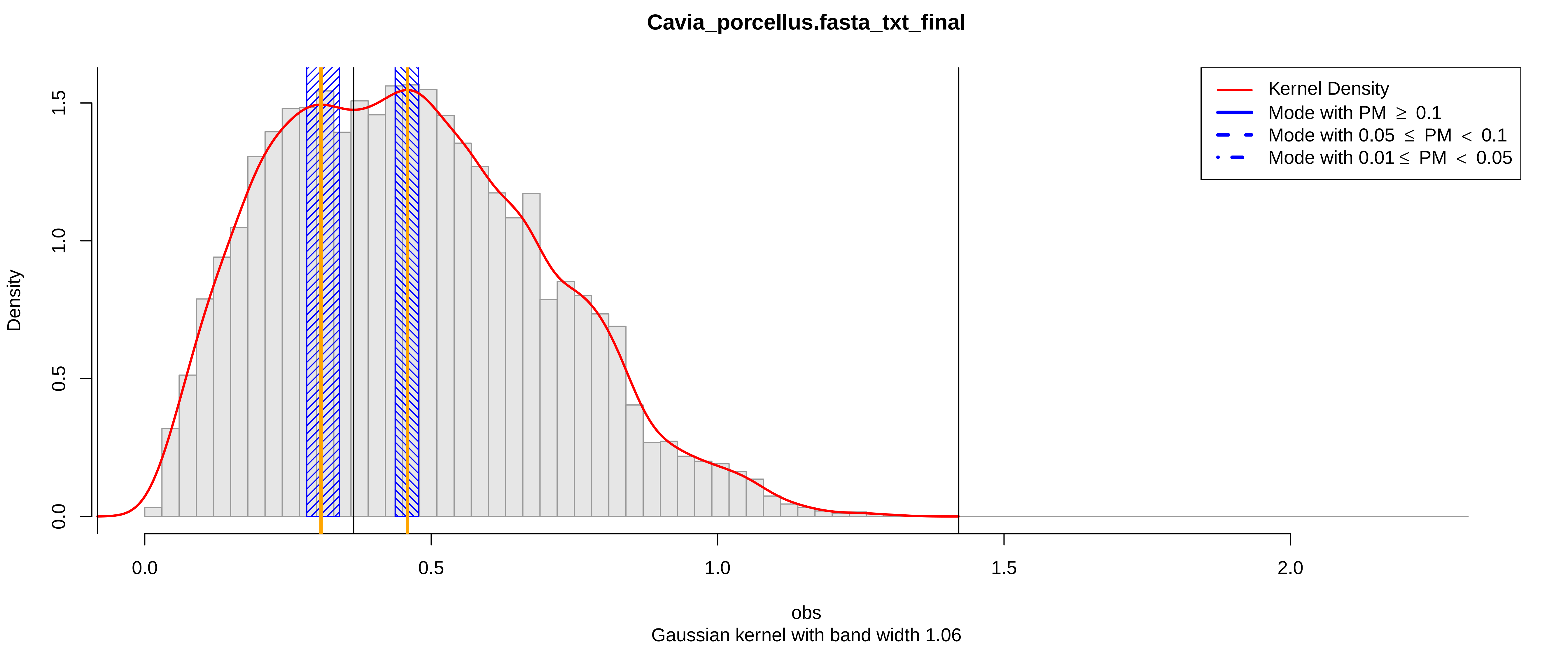
Cavia_porcellus.fasta_txt_final
Kernel Density
Mode with PM ≥ 0.1
Mode with 0.05 ≤ PM < 0.1
Mode with 0.01 ≤ PM < 0.05
Density
0.0
0.5
1.0
1.5
0.0
0.5
1.0
1.5
2.0
obs
Gaussian kernel with band width 1.06

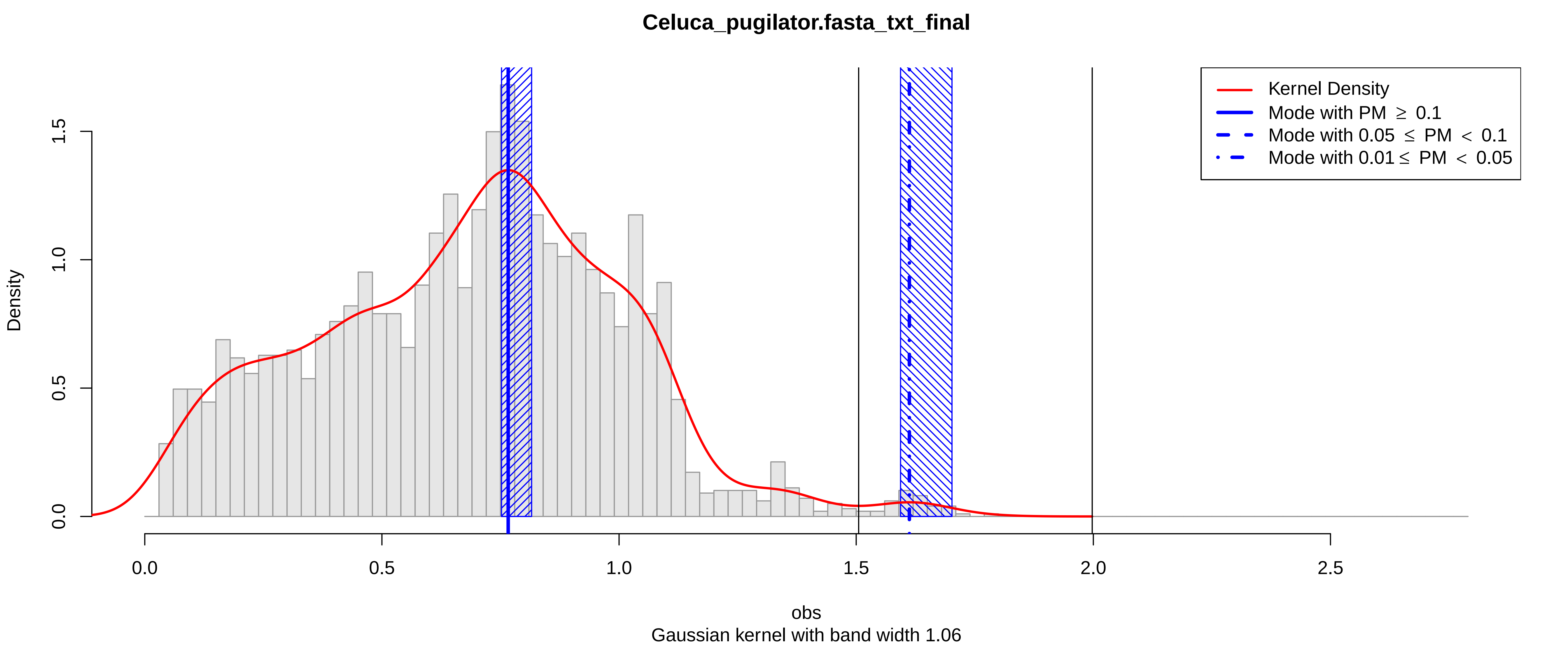
Celuca_pugilator.fasta_txt_final
Kernel Density
Mode with PM ≥ 0.1
Mode with 0.05 ≤ PM < 0.1
Mode with 0.01 ≤ PM < 0.05
Density
0.0
0.5
1.0
1.5
0.0
0.5
1.0
1.5
2.0
2.5
obs
Gaussian kernel with band width 1.06

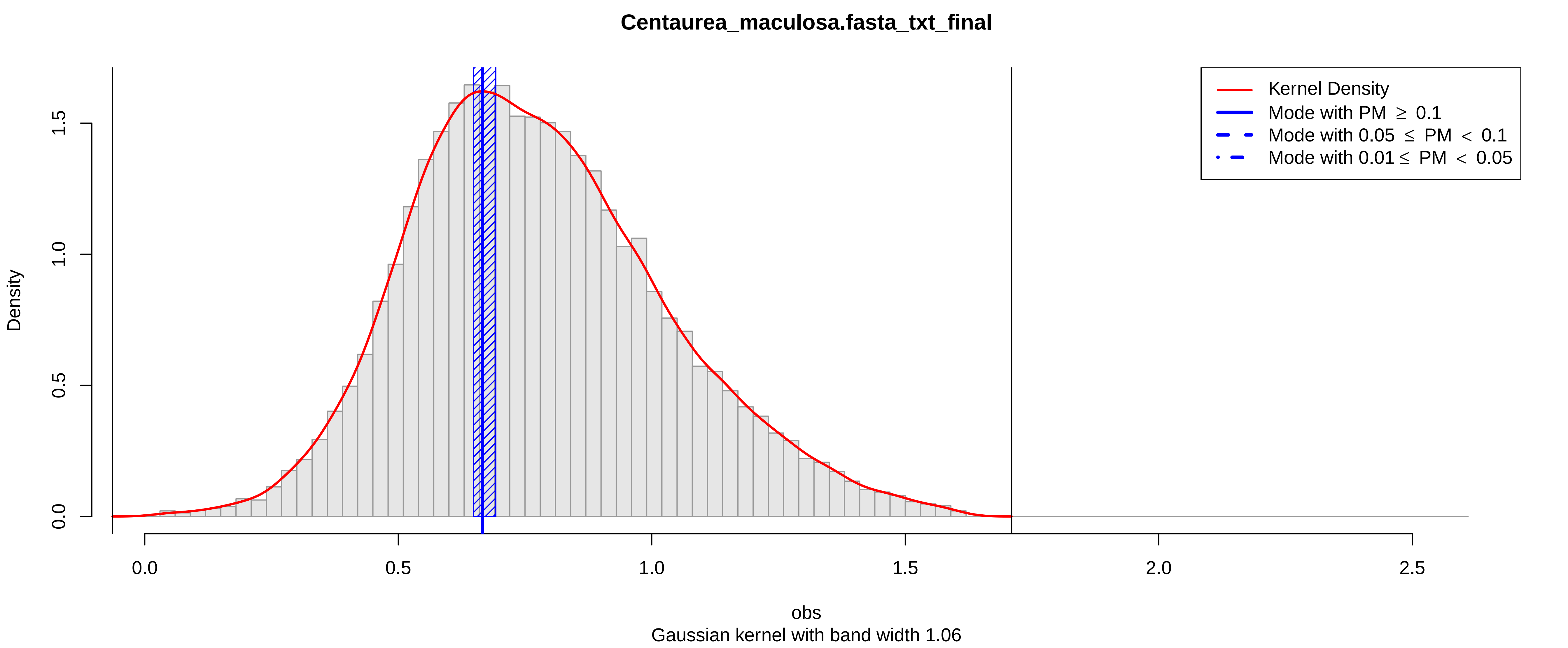
Centaurea_maculosa.fasta_txt_final
Kernel Density
Mode with PM ≥ 0.1
Mode with 0.05 ≤ PM < 0.1
Mode with 0.01 ≤ PM < 0.05
Density
0.0
0.5
1.0
1.5
0.0
0.5
1.0
1.5
2.0
2.5
obs
Gaussian kernel with band width 1.06

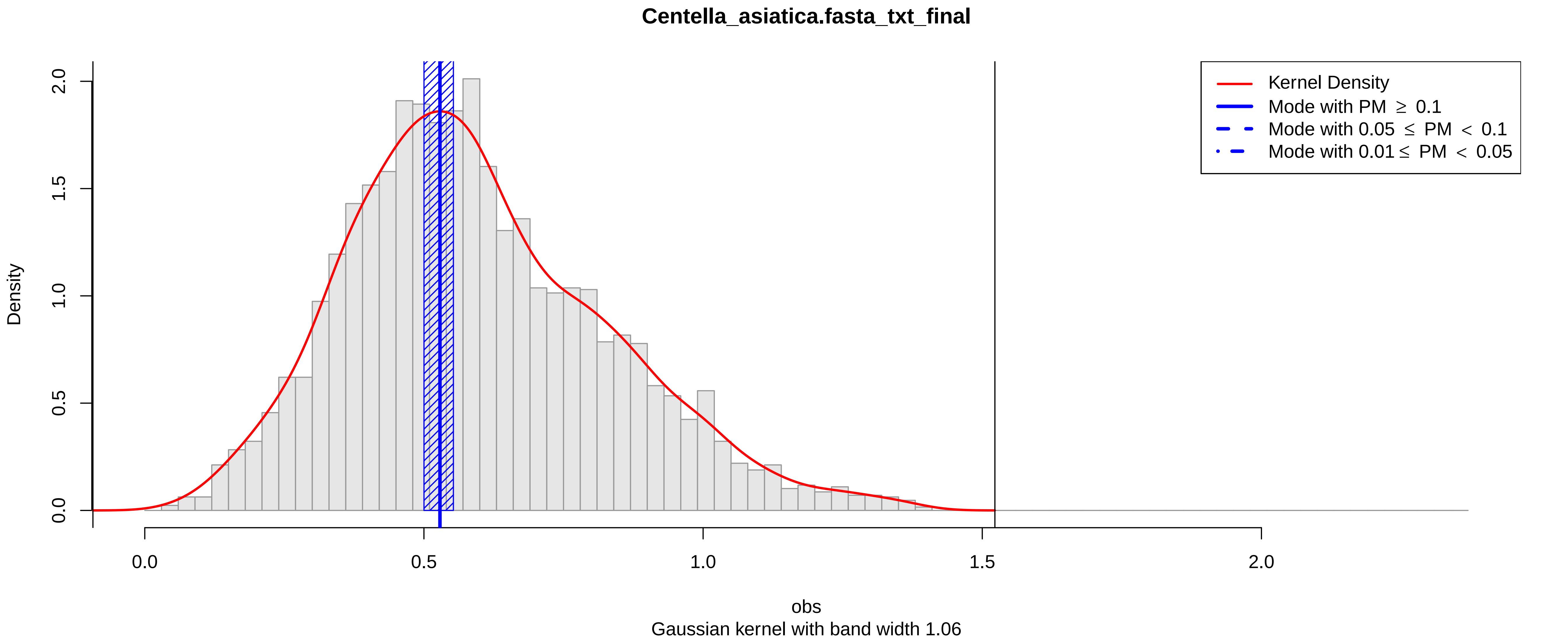

Centella_asiatica.fasta_txt_final
Density
0.0
0.5
1.0
1.5
2.0
0.0
0.5
1.0
1.5
2.0
obs
Gaussian kernel with band width 1.06
Kernel Density
Mode with PM ≥ 0.1
Mode with 0.05 ≤ PM < 0.1
Mode with 0.01 ≤ PM < 0.05

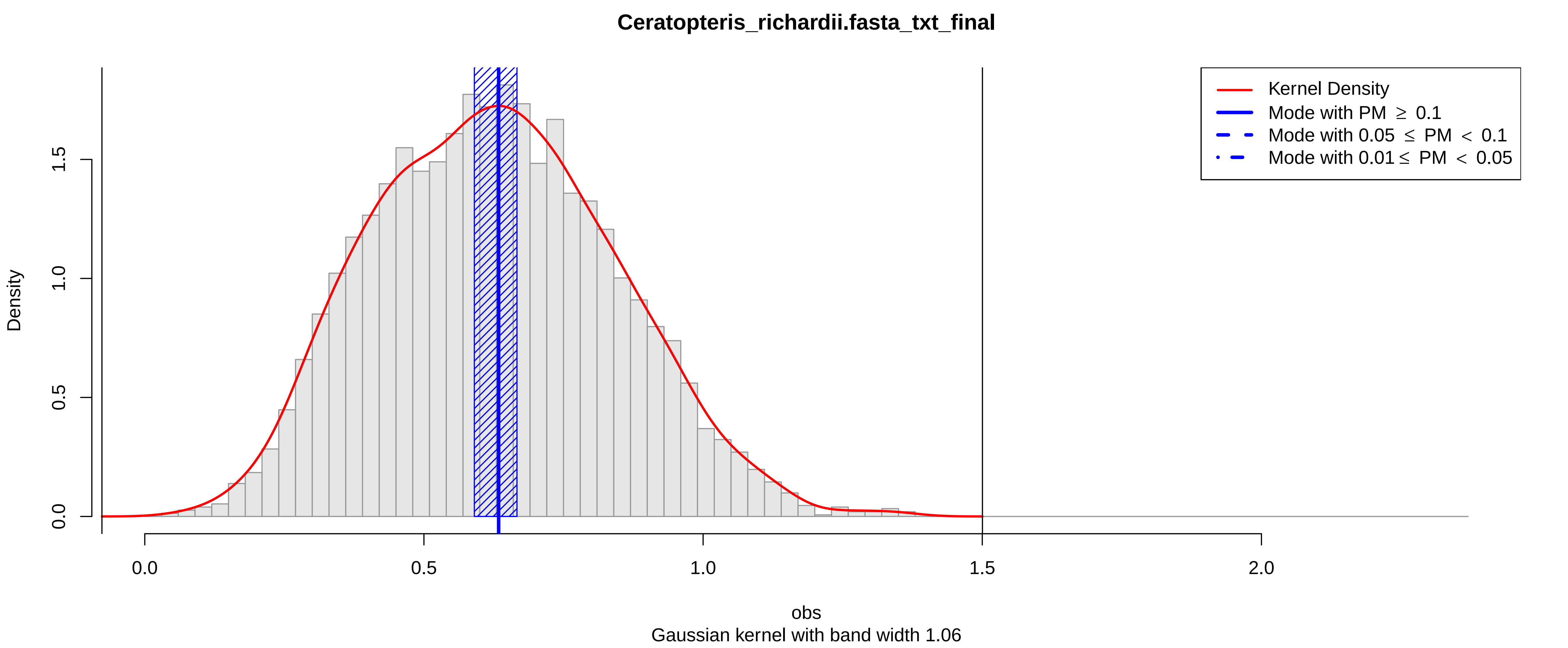

Ceratopteris_richardii.fasta_txt_final
Kernel Density
Mode with PM ≥ 0.1
Mode with 0.05 ≤ PM < 0.1
Mode with 0.01 ≤ PM < 0.05
Density
0.0
0.5
1.0
1.5
0.0
0.5
1.0
1.5
2.0
obs
Gaussian kernel with band width 1.06

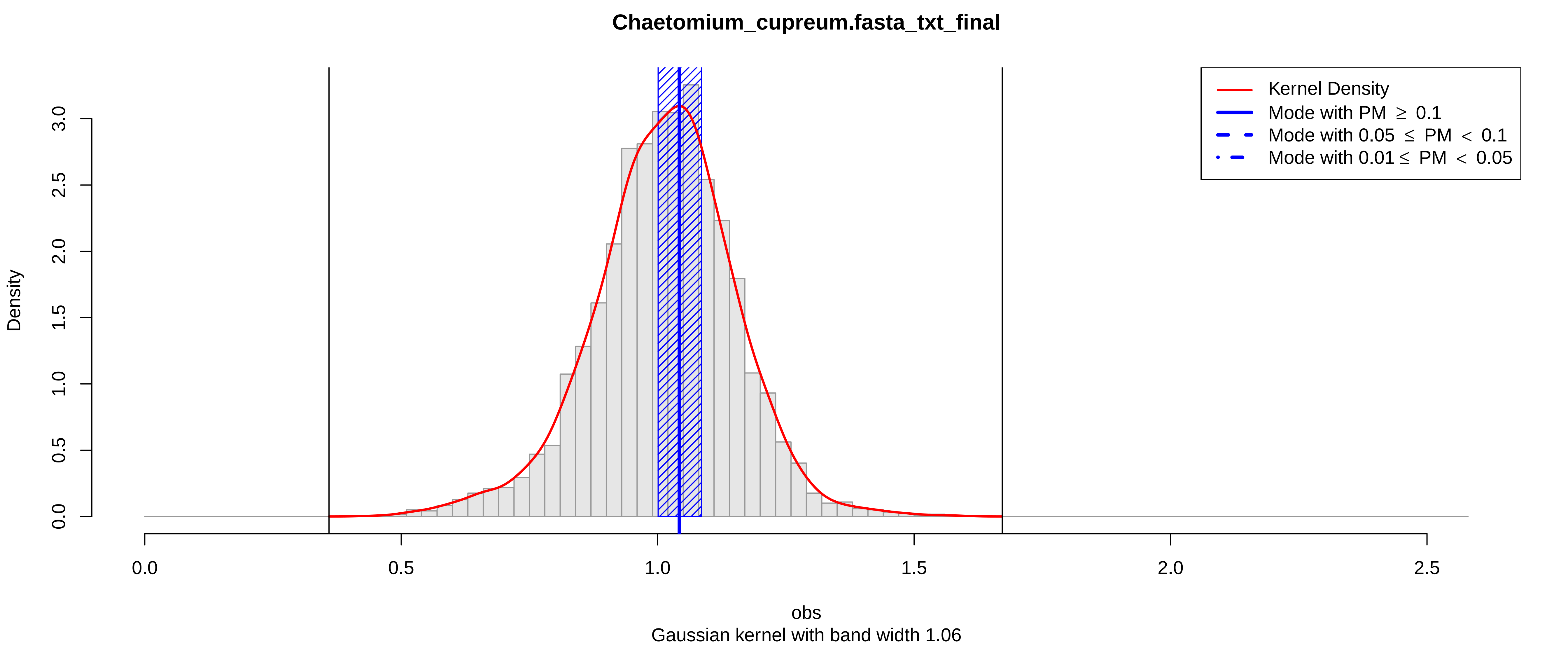
Chaetomium_cupreum.fasta_txt_final
Kernel Density
Mode with PM ≥ 0.1
Mode with 0.05 ≤ PM < 0.1
Mode with 0.01 ≤ PM < 0.05
Density
0.0
0.5
1.0
1.5
2.0
2.5
3.0
0.0
0.5
1.0
1.5
2.0
2.5
obs
Gaussian kernel with band width 1.06

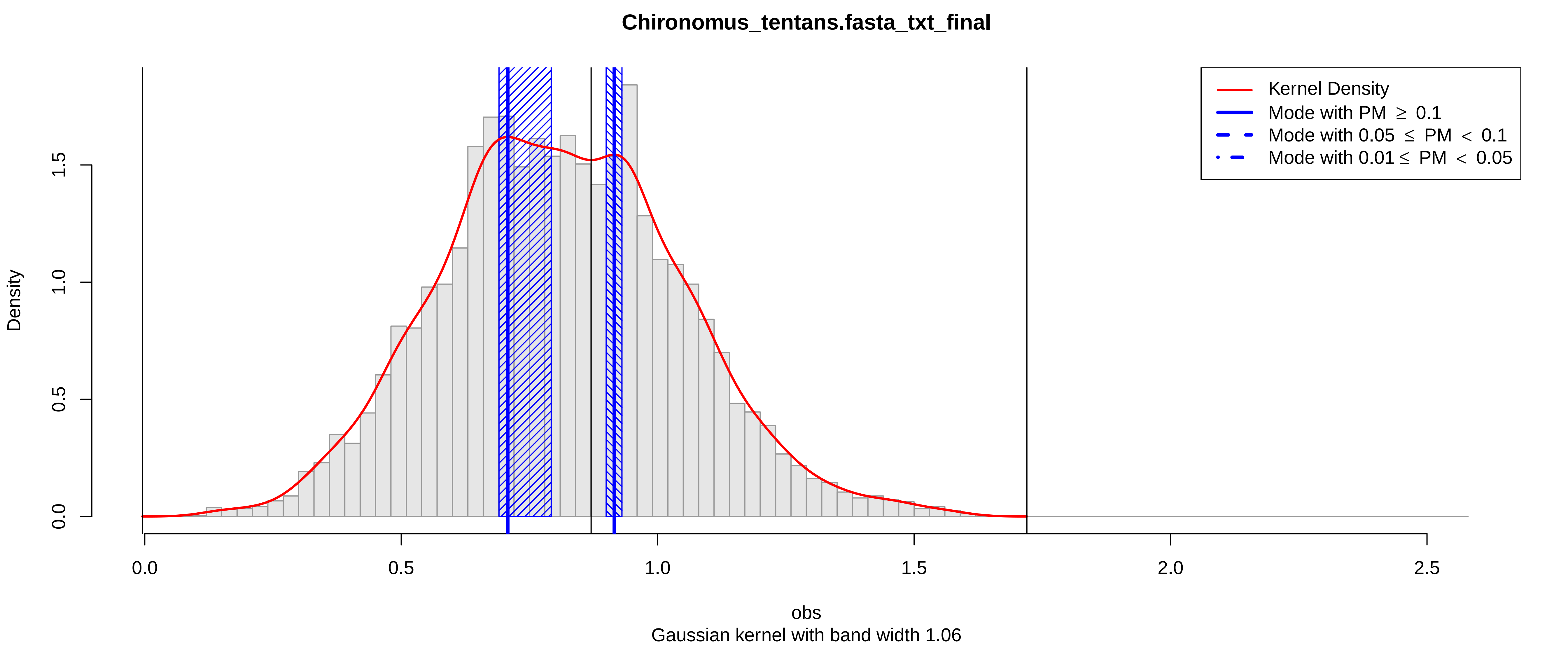

Chironomus_tentans.fasta_txt_final
Kernel Density
Mode with PM ≥ 0.1
Mode with 0.05 ≤ PM < 0.1
Mode with 0.01 ≤ PM < 0.05
Density
0.0
0.5
1.0
1.5
0.0
0.5
1.0
1.5
2.0
2.5
obs
Gaussian kernel with band width 1.06

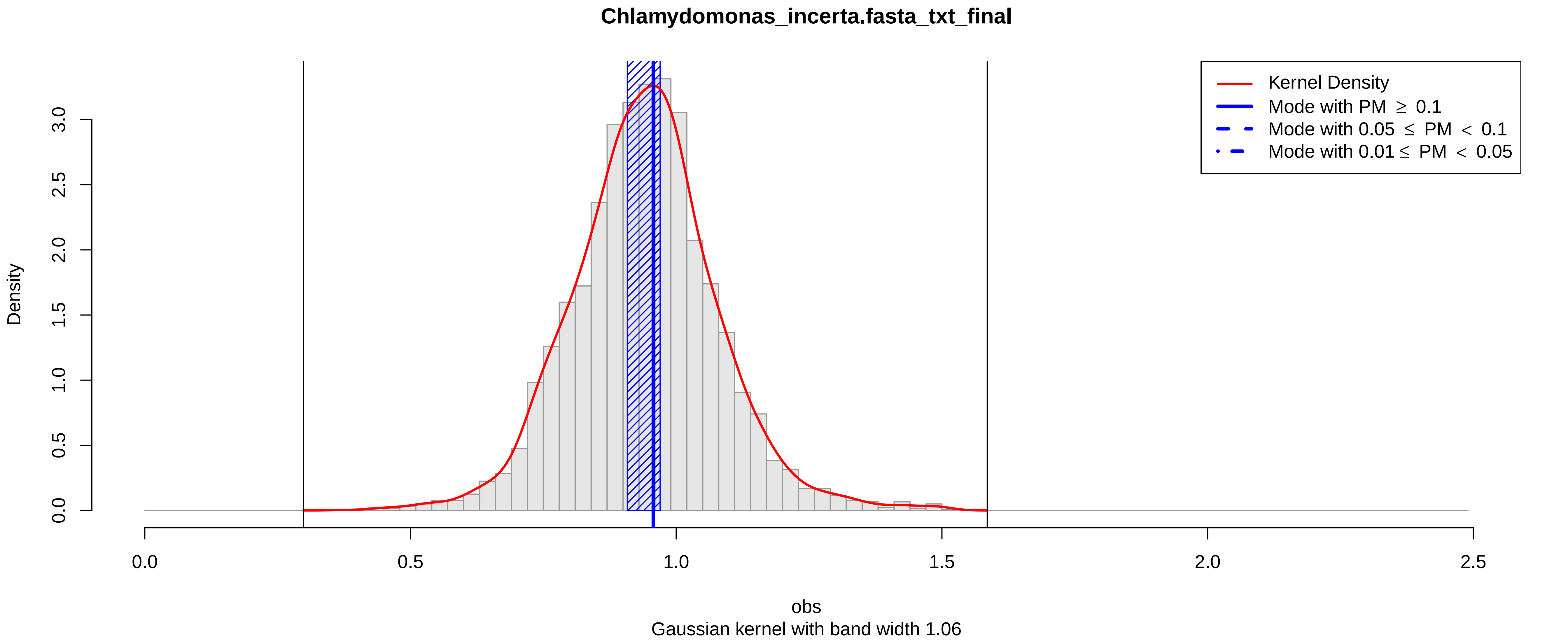
Chlamydomonas_incerta.fasta_txt_final
Kernel Density
Mode with PM ≥ 0.1
Mode with 0.05 ≤ PM < 0.1
Mode with 0.01 ≤ PM < 0.05
Density
0.0
0.5
1.0
1.5
2.0
2.5
3.0
0.0
0.5
1.0
1.5
2.0
2.5
obs
Gaussian kernel with band width 1.06

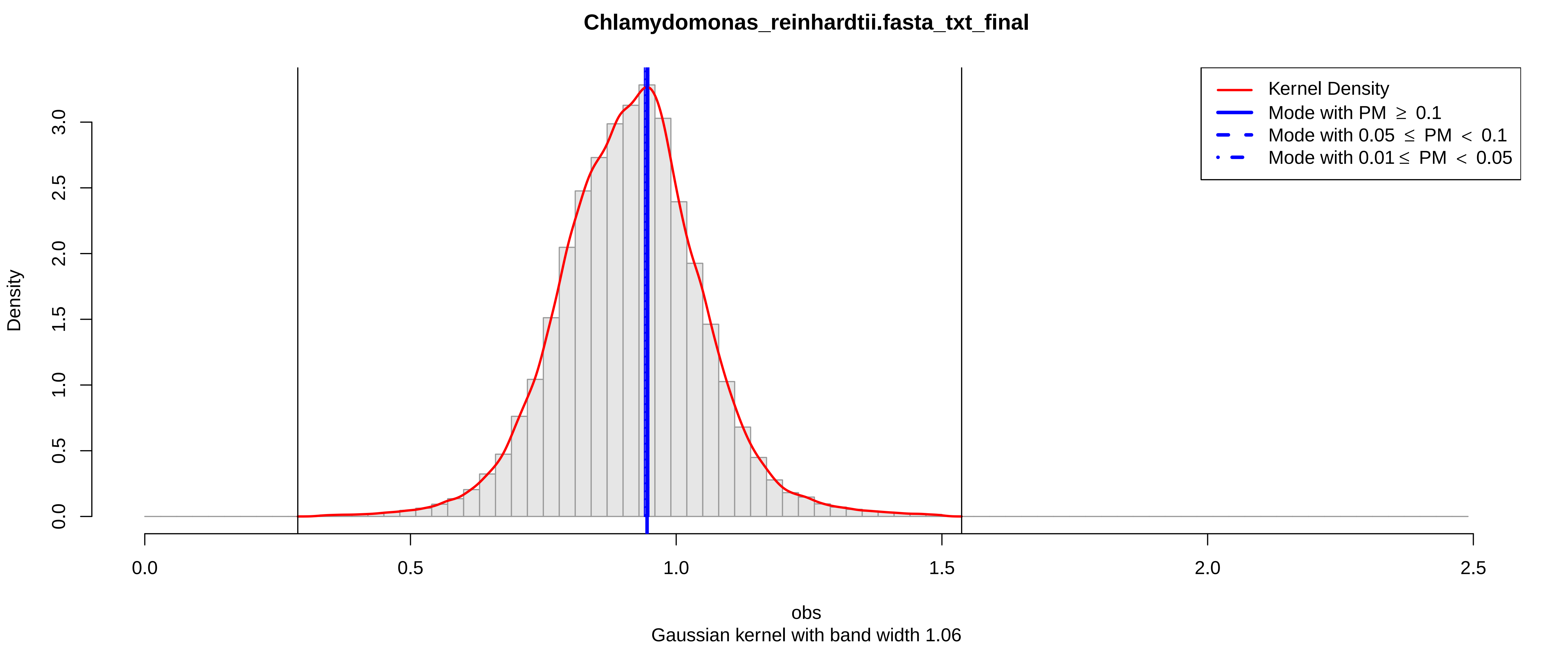
Chlamydomonas_reinhardtii.fasta_txt_final
Kernel Density
Mode with PM ≥ 0.1
Mode with 0.05 ≤ PM < 0.1
Mode with 0.01 ≤ PM < 0.05
Density
0.0
0.5
1.0
1.5
2.0
2.5
3.0
0.0
0.5
1.0
1.5
2.0
2.5
obs
Gaussian kernel with band width 1.06

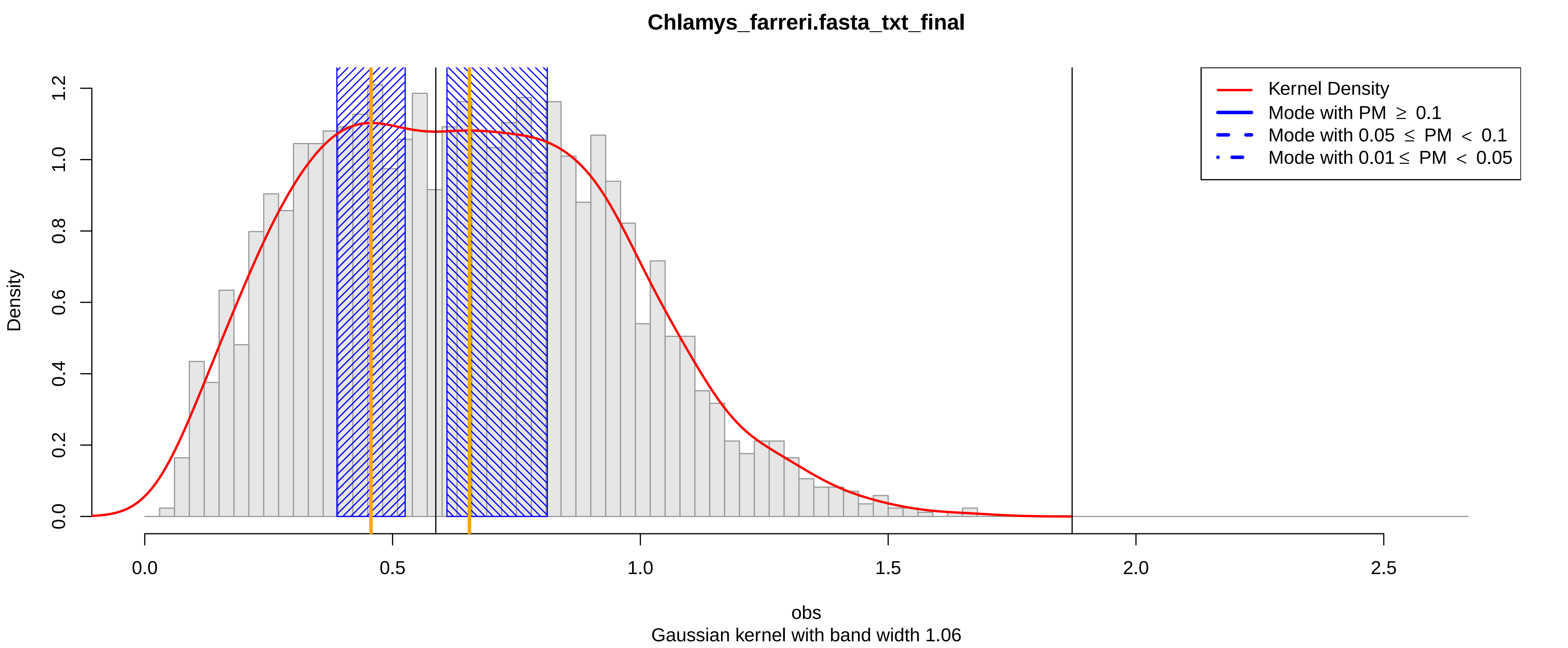
Chlamys_farreri.fasta_txt_final
Density
0.0
0.2
0.4
0.6
0.8
1.0
1.2
0.0
0.5
1.0
1.5
2.0
2.5
obs
Gaussian kernel with band width 1.06
Kernel Density
Mode with PM ≥ 0.1
Mode with 0.05 ≤ PM < 0.1
Mode with 0.01 ≤ PM < 0.05

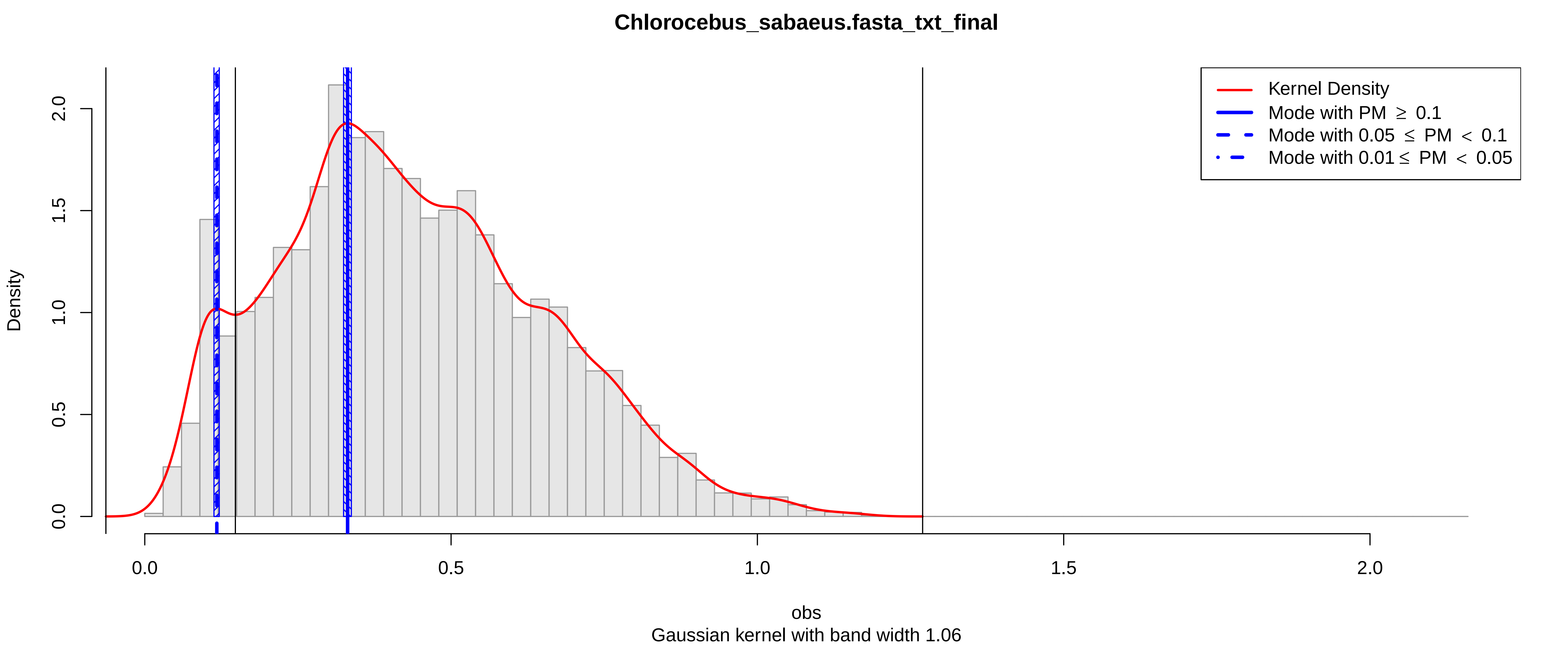

Chlorocebus_sabaeus.fasta_txt_final
Kernel Density
Mode with PM ≥ 0.1
Mode with 0.05 ≤ PM < 0.1
Mode with 0.01 ≤ PM < 0.05
Density
0.0
0.5
1.0
1.5
2.0
0.0
0.5
1.0
1.5
2.0
obs
Gaussian kernel with band width 1.06

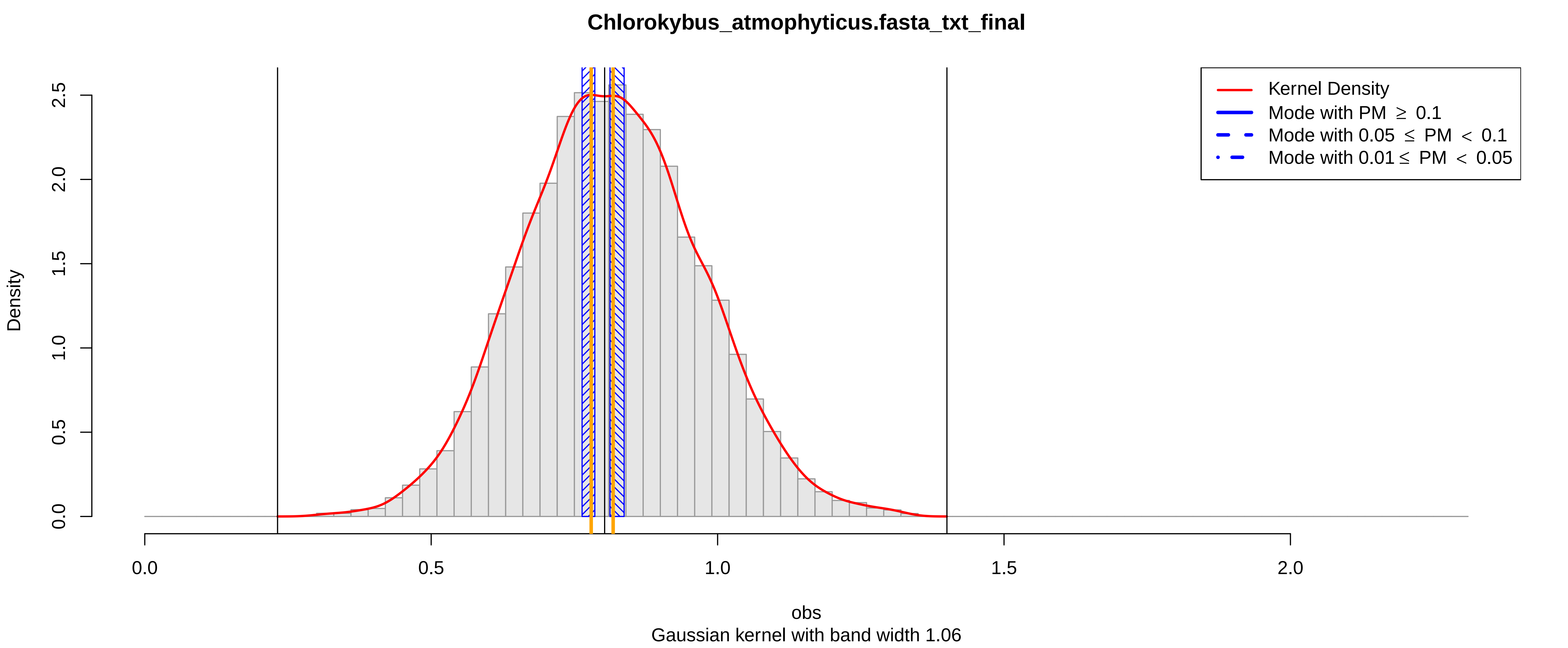
Chlorokybus_atmophyticus.fasta_txt_final
Density
0.0
0.5
1.0
1.5
2.0
2.5
Kernel Density
Mode with PM ≥ 0.1
Mode with 0.05 ≤ PM < 0.1
Mode with 0.01 ≤ PM < 0.05
0.0
0.5
1.0
1.5
2.0
obs
Gaussian kernel with band width 1.06

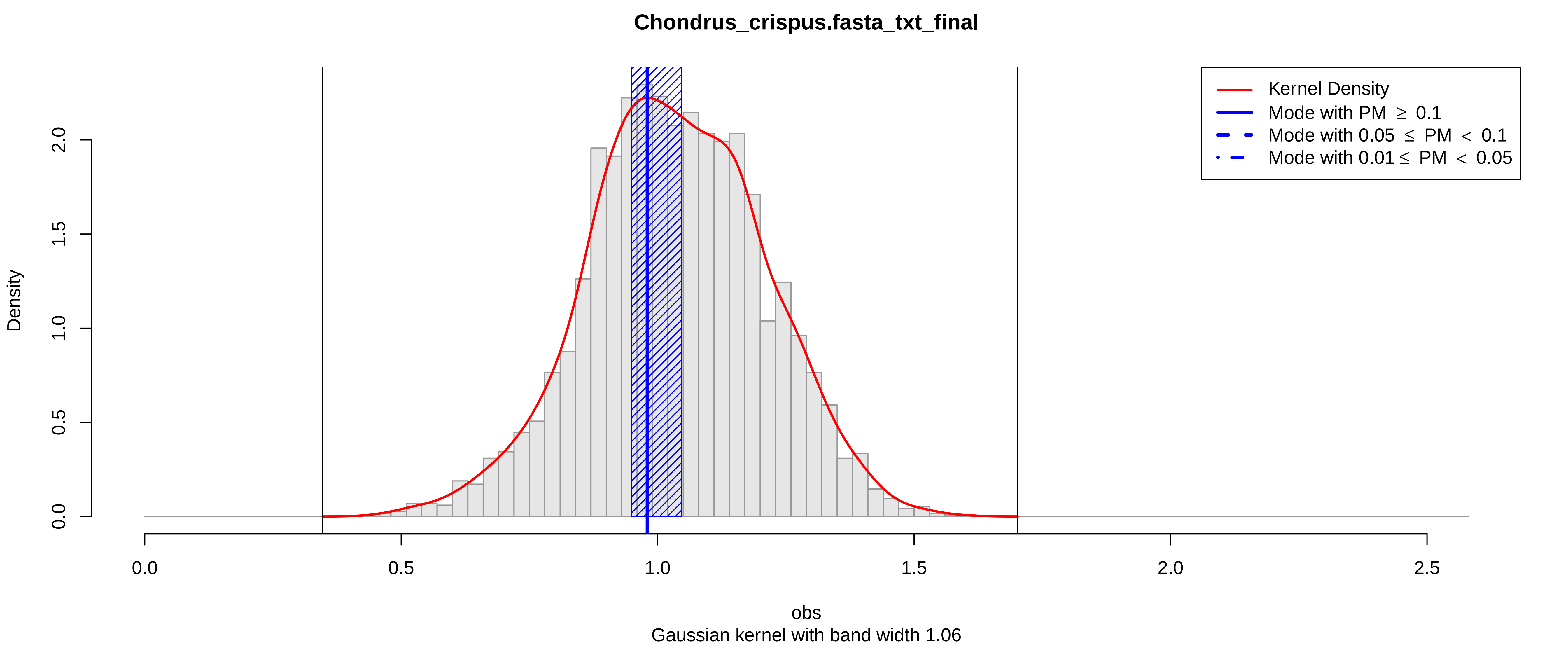
Chondrus_crispus.fasta_txt_final
Kernel Density
Mode with PM ≥ 0.1
Mode with 0.05 ≤ PM < 0.1
Mode with 0.01 ≤ PM < 0.05
Density
0.0
0.5
1.0
1.5
2.0
2.5
obs
Gaussian kernel with band width 1.06

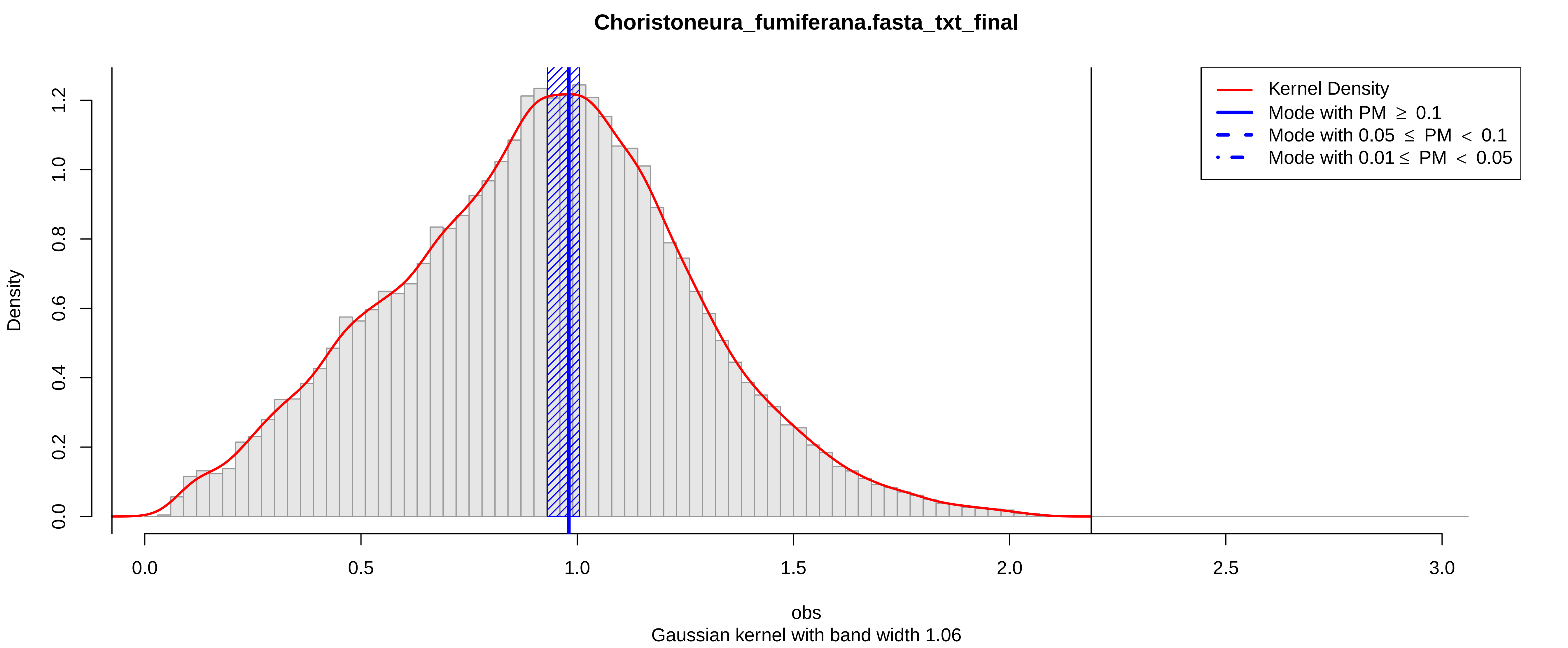

Choristoneura_fumiferana.fasta_txt_final
Kernel Density
Mode with PM ≥ 0.1
Mode with 0.05 ≤ PM < 0.1
Mode with 0.01 ≤ PM < 0.05
Density
0.0
0.2
0.4
0.6
0.8
1.0
1.2
0.0
0.5
1.0
1.5
2.0
2.5
3.0
obs
Gaussian kernel with band width 1.06

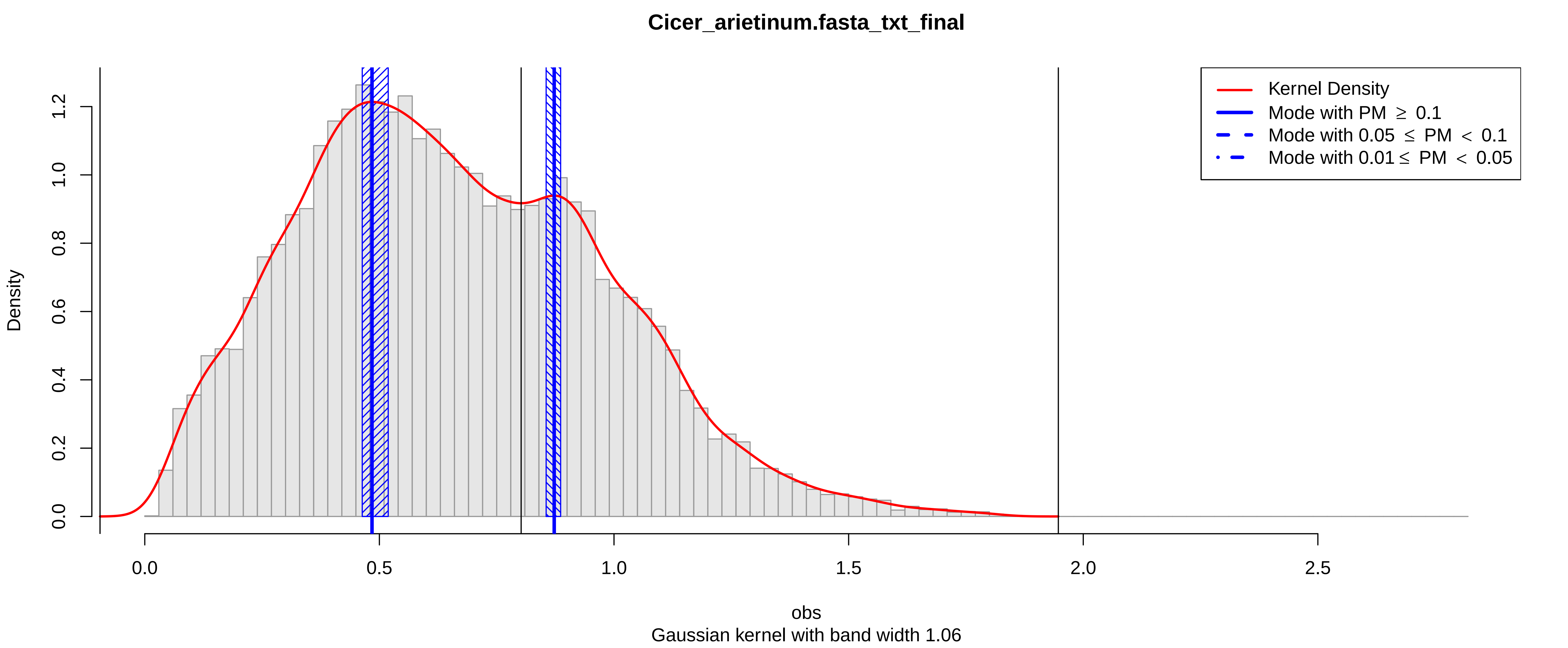
Cicer_arietinum.fasta_txt_final
Kernel Density
Mode with PM ≥ 0.1
Mode with 0.05 ≤ PM < 0.1
Mode with 0.01 ≤ PM < 0.05
Density
0.0
0.2
0.4
0.6
0.8
1.0
1.2
0.0
0.5
1.0
1.5
2.0
2.5
obs
Gaussian kernel with band width 1.06

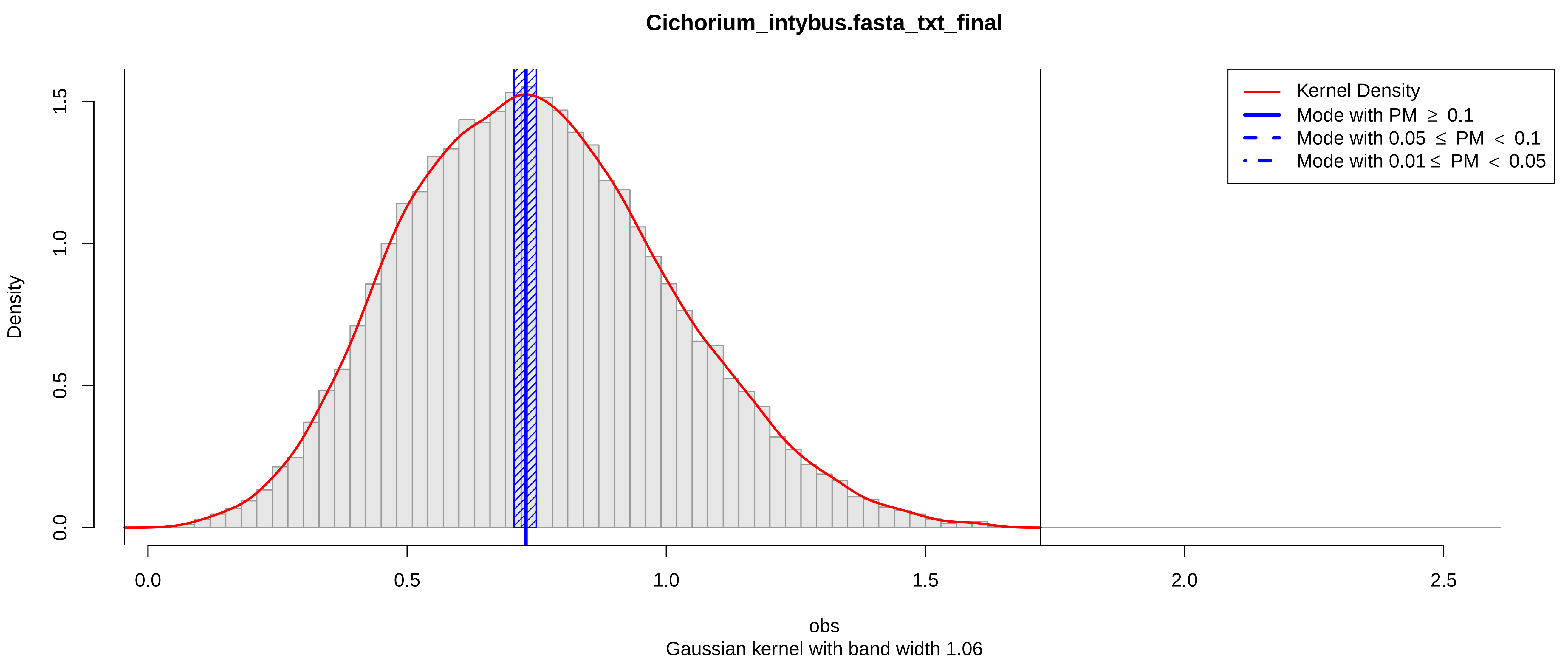

Cichorium_intybus.fasta_txt_final
Kernel Density
Mode with PM ≥ 0.1
Mode with 0.05 ≤ PM < 0.1
Mode with 0.01 ≤ PM < 0.05
Density
0.0
0.5
1.0
1.5
0.0
0.5
1.0
1.5
2.0
2.5
obs
Gaussian kernel with band width 1.06

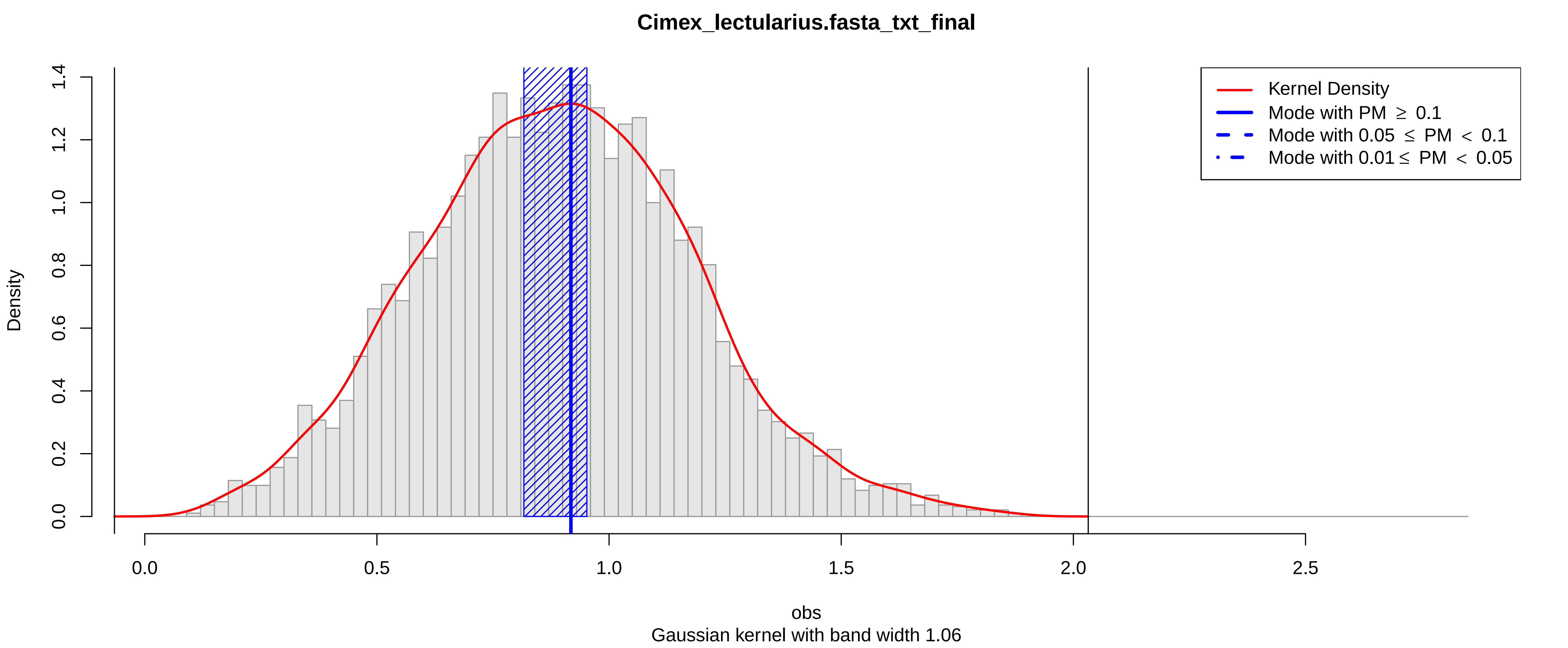

Cimex_lectularius.fasta_txt_final
Kernel Density
Mode with PM ≥ 0.1
Mode with 0.05 ≤ PM < 0.1
Mode with 0.01 ≤ PM < 0.05
Density
obs
Gaussian kernel with band width 1.06

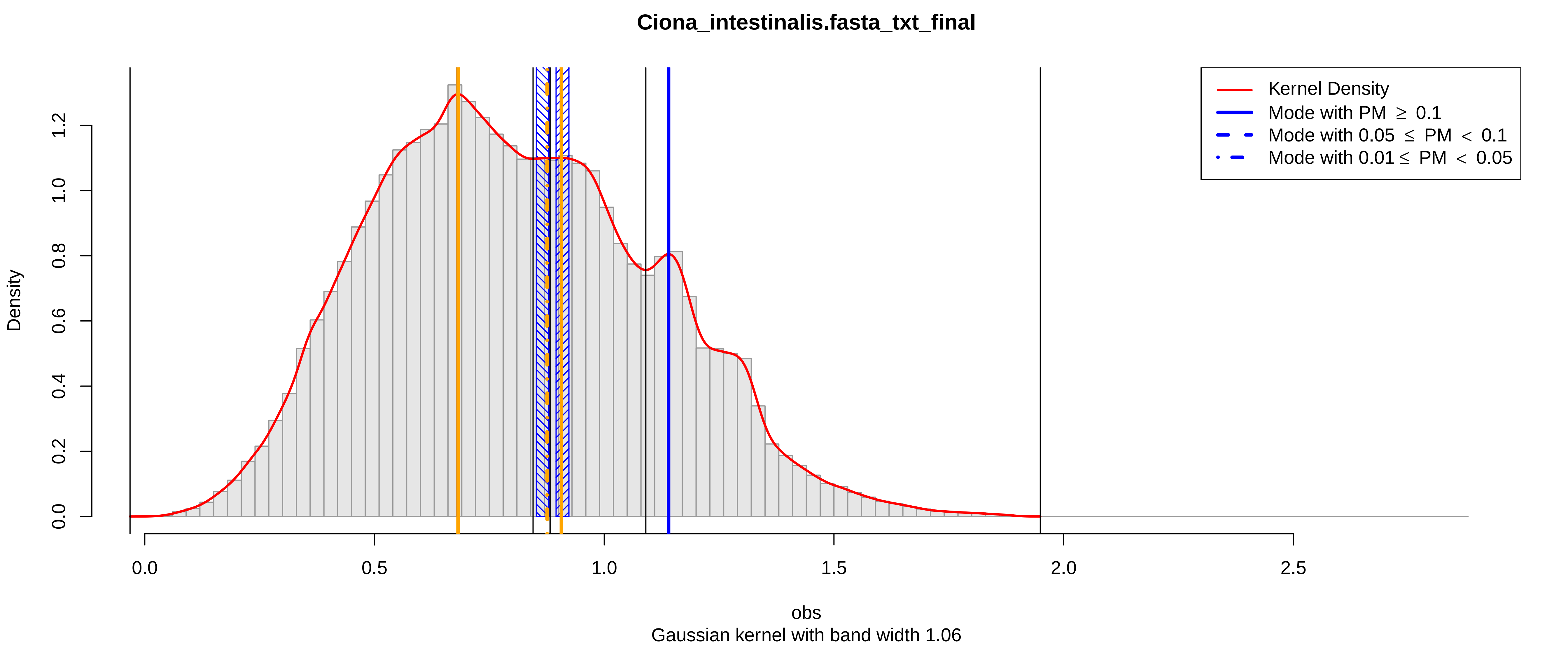

Ciona_intestinalis.fasta_txt_final
Kernel Density
Mode with PM ≥ 0.1
Mode with 0.05 ≤ PM < 0.1
Mode with 0.01 ≤ PM < 0.05
Density
0.0
0.2
0.4
0.6
0.8
1.0
1.2
0.0
0.5
1.0
1.5
2.0
2.5
obs
Gaussian kernel with band width 1.06

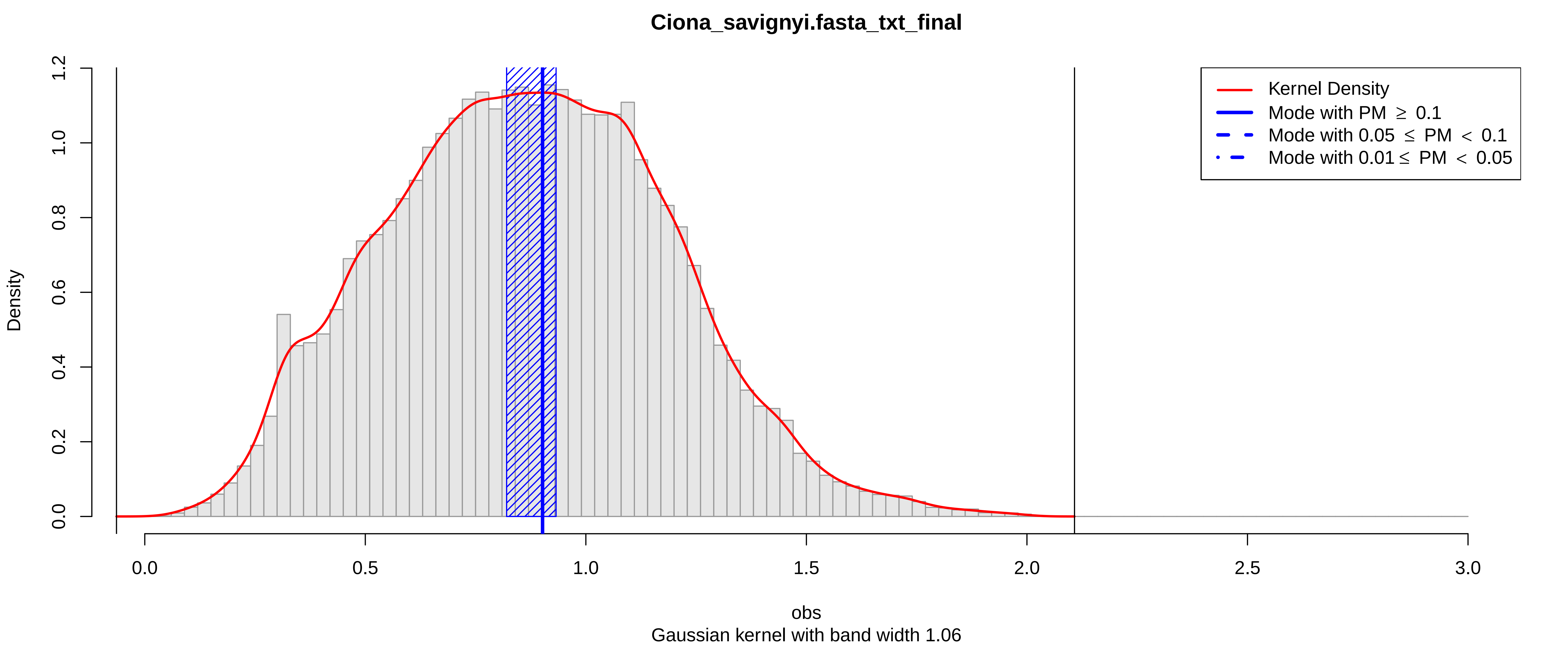

Ciona_savignyi.fasta_txt_final
Density
0.0
0.2
0.4
0.6
0.8
1.0
1.2
0.0
0.5
1.0
1.5
2.0
2.5
3.0
obs
Gaussian kernel with band width 1.06
Kernel Density
Mode with PM ≥ 0.1
Mode with 0.05 ≤ PM < 0.1
Mode with 0.01 ≤ PM < 0.05

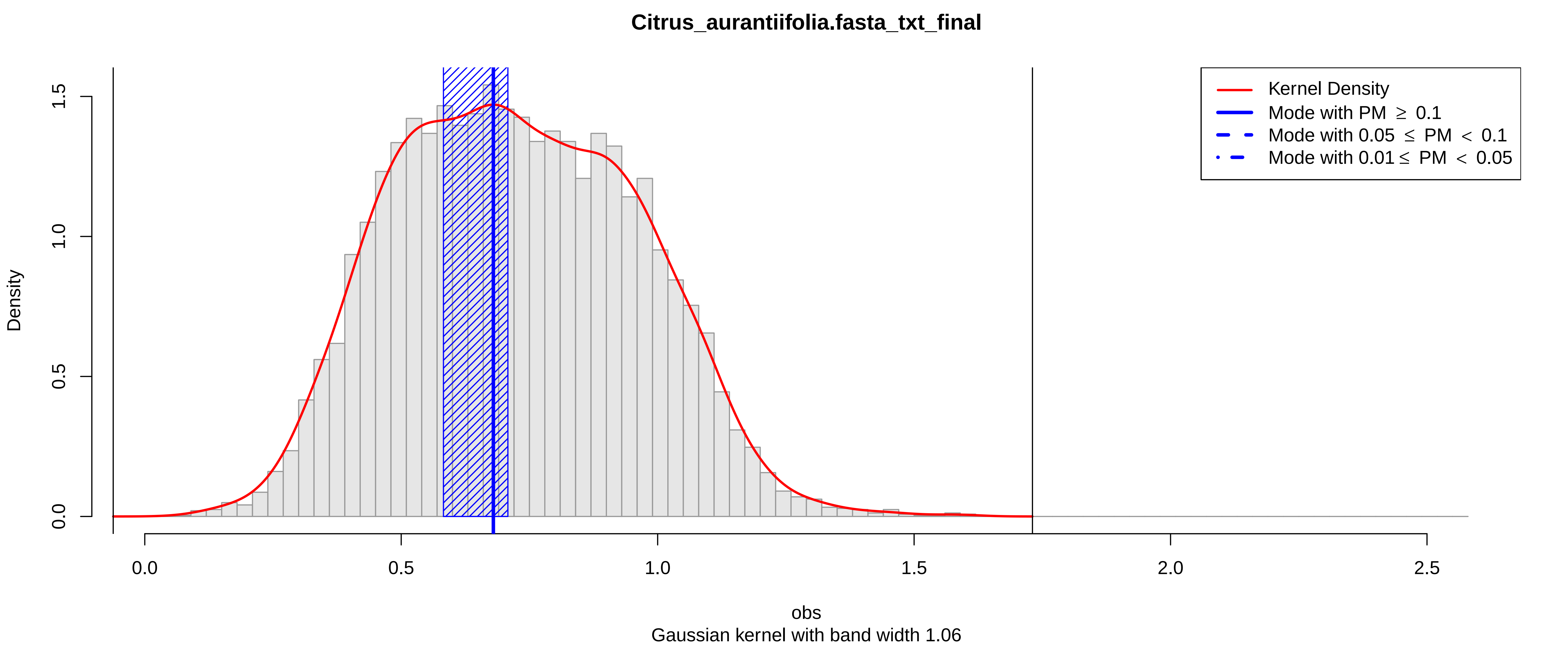

Citrus_aurantiifolia.fasta_txt_final
Kernel Density
Mode with PM ≥ 0.1
Mode with 0.05 ≤ PM < 0.1
Mode with 0.01 ≤ PM < 0.05
Density
0.0
0.5
1.0
1.5
2.0
2.5
obs
Gaussian kernel with band width 1.06

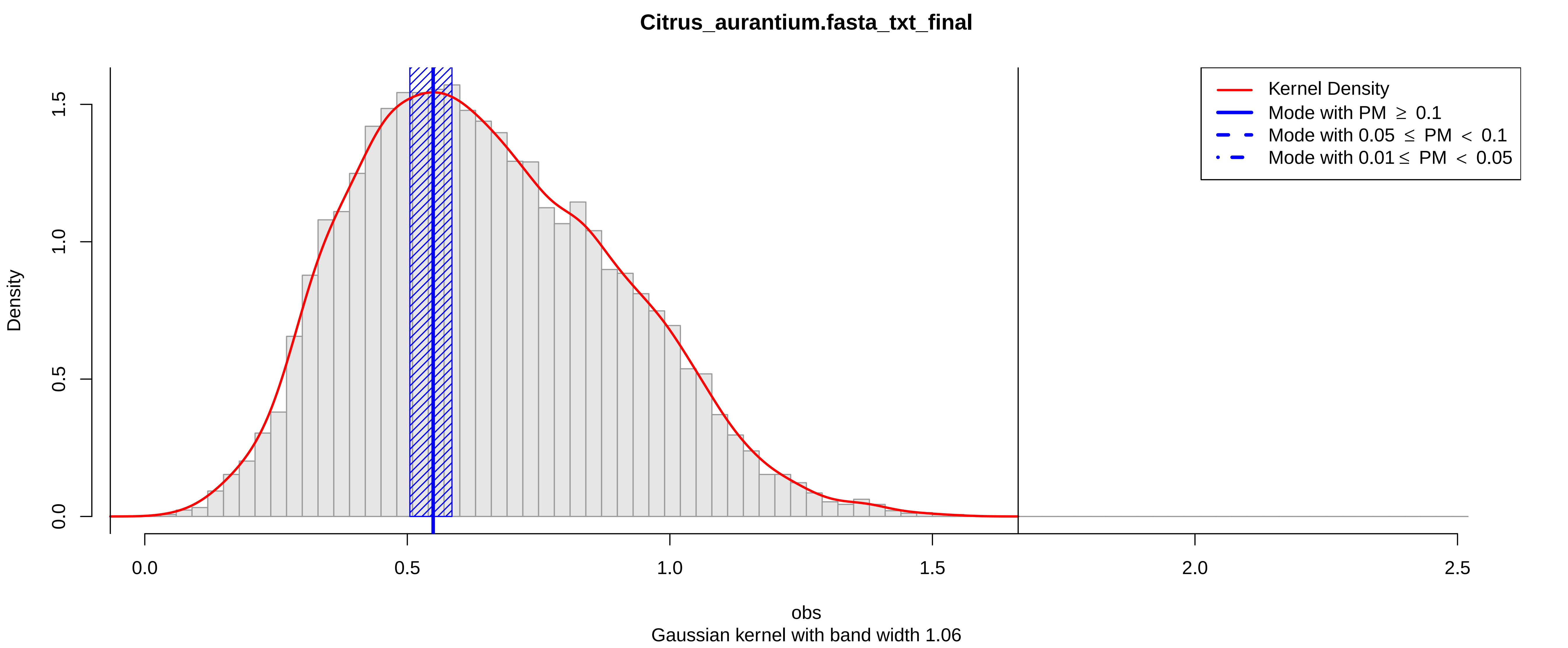
Citrus_aurantium.fasta_txt_final
Kernel Density
Mode with PM ≥ 0.1
Mode with 0.05 ≤ PM < 0.1
Mode with 0.01 ≤ PM < 0.05
Density
0.0
0.5
1.0
1.5
0.0
0.5
1.0
1.5
2.0
2.5
obs
Gaussian kernel with band width 1.06

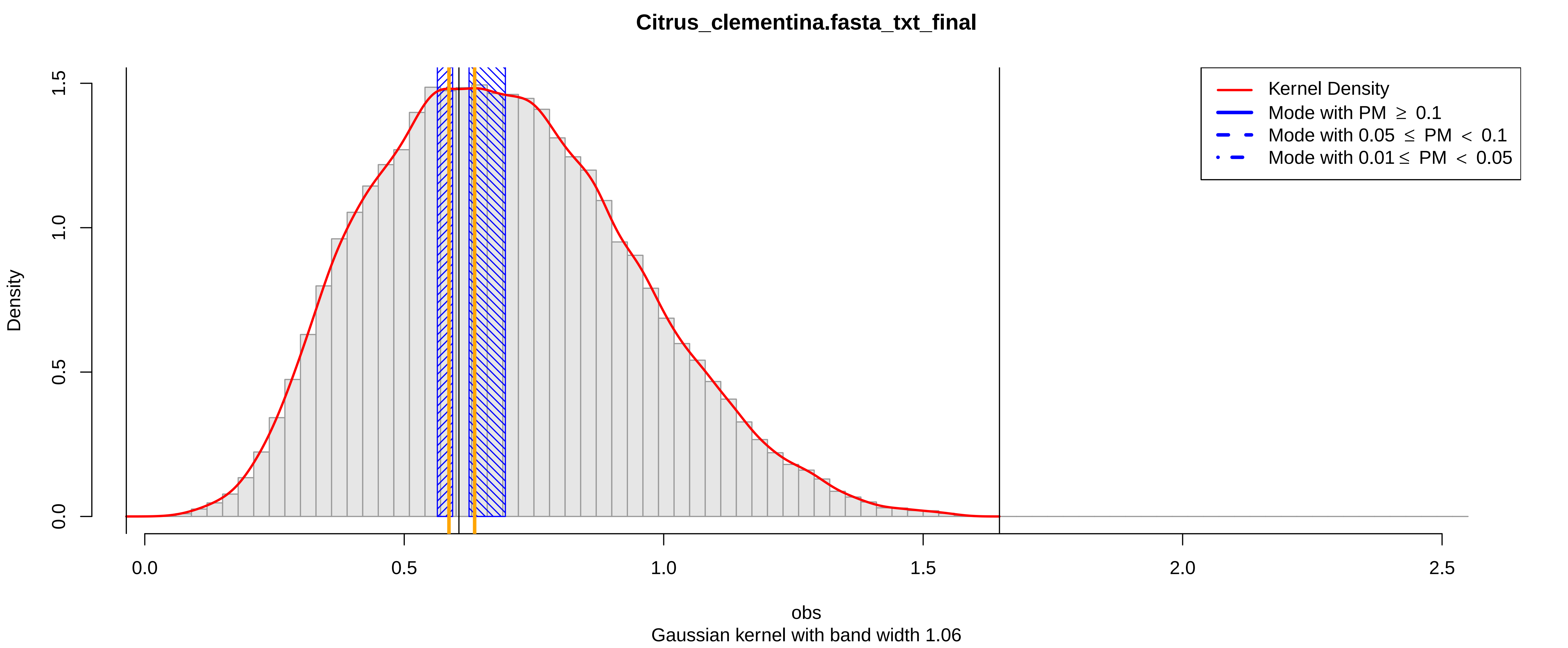

Citrus_clementina.fasta_txt_final
Kernel Density
Mode with PM ≥ 0.1
Mode with 0.05 ≤ PM < 0.1
Mode with 0.01 ≤ PM < 0.05
Density
0.0
0.5
1.0
1.5
0.0
0.5
1.0
1.5
2.0
2.5
obs
Gaussian kernel with band width 1.06

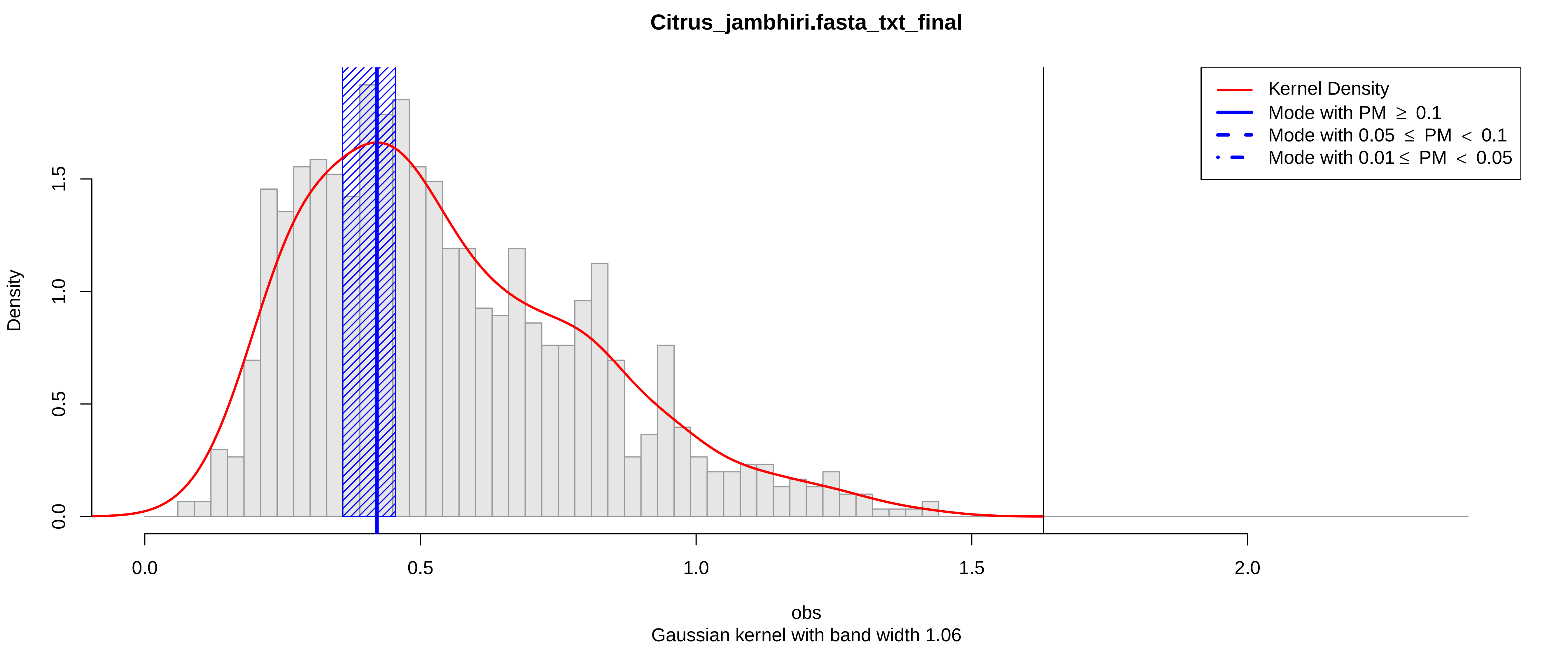
Citrus_jambhiri.fasta_txt_final
Kernel Density
Mode with PM ≥ 0.1
Mode with 0.05 ≤ PM < 0.1
Mode with 0.01 ≤ PM < 0.05
Density
0.0
0.5
1.0
1.5
0.0
0.5
1.0
1.5
2.0
obs
Gaussian kernel with band width 1.06

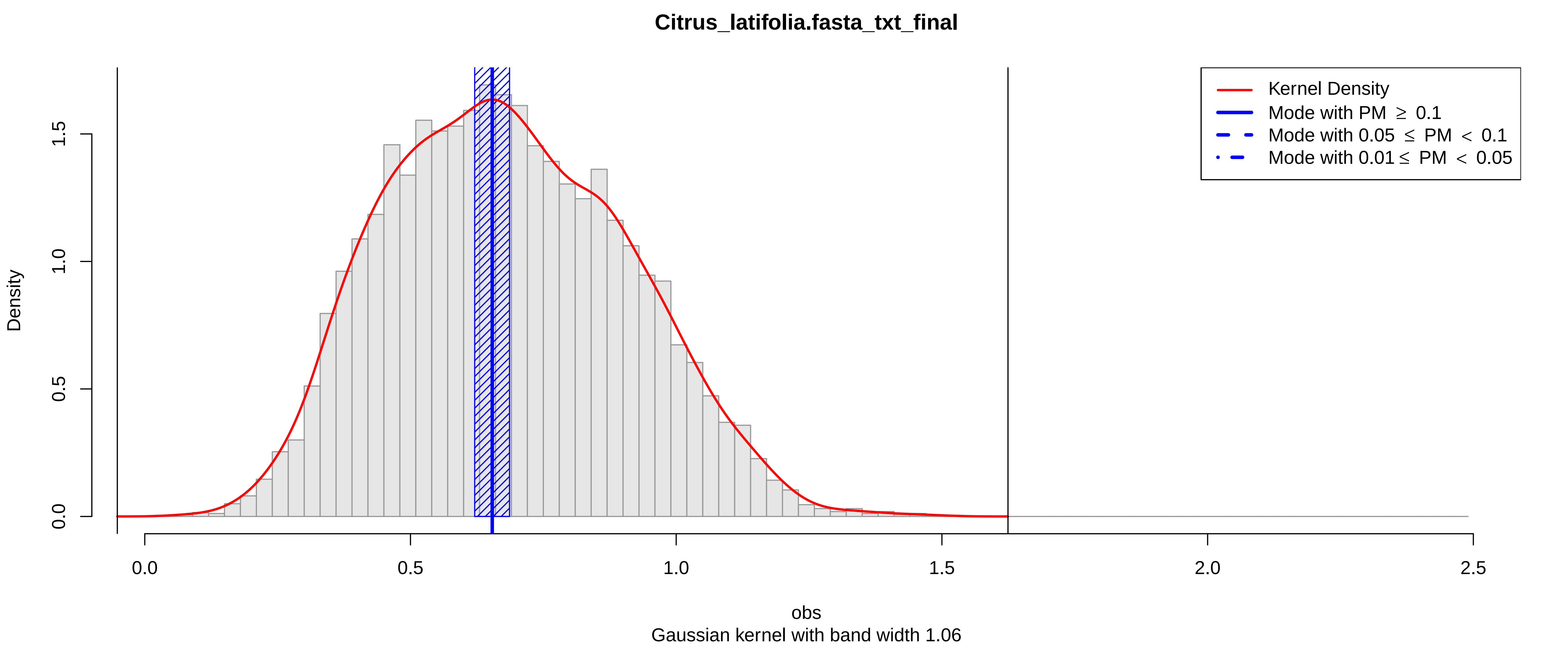

Citrus_latifolia.fasta_txt_final
Kernel Density
Mode with PM ≥ 0.1
Mode with 0.05 ≤ PM < 0.1
Mode with 0.01 ≤ PM < 0.05
Density
0.0
0.5
1.0
1.5
0.0
0.5
1.0
1.5
2.0
2.5
obs
Gaussian kernel with band width 1.06

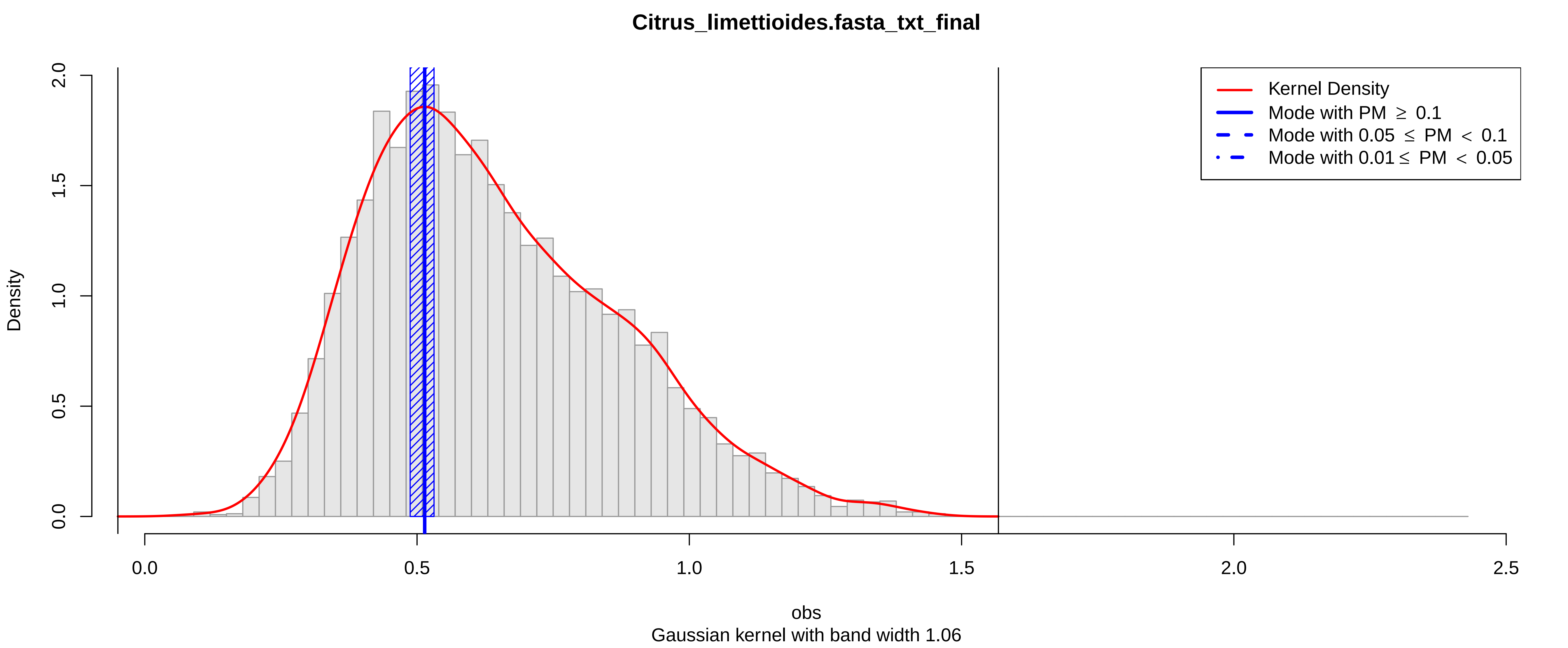

Citrus_limettioides.fasta_txt_final
Density
obs
Gaussian kernel with band width 1.06
Kernel Density
Mode with PM ≥ 0.1
Mode with 0.05 ≤ PM < 0.1
Mode with 0.01 ≤ PM < 0.05
0.0
0.5
1.0
1.5
2.0
2.5

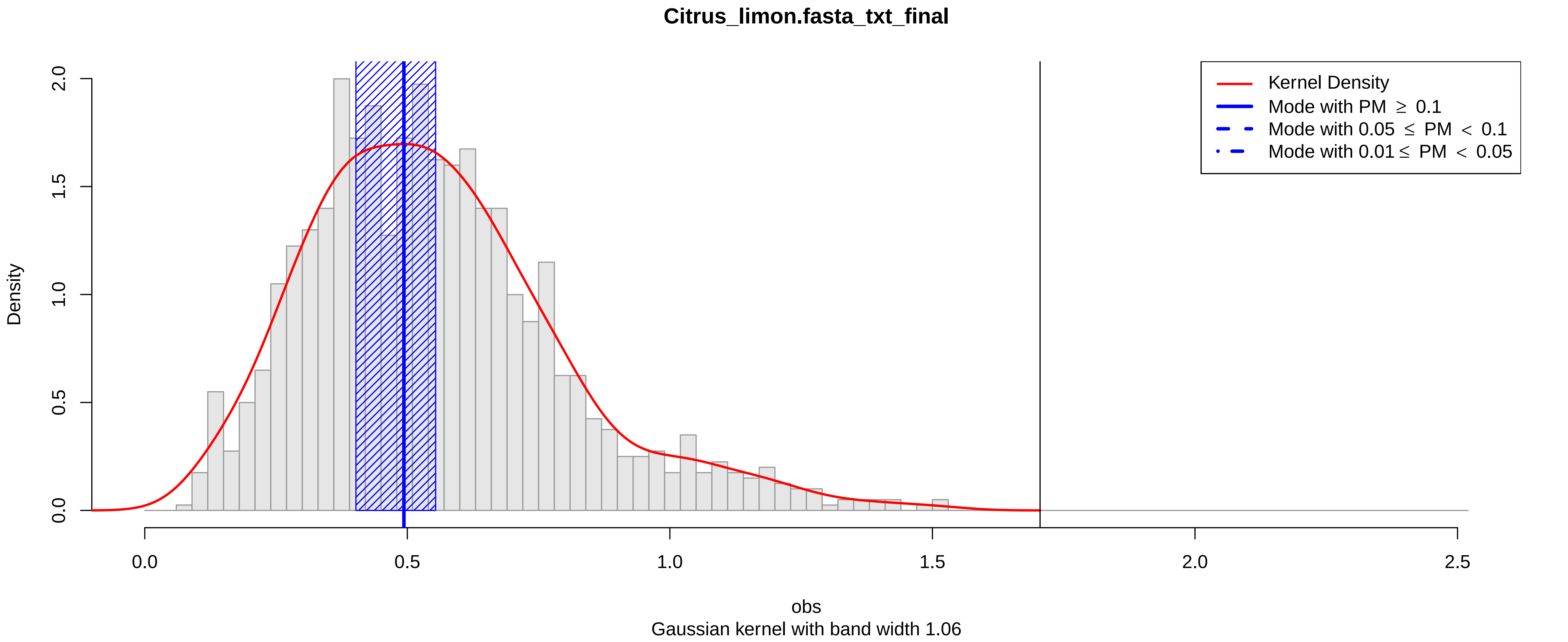
Citrus_limon.fasta_txt_final
Kernel Density
Mode with PM ≥ 0.1
Mode with 0.05 ≤ PM < 0.1
Mode with 0.01 ≤ PM < 0.05
Density
0.0
0.5
1.0
1.5
2.0
2.5
obs
Gaussian kernel with band width 1.06

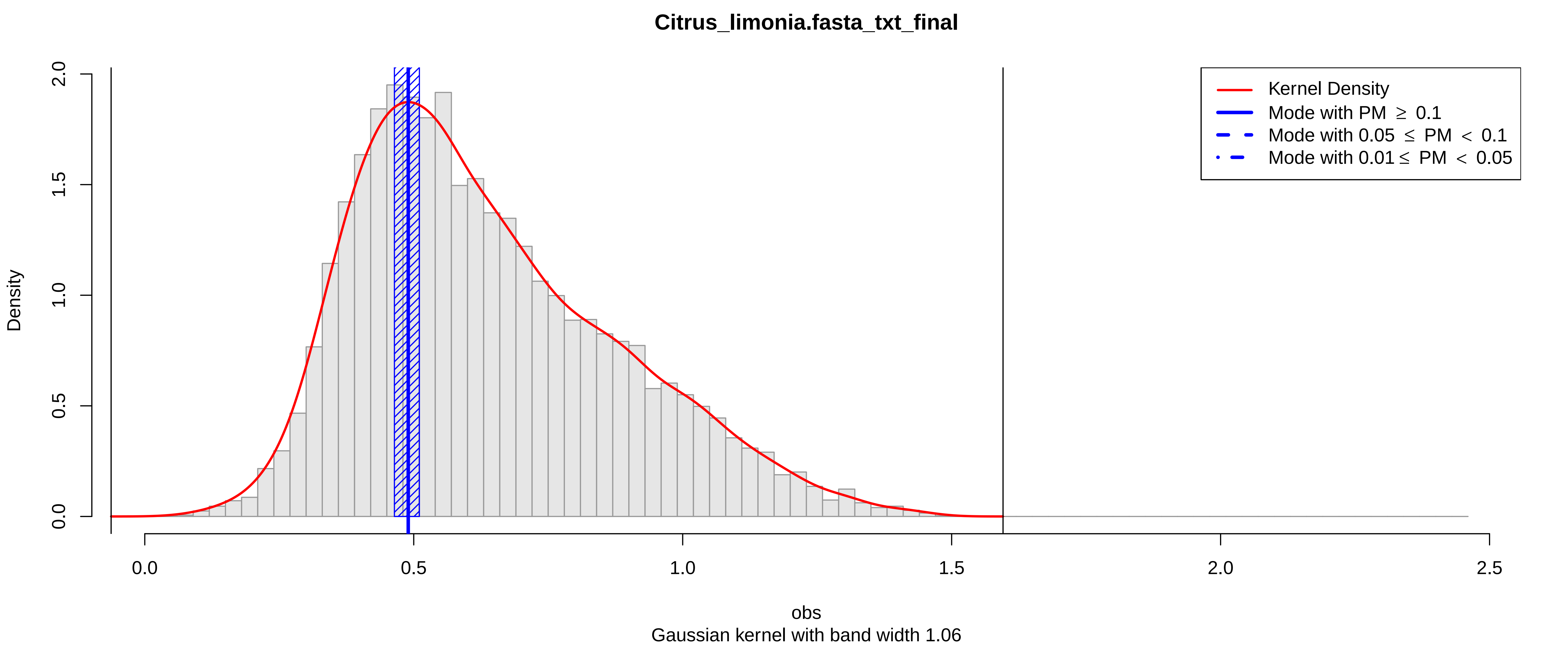

Citrus_limonia.fasta_txt_final
Density
2.0
1.5
1.0
0.5
0.0
0.0
0.5
1.0
1.5
2.0
2.5
obs
Gaussian kernel with band width 1.06
Kernel Density
Mode with PM ≥ 0.1
Mode with 0.05 ≤ PM < 0.1
Mode with 0.01 ≤ PM < 0.05

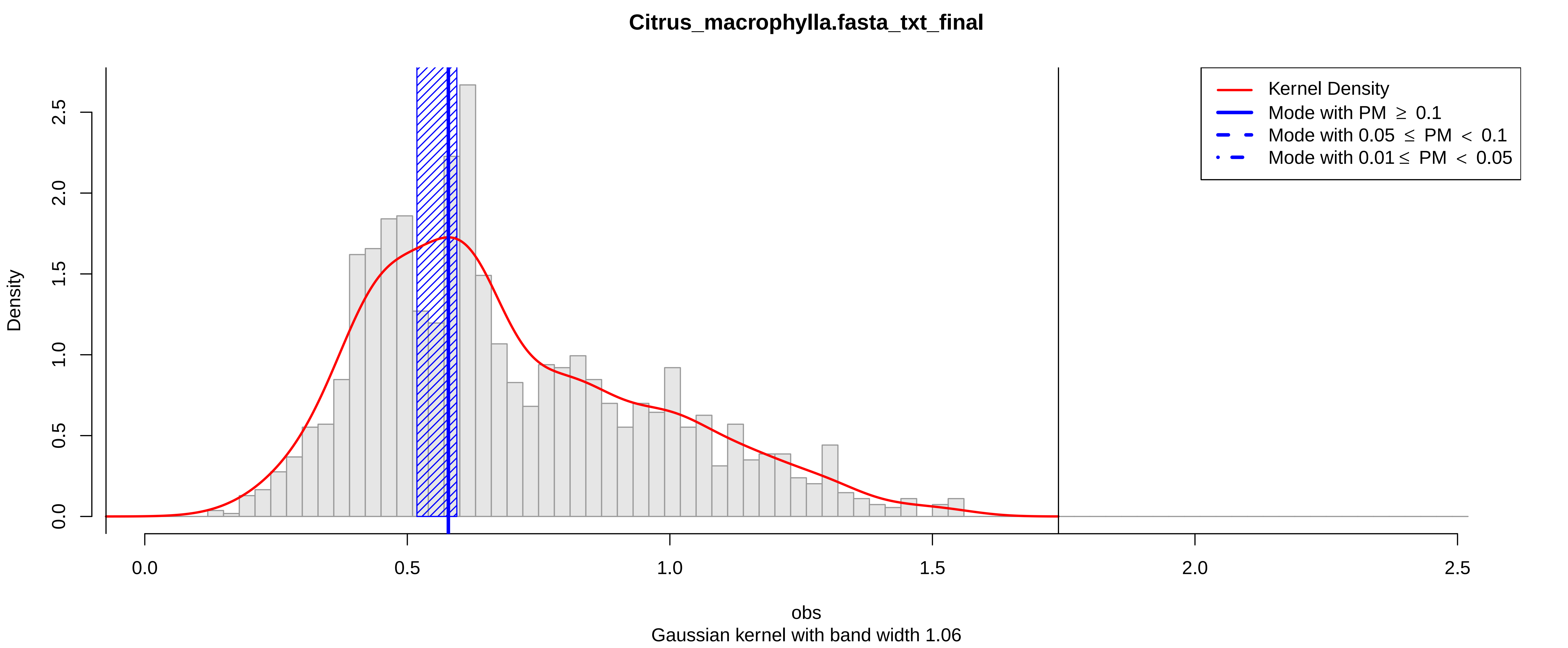

Citrus_macrophylla.fasta_txt_final
Kernel Density
Mode with PM ≥ 0.1
Mode with 0.05 ≤ PM < 0.1
Mode with 0.01 ≤ PM < 0.05
Density
0.0
0.5
1.0
1.5
2.0
2.5
obs
Gaussian kernel with band width 1.06

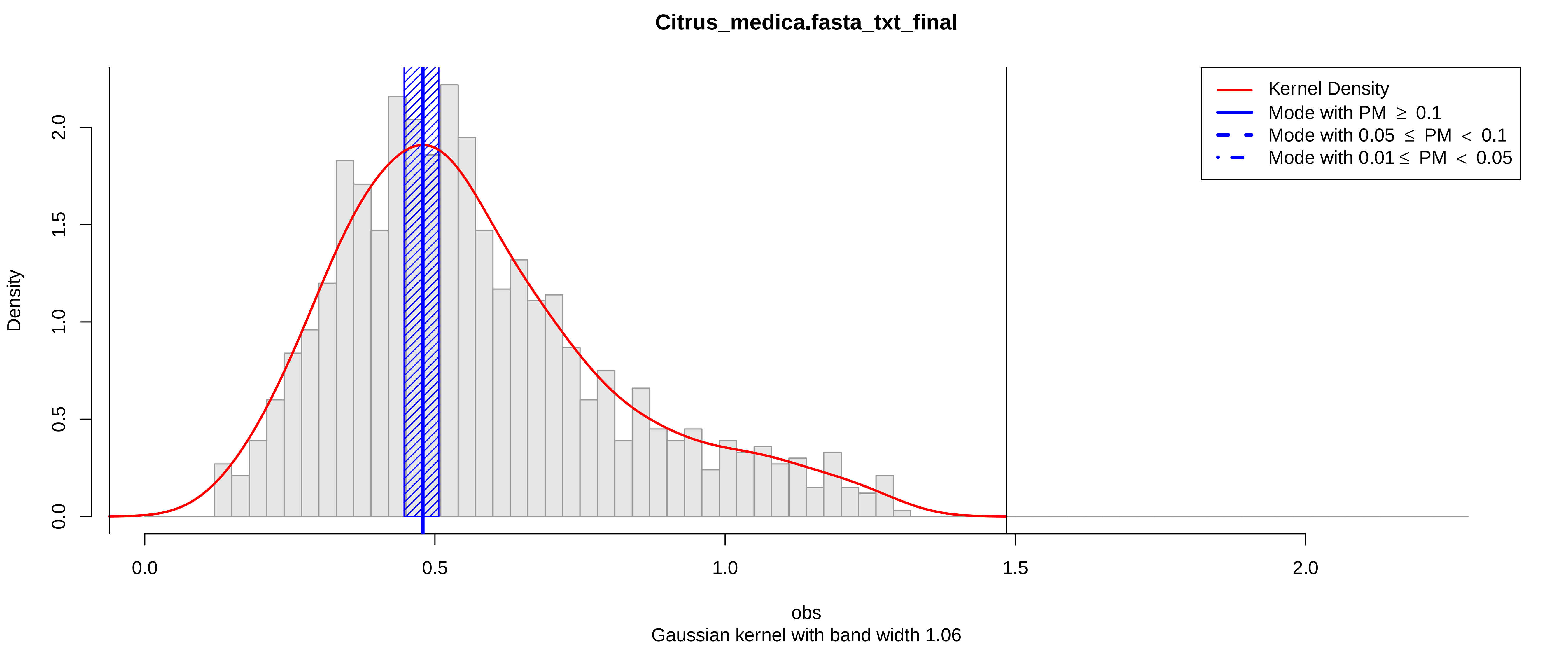
Citrus_medica.fasta_txt_final
Kernel Density
Mode with PM ≥ 0.1
Mode with 0.05 ≤ PM < 0.1
Mode with 0.01 ≤ PM < 0.05
Density
0.0
0.5
1.0
1.5
2.0
0.0
0.5
1.0
1.5
2.0
obs
Gaussian kernel with band width 1.06

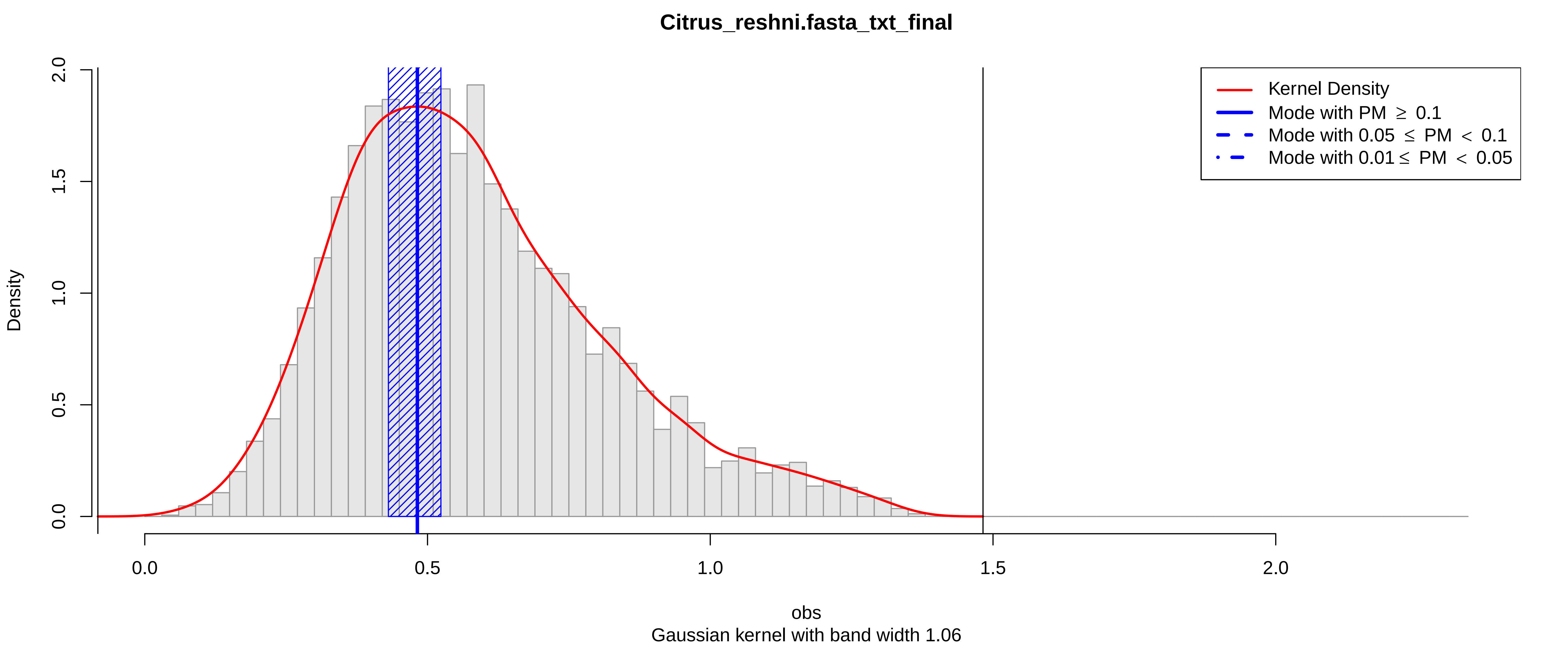
Citrus_reshni.fasta_txt_final
Density
2.0
1.5
1.0
0.5
0.0
0.0
0.5
1.0
1.5
2.0
obs
Gaussian kernel with band width 1.06
Kernel Density
Mode with PM ≥ 0.1
Mode with 0.05 ≤ PM < 0.1
Mode with 0.01 ≤ PM < 0.05

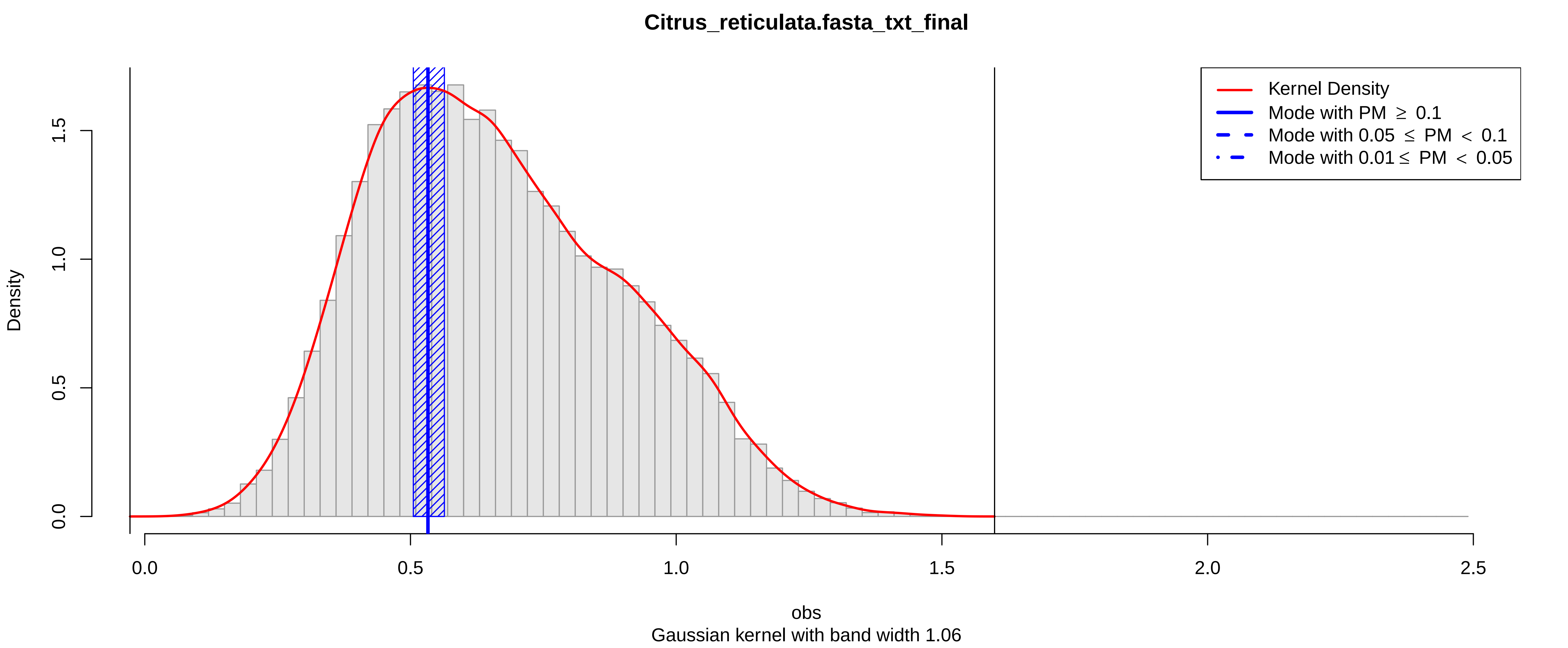

Citrus_reticulata.fasta_txt_final
Kernel Density
Mode with PM ≥ 0.1
Mode with 0.05 ≤ PM < 0.1
Mode with 0.01 ≤ PM < 0.05
Density
0.0
0.5
1.0
1.5
2.0
2.5
obs
Gaussian kernel with band width 1.06

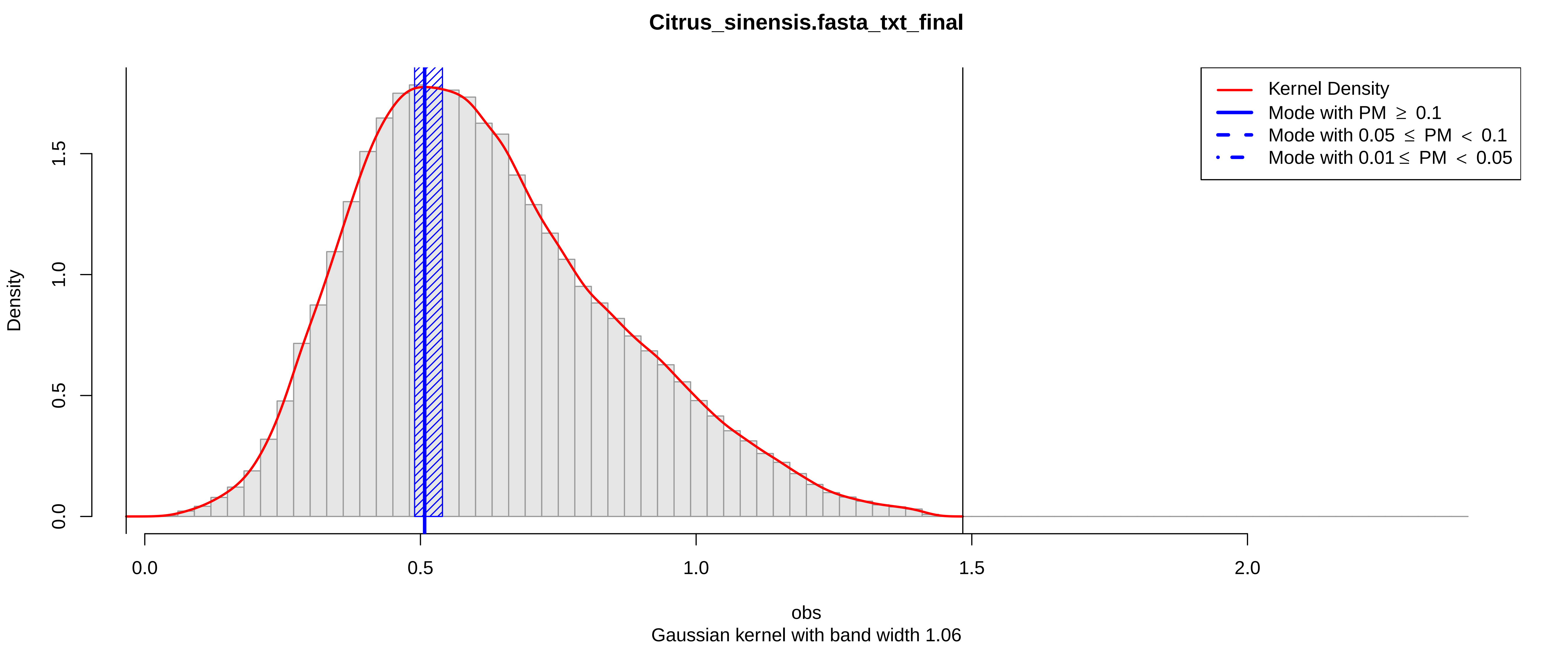
Citrus_sinensis.fasta_txt_final
Kernel Density
Mode with PM ≥ 0.1
Mode with 0.05 ≤ PM < 0.1
Mode with 0.01 ≤ PM < 0.05
Density
0.0
0.5
1.0
1.5
0.0
0.5
1.0
1.5
2.0
obs
Gaussian kernel with band width 1.06

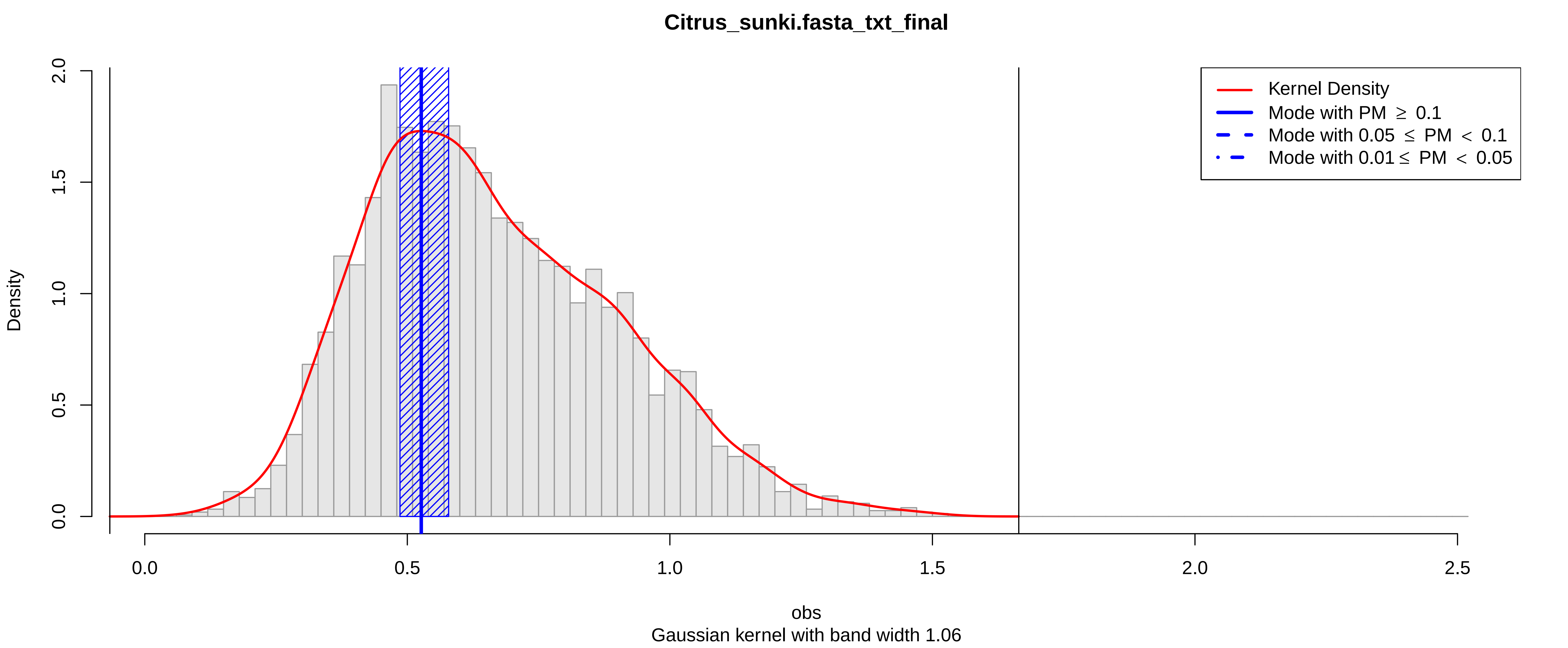
Citrus_sunki.fasta_txt_final
Kernel Density
Mode with PM ≥ 0.1
Mode with 0.05 ≤ PM < 0.1
Mode with 0.01 ≤ PM < 0.05
Density
0.0
0.5
1.0
1.5
2.0
0.0
0.5
1.0
1.5
2.0
2.5
obs
Gaussian kernel with band width 1.06

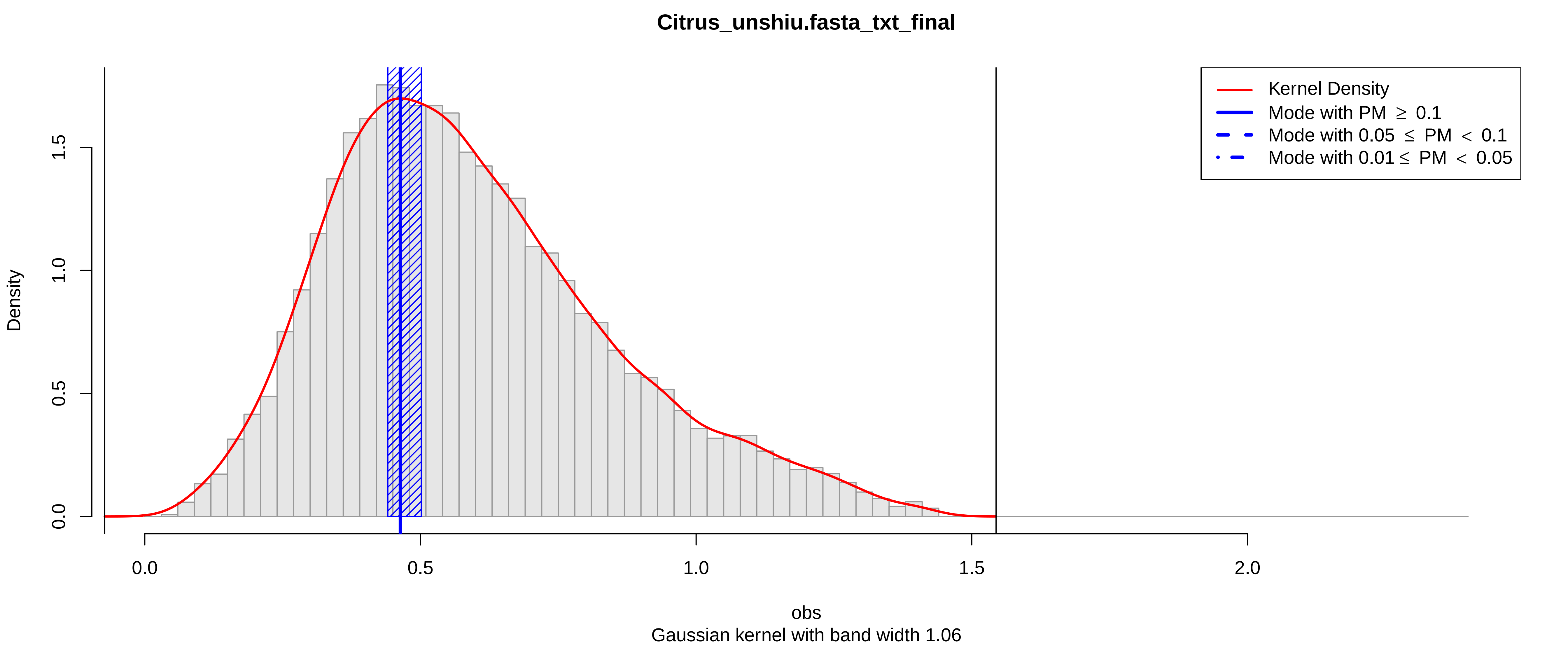
Citrus_unshiu.fasta_txt_final
Density
0.0
0.5
1.0
1.5
2.0
obs
Gaussian kernel with band width 1.06
Kernel Density
Mode with PM ≥ 0.1
Mode with 0.05 ≤ PM < 0.1
Mode with 0.01 ≤ PM < 0.05

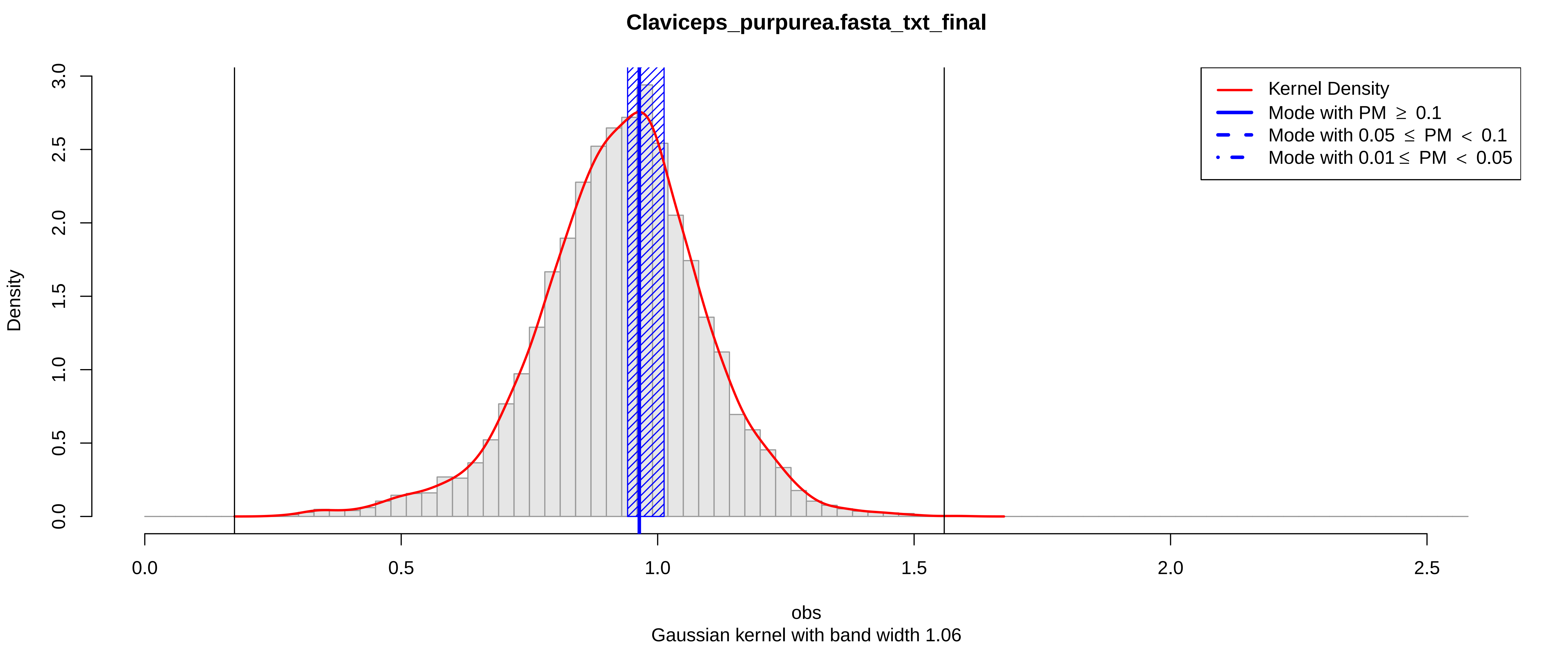
Claviceps_purpurea.fasta_txt_final
Kernel Density
Mode with PM ≥ 0.1
Mode with 0.05 ≤ PM < 0.1
Mode with 0.01 ≤ PM < 0.05
Density
0.0
0.5
1.0
1.5
2.0
2.5
3.0
obs
Gaussian kernel with band width 1.06

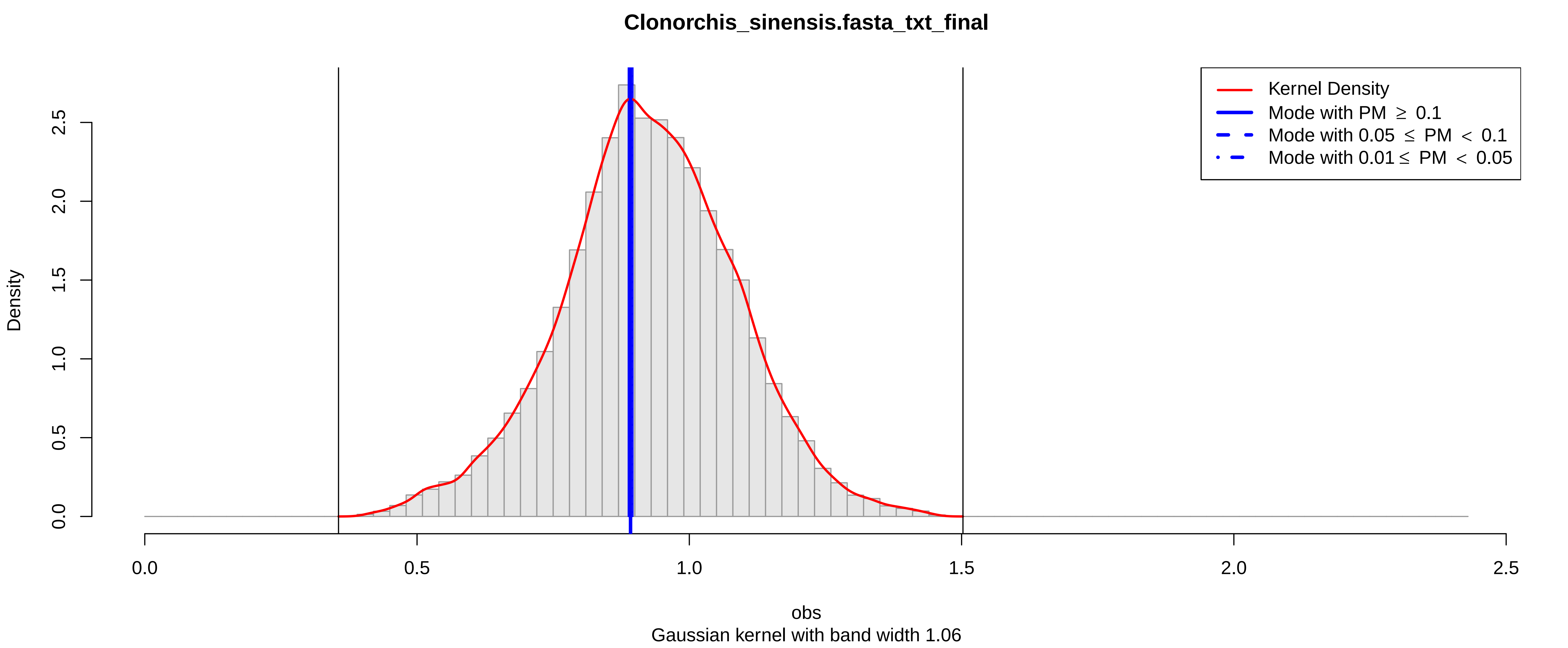

Clonorchis_sinensis.fasta_txt_final
Kernel Density
Mode with PM ≥ 0.1
Mode with 0.05 ≤ PM < 0.1
Mode with 0.01 ≤ PM < 0.05
Density
0.0
0.5
1.0
1.5
2.0
2.5
obs
Gaussian kernel with band width 1.06

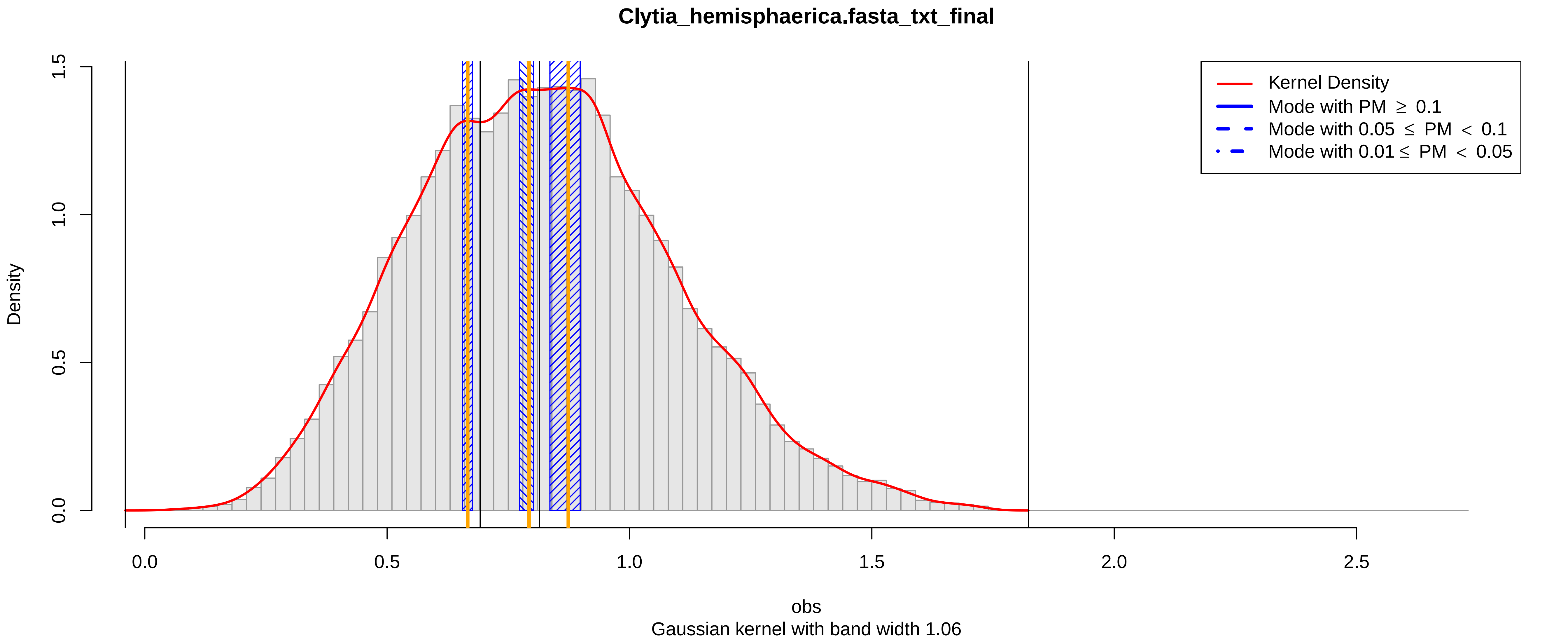

Clytia_hemisphaerica.fasta_txt_final
Density
1.5
1.0
0.5
0.0
0.0
0.5
1.0
1.5
2.0
2.5
obs
Gaussian kernel with band width 1.06
Kernel Density
Mode with PM ≥ 0.1
Mode with 0.05 ≤ PM < 0.1
Mode with 0.01 ≤ PM < 0.05

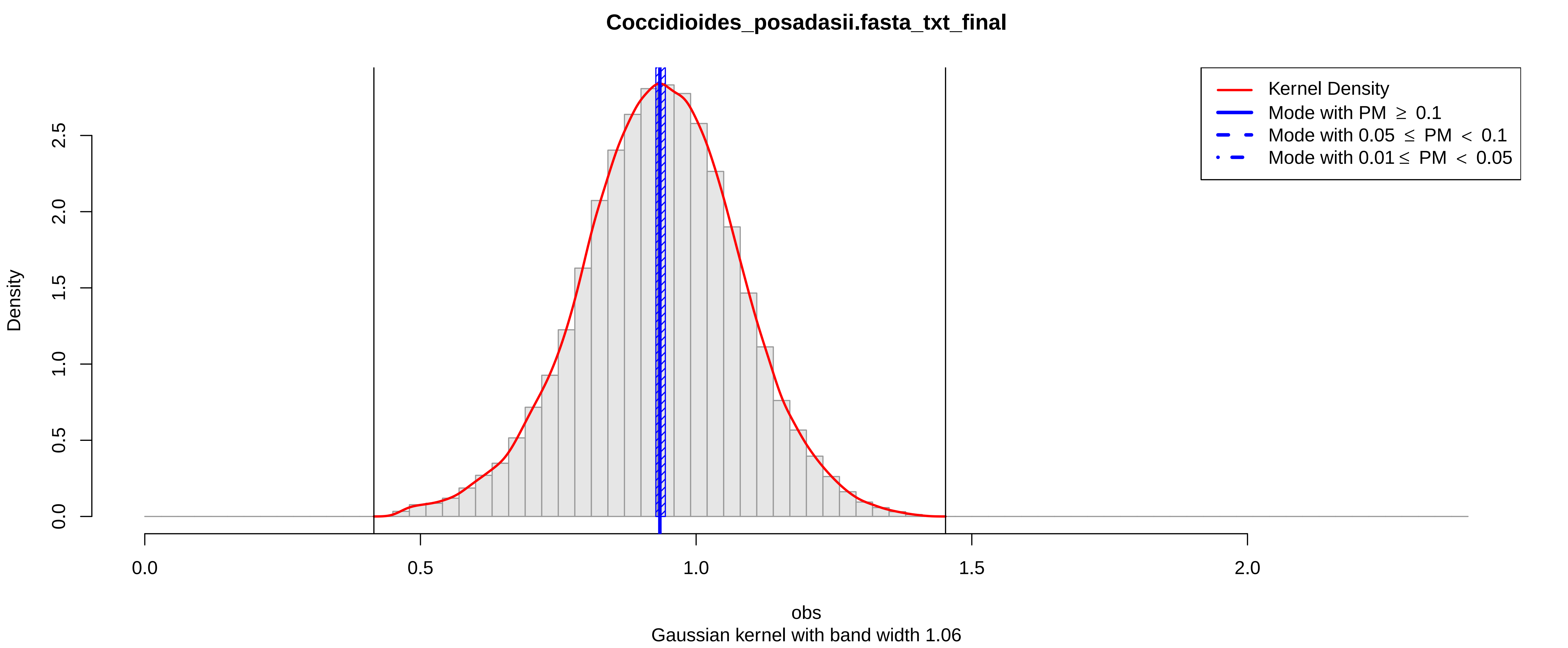
Coccidioides_posadasii.fasta_txt_final
Kernel Density
Mode with PM ≥ 0.1
Mode with 0.05 ≤ PM < 0.1
Mode with 0.01 ≤ PM < 0.05
Density
0.0
0.5
1.0
1.5
2.0
2.5
obs
Gaussian kernel with band width 1.06

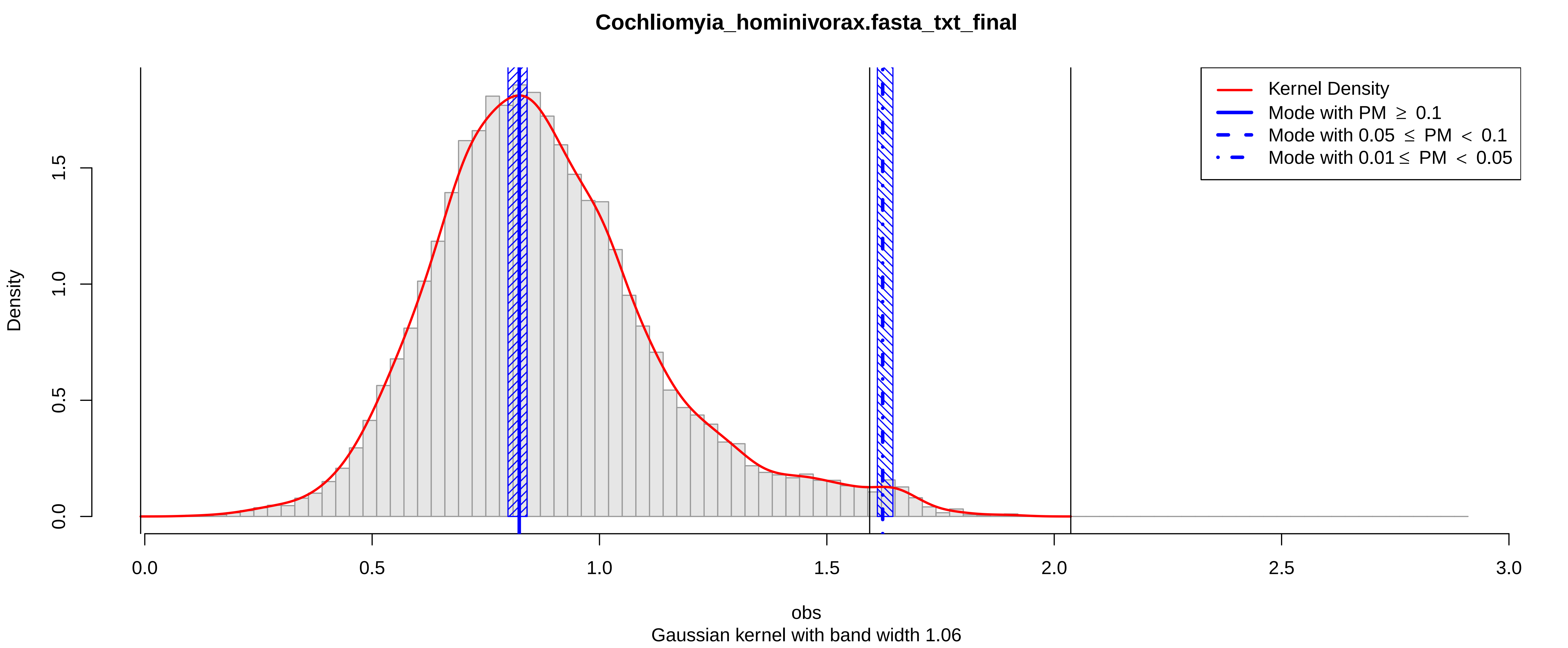

Cochliomyia_hominivorax.fasta_txt_final
Kernel Density
Mode with PM ≥ 0.1
Mode with 0.05 ≤ PM < 0.1
Mode with 0.01 ≤ PM < 0.05
Density
0.0
0.5
1.0
1.5
0.0
0.5
1.0
1.5
2.0
2.5
3.0
obs
Gaussian kernel with band width 1.06

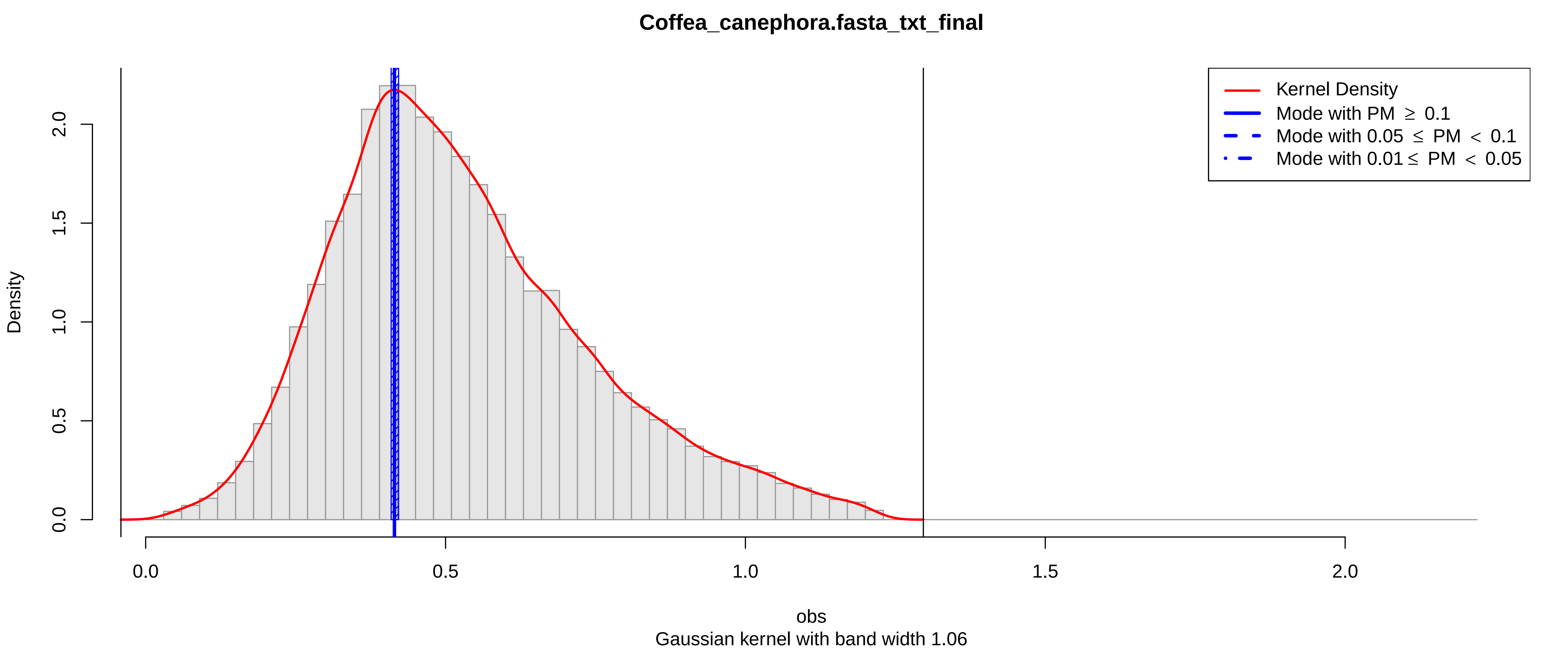

Coffea_canephora.fasta_txt_final
Kernel Density
Mode with PM ≥ 0.1
Mode with 0.05 ≤ PM < 0.1
Mode with 0.01 ≤ PM < 0.05
Density
0.0
0.5
1.0
1.5
2.0
obs
Gaussian kernel with band width 1.06

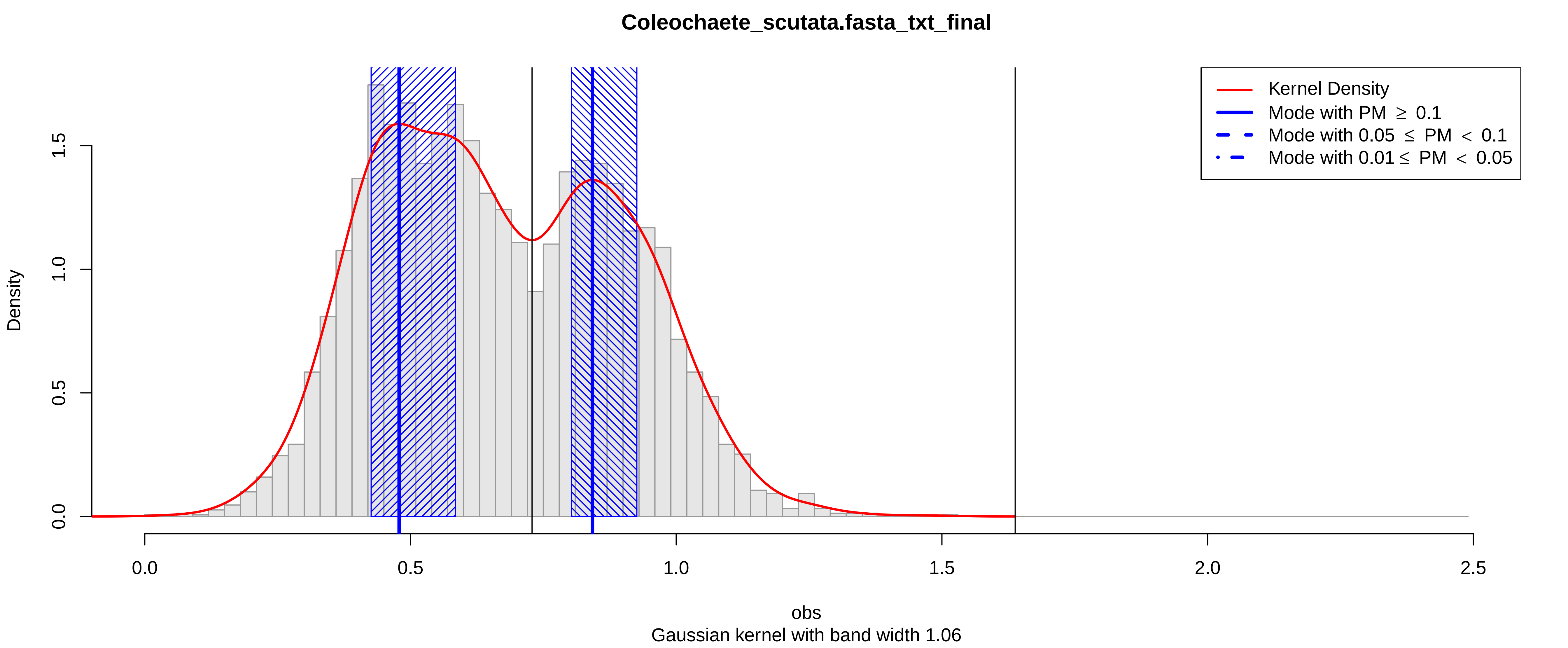

Coleochaete_scutata.fasta_txt_final
Kernel Density
Mode with PM ≥ 0.1
Mode with 0.05 ≤ PM < 0.1
Mode with 0.01 ≤ PM < 0.05
Density
0.0
0.5
1.0
1.5
0.0
0.5
1.0
1.5
2.0
2.5
obs
Gaussian kernel with band width 1.06

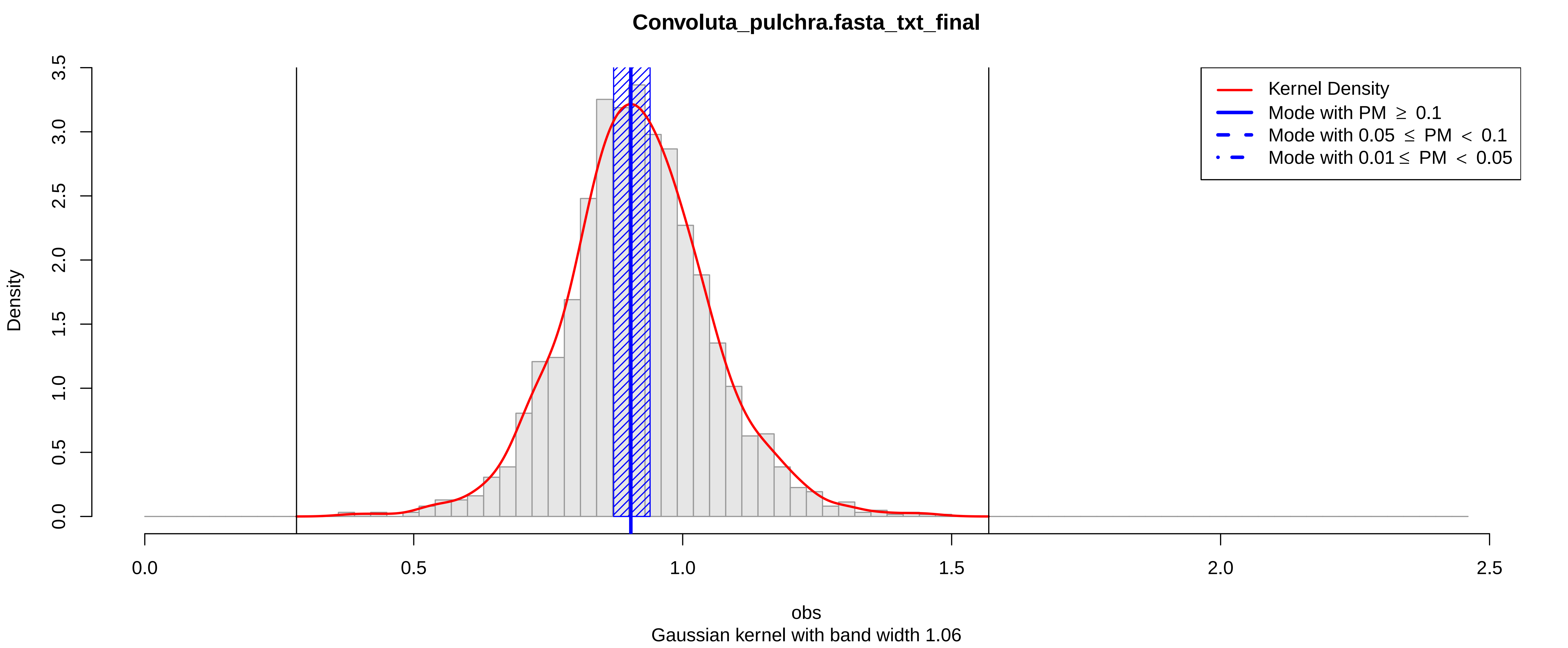

Convoluta_pulchra.fasta_txt_final
Density
0.0
0.5
1.0
1.5
2.0
2.5
3.0
3.5
Kernel Density
Mode with PM ≥ 0.1
Mode with 0.05 ≤ PM < 0.1
Mode with 0.01 ≤ PM < 0.05
0.0
0.5
1.0
1.5
2.0
2.5
obs
Gaussian kernel with band width 1.06

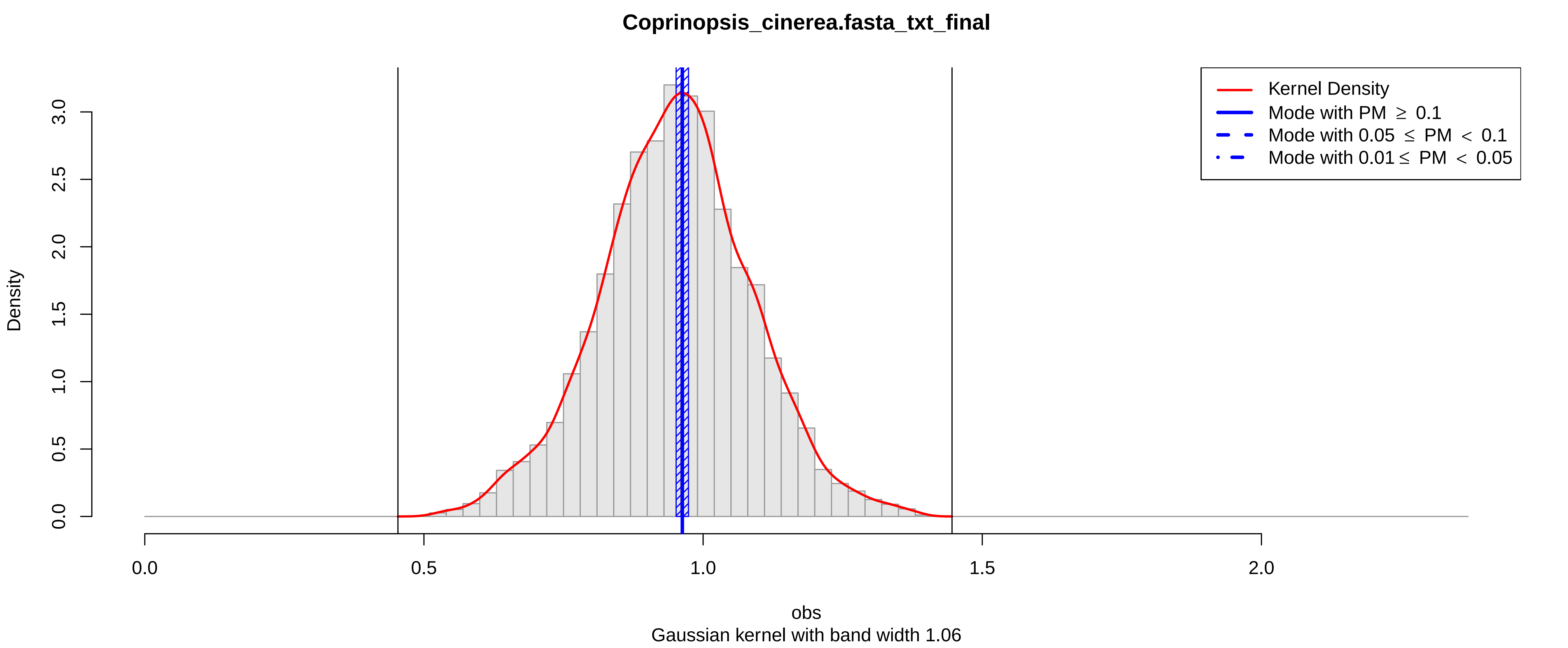

Coprinopsis_cinerea.fasta_txt_final
Kernel Density
Mode with PM ≥ 0.1
Mode with 0.05 ≤ PM < 0.1
Mode with 0.01 ≤ PM < 0.05
Density
0.0
0.5
1.0
1.5
2.0
2.5
3.0
0.0
0.5
1.0
1.5
2.0
obs
Gaussian kernel with band width 1.06

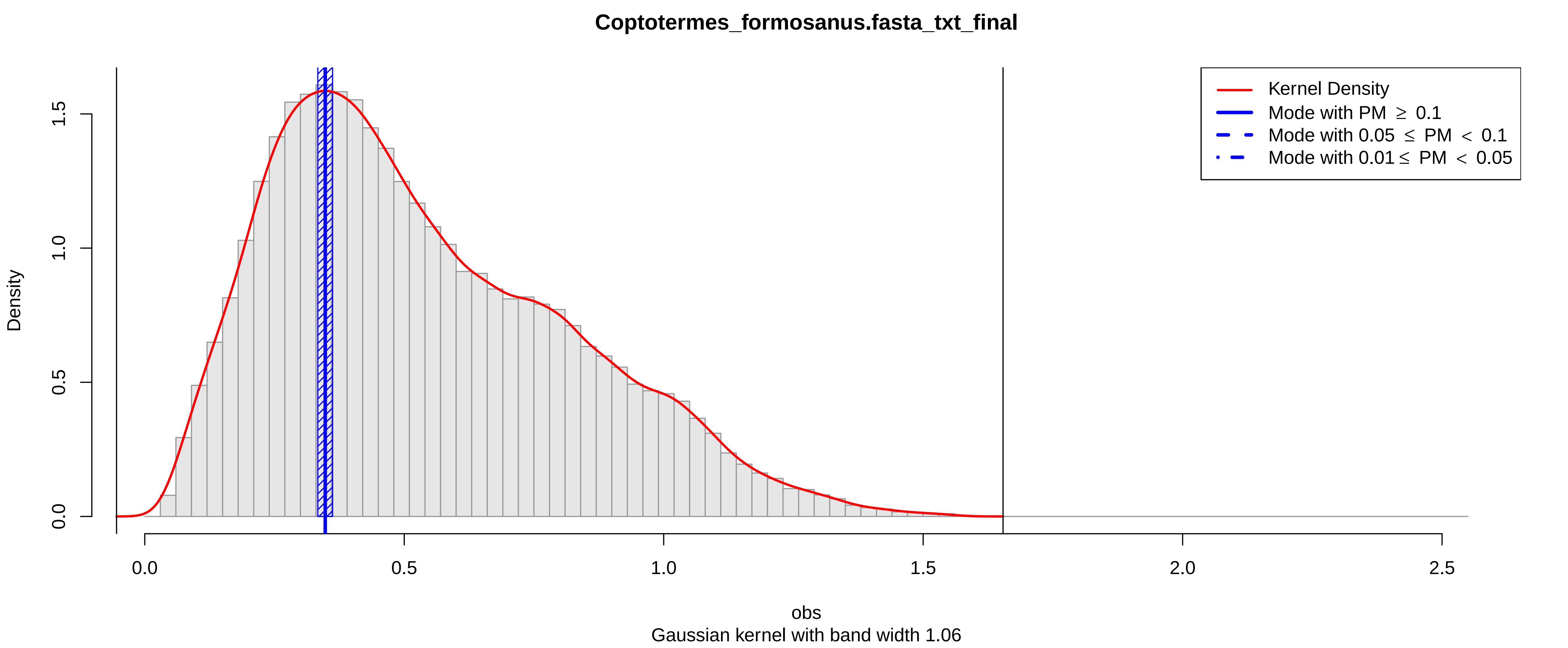
Coptotermes_formosanus.fasta_txt_final
Kernel Density
Mode with PM ≥ 0.1
Mode with 0.05 ≤ PM < 0.1
Mode with 0.01 ≤ PM < 0.05
Density
0.0
0.5
1.0
1.5
0.0
0.5
1.0
1.5
2.0
2.5
obs
Gaussian kernel with band width 1.06

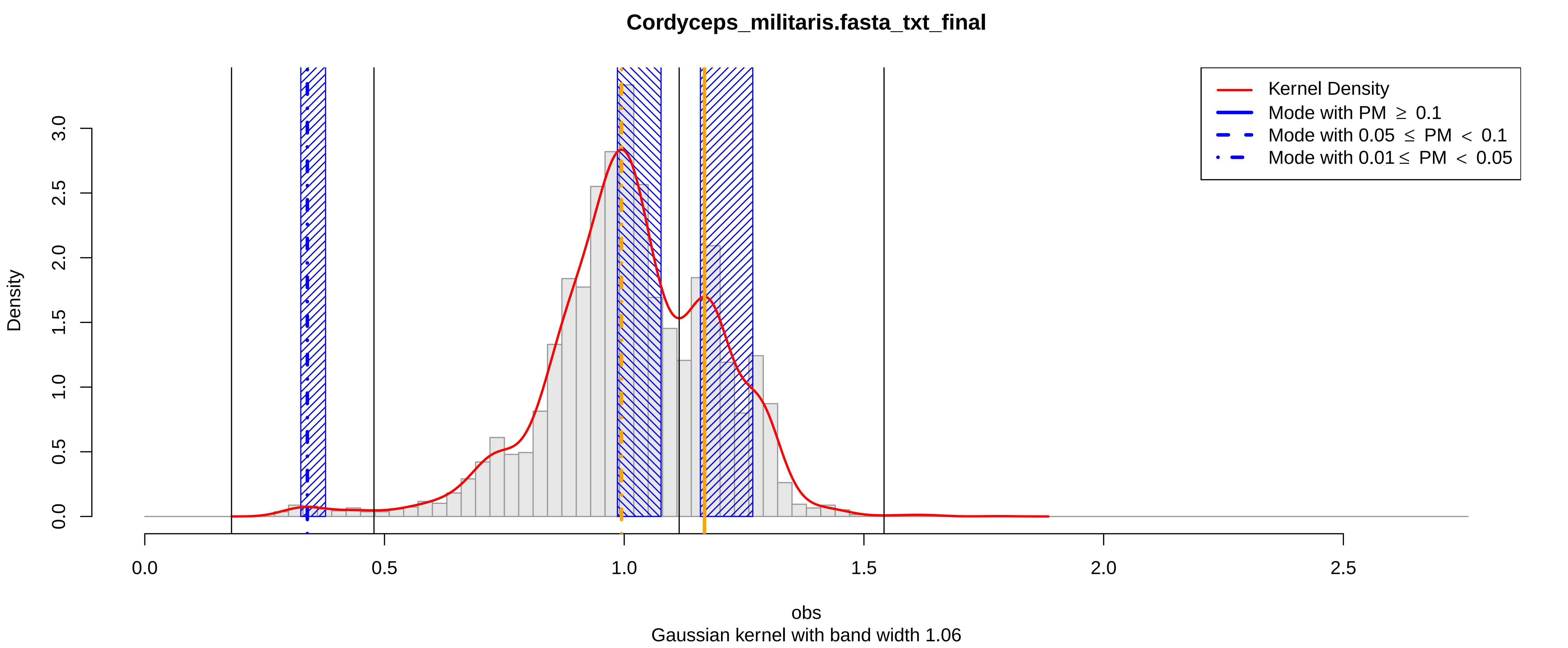
Cordyceps_militaris.fasta_txt_final
Kernel Density
Mode with PM ≥ 0.1
Mode with 0.05 ≤ PM < 0.1
Mode with 0.01 ≤ PM < 0.05
Density
0.0
0.5
1.0
1.5
2.0
2.5
3.0
0.0
0.5
1.0
1.5
2.0
2.5
obs
Gaussian kernel with band width 1.06

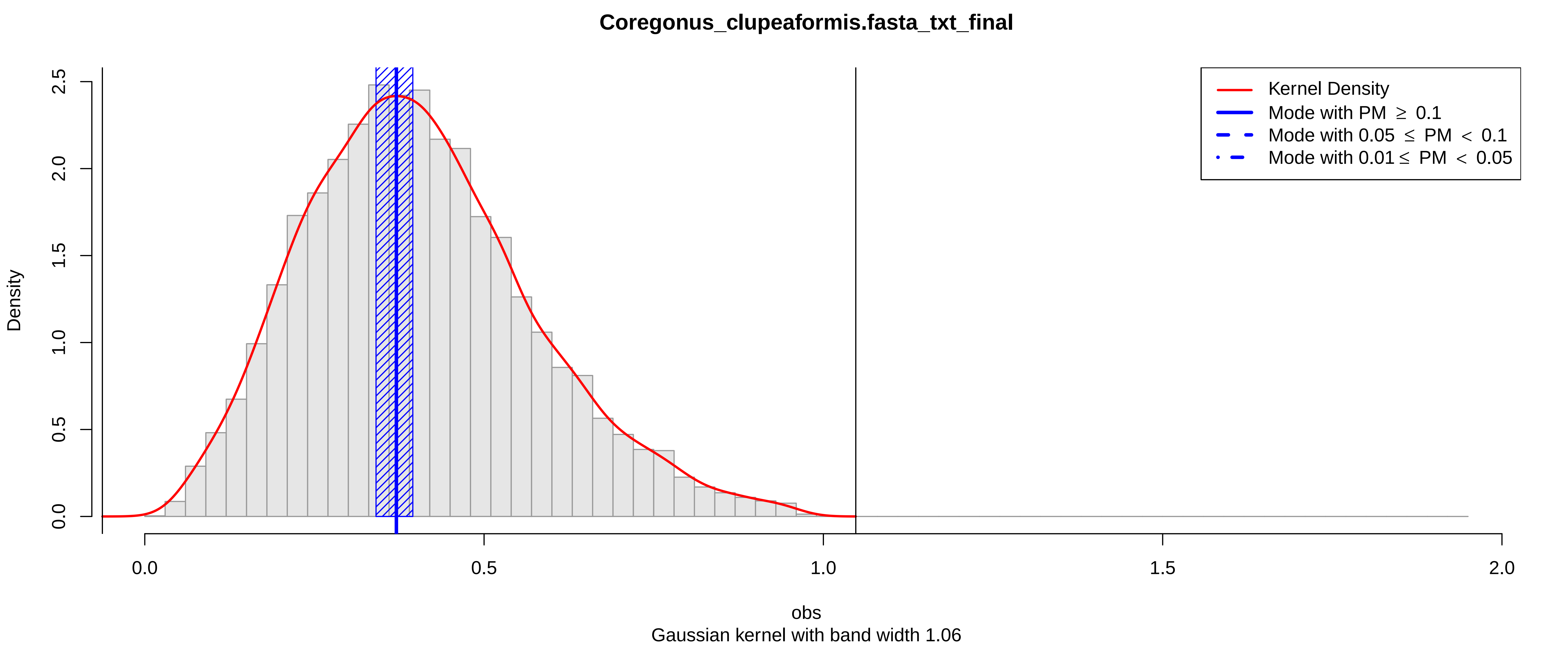
Coregonus_clupeaformis.fasta_txt_final
Density
2.5
2.0
1.5
1.0
0.5
0.0
0.0
0.5
1.0
1.5
2.0
obs
Gaussian kernel with band width 1.06
Kernel Density
Mode with PM ≥ 0.1
Mode with 0.05 ≤ PM < 0.1
Mode with 0.01 ≤ PM < 0.05

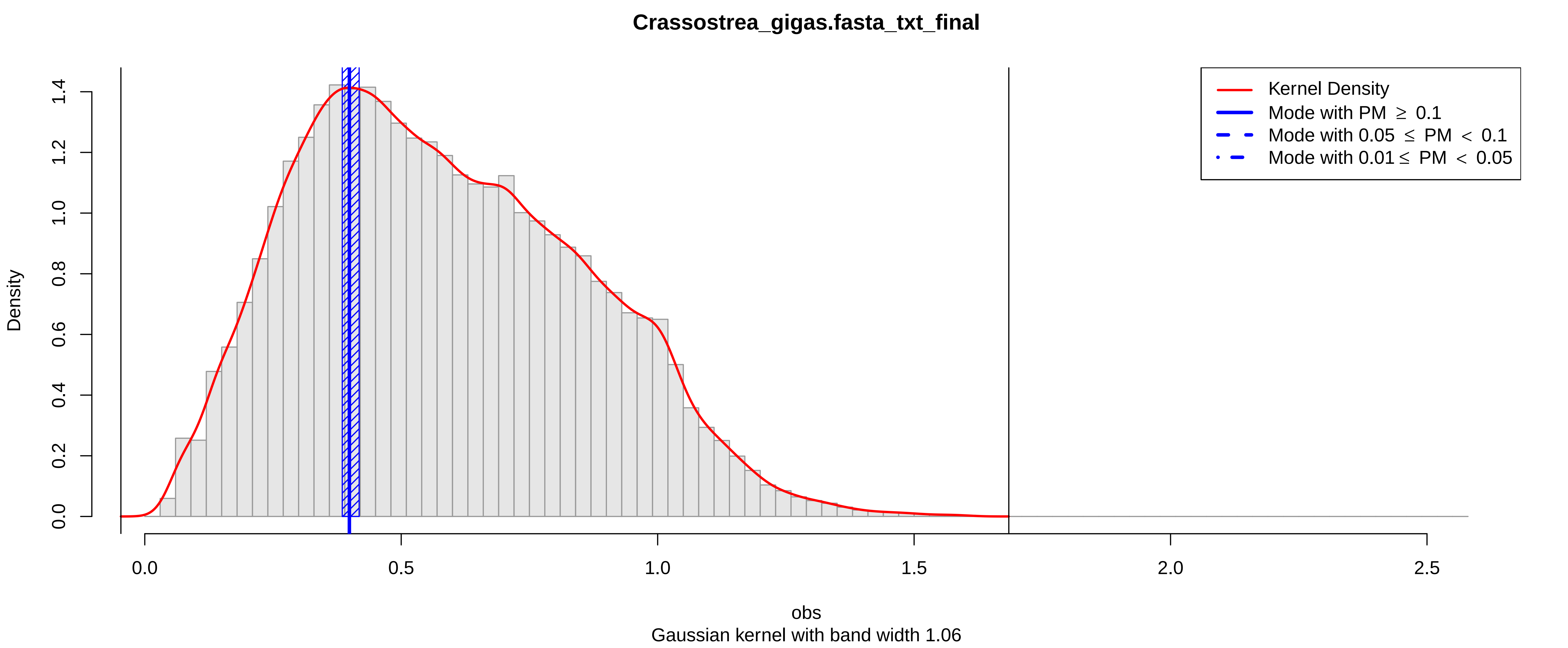
Crassostrea_gigas.fasta_txt_final
Kernel Density
Mode with PM ≥ 0.1
Mode with 0.05 ≤ PM < 0.1
Mode with 0.01 ≤ PM < 0.05
Density
0.0
0.2
0.4
0.6
0.8
1.0
1.2
1.4
0.0
0.5
1.0
1.5
2.0
2.5
obs
Gaussian kernel with band width 1.06

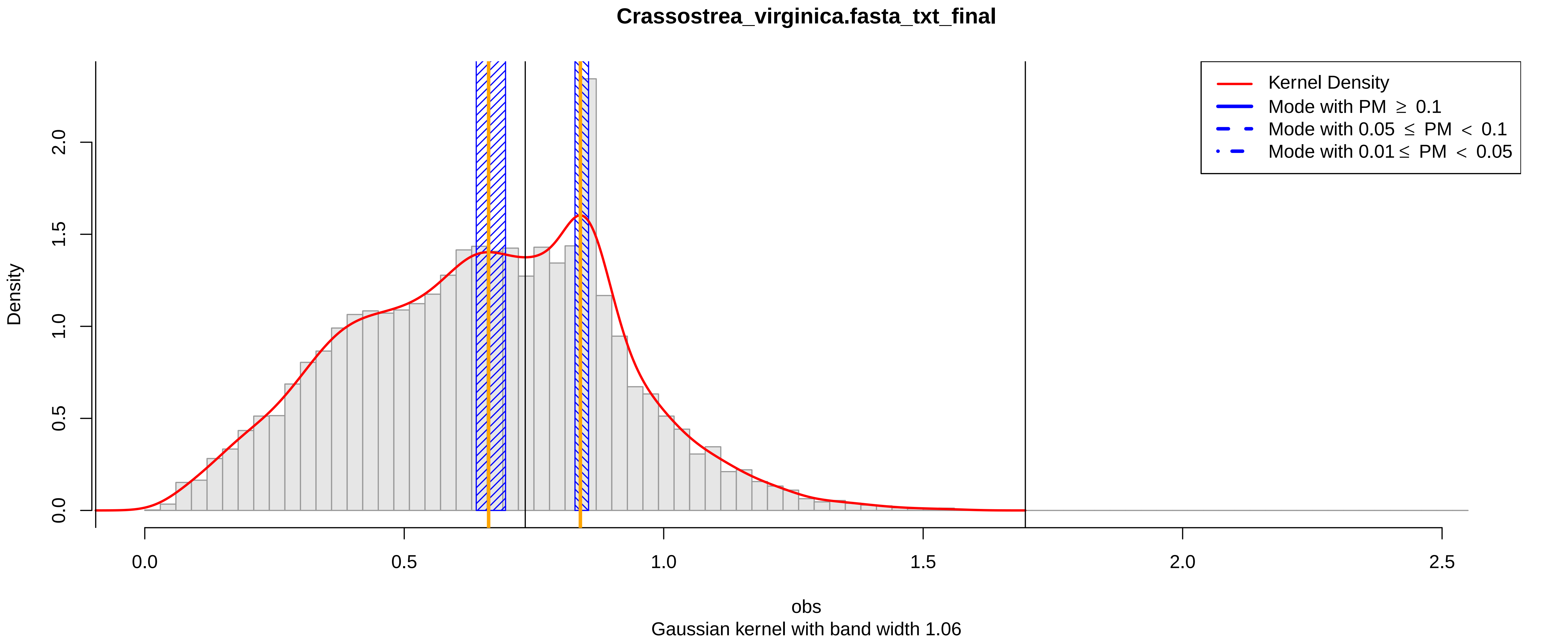
Crassostrea_virginica.fasta_txt_final
Kernel Density
Mode with PM ≥ 0.1
Mode with 0.05 ≤ PM < 0.1
Mode with 0.01 ≤ PM < 0.05
Density
0.0
0.5
1.0
1.5
2.0
0.0
0.5
1.0
1.5
2.0
2.5
obs
Gaussian kernel with band width 1.06

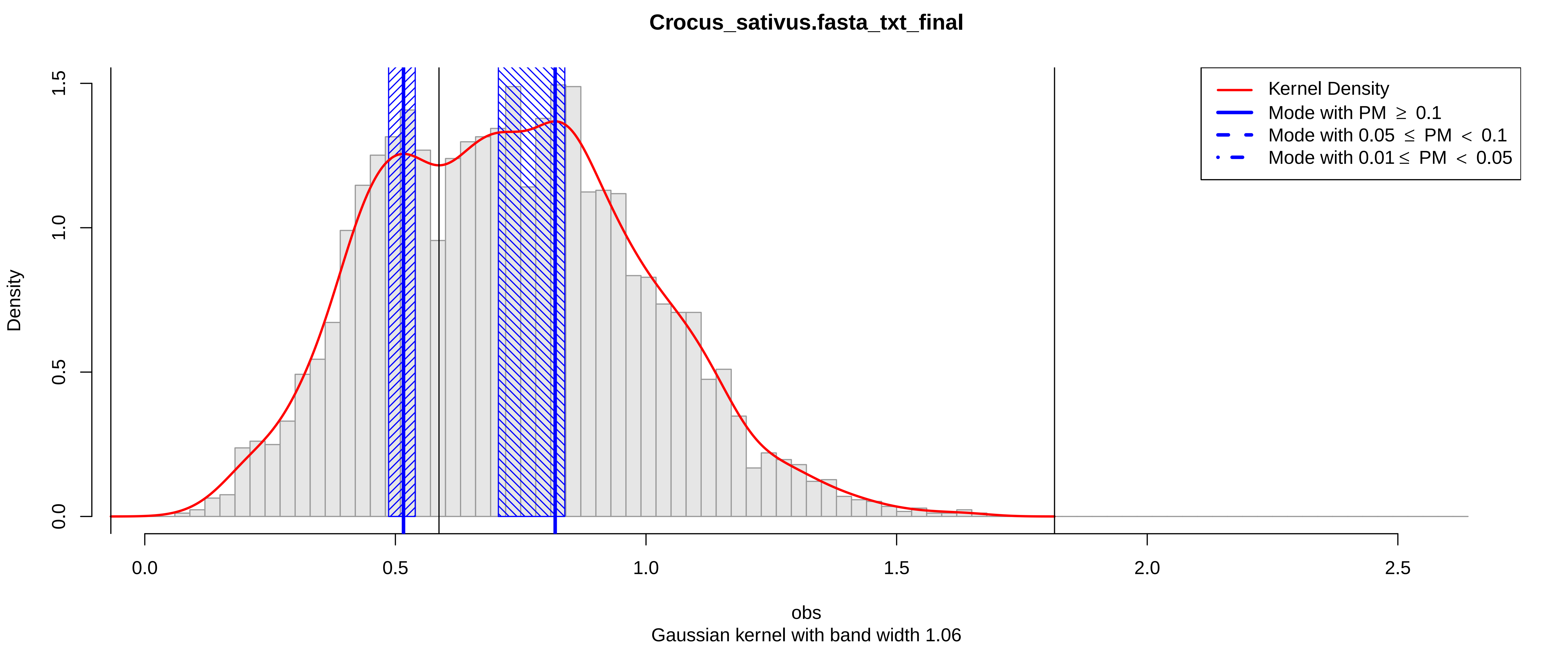

Crocus_sativus.fasta_txt_final
Kernel Density
Mode with PM ≥ 0.1
Mode with 0.05 ≤ PM < 0.1
Mode with 0.01 ≤ PM < 0.05
Density
0.0
0.5
1.0
1.5
0.0
0.5
1.0
1.5
2.0
2.5
obs
Gaussian kernel with band width 1.06

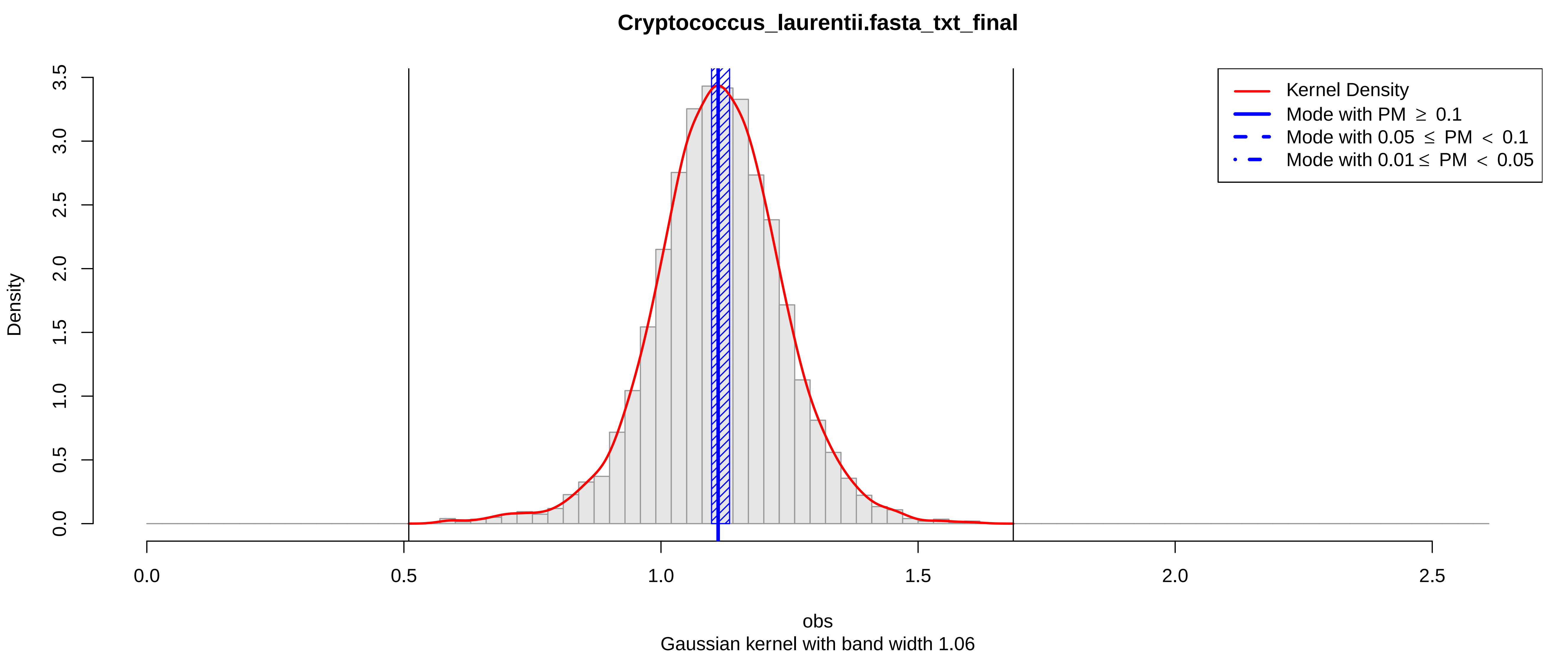

Cryptococcus_laurentii.fasta_txt_final
Kernel Density
Mode with PM ≥ 0.1
Mode with 0.05 ≤ PM < 0.1
Mode with 0.01 ≤ PM < 0.05
Density
0.0
0.5
1.0
1.5
2.0
2.5
3.0
3.5
obs
Gaussian kernel with band width 1.06

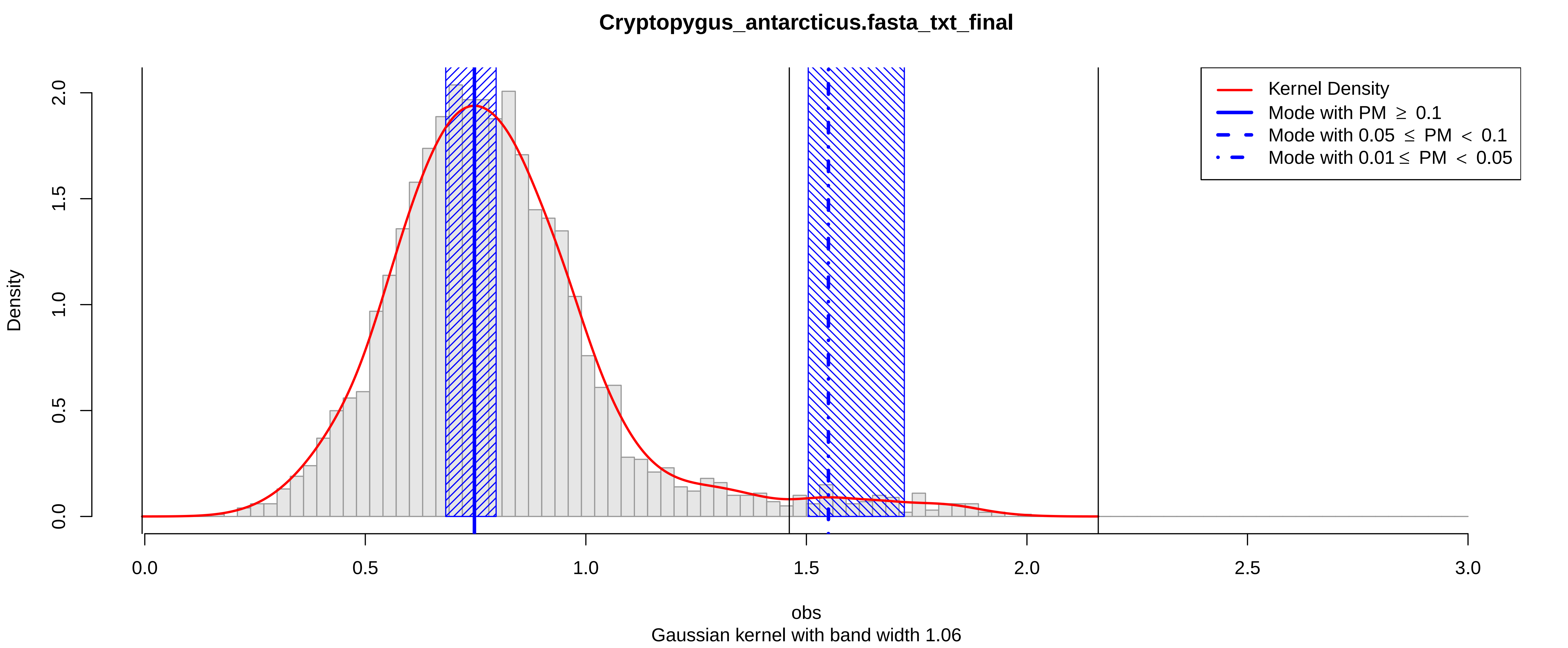

Cryptopygus_antarcticus.fasta_txt_final
Kernel Density
Mode with PM ≥ 0.1
Mode with 0.05 ≤ PM < 0.1
Mode with 0.01 ≤ PM < 0.05
Density
0.0
0.5
1.0
1.5
2.0
0.0
0.5
1.0
1.5
2.0
2.5
3.0
obs
Gaussian kernel with band width 1.06

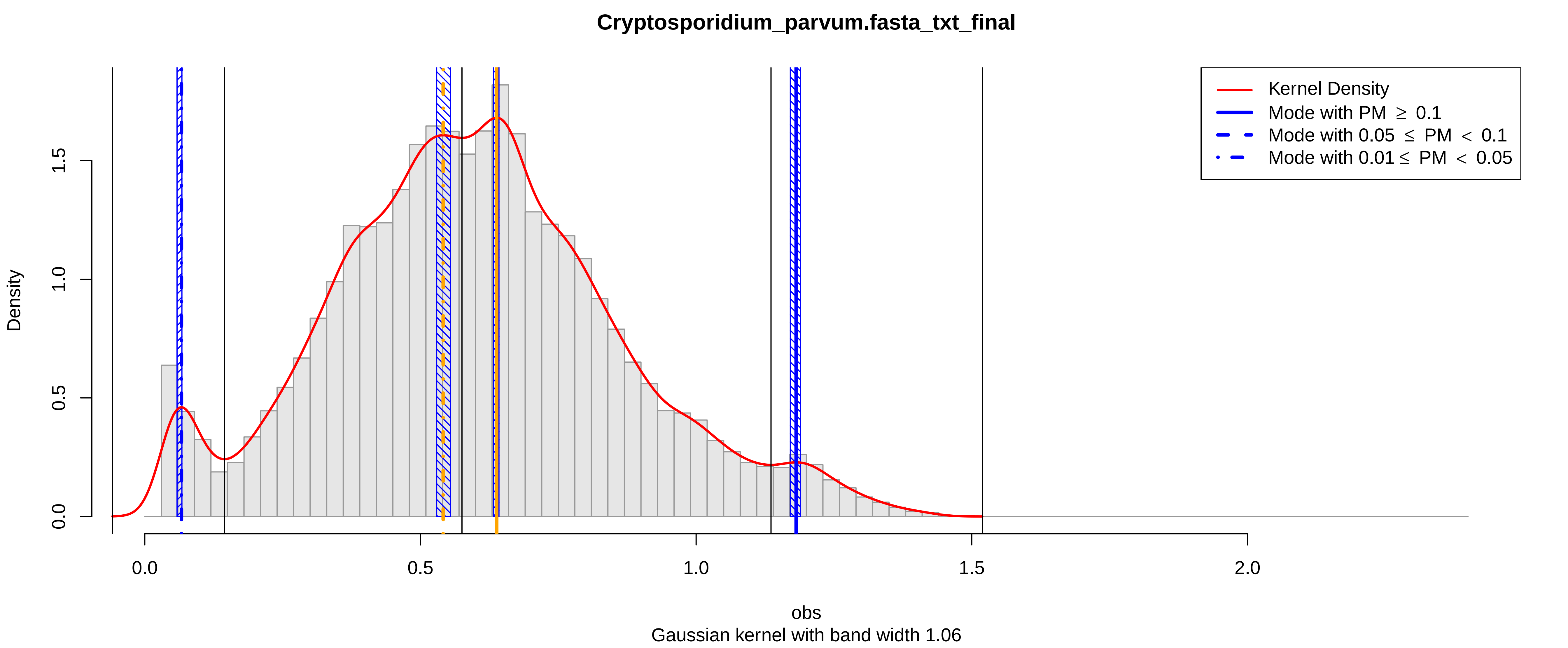
Cryptosporidium_parvum.fasta_txt_final
Kernel Density
Mode with PM ≥ 0.1
Mode with 0.05 ≤ PM < 0.1
Mode with 0.01 ≤ PM < 0.05
Density
0.0
0.5
1.0
1.5
0.0
0.5
1.0
1.5
2.0
obs
Gaussian kernel with band width 1.06

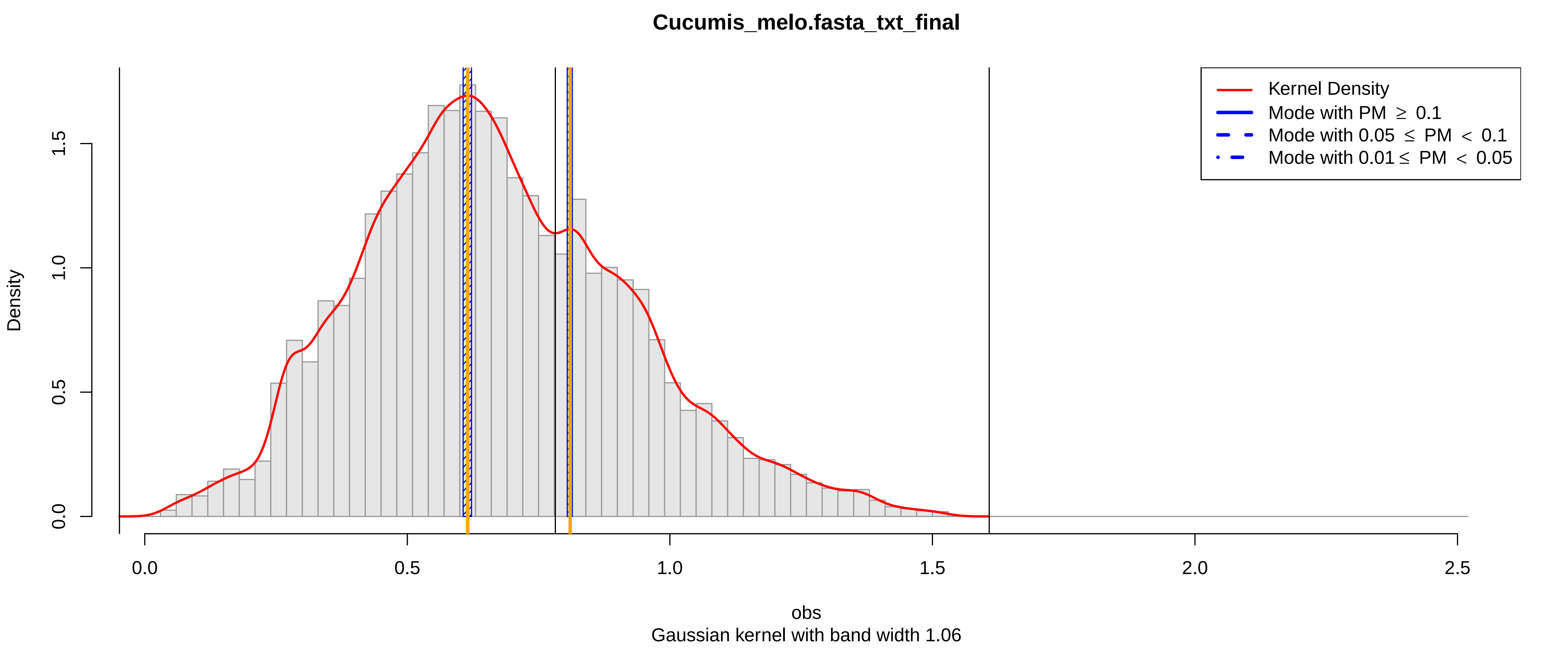

Cucumis_melo.fasta_txt_final
Kernel Density
Mode with PM ≥ 0.1
Mode with 0.05 ≤ PM < 0.1
Mode with 0.01 ≤ PM < 0.05
Density
0.0
0.5
1.0
1.5
2.0
2.5
obs
Gaussian kernel with band width 1.06

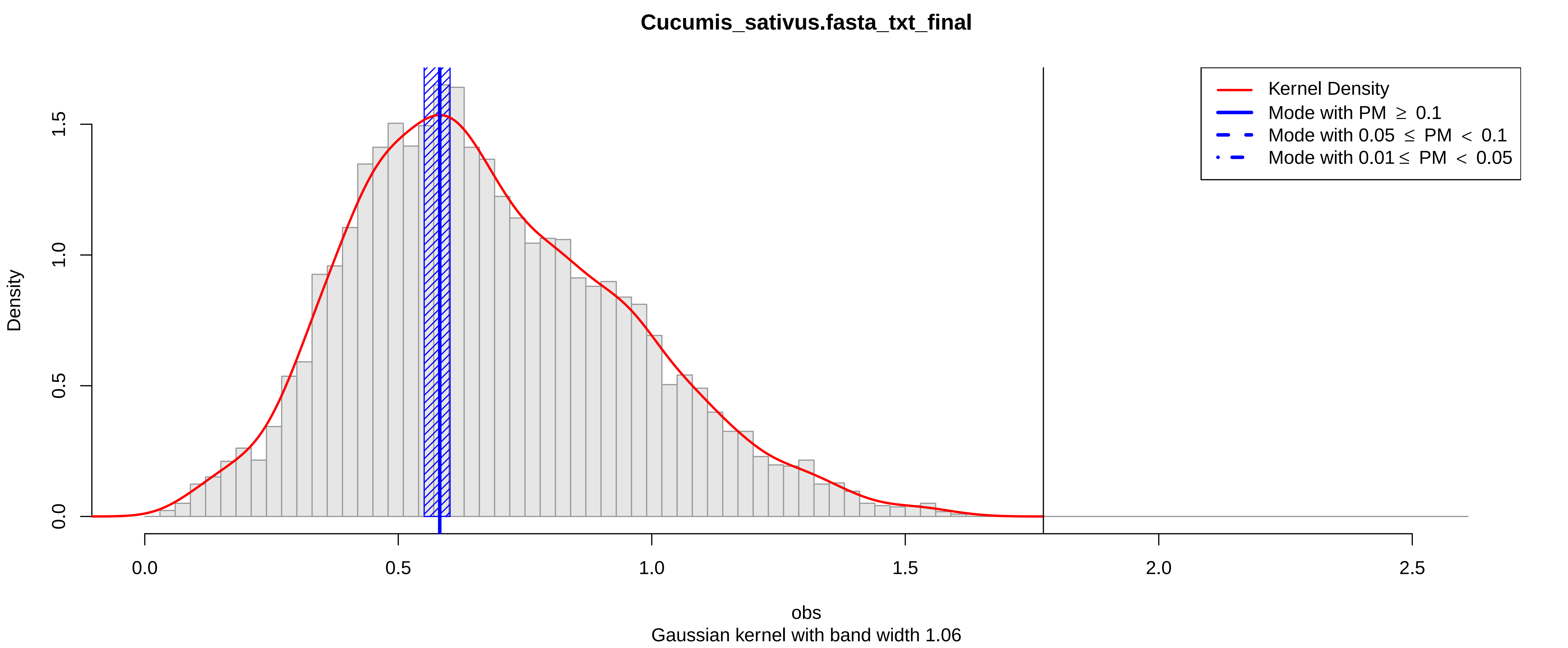
Cucumis_sativus.fasta_txt_final
Kernel Density
Mode with PM ≥ 0.1
Mode with 0.05 ≤ PM < 0.1
Mode with 0.01 ≤ PM < 0.05
Density
0.0
0.5
1.0
1.5
0.0
0.5
1.0
1.5
2.0
2.5
obs
Gaussian kernel with band width 1.06

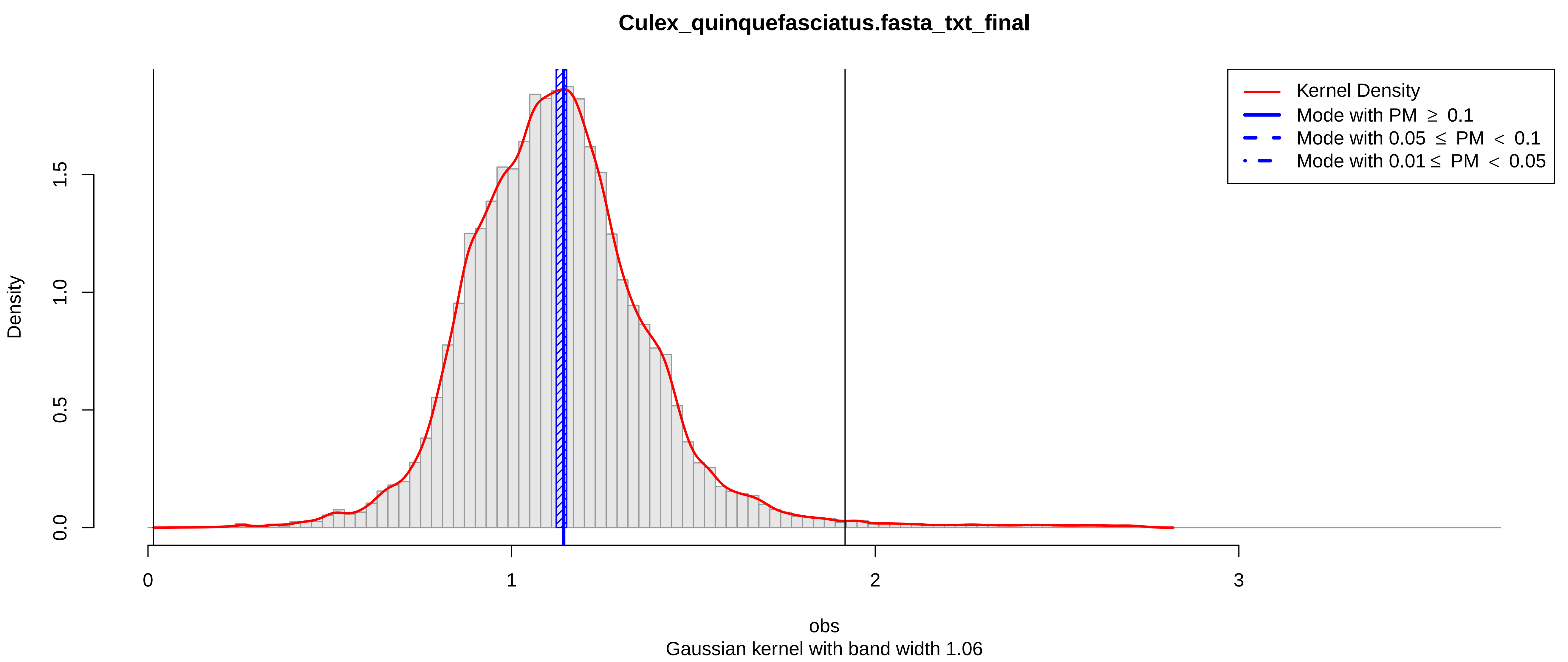

Culex_quinquefasciatus.fasta_txt_final
Kernel Density
Mode with PM ≥ 0.1
Mode with 0.05 ≤ PM < 0.1
Mode with 0.01 ≤ PM < 0.05
Density
0.0
0.5
1.0
1.5
0
1
2
3
obs
Gaussian kernel with band width 1.06

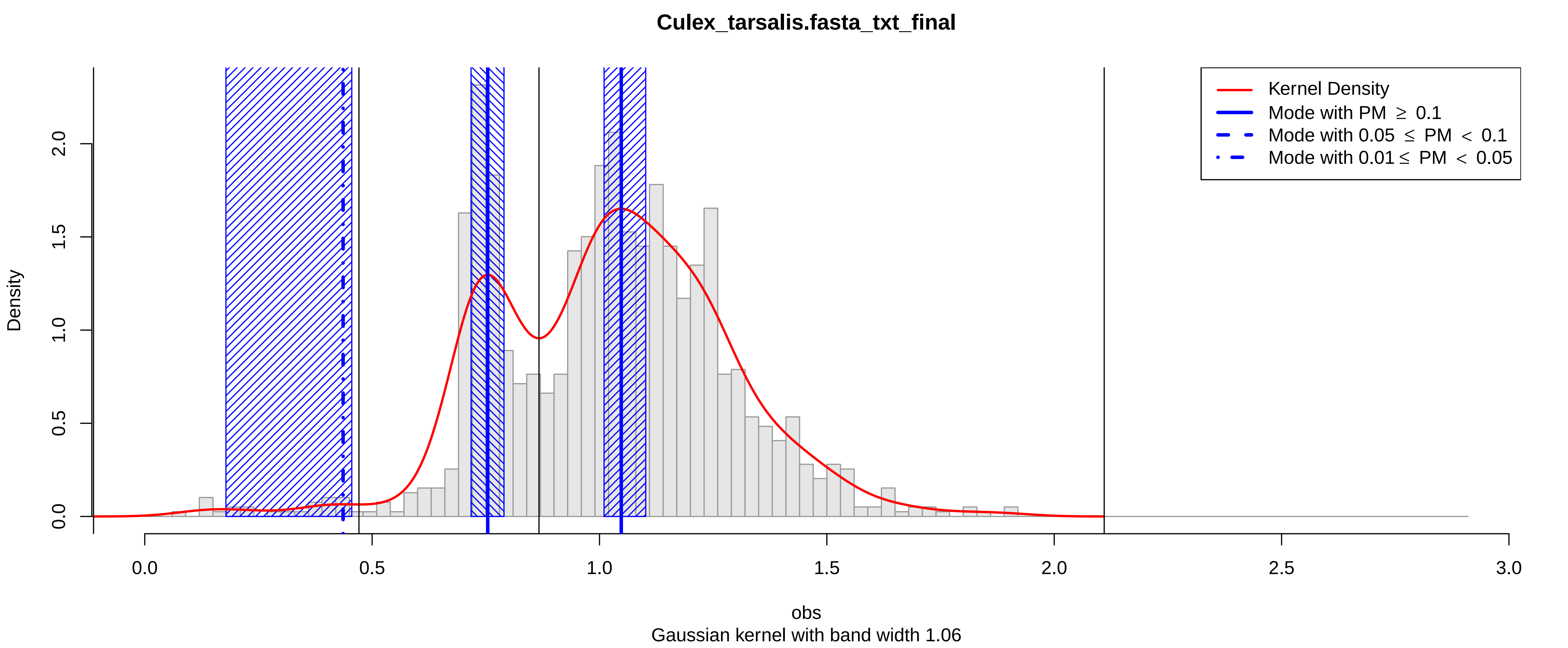

Culex_tarsalis.fasta_txt_final
Kernel Density
Mode with PM ≥ 0.1
Mode with 0.05 ≤ PM < 0.1
Mode with 0.01 ≤ PM < 0.05
Density
0.0
0.5
1.0
1.5
2.0
0.0
0.5
1.0
1.5
2.0
2.5
3.0
obs
Gaussian kernel with band width 1.06

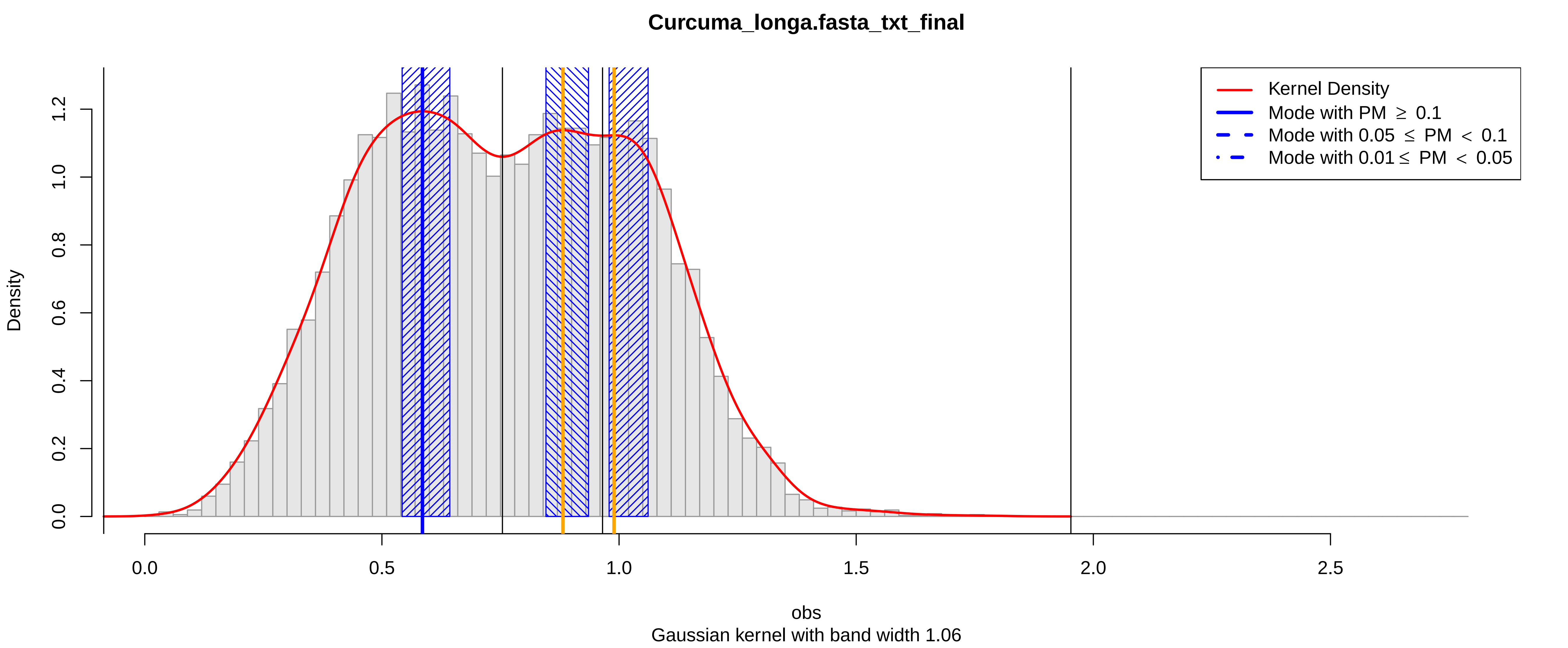

Curcuma_longa.fasta_txt_final
Kernel Density
Mode with PM ≥ 0.1
Mode with 0.05 ≤ PM < 0.1
Mode with 0.01 ≤ PM < 0.05
Density
0.0
0.2
0.4
0.6
0.8
1.0
1.2
0.0
0.5
1.0
1.5
2.0
2.5
obs
Gaussian kernel with band width 1.06

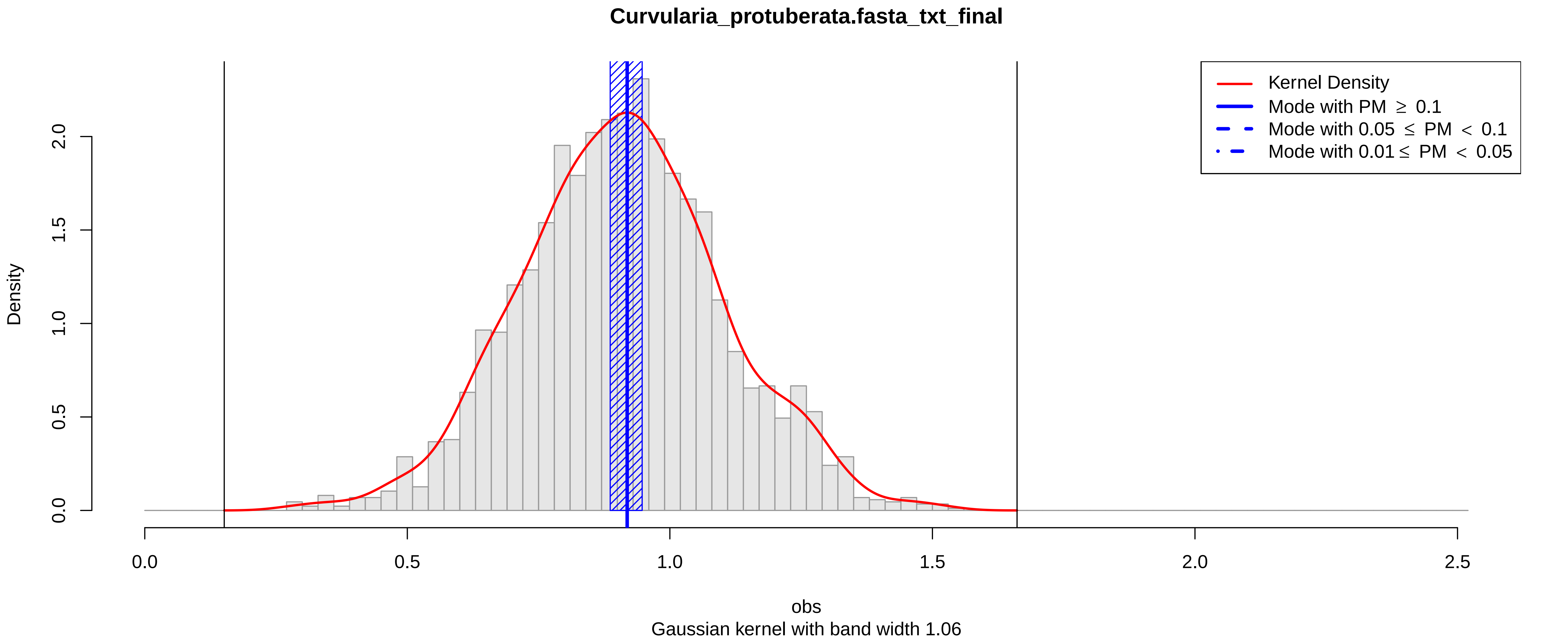

Curvularia_protuberata.fasta_txt_final
Kernel Density
Mode with PM ≥ 0.1
Mode with 0.05 ≤ PM < 0.1
Mode with 0.01 ≤ PM < 0.05
Density
0.0
0.5
1.0
1.5
2.0
0.0
0.5
1.0
1.5
2.0
2.5
obs
Gaussian kernel with band width 1.06

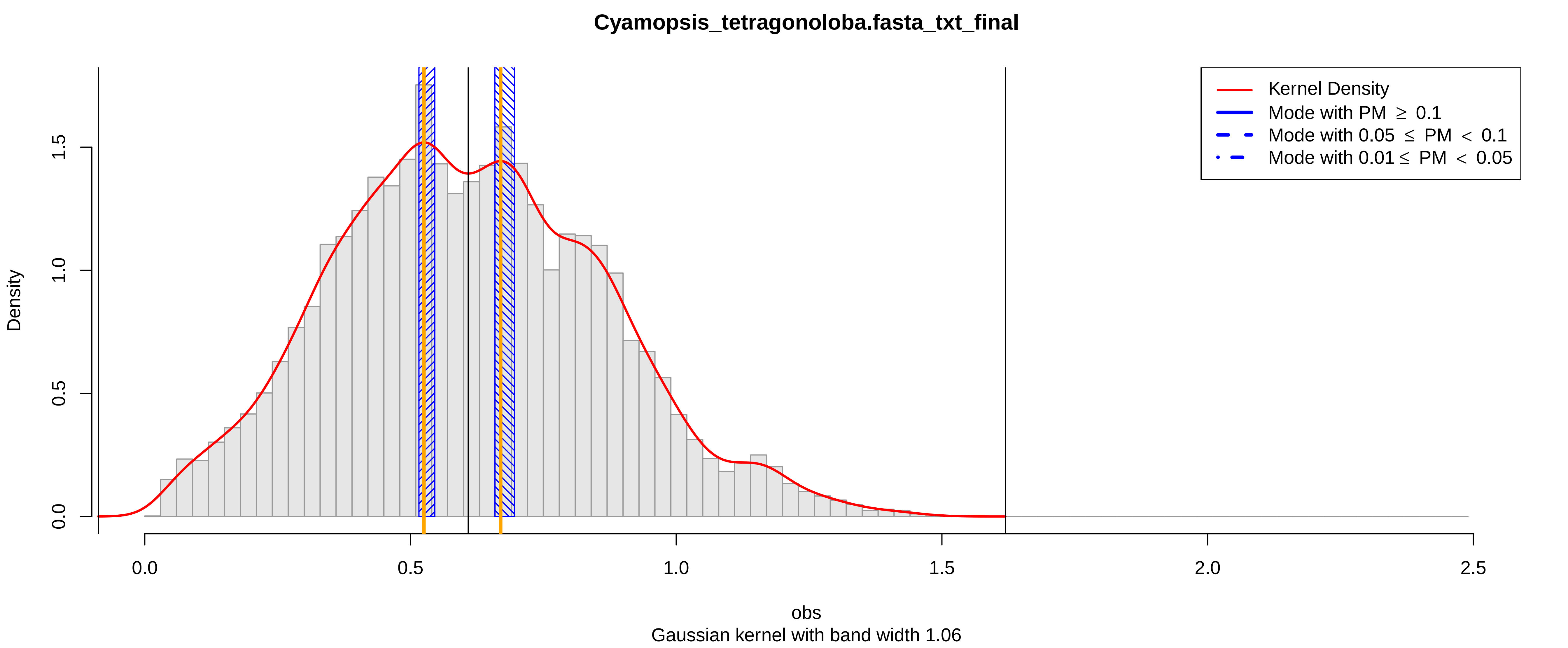

Cyamopsis_tetragonoloba.fasta_txt_final
Kernel Density
Mode with PM ≥ 0.1
Mode with 0.05 ≤ PM < 0.1
Mode with 0.01 ≤ PM < 0.05
Density
0.0
0.5
1.0
1.5
2.0
2.5
obs
Gaussian kernel with band width 1.06

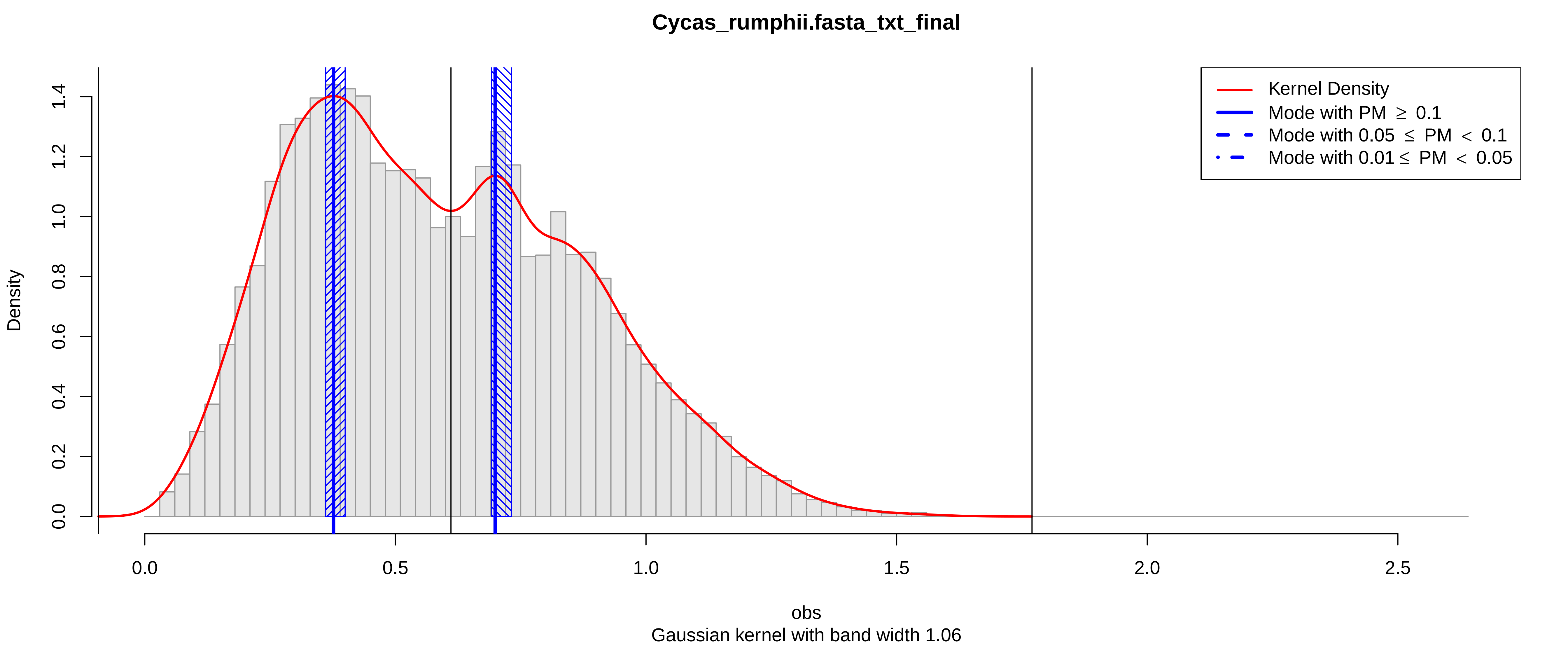

Cycas_rumphii.fasta_txt_final
Kernel Density
Mode with PM ≥ 0.1
Mode with 0.05 ≤ PM < 0.1
Mode with 0.01 ≤ PM < 0.05
Density
0.0
0.2
0.4
0.6
0.8
1.0
1.2
1.4
0.0
0.5
1.0
1.5
2.0
2.5
obs
Gaussian kernel with band width 1.06

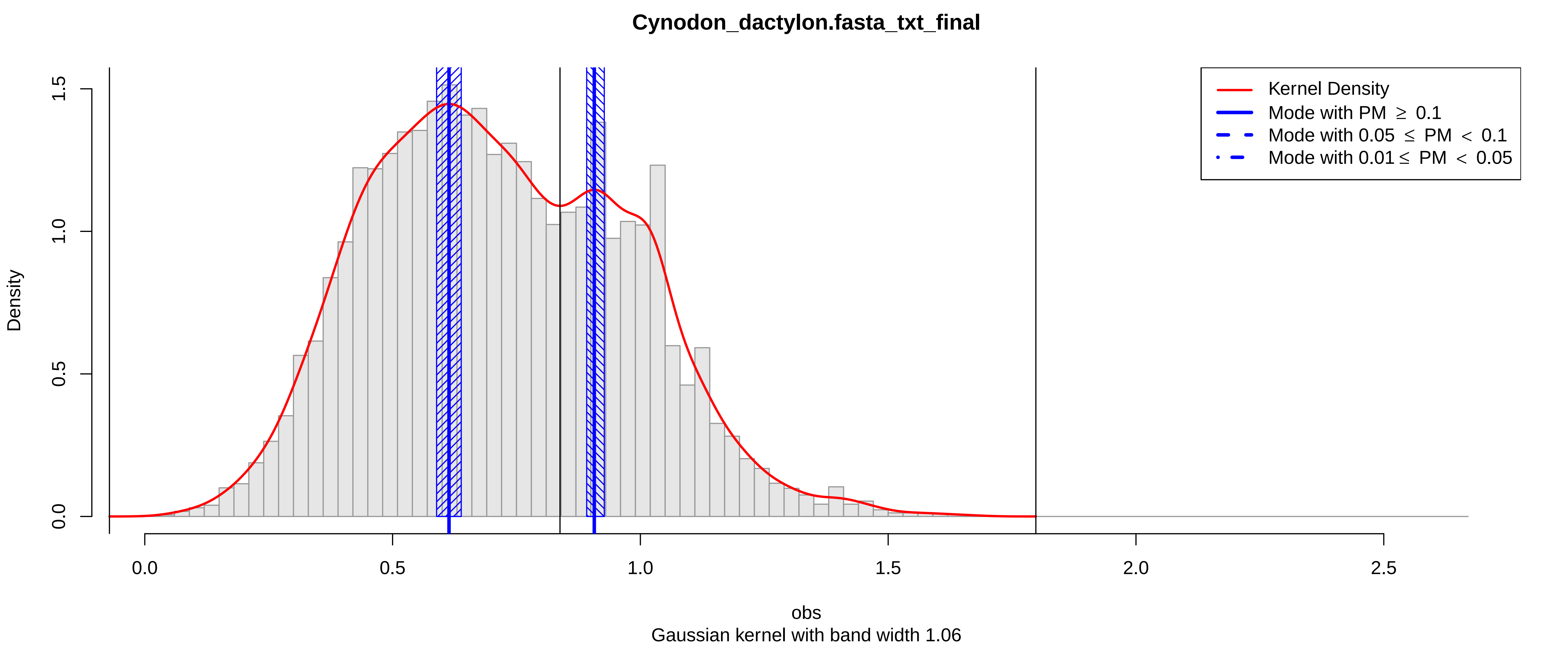

Cynodon_dactylon.fasta_txt_final
Kernel Density
Mode with PM ≥ 0.1
Mode with 0.05 ≤ PM < 0.1
Mode with 0.01 ≤ PM < 0.05
Density
0.0
0.5
1.0
1.5
0.0
0.5
1.0
1.5
2.0
2.5
obs
Gaussian kernel with band width 1.06

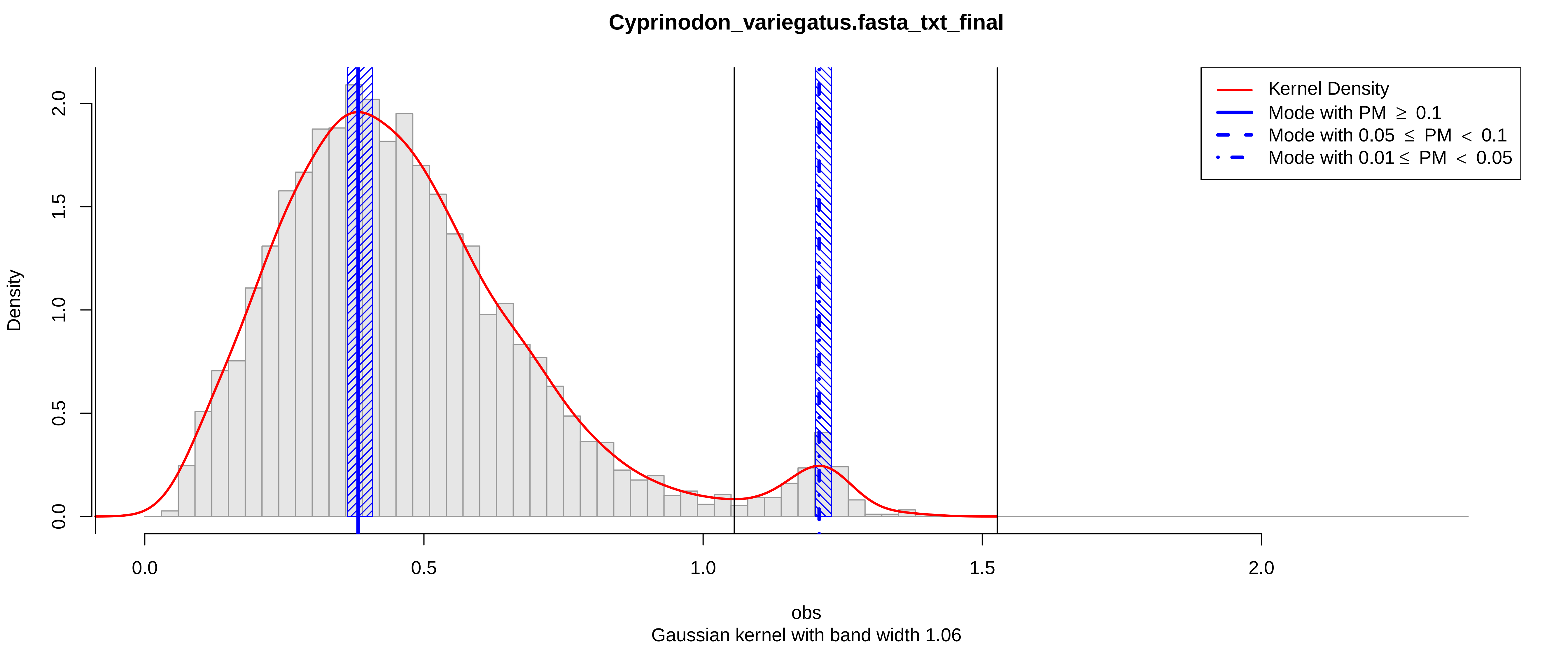
Cyprinodon_variegatus.fasta_txt_final
Kernel Density
Mode with PM ≥ 0.1
Mode with 0.05 ≤ PM < 0.1
Mode with 0.01 ≤ PM < 0.05
Density
0.0
0.5
1.0
1.5
2.0
obs
Gaussian kernel with band width 1.06

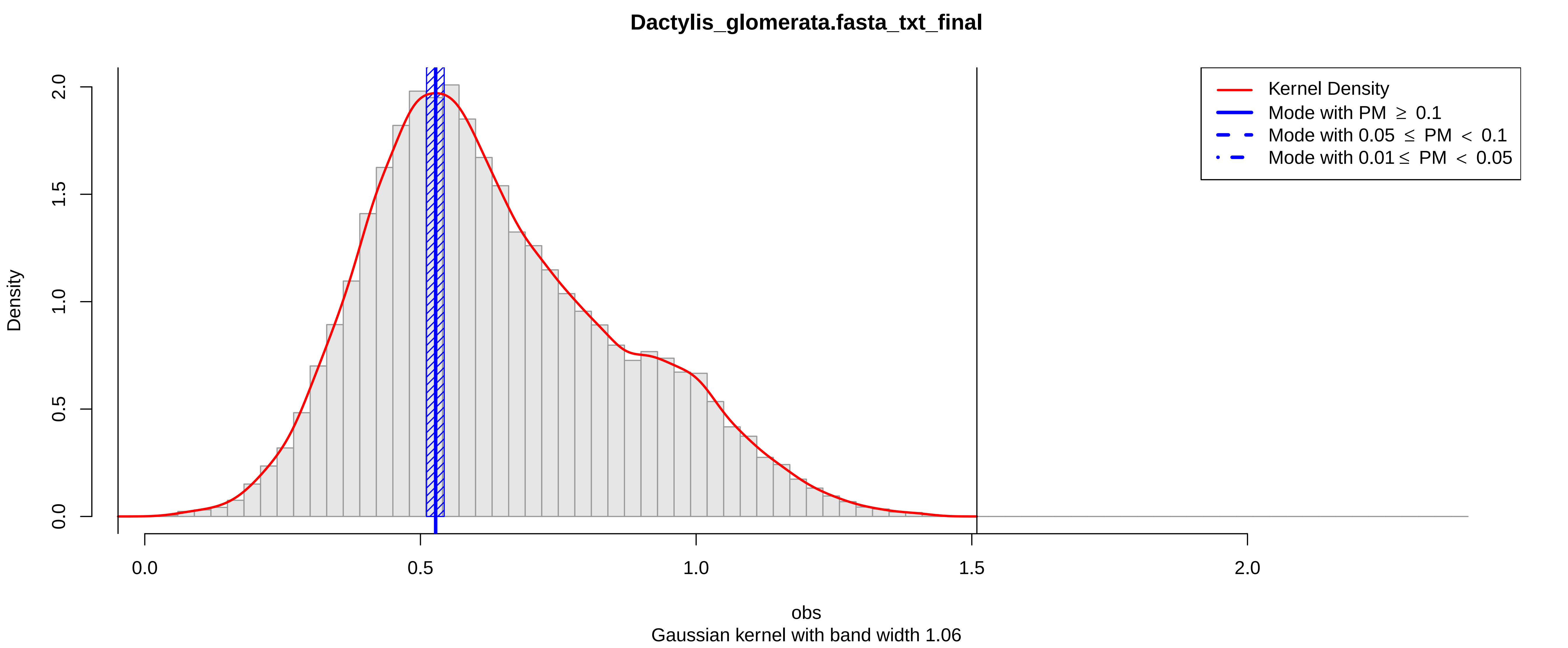

Dactylis_glomerata.fasta_txt_final
Kernel Density
Mode with PM ≥ 0.1
Mode with 0.05 ≤ PM < 0.1
Mode with 0.01 ≤ PM < 0.05
Density
0.0
0.5
1.0
1.5
2.0
0.0
0.5
1.0
1.5
2.0
obs
Gaussian kernel with band width 1.06

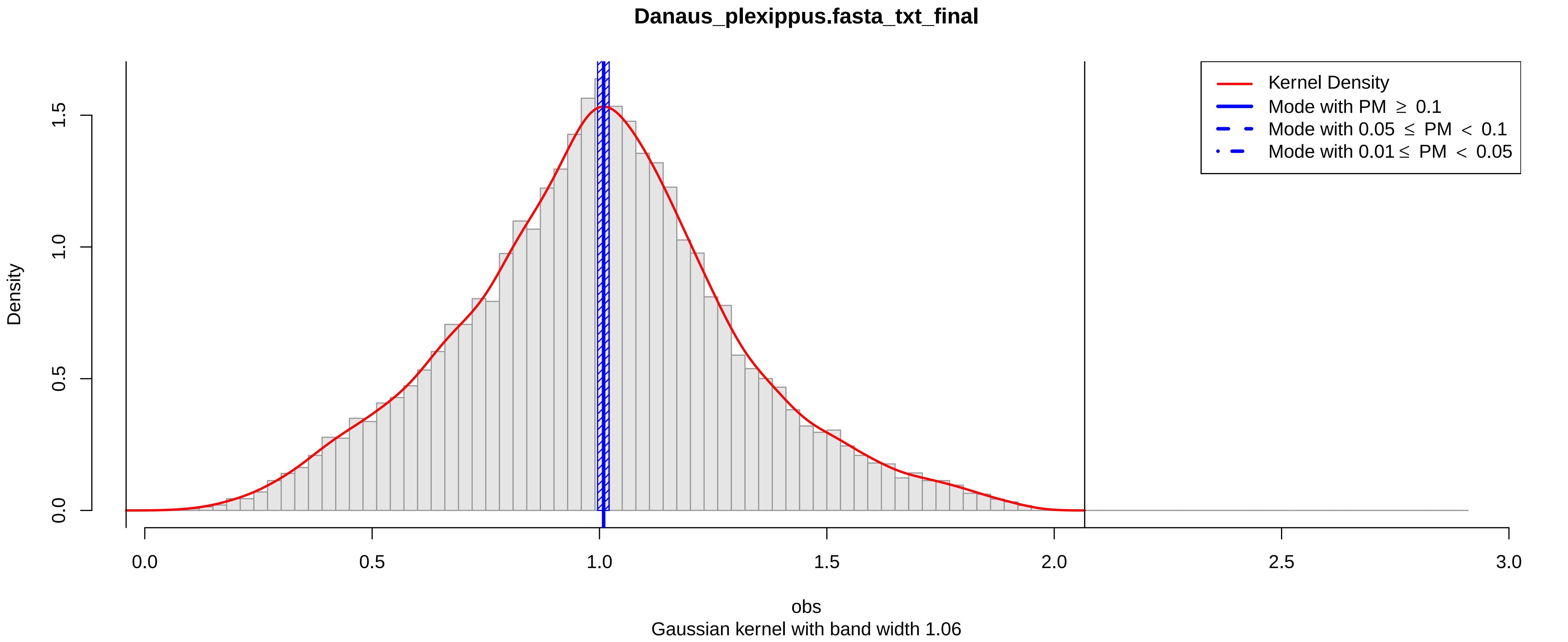
Danaus_plexippus.fasta_txt_final
Kernel Density
Mode with PM ≥ 0.1
Mode with 0.05 ≤ PM < 0.1
Mode with 0.01 ≤ PM < 0.05
Density
0.0
0.5
1.0
1.5
0.0
0.5
1.0
1.5
2.0
2.5
3.0
obs
Gaussian kernel with band width 1.06

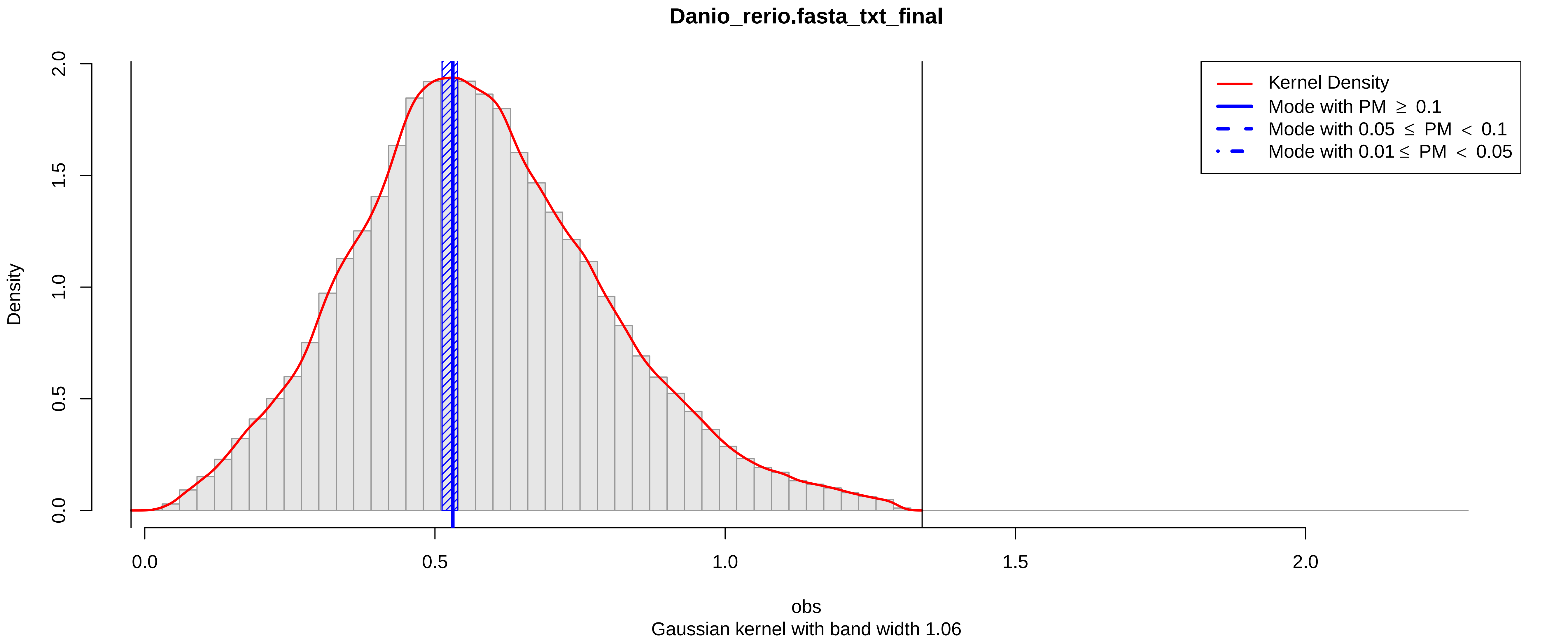
Danio_rerio.fasta_txt_final
Kernel Density
Mode with PM ≥ 0.1
Mode with 0.05 ≤ PM < 0.1
Mode with 0.01 ≤ PM < 0.05
Density
0.0
0.5
1.0
1.5
2.0
obs
Gaussian kernel with band width 1.06

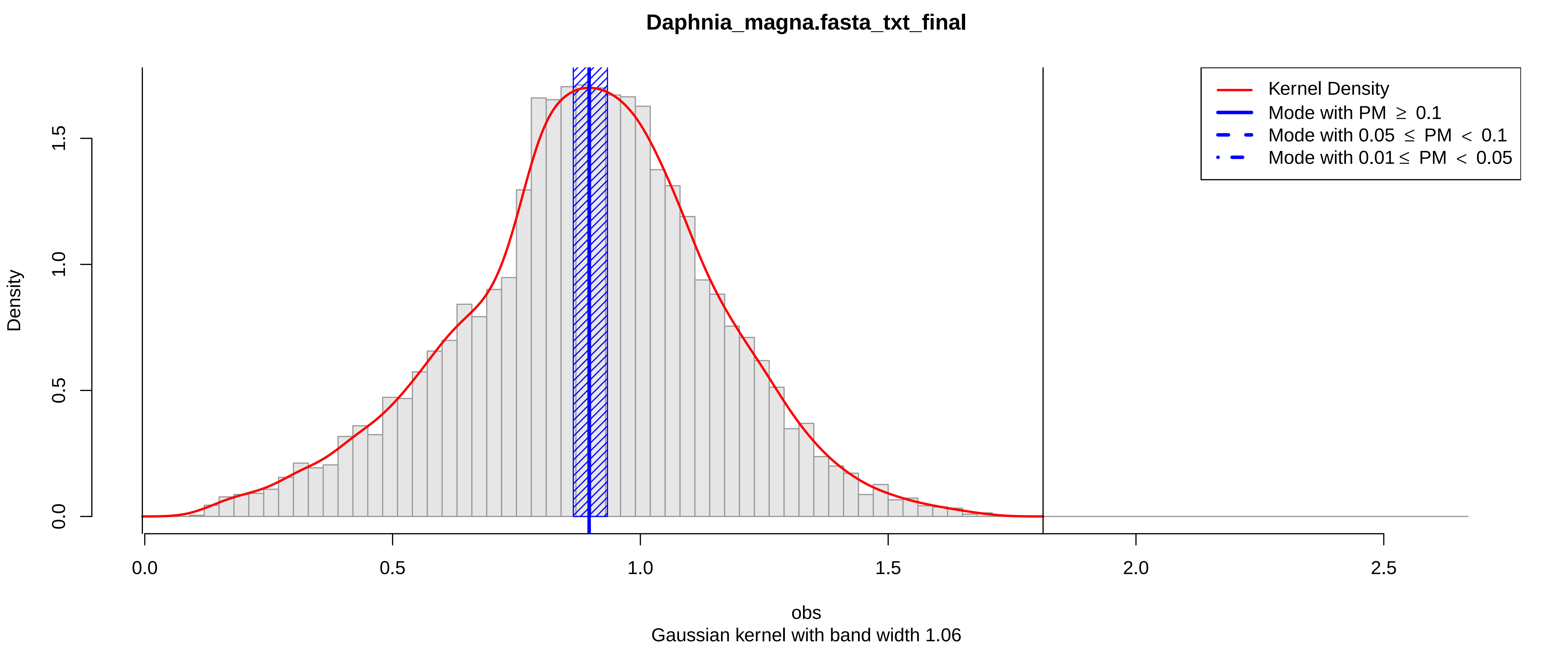

Daphnia_magna.fasta_txt_final
Kernel Density
Mode with PM ≥ 0.1
Mode with 0.05 ≤ PM < 0.1
Mode with 0.01 ≤ PM < 0.05
Density
0.0
0.5
1.0
1.5
0.0
0.5
1.0
1.5
2.0
2.5
obs
Gaussian kernel with band width 1.06

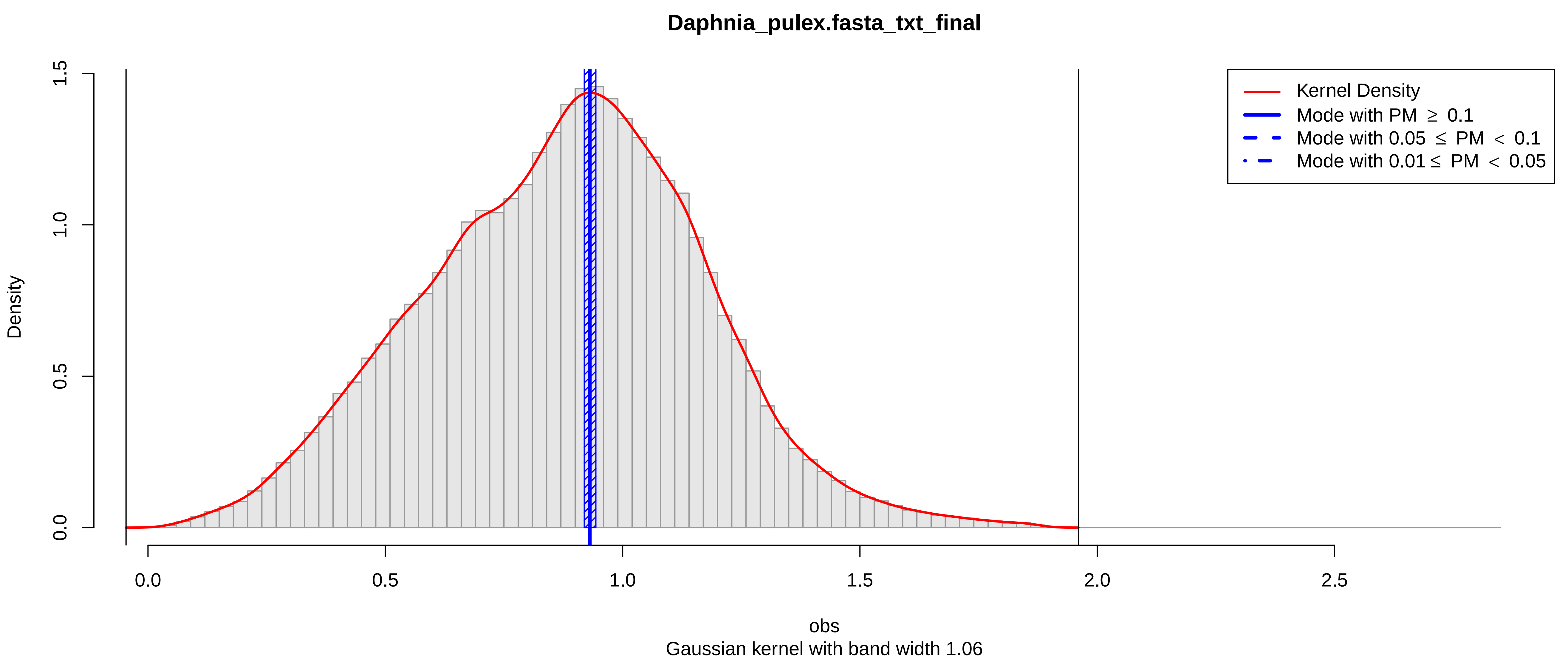

Daphnia_pulex.fasta_txt_final
Density
0.0
0.5
1.0
1.5
0.0
0.5
1.0
1.5
2.0
2.5
obs
Gaussian kernel with band width 1.06
Kernel Density
Mode with PM ≥ 0.1
Mode with 0.05 ≤ PM < 0.1
Mode with 0.01 ≤ PM < 0.05

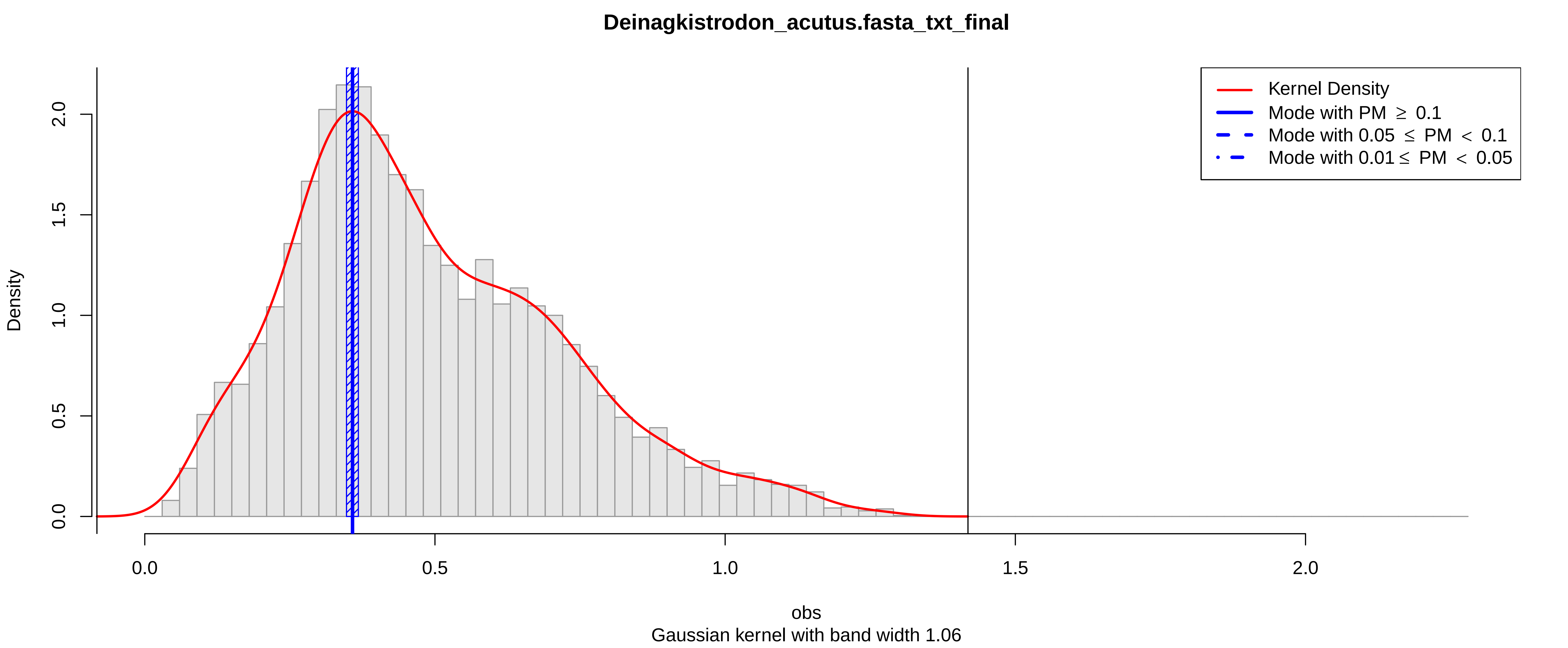

Deinagkistrodon_acutus.fasta_txt_final
Kernel Density
Mode with PM ≥ 0.1
Mode with 0.05 ≤ PM < 0.1
Mode with 0.01 ≤ PM < 0.05
Density
0.0
0.5
1.0
1.5
2.0
0.0
0.5
1.0
1.5
2.0
obs
Gaussian kernel with band width 1.06

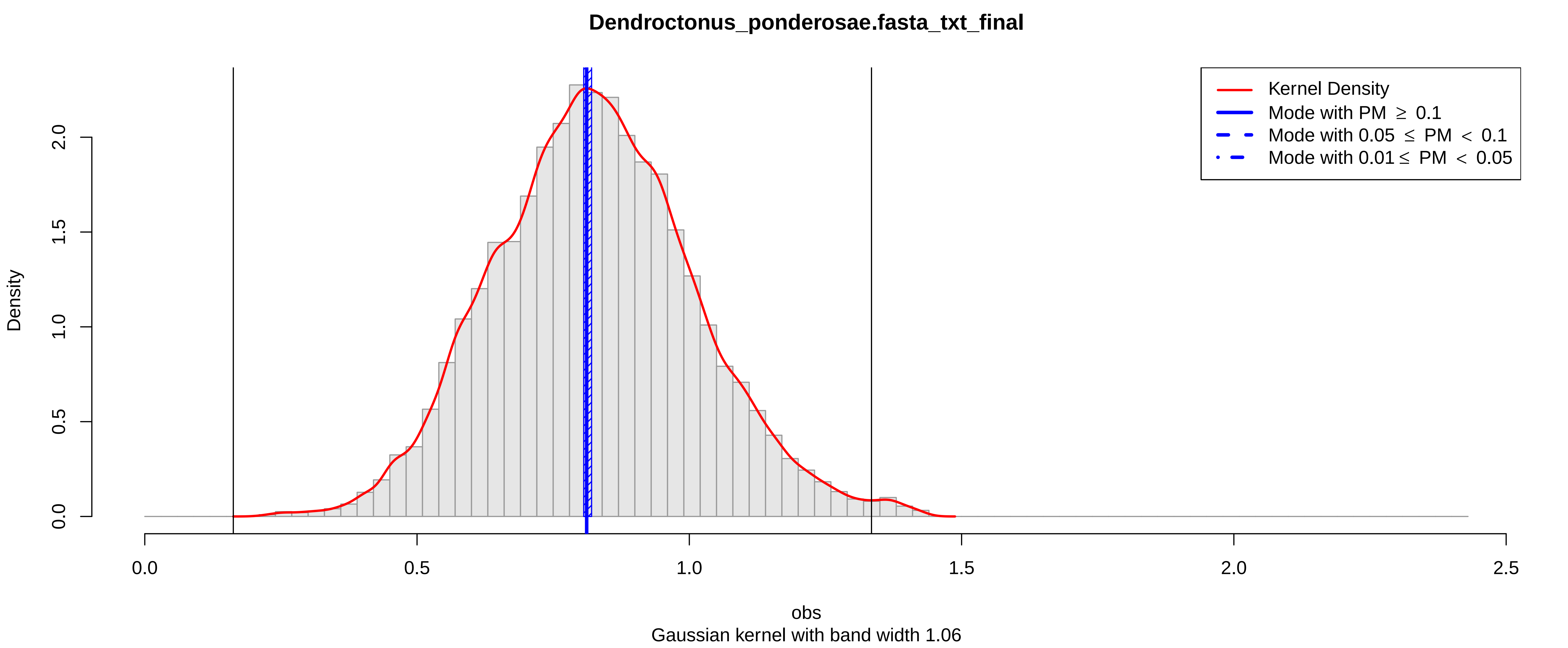
Dendroctonus_ponderosae.fasta_txt_final
Kernel Density
Mode with PM ≥ 0.1
Mode with 0.05 ≤ PM < 0.1
Mode with 0.01 ≤ PM < 0.05
Density
0.0
0.5
1.0
1.5
2.0
0.0
0.5
1.0
1.5
2.0
2.5
obs
Gaussian kernel with band width 1.06

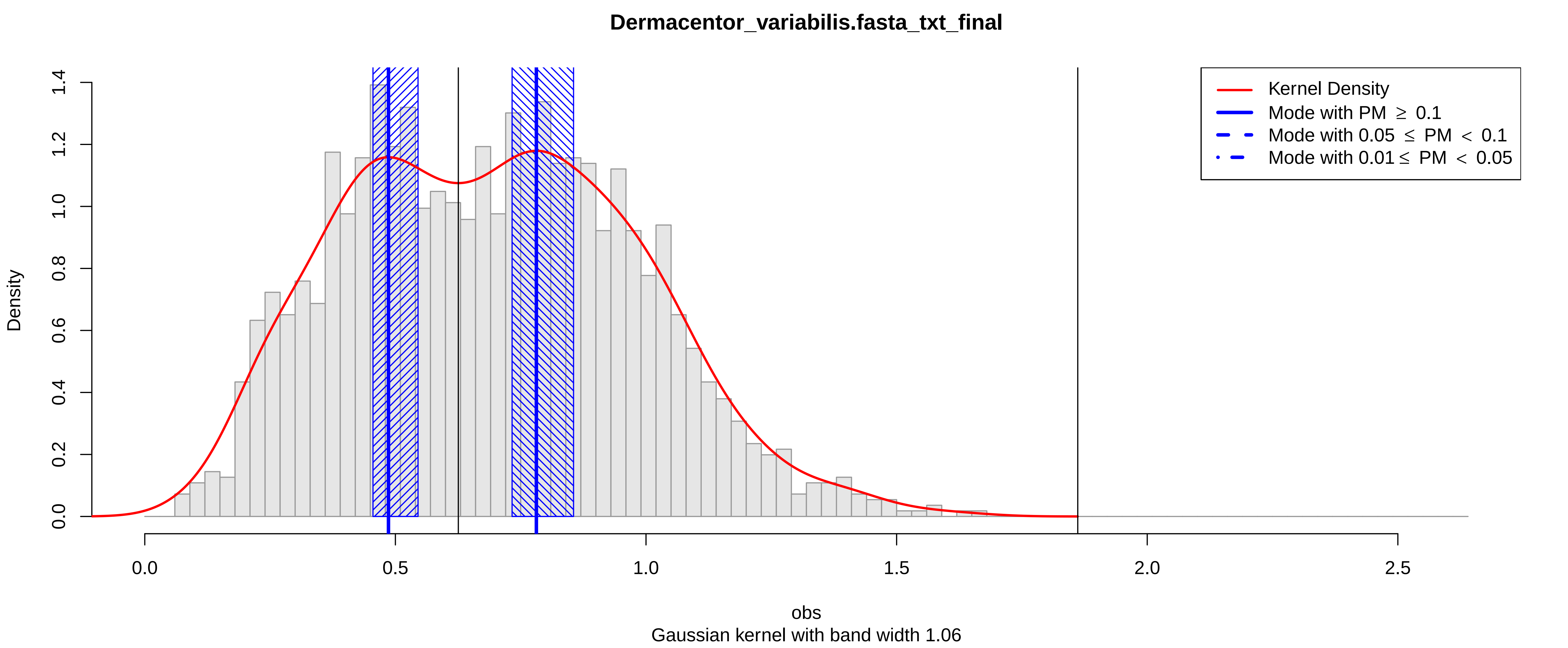

Dermacentor_variabilis.fasta_txt_final
Density
0.0
0.2
0.4
0.6
0.8
1.0
1.2
1.4
0.0
0.5
1.0
1.5
2.0
2.5
obs
Gaussian kernel with band width 1.06
Kernel Density
Mode with PM ≥ 0.1
Mode with 0.05 ≤ PM < 0.1
Mode with 0.01 ≤ PM < 0.05

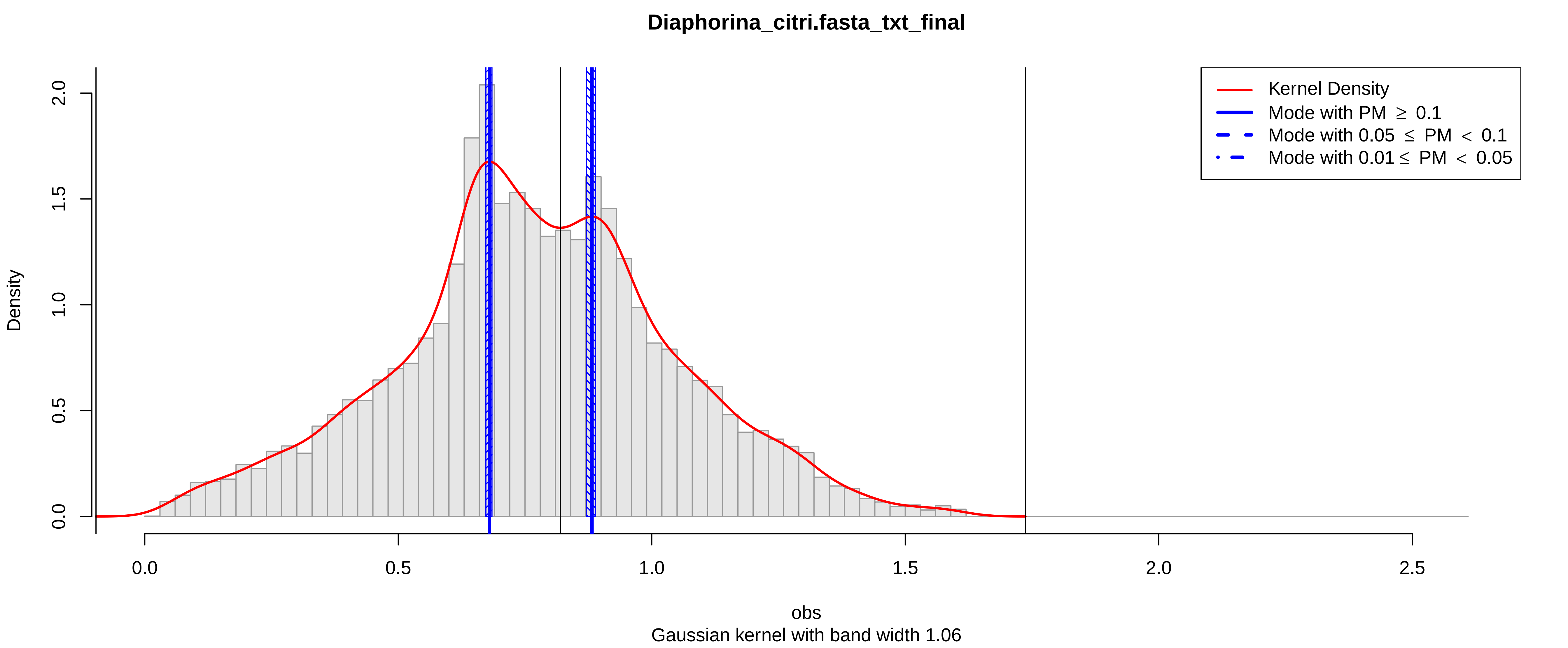

Diaphorina_citri.fasta_txt_final
Kernel Density
Mode with PM ≥ 0.1
Mode with 0.05 ≤ PM < 0.1
Mode with 0.01 ≤ PM < 0.05
Density
0.0
0.5
1.0
1.5
2.0
2.5
obs
Gaussian kernel with band width 1.06

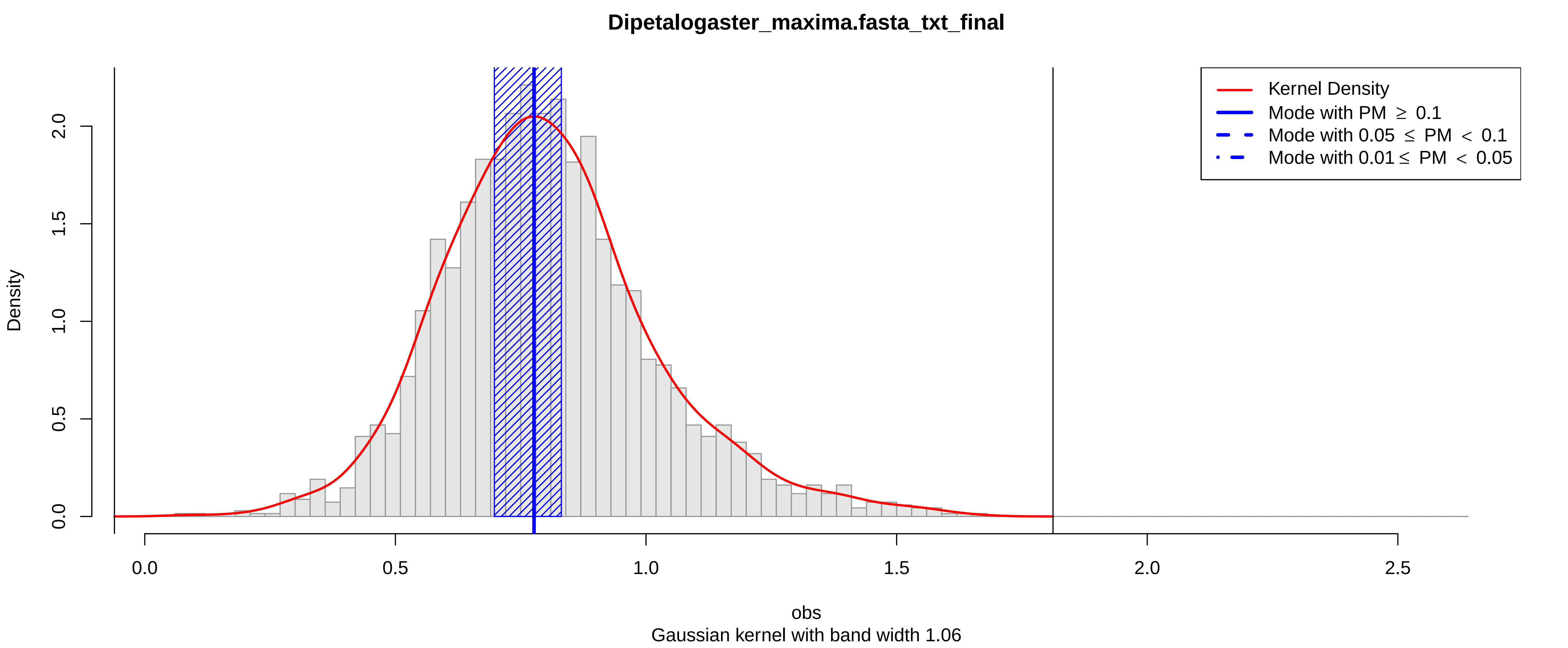
Dipetalogaster_maxima.fasta_txt_final
Kernel Density
Mode with PM ≥ 0.1
Mode with 0.05 ≤ PM < 0.1
Mode with 0.01 ≤ PM < 0.05
Density
0.0
0.5
1.0
1.5
2.0
0.0
0.5
1.0
1.5
2.0
2.5
obs
Gaussian kernel with band width 1.06

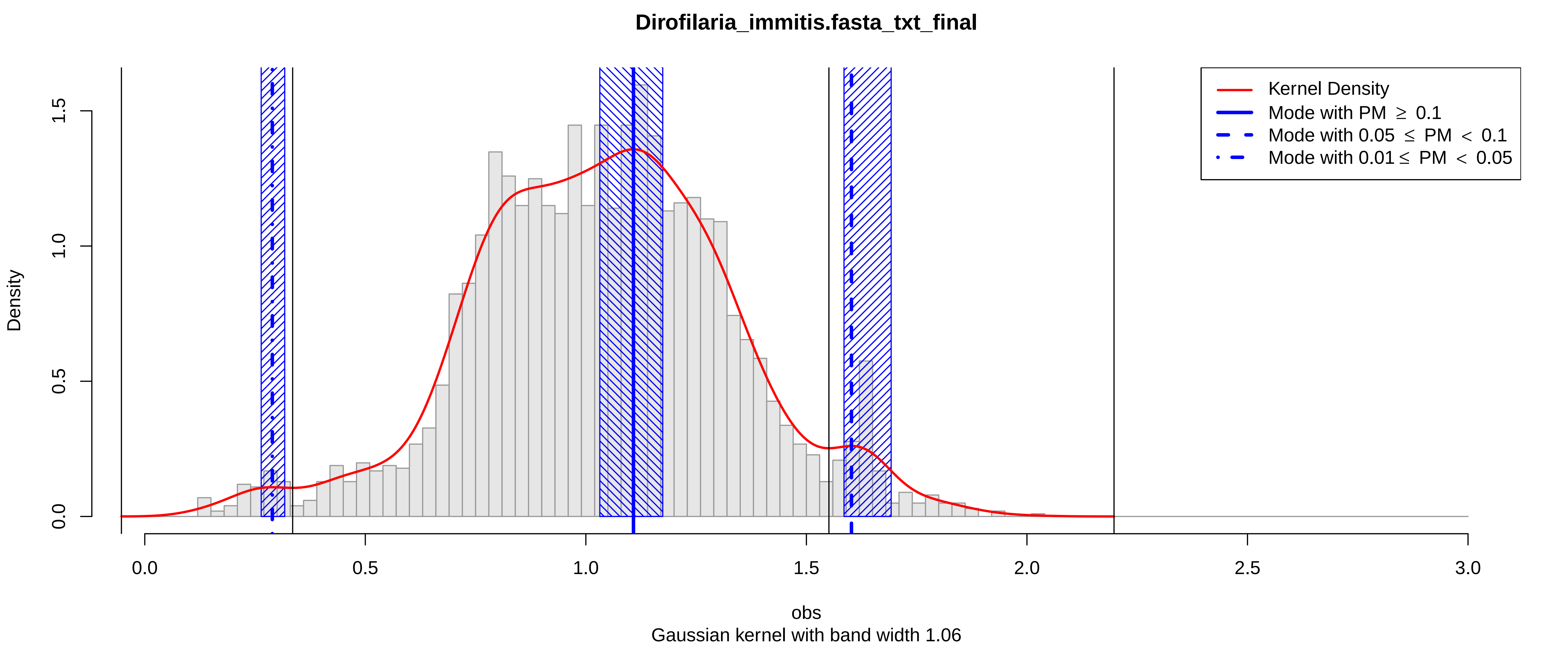

Dirofilaria_immitis.fasta_txt_final
Kernel Density
Mode with PM ≥ 0.1
Mode with 0.05 ≤ PM < 0.1
Mode with 0.01 ≤ PM < 0.05
Density
0.0
0.5
1.0
1.5
0.0
0.5
1.0
1.5
2.0
2.5
3.0
obs
Gaussian kernel with band width 1.06

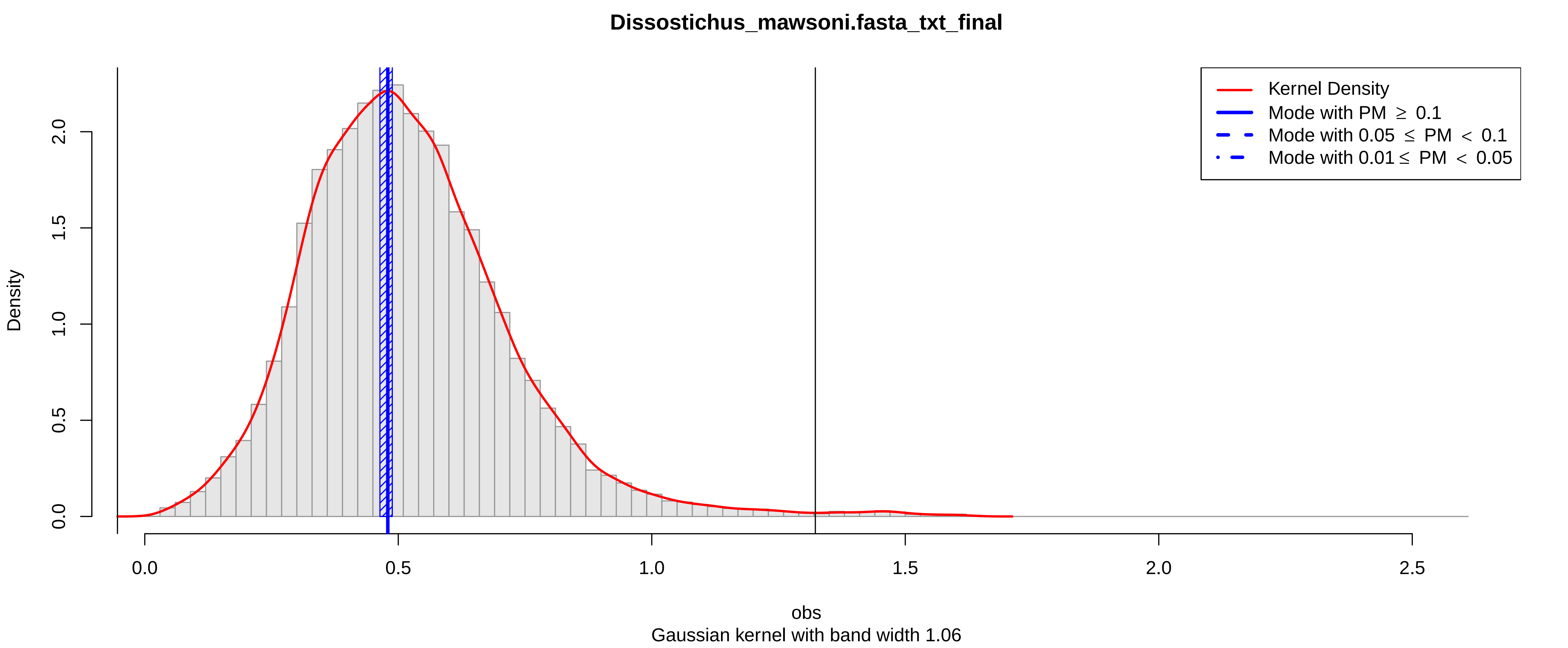
Dissostichus_mawsoni.fasta_txt_final
Kernel Density
Mode with PM ≥ 0.1
Mode with 0.05 ≤ PM < 0.1
Mode with 0.01 ≤ PM < 0.05
Density
0.0
0.5
1.0
1.5
2.0
0.0
0.5
1.0
1.5
2.0
2.5
obs
Gaussian kernel with band width 1.06

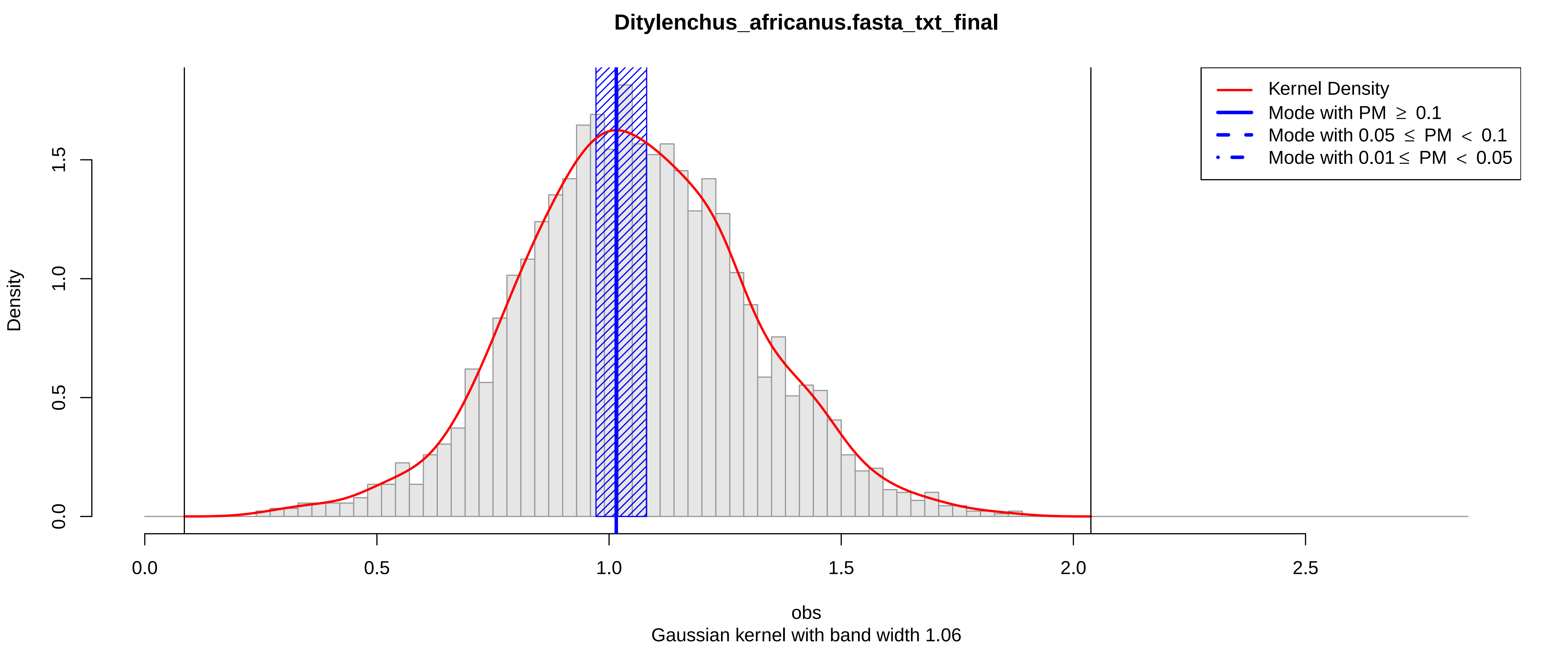

Ditylenchus_africanus.fasta_txt_final
Kernel Density
Mode with PM ≥ 0.1
Mode with 0.05 ≤ PM < 0.1
Mode with 0.01 ≤ PM < 0.05
Density
0.0
0.5
1.0
1.5
0.0
0.5
1.0
1.5
2.0
2.5
obs
Gaussian kernel with band width 1.06

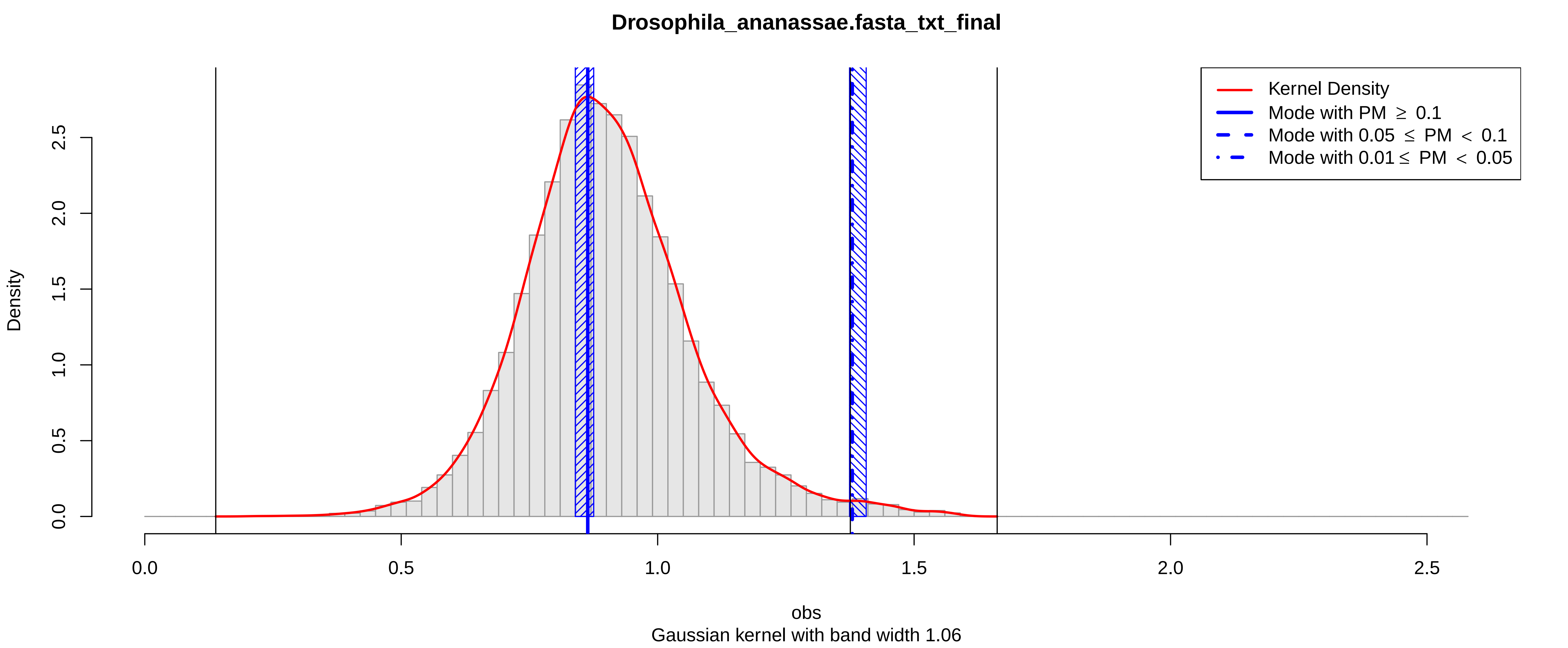
Drosophila_ananassae.fasta_txt_final
Kernel Density
Mode with PM ≥ 0.1
Mode with 0.05 ≤ PM < 0.1
Mode with 0.01 ≤ PM < 0.05
Density
0.0
0.5
1.0
1.5
2.0
2.5
0.0
0.5
1.0
1.5
2.0
2.5
obs
Gaussian kernel with band width 1.06

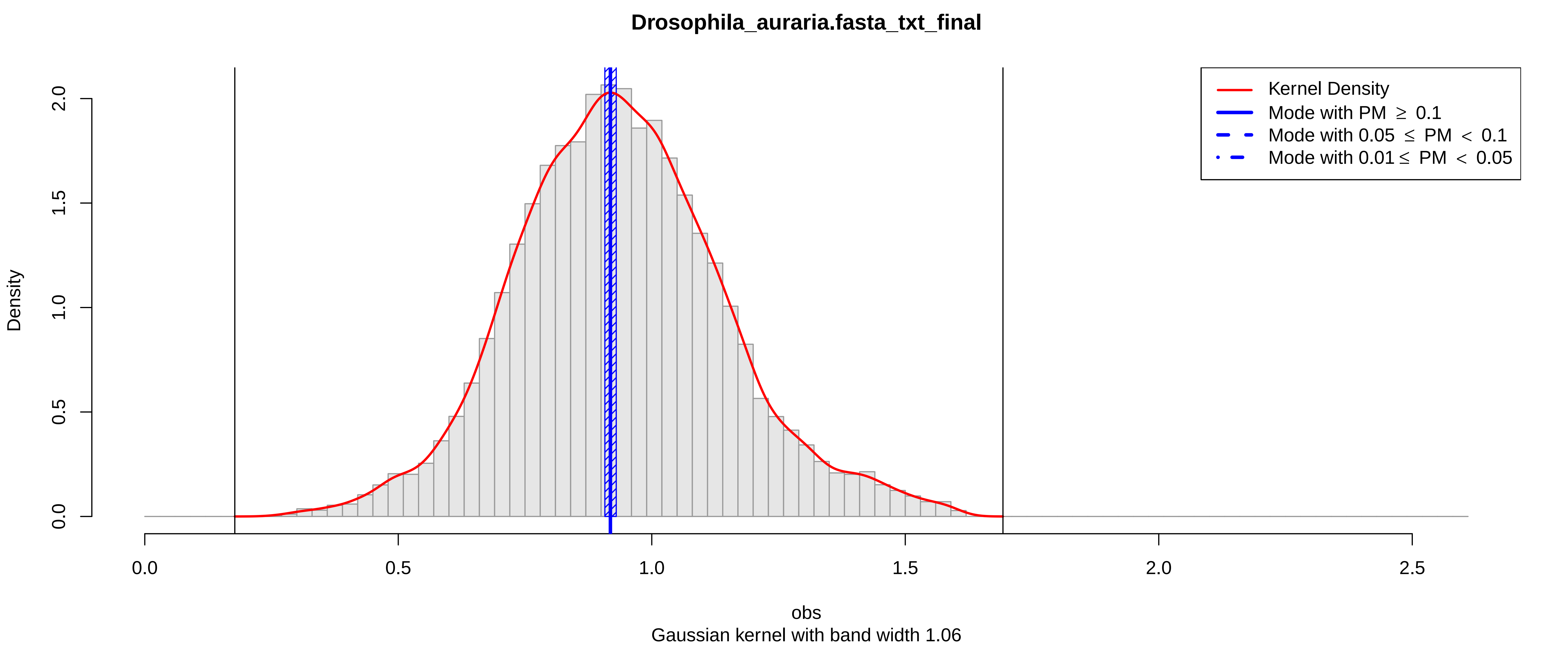

Drosophila_auraria.fasta_txt_final
Kernel Density
Mode with PM ≥ 0.1
Mode with 0.05 ≤ PM < 0.1
Mode with 0.01 ≤ PM < 0.05
Density
0.0
0.5
1.0
1.5
2.0
0.0
0.5
1.0
1.5
2.0
2.5
obs
Gaussian kernel with band width 1.06

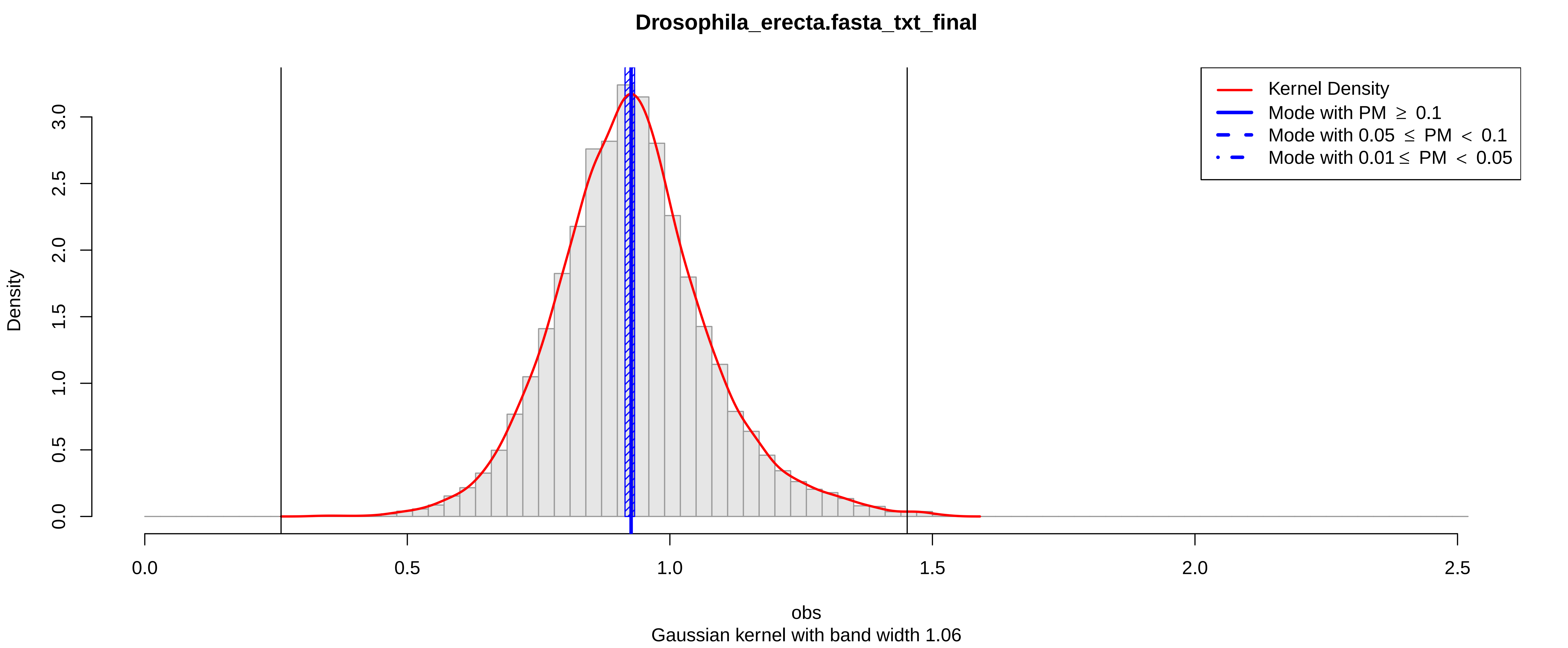

Drosophila_erecta.fasta_txt_final
Kernel Density
Mode with PM ≥ 0.1
Mode with 0.05 ≤ PM < 0.1
Mode with 0.01 ≤ PM < 0.05
Density
0.0
0.5
1.0
1.5
2.0
2.5
3.0
0.0
0.5
1.0
1.5
2.0
2.5
obs
Gaussian kernel with band width 1.06

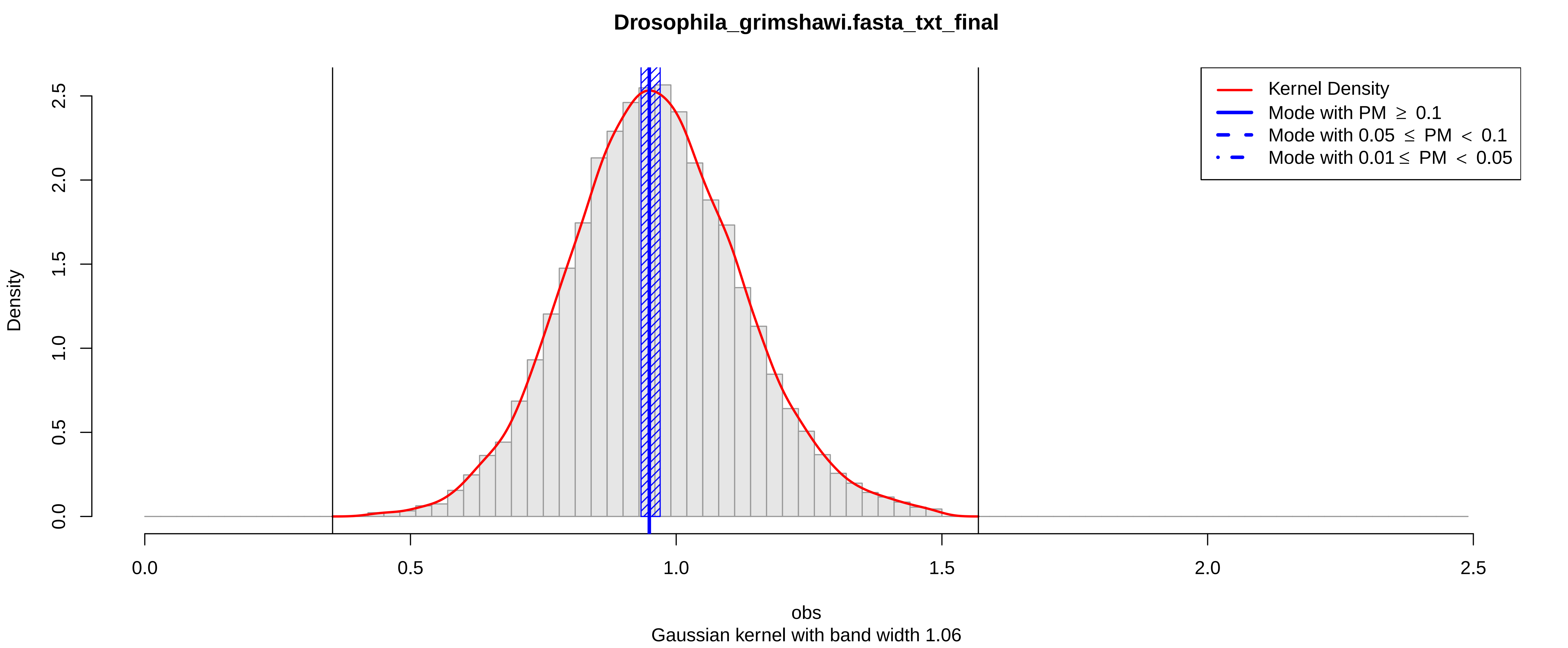

Drosophila_grimshawi.fasta_txt_final
Kernel Density
Mode with PM ≥ 0.1
Mode with 0.05 ≤ PM < 0.1
Mode with 0.01 ≤ PM < 0.05
Density
0.0
0.5
1.0
1.5
2.0
2.5
obs
Gaussian kernel with band width 1.06

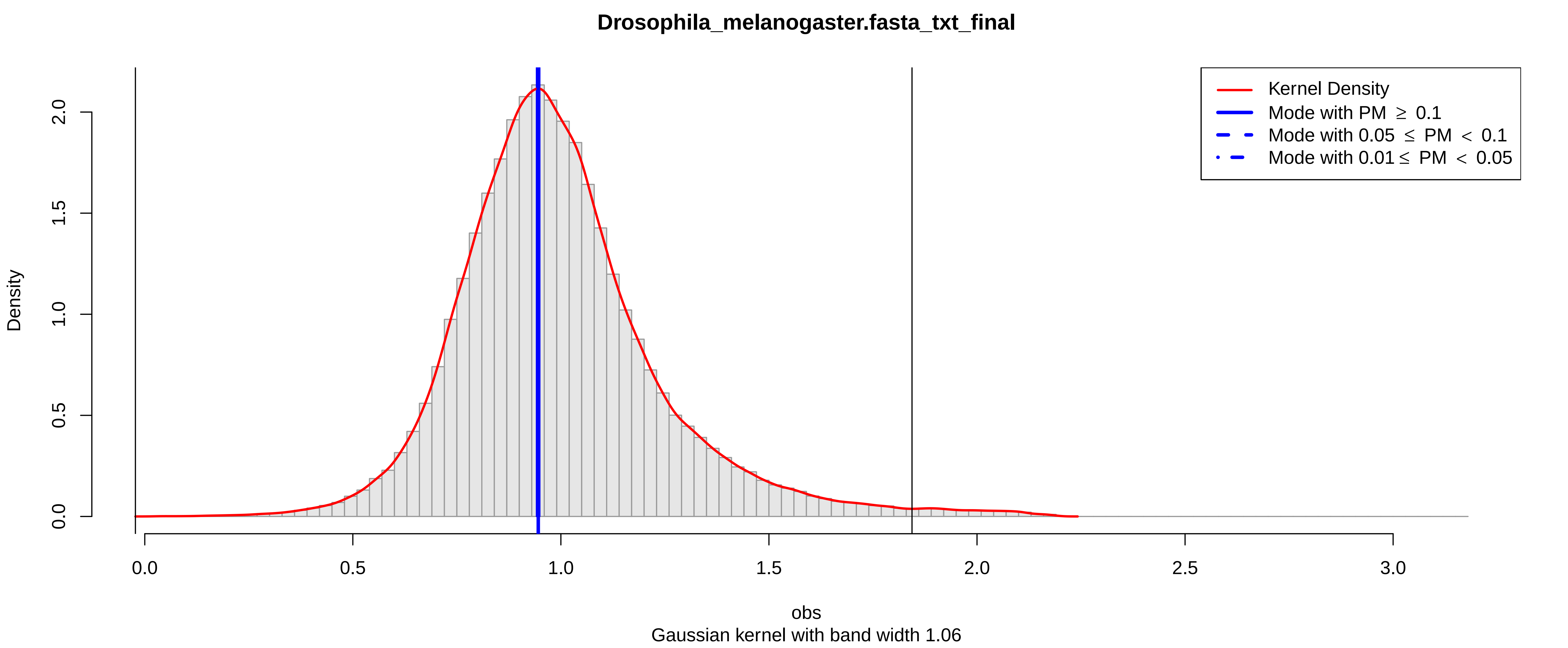

Drosophila_melanogaster.fasta_txt_final
Kernel Density
Mode with PM ≥ 0.1
Mode with 0.05 ≤ PM < 0.1
Mode with 0.01 ≤ PM < 0.05
Density
0.0
0.5
1.0
1.5
2.0
0.0
0.5
1.0
1.5
2.0
2.5
3.0
obs
Gaussian kernel with band width 1.06

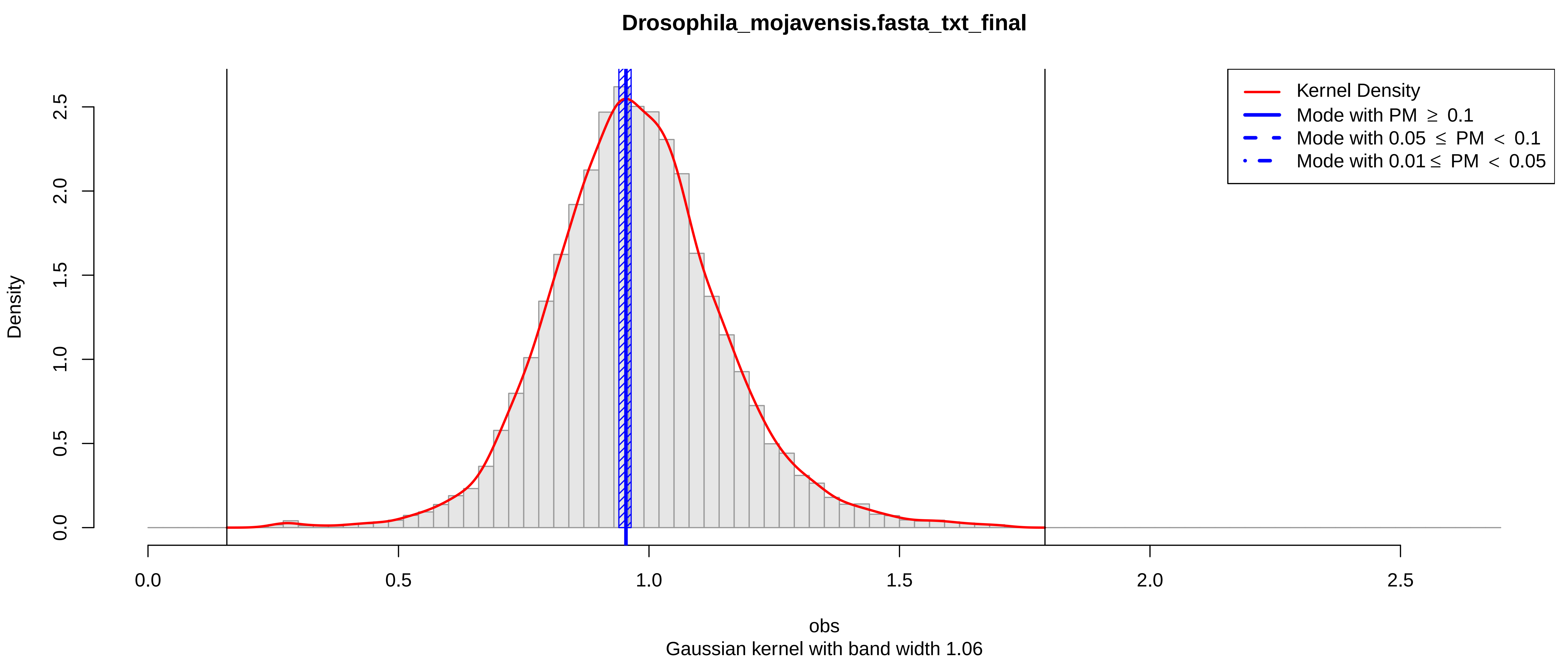

Drosophila_mojavensis.fasta_txt_final
Kernel Density
Mode with PM ≥ 0.1
Mode with 0.05 ≤ PM < 0.1
Mode with 0.01 ≤ PM < 0.05
Density
0.0
0.5
1.0
1.5
2.0
2.5
obs
Gaussian kernel with band width 1.06

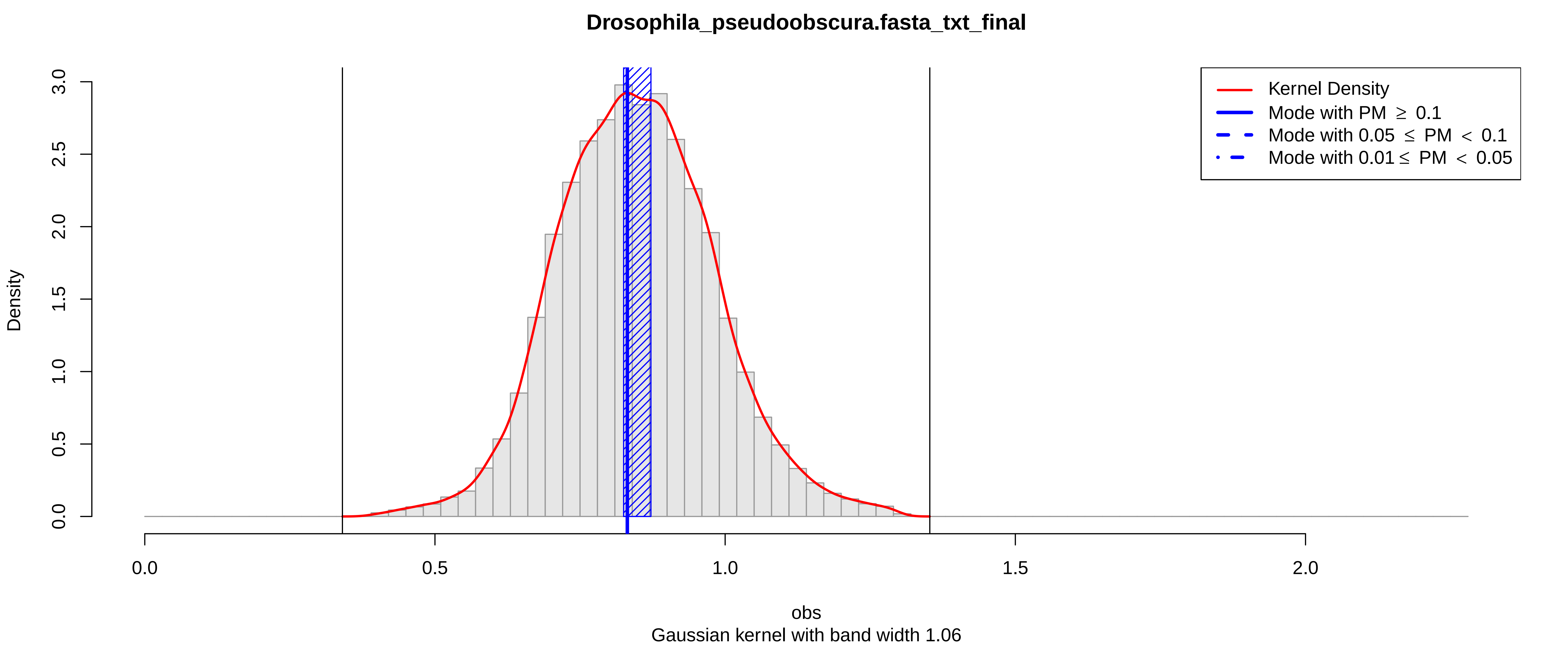

Drosophila_pseudoobscura.fasta_txt_final
Density
0.0
0.5
1.0
1.5
2.0
2.5
3.0
0.0
0.5
1.0
1.5
2.0
obs
Gaussian kernel with band width 1.06
Kernel Density
Mode with PM ≥ 0.1
Mode with 0.05 ≤ PM < 0.1
Mode with 0.01 ≤ PM < 0.05

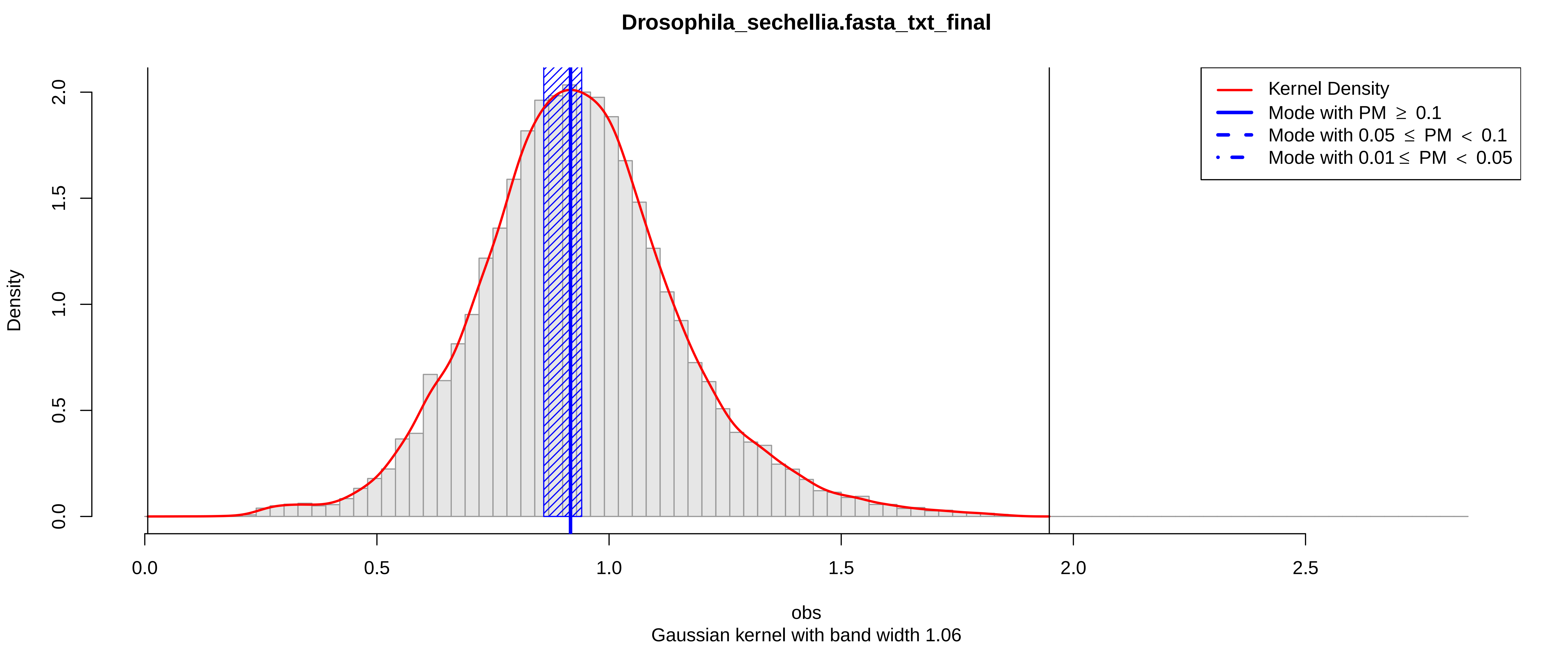

Drosophila_sechellia.fasta_txt_final
Kernel Density
Mode with PM ≥ 0.1
Mode with 0.05 ≤ PM < 0.1
Mode with 0.01 ≤ PM < 0.05
Density
0.0
0.5
1.0
1.5
2.0
0.0
0.5
1.0
1.5
2.0
2.5
obs
Gaussian kernel with band width 1.06

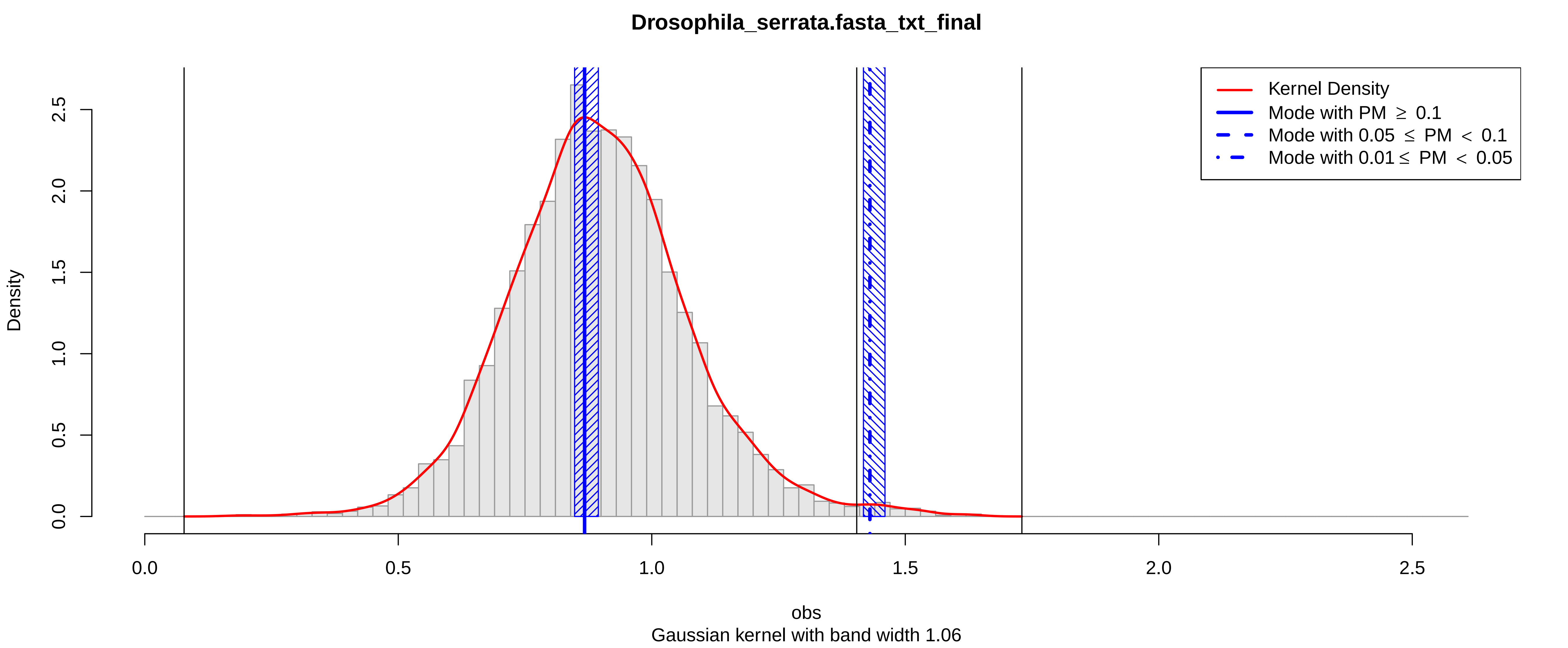
Drosophila_serrata.fasta_txt_final
Kernel Density
Mode with PM ≥ 0.1
Mode with 0.05 ≤ PM < 0.1
Mode with 0.01 ≤ PM < 0.05
Density
0.0
0.5
1.0
1.5
2.0
2.5
obs
Gaussian kernel with band width 1.06

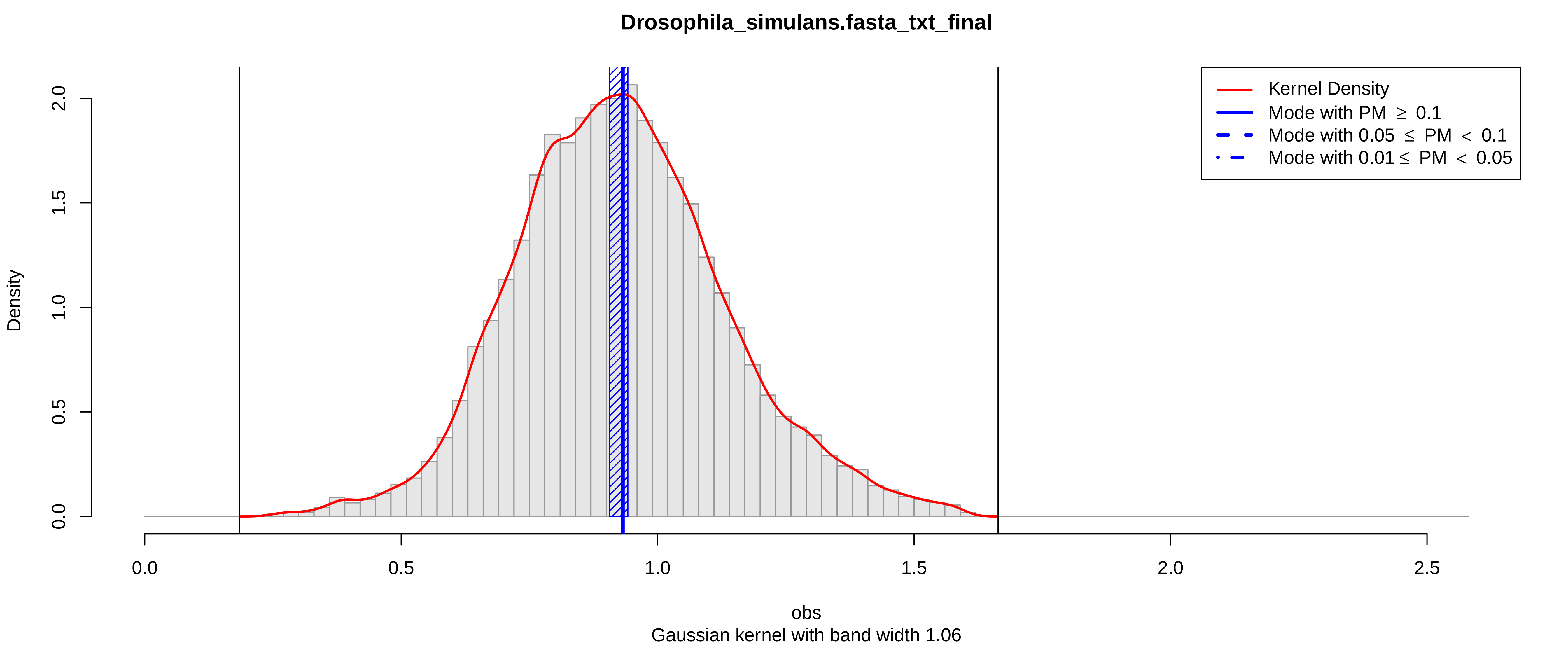
Drosophila_simulans.fasta_txt_final
Kernel Density
Mode with PM ≥ 0.1
Mode with 0.05 ≤ PM < 0.1
Mode with 0.01 ≤ PM < 0.05
Density
0.0
0.5
1.0
1.5
2.0
2.5
obs
Gaussian kernel with band width 1.06

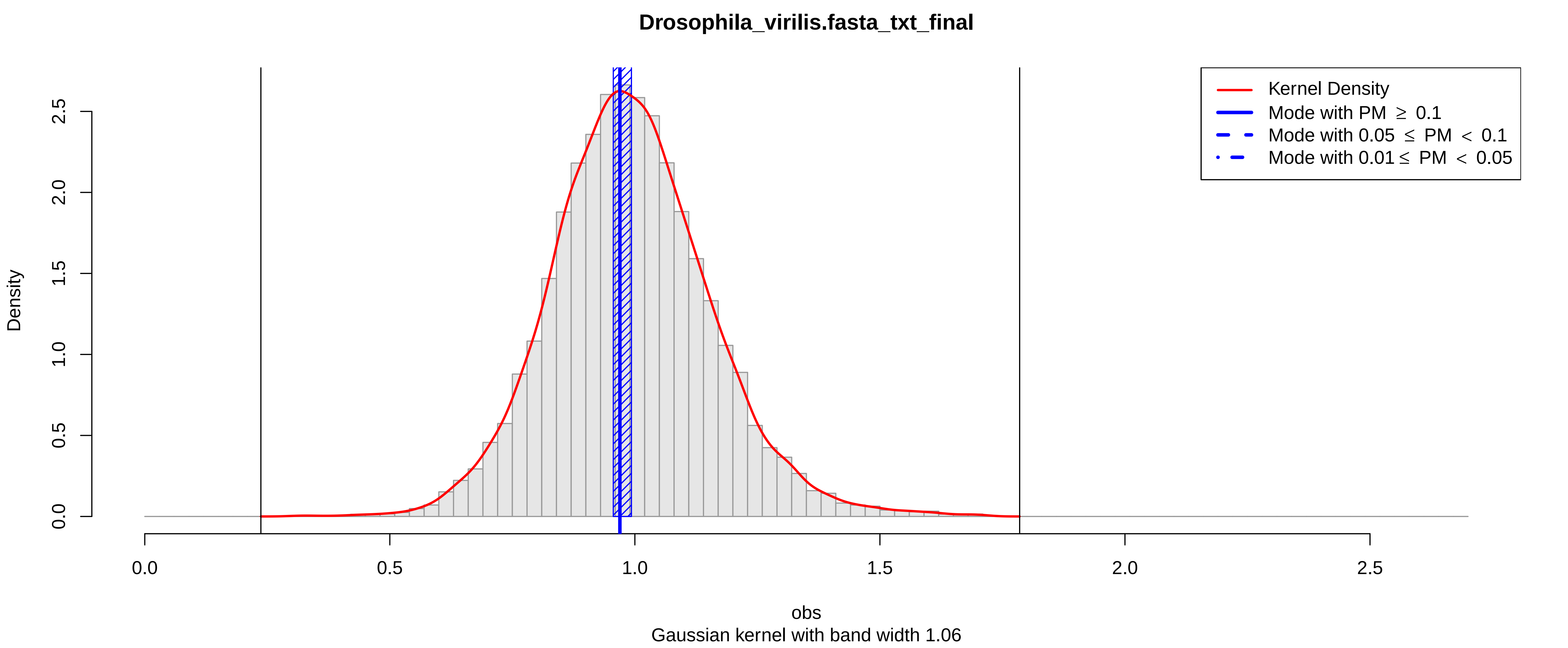

Drosophila_virilis.fasta_txt_final
Kernel Density
Mode with PM ≥ 0.1
Mode with 0.05 ≤ PM < 0.1
Mode with 0.01 ≤ PM < 0.05
Density
0.0
0.5
1.0
1.5
2.0
2.5
obs
Gaussian kernel with band width 1.06

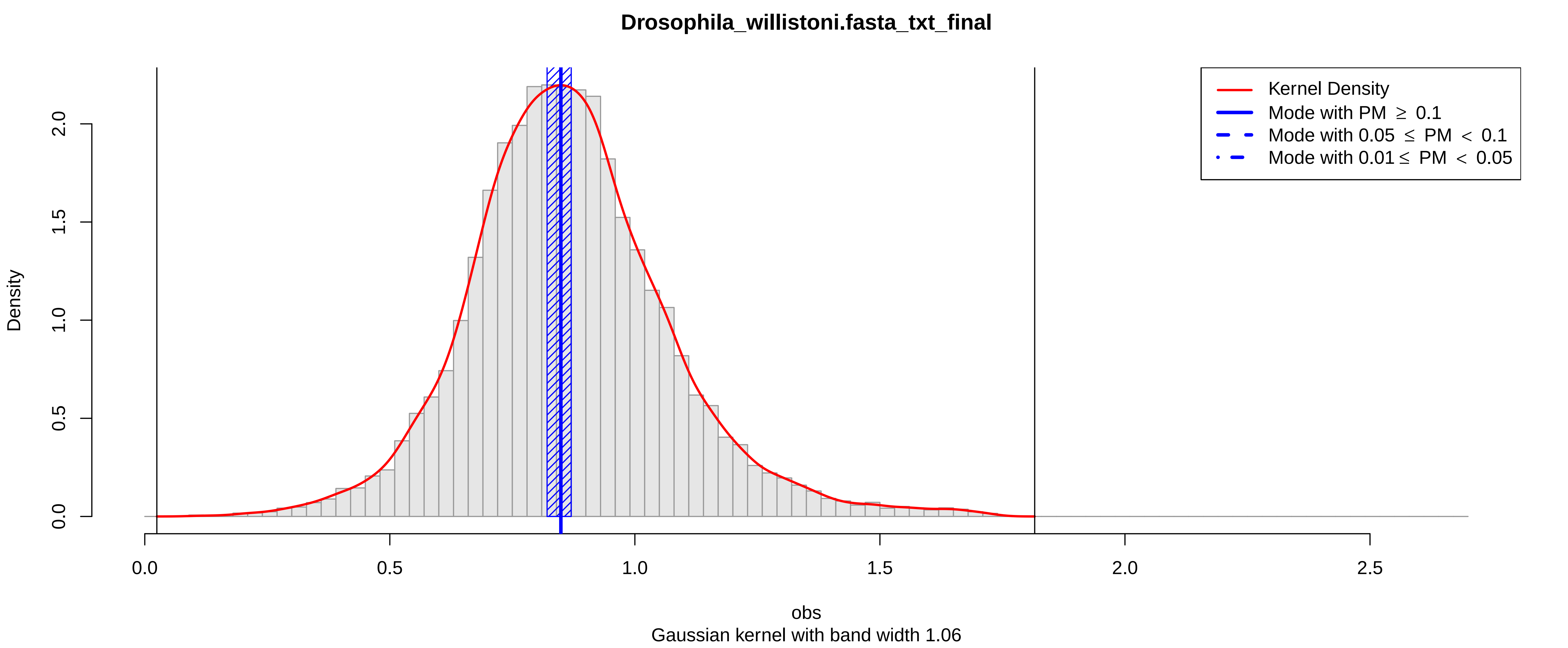
Drosophila_willistoni.fasta_txt_final
Kernel Density
Mode with PM ≥ 0.1
Mode with 0.05 ≤ PM < 0.1
Mode with 0.01 ≤ PM < 0.05
Density
0.0
0.5
1.0
1.5
2.0
0.0
0.5
1.0
1.5
2.0
2.5
obs
Gaussian kernel with band width 1.06

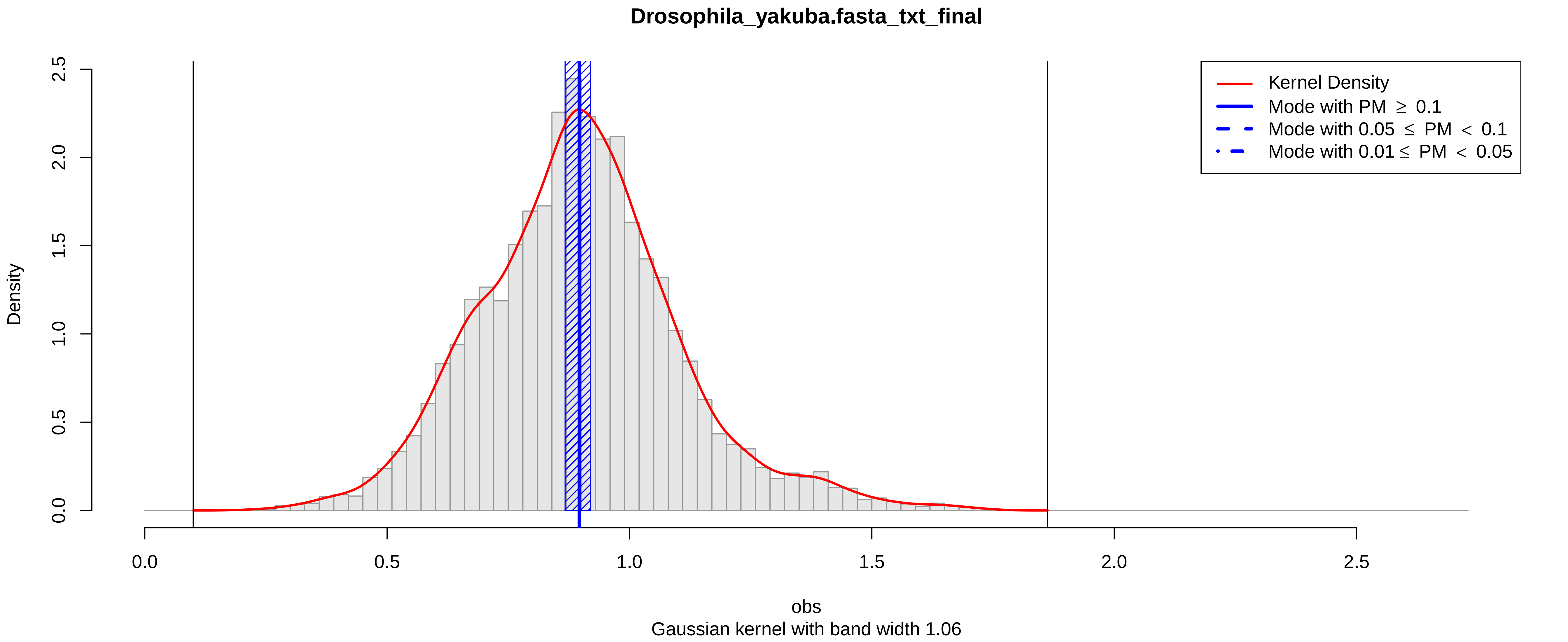
Drosophila_yakuba.fasta_txt_final
Kernel Density
Mode with PM ≥ 0.1
Mode with 0.05 ≤ PM < 0.1
Mode with 0.01 ≤ PM < 0.05
Density
0.0
0.5
1.0
1.5
2.0
2.5
obs
Gaussian kernel with band width 1.06

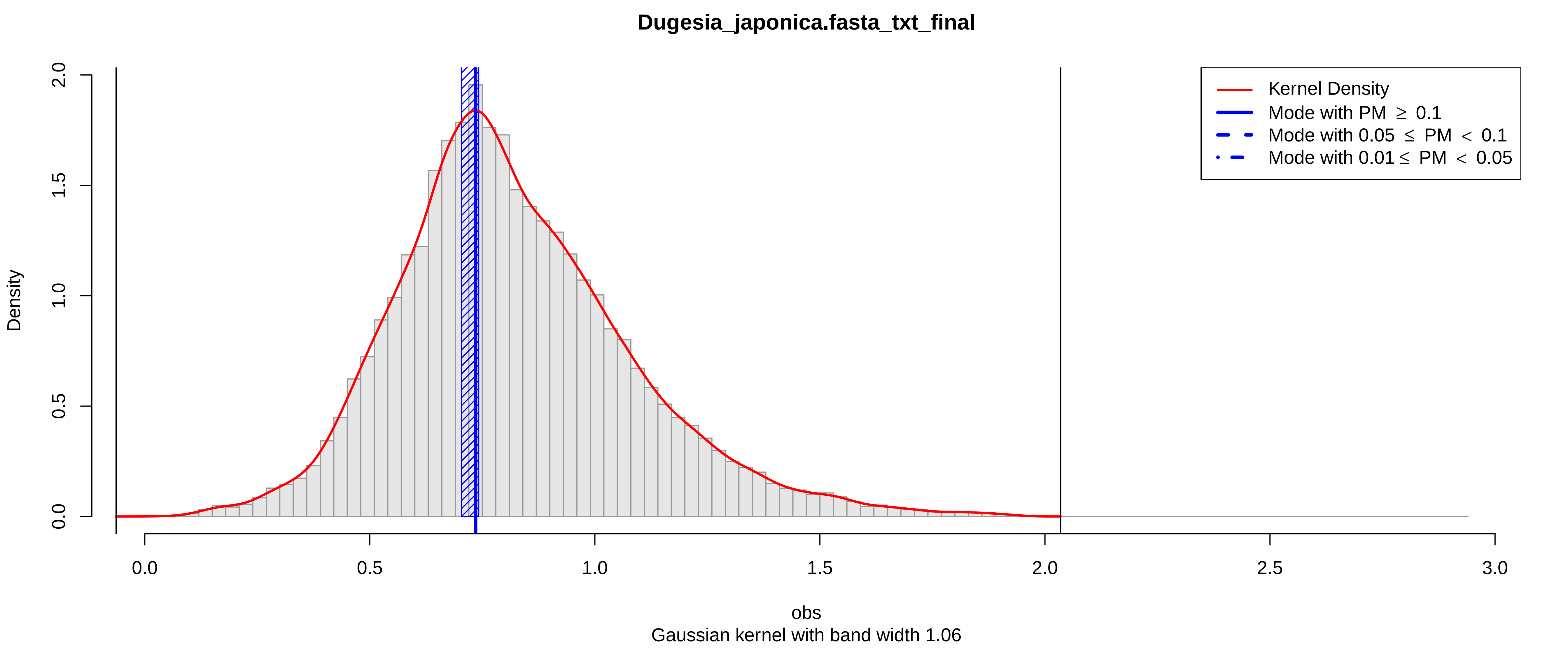
Dugesia_japonica.fasta_txt_final
Kernel Density
Mode with PM ≥ 0.1
Mode with 0.05 ≤ PM < 0.1
Mode with 0.01 ≤ PM < 0.05
Density
0.0
0.5
1.0
1.5
2.0
0.0
0.5
1.0
1.5
2.0
2.5
3.0
obs
Gaussian kernel with band width 1.06

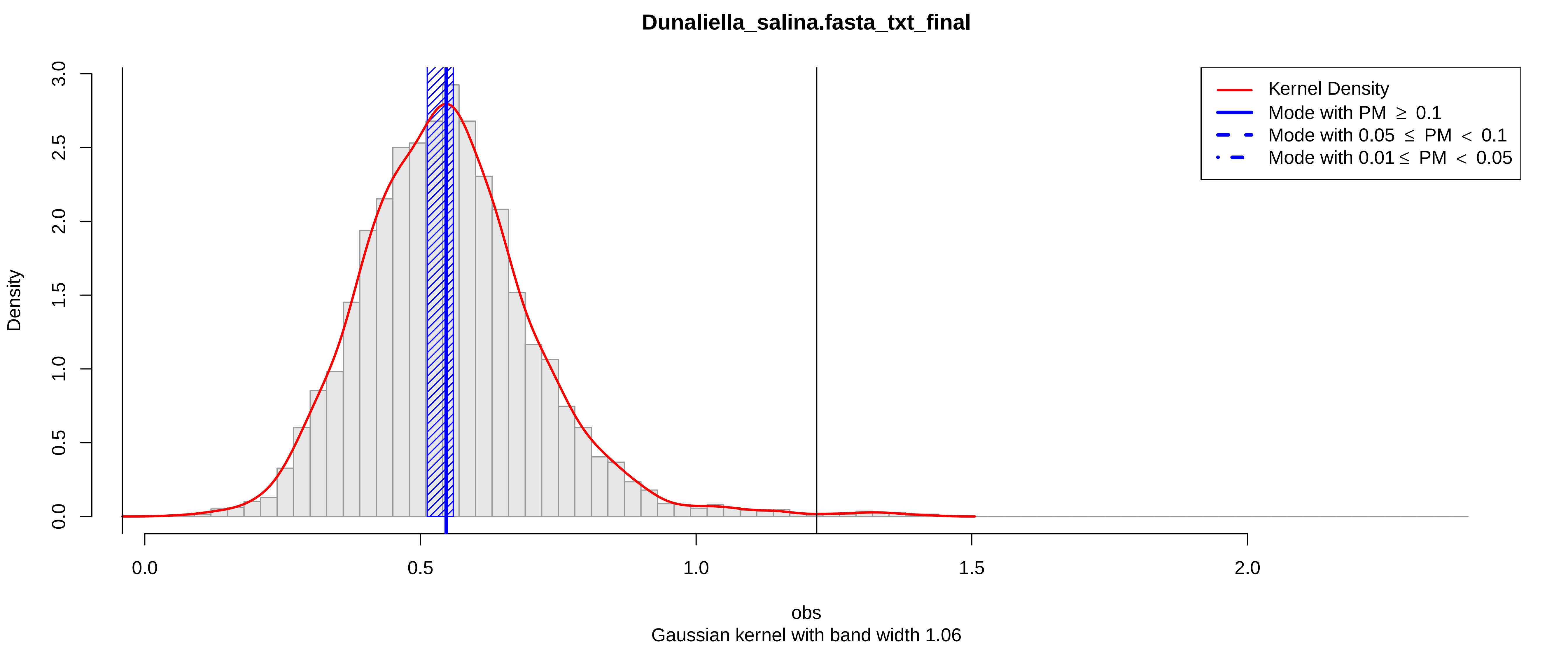
Dunaliella_salina.fasta_txt_final
Kernel Density
Mode with PM ≥ 0.1
Mode with 0.05 ≤ PM < 0.1
Mode with 0.01 ≤ PM < 0.05
Density
0.0
0.5
1.0
1.5
2.0
2.5
3.0
0.0
0.5
1.0
1.5
2.0
obs
Gaussian kernel with band width 1.06

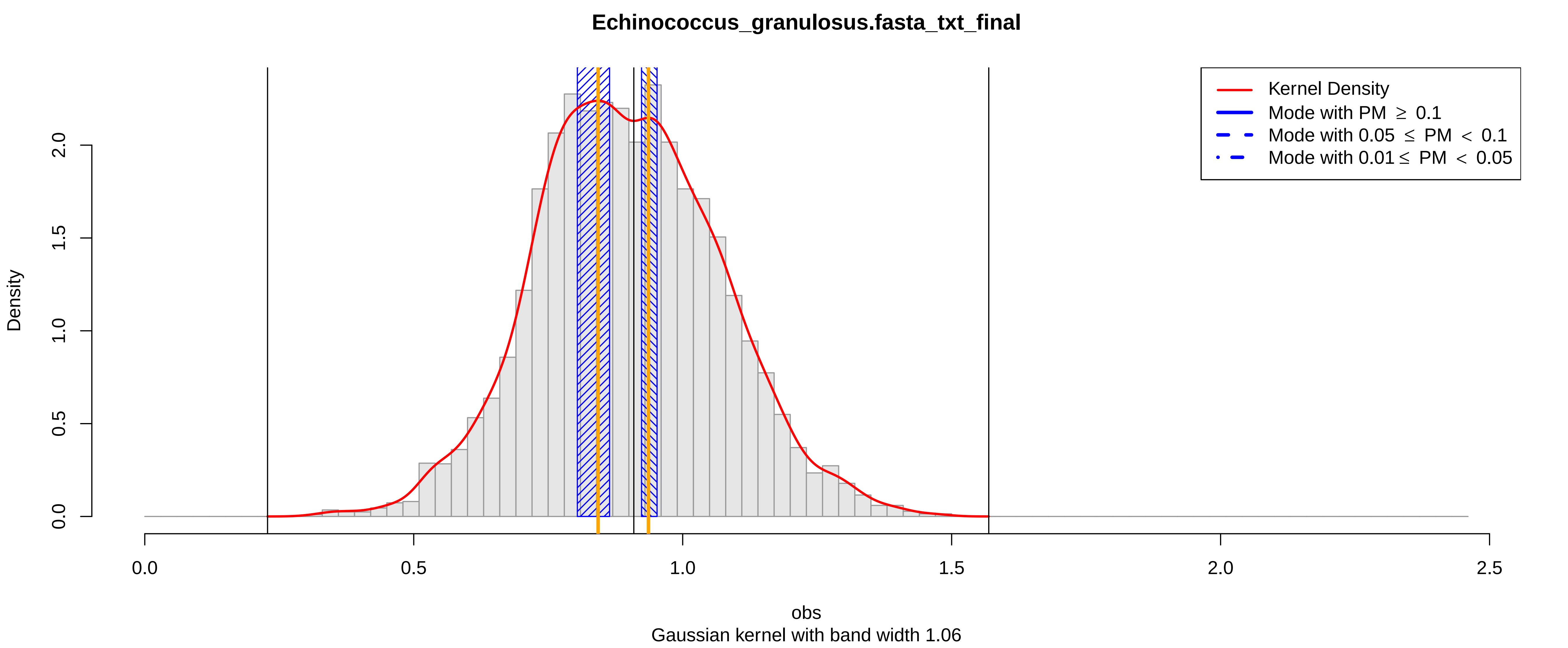
Echinococcus_granulosus.fasta_txt_final
Kernel Density
Mode with PM ≥ 0.1
Mode with 0.05 ≤ PM < 0.1
Mode with 0.01 ≤ PM < 0.05
Density
0.0
0.5
1.0
1.5
2.0
0.0
0.5
1.0
1.5
2.0
2.5
obs
Gaussian kernel with band width 1.06

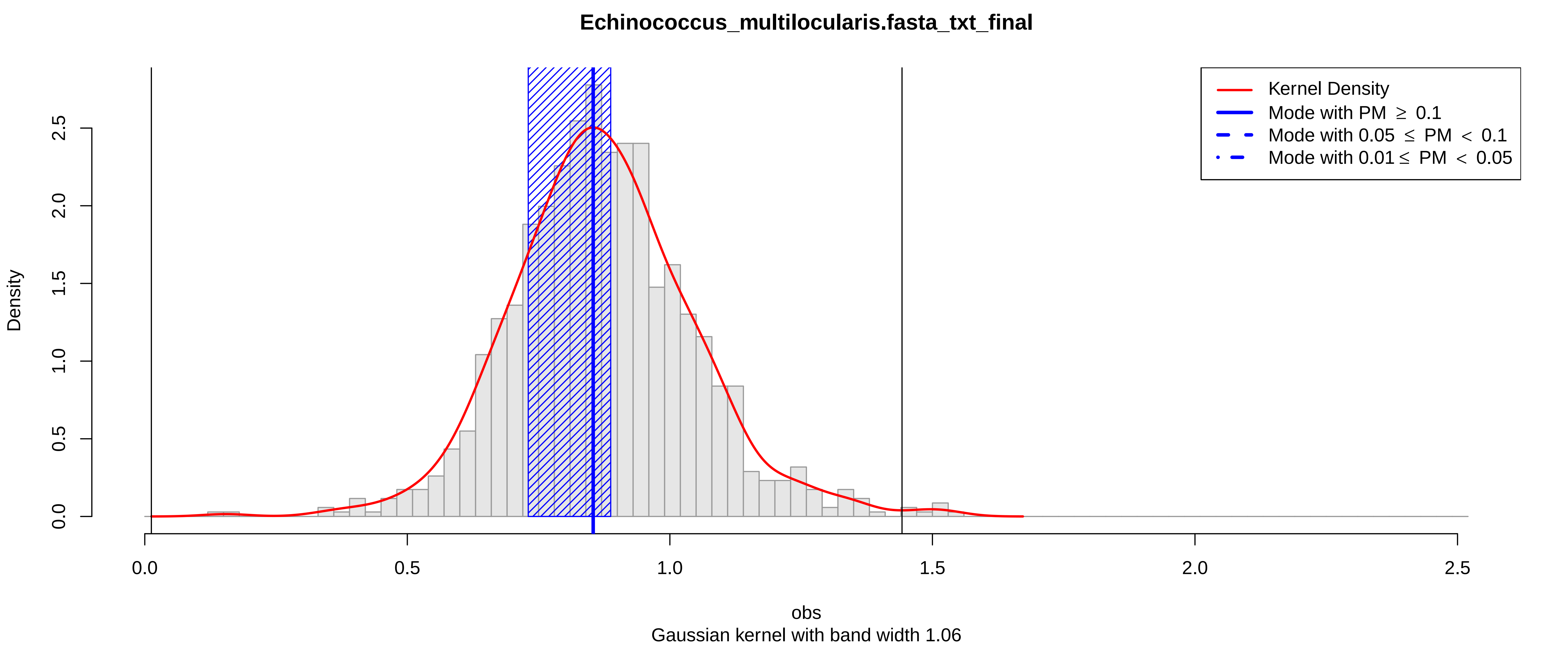

Echinococcus_multilocularis.fasta_txt_final
Kernel Density
Mode with PM ≥ 0.1
Mode with 0.05 ≤ PM < 0.1
Mode with 0.01 ≤ PM < 0.05
Density
0.0
0.5
1.0
1.5
2.0
2.5
obs
Gaussian kernel with band width 1.06

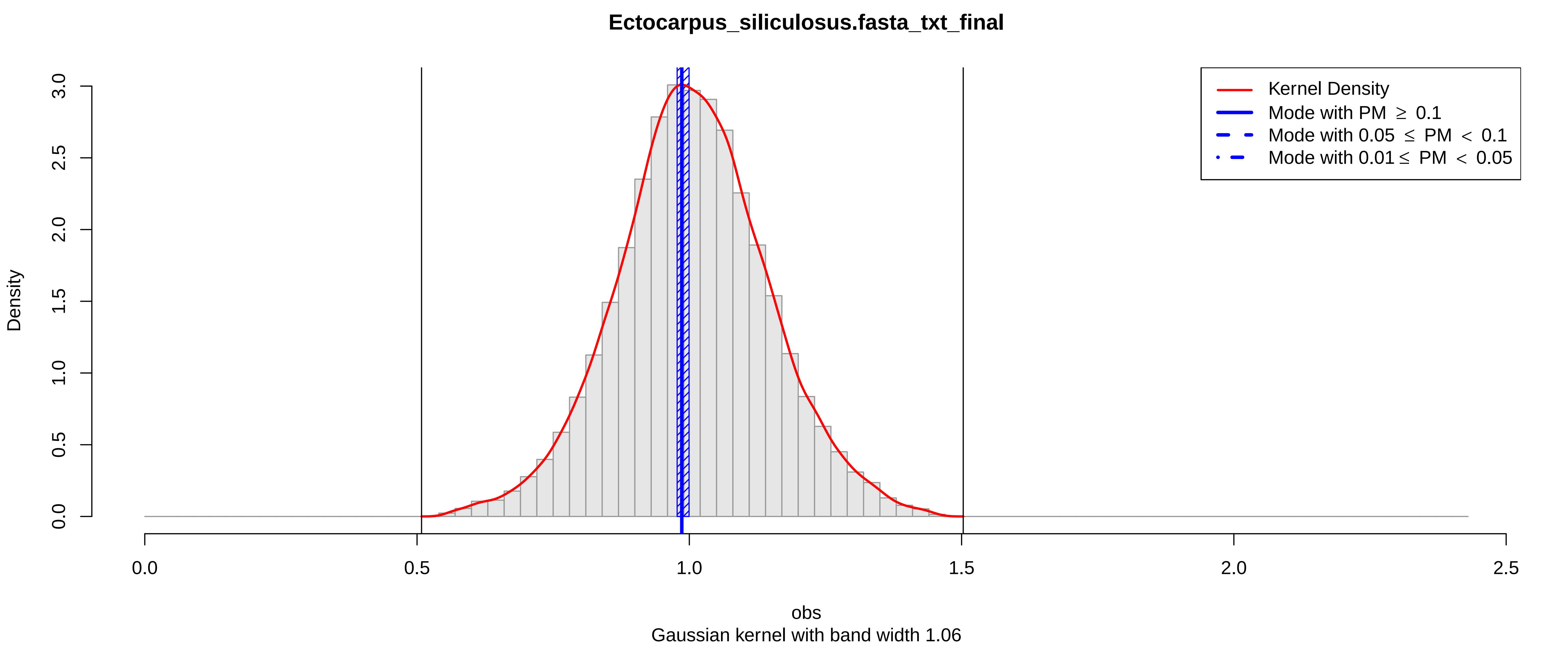
Ectocarpus_siliculosus.fasta_txt_final
Kernel Density
Mode with PM ≥ 0.1
Mode with 0.05 ≤ PM < 0.1
Mode with 0.01 ≤ PM < 0.05
Density
0.0
0.5
1.0
1.5
2.0
2.5
3.0
0.0
0.5
1.0
1.5
2.0
2.5
obs
Gaussian kernel with band width 1.06

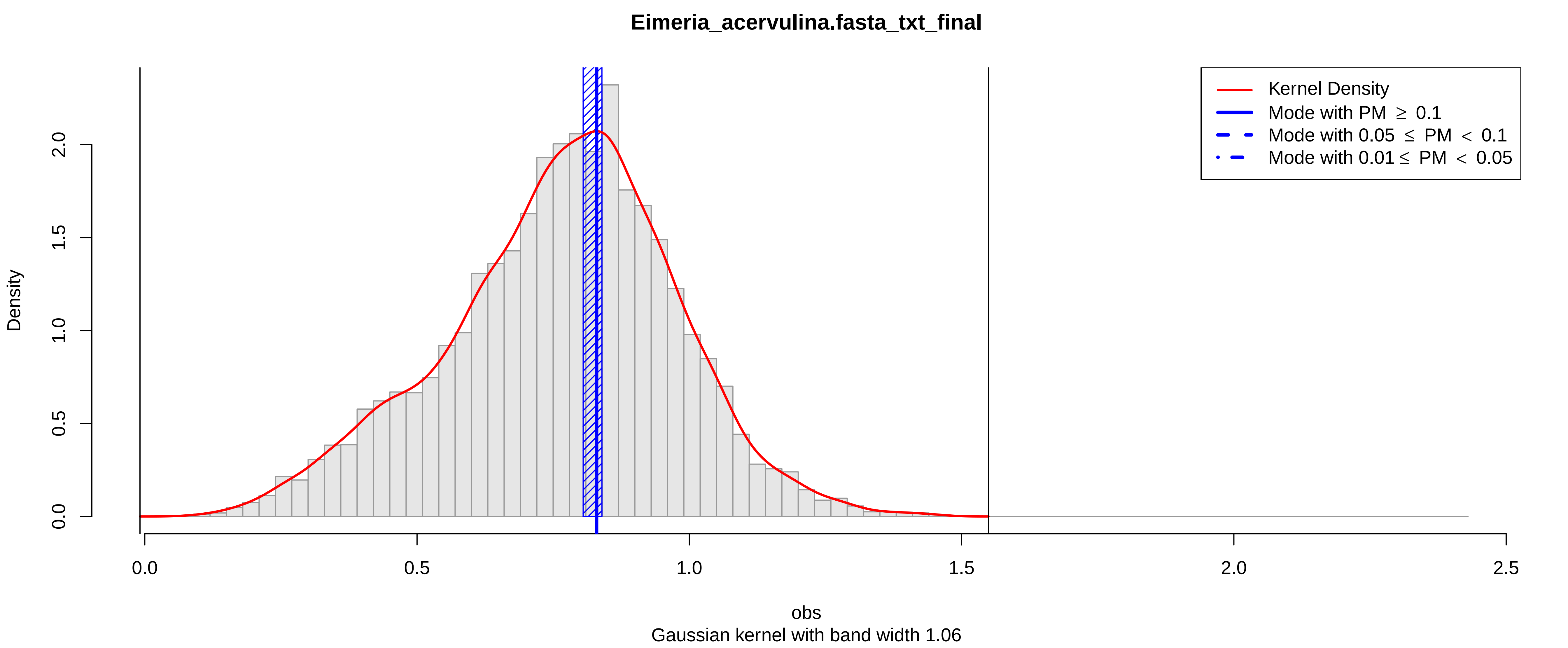
Eimeria_acervulina.fasta_txt_final
Kernel Density
Mode with PM ≥ 0.1
Mode with 0.05 ≤ PM < 0.1
Mode with 0.01 ≤ PM < 0.05
Density
0.0
0.5
1.0
1.5
2.0
2.5
obs
Gaussian kernel with band width 1.06

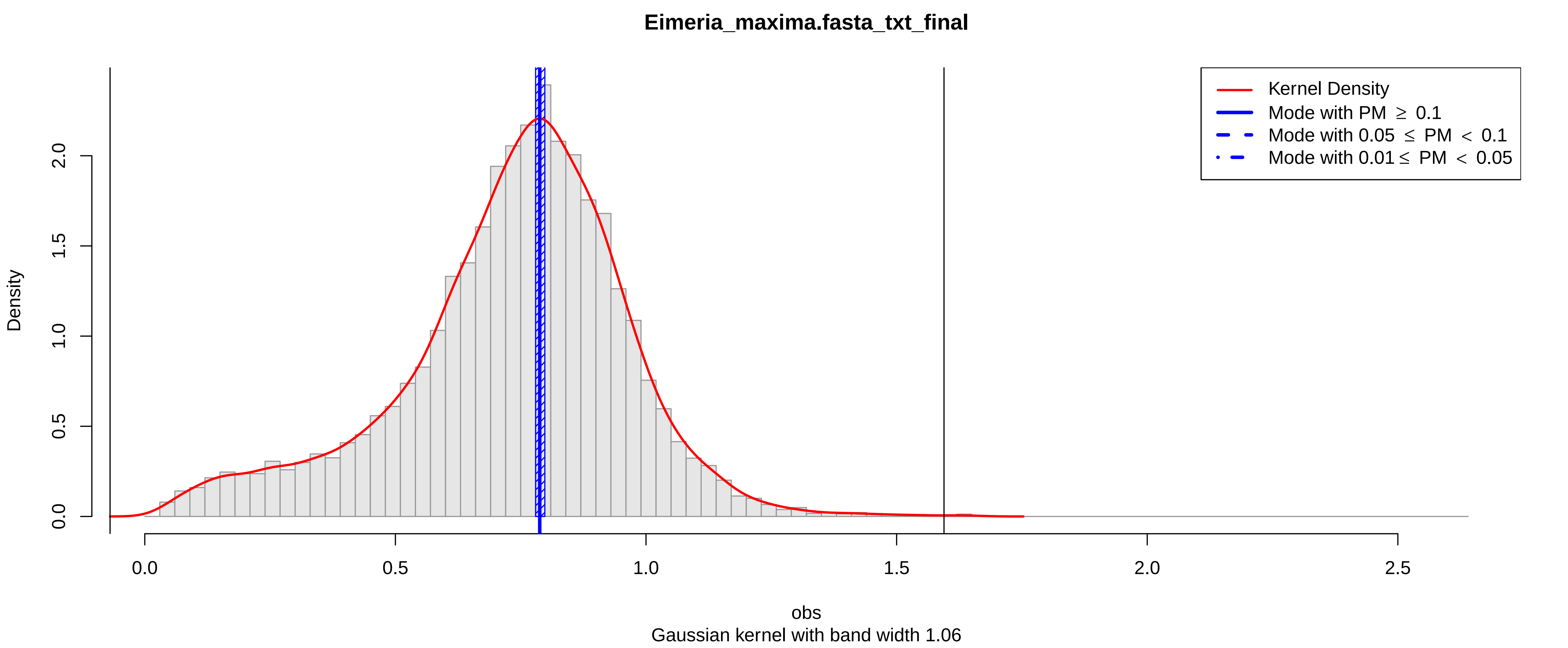
Eimeria_maxima.fasta_txt_final
Kernel Density
Mode with PM ≥ 0.1
Mode with 0.05 ≤ PM < 0.1
Mode with 0.01 ≤ PM < 0.05
Density
0.0
0.5
1.0
1.5
2.0
2.5
obs
Gaussian kernel with band width 1.06

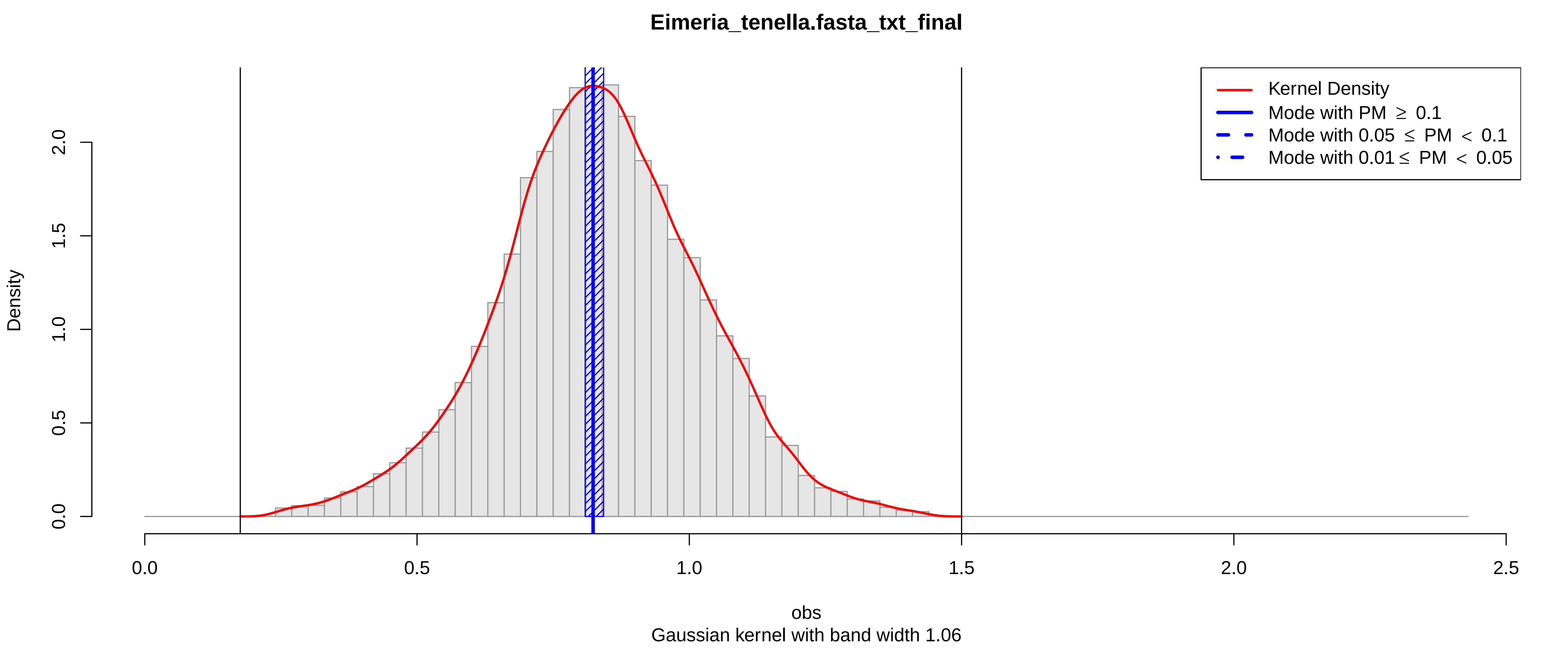

Eimeria_tenella.fasta_txt_final
Kernel Density
Mode with PM ≥ 0.1
Mode with 0.05 ≤ PM < 0.1
Mode with 0.01 ≤ PM < 0.05
Density
0.0
0.5
1.0
1.5
2.0
2.5
obs
Gaussian kernel with band width 1.06

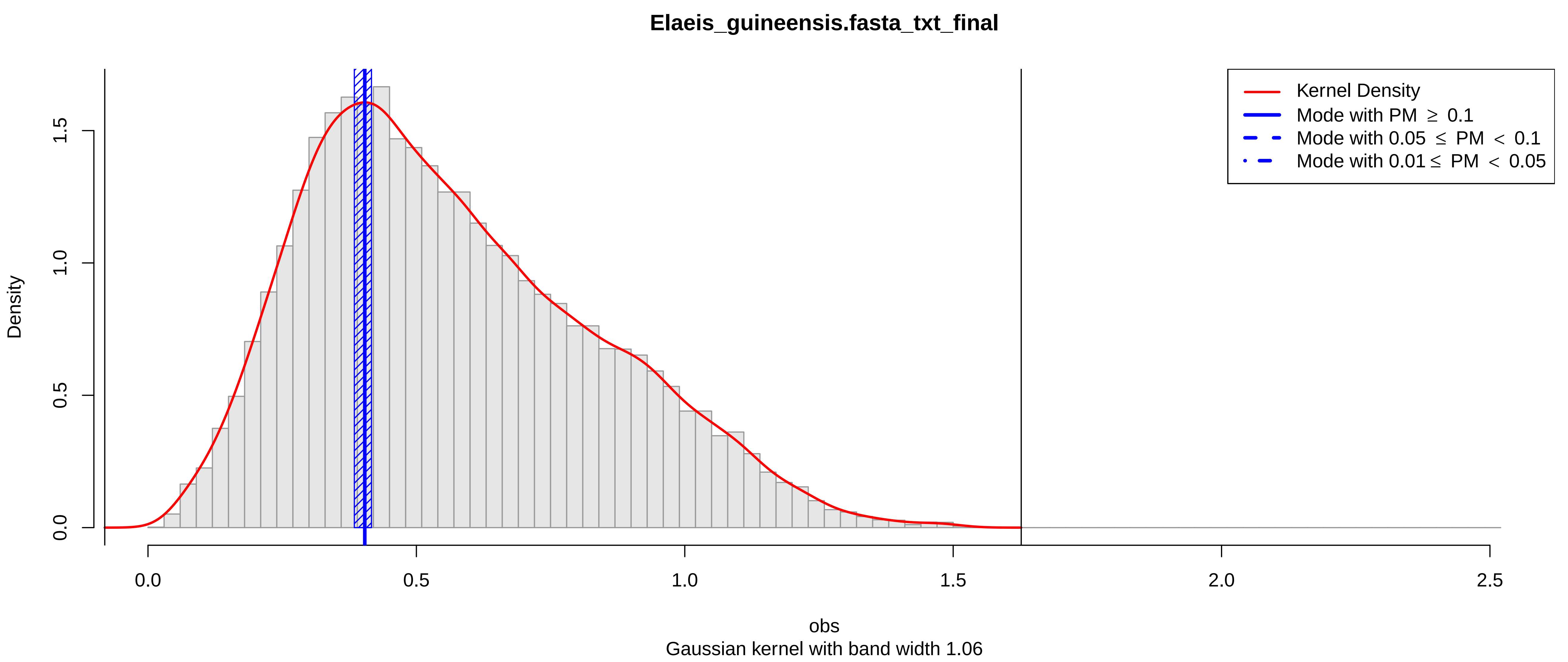
Elaeis_guineensis.fasta_txt_final
Kernel Density
Mode with PM ≥ 0.1
Mode with 0.05 ≤ PM < 0.1
Mode with 0.01 ≤ PM < 0.05
Density
0.0
0.5
1.0
1.5
2.0
2.5
obs
Gaussian kernel with band width 1.06

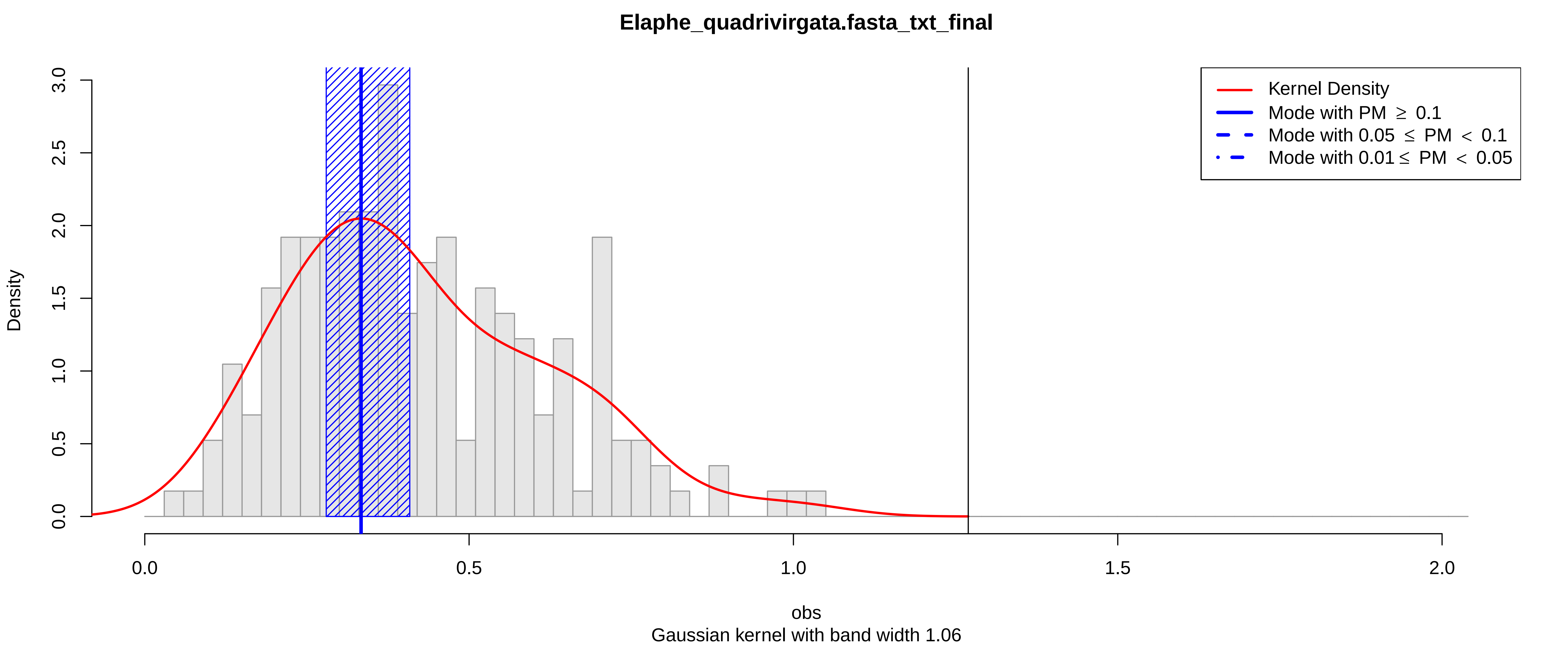
Elaphe_quadrivirgata.fasta_txt_final
Kernel Density
Mode with PM ≥ 0.1
Mode with 0.05 ≤ PM < 0.1
Mode with 0.01 ≤ PM < 0.05
Density
0.0
0.5
1.0
1.5
2.0
2.5
3.0
0.0
0.5
1.0
1.5
2.0
obs
Gaussian kernel with band width 1.06

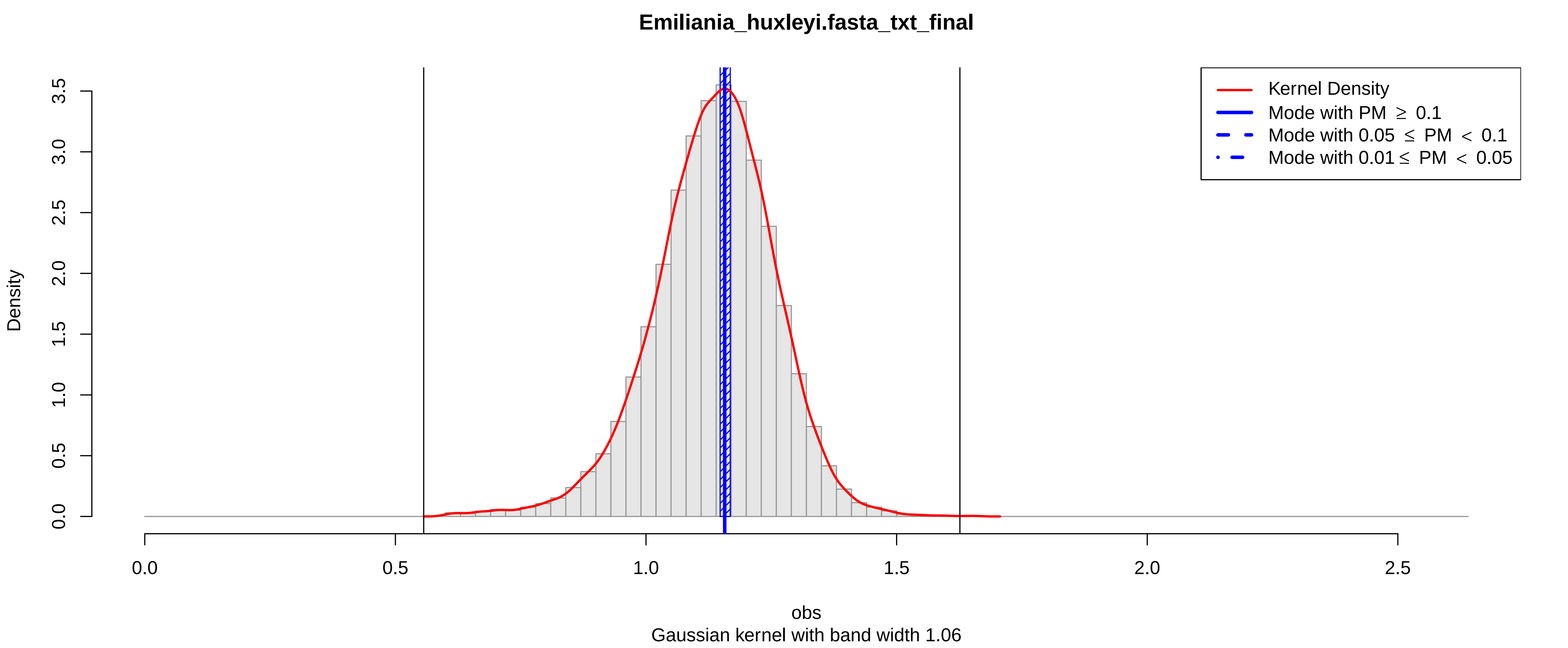

Emiliania_huxleyi.fasta_txt_final
Kernel Density
Mode with PM ≥ 0.1
Mode with 0.05 ≤ PM < 0.1
Mode with 0.01 ≤ PM < 0.05
Density
0.0
0.5
1.0
1.5
2.0
2.5
3.0
3.5
0.0
0.5
1.0
1.5
2.0
2.5
obs
Gaussian kernel with band width 1.06

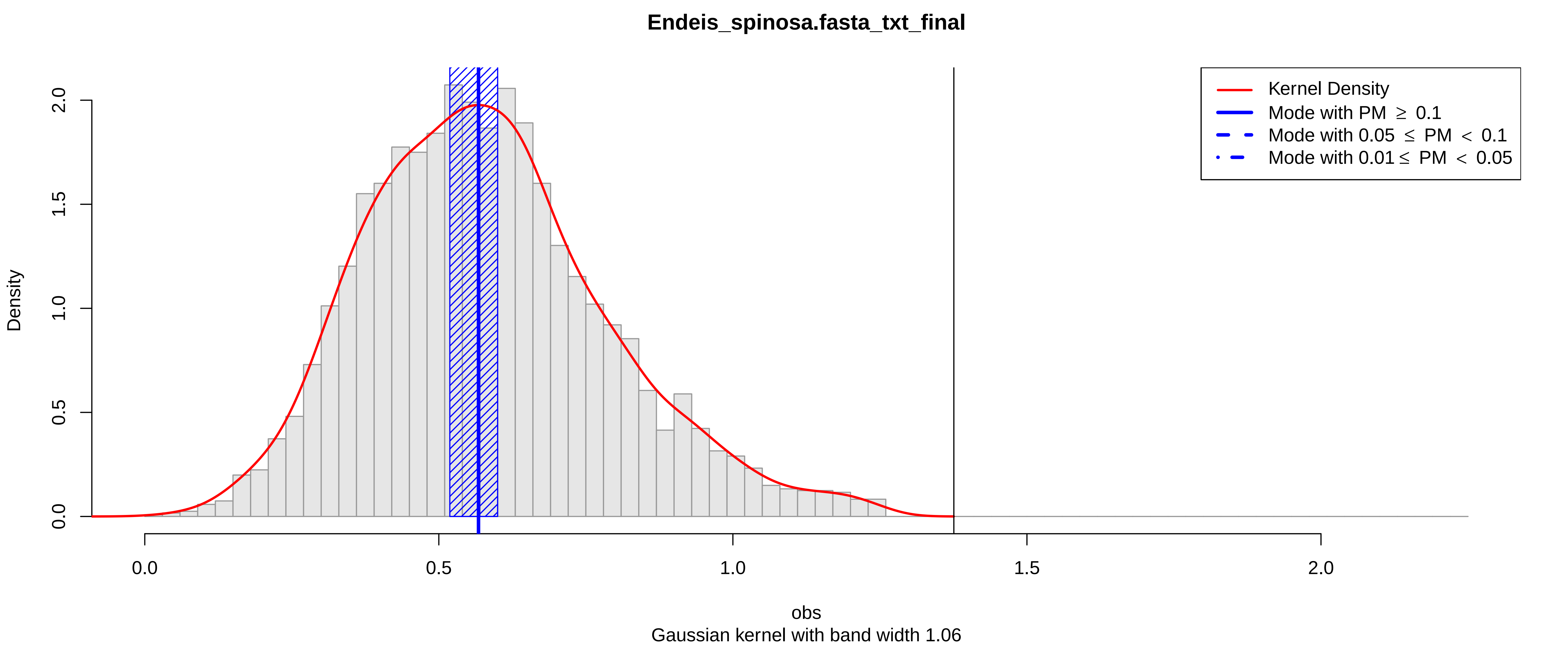
Endeis_spinosa.fasta_txt_final
Kernel Density
Mode with PM ≥ 0.1
Mode with 0.05 ≤ PM < 0.1
Mode with 0.01 ≤ PM < 0.05
Density
0.0
0.5
1.0
1.5
2.0
0.0
0.5
1.0
1.5
2.0
obs
Gaussian kernel with band width 1.06

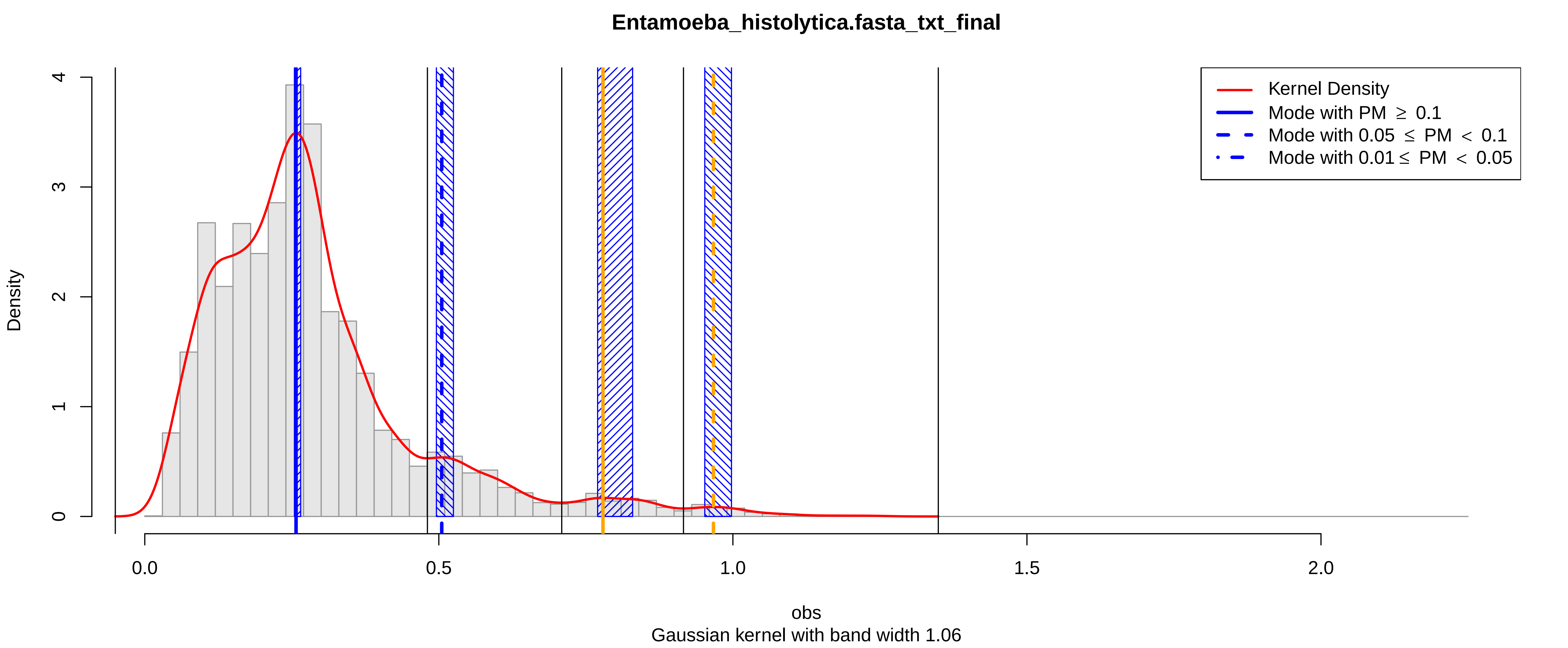

Entamoeba_histolytica.fasta_txt_final
Kernel Density
Mode with PM ≥ 0.1
Mode with 0.05 ≤ PM < 0.1
Mode with 0.01 ≤ PM < 0.05
Density
0
1
2
3
4
0.0
0.5
1.0
1.5
2.0
obs
Gaussian kernel with band width 1.06

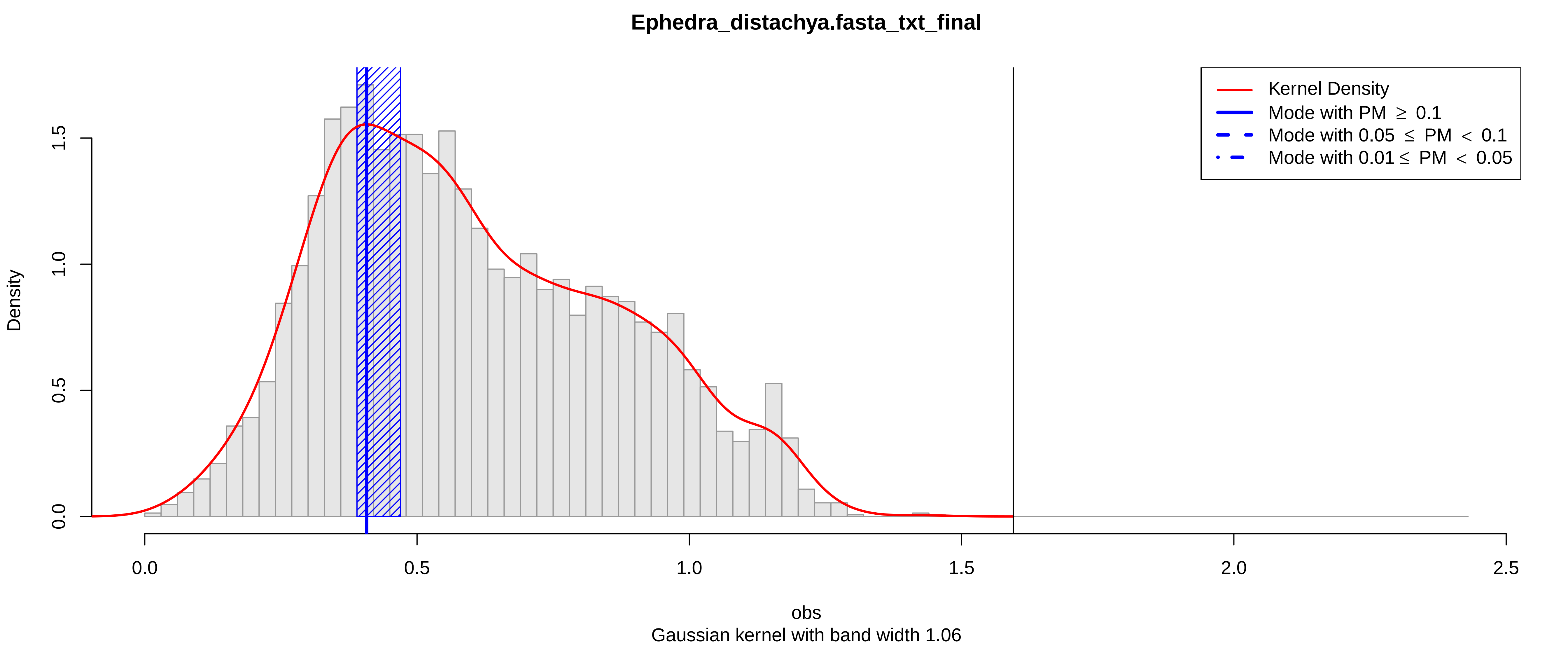
Ephedra_distachya.fasta_txt_final
Kernel Density
Mode with PM ≥ 0.1
Mode with 0.05 ≤ PM < 0.1
Mode with 0.01 ≤ PM < 0.05
Density
0.0
0.5
1.0
1.5
0.0
0.5
1.0
1.5
2.0
2.5
obs
Gaussian kernel with band width 1.06

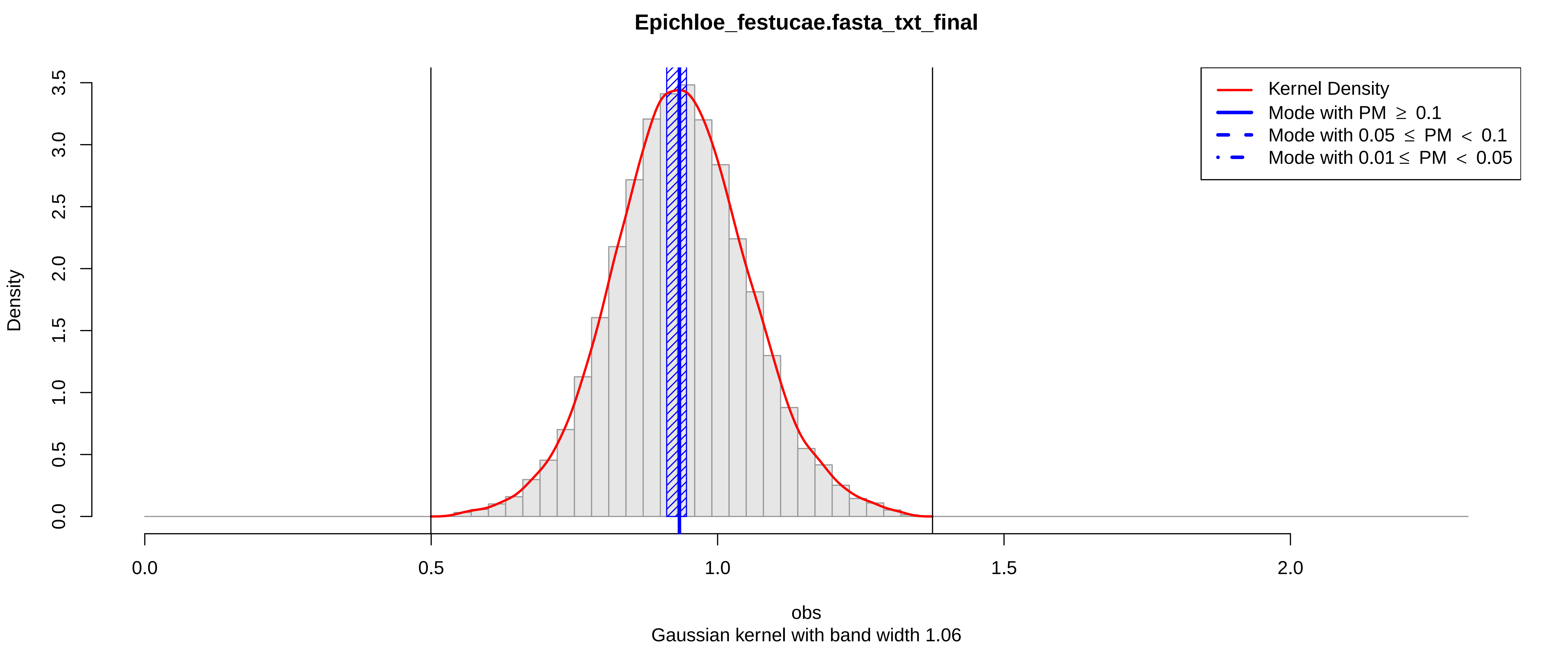

Epichloe_festucae.fasta_txt_final
Density
0.0
0.5
1.0
1.5
2.0
2.5
3.0
3.5
0.0
0.5
1.0
1.5
2.0
Kernel Density
Mode with PM ≥ 0.1
Mode with 0.05 ≤ PM < 0.1
Mode with 0.01 ≤ PM < 0.05
obs
Gaussian kernel with band width 1.06

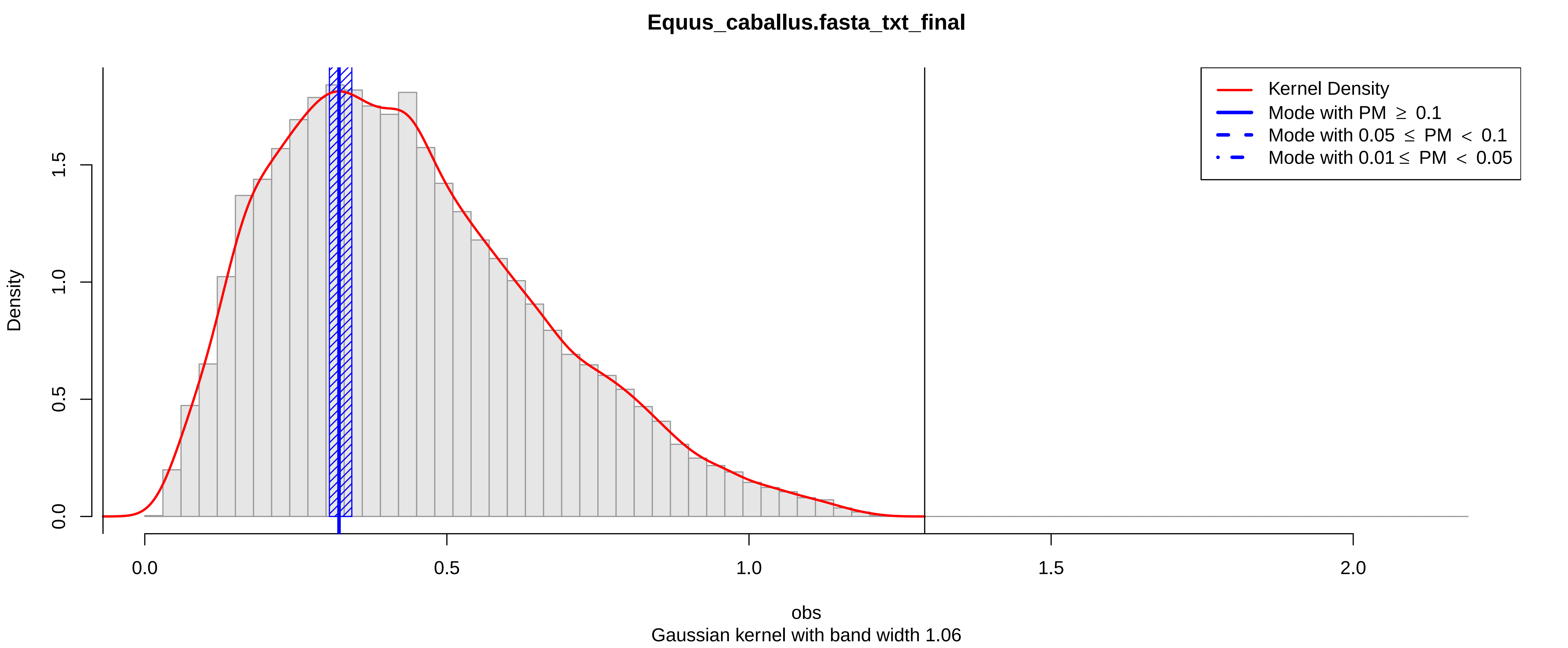

Equus_caballus.fasta_txt_final
Kernel Density
Mode with PM ≥ 0.1
Mode with 0.05 ≤ PM < 0.1
Mode with 0.01 ≤ PM < 0.05
Density
0.0
0.5
1.0
1.5
0.0
0.5
1.0
1.5
2.0
obs
Gaussian kernel with band width 1.06

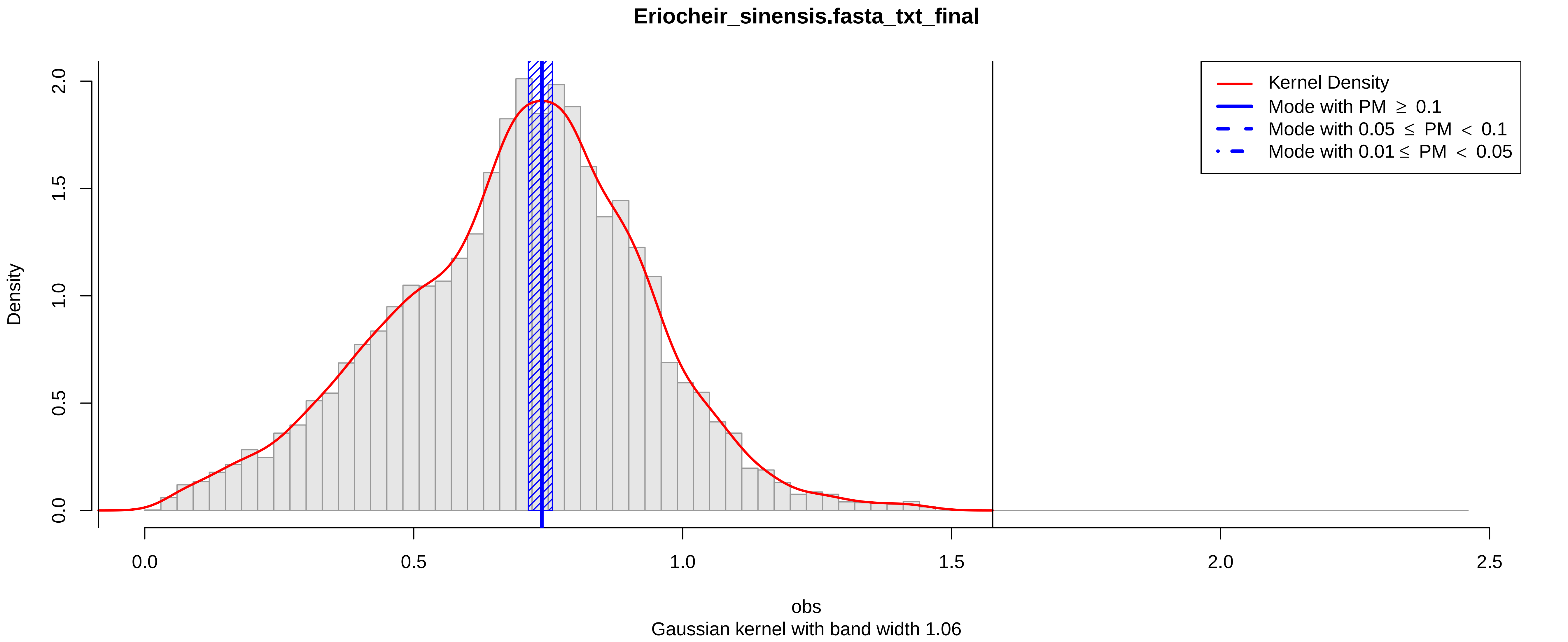
Eriocheir_sinensis.fasta_txt_final
Kernel Density
Mode with PM ≥ 0.1
Mode with 0.05 ≤ PM < 0.1
Mode with 0.01 ≤ PM < 0.05
Density
0.0
0.5
1.0
1.5
2.0
0.0
0.5
1.0
1.5
2.0
2.5
obs
Gaussian kernel with band width 1.06

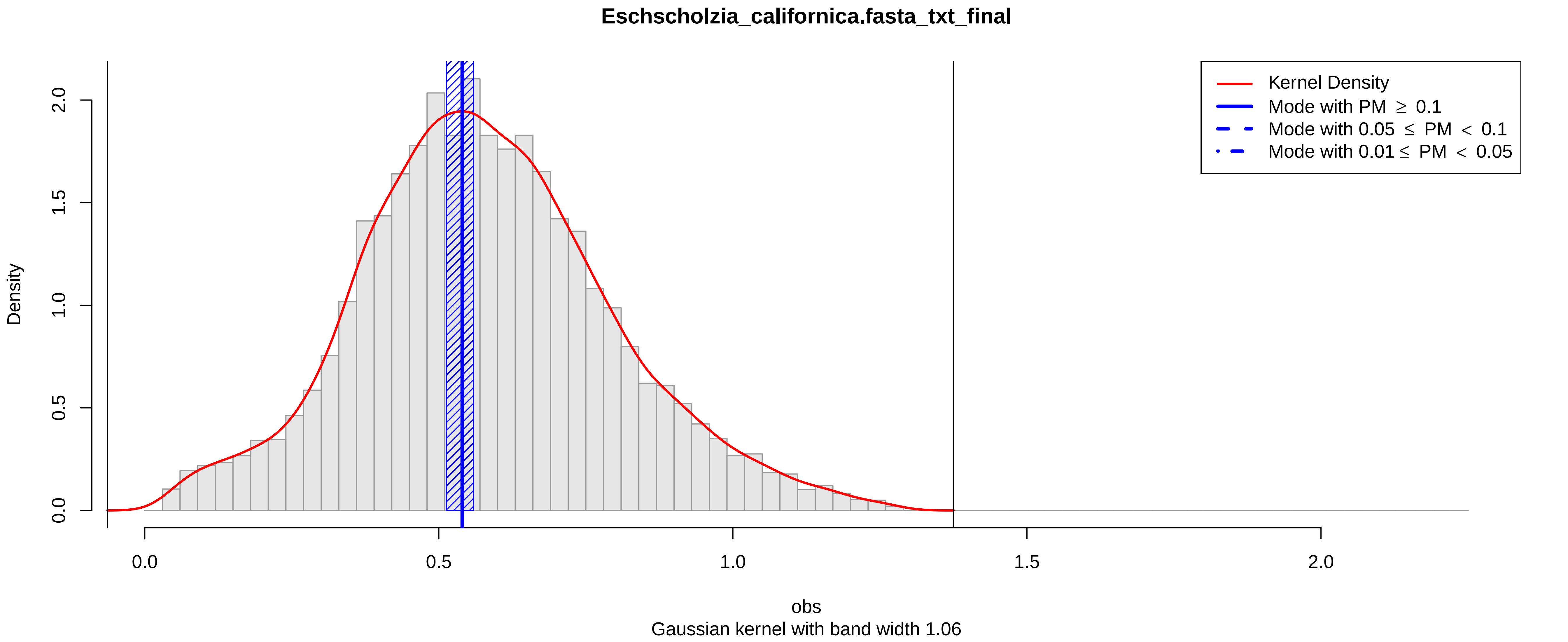

Eschscholzia_californica.fasta_txt_final
Kernel Density
Mode with PM ≥ 0.1
Mode with 0.05 ≤ PM < 0.1
Mode with 0.01 ≤ PM < 0.05
Density
0.0
0.5
1.0
1.5
2.0
obs
Gaussian kernel with band width 1.06

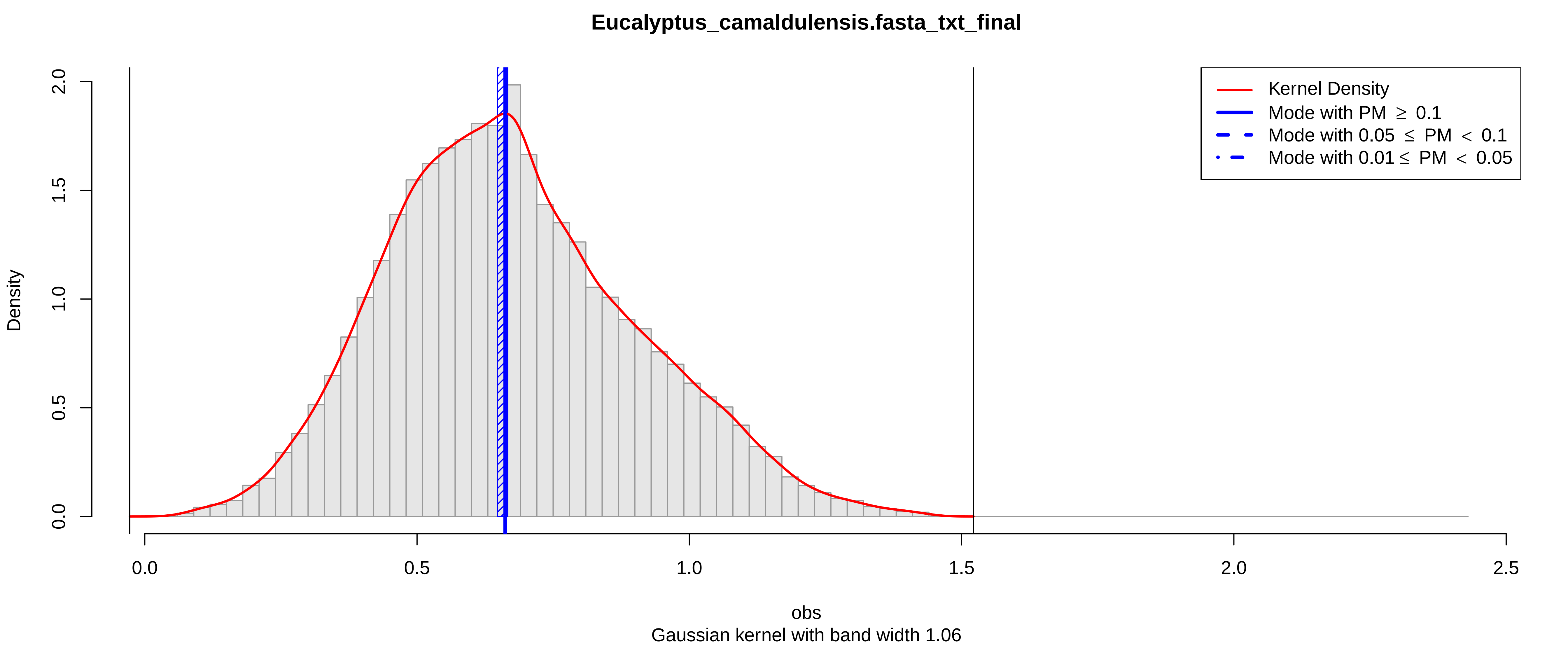

Eucalyptus_camaldulensis.fasta_txt_final
Kernel Density
Mode with PM ≥ 0.1
Mode with 0.05 ≤ PM < 0.1
Mode with 0.01 ≤ PM < 0.05
Density
0.0
0.5
1.0
1.5
2.0
2.5
obs
Gaussian kernel with band width 1.06

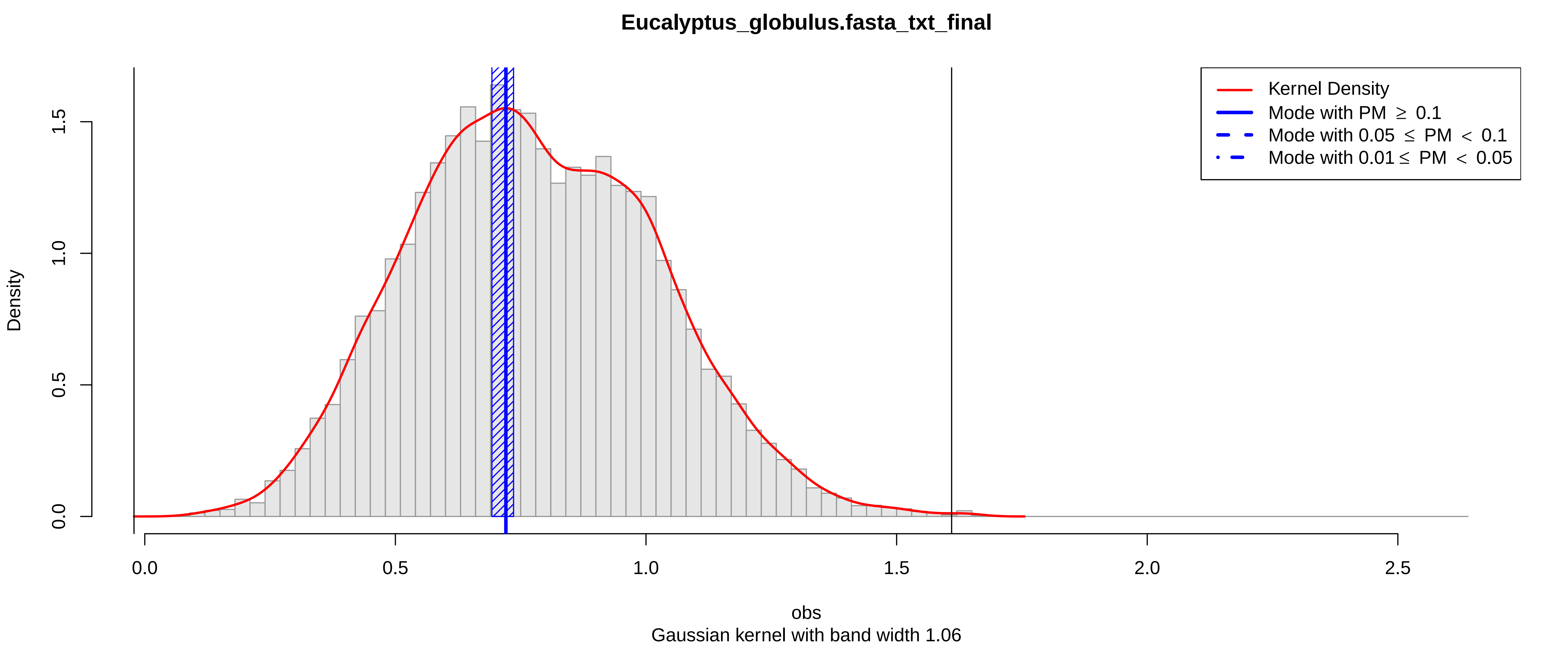

Eucalyptus_globulus.fasta_txt_final
Kernel Density
Mode with PM ≥ 0.1
Mode with 0.05 ≤ PM < 0.1
Mode with 0.01 ≤ PM < 0.05
Density
0.0
0.5
1.0
1.5
0.0
0.5
1.0
1.5
2.0
2.5
obs
Gaussian kernel with band width 1.06

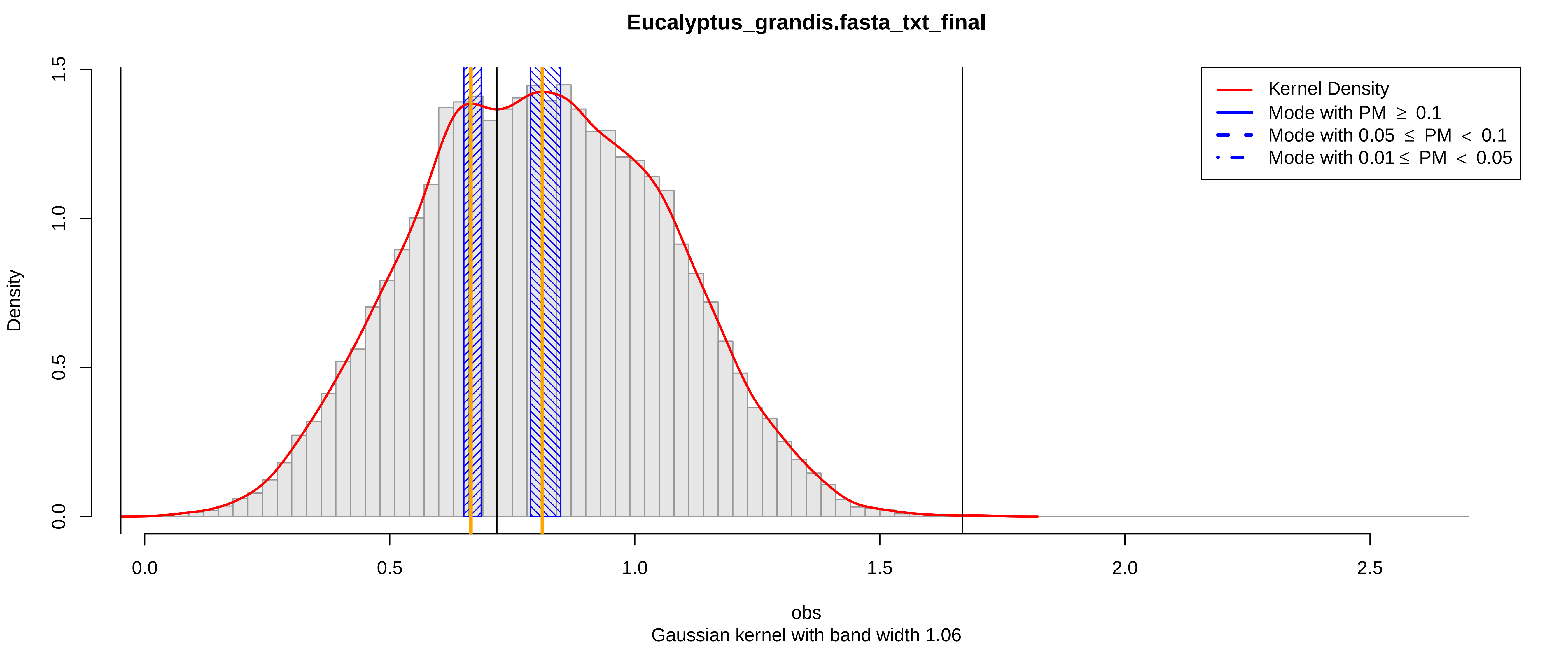

Eucalyptus_grandis.fasta_txt_final
Kernel Density
Mode with PM ≥ 0.1
Mode with 0.05 ≤ PM < 0.1
Mode with 0.01 ≤ PM < 0.05
Density
0.0
0.5
1.0
1.5
0.0
0.5
1.0
1.5
2.0
2.5
obs
Gaussian kernel with band width 1.06

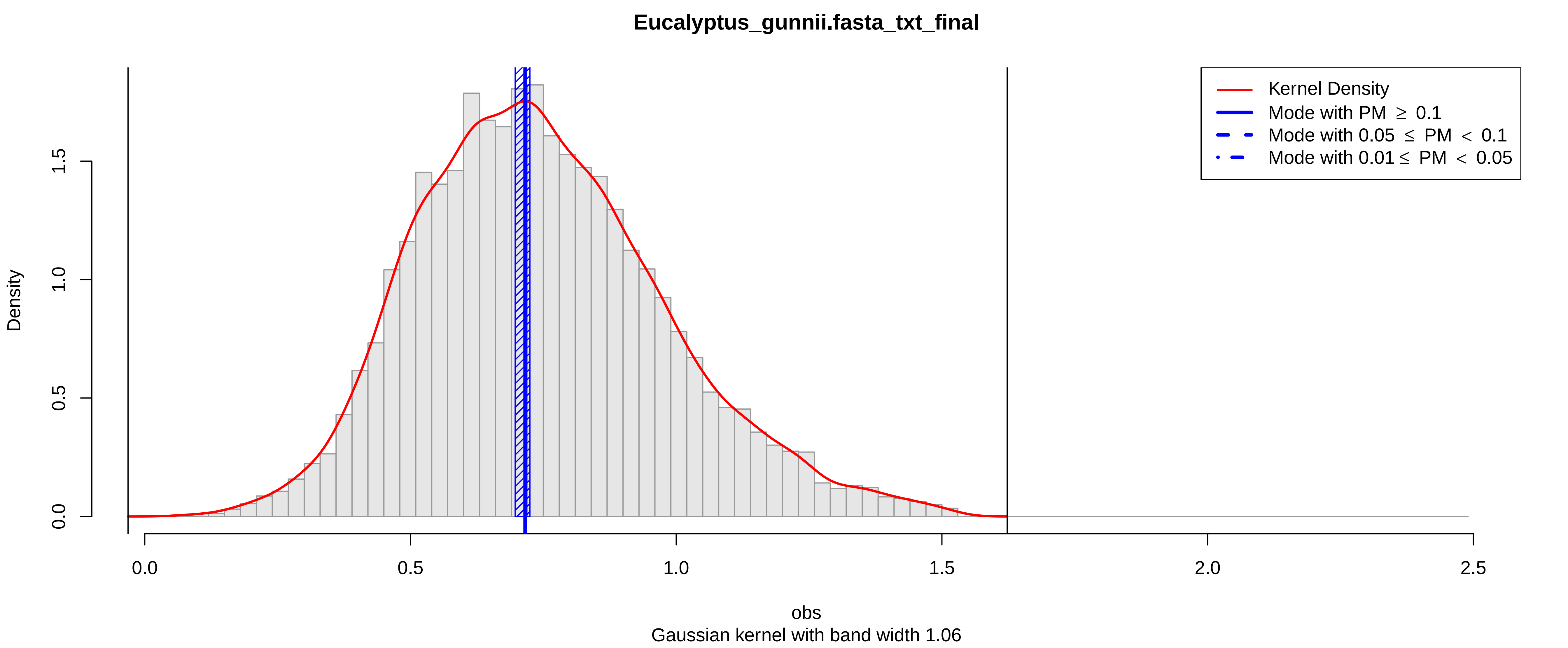
Eucalyptus_gunnii.fasta_txt_final
Kernel Density
Mode with PM ≥ 0.1
Mode with 0.05 ≤ PM < 0.1
Mode with 0.01 ≤ PM < 0.05
Density
0.0
0.5
1.0
1.5
0.0
0.5
1.0
1.5
2.0
2.5
obs
Gaussian kernel with band width 1.06

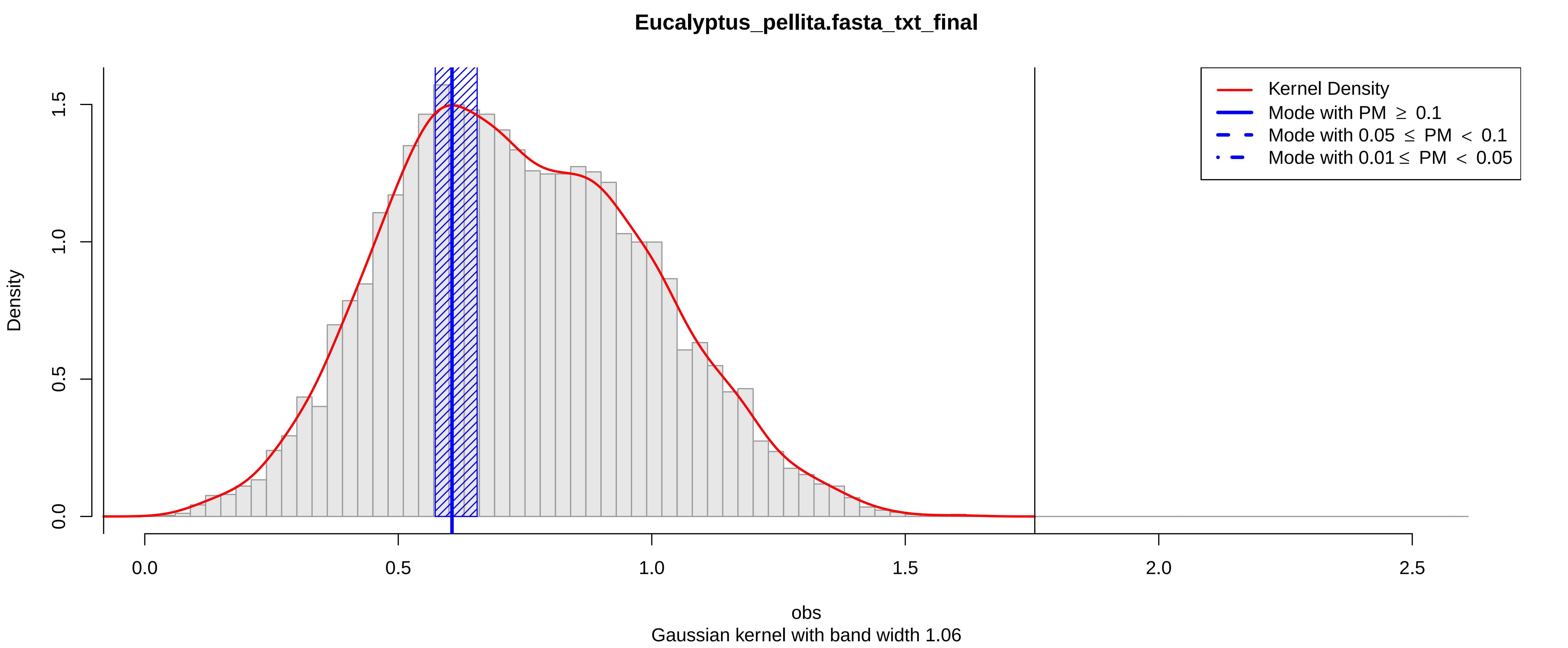

Eucalyptus_pellita.fasta_txt_final
Kernel Density
Mode with PM ≥ 0.1
Mode with 0.05 ≤ PM < 0.1
Mode with 0.01 ≤ PM < 0.05
Density
0.0
0.5
1.0
1.5
0.0
0.5
1.0
1.5
2.0
2.5
obs
Gaussian kernel with band width 1.06

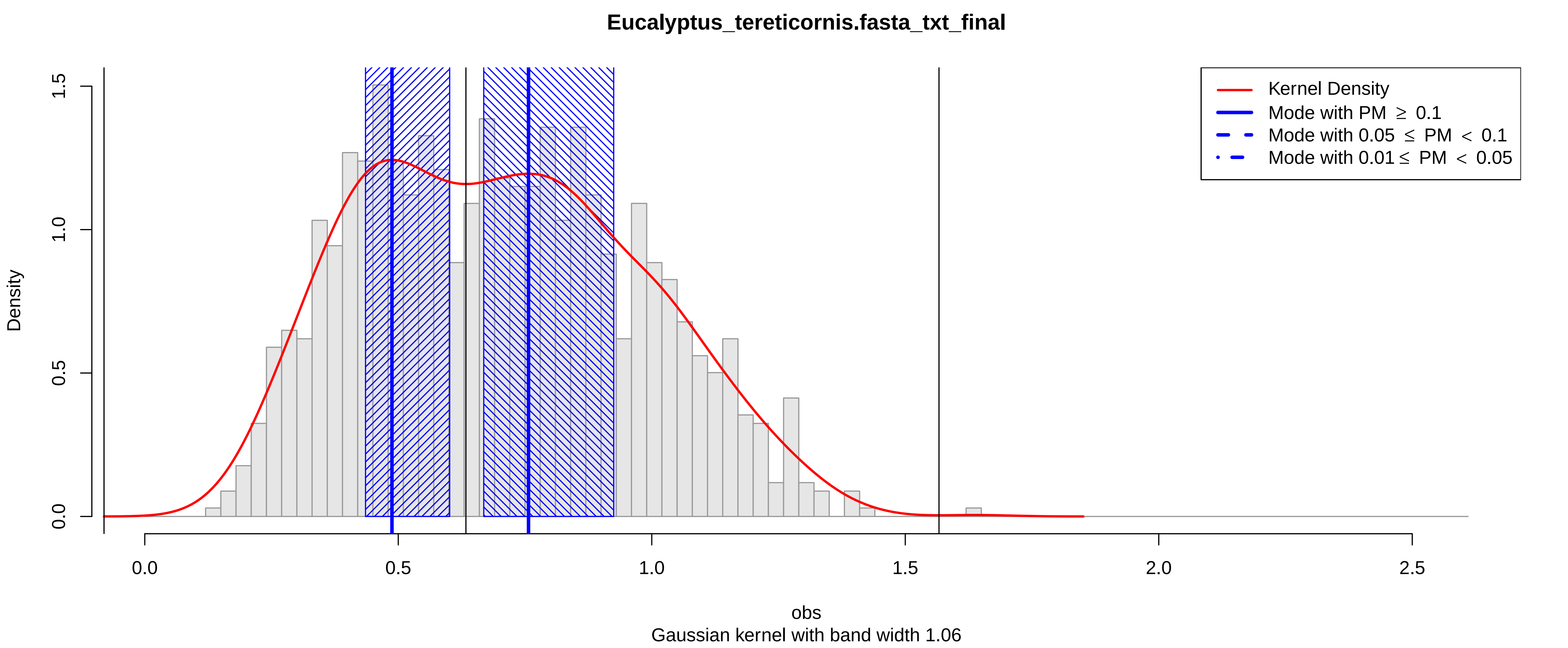

Eucalyptus_tereticornis.fasta_txt_final
Density
0.0
0.5
1.0
1.5
0.0
0.5
1.0
1.5
2.0
2.5
obs
Gaussian kernel with band width 1.06
Kernel Density
Mode with PM ≥ 0.1
Mode with 0.05 ≤ PM < 0.1
Mode with 0.01 ≤ PM < 0.05

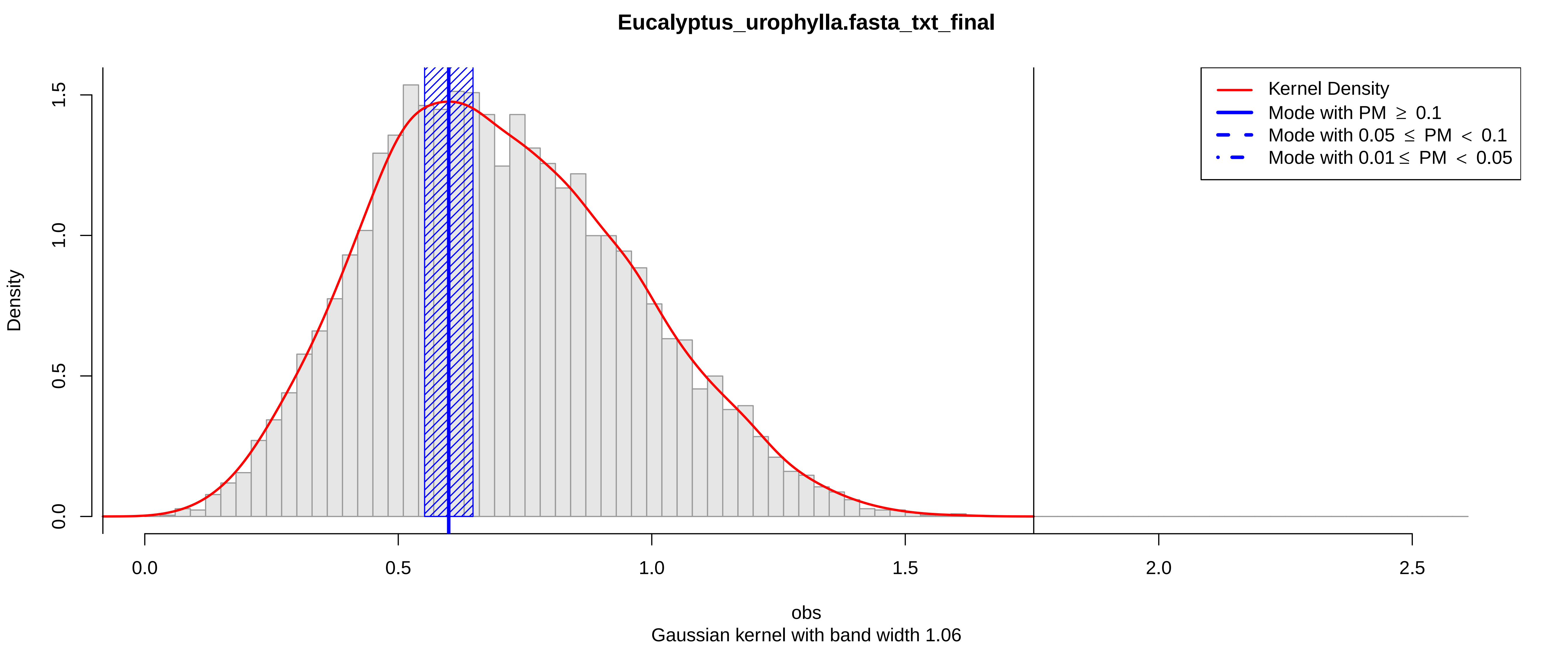

Eucalyptus_urophylla.fasta_txt_final
Kernel Density
Mode with PM ≥ 0.1
Mode with 0.05 ≤ PM < 0.1
Mode with 0.01 ≤ PM < 0.05
Density
0.0
0.5
1.0
1.5
0.0
0.5
1.0
1.5
2.0
2.5
obs
Gaussian kernel with band width 1.06

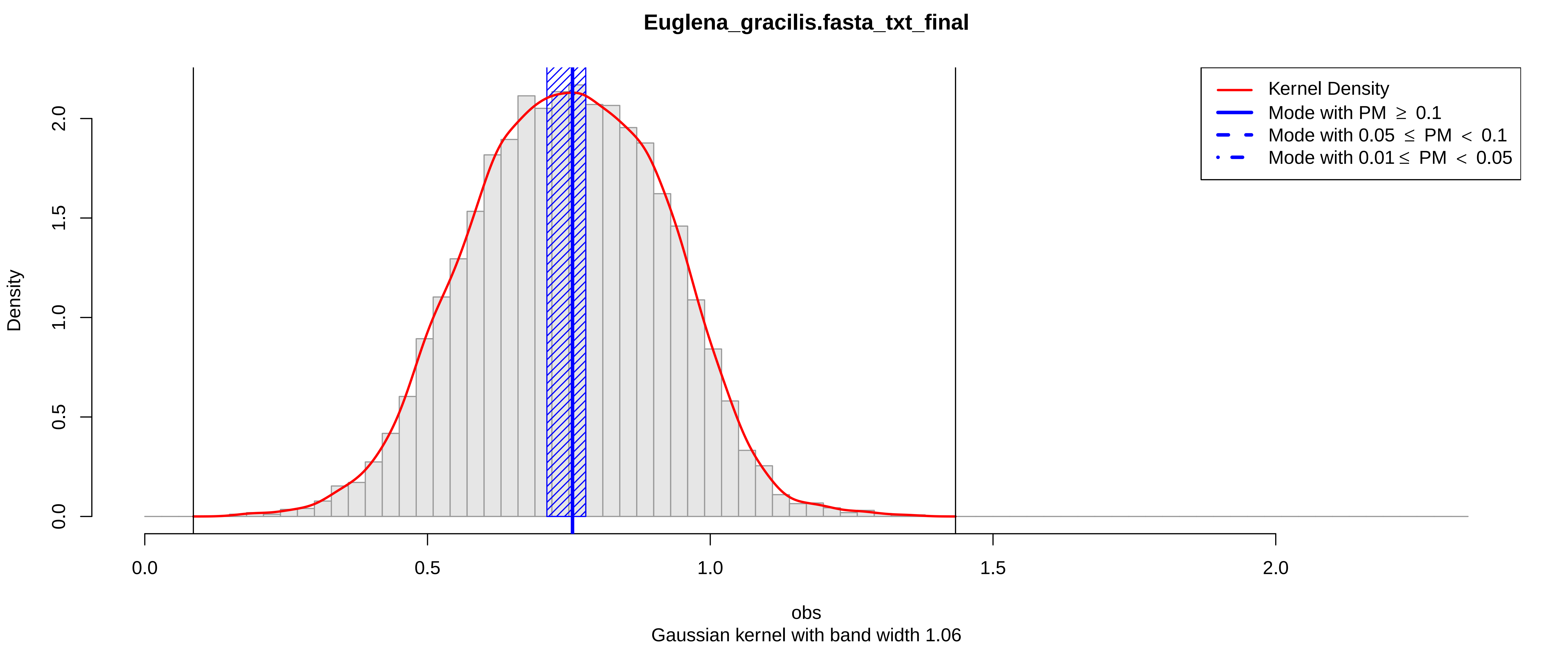

Euglena_gracilis.fasta_txt_final
Kernel Density
Mode with PM ≥ 0.1
Mode with 0.05 ≤ PM < 0.1
Mode with 0.01 ≤ PM < 0.05
Density
0.0
0.5
1.0
1.5
2.0
0.0
0.5
1.0
1.5
2.0
obs
Gaussian kernel with band width 1.06

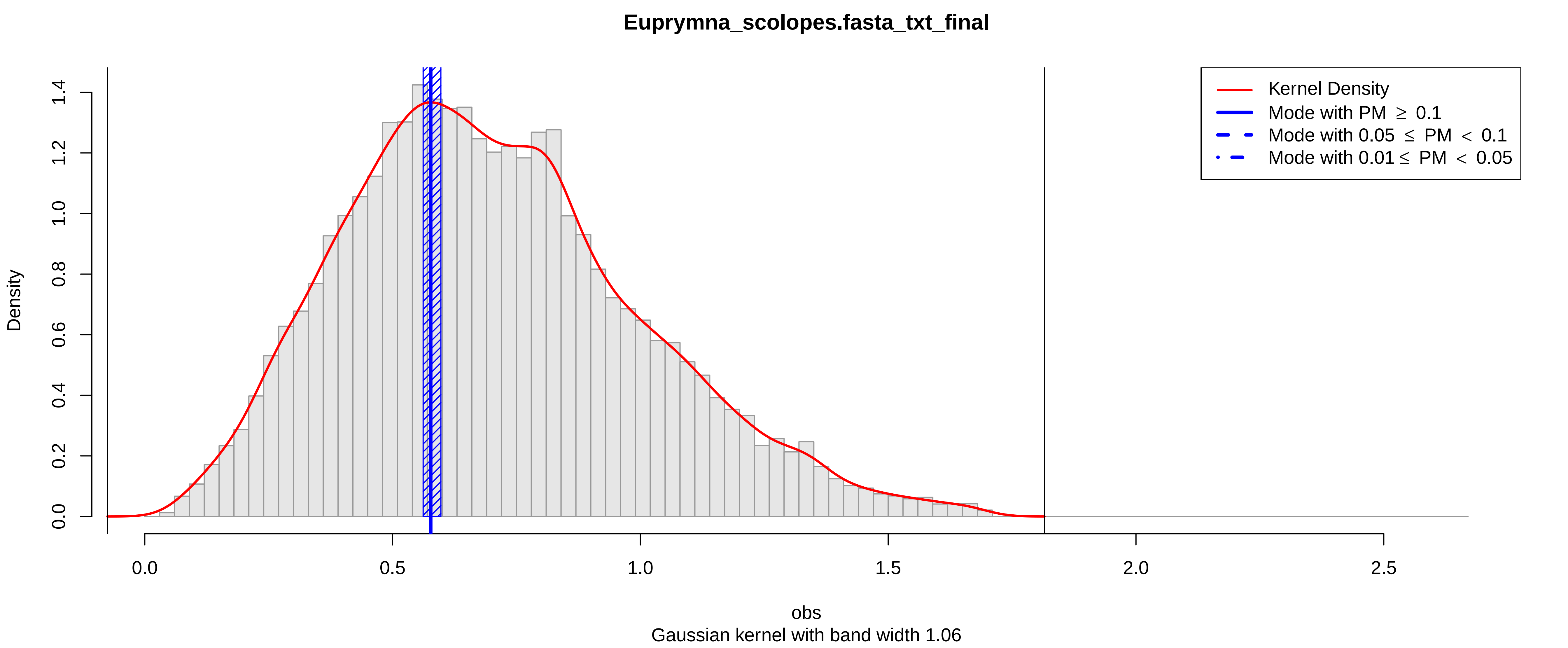

Euprymna_scolopes.fasta_txt_final
Kernel Density
Mode with PM ≥ 0.1
Mode with 0.05 ≤ PM < 0.1
Mode with 0.01 ≤ PM < 0.05
Density
0.0
0.2
0.4
0.6
0.8
1.0
1.2
1.4
0.0
0.5
1.0
1.5
2.0
2.5
obs
Gaussian kernel with band width 1.06

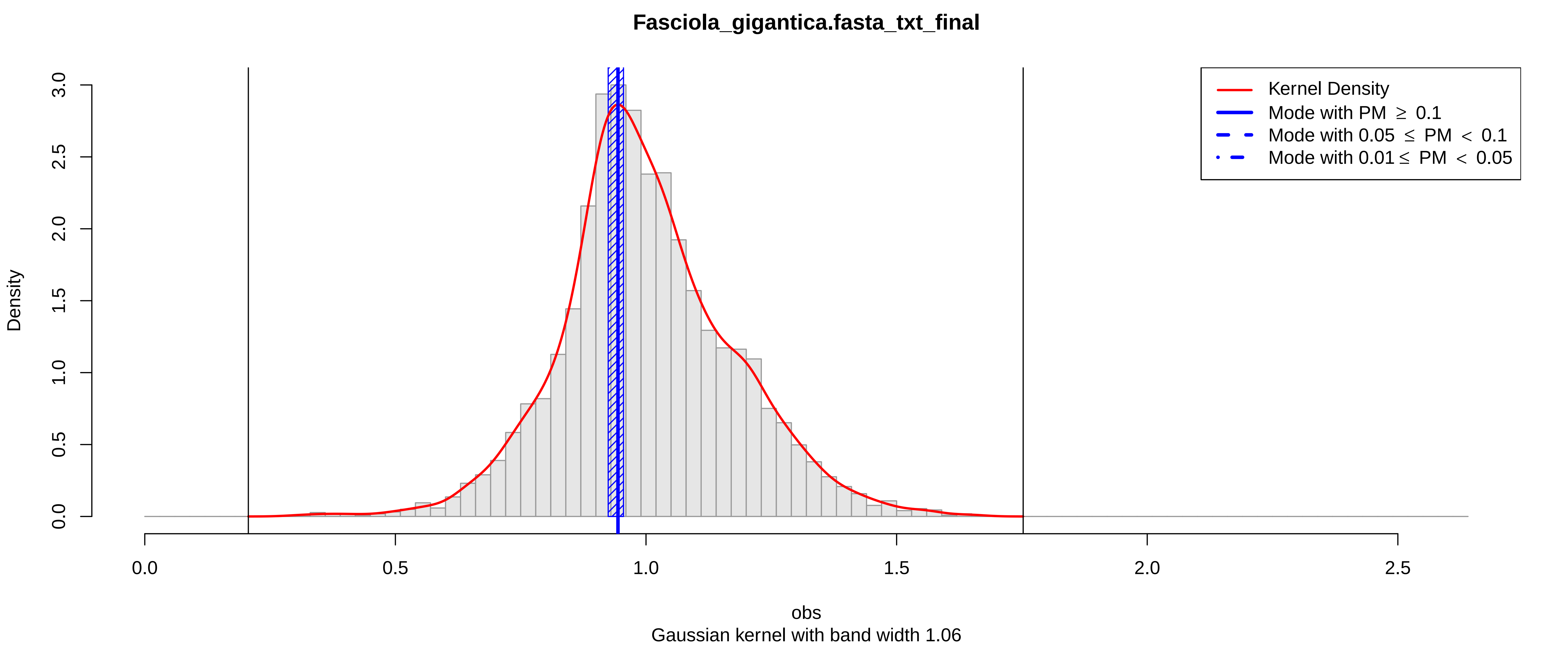

Fasciola_gigantica.fasta_txt_final
Kernel Density
Mode with PM ≥ 0.1
Mode with 0.05 ≤ PM < 0.1
Mode with 0.01 ≤ PM < 0.05
Density
0.0
0.5
1.0
1.5
2.0
2.5
3.0
obs
Gaussian kernel with band width 1.06

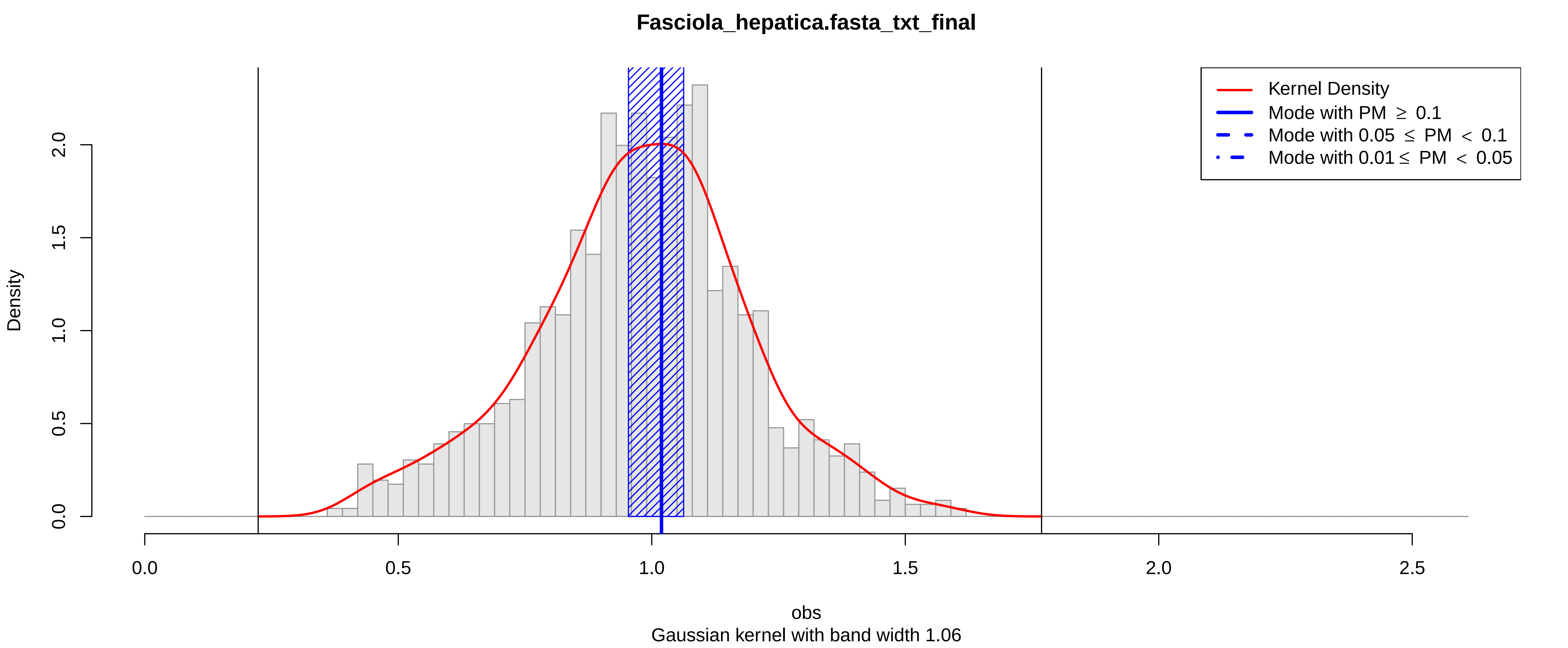

Fasciola_hepatica.fasta_txt_final
Kernel Density
Mode with PM ≥ 0.1
Mode with 0.05 ≤ PM < 0.1
Mode with 0.01 ≤ PM < 0.05
Density
0.0
0.5
1.0
1.5
2.0
2.5
obs
Gaussian kernel with band width 1.06

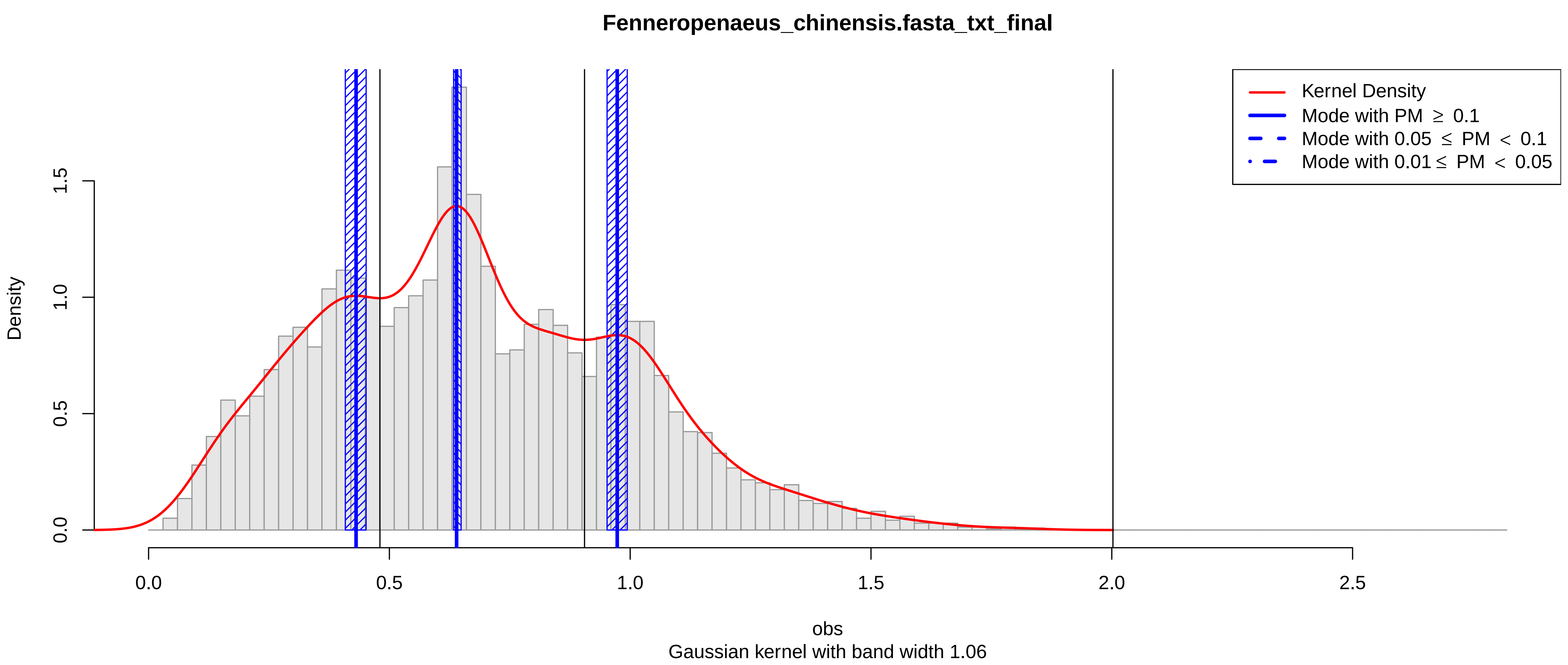
Fenneropenaeus_chinensis.fasta_txt_final
Kernel Density
Mode with PM ≥ 0.1
Mode with 0.05 ≤ PM < 0.1
Mode with 0.01 ≤ PM < 0.05
Density
0.0
0.5
1.0
1.5
0.0
0.5
1.0
1.5
2.0
2.5
obs
Gaussian kernel with band width 1.06

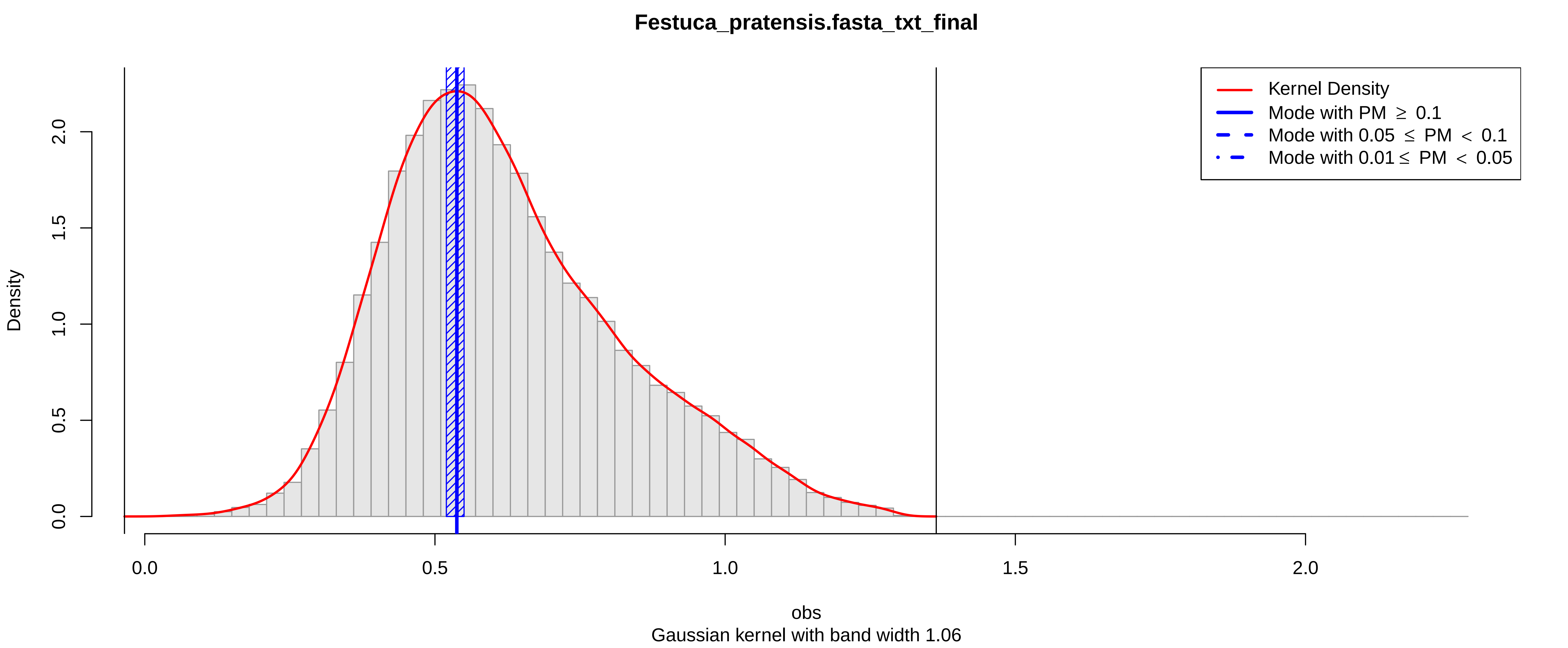

Festuca_pratensis.fasta_txt_final
Kernel Density
Mode with PM ≥ 0.1
Mode with 0.05 ≤ PM < 0.1
Mode with 0.01 ≤ PM < 0.05
Density
0.0
0.5
1.0
1.5
2.0
0.0
0.5
1.0
1.5
2.0
obs
Gaussian kernel with band width 1.06

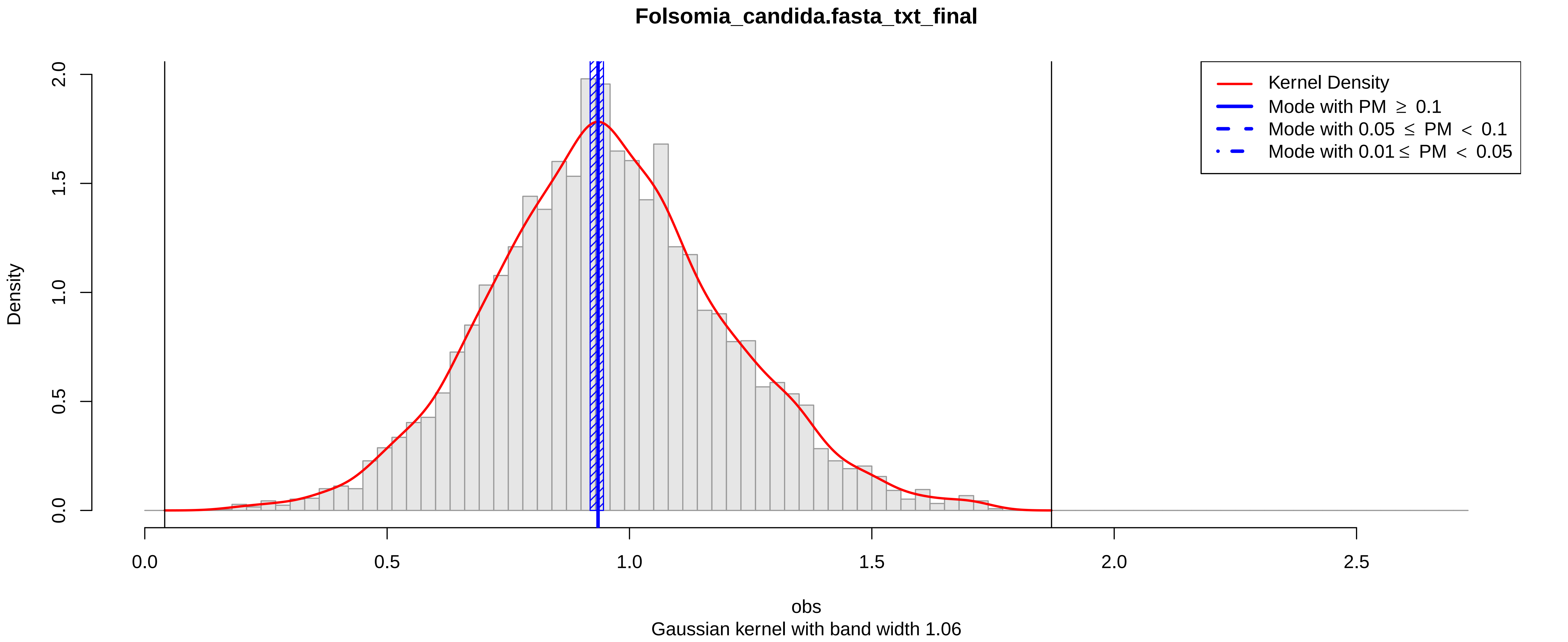
Folsomia_candida.fasta_txt_final
Kernel Density
Mode with PM ≥ 0.1
Mode with 0.05 ≤ PM < 0.1
Mode with 0.01 ≤ PM < 0.05
Density
0.0
0.5
1.0
1.5
2.0
2.5
obs
Gaussian kernel with band width 1.06

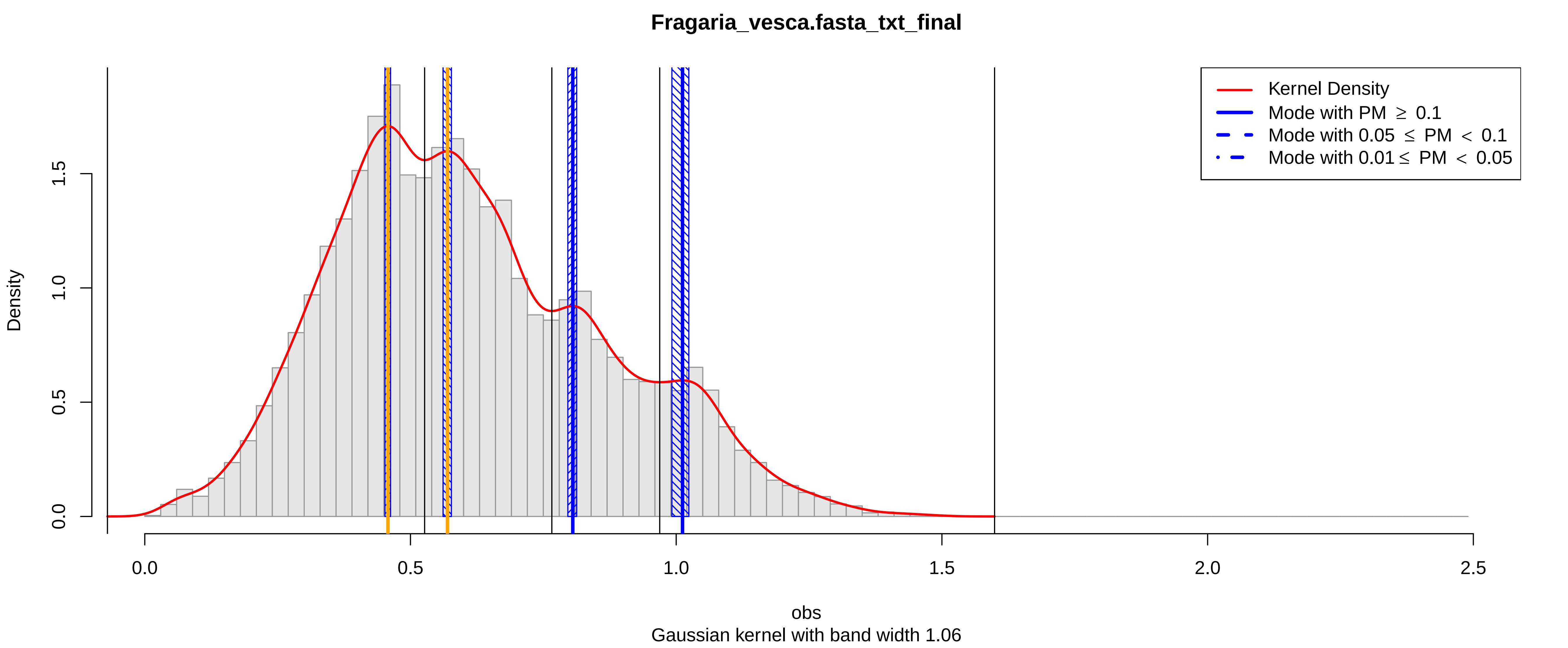

Fragaria_vesca.fasta_txt_final
Kernel Density
Mode with PM ≥ 0.1
Mode with 0.05 ≤ PM < 0.1
Mode with 0.01 ≤ PM < 0.05
Density
0.0
0.5
1.0
1.5
2.0
2.5
obs
Gaussian kernel with band width 1.06

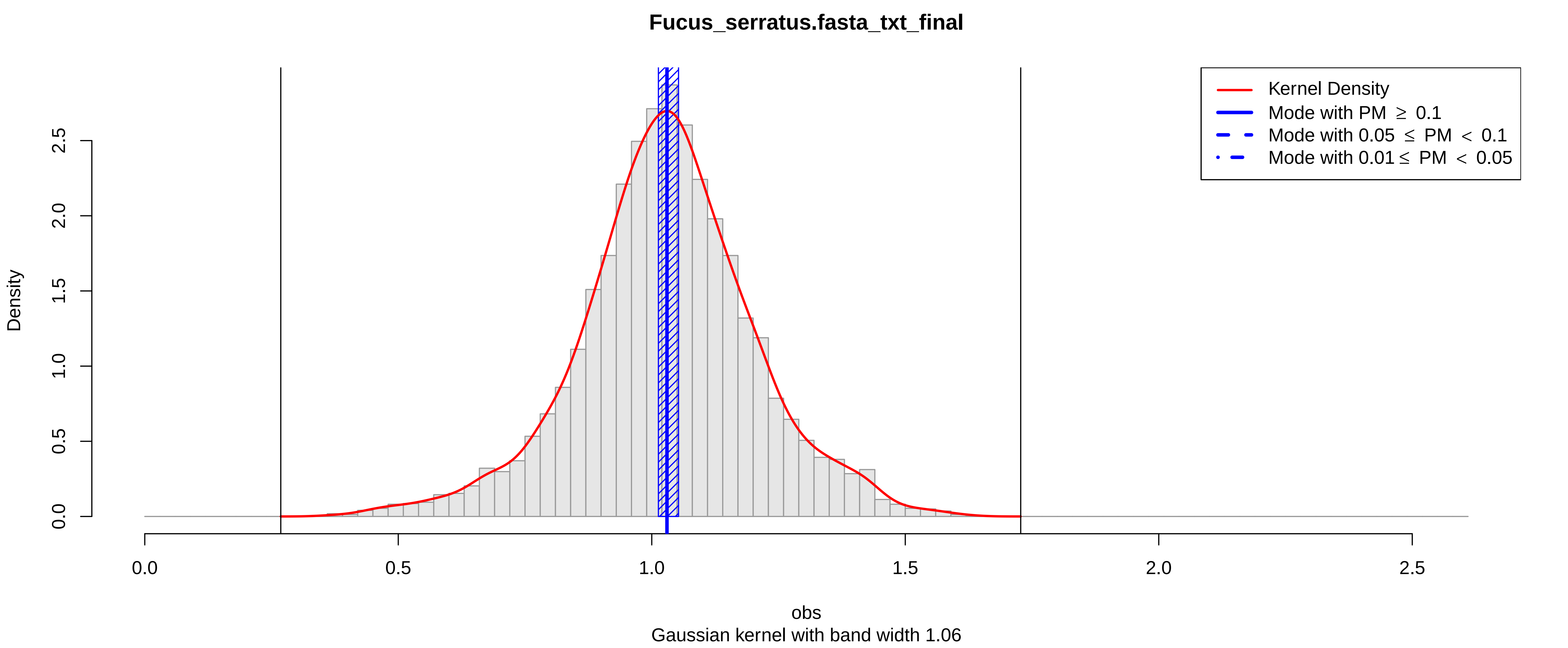
Fucus_serratus.fasta_txt_final
Kernel Density
Mode with PM ≥ 0.1
Mode with 0.05 ≤ PM < 0.1
Mode with 0.01 ≤ PM < 0.05
Density
0.0
0.5
1.0
1.5
2.0
2.5
obs
Gaussian kernel with band width 1.06

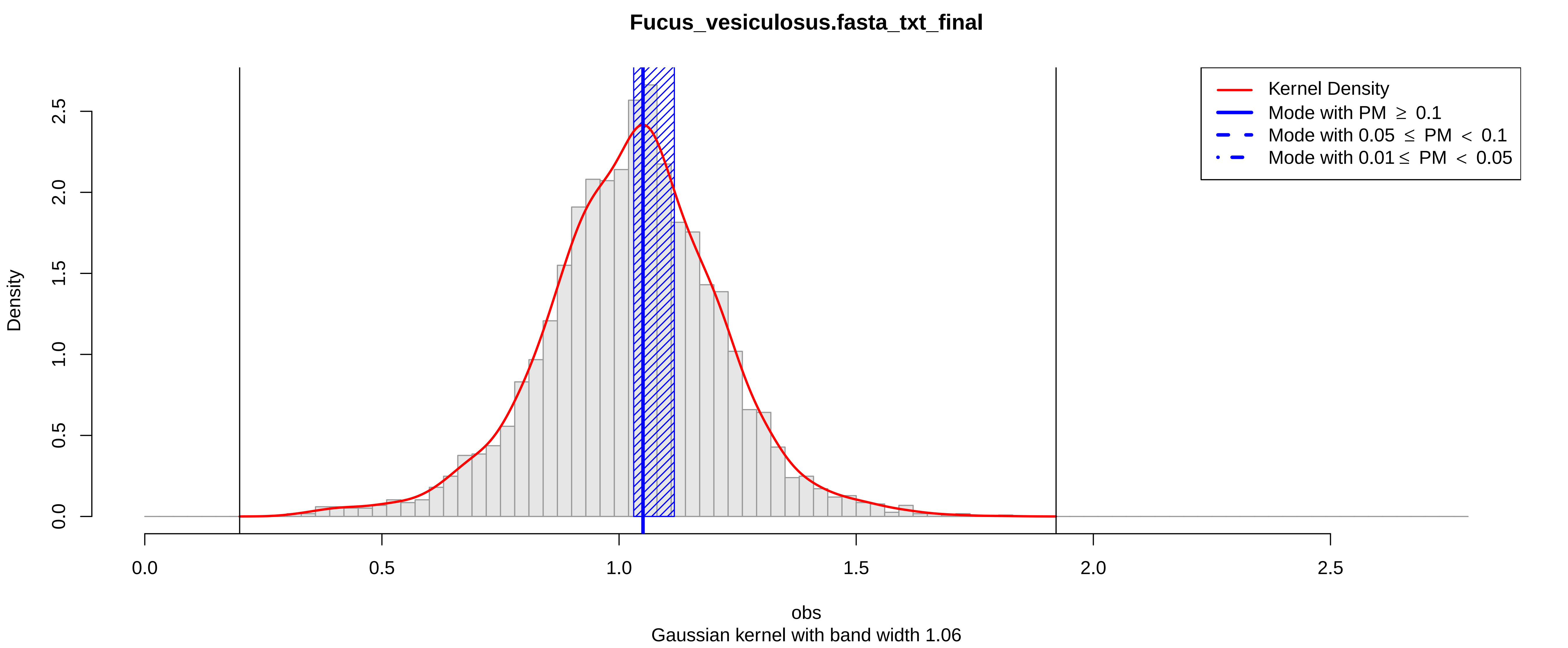
Fucus_vesiculosus.fasta_txt_final
Kernel Density
Mode with PM ≥ 0.1
Mode with 0.05 ≤ PM < 0.1
Mode with 0.01 ≤ PM < 0.05
Density
0.0
0.5
1.0
1.5
2.0
2.5
0.0
0.5
1.0
1.5
2.0
2.5
obs
Gaussian kernel with band width 1.06

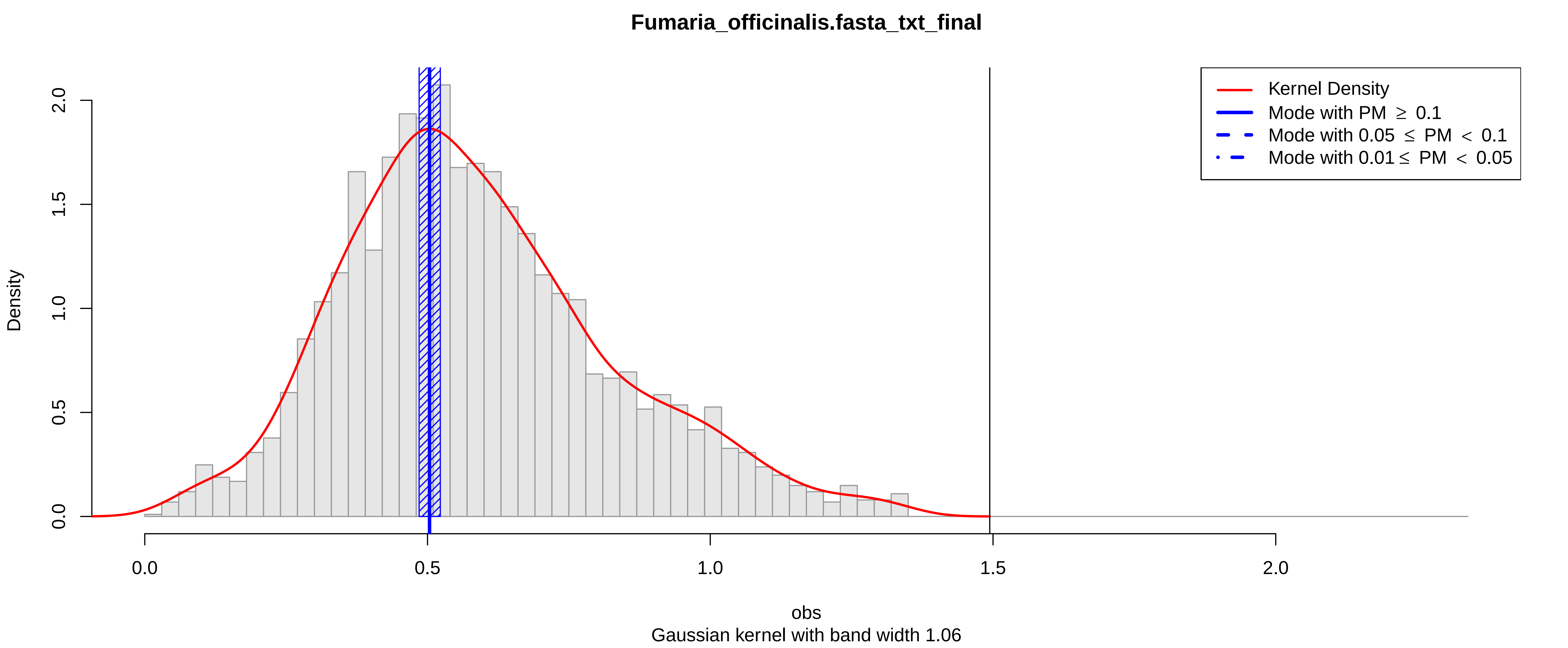

Fumaria_officinalis.fasta_txt_final
Kernel Density
Mode with PM ≥ 0.1
Mode with 0.05 ≤ PM < 0.1
Mode with 0.01 ≤ PM < 0.05
Density
0.0
0.5
1.0
1.5
2.0
obs
Gaussian kernel with band width 1.06

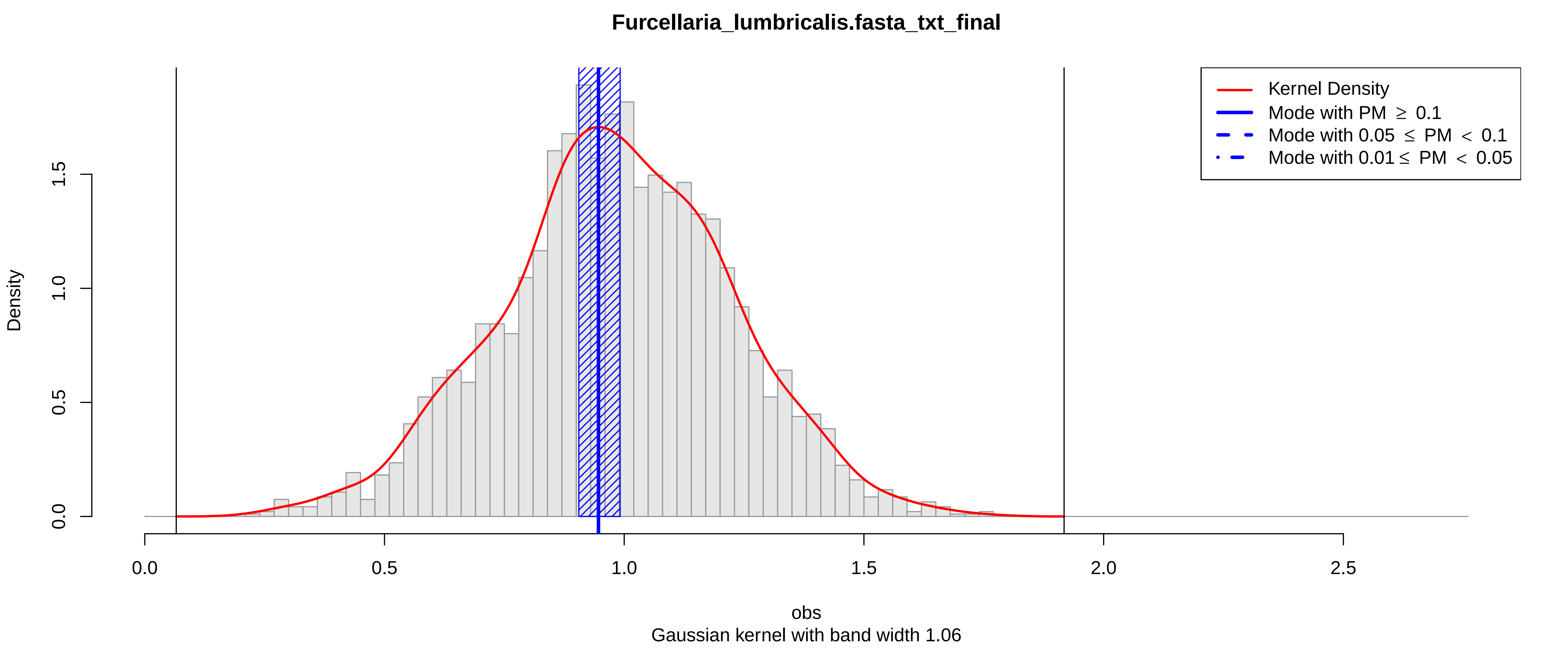

Furcellaria_lumbricalis.fasta_txt_final
Kernel Density
Mode with PM ≥ 0.1
Mode with 0.05 ≤ PM < 0.1
Mode with 0.01 ≤ PM < 0.05
Density
0.0
0.5
1.0
1.5
0.0
0.5
1.0
1.5
2.0
2.5
obs
Gaussian kernel with band width 1.06

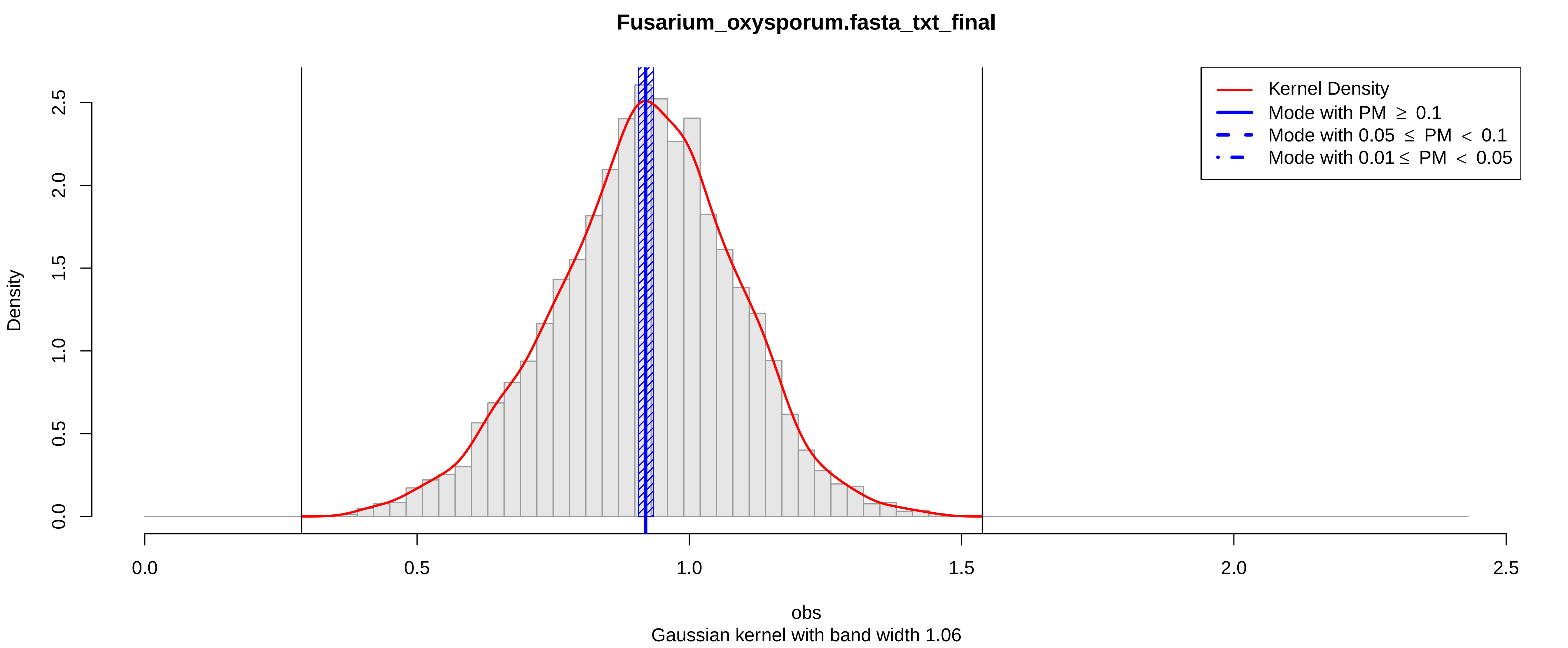

Fusarium_oxysporum.fasta_txt_final
Kernel Density
Mode with PM ≥ 0.1
Mode with 0.05 ≤ PM < 0.1
Mode with 0.01 ≤ PM < 0.05
Density
0.0
0.5
1.0
1.5
2.0
2.5
obs
Gaussian kernel with band width 1.06

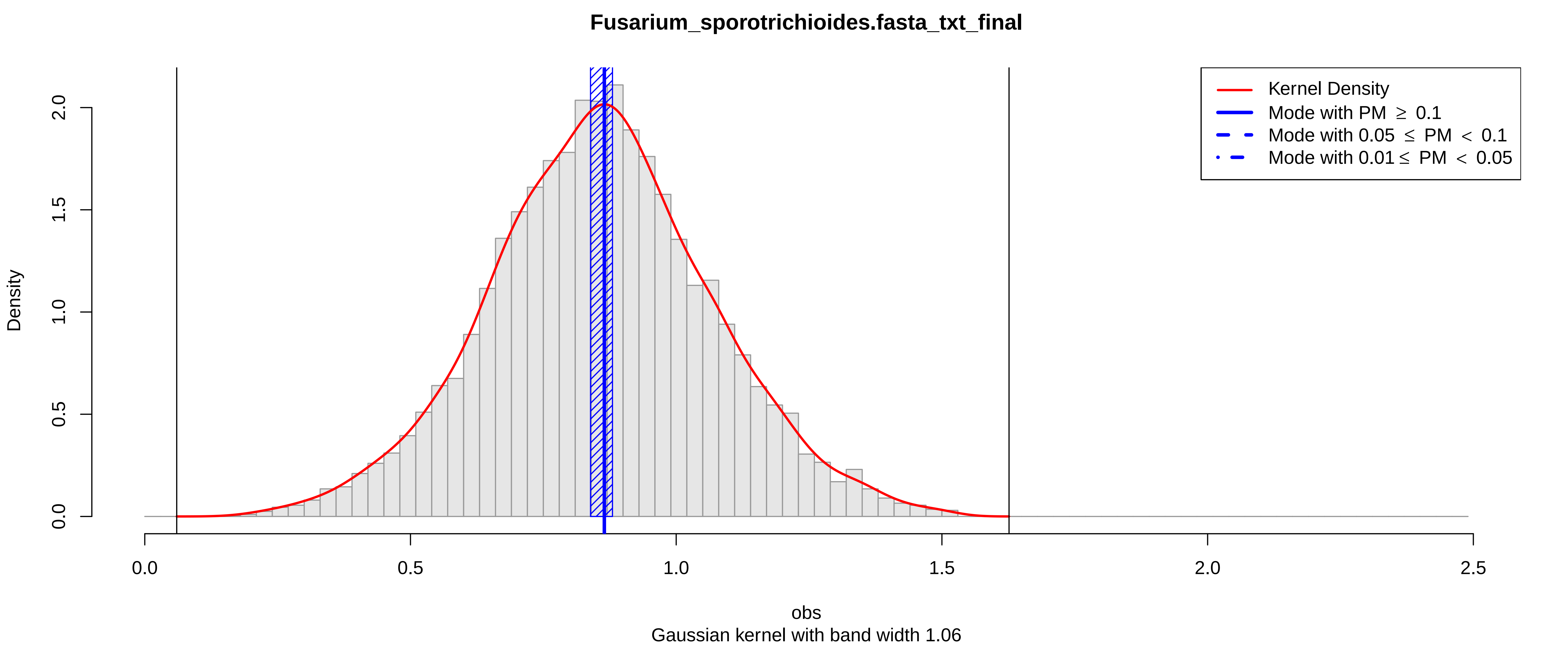

Fusarium_sporotrichioides.fasta_txt_final
Kernel Density
Mode with PM ≥ 0.1
Mode with 0.05 ≤ PM < 0.1
Mode with 0.01 ≤ PM < 0.05
Density
0.0
0.5
1.0
1.5
2.0
2.5
obs
Gaussian kernel with band width 1.06

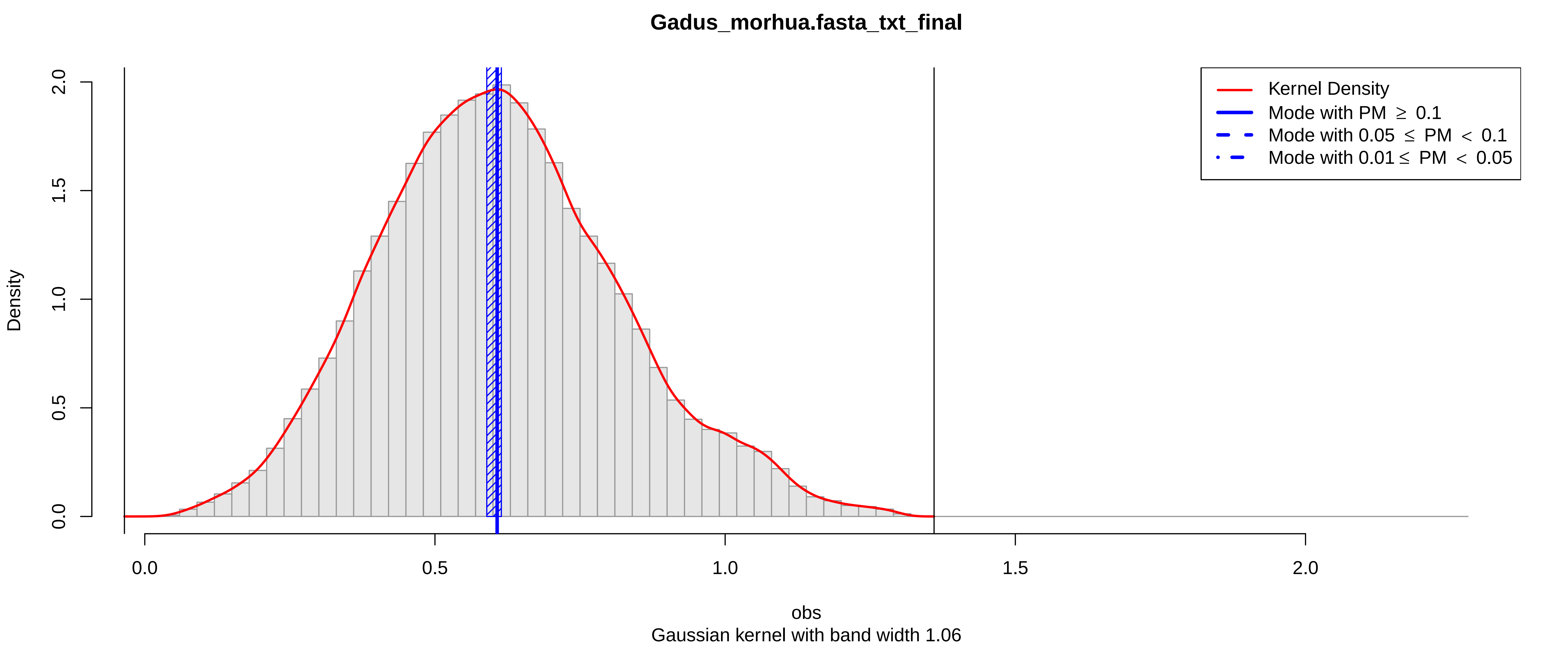

Gadus_morhua.fasta_txt_final
Kernel Density
Mode with PM ≥ 0.1
Mode with 0.05 ≤ PM < 0.1
Mode with 0.01 ≤ PM < 0.05
Density
0.0
0.5
1.0
1.5
2.0
0.0
0.5
1.0
1.5
2.0
obs
Gaussian kernel with band width 1.06

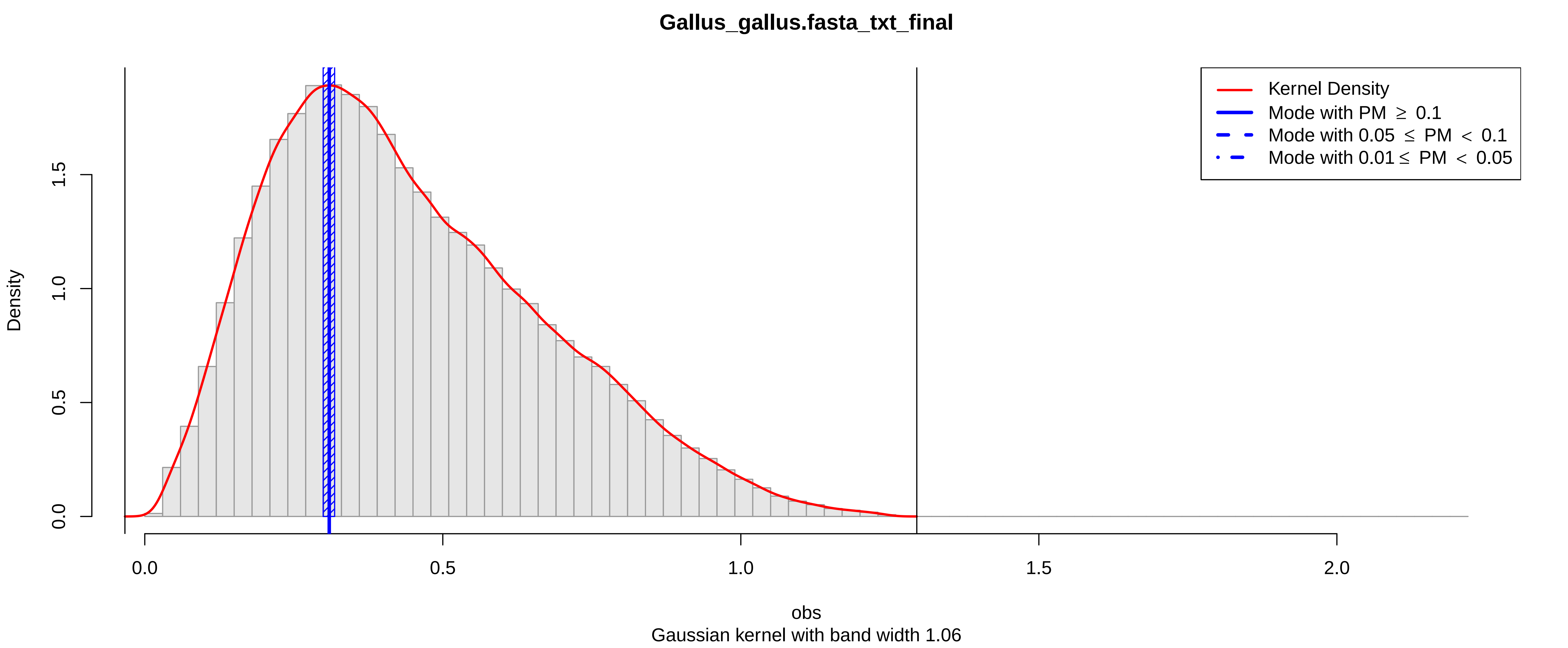

Gallus_gallus.fasta_txt_final
Kernel Density
Mode with PM ≥ 0.1
Mode with 0.05 ≤ PM < 0.1
Mode with 0.01 ≤ PM < 0.05
Density
0.0
0.5
1.0
1.5
0.0
0.5
1.0
1.5
2.0
obs
Gaussian kernel with band width 1.06

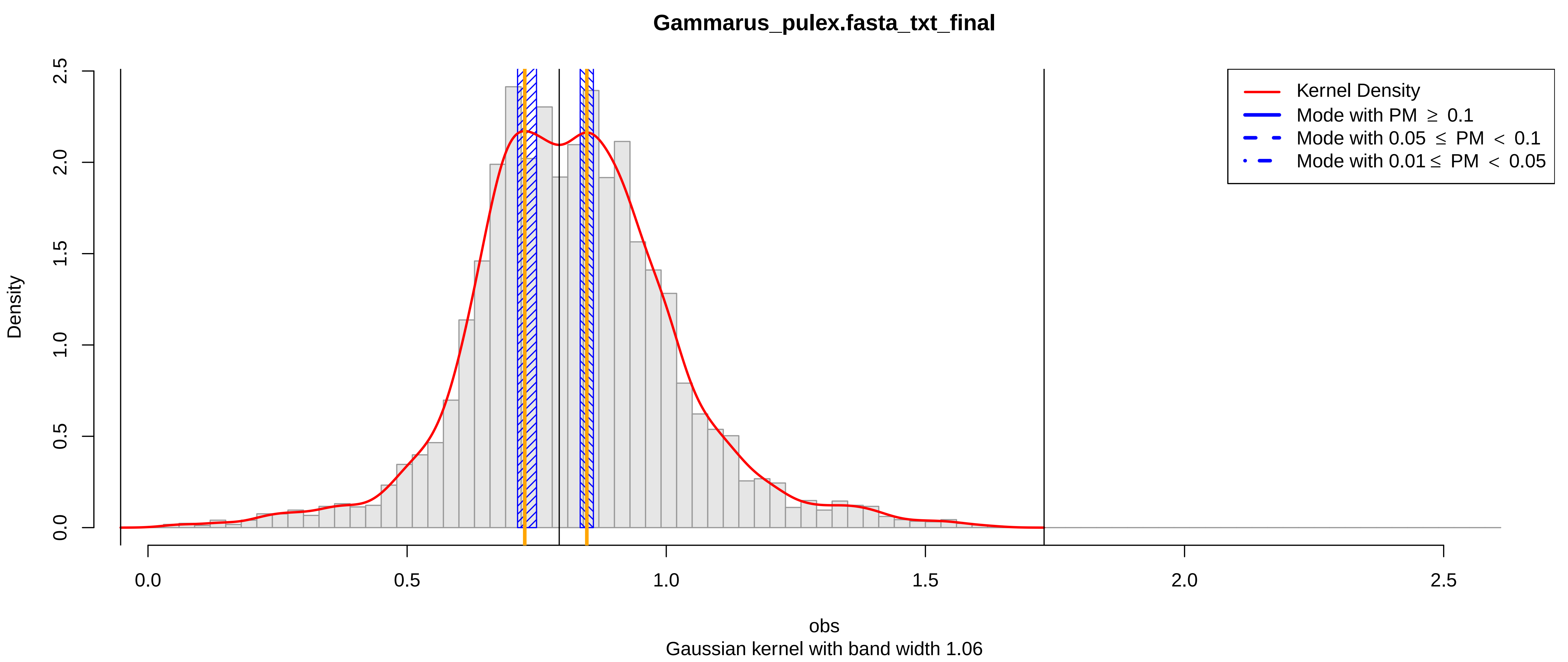

Gammarus_pulex.fasta_txt_final
Density
0.0
0.5
1.0
1.5
2.0
2.5
Kernel Density
Mode with PM ≥ 0.1
Mode with 0.05 ≤ PM < 0.1
Mode with 0.01 ≤ PM < 0.05
obs
Gaussian kernel with band width 1.06

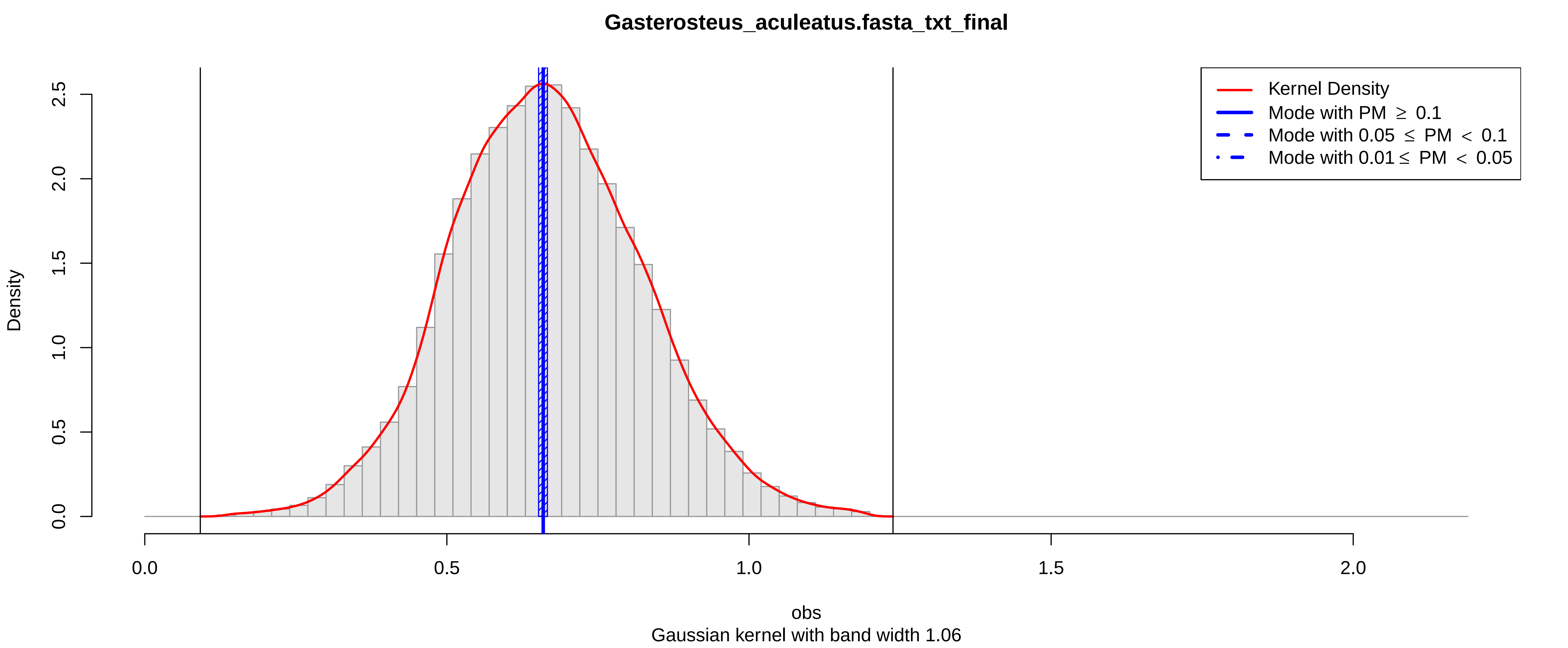

Gasterosteus_aculeatus.fasta_txt_final
Kernel Density
Mode with PM ≥ 0.1
Mode with 0.05 ≤ PM < 0.1
Mode with 0.01 ≤ PM < 0.05
Density
0.0
0.5
1.0
1.5
2.0
2.5
0.0
0.5
1.0
1.5
2.0
obs
Gaussian kernel with band width 1.06

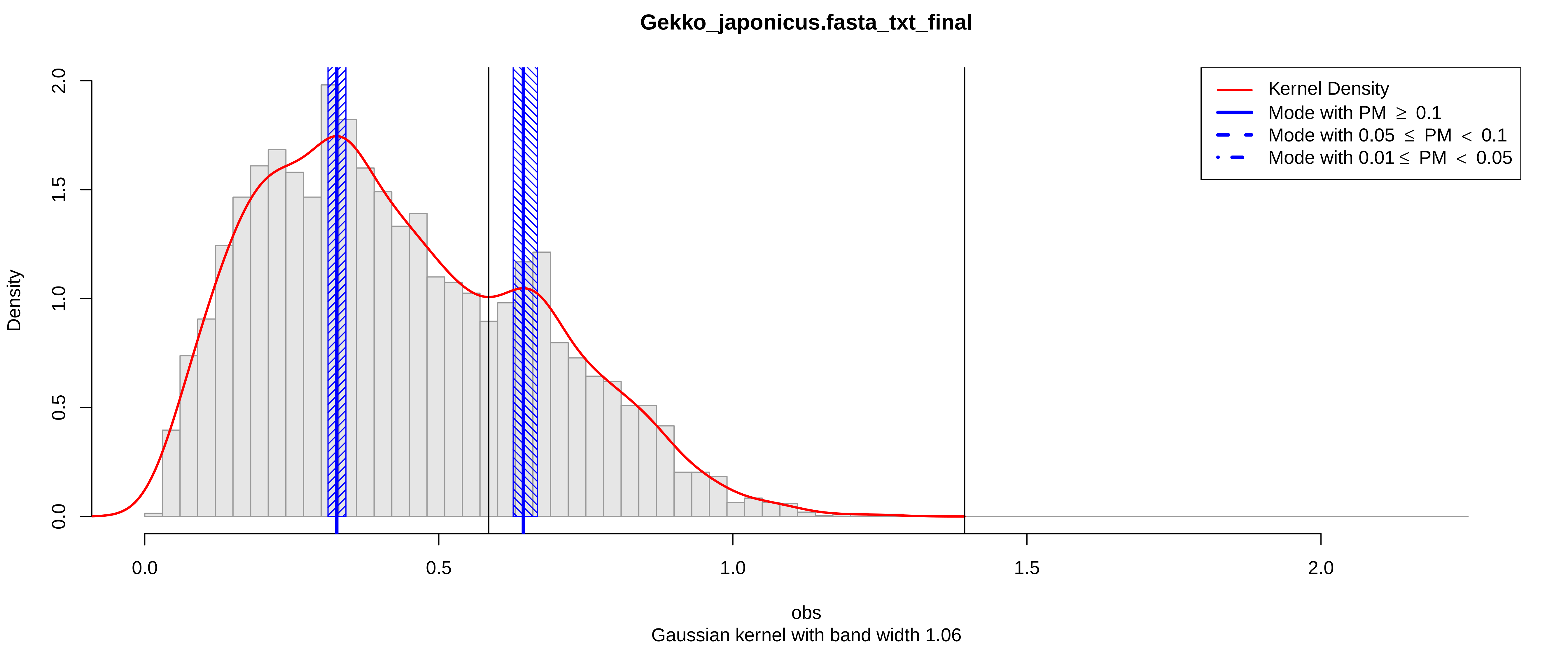

Gekko_japonicus.fasta_txt_final
Kernel Density
Mode with PM ≥ 0.1
Mode with 0.05 ≤ PM < 0.1
Mode with 0.01 ≤ PM < 0.05
Density
0.0
0.5
1.0
1.5
2.0
0.0
0.5
1.0
1.5
2.0
obs
Gaussian kernel with band width 1.06

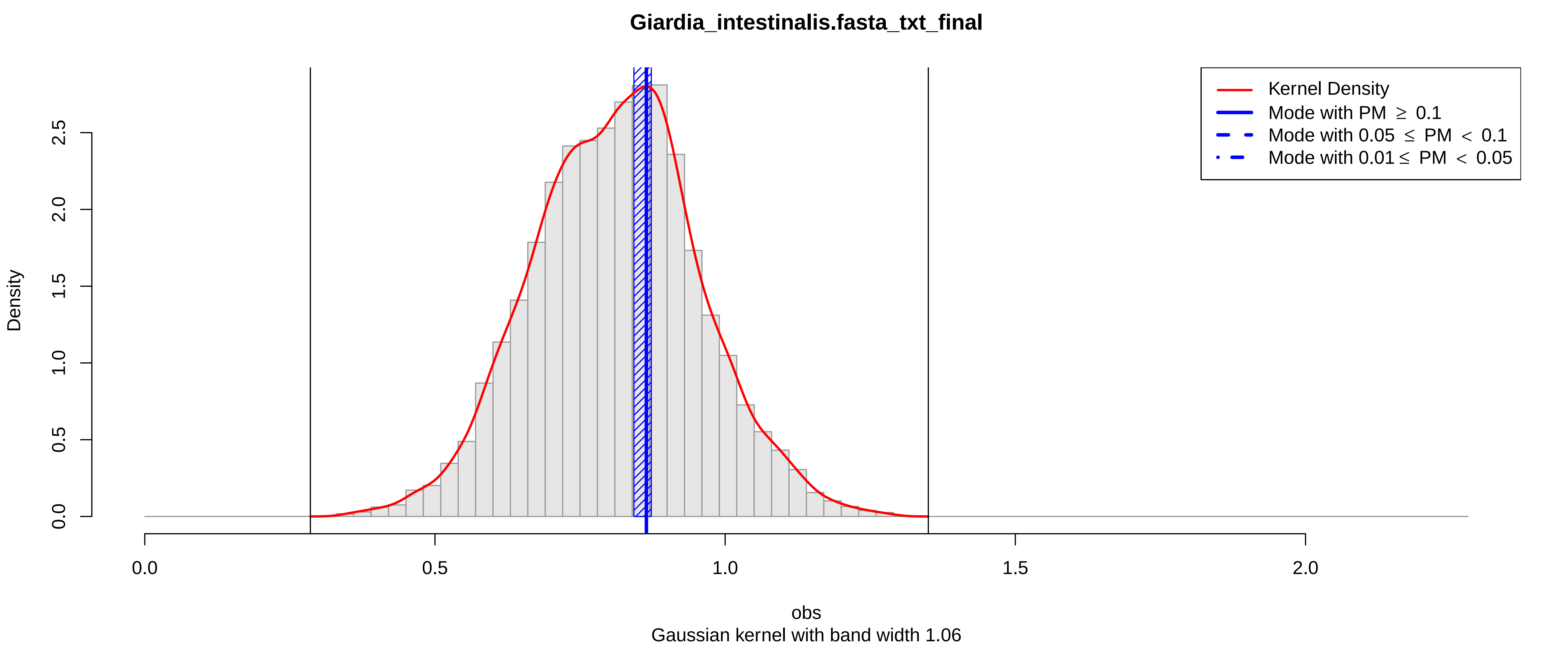
Giardia_intestinalis.fasta_txt_final
Kernel Density
Mode with PM ≥ 0.1
Mode with 0.05 ≤ PM < 0.1
Mode with 0.01 ≤ PM < 0.05
Density
0.0
0.5
1.0
1.5
2.0
2.5
obs
Gaussian kernel with band width 1.06

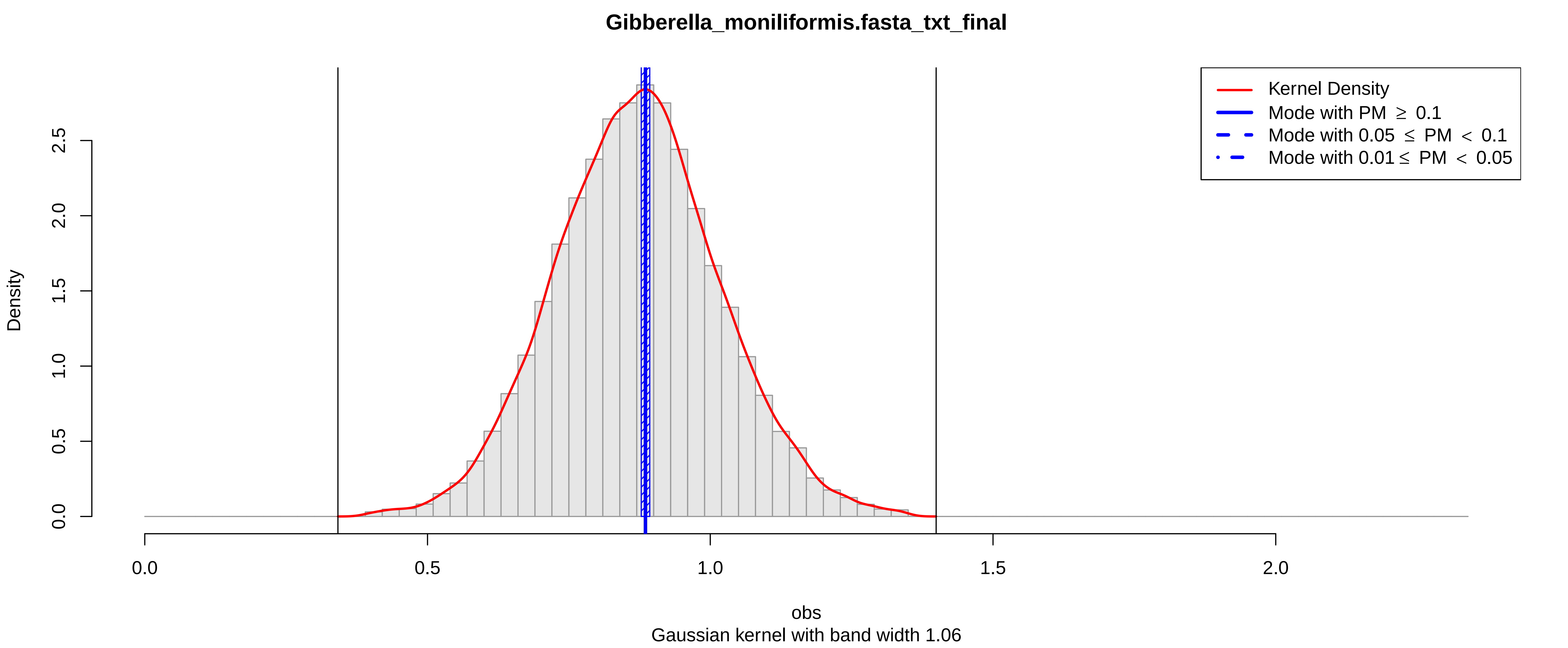

Gibberella_moniliformis.fasta_txt_final
Kernel Density
Mode with PM ≥ 0.1
Mode with 0.05 ≤ PM < 0.1
Mode with 0.01 ≤ PM < 0.05
Density
0.0
0.5
1.0
1.5
2.0
2.5
0.0
0.5
1.0
1.5
2.0
obs
Gaussian kernel with band width 1.06

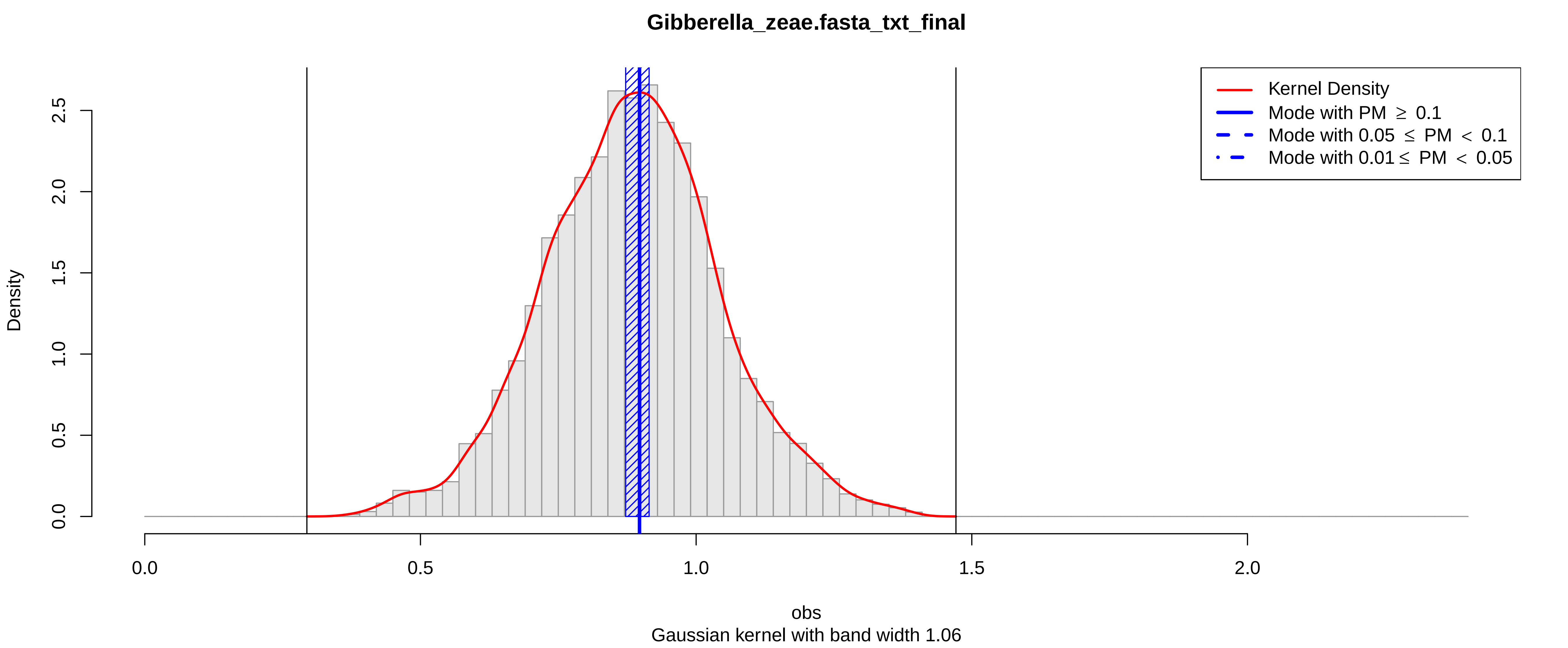
Gibberella_zeae.fasta_txt_final
Kernel Density
Mode with PM ≥ 0.1
Mode with 0.05 ≤ PM < 0.1
Mode with 0.01 ≤ PM < 0.05
Density
0.0
0.5
1.0
1.5
2.0
2.5
0.0
0.5
1.0
1.5
2.0
obs
Gaussian kernel with band width 1.06

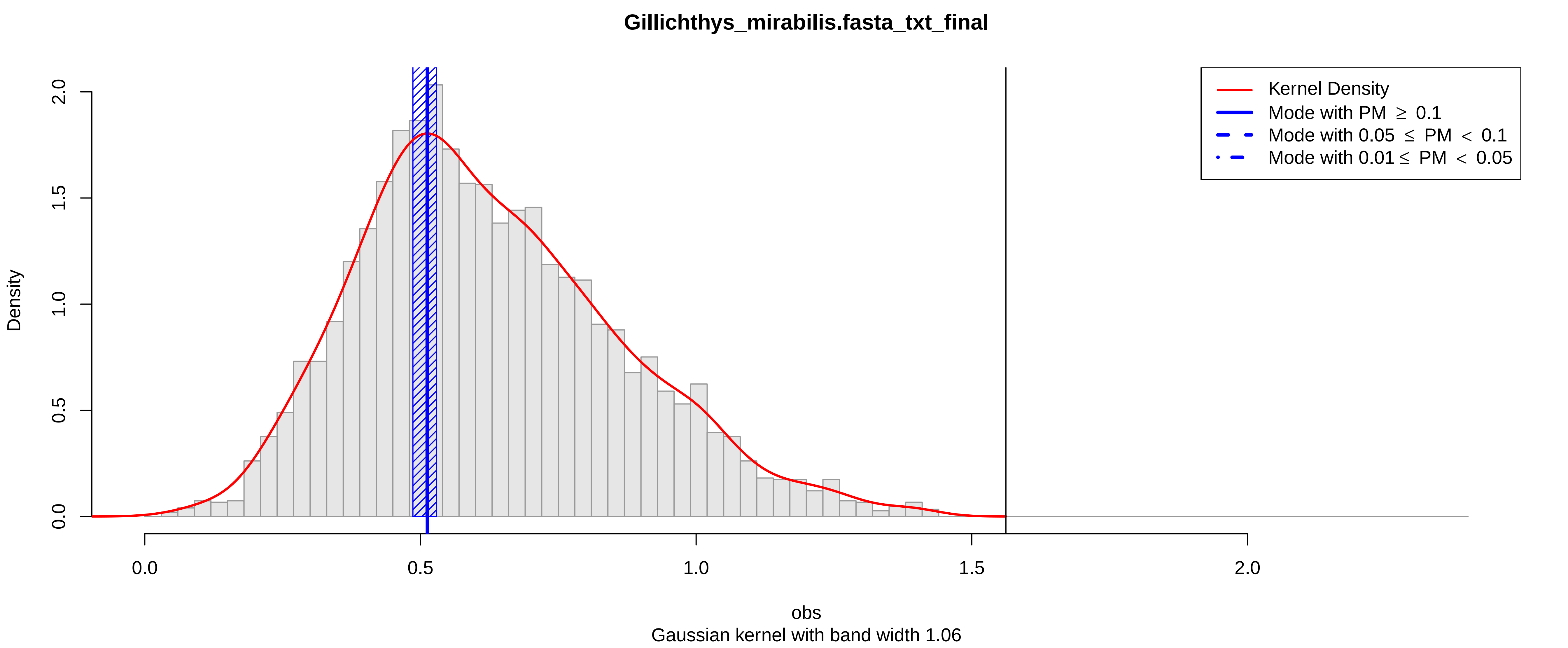
Gillichthys_mirabilis.fasta_txt_final
Kernel Density
Mode with PM ≥ 0.1
Mode with 0.05 ≤ PM < 0.1
Mode with 0.01 ≤ PM < 0.05
Density
0.0
0.5
1.0
1.5
2.0
0.0
0.5
1.0
1.5
2.0
obs
Gaussian kernel with band width 1.06

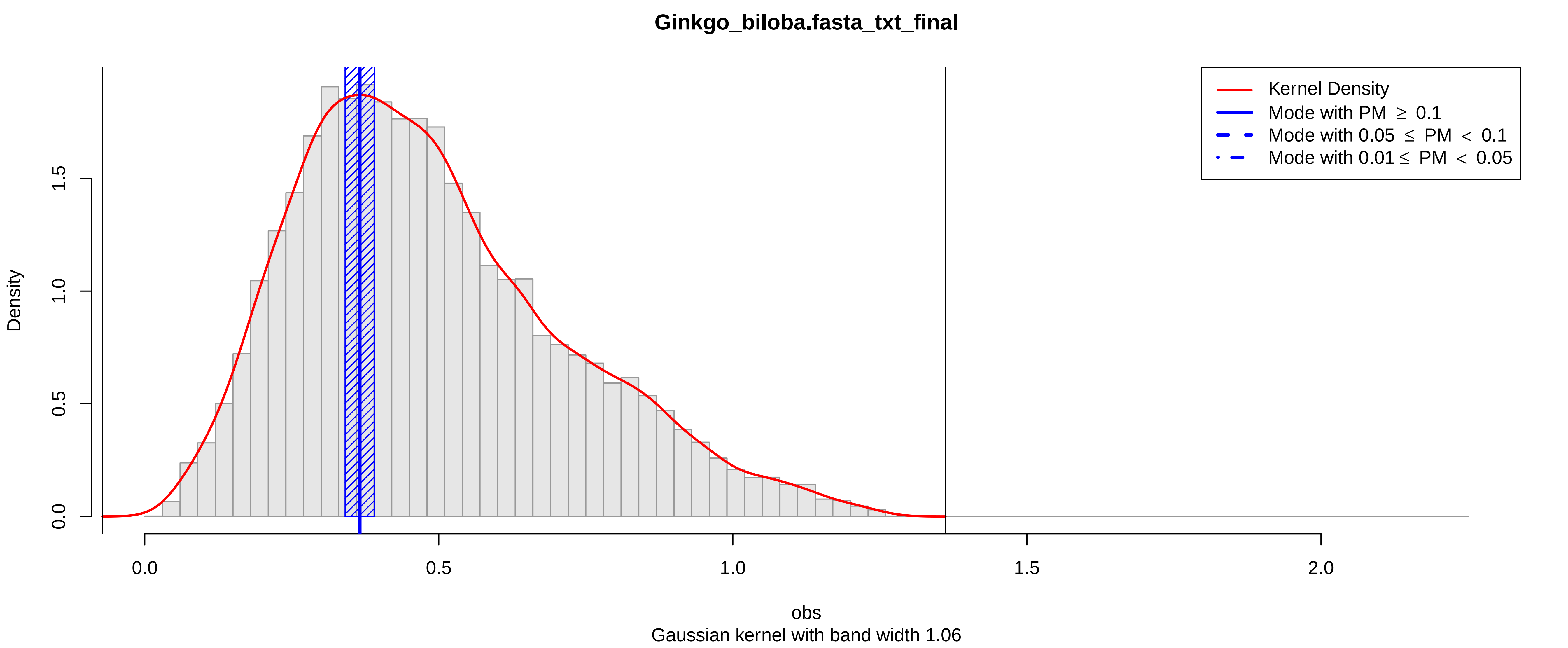

Ginkgo_biloba.fasta_txt_final
Kernel Density
Mode with PM ≥ 0.1
Mode with 0.05 ≤ PM < 0.1
Mode with 0.01 ≤ PM < 0.05
Density
0.0
0.5
1.0
1.5
0.0
0.5
1.0
1.5
2.0
obs
Gaussian kernel with band width 1.06

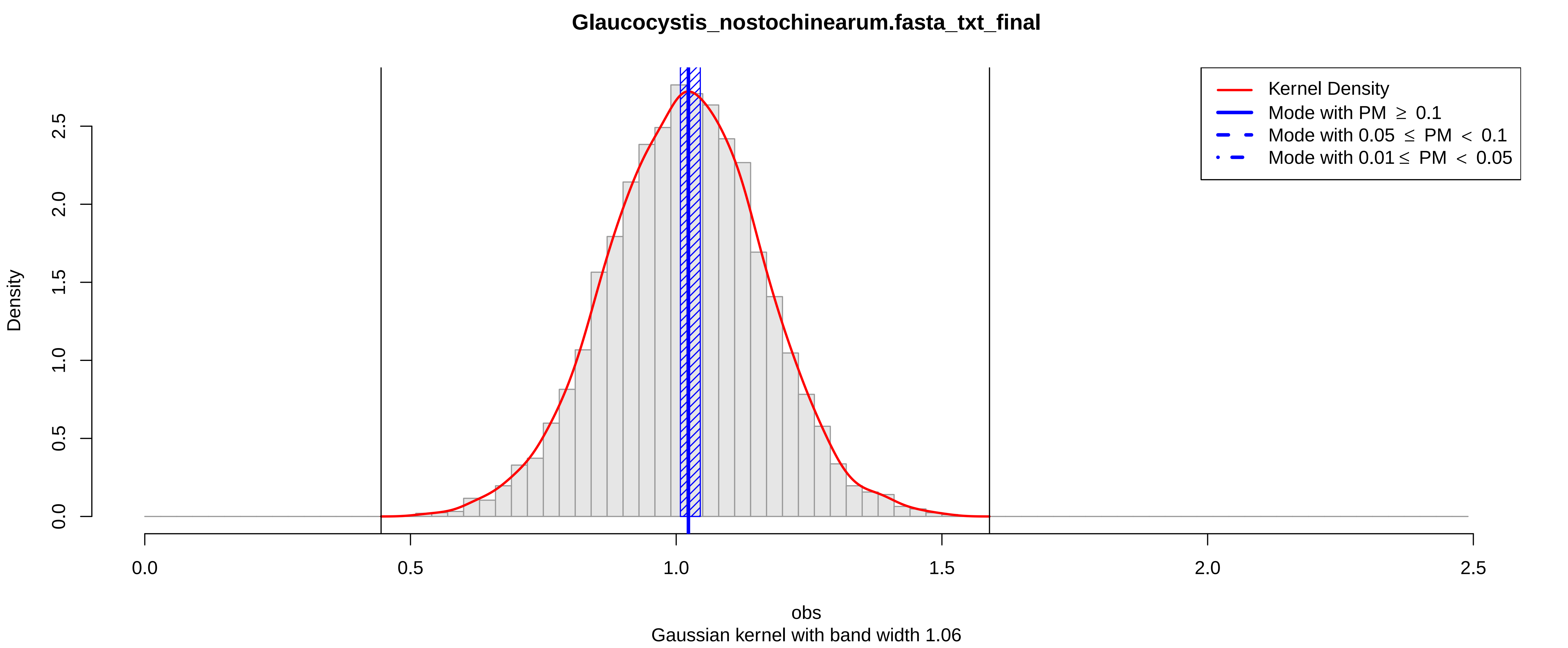

Glaucocystis_nostochinearum.fasta_txt_final
Kernel Density
Mode with PM ≥ 0.1
Mode with 0.05 ≤ PM < 0.1
Mode with 0.01 ≤ PM < 0.05
Density
0.0
0.5
1.0
1.5
2.0
2.5
obs
Gaussian kernel with band width 1.06

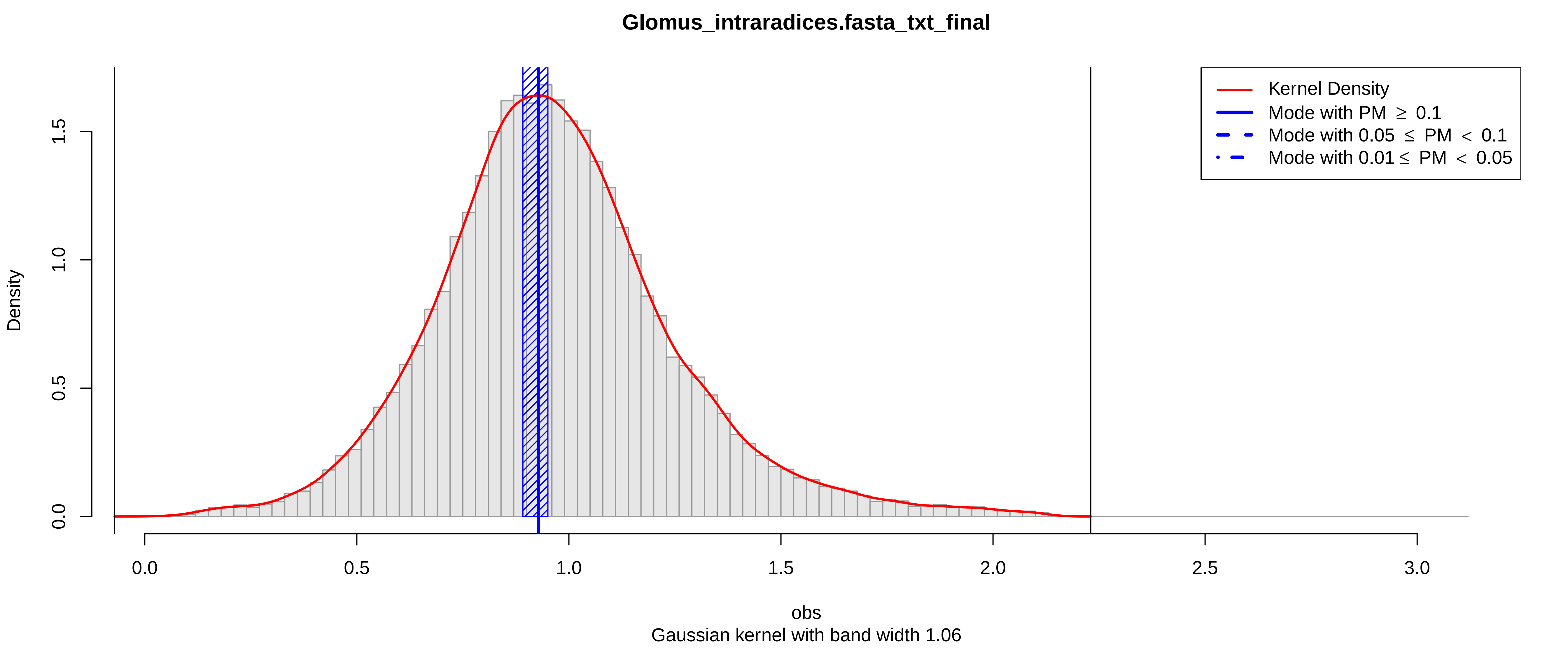

Glomus_intraradices.fasta_txt_final
Kernel Density
Mode with PM ≥ 0.1
Mode with 0.05 ≤ PM < 0.1
Mode with 0.01 ≤ PM < 0.05
Density
0.0
0.5
1.0
1.5
0.0
0.5
1.0
1.5
2.0
2.5
3.0
obs
Gaussian kernel with band width 1.06

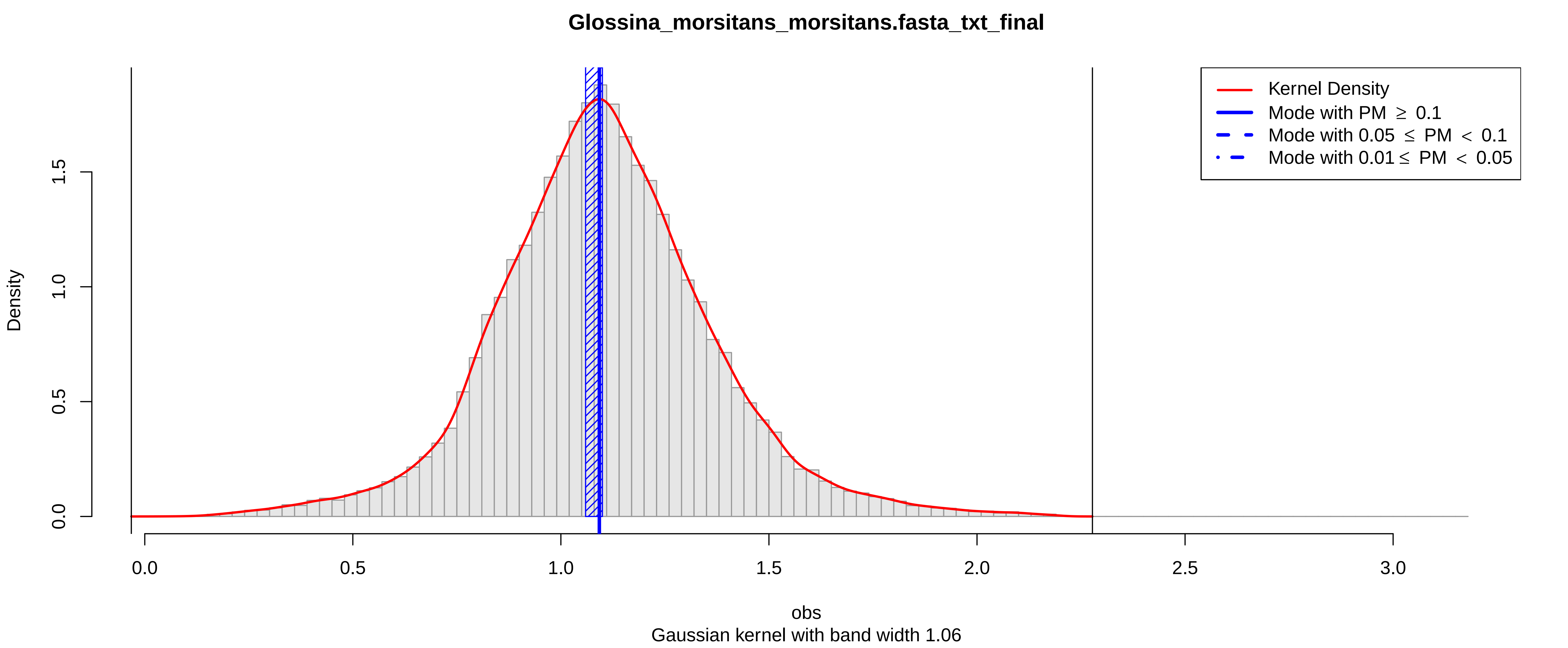

Glossina_morsitans_morsitans.fasta_txt_final
Kernel Density
Mode with PM ≥ 0.1
Mode with 0.05 ≤ PM < 0.1
Mode with 0.01 ≤ PM < 0.05
Density
0.0
0.5
1.0
1.5
0.0
0.5
1.0
1.5
2.0
2.5
3.0
obs
Gaussian kernel with band width 1.06

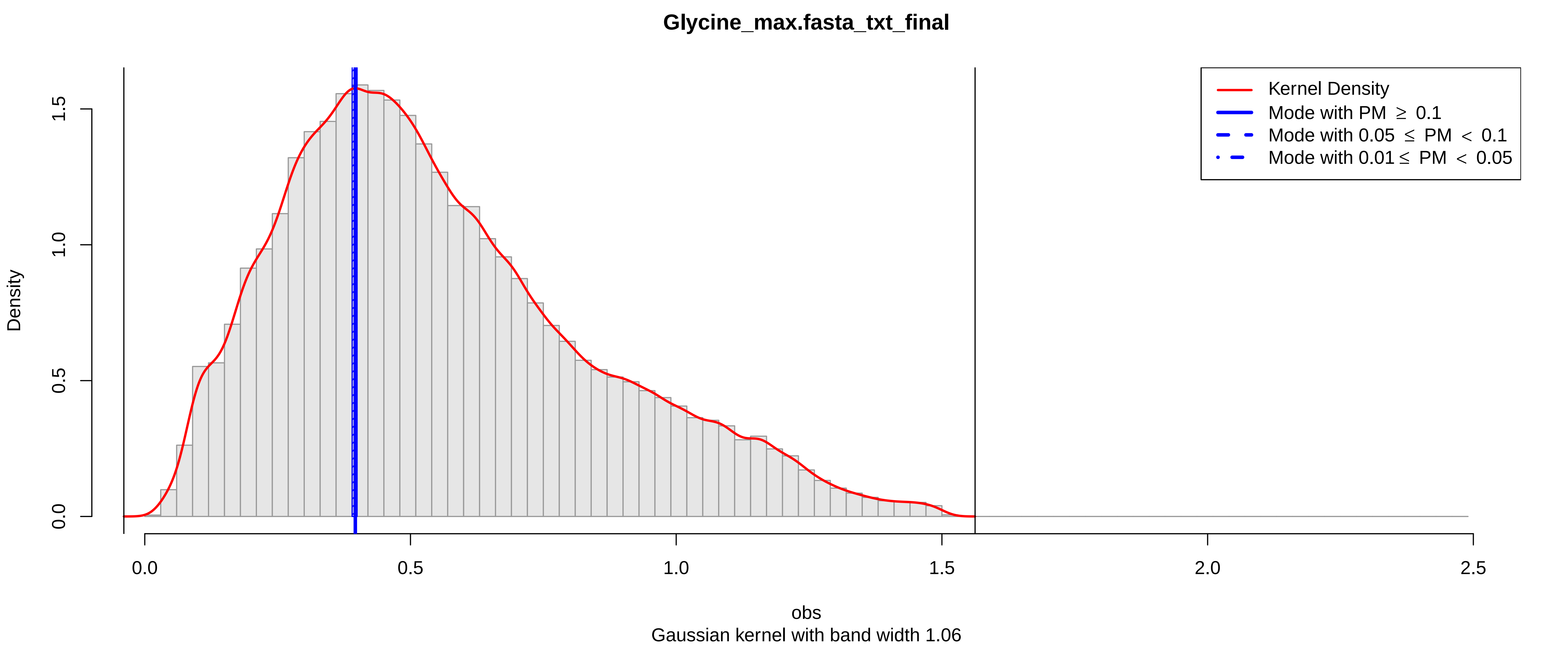
Glycine_max.fasta_txt_final
Kernel Density
Mode with PM ≥ 0.1
Mode with 0.05 ≤ PM < 0.1
Mode with 0.01 ≤ PM < 0.05
Density
0.0
0.5
1.0
1.5
0.0
0.5
1.0
1.5
2.0
2.5
obs
Gaussian kernel with band width 1.06

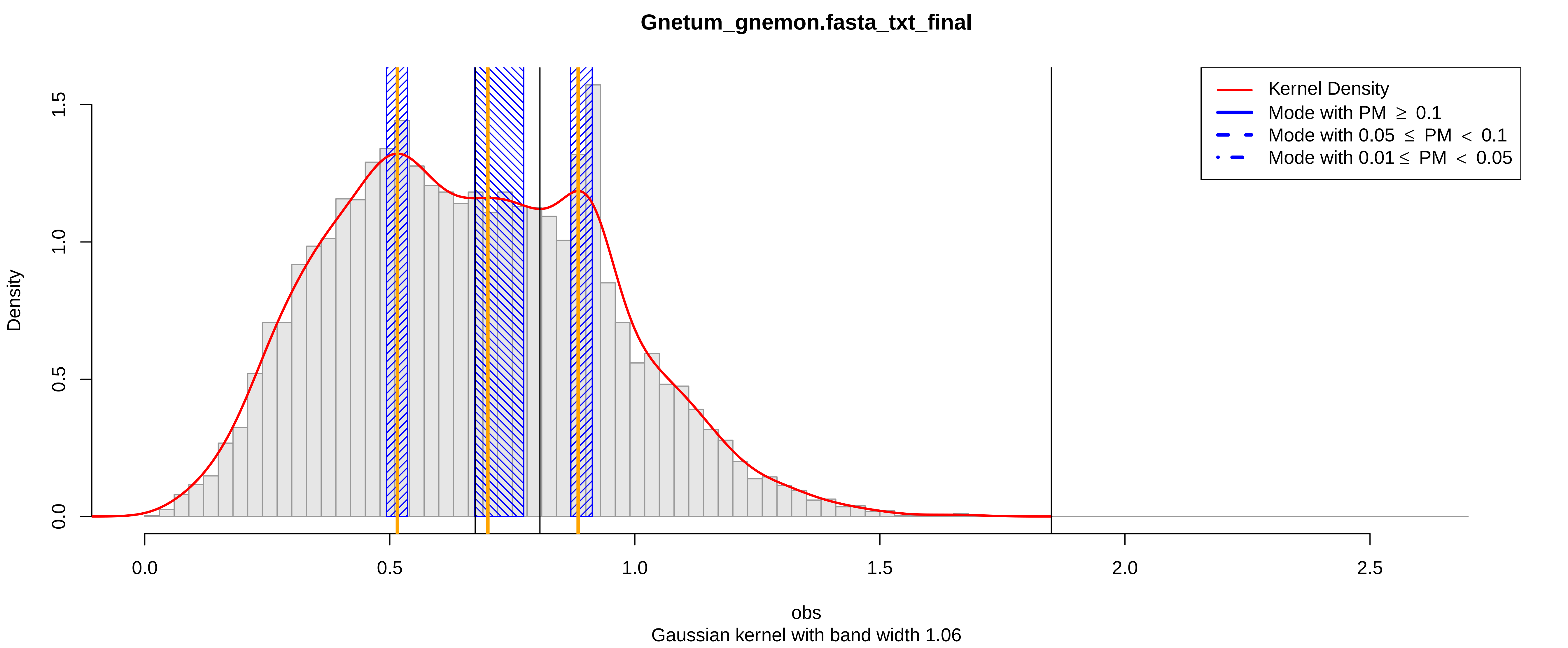

Gnetum_gnemon.fasta_txt_final
Kernel Density
Mode with PM ≥ 0.1
Mode with 0.05 ≤ PM < 0.1
Mode with 0.01 ≤ PM < 0.05
Density
0.0
0.5
1.0
1.5
0.0
0.5
1.0
1.5
2.0
2.5
obs
Gaussian kernel with band width 1.06

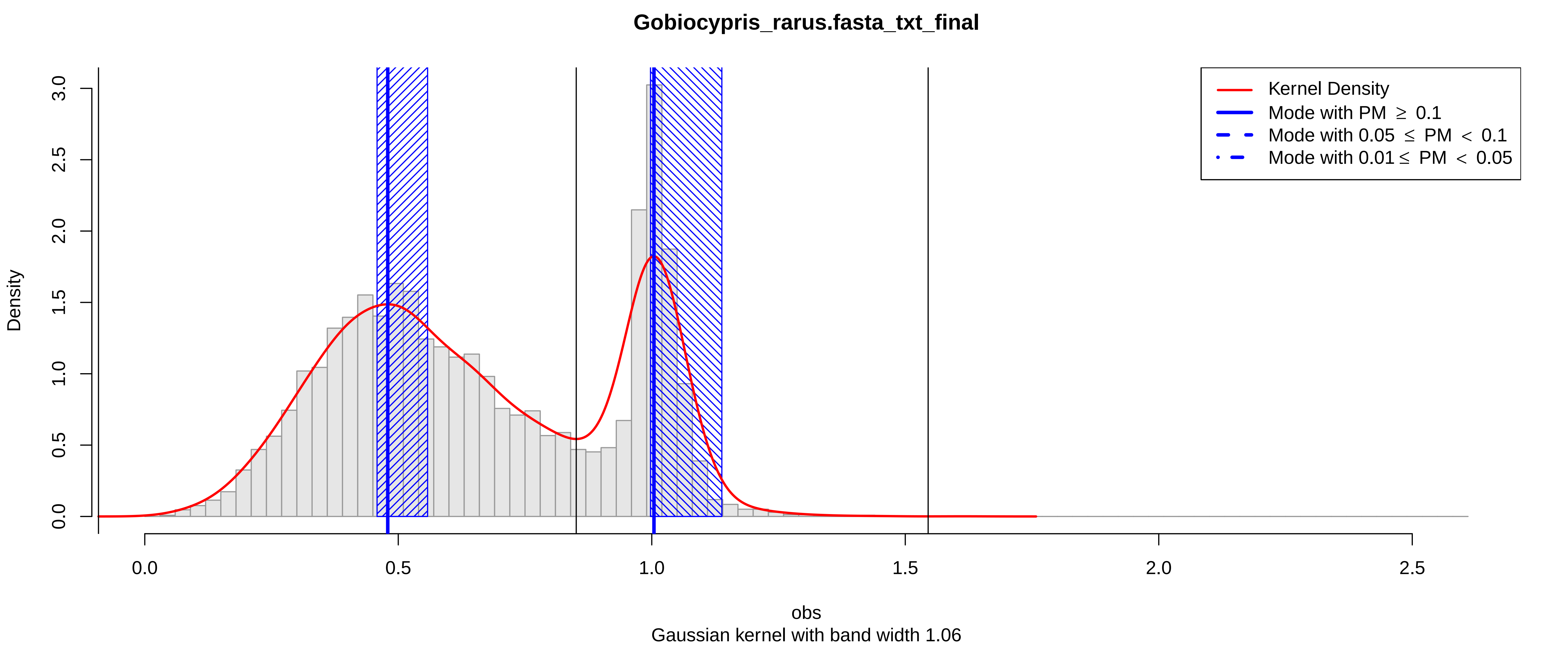

Gobiocypris_rarus.fasta_txt_final
Density
obs
Gaussian kernel with band width 1.06
Kernel Density
Mode with PM ≥ 0.1
Mode with 0.05 ≤ PM < 0.1
Mode with 0.01 ≤ PM < 0.05
0.0
0.5
1.0
1.5
2.0
2.5
3.0

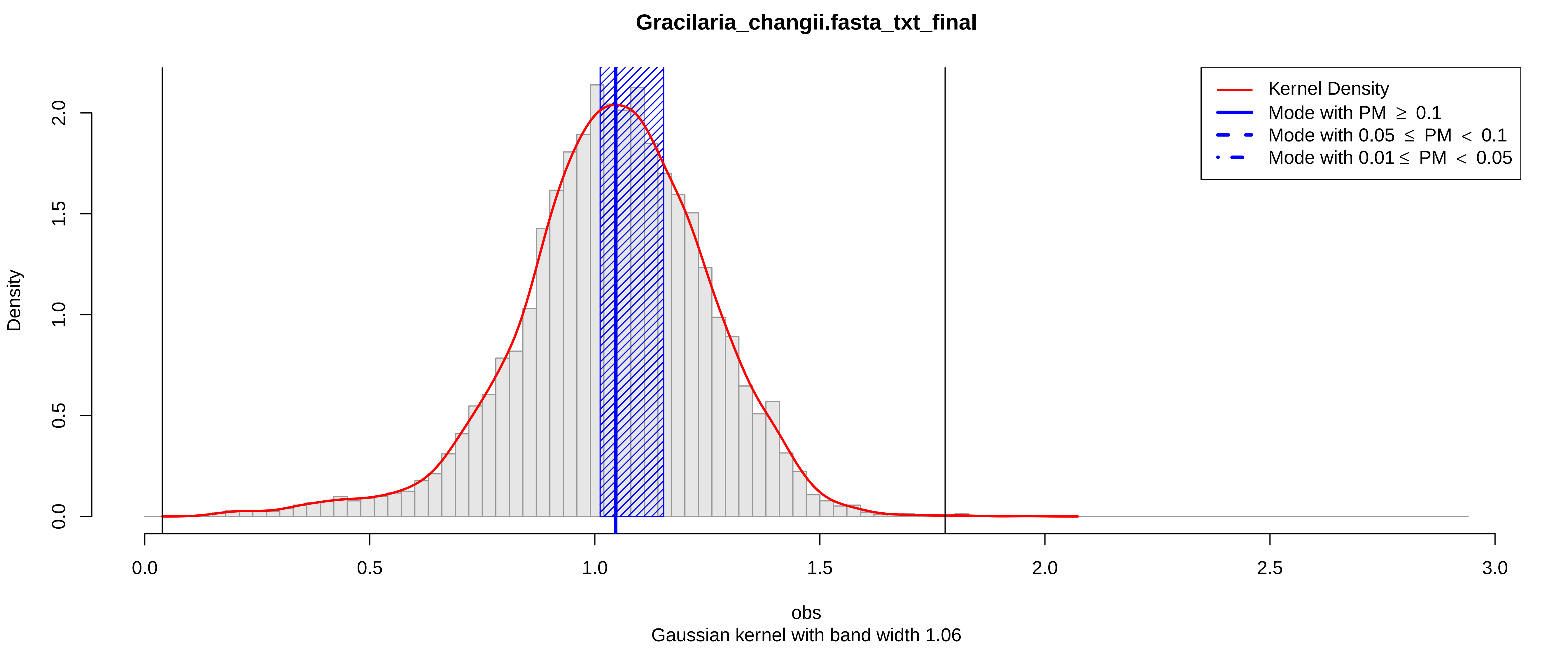

Gracilaria_changii.fasta_txt_final
Kernel Density
Mode with PM ≥ 0.1
Mode with 0.05 ≤ PM < 0.1
Mode with 0.01 ≤ PM < 0.05
Density
0.0
0.5
1.0
1.5
2.0
0.0
0.5
1.0
1.5
2.0
2.5
3.0
obs
Gaussian kernel with band width 1.06

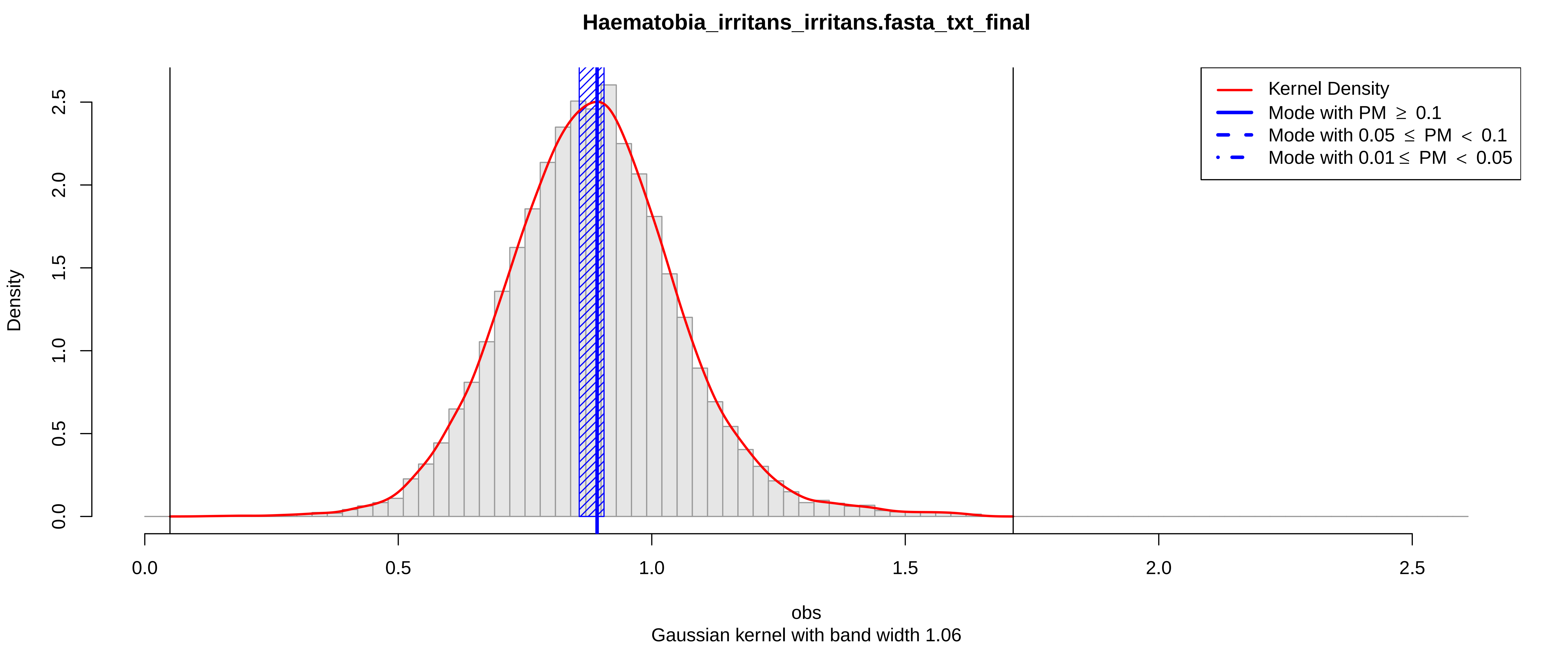
Haematobia_irritans_irritans.fasta_txt_final
Kernel Density
Mode with PM ≥ 0.1
Mode with 0.05 ≤ PM < 0.1
Mode with 0.01 ≤ PM < 0.05
Density
0.0
0.5
1.0
1.5
2.0
2.5
obs
Gaussian kernel with band width 1.06

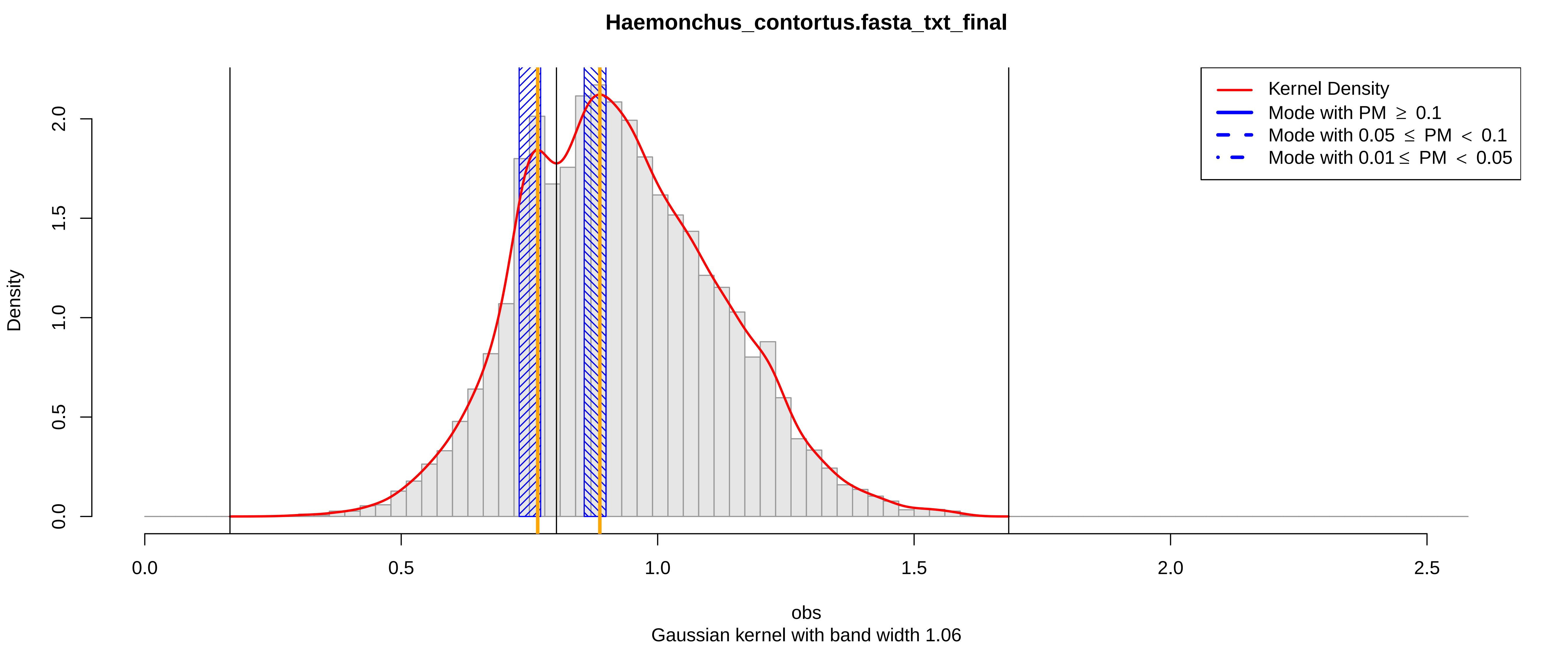
Haemonchus_contortus.fasta_txt_final
Kernel Density
Mode with PM ≥ 0.1
Mode with 0.05 ≤ PM < 0.1
Mode with 0.01 ≤ PM < 0.05
Density
0.0
0.5
1.0
1.5
2.0
0.0
0.5
1.0
1.5
2.0
2.5
obs
Gaussian kernel with band width 1.06

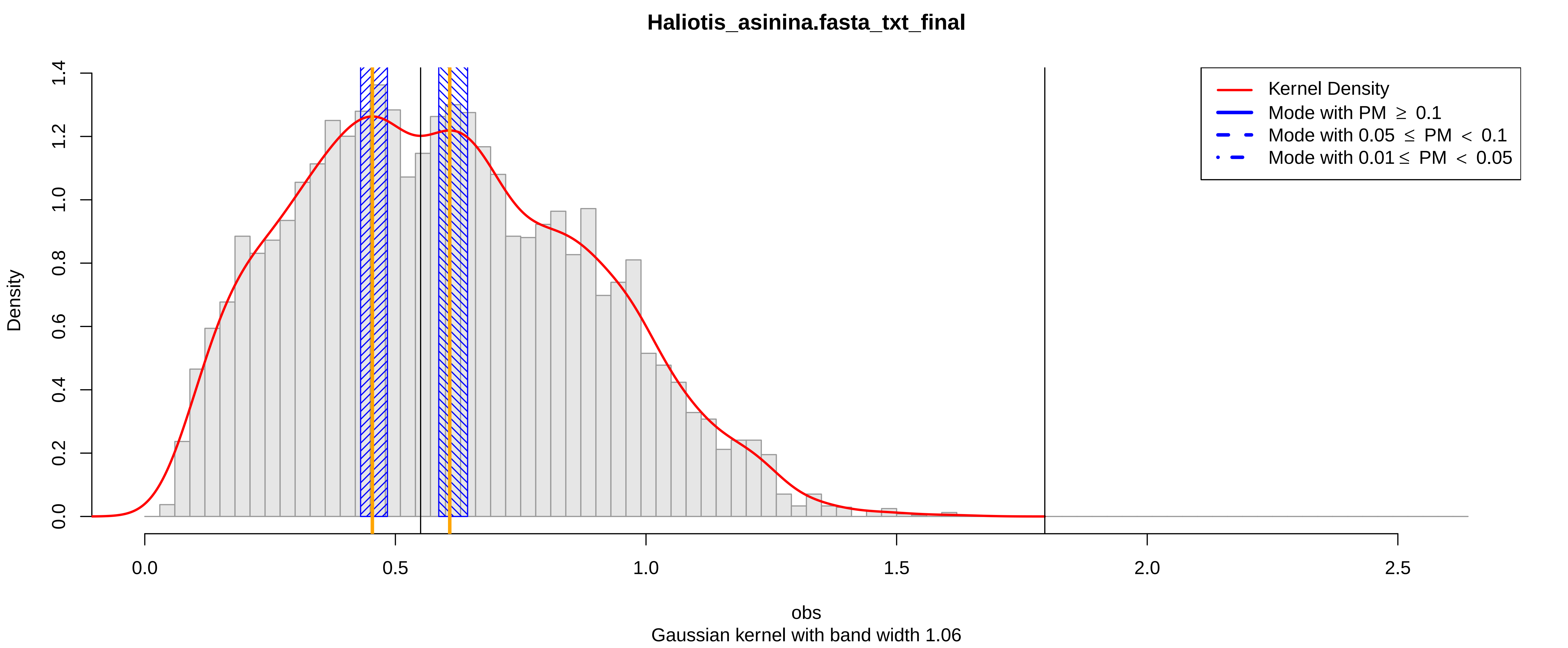

Haliotis_asinina.fasta_txt_final
Kernel Density
Mode with PM ≥ 0.1
Mode with 0.05 ≤ PM < 0.1
Mode with 0.01 ≤ PM < 0.05
Density
0.0
0.2
0.4
0.6
0.8
1.0
1.2
1.4
0.0
0.5
1.0
1.5
2.0
2.5
obs
Gaussian kernel with band width 1.06

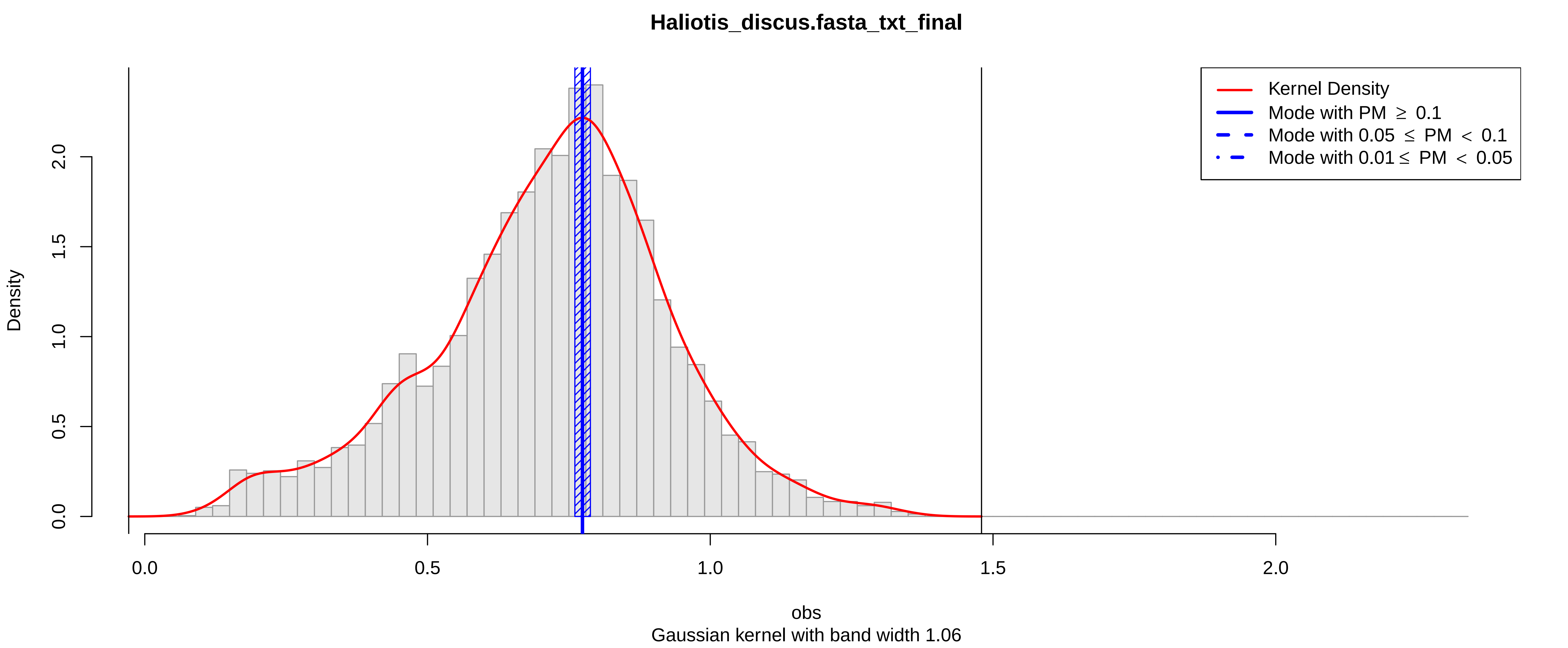
Haliotis_discus.fasta_txt_final
Kernel Density
Mode with PM ≥ 0.1
Mode with 0.05 ≤ PM < 0.1
Mode with 0.01 ≤ PM < 0.05
Density
0.0
0.5
1.0
1.5
2.0
obs
Gaussian kernel with band width 1.06

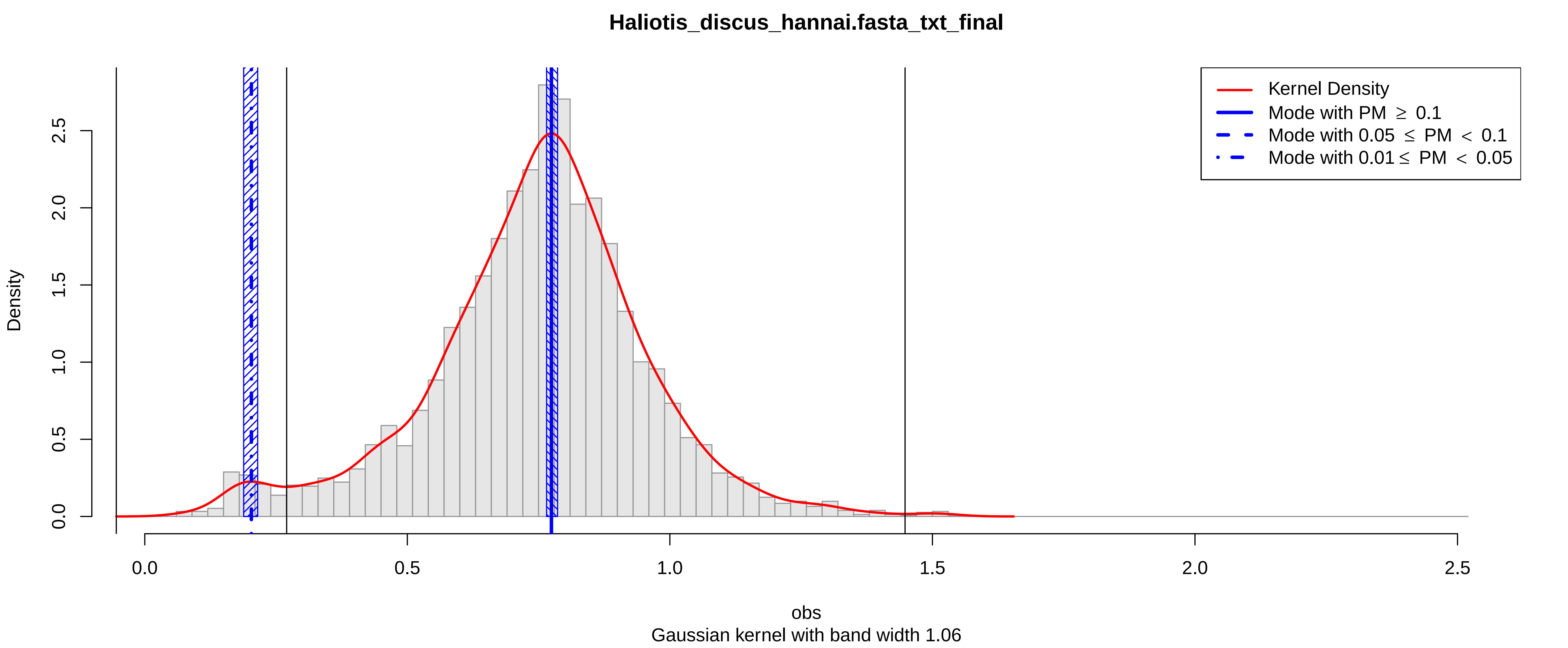

Haliotis_discus_hannai.fasta_txt_final
Kernel Density
Mode with PM ≥ 0.1
Mode with 0.05 ≤ PM < 0.1
Mode with 0.01 ≤ PM < 0.05
Density
0.0
0.5
1.0
1.5
2.0
2.5
0.0
0.5
1.0
1.5
2.0
2.5
obs
Gaussian kernel with band width 1.06

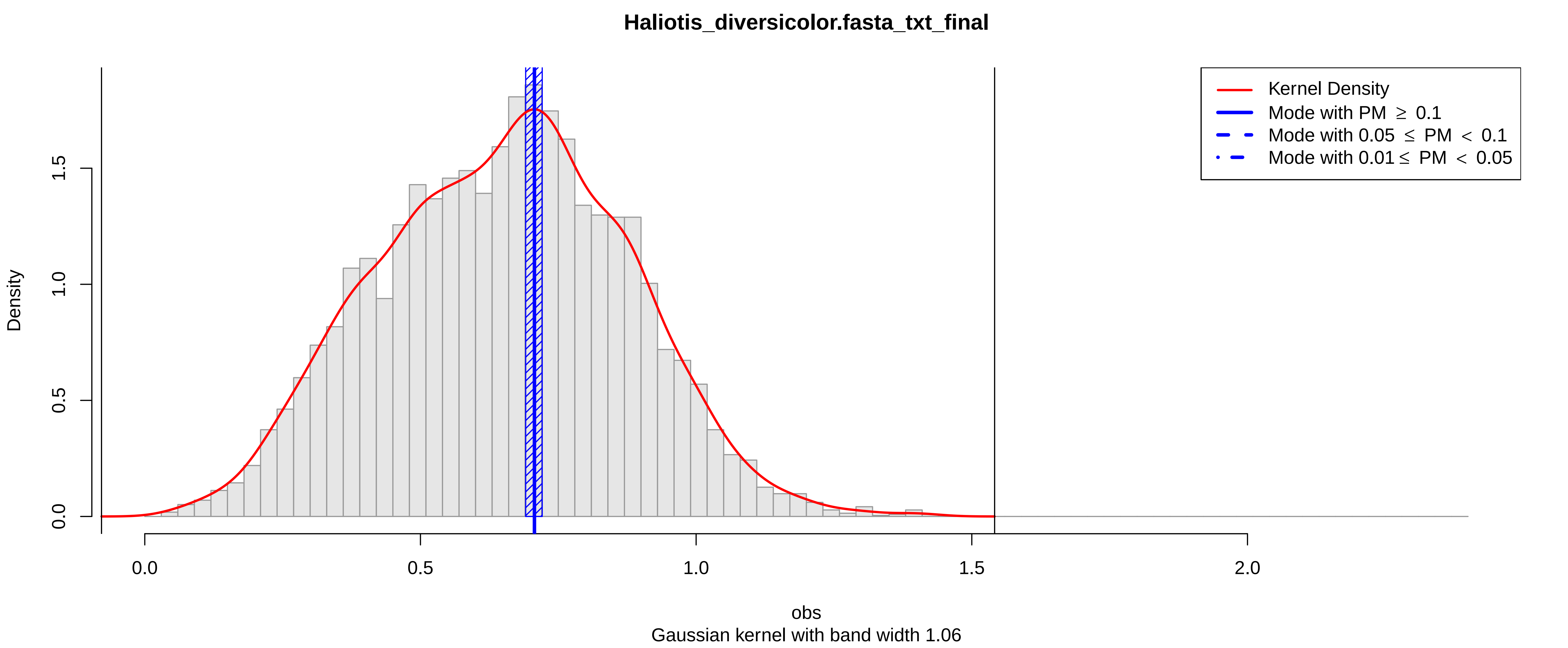

Haliotis_diversicolor.fasta_txt_final
Kernel Density
Mode with PM ≥ 0.1
Mode with 0.05 ≤ PM < 0.1
Mode with 0.01 ≤ PM < 0.05
Density
0.0
0.5
1.0
1.5
2.0
obs
Gaussian kernel with band width 1.06

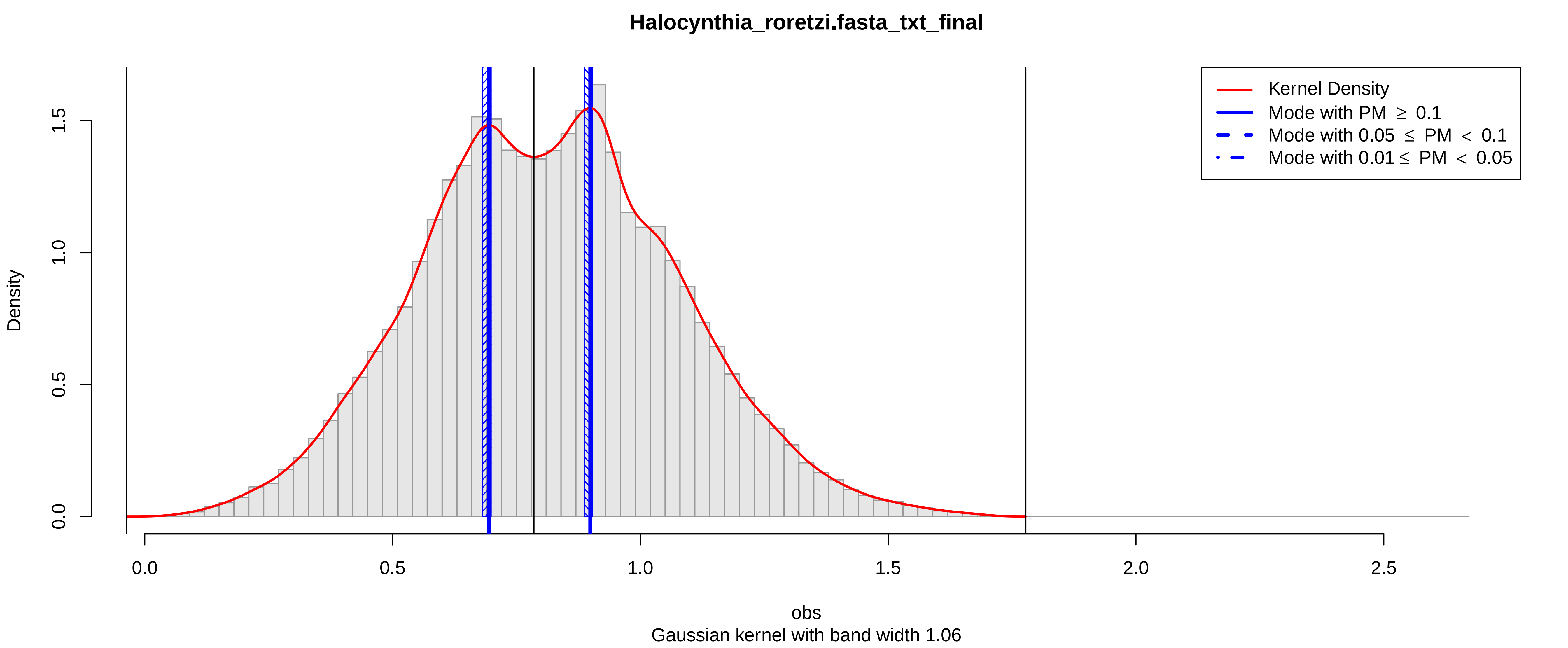

Halocynthia_roretzi.fasta_txt_final
Kernel Density
Mode with PM ≥ 0.1
Mode with 0.05 ≤ PM < 0.1
Mode with 0.01 ≤ PM < 0.05
Density
0.0
0.5
1.0
1.5
0.0
0.5
1.0
1.5
2.0
2.5
obs
Gaussian kernel with band width 1.06

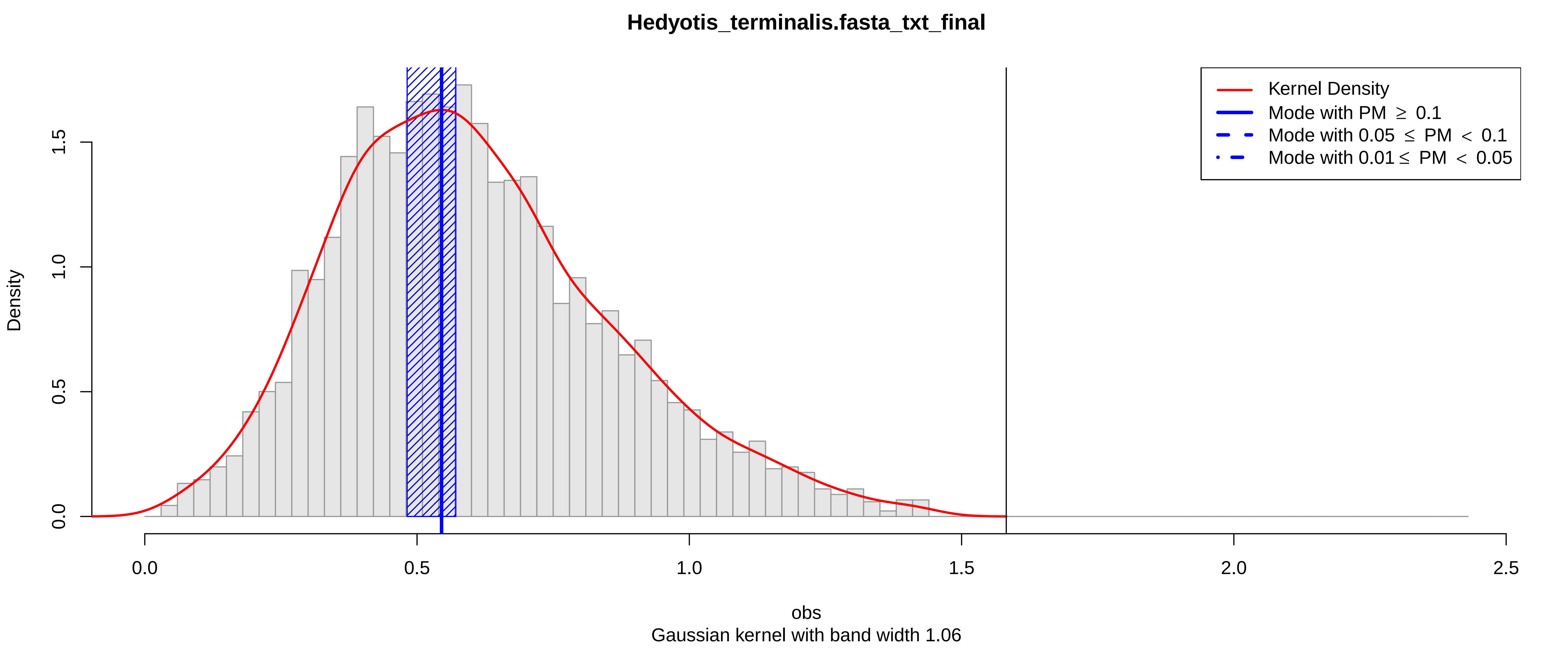

Hedyotis_terminalis.fasta_txt_final
Kernel Density
Mode with PM ≥ 0.1
Mode with 0.05 ≤ PM < 0.1
Mode with 0.01 ≤ PM < 0.05
Density
0.0
0.5
1.0
1.5
0.0
0.5
1.0
1.5
2.0
2.5
obs
Gaussian kernel with band width 1.06

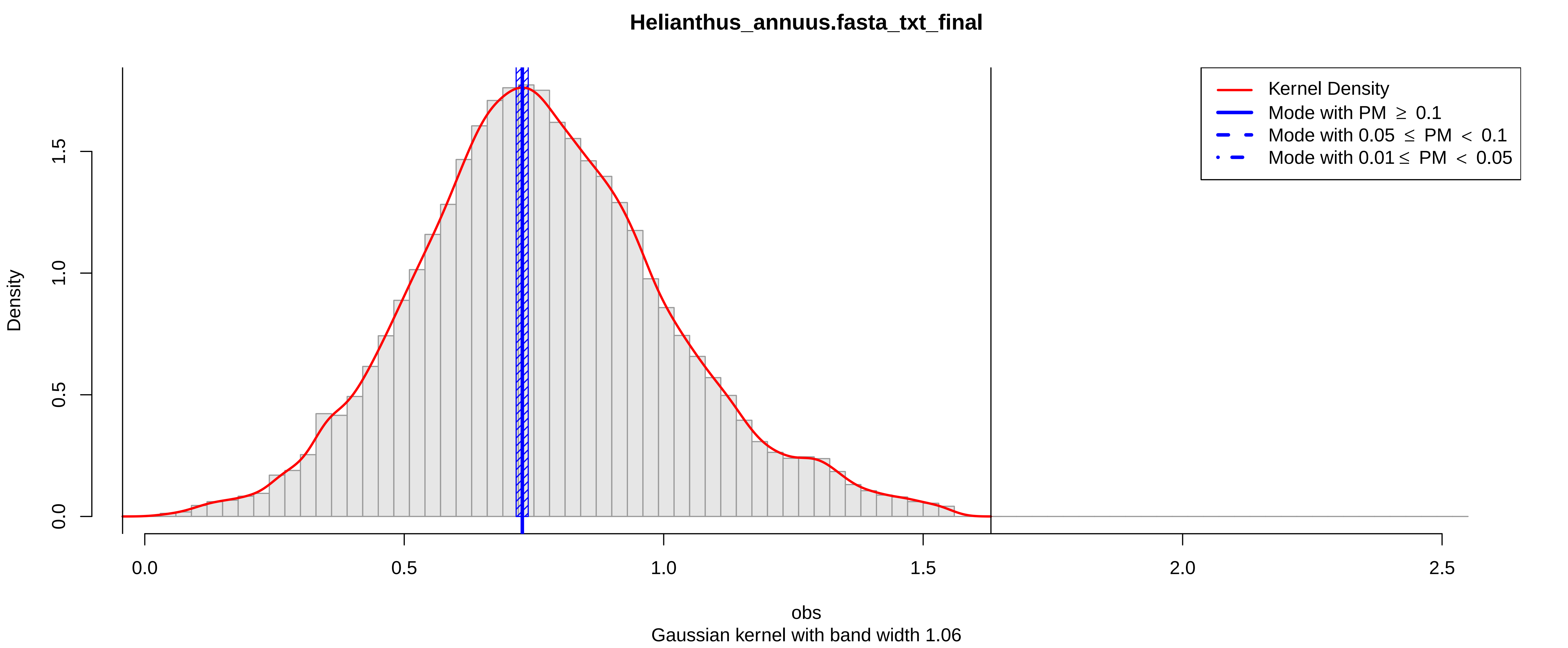

Helianthus_annuus.fasta_txt_final
Kernel Density
Mode with PM ≥ 0.1
Mode with 0.05 ≤ PM < 0.1
Mode with 0.01 ≤ PM < 0.05
Density
0.0
0.5
1.0
1.5
0.0
0.5
1.0
1.5
2.0
2.5
obs
Gaussian kernel with band width 1.06

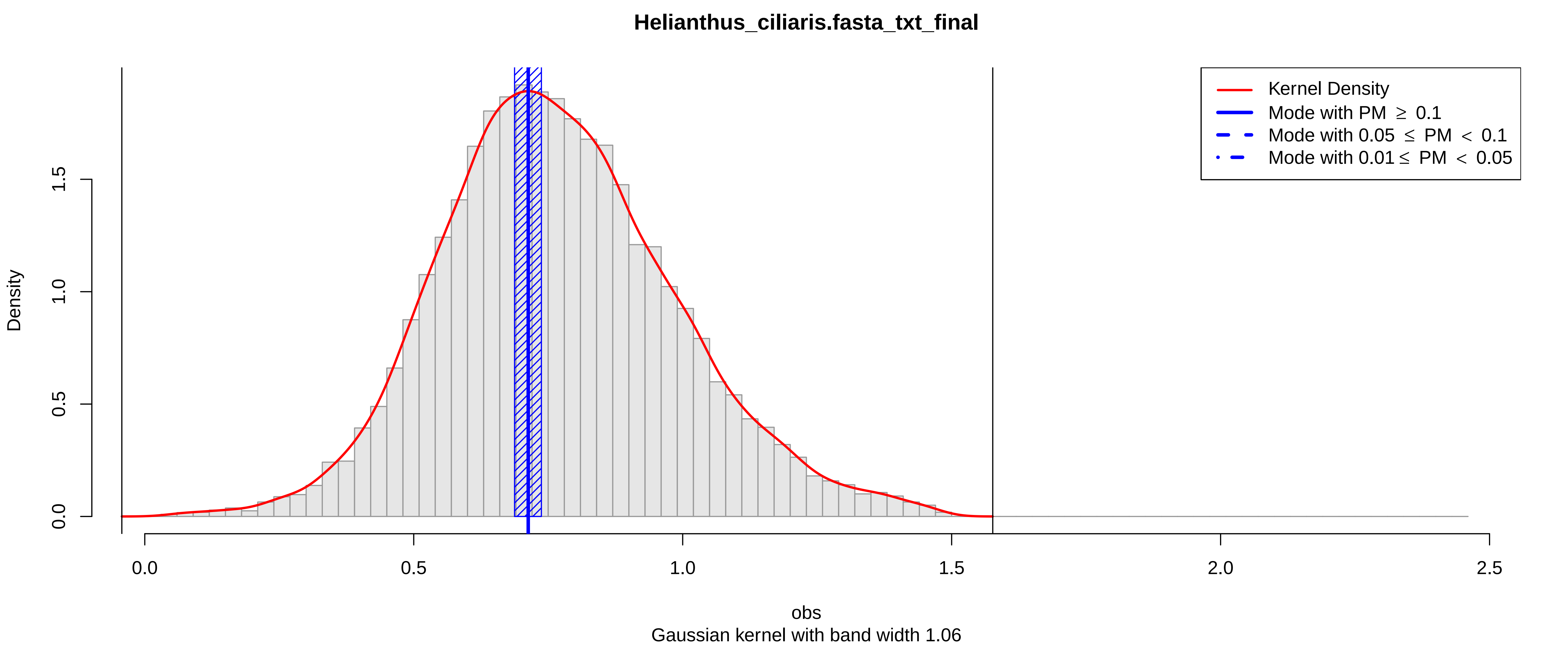
Helianthus_ciliaris.fasta_txt_final
Kernel Density
Mode with PM ≥ 0.1
Mode with 0.05 ≤ PM < 0.1
Mode with 0.01 ≤ PM < 0.05
Density
0.0
0.5
1.0
1.5
0.0
0.5
1.0
1.5
2.0
2.5
obs
Gaussian kernel with band width 1.06

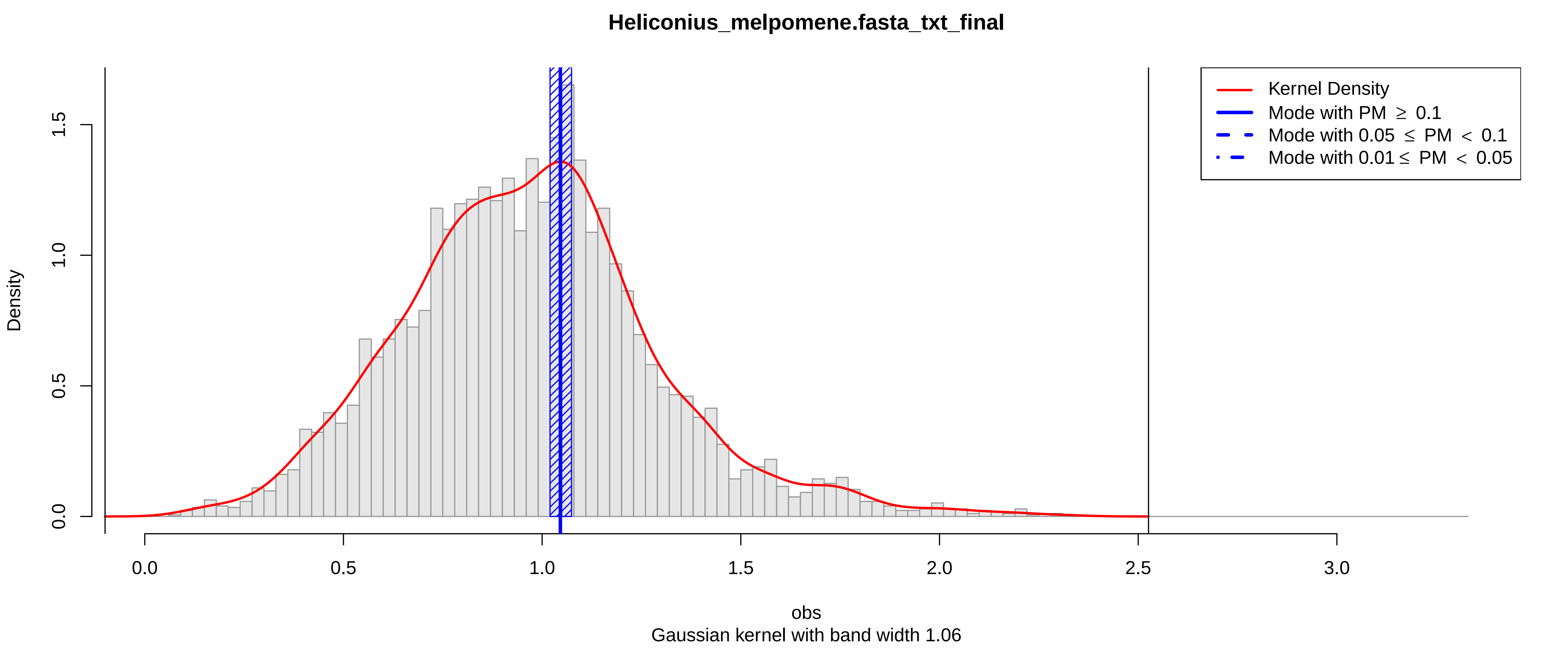

Heliconius_melpomene.fasta_txt_final
Kernel Density
Mode with PM ≥ 0.1
Mode with 0.05 ≤ PM < 0.1
Mode with 0.01 ≤ PM < 0.05
Density
0.0
0.5
1.0
1.5
0.0
0.5
1.0
1.5
2.0
2.5
3.0
obs
Gaussian kernel with band width 1.06

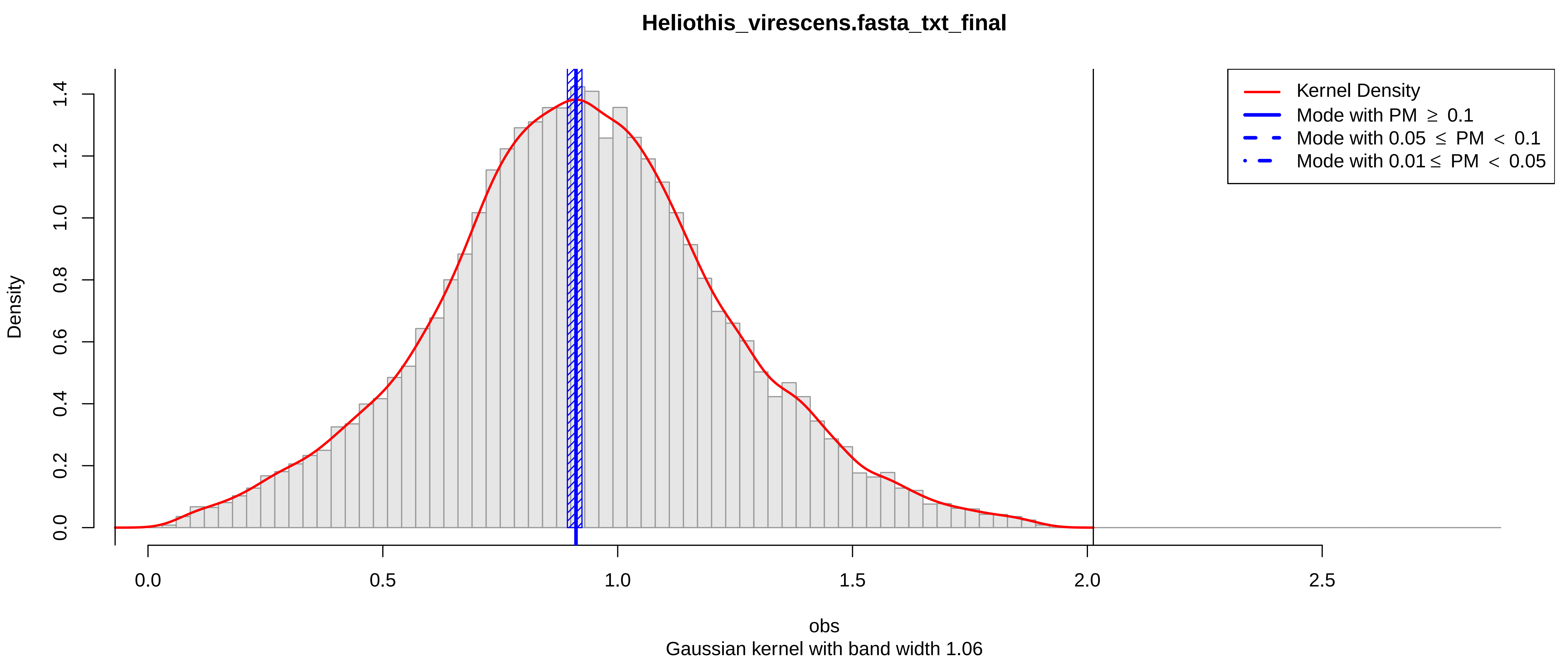

Heliothis_virescens.fasta_txt_final
Kernel Density
Mode with PM ≥ 0.1
Mode with 0.05 ≤ PM < 0.1
Mode with 0.01 ≤ PM < 0.05
Density
0.0
0.2
0.4
0.6
0.8
1.0
1.2
1.4
0.0
0.5
1.0
1.5
2.0
2.5
obs
Gaussian kernel with band width 1.06

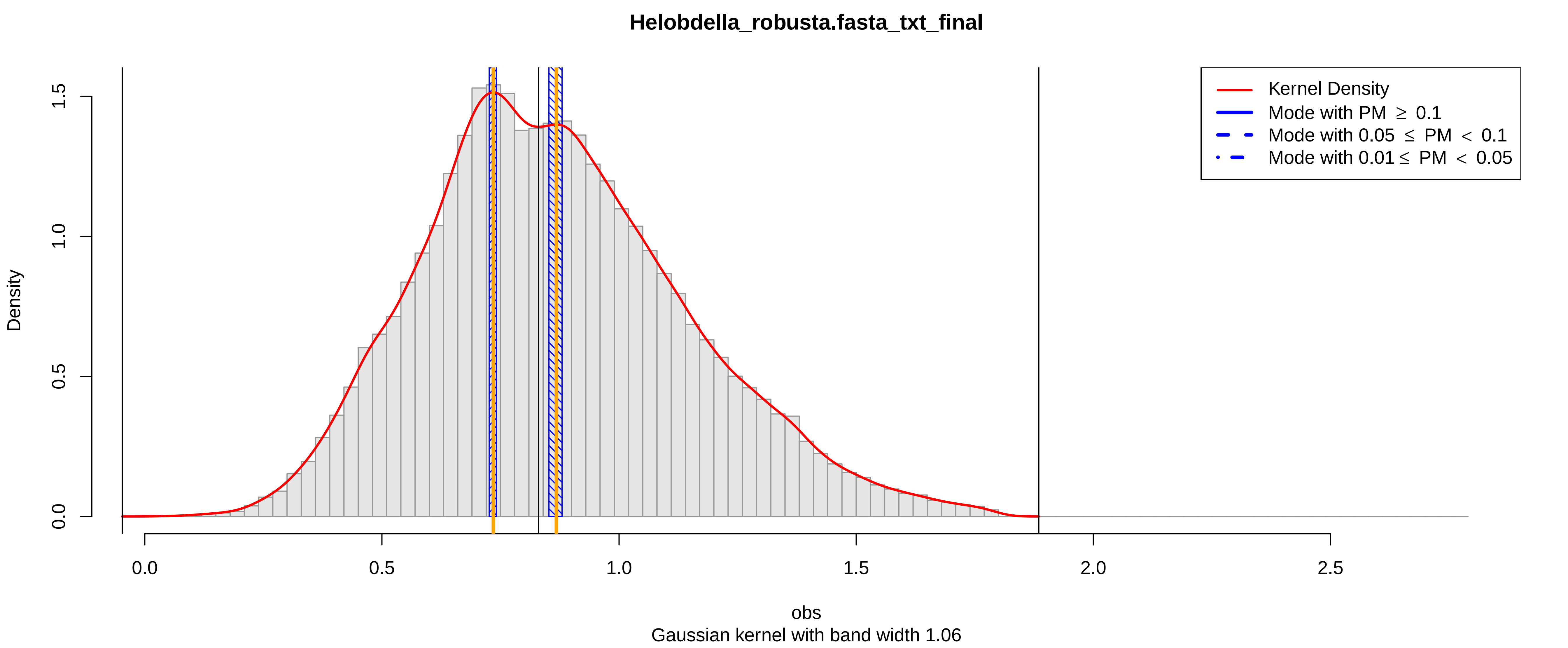

Helobdella_robusta.fasta_txt_final
Kernel Density
Mode with PM ≥ 0.1
Mode with 0.05 ≤ PM < 0.1
Mode with 0.01 ≤ PM < 0.05
Density
0.0
0.5
1.0
1.5
0.0
0.5
1.0
1.5
2.0
2.5
obs
Gaussian kernel with band width 1.06

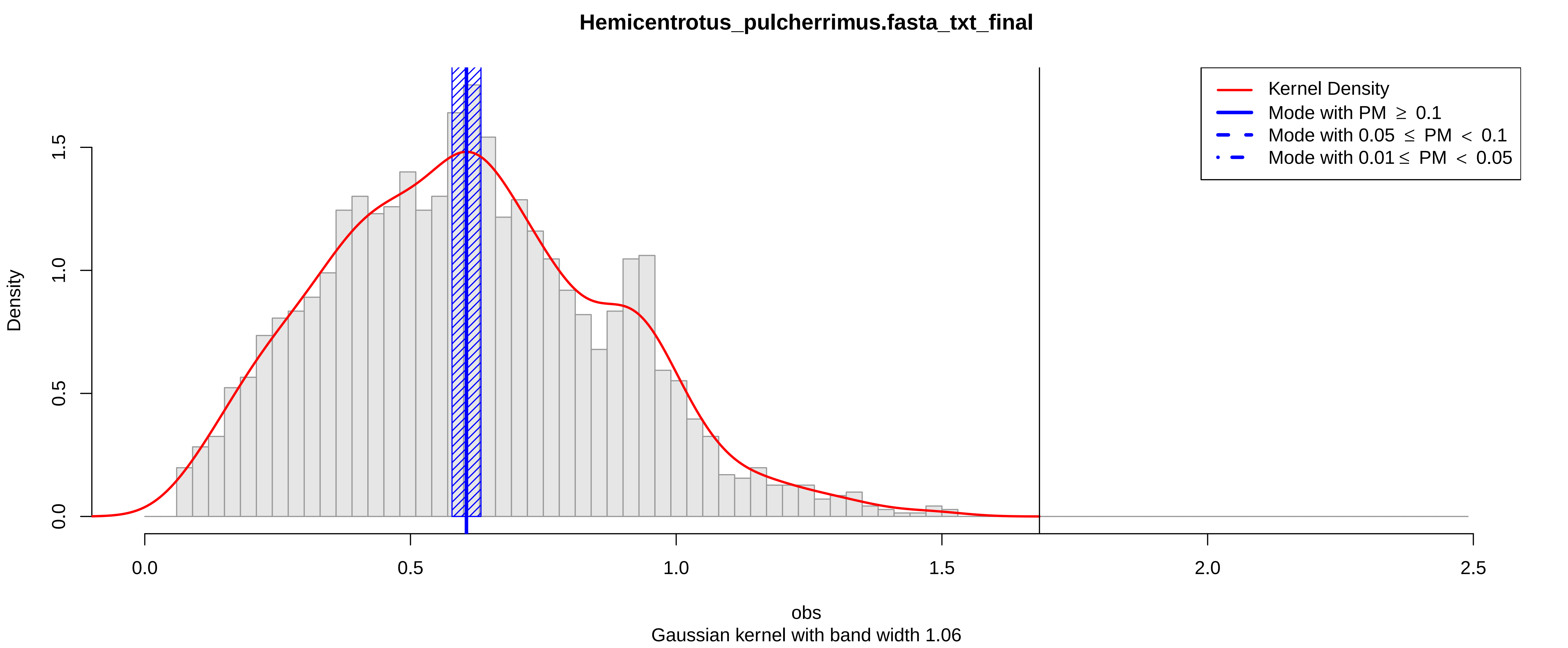

Hemicentrotus_pulcherrimus.fasta_txt_final
Kernel Density
Mode with PM ≥ 0.1
Mode with 0.05 ≤ PM < 0.1
Mode with 0.01 ≤ PM < 0.05
Density
0.0
0.5
1.0
1.5
0.0
0.5
1.0
1.5
2.0
2.5
obs
Gaussian kernel with band width 1.06

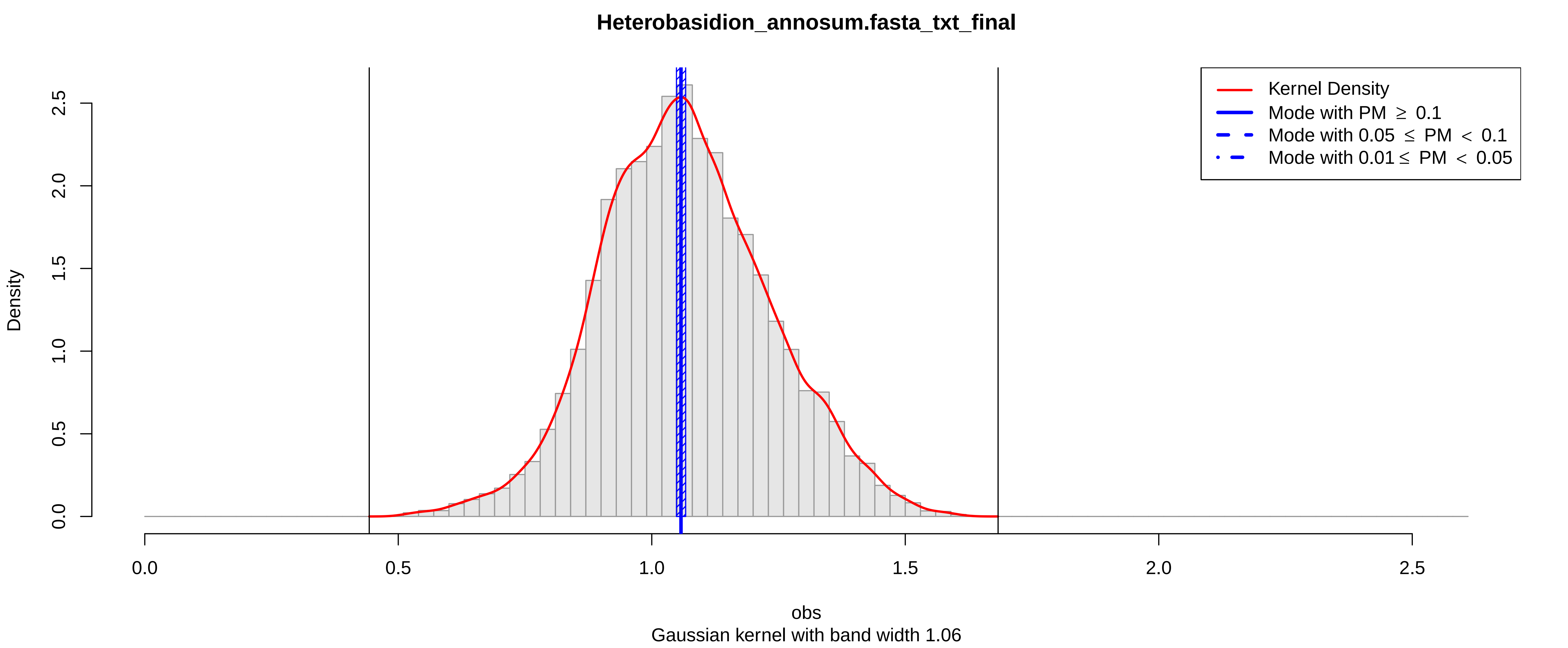

Heterobasidion_annosum.fasta_txt_final
Kernel Density
Mode with PM ≥ 0.1
Mode with 0.05 ≤ PM < 0.1
Mode with 0.01 ≤ PM < 0.05
Density
0.0
0.5
1.0
1.5
2.0
2.5
obs
Gaussian kernel with band width 1.06

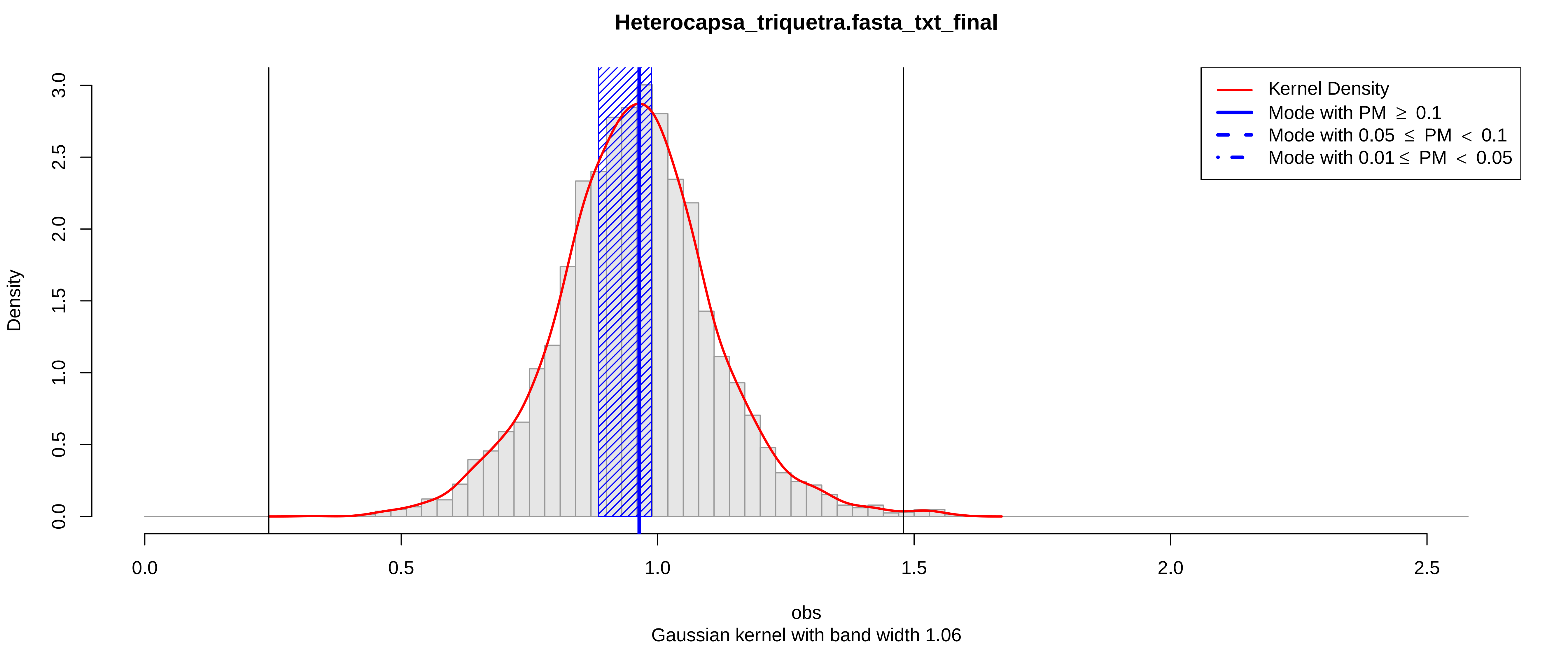

Heterocapsa_triquetra.fasta_txt_final
Density
0.0
0.5
1.0
1.5
2.0
2.5
3.0
0.0
0.5
1.0
1.5
2.0
2.5
Kernel Density
Mode with PM ≥ 0.1
Mode with 0.05 ≤ PM < 0.1
Mode with 0.01 ≤ PM < 0.05
obs
Gaussian kernel with band width 1.06

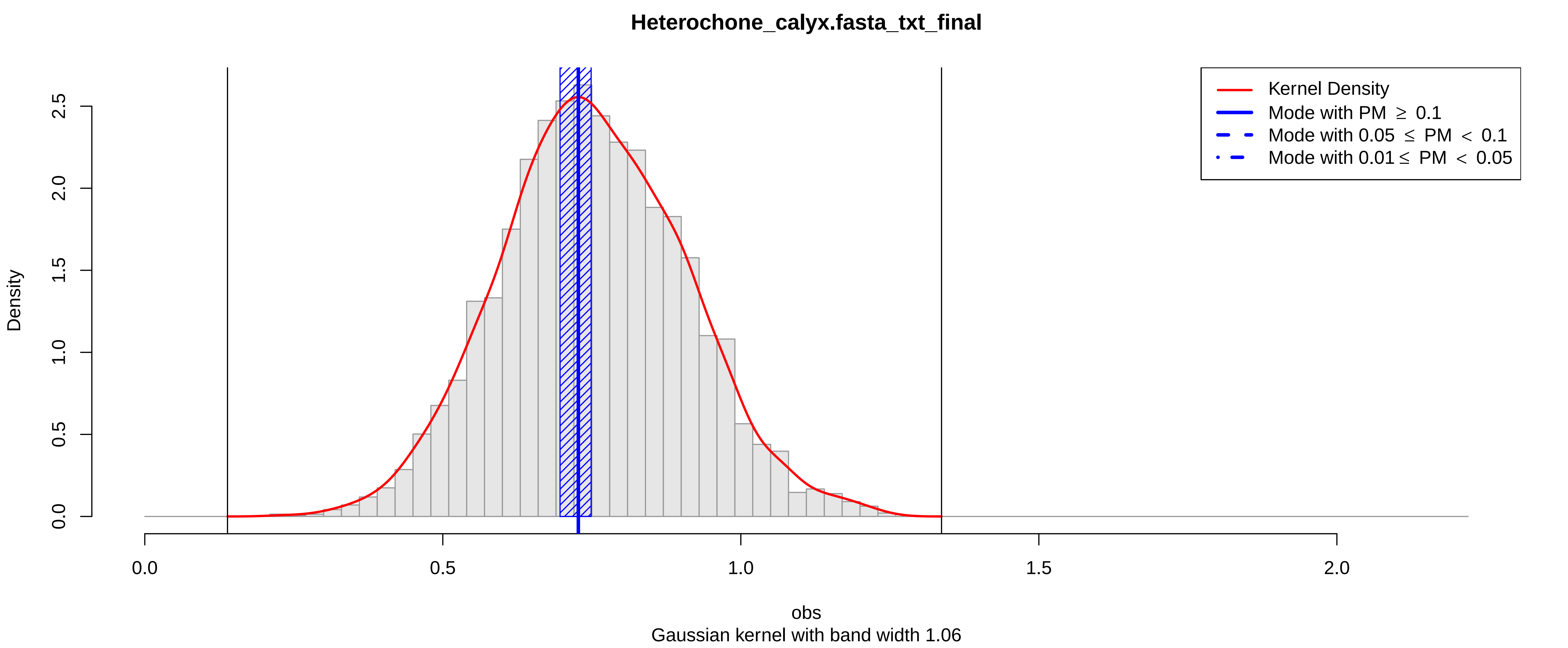

Heterochone_calyx.fasta_txt_final
Kernel Density
Mode with PM ≥ 0.1
Mode with 0.05 ≤ PM < 0.1
Mode with 0.01 ≤ PM < 0.05
Density
0.0
0.5
1.0
1.5
2.0
2.5
0.0
0.5
1.0
1.5
2.0
obs
Gaussian kernel with band width 1.06

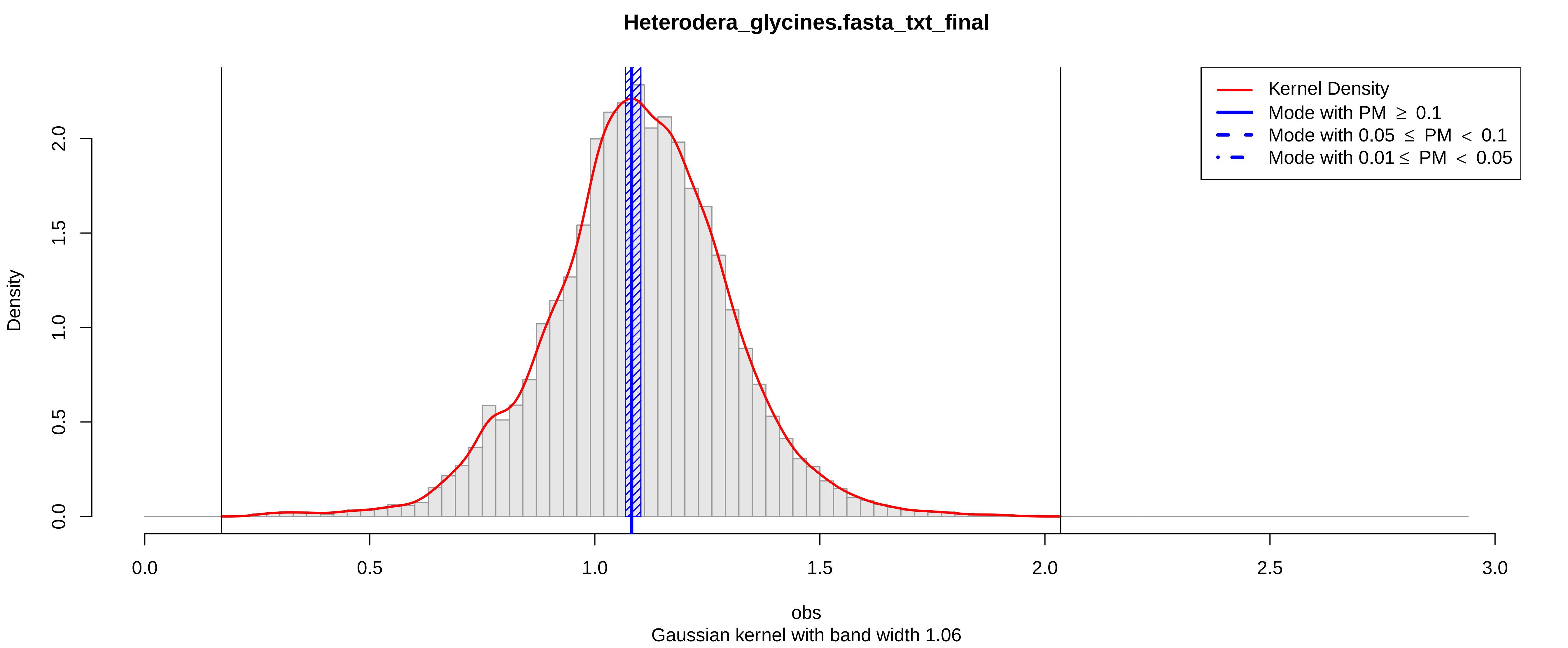

Heterodera_glycines.fasta_txt_final
Kernel Density
Mode with PM ≥ 0.1
Mode with 0.05 ≤ PM < 0.1
Mode with 0.01 ≤ PM < 0.05
Density
0.0
0.5
1.0
1.5
2.0
0.0
0.5
1.0
1.5
2.0
2.5
3.0
obs
Gaussian kernel with band width 1.06

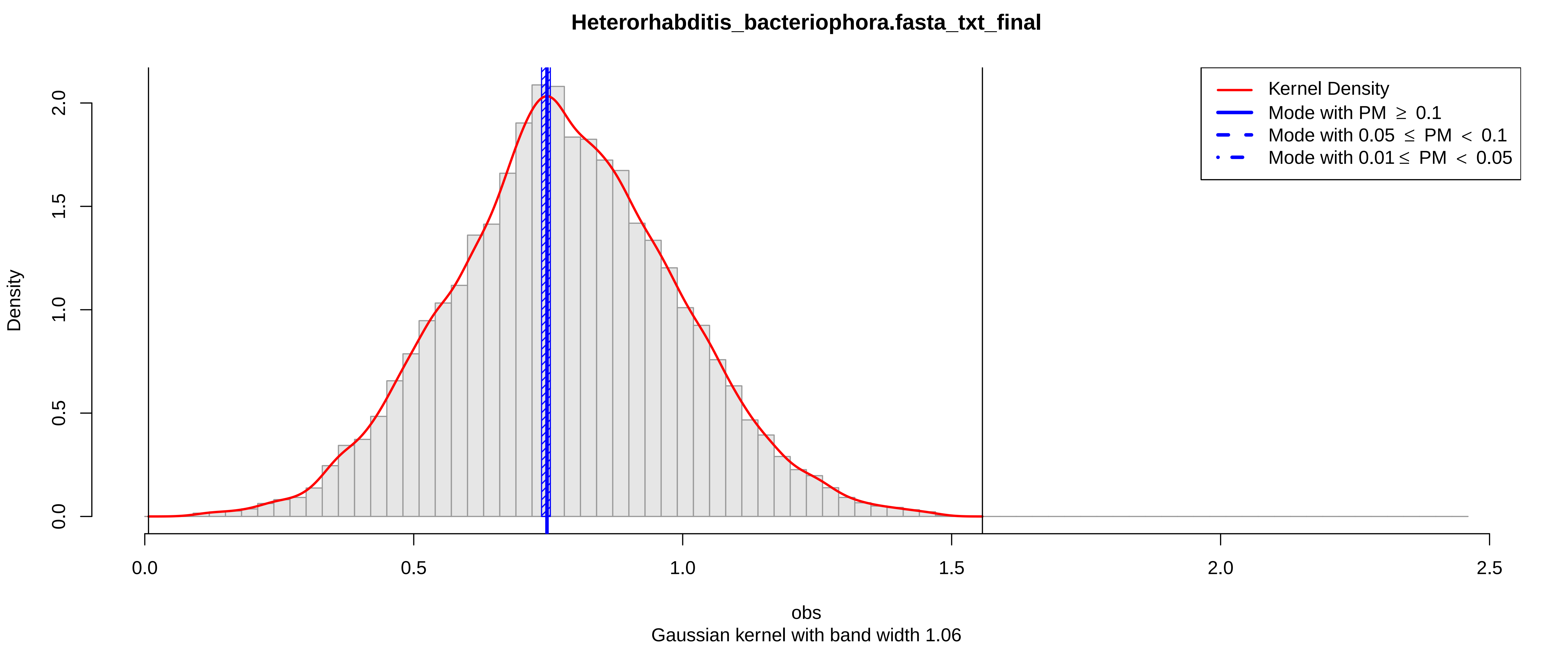

Heterorhabditis_bacteriophora.fasta_txt_final
Kernel Density
Mode with PM ≥ 0.1
Mode with 0.05 ≤ PM < 0.1
Mode with 0.01 ≤ PM < 0.05
Density
0.0
0.5
1.0
1.5
2.0
2.5
obs
Gaussian kernel with band width 1.06

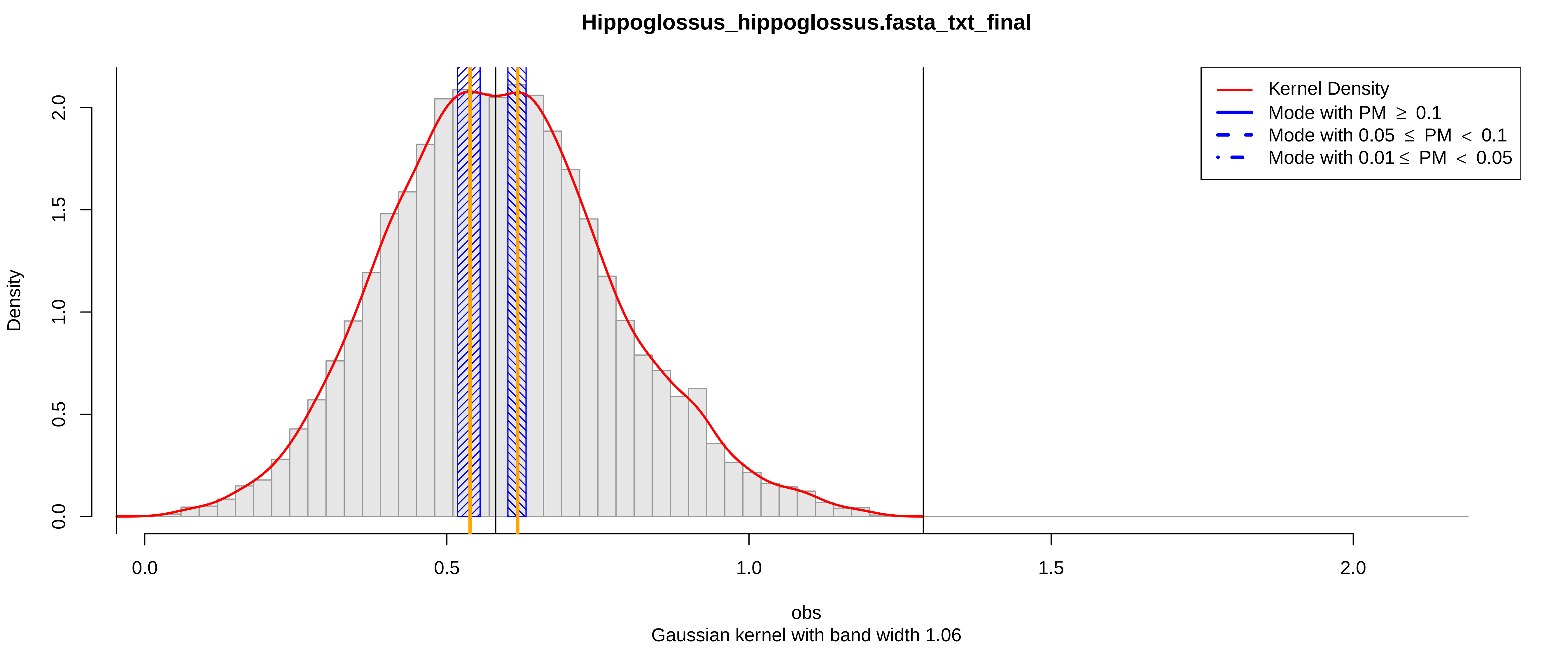

Hippoglossus_hippoglossus.fasta_txt_final
Kernel Density
Mode with PM ≥ 0.1
Mode with 0.05 ≤ PM < 0.1
Mode with 0.01 ≤ PM < 0.05
Density
0.0
0.5
1.0
1.5
2.0
0.0
0.5
1.0
1.5
2.0
obs
Gaussian kernel with band width 1.06

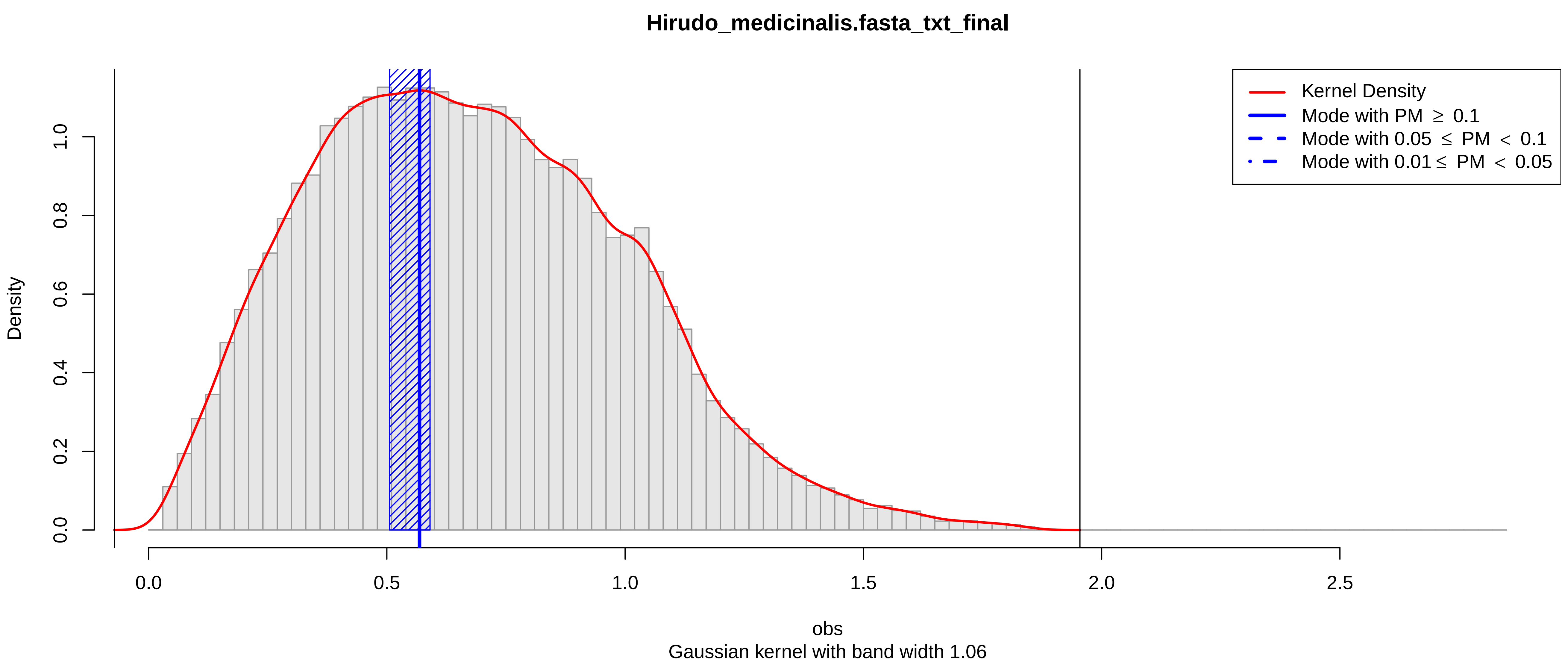

Hirudo_medicinalis.fasta_txt_final
Kernel Density
Mode with PM ≥ 0.1
Mode with 0.05 ≤ PM < 0.1
Mode with 0.01 ≤ PM < 0.05
Density
0.0
0.2
0.4
0.6
0.8
1.0
0.0
0.5
1.0
1.5
2.0
2.5
obs
Gaussian kernel with band width 1.06

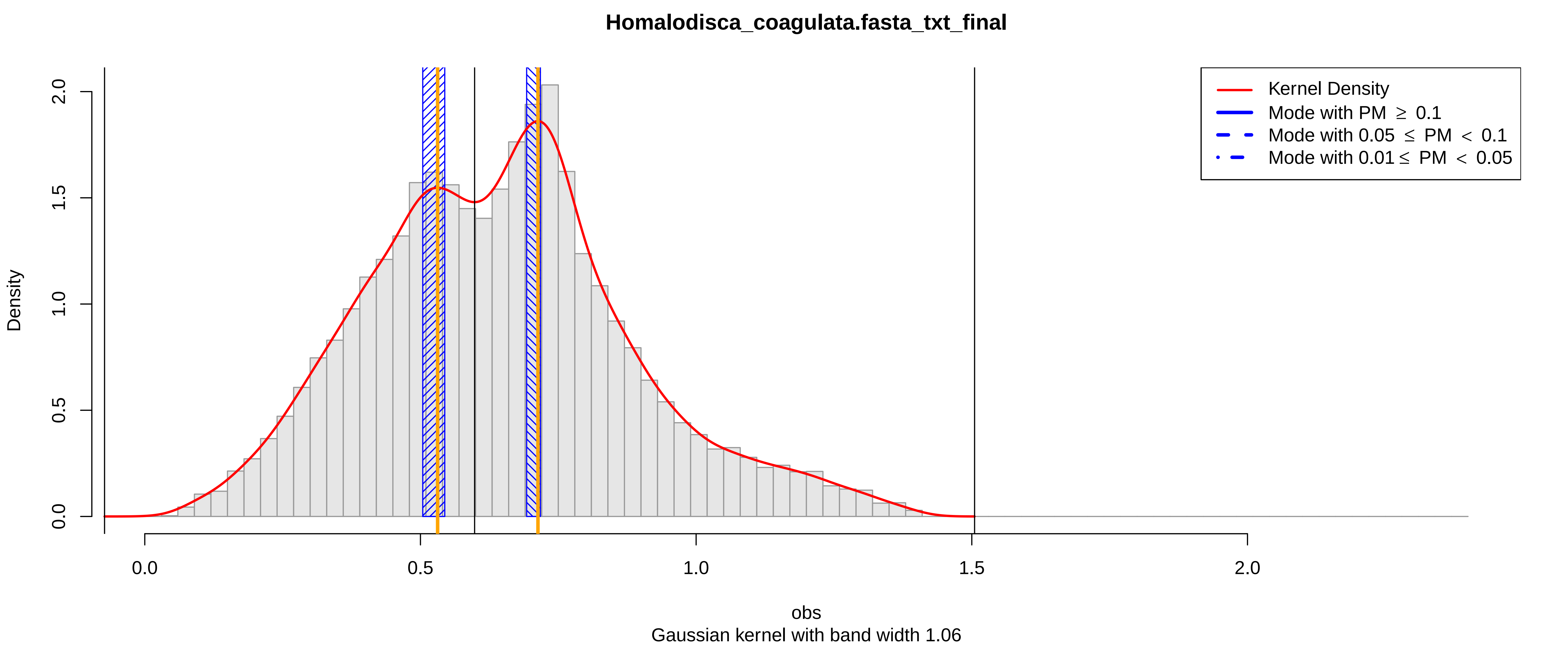
Homalodisca_coagulata.fasta_txt_final
Kernel Density
Mode with PM ≥ 0.1
Mode with 0.05 ≤ PM < 0.1
Mode with 0.01 ≤ PM < 0.05
Density
0.0
0.5
1.0
1.5
2.0
obs
Gaussian kernel with band width 1.06

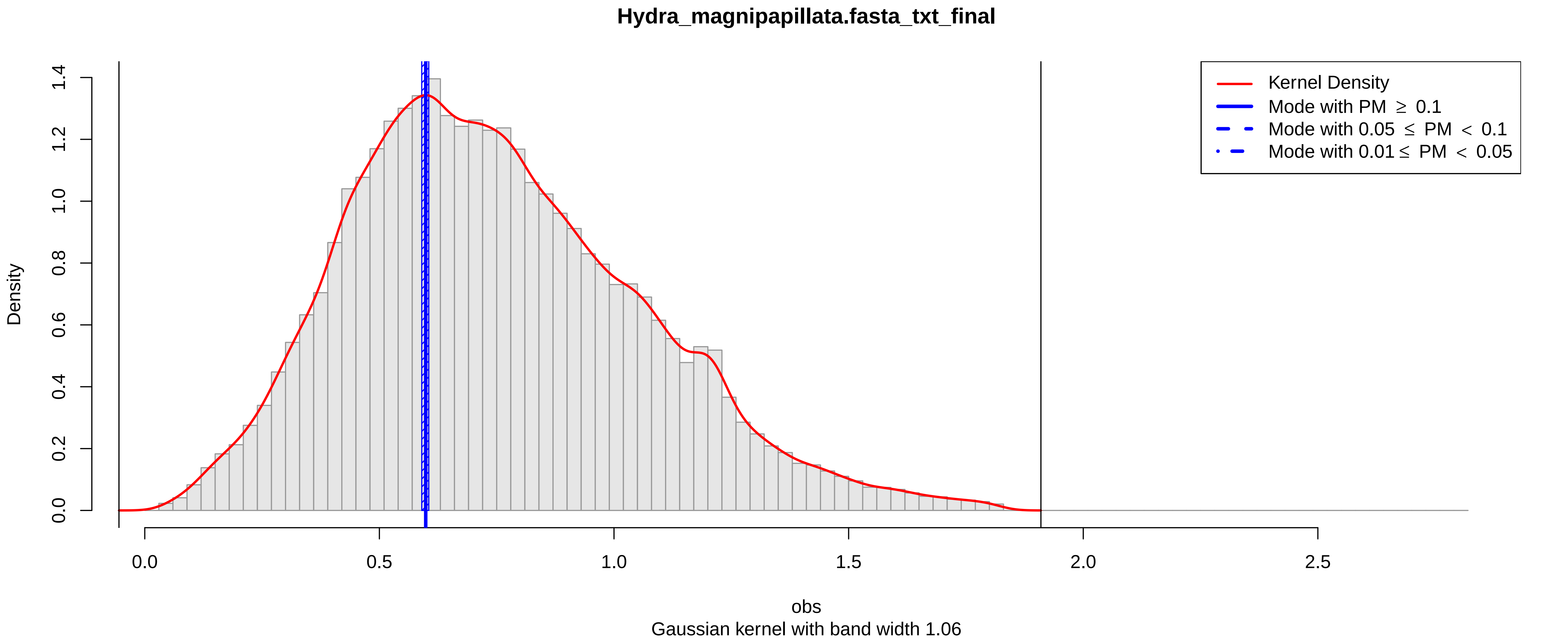
Hydra_magnipapillata.fasta_txt_final
Kernel Density
Mode with PM ≥ 0.1
Mode with 0.05 ≤ PM < 0.1
Mode with 0.01 ≤ PM < 0.05
Density
0.0
0.2
0.4
0.6
0.8
1.0
1.2
1.4
0.0
0.5
1.0
1.5
2.0
2.5
obs
Gaussian kernel with band width 1.06

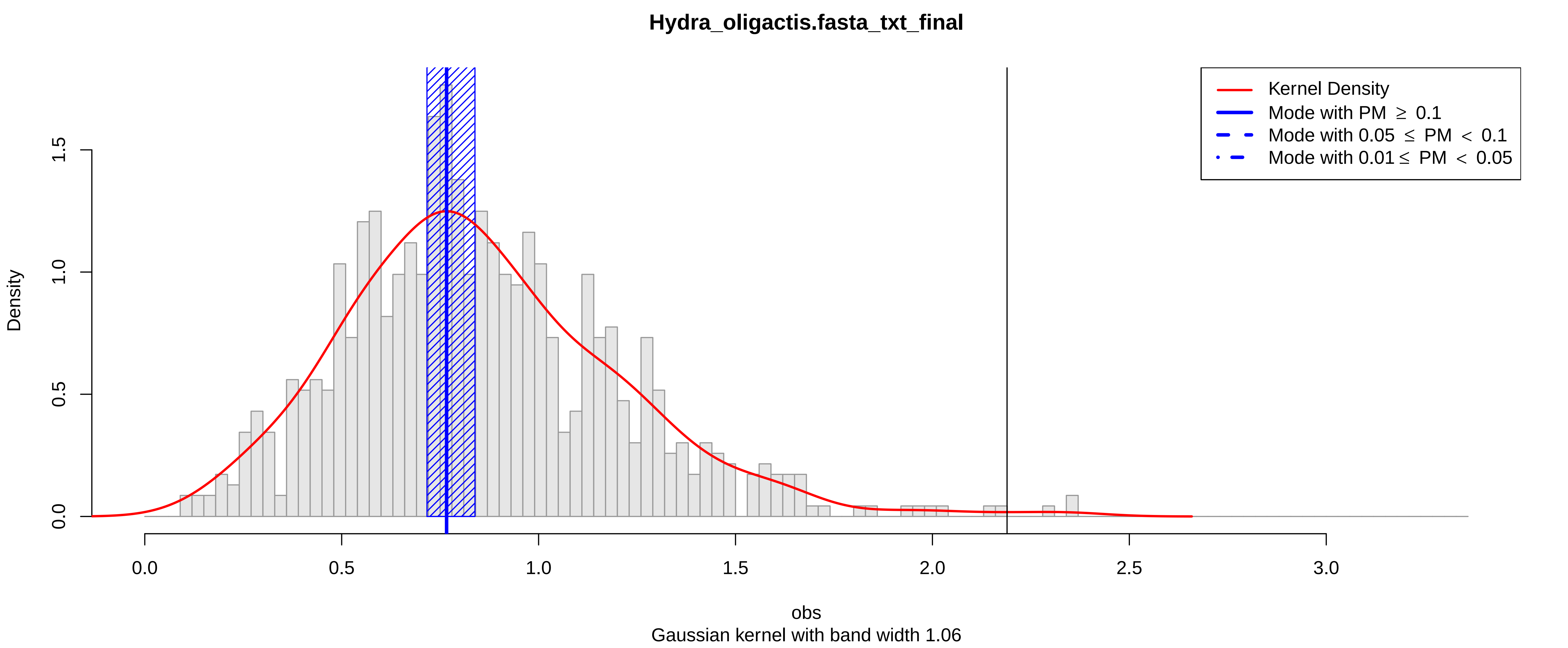

Hydra_oligactis.fasta_txt_final
Kernel Density
Mode with PM ≥ 0.1
Mode with 0.05 ≤ PM < 0.1
Mode with 0.01 ≤ PM < 0.05
Density
0.0
0.5
1.0
1.5
0.0
0.5
1.0
1.5
2.0
2.5
3.0
obs
Gaussian kernel with band width 1.06

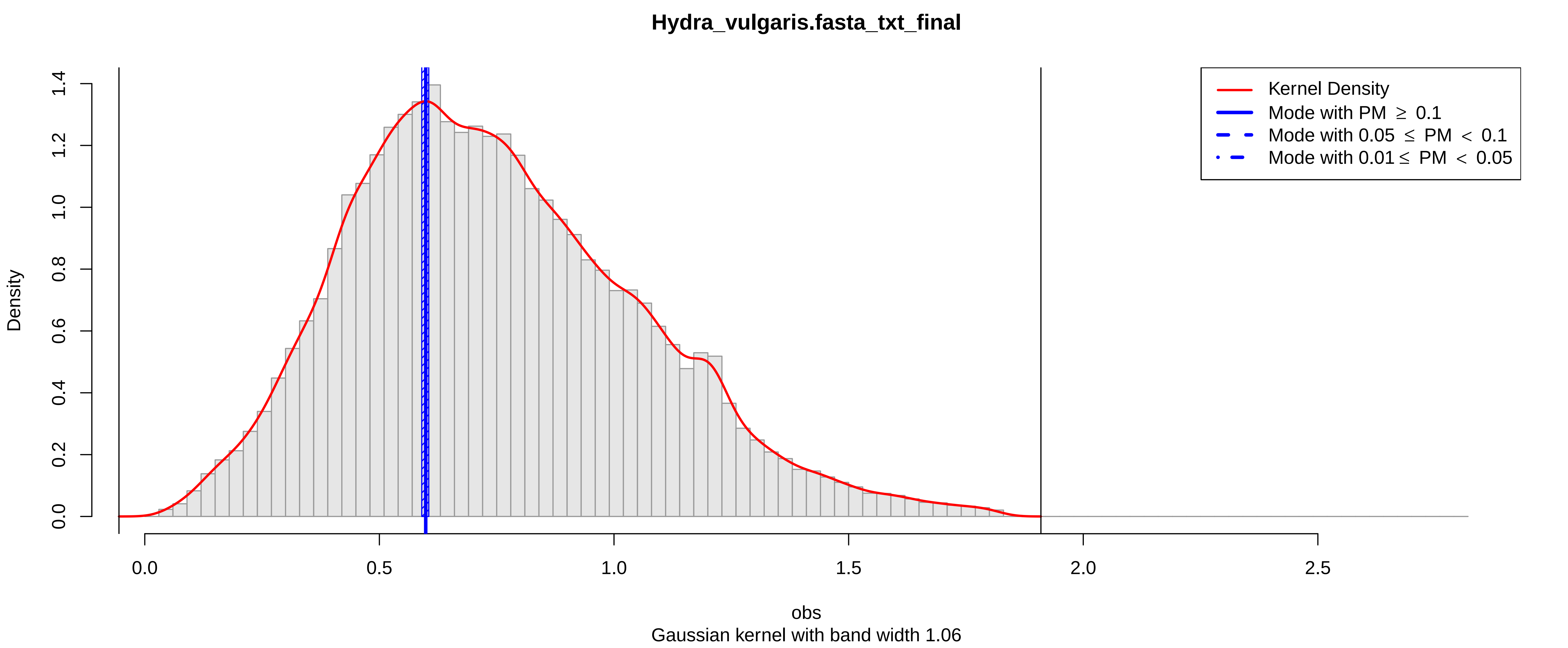

Hydra_vulgaris.fasta_txt_final
Kernel Density
Mode with PM ≥ 0.1
Mode with 0.05 ≤ PM < 0.1
Mode with 0.01 ≤ PM < 0.05
Density
0.0
0.2
0.4
0.6
0.8
1.0
1.2
1.4
0.0
0.5
1.0
1.5
2.0
2.5
obs
Gaussian kernel with band width 1.06

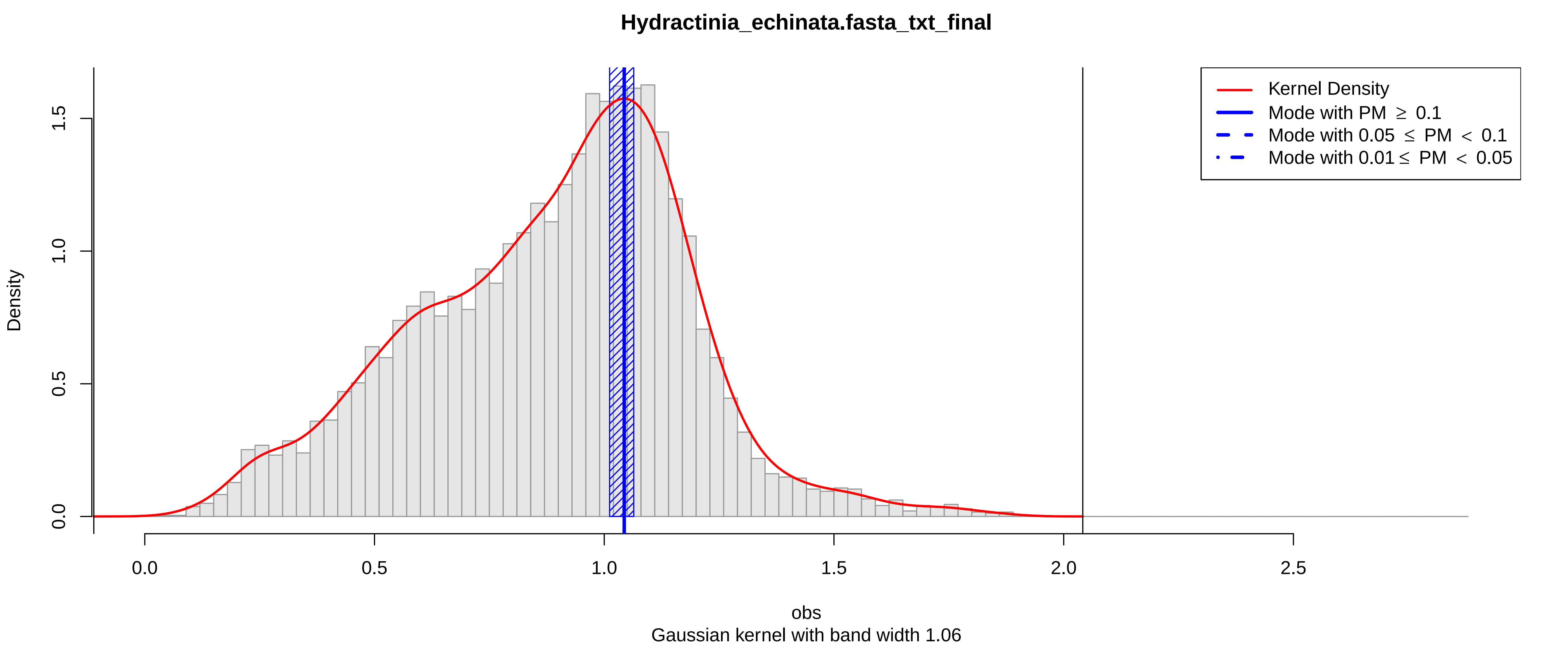

Hydractinia_echinata.fasta_txt_final
Kernel Density
Mode with PM ≥ 0.1
Mode with 0.05 ≤ PM < 0.1
Mode with 0.01 ≤ PM < 0.05
Density
0.0
0.5
1.0
1.5
0.0
0.5
1.0
1.5
2.0
2.5
obs
Gaussian kernel with band width 1.06

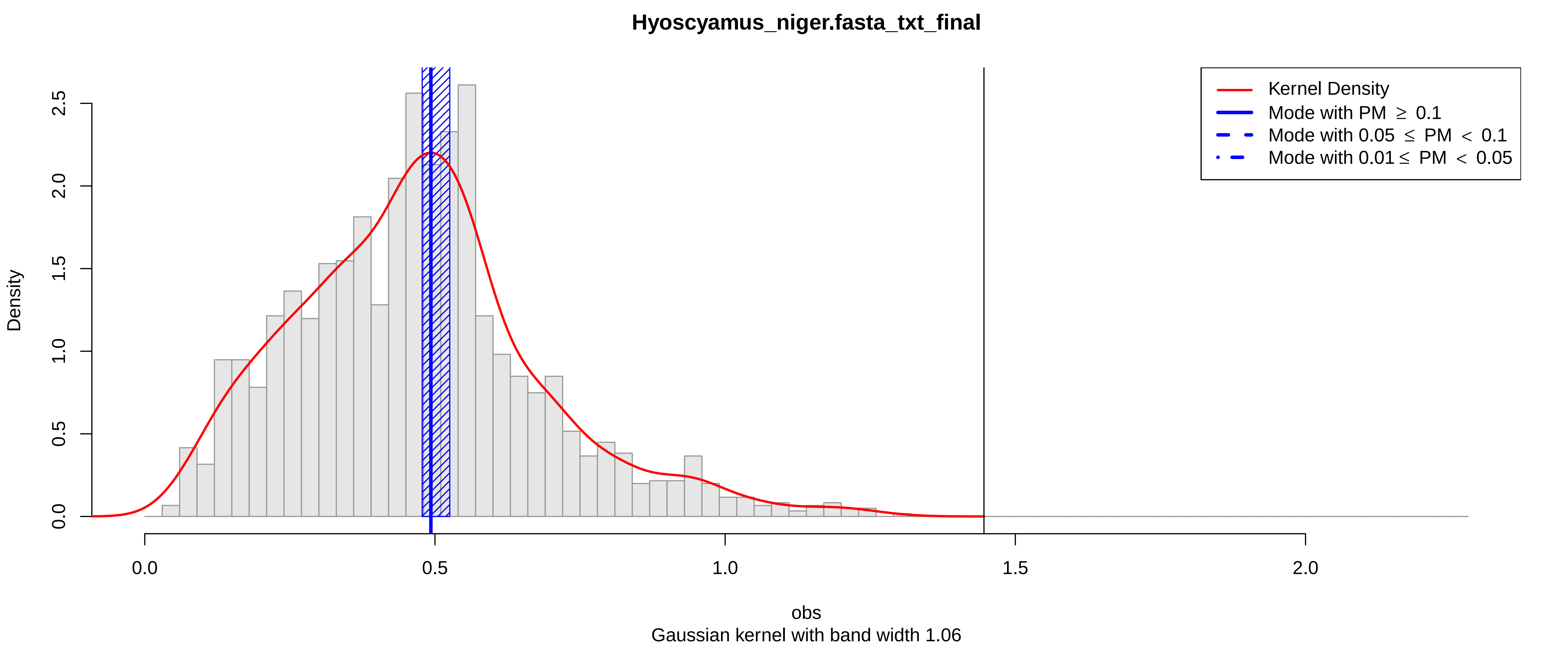
Hyoscyamus_niger.fasta_txt_final
Kernel Density
Mode with PM ≥ 0.1
Mode with 0.05 ≤ PM < 0.1
Mode with 0.01 ≤ PM < 0.05
Density
0.0
0.5
1.0
1.5
2.0
2.5
0.0
0.5
1.0
1.5
2.0
obs
Gaussian kernel with band width 1.06

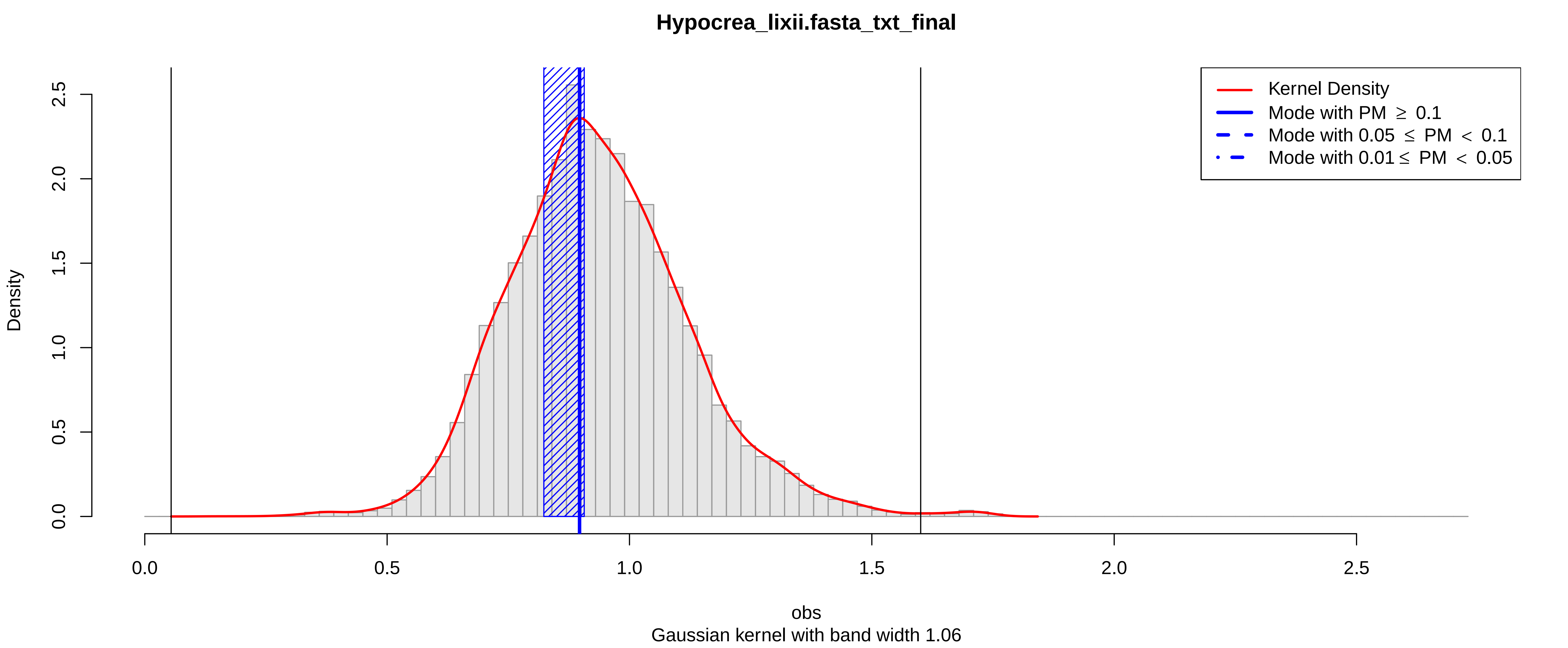
Hypocrea_lixii.fasta_txt_final
Kernel Density
Mode with PM ≥ 0.1
Mode with 0.05 ≤ PM < 0.1
Mode with 0.01 ≤ PM < 0.05
Density
0.0
0.5
1.0
1.5
2.0
2.5
obs
Gaussian kernel with band width 1.06

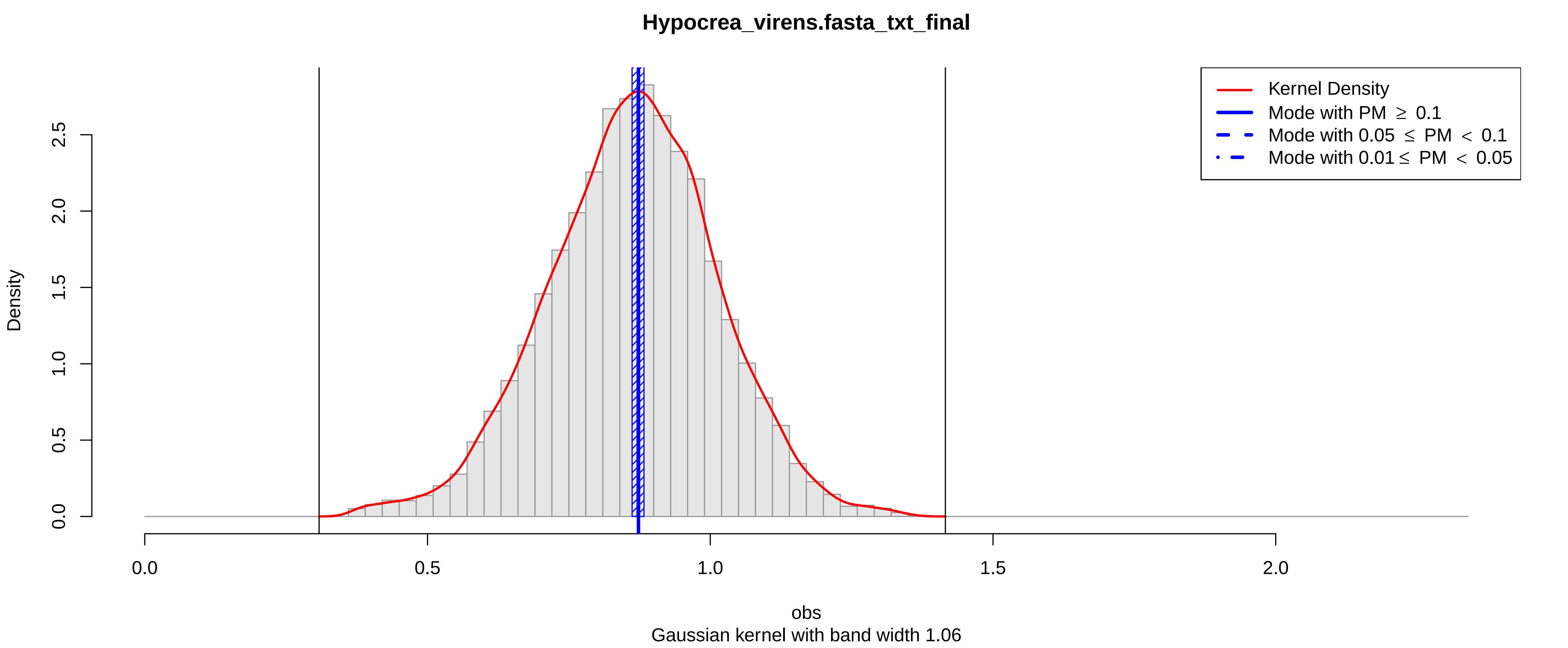
Hypocrea_virens.fasta_txt_final
Kernel Density
Mode with PM ≥ 0.1
Mode with 0.05 ≤ PM < 0.1
Mode with 0.01 ≤ PM < 0.05
Density
0.0
0.5
1.0
1.5
2.0
2.5
0.0
0.5
1.0
1.5
2.0
obs
Gaussian kernel with band width 1.06

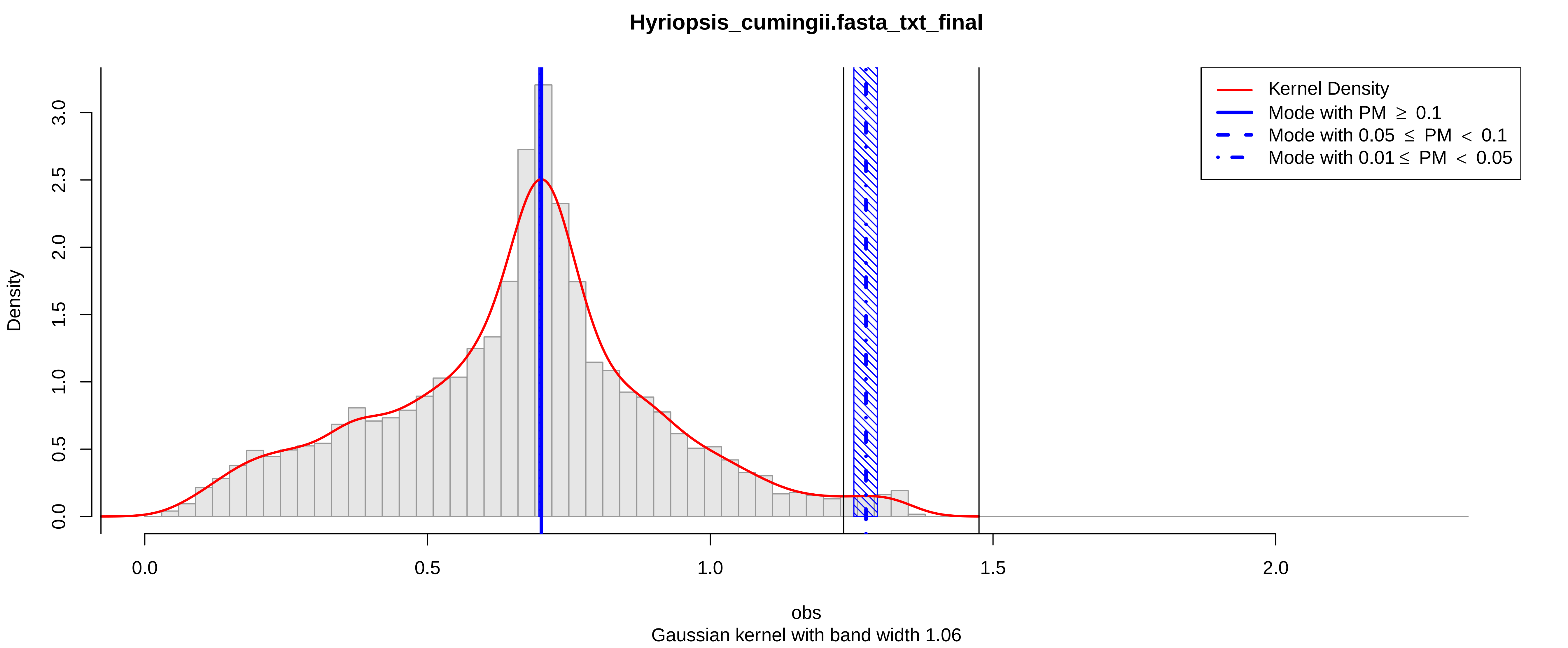

Hyriopsis_cumingii.fasta_txt_final
Kernel Density
Mode with PM ≥ 0.1
Mode with 0.05 ≤ PM < 0.1
Mode with 0.01 ≤ PM < 0.05
Density
0.0
0.5
1.0
1.5
2.0
2.5
3.0
0.0
0.5
1.0
1.5
2.0
obs
Gaussian kernel with band width 1.06

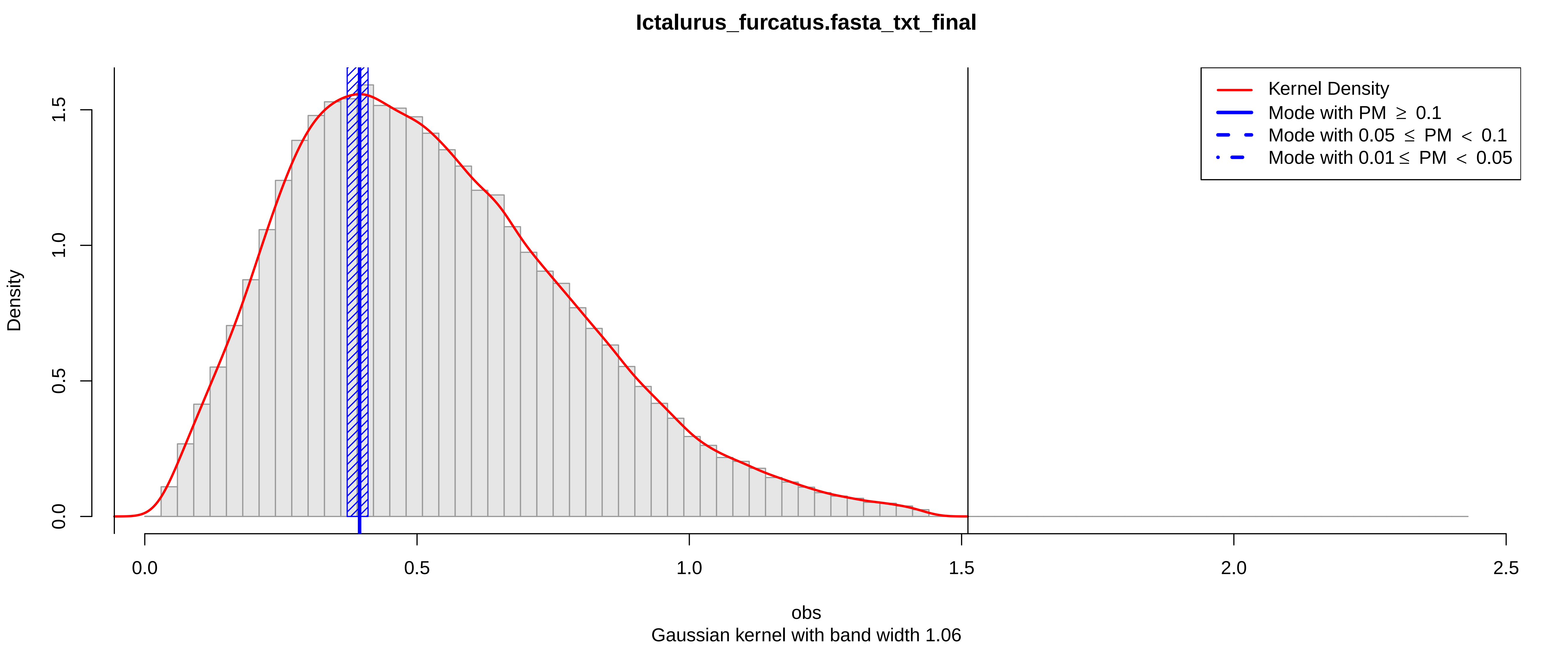
Ictalurus_furcatus.fasta_txt_final
Kernel Density
Mode with PM ≥ 0.1
Mode with 0.05 ≤ PM < 0.1
Mode with 0.01 ≤ PM < 0.05
Density
0.0
0.5
1.0
1.5
0.0
0.5
1.0
1.5
2.0
2.5
obs
Gaussian kernel with band width 1.06

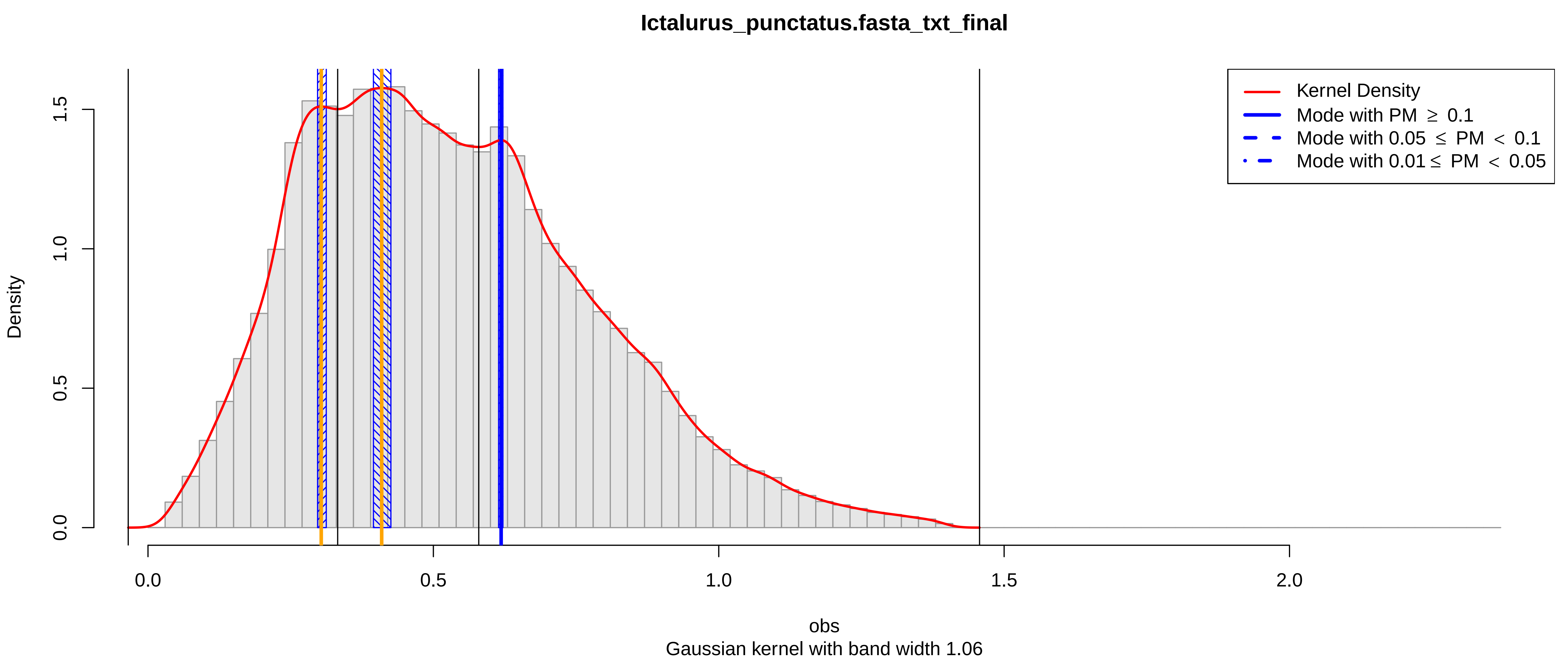
Ictalurus_punctatus.fasta_txt_final
Kernel Density
Mode with PM ≥ 0.1
Mode with 0.05 ≤ PM < 0.1
Mode with 0.01 ≤ PM < 0.05
Density
0.0
0.5
1.0
1.5
0.0
0.5
1.0
1.5
2.0
obs
Gaussian kernel with band width 1.06

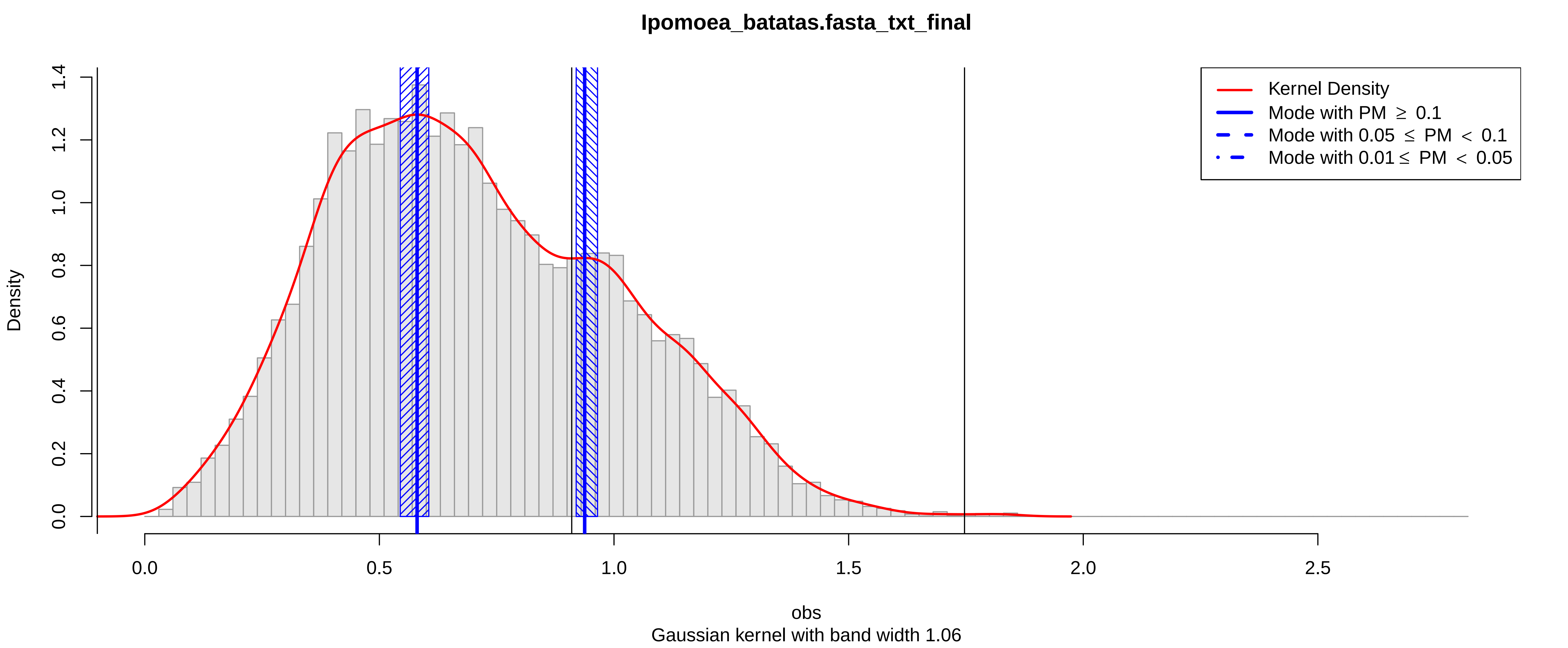
Ipomoea_batatas.fasta_txt_final
Kernel Density
Mode with PM ≥ 0.1
Mode with 0.05 ≤ PM < 0.1
Mode with 0.01 ≤ PM < 0.05
Density
0.0
0.2
0.4
0.6
0.8
1.0
1.2
1.4
0.0
0.5
1.0
1.5
2.0
2.5
obs
Gaussian kernel with band width 1.06

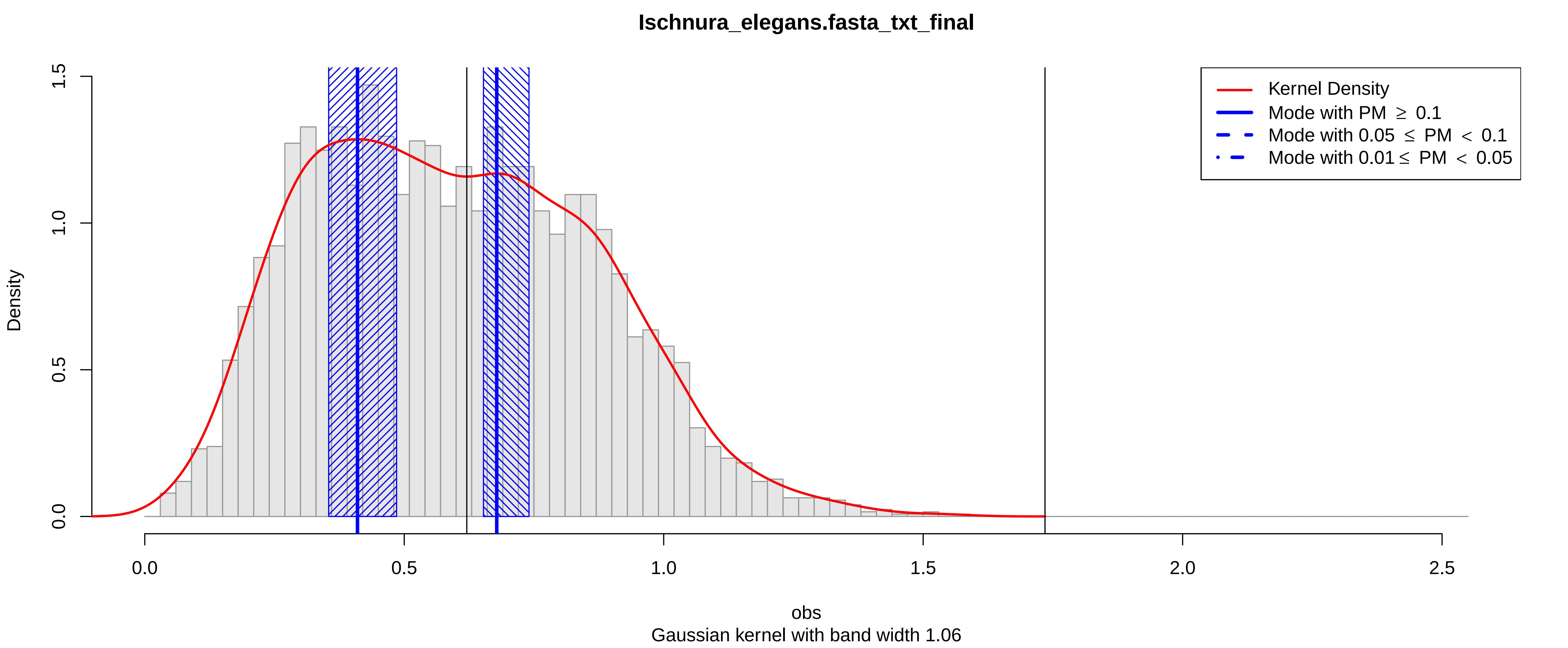

Ischnura_elegans.fasta_txt_final
Density
0.0
0.5
1.0
1.5
0.0
0.5
1.0
1.5
2.0
2.5
obs
Gaussian kernel with band width 1.06
Kernel Density
Mode with PM ≥ 0.1
Mode with 0.05 ≤ PM < 0.1
Mode with 0.01 ≤ PM < 0.05

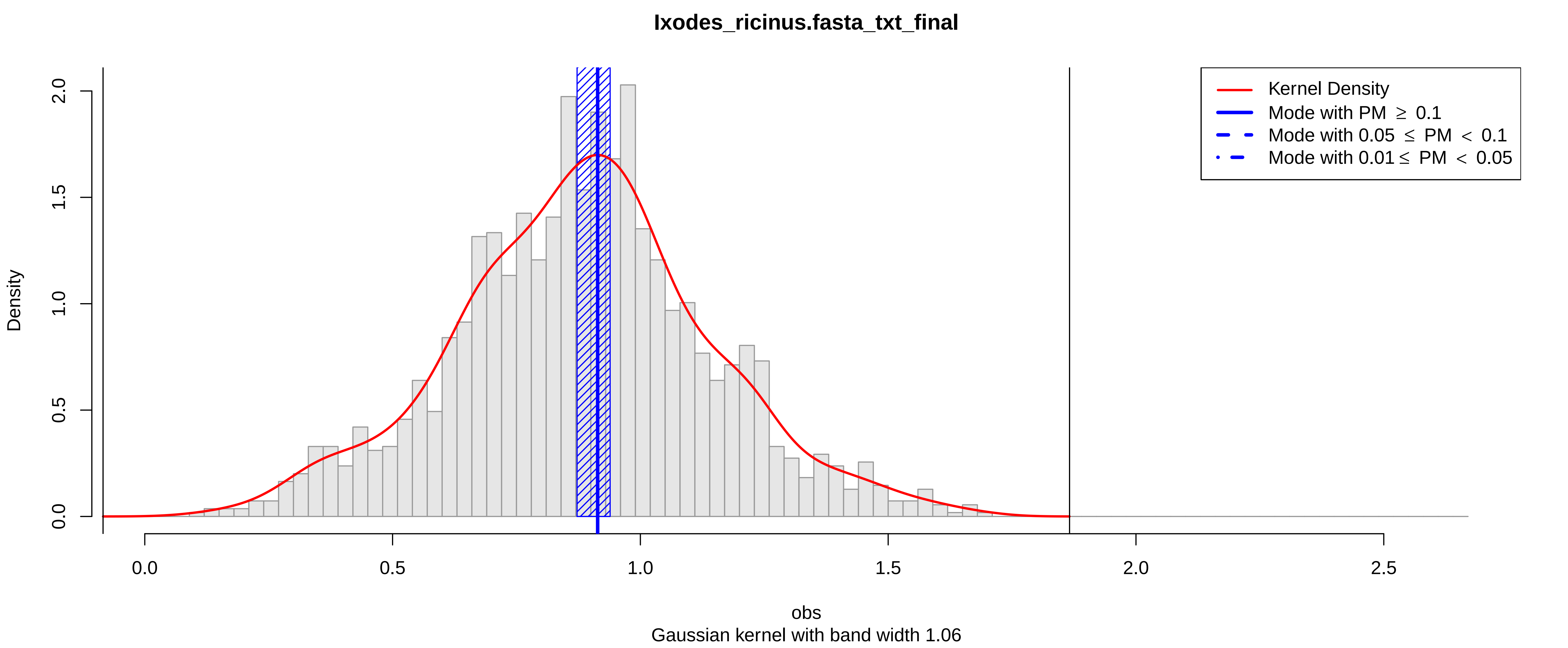
Ixodes_ricinus.fasta_txt_final
Kernel Density
Mode with PM ≥ 0.1
Mode with 0.05 ≤ PM < 0.1
Mode with 0.01 ≤ PM < 0.05
Density
0.0
0.5
1.0
1.5
2.0
0.0
0.5
1.0
1.5
2.0
2.5
obs
Gaussian kernel with band width 1.06

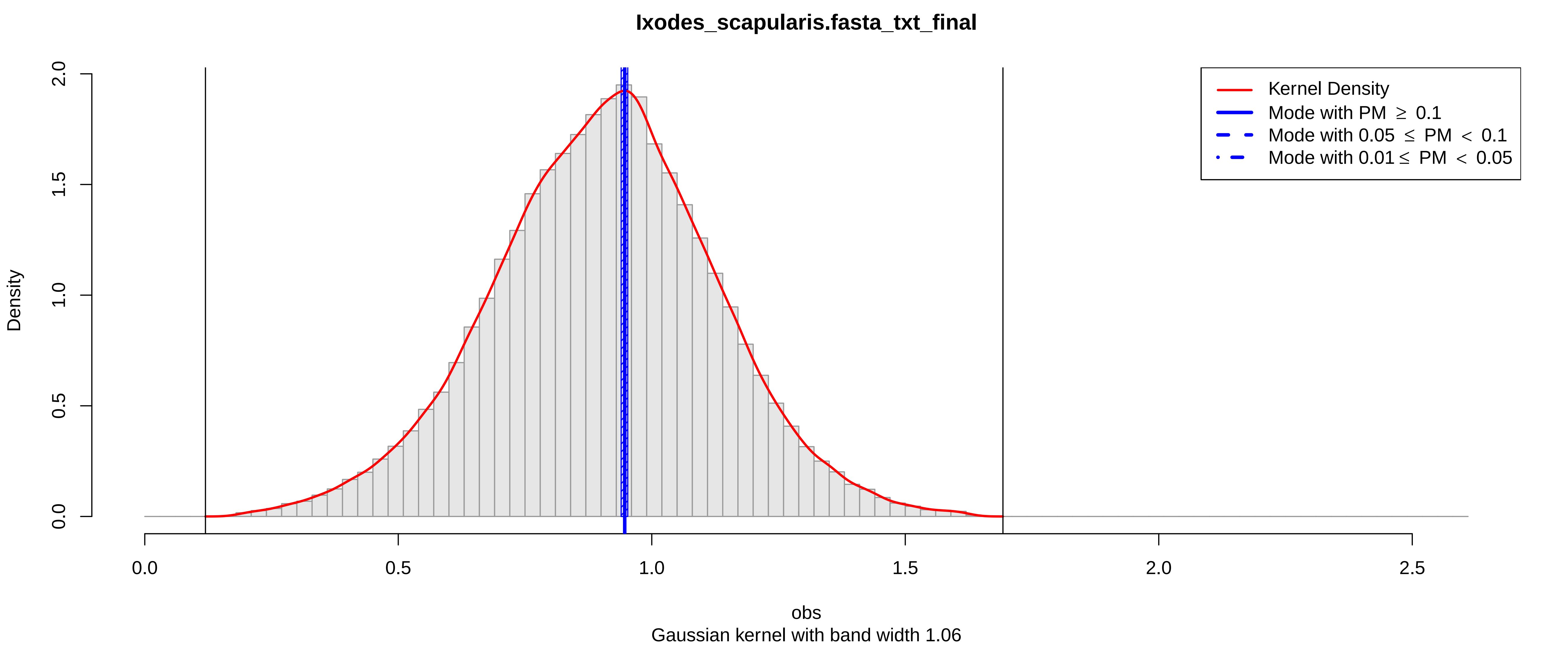
Ixodes_scapularis.fasta_txt_final
Kernel Density
Mode with PM ≥ 0.1
Mode with 0.05 ≤ PM < 0.1
Mode with 0.01 ≤ PM < 0.05
Density
0.0
0.5
1.0
1.5
2.0
0.0
0.5
1.0
1.5
2.0
2.5
obs
Gaussian kernel with band width 1.06

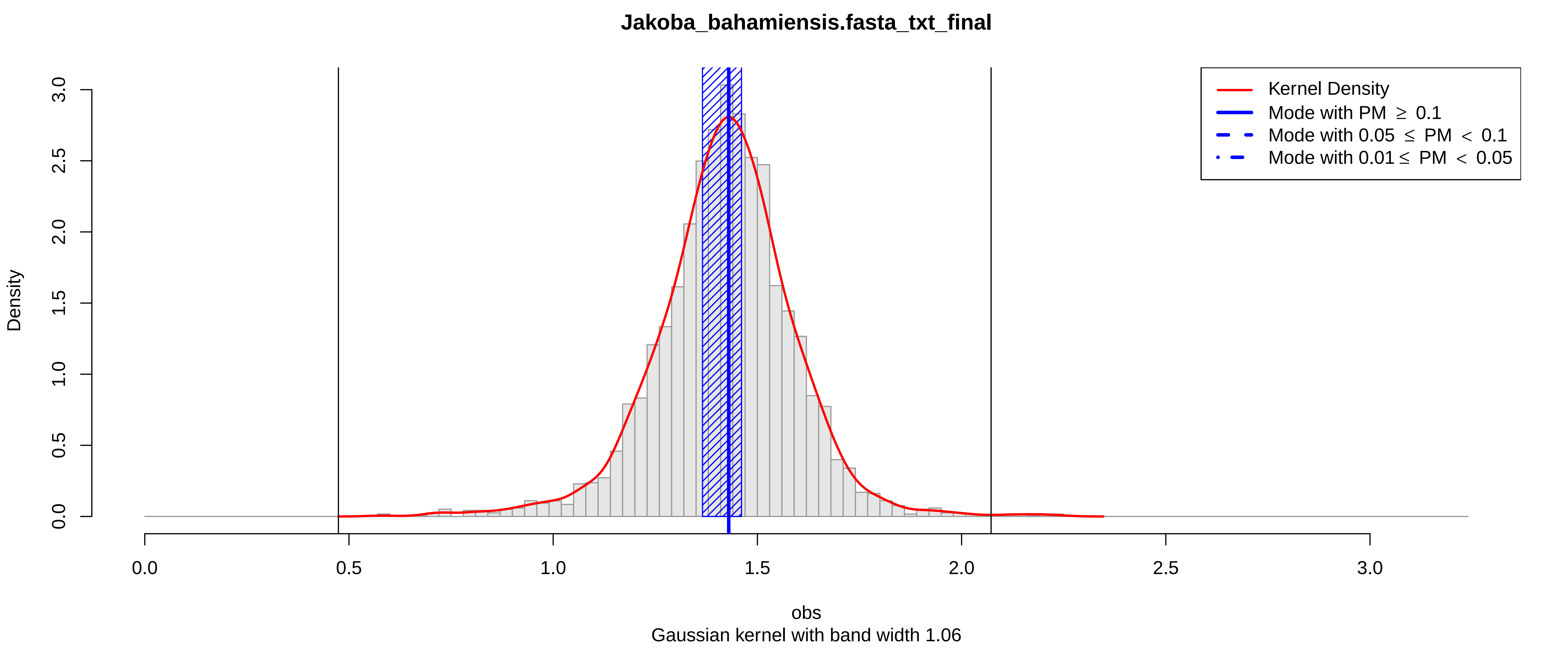

Jakoba_bahamiensis.fasta_txt_final
Kernel Density
Mode with PM ≥ 0.1
Mode with 0.05 ≤ PM < 0.1
Mode with 0.01 ≤ PM < 0.05
Density
0.0
0.5
1.0
1.5
2.0
2.5
3.0
obs
Gaussian kernel with band width 1.06

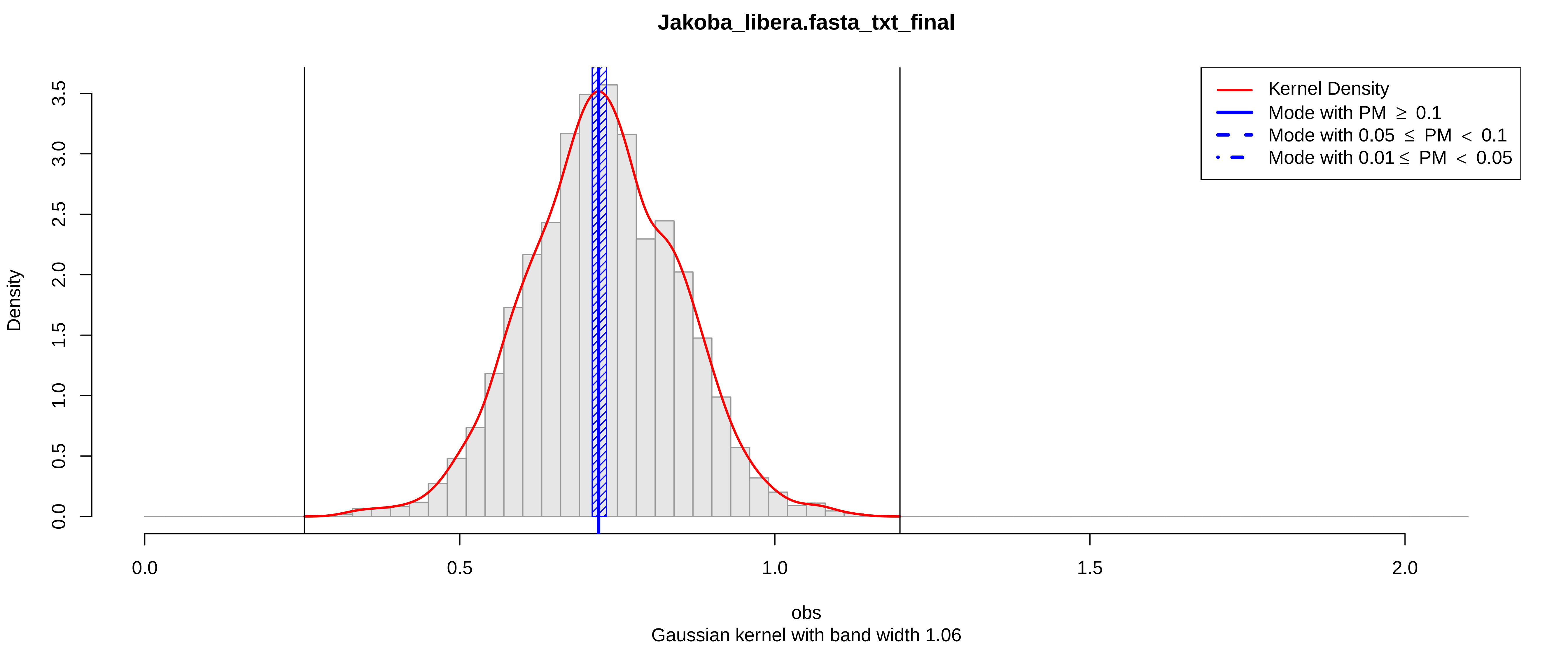
Jakoba_libera.fasta_txt_final
Kernel Density
Mode with PM ≥ 0.1
Mode with 0.05 ≤ PM < 0.1
Mode with 0.01 ≤ PM < 0.05
Density
0.0
0.5
1.0
1.5
2.0
2.5
3.0
3.5
0.0
0.5
1.0
1.5
2.0
obs
Gaussian kernel with band width 1.06

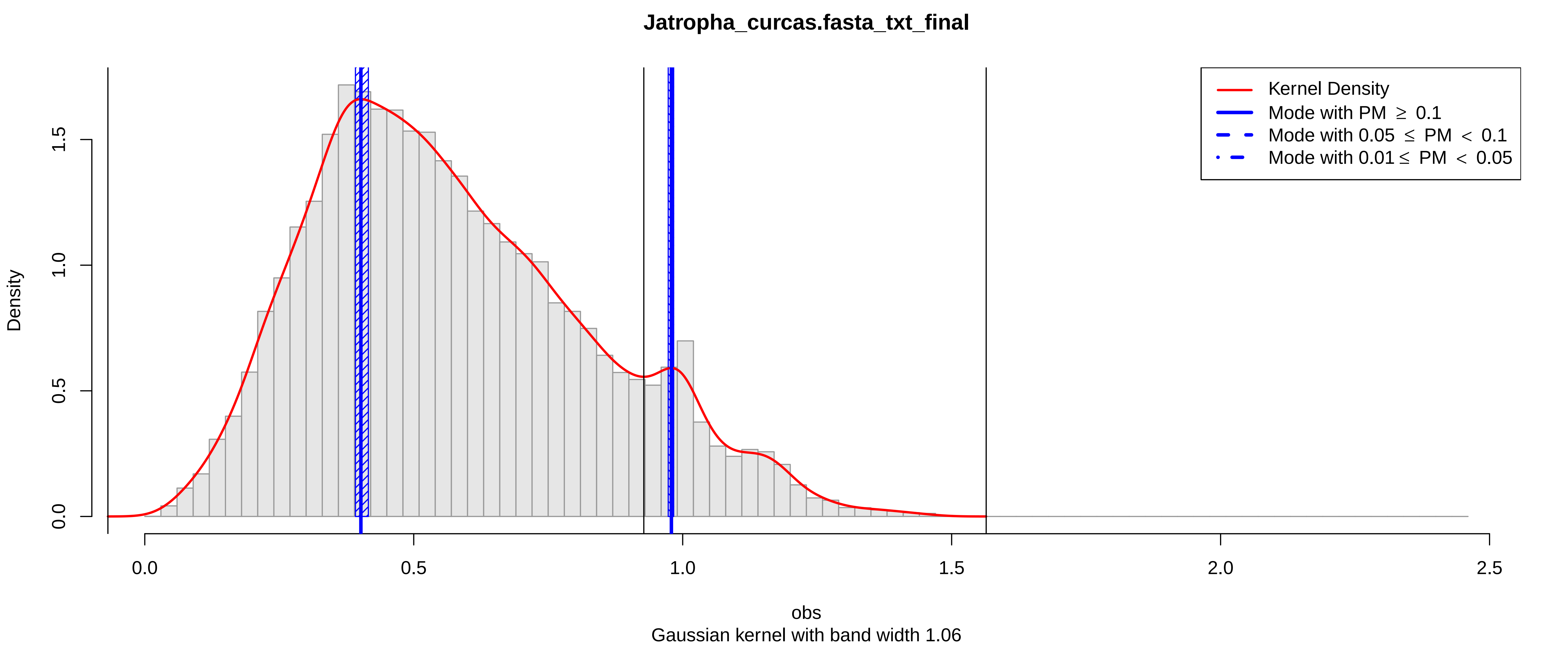

Jatropha_curcas.fasta_txt_final
Kernel Density
Mode with PM ≥ 0.1
Mode with 0.05 ≤ PM < 0.1
Mode with 0.01 ≤ PM < 0.05
Density
0.0
0.5
1.0
1.5
0.0
0.5
1.0
1.5
2.0
2.5
obs
Gaussian kernel with band width 1.06

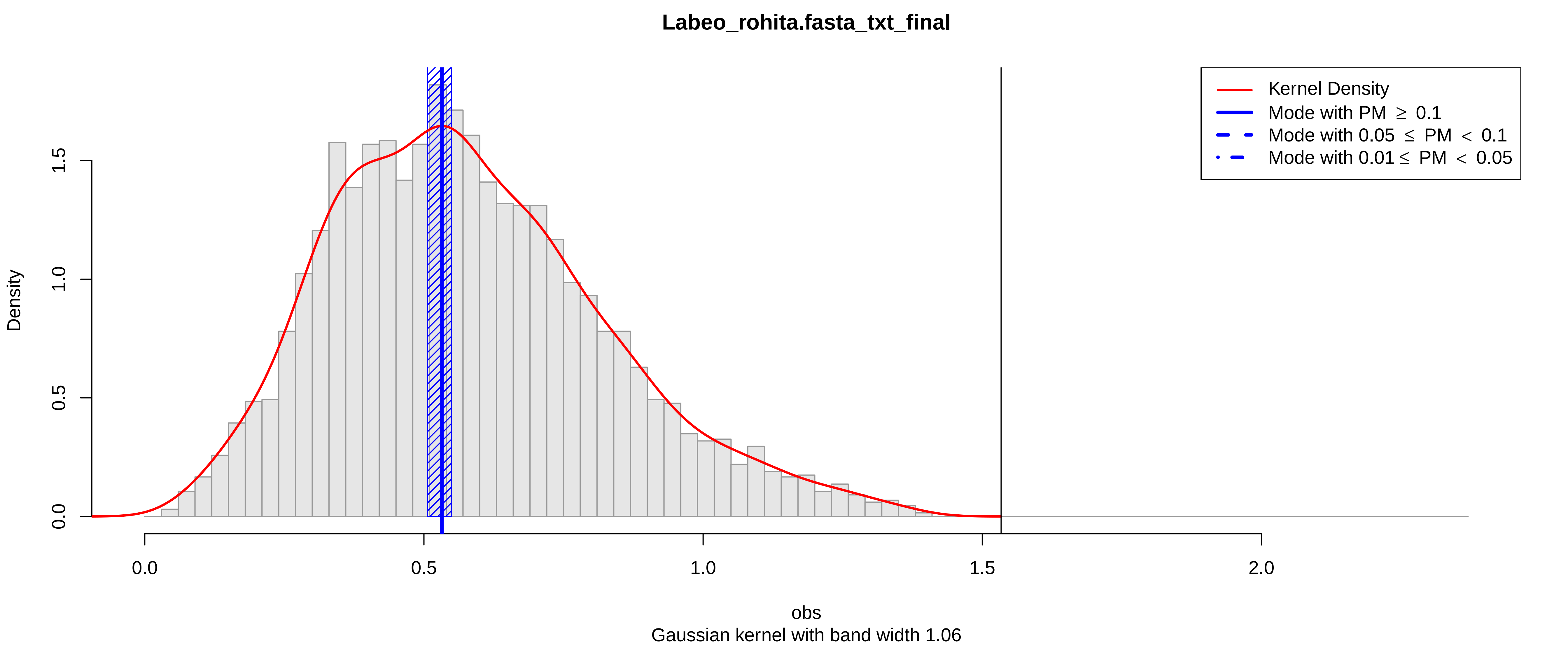
Labeo_rohita.fasta_txt_final
Kernel Density
Mode with PM ≥ 0.1
Mode with 0.05 ≤ PM < 0.1
Mode with 0.01 ≤ PM < 0.05
Density
0.0
0.5
1.0
1.5
0.0
0.5
1.0
1.5
2.0
obs
Gaussian kernel with band width 1.06

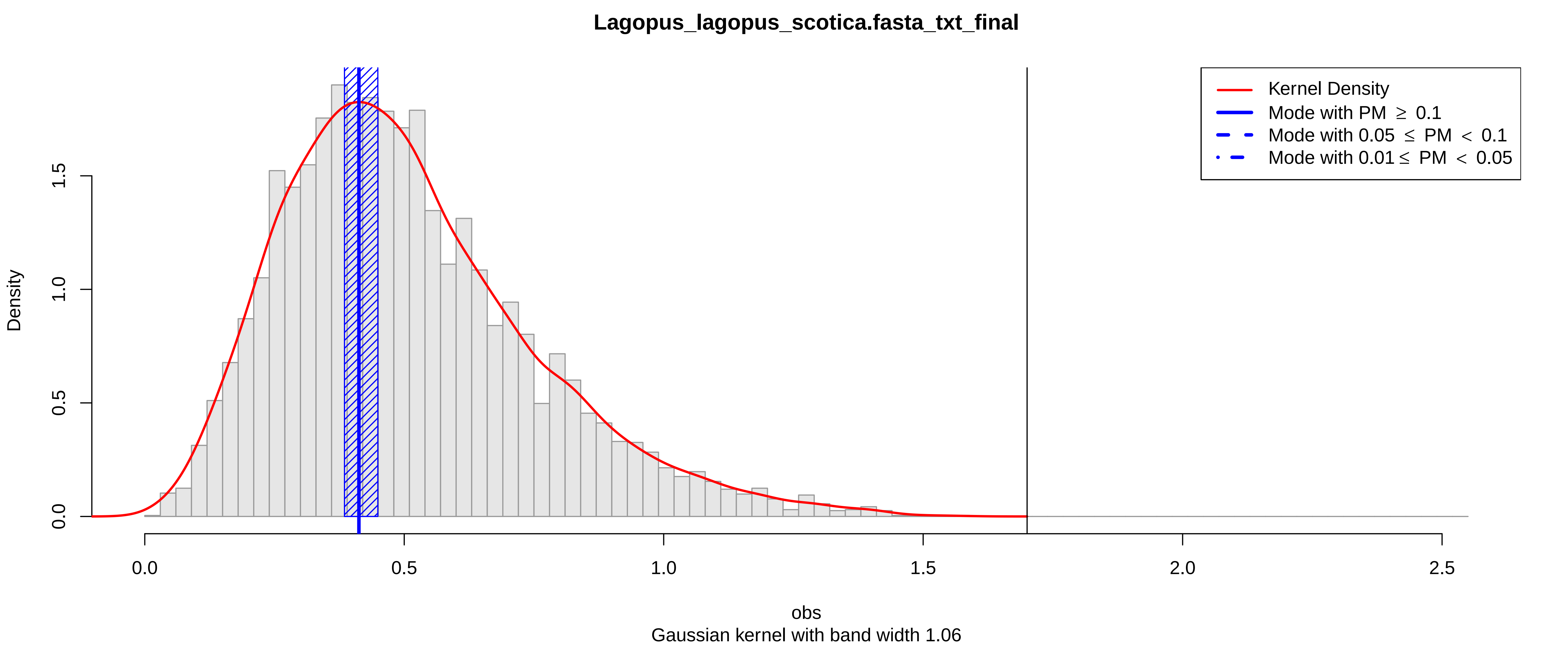
Lagopus_lagopus_scotica.fasta_txt_final
Kernel Density
Mode with PM ≥ 0.1
Mode with 0.05 ≤ PM < 0.1
Mode with 0.01 ≤ PM < 0.05
Density
0.0
0.5
1.0
1.5
0.0
0.5
1.0
1.5
2.0
2.5
obs
Gaussian kernel with band width 1.06

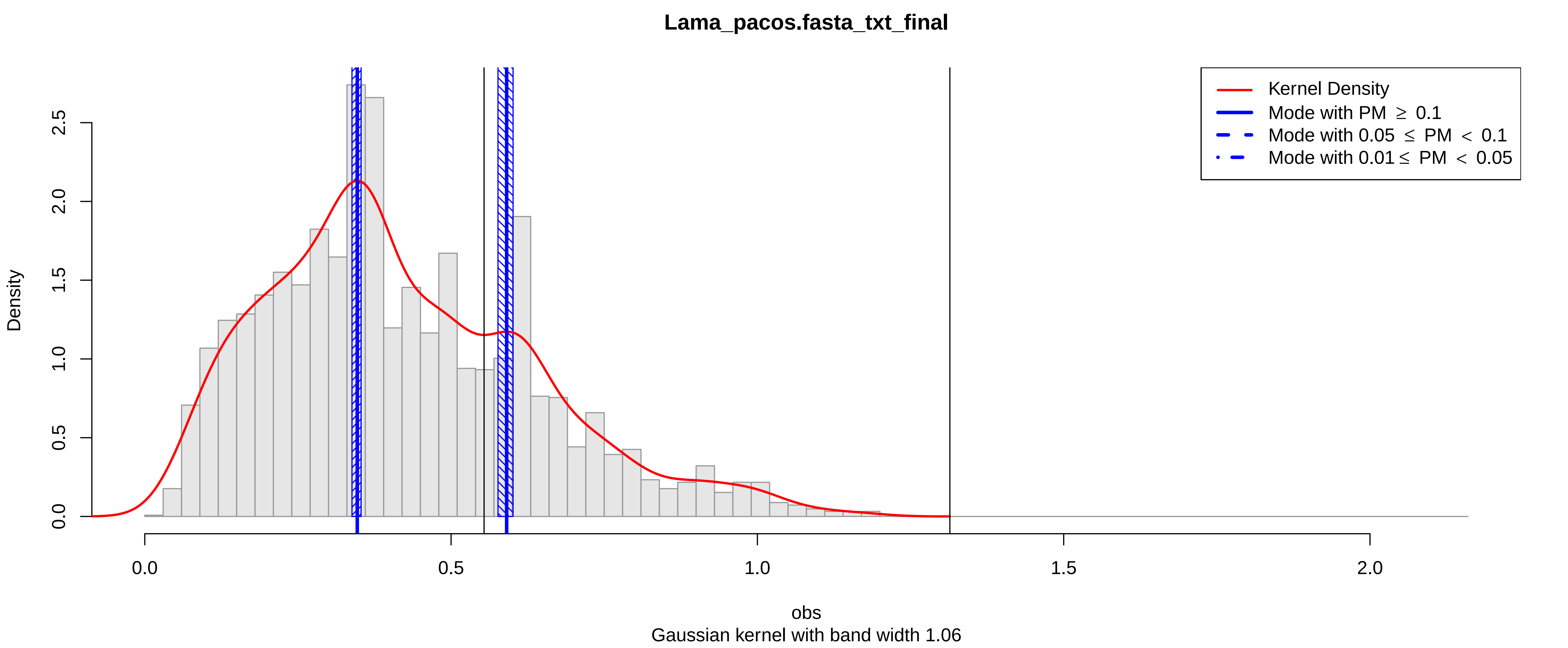

Lama_pacos.fasta_txt_final
Kernel Density
Mode with PM ≥ 0.1
Mode with 0.05 ≤ PM < 0.1
Mode with 0.01 ≤ PM < 0.05
Density
0.0
0.5
1.0
1.5
2.0
2.5
0.0
0.5
1.0
1.5
2.0
obs
Gaussian kernel with band width 1.06

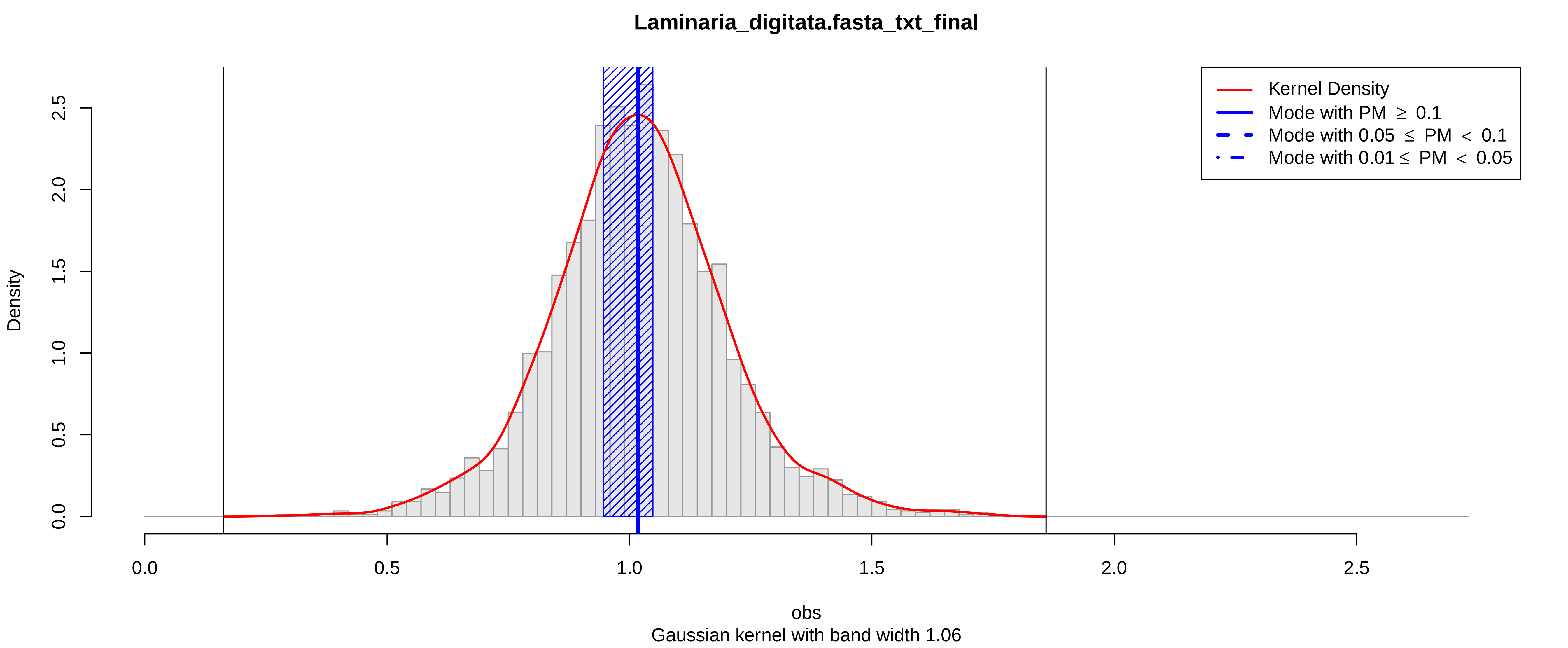

Laminaria_digitata.fasta_txt_final
Kernel Density
Mode with PM ≥ 0.1
Mode with 0.05 ≤ PM < 0.1
Mode with 0.01 ≤ PM < 0.05
Density
0.0
0.5
1.0
1.5
2.0
2.5
obs
Gaussian kernel with band width 1.06

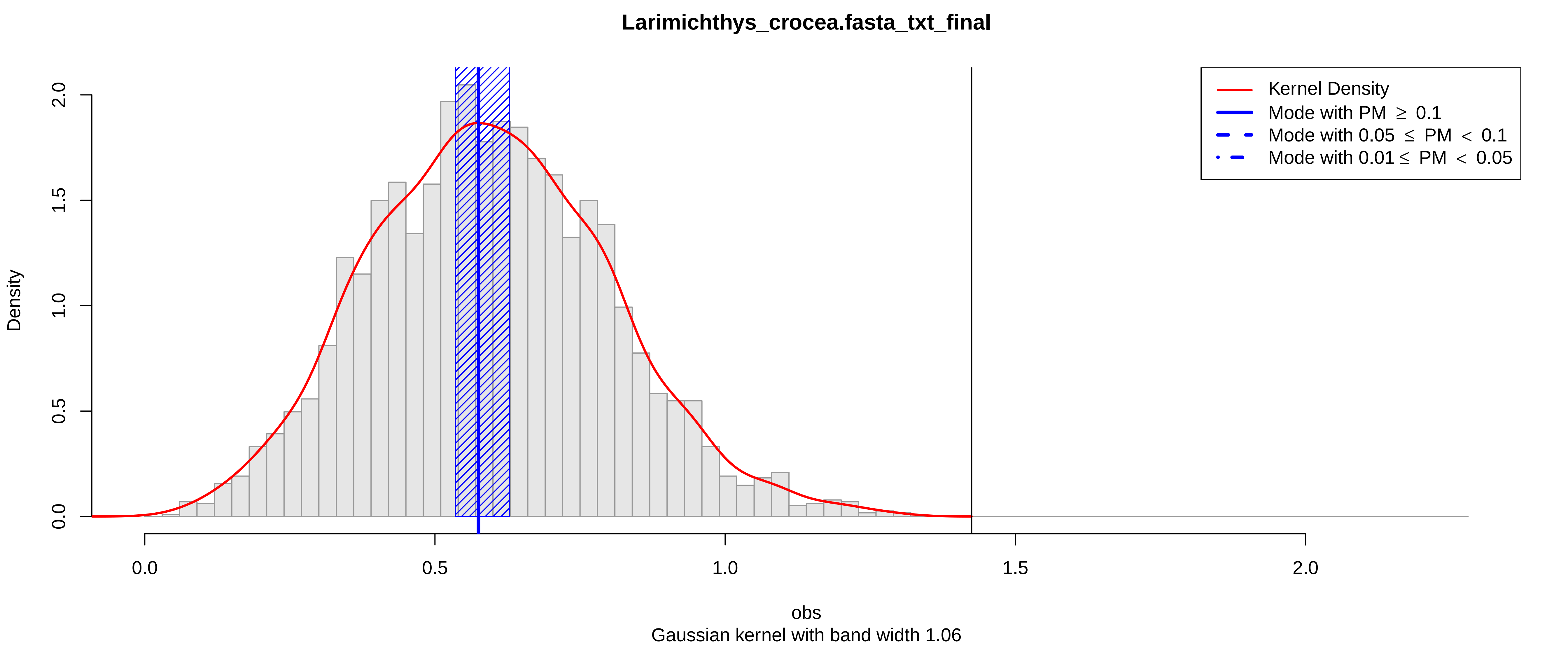

Larimichthys_crocea.fasta_txt_final
Kernel Density
Mode with PM ≥ 0.1
Mode with 0.05 ≤ PM < 0.1
Mode with 0.01 ≤ PM < 0.05
Density
0.0
0.5
1.0
1.5
2.0
0.0
0.5
1.0
1.5
2.0
obs
Gaussian kernel with band width 1.06

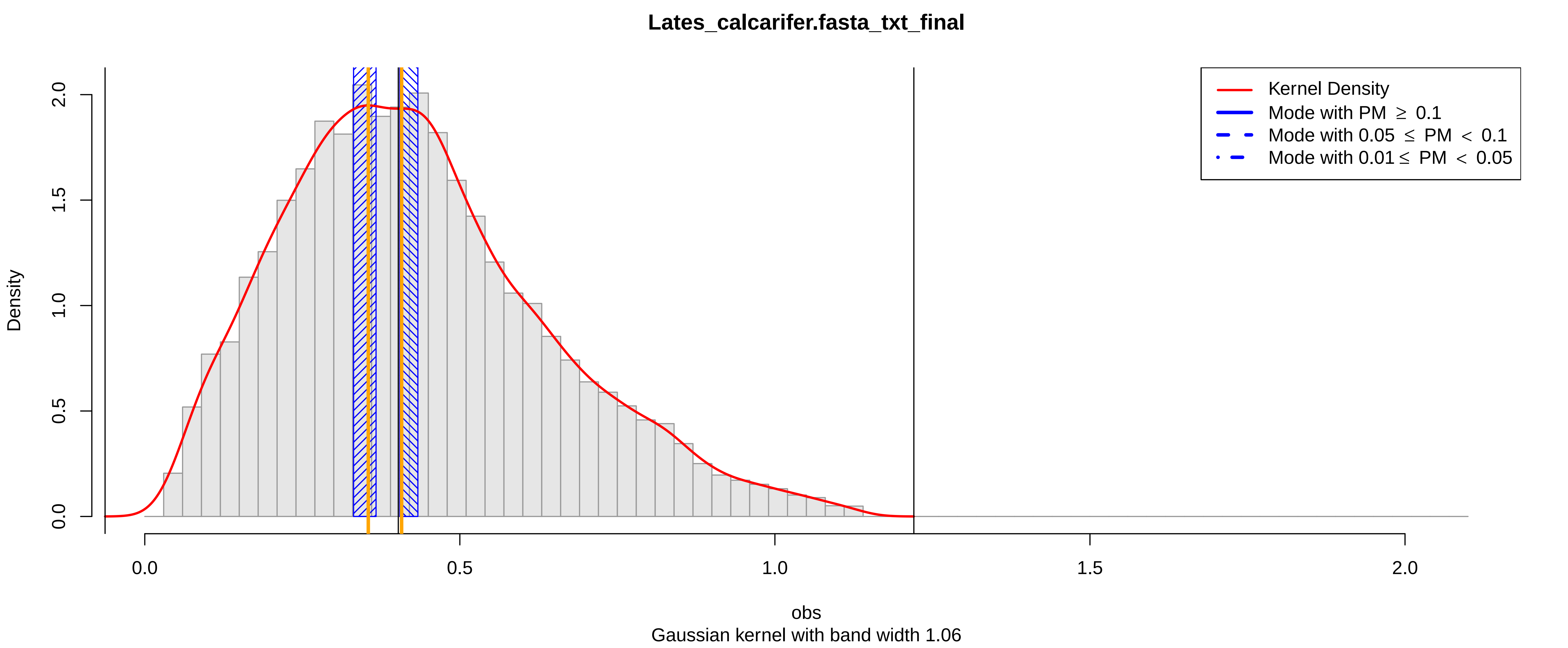

Lates_calcarifer.fasta_txt_final
Kernel Density
Mode with PM ≥ 0.1
Mode with 0.05 ≤ PM < 0.1
Mode with 0.01 ≤ PM < 0.05
Density
0.0
0.5
1.0
1.5
2.0
0.0
0.5
1.0
1.5
2.0
obs
Gaussian kernel with band width 1.06

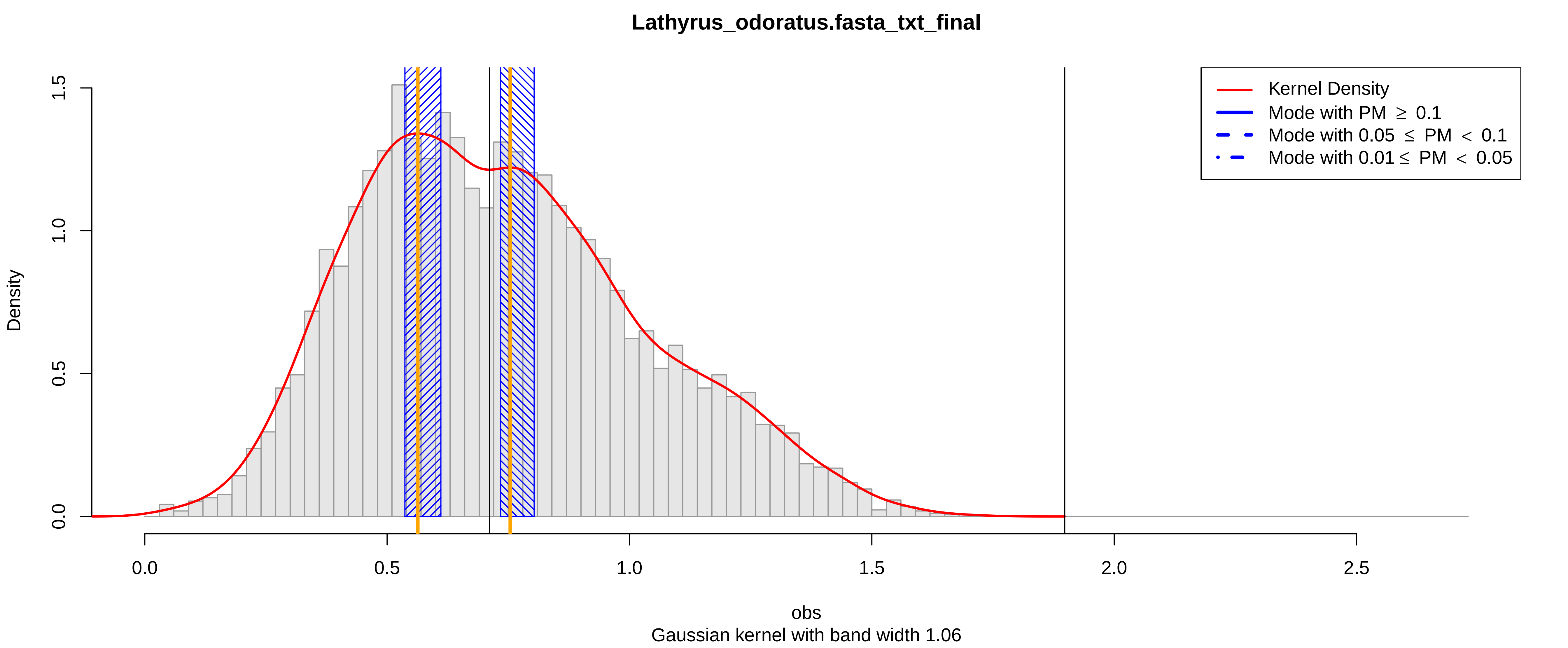

Lathyrus_odoratus.fasta_txt_final
Kernel Density
Mode with PM ≥ 0.1
Mode with 0.05 ≤ PM < 0.1
Mode with 0.01 ≤ PM < 0.05
Density
0.0
0.5
1.0
1.5
0.0
0.5
1.0
1.5
2.0
2.5
obs
Gaussian kernel with band width 1.06

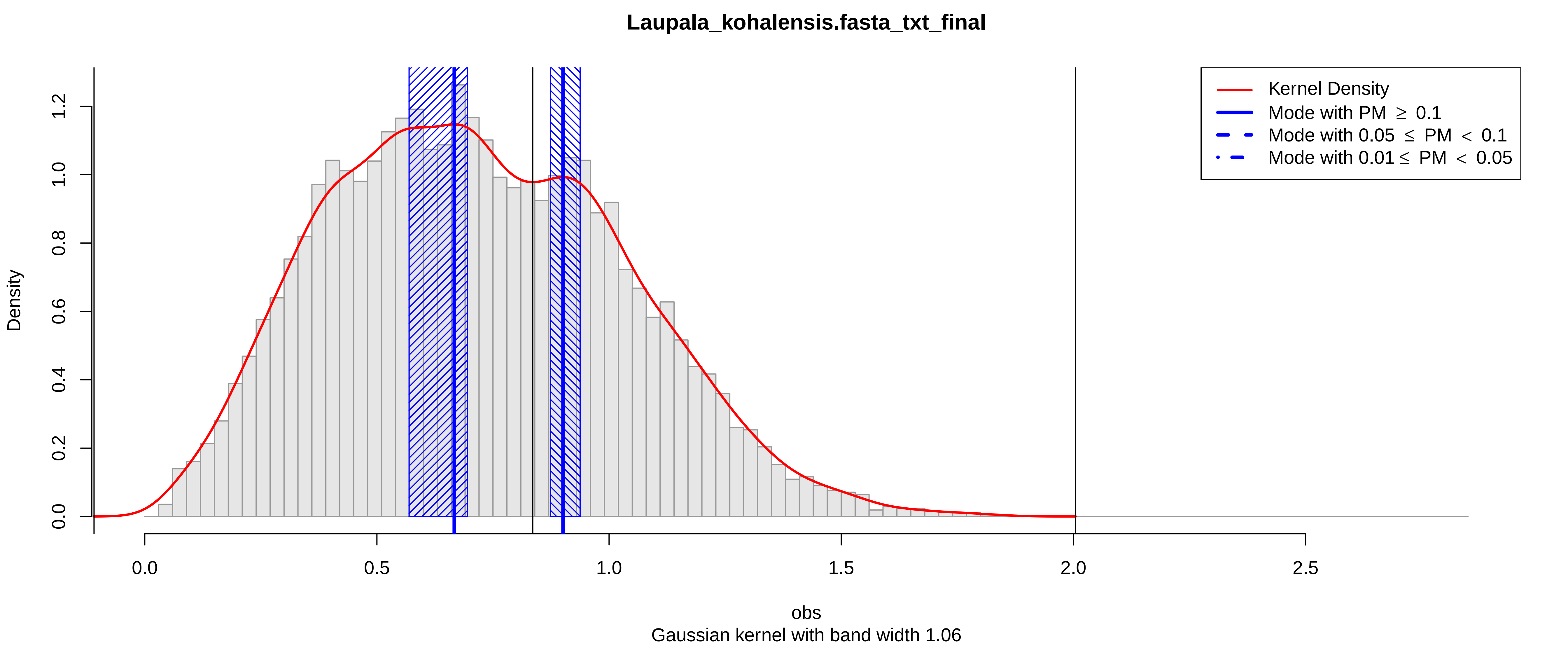
Laupala_kohalensis.fasta_txt_final
Kernel Density
Mode with PM ≥ 0.1
Mode with 0.05 ≤ PM < 0.1
Mode with 0.01 ≤ PM < 0.05
Density
0.0
0.2
0.4
0.6
0.8
1.0
1.2
0.0
0.5
1.0
1.5
2.0
2.5
obs
Gaussian kernel with band width 1.06

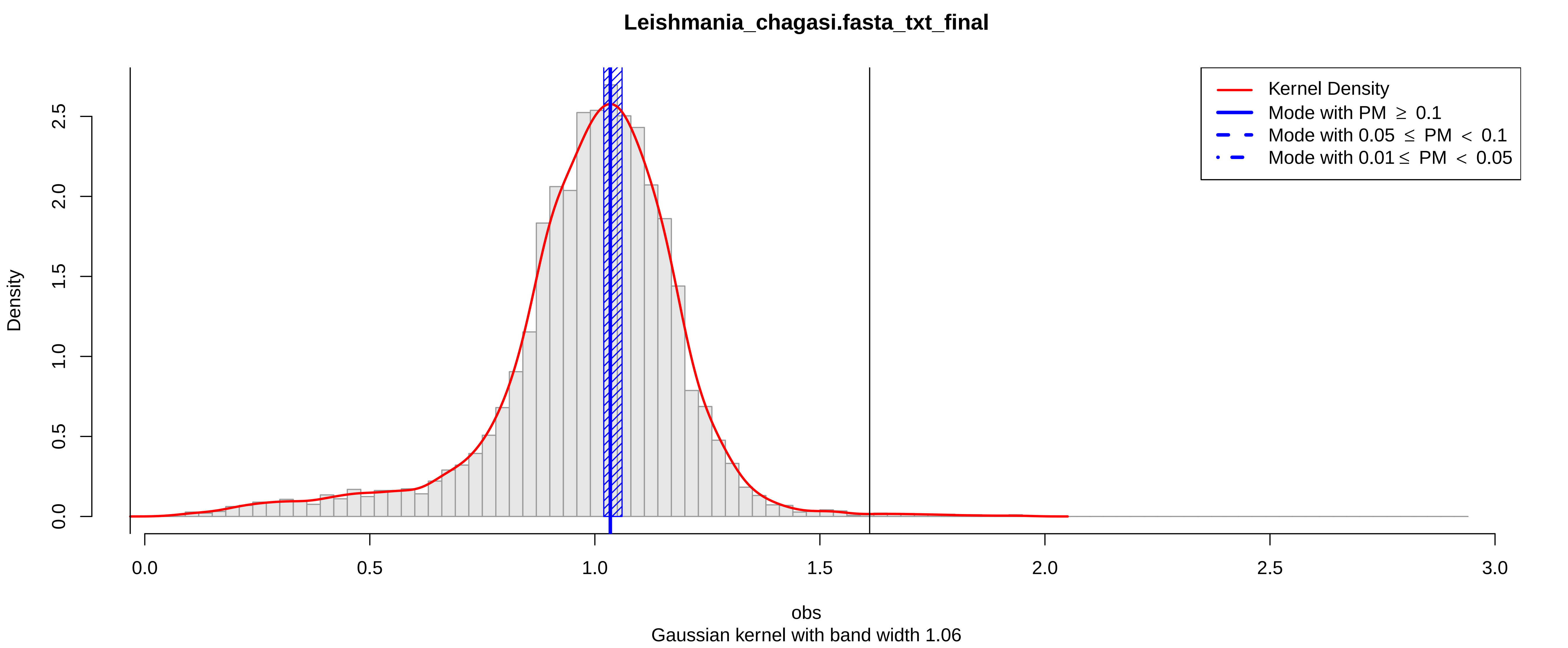

Leishmania_chagasi.fasta_txt_final
Kernel Density
Mode with PM ≥ 0.1
Mode with 0.05 ≤ PM < 0.1
Mode with 0.01 ≤ PM < 0.05
Density
0.0
0.5
1.0
1.5
2.0
2.5
0.0
0.5
1.0
1.5
2.0
2.5
3.0
obs
Gaussian kernel with band width 1.06

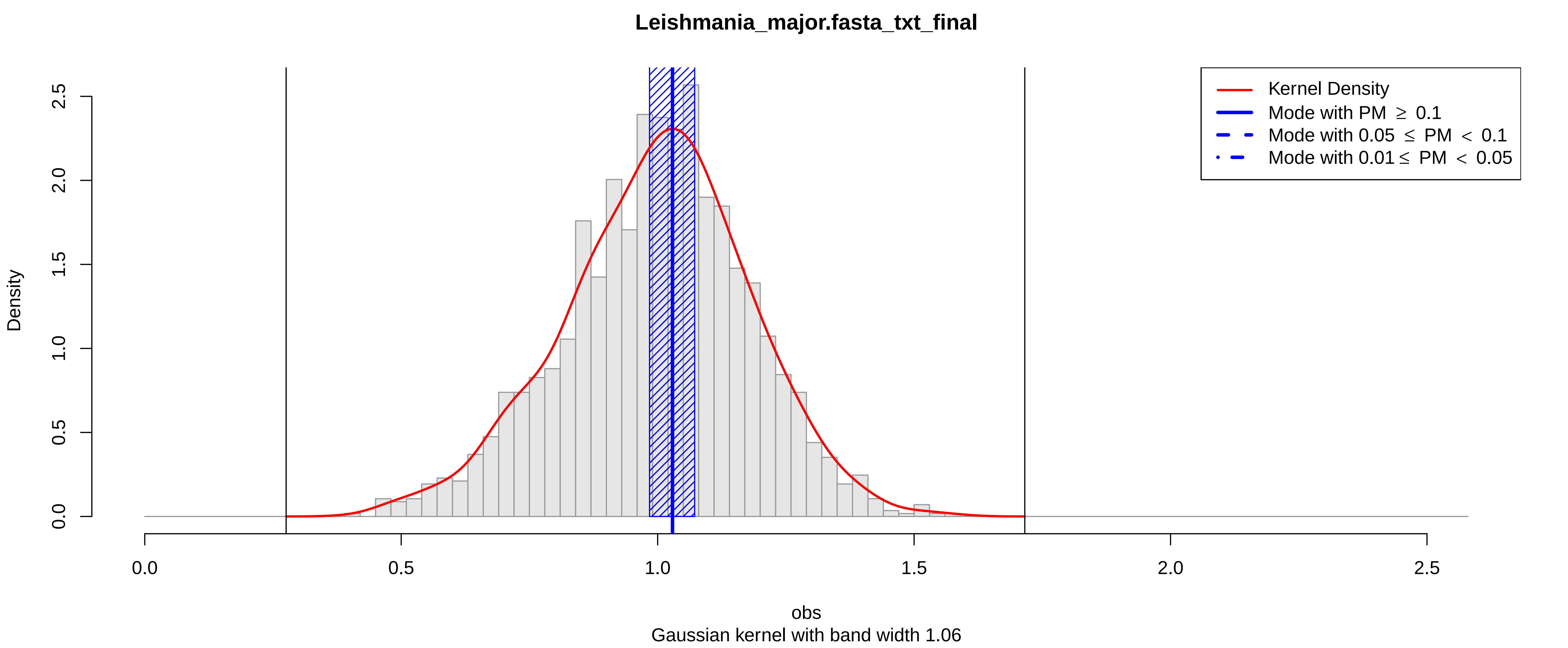

Leishmania_major.fasta_txt_final
Density
0.0
0.5
1.0
1.5
2.0
2.5
Kernel Density
Mode with PM ≥ 0.1
Mode with 0.05 ≤ PM < 0.1
Mode with 0.01 ≤ PM < 0.05
0.0
0.5
1.0
1.5
2.0
2.5
obs
Gaussian kernel with band width 1.06

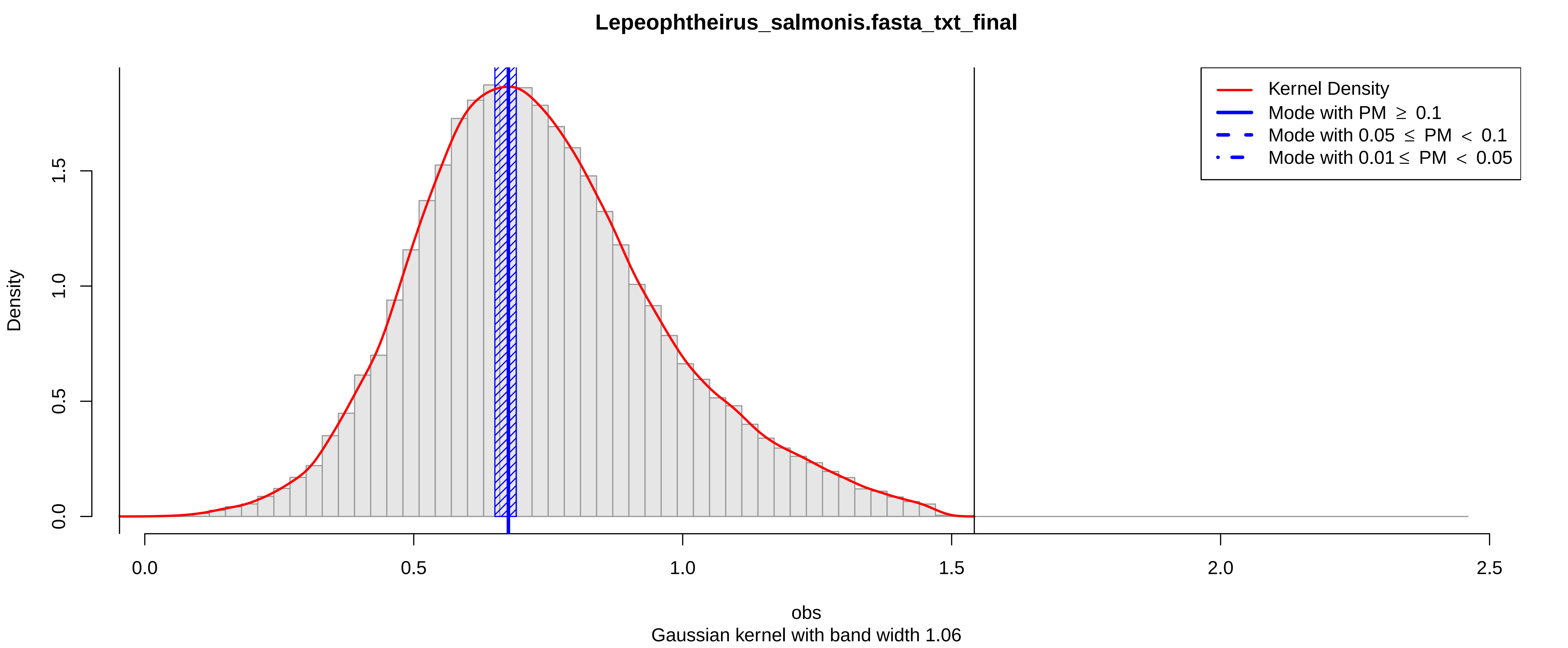

Lepeophtheirus_salmonis.fasta_txt_final
Kernel Density
Mode with PM ≥ 0.1
Mode with 0.05 ≤ PM < 0.1
Mode with 0.01 ≤ PM < 0.05
Density
0.0
0.5
1.0
1.5
0.0
0.5
1.0
1.5
2.0
2.5
obs
Gaussian kernel with band width 1.06

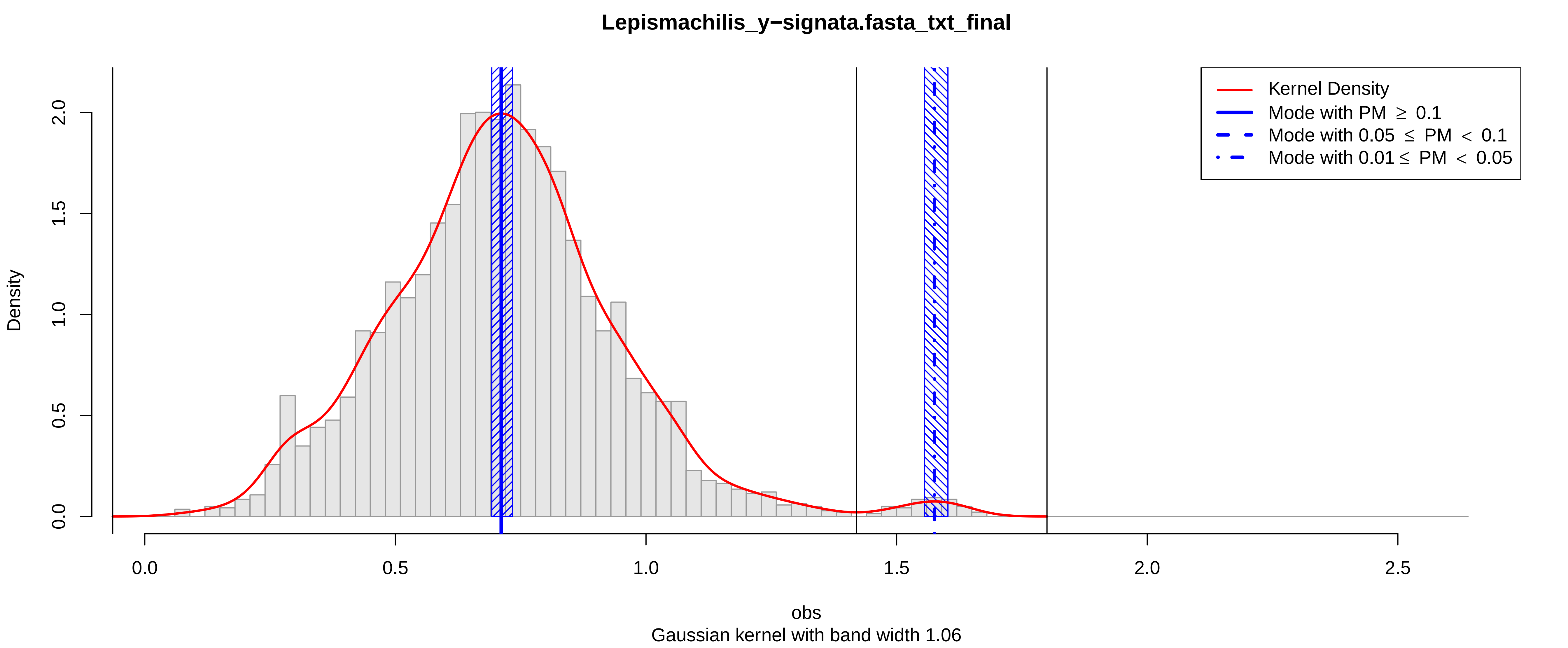

Lepismachilis_y−signata.fasta_txt_final
Kernel Density
Mode with PM ≥ 0.1
Mode with 0.05 ≤ PM < 0.1
Mode with 0.01 ≤ PM < 0.05
Density
0.0
0.5
1.0
1.5
2.0
2.5
obs
Gaussian kernel with band width 1.06

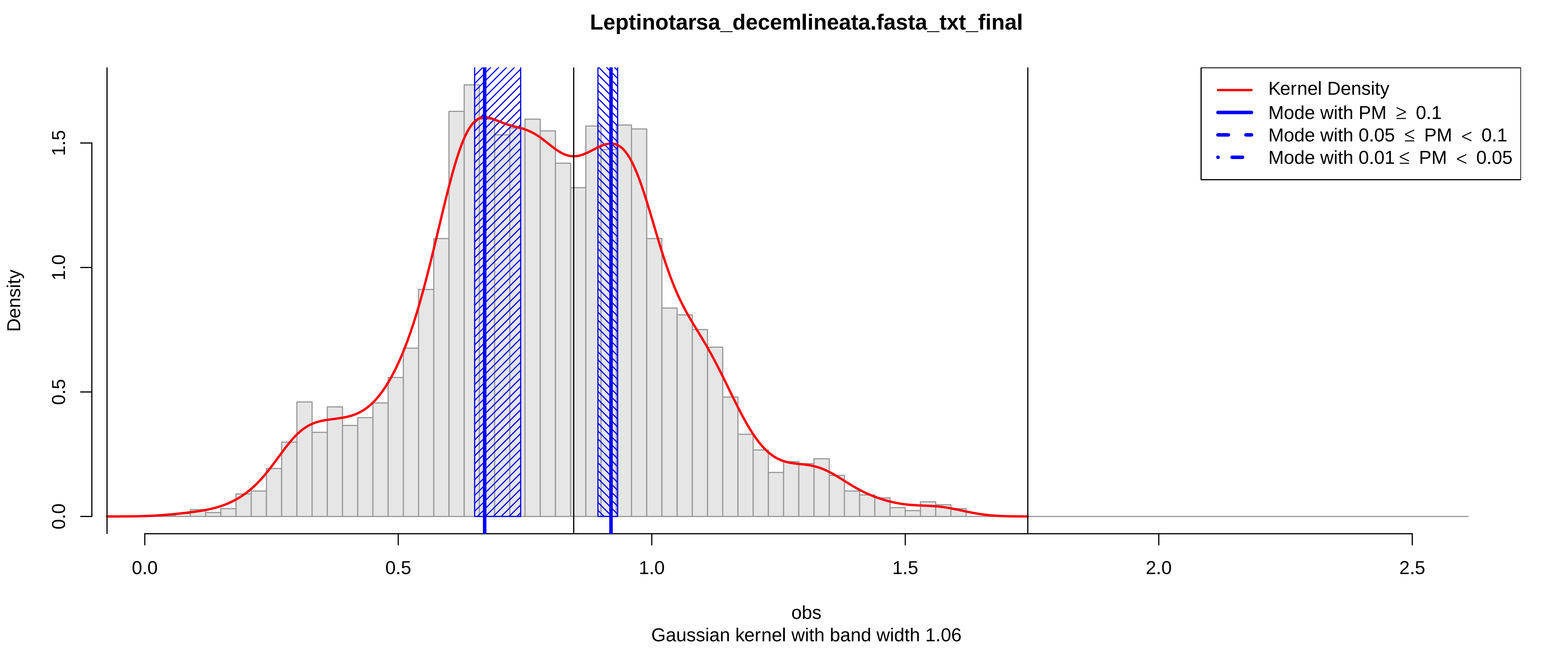
Leptinotarsa_decemlineata.fasta_txt_final
Density
obs
Gaussian kernel with band width 1.06
Kernel Density
Mode with PM ≥ 0.1
Mode with 0.05 ≤ PM < 0.1
Mode with 0.01 ≤ PM < 0.05
0.0
0.5
1.0
1.5
2.0
2.5

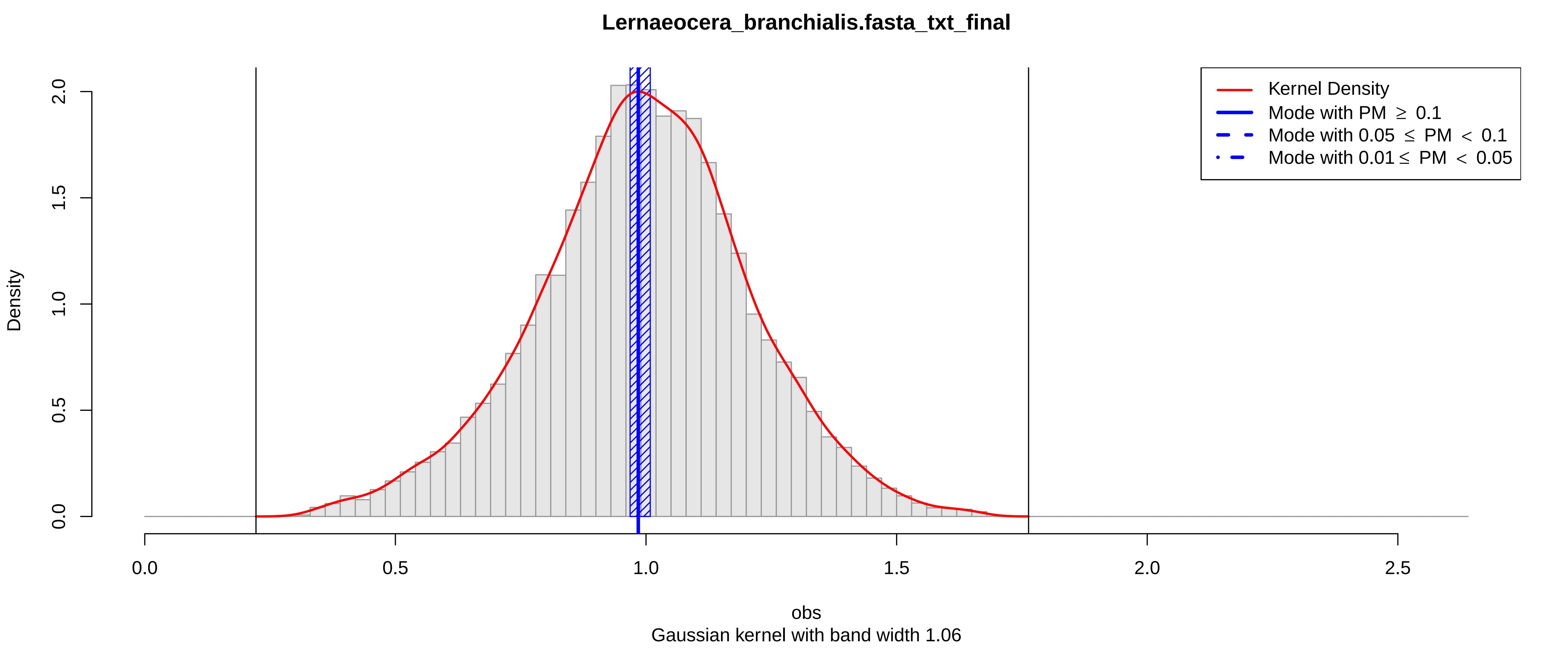

Lernaeocera_branchialis.fasta_txt_final
Kernel Density
Mode with PM ≥ 0.1
Mode with 0.05 ≤ PM < 0.1
Mode with 0.01 ≤ PM < 0.05
Density
0.0
0.5
1.0
1.5
2.0
2.5
obs
Gaussian kernel with band width 1.06

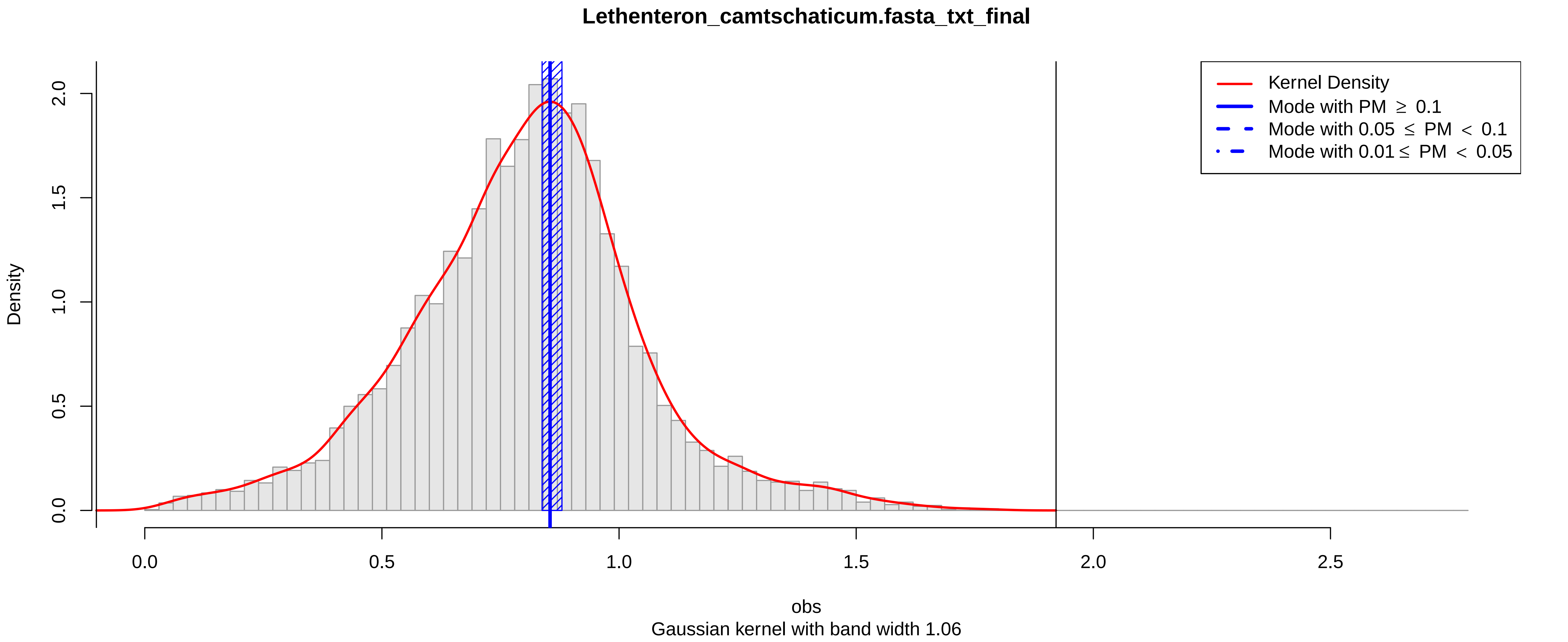
Lethenteron_camtschaticum.fasta_txt_final
Kernel Density
Mode with PM ≥ 0.1
Mode with 0.05 ≤ PM < 0.1
Mode with 0.01 ≤ PM < 0.05
Density
0.0
0.5
1.0
1.5
2.0
0.0
0.5
1.0
1.5
2.0
2.5
obs
Gaussian kernel with band width 1.06

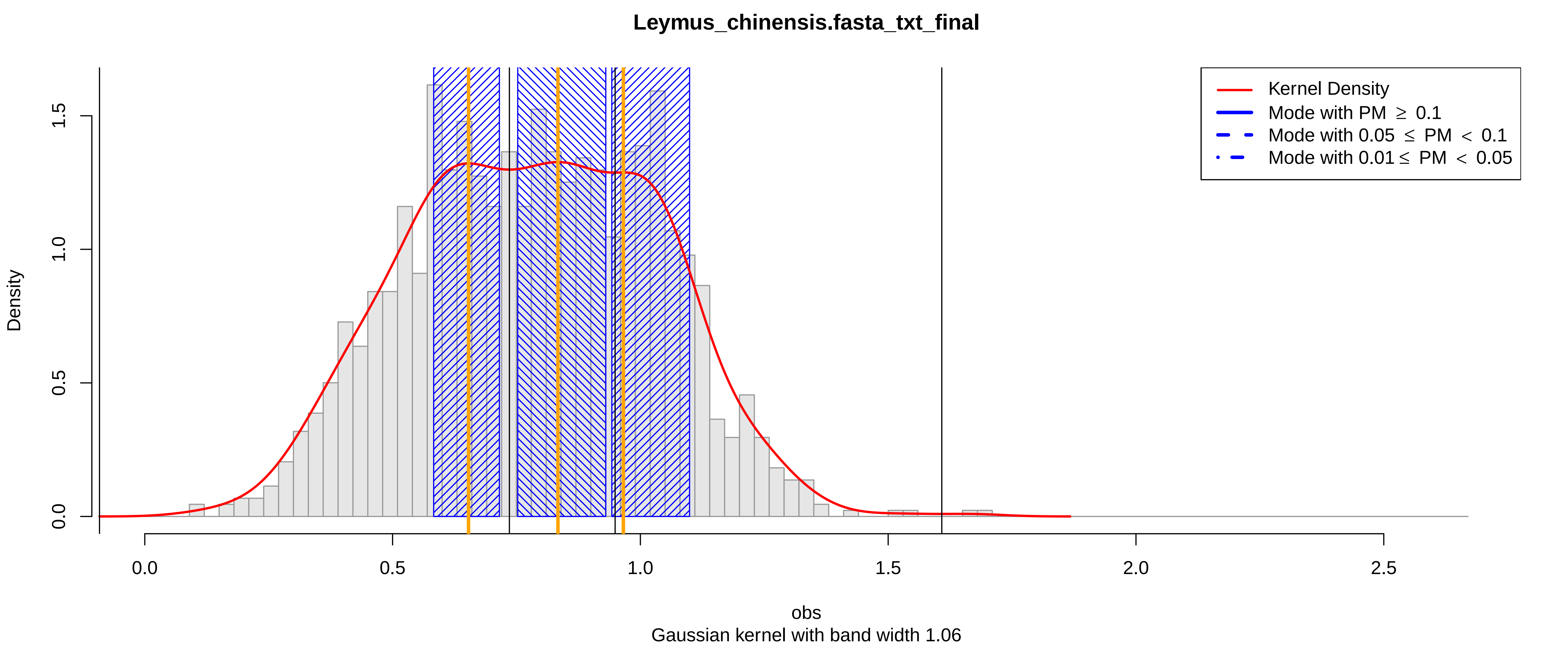

Leymus_chinensis.fasta_txt_final
Kernel Density
Mode with PM ≥ 0.1
Mode with 0.05 ≤ PM < 0.1
Mode with 0.01 ≤ PM < 0.05
Density
0.0
0.5
1.0
1.5
2.0
2.5
obs
Gaussian kernel with band width 1.06

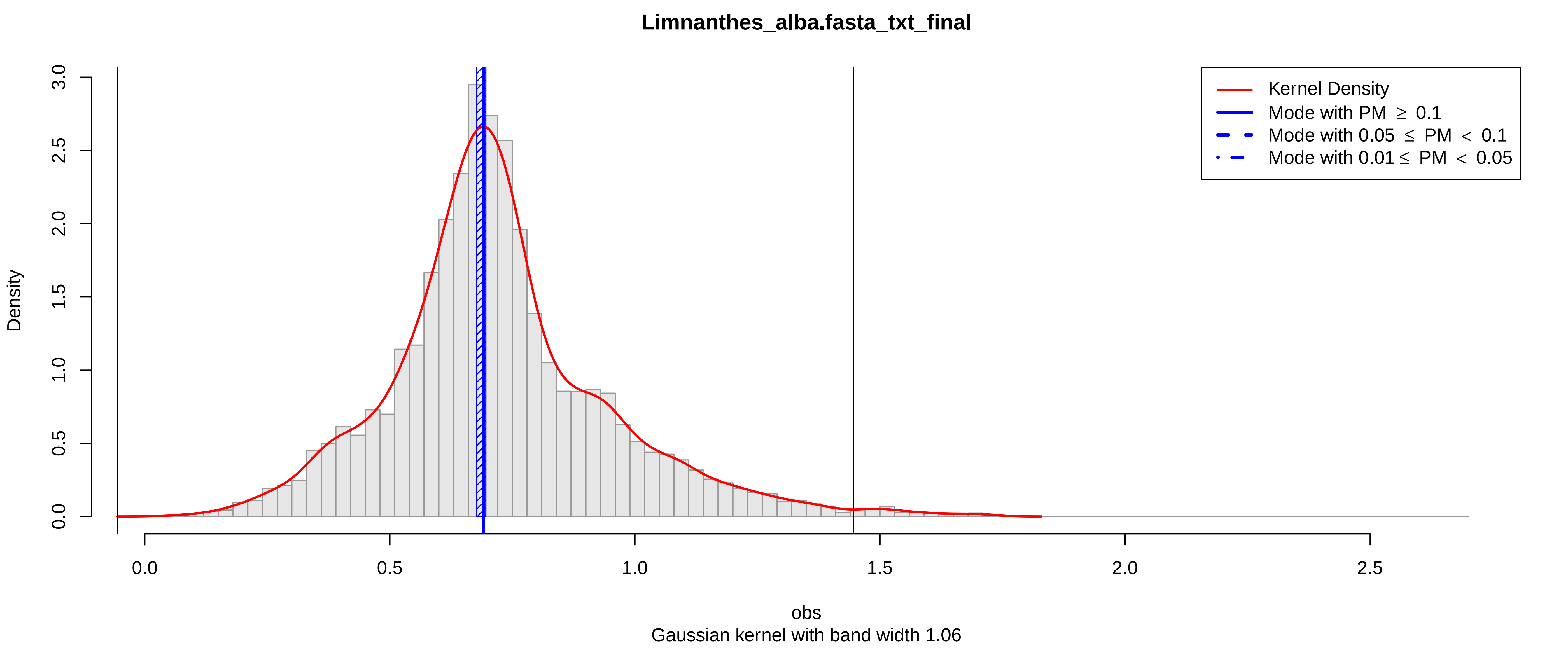
Limnanthes_alba.fasta_txt_final
Kernel Density
Mode with PM ≥ 0.1
Mode with 0.05 ≤ PM < 0.1
Mode with 0.01 ≤ PM < 0.05
Density
0.0
0.5
1.0
1.5
2.0
2.5
3.0
obs
Gaussian kernel with band width 1.06

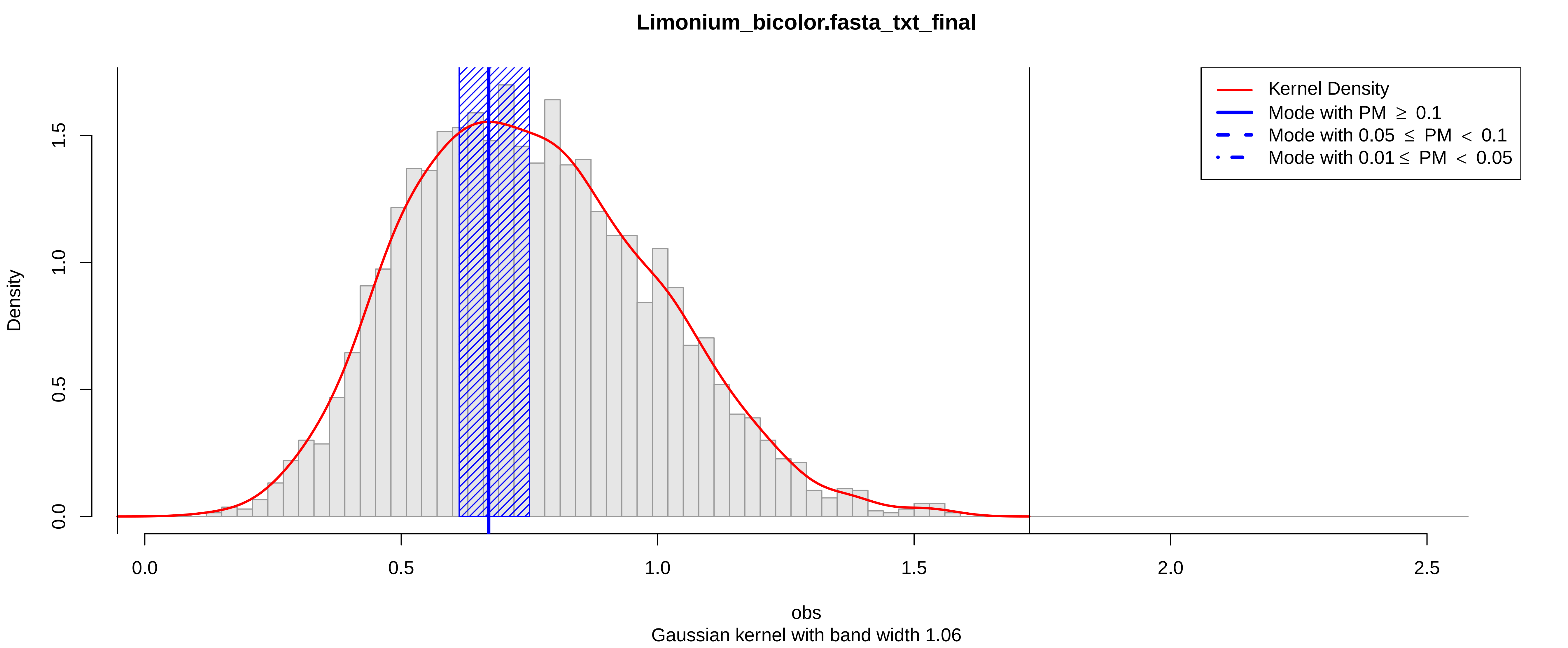

Limonium_bicolor.fasta_txt_final
Kernel Density
Mode with PM ≥ 0.1
Mode with 0.05 ≤ PM < 0.1
Mode with 0.01 ≤ PM < 0.05
Density
0.0
0.5
1.0
1.5
2.0
2.5
obs
Gaussian kernel with band width 1.06

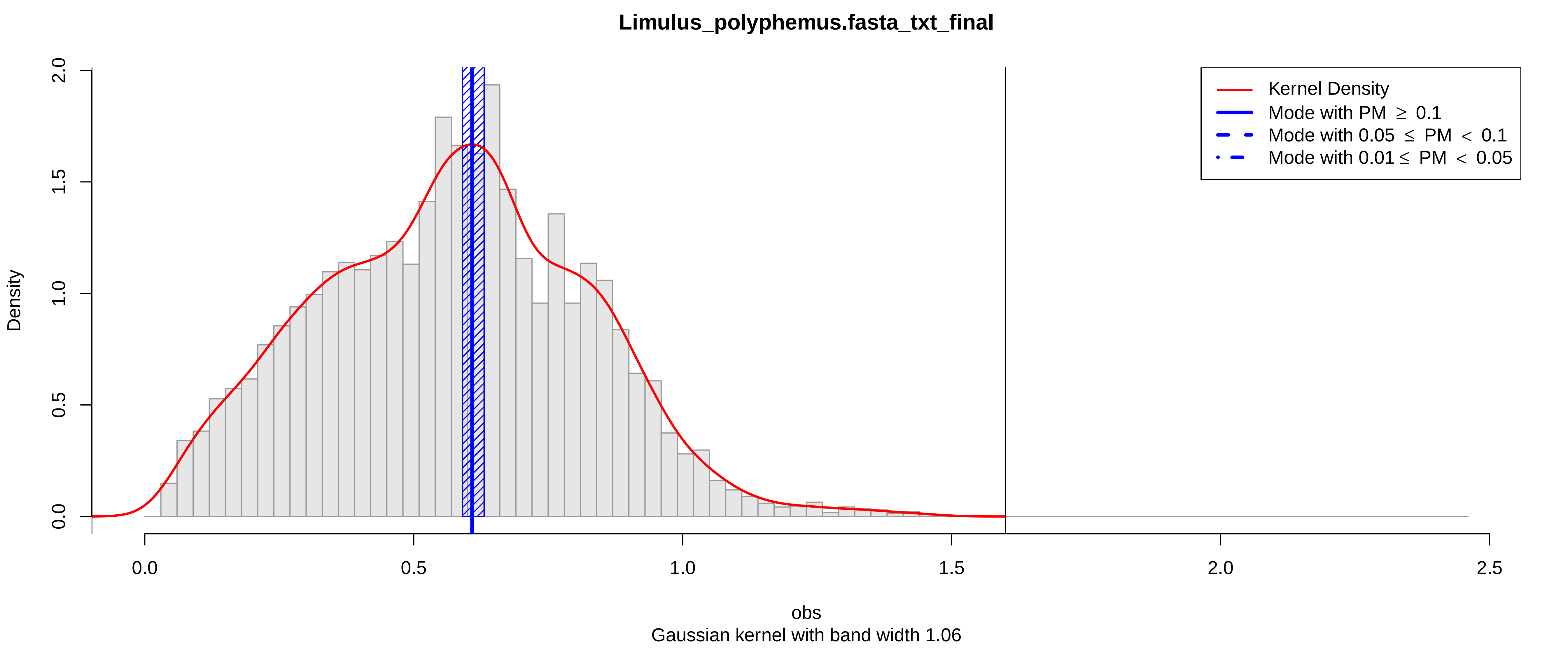

Limulus_polyphemus.fasta_txt_final
Density
obs
Gaussian kernel with band width 1.06
Kernel Density
Mode with PM ≥ 0.1
Mode with 0.05 ≤ PM < 0.1
Mode with 0.01 ≤ PM < 0.05
0.0
0.5
1.0
1.5
2.0
2.5

Lithognathus_mormyrus.fasta_txt_final

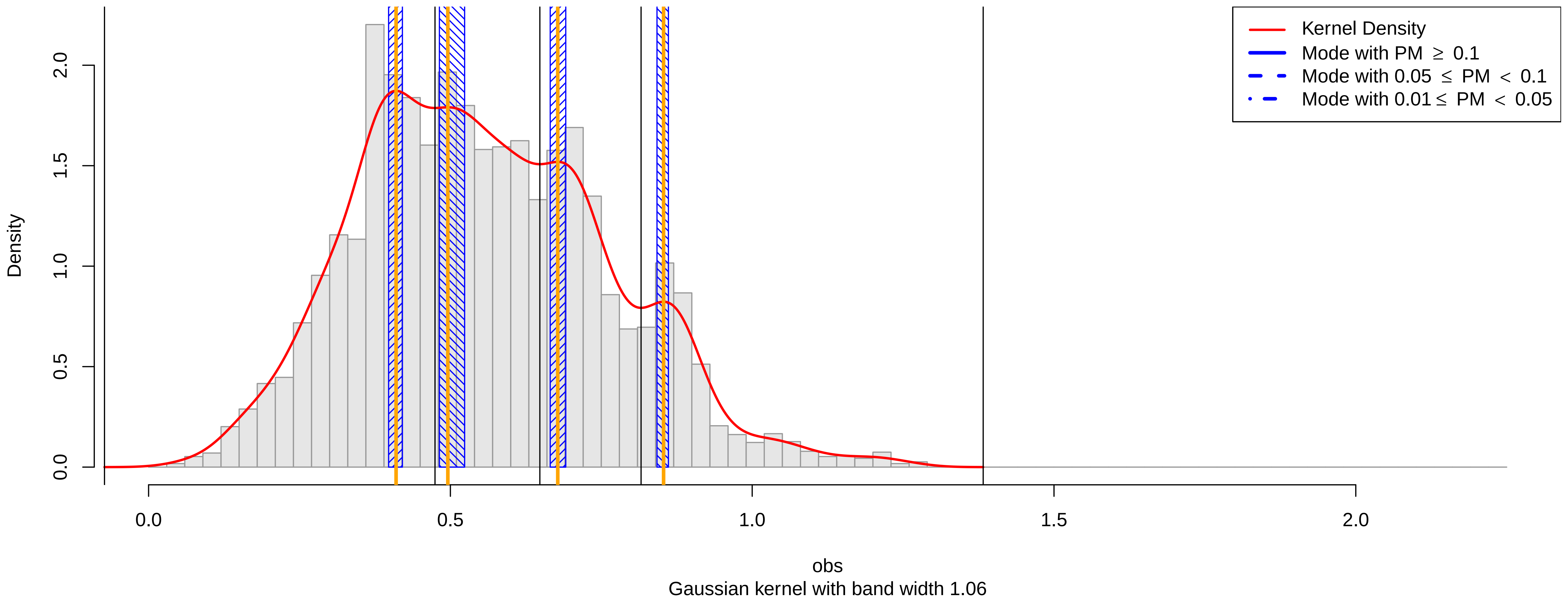

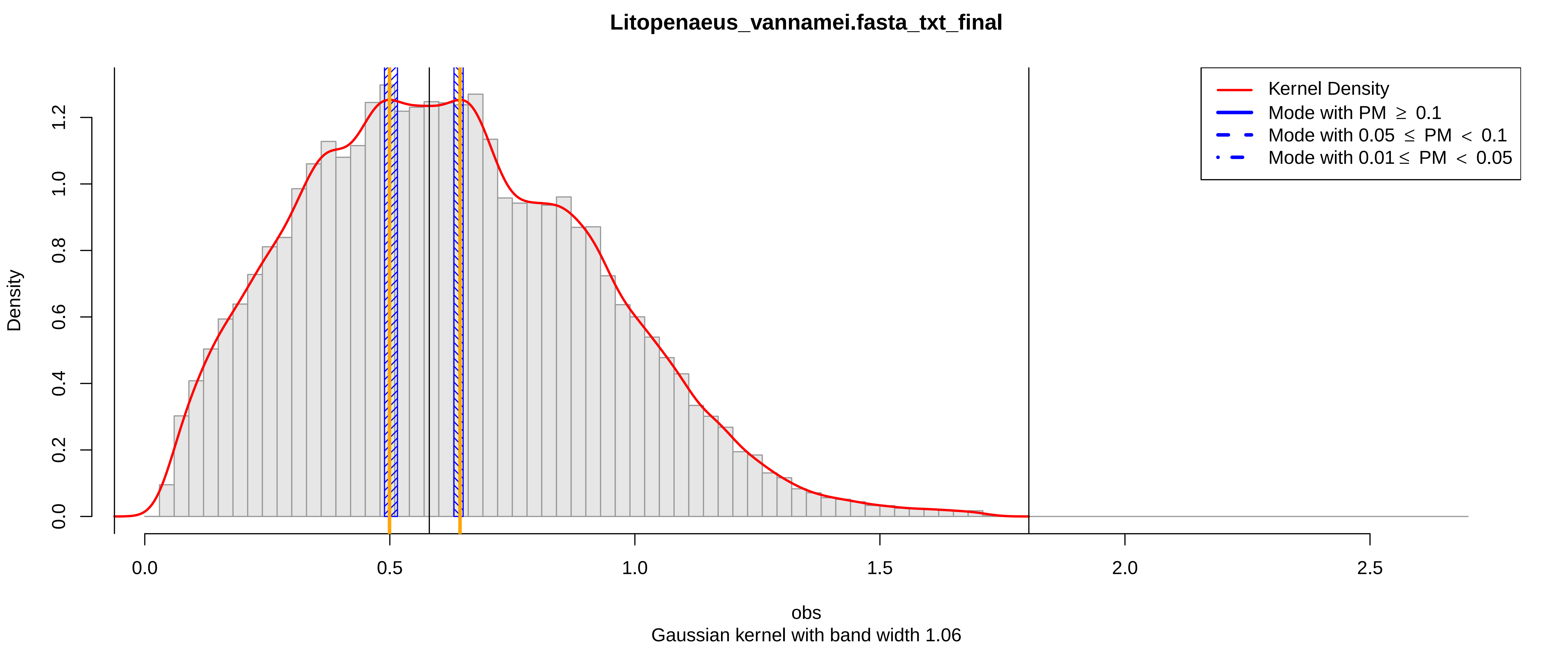

Litopenaeus_vannamei.fasta_txt_final
Kernel Density
Mode with PM ≥ 0.1
Mode with 0.05 ≤ PM < 0.1
Mode with 0.01 ≤ PM < 0.05
Density
0.0
0.2
0.4
0.6
0.8
1.0
1.2
0.0
0.5
1.0
1.5
2.0
2.5
obs
Gaussian kernel with band width 1.06

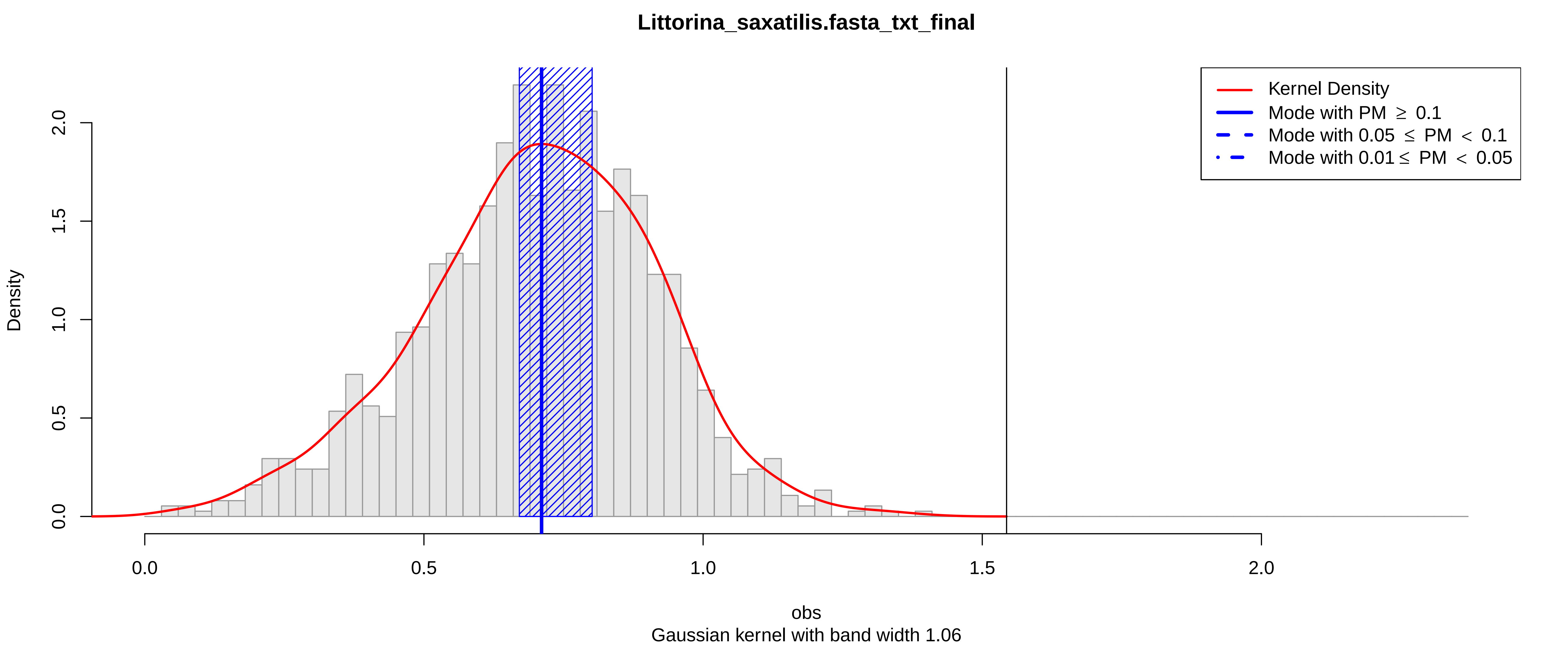
Littorina_saxatilis.fasta_txt_final
Kernel Density
Mode with PM ≥ 0.1
Mode with 0.05 ≤ PM < 0.1
Mode with 0.01 ≤ PM < 0.05
Density
0.0
0.5
1.0
1.5
2.0
obs
Gaussian kernel with band width 1.06

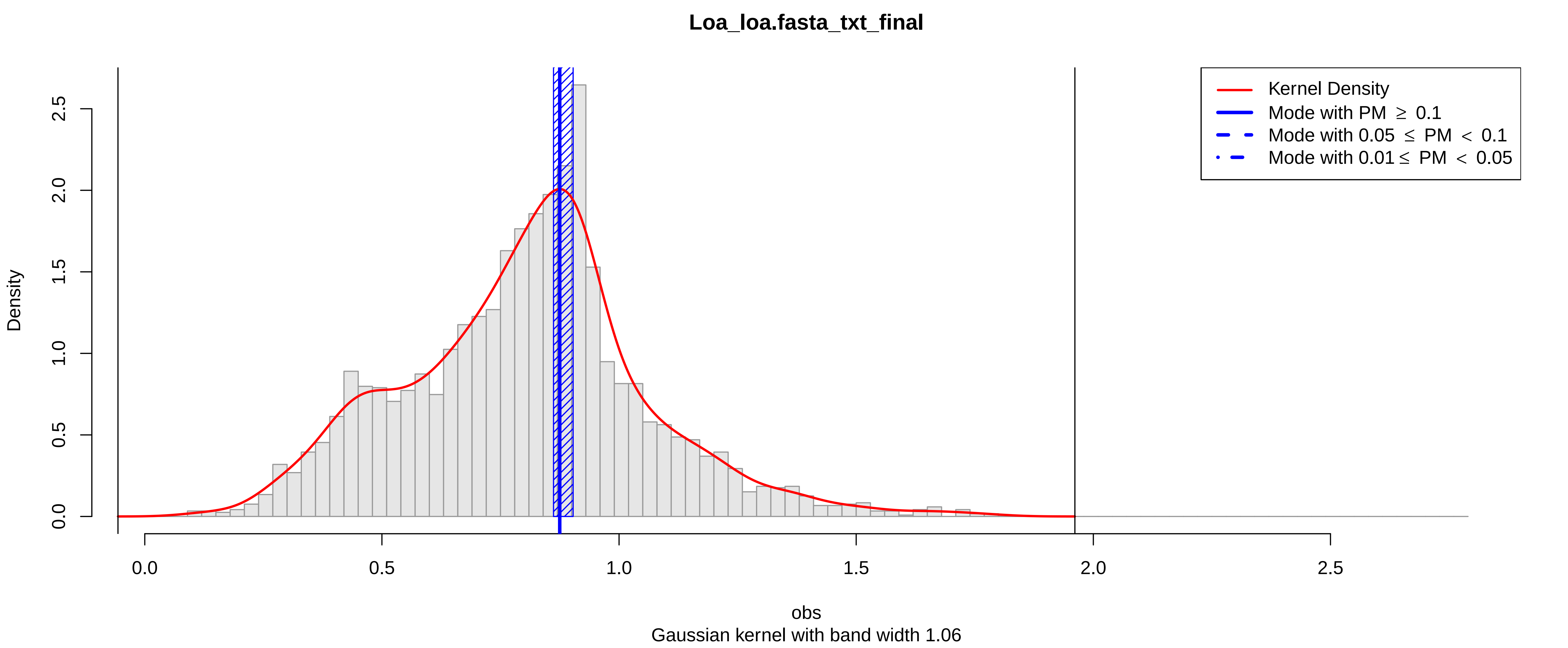

Loa_loa.fasta_txt_final
Kernel Density
Mode with PM ≥ 0.1
Mode with 0.05 ≤ PM < 0.1
Mode with 0.01 ≤ PM < 0.05
Density
0.0
0.5
1.0
1.5
2.0
2.5
obs
Gaussian kernel with band width 1.06

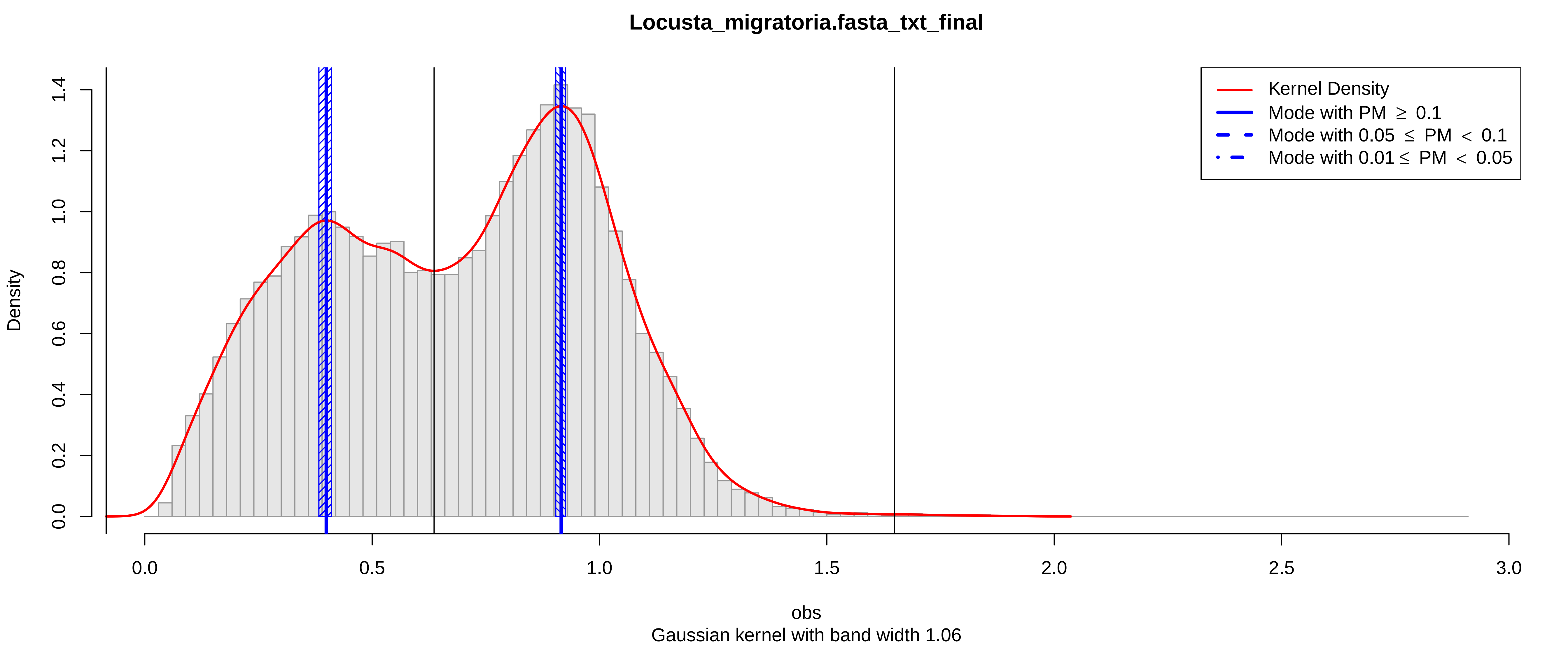

Locusta_migratoria.fasta_txt_final
Kernel Density
Mode with PM ≥ 0.1
Mode with 0.05 ≤ PM < 0.1
Mode with 0.01 ≤ PM < 0.05
Density
0.0
0.2
0.4
0.6
0.8
1.0
1.2
1.4
0.0
0.5
1.0
1.5
2.0
2.5
3.0
obs
Gaussian kernel with band width 1.06

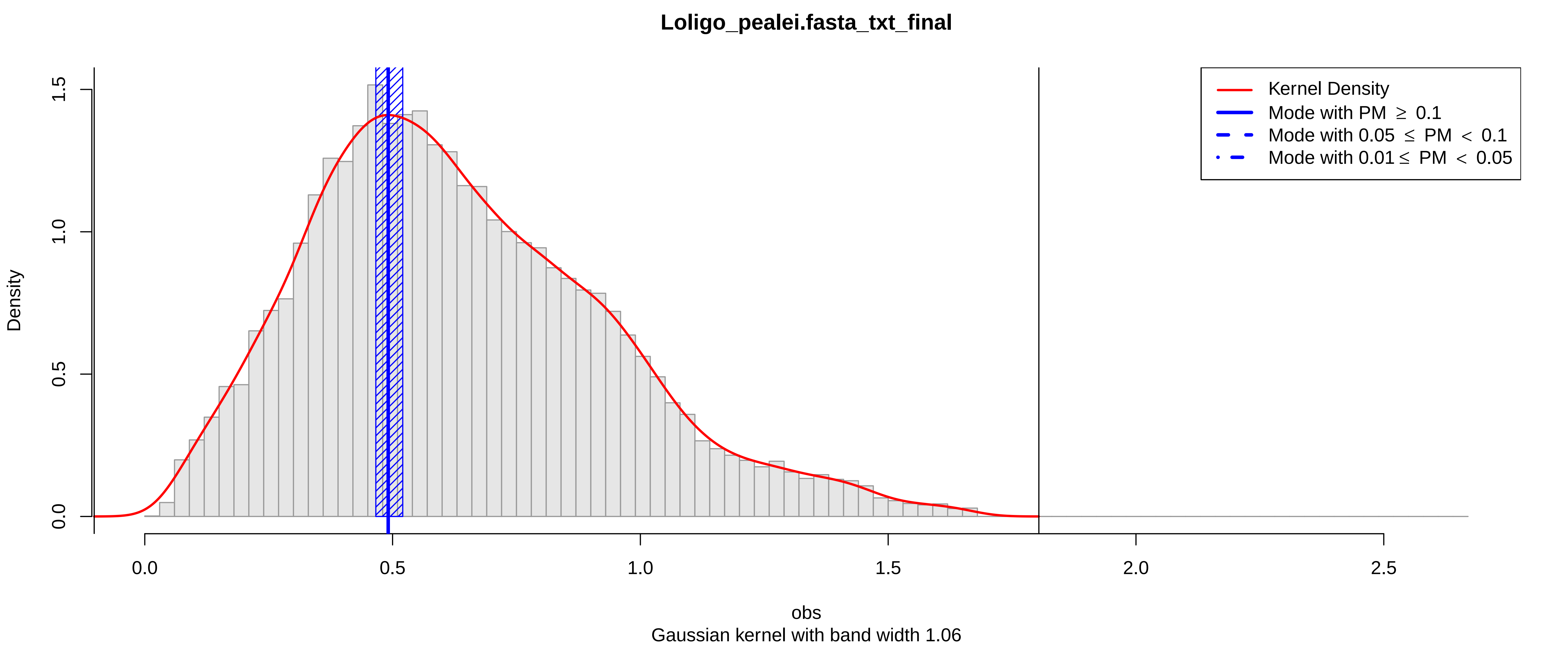

Loligo_pealei.fasta_txt_final
Density
0.0
0.5
1.0
1.5
0.0
0.5
1.0
1.5
2.0
2.5
Kernel Density
Mode with PM ≥ 0.1
Mode with 0.05 ≤ PM < 0.1
Mode with 0.01 ≤ PM < 0.05
obs
Gaussian kernel with band width 1.06

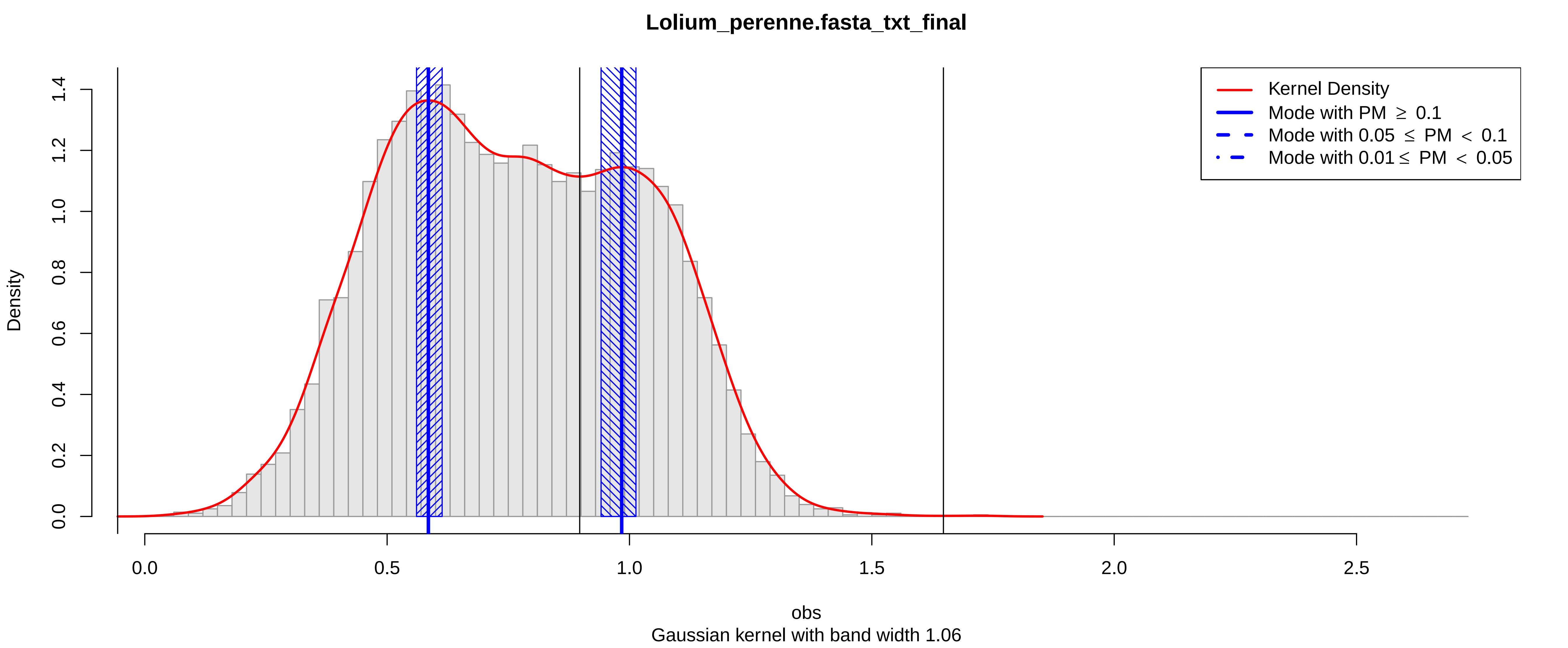

Lolium_perenne.fasta_txt_final
Kernel Density
Mode with PM ≥ 0.1
Mode with 0.05 ≤ PM < 0.1
Mode with 0.01 ≤ PM < 0.05
Density
0.0
0.2
0.4
0.6
0.8
1.0
1.2
1.4
0.0
0.5
1.0
1.5
2.0
2.5
obs
Gaussian kernel with band width 1.06

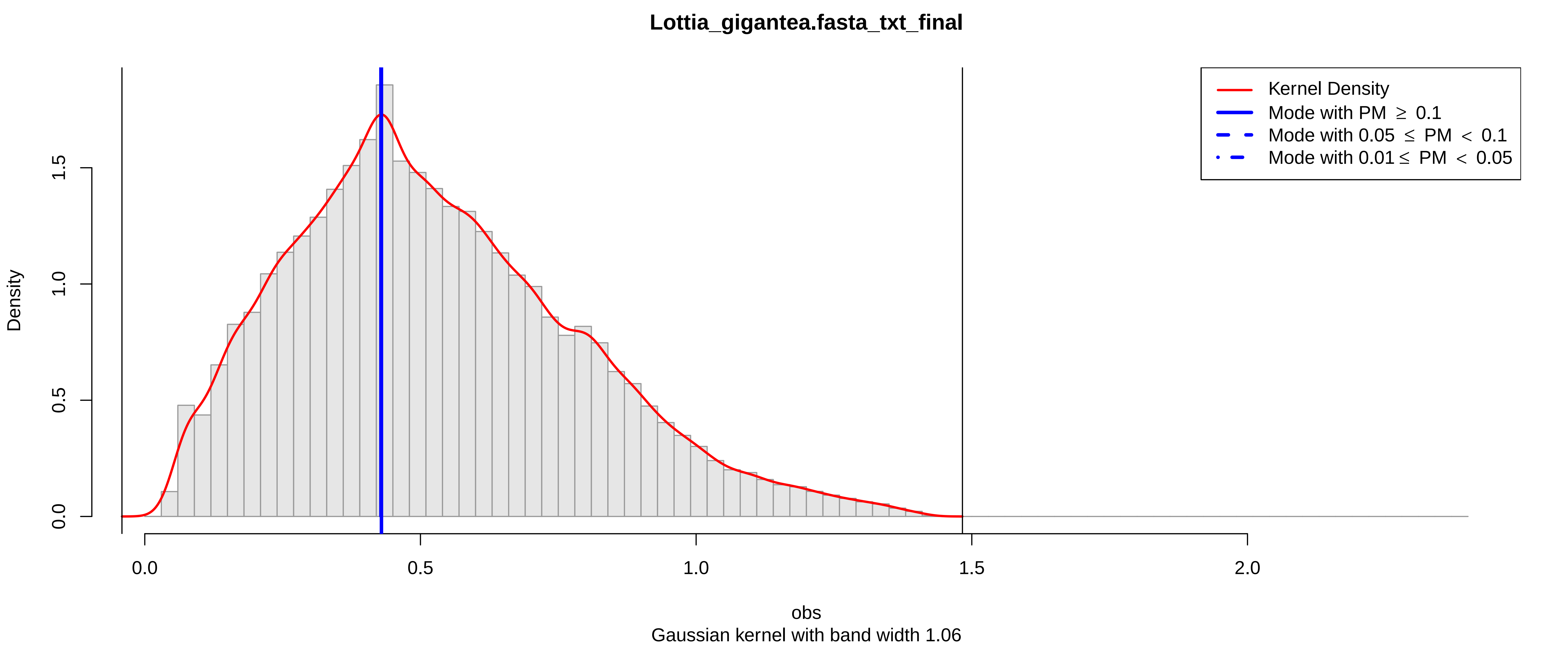

Lottia_gigantea.fasta_txt_final
Kernel Density
Mode with PM ≥ 0.1
Mode with 0.05 ≤ PM < 0.1
Mode with 0.01 ≤ PM < 0.05
Density
0.0
0.5
1.0
1.5
0.0
0.5
1.0
1.5
2.0
obs
Gaussian kernel with band width 1.06

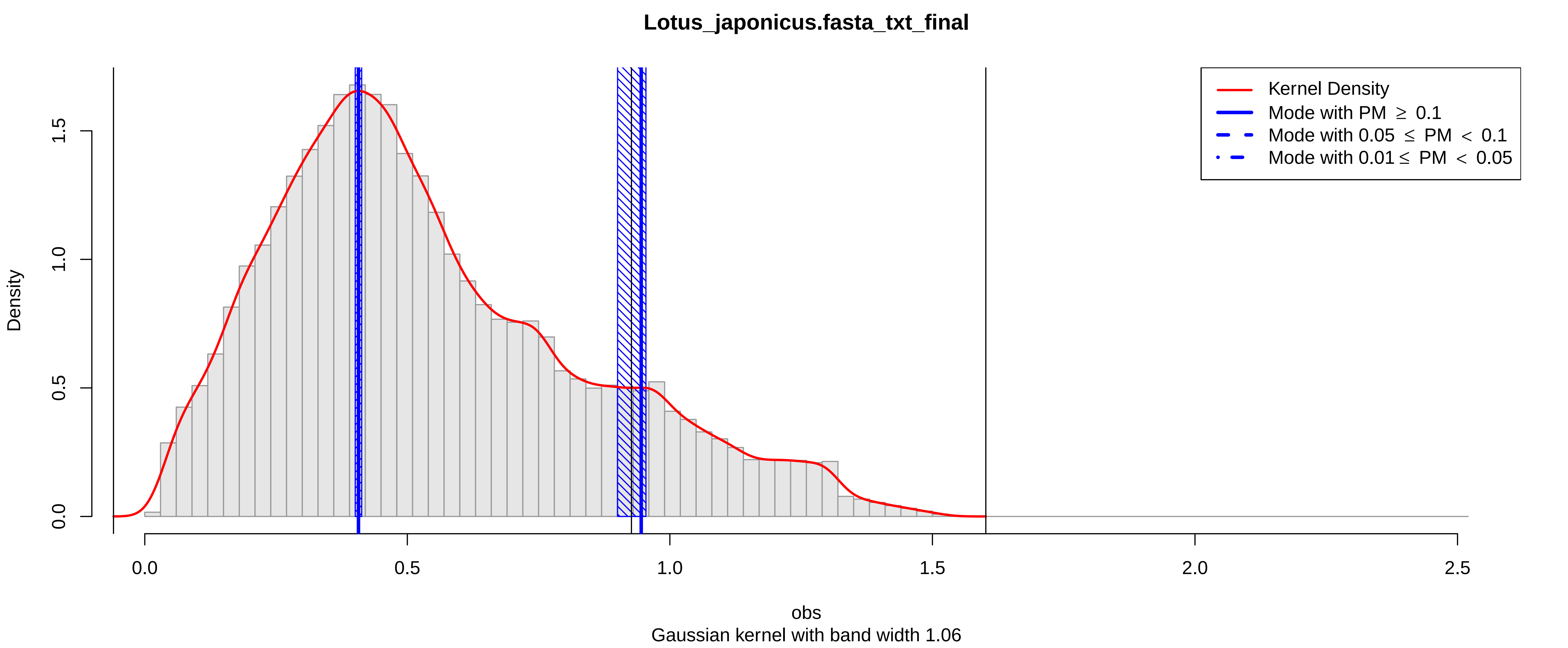

Lotus_japonicus.fasta_txt_final
Kernel Density
Mode with PM ≥ 0.1
Mode with 0.05 ≤ PM < 0.1
Mode with 0.01 ≤ PM < 0.05
Density
0.0
0.5
1.0
1.5
2.0
2.5
obs
Gaussian kernel with band width 1.06

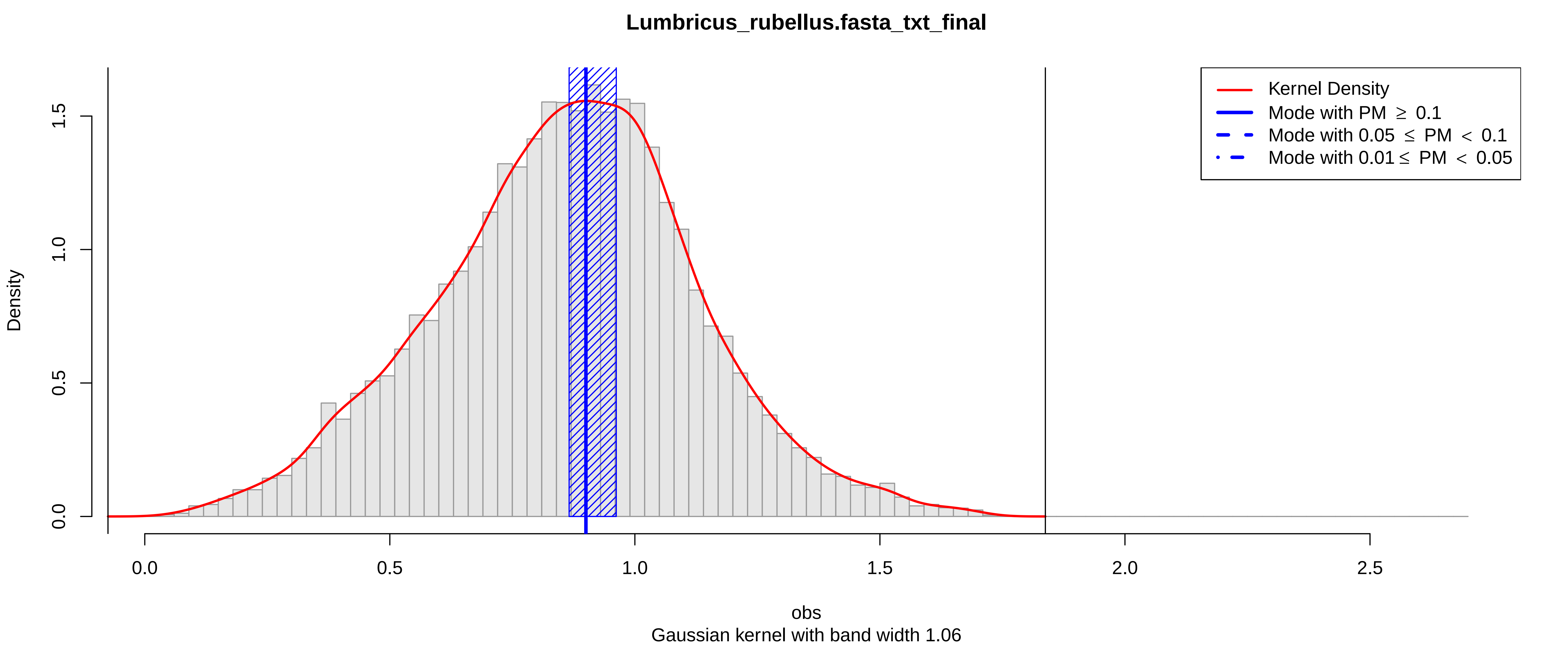
Lumbricus_rubellus.fasta_txt_final
Kernel Density
Mode with PM ≥ 0.1
Mode with 0.05 ≤ PM < 0.1
Mode with 0.01 ≤ PM < 0.05
Density
0.0
0.5
1.0
1.5
0.0
0.5
1.0
1.5
2.0
2.5
obs
Gaussian kernel with band width 1.06

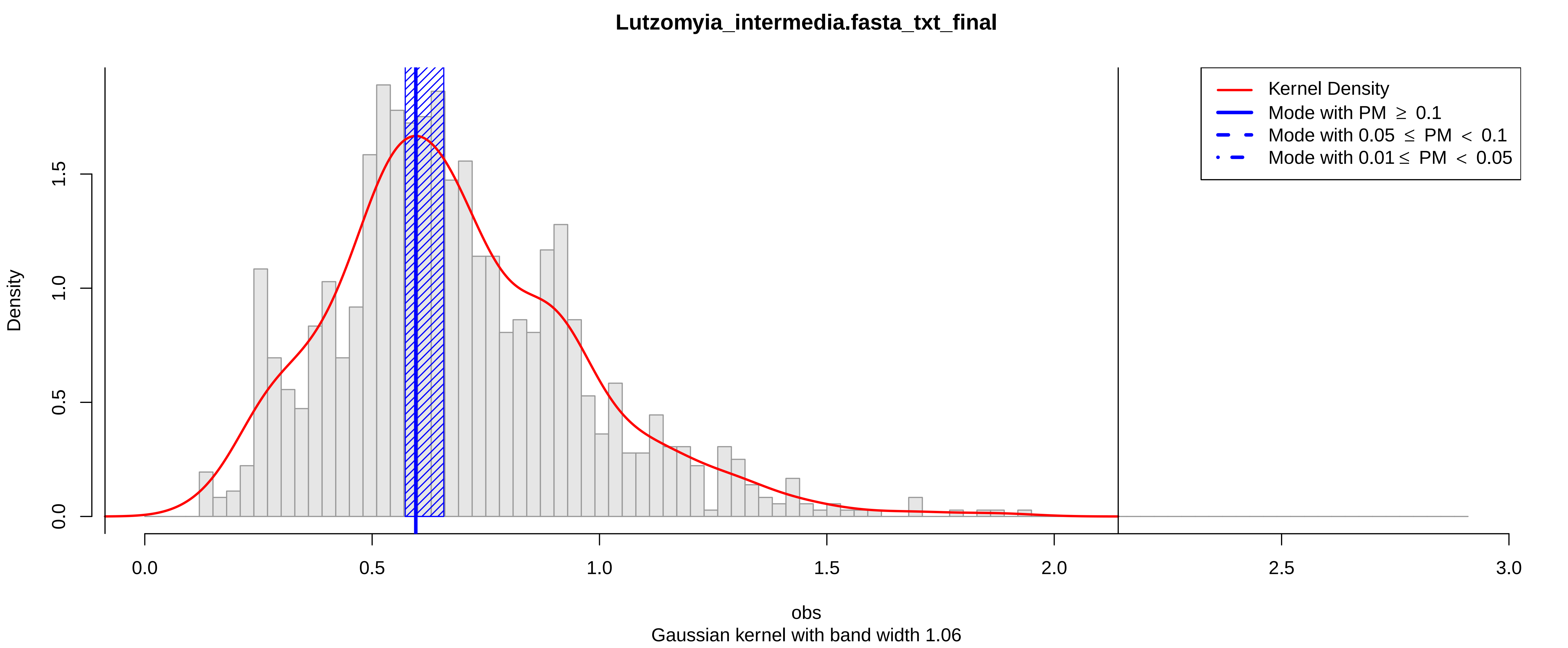
Lutzomyia_intermedia.fasta_txt_final
Kernel Density
Mode with PM ≥ 0.1
Mode with 0.05 ≤ PM < 0.1
Mode with 0.01 ≤ PM < 0.05
Density
obs
Gaussian kernel with band width 1.06
0.0
0.5
1.0
1.5
2.0
2.5
3.0

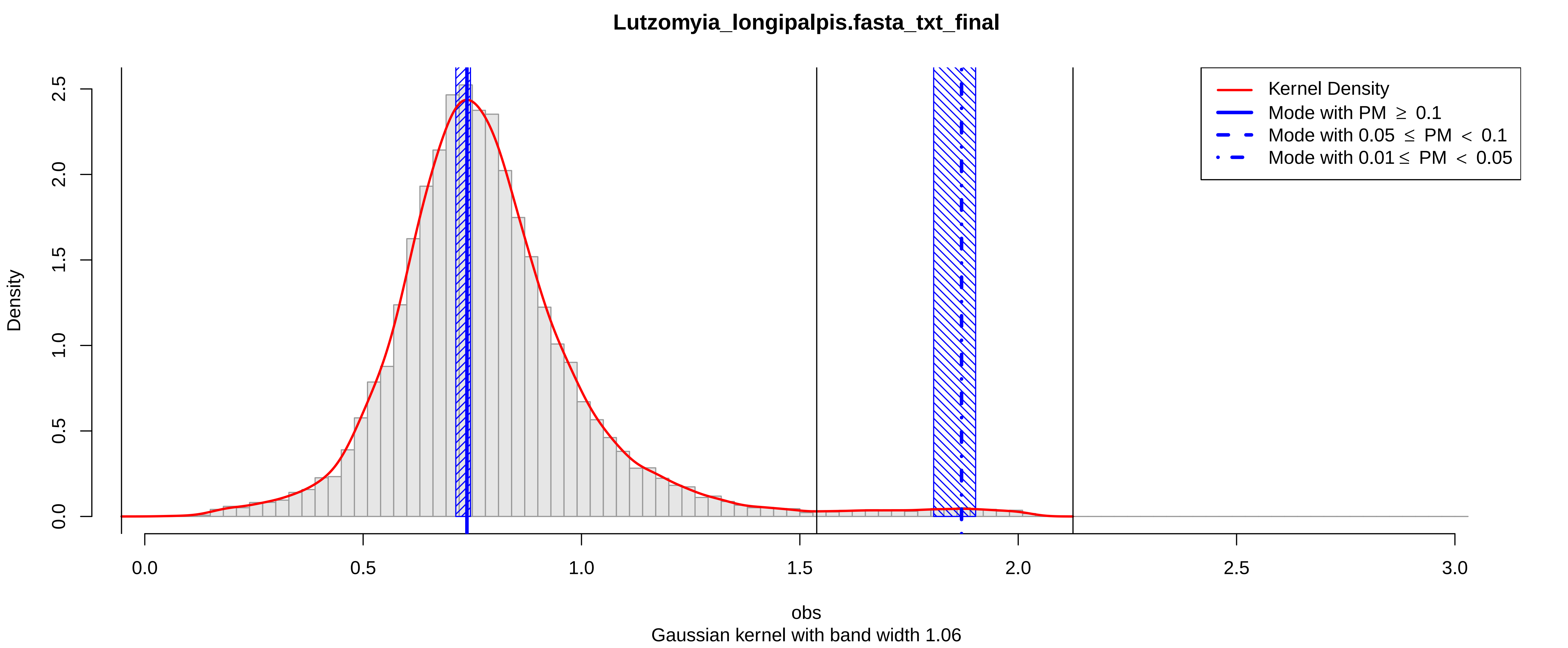

Lutzomyia_longipalpis.fasta_txt_final
Kernel Density
Mode with PM ≥ 0.1
Mode with 0.05 ≤ PM < 0.1
Mode with 0.01 ≤ PM < 0.05
Density
0.0
0.5
1.0
1.5
2.0
2.5
3.0
obs
Gaussian kernel with band width 1.06

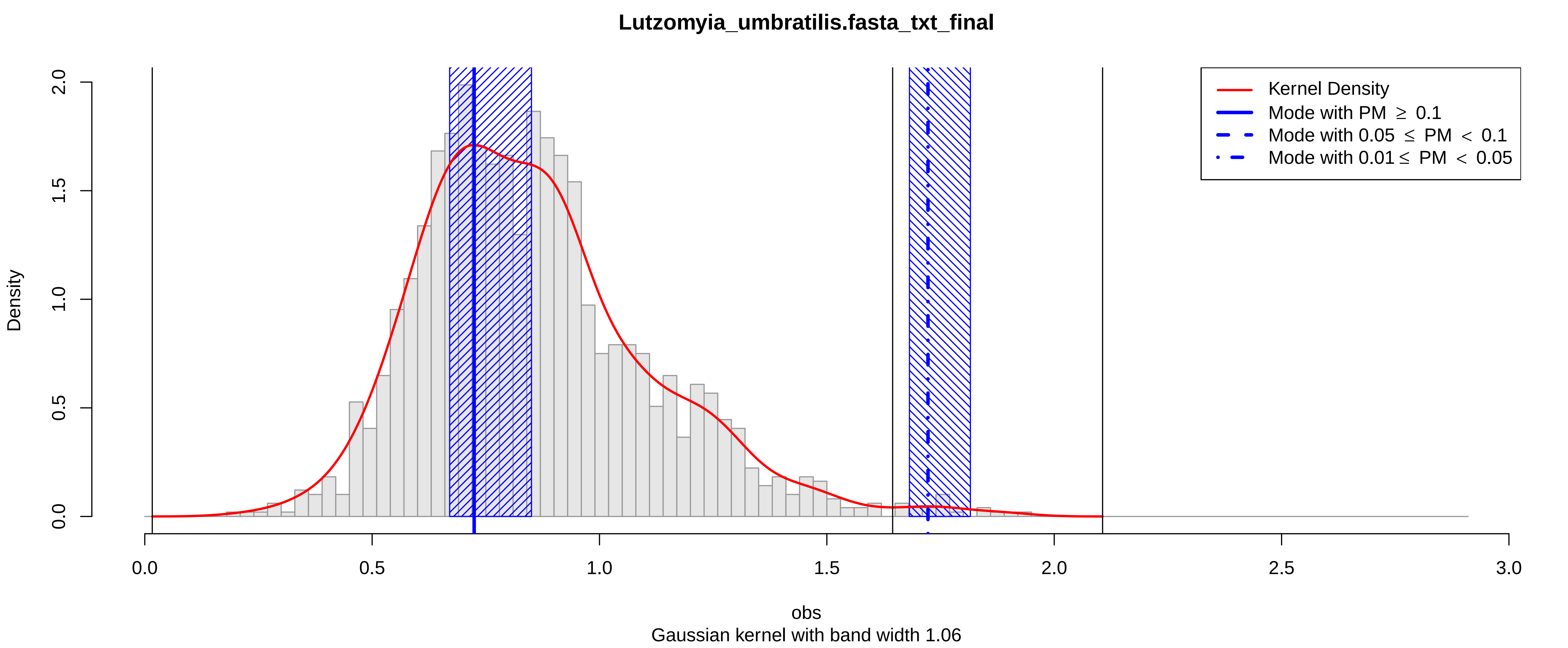

Lutzomyia_umbratilis.fasta_txt_final
Kernel Density
Mode with PM ≥ 0.1
Mode with 0.05 ≤ PM < 0.1
Mode with 0.01 ≤ PM < 0.05
Density
0.0
0.5
1.0
1.5
2.0
2.5
3.0
obs
Gaussian kernel with band width 1.06

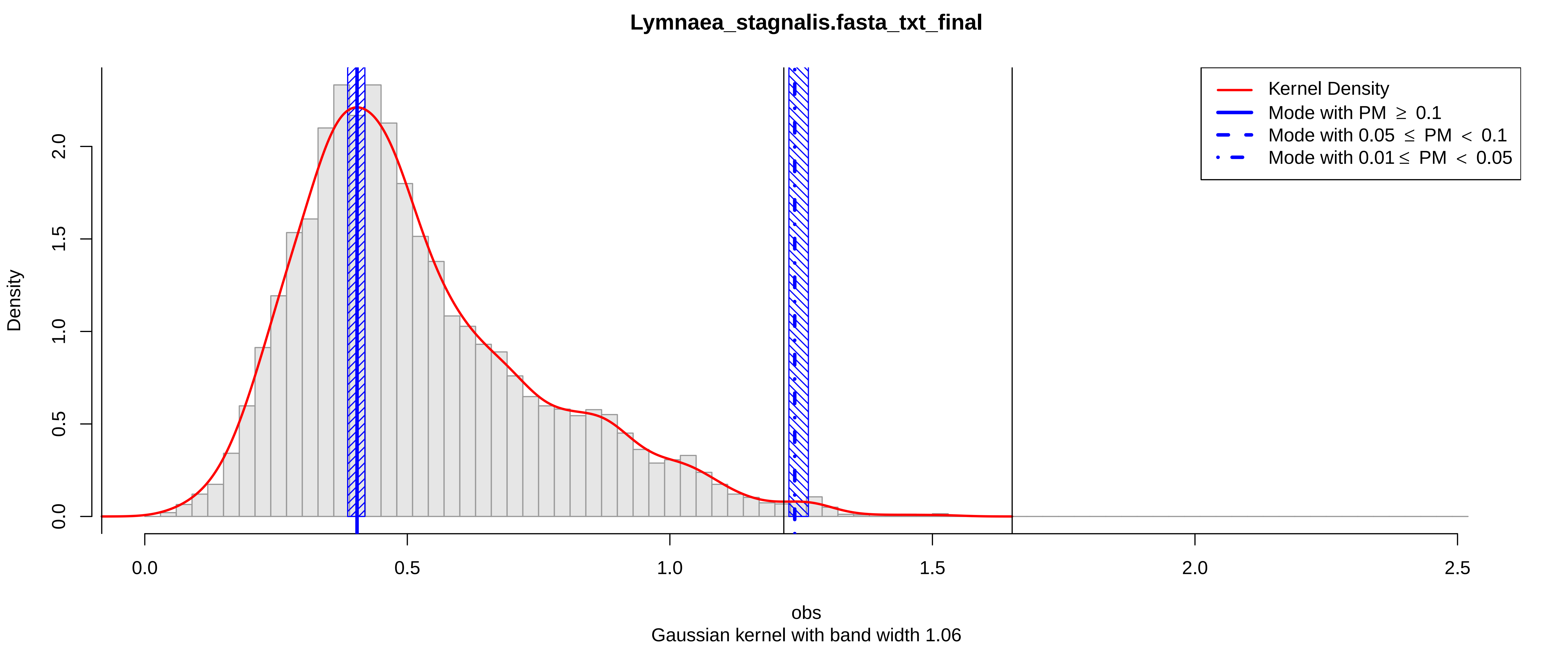

Lymnaea_stagnalis.fasta_txt_final
Kernel Density
Mode with PM ≥ 0.1
Mode with 0.05 ≤ PM < 0.1
Mode with 0.01 ≤ PM < 0.05
Density
0.0
0.5
1.0
1.5
2.0
2.5
obs
Gaussian kernel with band width 1.06

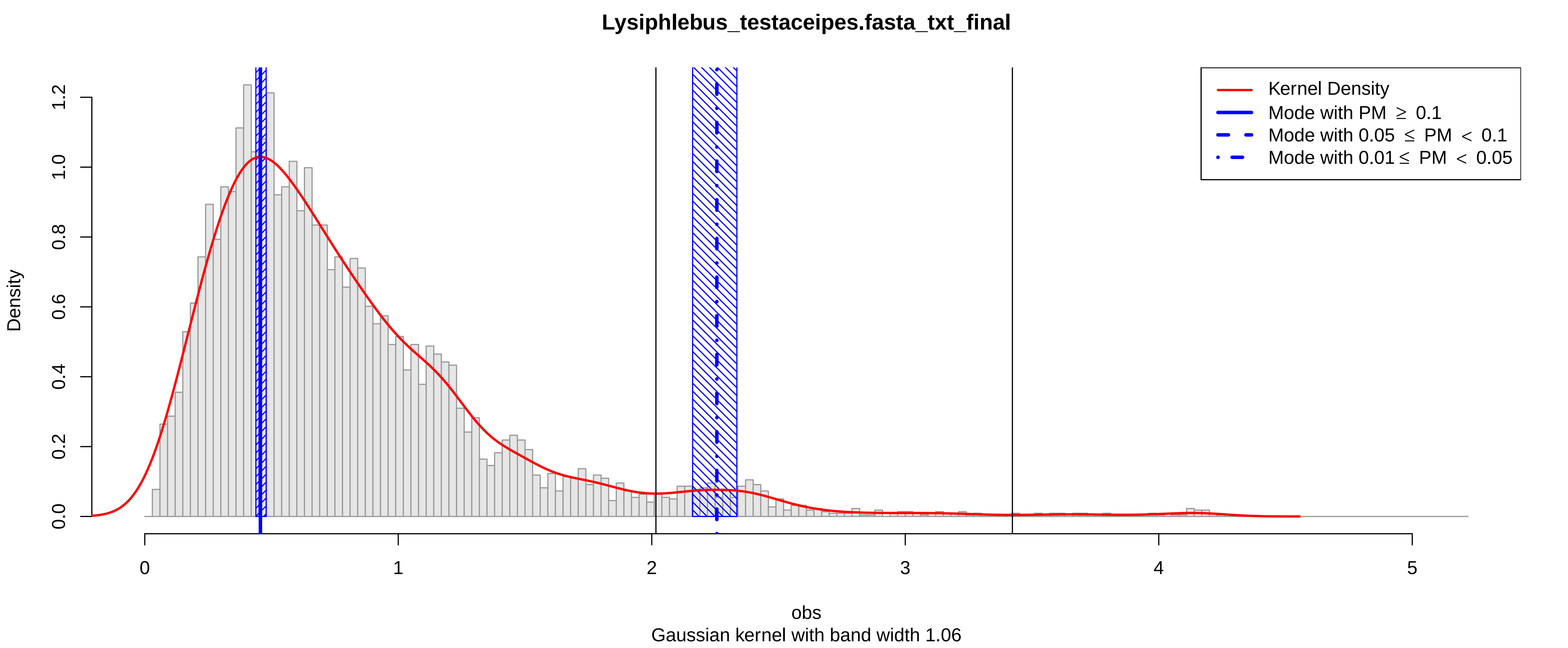

Lysiphlebus_testaceipes.fasta_txt_final
Kernel Density
Mode with PM ≥ 0.1
Mode with 0.05 ≤ PM < 0.1
Mode with 0.01 ≤ PM < 0.05
Density
0.0
0.2
0.4
0.6
0.8
1.0
1.2
0
1
2
3
4
5
obs
Gaussian kernel with band width 1.06

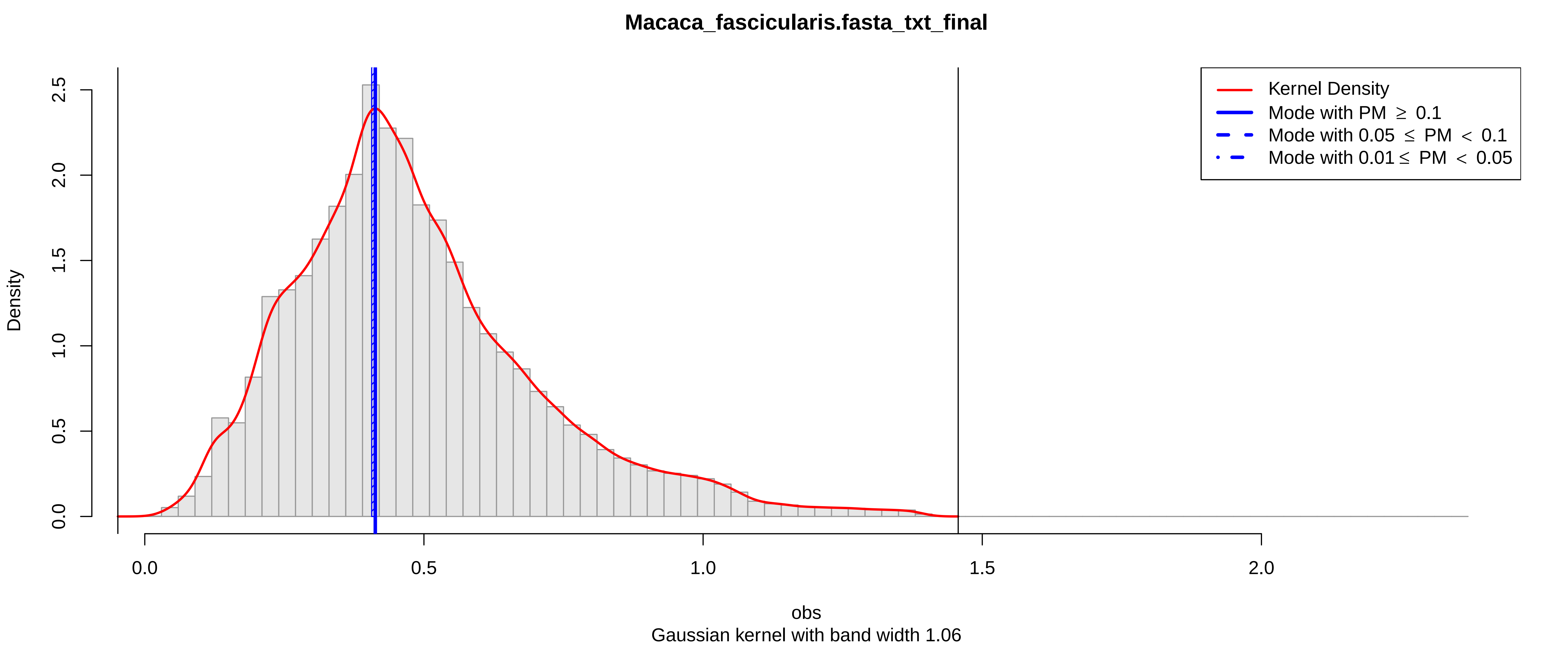
Macaca_fascicularis.fasta_txt_final
Kernel Density
Mode with PM ≥ 0.1
Mode with 0.05 ≤ PM < 0.1
Mode with 0.01 ≤ PM < 0.05
Density
0.0
0.5
1.0
1.5
2.0
2.5
0.0
0.5
1.0
1.5
2.0
obs
Gaussian kernel with band width 1.06

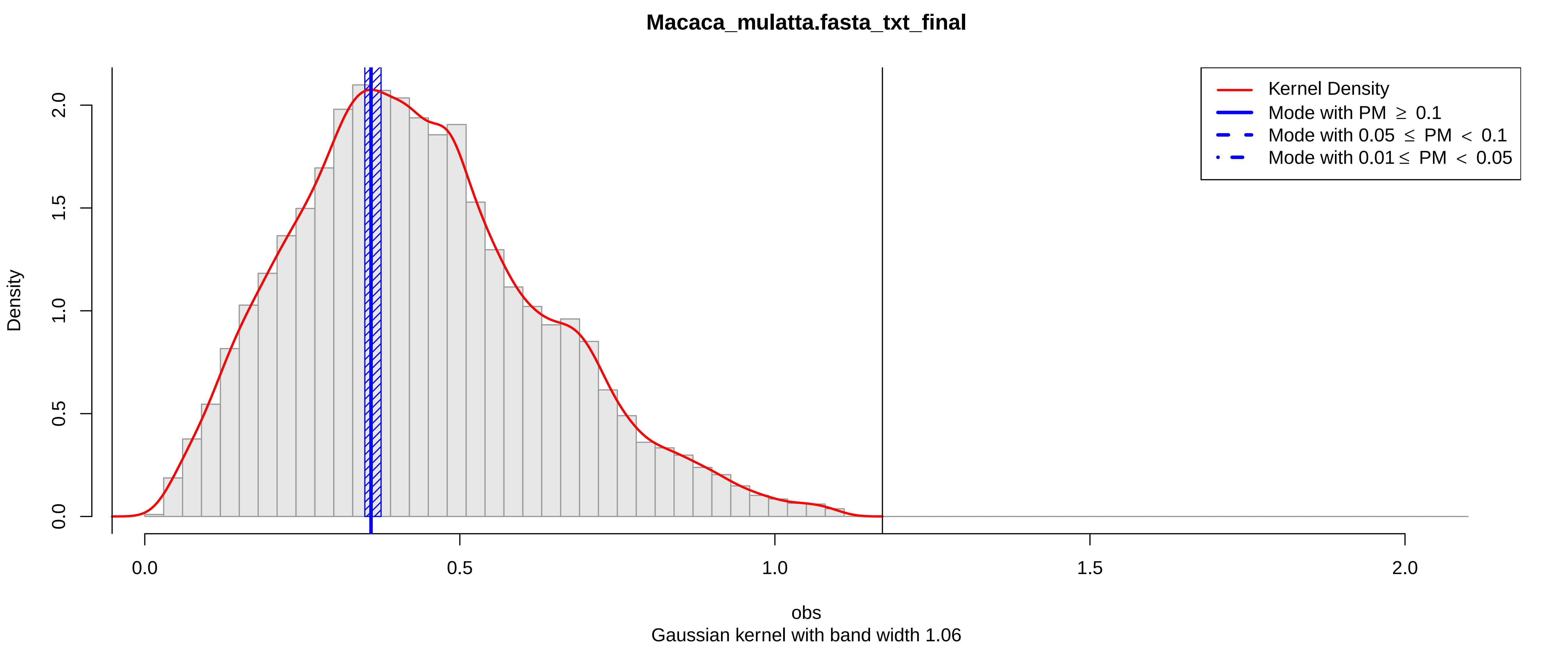

Macaca_mulatta.fasta_txt_final
Kernel Density
Mode with PM ≥ 0.1
Mode with 0.05 ≤ PM < 0.1
Mode with 0.01 ≤ PM < 0.05
Density
0.0
0.5
1.0
1.5
2.0
obs
Gaussian kernel with band width 1.06

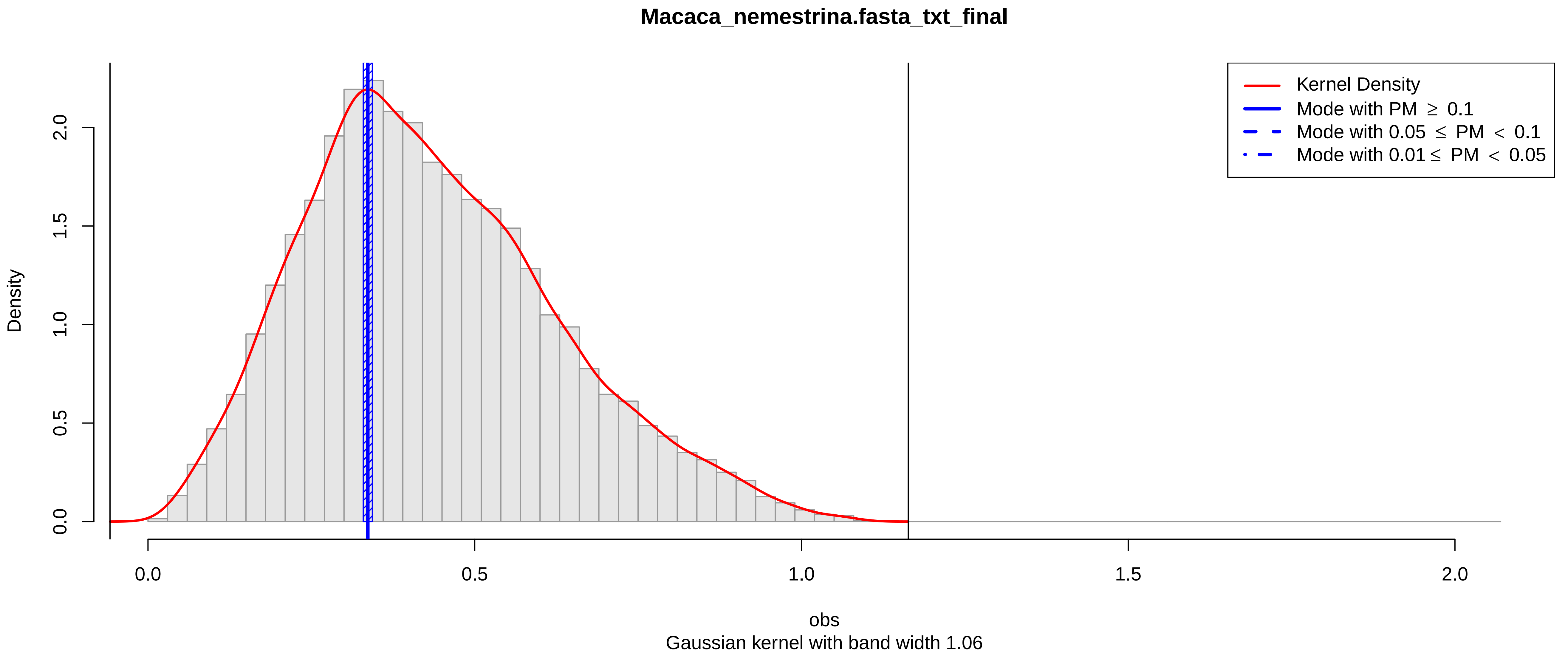

Macaca_nemestrina.fasta_txt_final
Kernel Density
Mode with PM ≥ 0.1
Mode with 0.05 ≤ PM < 0.1
Mode with 0.01 ≤ PM < 0.05
Density
0.0
0.5
1.0
1.5
2.0
obs
Gaussian kernel with band width 1.06

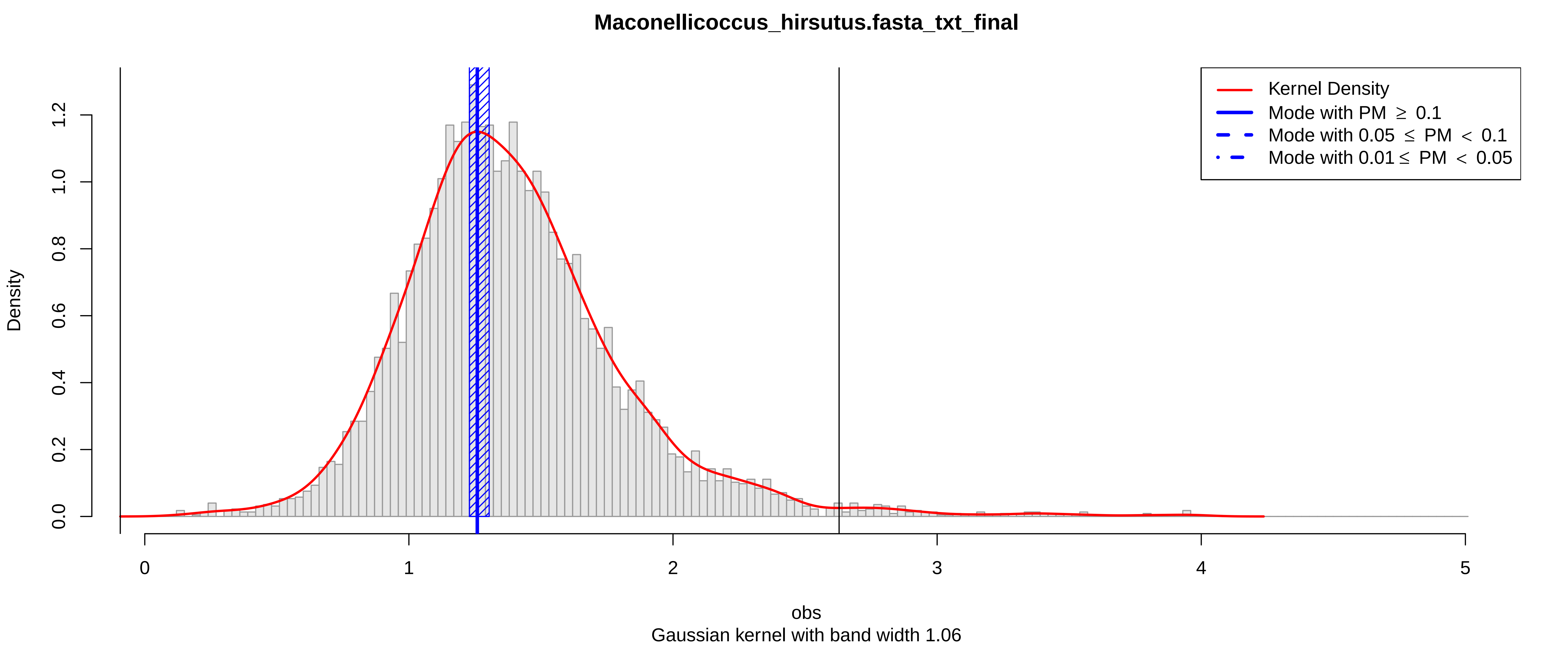

Maconellicoccus_hirsutus.fasta_txt_final
Kernel Density
Mode with PM ≥ 0.1
Mode with 0.05 ≤ PM < 0.1
Mode with 0.01 ≤ PM < 0.05
Density
0.0
0.2
0.4
0.6
0.8
1.0
1.2
0
1
2
3
4
5
obs
Gaussian kernel with band width 1.06

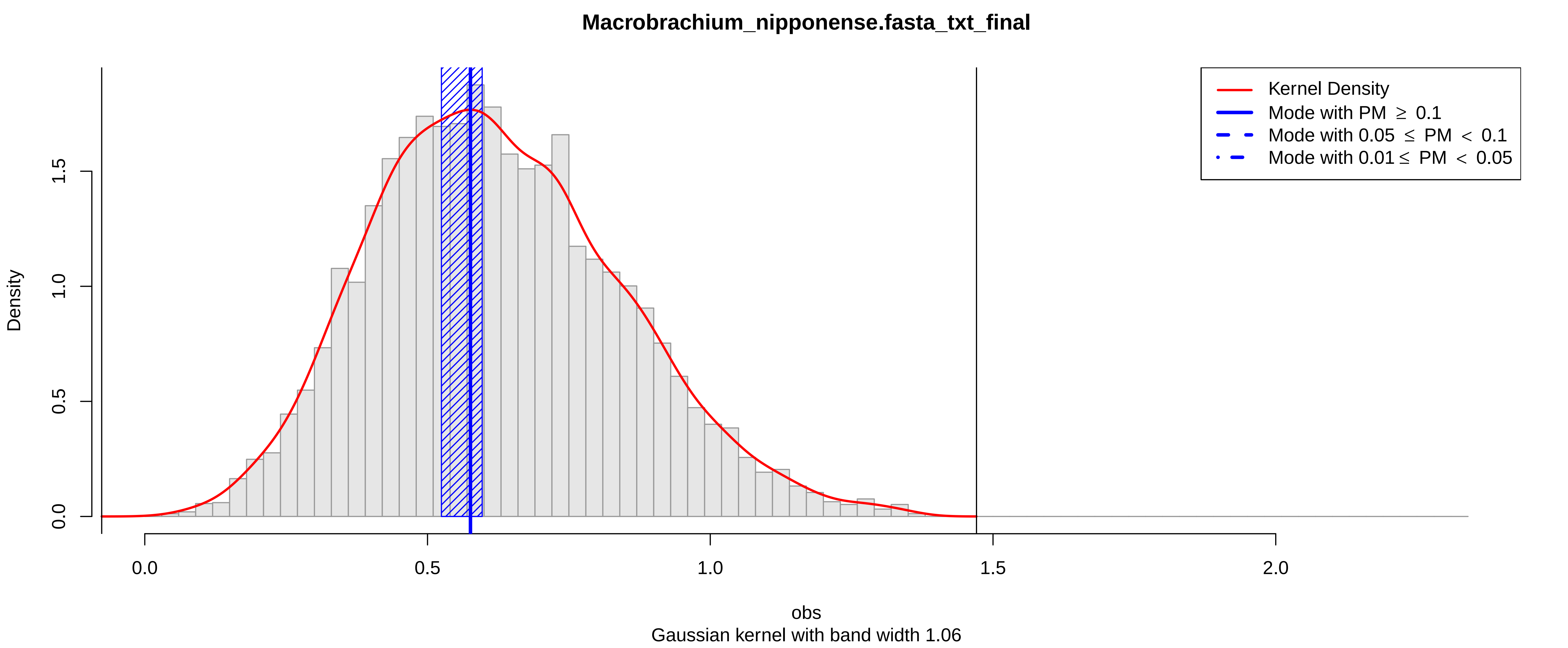

Macrobrachium_nipponense.fasta_txt_final
Kernel Density
Mode with PM ≥ 0.1
Mode with 0.05 ≤ PM < 0.1
Mode with 0.01 ≤ PM < 0.05
Density
0.0
0.5
1.0
1.5
2.0
obs
Gaussian kernel with band width 1.06

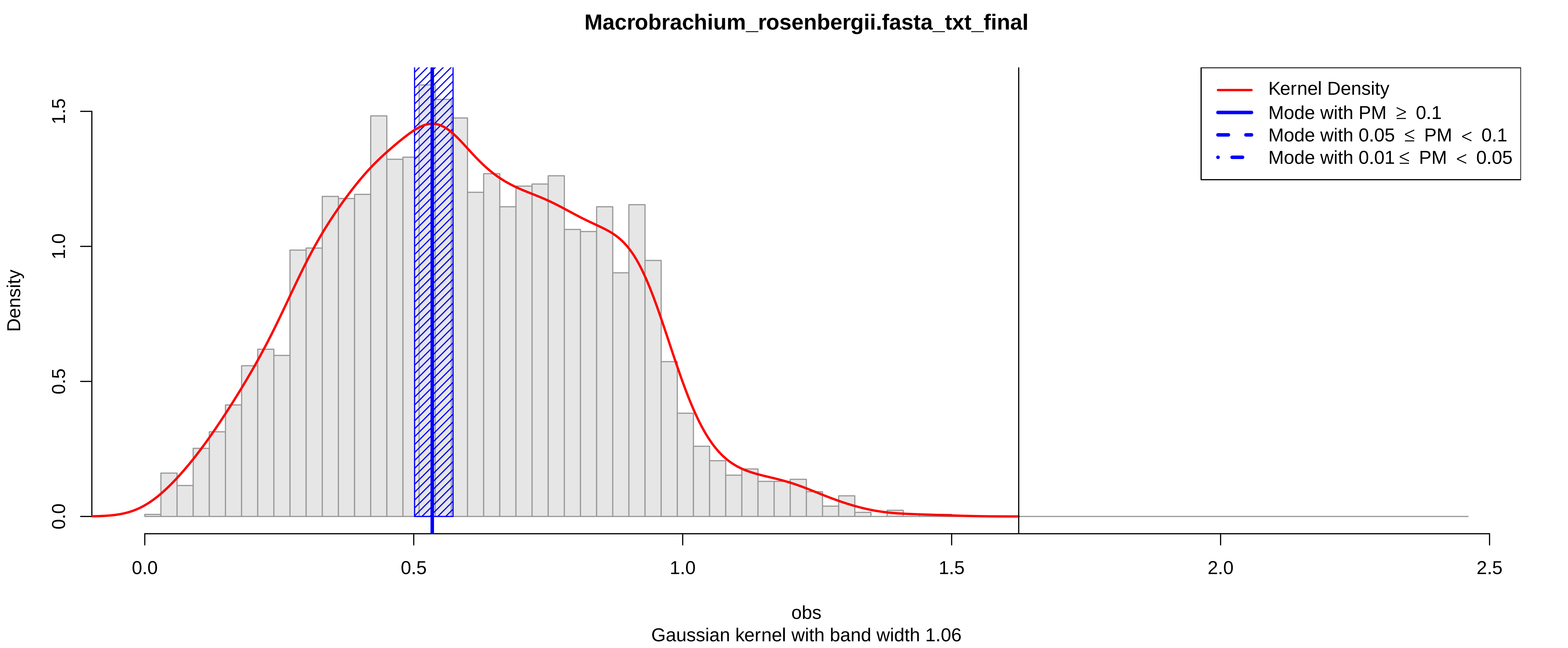
Macrobrachium_rosenbergii.fasta_txt_final
Kernel Density
Mode with PM ≥ 0.1
Mode with 0.05 ≤ PM < 0.1
Mode with 0.01 ≤ PM < 0.05
Density
0.0
0.5
1.0
1.5
0.0
0.5
1.0
1.5
2.0
2.5
obs
Gaussian kernel with band width 1.06

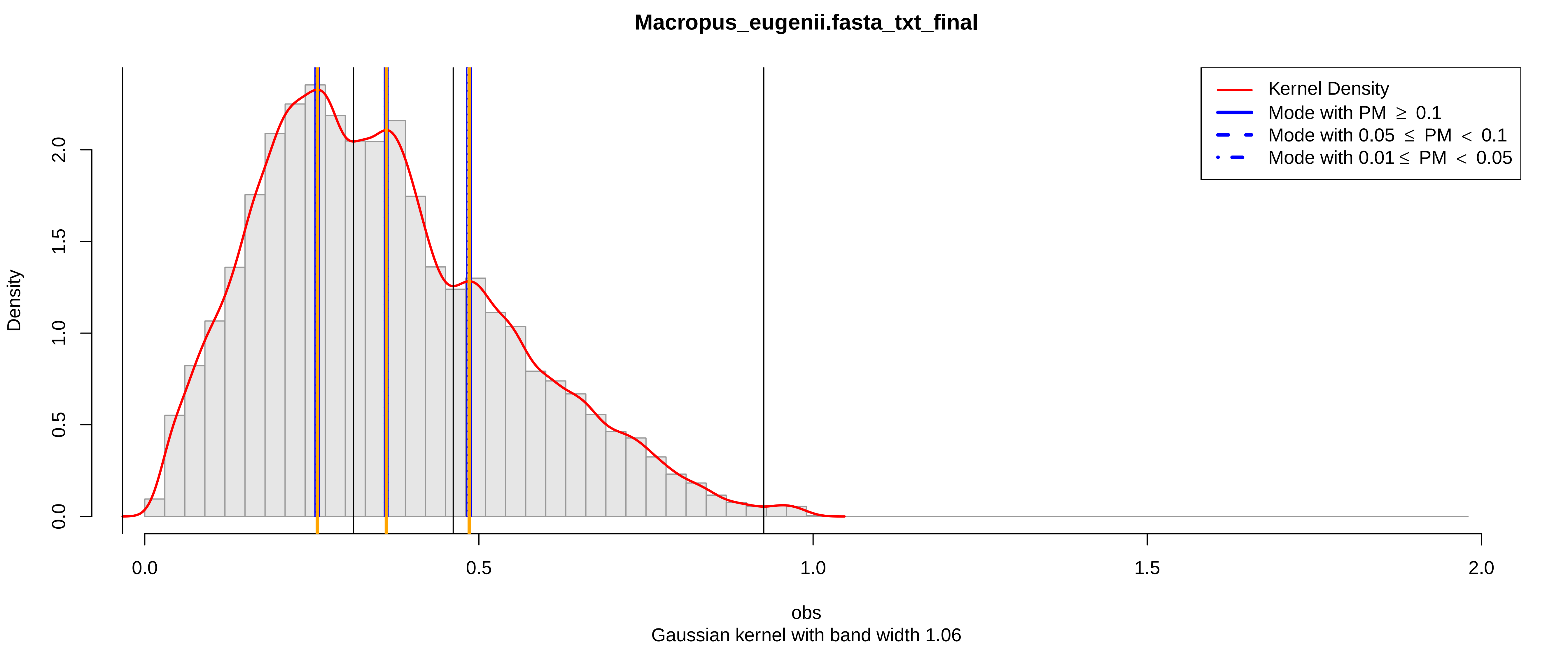
Macropus_eugenii.fasta_txt_final
Kernel Density
Mode with PM ≥ 0.1
Mode with 0.05 ≤ PM < 0.1
Mode with 0.01 ≤ PM < 0.05
Density
0.0
0.5
1.0
1.5
2.0
0.0
0.5
1.0
1.5
2.0
obs
Gaussian kernel with band width 1.06

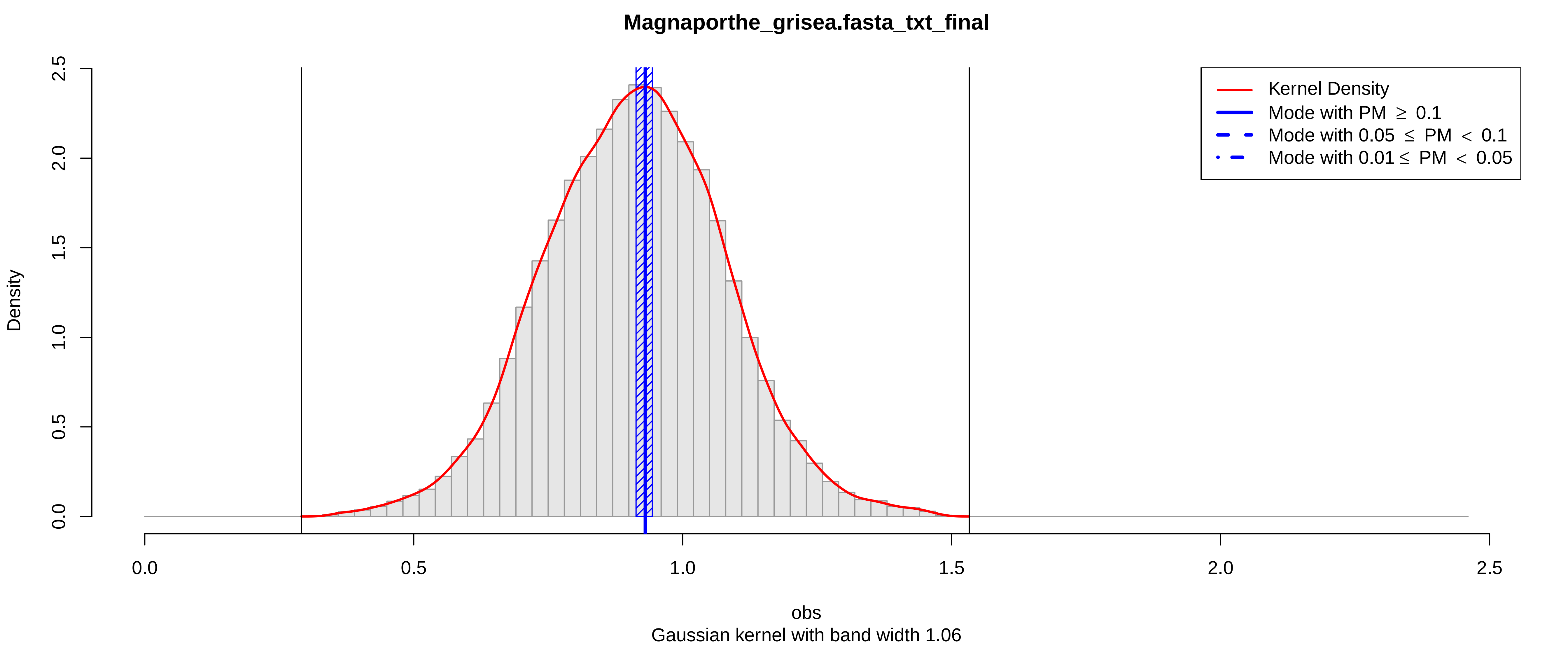
Magnaporthe_grisea.fasta_txt_final
Density
obs
Gaussian kernel with band width 1.06
0.0
0.5
1.0
1.5
2.0
2.5
Kernel Density
Mode with PM ≥ 0.1
Mode with 0.05 ≤ PM < 0.1
Mode with 0.01 ≤ PM < 0.05

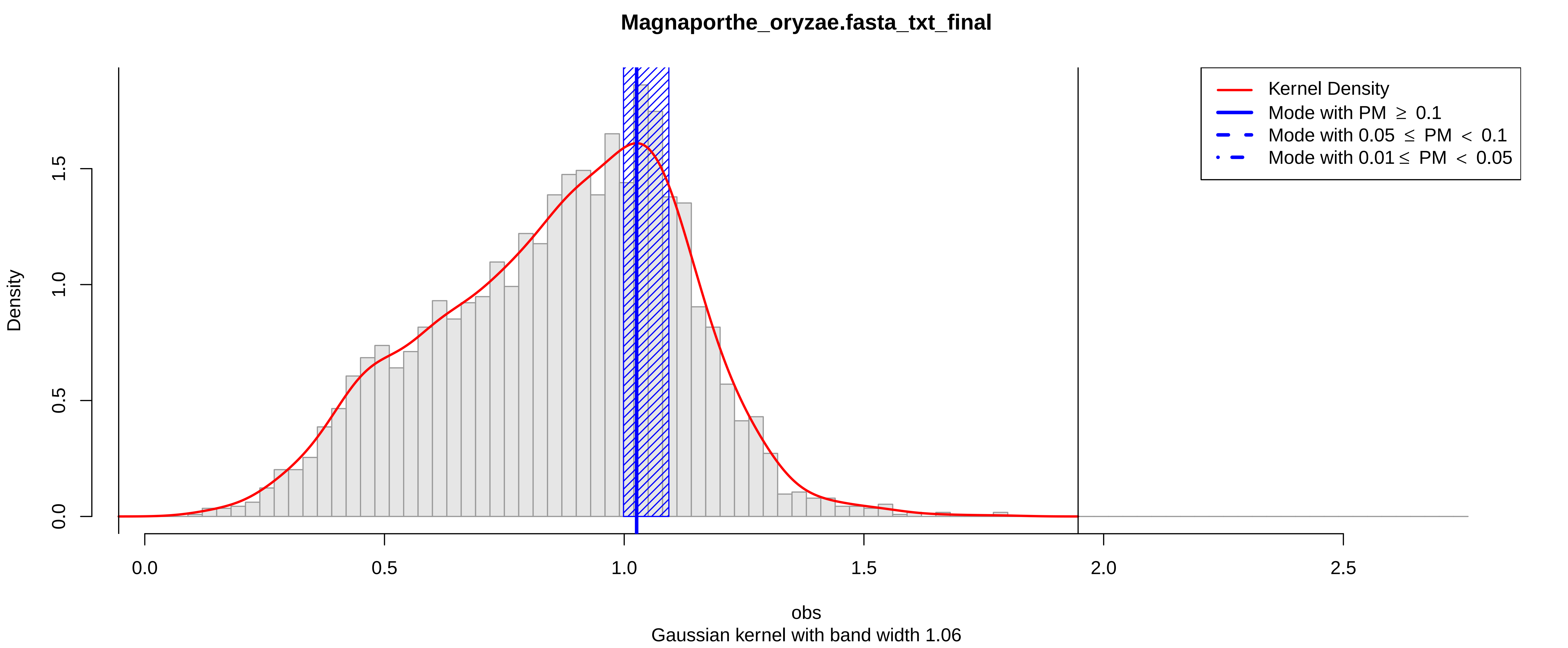

Magnaporthe_oryzae.fasta_txt_final
Kernel Density
Mode with PM ≥ 0.1
Mode with 0.05 ≤ PM < 0.1
Mode with 0.01 ≤ PM < 0.05
Density
0.0
0.5
1.0
1.5
0.0
0.5
1.0
1.5
2.0
2.5
obs
Gaussian kernel with band width 1.06

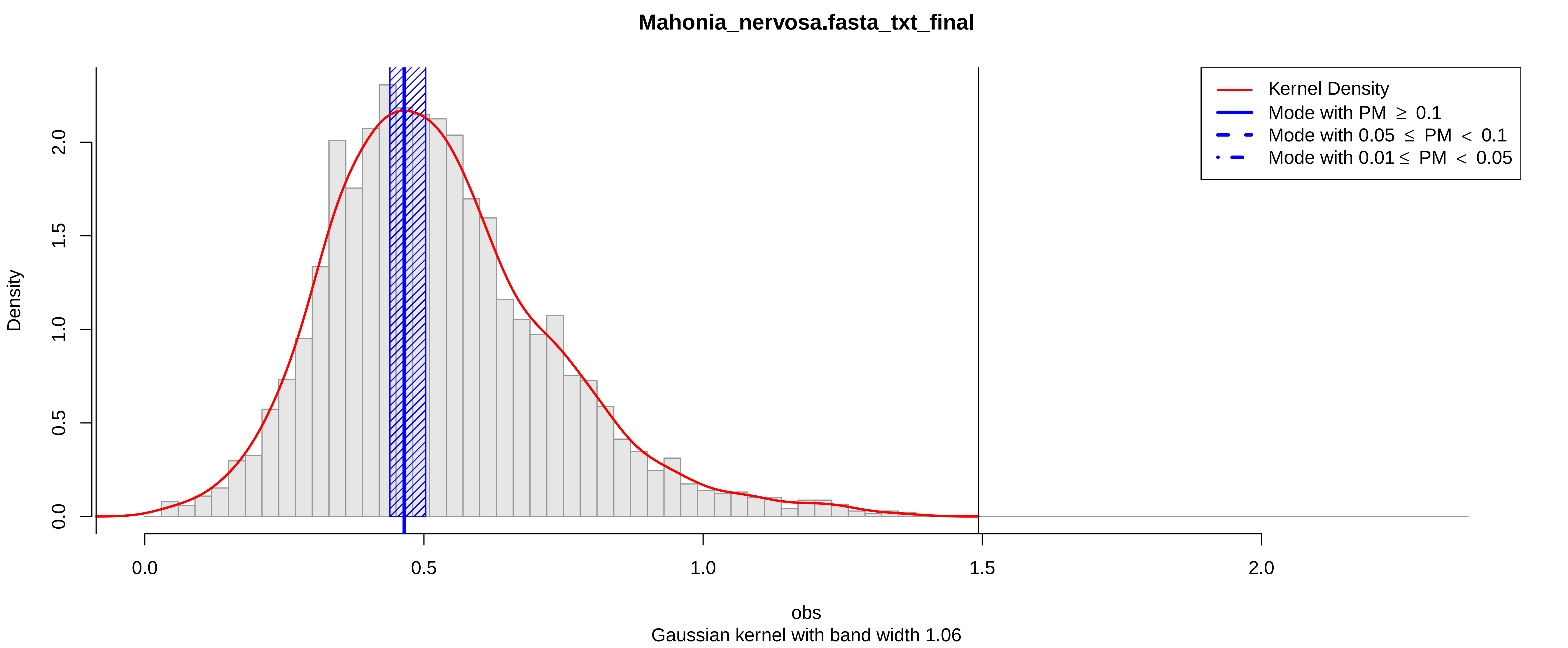

Mahonia_nervosa.fasta_txt_final
Kernel Density
Mode with PM ≥ 0.1
Mode with 0.05 ≤ PM < 0.1
Mode with 0.01 ≤ PM < 0.05
Density
0.0
0.5
1.0
1.5
2.0
obs
Gaussian kernel with band width 1.06

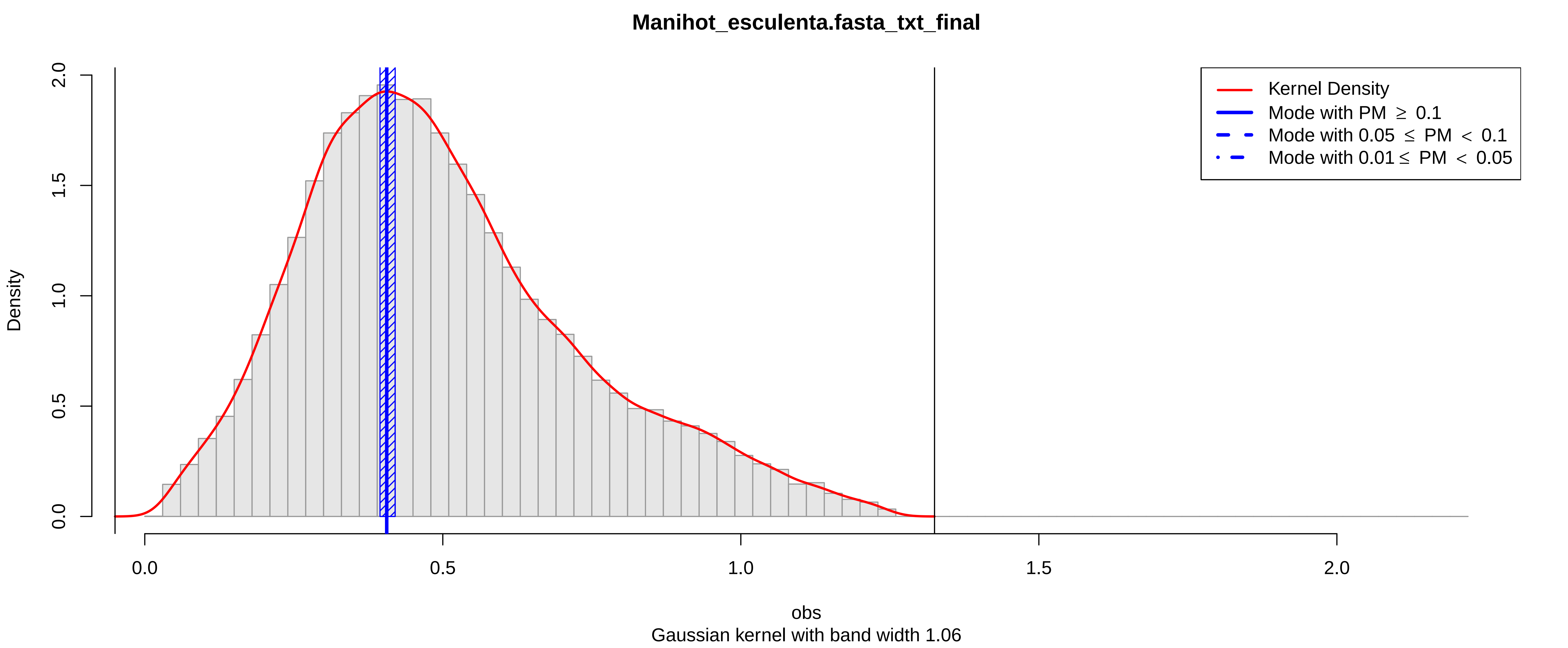
Manihot_esculenta.fasta_txt_final
Kernel Density
Mode with PM ≥ 0.1
Mode with 0.05 ≤ PM < 0.1
Mode with 0.01 ≤ PM < 0.05
Density
0.0
0.5
1.0
1.5
2.0
obs
Gaussian kernel with band width 1.06

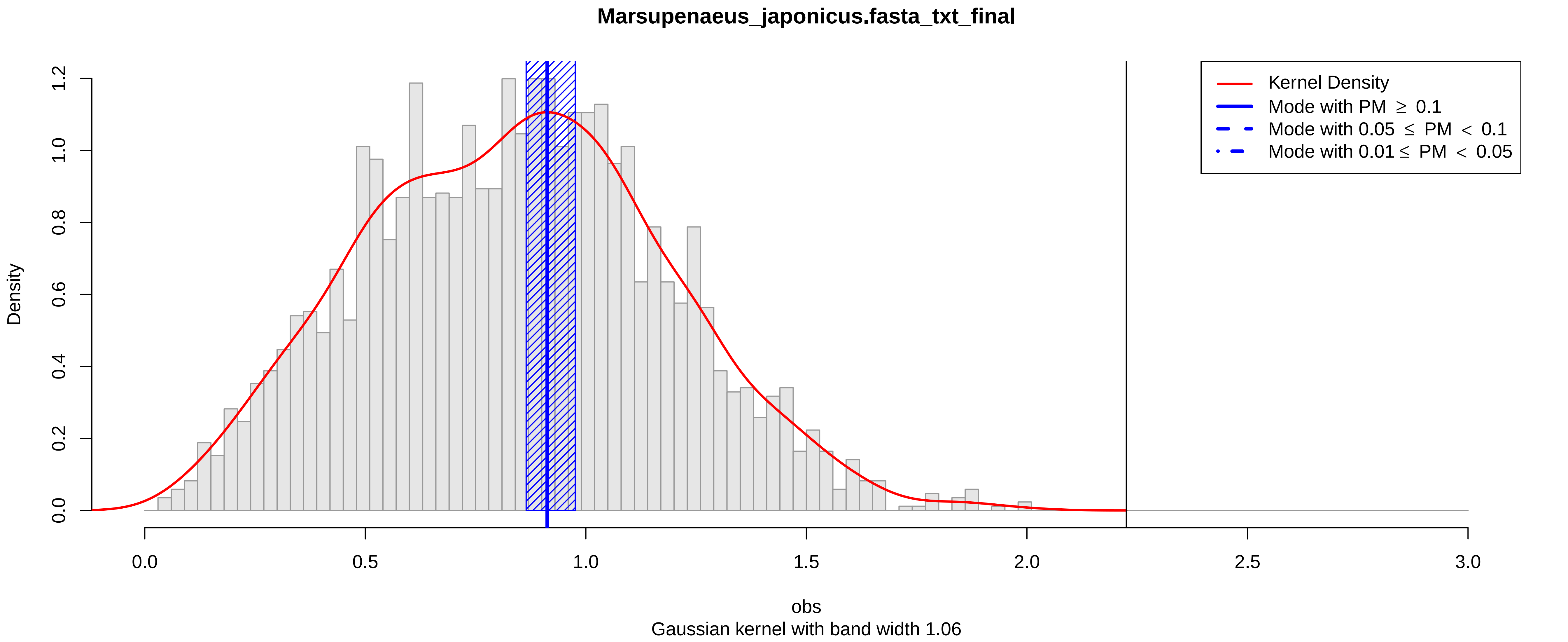
Marsupenaeus_japonicus.fasta_txt_final
Density
1.2
1.0
0.8
0.6
0.4
0.2
0.0
0.0
0.5
1.0
1.5
2.0
2.5
3.0
obs
Gaussian kernel with band width 1.06
Kernel Density
Mode with PM ≥ 0.1
Mode with 0.05 ≤ PM < 0.1
Mode with 0.01 ≤ PM < 0.05

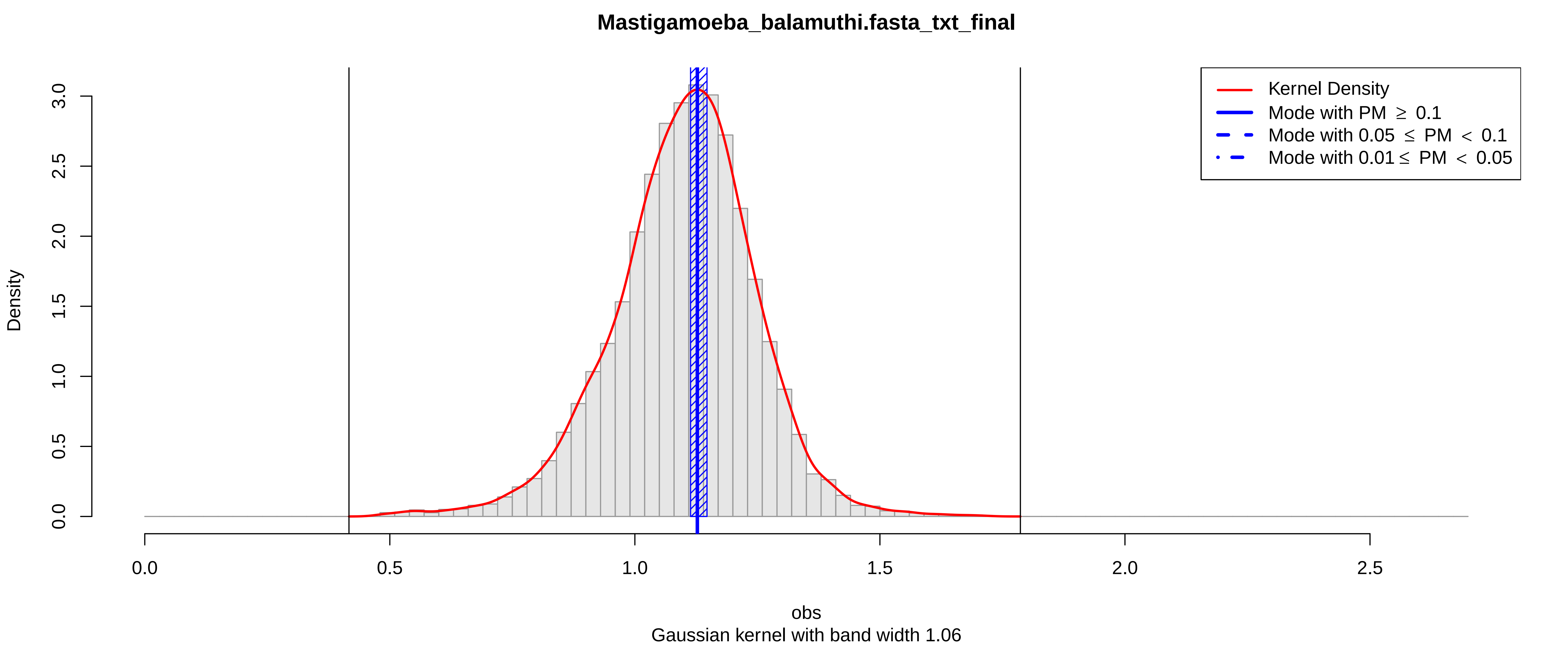

Mastigamoeba_balamuthi.fasta_txt_final
Kernel Density
Mode with PM ≥ 0.1
Mode with 0.05 ≤ PM < 0.1
Mode with 0.01 ≤ PM < 0.05
Density
0.0
0.5
1.0
1.5
2.0
2.5
3.0
obs
Gaussian kernel with band width 1.06

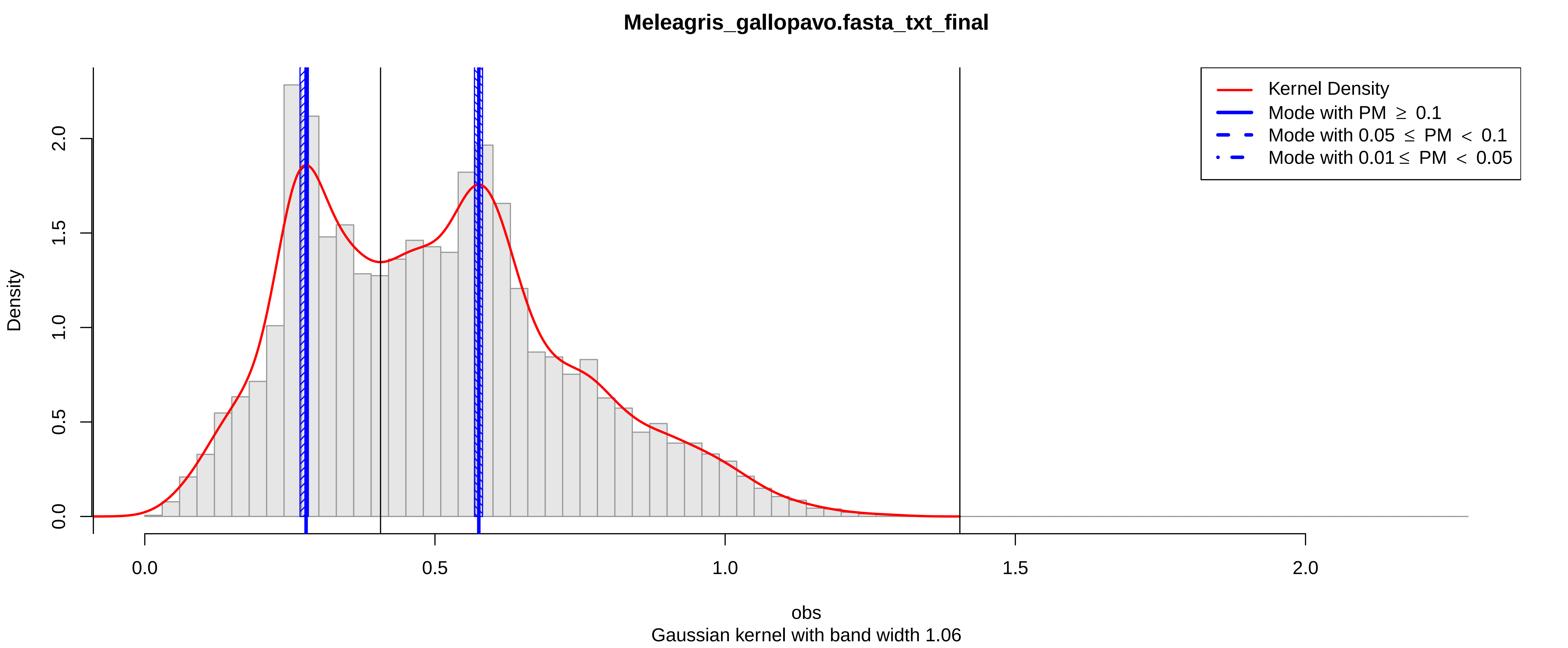

Meleagris_gallopavo.fasta_txt_final
Kernel Density
Mode with PM ≥ 0.1
Mode with 0.05 ≤ PM < 0.1
Mode with 0.01 ≤ PM < 0.05
Density
0.0
0.5
1.0
1.5
2.0
obs
Gaussian kernel with band width 1.06

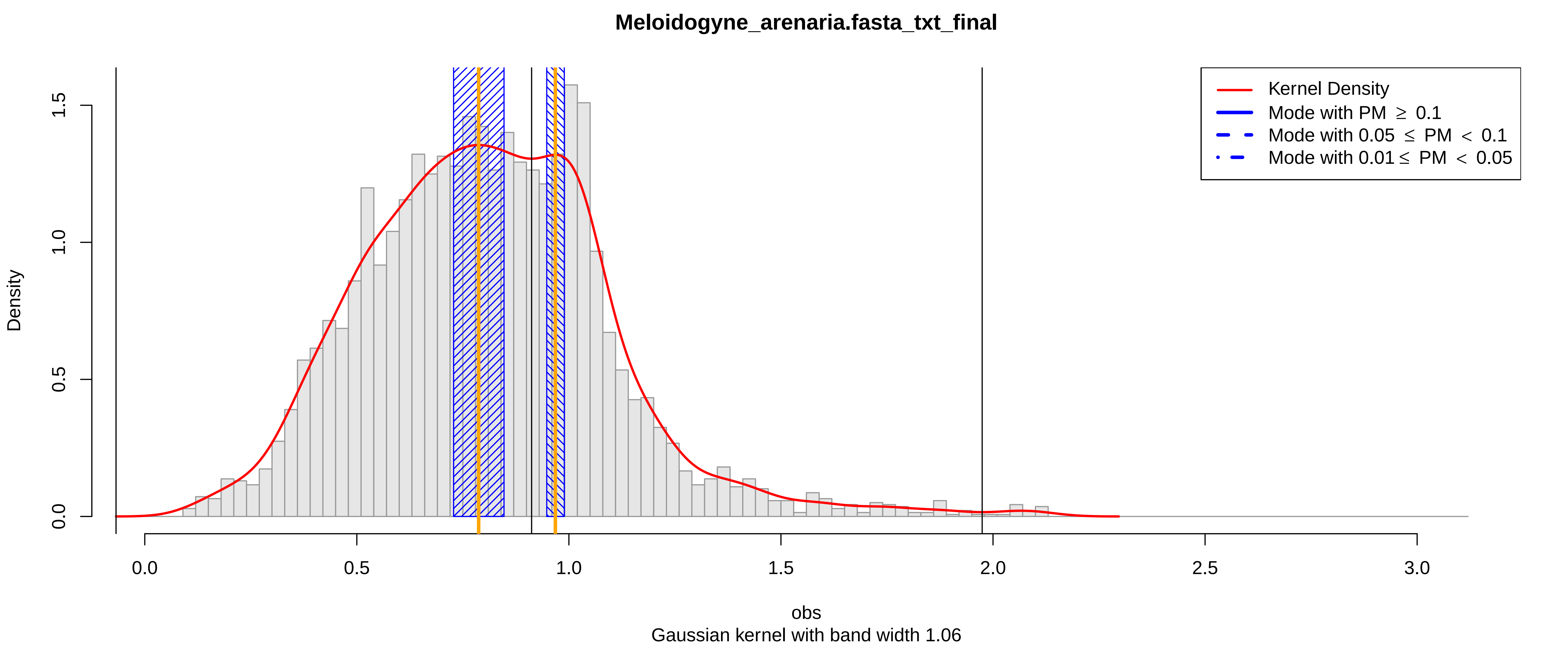

Meloidogyne_arenaria.fasta_txt_final
Kernel Density
Mode with PM ≥ 0.1
Mode with 0.05 ≤ PM < 0.1
Mode with 0.01 ≤ PM < 0.05
Density
0.0
0.5
1.0
1.5
0.0
0.5
1.0
1.5
2.0
2.5
3.0
obs
Gaussian kernel with band width 1.06

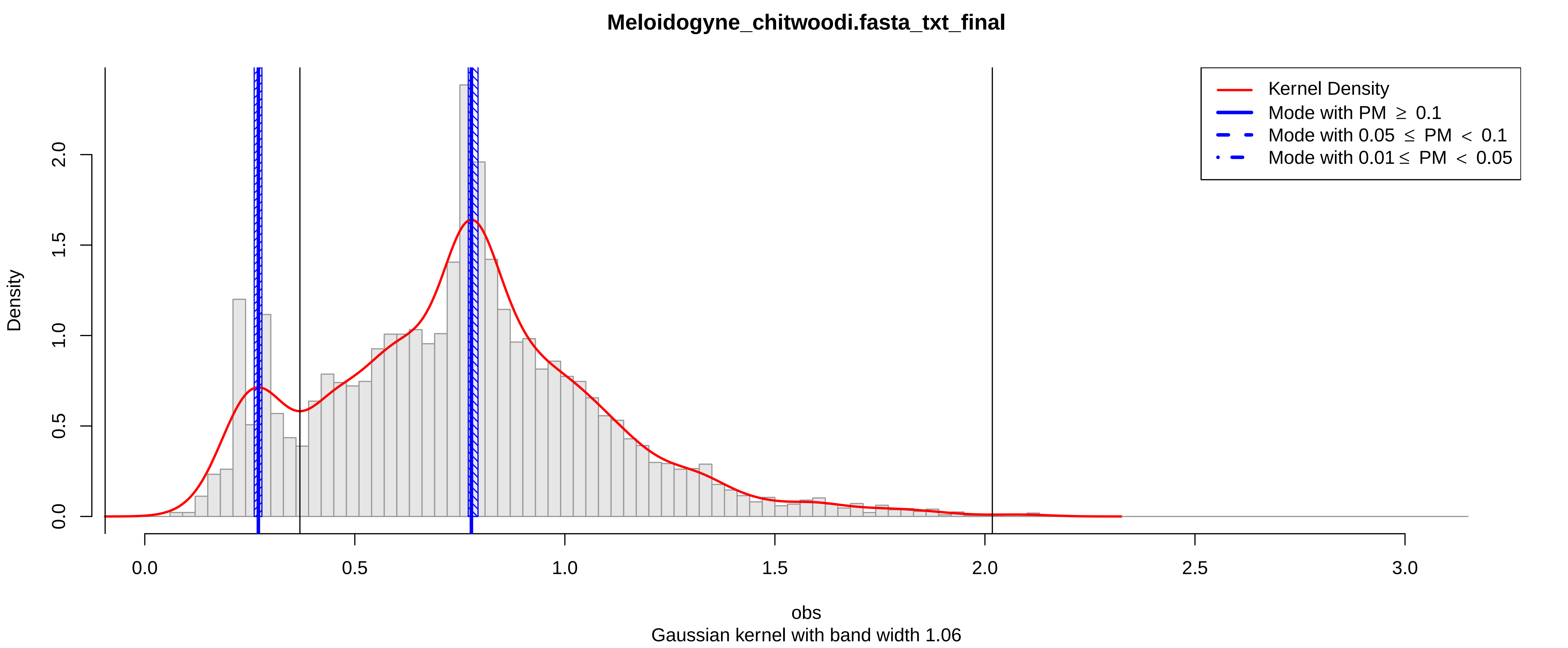

Meloidogyne_chitwoodi.fasta_txt_final
Kernel Density
Mode with PM ≥ 0.1
Mode with 0.05 ≤ PM < 0.1
Mode with 0.01 ≤ PM < 0.05
Density
0.0
0.5
1.0
1.5
2.0
2.5
3.0
obs
Gaussian kernel with band width 1.06

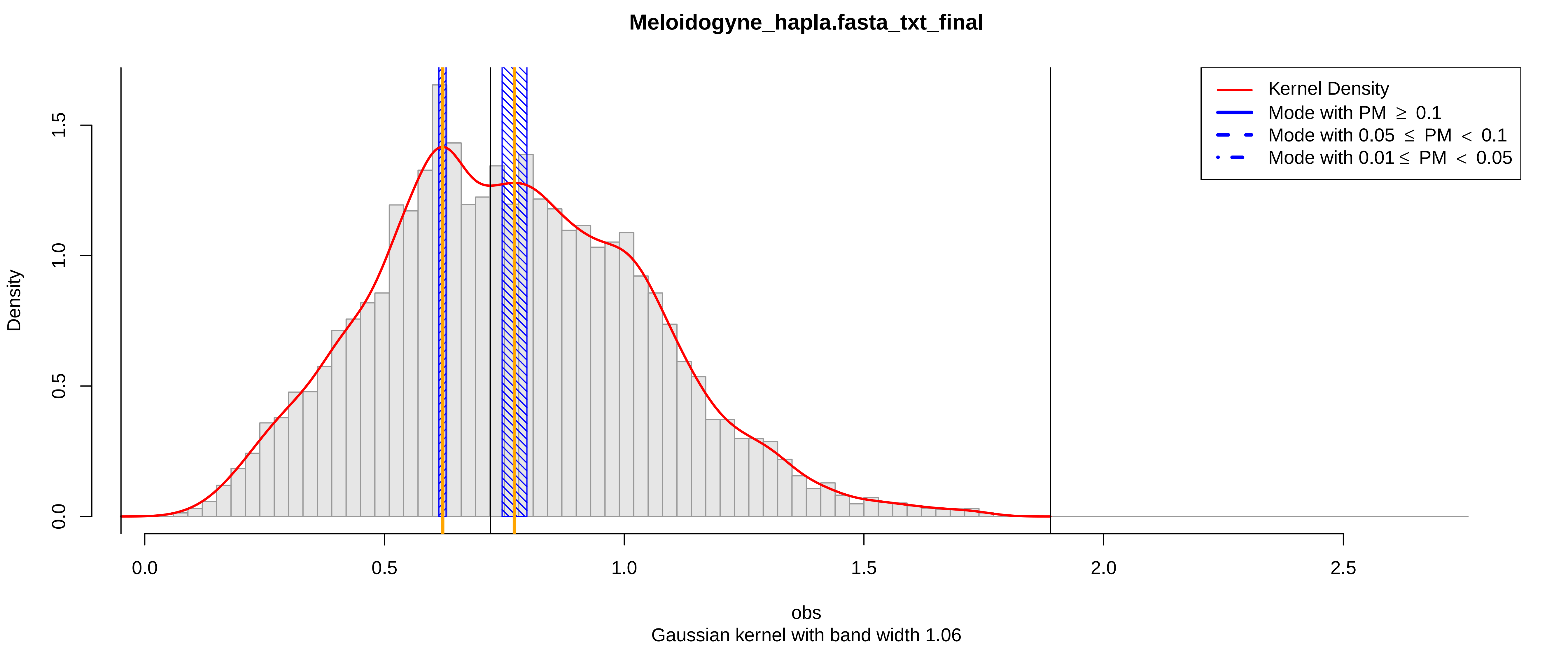

Meloidogyne_hapla.fasta_txt_final
Kernel Density
Mode with PM ≥ 0.1
Mode with 0.05 ≤ PM < 0.1
Mode with 0.01 ≤ PM < 0.05
Density
0.0
0.5
1.0
1.5
0.0
0.5
1.0
1.5
2.0
2.5
obs
Gaussian kernel with band width 1.06

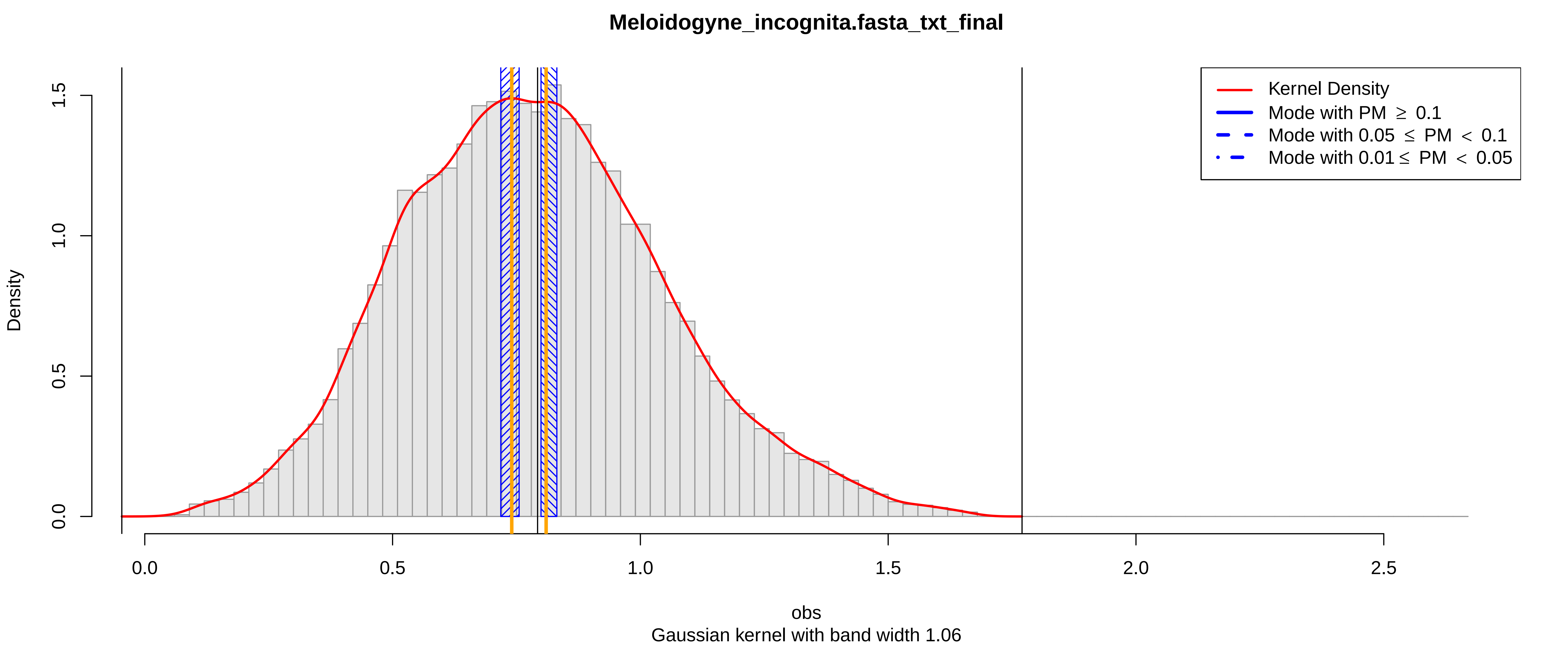

Meloidogyne_incognita.fasta_txt_final
Kernel Density
Mode with PM ≥ 0.1
Mode with 0.05 ≤ PM < 0.1
Mode with 0.01 ≤ PM < 0.05
Density
0.0
0.5
1.0
1.5
0.0
0.5
1.0
1.5
2.0
2.5
obs
Gaussian kernel with band width 1.06

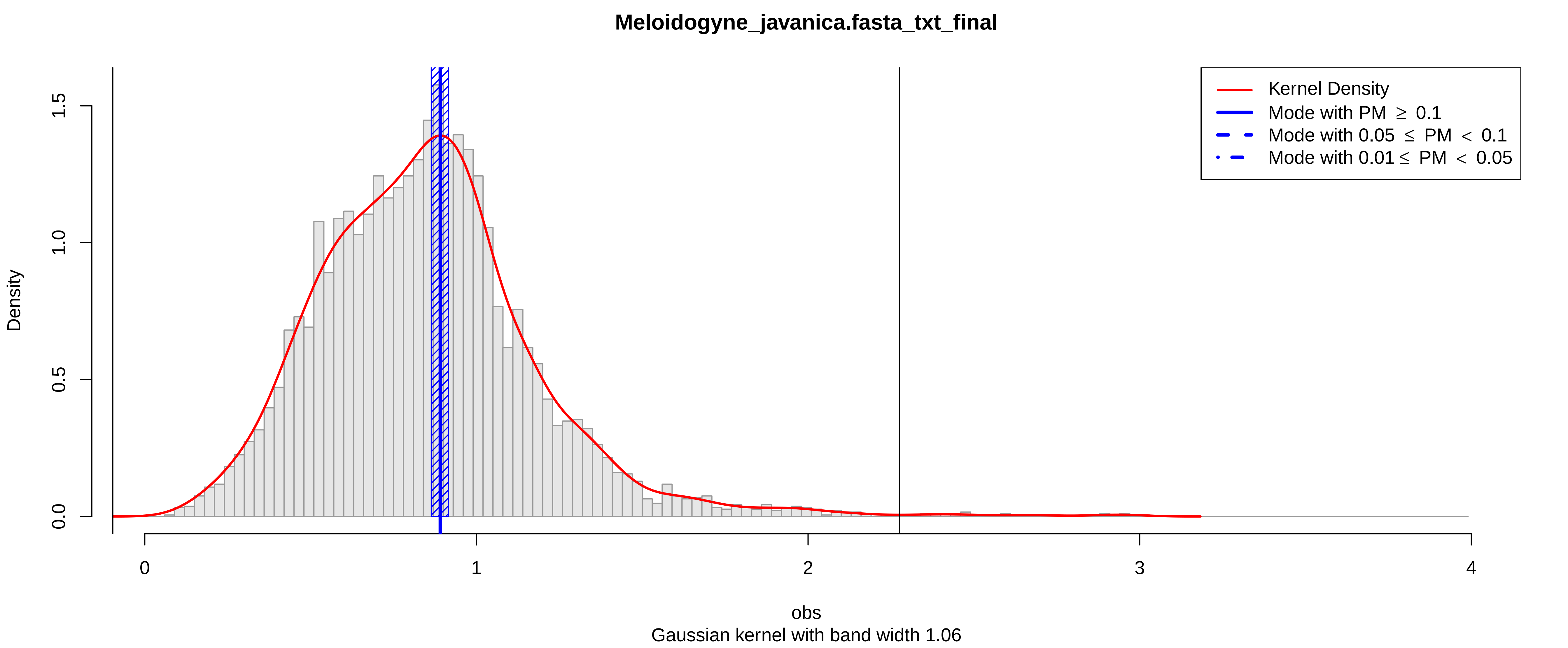

Meloidogyne_javanica.fasta_txt_final
Kernel Density
Mode with PM ≥ 0.1
Mode with 0.05 ≤ PM < 0.1
Mode with 0.01 ≤ PM < 0.05
Density
0.0
0.5
1.0
1.5
0
1
2
3
4
obs
Gaussian kernel with band width 1.06

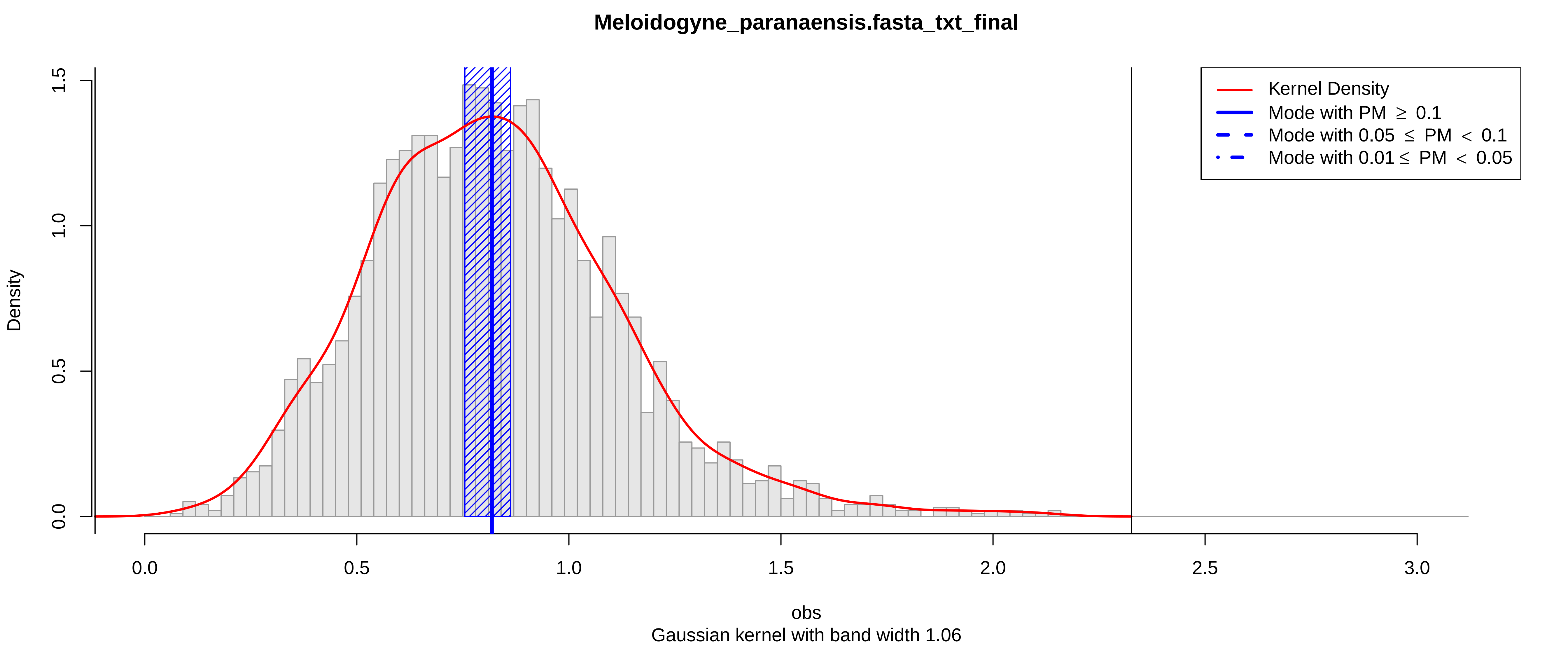

Meloidogyne_paranaensis.fasta_txt_final
Kernel Density
Mode with PM ≥ 0.1
Mode with 0.05 ≤ PM < 0.1
Mode with 0.01 ≤ PM < 0.05
Density
0.0
0.5
1.0
1.5
0.0
0.5
1.0
1.5
2.0
2.5
3.0
obs
Gaussian kernel with band width 1.06

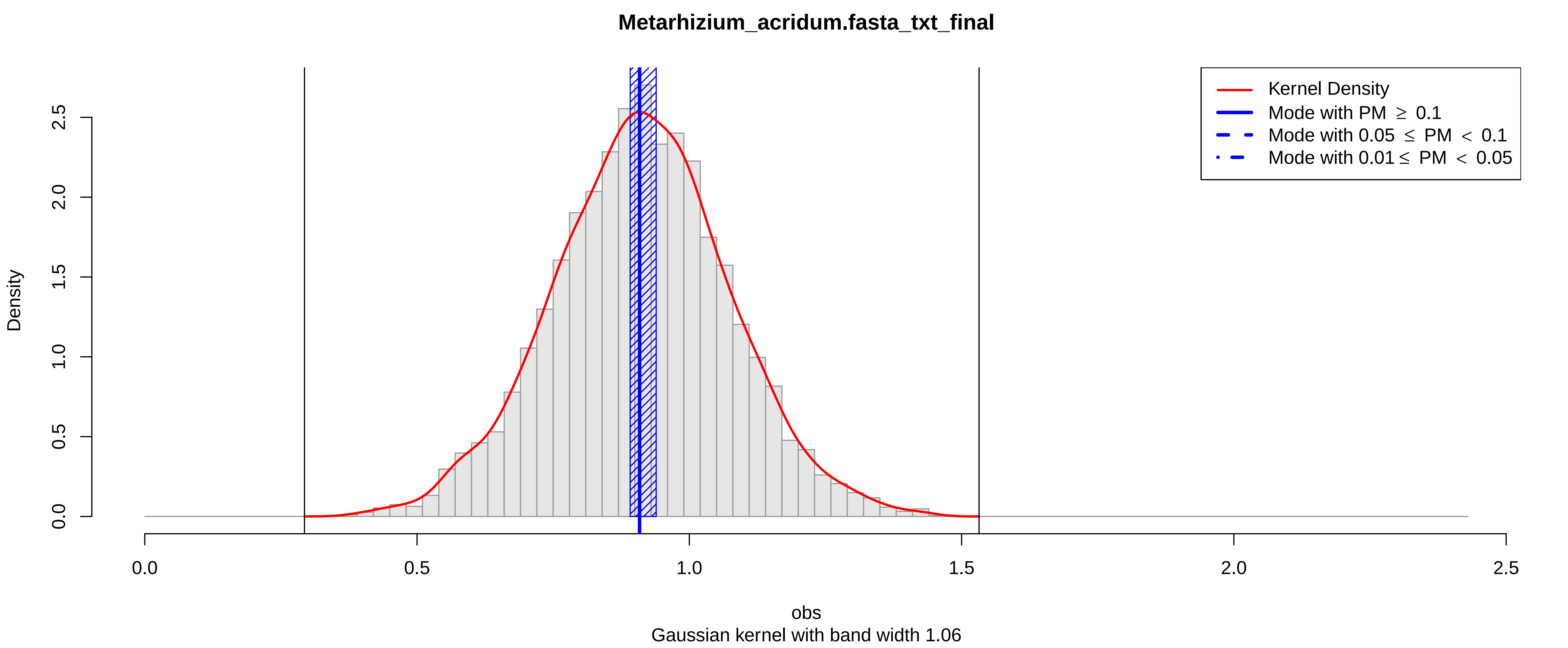

Metarhizium_acridum.fasta_txt_final
Kernel Density
Mode with PM ≥ 0.1
Mode with 0.05 ≤ PM < 0.1
Mode with 0.01 ≤ PM < 0.05
Density
0.0
0.5
1.0
1.5
2.0
2.5
obs
Gaussian kernel with band width 1.06

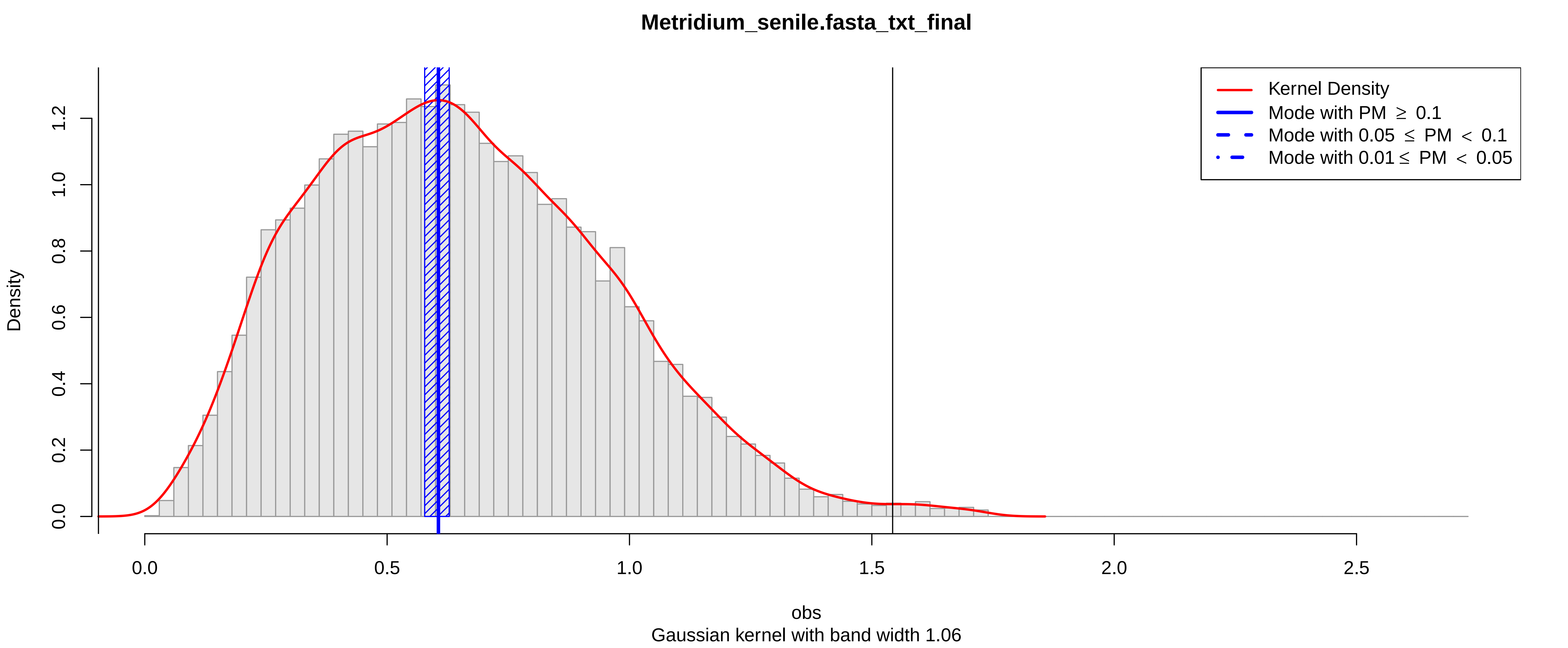

Metridium_senile.fasta_txt_final
Kernel Density
Mode with PM ≥ 0.1
Mode with 0.05 ≤ PM < 0.1
Mode with 0.01 ≤ PM < 0.05
Density
0.0
0.2
0.4
0.6
0.8
1.0
1.2
0.0
0.5
1.0
1.5
2.0
2.5
obs
Gaussian kernel with band width 1.06

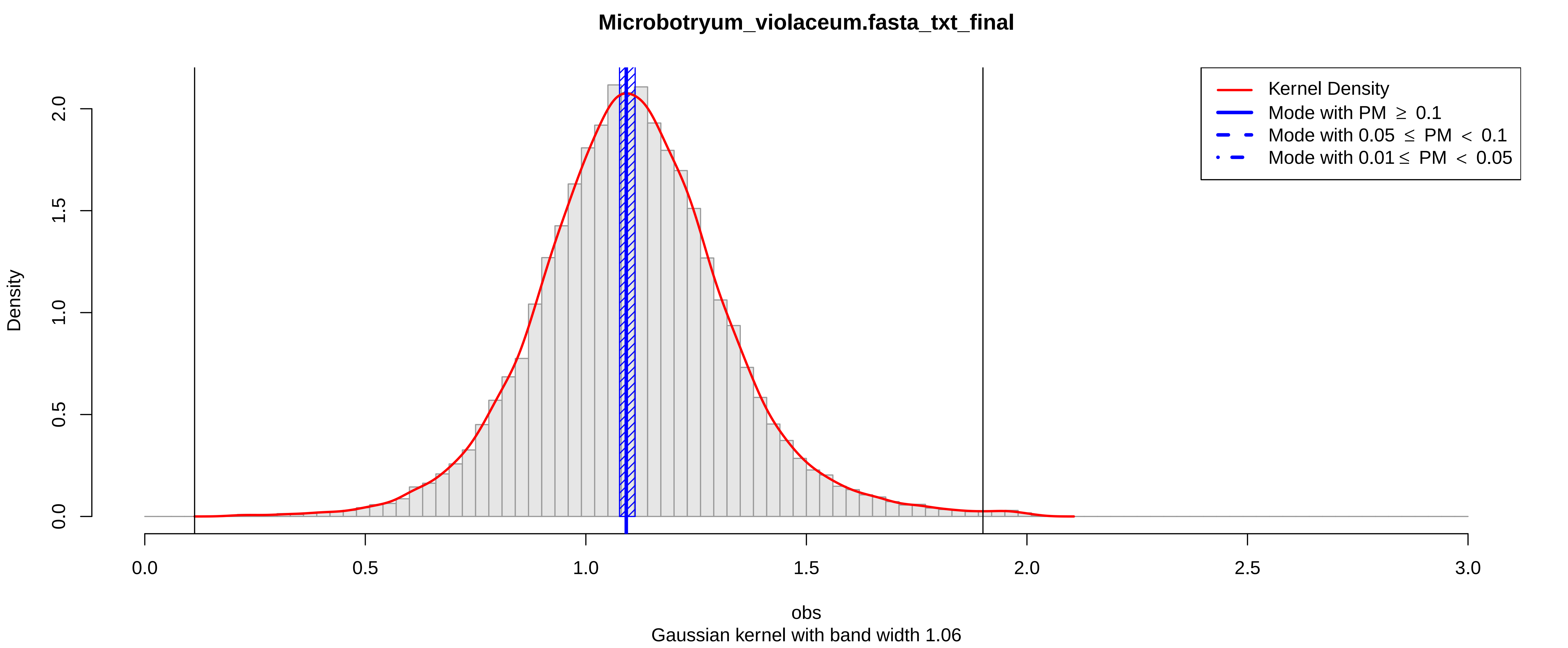

Microbotryum_violaceum.fasta_txt_final
Kernel Density
Mode with PM ≥ 0.1
Mode with 0.05 ≤ PM < 0.1
Mode with 0.01 ≤ PM < 0.05
Density
0.0
0.5
1.0
1.5
2.0
0.0
0.5
1.0
1.5
2.0
2.5
3.0
obs
Gaussian kernel with band width 1.06

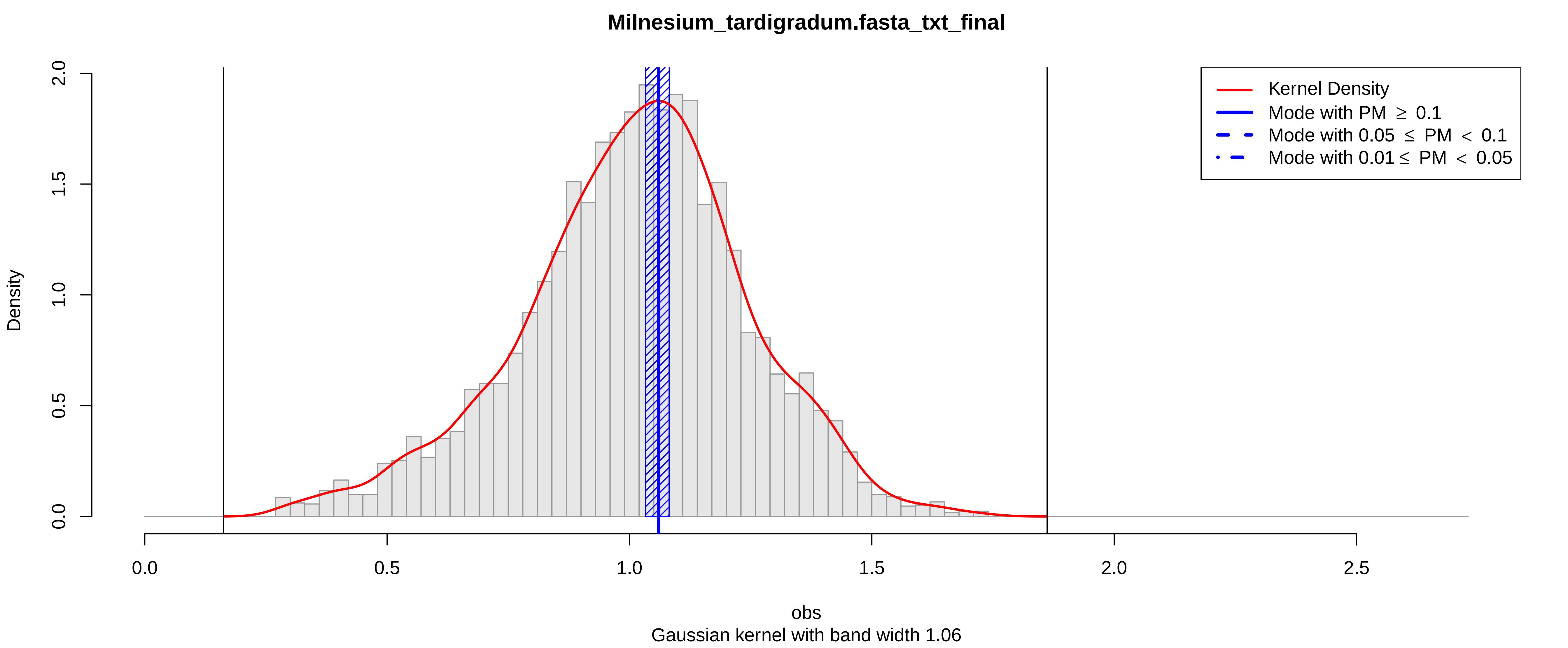

Milnesium_tardigradum.fasta_txt_final
Kernel Density
Mode with PM ≥ 0.1
Mode with 0.05 ≤ PM < 0.1
Mode with 0.01 ≤ PM < 0.05
Density
0.0
0.5
1.0
1.5
2.0
2.5
obs
Gaussian kernel with band width 1.06

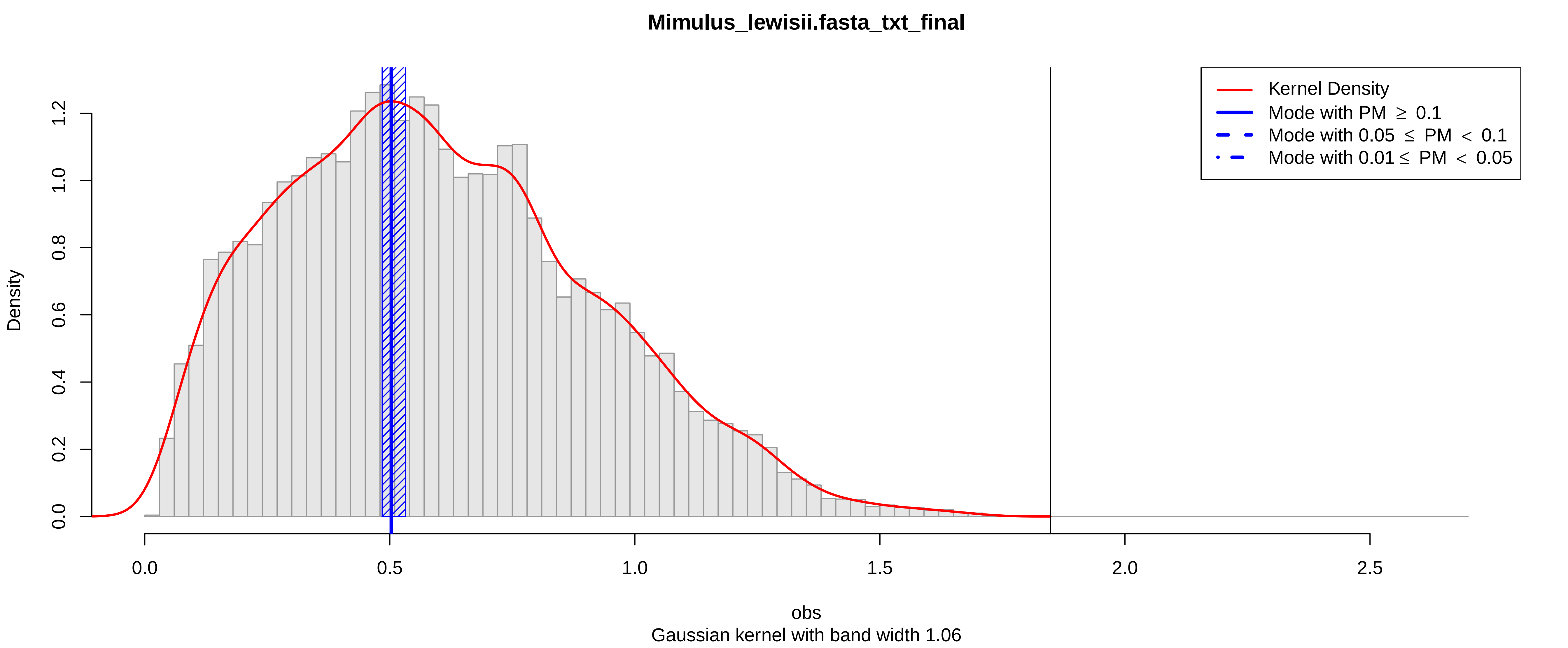
Mimulus_lewisii.fasta_txt_final
Kernel Density
Mode with PM ≥ 0.1
Mode with 0.05 ≤ PM < 0.1
Mode with 0.01 ≤ PM < 0.05
Density
0.0
0.2
0.4
0.6
0.8
1.0
1.2
0.0
0.5
1.0
1.5
2.0
2.5
obs
Gaussian kernel with band width 1.06

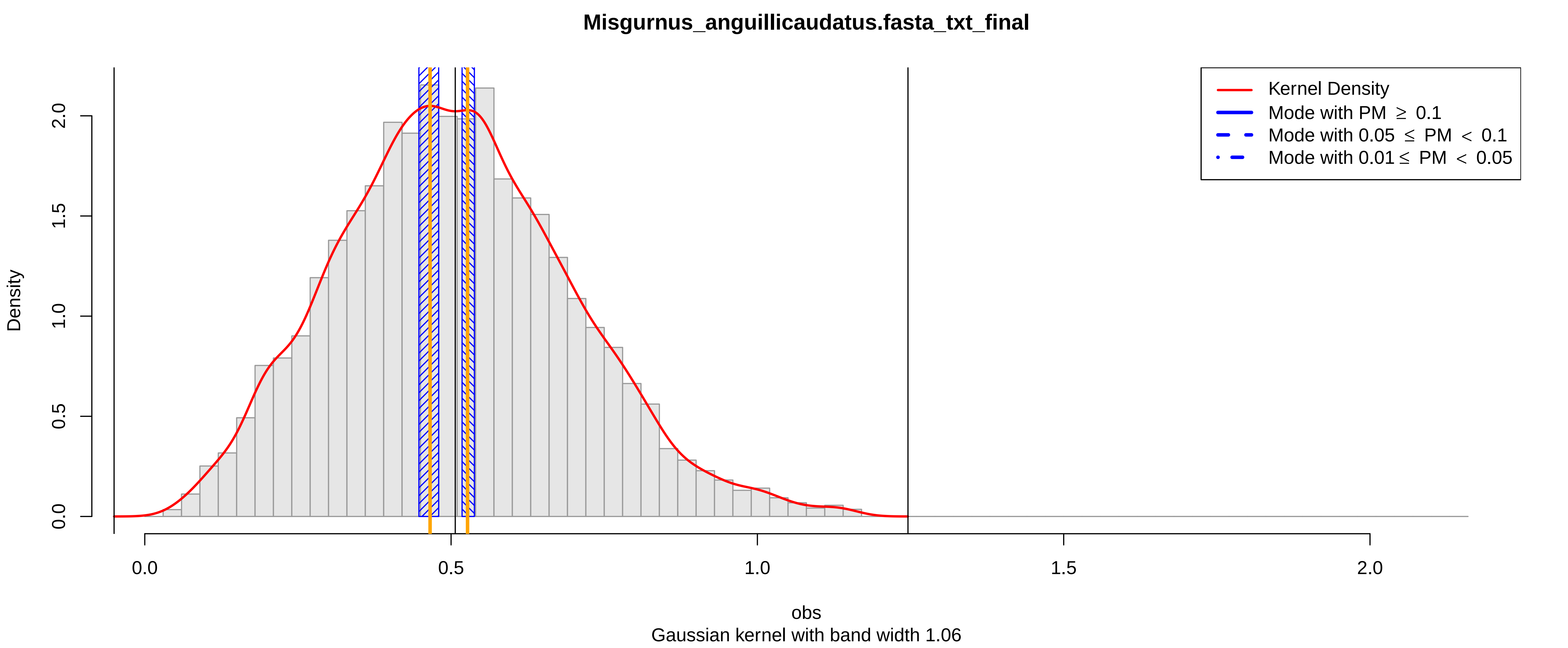

Misgurnus_anguillicaudatus.fasta_txt_final
Density
0.0
0.5
1.0
1.5
2.0
Kernel Density
Mode with PM ≥ 0.1
Mode with 0.05 ≤ PM < 0.1
Mode with 0.01 ≤ PM < 0.05
0.0
0.5
1.0
1.5
2.0
obs
Gaussian kernel with band width 1.06

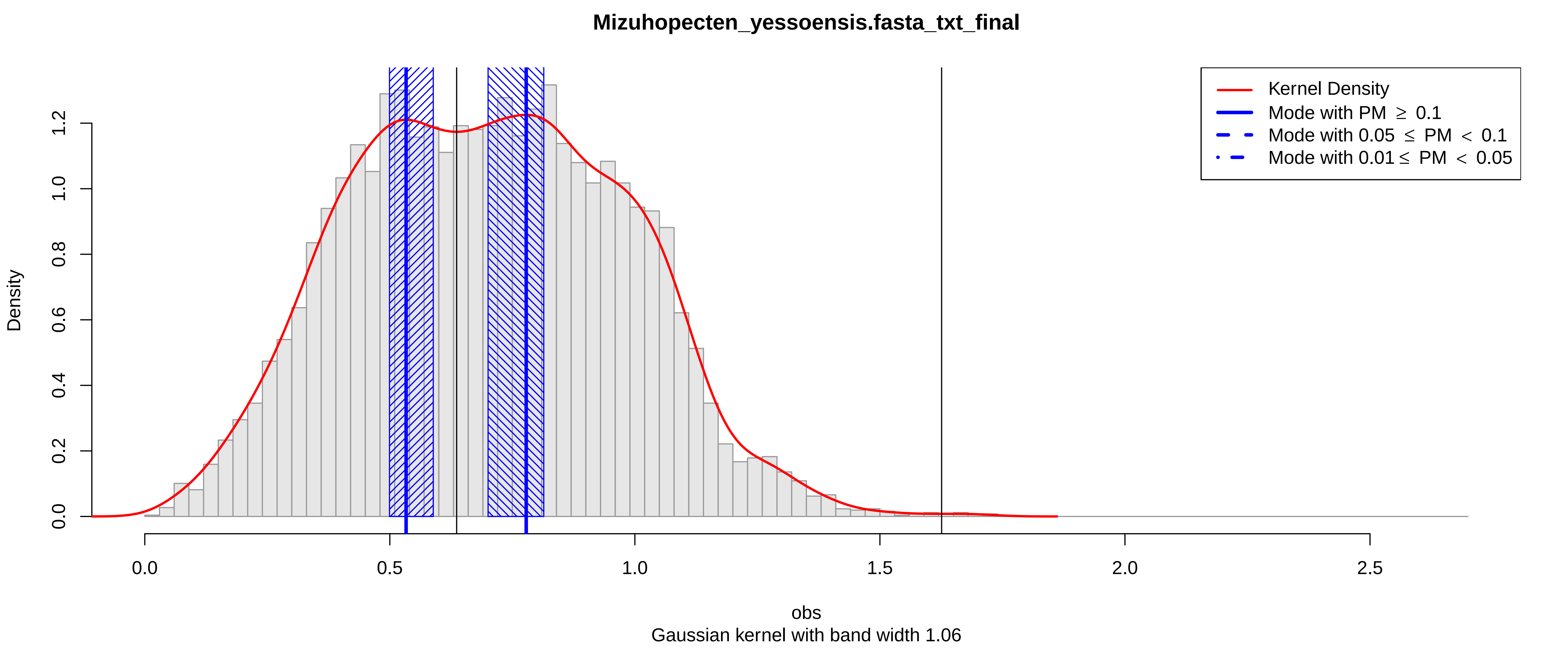

Mizuhopecten_yessoensis.fasta_txt_final
Kernel Density
Mode with PM ≥ 0.1
Mode with 0.05 ≤ PM < 0.1
Mode with 0.01 ≤ PM < 0.05
Density
0.0
0.2
0.4
0.6
0.8
1.0
1.2
0.0
0.5
1.0
1.5
2.0
2.5
obs
Gaussian kernel with band width 1.06

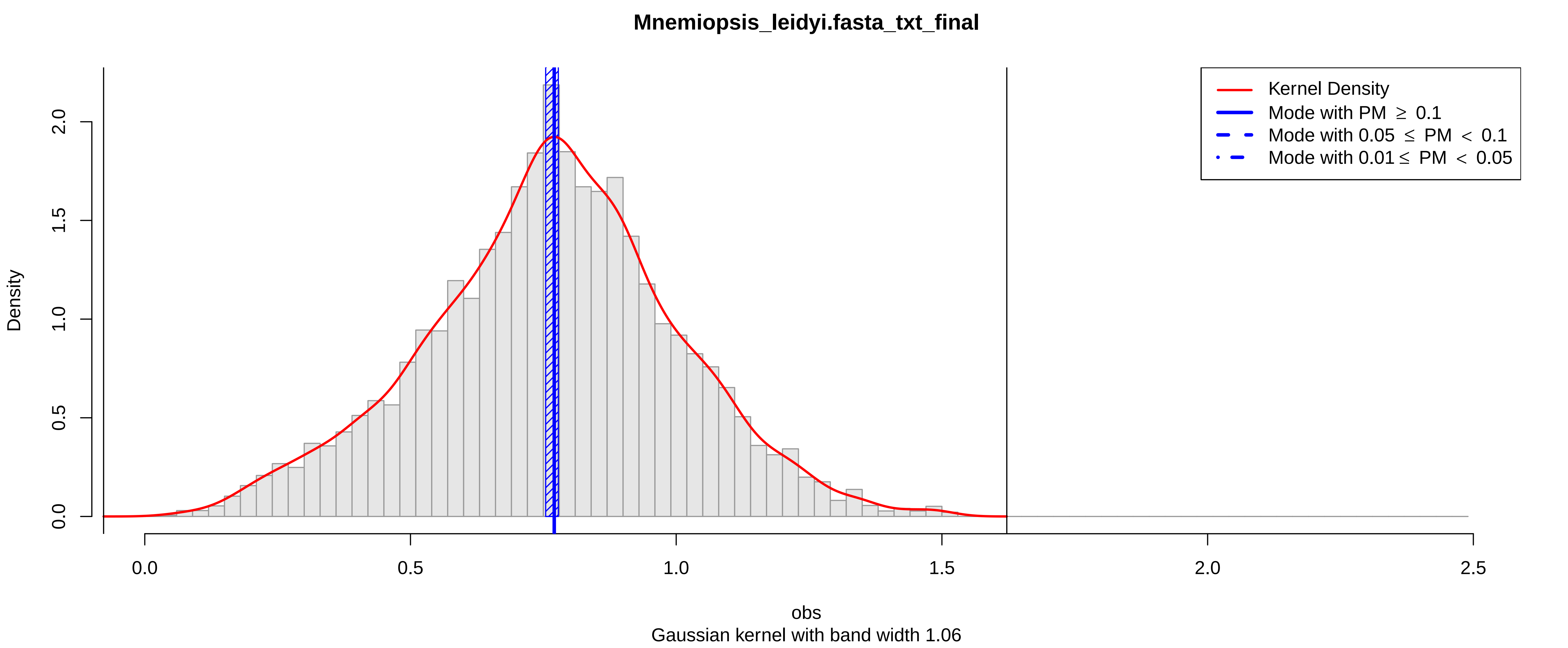

Mnemiopsis_leidyi.fasta_txt_final
Kernel Density
Mode with PM ≥ 0.1
Mode with 0.05 ≤ PM < 0.1
Mode with 0.01 ≤ PM < 0.05
Density
0.0
0.5
1.0
1.5
2.0
0.0
0.5
1.0
1.5
2.0
2.5
obs
Gaussian kernel with band width 1.06

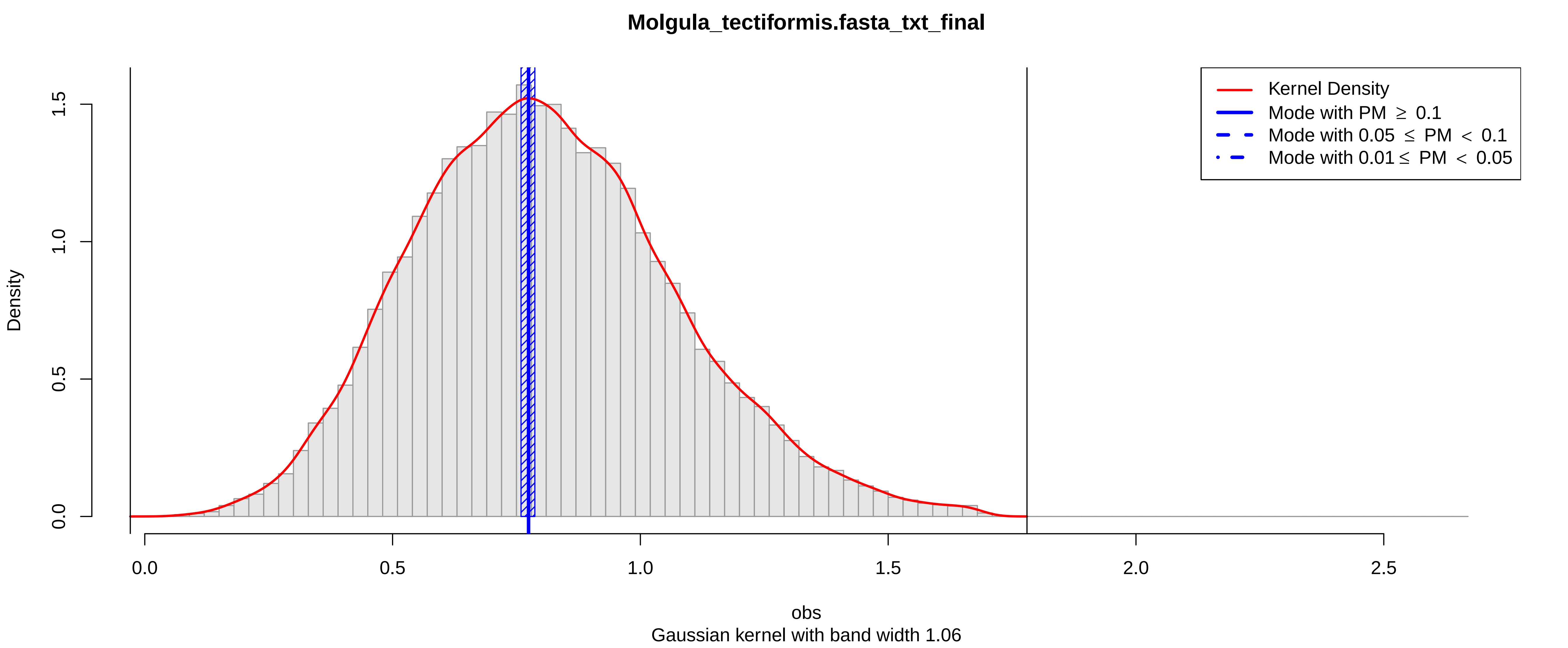
Molgula_tectiformis.fasta_txt_final
Kernel Density
Mode with PM ≥ 0.1
Mode with 0.05 ≤ PM < 0.1
Mode with 0.01 ≤ PM < 0.05
Density
0.0
0.5
1.0
1.5
0.0
0.5
1.0
1.5
2.0
2.5
obs
Gaussian kernel with band width 1.06

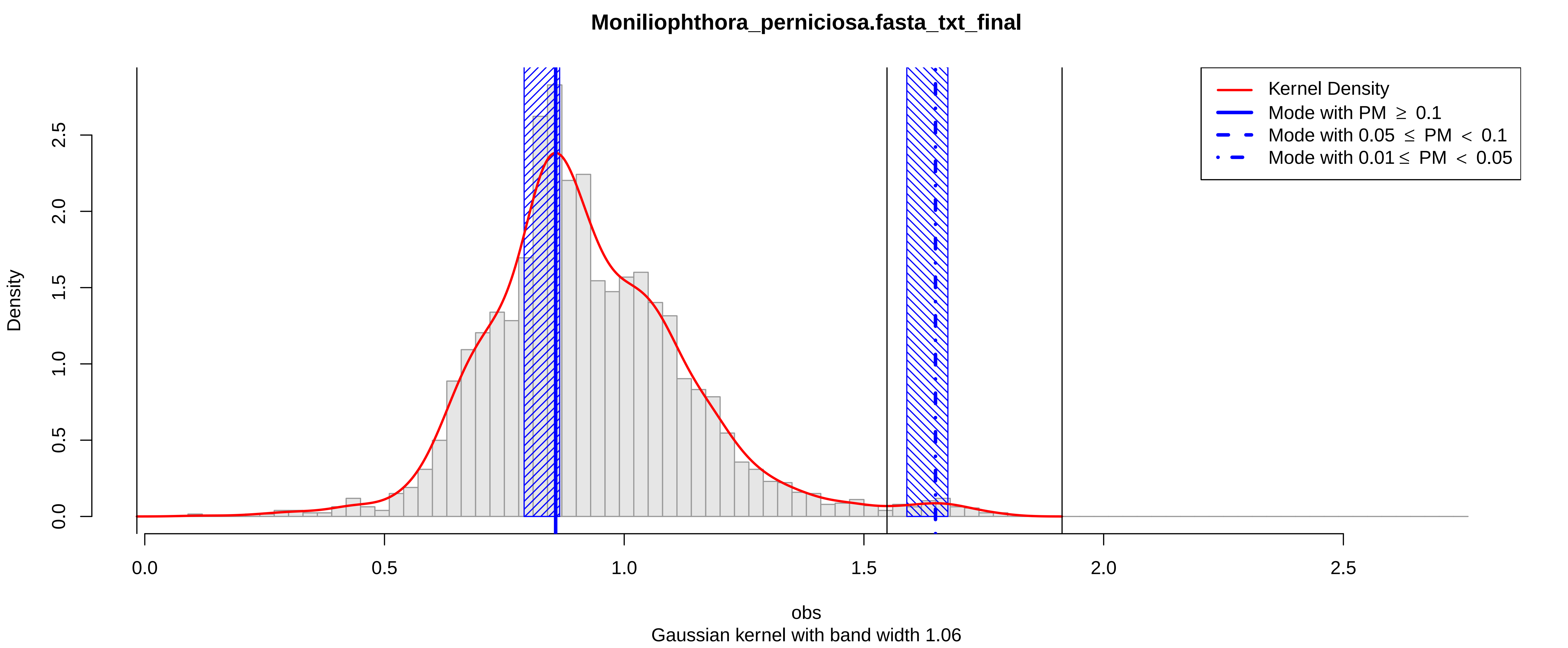
Moniliophthora_perniciosa.fasta_txt_final
Kernel Density
Mode with PM ≥ 0.1
Mode with 0.05 ≤ PM < 0.1
Mode with 0.01 ≤ PM < 0.05
Density
0.0
0.5
1.0
1.5
2.0
2.5
obs
Gaussian kernel with band width 1.06

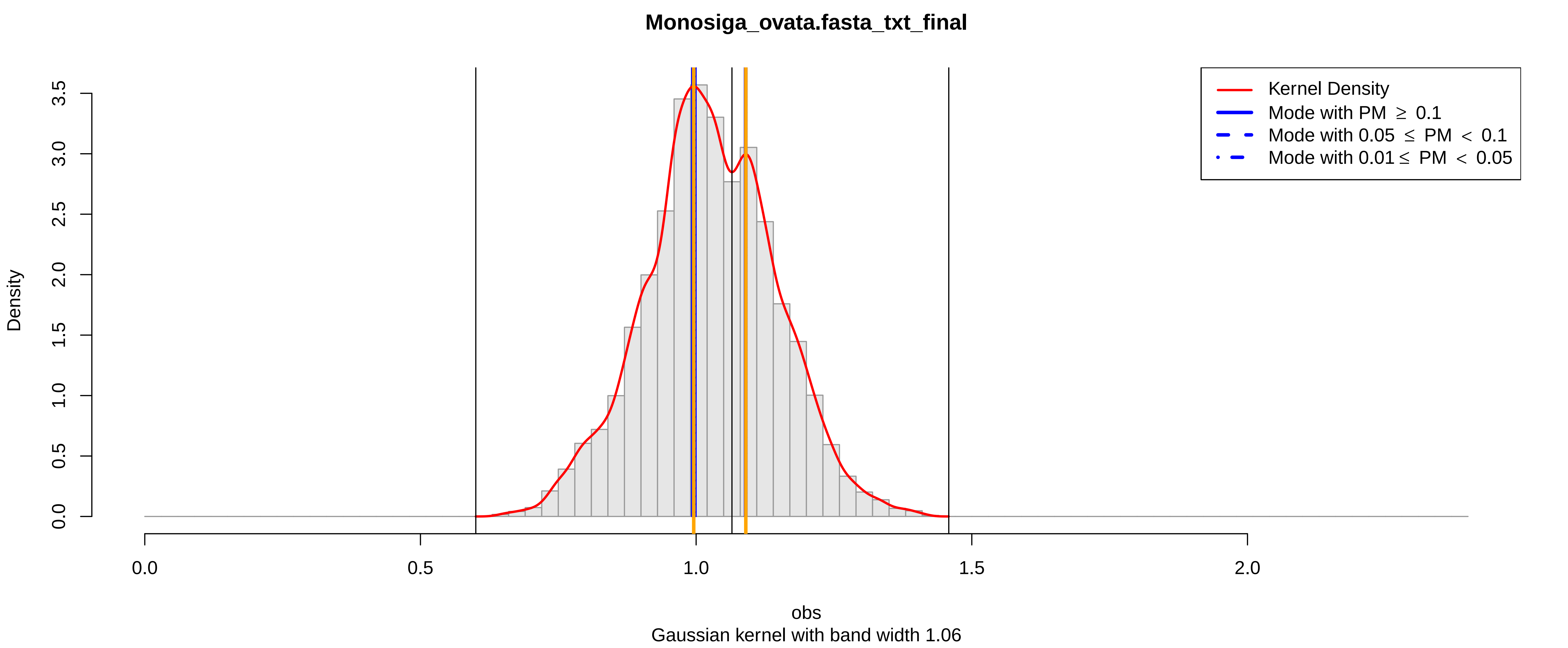
Monosiga_ovata.fasta_txt_final
Kernel Density
Mode with PM ≥ 0.1
Mode with 0.05 ≤ PM < 0.1
Mode with 0.01 ≤ PM < 0.05
Density
0.0
0.5
1.0
1.5
2.0
2.5
3.0
3.5
obs
Gaussian kernel with band width 1.06

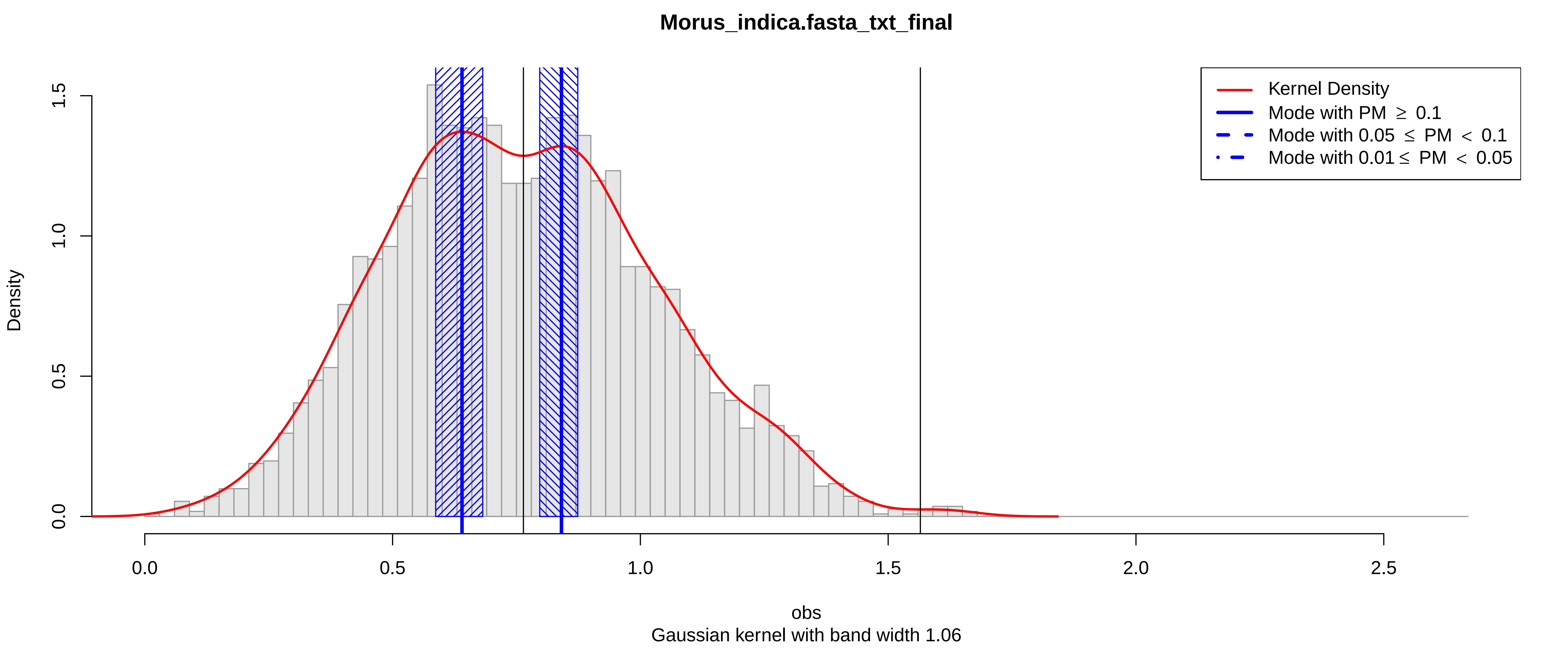
Morus_indica.fasta_txt_final
Density
0.0
0.5
1.0
1.5
0.0
0.5
1.0
1.5
2.0
2.5
obs
Gaussian kernel with band width 1.06
Kernel Density
Mode with PM ≥ 0.1
Mode with 0.05 ≤ PM < 0.1
Mode with 0.01 ≤ PM < 0.05

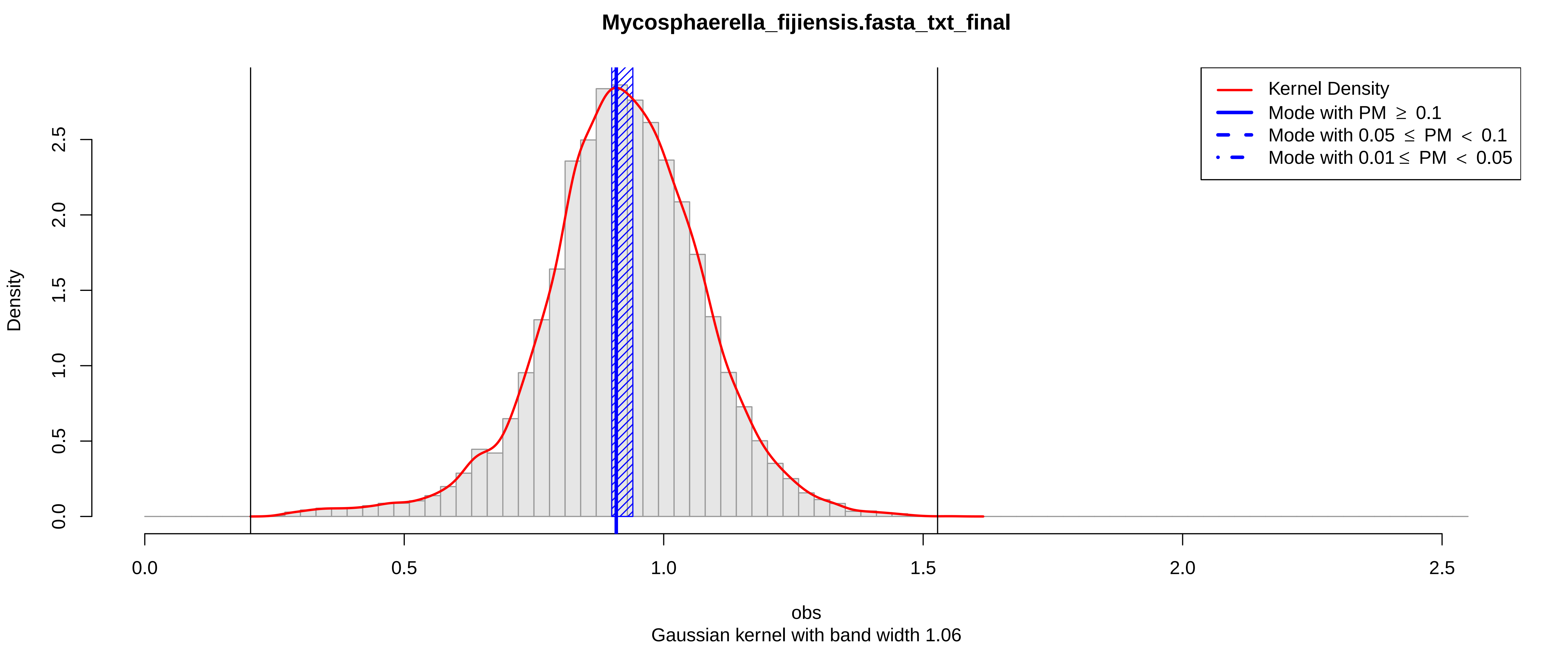
Mycosphaerella_fijiensis.fasta_txt_final
Kernel Density
Mode with PM ≥ 0.1
Mode with 0.05 ≤ PM < 0.1
Mode with 0.01 ≤ PM < 0.05
Density
0.0
0.5
1.0
1.5
2.0
2.5
obs
Gaussian kernel with band width 1.06

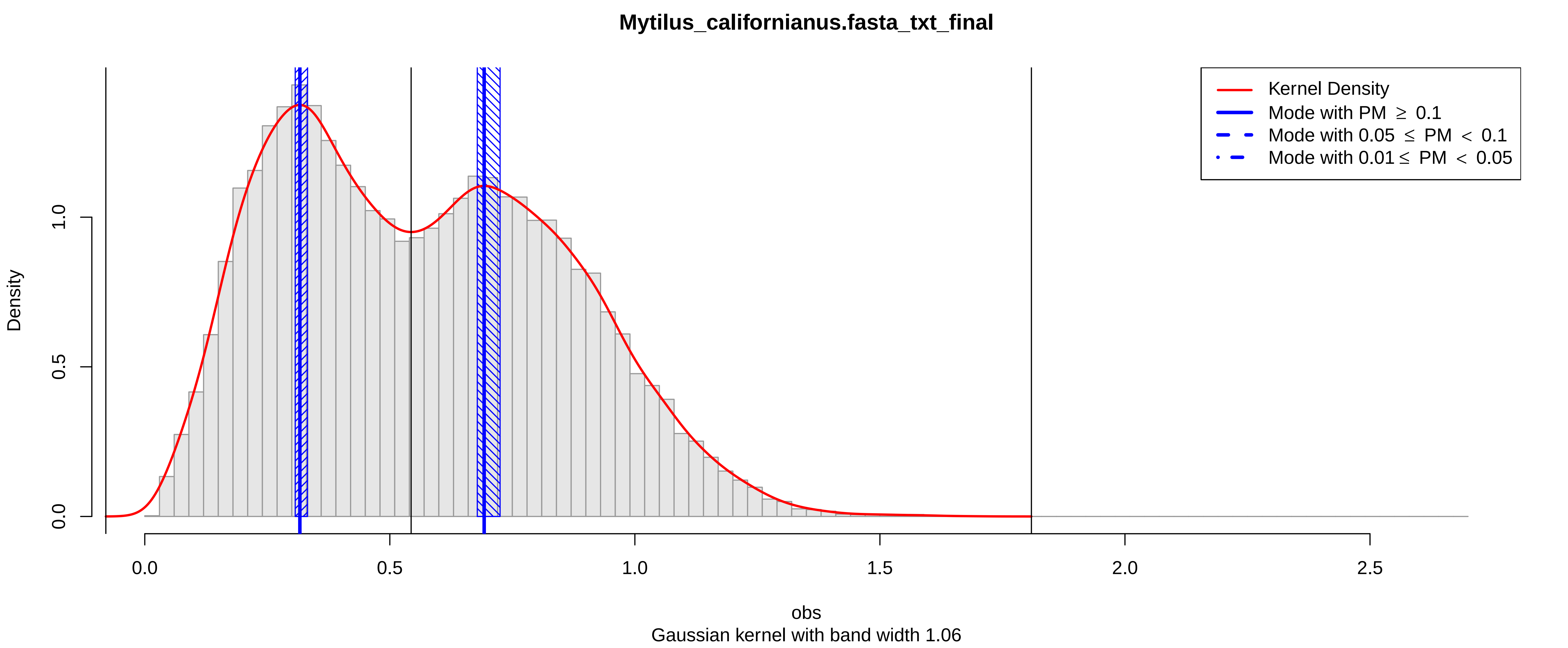
Mytilus_californianus.fasta_txt_final
Kernel Density
Mode with PM ≥ 0.1
Mode with 0.05 ≤ PM < 0.1
Mode with 0.01 ≤ PM < 0.05
Density
0.0
0.5
1.0
0.0
0.5
1.0
1.5
2.0
2.5
obs
Gaussian kernel with band width 1.06

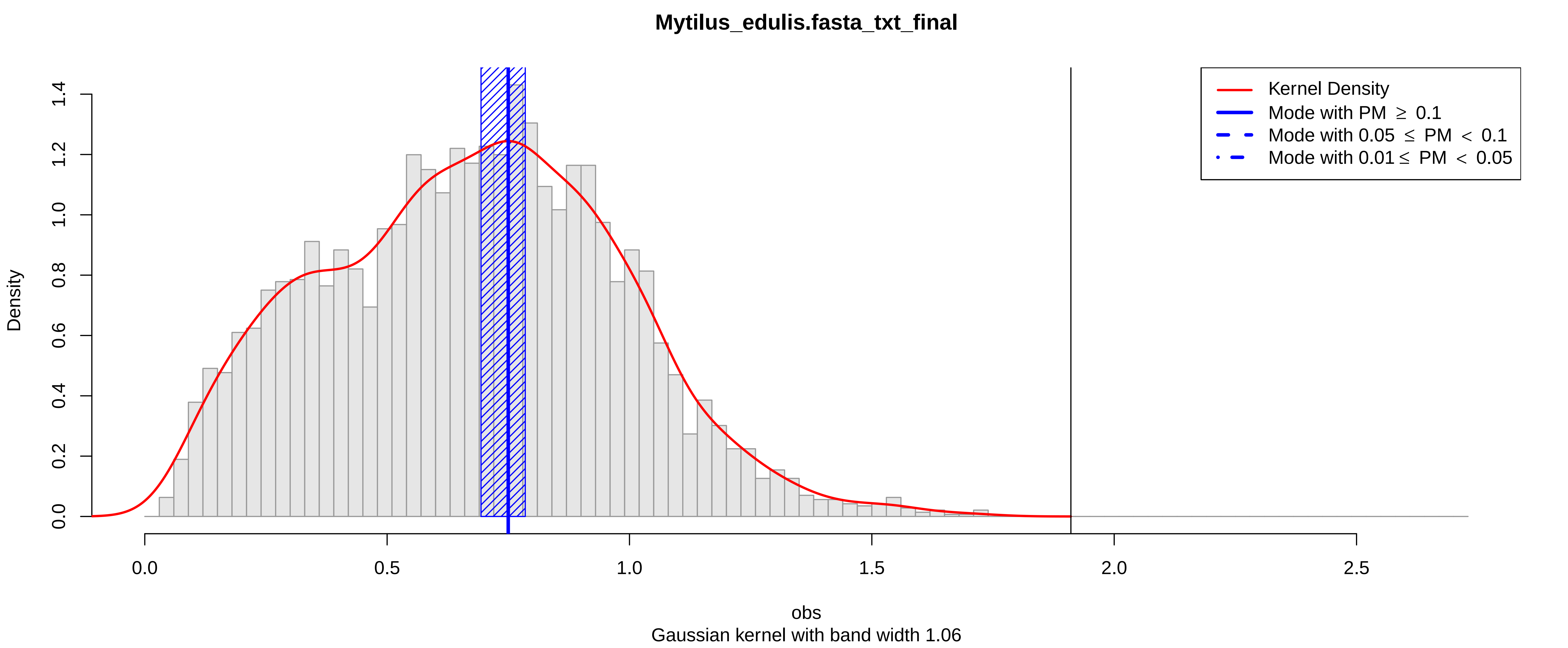

Mytilus_edulis.fasta_txt_final
Density
0.0
0.2
0.4
0.6
0.8
1.0
1.2
1.4
0.0
0.5
1.0
1.5
2.0
2.5
obs
Gaussian kernel with band width 1.06
Kernel Density
Mode with PM ≥ 0.1
Mode with 0.05 ≤ PM < 0.1
Mode with 0.01 ≤ PM < 0.05

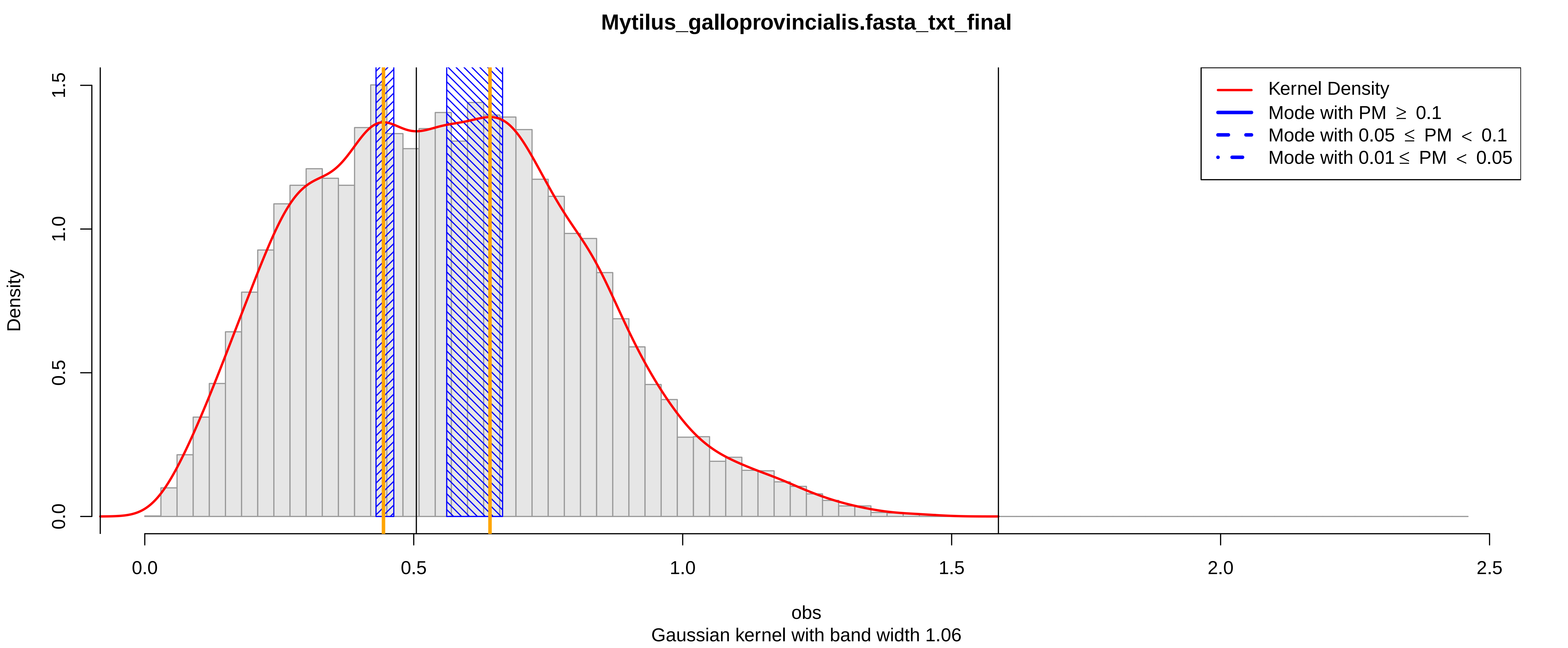

Mytilus_galloprovincialis.fasta_txt_final
Density
0.0
0.5
1.0
1.5
2.0
2.5
obs
Gaussian kernel with band width 1.06
Kernel Density
Mode with PM ≥ 0.1
Mode with 0.05 ≤ PM < 0.1
Mode with 0.01 ≤ PM < 0.05

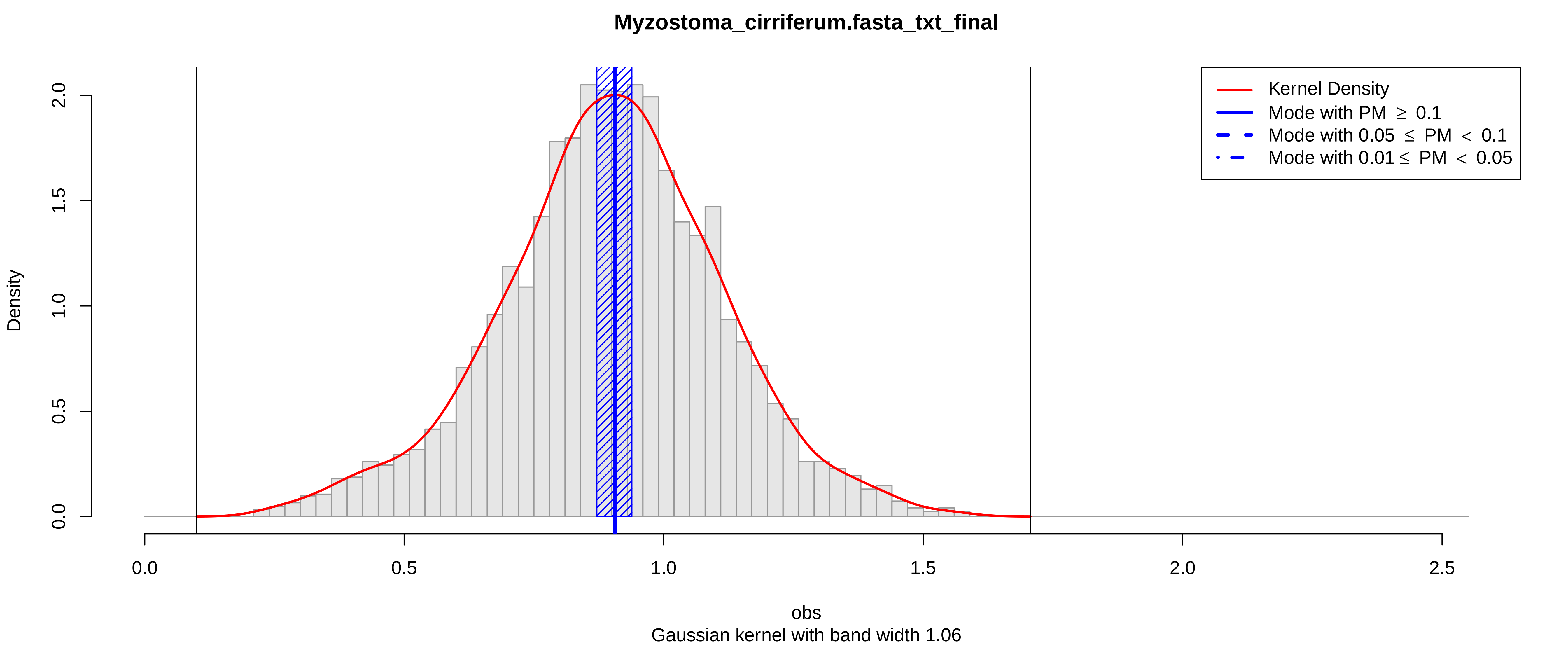
Myzostoma_cirriferum.fasta_txt_final
Kernel Density
Mode with PM ≥ 0.1
Mode with 0.05 ≤ PM < 0.1
Mode with 0.01 ≤ PM < 0.05
Density
0.0
0.5
1.0
1.5
2.0
0.0
0.5
1.0
1.5
2.0
2.5
obs
Gaussian kernel with band width 1.06

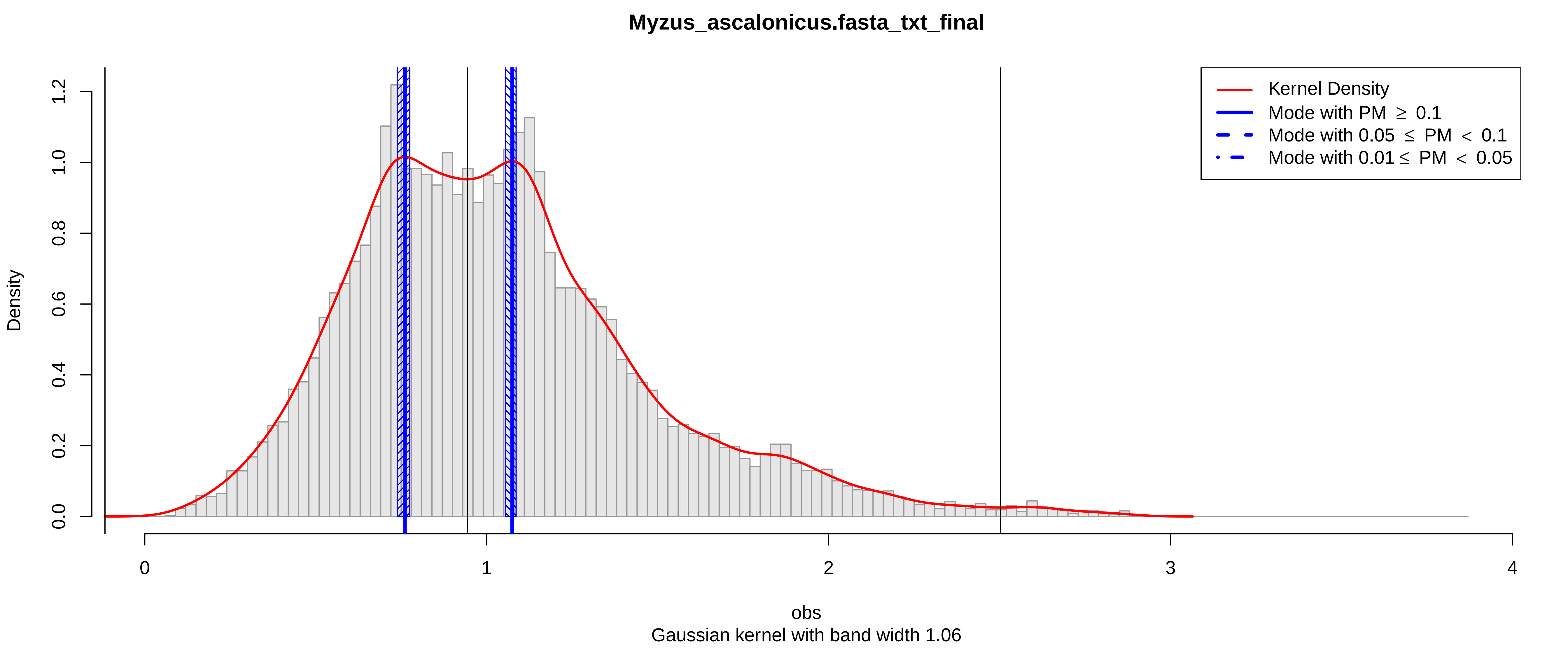
Myzus_ascalonicus.fasta_txt_final
Density
0.0
0.2
0.4
0.6
0.8
1.0
1.2
0
1
2
3
4
obs
Gaussian kernel with band width 1.06
Kernel Density
Mode with PM ≥ 0.1
Mode with 0.05 ≤ PM < 0.1
Mode with 0.01 ≤ PM < 0.05

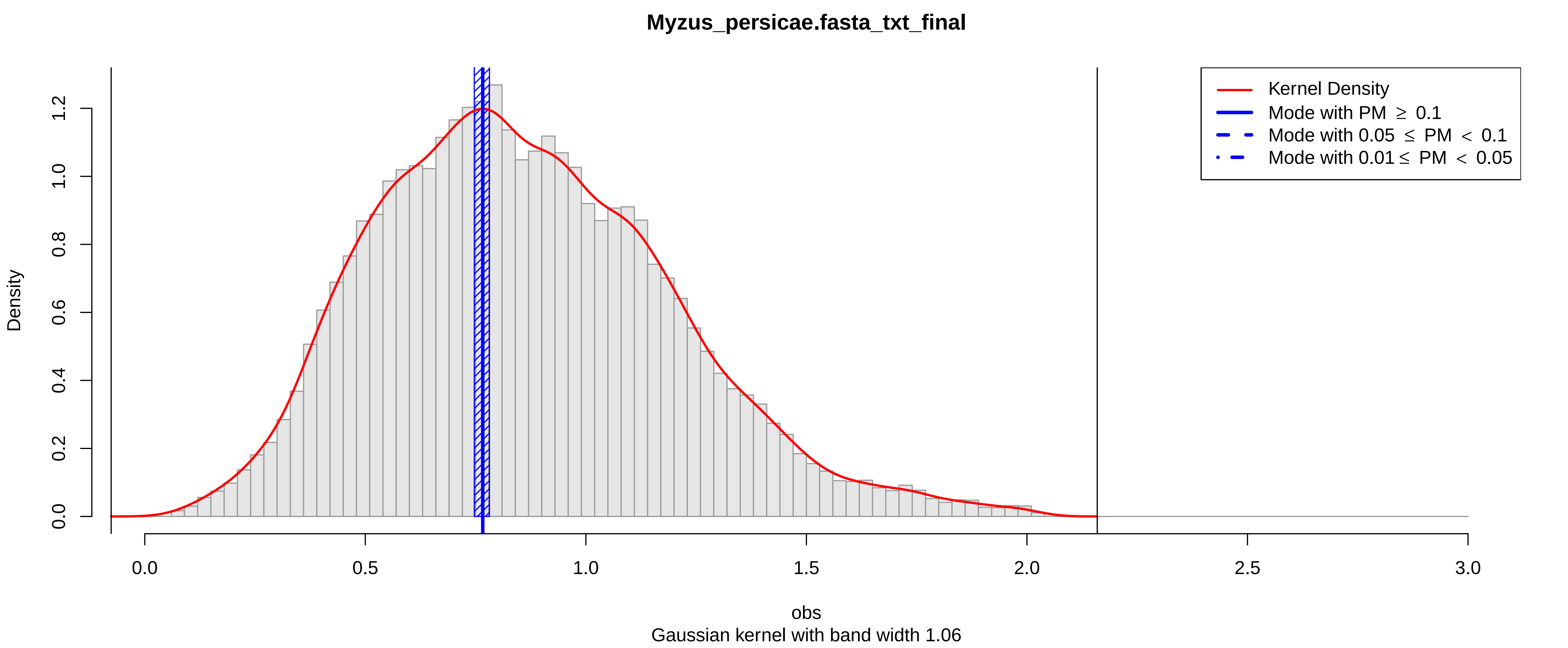

Myzus_persicae.fasta_txt_final
Density
0.0
0.2
0.4
0.6
0.8
1.0
1.2
0.0
0.5
1.0
1.5
2.0
2.5
3.0
obs
Gaussian kernel with band width 1.06
Kernel Density
Mode with PM ≥ 0.1
Mode with 0.05 ≤ PM < 0.1
Mode with 0.01 ≤ PM < 0.05

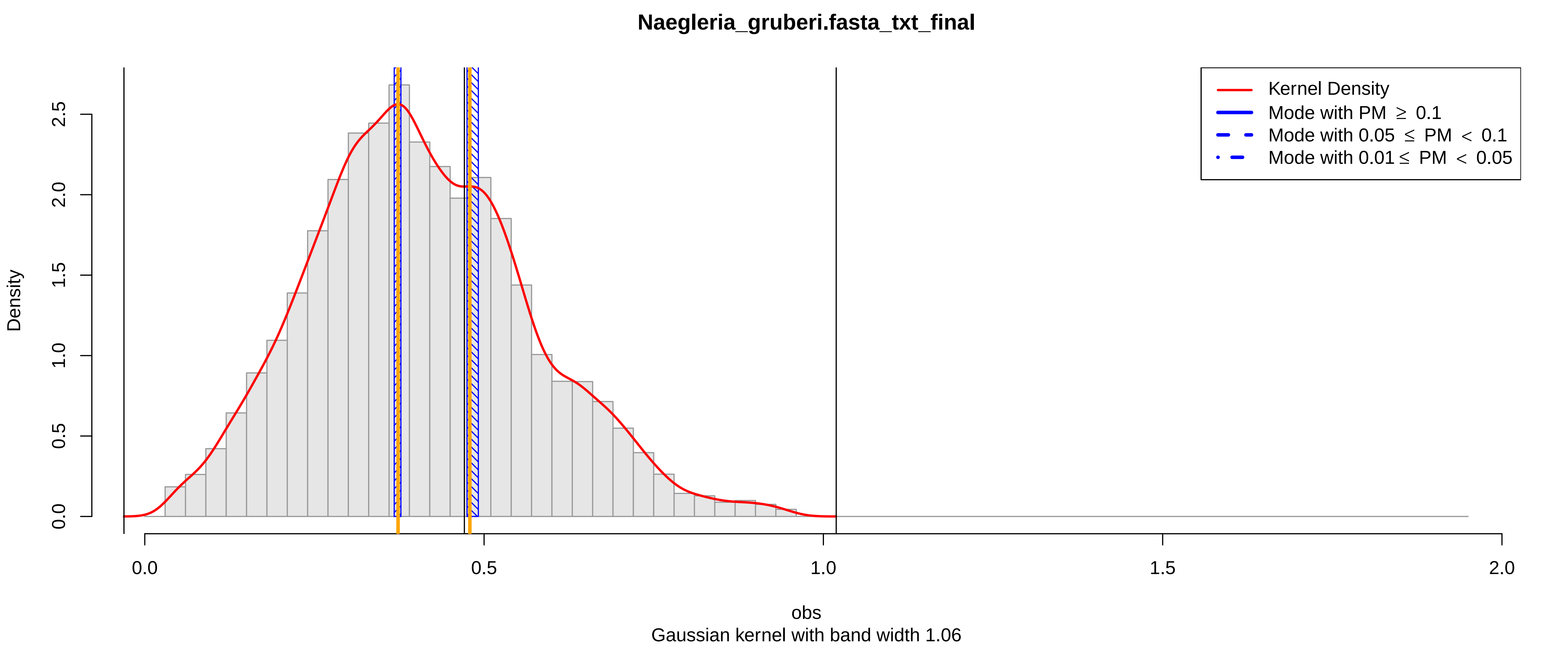

Naegleria_gruberi.fasta_txt_final
Kernel Density
Mode with PM ≥ 0.1
Mode with 0.05 ≤ PM < 0.1
Mode with 0.01 ≤ PM < 0.05
Density
0.0
0.5
1.0
1.5
2.0
2.5
0.0
0.5
1.0
1.5
2.0
obs
Gaussian kernel with band width 1.06

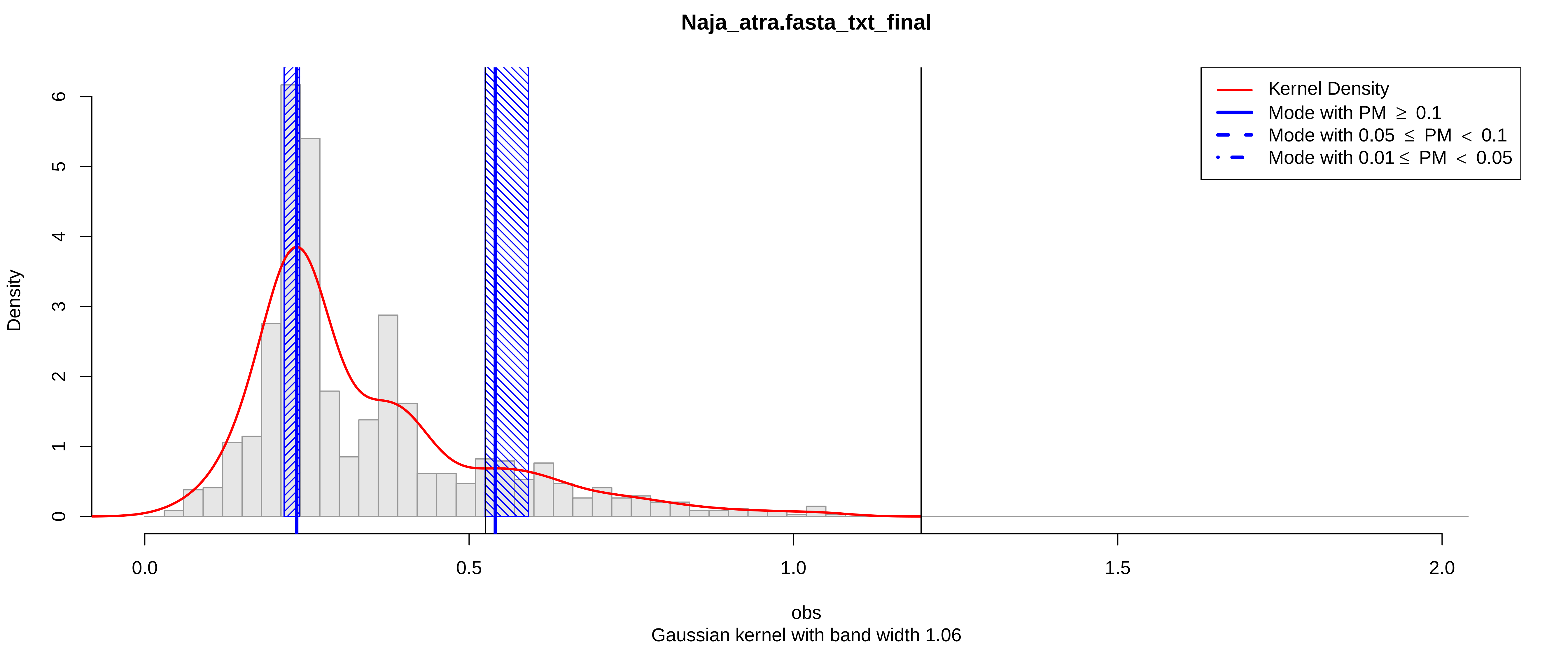
Naja_atra.fasta_txt_final
Kernel Density
Mode with PM ≥ 0.1
Mode with 0.05 ≤ PM < 0.1
Mode with 0.01 ≤ PM < 0.05
Density
0
1
2
3
4
5
6
0.0
0.5
1.0
1.5
2.0
obs
Gaussian kernel with band width 1.06

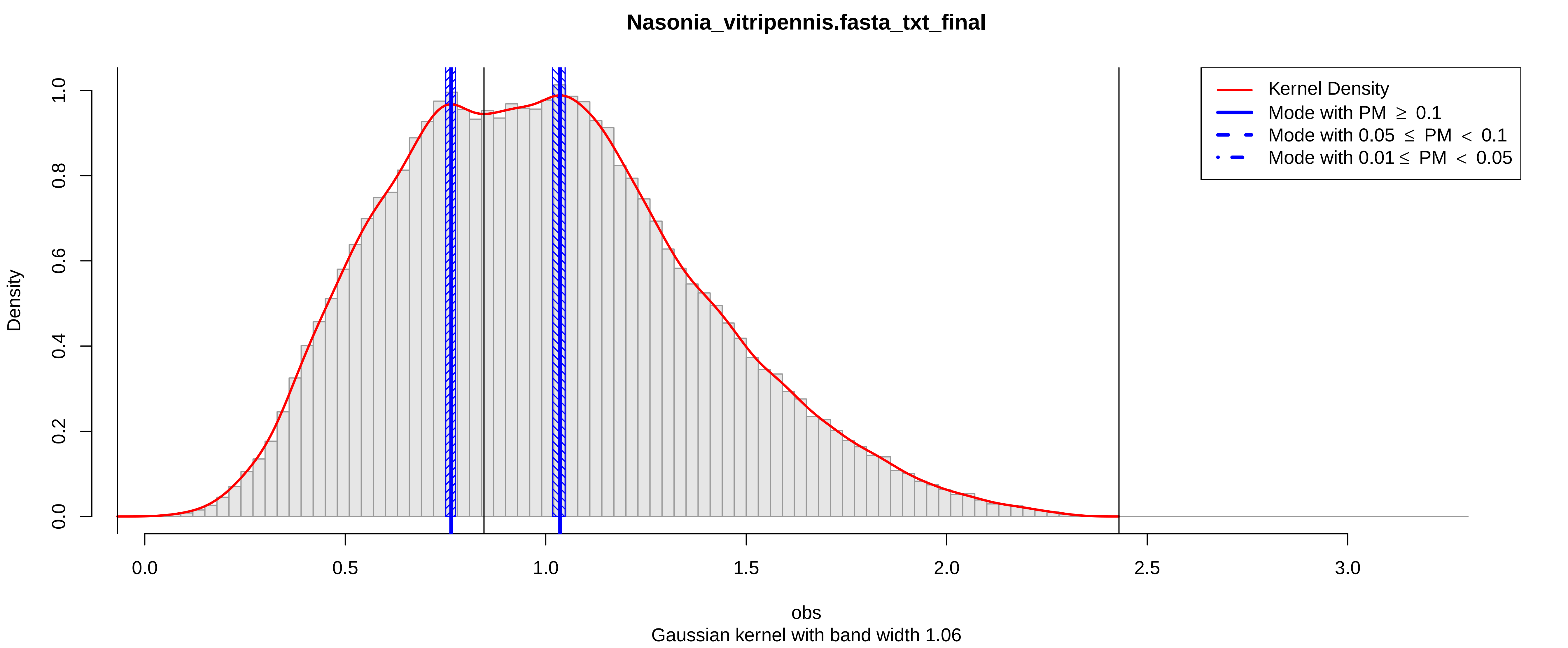

Nasonia_vitripennis.fasta_txt_final
Kernel Density
Mode with PM ≥ 0.1
Mode with 0.05 ≤ PM < 0.1
Mode with 0.01 ≤ PM < 0.05
Density
0.0
0.2
0.4
0.6
0.8
1.0
0.0
0.5
1.0
1.5
2.0
2.5
3.0
obs
Gaussian kernel with band width 1.06

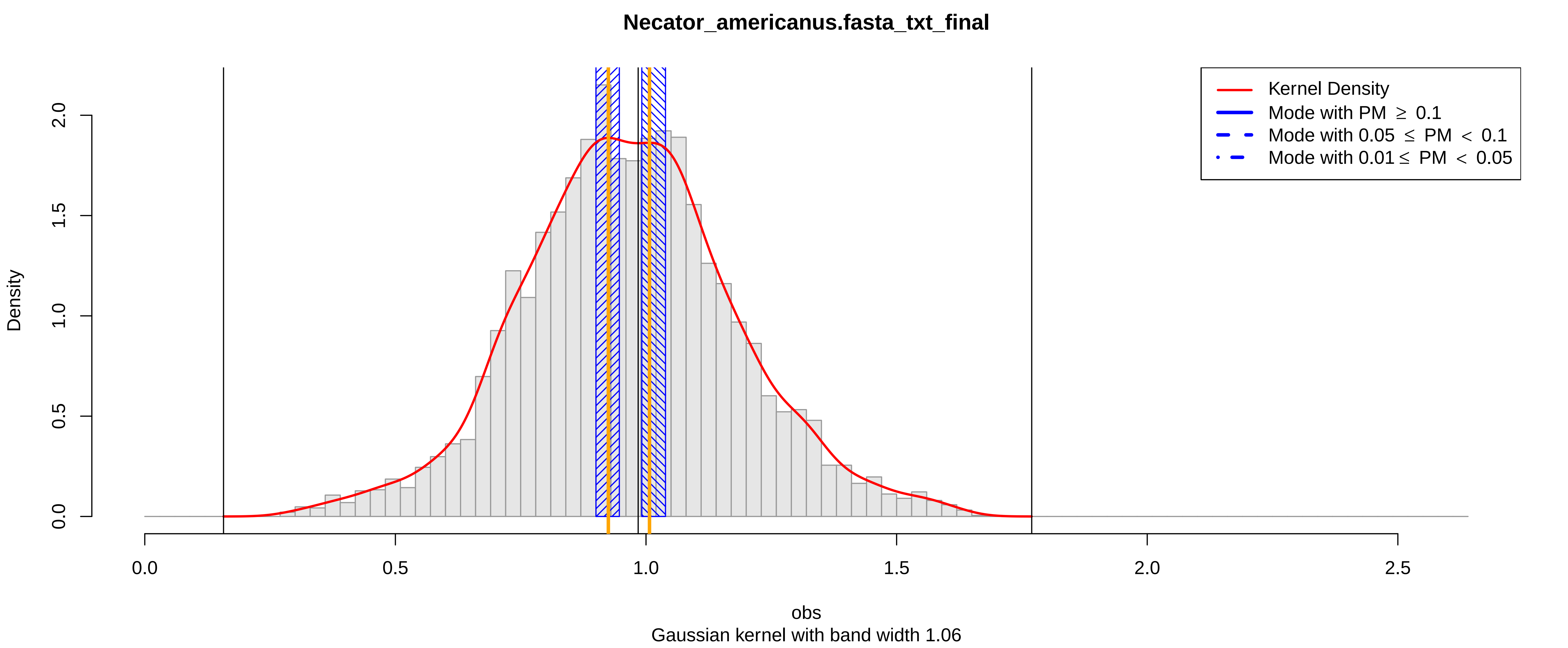

Necator_americanus.fasta_txt_final
Kernel Density
Mode with PM ≥ 0.1
Mode with 0.05 ≤ PM < 0.1
Mode with 0.01 ≤ PM < 0.05
Density
0.0
0.5
1.0
1.5
2.0
2.5
obs
Gaussian kernel with band width 1.06

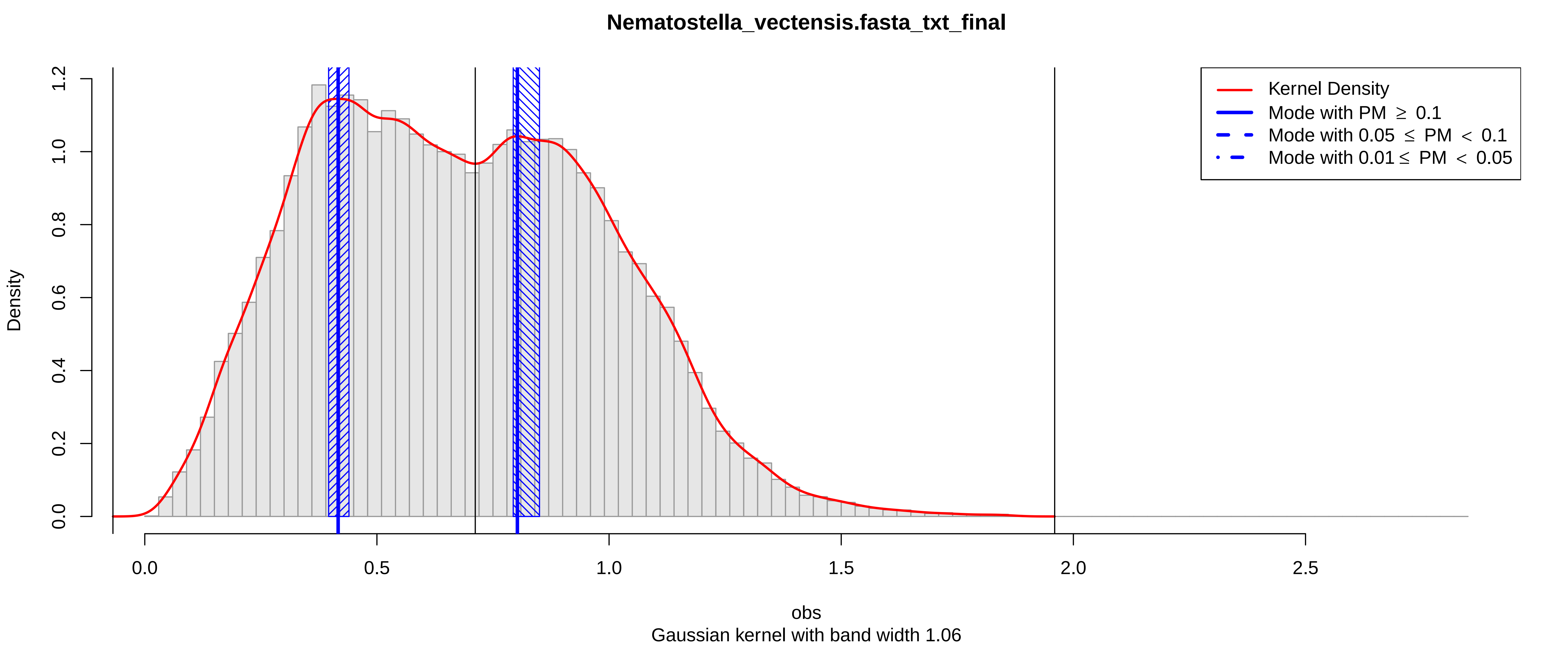

Nematostella_vectensis.fasta_txt_final
Density
0.0
0.2
0.4
0.6
0.8
1.0
1.2
0.0
0.5
1.0
1.5
2.0
2.5
obs
Gaussian kernel with band width 1.06
Kernel Density
Mode with PM ≥ 0.1
Mode with 0.05 ≤ PM < 0.1
Mode with 0.01 ≤ PM < 0.05

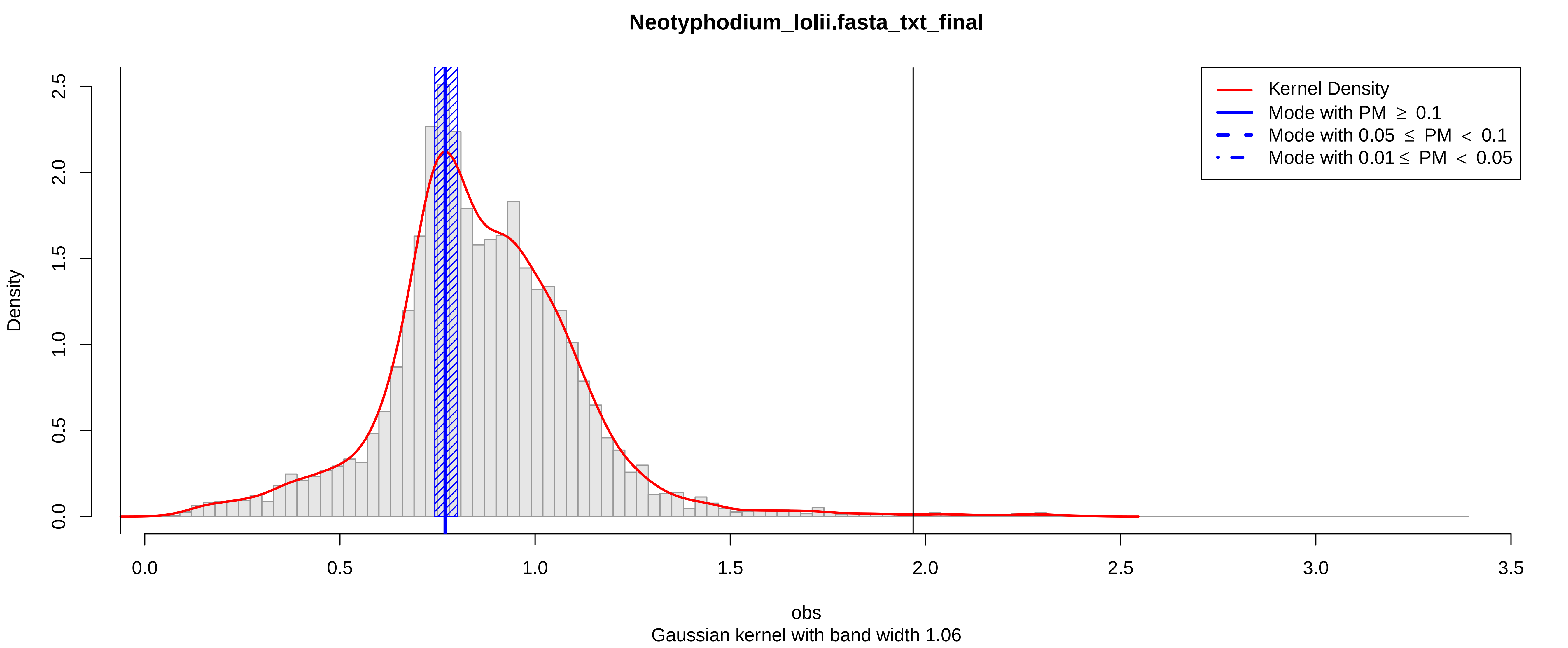
Neotyphodium_lolii.fasta_txt_final
Density
0.0
0.5
1.0
1.5
2.0
2.5
3.0
3.5
obs
Gaussian kernel with band width 1.06
Kernel Density
Mode with PM ≥ 0.1
Mode with 0.05 ≤ PM < 0.1
Mode with 0.01 ≤ PM < 0.05

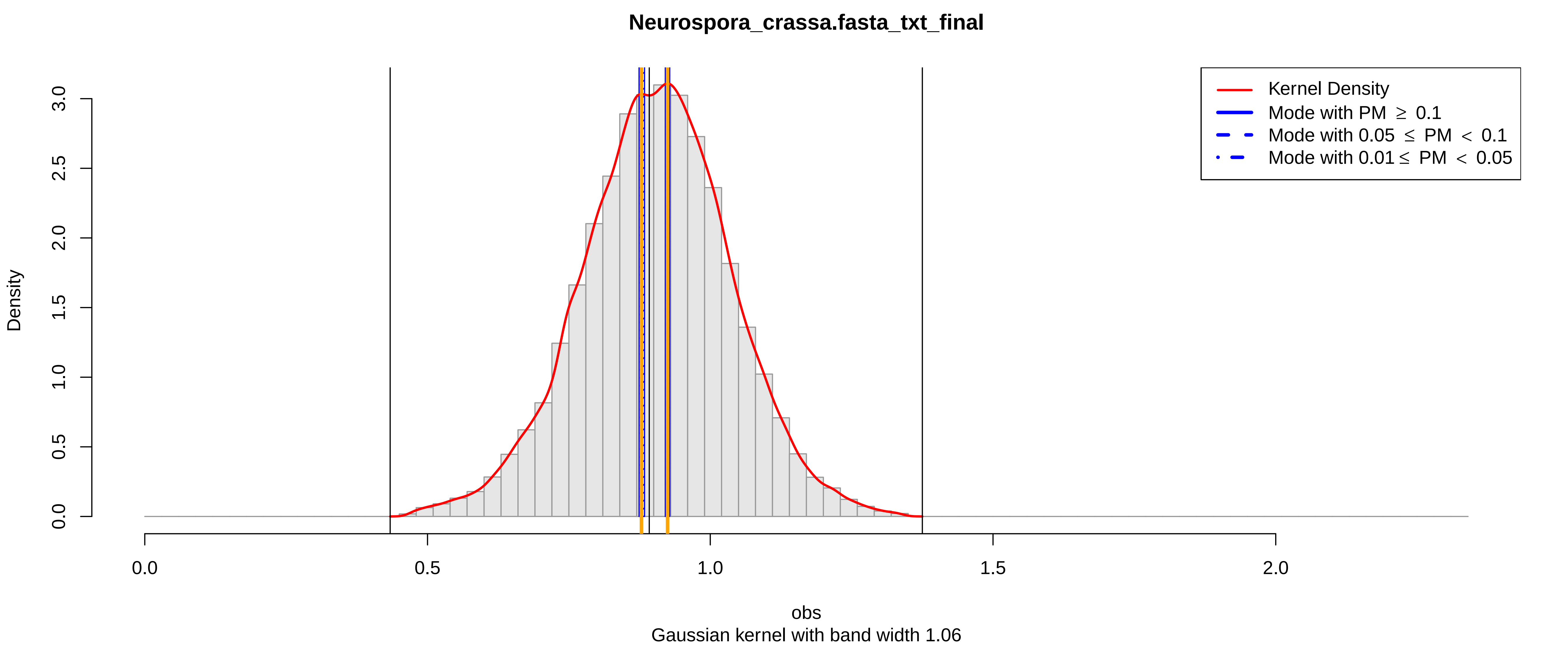
Neurospora_crassa.fasta_txt_final
Kernel Density
Mode with PM ≥ 0.1
Mode with 0.05 ≤ PM < 0.1
Mode with 0.01 ≤ PM < 0.05
Density
0.0
0.5
1.0
1.5
2.0
2.5
3.0
obs
Gaussian kernel with band width 1.06

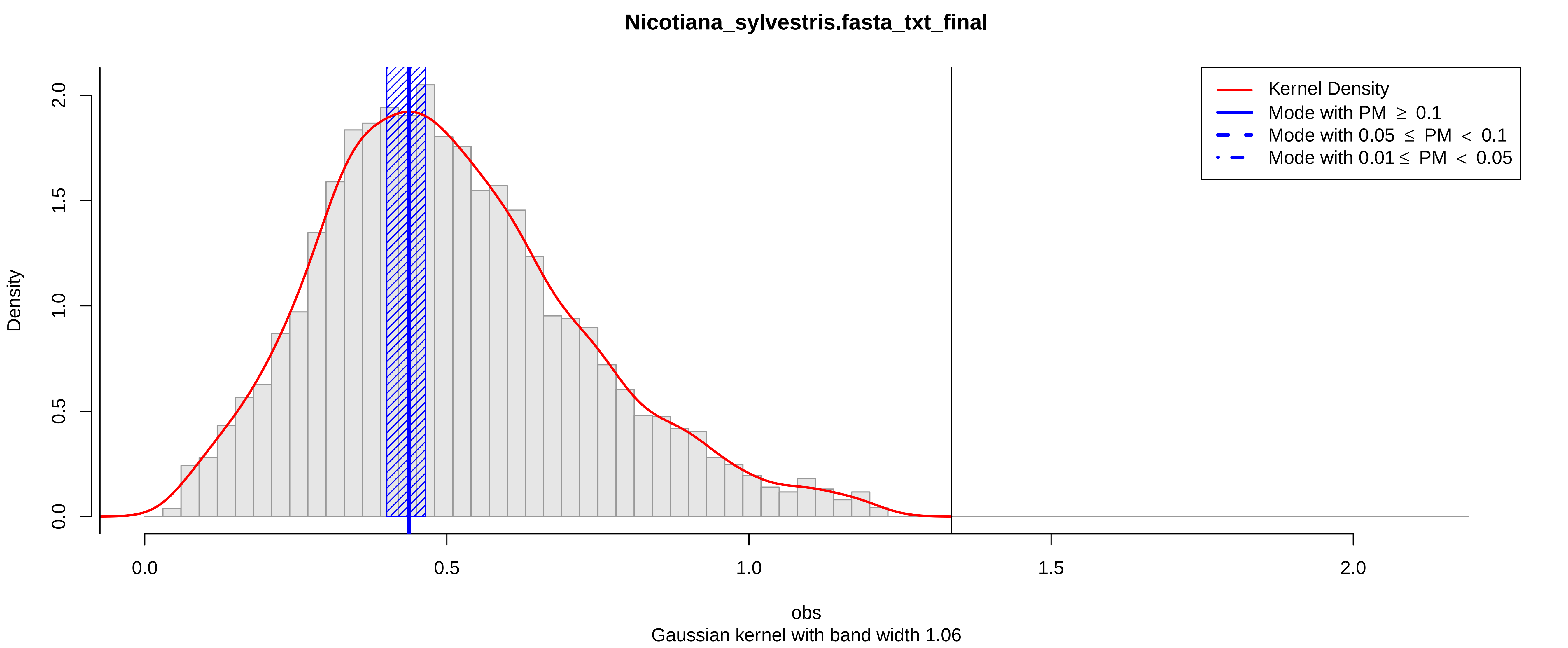

Nicotiana_sylvestris.fasta_txt_final
Kernel Density
Mode with PM ≥ 0.1
Mode with 0.05 ≤ PM < 0.1
Mode with 0.01 ≤ PM < 0.05
Density
0.0
0.5
1.0
1.5
2.0
obs
Gaussian kernel with band width 1.06

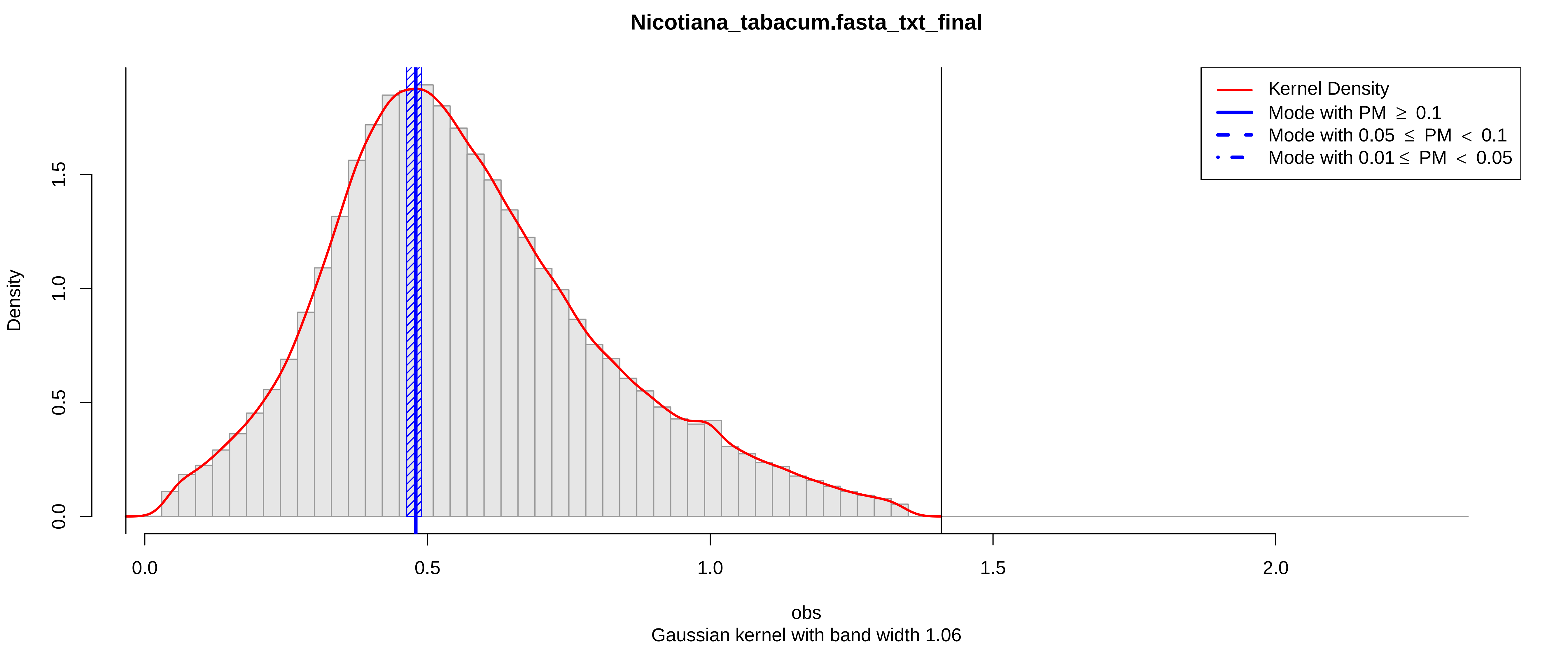
Nicotiana_tabacum.fasta_txt_final
Kernel Density
Mode with PM ≥ 0.1
Mode with 0.05 ≤ PM < 0.1
Mode with 0.01 ≤ PM < 0.05
Density
0.0
0.5
1.0
1.5
0.0
0.5
1.0
1.5
2.0
obs
Gaussian kernel with band width 1.06

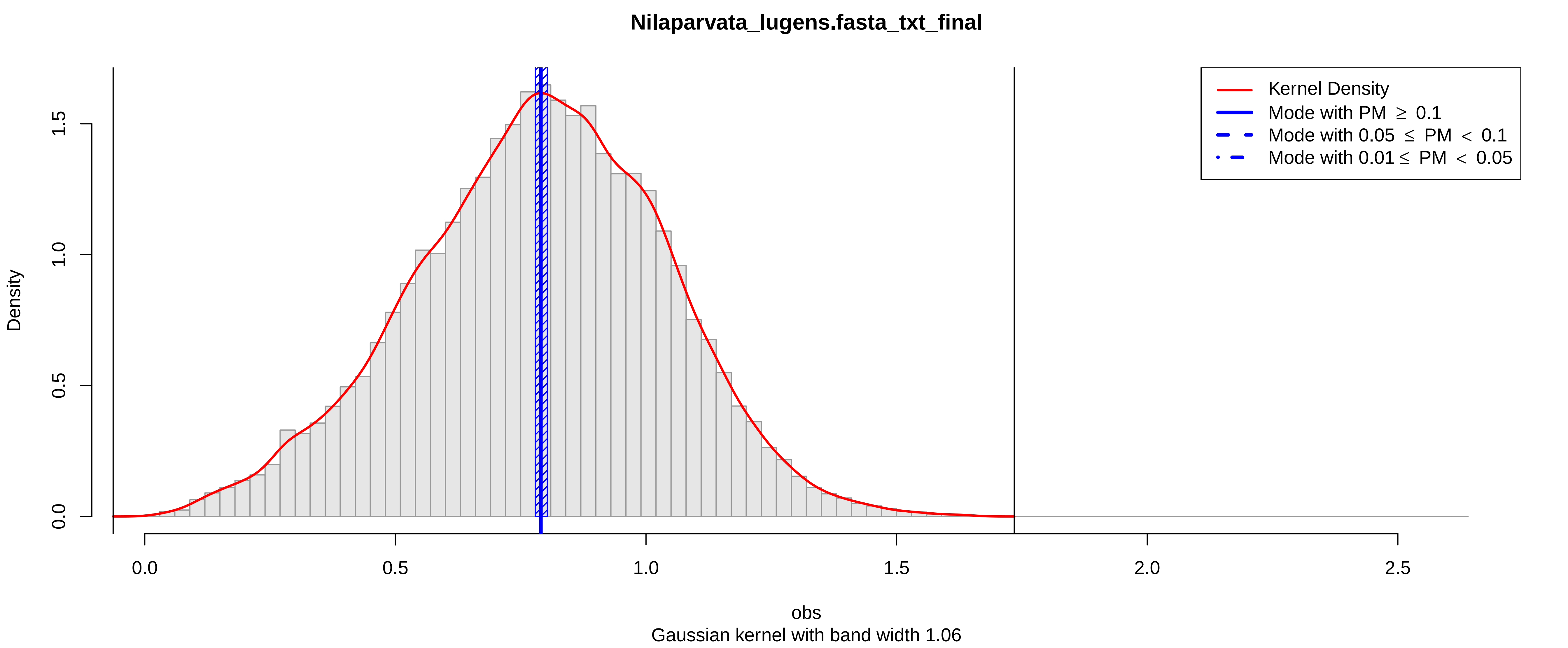
Nilaparvata_lugens.fasta_txt_final
Kernel Density
Mode with PM ≥ 0.1
Mode with 0.05 ≤ PM < 0.1
Mode with 0.01 ≤ PM < 0.05
Density
0.0
0.5
1.0
1.5
0.0
0.5
1.0
1.5
2.0
2.5
obs
Gaussian kernel with band width 1.06

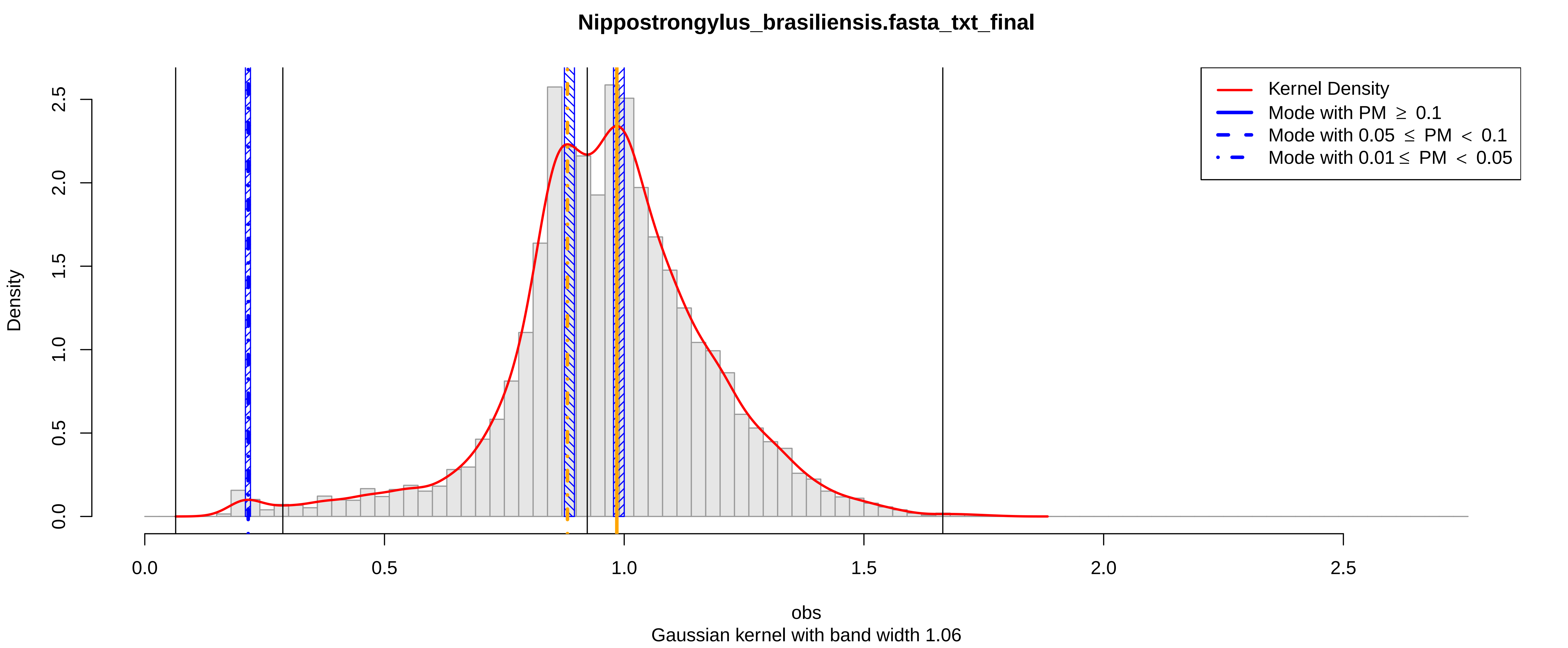

Nippostrongylus_brasiliensis.fasta_txt_final
Kernel Density
Mode with PM ≥ 0.1
Mode with 0.05 ≤ PM < 0.1
Mode with 0.01 ≤ PM < 0.05
Density
0.0
0.5
1.0
1.5
2.0
2.5
obs
Gaussian kernel with band width 1.06

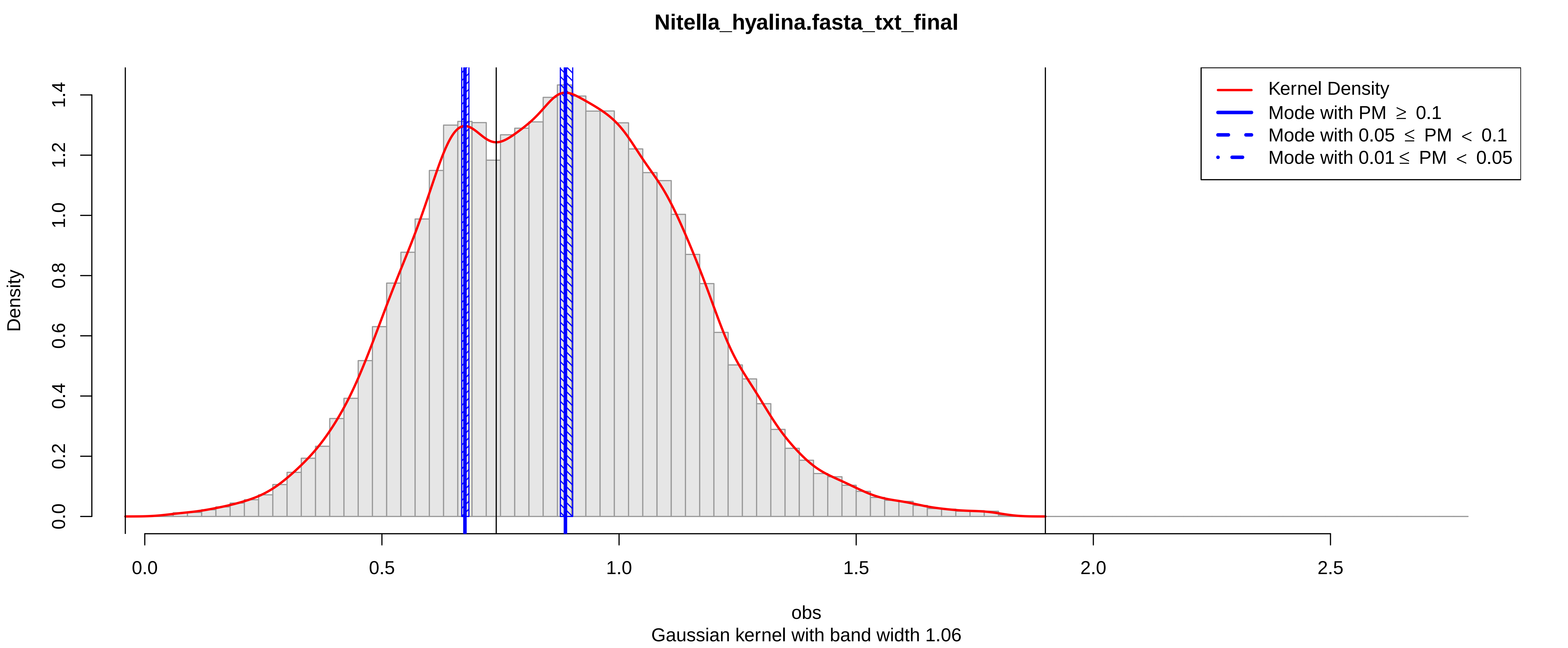
Nitella_hyalina.fasta_txt_final
Kernel Density
Mode with PM ≥ 0.1
Mode with 0.05 ≤ PM < 0.1
Mode with 0.01 ≤ PM < 0.05
Density
0.0
0.2
0.4
0.6
0.8
1.0
1.2
1.4
0.0
0.5
1.0
1.5
2.0
2.5
obs
Gaussian kernel with band width 1.06

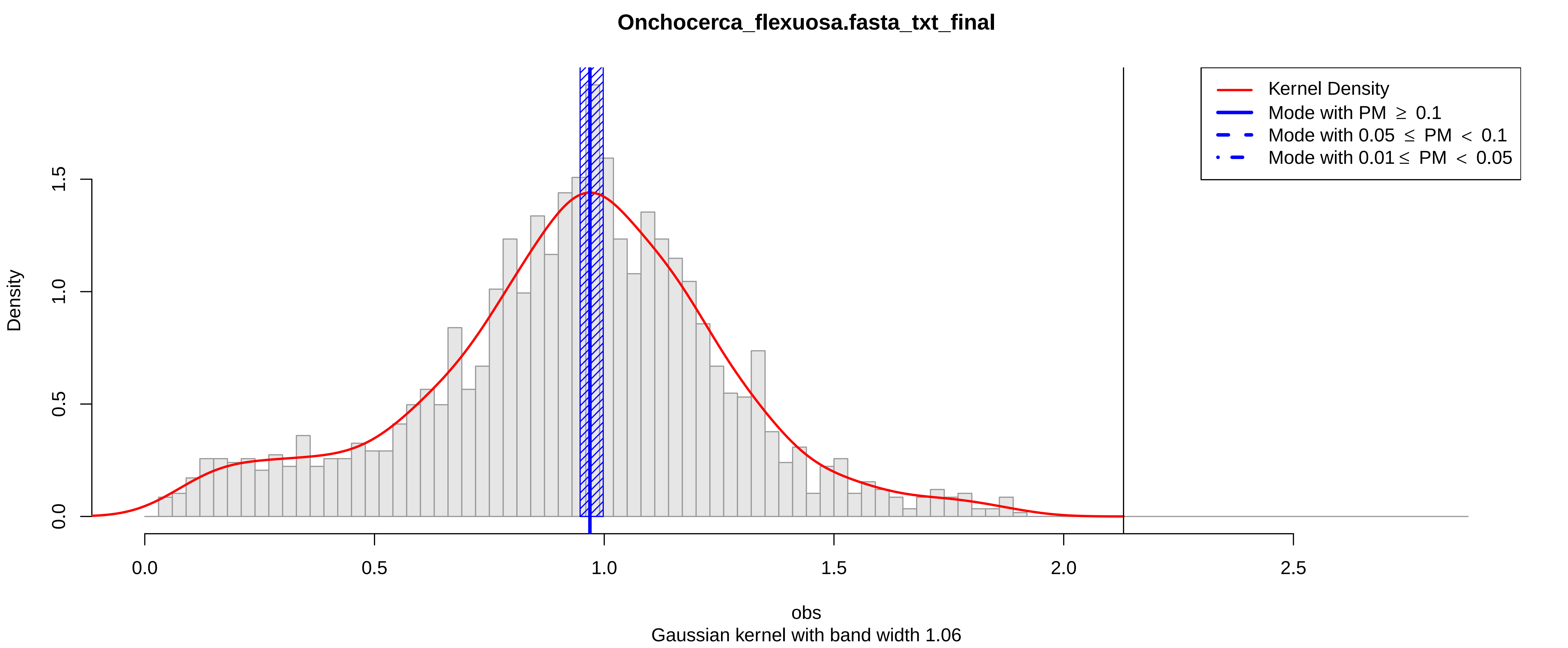

Onchocerca_flexuosa.fasta_txt_final
Kernel Density
Mode with PM ≥ 0.1
Mode with 0.05 ≤ PM < 0.1
Mode with 0.01 ≤ PM < 0.05
Density
0.0
0.5
1.0
1.5
0.0
0.5
1.0
1.5
2.0
2.5
obs
Gaussian kernel with band width 1.06

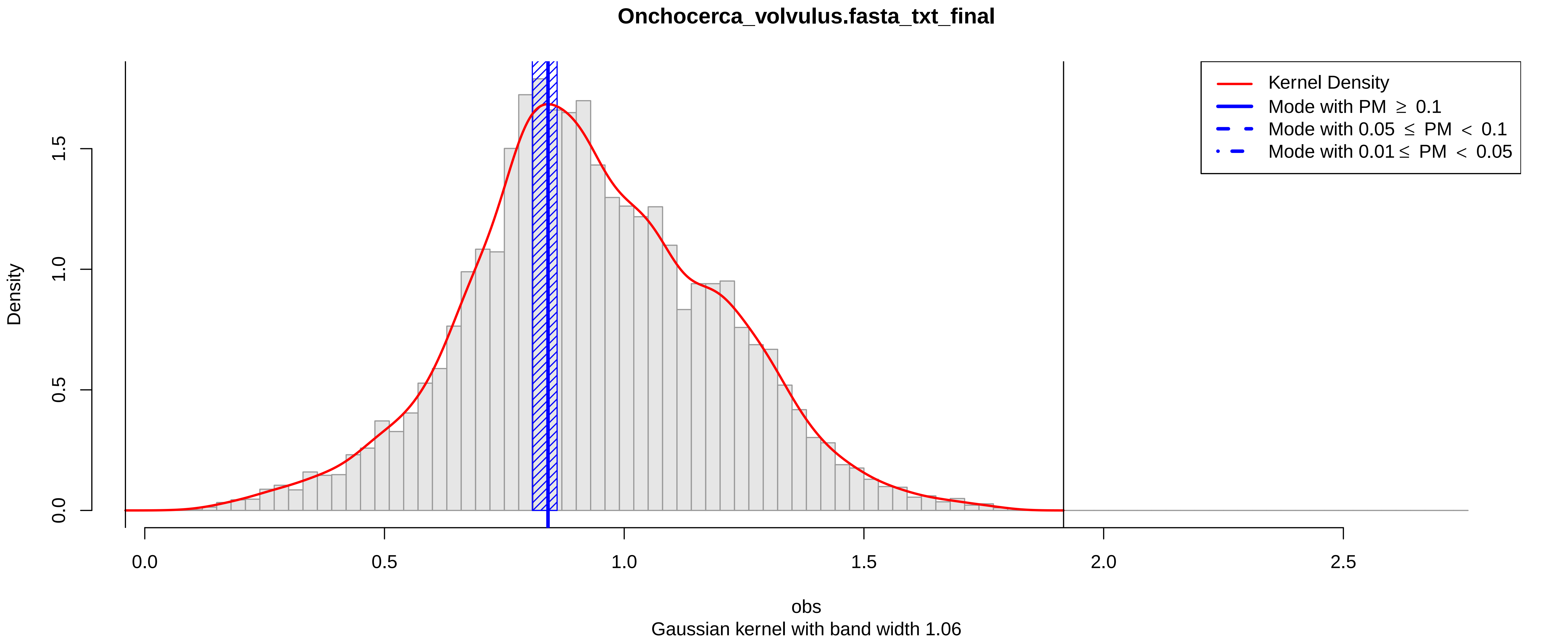

Onchocerca_volvulus.fasta_txt_final
Kernel Density
Mode with PM ≥ 0.1
Mode with 0.05 ≤ PM < 0.1
Mode with 0.01 ≤ PM < 0.05
Density
0.0
0.5
1.0
1.5
0.0
0.5
1.0
1.5
2.0
2.5
obs
Gaussian kernel with band width 1.06

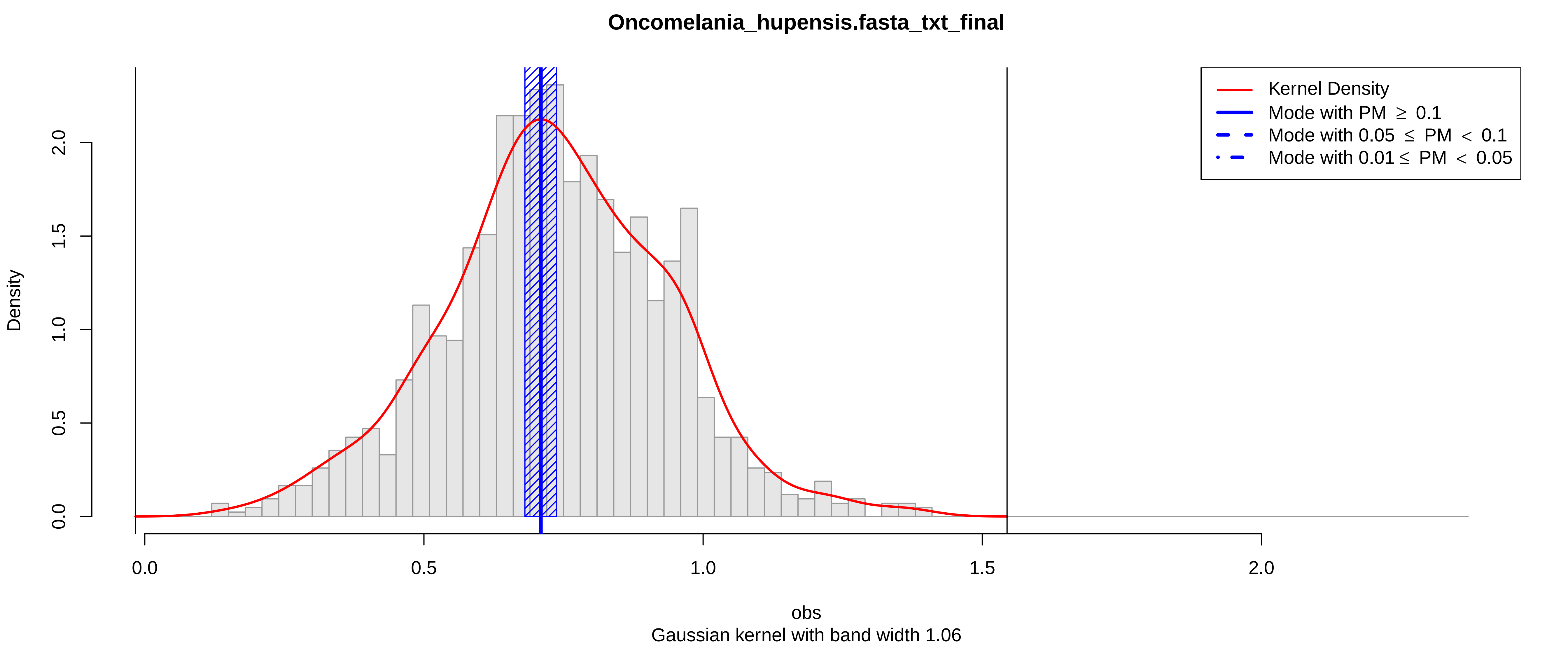

Oncomelania_hupensis.fasta_txt_final
Kernel Density
Mode with PM ≥ 0.1
Mode with 0.05 ≤ PM < 0.1
Mode with 0.01 ≤ PM < 0.05
Density
0.0
0.5
1.0
1.5
2.0
0.0
0.5
1.0
1.5
2.0
obs
Gaussian kernel with band width 1.06

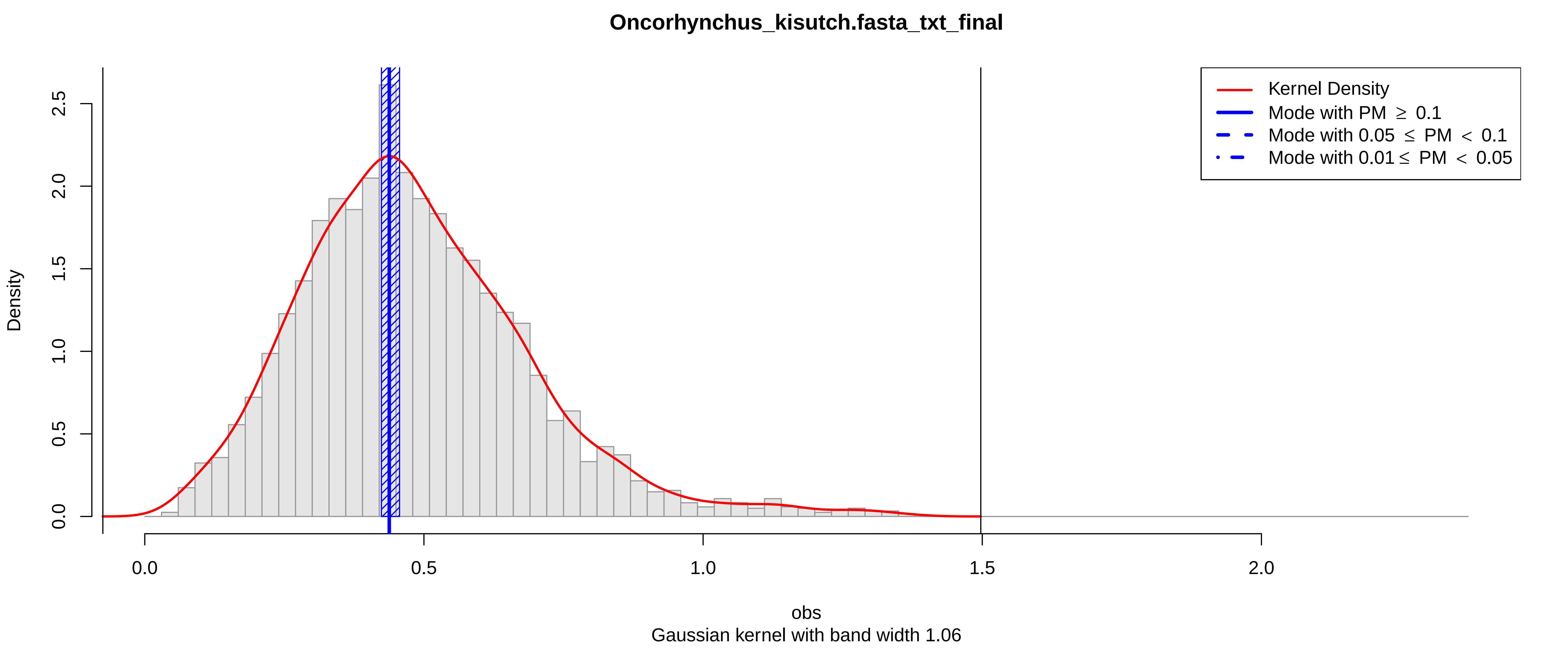
Oncorhynchus_kisutch.fasta_txt_final
Kernel Density
Mode with PM ≥ 0.1
Mode with 0.05 ≤ PM < 0.1
Mode with 0.01 ≤ PM < 0.05
Density
0.0
0.5
1.0
1.5
2.0
2.5
obs
Gaussian kernel with band width 1.06

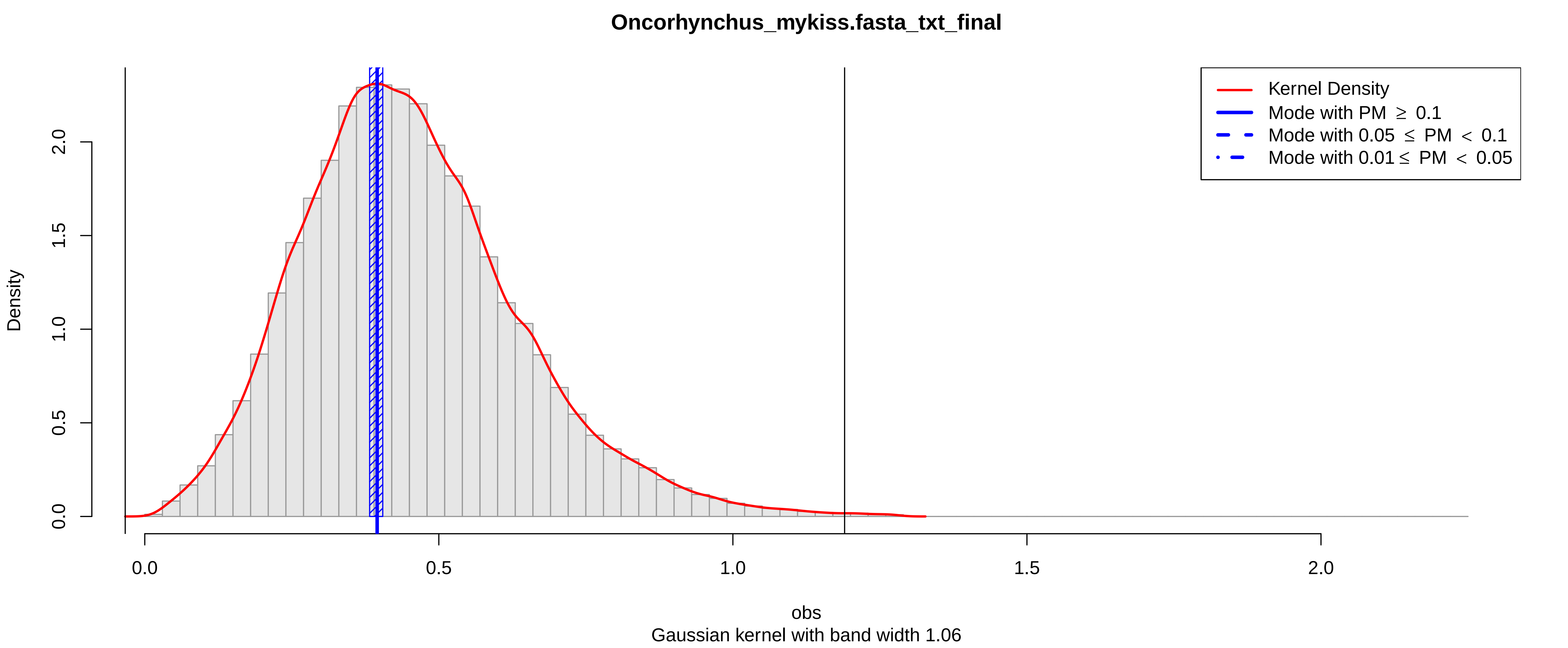
Oncorhynchus_mykiss.fasta_txt_final
Kernel Density
Mode with PM ≥ 0.1
Mode with 0.05 ≤ PM < 0.1
Mode with 0.01 ≤ PM < 0.05
Density
0.0
0.5
1.0
1.5
2.0
0.0
0.5
1.0
1.5
2.0
obs
Gaussian kernel with band width 1.06

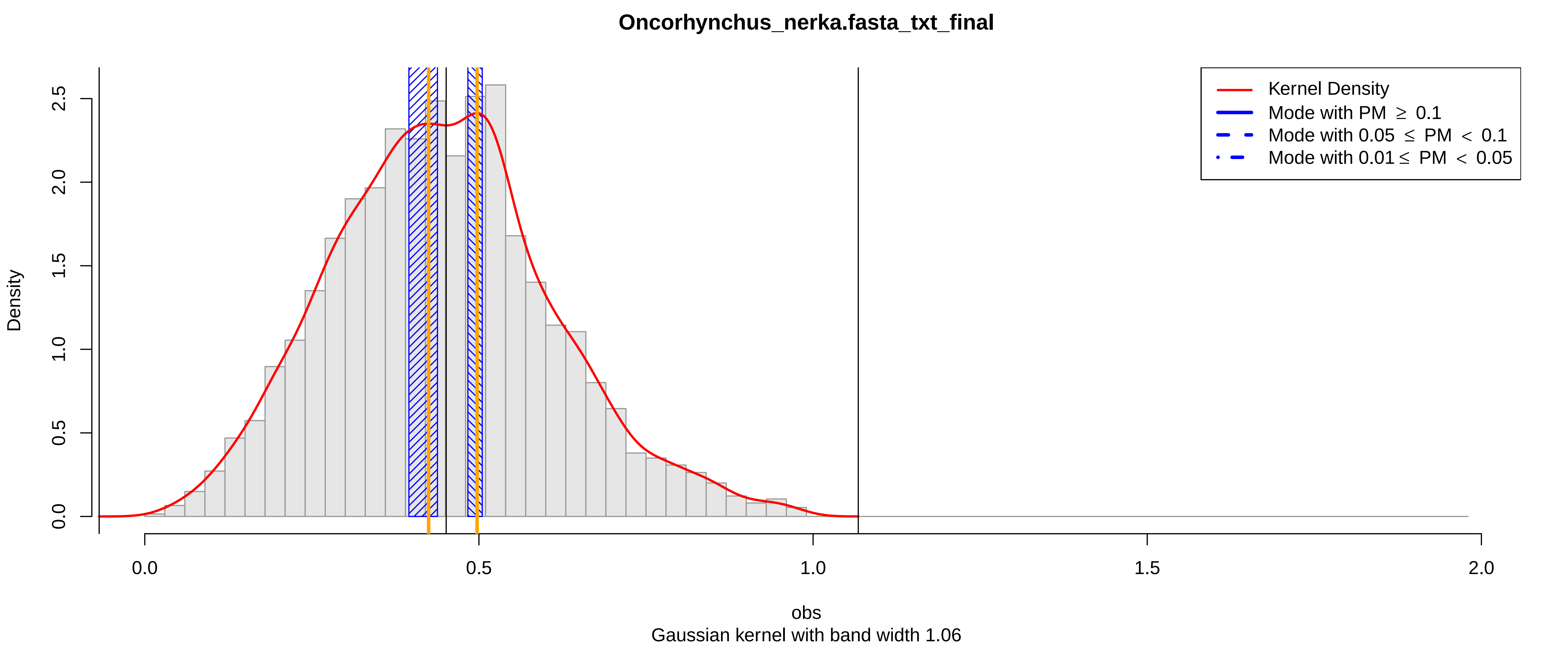

Oncorhynchus_nerka.fasta_txt_final
Kernel Density
Mode with PM ≥ 0.1
Mode with 0.05 ≤ PM < 0.1
Mode with 0.01 ≤ PM < 0.05
Density
0.0
0.5
1.0
1.5
2.0
2.5
0.0
0.5
1.0
1.5
2.0
obs
Gaussian kernel with band width 1.06

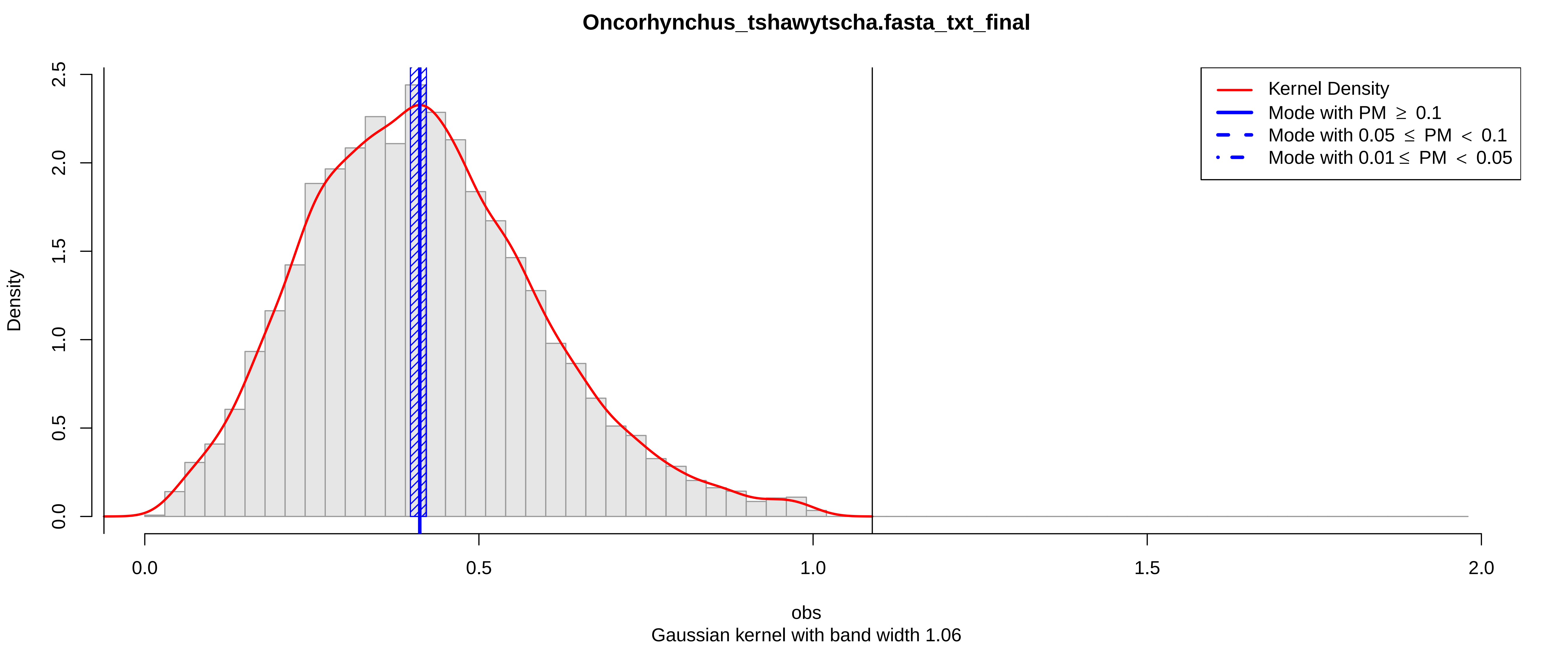

Oncorhynchus_tshawytscha.fasta_txt_final
Kernel Density
Mode with PM ≥ 0.1
Mode with 0.05 ≤ PM < 0.1
Mode with 0.01 ≤ PM < 0.05
Density
0.0
0.5
1.0
1.5
2.0
2.5
0.0
0.5
1.0
1.5
2.0
obs
Gaussian kernel with band width 1.06

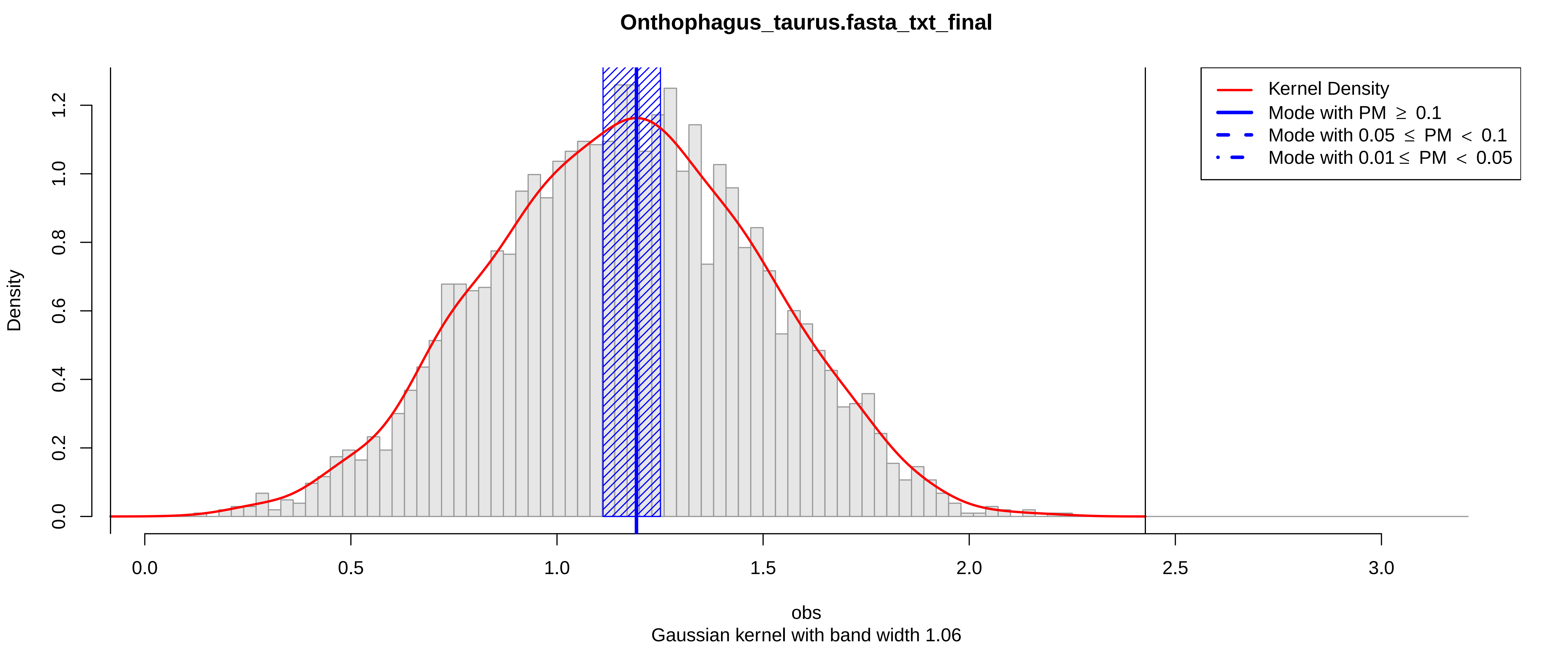
Onthophagus_taurus.fasta_txt_final
Kernel Density
Mode with PM ≥ 0.1
Mode with 0.05 ≤ PM < 0.1
Mode with 0.01 ≤ PM < 0.05
Density
0.0
0.2
0.4
0.6
0.8
1.0
1.2
0.0
0.5
1.0
1.5
2.0
2.5
3.0
obs
Gaussian kernel with band width 1.06

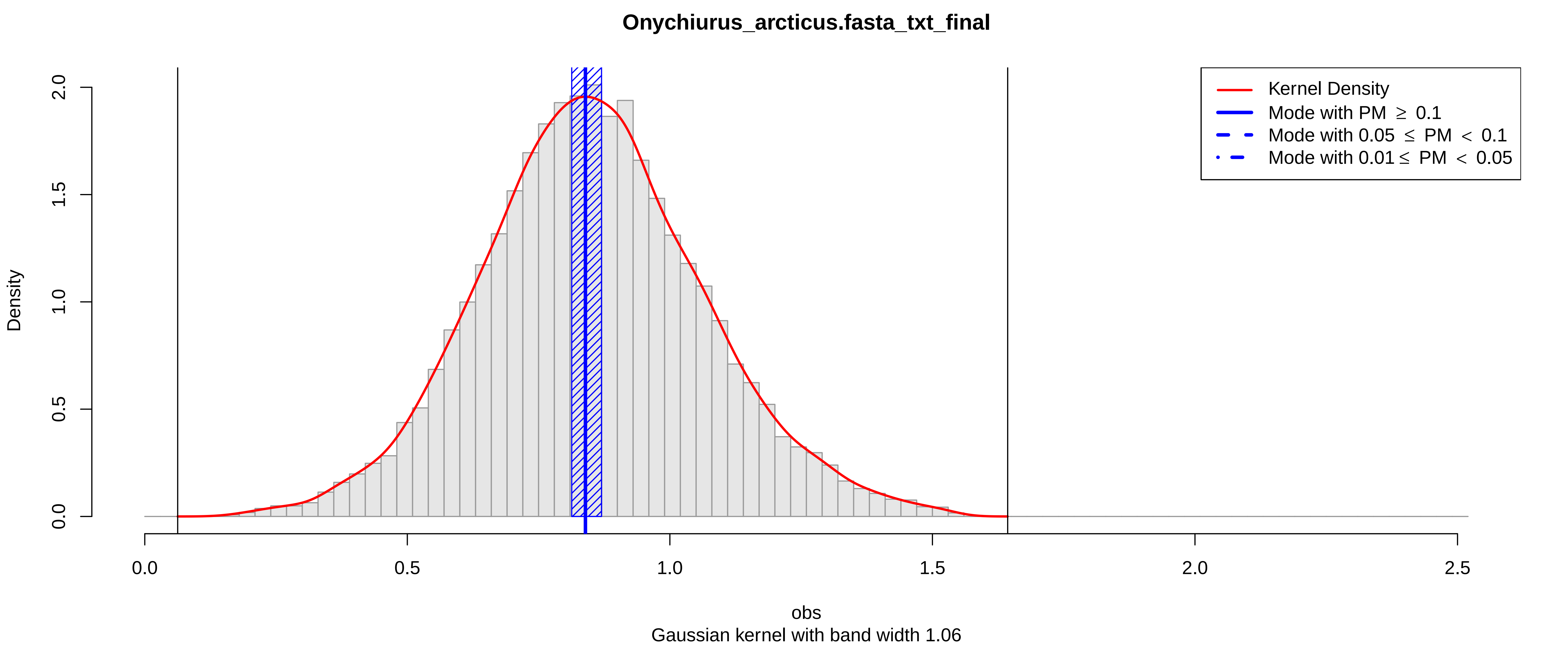
Onychiurus_arcticus.fasta_txt_final
Density
0.0
0.5
1.0
1.5
2.0
0.0
0.5
1.0
1.5
2.0
2.5
obs
Gaussian kernel with band width 1.06
Kernel Density
Mode with PM ≥ 0.1
Mode with 0.05 ≤ PM < 0.1
Mode with 0.01 ≤ PM < 0.05

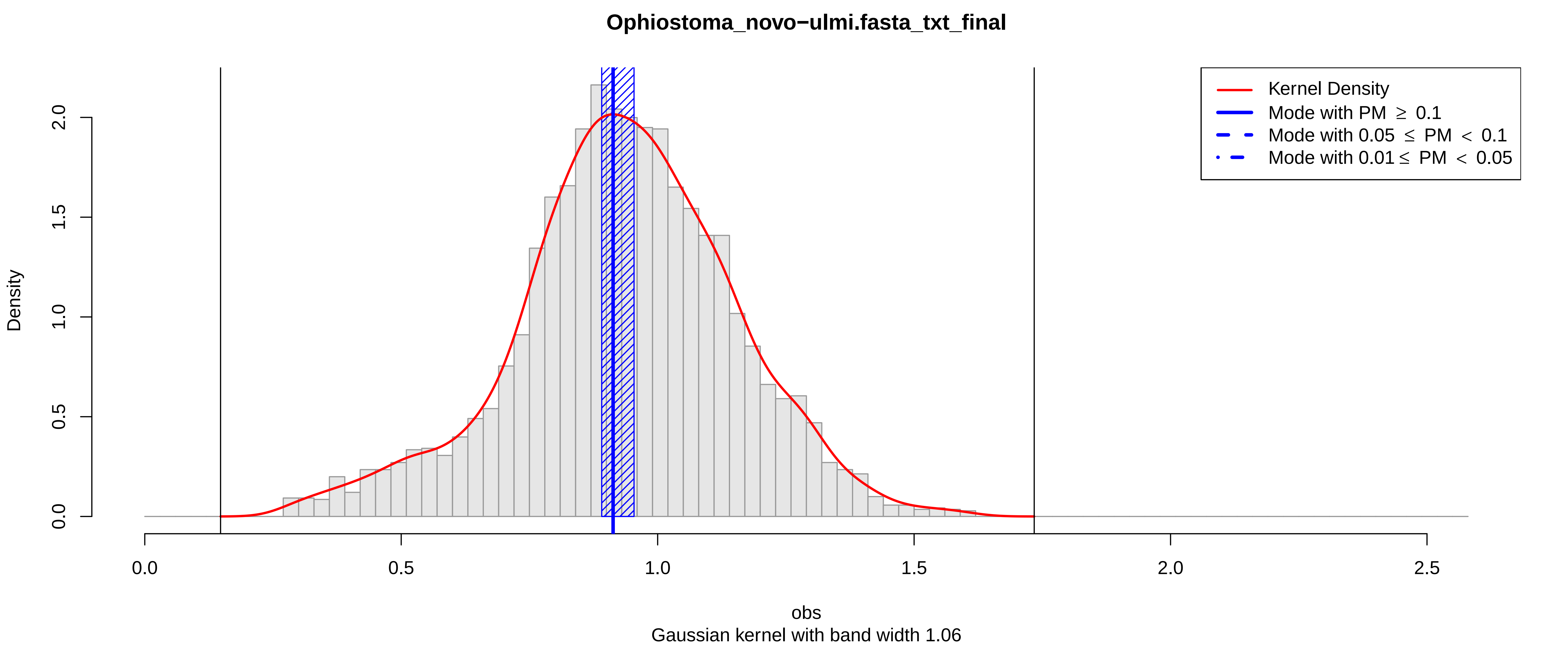
Ophiostoma_novo−ulmi.fasta_txt_final
Kernel Density
Mode with PM ≥ 0.1
Mode with 0.05 ≤ PM < 0.1
Mode with 0.01 ≤ PM < 0.05
Density
0.0
0.5
1.0
1.5
2.0
0.0
0.5
1.0
1.5
2.0
2.5
obs
Gaussian kernel with band width 1.06

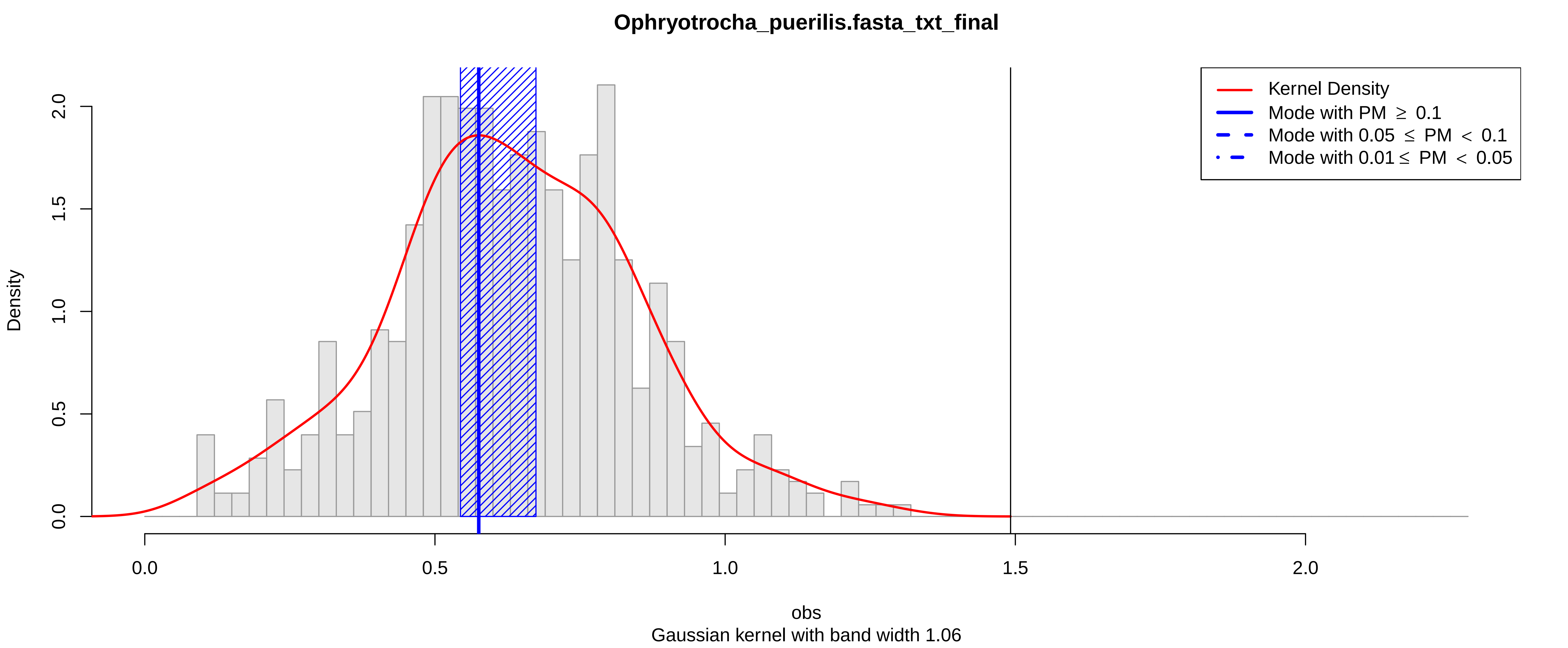
Ophryotrocha_puerilis.fasta_txt_final
Kernel Density
Mode with PM ≥ 0.1
Mode with 0.05 ≤ PM < 0.1
Mode with 0.01 ≤ PM < 0.05
Density
0.0
0.5
1.0
1.5
2.0
0.0
0.5
1.0
1.5
2.0
obs
Gaussian kernel with band width 1.06

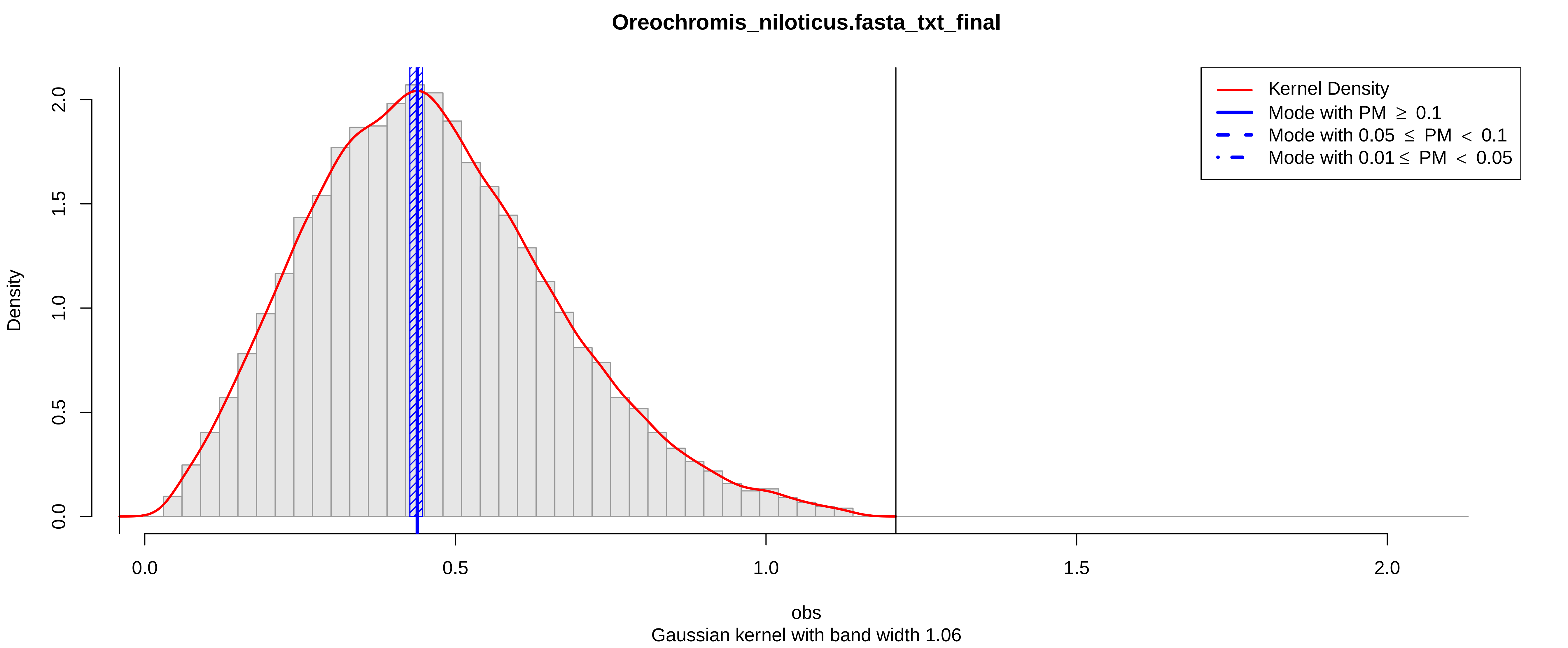
Oreochromis_niloticus.fasta_txt_final
Kernel Density
Mode with PM ≥ 0.1
Mode with 0.05 ≤ PM < 0.1
Mode with 0.01 ≤ PM < 0.05
Density
0.0
0.5
1.0
1.5
2.0
obs
Gaussian kernel with band width 1.06

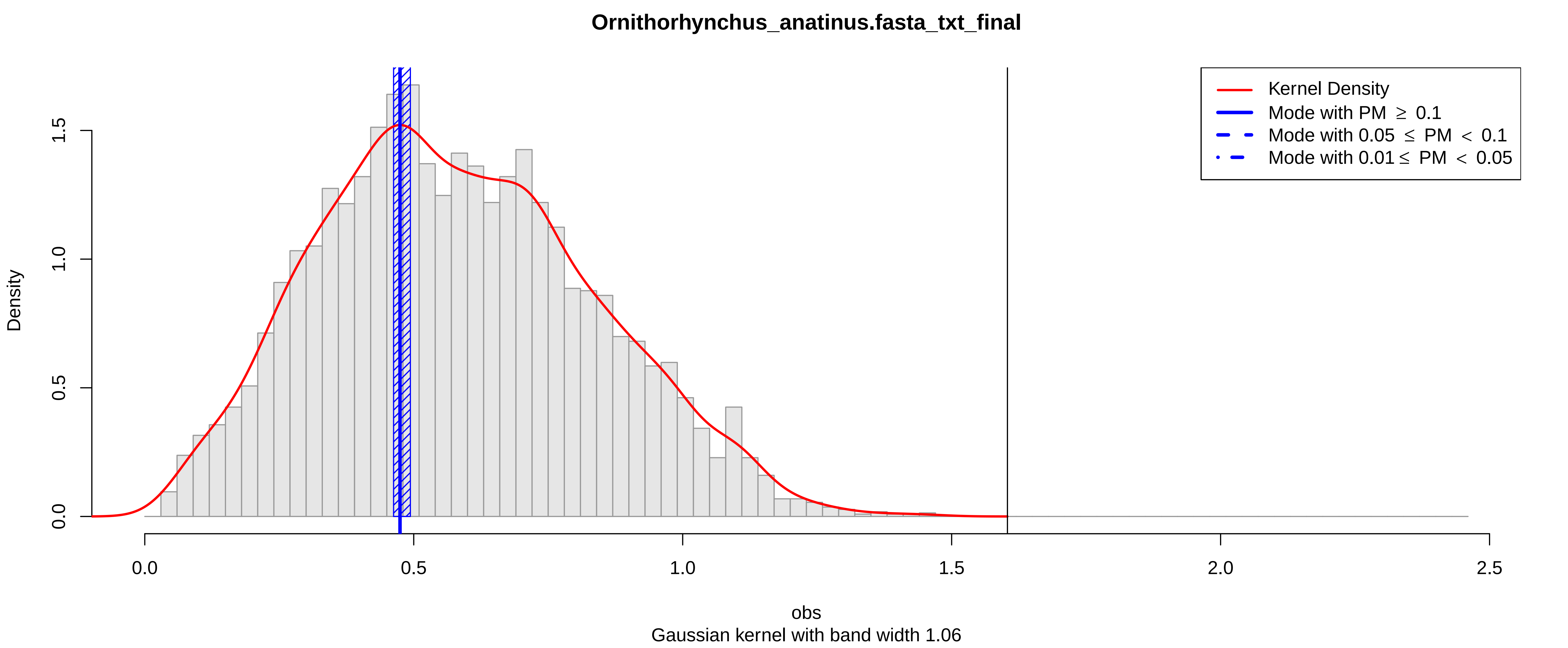

Ornithorhynchus_anatinus.fasta_txt_final
Kernel Density
Mode with PM ≥ 0.1
Mode with 0.05 ≤ PM < 0.1
Mode with 0.01 ≤ PM < 0.05
Density
0.0
0.5
1.0
1.5
0.0
0.5
1.0
1.5
2.0
2.5
obs
Gaussian kernel with band width 1.06

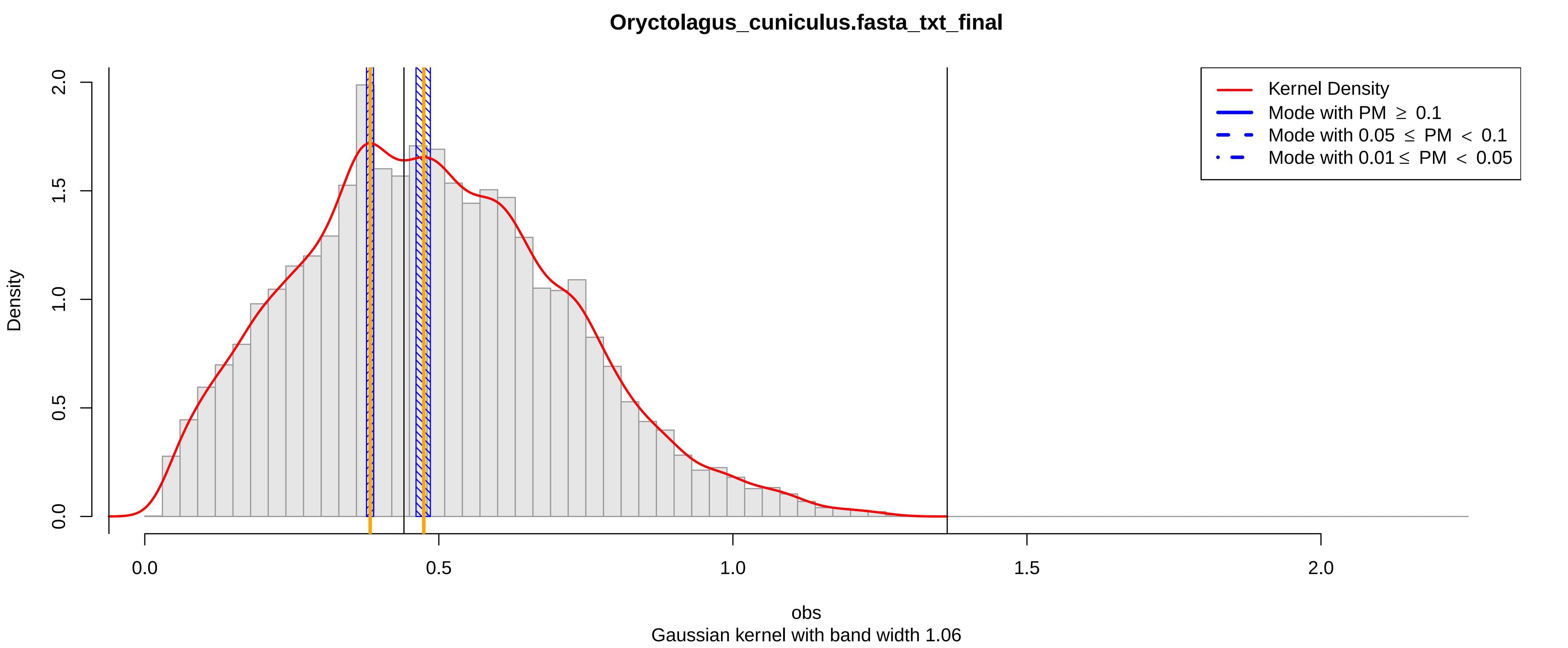

Oryctolagus_cuniculus.fasta_txt_final
Kernel Density
Mode with PM ≥ 0.1
Mode with 0.05 ≤ PM < 0.1
Mode with 0.01 ≤ PM < 0.05
Density
0.0
0.5
1.0
1.5
2.0
obs
Gaussian kernel with band width 1.06

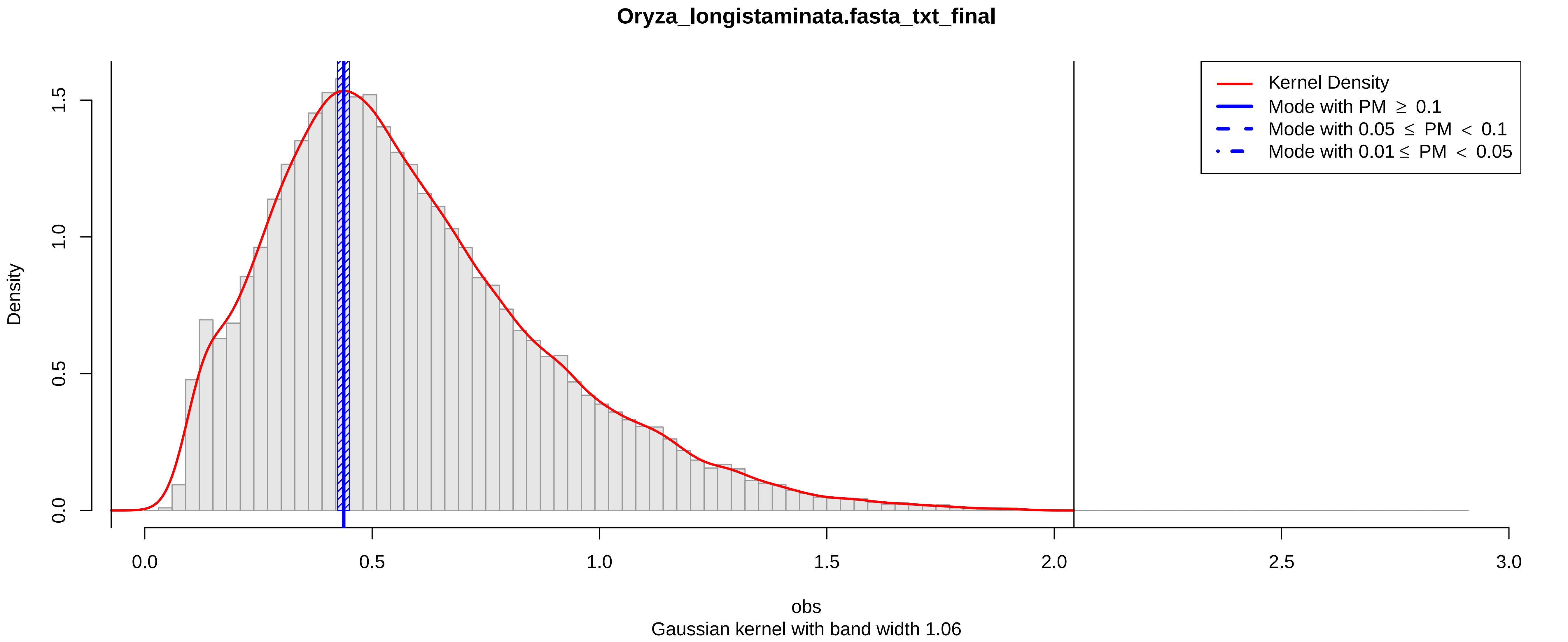
Oryza_longistaminata.fasta_txt_final
Kernel Density
Mode with PM ≥ 0.1
Mode with 0.05 ≤ PM < 0.1
Mode with 0.01 ≤ PM < 0.05
Density
0.0
0.5
1.0
1.5
0.0
0.5
1.0
1.5
2.0
2.5
3.0
obs
Gaussian kernel with band width 1.06

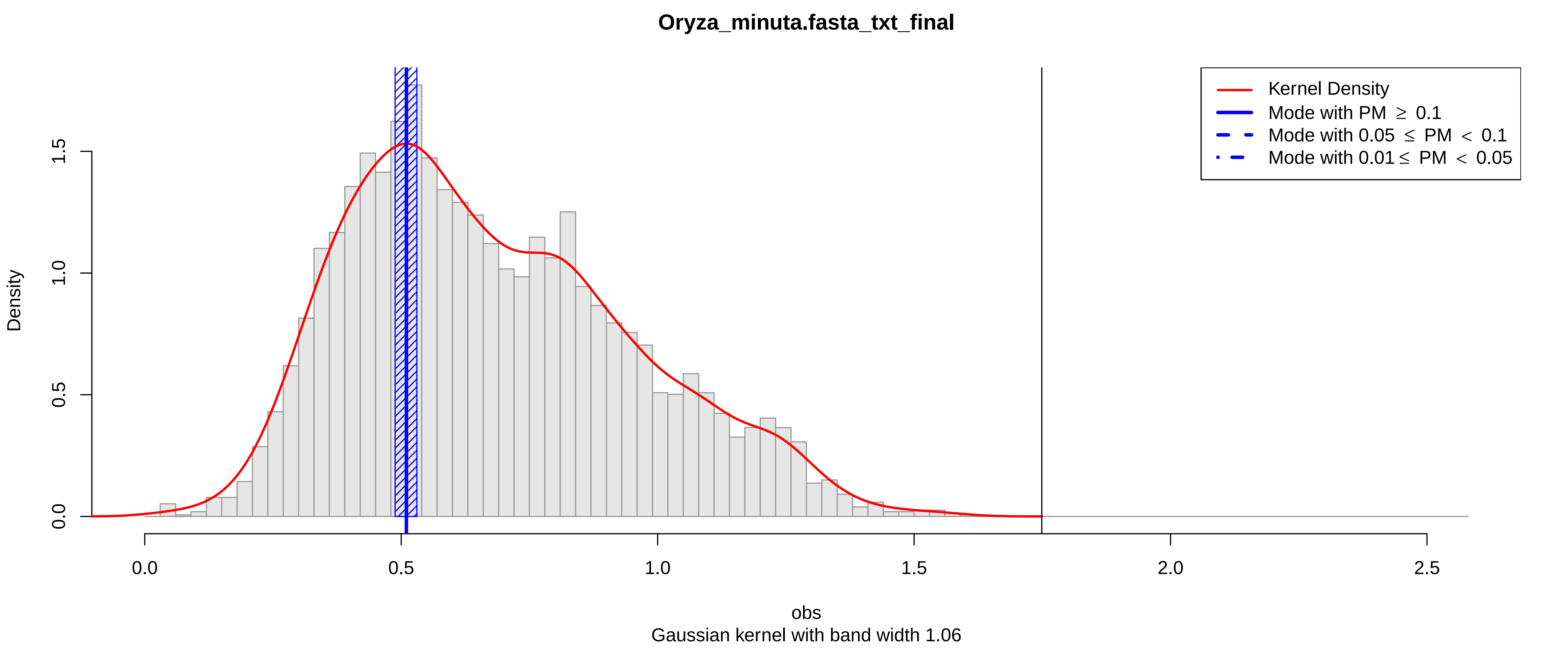

Oryza_minuta.fasta_txt_final
Kernel Density
Mode with PM ≥ 0.1
Mode with 0.05 ≤ PM < 0.1
Mode with 0.01 ≤ PM < 0.05
Density
0.0
0.5
1.0
1.5
0.0
0.5
1.0
1.5
2.0
2.5
obs
Gaussian kernel with band width 1.06

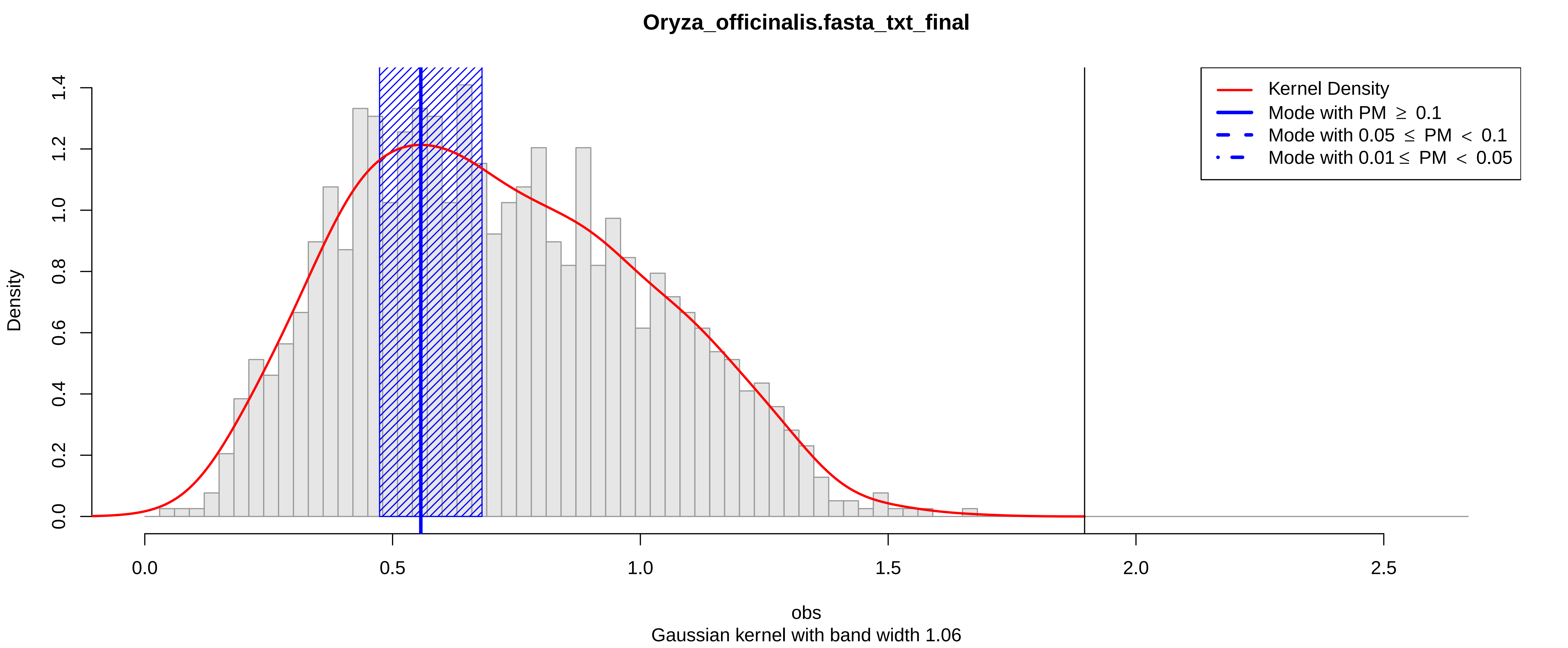
Oryza_officinalis.fasta_txt_final
Kernel Density
Mode with PM ≥ 0.1
Mode with 0.05 ≤ PM < 0.1
Mode with 0.01 ≤ PM < 0.05
Density
0.0
0.2
0.4
0.6
0.8
1.0
1.2
1.4
0.0
0.5
1.0
1.5
2.0
2.5
obs
Gaussian kernel with band width 1.06

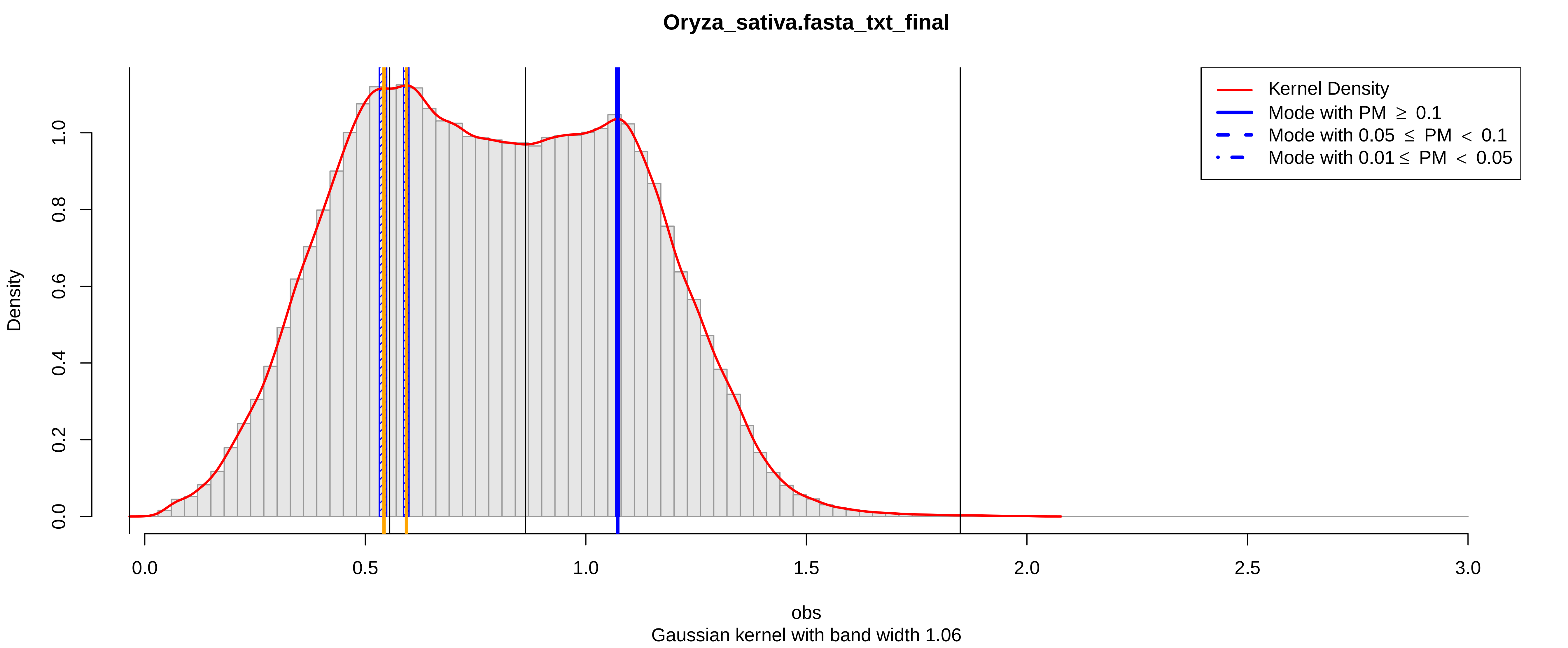

Oryza_sativa.fasta_txt_final
Kernel Density
Mode with PM ≥ 0.1
Mode with 0.05 ≤ PM < 0.1
Mode with 0.01 ≤ PM < 0.05
Density
0.0
0.2
0.4
0.6
0.8
1.0
0.0
0.5
1.0
1.5
2.0
2.5
3.0
obs
Gaussian kernel with band width 1.06

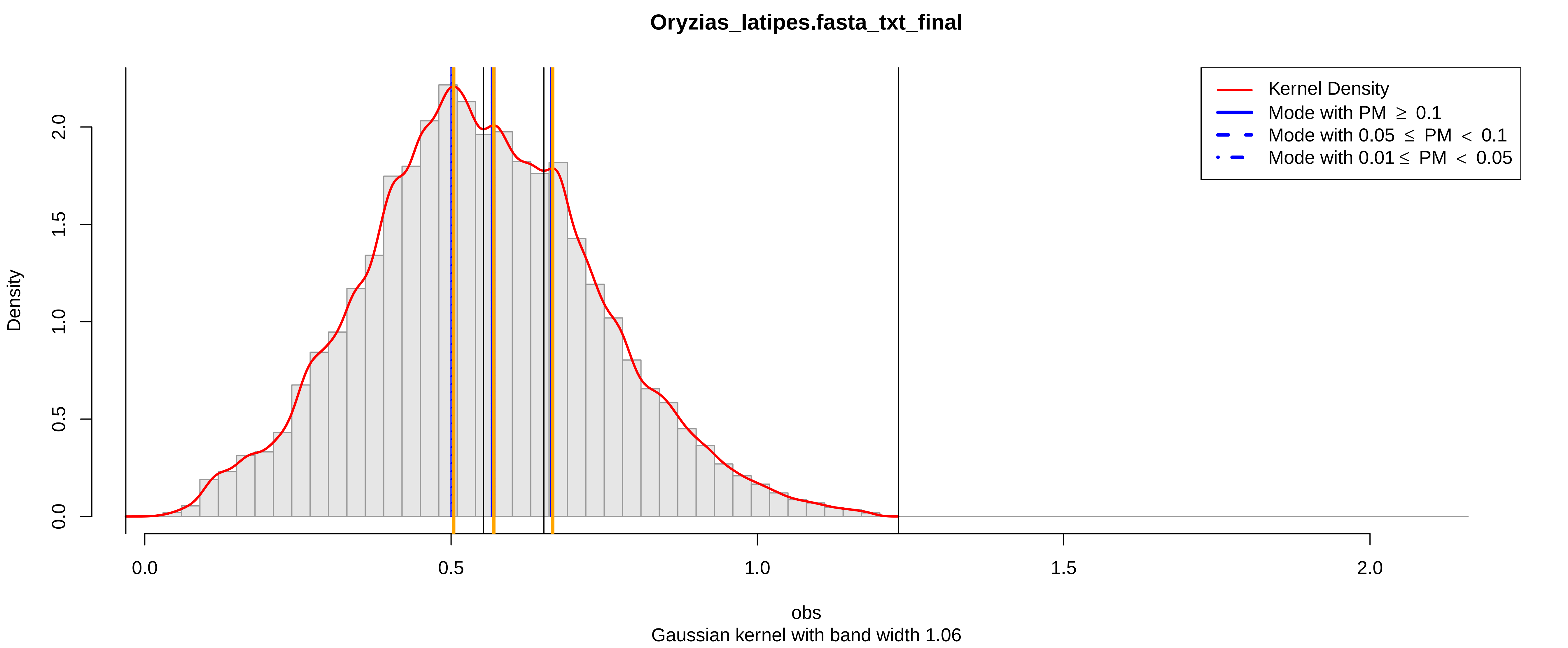

Oryzias_latipes.fasta_txt_final
Kernel Density
Mode with PM ≥ 0.1
Mode with 0.05 ≤ PM < 0.1
Mode with 0.01 ≤ PM < 0.05
Density
0.0
0.5
1.0
1.5
2.0
0.0
0.5
1.0
1.5
2.0
obs
Gaussian kernel with band width 1.06

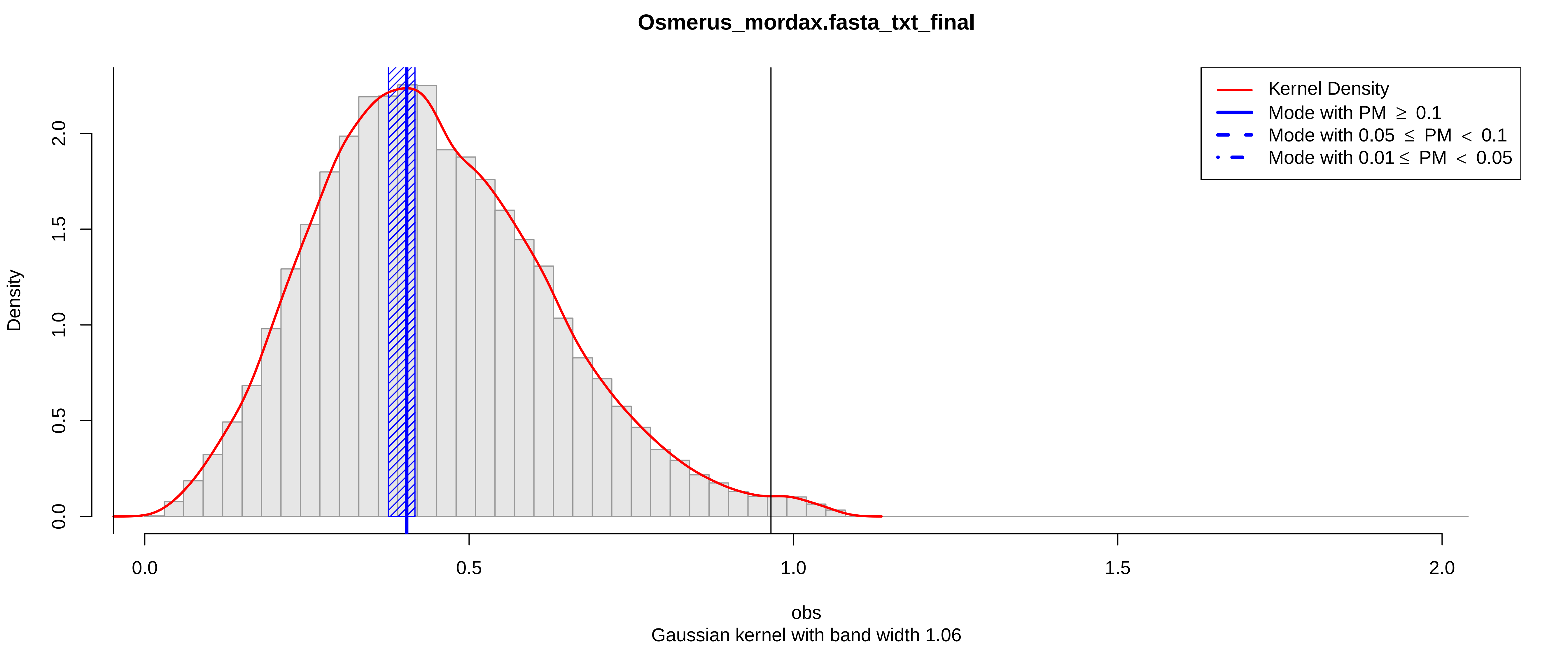

Osmerus_mordax.fasta_txt_final
Kernel Density
Mode with PM ≥ 0.1
Mode with 0.05 ≤ PM < 0.1
Mode with 0.01 ≤ PM < 0.05
Density
0.0
0.5
1.0
1.5
2.0
0.0
0.5
1.0
1.5
2.0
obs
Gaussian kernel with band width 1.06

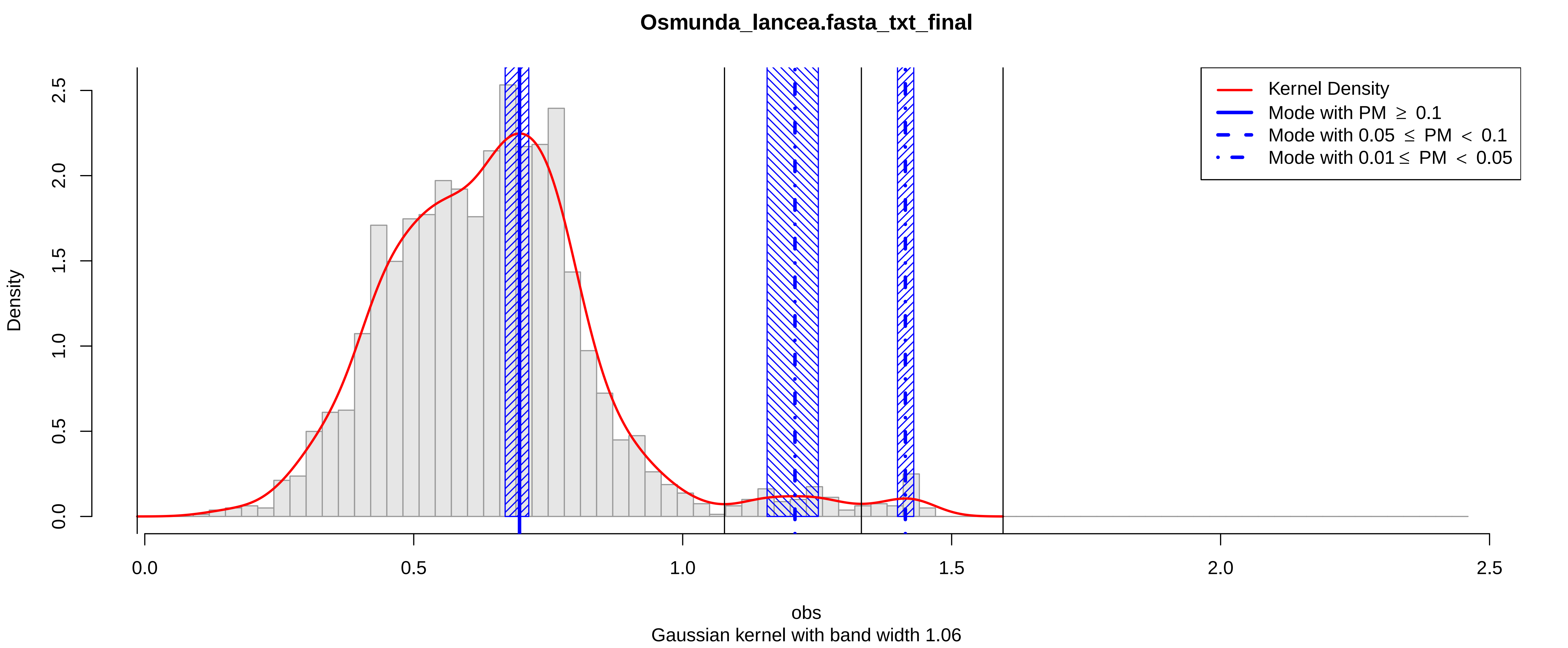
Osmunda_lancea.fasta_txt_final
Kernel Density
Mode with PM ≥ 0.1
Mode with 0.05 ≤ PM < 0.1
Mode with 0.01 ≤ PM < 0.05
Density
0.0
0.5
1.0
1.5
2.0
2.5
obs
Gaussian kernel with band width 1.06

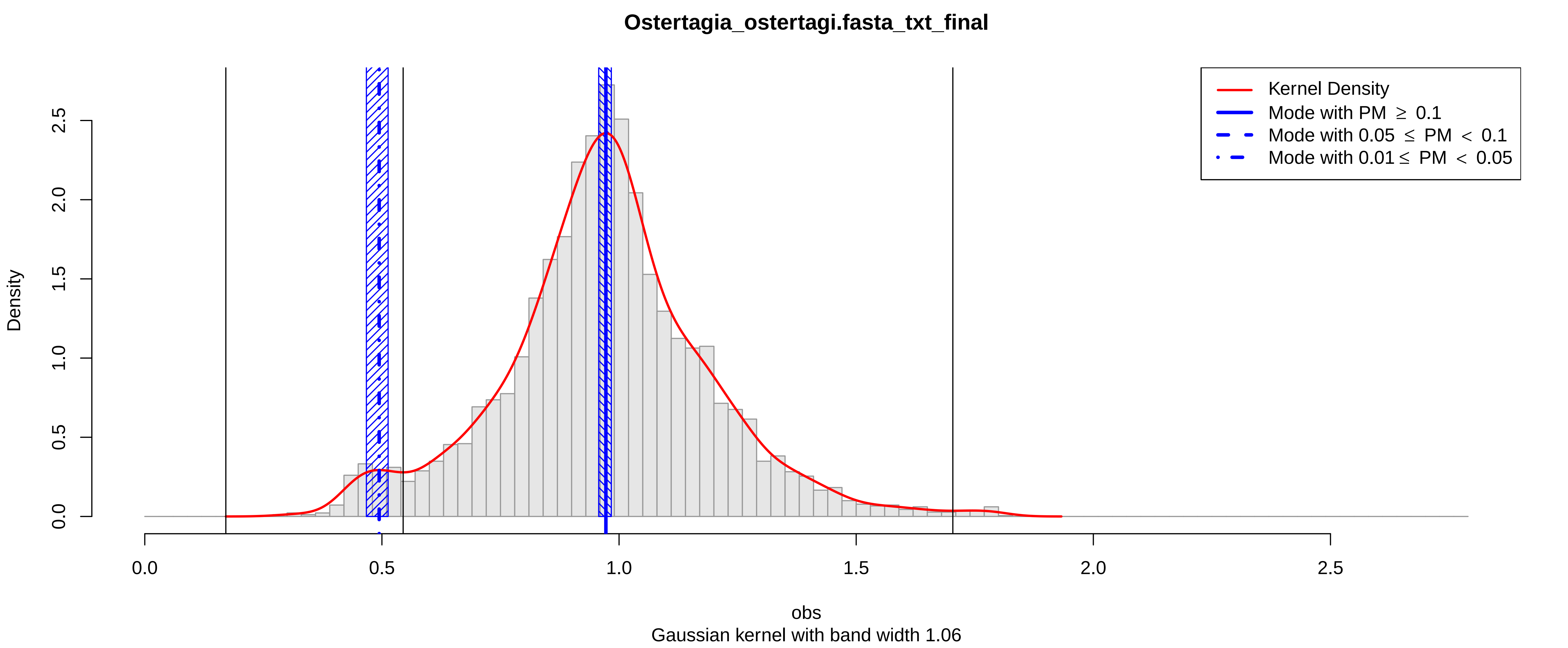

Ostertagia_ostertagi.fasta_txt_final
Kernel Density
Mode with PM ≥ 0.1
Mode with 0.05 ≤ PM < 0.1
Mode with 0.01 ≤ PM < 0.05
Density
0.0
0.5
1.0
1.5
2.0
2.5
obs
Gaussian kernel with band width 1.06

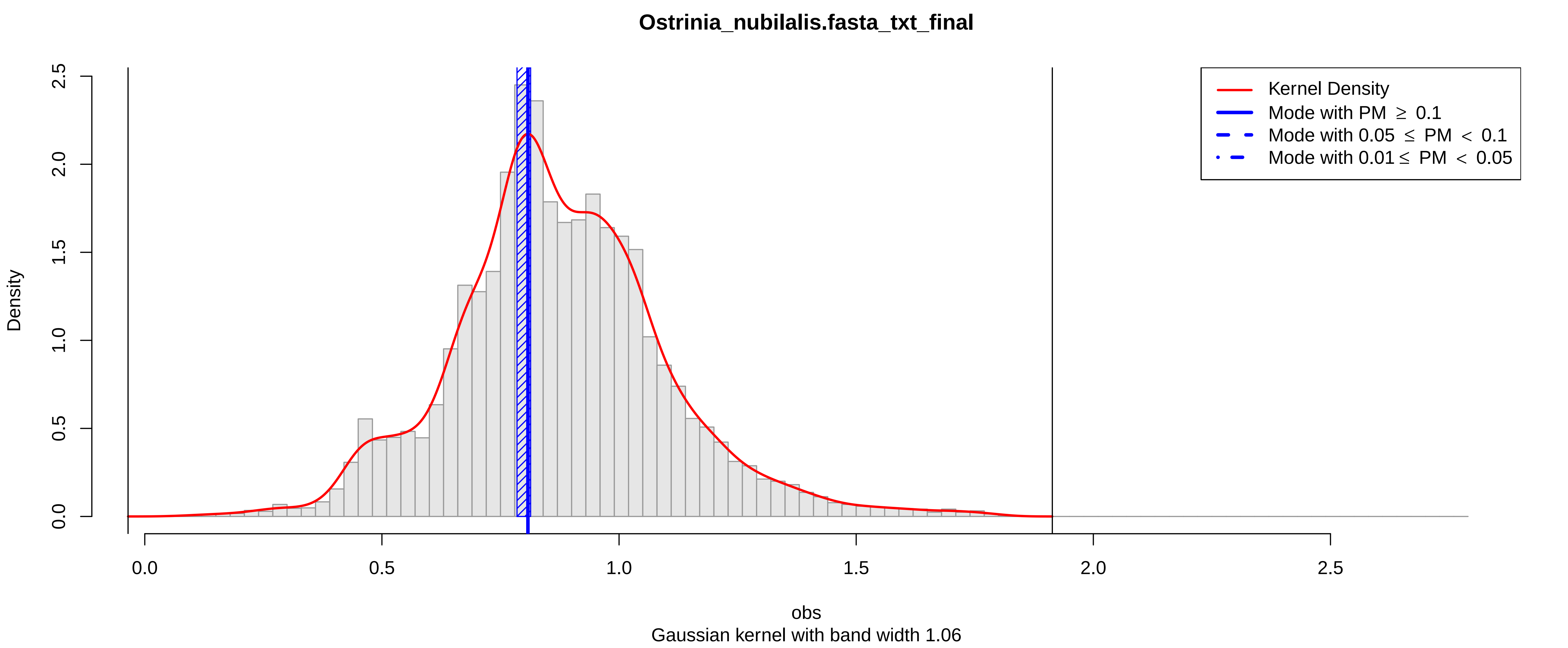

Ostrinia_nubilalis.fasta_txt_final
Kernel Density
Mode with PM ≥ 0.1
Mode with 0.05 ≤ PM < 0.1
Mode with 0.01 ≤ PM < 0.05
Density
0.0
0.5
1.0
1.5
2.0
2.5
obs
Gaussian kernel with band width 1.06

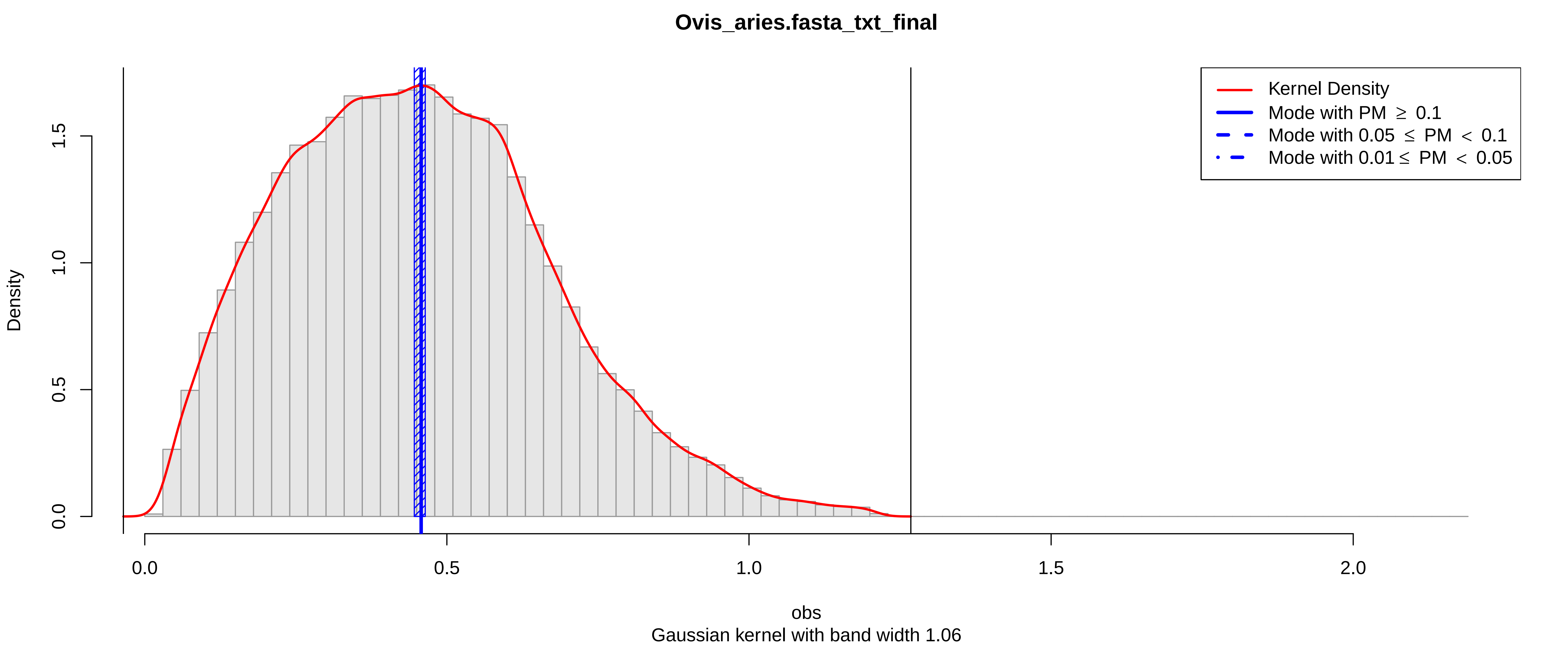
Ovis_aries.fasta_txt_final
Kernel Density
Mode with PM ≥ 0.1
Mode with 0.05 ≤ PM < 0.1
Mode with 0.01 ≤ PM < 0.05
Density
0.0
0.5
1.0
1.5
0.0
0.5
1.0
1.5
2.0
obs
Gaussian kernel with band width 1.06

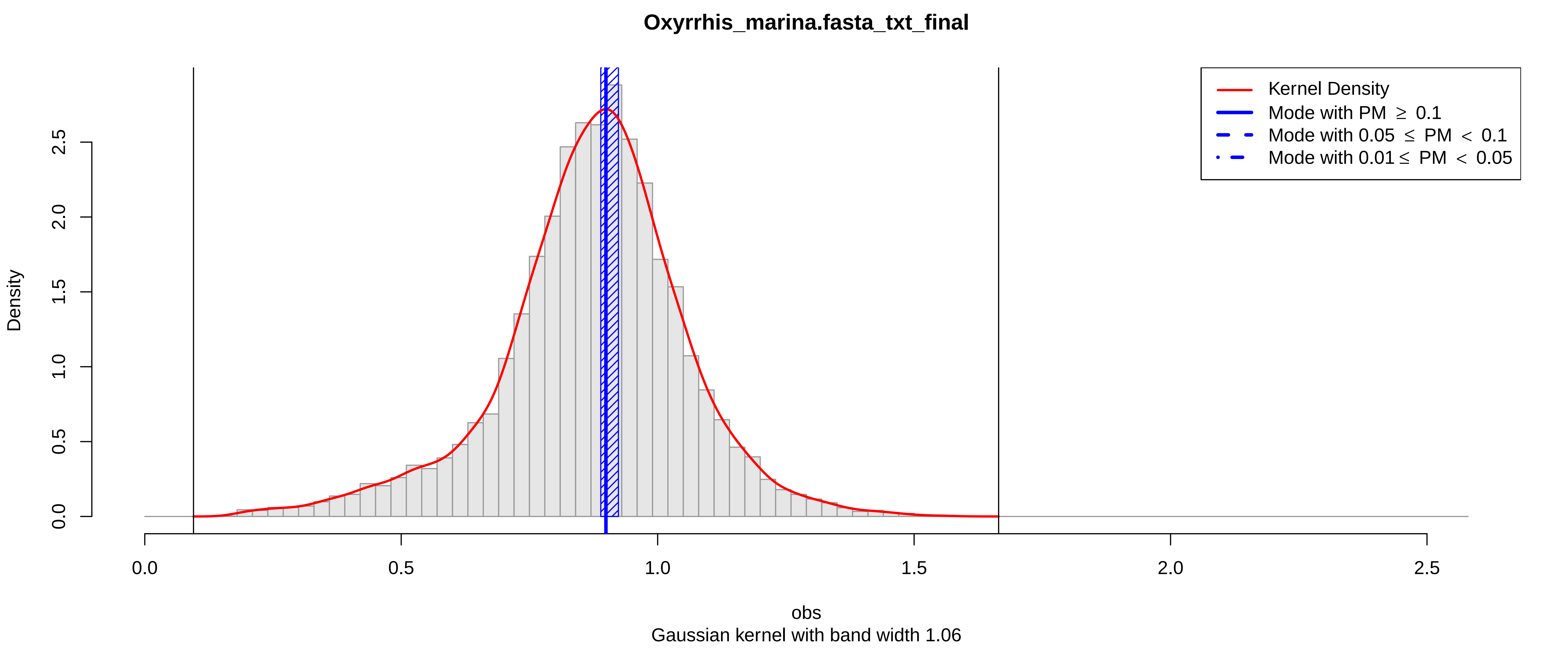
Oxyrrhis_marina.fasta_txt_final
Kernel Density
Mode with PM ≥ 0.1
Mode with 0.05 ≤ PM < 0.1
Mode with 0.01 ≤ PM < 0.05
Density
0.0
0.5
1.0
1.5
2.0
2.5
obs
Gaussian kernel with band width 1.06

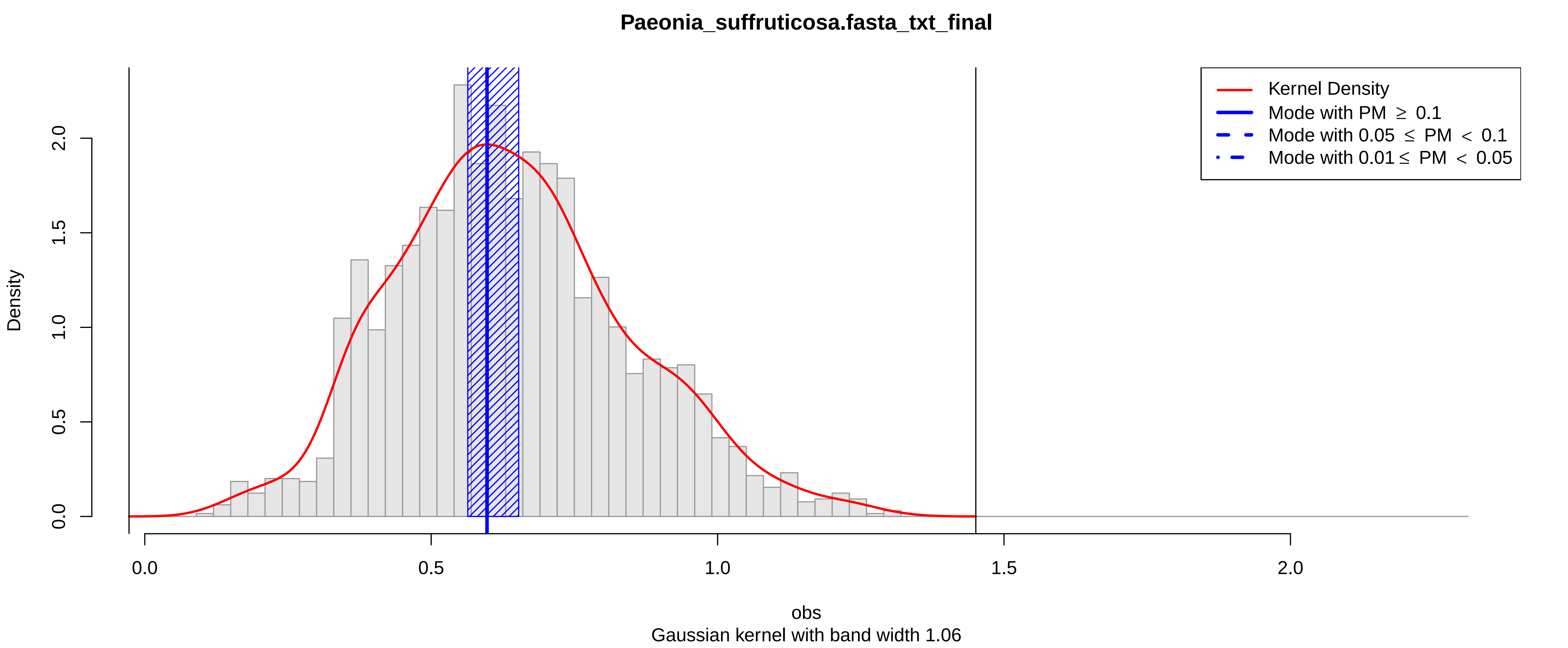

Paeonia_suffruticosa.fasta_txt_final
Kernel Density
Mode with PM ≥ 0.1
Mode with 0.05 ≤ PM < 0.1
Mode with 0.01 ≤ PM < 0.05
Density
0.0
0.5
1.0
1.5
2.0
0.0
0.5
1.0
1.5
2.0
obs
Gaussian kernel with band width 1.06

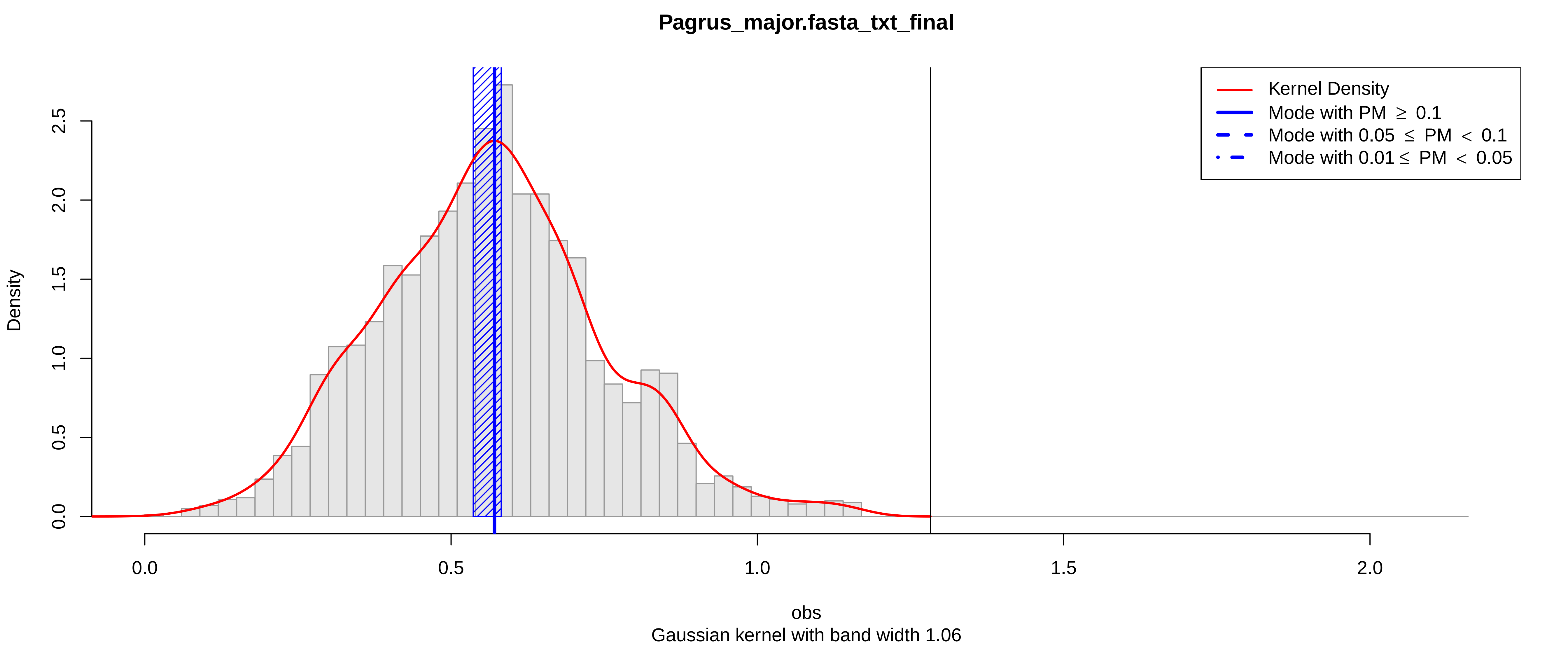

Pagrus_major.fasta_txt_final
Kernel Density
Mode with PM ≥ 0.1
Mode with 0.05 ≤ PM < 0.1
Mode with 0.01 ≤ PM < 0.05
Density
0.0
0.5
1.0
1.5
2.0
2.5
0.0
0.5
1.0
1.5
2.0
obs
Gaussian kernel with band width 1.06

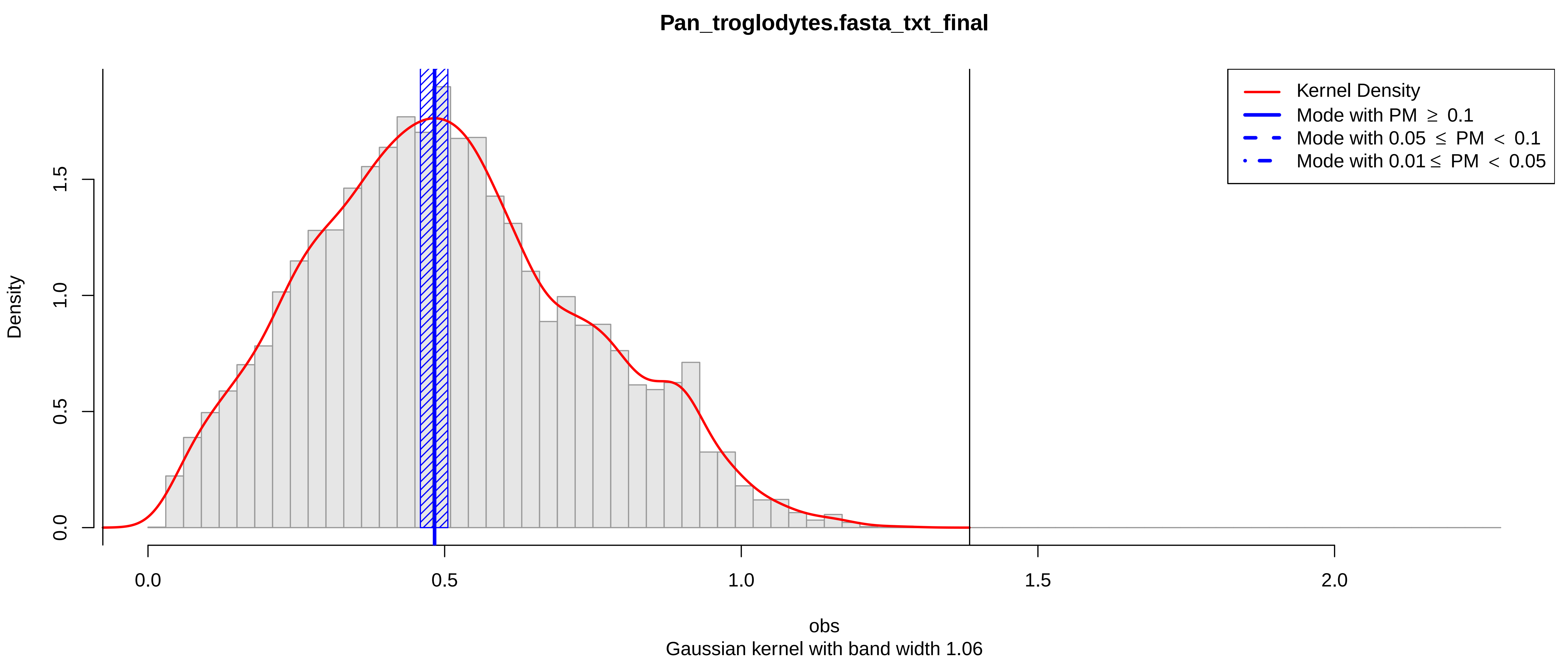

Pan_troglodytes.fasta_txt_final
Kernel Density
Mode with PM ≥ 0.1
Mode with 0.05 ≤ PM < 0.1
Mode with 0.01 ≤ PM < 0.05
Density
0.0
0.5
1.0
1.5
0.0
0.5
1.0
1.5
2.0
obs
Gaussian kernel with band width 1.06

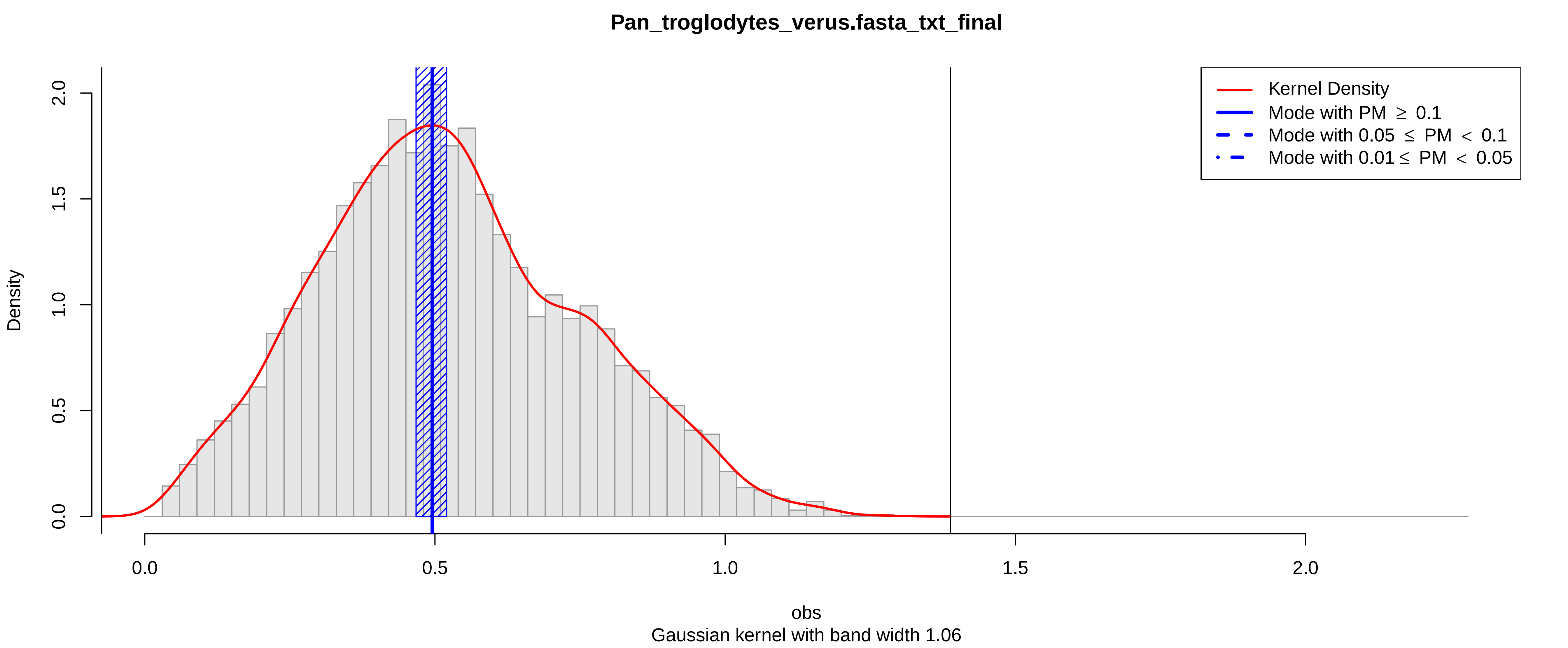

Pan_troglodytes_verus.fasta_txt_final
Kernel Density
Mode with PM ≥ 0.1
Mode with 0.05 ≤ PM < 0.1
Mode with 0.01 ≤ PM < 0.05
Density
0.0
0.5
1.0
1.5
2.0
obs
Gaussian kernel with band width 1.06

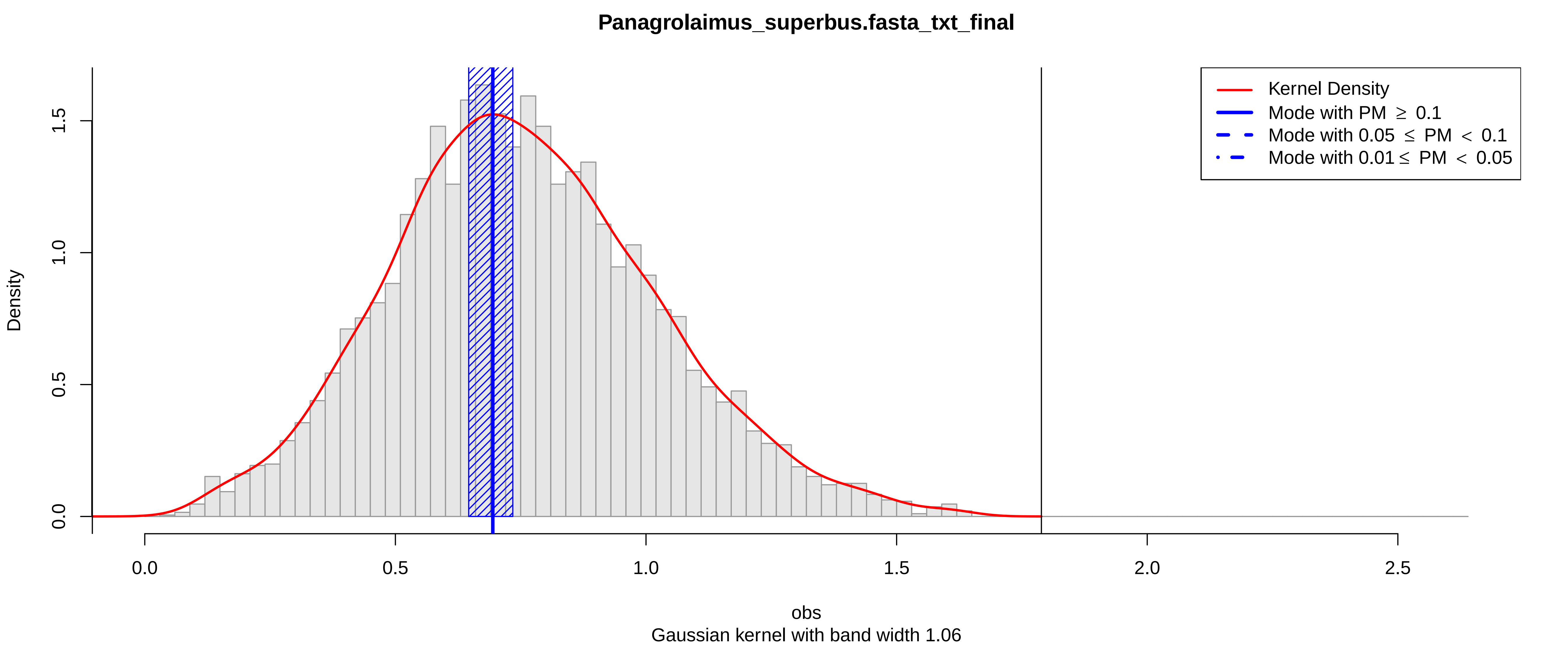
Panagrolaimus_superbus.fasta_txt_final
Kernel Density
Mode with PM ≥ 0.1
Mode with 0.05 ≤ PM < 0.1
Mode with 0.01 ≤ PM < 0.05
Density
0.0
0.5
1.0
1.5
2.0
2.5
obs
Gaussian kernel with band width 1.06

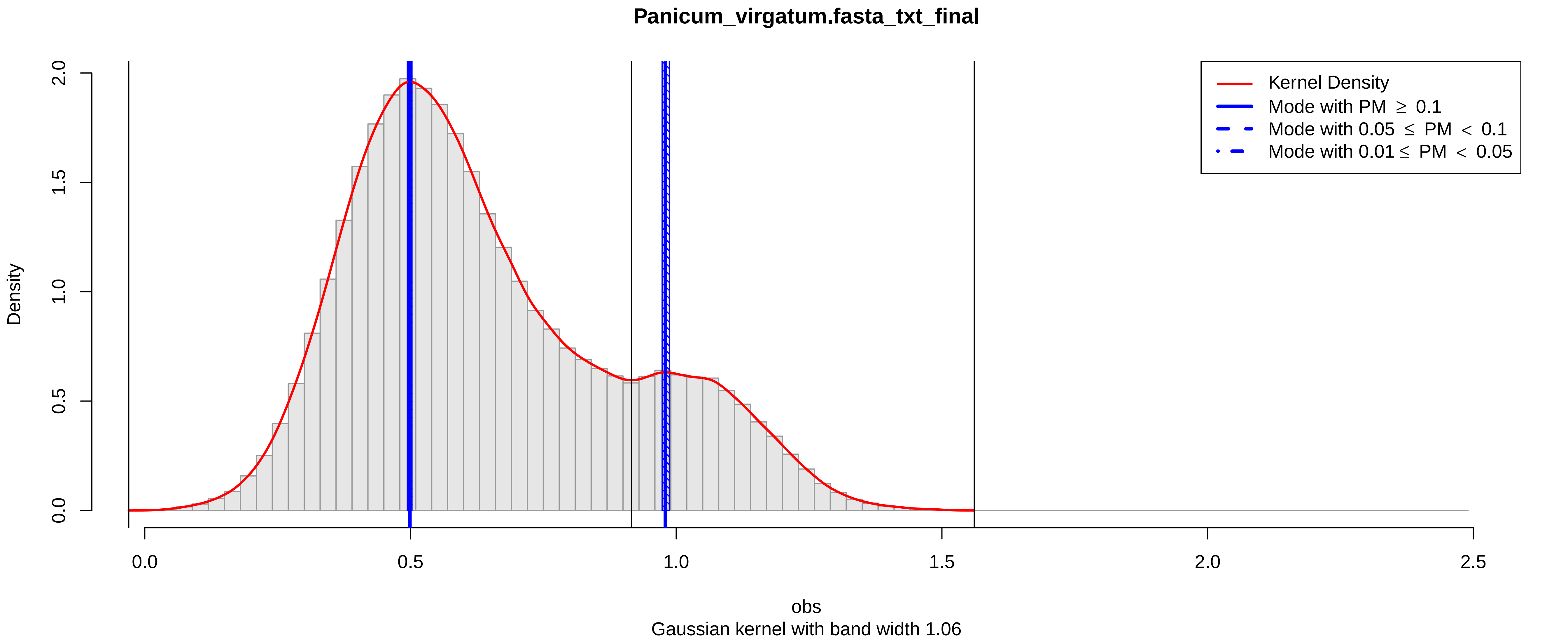

Panicum_virgatum.fasta_txt_final
Kernel Density
Mode with PM ≥ 0.1
Mode with 0.05 ≤ PM < 0.1
Mode with 0.01 ≤ PM < 0.05
Density
0.0
0.5
1.0
1.5
2.0
2.5
obs
Gaussian kernel with band width 1.06

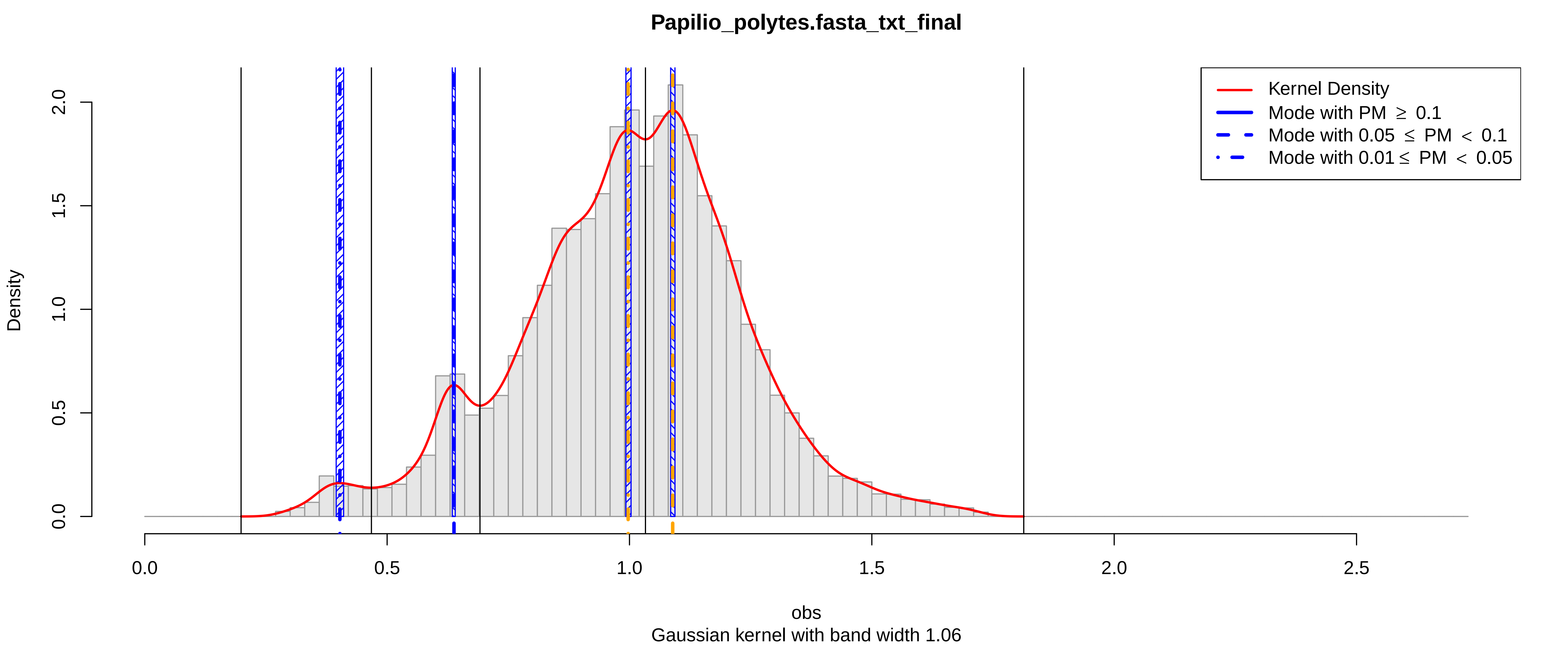

Papilio_polytes.fasta_txt_final
Kernel Density
Mode with PM ≥ 0.1
Mode with 0.05 ≤ PM < 0.1
Mode with 0.01 ≤ PM < 0.05
Density
0.0
0.5
1.0
1.5
2.0
2.5
obs
Gaussian kernel with band width 1.06

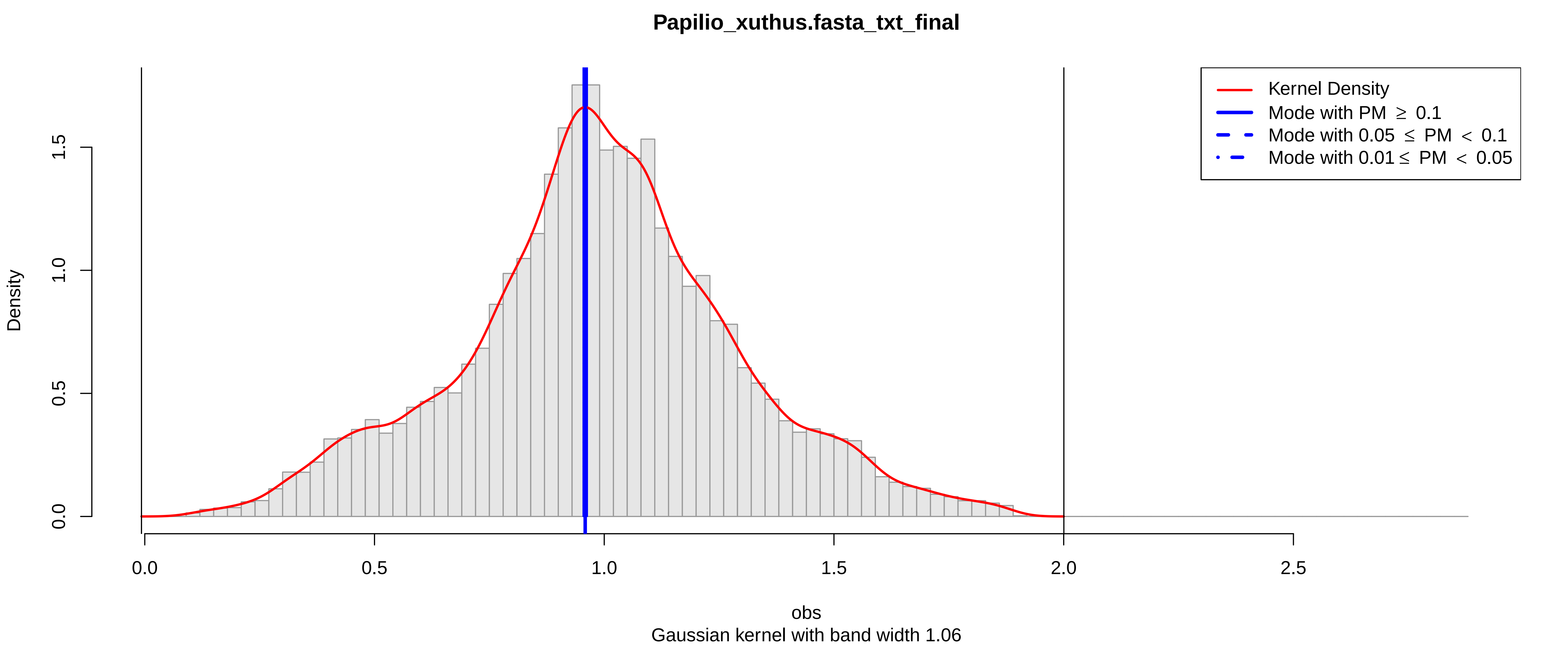

Papilio_xuthus.fasta_txt_final
Kernel Density
Mode with PM ≥ 0.1
Mode with 0.05 ≤ PM < 0.1
Mode with 0.01 ≤ PM < 0.05
Density
0.0
0.5
1.0
1.5
0.0
0.5
1.0
1.5
2.0
2.5
obs
Gaussian kernel with band width 1.06

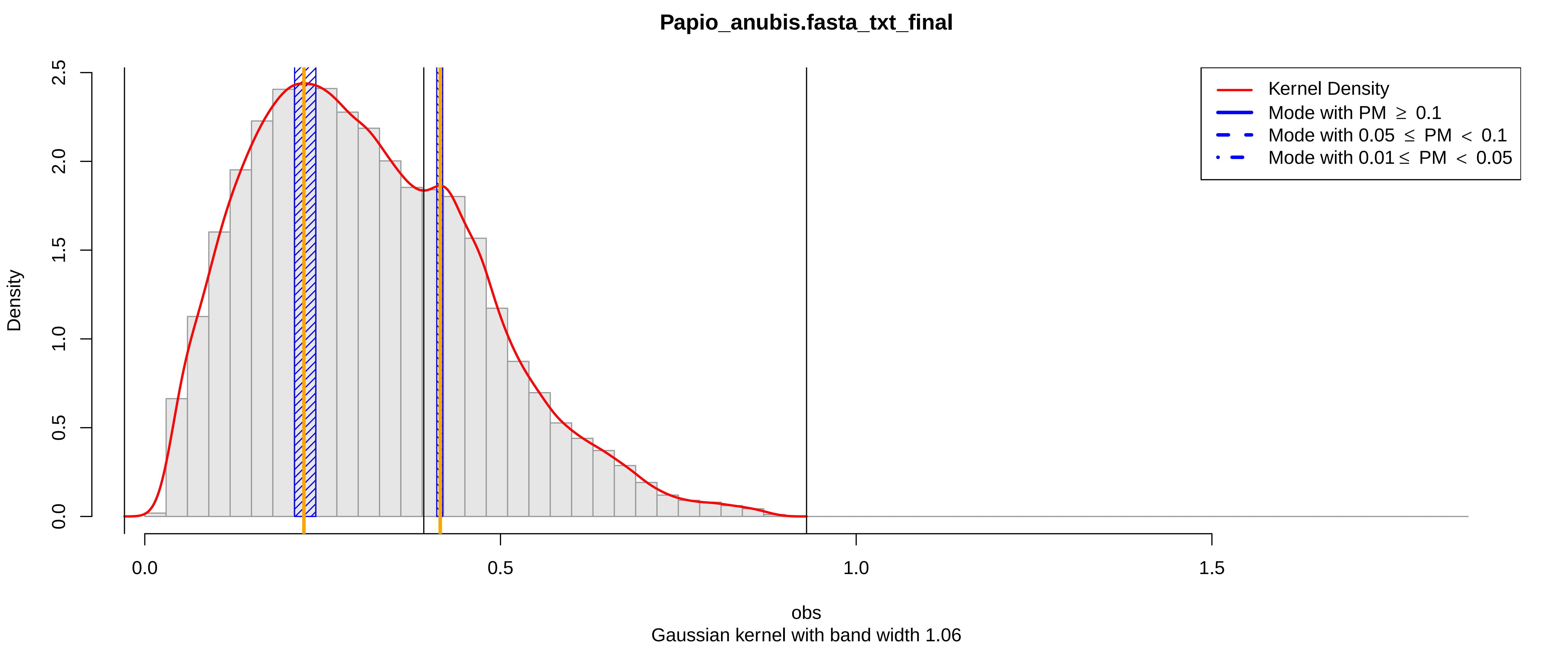

Papio_anubis.fasta_txt_final
Kernel Density
Mode with PM ≥ 0.1
Mode with 0.05 ≤ PM < 0.1
Mode with 0.01 ≤ PM < 0.05
Density
0.0
0.5
1.0
1.5
2.0
2.5
0.0
0.5
1.0
1.5
obs
Gaussian kernel with band width 1.06

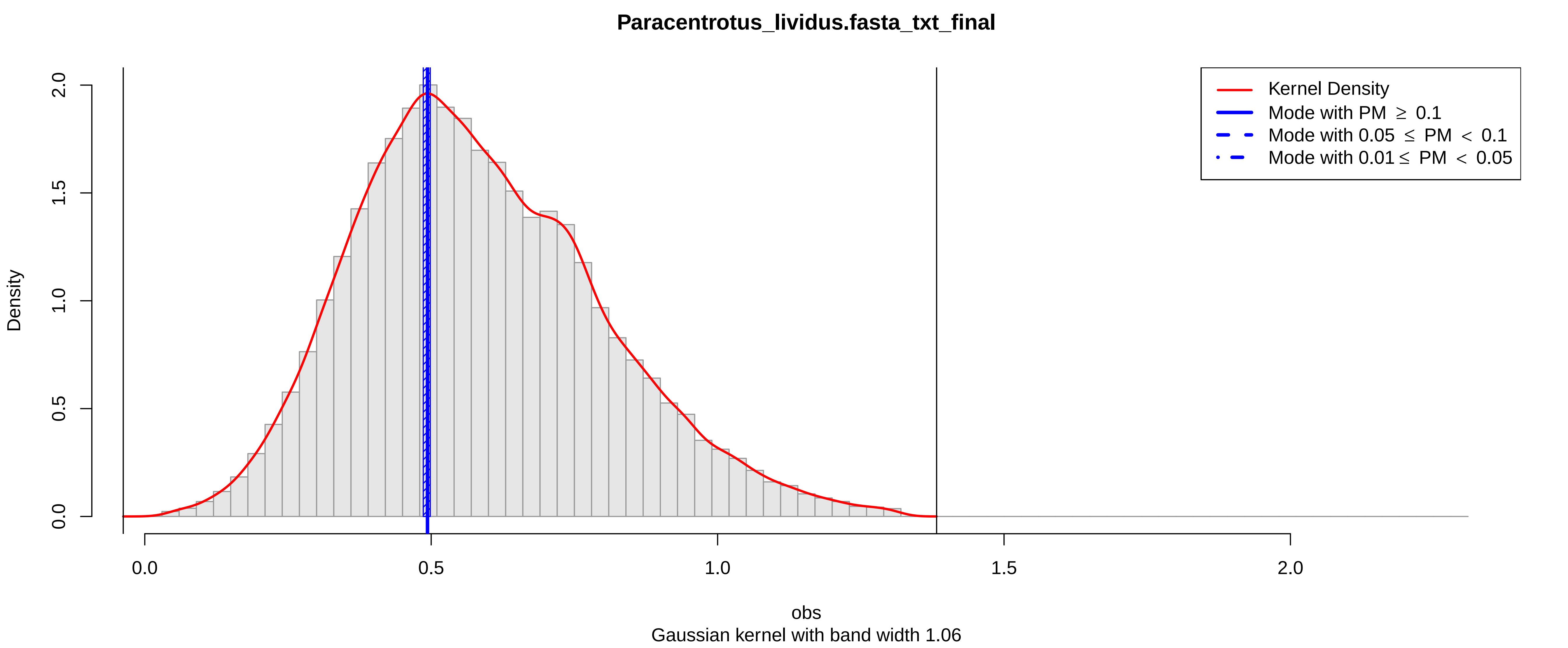

Paracentrotus_lividus.fasta_txt_final
Kernel Density
Mode with PM ≥ 0.1
Mode with 0.05 ≤ PM < 0.1
Mode with 0.01 ≤ PM < 0.05
Density
0.0
0.5
1.0
1.5
2.0
0.0
0.5
1.0
1.5
2.0
obs
Gaussian kernel with band width 1.06

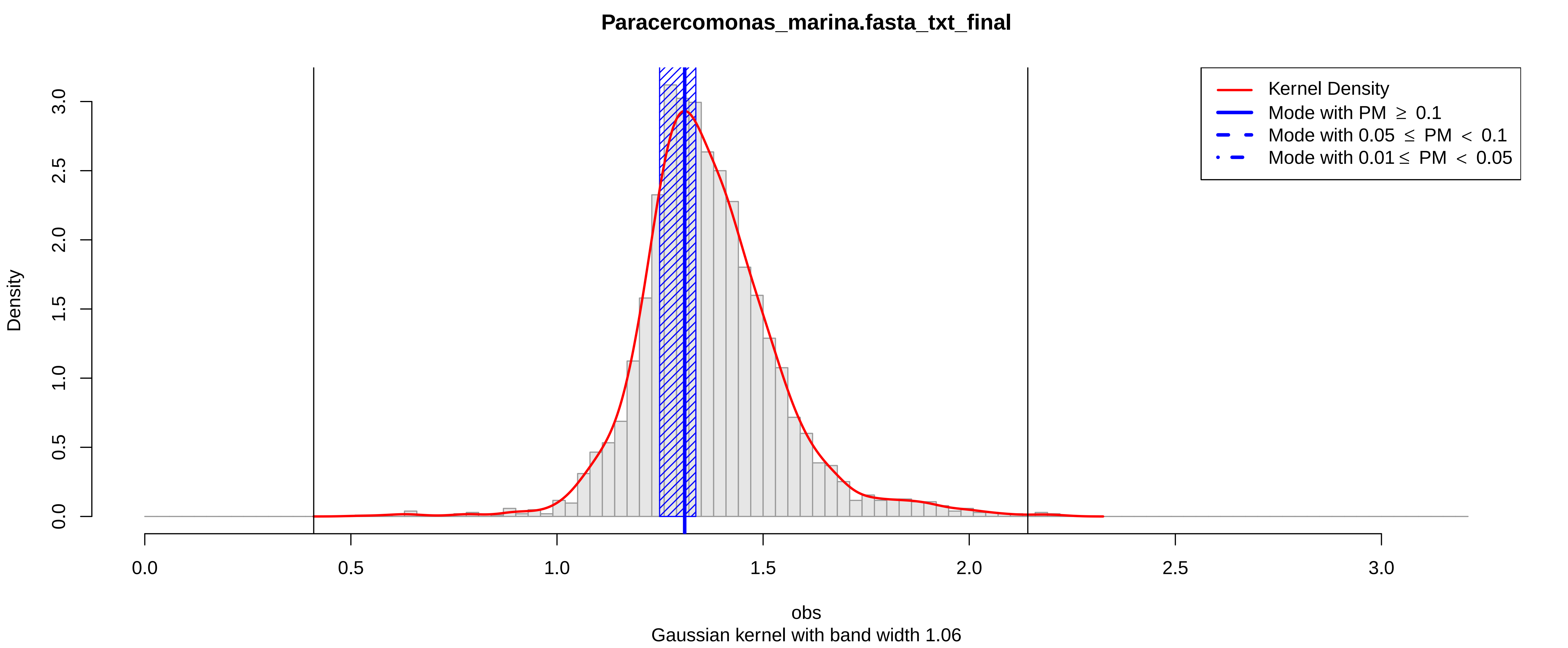

Paracercomonas_marina.fasta_txt_final
Kernel Density
Mode with PM ≥ 0.1
Mode with 0.05 ≤ PM < 0.1
Mode with 0.01 ≤ PM < 0.05
Density
0.0
0.5
1.0
1.5
2.0
2.5
3.0
0.0
0.5
1.0
1.5
2.0
2.5
3.0
obs
Gaussian kernel with band width 1.06

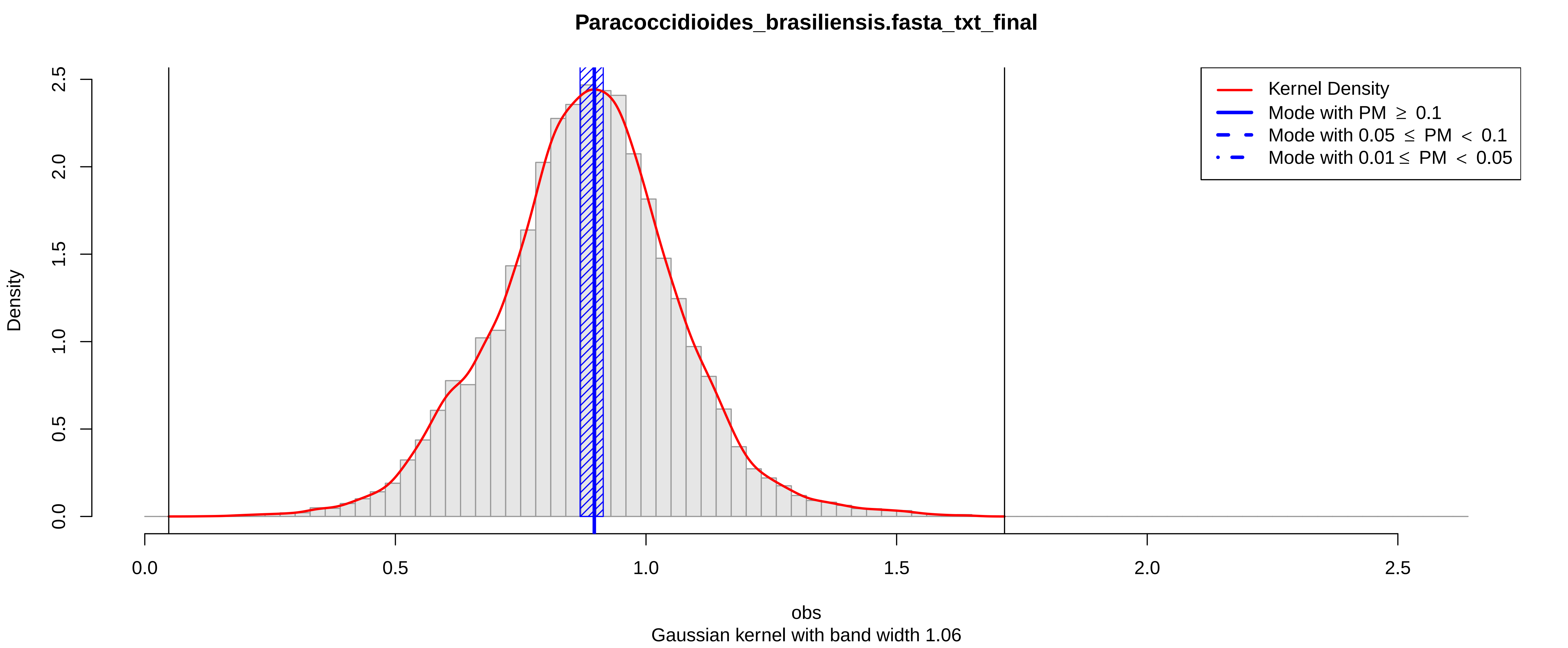
Paracoccidioides_brasiliensis.fasta_txt_final
Kernel Density
Mode with PM ≥ 0.1
Mode with 0.05 ≤ PM < 0.1
Mode with 0.01 ≤ PM < 0.05
Density
0.0
0.5
1.0
1.5
2.0
2.5
obs
Gaussian kernel with band width 1.06

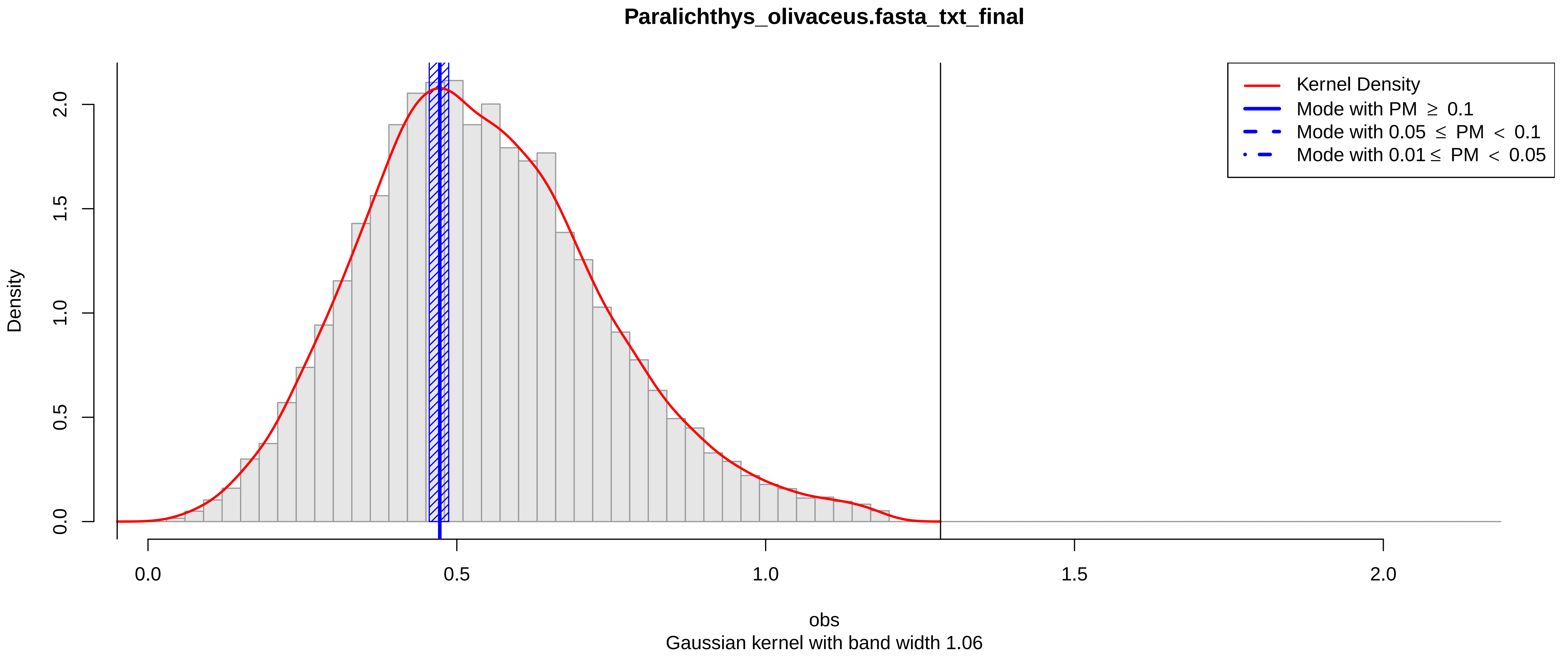

Paralichthys_olivaceus.fasta_txt_final
Kernel Density
Mode with PM ≥ 0.1
Mode with 0.05 ≤ PM < 0.1
Mode with 0.01 ≤ PM < 0.05
Density
0.0
0.5
1.0
1.5
2.0
obs
Gaussian kernel with band width 1.06

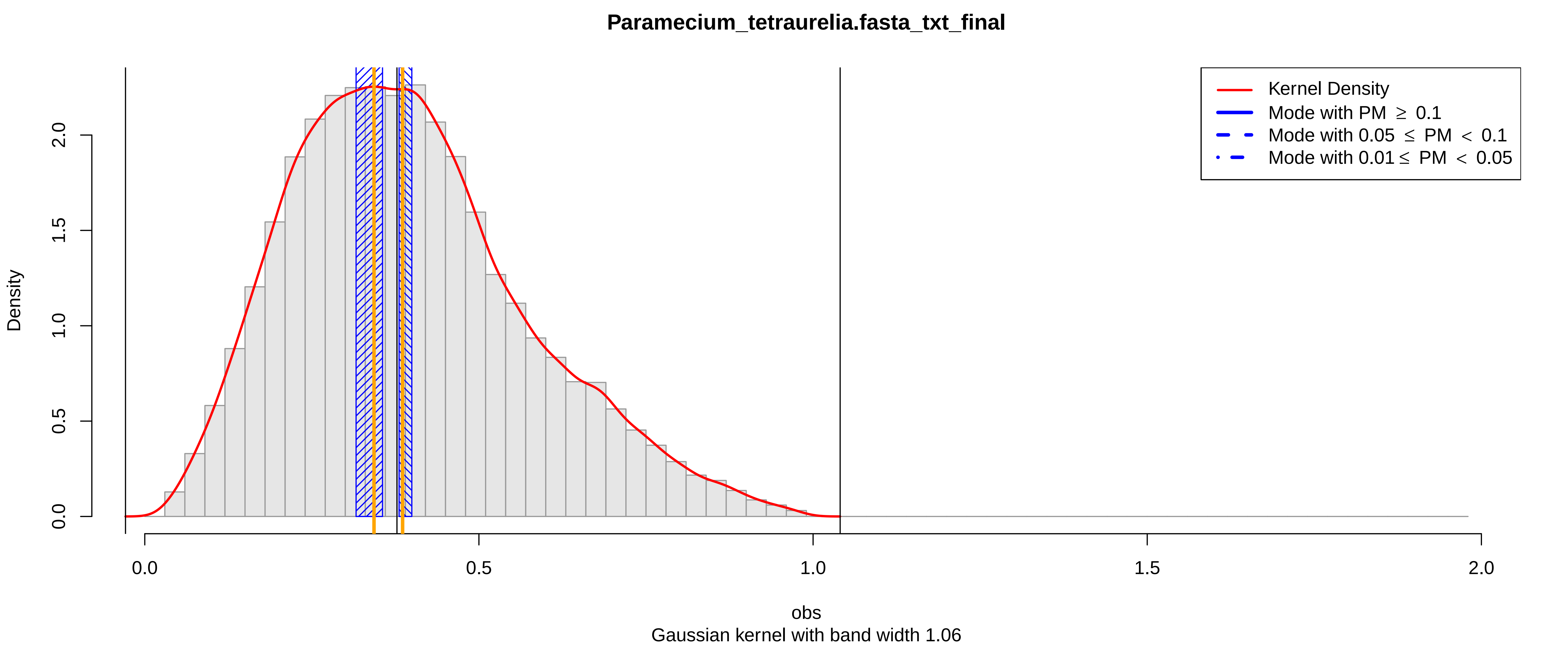

Paramecium_tetraurelia.fasta_txt_final
Kernel Density
Mode with PM ≥ 0.1
Mode with 0.05 ≤ PM < 0.1
Mode with 0.01 ≤ PM < 0.05
Density
0.0
0.5
1.0
1.5
2.0
0.0
0.5
1.0
1.5
2.0
obs
Gaussian kernel with band width 1.06

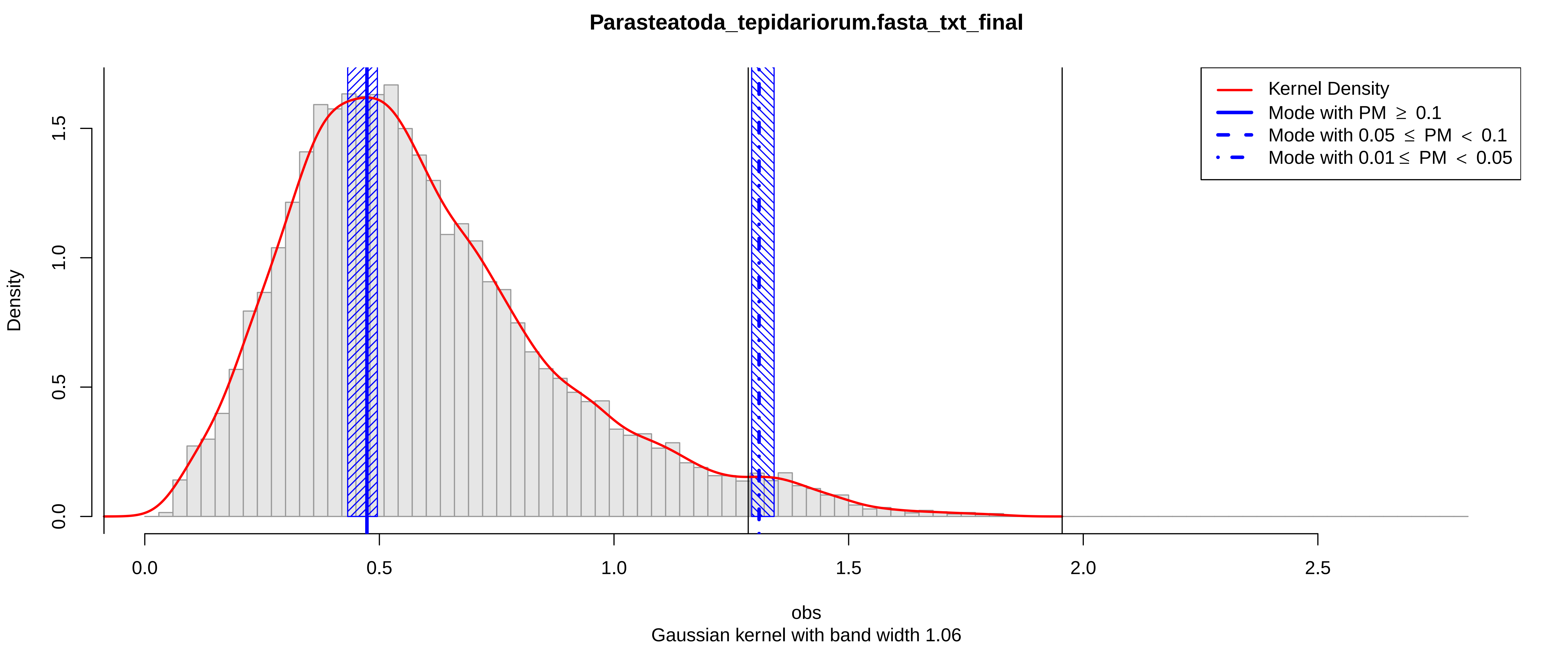

Parasteatoda_tepidariorum.fasta_txt_final
Kernel Density
Mode with PM ≥ 0.1
Mode with 0.05 ≤ PM < 0.1
Mode with 0.01 ≤ PM < 0.05
Density
0.0
0.5
1.0
1.5
0.0
0.5
1.0
1.5
2.0
2.5
obs
Gaussian kernel with band width 1.06

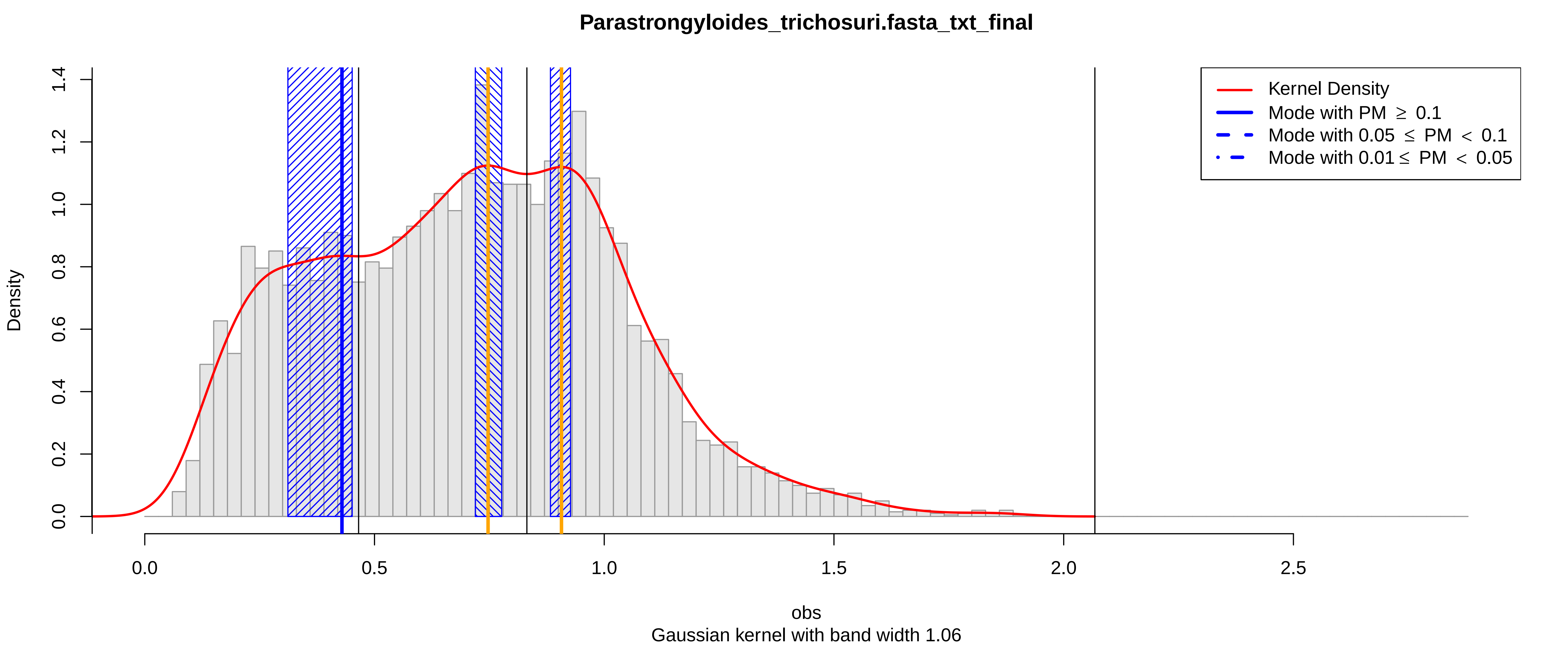
Parastrongyloides_trichosuri.fasta_txt_final
Density
obs
Gaussian kernel with band width 1.06
Kernel Density
Mode with PM ≥ 0.1
Mode with 0.05 ≤ PM < 0.1
Mode with 0.01 ≤ PM < 0.05
0.0
0.2
0.4
0.6
0.8
1.0
1.2
1.4
0.0
0.5
1.0
1.5
2.0
2.5

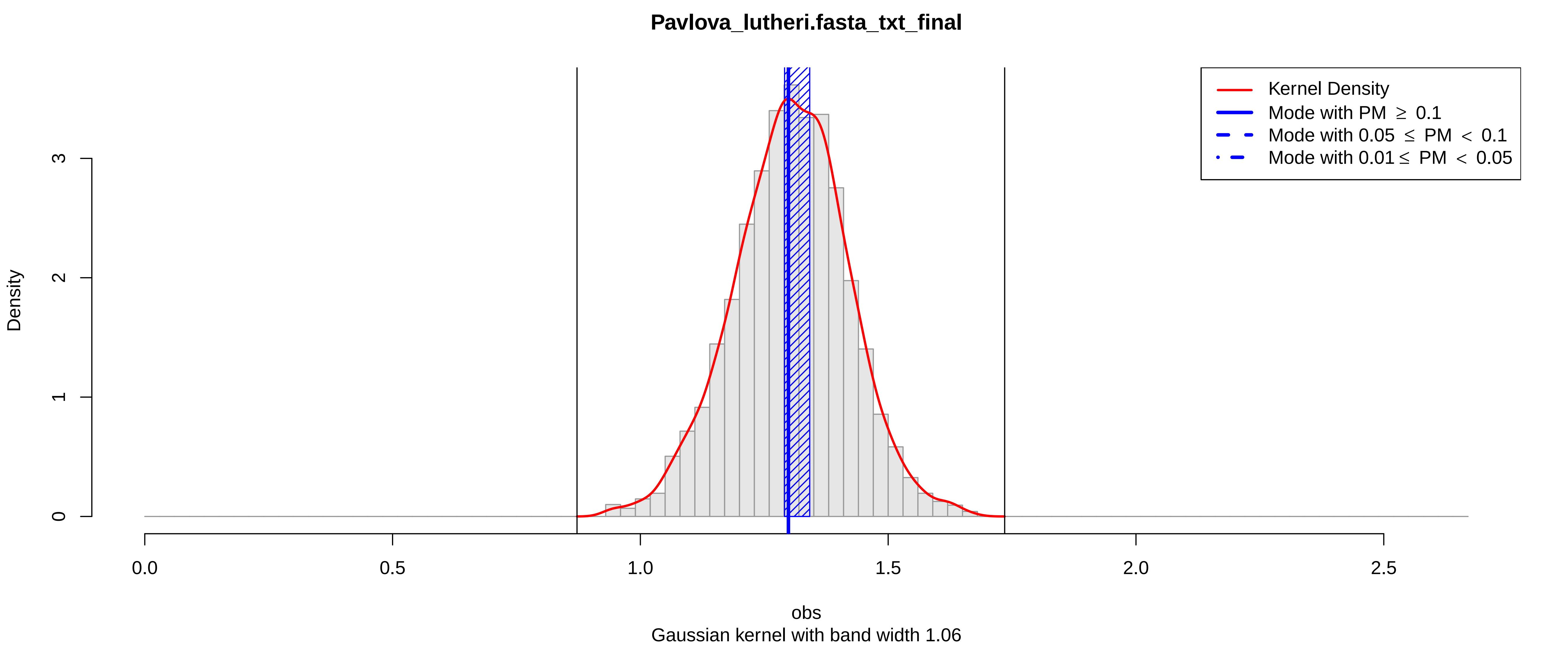

Pavlova_lutheri.fasta_txt_final
Kernel Density
Mode with PM ≥ 0.1
Mode with 0.05 ≤ PM < 0.1
Mode with 0.01 ≤ PM < 0.05
Density
0
1
2
3
0.0
0.5
1.0
1.5
2.0
2.5
obs
Gaussian kernel with band width 1.06

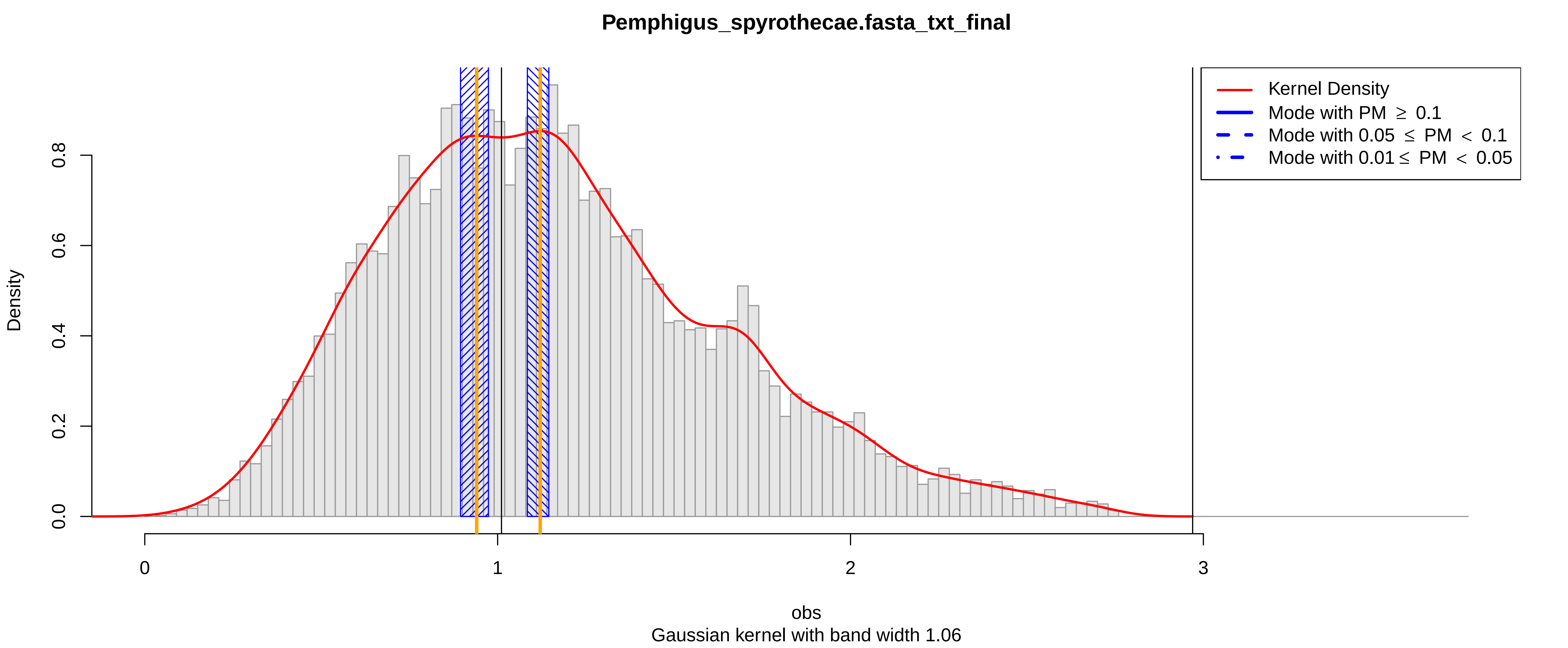
Pemphigus_spyrothecae.fasta_txt_final
Kernel Density
Mode with PM ≥ 0.1
Mode with 0.05 ≤ PM < 0.1
Mode with 0.01 ≤ PM < 0.05
Density
0.0
0.2
0.4
0.6
0.8
0
1
2
3
obs
Gaussian kernel with band width 1.06

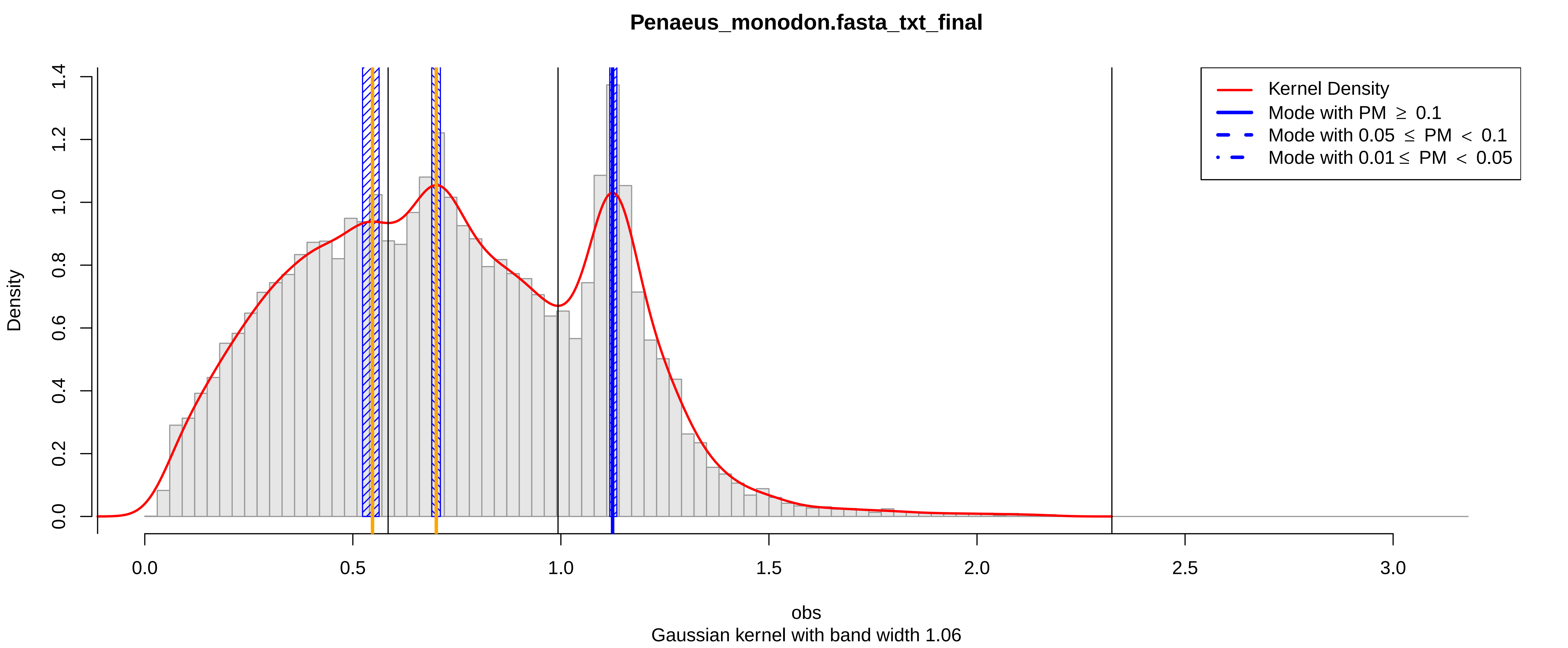

Penaeus_monodon.fasta_txt_final
Kernel Density
Mode with PM ≥ 0.1
Mode with 0.05 ≤ PM < 0.1
Mode with 0.01 ≤ PM < 0.05
Density
0.0
0.2
0.4
0.6
0.8
1.0
1.2
1.4
0.0
0.5
1.0
1.5
2.0
2.5
3.0
obs
Gaussian kernel with band width 1.06

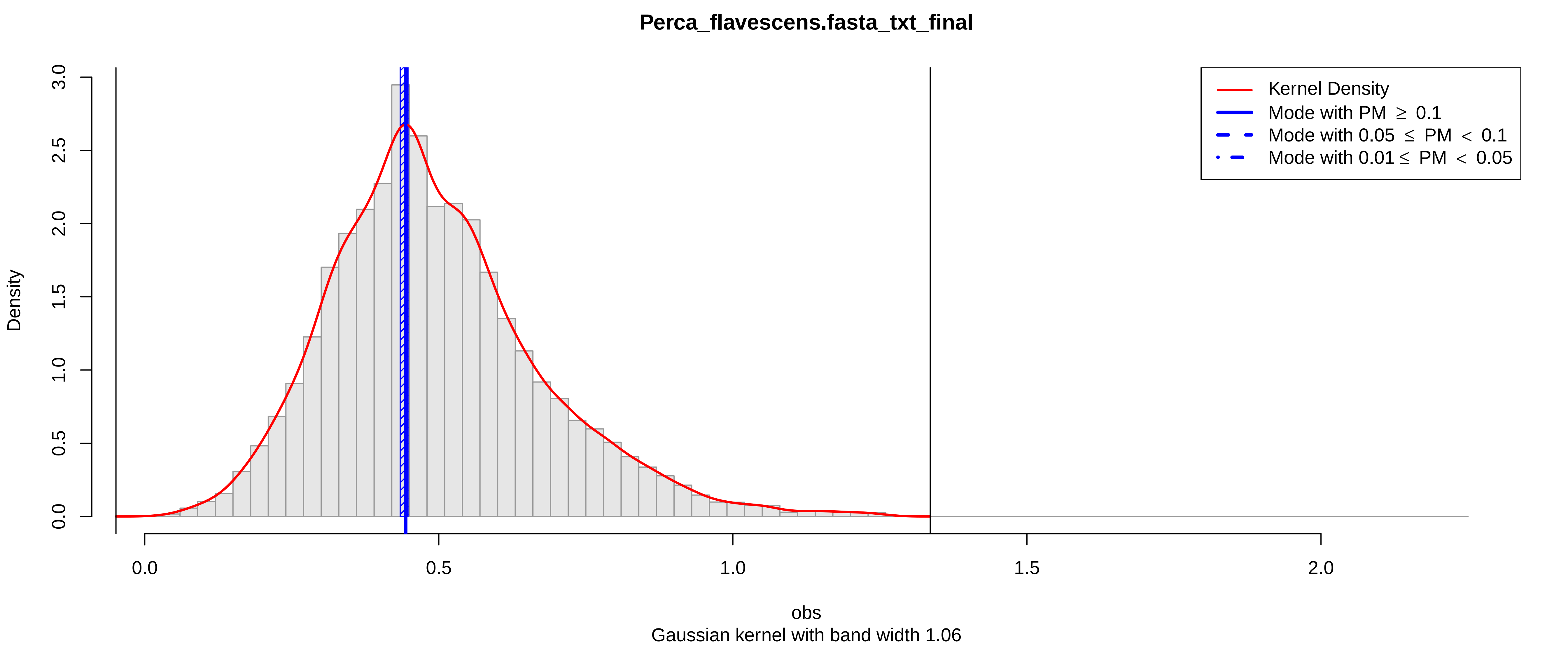
Perca_flavescens.fasta_txt_final
Density
obs
Gaussian kernel with band width 1.06
Kernel Density
Mode with PM ≥ 0.1
Mode with 0.05 ≤ PM < 0.1
Mode with 0.01 ≤ PM < 0.05
0.0
0.5
1.0
1.5
2.0
2.5
3.0

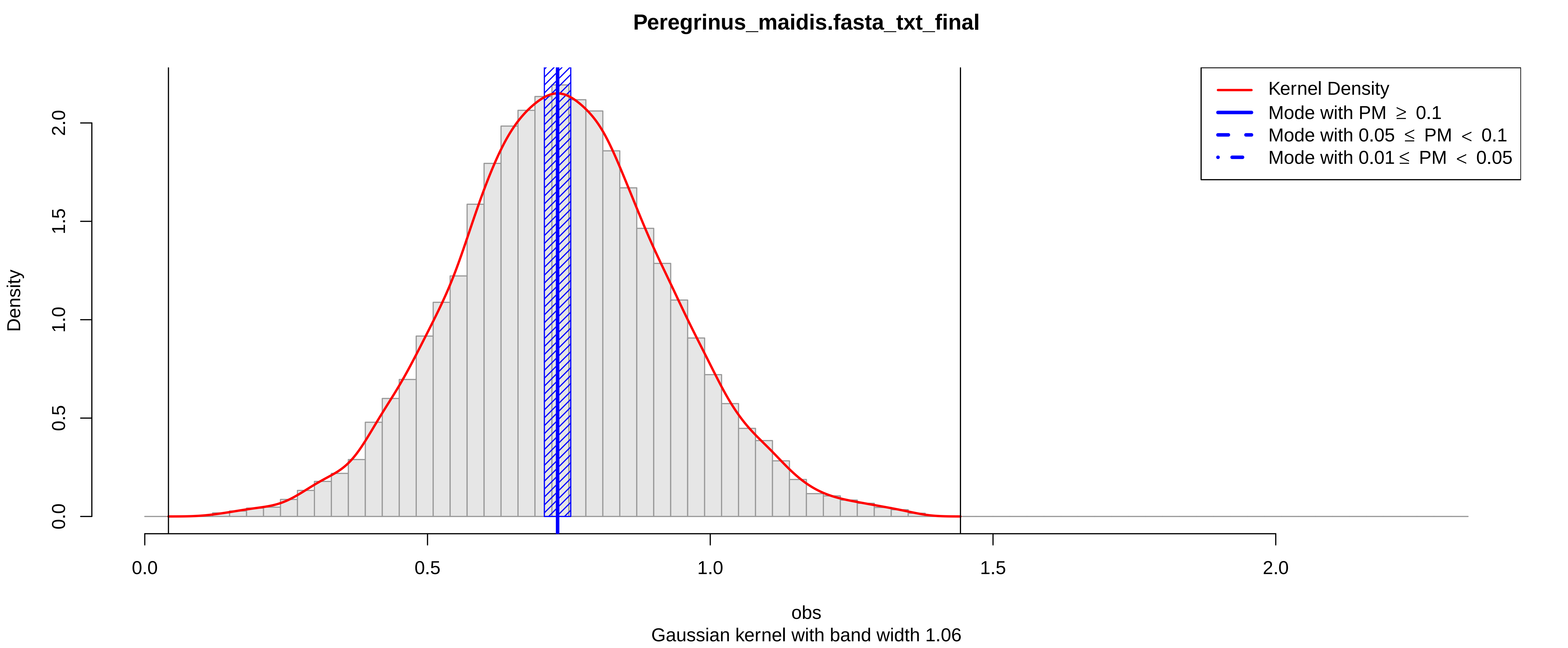

Peregrinus_maidis.fasta_txt_final
Kernel Density
Mode with PM ≥ 0.1
Mode with 0.05 ≤ PM < 0.1
Mode with 0.01 ≤ PM < 0.05
Density
0.0
0.5
1.0
1.5
2.0
0.0
0.5
1.0
1.5
2.0
obs
Gaussian kernel with band width 1.06

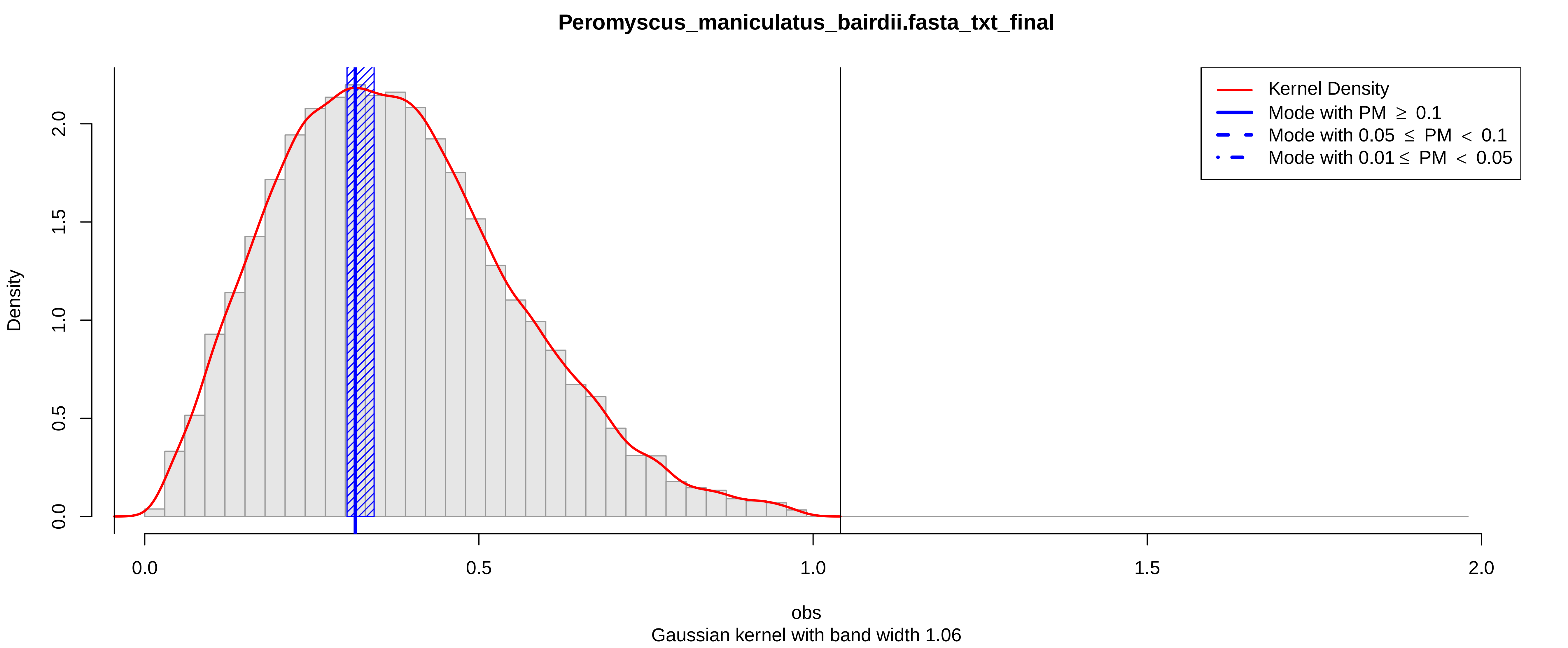
Peromyscus_maniculatus_bairdii.fasta_txt_final
Kernel Density
Mode with PM ≥ 0.1
Mode with 0.05 ≤ PM < 0.1
Mode with 0.01 ≤ PM < 0.05
Density
0.0
0.5
1.0
1.5
2.0
0.0
0.5
1.0
1.5
2.0
obs
Gaussian kernel with band width 1.06

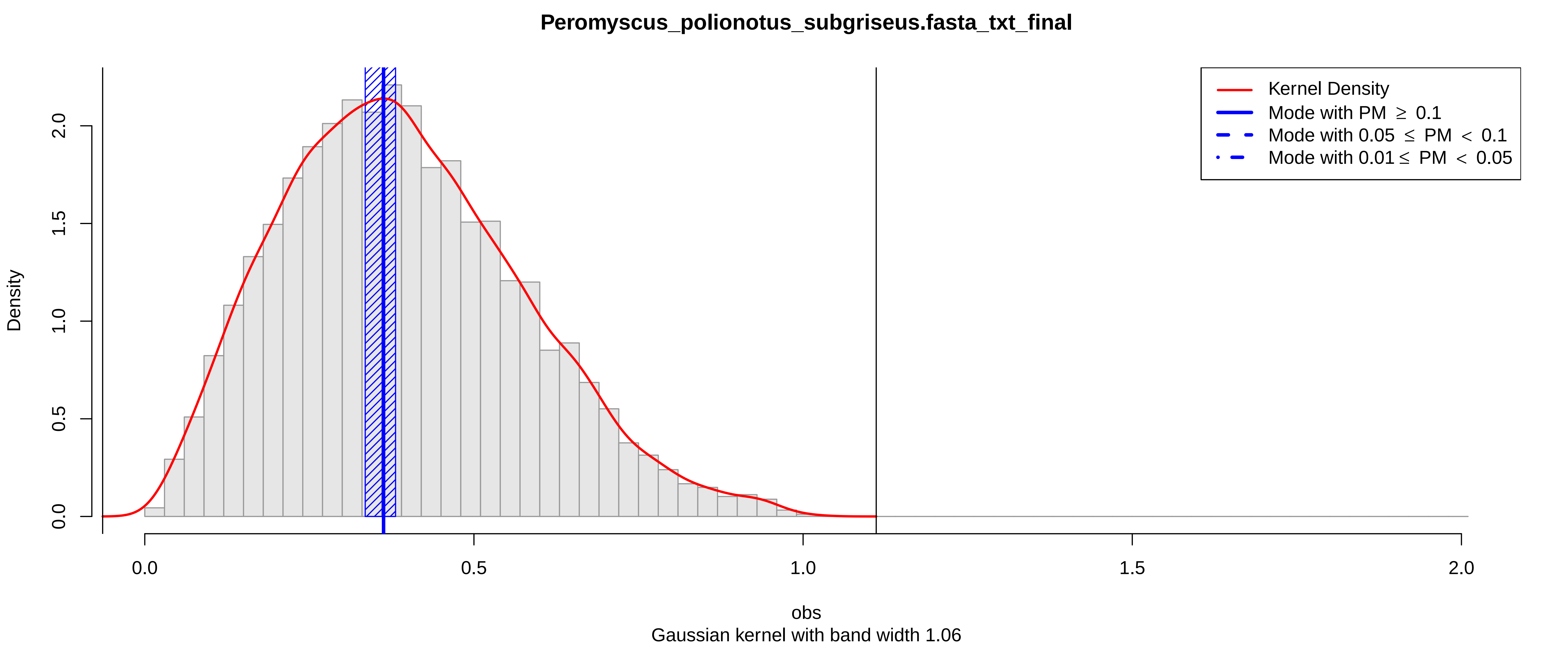

Peromyscus_polionotus_subgriseus.fasta_txt_final
Kernel Density
Mode with PM ≥ 0.1
Mode with 0.05 ≤ PM < 0.1
Mode with 0.01 ≤ PM < 0.05
Density
0.0
0.5
1.0
1.5
2.0
obs
Gaussian kernel with band width 1.06

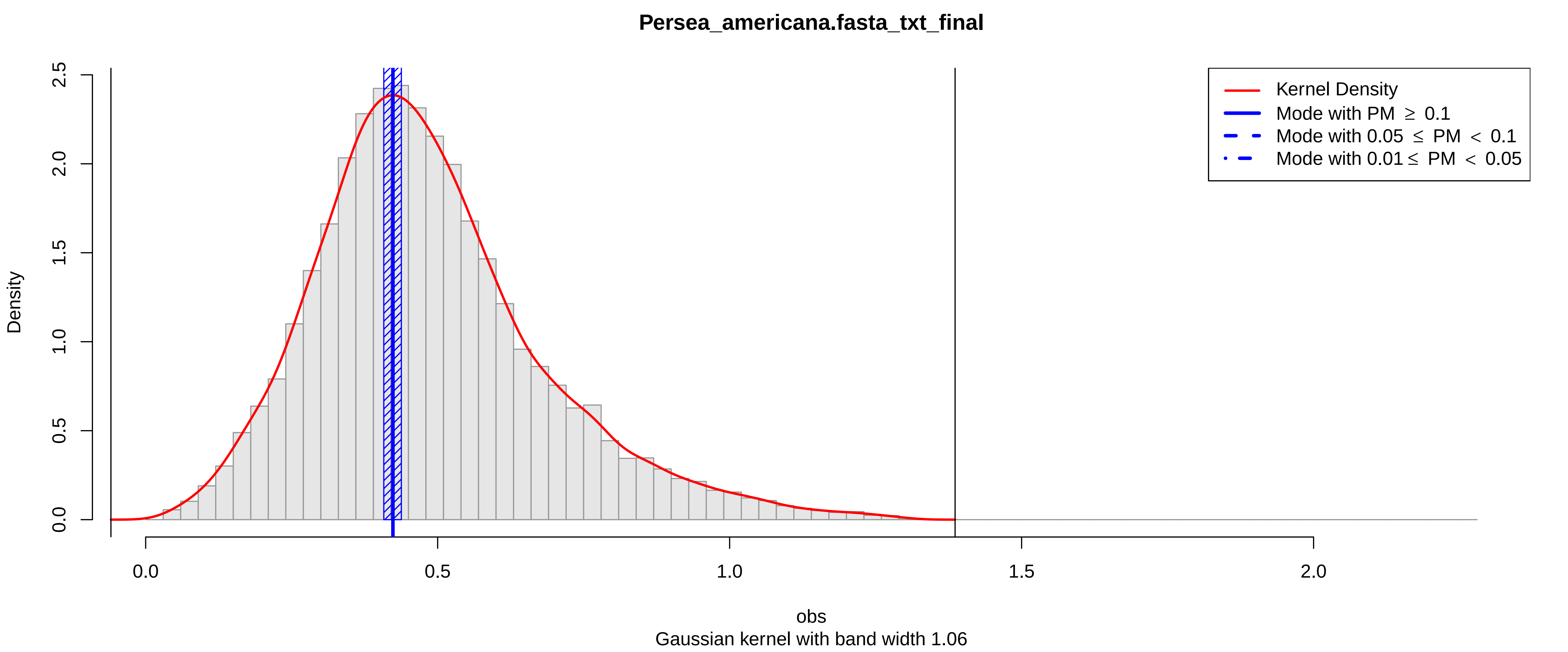

Persea_americana.fasta_txt_final
Kernel Density
Mode with PM ≥ 0.1
Mode with 0.05 ≤ PM < 0.1
Mode with 0.01 ≤ PM < 0.05
Density
0.0
0.5
1.0
1.5
2.0
2.5
obs
Gaussian kernel with band width 1.06

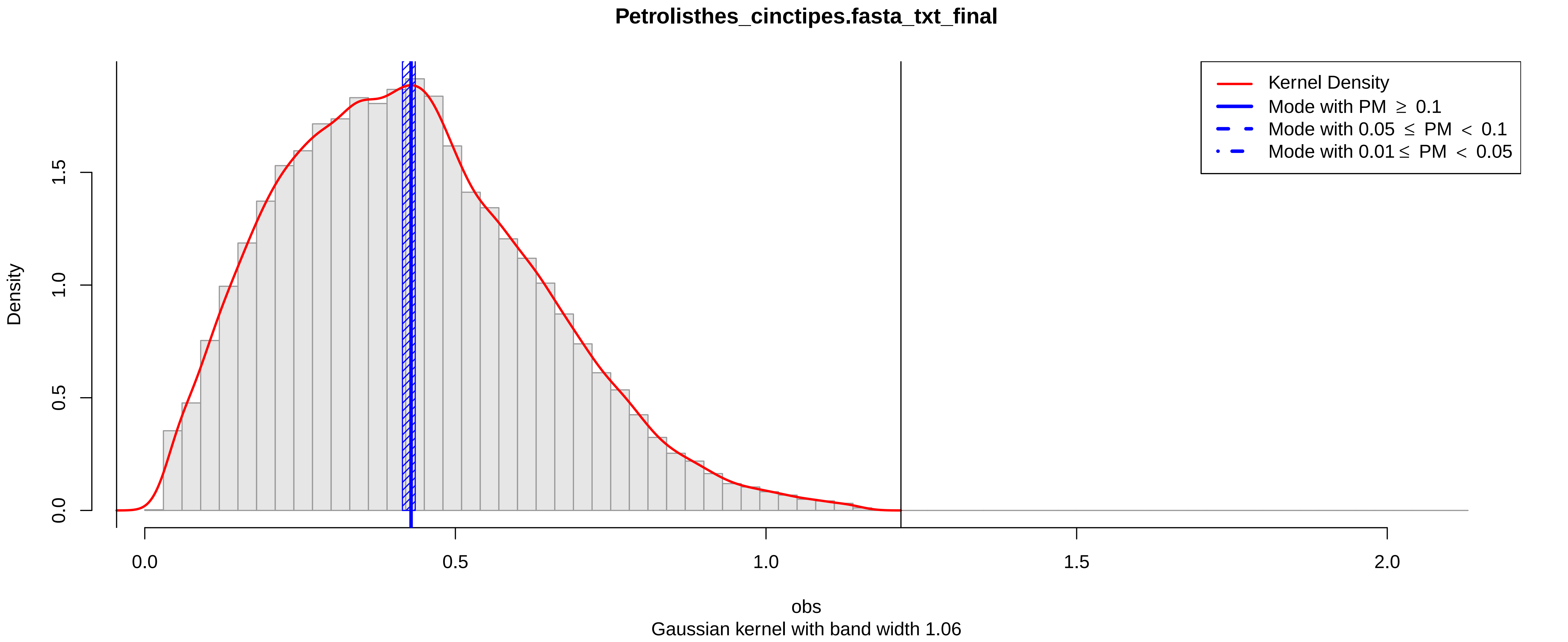

Petrolisthes_cinctipes.fasta_txt_final
Kernel Density
Mode with PM ≥ 0.1
Mode with 0.05 ≤ PM < 0.1
Mode with 0.01 ≤ PM < 0.05
Density
0.0
0.5
1.0
1.5
2.0
obs
Gaussian kernel with band width 1.06

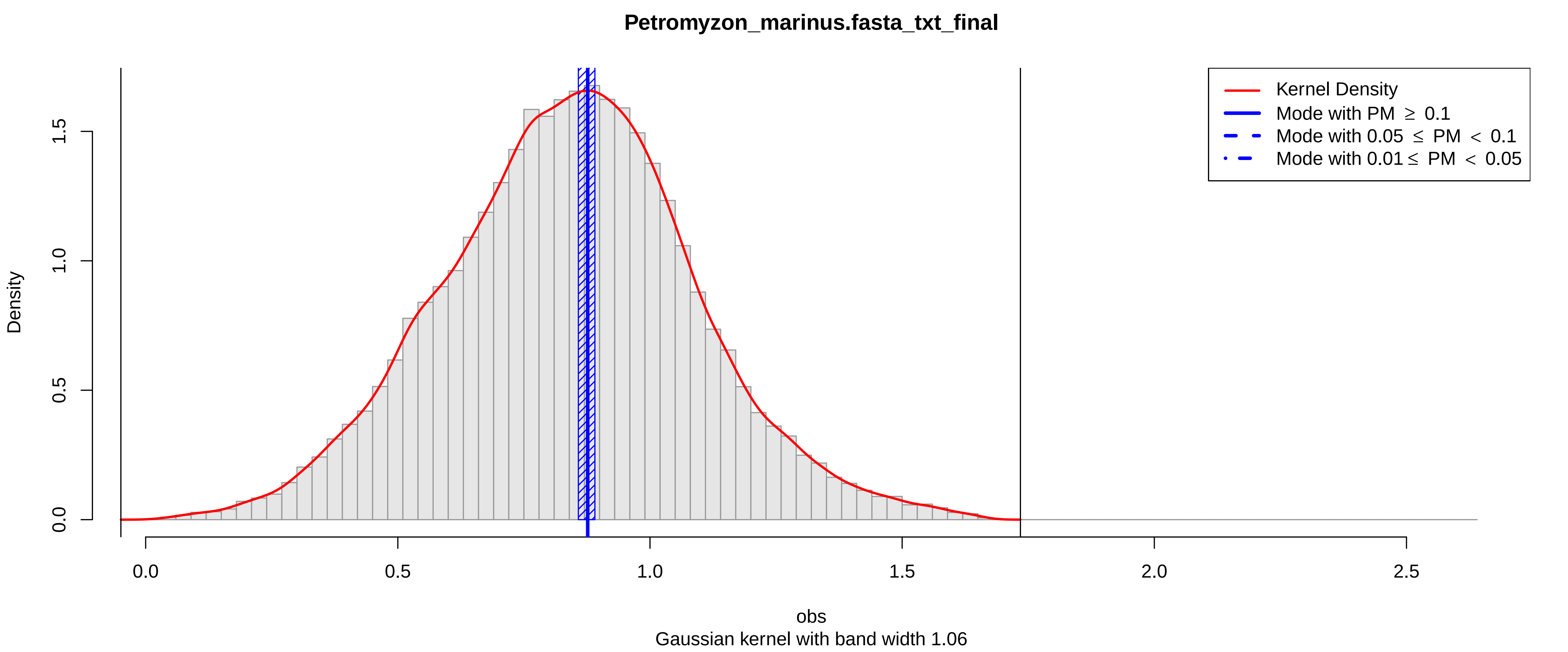

Petromyzon_marinus.fasta_txt_final
Kernel Density
Mode with PM ≥ 0.1
Mode with 0.05 ≤ PM < 0.1
Mode with 0.01 ≤ PM < 0.05
Density
0.0
0.5
1.0
1.5
0.0
0.5
1.0
1.5
2.0
2.5
obs
Gaussian kernel with band width 1.06

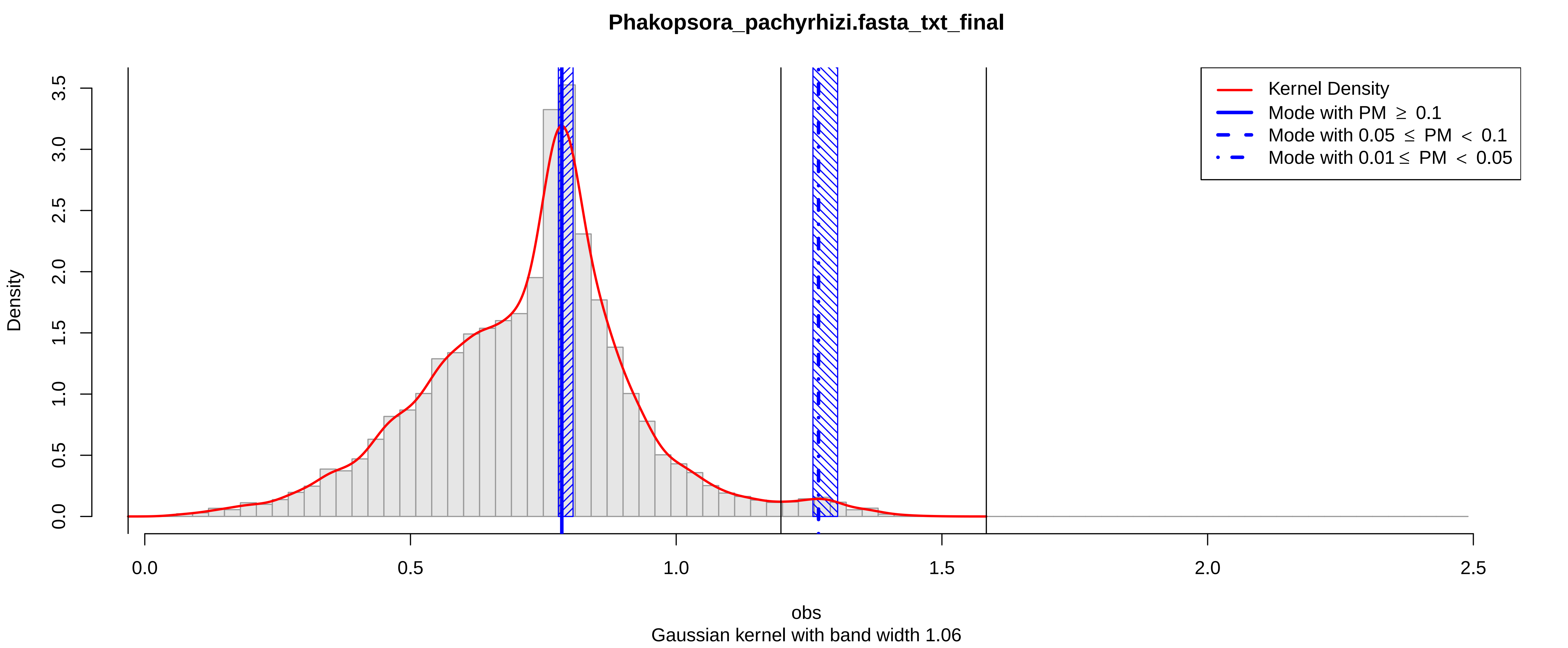

Phakopsora_pachyrhizi.fasta_txt_final
Density
0.0
0.5
1.0
1.5
2.0
2.5
3.0
3.5
Kernel Density
Mode with PM ≥ 0.1
Mode with 0.05 ≤ PM < 0.1
Mode with 0.01 ≤ PM < 0.05
0.0
0.5
1.0
1.5
2.0
2.5
obs
Gaussian kernel with band width 1.06

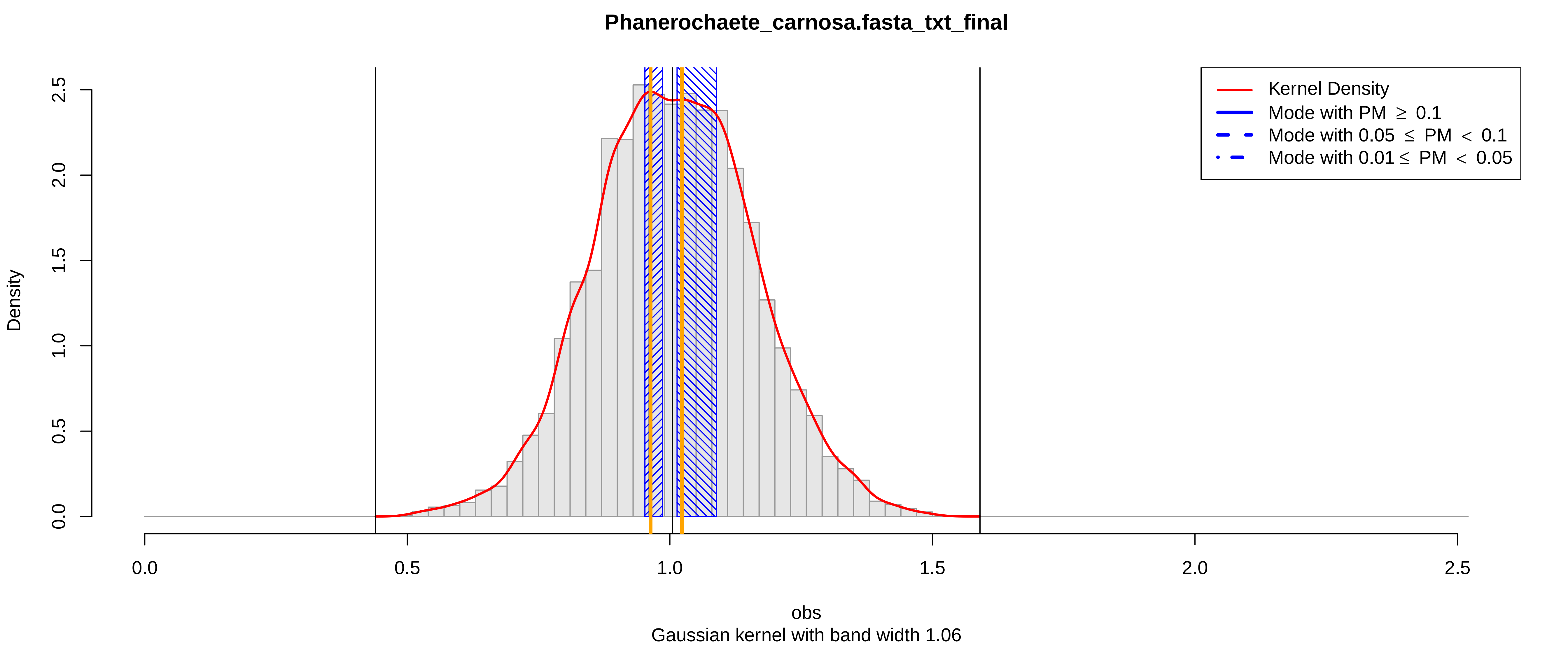

Phanerochaete_carnosa.fasta_txt_final
Kernel Density
Mode with PM ≥ 0.1
Mode with 0.05 ≤ PM < 0.1
Mode with 0.01 ≤ PM < 0.05
Density
0.0
0.5
1.0
1.5
2.0
2.5
obs
Gaussian kernel with band width 1.06

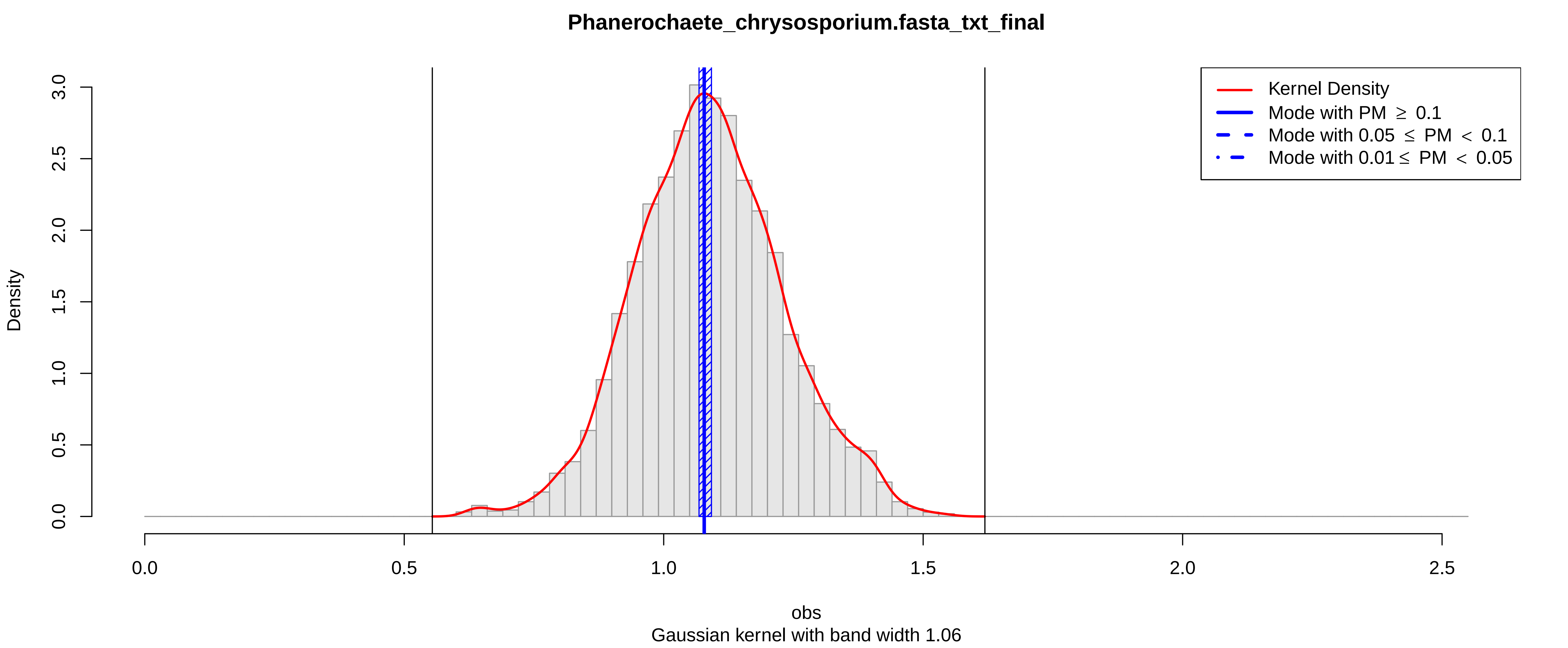

Phanerochaete_chrysosporium.fasta_txt_final
Kernel Density
Mode with PM ≥ 0.1
Mode with 0.05 ≤ PM < 0.1
Mode with 0.01 ≤ PM < 0.05
Density
0.0
0.5
1.0
1.5
2.0
2.5
3.0
0.0
0.5
1.0
1.5
2.0
2.5
obs
Gaussian kernel with band width 1.06

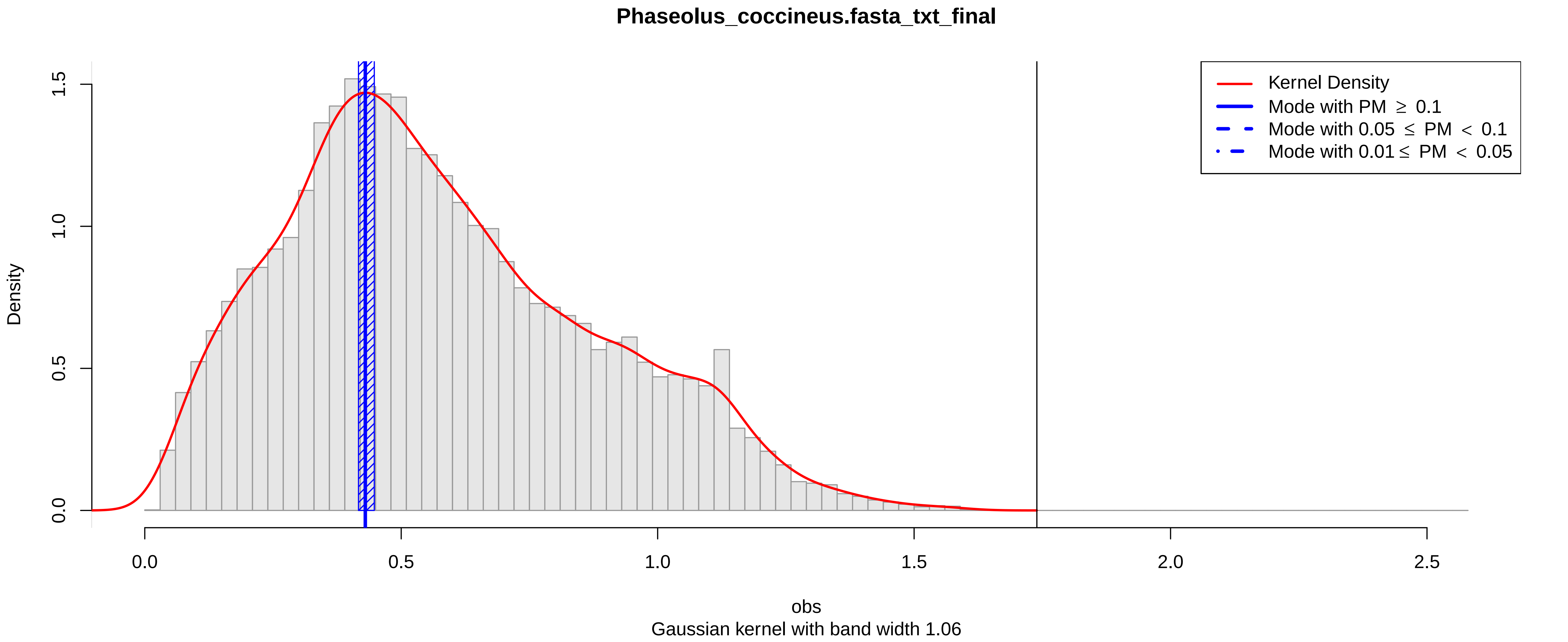
Phaseolus_coccineus.fasta_txt_final
Kernel Density
Mode with PM ≥ 0.1
Mode with 0.05 ≤ PM < 0.1
Mode with 0.01 ≤ PM < 0.05
Density
0.0
0.5
1.0
1.5
0.0
0.5
1.0
1.5
2.0
2.5
obs
Gaussian kernel with band width 1.06

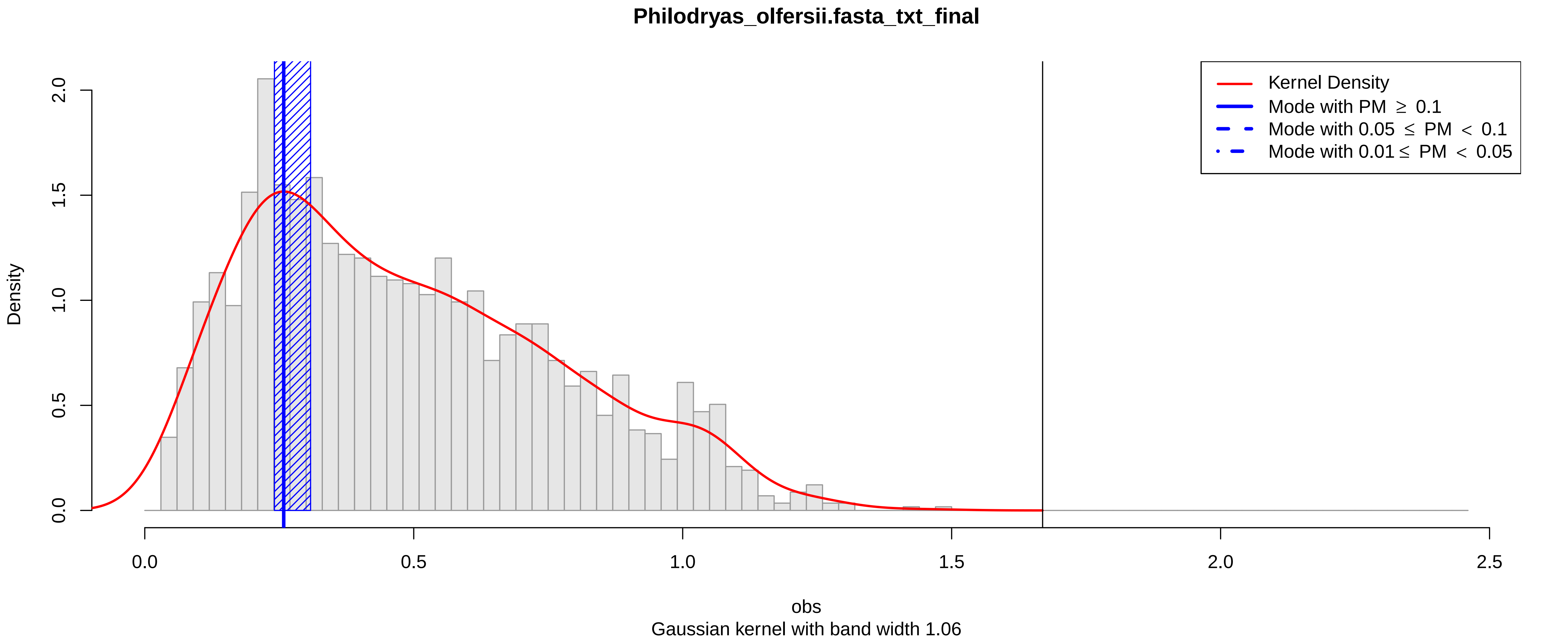
Philodryas_olfersii.fasta_txt_final
Kernel Density
Mode with PM ≥ 0.1
Mode with 0.05 ≤ PM < 0.1
Mode with 0.01 ≤ PM < 0.05
Density
0.0
0.5
1.0
1.5
2.0
0.0
0.5
1.0
1.5
2.0
2.5
obs
Gaussian kernel with band width 1.06

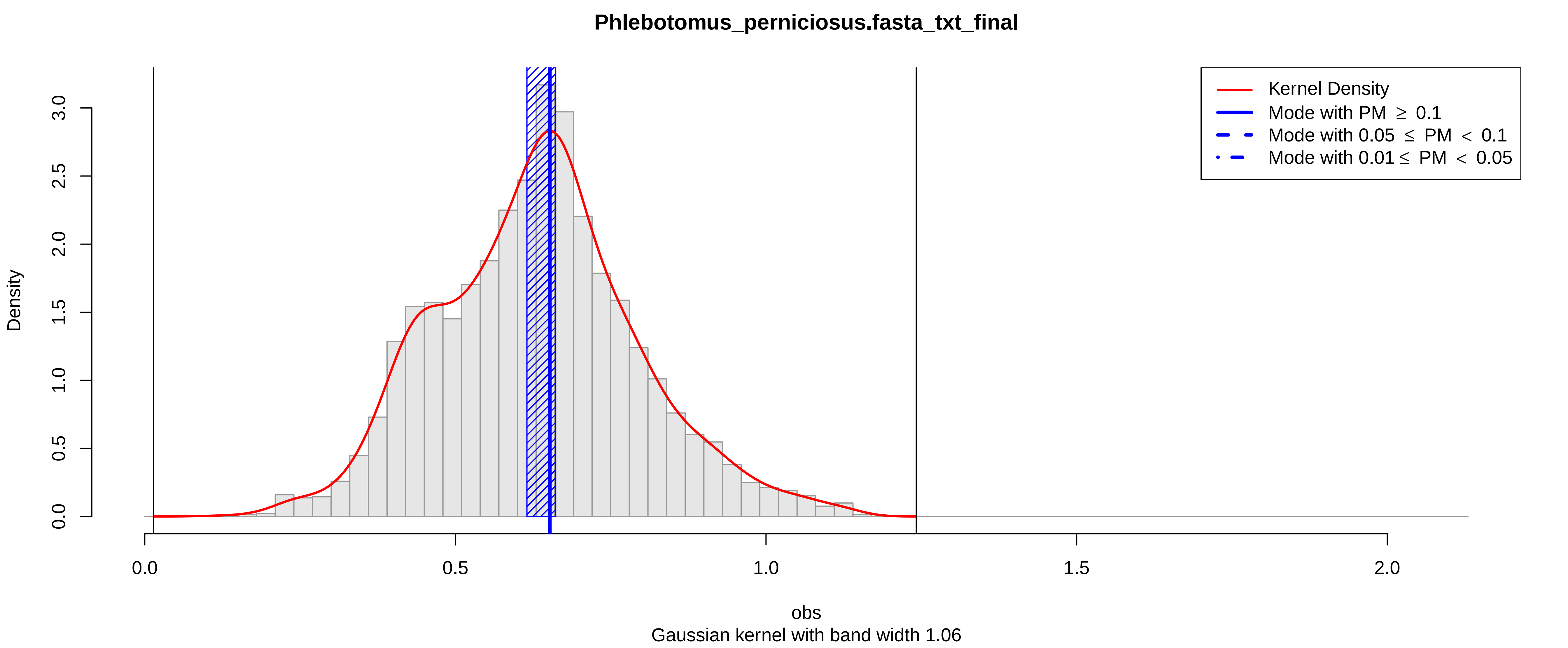

Phlebotomus_perniciosus.fasta_txt_final
Kernel Density
Mode with PM ≥ 0.1
Mode with 0.05 ≤ PM < 0.1
Mode with 0.01 ≤ PM < 0.05
Density
0.0
0.5
1.0
1.5
2.0
2.5
3.0
0.0
0.5
1.0
1.5
2.0
obs
Gaussian kernel with band width 1.06

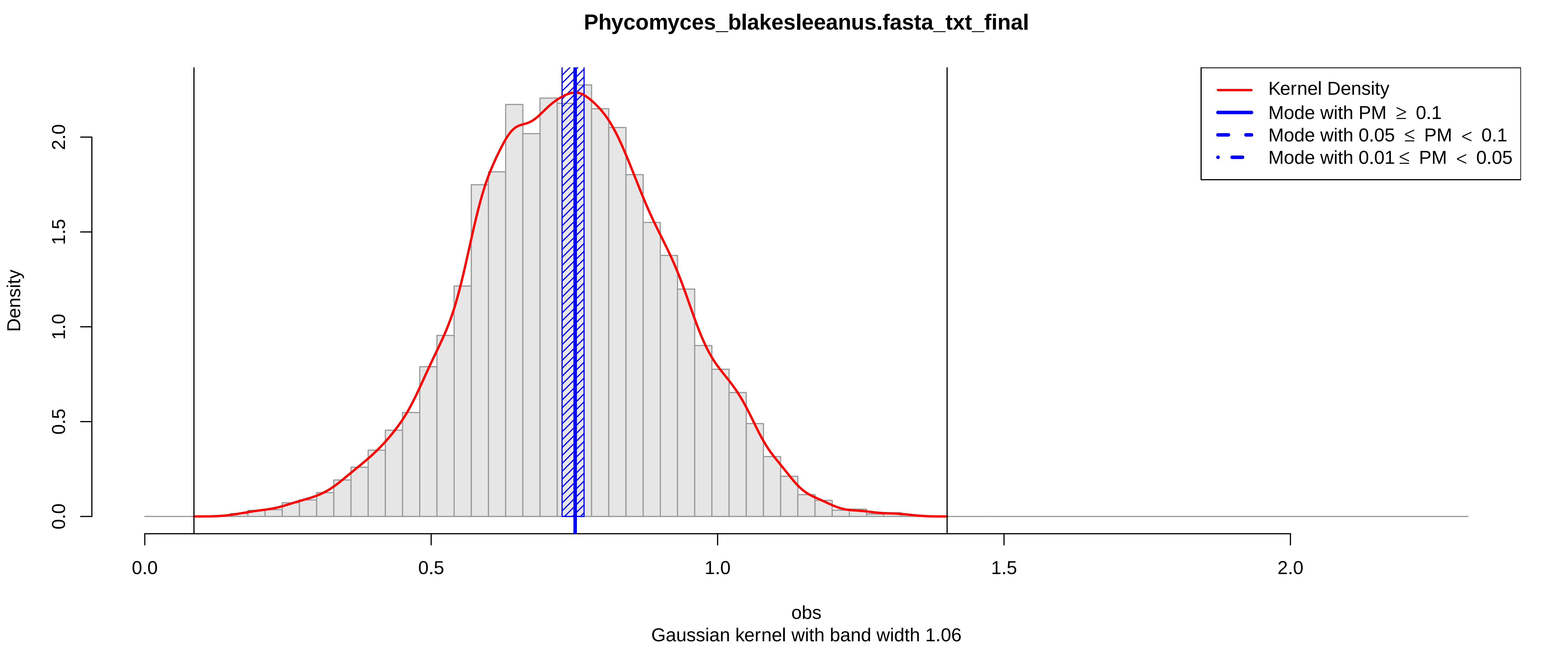

Phycomyces_blakesleeanus.fasta_txt_final
Kernel Density
Mode with PM ≥ 0.1
Mode with 0.05 ≤ PM < 0.1
Mode with 0.01 ≤ PM < 0.05
Density
0.0
0.5
1.0
1.5
2.0
0.0
0.5
1.0
1.5
2.0
obs
Gaussian kernel with band width 1.06

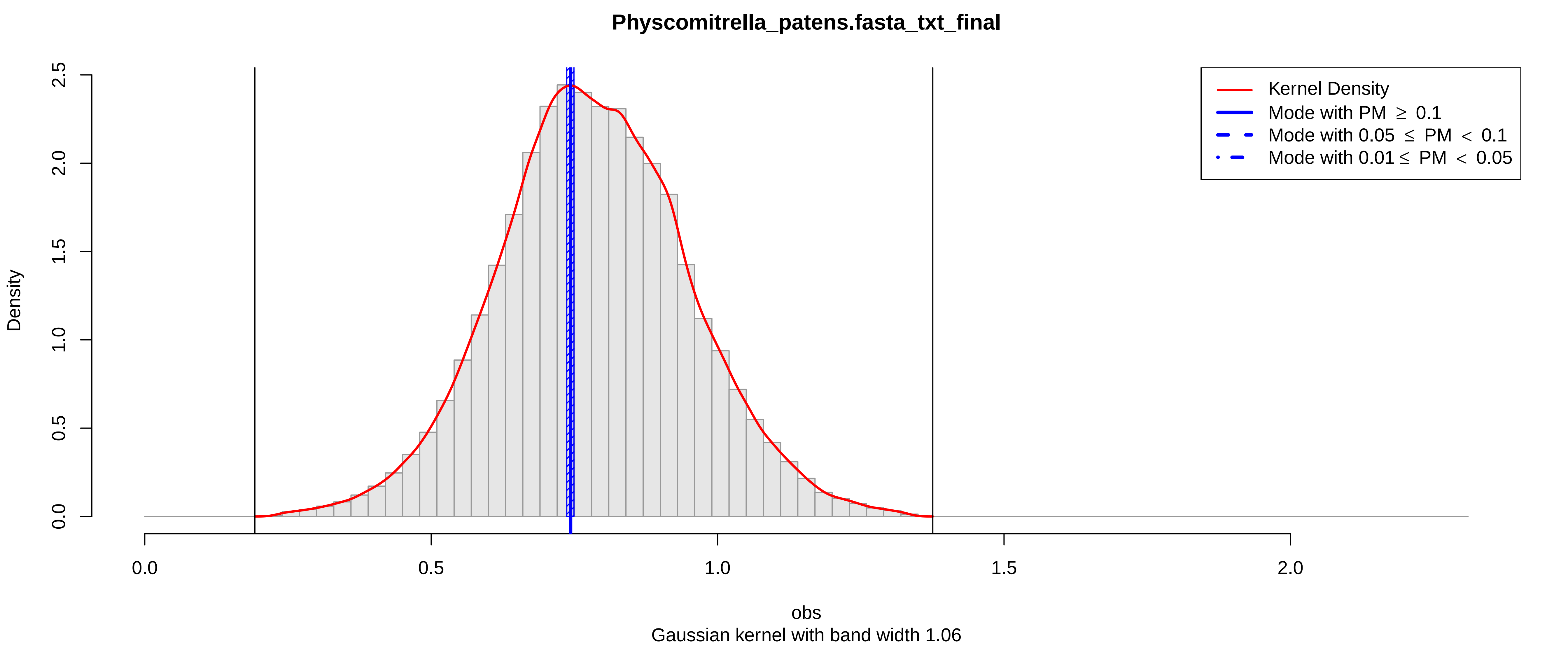

Physcomitrella_patens.fasta_txt_final
Density
0.0
0.5
1.0
1.5
2.0
2.5
Kernel Density
Mode with PM ≥ 0.1
Mode with 0.05 ≤ PM < 0.1
Mode with 0.01 ≤ PM < 0.05
0.0
0.5
1.0
1.5
2.0
obs
Gaussian kernel with band width 1.06

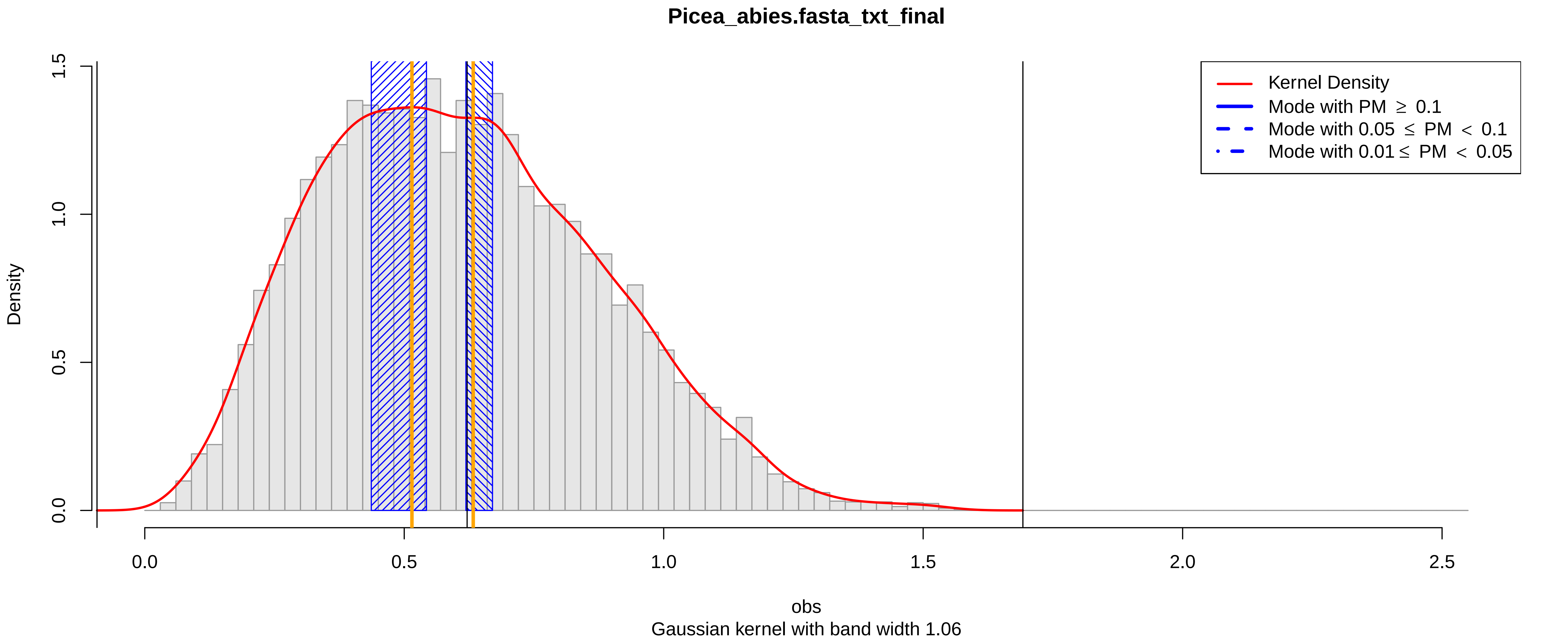

Picea_abies.fasta_txt_final
Kernel Density
Mode with PM ≥ 0.1
Mode with 0.05 ≤ PM < 0.1
Mode with 0.01 ≤ PM < 0.05
Density
0.0
0.5
1.0
1.5
0.0
0.5
1.0
1.5
2.0
2.5
obs
Gaussian kernel with band width 1.06

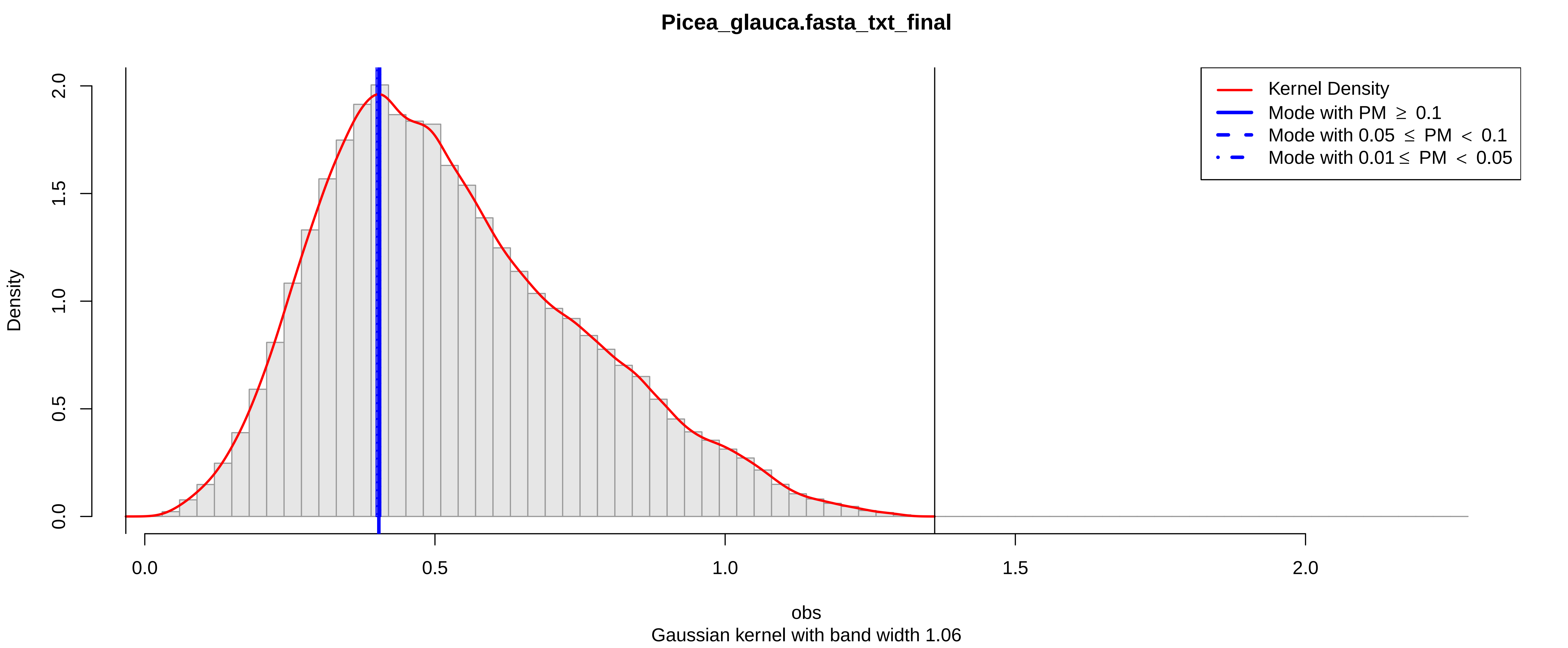
Picea_glauca.fasta_txt_final
Kernel Density
Mode with PM ≥ 0.1
Mode with 0.05 ≤ PM < 0.1
Mode with 0.01 ≤ PM < 0.05
Density
0.0
0.5
1.0
1.5
2.0
obs
Gaussian kernel with band width 1.06

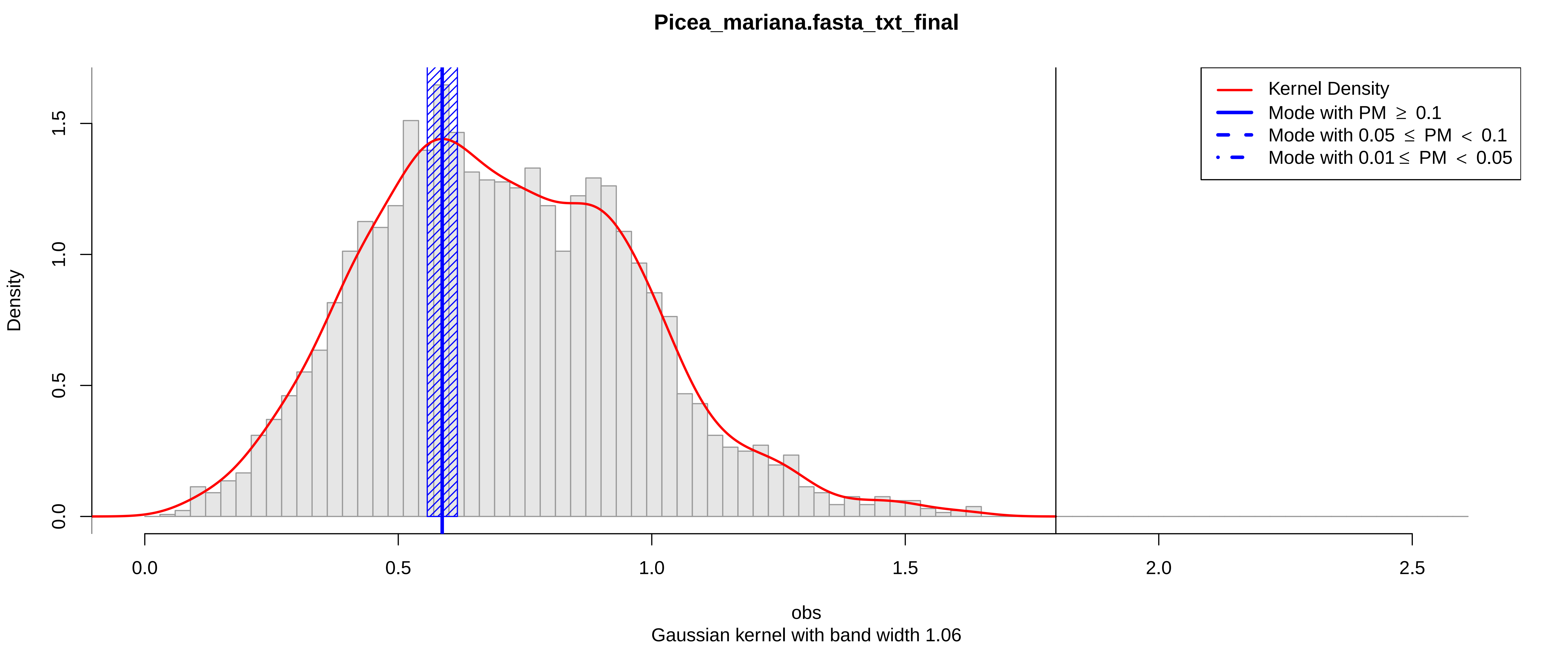
Picea_mariana.fasta_txt_final
Kernel Density
Mode with PM ≥ 0.1
Mode with 0.05 ≤ PM < 0.1
Mode with 0.01 ≤ PM < 0.05
Density
0.0
0.5
1.0
1.5
2.0
2.5
obs
Gaussian kernel with band width 1.06

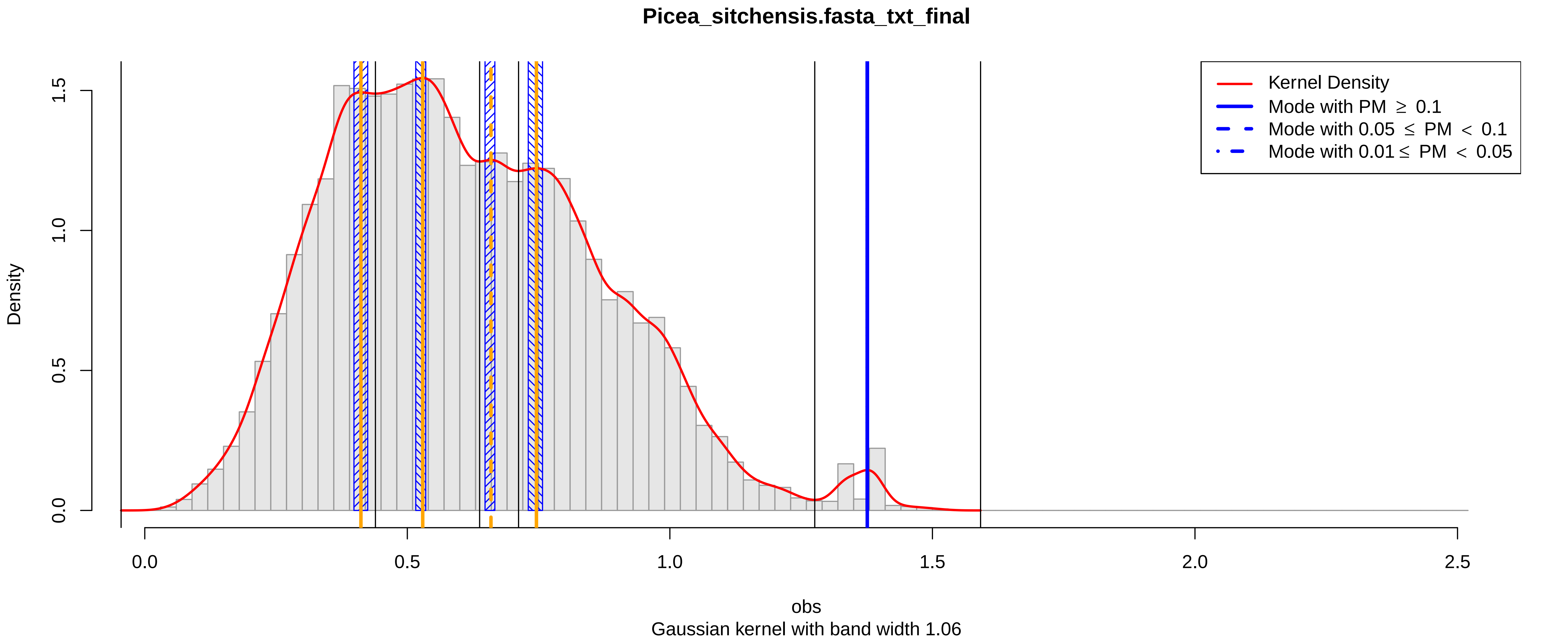
Picea_sitchensis.fasta_txt_final
Kernel Density
Mode with PM ≥ 0.1
Mode with 0.05 ≤ PM < 0.1
Mode with 0.01 ≤ PM < 0.05
Density
0.0
0.5
1.0
1.5
2.0
2.5
obs
Gaussian kernel with band width 1.06

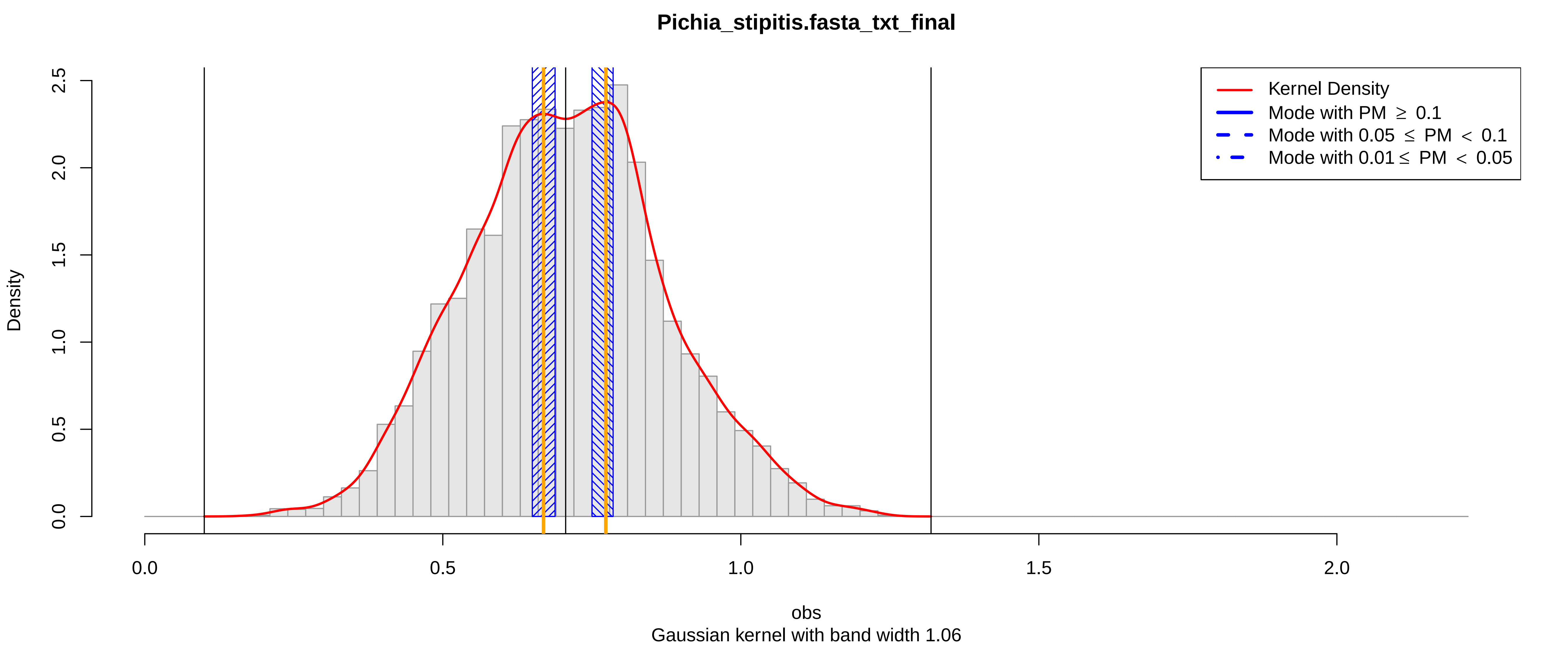
Pichia_stipitis.fasta_txt_final
Kernel Density
Mode with PM ≥ 0.1
Mode with 0.05 ≤ PM < 0.1
Mode with 0.01 ≤ PM < 0.05
Density
0.0
0.5
1.0
1.5
2.0
2.5
0.0
0.5
1.0
1.5
2.0
obs
Gaussian kernel with band width 1.06

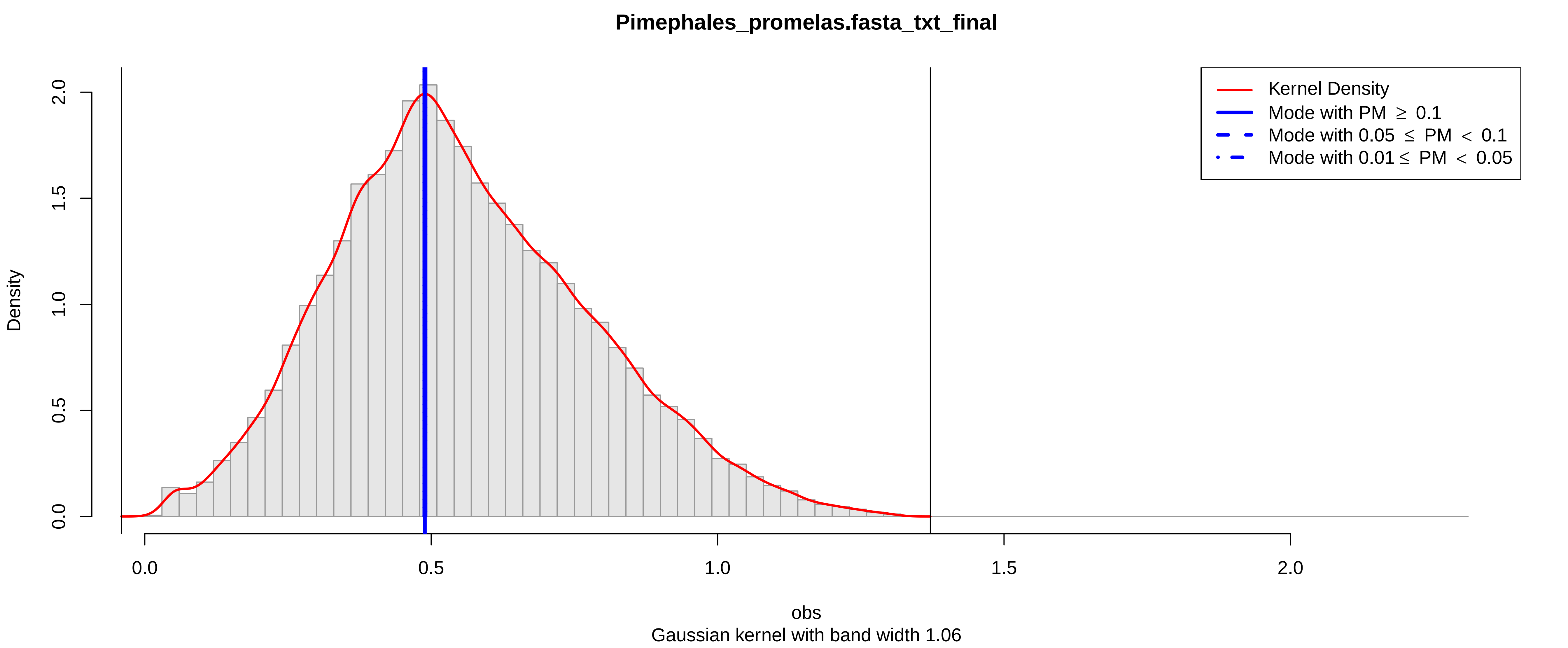
Pimephales_promelas.fasta_txt_final
Kernel Density
Mode with PM ≥ 0.1
Mode with 0.05 ≤ PM < 0.1
Mode with 0.01 ≤ PM < 0.05
Density
0.0
0.5
1.0
1.5
2.0
0.0
0.5
1.0
1.5
2.0
obs
Gaussian kernel with band width 1.06

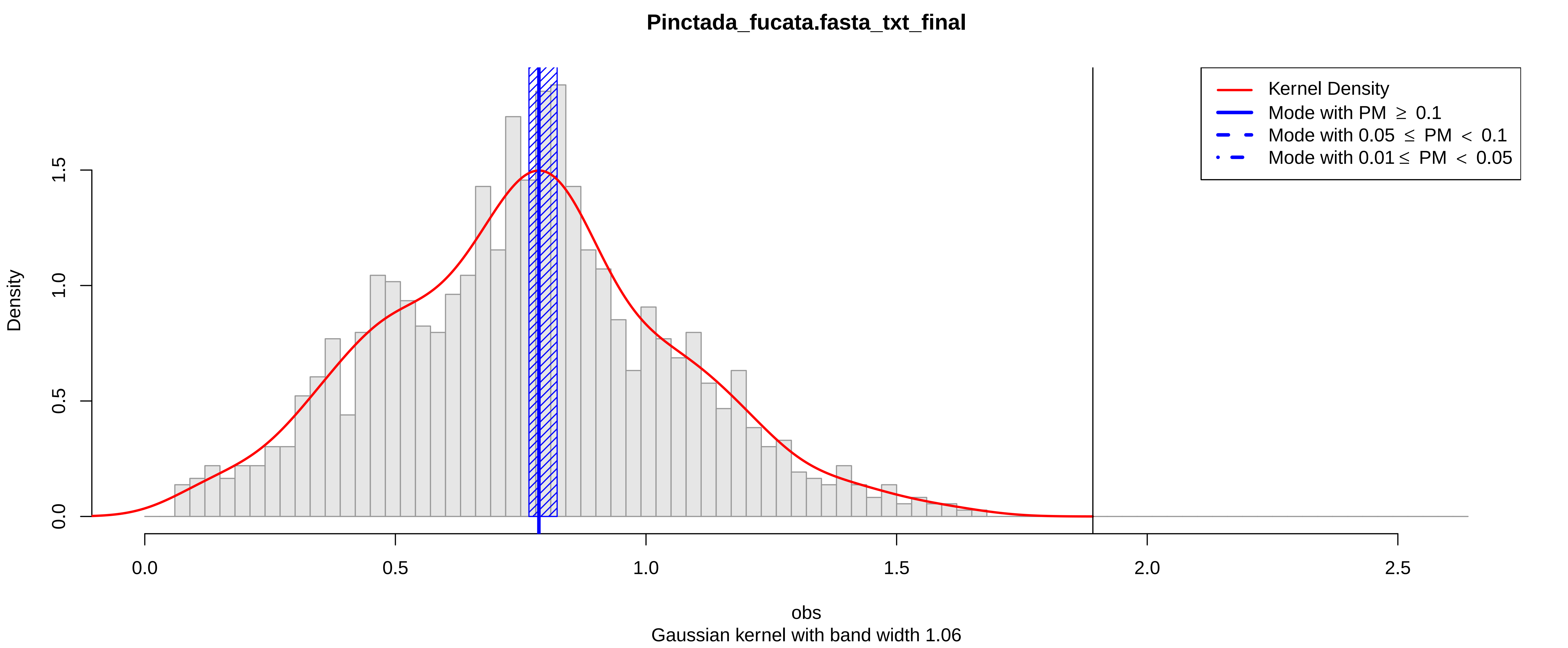

Pinctada_fucata.fasta_txt_final
Kernel Density
Mode with PM ≥ 0.1
Mode with 0.05 ≤ PM < 0.1
Mode with 0.01 ≤ PM < 0.05
Density
0.0
0.5
1.0
1.5
0.0
0.5
1.0
1.5
2.0
2.5
obs
Gaussian kernel with band width 1.06

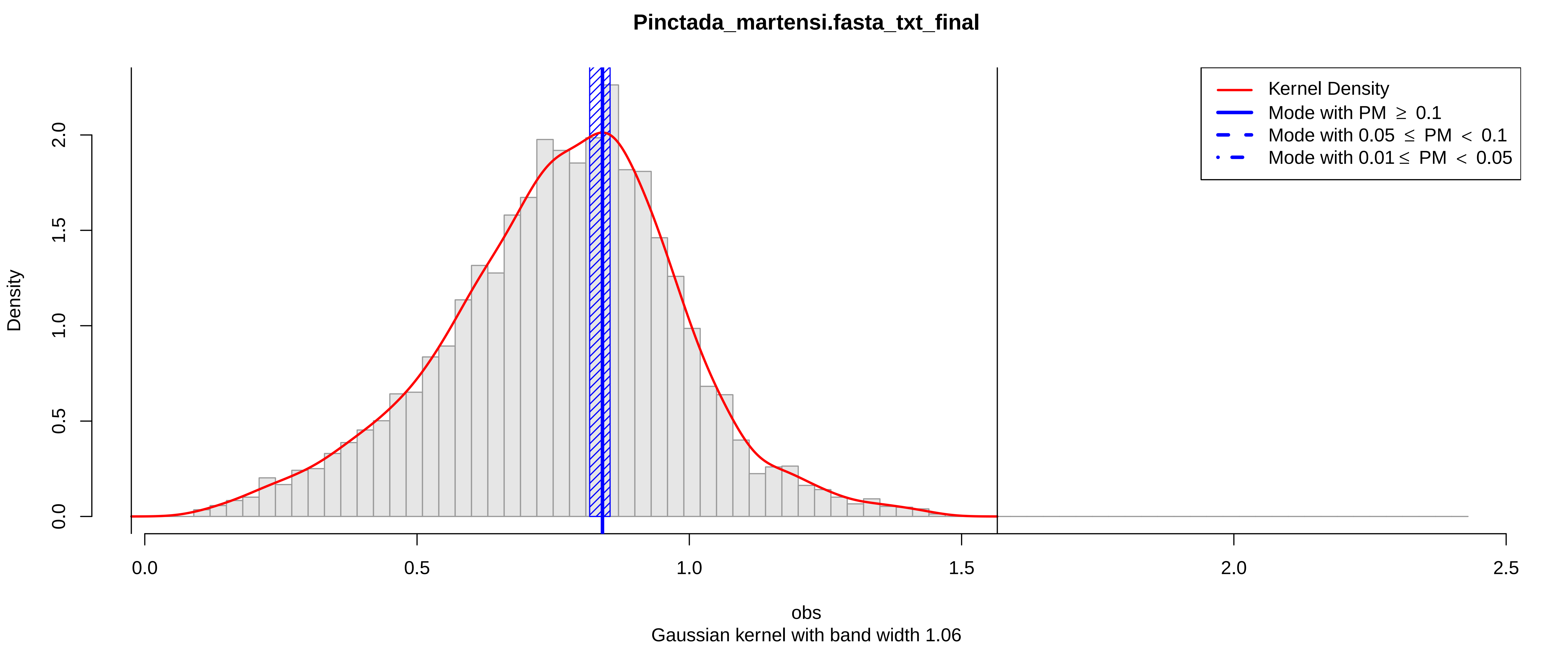
Pinctada_martensi.fasta_txt_final
Kernel Density
Mode with PM ≥ 0.1
Mode with 0.05 ≤ PM < 0.1
Mode with 0.01 ≤ PM < 0.05
Density
0.0
0.5
1.0
1.5
2.0
2.5
obs
Gaussian kernel with band width 1.06

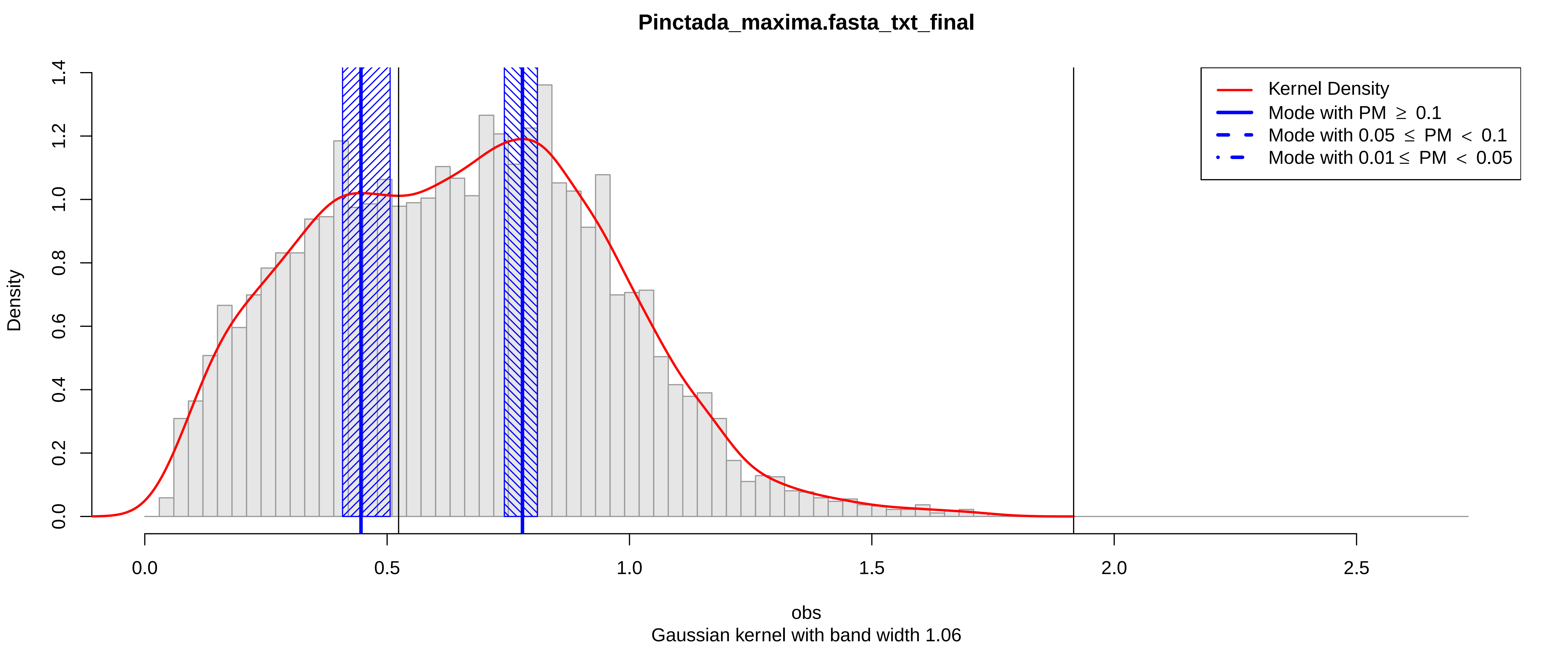

Pinctada_maxima.fasta_txt_final
Kernel Density
Mode with PM ≥ 0.1
Mode with 0.05 ≤ PM < 0.1
Mode with 0.01 ≤ PM < 0.05
Density
0.0
0.2
0.4
0.6
0.8
1.0
1.2
1.4
0.0
0.5
1.0
1.5
2.0
2.5
obs
Gaussian kernel with band width 1.06

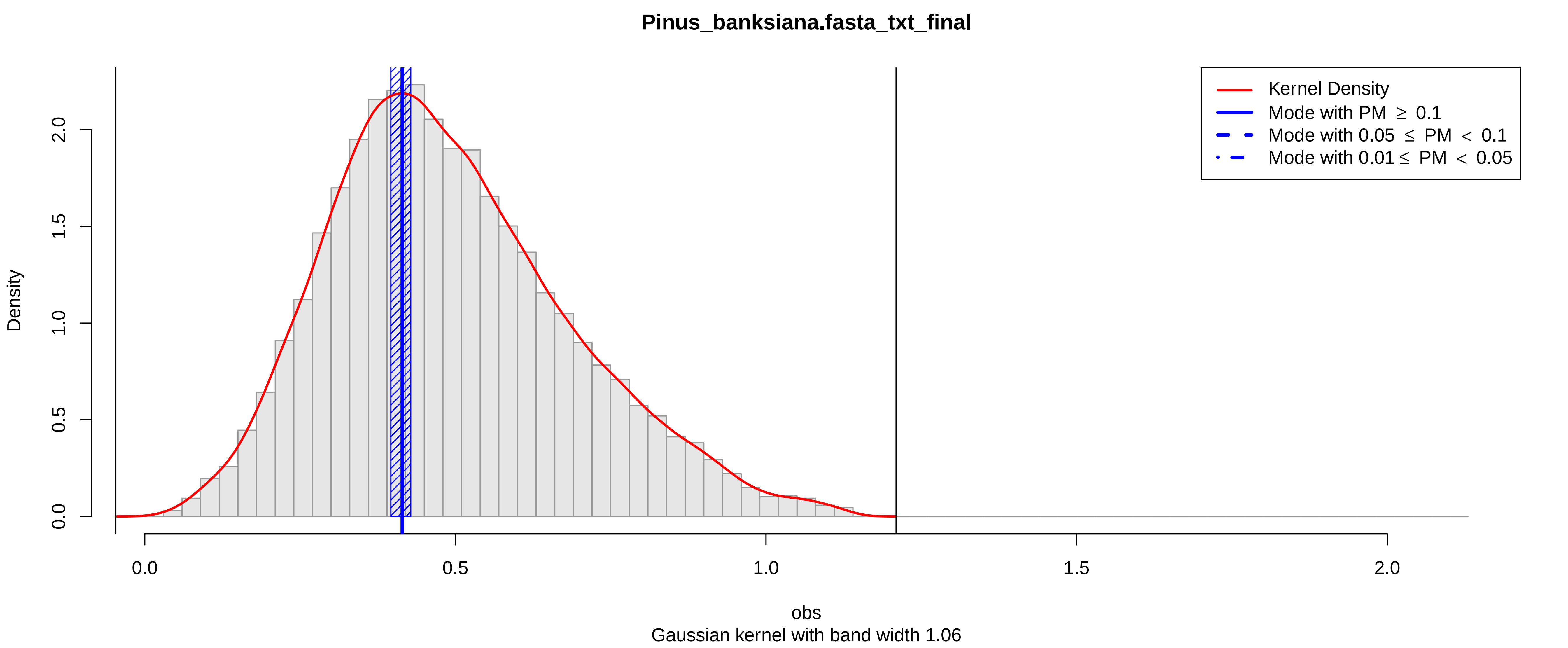

Pinus_banksiana.fasta_txt_final
Kernel Density
Mode with PM ≥ 0.1
Mode with 0.05 ≤ PM < 0.1
Mode with 0.01 ≤ PM < 0.05
Density
0.0
0.5
1.0
1.5
2.0
obs
Gaussian kernel with band width 1.06

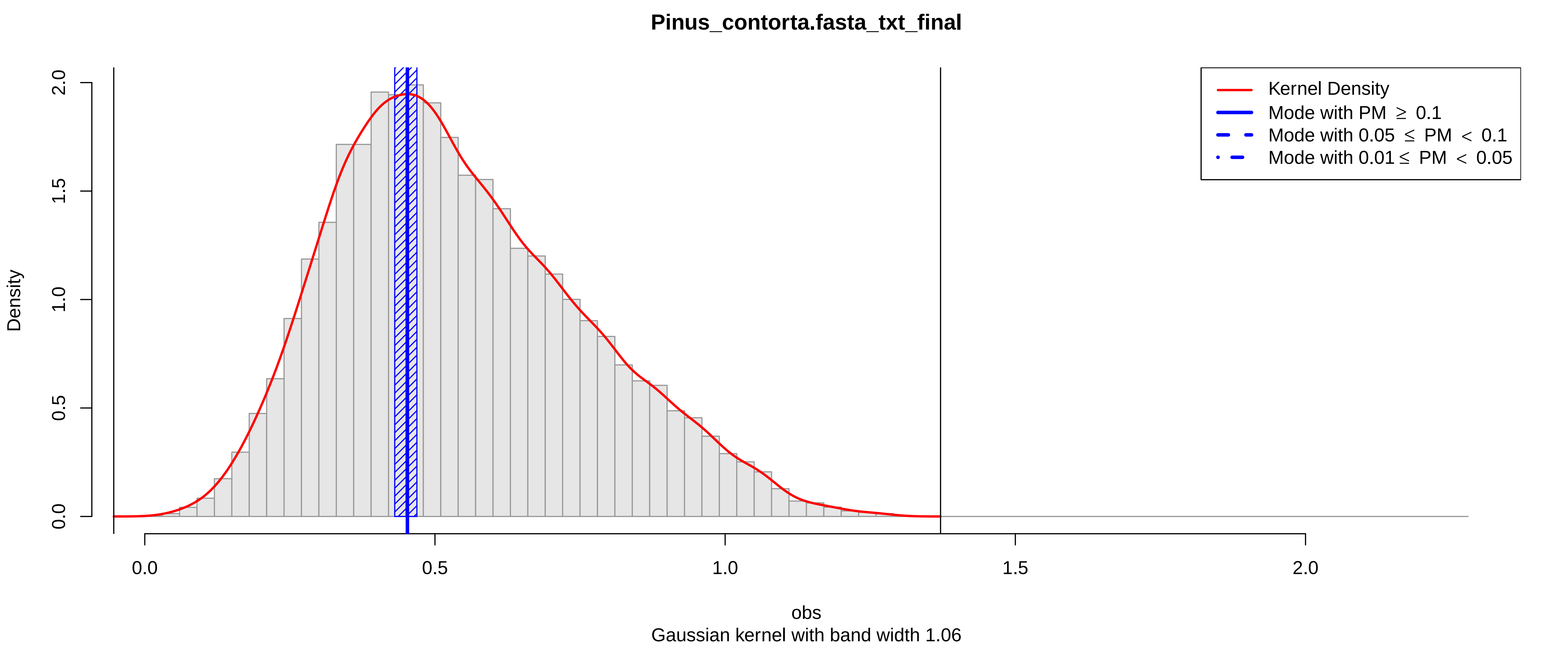

Pinus_contorta.fasta_txt_final
Kernel Density
Mode with PM ≥ 0.1
Mode with 0.05 ≤ PM < 0.1
Mode with 0.01 ≤ PM < 0.05
Density
0.0
0.5
1.0
1.5
2.0
0.0
0.5
1.0
1.5
2.0
obs
Gaussian kernel with band width 1.06

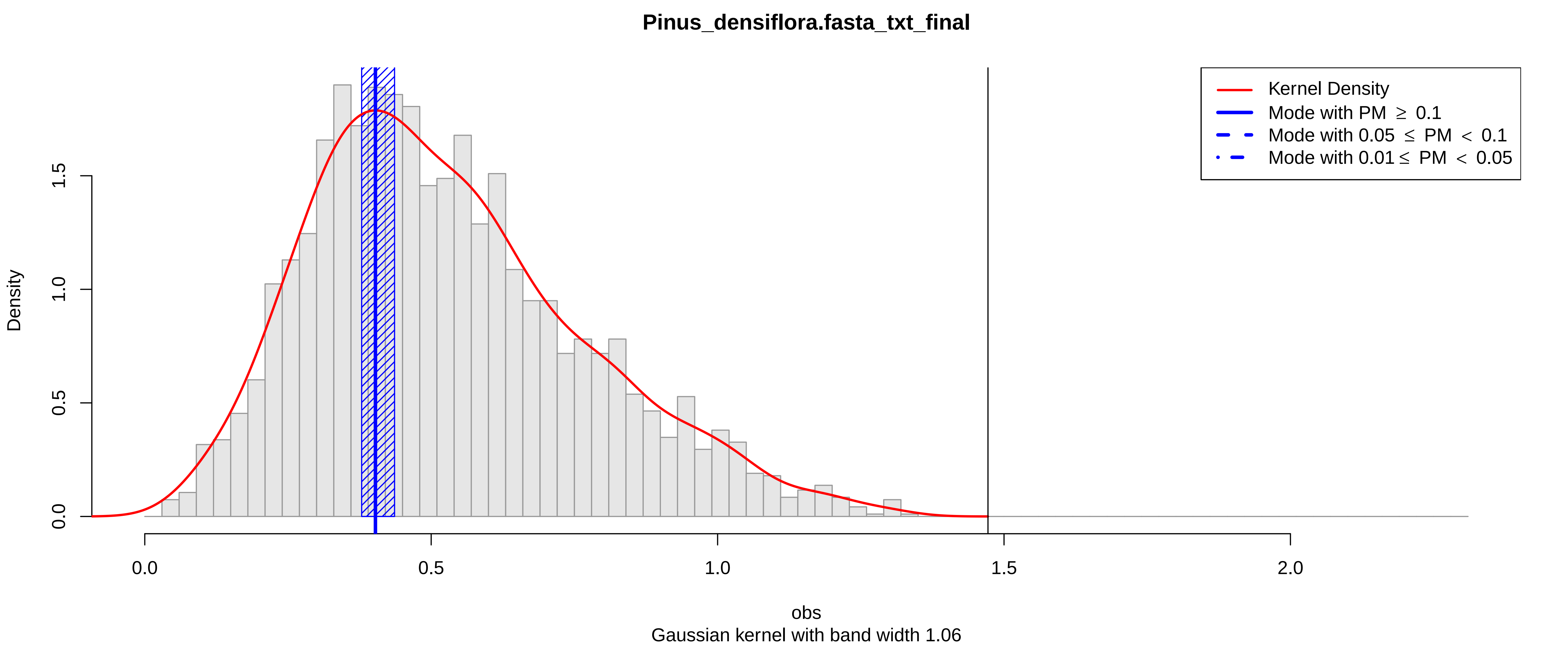
Pinus_densiflora.fasta_txt_final
Kernel Density
Mode with PM ≥ 0.1
Mode with 0.05 ≤ PM < 0.1
Mode with 0.01 ≤ PM < 0.05
Density
0.0
0.5
1.0
1.5
0.0
0.5
1.0
1.5
2.0
obs
Gaussian kernel with band width 1.06

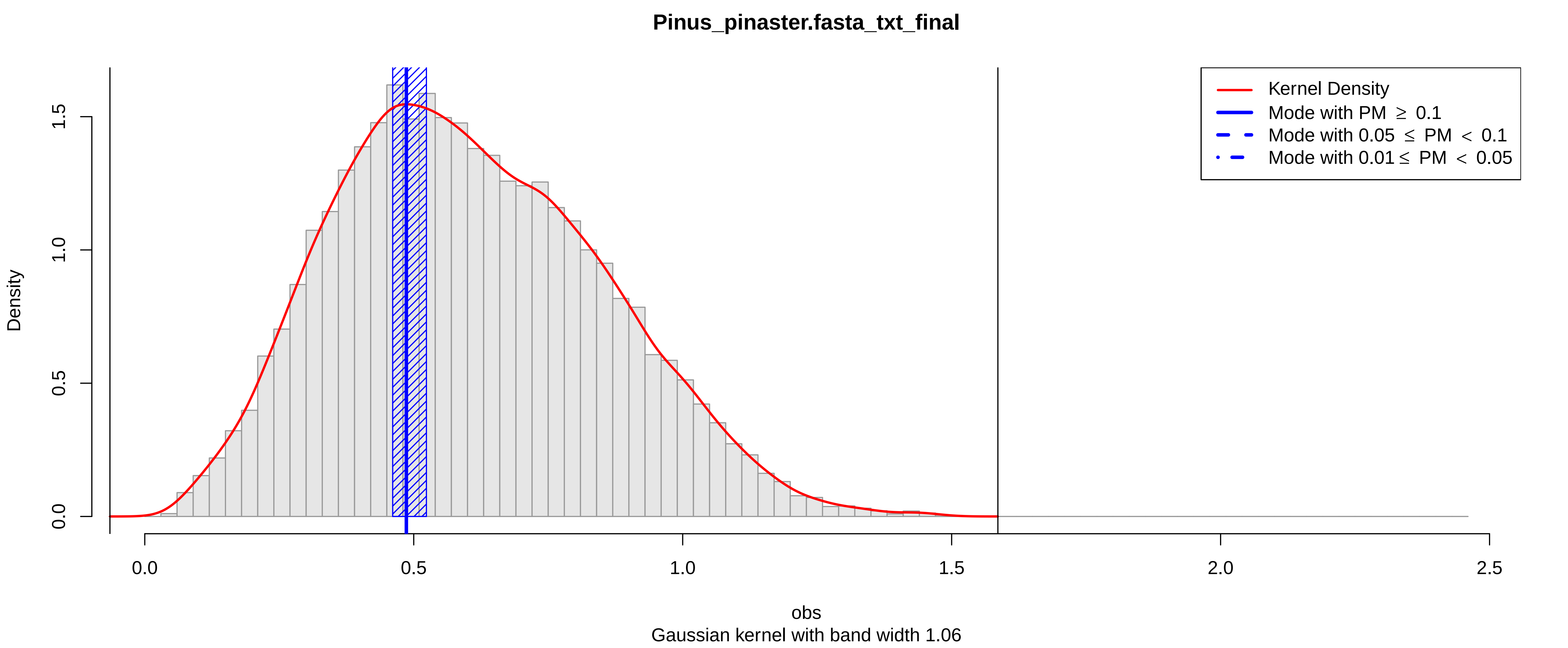
Pinus_pinaster.fasta_txt_final
Kernel Density
Mode with PM ≥ 0.1
Mode with 0.05 ≤ PM < 0.1
Mode with 0.01 ≤ PM < 0.05
Density
0.0
0.5
1.0
1.5
0.0
0.5
1.0
1.5
2.0
2.5
obs
Gaussian kernel with band width 1.06

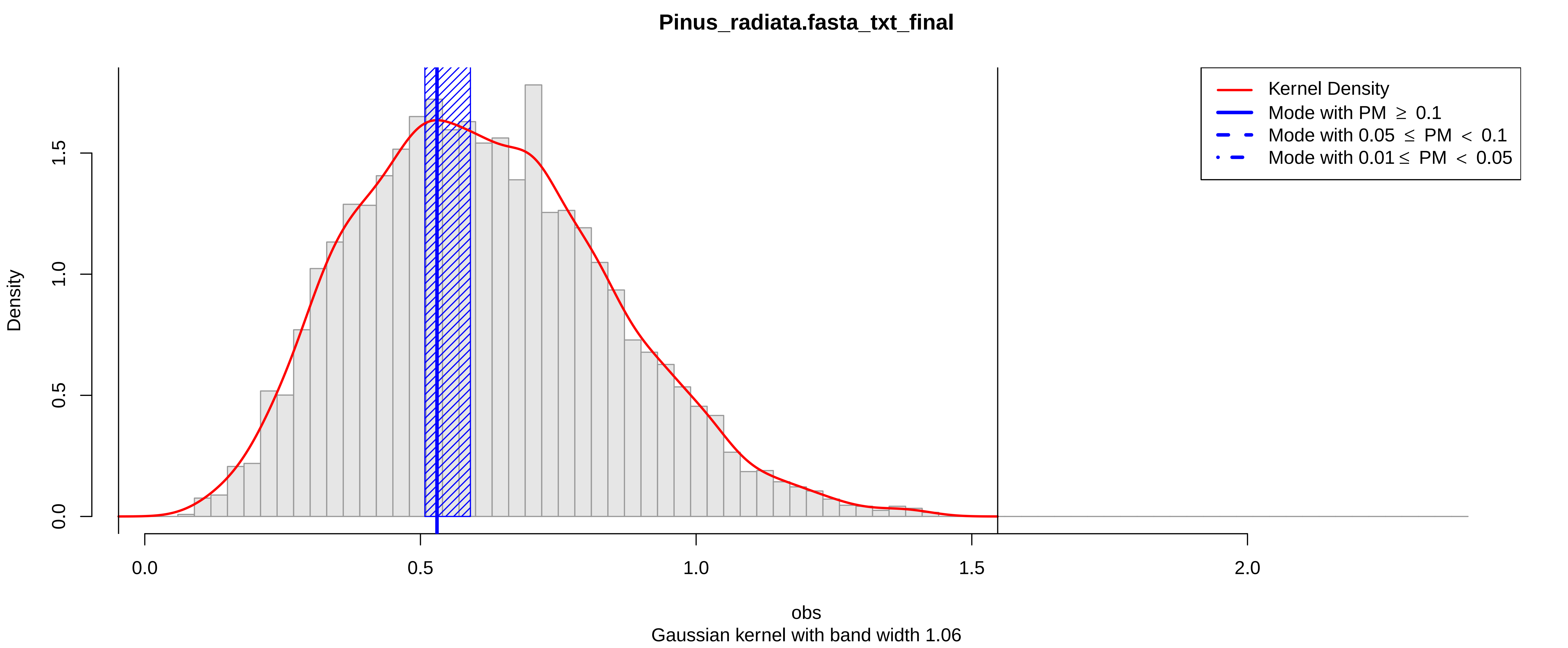

Pinus_radiata.fasta_txt_final
Kernel Density
Mode with PM ≥ 0.1
Mode with 0.05 ≤ PM < 0.1
Mode with 0.01 ≤ PM < 0.05
Density
0.0
0.5
1.0
1.5
2.0
obs
Gaussian kernel with band width 1.06

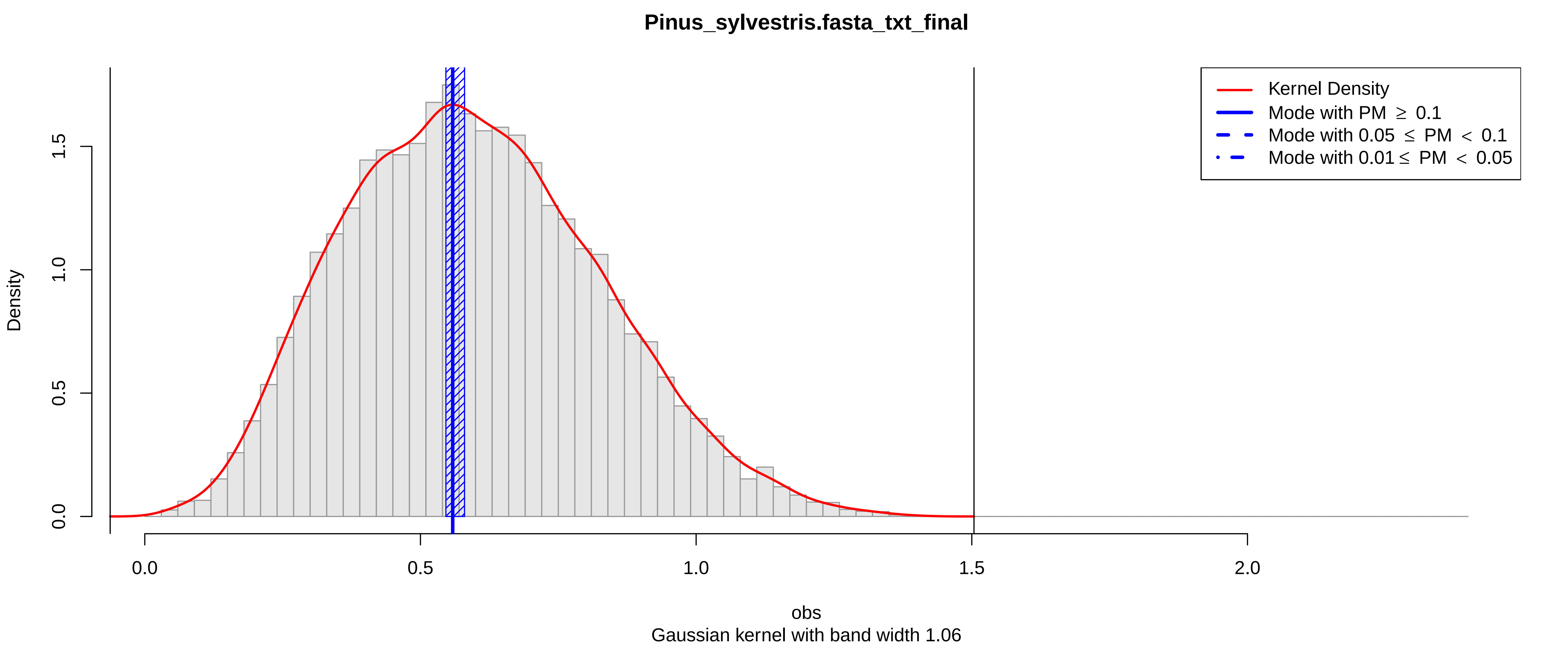

Pinus_sylvestris.fasta_txt_final
Kernel Density
Mode with PM ≥ 0.1
Mode with 0.05 ≤ PM < 0.1
Mode with 0.01 ≤ PM < 0.05
Density
0.0
0.5
1.0
1.5
2.0
obs
Gaussian kernel with band width 1.06

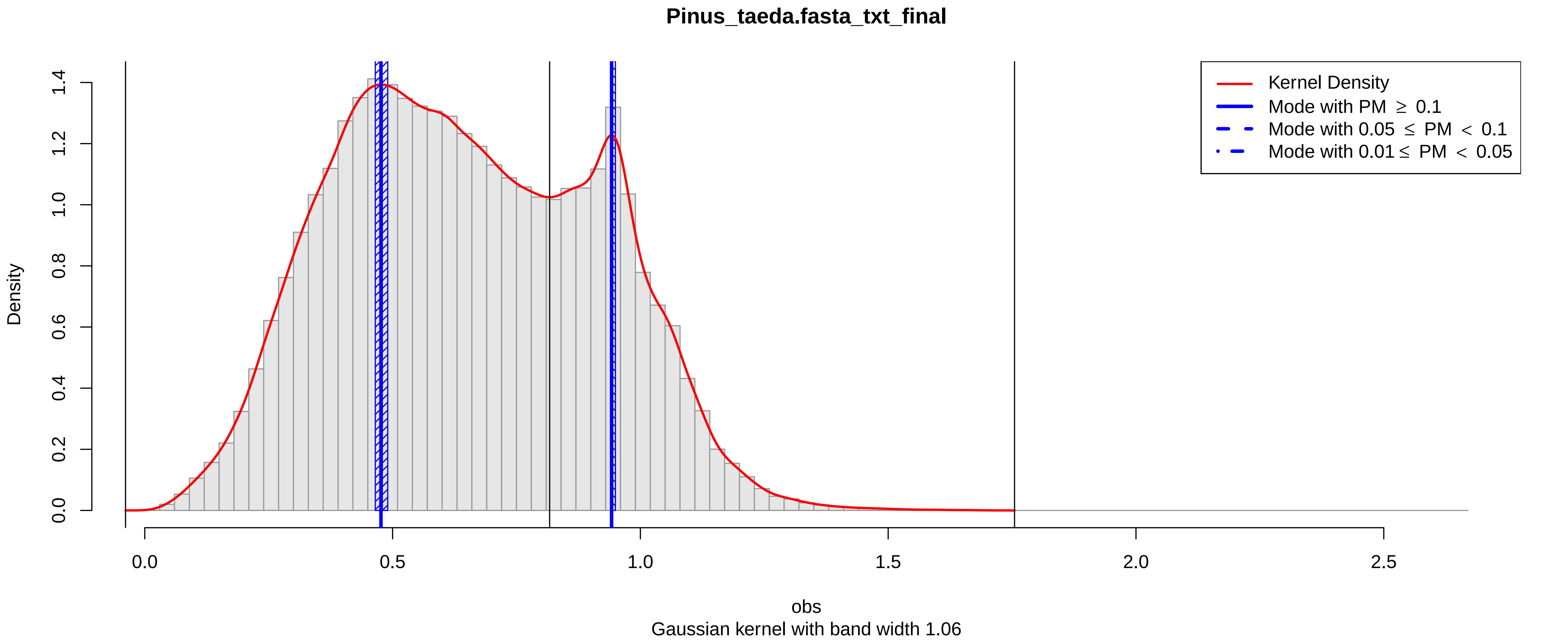

Pinus_taeda.fasta_txt_final
Kernel Density
Mode with PM ≥ 0.1
Mode with 0.05 ≤ PM < 0.1
Mode with 0.01 ≤ PM < 0.05
Density
0.0
0.2
0.4
0.6
0.8
1.0
1.2
1.4
0.0
0.5
1.0
1.5
2.0
2.5
obs
Gaussian kernel with band width 1.06

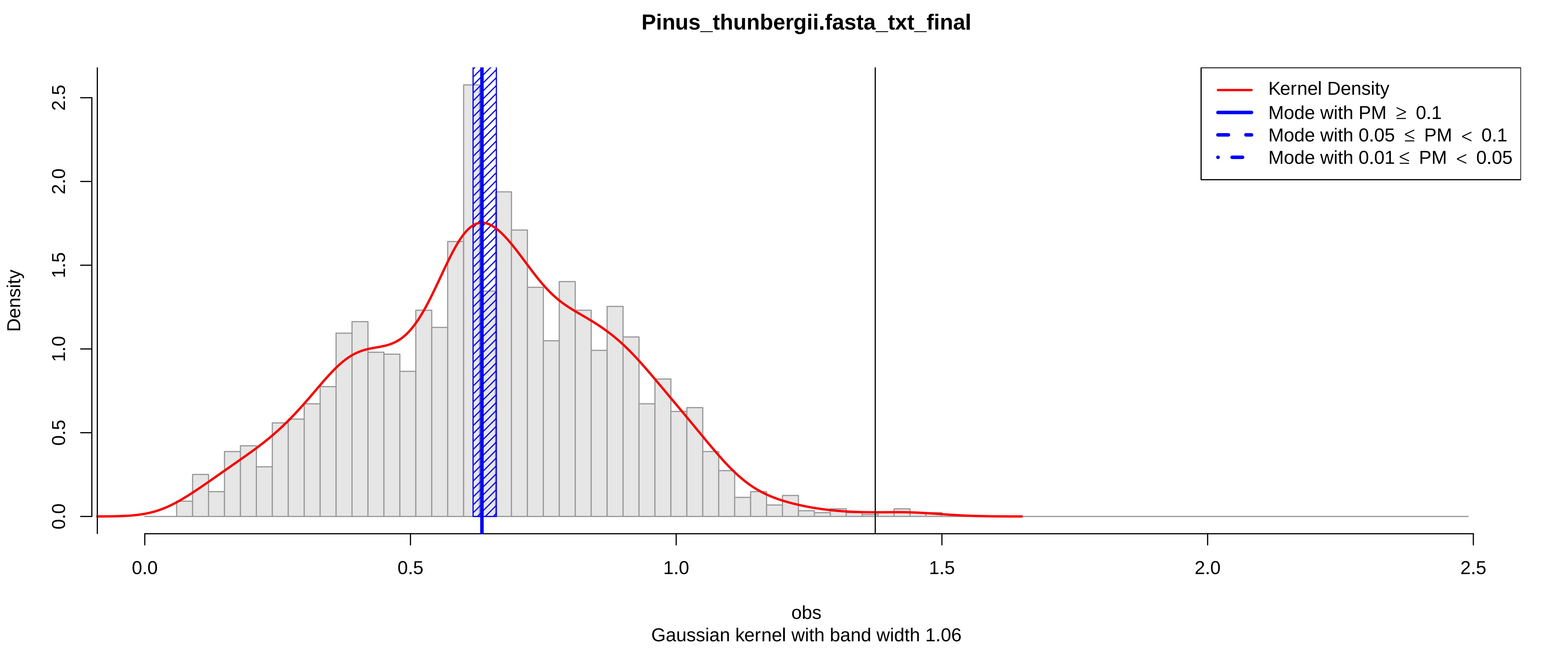

Pinus_thunbergii.fasta_txt_final
Kernel Density
Mode with PM ≥ 0.1
Mode with 0.05 ≤ PM < 0.1
Mode with 0.01 ≤ PM < 0.05
Density
0.0
0.5
1.0
1.5
2.0
2.5
obs
Gaussian kernel with band width 1.06

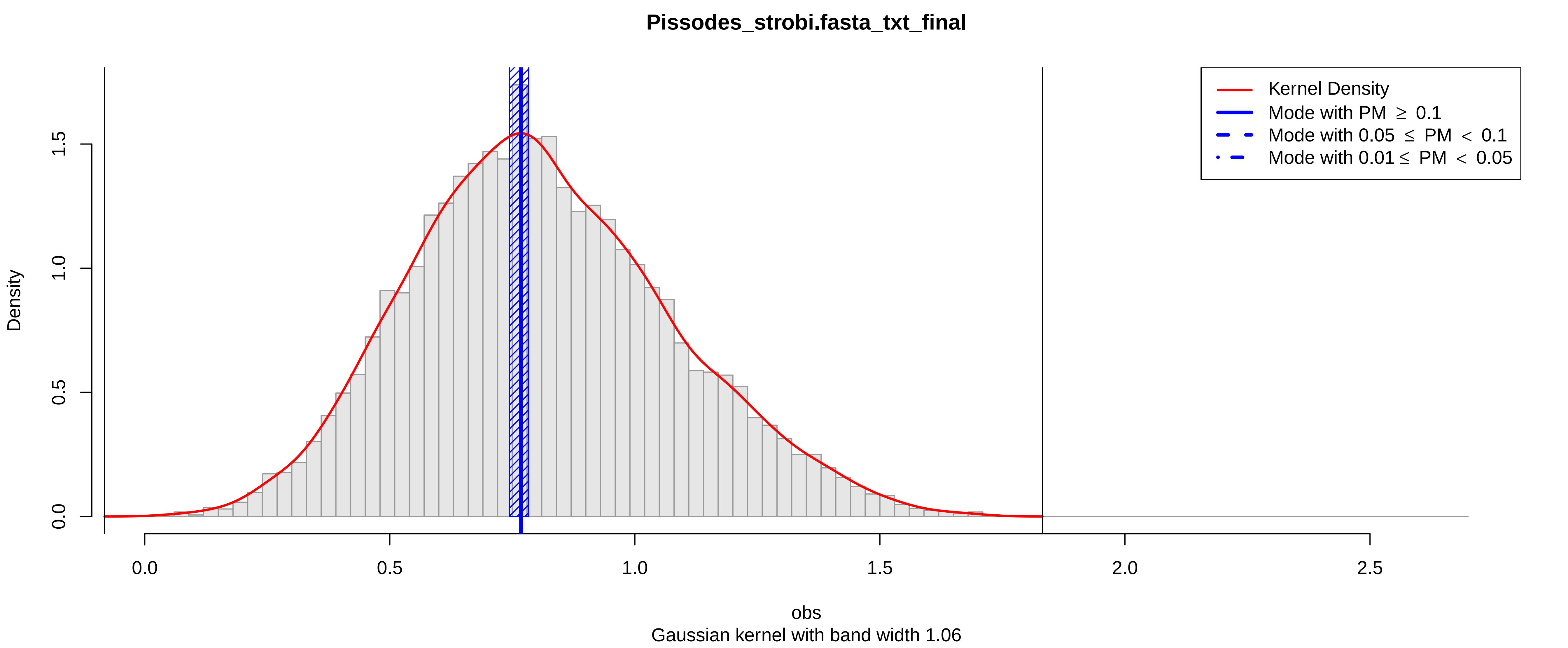

Pissodes_strobi.fasta_txt_final
Kernel Density
Mode with PM ≥ 0.1
Mode with 0.05 ≤ PM < 0.1
Mode with 0.01 ≤ PM < 0.05
Density
0.0
0.5
1.0
1.5
0.0
0.5
1.0
1.5
2.0
2.5
obs
Gaussian kernel with band width 1.06

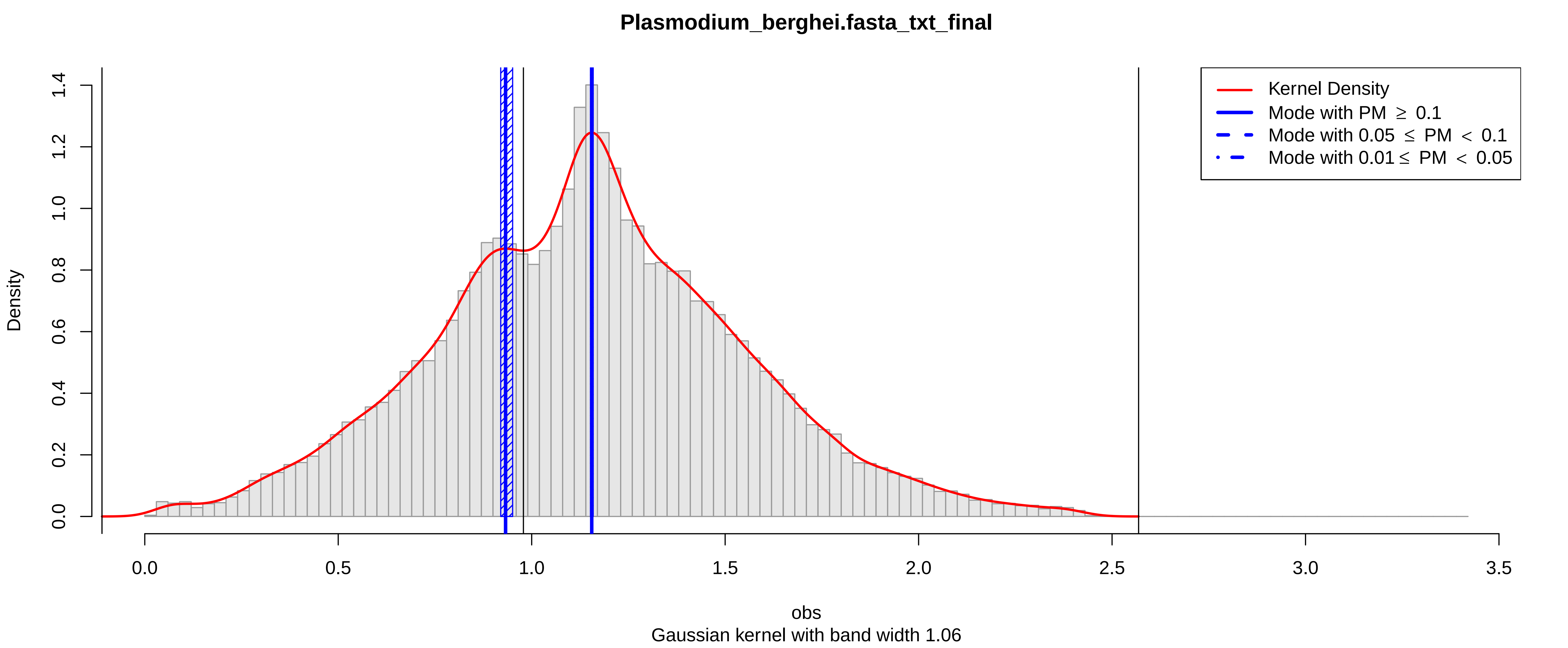

Plasmodium_berghei.fasta_txt_final
Kernel Density
Mode with PM ≥ 0.1
Mode with 0.05 ≤ PM < 0.1
Mode with 0.01 ≤ PM < 0.05
Density
0.0
0.2
0.4
0.6
0.8
1.0
1.2
1.4
0.0
0.5
1.0
1.5
2.0
2.5
3.0
3.5
obs
Gaussian kernel with band width 1.06

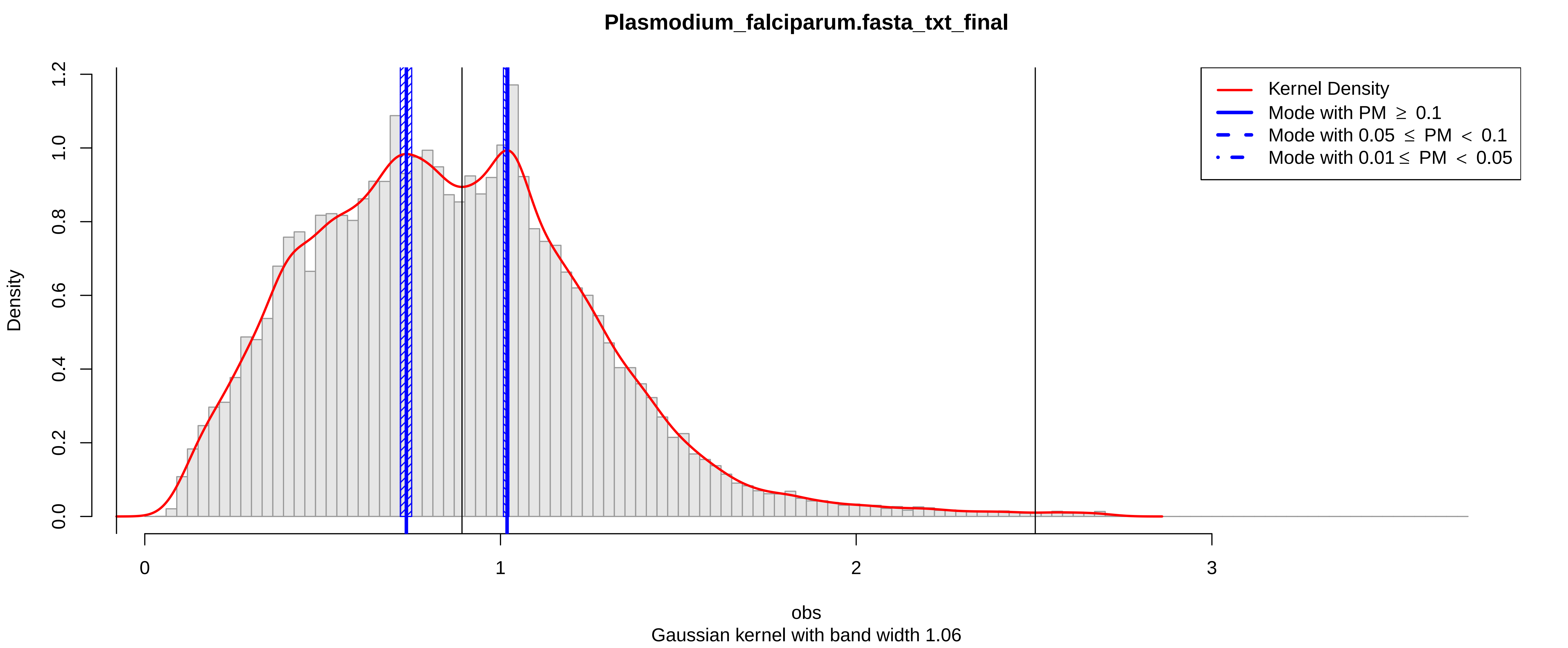

Plasmodium_falciparum.fasta_txt_final
Density
0.0
0.2
0.4
0.6
0.8
1.0
1.2
0
1
2
3
obs
Gaussian kernel with band width 1.06
Kernel Density
Mode with PM ≥ 0.1
Mode with 0.05 ≤ PM < 0.1
Mode with 0.01 ≤ PM < 0.05

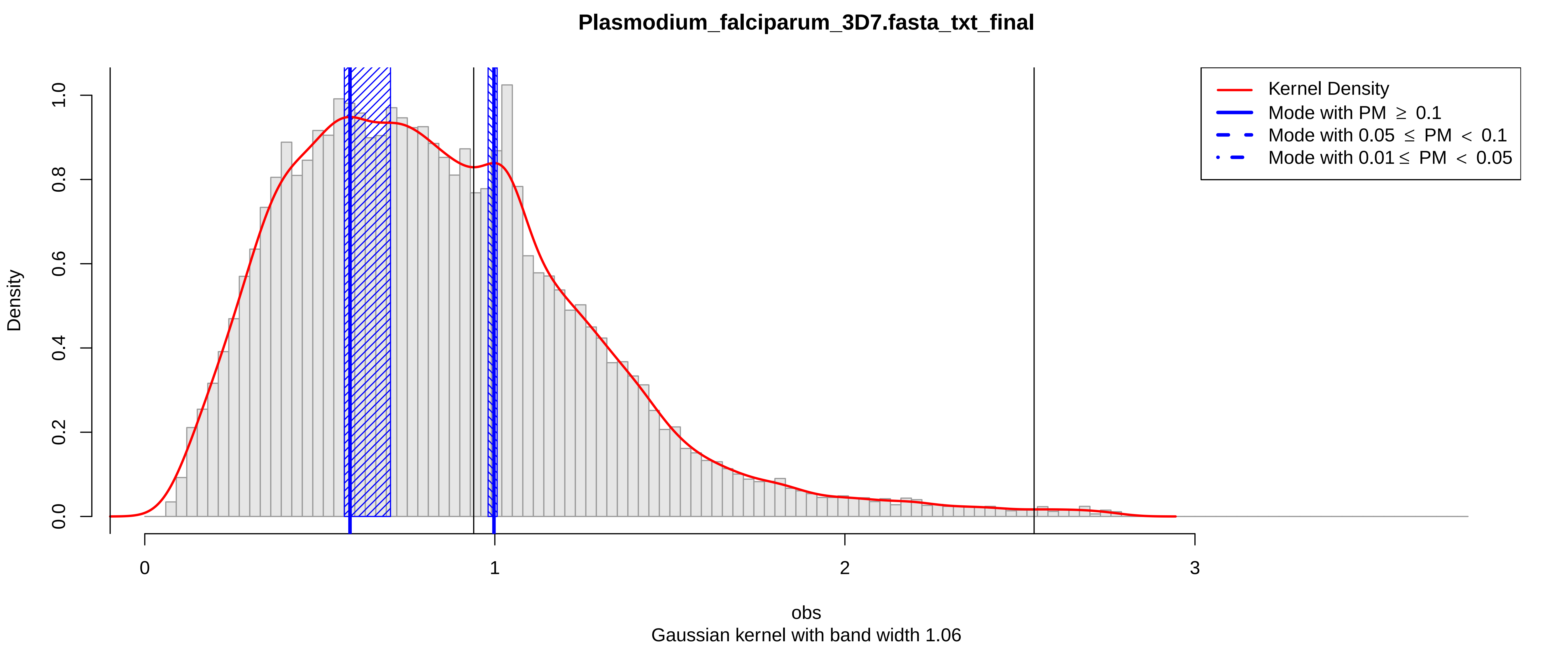

Plasmodium_falciparum_3D7.fasta_txt_final
Kernel Density
Mode with PM ≥ 0.1
Mode with 0.05 ≤ PM < 0.1
Mode with 0.01 ≤ PM < 0.05
Density
0.0
0.2
0.4
0.6
0.8
1.0
0
1
2
3
obs
Gaussian kernel with band width 1.06

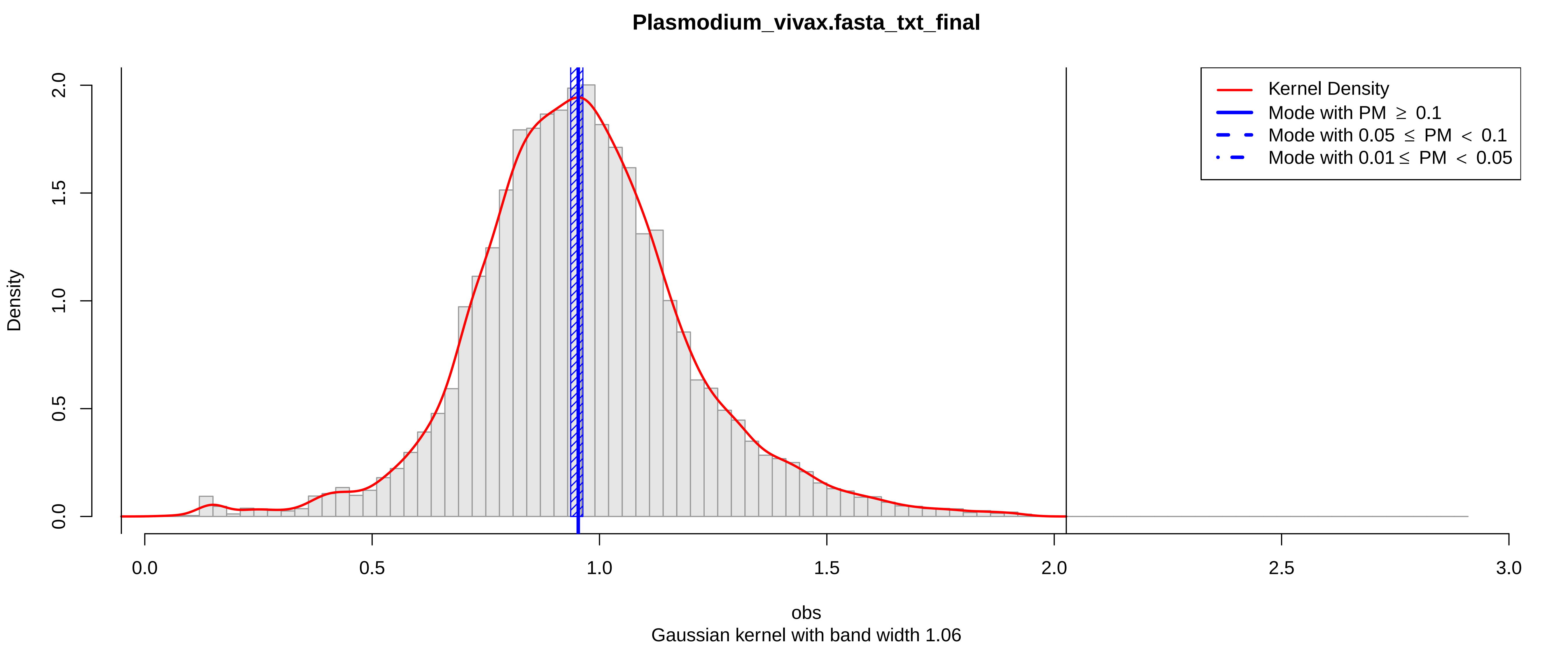
Plasmodium_vivax.fasta_txt_final
Kernel Density
Mode with PM ≥ 0.1
Mode with 0.05 ≤ PM < 0.1
Mode with 0.01 ≤ PM < 0.05
Density
0.0
0.5
1.0
1.5
2.0
0.0
0.5
1.0
1.5
2.0
2.5
3.0
obs
Gaussian kernel with band width 1.06

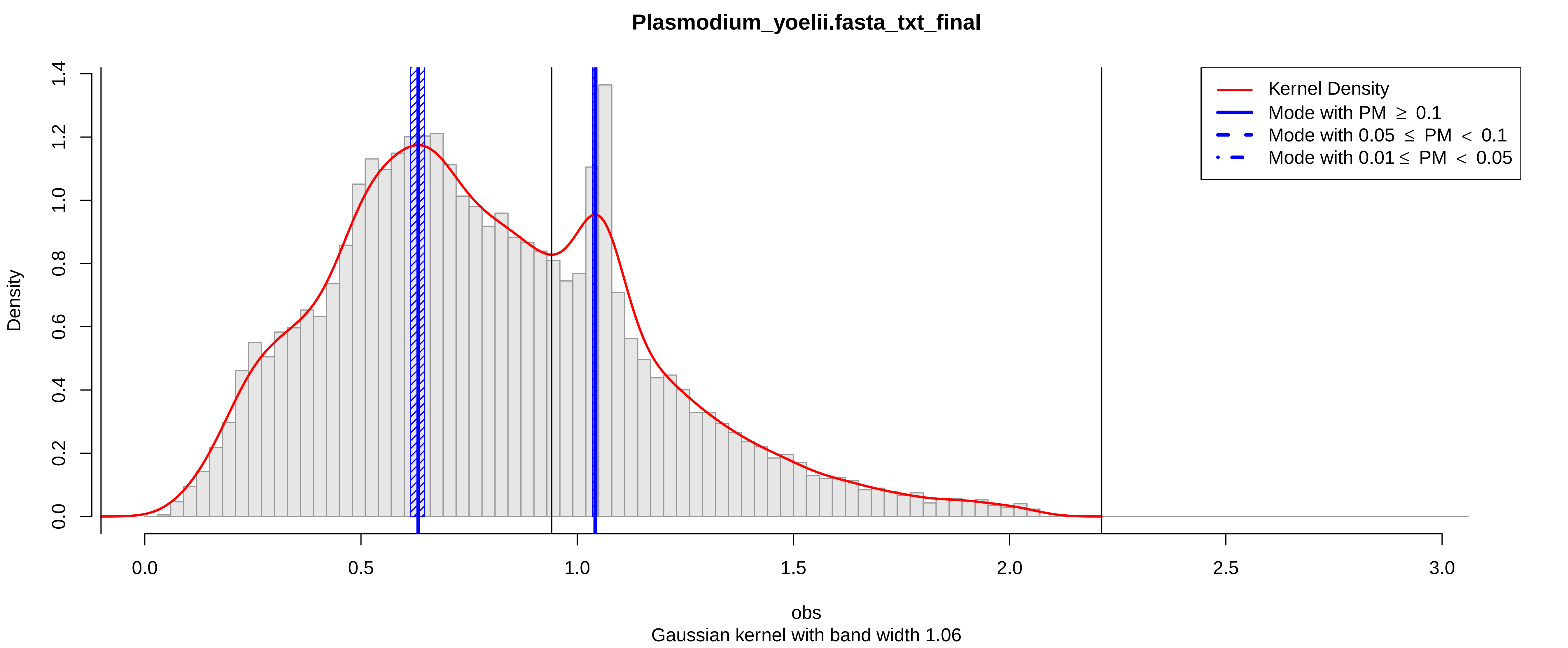

Plasmodium_yoelii.fasta_txt_final
Kernel Density
Mode with PM ≥ 0.1
Mode with 0.05 ≤ PM < 0.1
Mode with 0.01 ≤ PM < 0.05
Density
obs
Gaussian kernel with band width 1.06

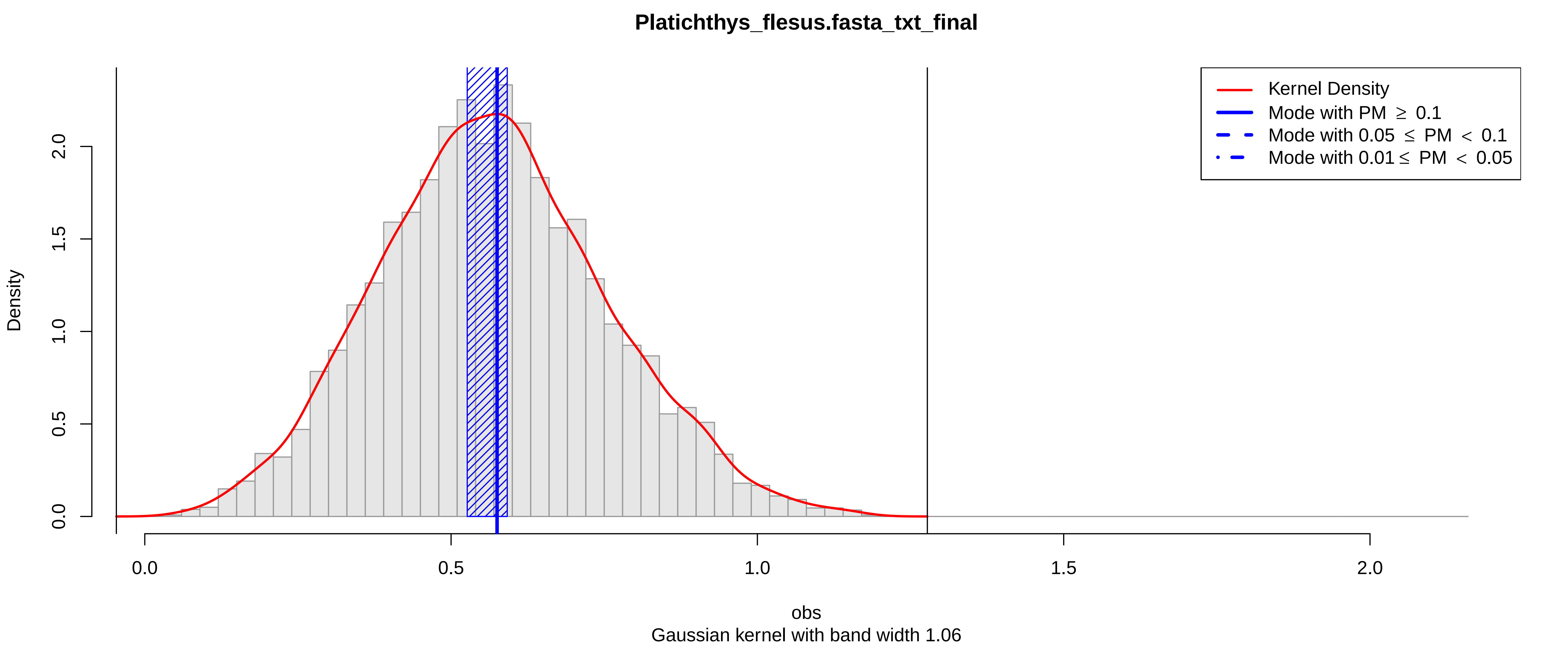

Platichthys_flesus.fasta_txt_final
Kernel Density
Mode with PM ≥ 0.1
Mode with 0.05 ≤ PM < 0.1
Mode with 0.01 ≤ PM < 0.05
Density
0.0
0.5
1.0
1.5
2.0
0.0
0.5
1.0
1.5
2.0
obs
Gaussian kernel with band width 1.06

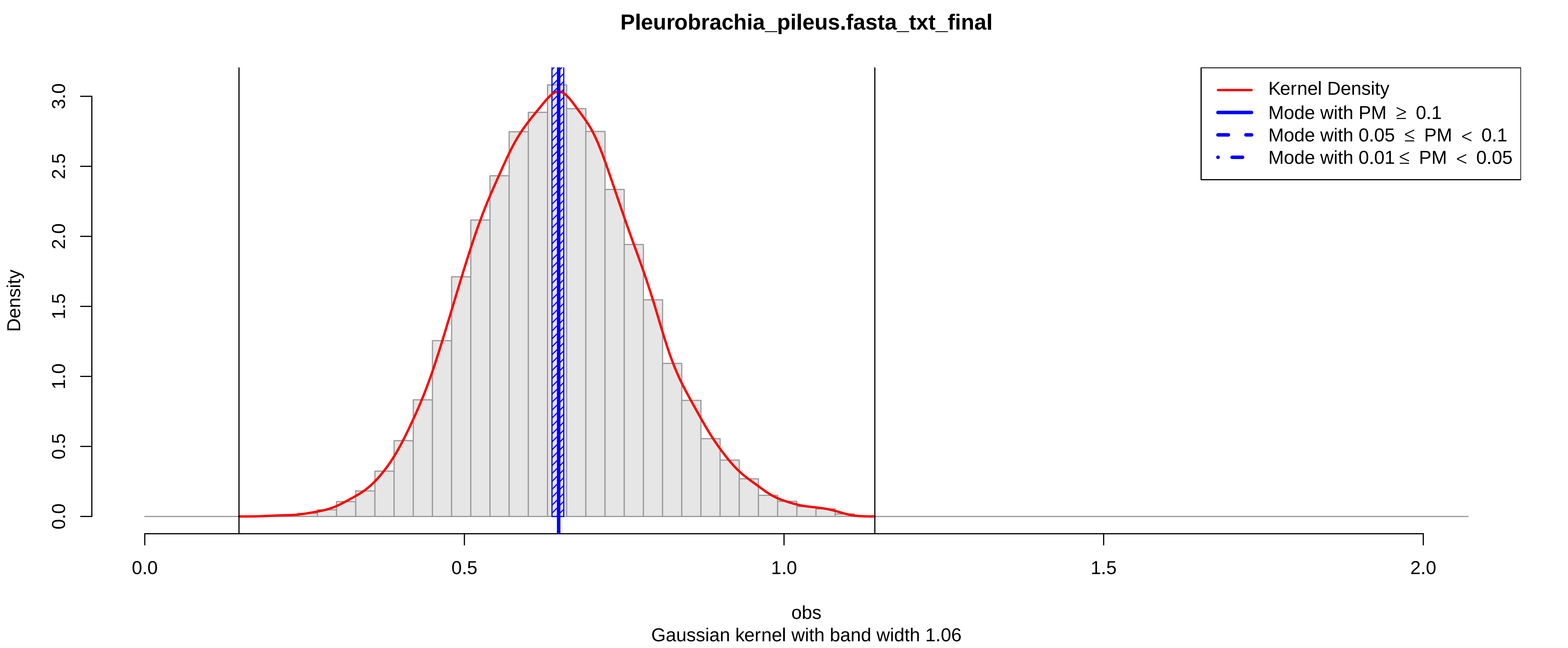
Pleurobrachia_pileus.fasta_txt_final
Kernel Density
Mode with PM ≥ 0.1
Mode with 0.05 ≤ PM < 0.1
Mode with 0.01 ≤ PM < 0.05
Density
obs
Gaussian kernel with band width 1.06
0.0
0.5
1.0
1.5
2.0
2.5
3.0

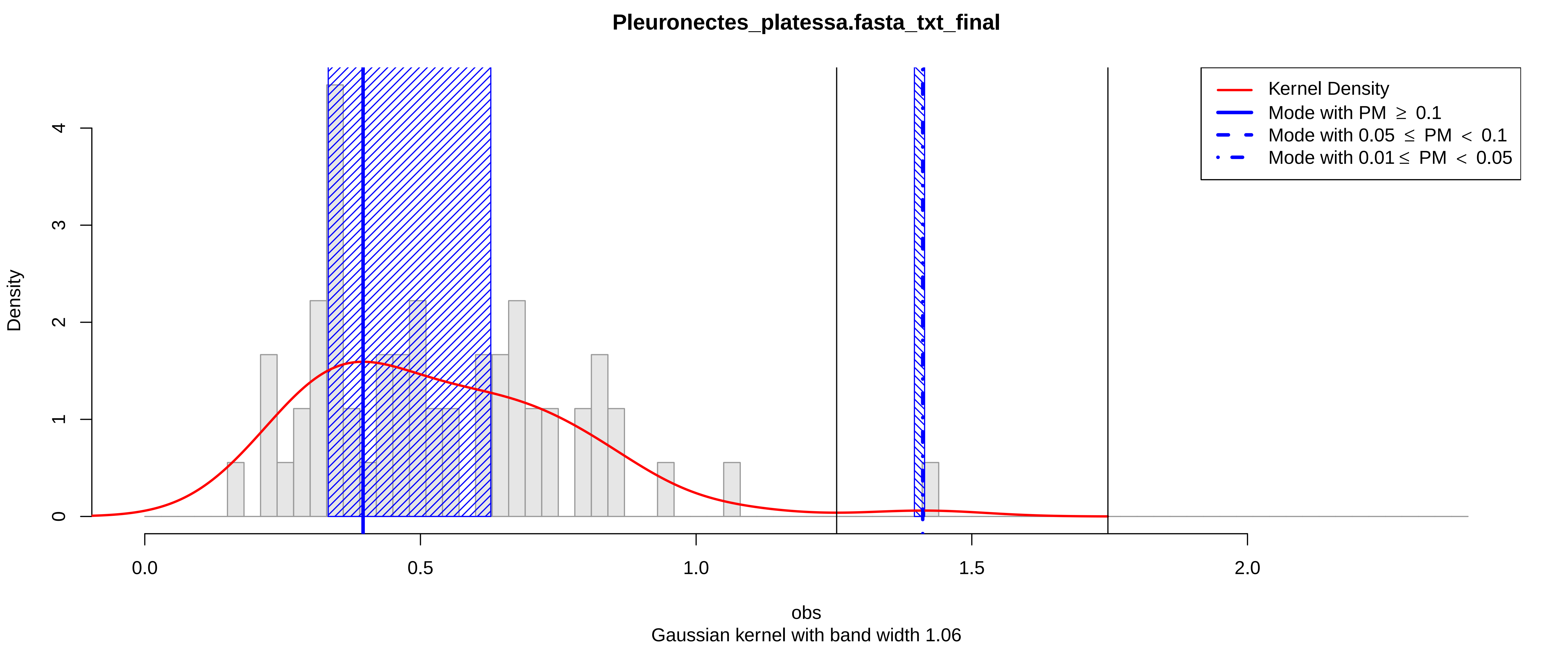

Pleuronectes_platessa.fasta_txt_final
Kernel Density
Mode with PM ≥ 0.1
Mode with 0.05 ≤ PM < 0.1
Mode with 0.01 ≤ PM < 0.05
Density
0
1
2
3
4
0.0
0.5
1.0
1.5
2.0
obs
Gaussian kernel with band width 1.06

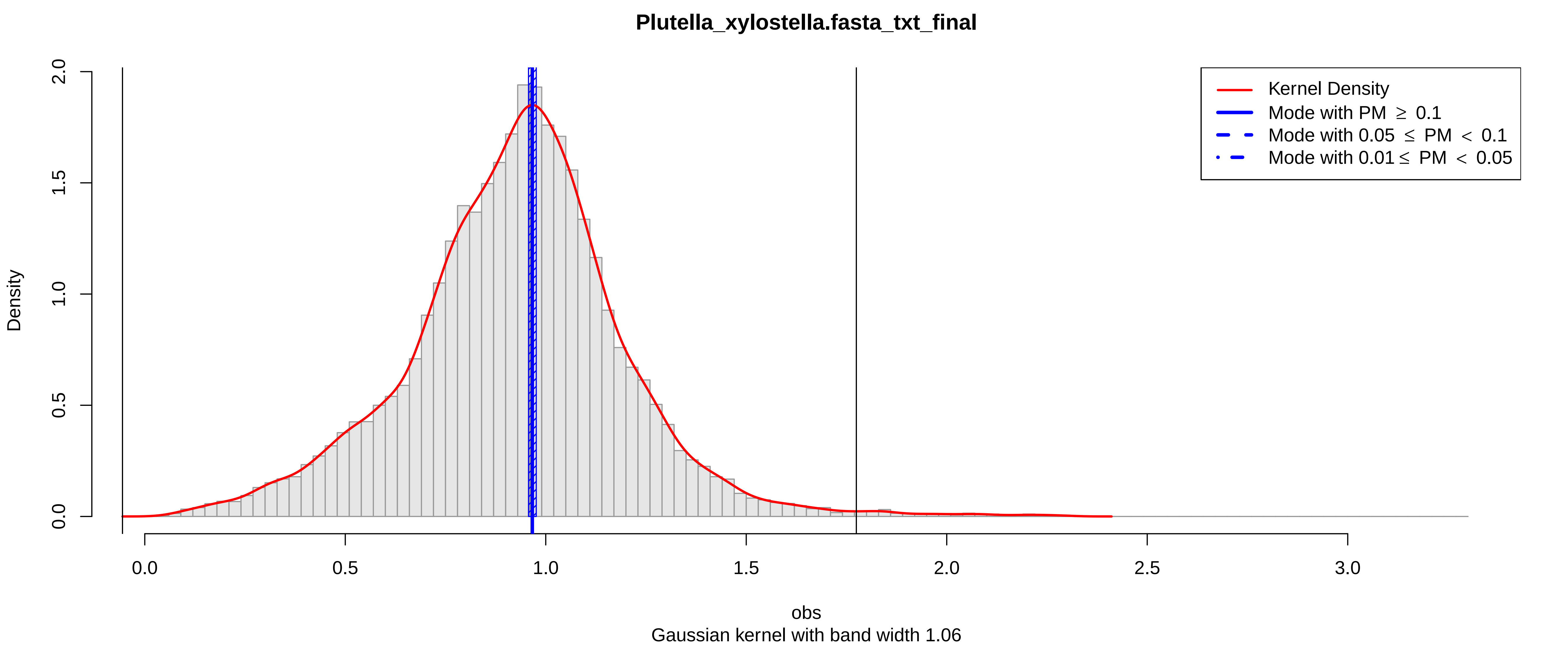
Plutella_xylostella.fasta_txt_final
Kernel Density
Mode with PM ≥ 0.1
Mode with 0.05 ≤ PM < 0.1
Mode with 0.01 ≤ PM < 0.05
Density
0.0
0.5
1.0
1.5
2.0
0.0
0.5
1.0
1.5
2.0
2.5
3.0
obs
Gaussian kernel with band width 1.06

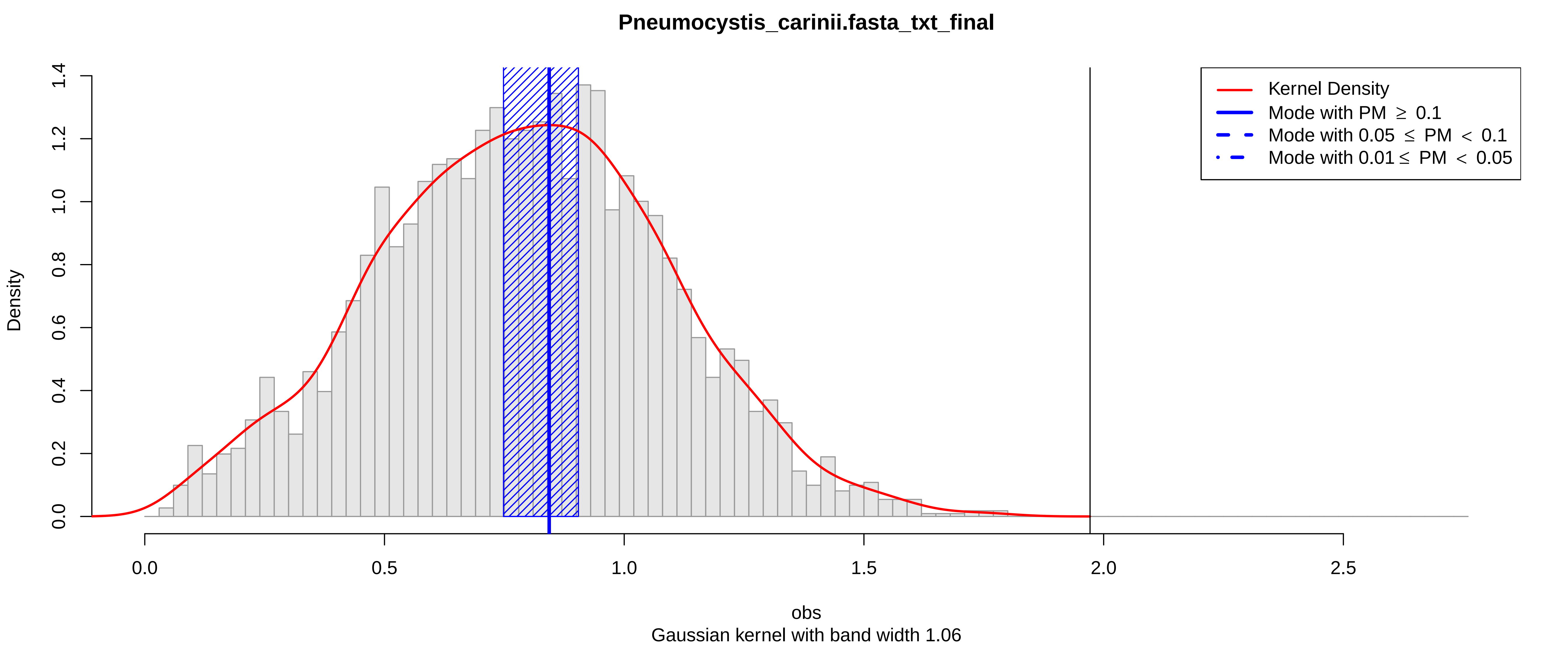
Pneumocystis_carinii.fasta_txt_final
Kernel Density
Mode with PM ≥ 0.1
Mode with 0.05 ≤ PM < 0.1
Mode with 0.01 ≤ PM < 0.05
Density
0.0
0.2
0.4
0.6
0.8
1.0
1.2
1.4
0.0
0.5
1.0
1.5
2.0
2.5
obs
Gaussian kernel with band width 1.06

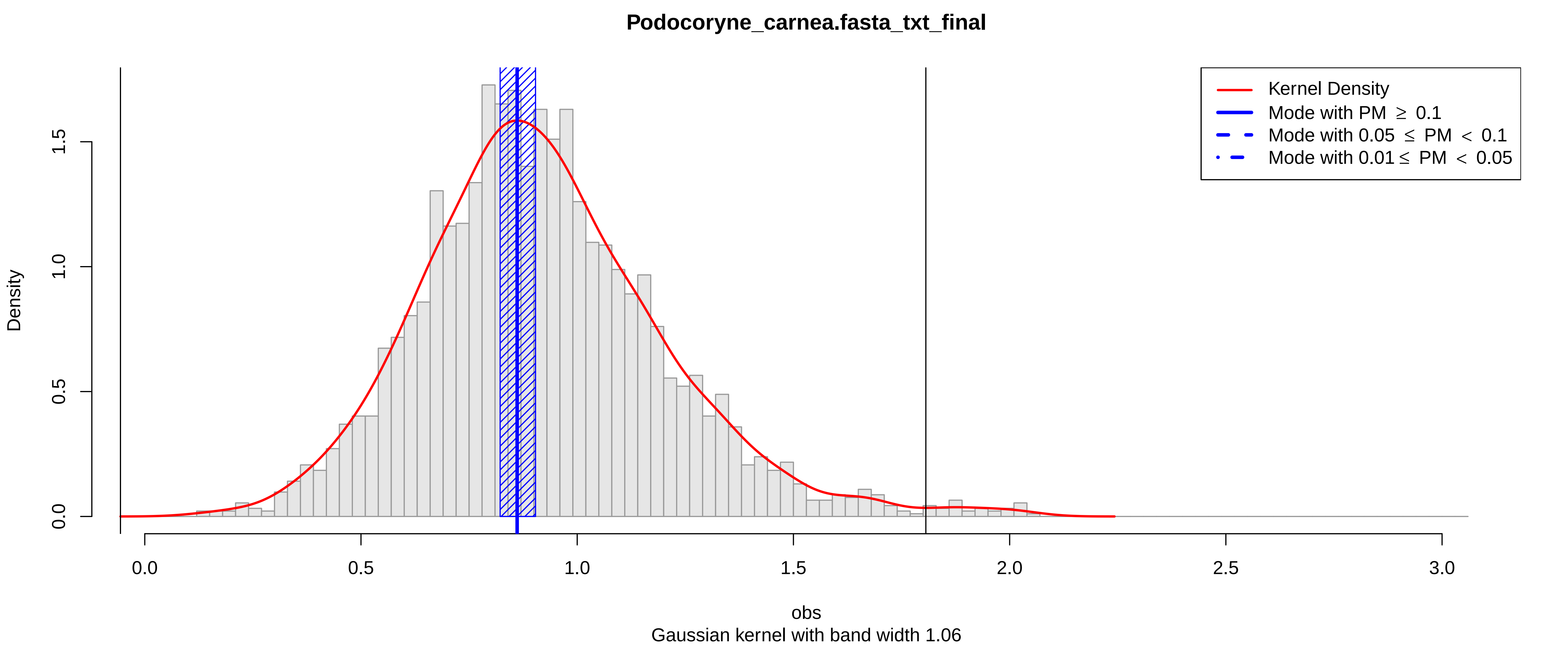

Podocoryne_carnea.fasta_txt_final
Kernel Density
Mode with PM ≥ 0.1
Mode with 0.05 ≤ PM < 0.1
Mode with 0.01 ≤ PM < 0.05
Density
0.0
0.5
1.0
1.5
0.0
0.5
1.0
1.5
2.0
2.5
3.0
obs
Gaussian kernel with band width 1.06

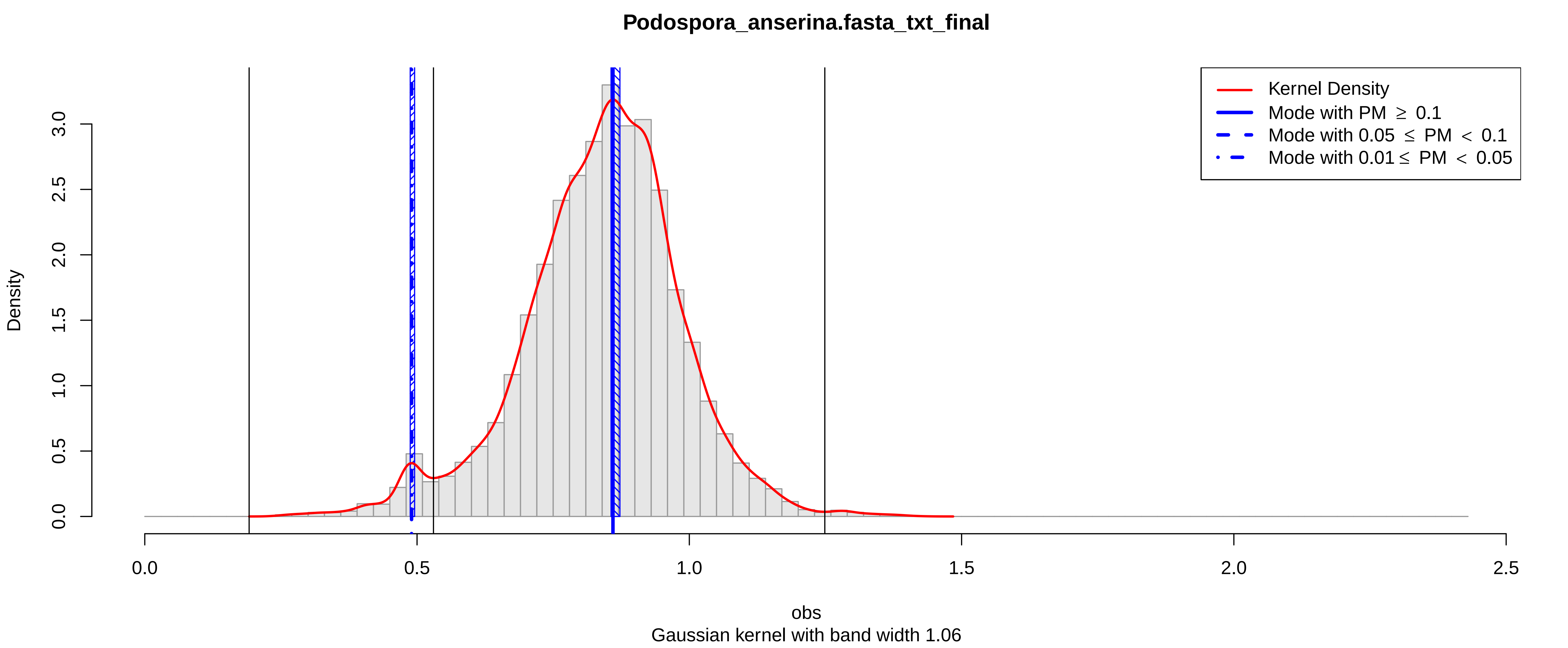
Podospora_anserina.fasta_txt_final
Kernel Density
Mode with PM ≥ 0.1
Mode with 0.05 ≤ PM < 0.1
Mode with 0.01 ≤ PM < 0.05
Density
0.0
0.5
1.0
1.5
2.0
2.5
3.0
obs
Gaussian kernel with band width 1.06

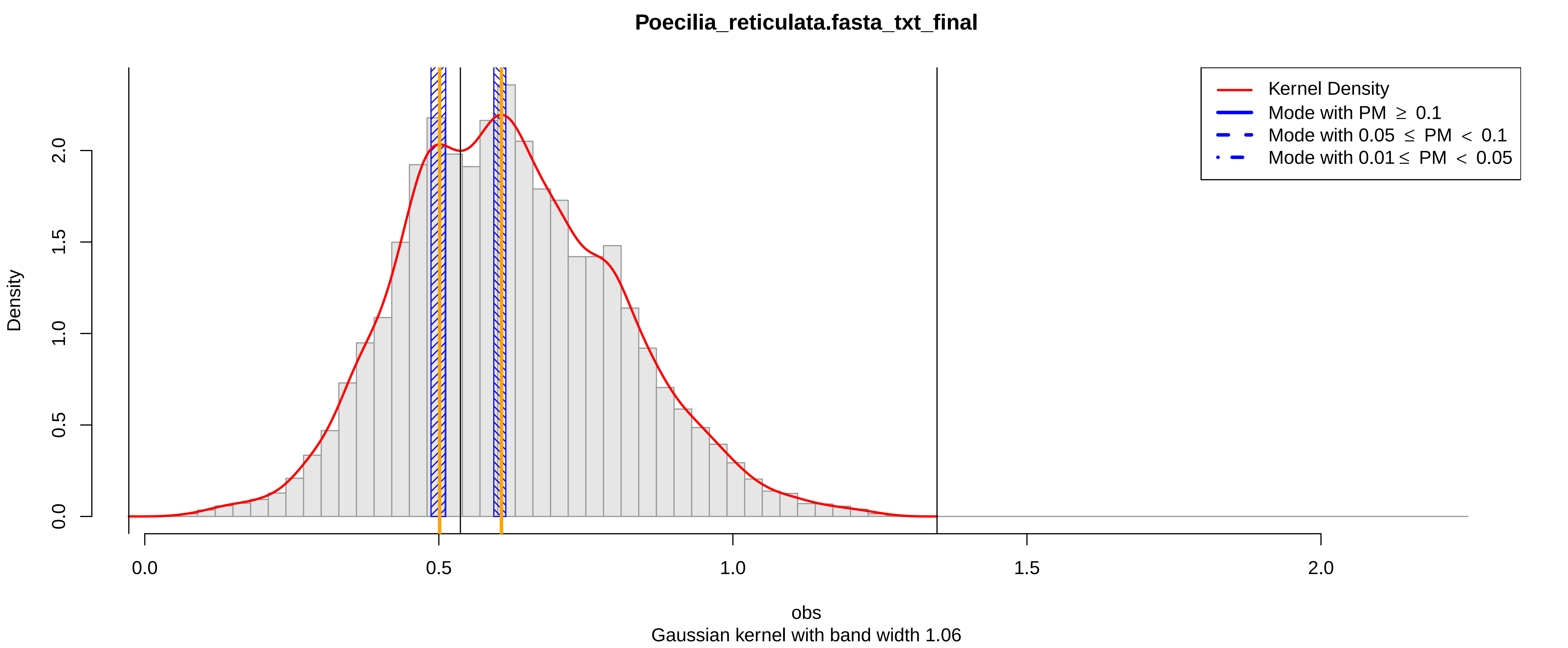
Poecilia_reticulata.fasta_txt_final
Kernel Density
Mode with PM ≥ 0.1
Mode with 0.05 ≤ PM < 0.1
Mode with 0.01 ≤ PM < 0.05
Density
0.0
0.5
1.0
1.5
2.0
0.0
0.5
1.0
1.5
2.0
obs
Gaussian kernel with band width 1.06

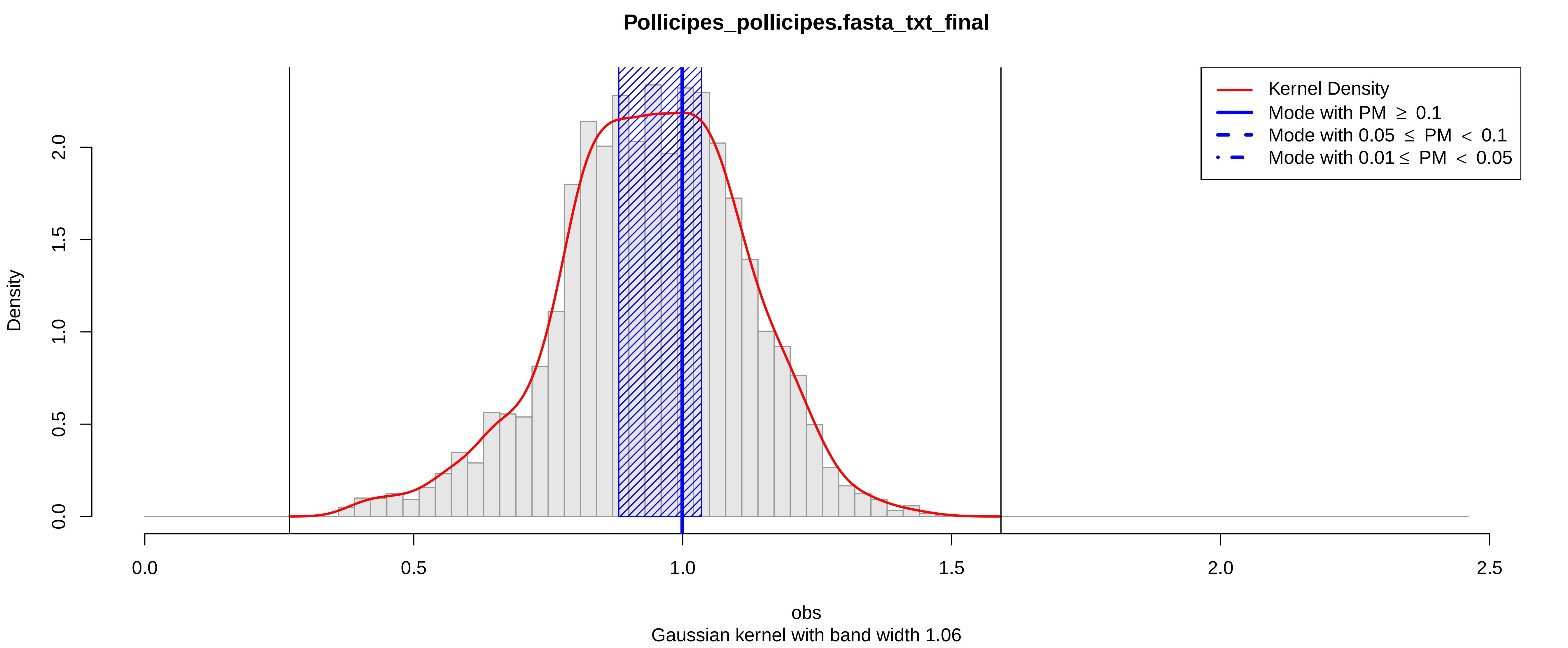

Pollicipes_pollicipes.fasta_txt_final
Kernel Density
Mode with PM ≥ 0.1
Mode with 0.05 ≤ PM < 0.1
Mode with 0.01 ≤ PM < 0.05
Density
0.0
0.5
1.0
1.5
2.0
2.5
obs
Gaussian kernel with band width 1.06

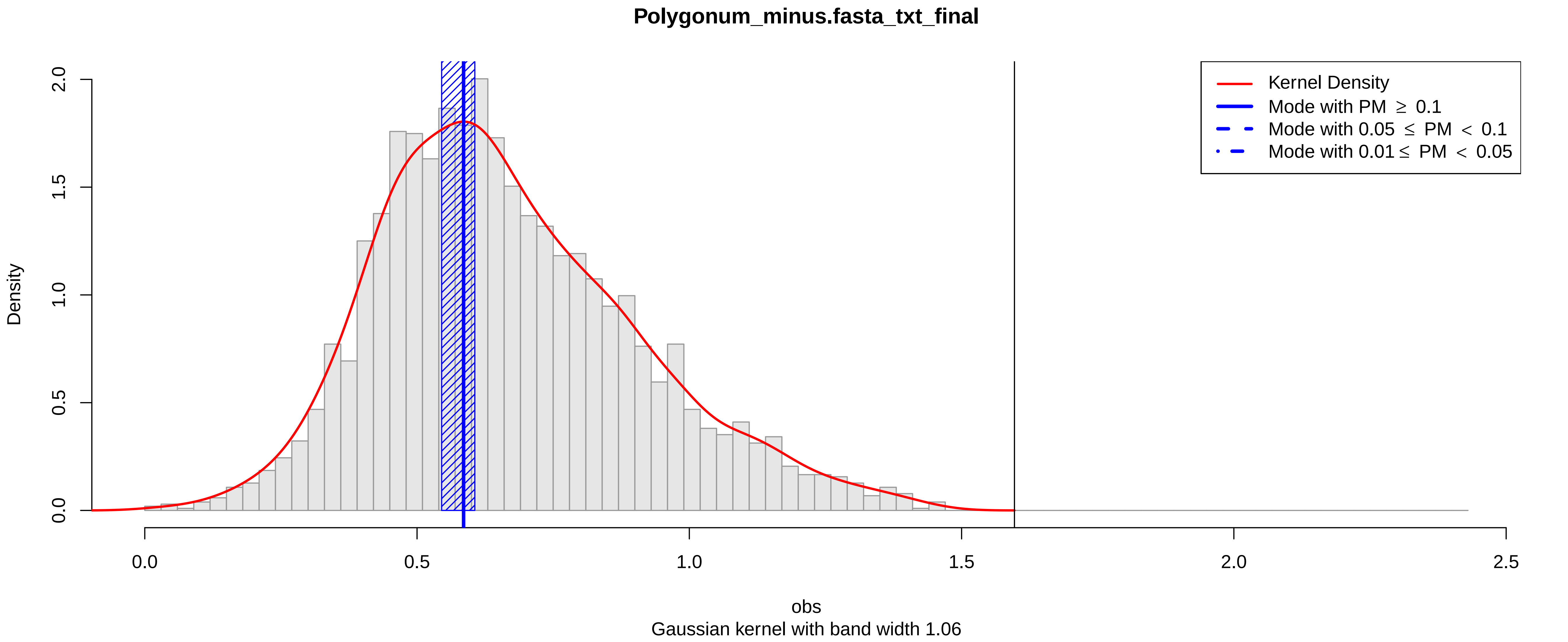

Polygonum_minus.fasta_txt_final
Kernel Density
Mode with PM ≥ 0.1
Mode with 0.05 ≤ PM < 0.1
Mode with 0.01 ≤ PM < 0.05
Density
0.0
0.5
1.0
1.5
2.0
0.0
0.5
1.0
1.5
2.0
2.5
obs
Gaussian kernel with band width 1.06

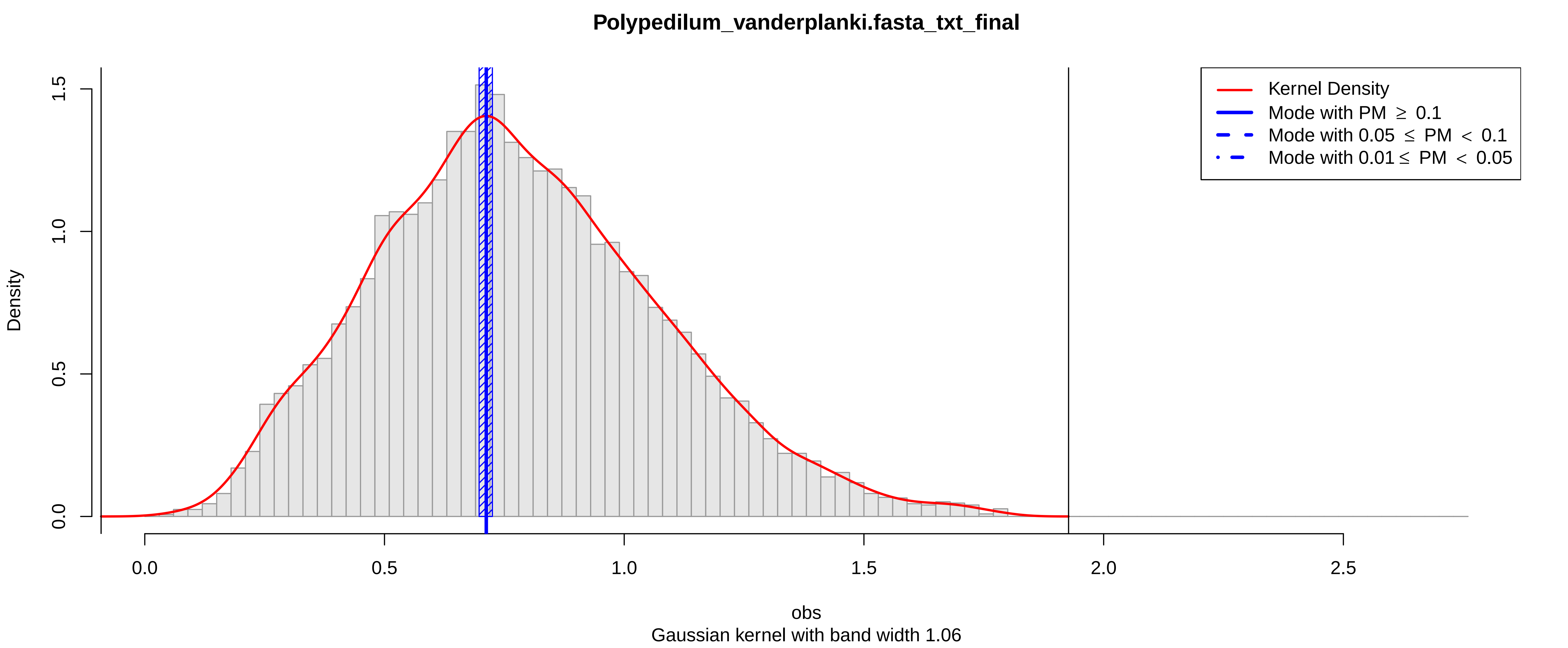
Polypedilum_vanderplanki.fasta_txt_final
Kernel Density
Mode with PM ≥ 0.1
Mode with 0.05 ≤ PM < 0.1
Mode with 0.01 ≤ PM < 0.05
Density
0.0
0.5
1.0
1.5
0.0
0.5
1.0
1.5
2.0
2.5
obs
Gaussian kernel with band width 1.06

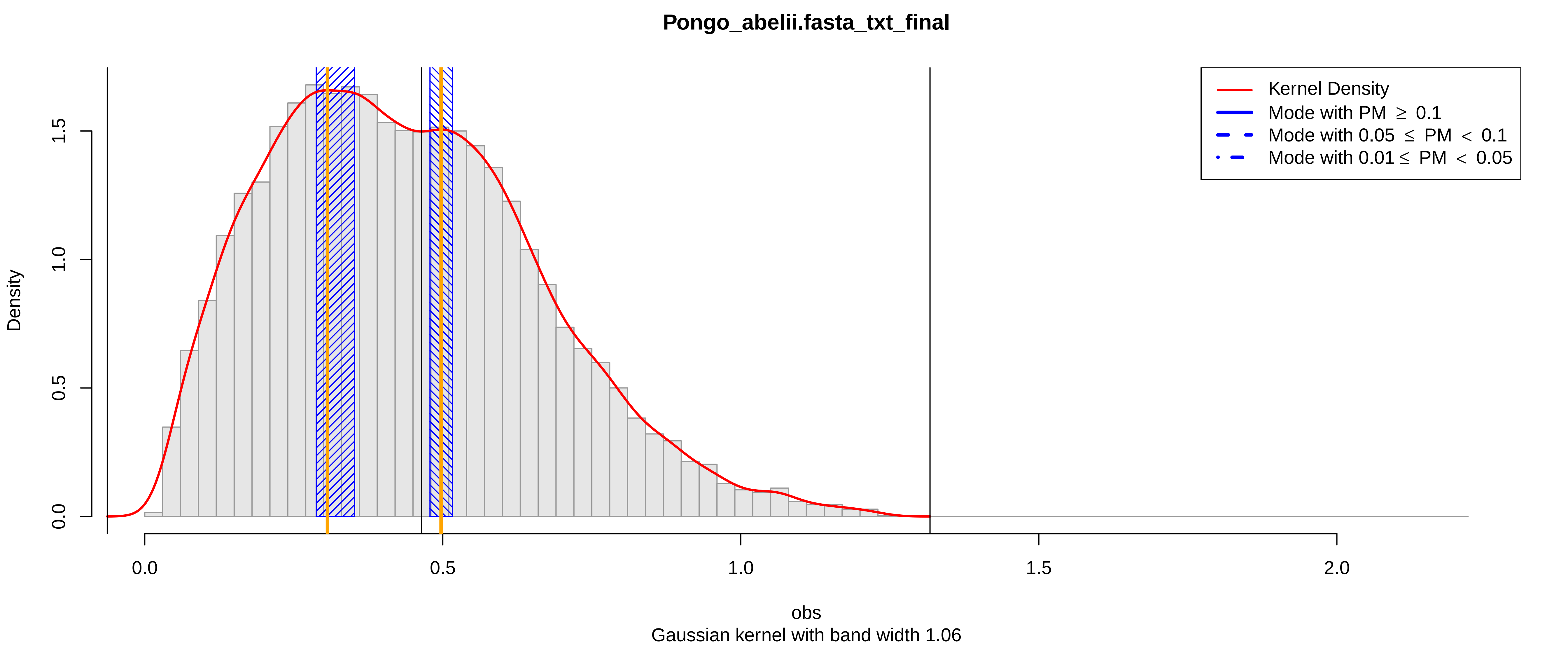

Pongo_abelii.fasta_txt_final
Kernel Density
Mode with PM ≥ 0.1
Mode with 0.05 ≤ PM < 0.1
Mode with 0.01 ≤ PM < 0.05
Density
0.0
0.5
1.0
1.5
2.0
obs
Gaussian kernel with band width 1.06

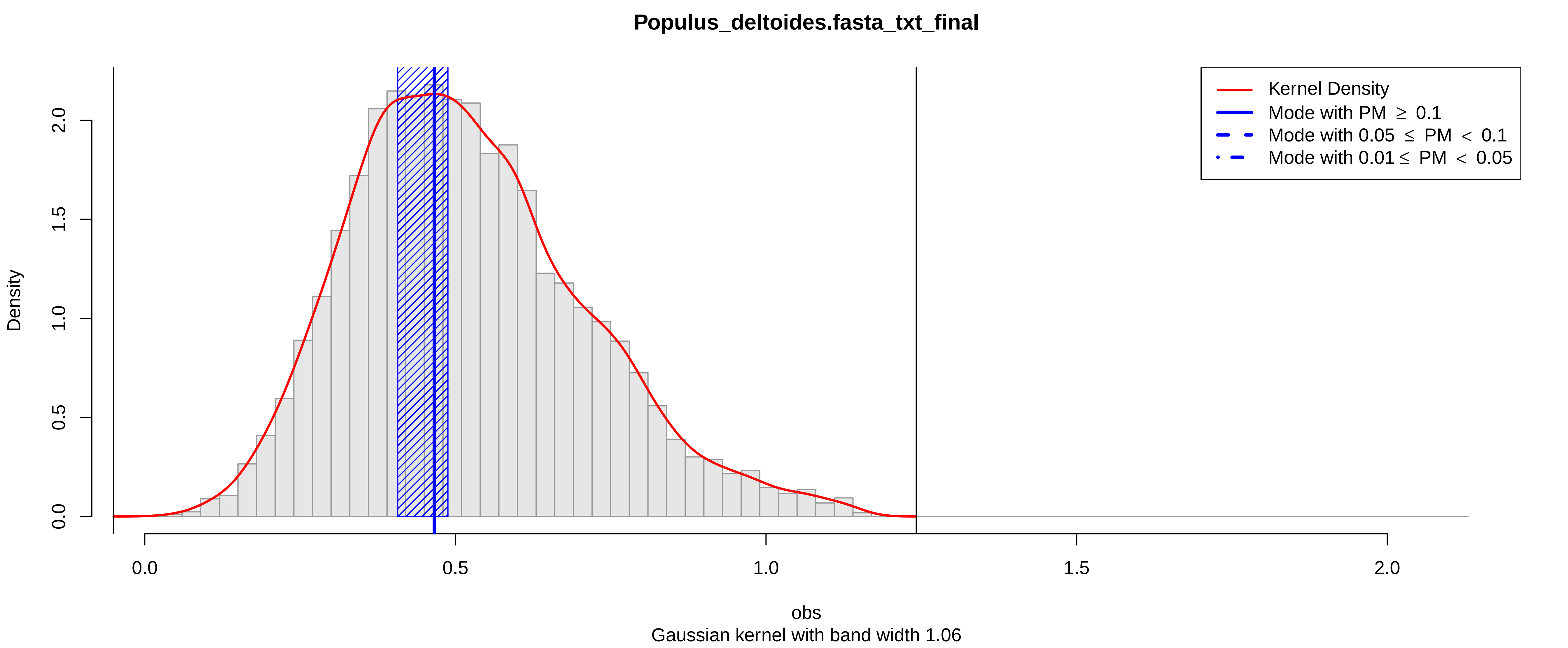
Populus_deltoides.fasta_txt_final
Density
obs
Gaussian kernel with band width 1.06
Kernel Density
Mode with PM ≥ 0.1
Mode with 0.05 ≤ PM < 0.1
Mode with 0.01 ≤ PM < 0.05
0.0
0.5
1.0
1.5
2.0

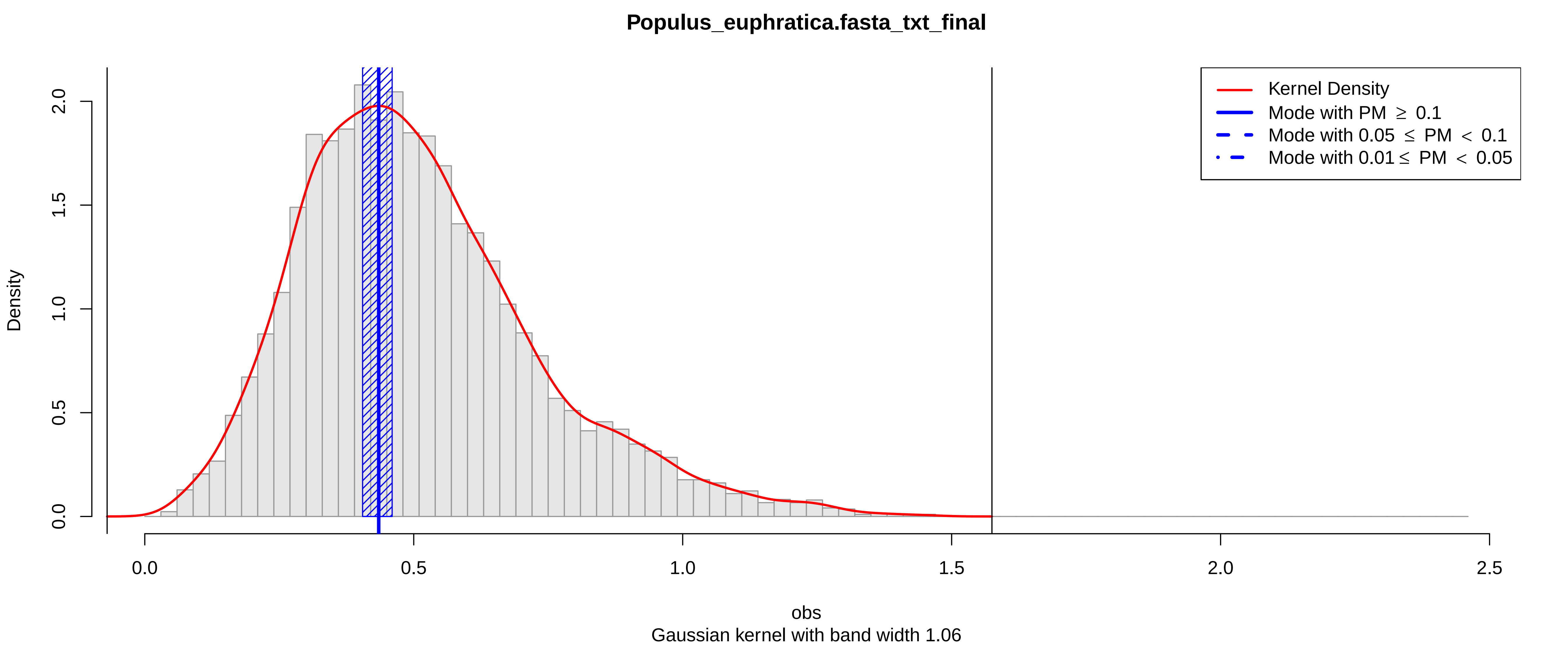
Populus_euphratica.fasta_txt_final
Kernel Density
Mode with PM ≥ 0.1
Mode with 0.05 ≤ PM < 0.1
Mode with 0.01 ≤ PM < 0.05
Density
0.0
0.5
1.0
1.5
2.0
2.5
obs
Gaussian kernel with band width 1.06

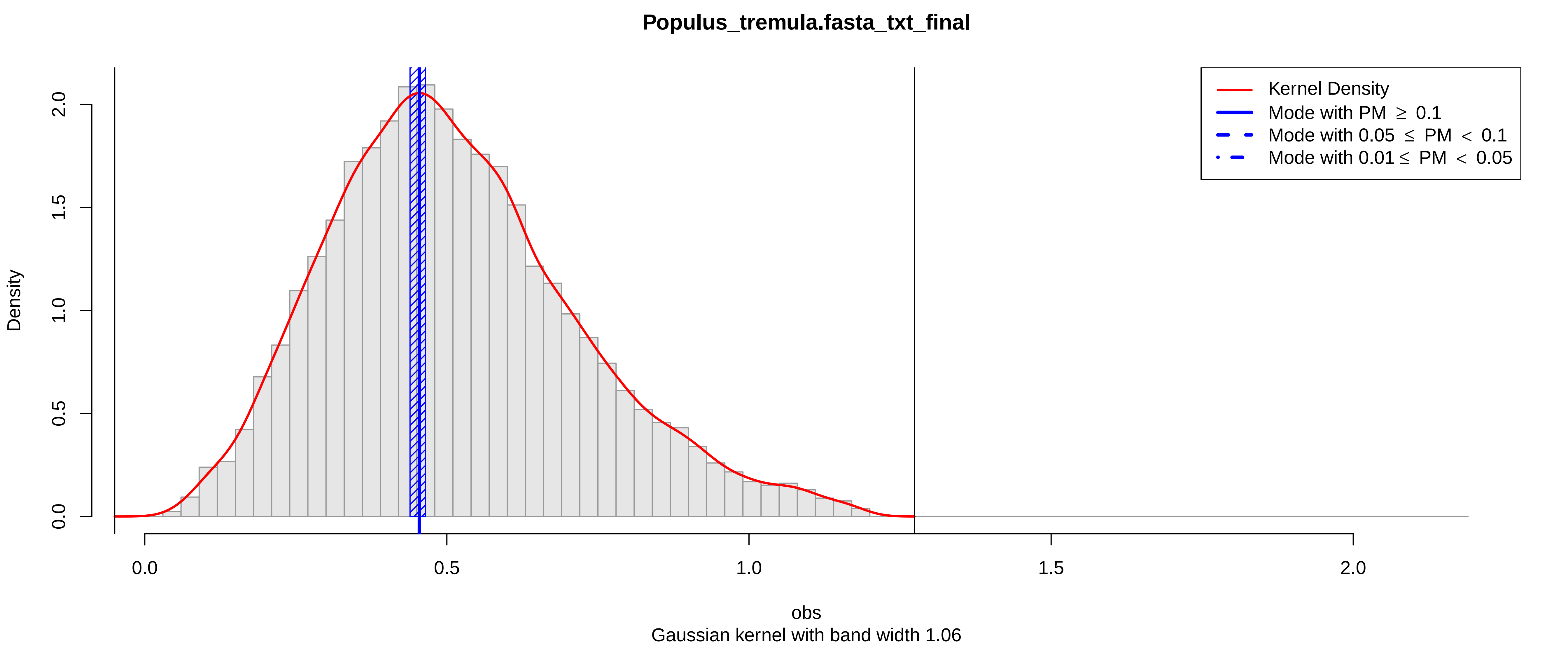

Populus_tremula.fasta_txt_final
Kernel Density
Mode with PM ≥ 0.1
Mode with 0.05 ≤ PM < 0.1
Mode with 0.01 ≤ PM < 0.05
Density
0.0
0.5
1.0
1.5
2.0
0.0
0.5
1.0
1.5
2.0
obs
Gaussian kernel with band width 1.06

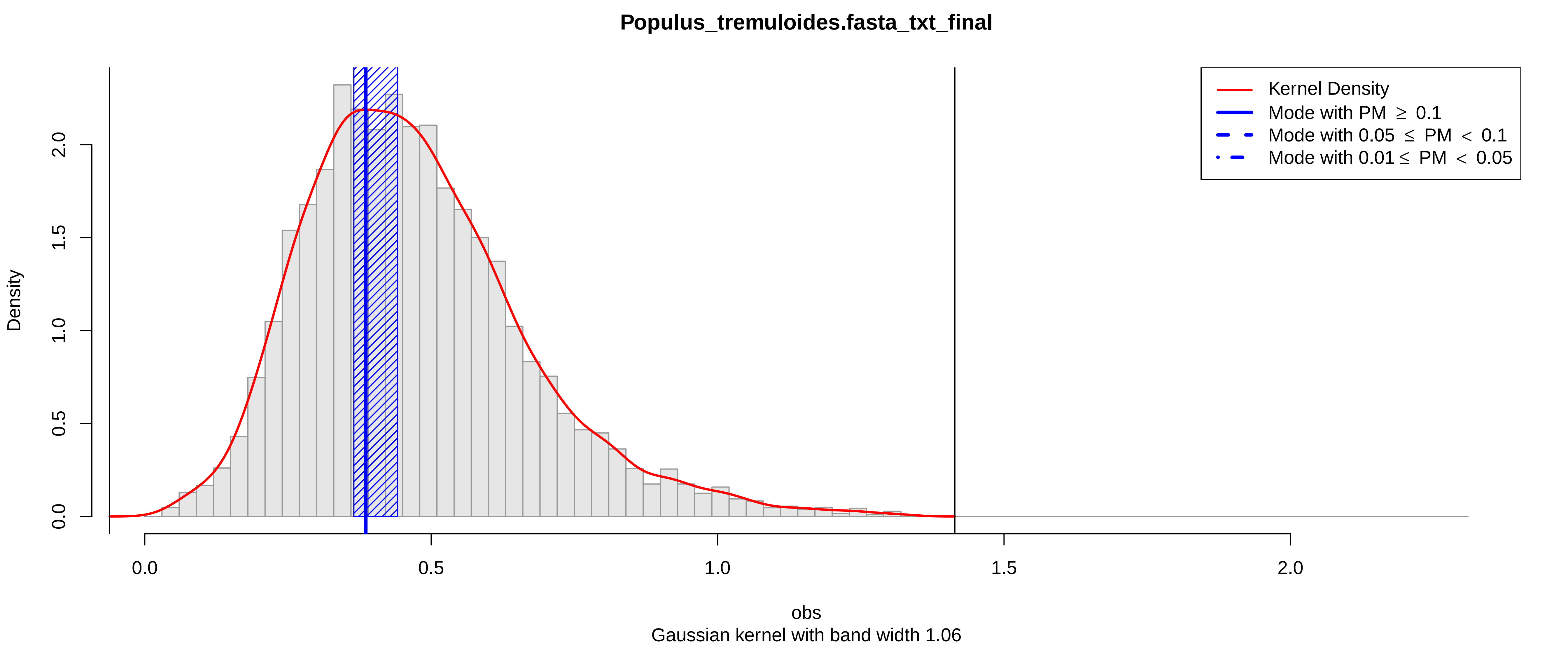

Populus_tremuloides.fasta_txt_final
Kernel Density
Mode with PM ≥ 0.1
Mode with 0.05 ≤ PM < 0.1
Mode with 0.01 ≤ PM < 0.05
Density
0.0
0.5
1.0
1.5
2.0
0.0
0.5
1.0
1.5
2.0
obs
Gaussian kernel with band width 1.06

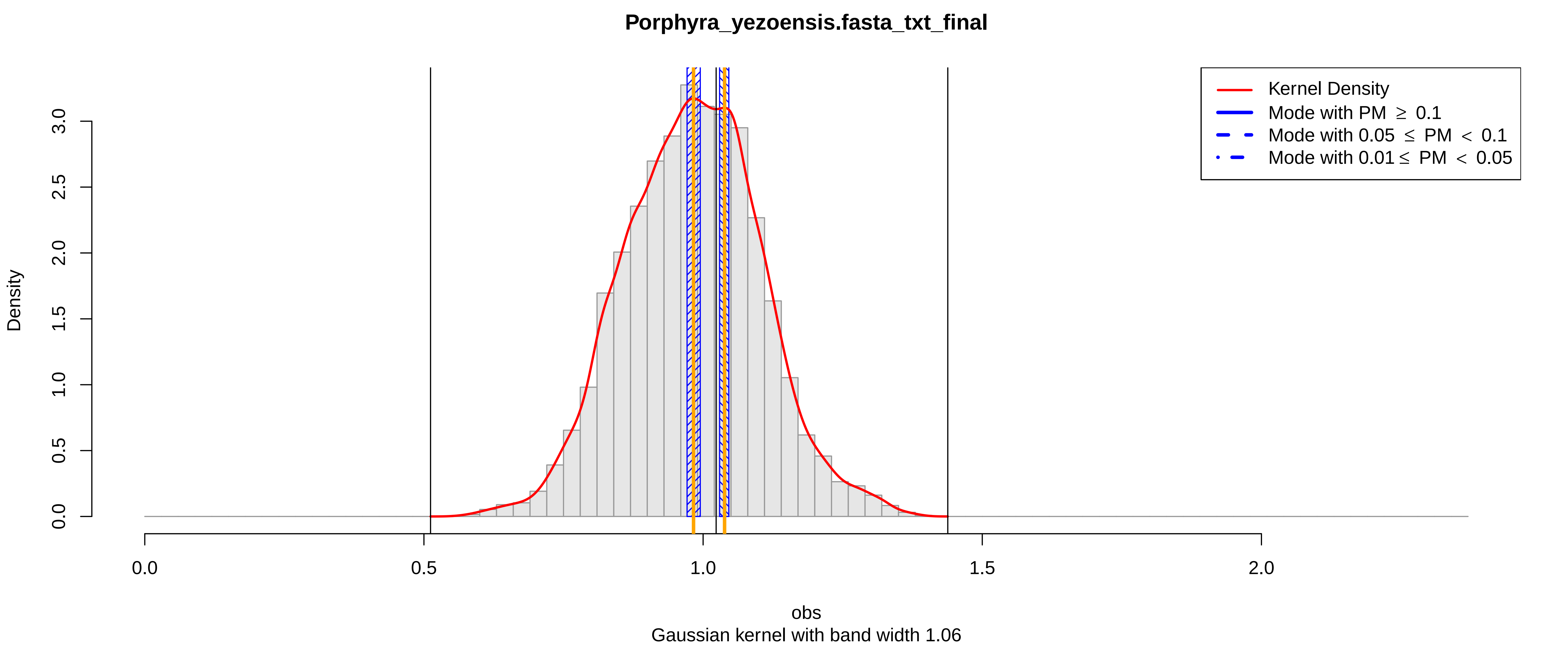

Porphyra_yezoensis.fasta_txt_final
Kernel Density
Mode with PM ≥ 0.1
Mode with 0.05 ≤ PM < 0.1
Mode with 0.01 ≤ PM < 0.05
Density
0.0
0.5
1.0
1.5
2.0
2.5
3.0
0.0
0.5
1.0
1.5
2.0
obs
Gaussian kernel with band width 1.06

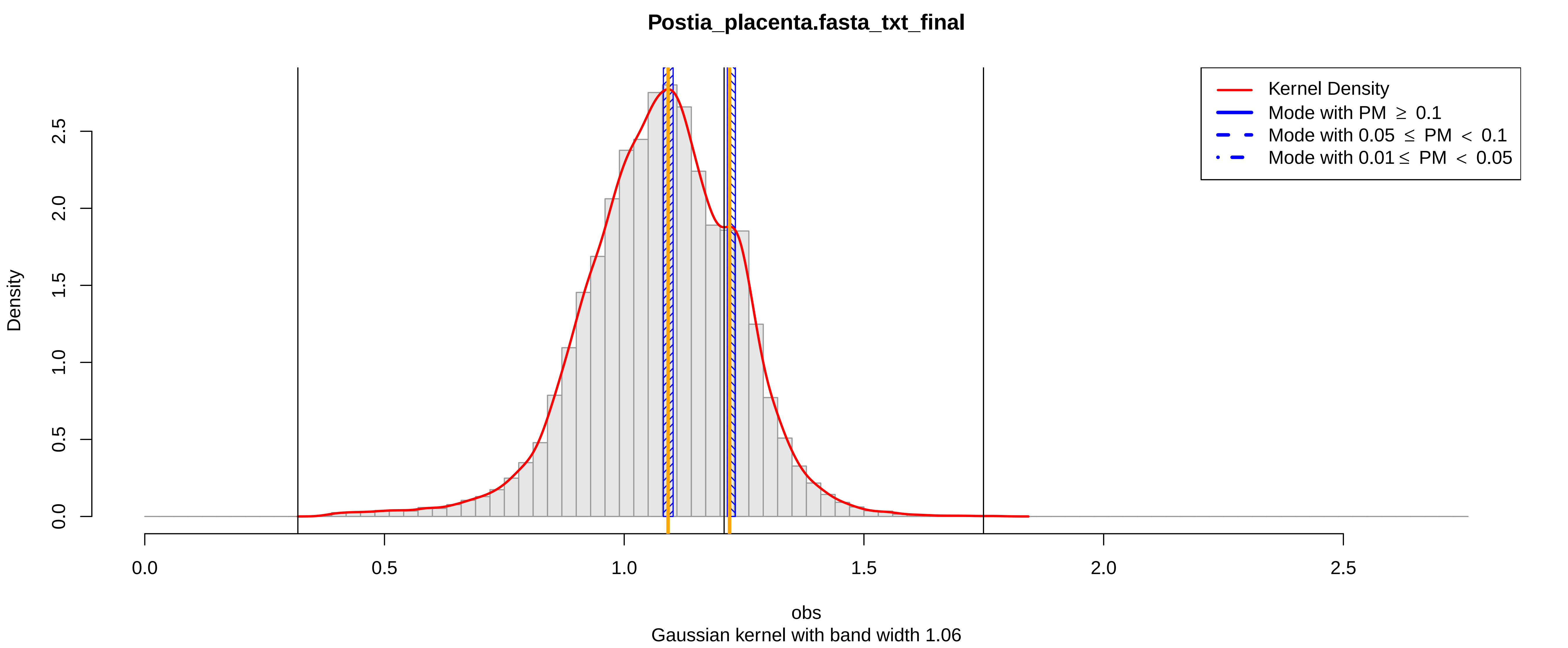

Postia_placenta.fasta_txt_final
Kernel Density
Mode with PM ≥ 0.1
Mode with 0.05 ≤ PM < 0.1
Mode with 0.01 ≤ PM < 0.05
Density
0.0
0.5
1.0
1.5
2.0
2.5
0.0
0.5
1.0
1.5
2.0
2.5
obs
Gaussian kernel with band width 1.06

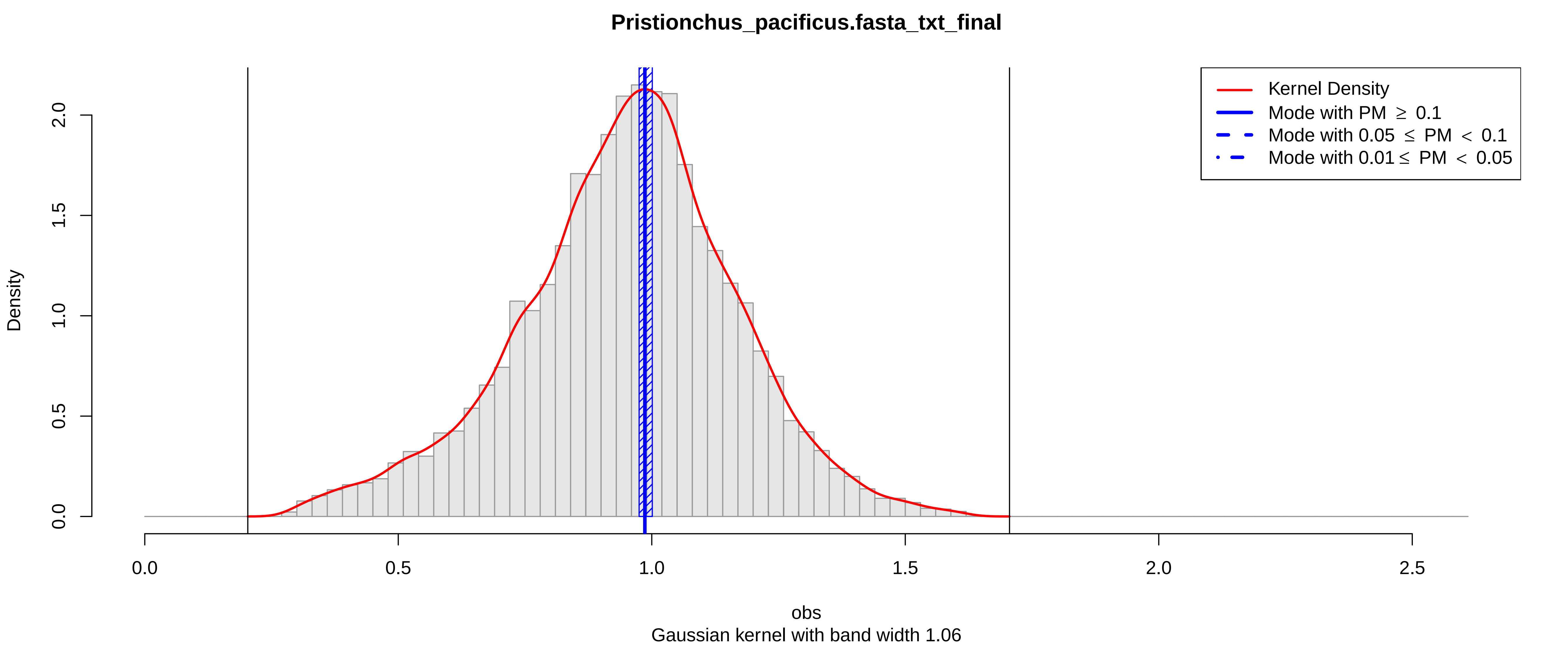

Pristionchus_pacificus.fasta_txt_final
Density
0.0
0.5
1.0
1.5
2.0
0.0
0.5
1.0
1.5
2.0
2.5
Kernel Density
Mode with PM ≥ 0.1
Mode with 0.05 ≤ PM < 0.1
Mode with 0.01 ≤ PM < 0.05
obs
Gaussian kernel with band width 1.06

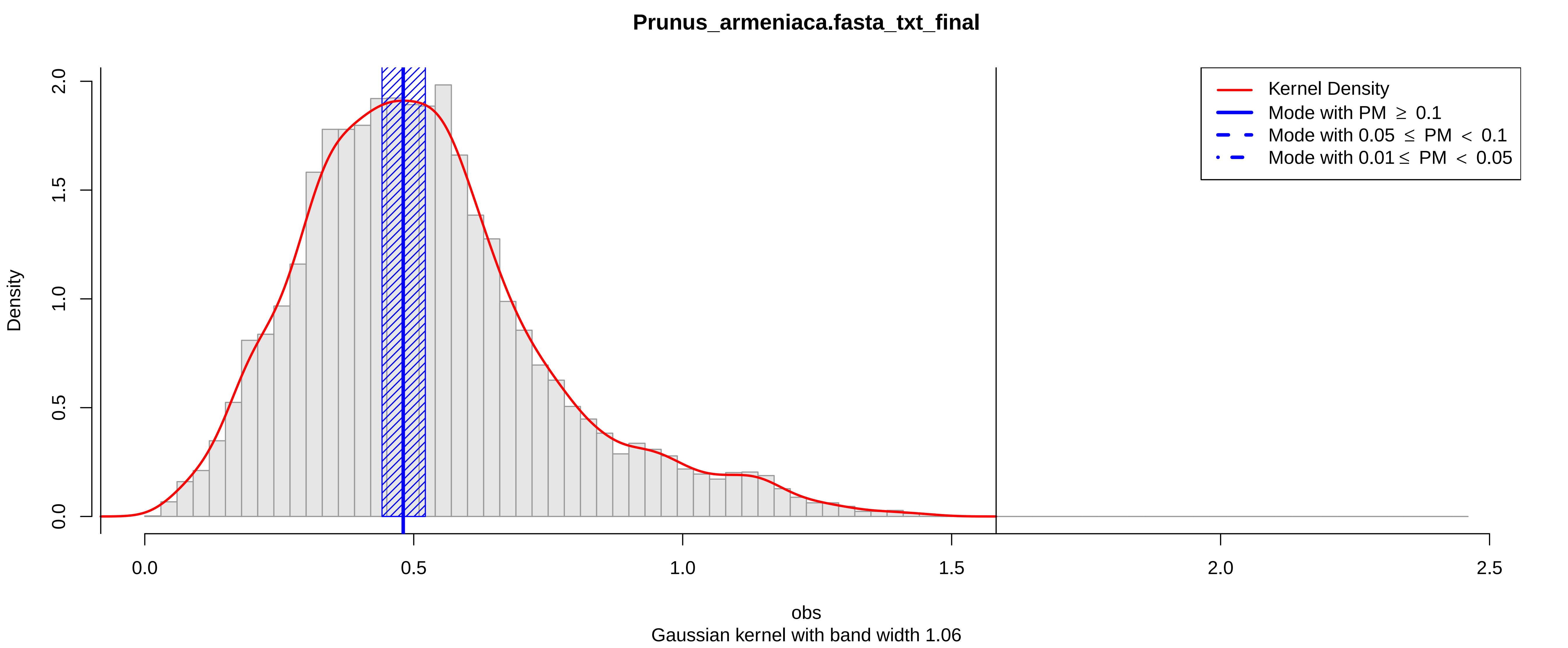

Prunus_armeniaca.fasta_txt_final
Kernel Density
Mode with PM ≥ 0.1
Mode with 0.05 ≤ PM < 0.1
Mode with 0.01 ≤ PM < 0.05
Density
0.0
0.5
1.0
1.5
2.0
0.0
0.5
1.0
1.5
2.0
2.5
obs
Gaussian kernel with band width 1.06

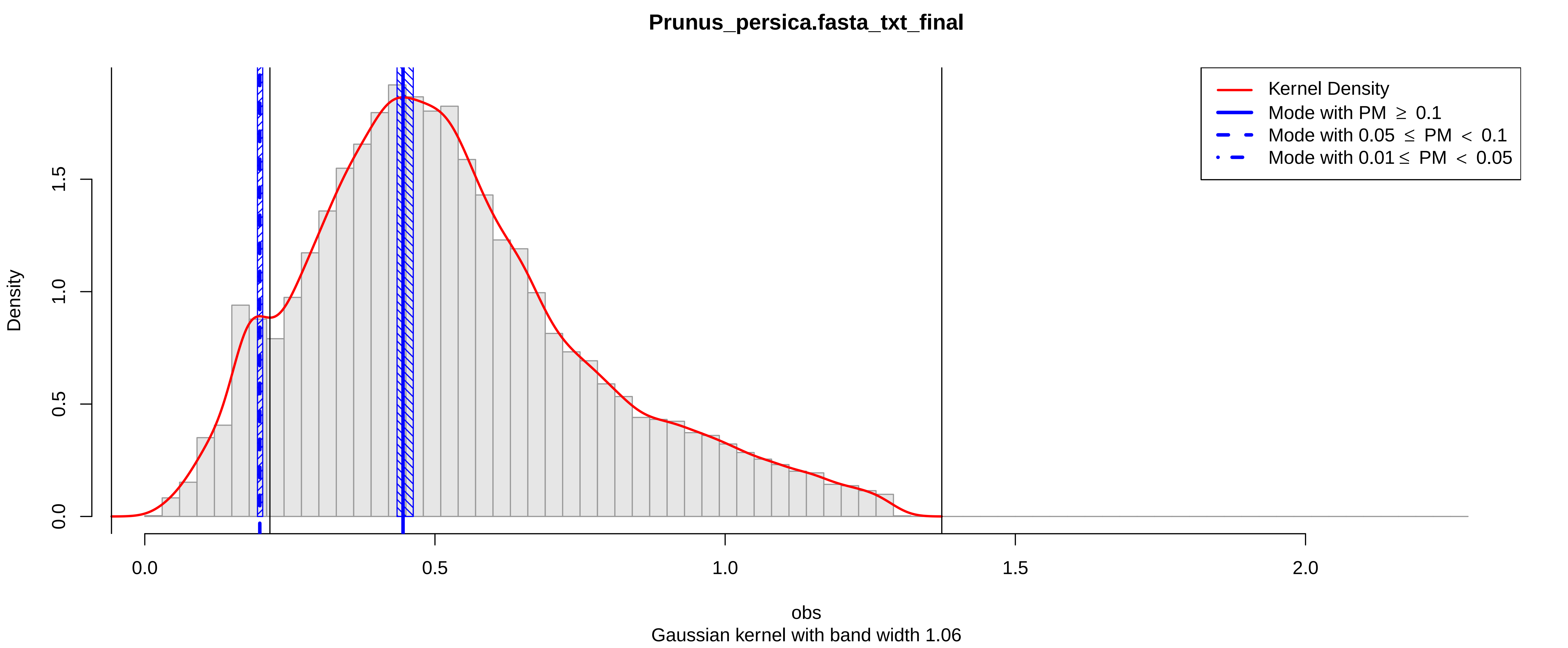

Prunus_persica.fasta_txt_final
Kernel Density
Mode with PM ≥ 0.1
Mode with 0.05 ≤ PM < 0.1
Mode with 0.01 ≤ PM < 0.05
Density
0.0
0.5
1.0
1.5
2.0
obs
Gaussian kernel with band width 1.06

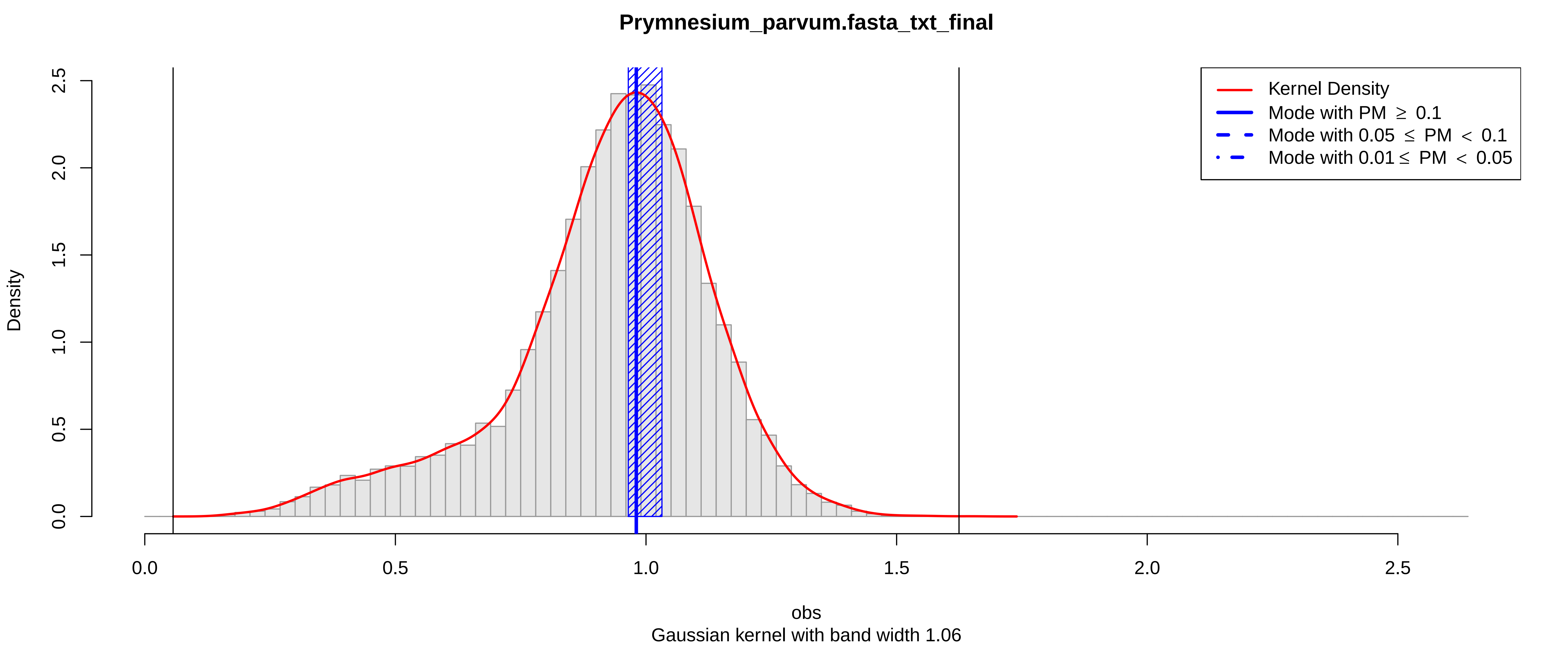

Prymnesium_parvum.fasta_txt_final
Kernel Density
Mode with PM ≥ 0.1
Mode with 0.05 ≤ PM < 0.1
Mode with 0.01 ≤ PM < 0.05
Density
0.0
0.5
1.0
1.5
2.0
2.5
obs
Gaussian kernel with band width 1.06

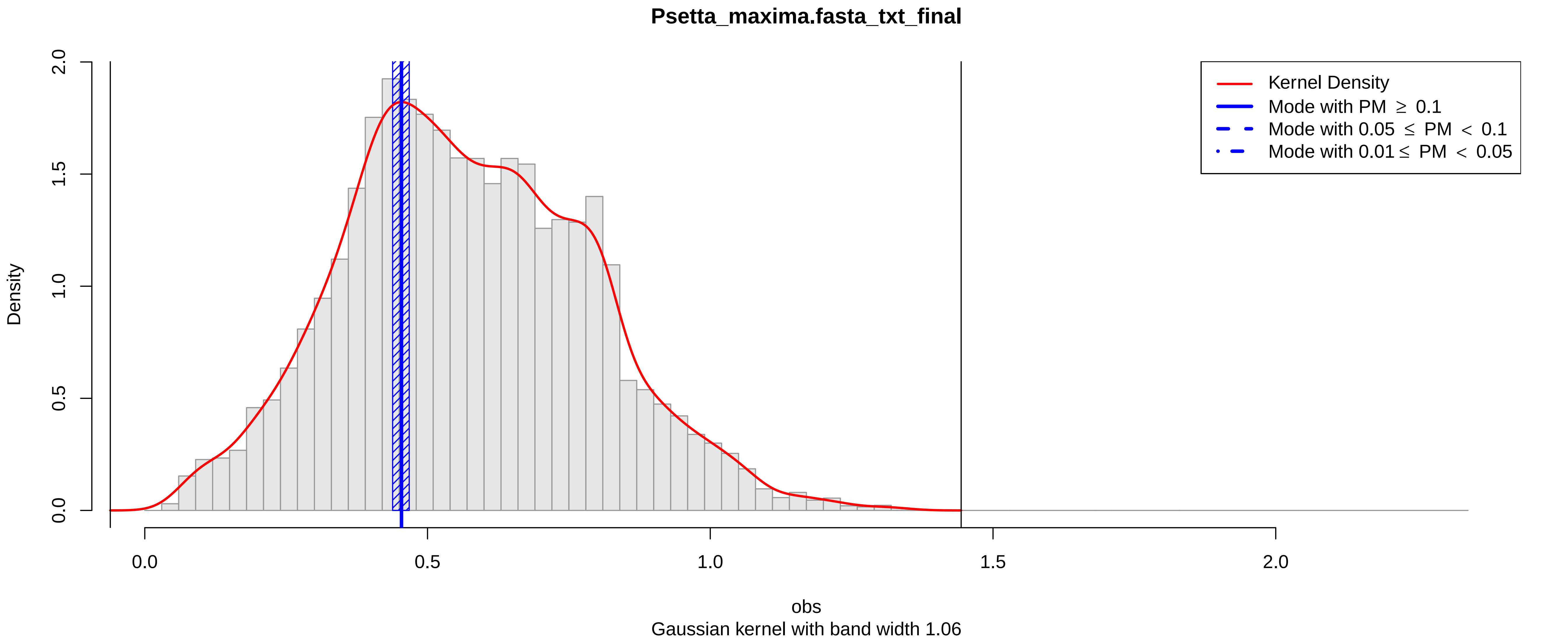
Psetta_maxima.fasta_txt_final
Density
0.0
0.5
1.0
1.5
2.0
0.0
0.5
1.0
1.5
2.0
obs
Gaussian kernel with band width 1.06
Kernel Density
Mode with PM ≥ 0.1
Mode with 0.05 ≤ PM < 0.1
Mode with 0.01 ≤ PM < 0.05

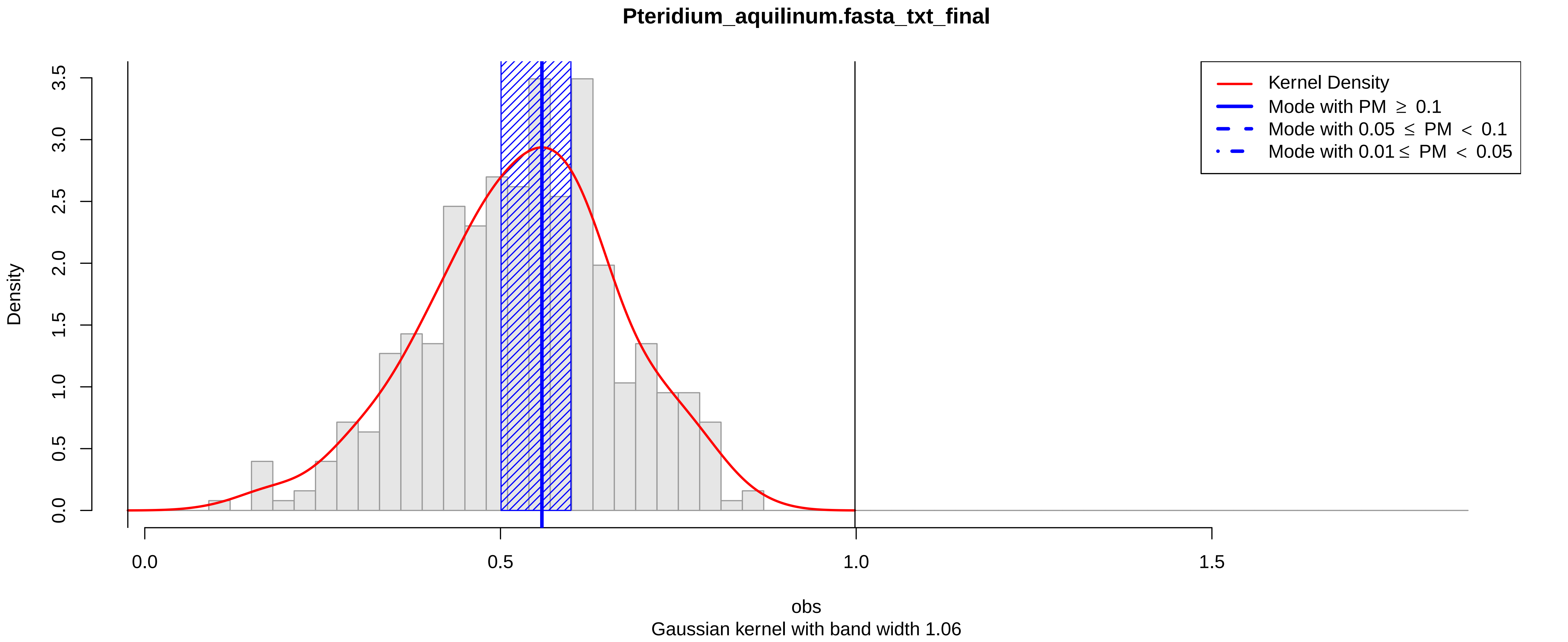
Pteridium_aquilinum.fasta_txt_final
Density
0.0
0.5
1.0
1.5
2.0
2.5
3.0
3.5
0.0
0.5
1.0
1.5
obs
Gaussian kernel with band width 1.06
Kernel Density
Mode with PM ≥ 0.1
Mode with 0.05 ≤ PM < 0.1
Mode with 0.01 ≤ PM < 0.05

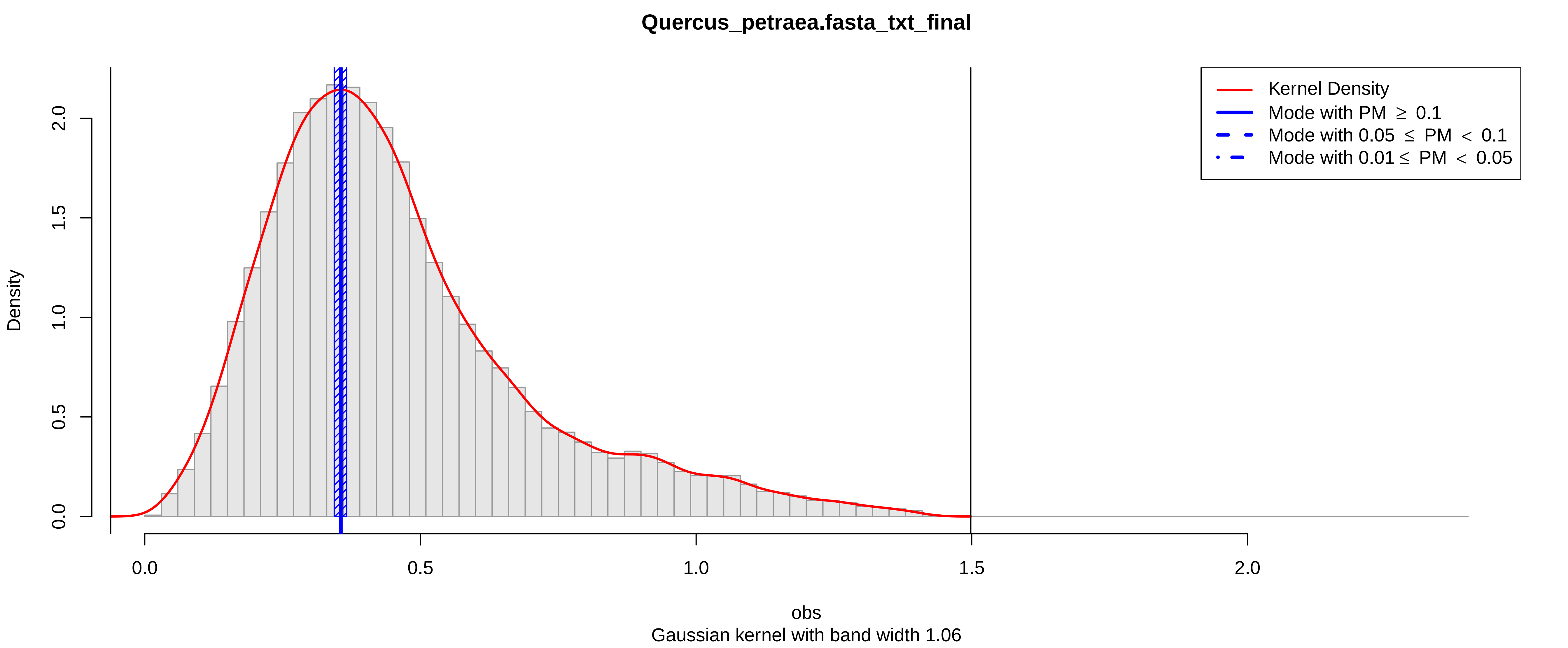

Quercus_petraea.fasta_txt_final
Kernel Density
Mode with PM ≥ 0.1
Mode with 0.05 ≤ PM < 0.1
Mode with 0.01 ≤ PM < 0.05
Density
0.0
0.5
1.0
1.5
2.0
0.0
0.5
1.0
1.5
2.0
obs
Gaussian kernel with band width 1.06

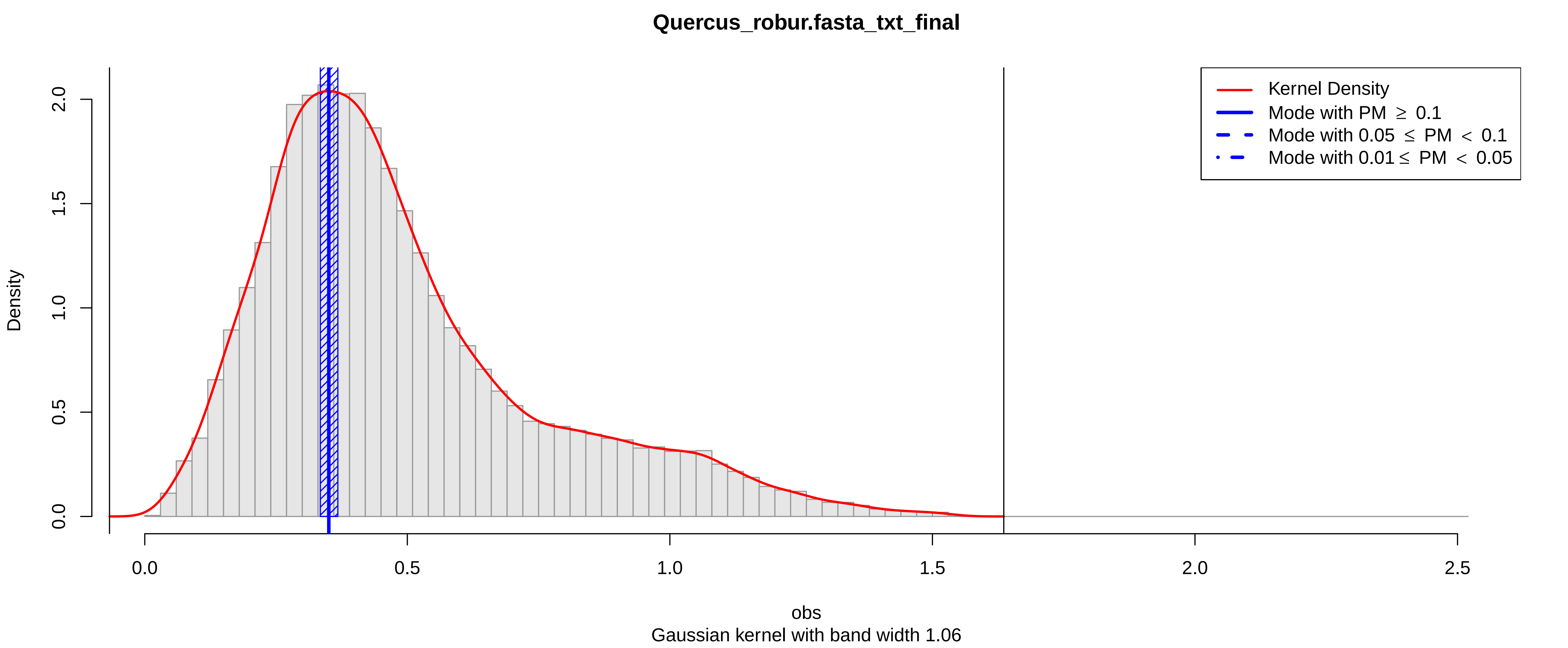
Quercus_robur.fasta_txt_final
Density
obs
Gaussian kernel with band width 1.06
0.0
0.5
1.0
1.5
2.0
2.5
Kernel Density
Mode with PM ≥ 0.1
Mode with 0.05 ≤ PM < 0.1
Mode with 0.01 ≤ PM < 0.05

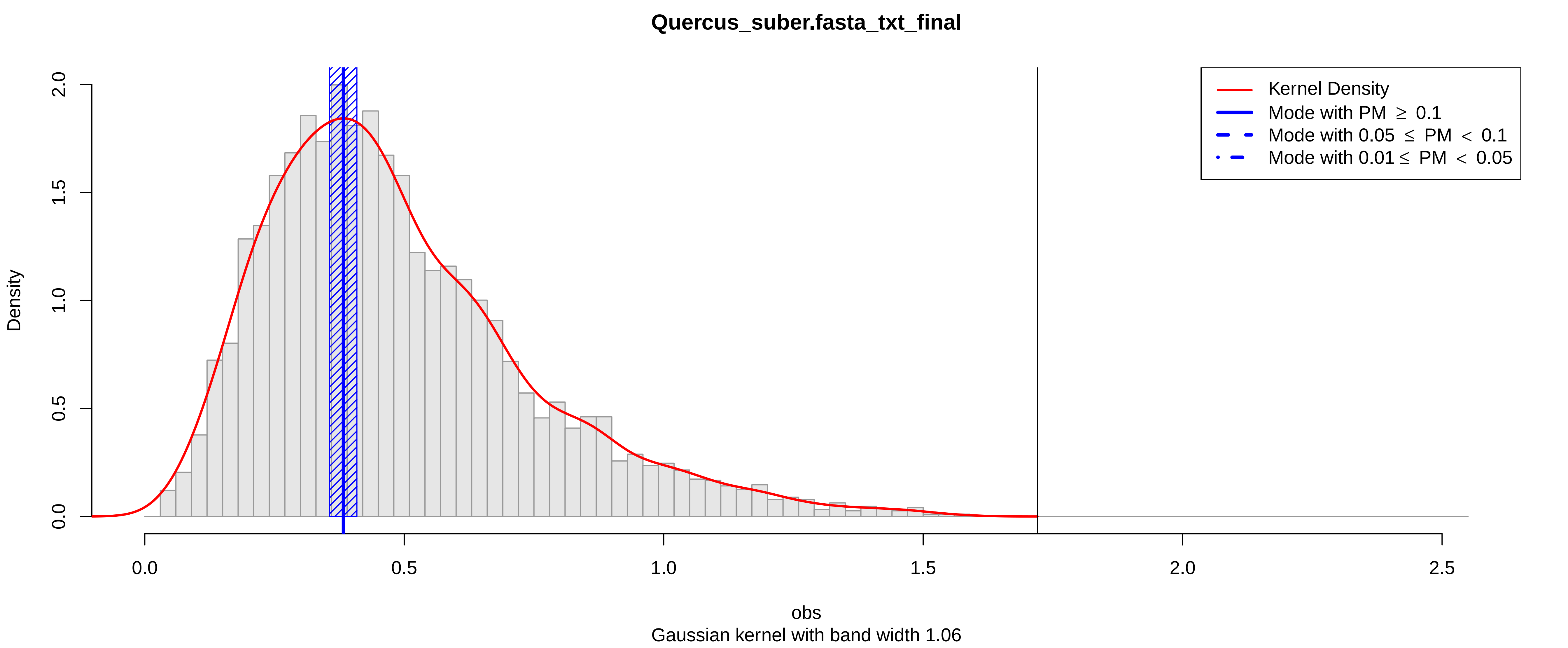
Quercus_suber.fasta_txt_final
Kernel Density
Mode with PM ≥ 0.1
Mode with 0.05 ≤ PM < 0.1
Mode with 0.01 ≤ PM < 0.05
Density
0.0
0.5
1.0
1.5
2.0
2.5
obs
Gaussian kernel with band width 1.06

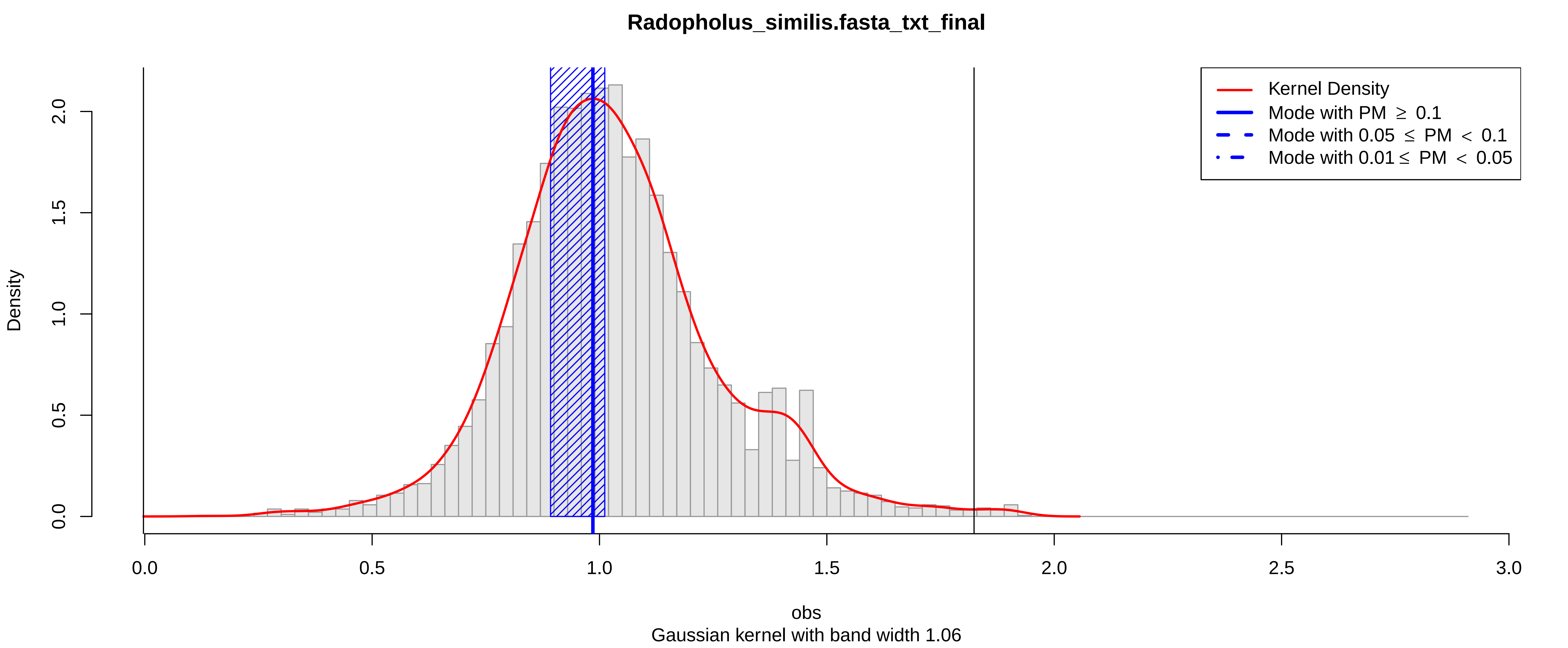

Radopholus_similis.fasta_txt_final
Kernel Density
Mode with PM ≥ 0.1
Mode with 0.05 ≤ PM < 0.1
Mode with 0.01 ≤ PM < 0.05
Density
0.0
0.5
1.0
1.5
2.0
0.0
0.5
1.0
1.5
2.0
2.5
3.0
obs
Gaussian kernel with band width 1.06

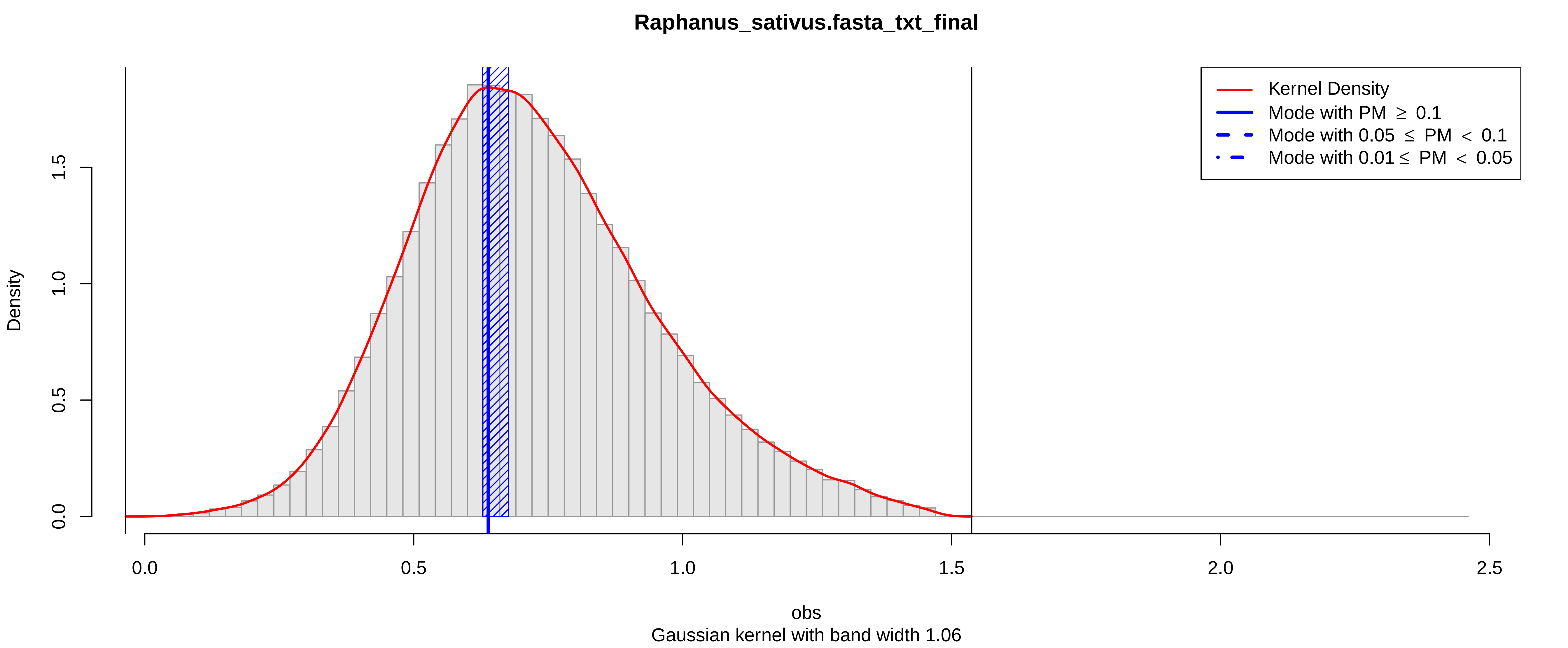
Raphanus_sativus.fasta_txt_final
Kernel Density
Mode with PM ≥ 0.1
Mode with 0.05 ≤ PM < 0.1
Mode with 0.01 ≤ PM < 0.05
Density
0.0
0.5
1.0
1.5
0.0
0.5
1.0
1.5
2.0
2.5
obs
Gaussian kernel with band width 1.06

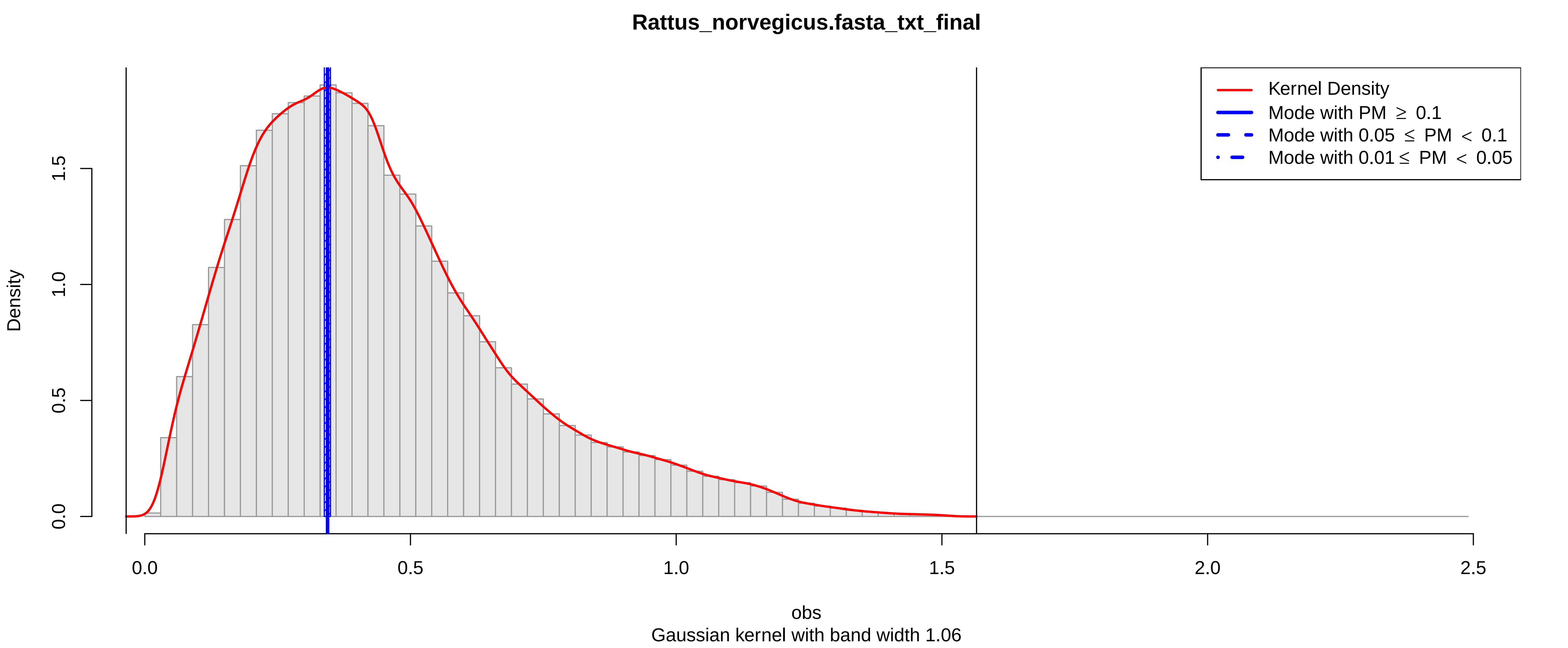

Rattus_norvegicus.fasta_txt_final
Kernel Density
Mode with PM ≥ 0.1
Mode with 0.05 ≤ PM < 0.1
Mode with 0.01 ≤ PM < 0.05
Density
0.0
0.5
1.0
1.5
0.0
0.5
1.0
1.5
2.0
2.5
obs
Gaussian kernel with band width 1.06

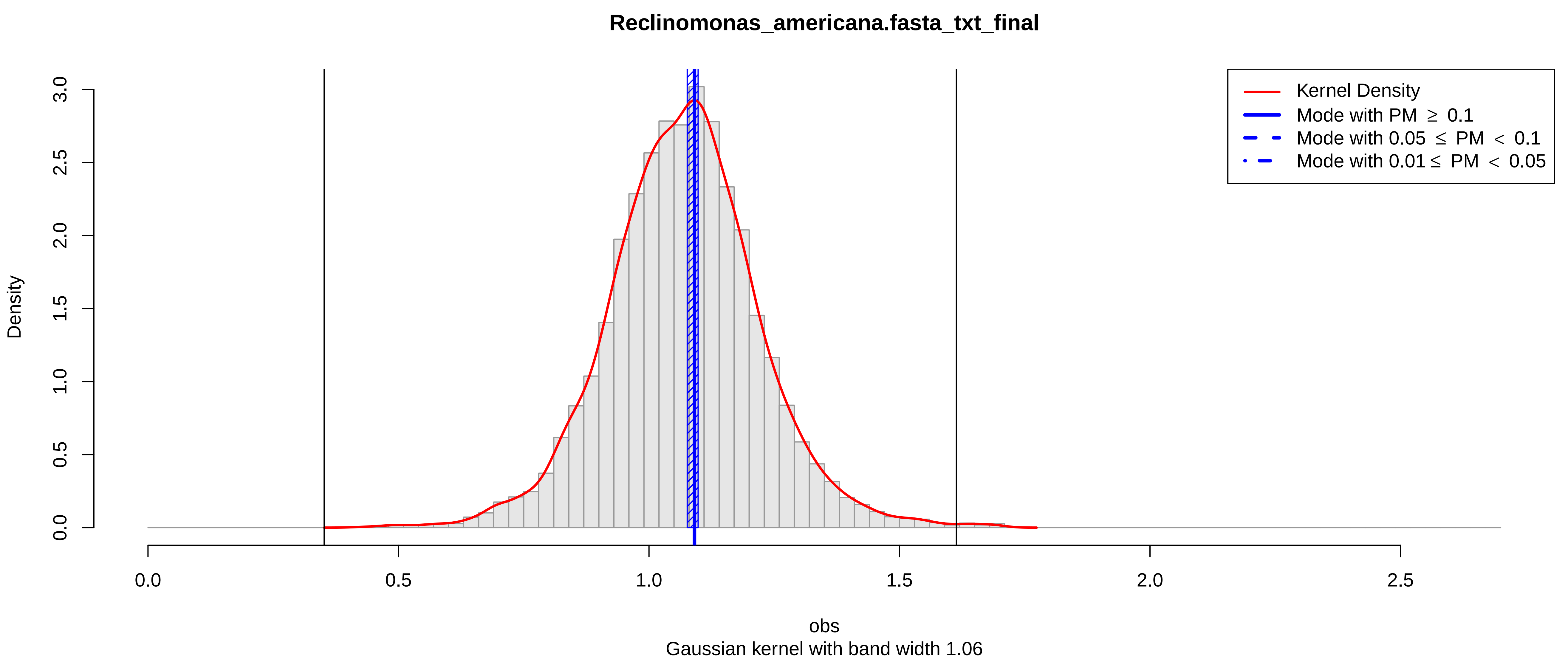

Reclinomonas_americana.fasta_txt_final
Kernel Density
Mode with PM ≥ 0.1
Mode with 0.05 ≤ PM < 0.1
Mode with 0.01 ≤ PM < 0.05
Density
0.0
0.5
1.0
1.5
2.0
2.5
3.0
obs
Gaussian kernel with band width 1.06

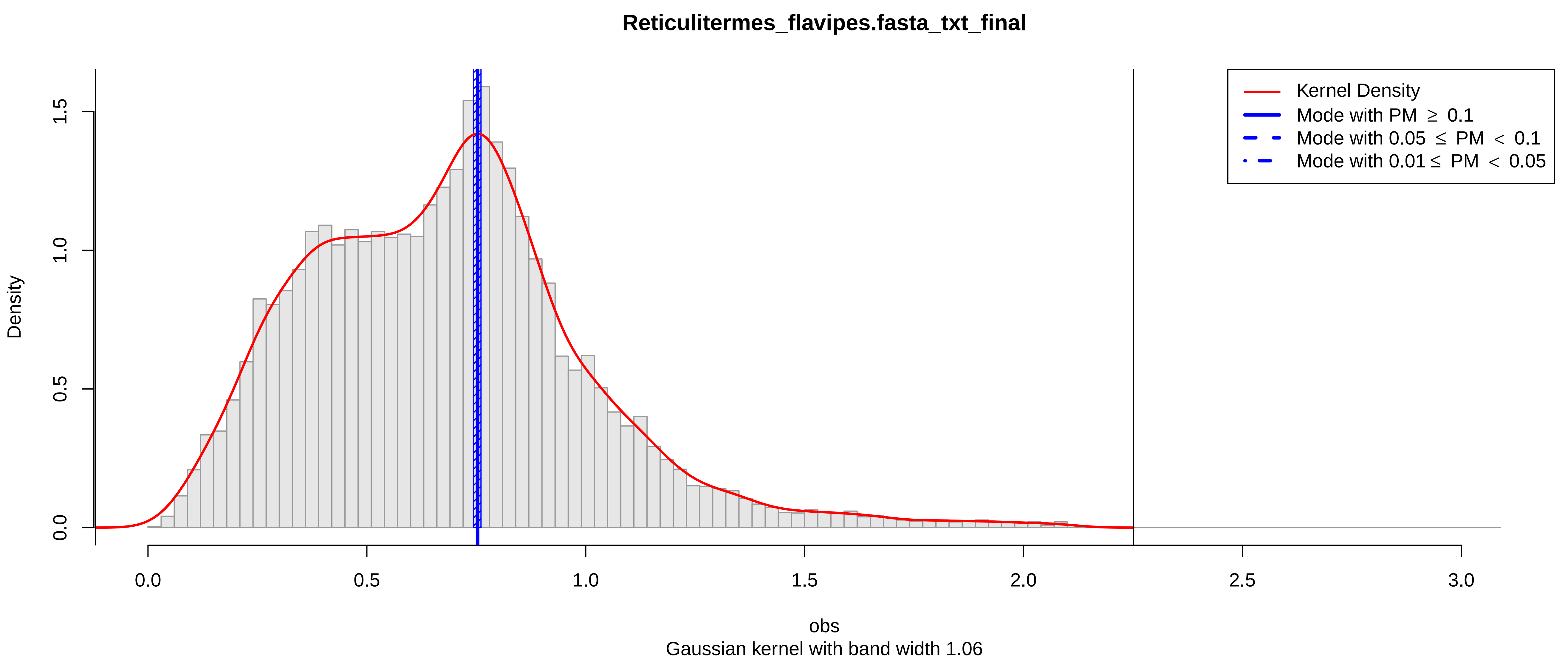

Reticulitermes_flavipes.fasta_txt_final
Kernel Density
Mode with PM ≥ 0.1
Mode with 0.05 ≤ PM < 0.1
Mode with 0.01 ≤ PM < 0.05
Density
0.0
0.5
1.0
1.5
0.0
0.5
1.0
1.5
2.0
2.5
3.0
obs
Gaussian kernel with band width 1.06

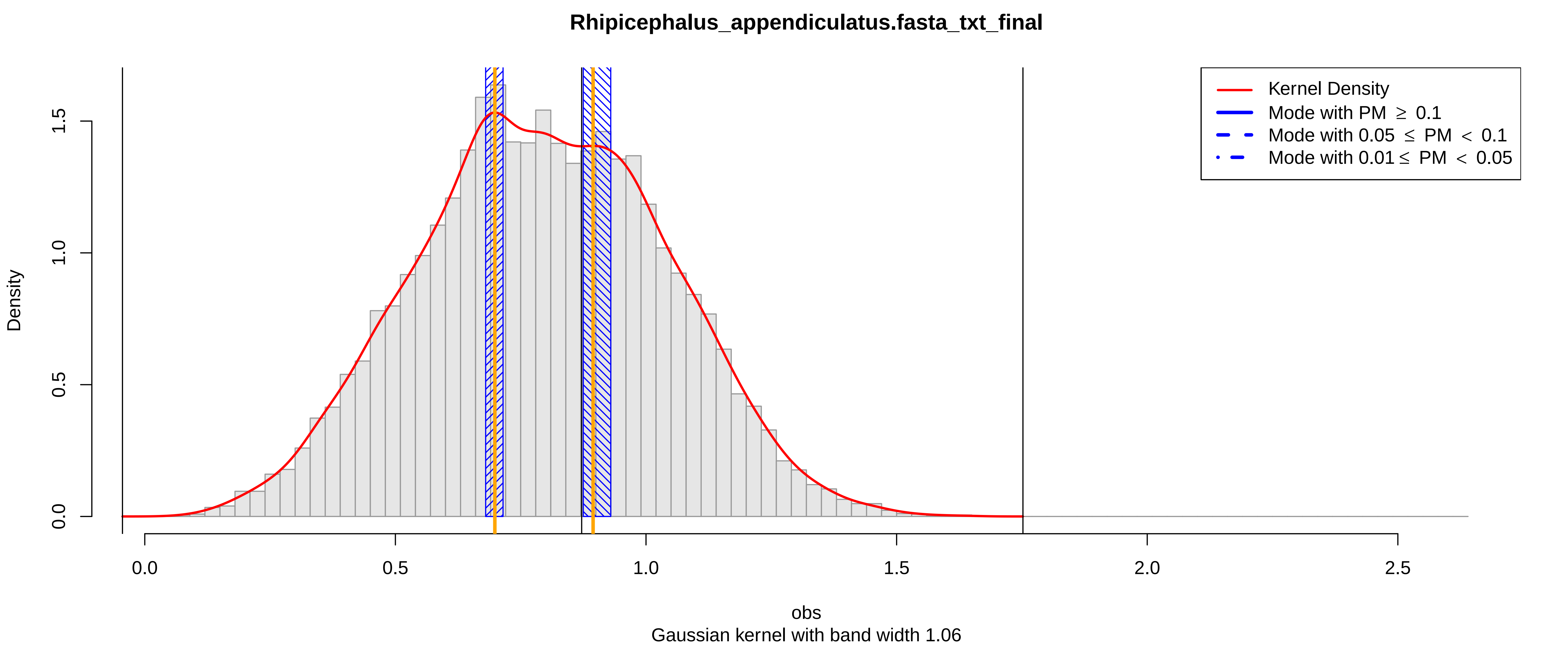

Rhipicephalus_appendiculatus.fasta_txt_final
Density
0.0
0.5
1.0
1.5
Kernel Density
Mode with PM ≥ 0.1
Mode with 0.05 ≤ PM < 0.1
Mode with 0.01 ≤ PM < 0.05
0.0
0.5
1.0
1.5
2.0
2.5
obs
Gaussian kernel with band width 1.06

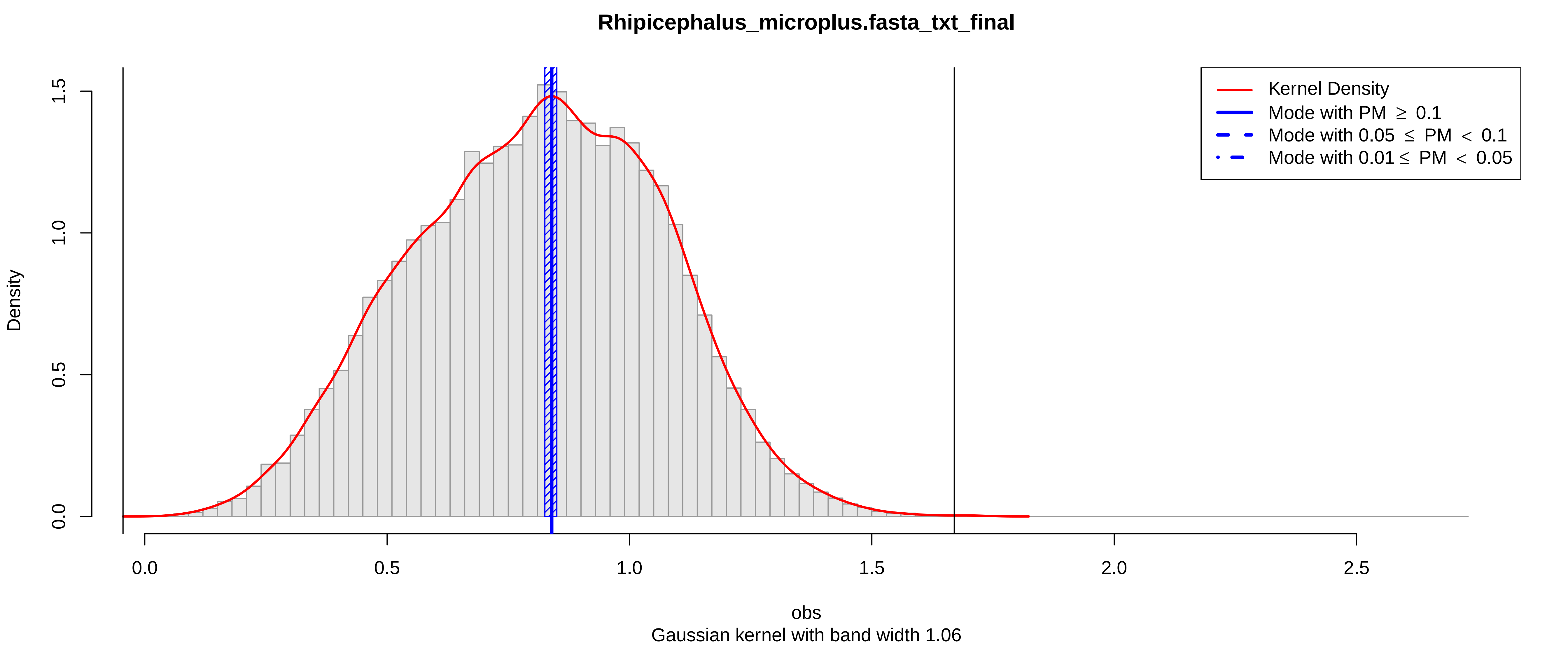
Rhipicephalus_microplus.fasta_txt_final
Density
0.0
0.5
1.0
1.5
0.0
0.5
1.0
1.5
2.0
2.5
Kernel Density
Mode with PM ≥ 0.1
Mode with 0.05 ≤ PM < 0.1
Mode with 0.01 ≤ PM < 0.05
obs
Gaussian kernel with band width 1.06

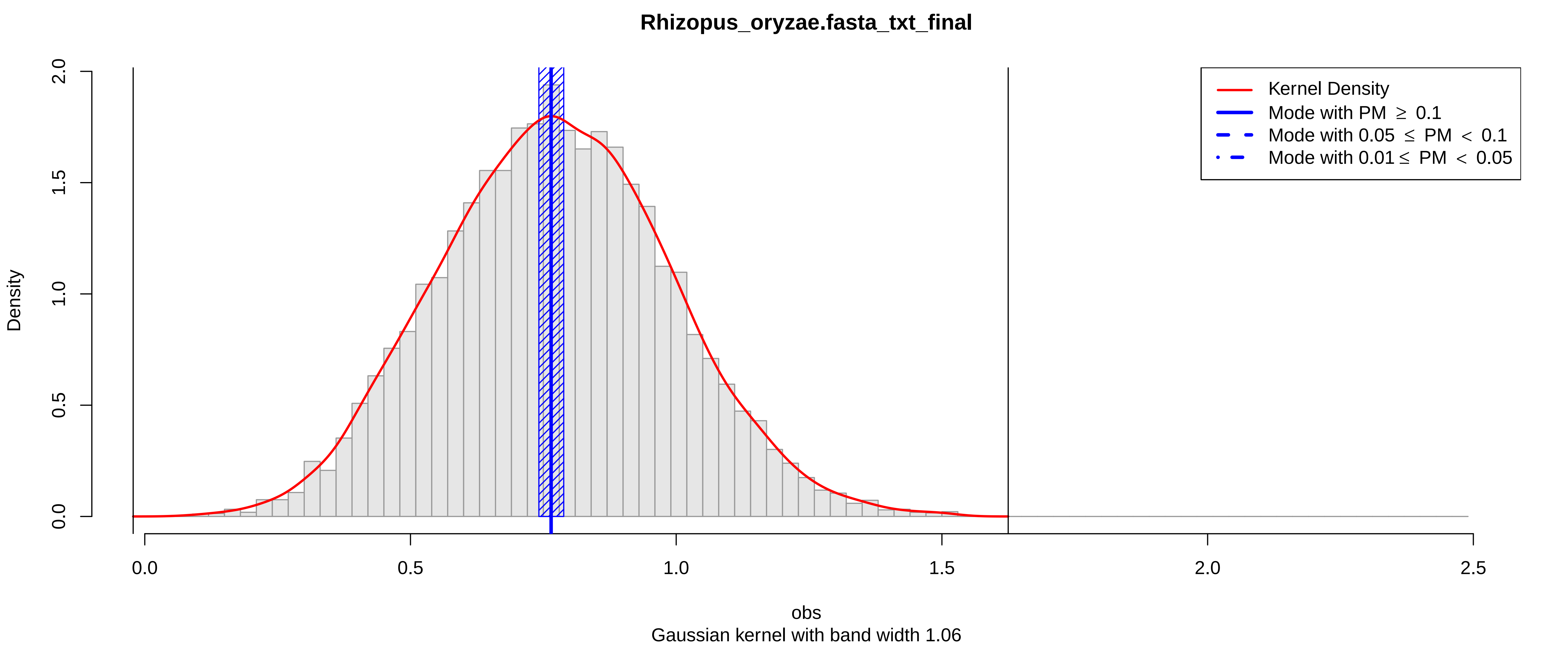
Rhizopus_oryzae.fasta_txt_final
Kernel Density
Mode with PM ≥ 0.1
Mode with 0.05 ≤ PM < 0.1
Mode with 0.01 ≤ PM < 0.05
Density
0.0
0.5
1.0
1.5
2.0
0.0
0.5
1.0
1.5
2.0
2.5
obs
Gaussian kernel with band width 1.06

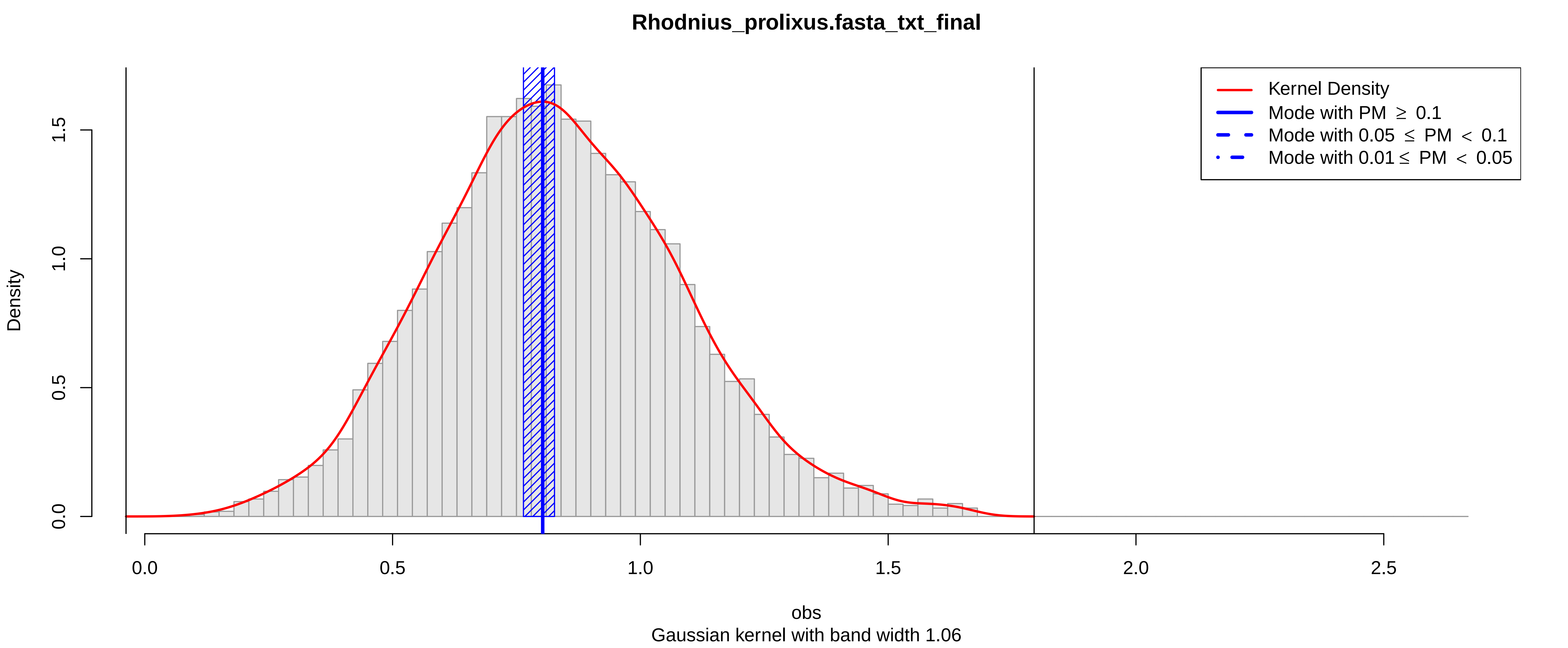
Rhodnius_prolixus.fasta_txt_final
Kernel Density
Mode with PM ≥ 0.1
Mode with 0.05 ≤ PM < 0.1
Mode with 0.01 ≤ PM < 0.05
Density
0.0
0.5
1.0
1.5
0.0
0.5
1.0
1.5
2.0
2.5
obs
Gaussian kernel with band width 1.06

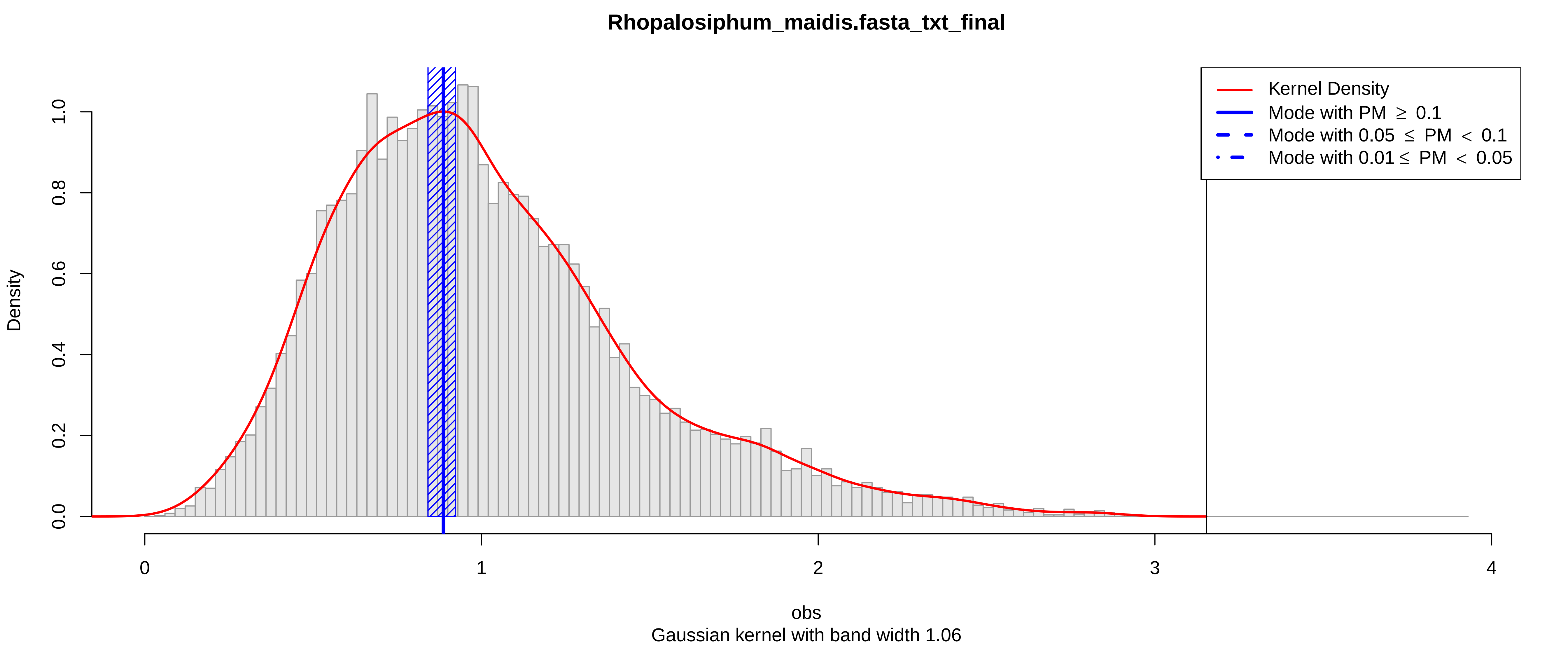
Rhopalosiphum_maidis.fasta_txt_final
Kernel Density
Mode with PM ≥ 0.1
Mode with 0.05 ≤ PM < 0.1
Mode with 0.01 ≤ PM < 0.05
Density
0.0
0.2
0.4
0.6
0.8
1.0
0
1
2
3
4
obs
Gaussian kernel with band width 1.06

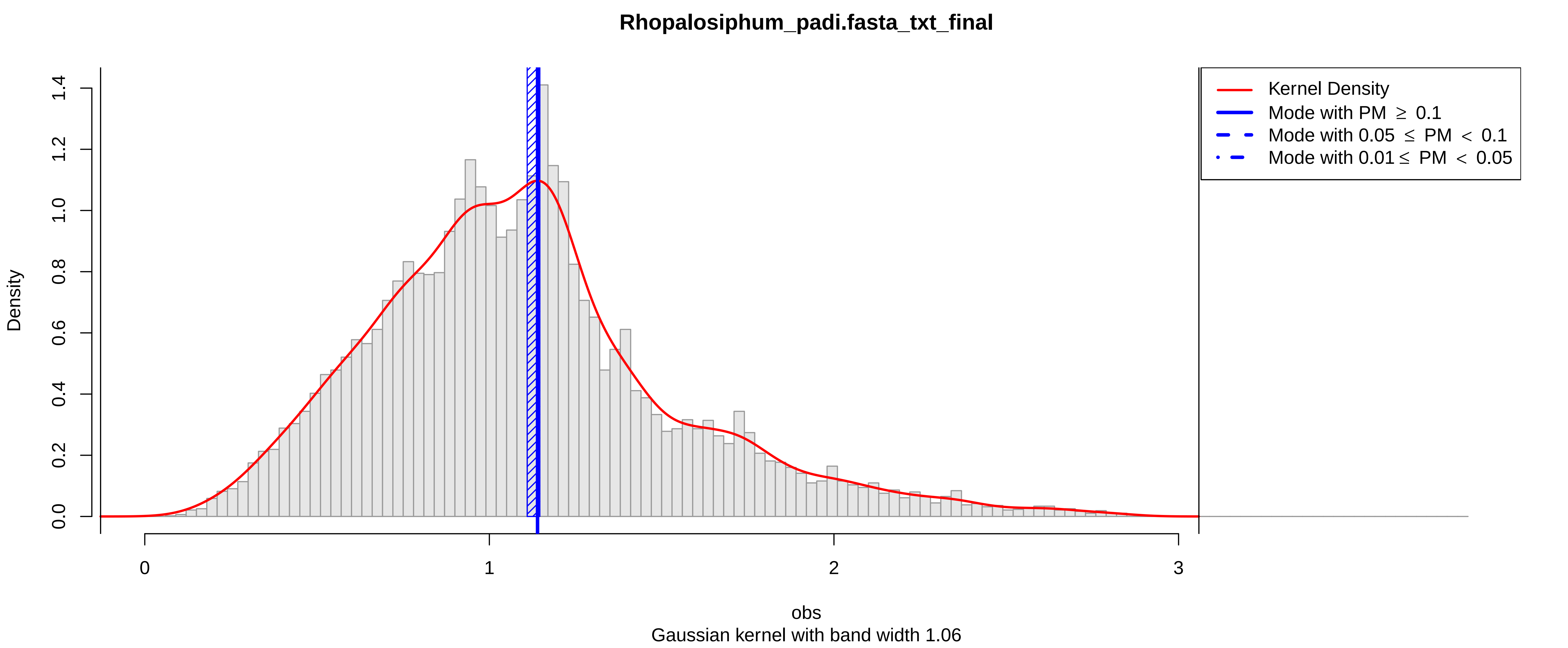

Rhopalosiphum_padi.fasta_txt_final
Density
0.0
0.2
0.4
0.6
0.8
1.0
1.2
1.4
0
1
2
3
obs
Gaussian kernel with band width 1.06
Kernel Density
Mode with PM ≥ 0.1
Mode with 0.05 ≤ PM < 0.1
Mode with 0.01 ≤ PM < 0.05

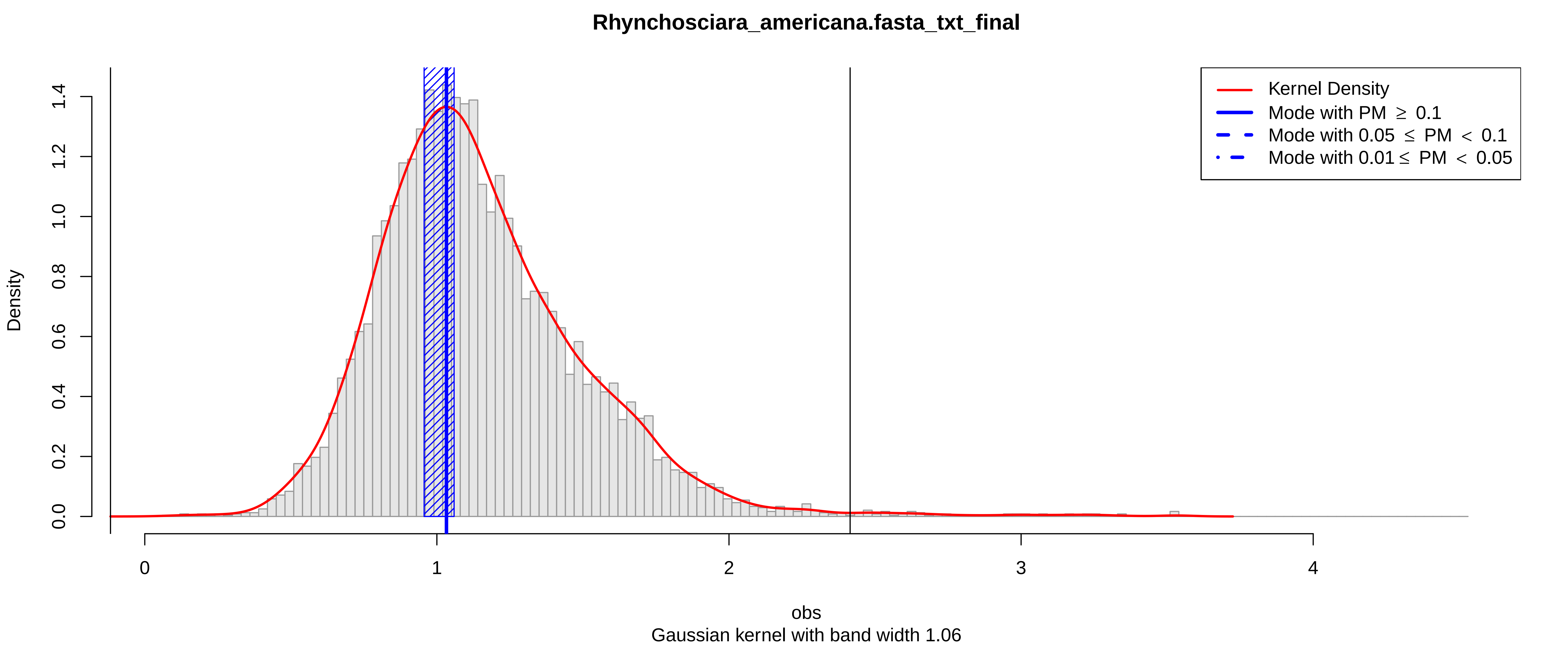
Rhynchosciara_americana.fasta_txt_final
Kernel Density
Mode with PM ≥ 0.1
Mode with 0.05 ≤ PM < 0.1
Mode with 0.01 ≤ PM < 0.05
Density
0.0
0.2
0.4
0.6
0.8
1.0
1.2
1.4
0
1
2
3
4
obs
Gaussian kernel with band width 1.06

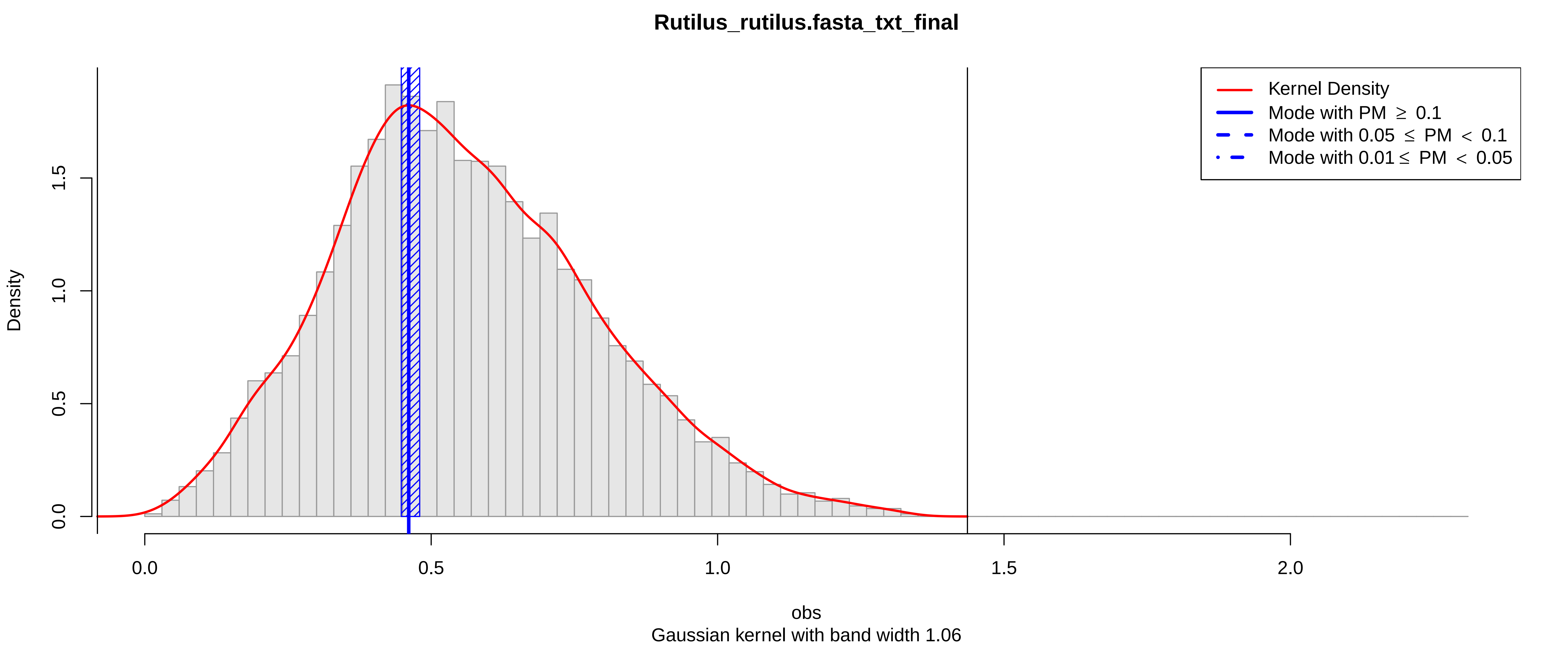

Rutilus_rutilus.fasta_txt_final
Kernel Density
Mode with PM ≥ 0.1
Mode with 0.05 ≤ PM < 0.1
Mode with 0.01 ≤ PM < 0.05
Density
0.0
0.5
1.0
1.5
2.0
obs
Gaussian kernel with band width 1.06

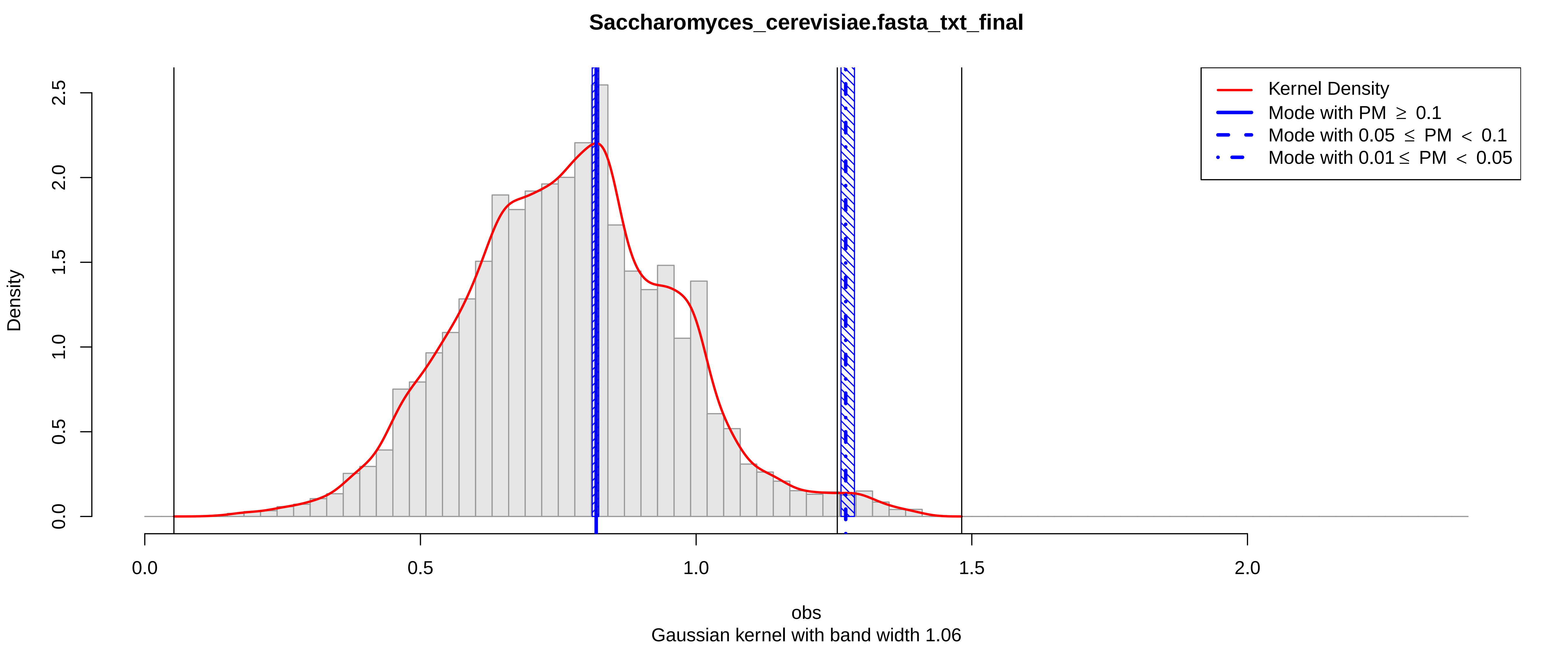
Saccharomyces_cerevisiae.fasta_txt_final
Kernel Density
Mode with PM ≥ 0.1
Mode with 0.05 ≤ PM < 0.1
Mode with 0.01 ≤ PM < 0.05
Density
0.0
0.5
1.0
1.5
2.0
2.5
0.0
0.5
1.0
1.5
2.0
obs
Gaussian kernel with band width 1.06

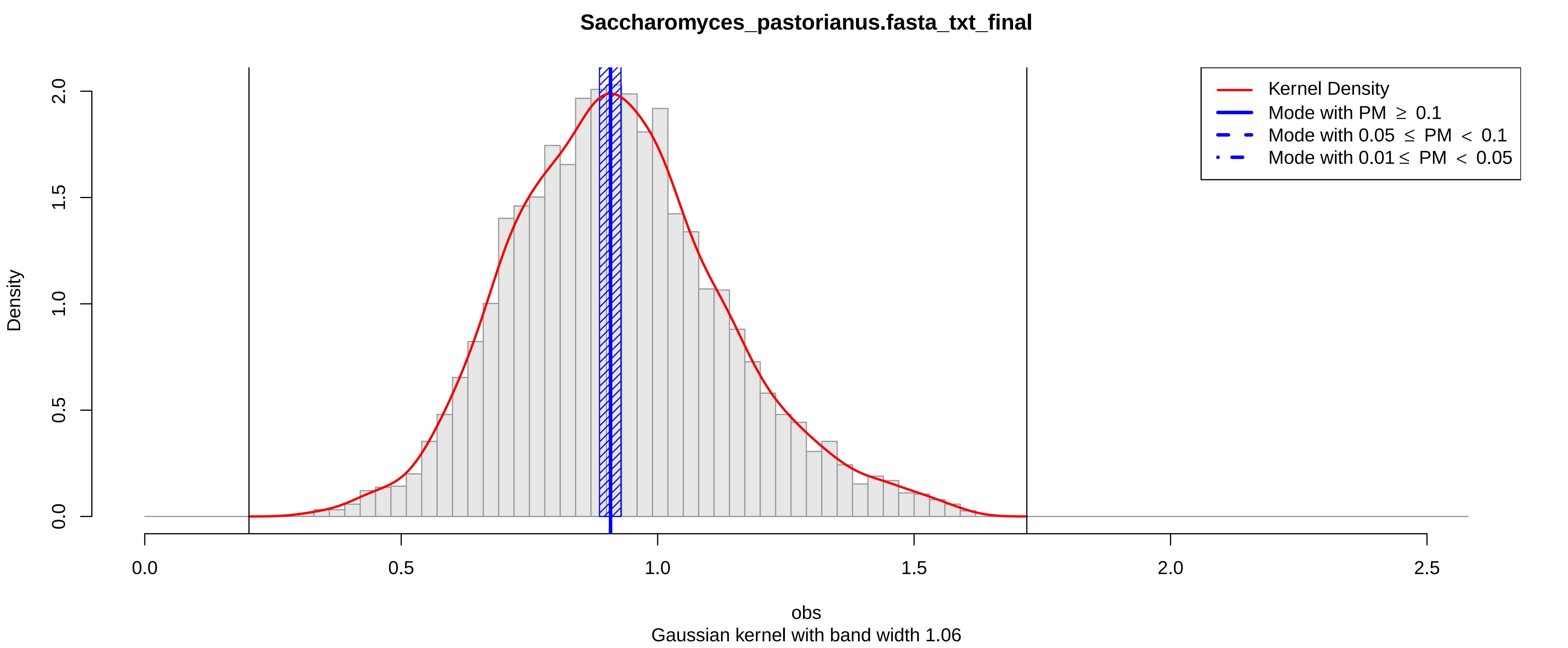

Saccharomyces_pastorianus.fasta_txt_final
Density
0.0
0.5
1.0
1.5
2.0
2.5
obs
Gaussian kernel with band width 1.06
Kernel Density
Mode with PM ≥ 0.1
Mode with 0.05 ≤ PM < 0.1
Mode with 0.01 ≤ PM < 0.05

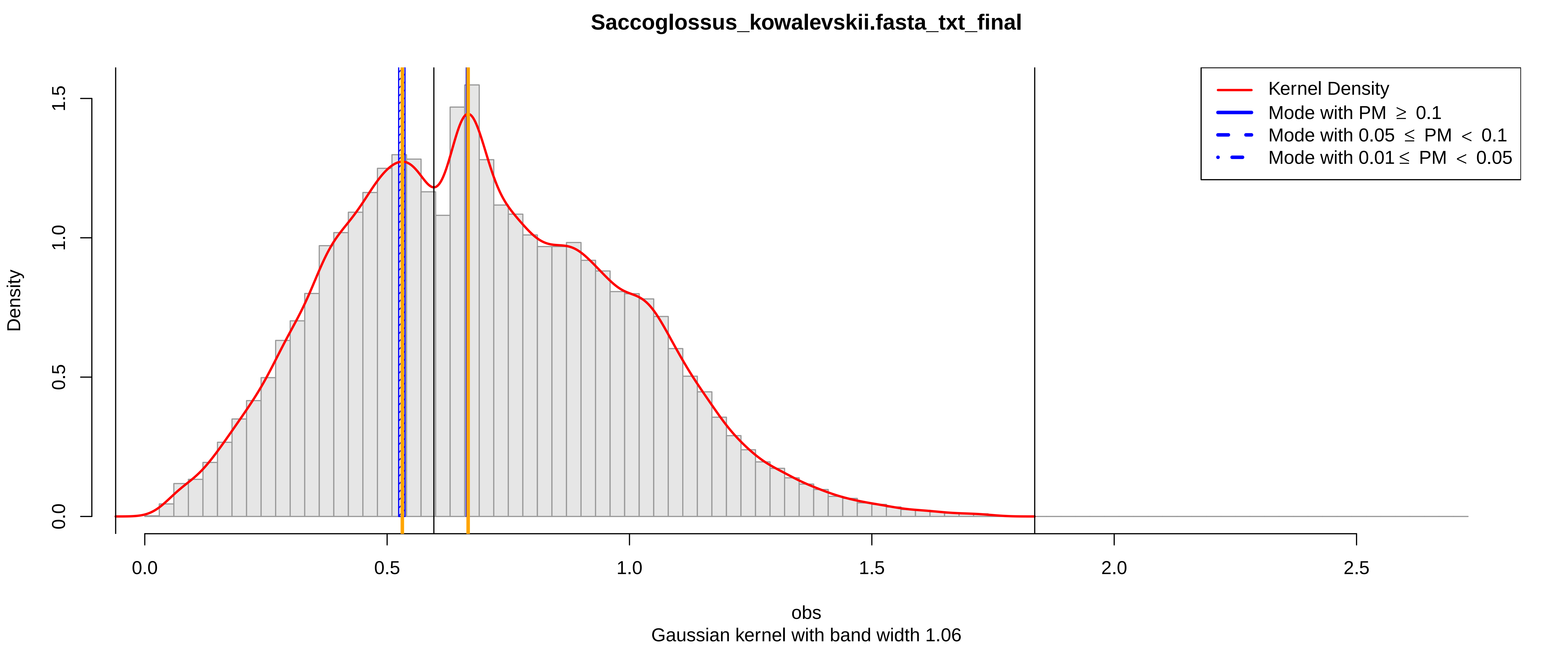
Saccoglossus_kowalevskii.fasta_txt_final
Kernel Density
Mode with PM ≥ 0.1
Mode with 0.05 ≤ PM < 0.1
Mode with 0.01 ≤ PM < 0.05
Density
0.0
0.5
1.0
1.5
0.0
0.5
1.0
1.5
2.0
2.5
obs
Gaussian kernel with band width 1.06

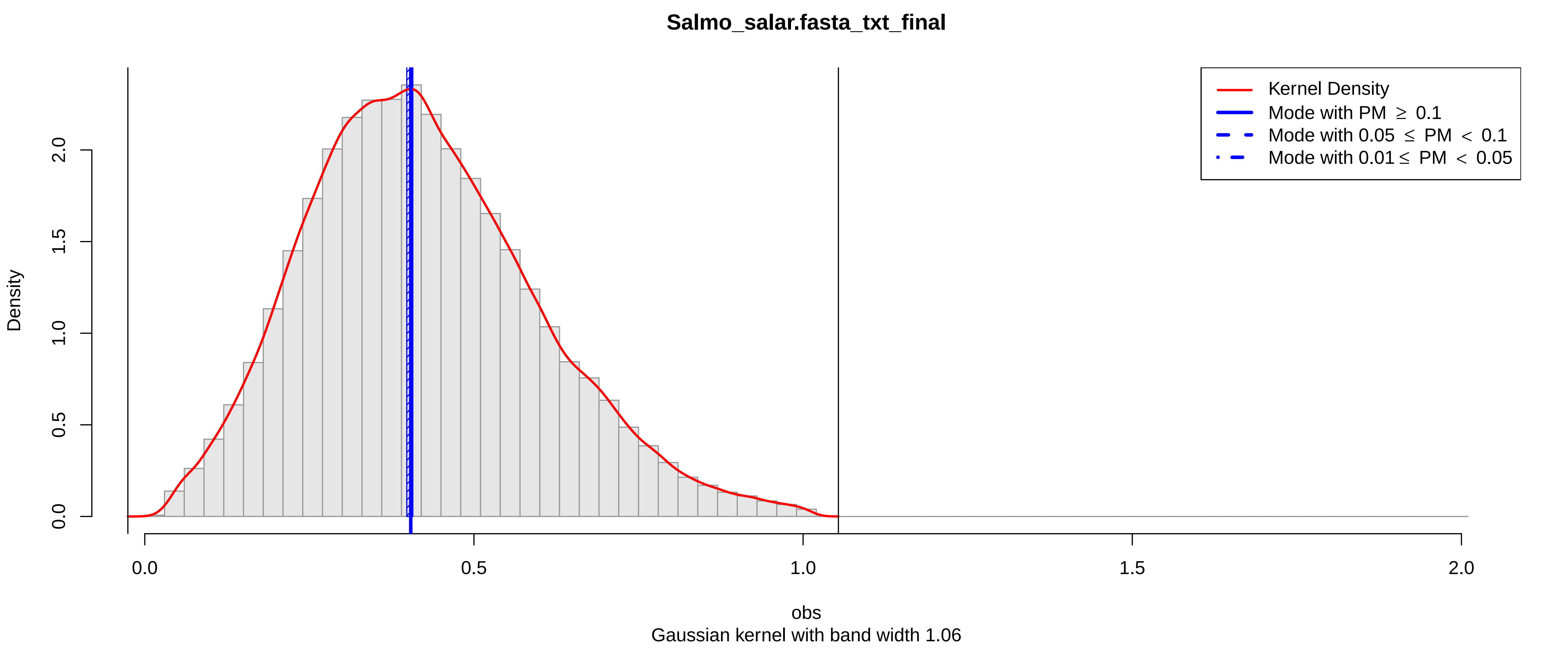
Salmo_salar.fasta_txt_final
Kernel Density
Mode with PM ≥ 0.1
Mode with 0.05 ≤ PM < 0.1
Mode with 0.01 ≤ PM < 0.05
Density
0.0
0.5
1.0
1.5
2.0
obs
Gaussian kernel with band width 1.06

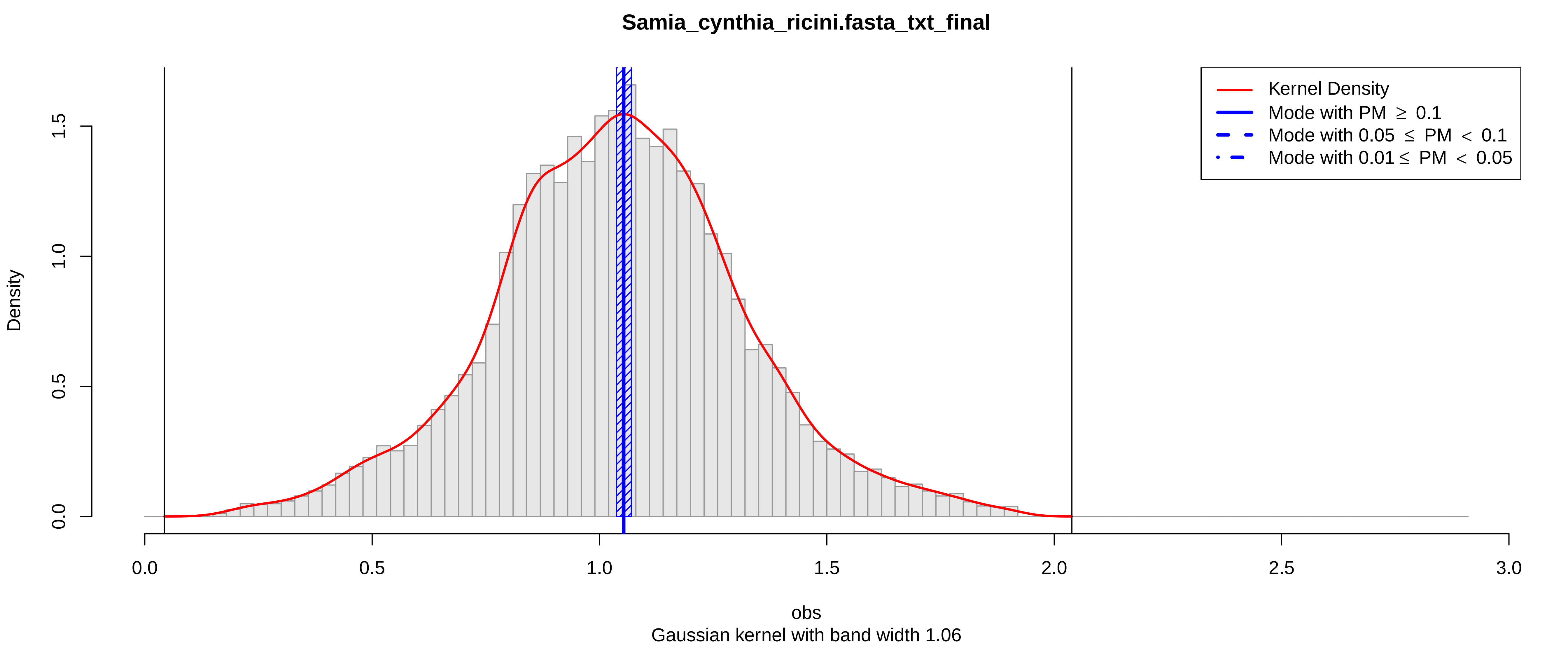

Samia_cynthia_ricini.fasta_txt_final
Kernel Density
Mode with PM ≥ 0.1
Mode with 0.05 ≤ PM < 0.1
Mode with 0.01 ≤ PM < 0.05
Density
0.0
0.5
1.0
1.5
0.0
0.5
1.0
1.5
2.0
2.5
3.0
obs
Gaussian kernel with band width 1.06

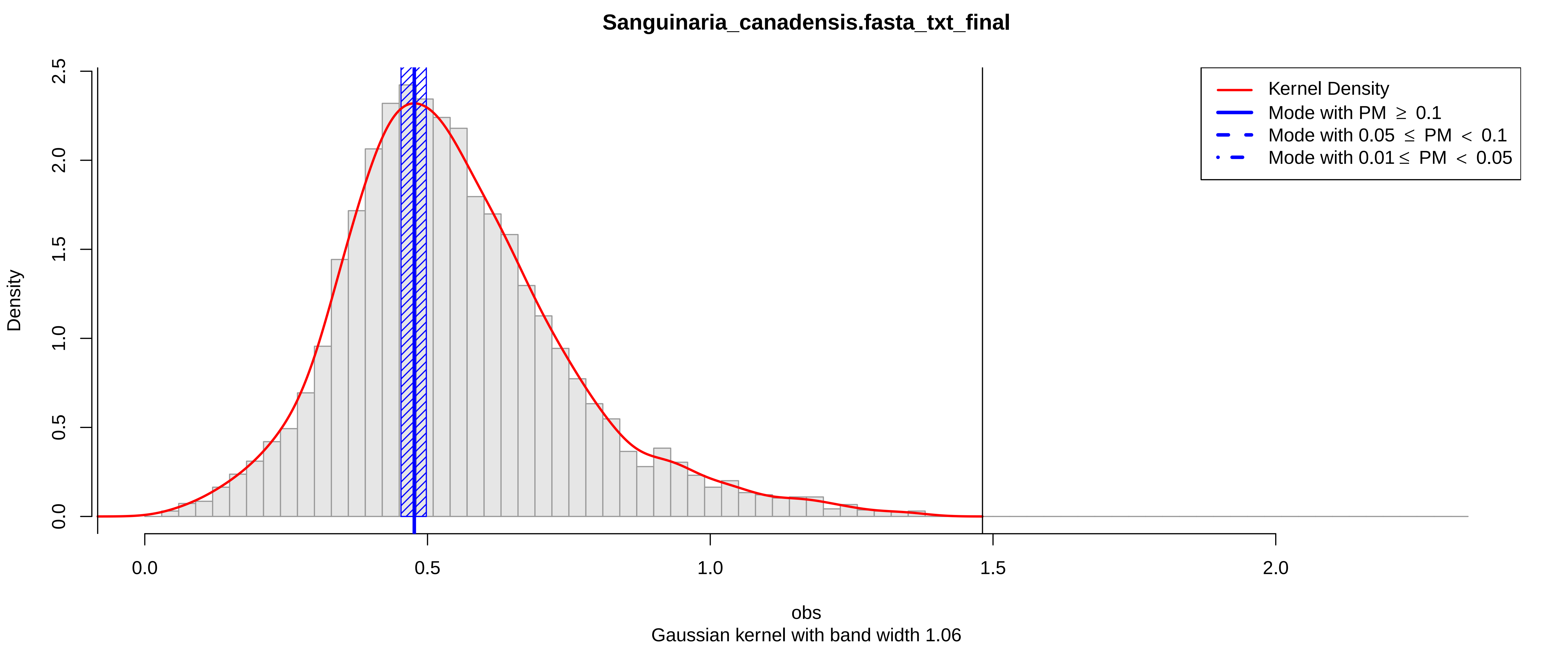
Sanguinaria_canadensis.fasta_txt_final
Kernel Density
Mode with PM ≥ 0.1
Mode with 0.05 ≤ PM < 0.1
Mode with 0.01 ≤ PM < 0.05
Density
0.0
0.5
1.0
1.5
2.0
2.5
0.0
0.5
1.0
1.5
2.0
obs
Gaussian kernel with band width 1.06

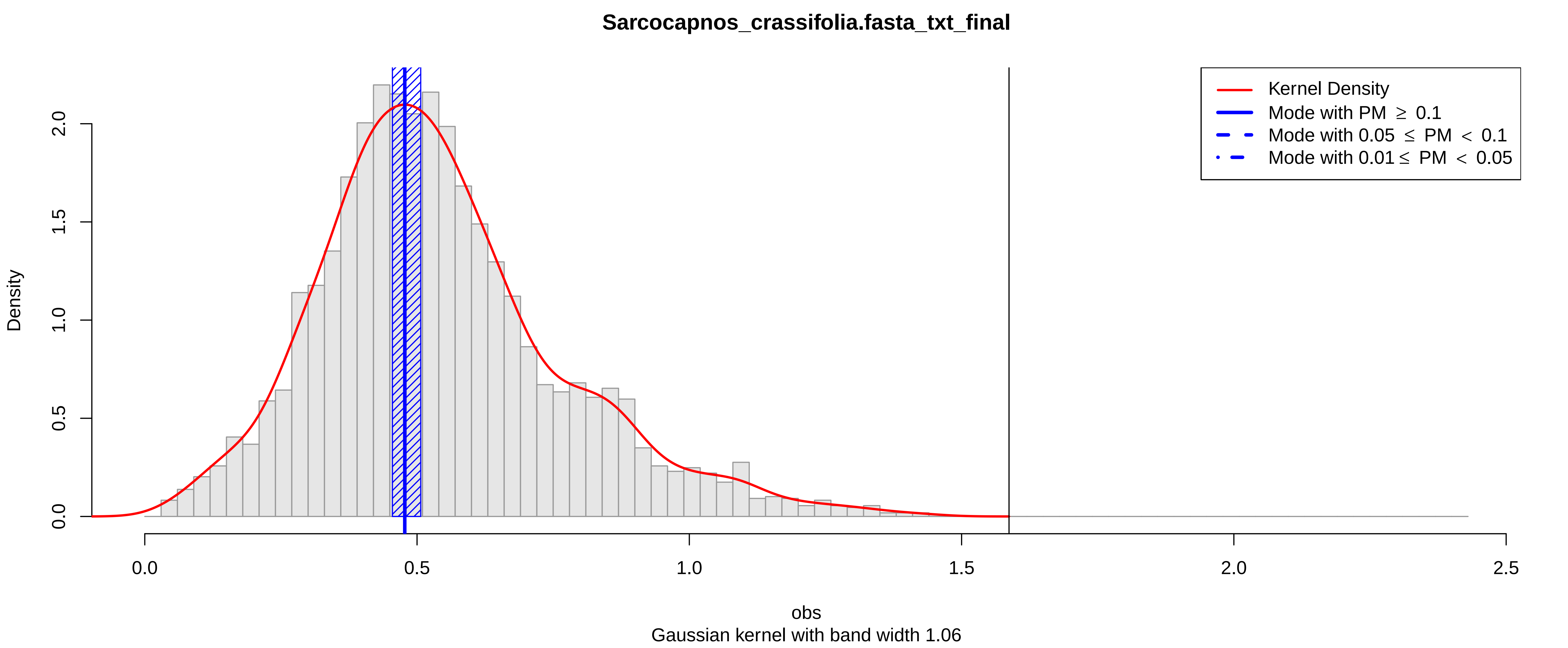
Sarcocapnos_crassifolia.fasta_txt_final
Density
obs
Gaussian kernel with band width 1.06
Kernel Density
Mode with PM ≥ 0.1
Mode with 0.05 ≤ PM < 0.1
Mode with 0.01 ≤ PM < 0.05
0.0
0.5
1.0
1.5
2.0
2.5

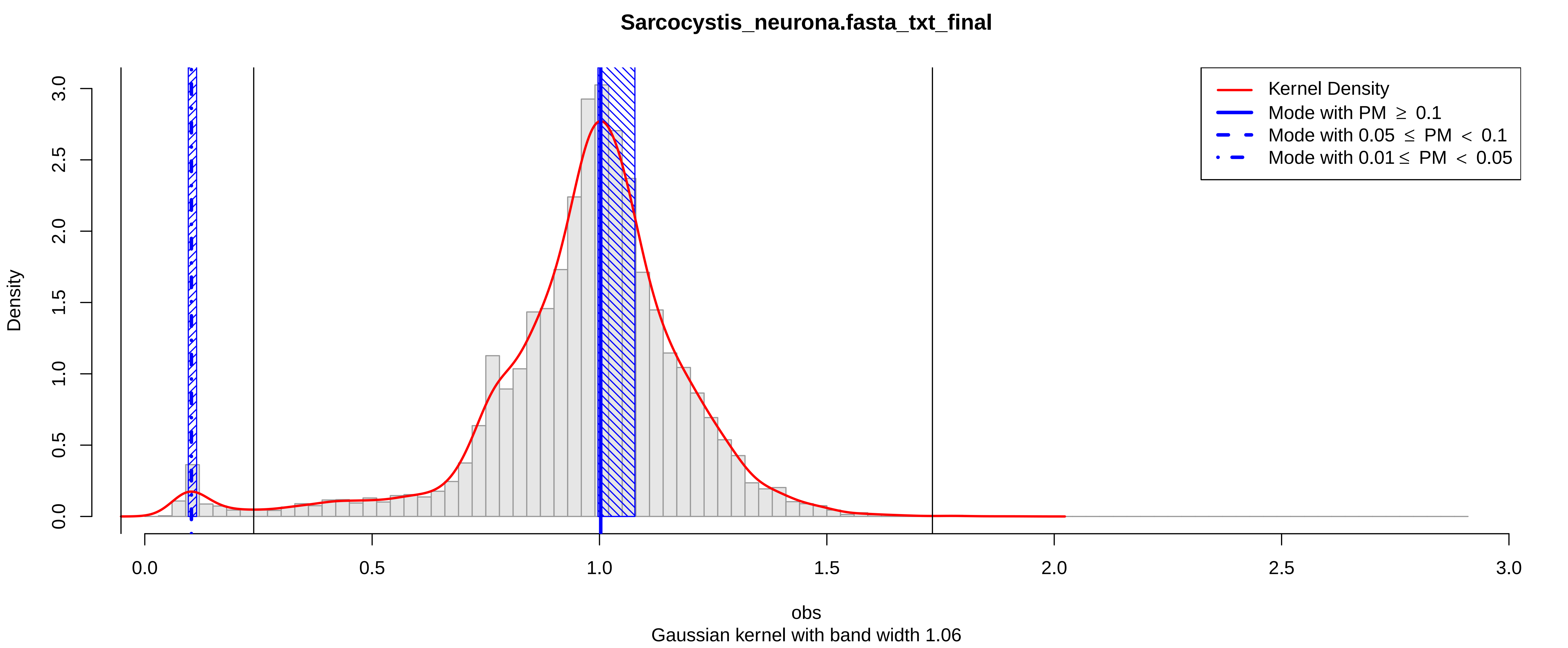
Sarcocystis_neurona.fasta_txt_final
Density
obs
Gaussian kernel with band width 1.06
Kernel Density
Mode with PM ≥ 0.1
Mode with 0.05 ≤ PM < 0.1
Mode with 0.01 ≤ PM < 0.05
0.0
0.5
1.0
1.5
2.0
2.5
3.0

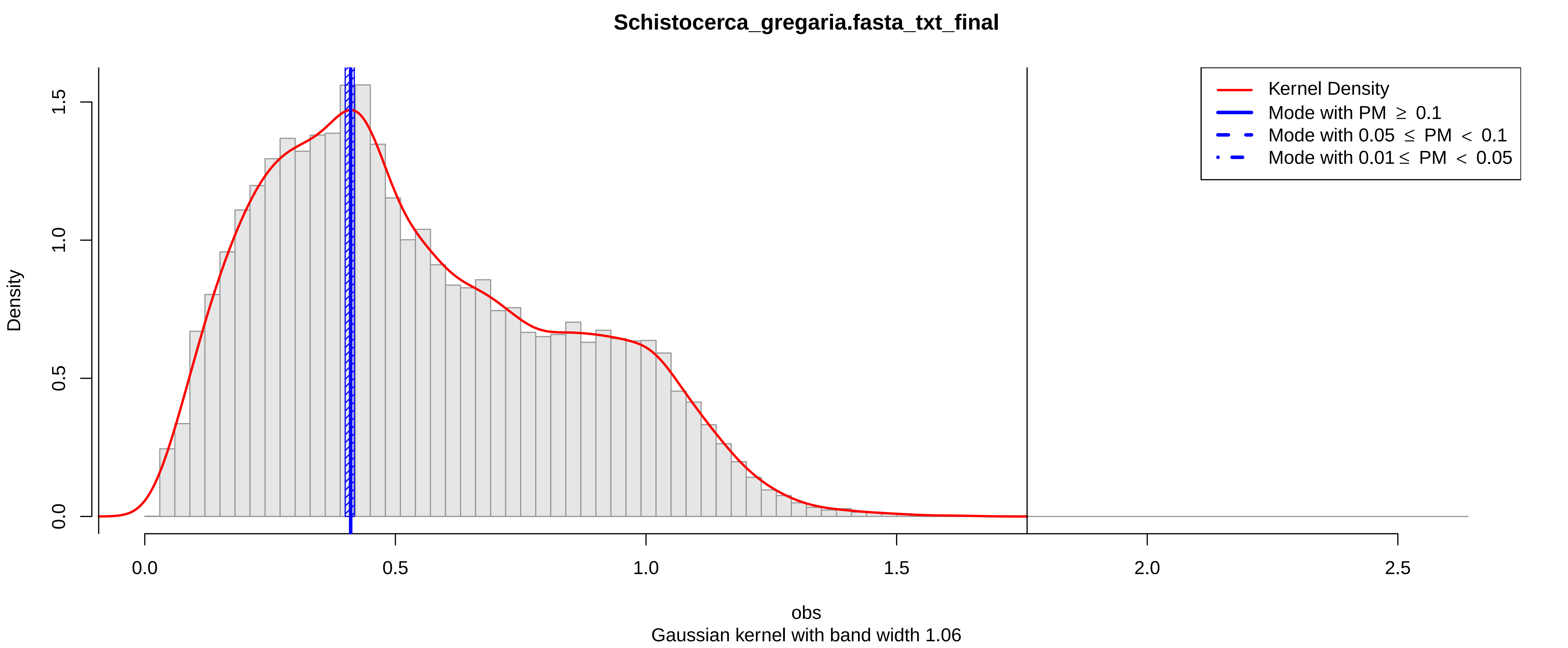

Schistocerca_gregaria.fasta_txt_final
Kernel Density
Mode with PM ≥ 0.1
Mode with 0.05 ≤ PM < 0.1
Mode with 0.01 ≤ PM < 0.05
Density
0.0
0.5
1.0
1.5
0.0
0.5
1.0
1.5
2.0
2.5
obs
Gaussian kernel with band width 1.06

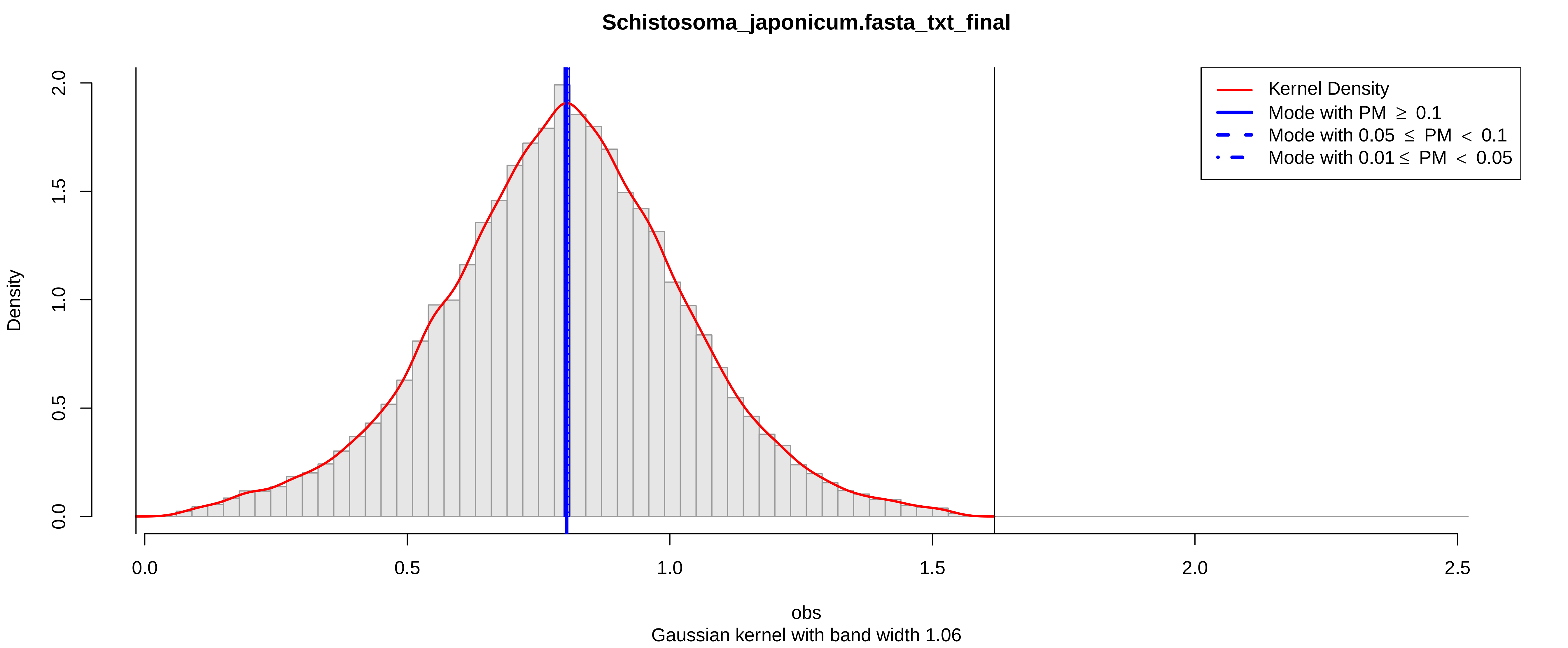

Schistosoma_japonicum.fasta_txt_final
Kernel Density
Mode with PM ≥ 0.1
Mode with 0.05 ≤ PM < 0.1
Mode with 0.01 ≤ PM < 0.05
Density
0.0
0.5
1.0
1.5
2.0
0.0
0.5
1.0
1.5
2.0
2.5
obs
Gaussian kernel with band width 1.06

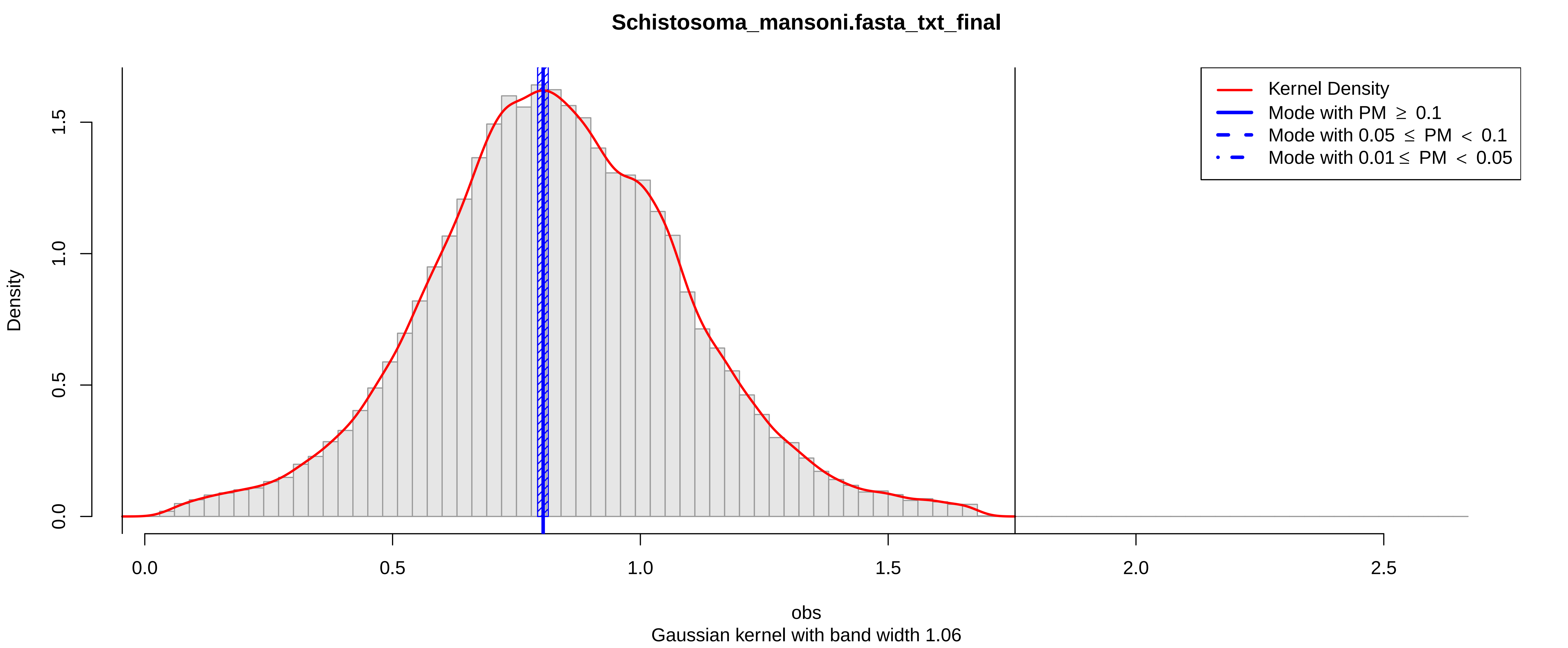

Schistosoma_mansoni.fasta_txt_final
Kernel Density
Mode with PM ≥ 0.1
Mode with 0.05 ≤ PM < 0.1
Mode with 0.01 ≤ PM < 0.05
Density
0.0
0.5
1.0
1.5
0.0
0.5
1.0
1.5
2.0
2.5
obs
Gaussian kernel with band width 1.06

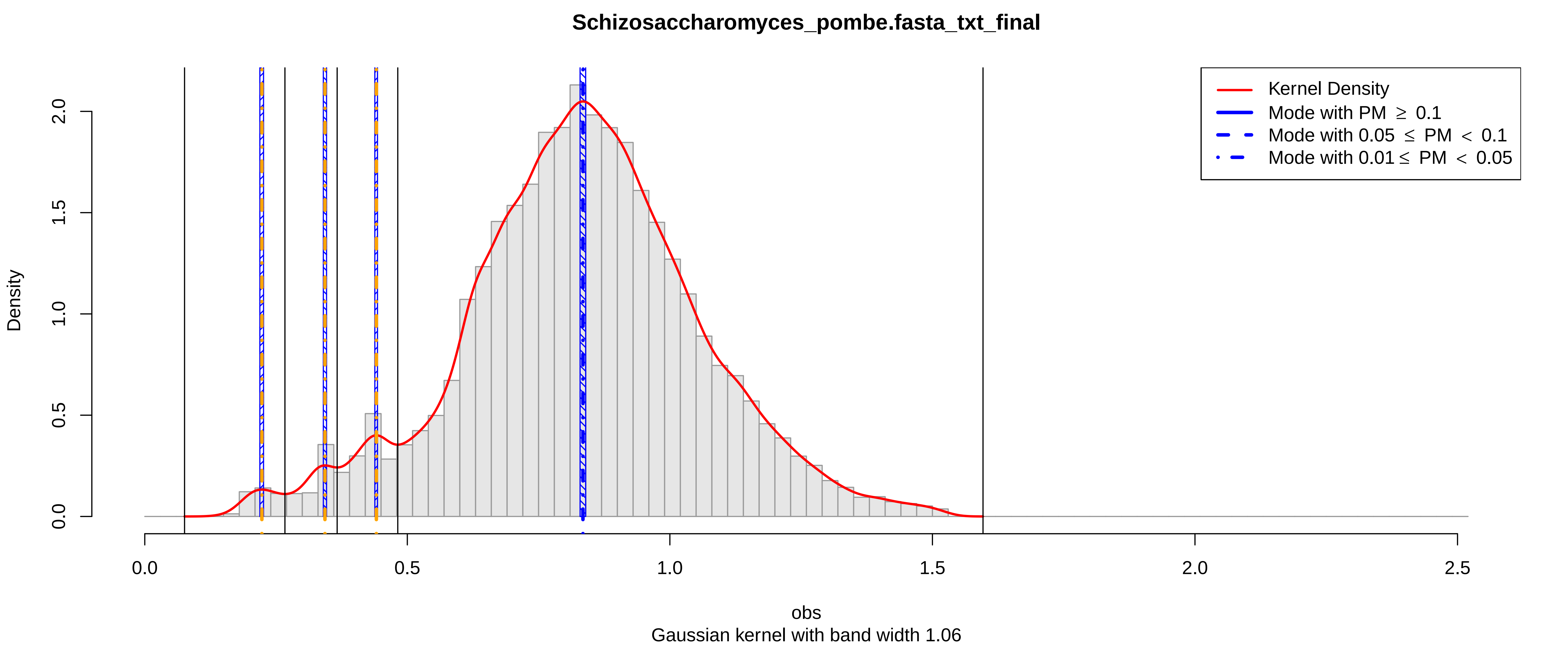

Schizosaccharomyces_pombe.fasta_txt_final
Kernel Density
Mode with PM ≥ 0.1
Mode with 0.05 ≤ PM < 0.1
Mode with 0.01 ≤ PM < 0.05
Density
0.0
0.5
1.0
1.5
2.0
0.0
0.5
1.0
1.5
2.0
2.5
obs
Gaussian kernel with band width 1.06

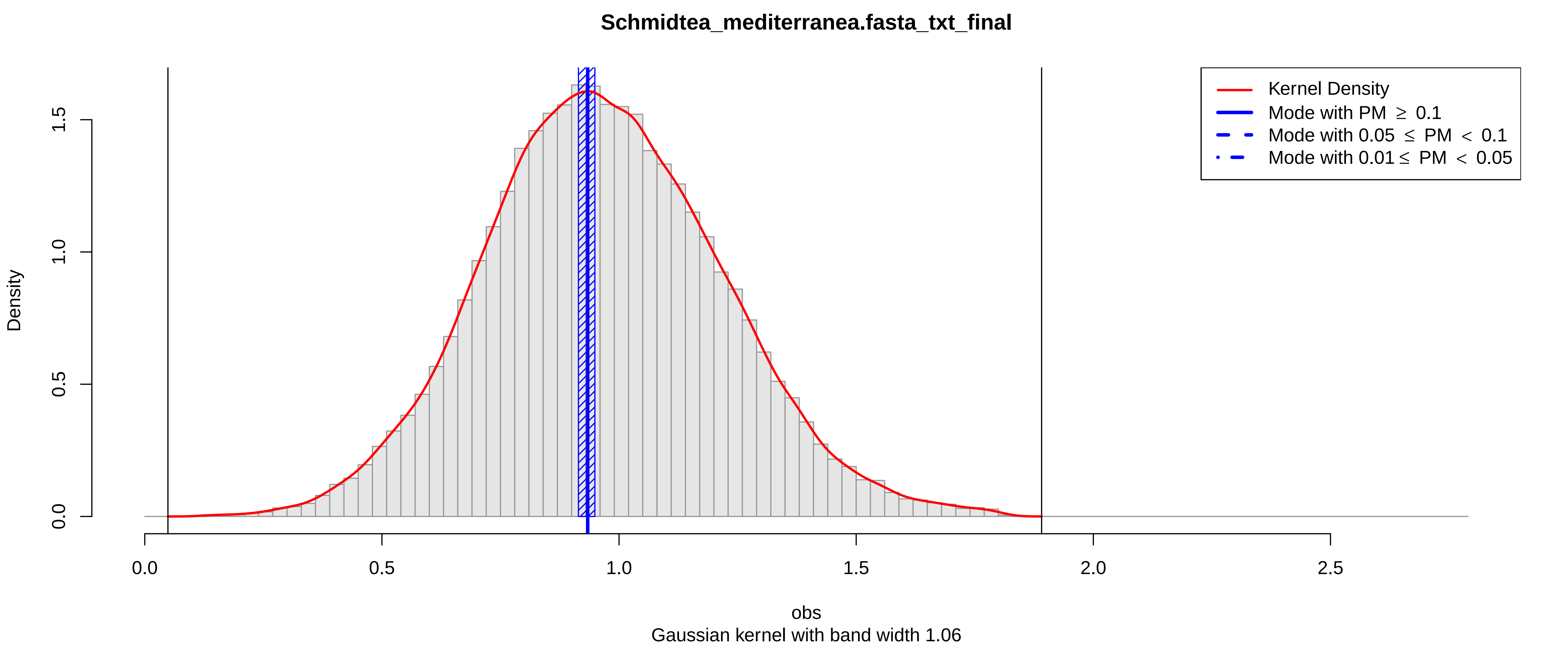

Schmidtea_mediterranea.fasta_txt_final
Density
0.0
0.5
1.0
1.5
0.0
0.5
1.0
1.5
2.0
2.5
obs
Gaussian kernel with band width 1.06
Kernel Density
Mode with PM ≥ 0.1
Mode with 0.05 ≤ PM < 0.1
Mode with 0.01 ≤ PM < 0.05

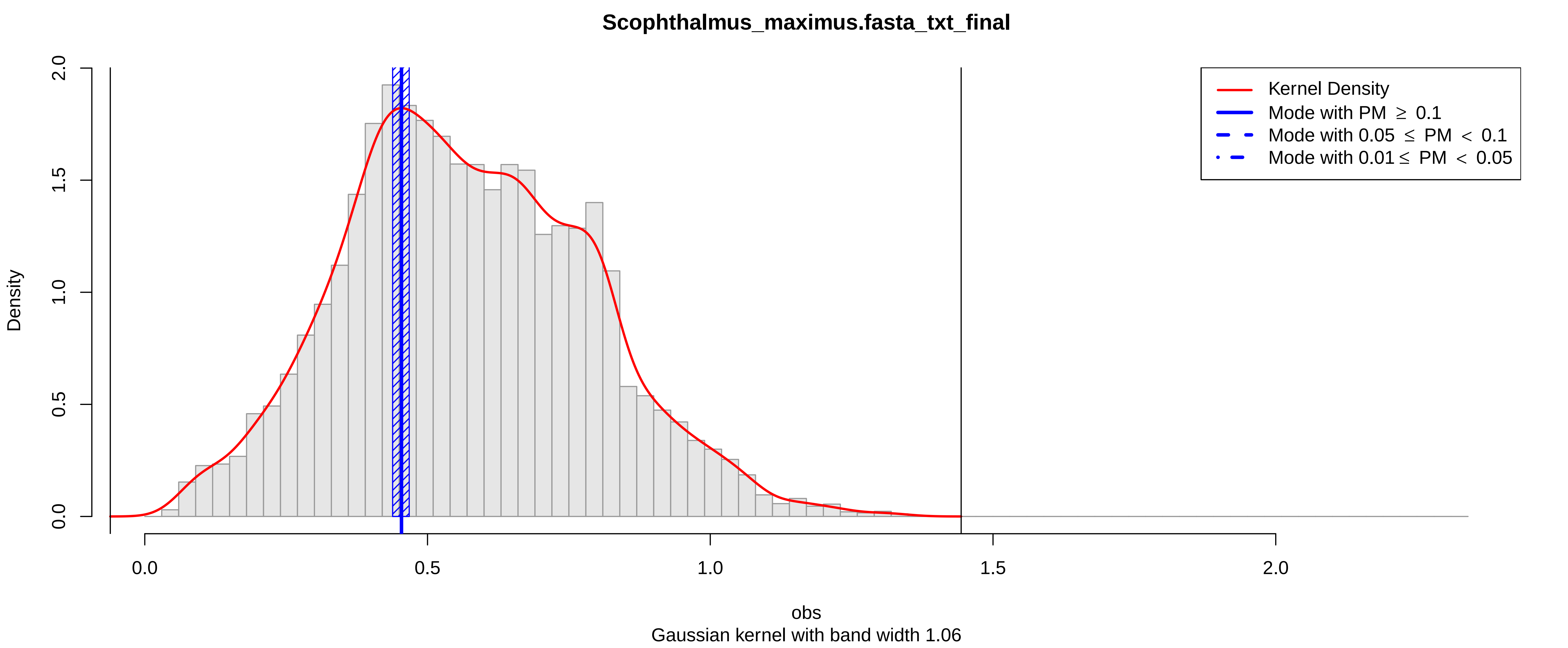
Scophthalmus_maximus.fasta_txt_final
Density
obs
Gaussian kernel with band width 1.06
Kernel Density
Mode with PM ≥ 0.1
Mode with 0.05 ≤ PM < 0.1
Mode with 0.01 ≤ PM < 0.05
0.0
0.5
1.0
1.5
2.0

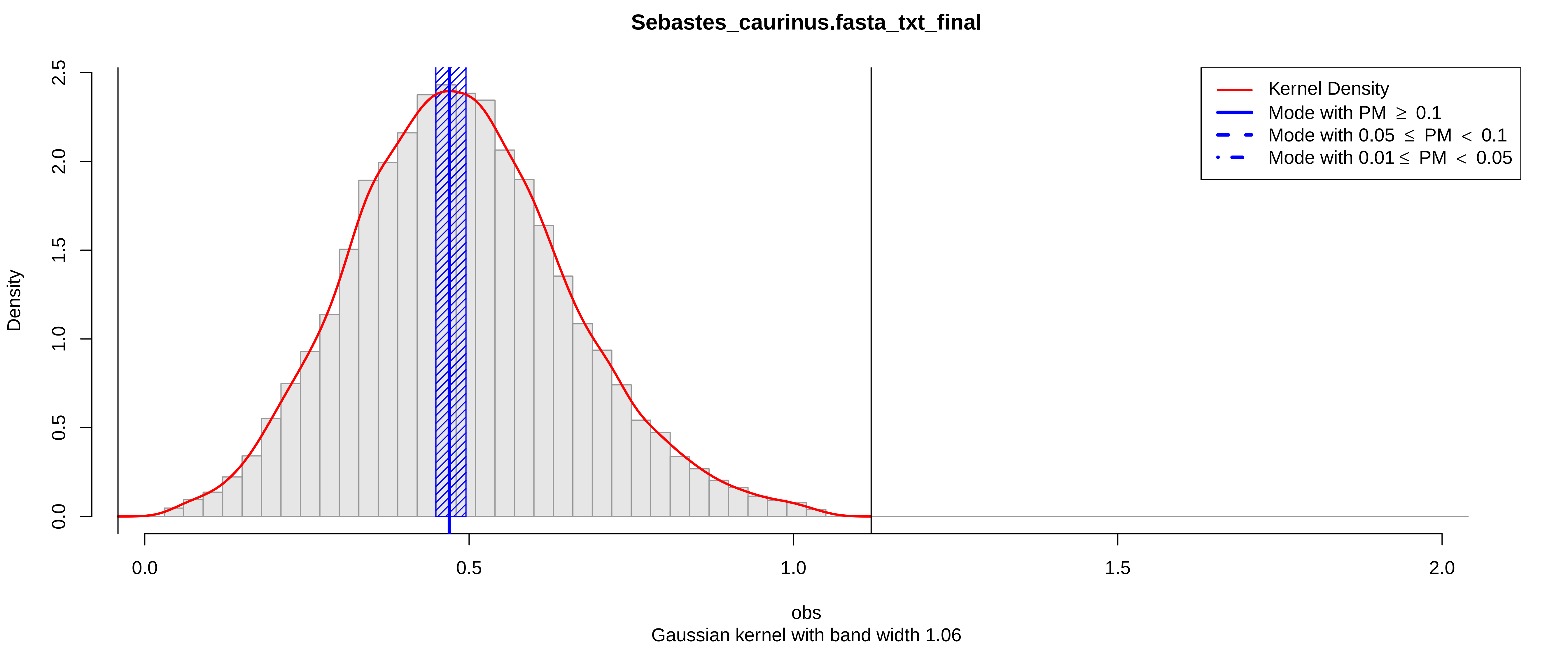

Sebastes_caurinus.fasta_txt_final
Density
0.0
0.5
1.0
1.5
2.0
2.5
0.0
0.5
1.0
1.5
2.0
obs
Gaussian kernel with band width 1.06
Kernel Density
Mode with PM ≥ 0.1
Mode with 0.05 ≤ PM < 0.1
Mode with 0.01 ≤ PM < 0.05

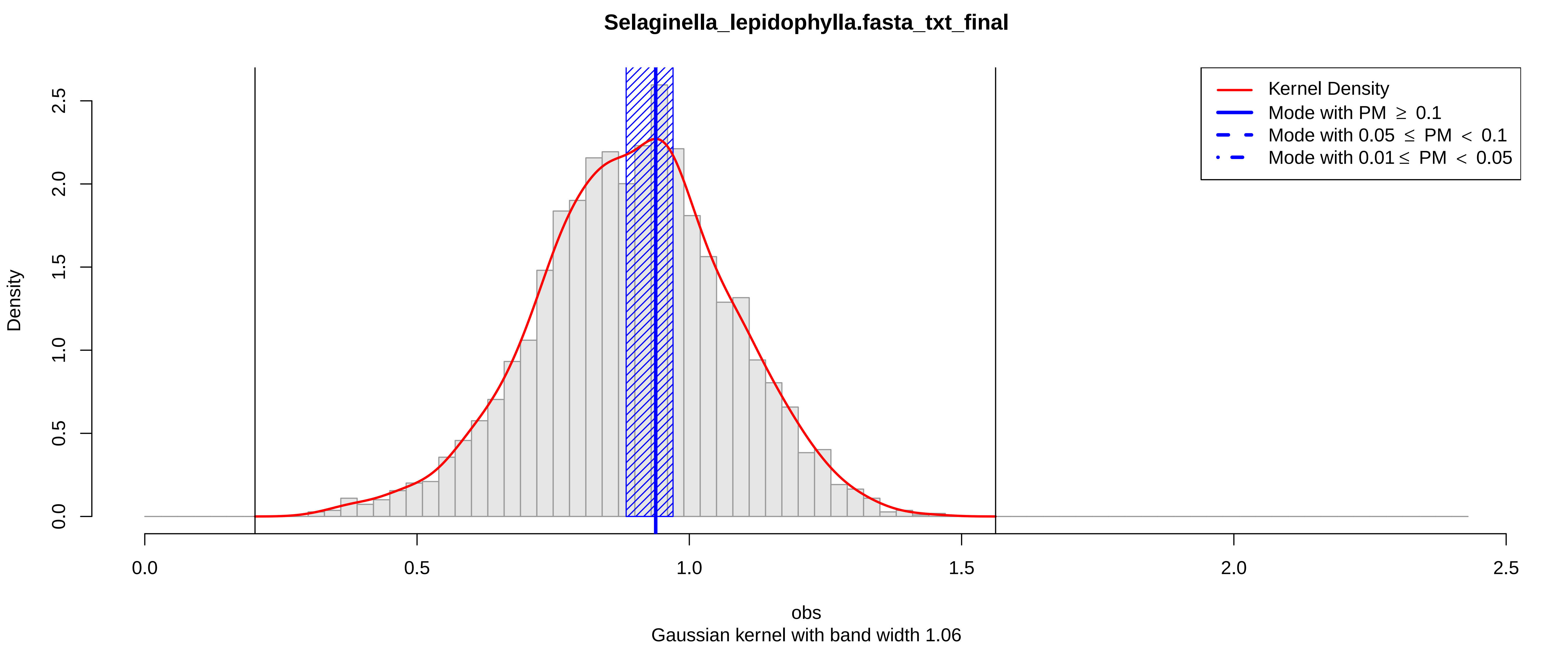
Selaginella_lepidophylla.fasta_txt_final
Kernel Density
Mode with PM ≥ 0.1
Mode with 0.05 ≤ PM < 0.1
Mode with 0.01 ≤ PM < 0.05
Density
0.0
0.5
1.0
1.5
2.0
2.5
obs
Gaussian kernel with band width 1.06

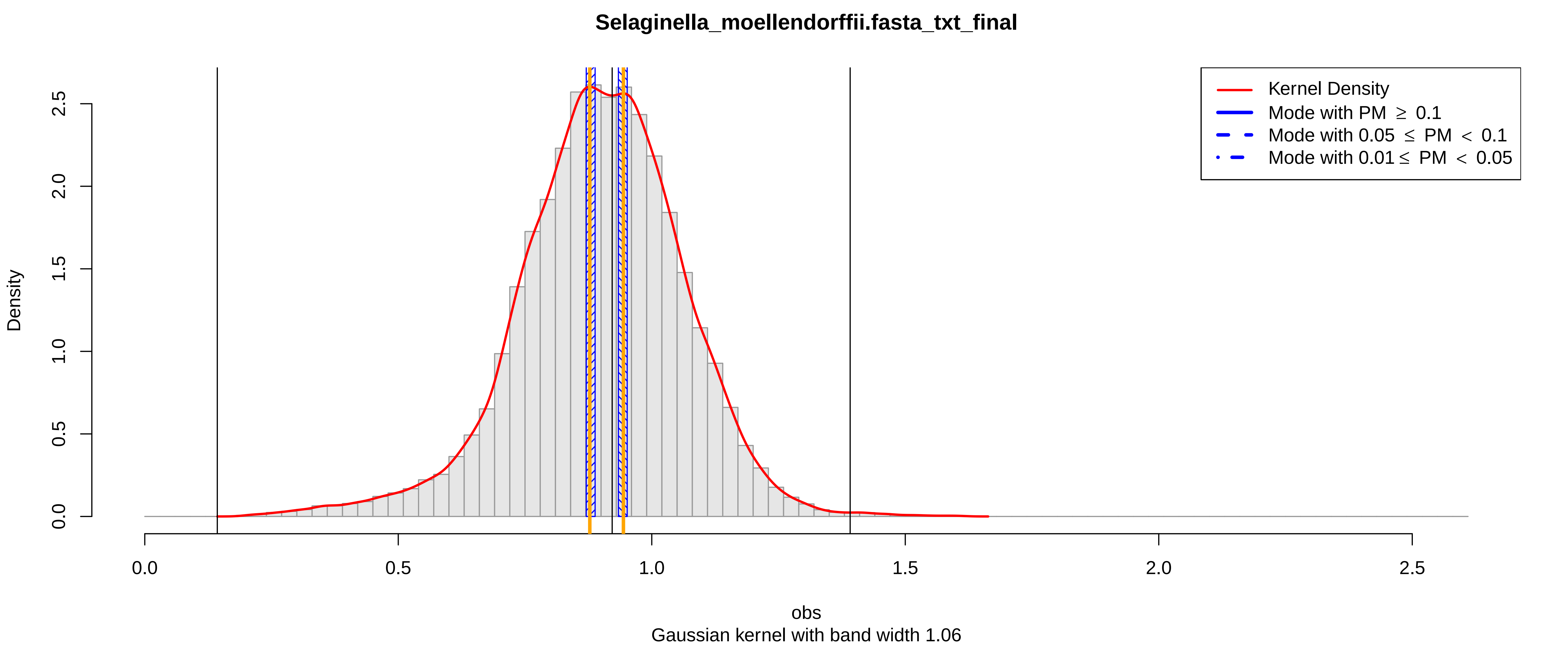

Selaginella_moellendorffii.fasta_txt_final
Kernel Density
Mode with PM ≥ 0.1
Mode with 0.05 ≤ PM < 0.1
Mode with 0.01 ≤ PM < 0.05
Density
0.0
0.5
1.0
1.5
2.0
2.5
obs
Gaussian kernel with band width 1.06

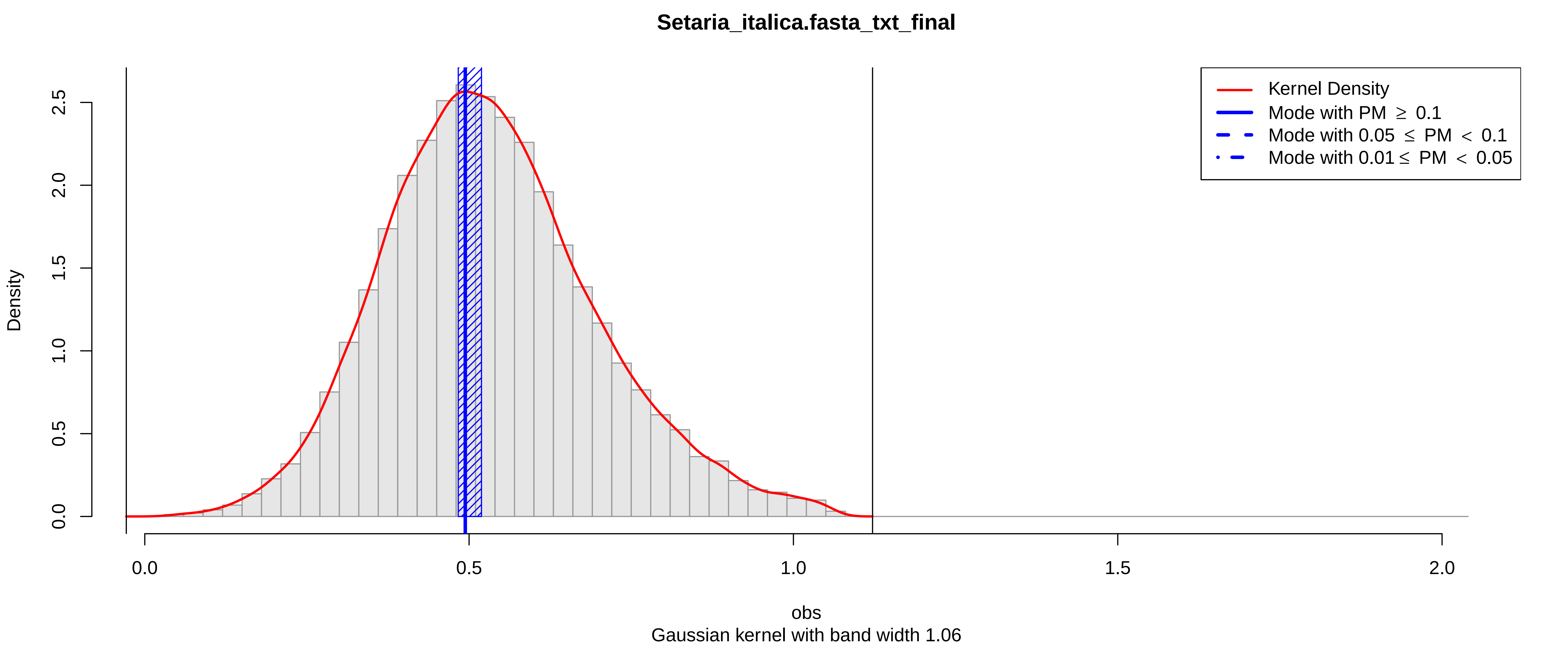

Setaria_italica.fasta_txt_final
Kernel Density
Mode with PM ≥ 0.1
Mode with 0.05 ≤ PM < 0.1
Mode with 0.01 ≤ PM < 0.05
Density
0.0
0.5
1.0
1.5
2.0
2.5
0.0
0.5
1.0
1.5
2.0
obs
Gaussian kernel with band width 1.06

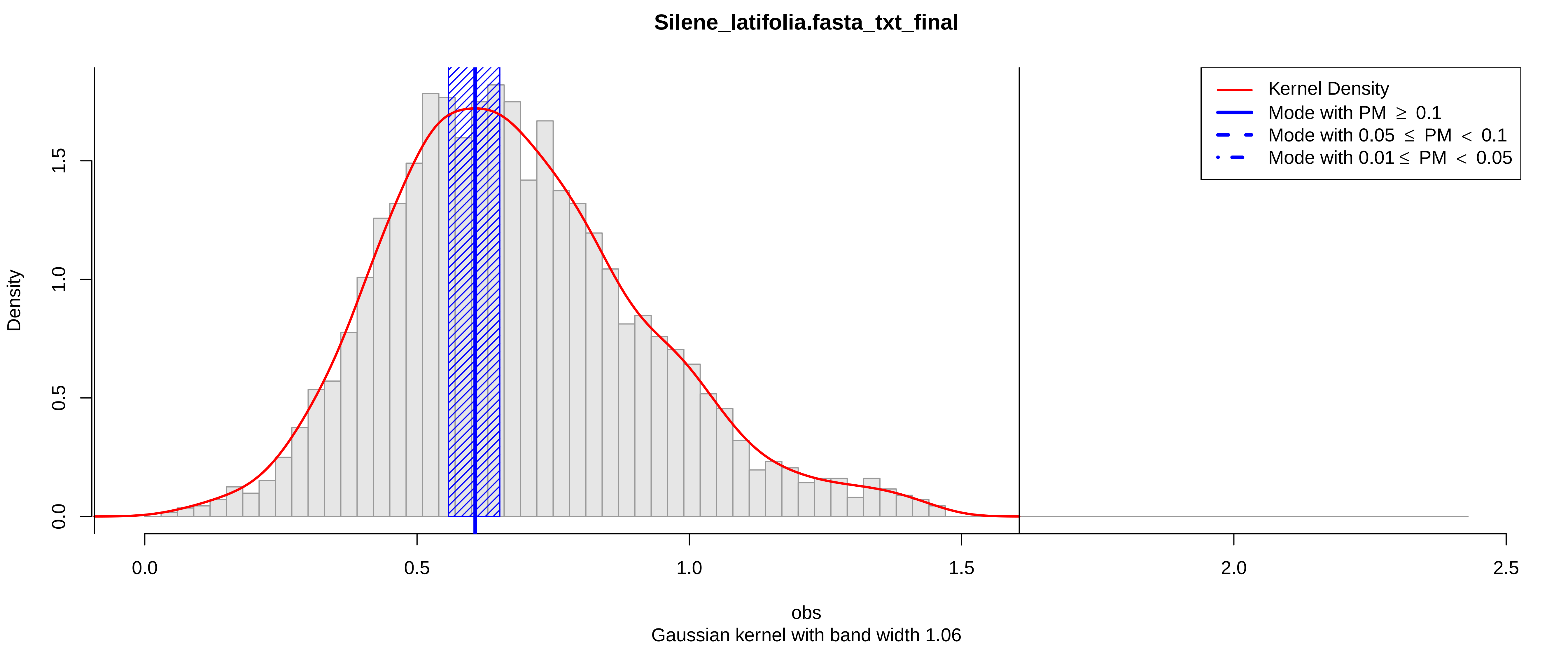

Silene_latifolia.fasta_txt_final
Kernel Density
Mode with PM ≥ 0.1
Mode with 0.05 ≤ PM < 0.1
Mode with 0.01 ≤ PM < 0.05
Density
0.0
0.5
1.0
1.5
0.0
0.5
1.0
1.5
2.0
2.5
obs
Gaussian kernel with band width 1.06

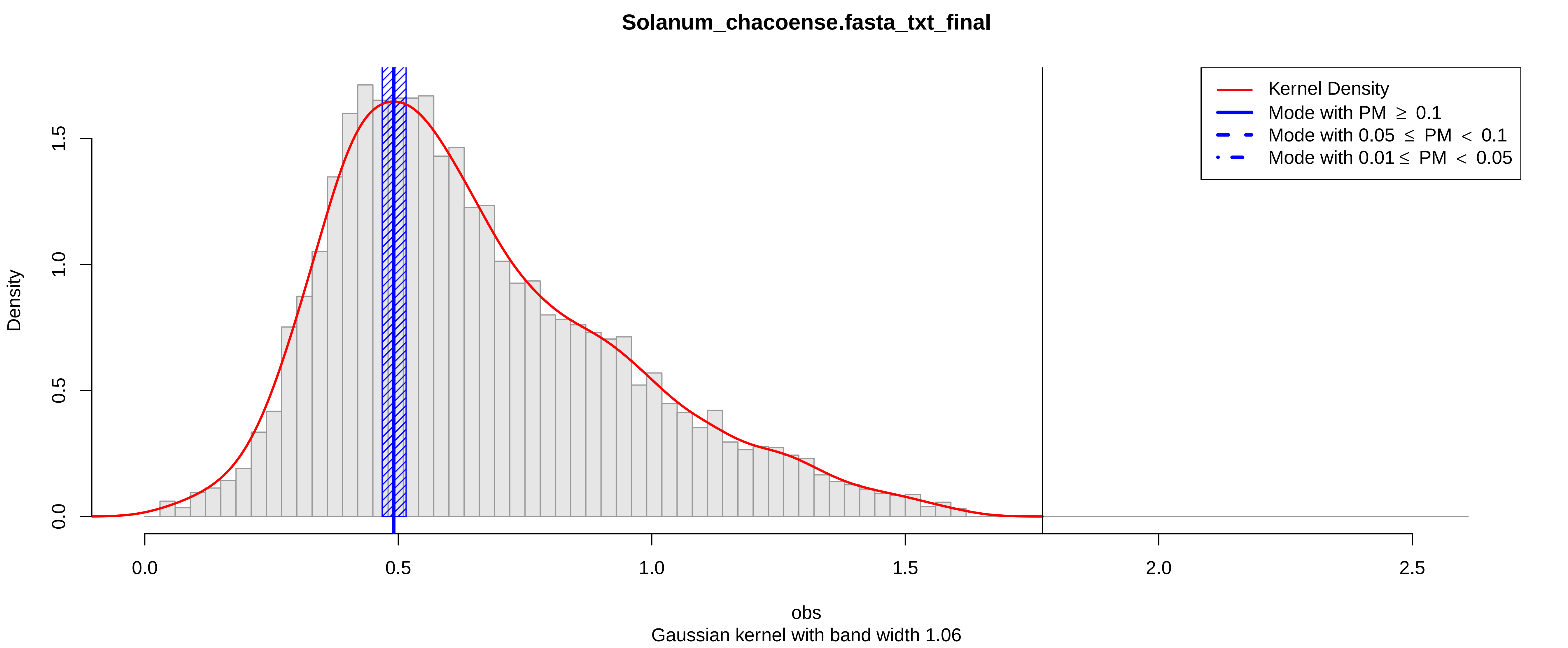

Solanum_chacoense.fasta_txt_final
Kernel Density
Mode with PM ≥ 0.1
Mode with 0.05 ≤ PM < 0.1
Mode with 0.01 ≤ PM < 0.05
Density
0.0
0.5
1.0
1.5
0.0
0.5
1.0
1.5
2.0
2.5
obs
Gaussian kernel with band width 1.06

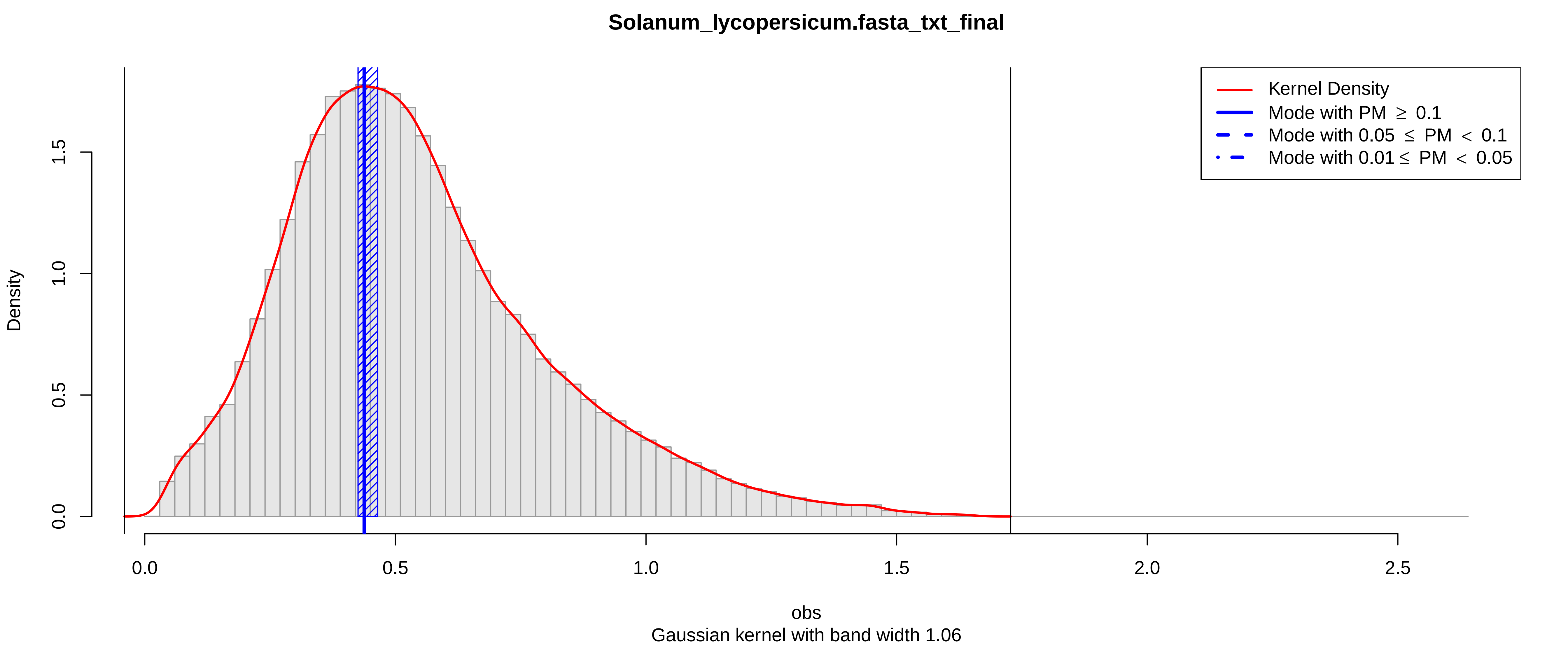

Solanum_lycopersicum.fasta_txt_final
Kernel Density
Mode with PM ≥ 0.1
Mode with 0.05 ≤ PM < 0.1
Mode with 0.01 ≤ PM < 0.05
Density
0.0
0.5
1.0
1.5
0.0
0.5
1.0
1.5
2.0
2.5
obs
Gaussian kernel with band width 1.06

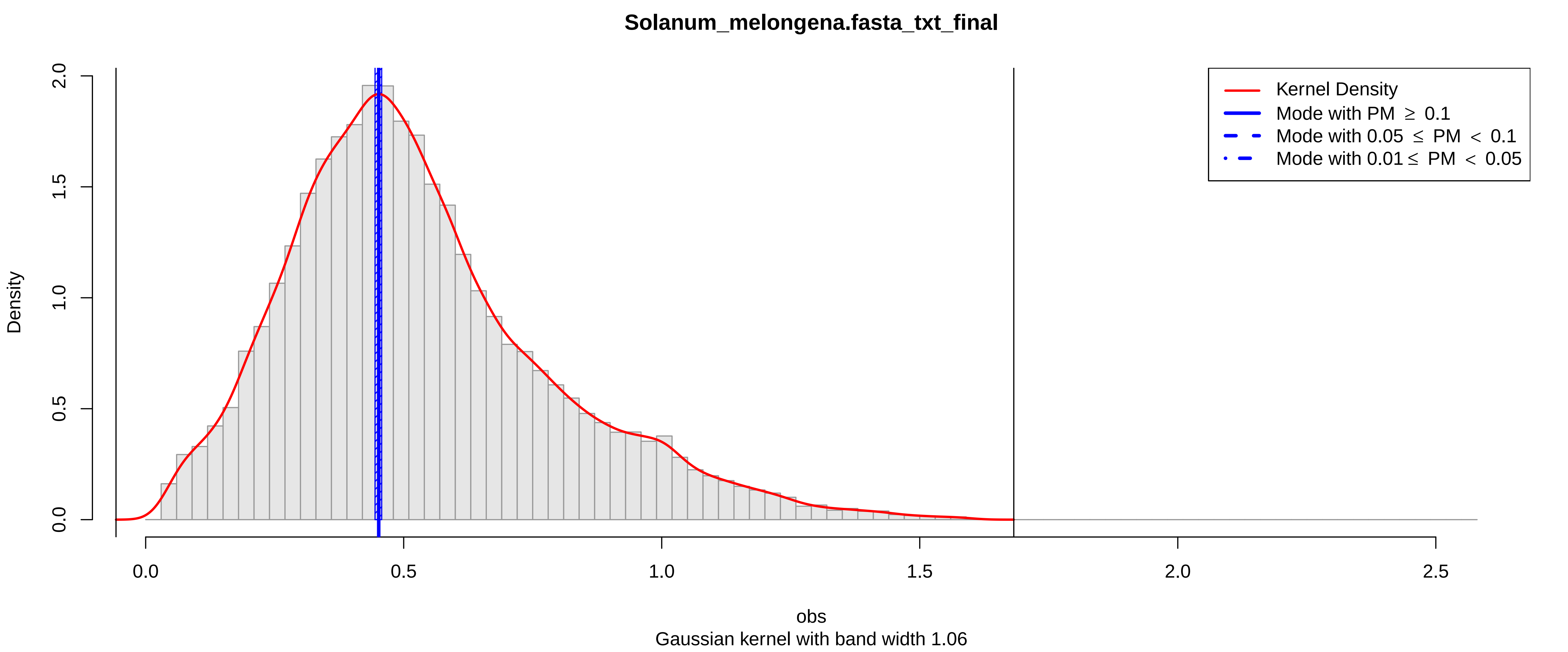
Solanum_melongena.fasta_txt_final
Kernel Density
Mode with PM ≥ 0.1
Mode with 0.05 ≤ PM < 0.1
Mode with 0.01 ≤ PM < 0.05
Density
0.0
0.5
1.0
1.5
2.0
2.5
obs
Gaussian kernel with band width 1.06

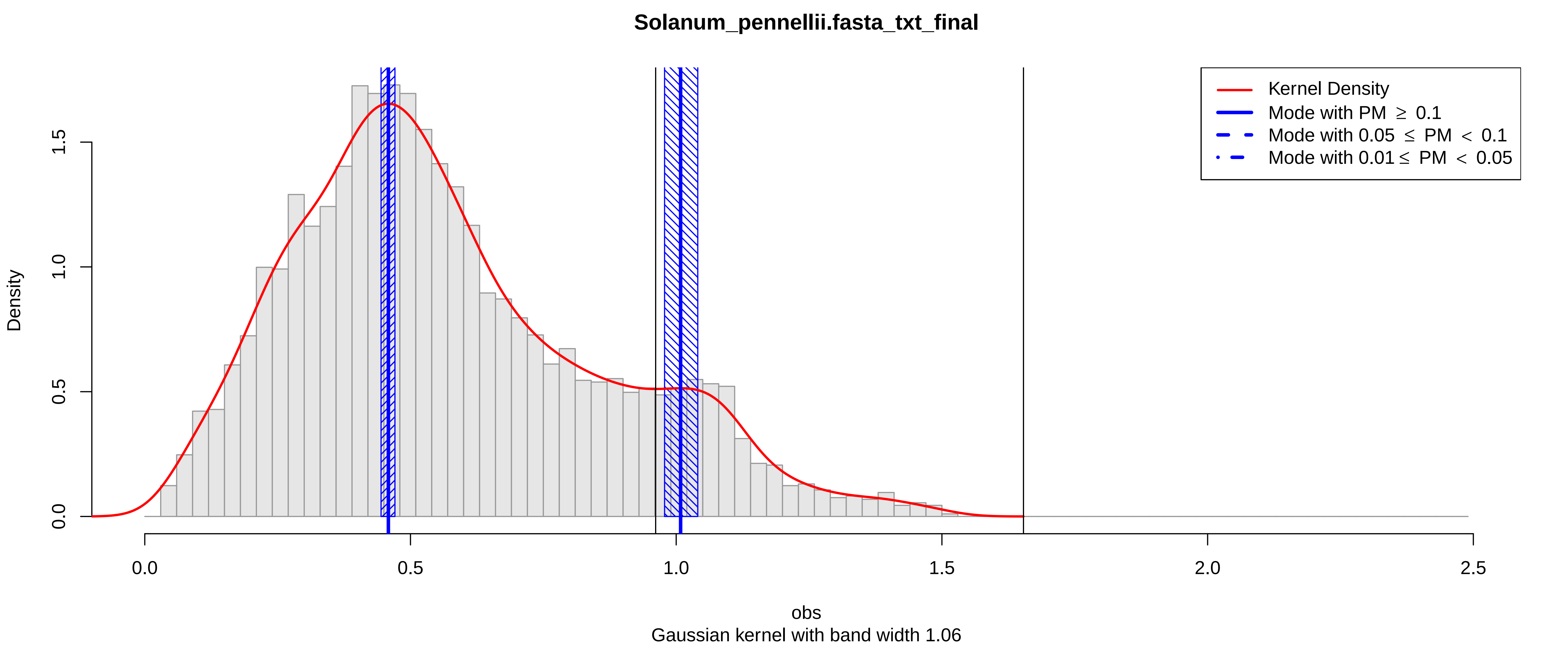

Solanum_pennellii.fasta_txt_final
Kernel Density
Mode with PM ≥ 0.1
Mode with 0.05 ≤ PM < 0.1
Mode with 0.01 ≤ PM < 0.05
Density
0.0
0.5
1.0
1.5
0.0
0.5
1.0
1.5
2.0
2.5
obs
Gaussian kernel with band width 1.06

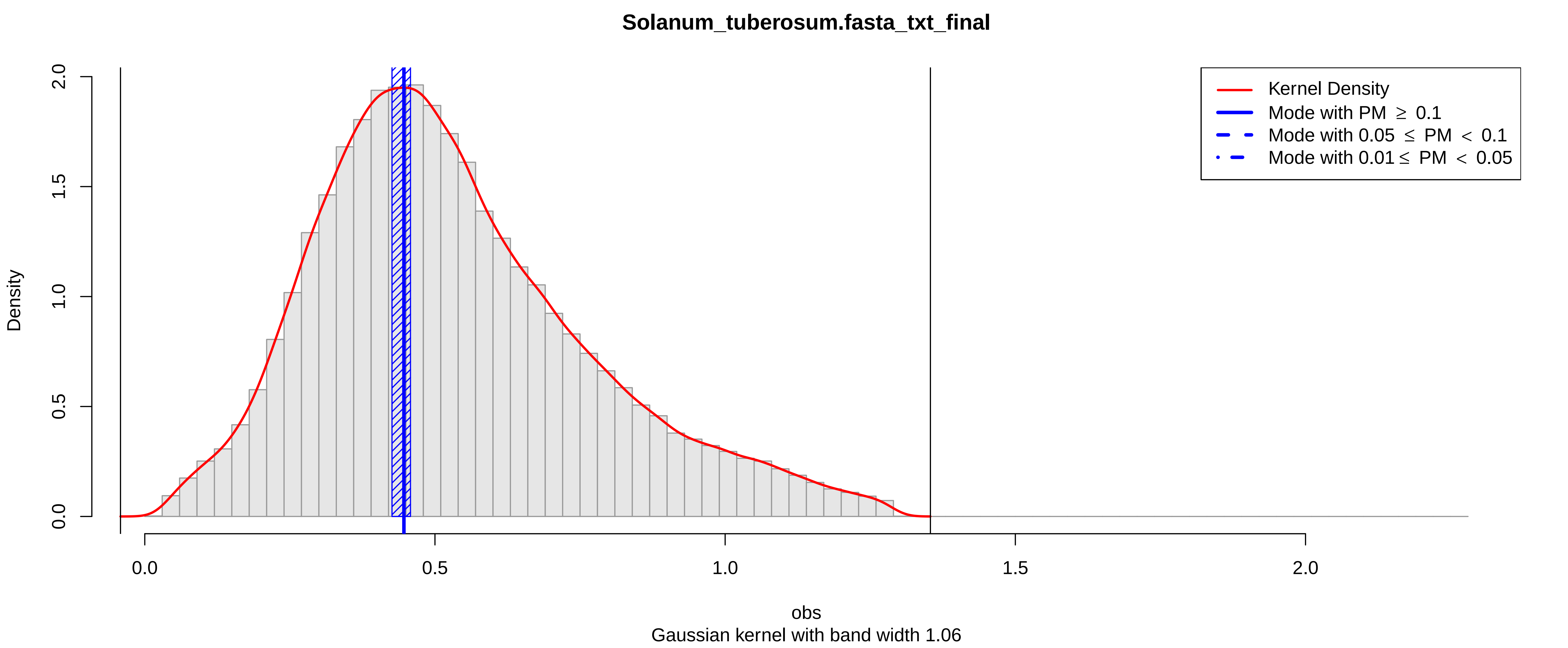

Solanum_tuberosum.fasta_txt_final
Density
0.0
0.5
1.0
1.5
2.0
0.0
0.5
1.0
1.5
2.0
obs
Gaussian kernel with band width 1.06
Kernel Density
Mode with PM ≥ 0.1
Mode with 0.05 ≤ PM < 0.1
Mode with 0.01 ≤ PM < 0.05

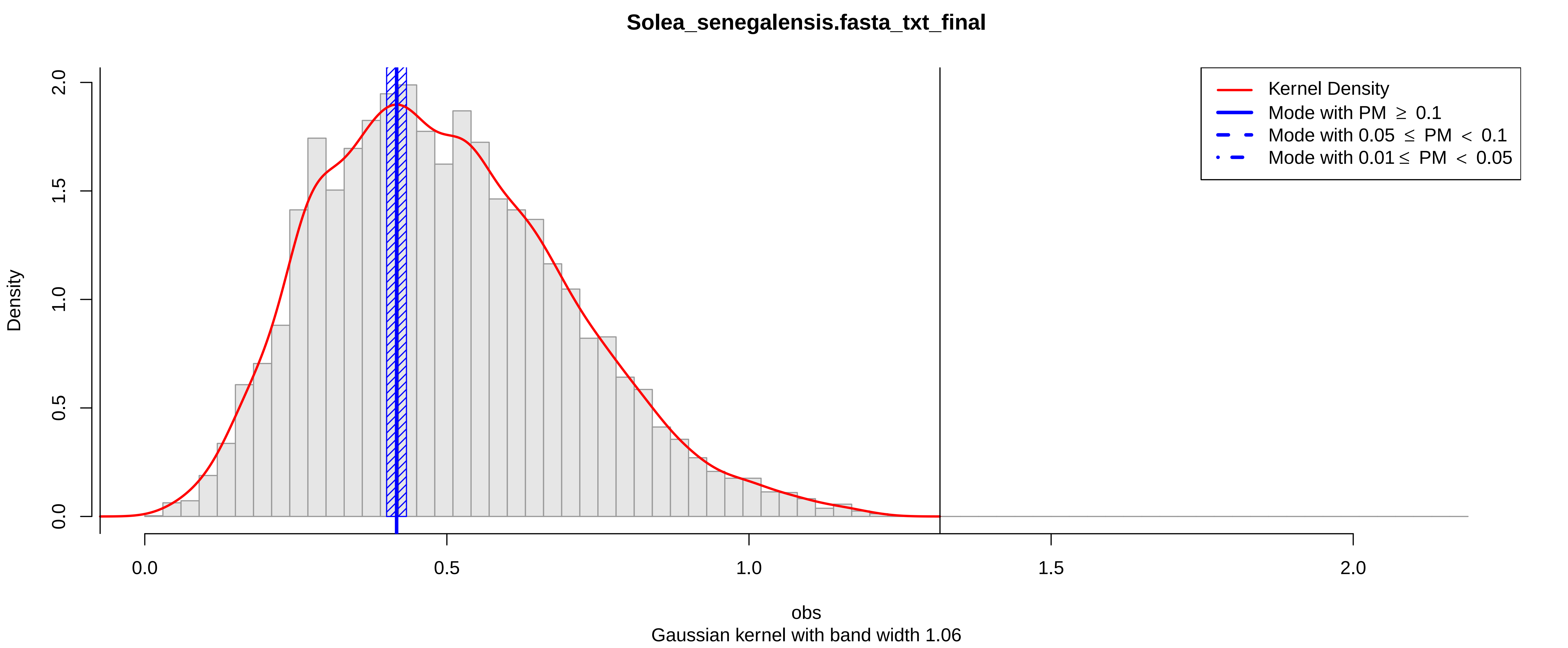

Solea_senegalensis.fasta_txt_final
Kernel Density
Mode with PM ≥ 0.1
Mode with 0.05 ≤ PM < 0.1
Mode with 0.01 ≤ PM < 0.05
Density
0.0
0.5
1.0
1.5
2.0
obs
Gaussian kernel with band width 1.06

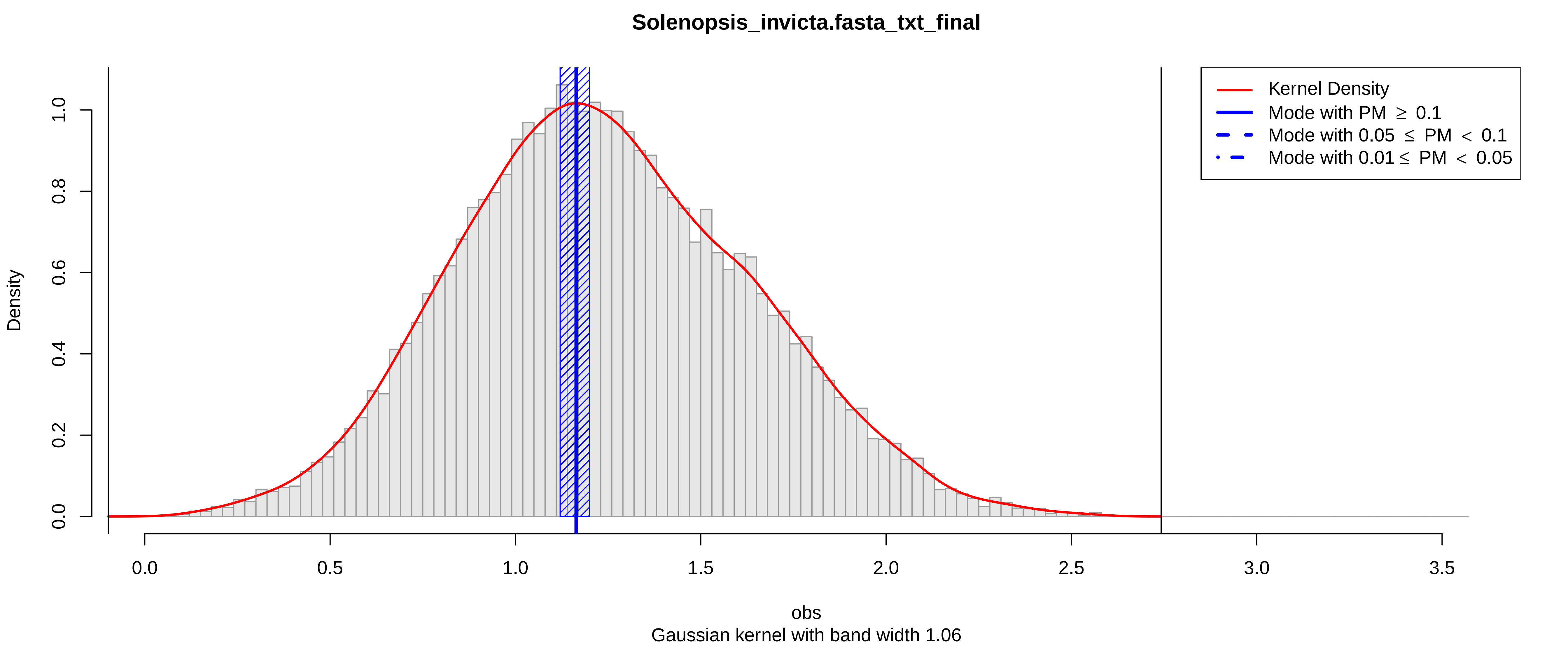
Solenopsis_invicta.fasta_txt_final
Kernel Density
Mode with PM ≥ 0.1
Mode with 0.05 ≤ PM < 0.1
Mode with 0.01 ≤ PM < 0.05
Density
0.0
0.2
0.4
0.6
0.8
1.0
0.0
0.5
1.0
1.5
2.0
2.5
3.0
3.5
obs
Gaussian kernel with band width 1.06

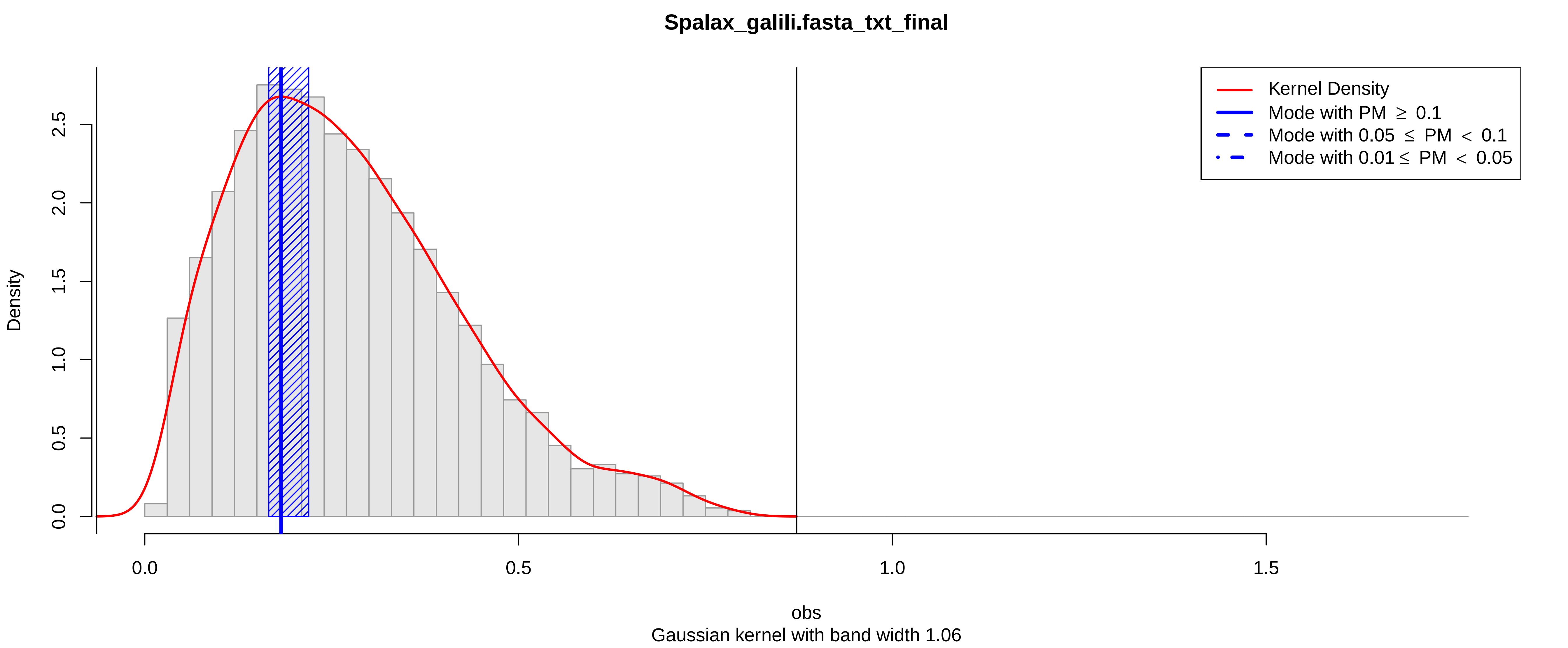
Spalax_galili.fasta_txt_final
Kernel Density
Mode with PM ≥ 0.1
Mode with 0.05 ≤ PM < 0.1
Mode with 0.01 ≤ PM < 0.05
Density
0.0
0.5
1.0
1.5
2.0
2.5
0.0
0.5
1.0
1.5
obs
Gaussian kernel with band width 1.06

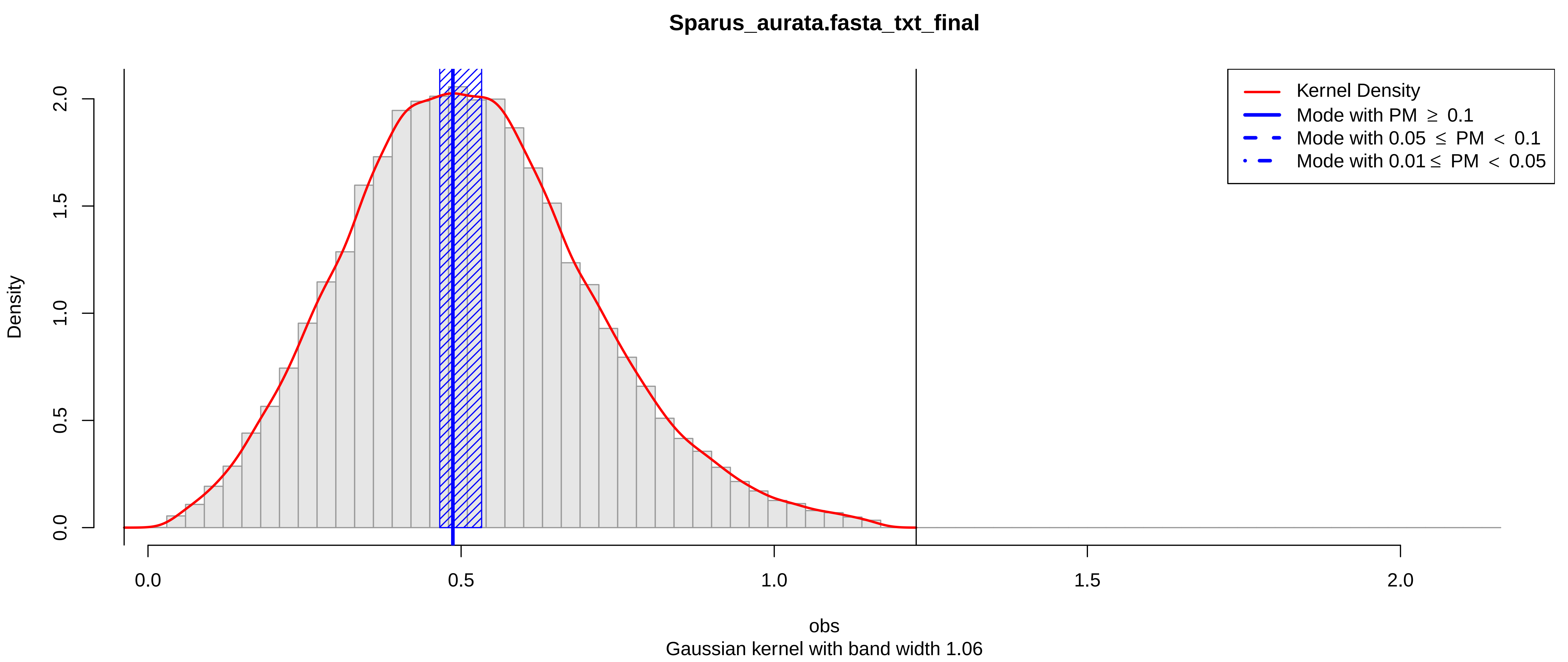
Sparus_aurata.fasta_txt_final
Kernel Density
Mode with PM ≥ 0.1
Mode with 0.05 ≤ PM < 0.1
Mode with 0.01 ≤ PM < 0.05
Density
0.0
0.5
1.0
1.5
2.0
0.0
0.5
1.0
1.5
2.0
obs
Gaussian kernel with band width 1.06

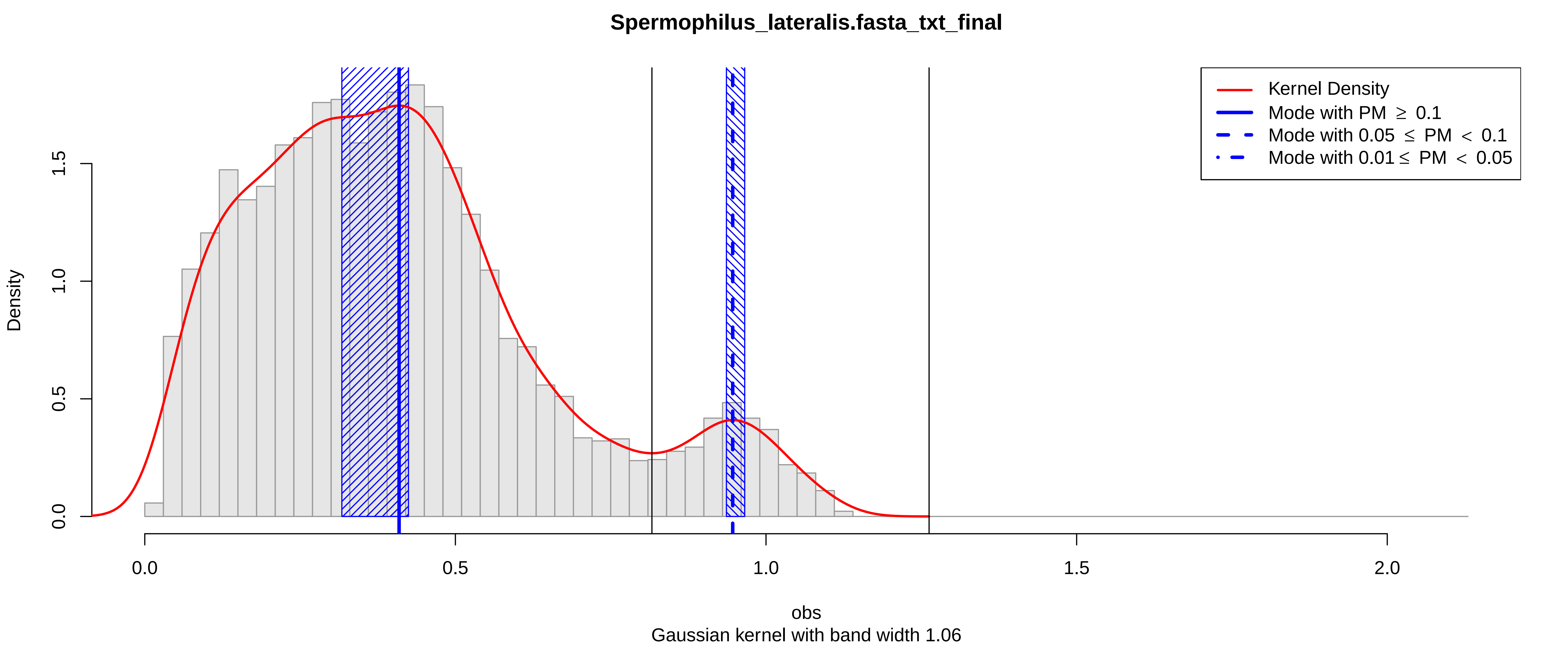

Spermophilus_lateralis.fasta_txt_final
Kernel Density
Mode with PM ≥ 0.1
Mode with 0.05 ≤ PM < 0.1
Mode with 0.01 ≤ PM < 0.05
Density
0.0
0.5
1.0
1.5
2.0
obs
Gaussian kernel with band width 1.06

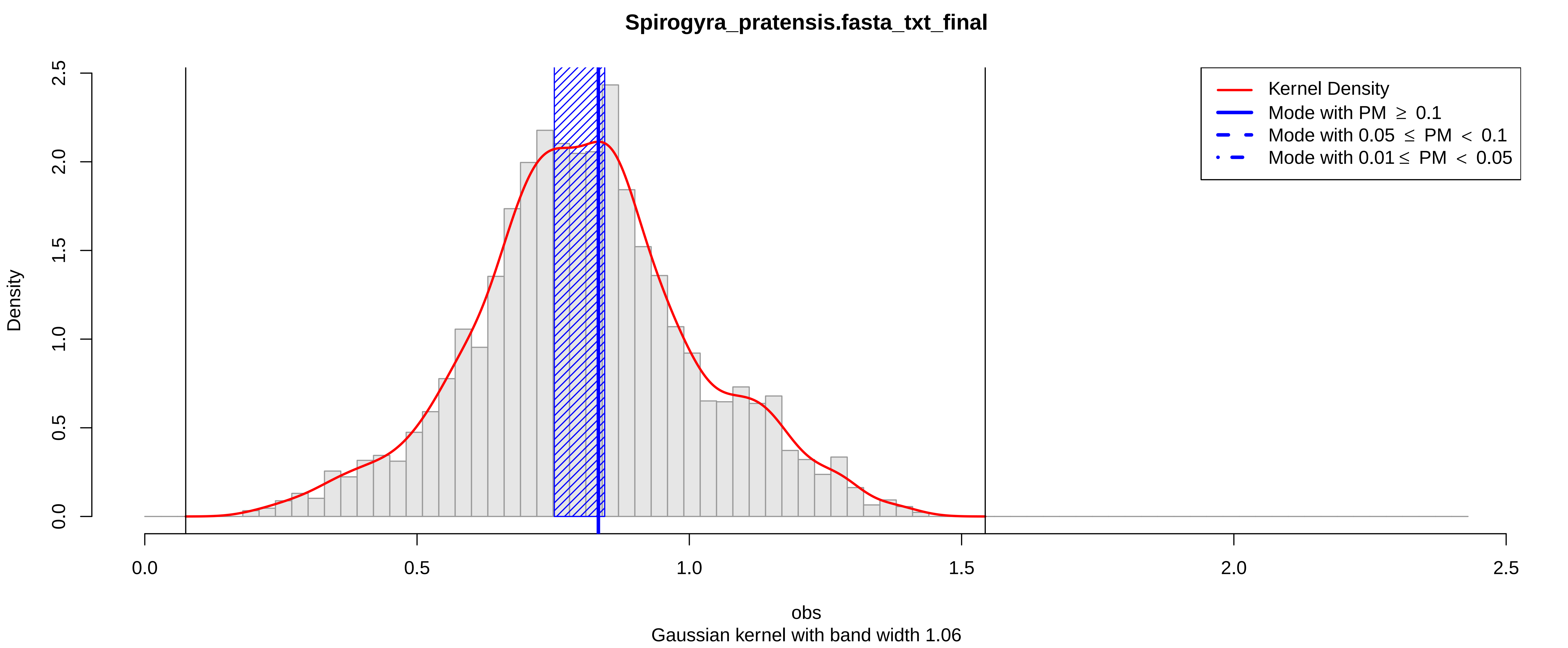
Spirogyra_pratensis.fasta_txt_final
Density
0.0
0.5
1.0
1.5
2.0
2.5
Kernel Density
Mode with PM ≥ 0.1
Mode with 0.05 ≤ PM < 0.1
Mode with 0.01 ≤ PM < 0.05
obs
Gaussian kernel with band width 1.06

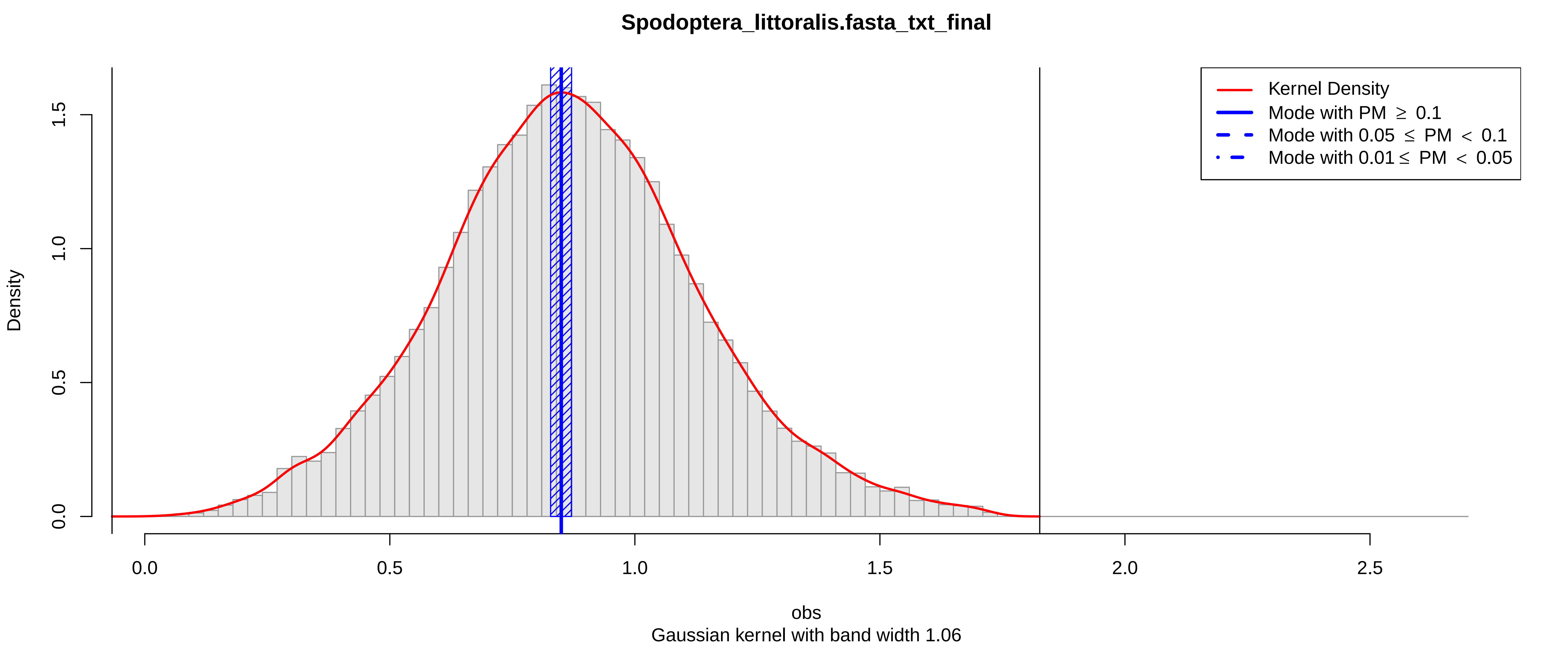

Spodoptera_littoralis.fasta_txt_final
Kernel Density
Mode with PM ≥ 0.1
Mode with 0.05 ≤ PM < 0.1
Mode with 0.01 ≤ PM < 0.05
Density
0.0
0.5
1.0
1.5
0.0
0.5
1.0
1.5
2.0
2.5
obs
Gaussian kernel with band width 1.06

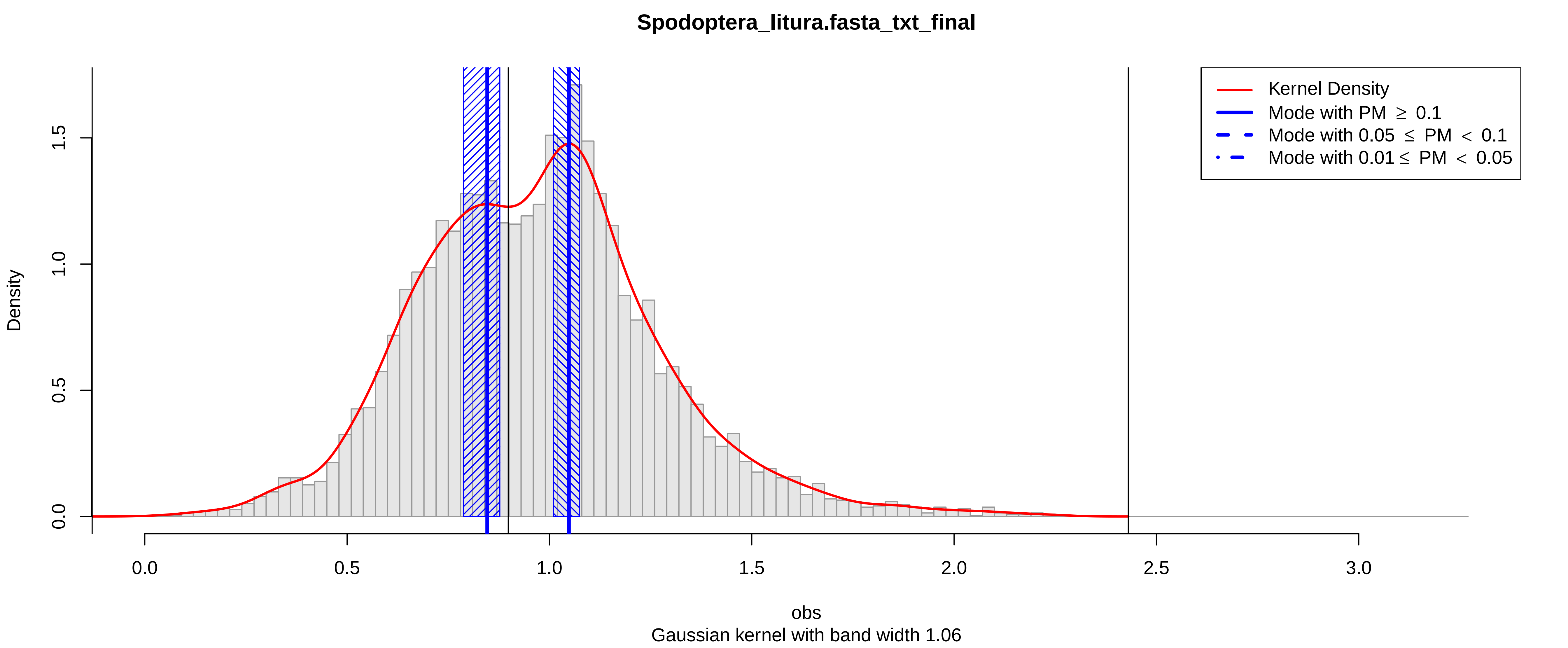

Spodoptera_litura.fasta_txt_final
Kernel Density
Mode with PM ≥ 0.1
Mode with 0.05 ≤ PM < 0.1
Mode with 0.01 ≤ PM < 0.05
Density
0.0
0.5
1.0
1.5
0.0
0.5
1.0
1.5
2.0
2.5
3.0
obs
Gaussian kernel with band width 1.06

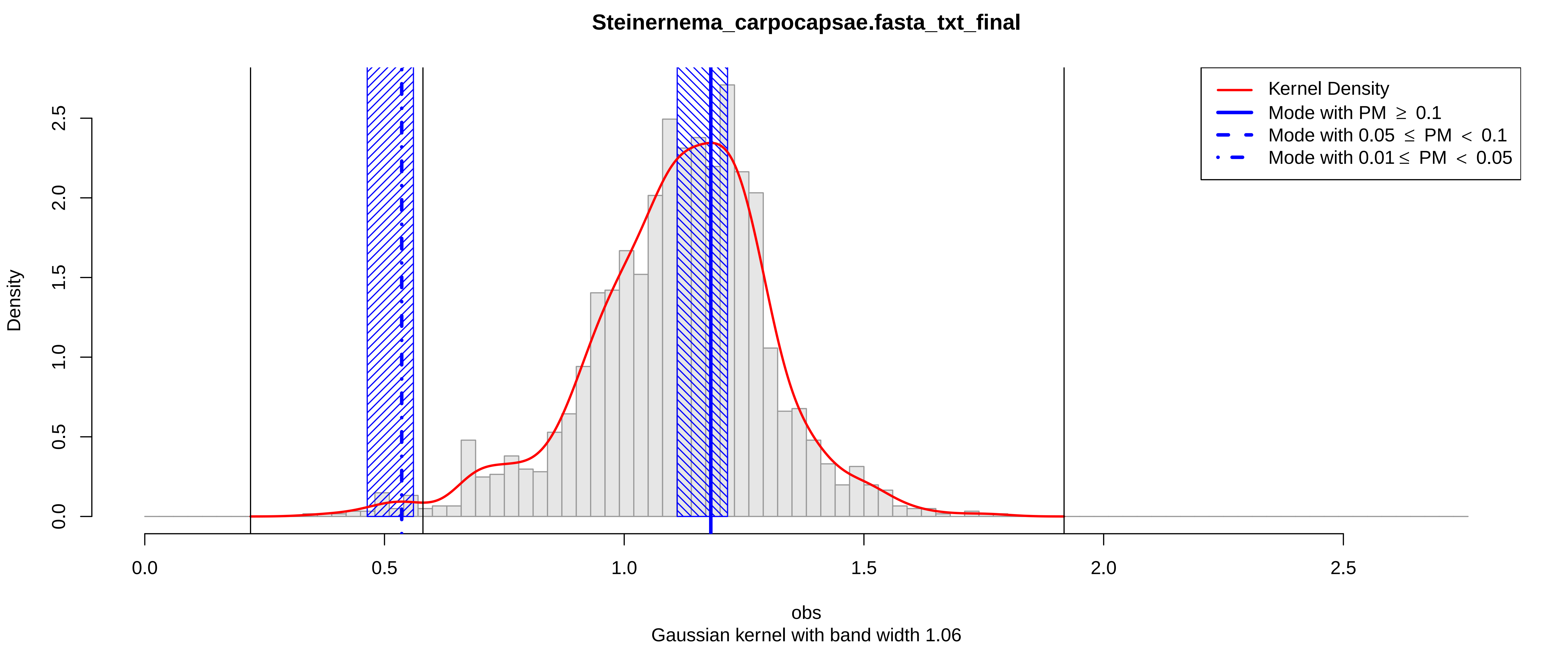
Steinernema_carpocapsae.fasta_txt_final
Kernel Density
Mode with PM ≥ 0.1
Mode with 0.05 ≤ PM < 0.1
Mode with 0.01 ≤ PM < 0.05
Density
0.0
0.5
1.0
1.5
2.0
2.5
obs
Gaussian kernel with band width 1.06

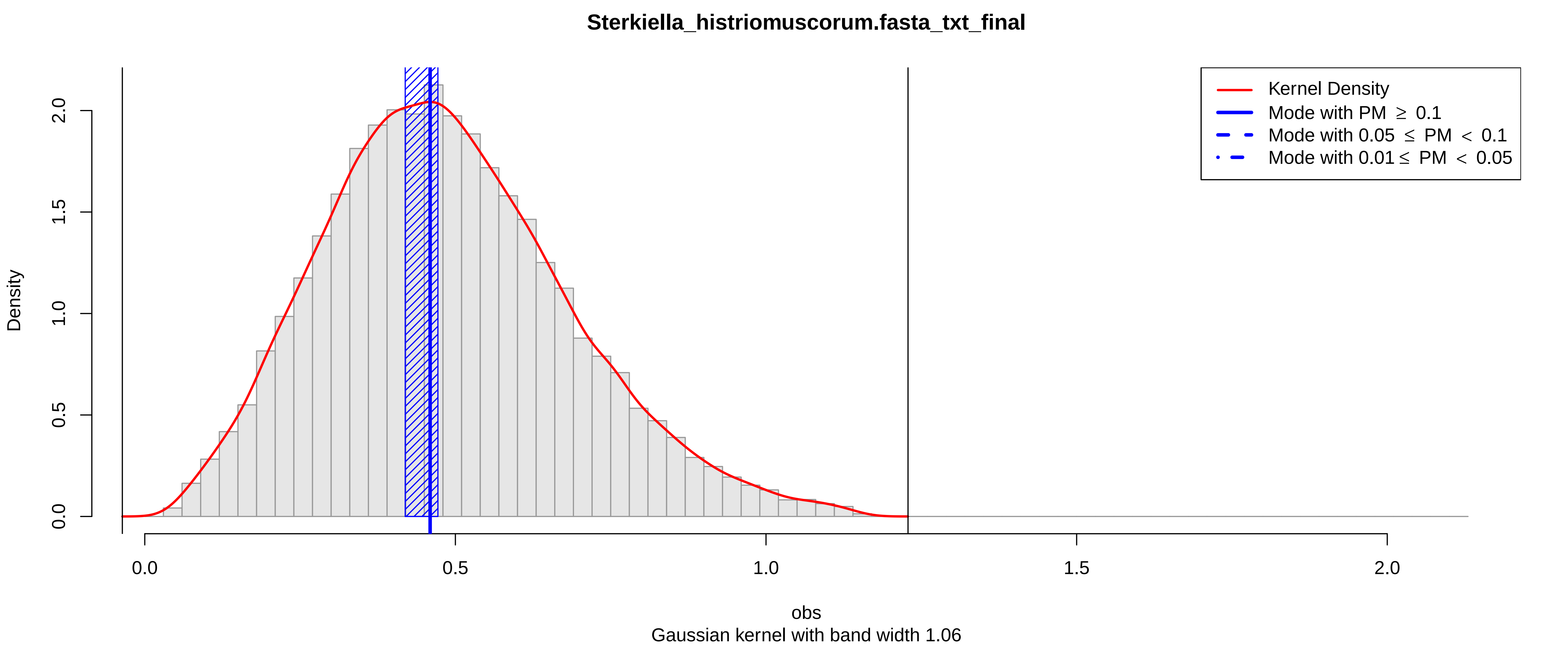

Sterkiella_histriomuscorum.fasta_txt_final
Kernel Density
Mode with PM ≥ 0.1
Mode with 0.05 ≤ PM < 0.1
Mode with 0.01 ≤ PM < 0.05
Density
0.0
0.5
1.0
1.5
2.0
obs
Gaussian kernel with band width 1.06

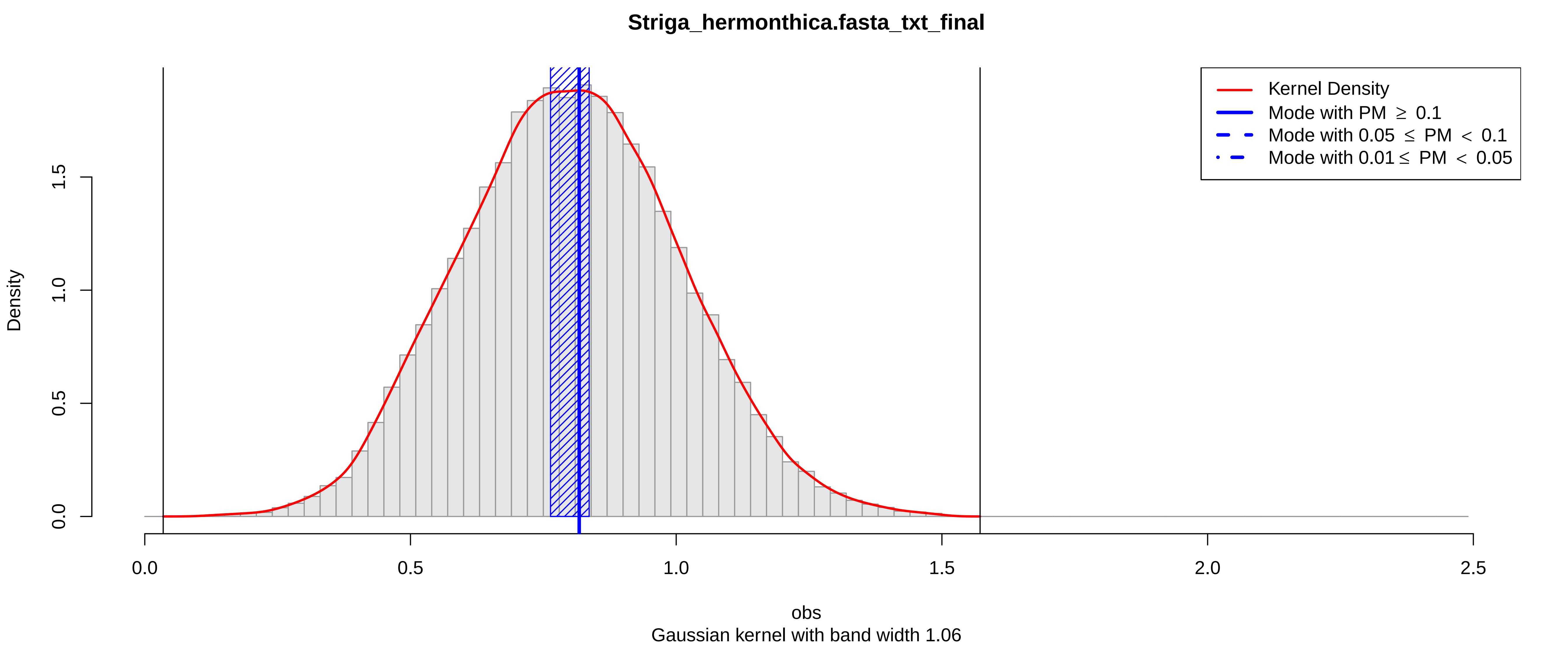
Striga_hermonthica.fasta_txt_final
Kernel Density
Mode with PM ≥ 0.1
Mode with 0.05 ≤ PM < 0.1
Mode with 0.01 ≤ PM < 0.05
Density
0.0
0.5
1.0
1.5
0.0
0.5
1.0
1.5
2.0
2.5
obs
Gaussian kernel with band width 1.06

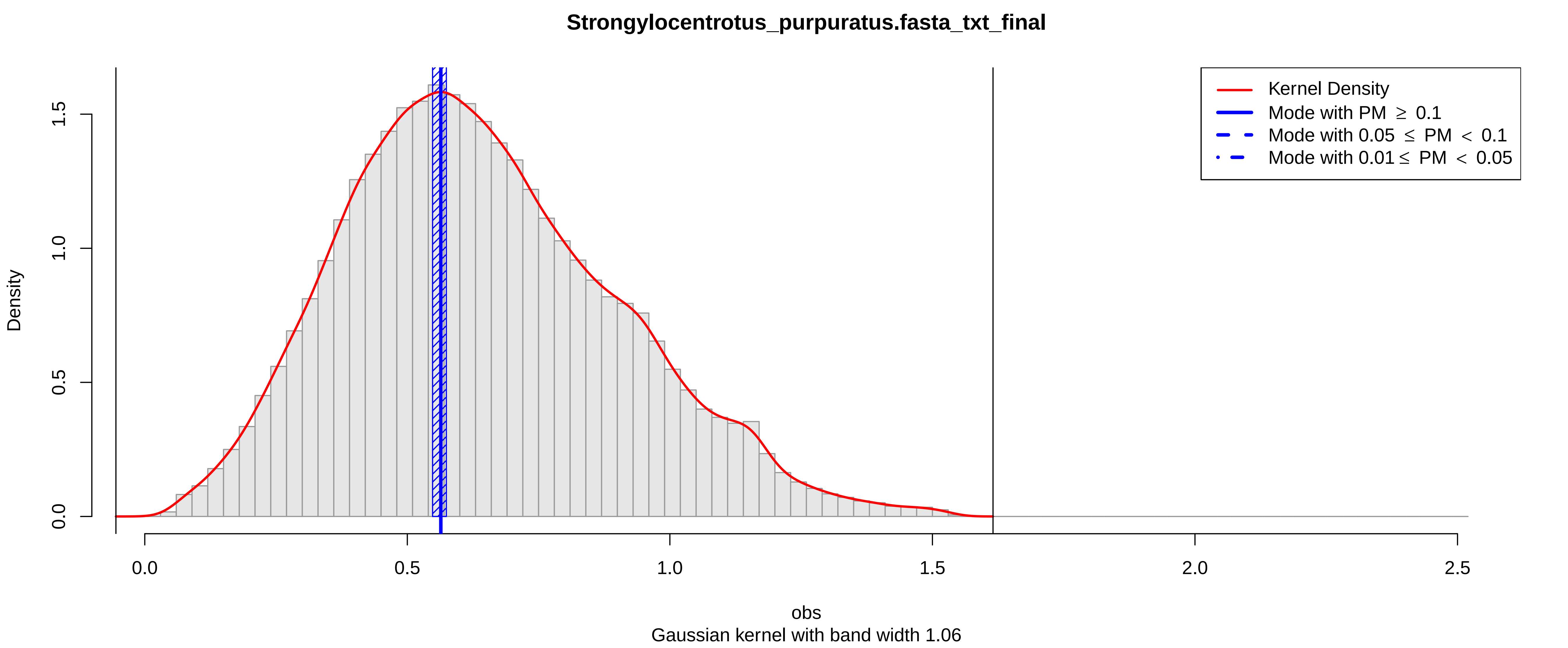
Strongylocentrotus_purpuratus.fasta_txt_final
Density
0.0
0.5
1.0
1.5
0.0
0.5
1.0
1.5
2.0
2.5
Kernel Density
Mode with PM ≥ 0.1
Mode with 0.05 ≤ PM < 0.1
Mode with 0.01 ≤ PM < 0.05
obs
Gaussian kernel with band width 1.06

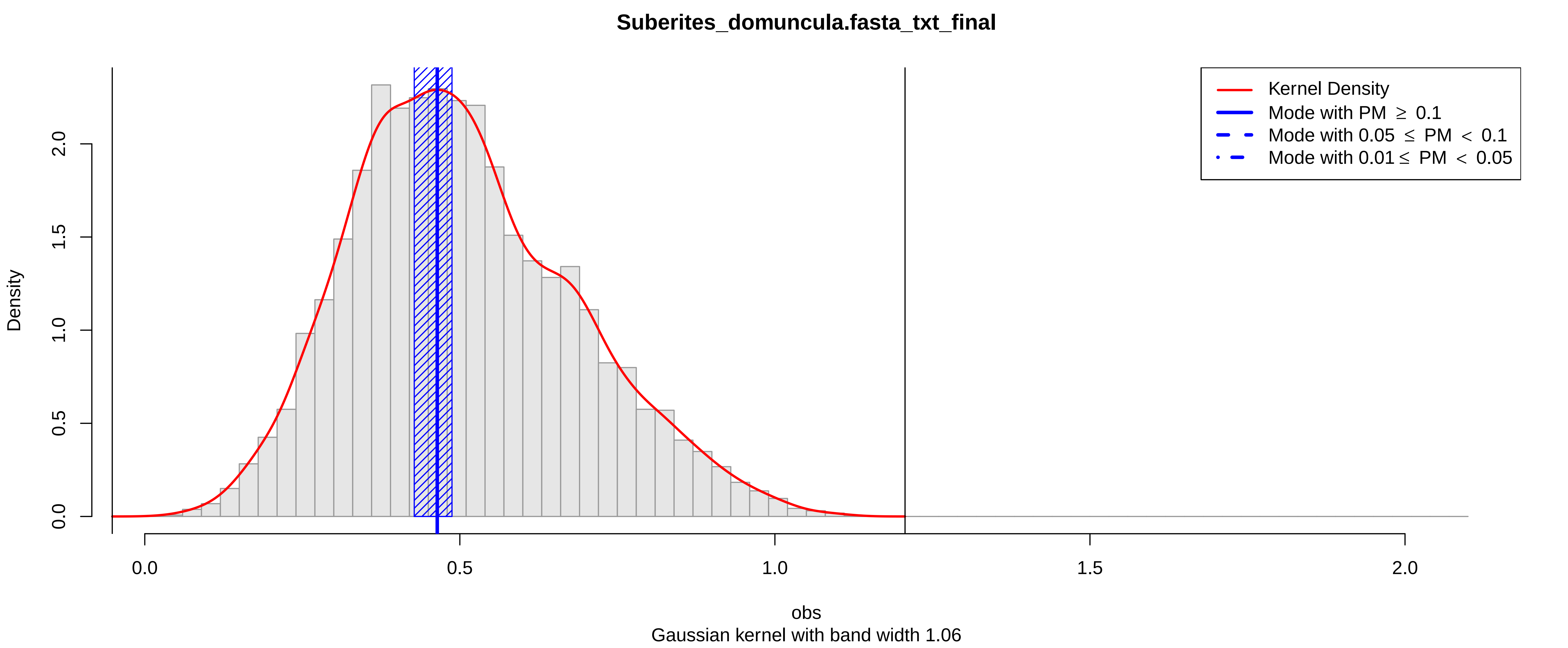
Suberites_domuncula.fasta_txt_final
Kernel Density
Mode with PM ≥ 0.1
Mode with 0.05 ≤ PM < 0.1
Mode with 0.01 ≤ PM < 0.05
Density
0.0
0.5
1.0
1.5
2.0
obs
Gaussian kernel with band width 1.06

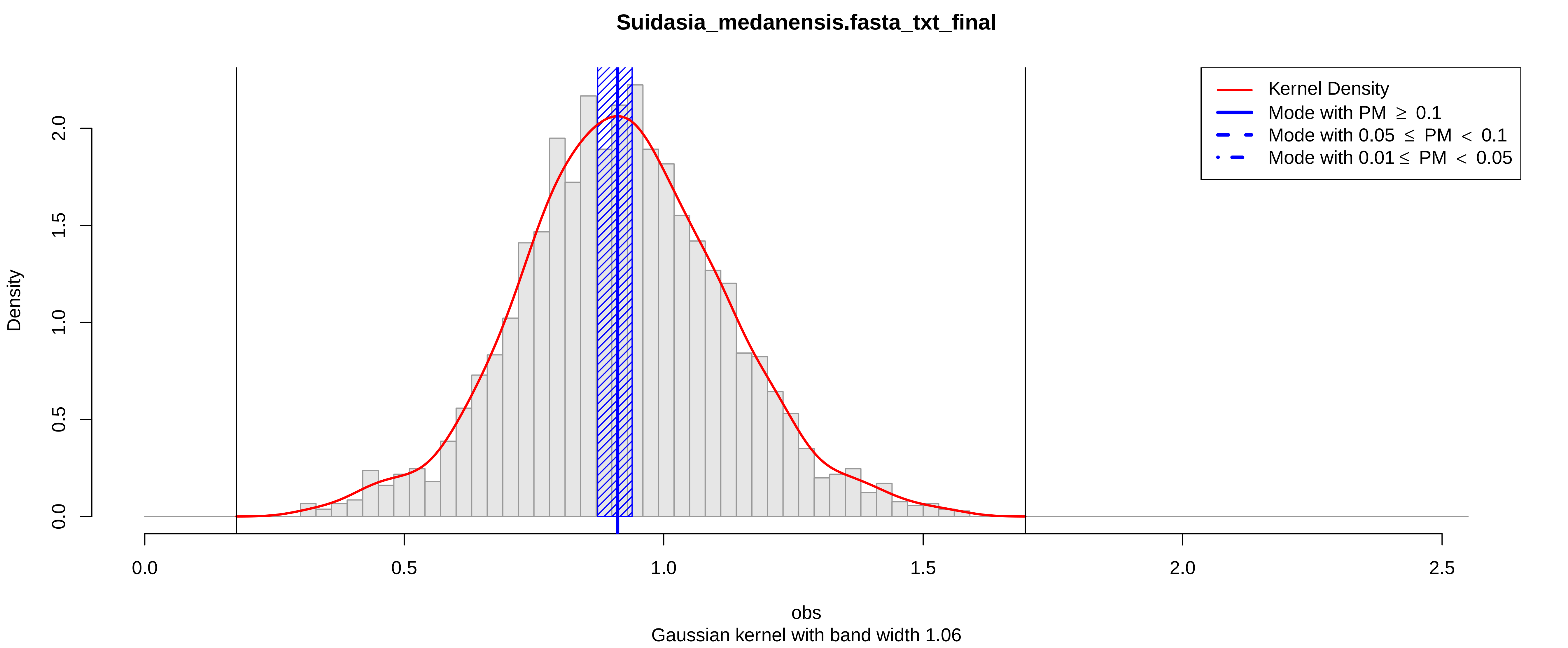

Suidasia_medanensis.fasta_txt_final
Kernel Density
Mode with PM ≥ 0.1
Mode with 0.05 ≤ PM < 0.1
Mode with 0.01 ≤ PM < 0.05
Density
0.0
0.5
1.0
1.5
2.0
0.0
0.5
1.0
1.5
2.0
2.5
obs
Gaussian kernel with band width 1.06

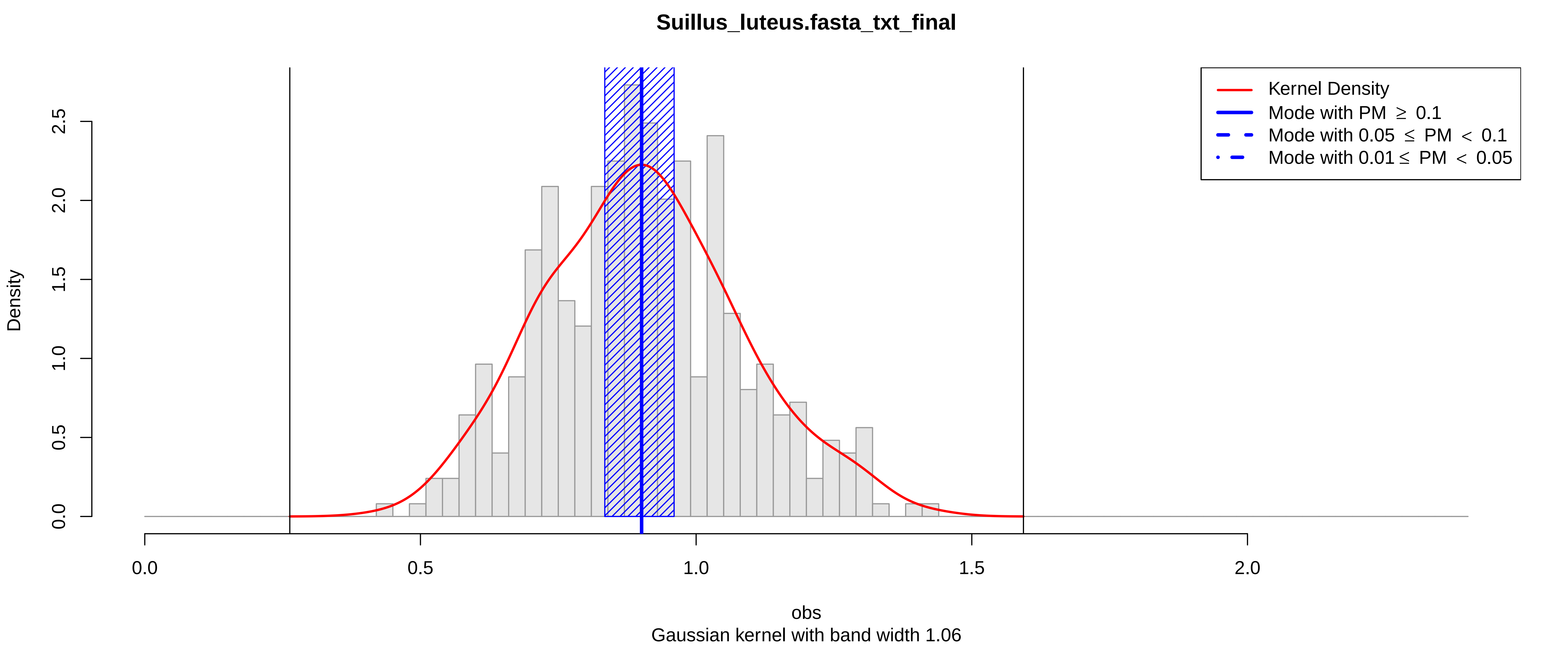
Suillus_luteus.fasta_txt_final
Kernel Density
Mode with PM ≥ 0.1
Mode with 0.05 ≤ PM < 0.1
Mode with 0.01 ≤ PM < 0.05
Density
0.0
0.5
1.0
1.5
2.0
2.5
0.0
0.5
1.0
1.5
2.0
obs
Gaussian kernel with band width 1.06

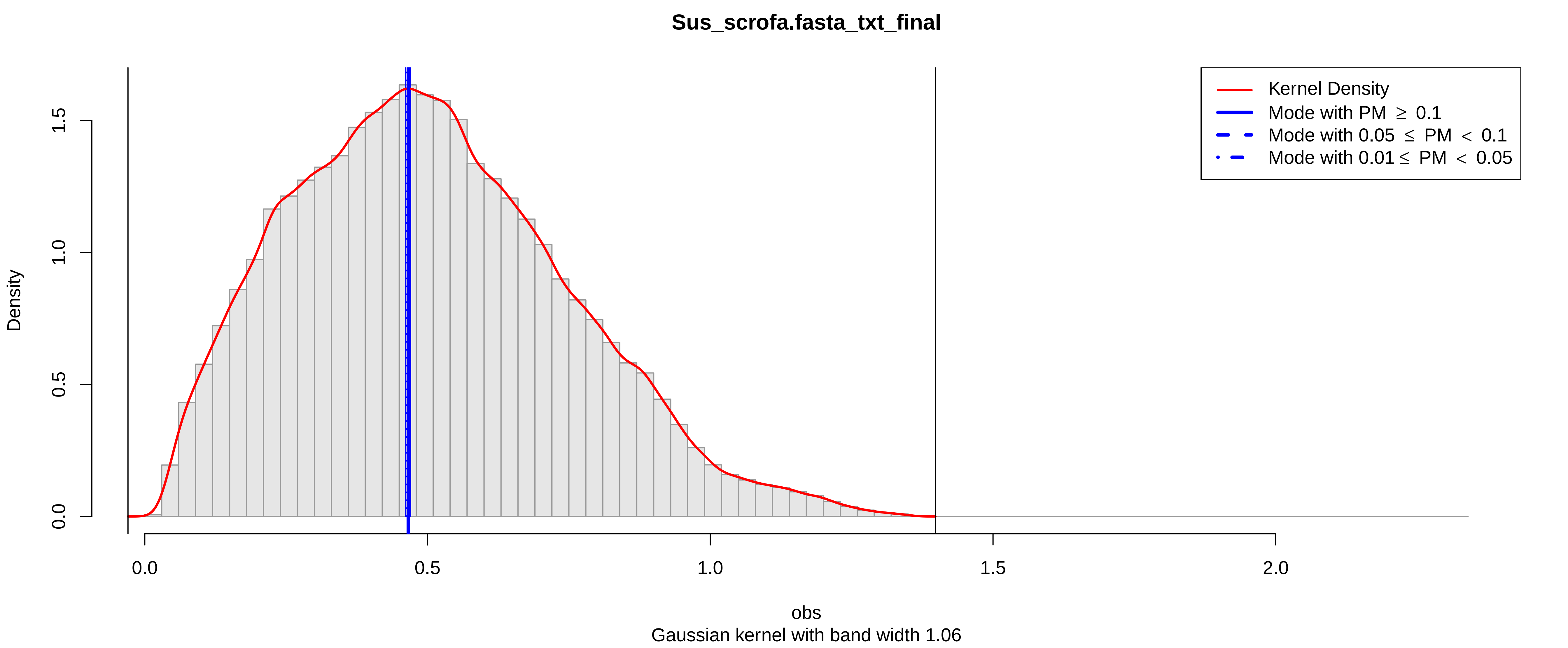
Sus_scrofa.fasta_txt_final
Kernel Density
Mode with PM ≥ 0.1
Mode with 0.05 ≤ PM < 0.1
Mode with 0.01 ≤ PM < 0.05
Density
0.0
0.5
1.0
1.5
0.0
0.5
1.0
1.5
2.0
obs
Gaussian kernel with band width 1.06

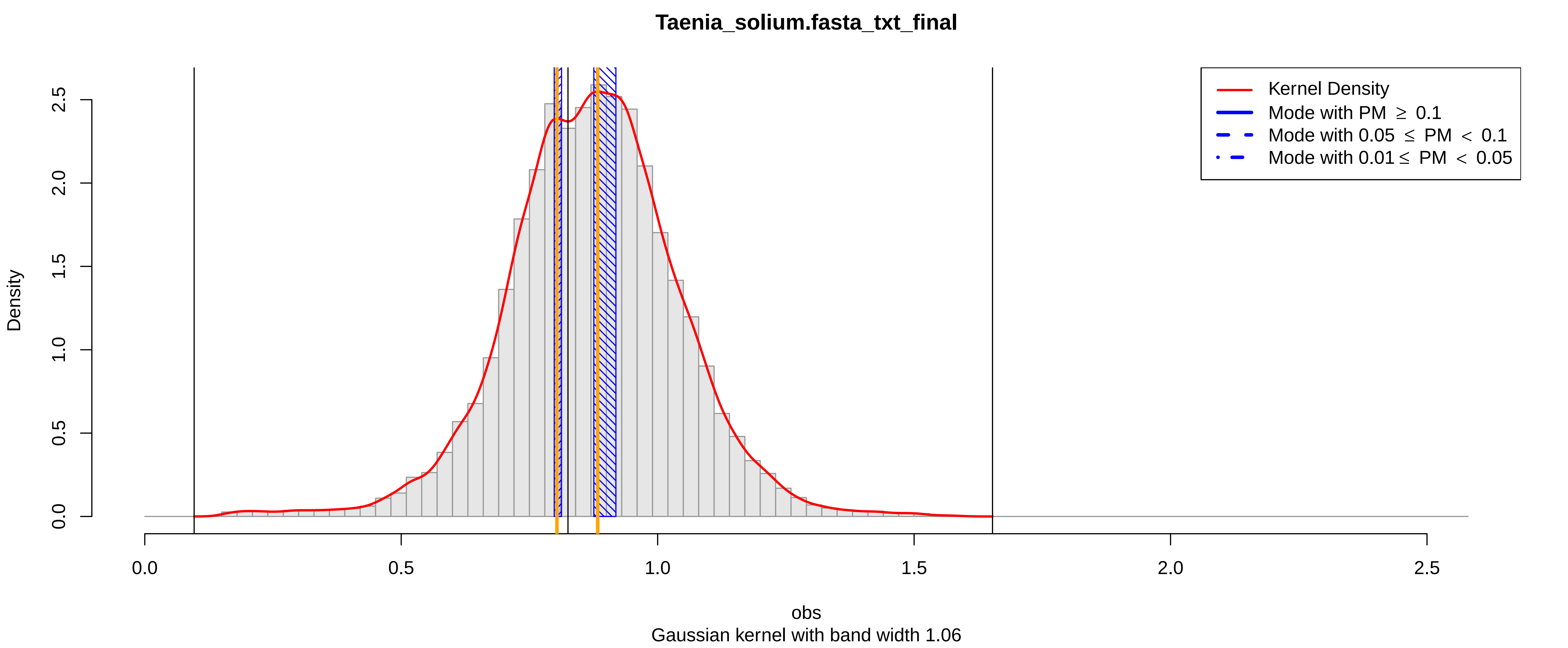

Taenia_solium.fasta_txt_final
Kernel Density
Mode with PM ≥ 0.1
Mode with 0.05 ≤ PM < 0.1
Mode with 0.01 ≤ PM < 0.05
Density
0.0
0.5
1.0
1.5
2.0
2.5
obs
Gaussian kernel with band width 1.06

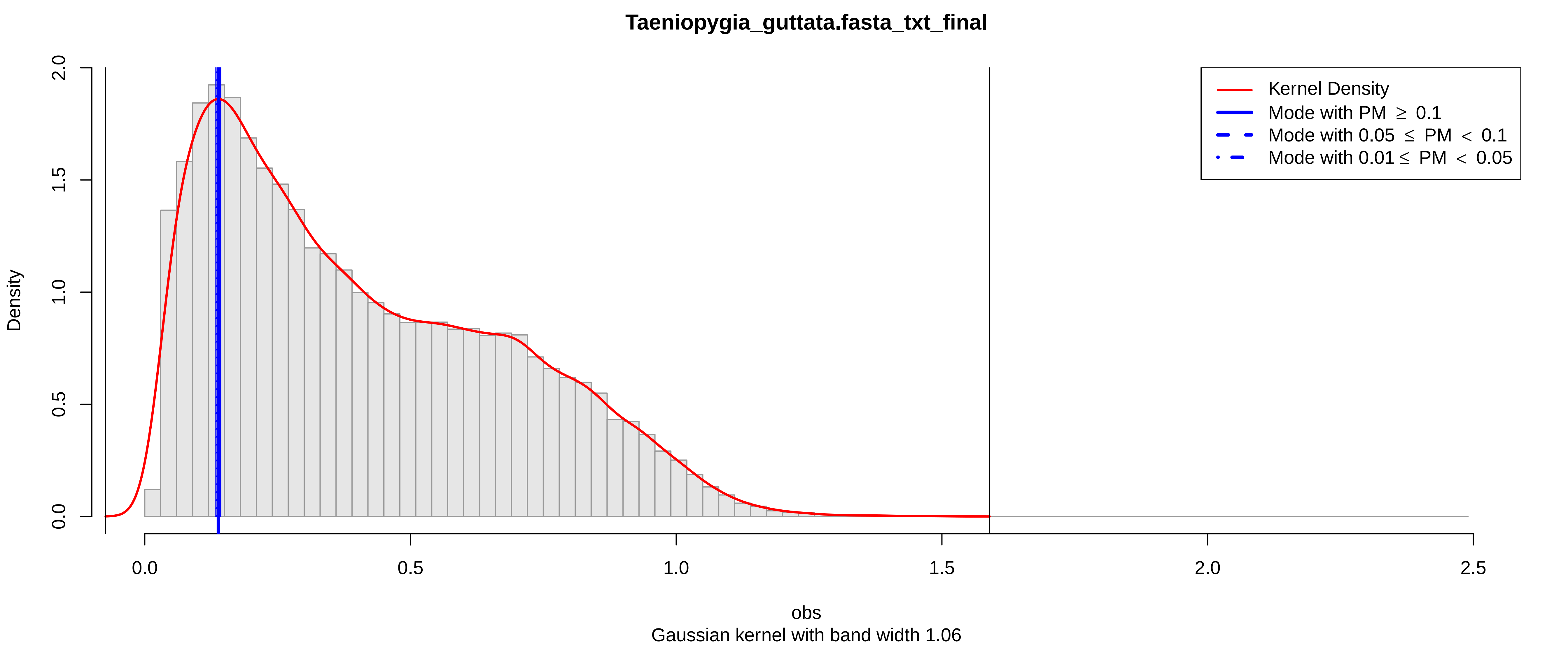
Taeniopygia_guttata.fasta_txt_final
Density
0.0
0.5
1.0
1.5
2.0
2.5
obs
Gaussian kernel with band width 1.06
Kernel Density
Mode with PM ≥ 0.1
Mode with 0.05 ≤ PM < 0.1
Mode with 0.01 ≤ PM < 0.05

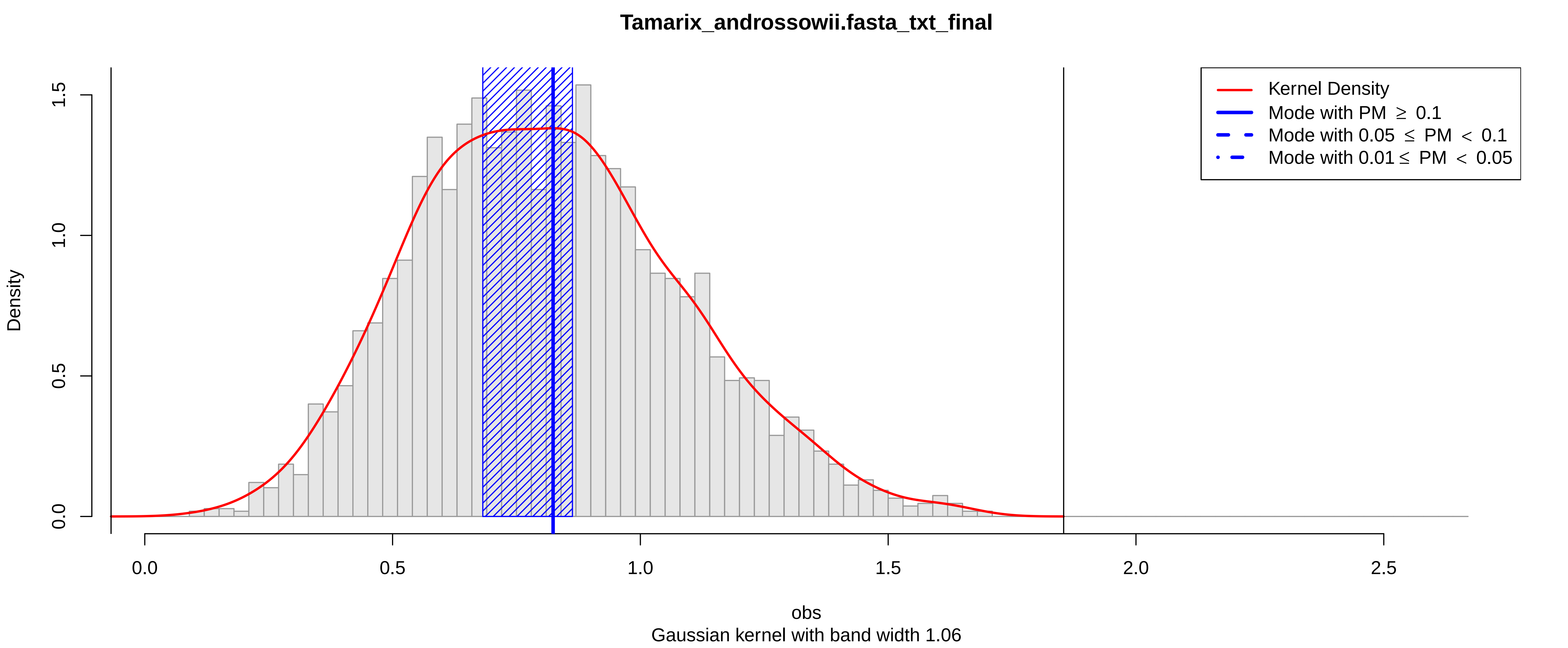

Tamarix_androssowii.fasta_txt_final
Kernel Density
Mode with PM ≥ 0.1
Mode with 0.05 ≤ PM < 0.1
Mode with 0.01 ≤ PM < 0.05
Density
0.0
0.5
1.0
1.5
0.0
0.5
1.0
1.5
2.0
2.5
obs
Gaussian kernel with band width 1.06

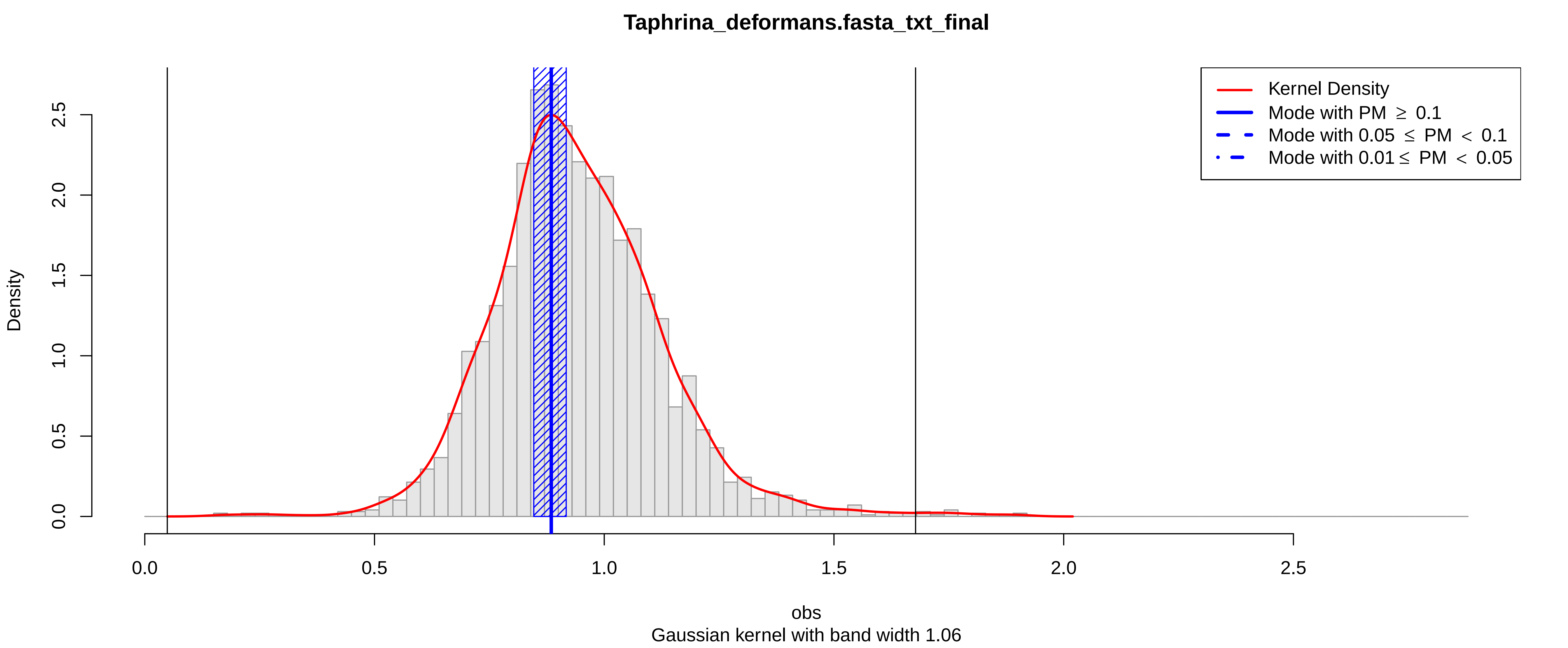
Taphrina_deformans.fasta_txt_final
Kernel Density
Mode with PM ≥ 0.1
Mode with 0.05 ≤ PM < 0.1
Mode with 0.01 ≤ PM < 0.05
Density
0.0
0.5
1.0
1.5
2.0
2.5
0.0
0.5
1.0
1.5
2.0
2.5
obs
Gaussian kernel with band width 1.06

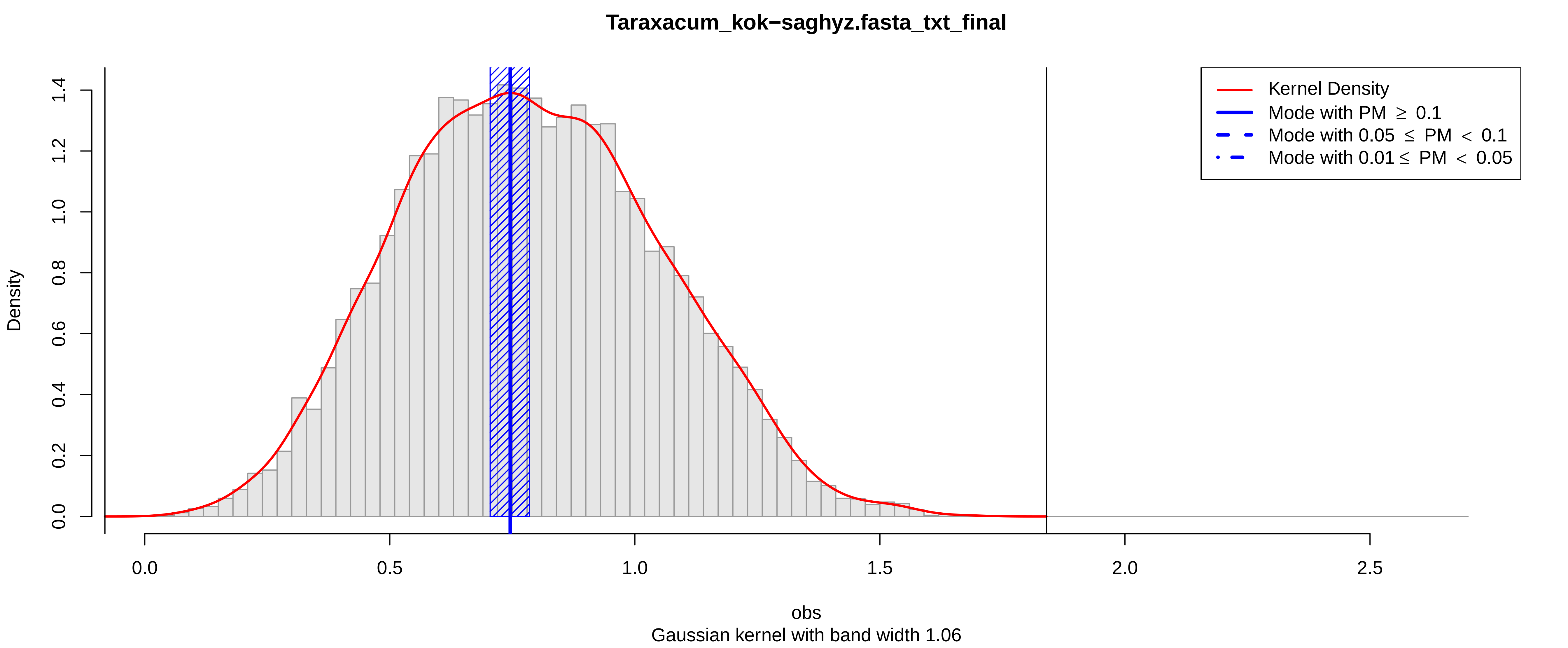

Taraxacum_kok−saghyz.fasta_txt_final
Kernel Density
Mode with PM ≥ 0.1
Mode with 0.05 ≤ PM < 0.1
Mode with 0.01 ≤ PM < 0.05
Density
0.0
0.2
0.4
0.6
0.8
1.0
1.2
1.4
0.0
0.5
1.0
1.5
2.0
2.5
obs
Gaussian kernel with band width 1.06

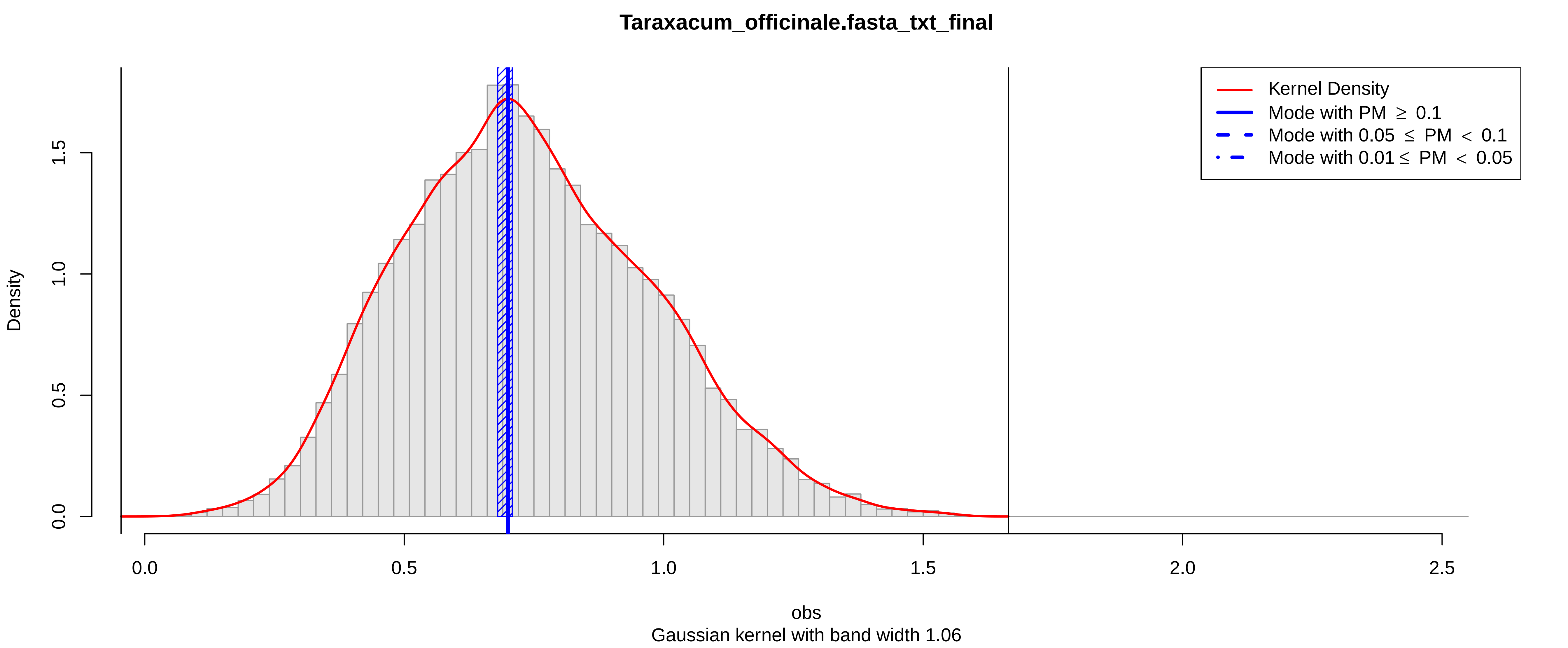

Taraxacum_officinale.fasta_txt_final
Kernel Density
Mode with PM ≥ 0.1
Mode with 0.05 ≤ PM < 0.1
Mode with 0.01 ≤ PM < 0.05
Density
0.0
0.5
1.0
1.5
0.0
0.5
1.0
1.5
2.0
2.5
obs
Gaussian kernel with band width 1.06

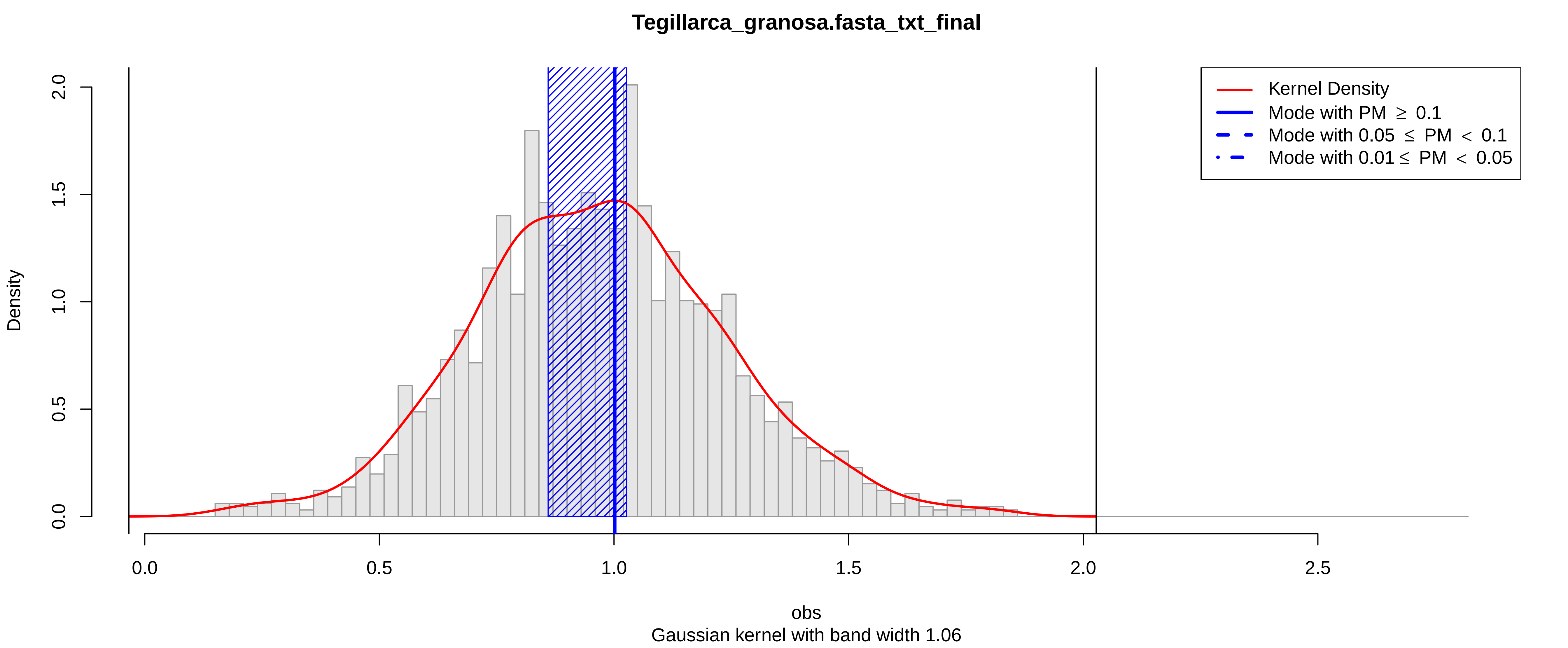

Tegillarca_granosa.fasta_txt_final
Kernel Density
Mode with PM ≥ 0.1
Mode with 0.05 ≤ PM < 0.1
Mode with 0.01 ≤ PM < 0.05
Density
0.0
0.5
1.0
1.5
2.0
0.0
0.5
1.0
1.5
2.0
2.5
obs
Gaussian kernel with band width 1.06

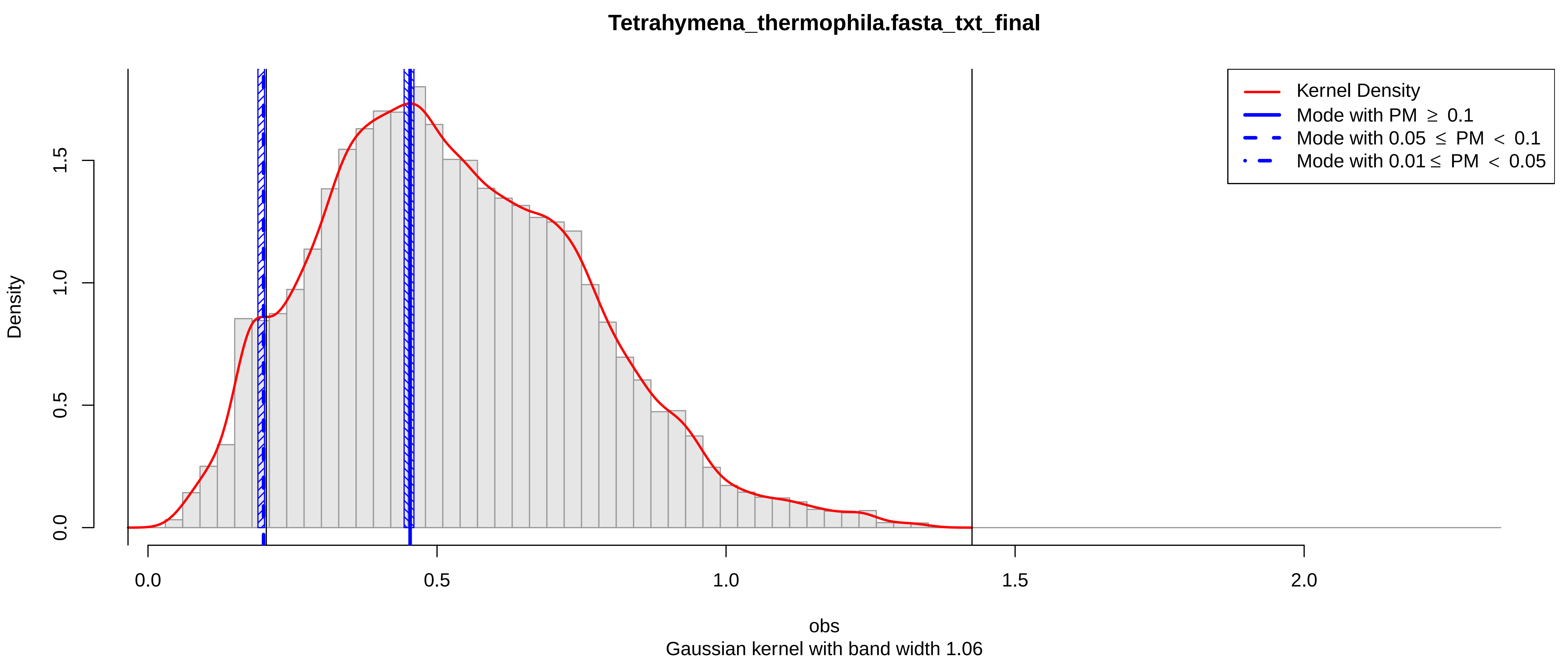

Tetrahymena_thermophila.fasta_txt_final
Kernel Density
Mode with PM ≥ 0.1
Mode with 0.05 ≤ PM < 0.1
Mode with 0.01 ≤ PM < 0.05
Density
0.0
0.5
1.0
1.5
0.0
0.5
1.0
1.5
2.0
obs
Gaussian kernel with band width 1.06

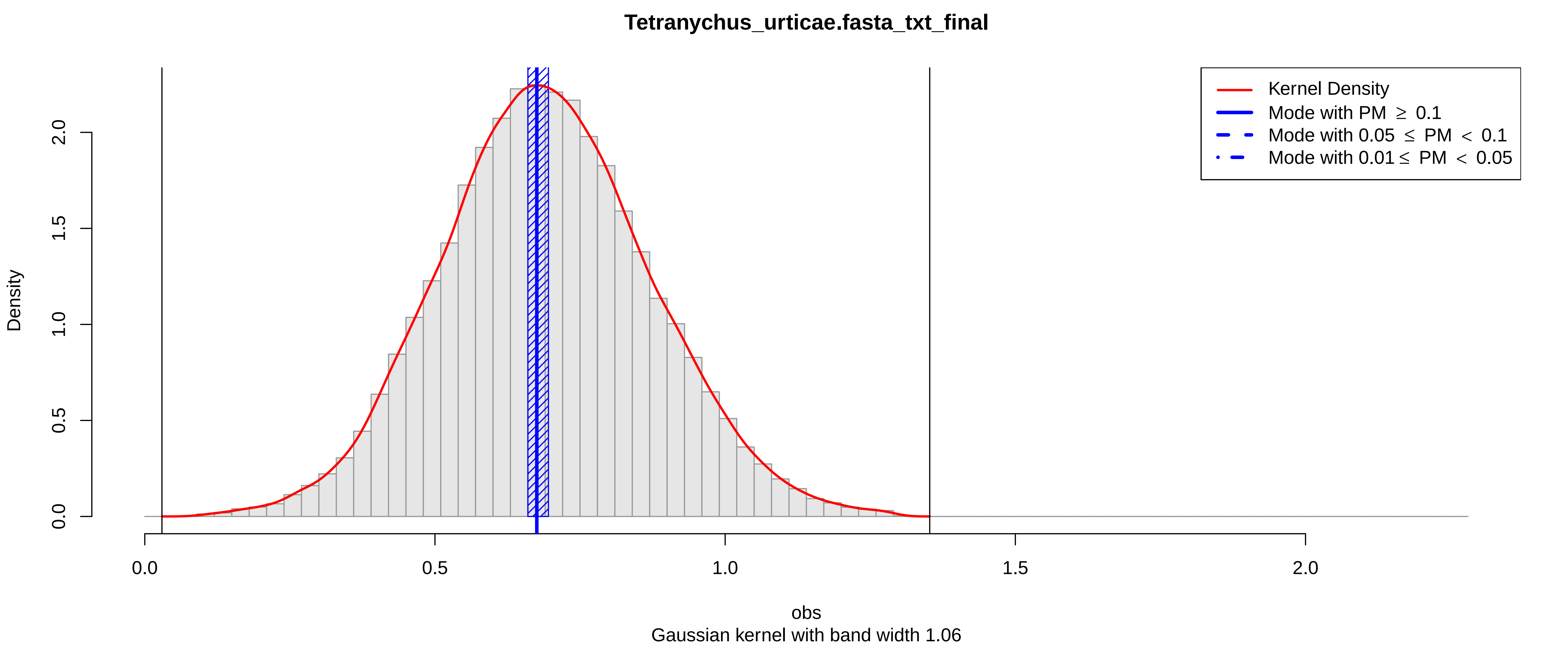

Tetranychus_urticae.fasta_txt_final
Kernel Density
Mode with PM ≥ 0.1
Mode with 0.05 ≤ PM < 0.1
Mode with 0.01 ≤ PM < 0.05
Density
0.0
0.5
1.0
1.5
2.0
obs
Gaussian kernel with band width 1.06

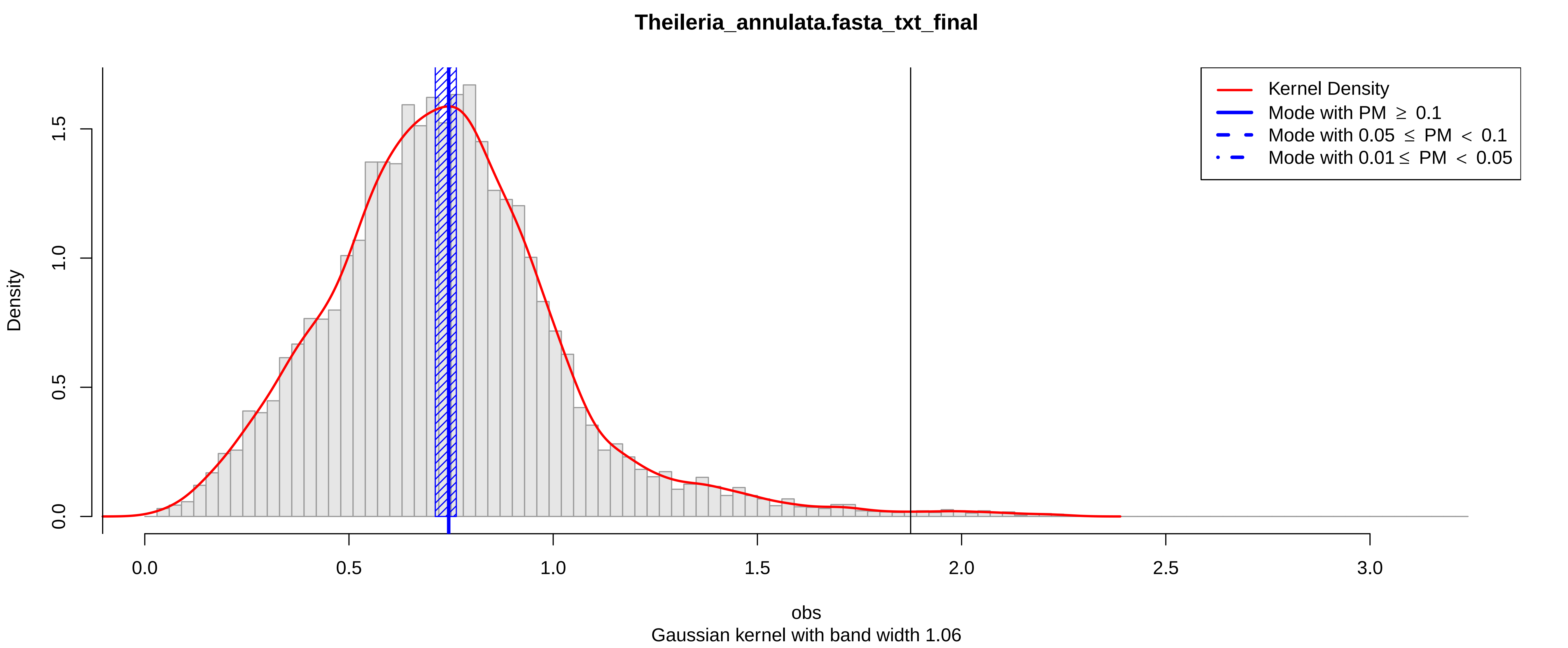

Theileria_annulata.fasta_txt_final
Kernel Density
Mode with PM ≥ 0.1
Mode with 0.05 ≤ PM < 0.1
Mode with 0.01 ≤ PM < 0.05
Density
0.0
0.5
1.0
1.5
0.0
0.5
1.0
1.5
2.0
2.5
3.0
obs
Gaussian kernel with band width 1.06

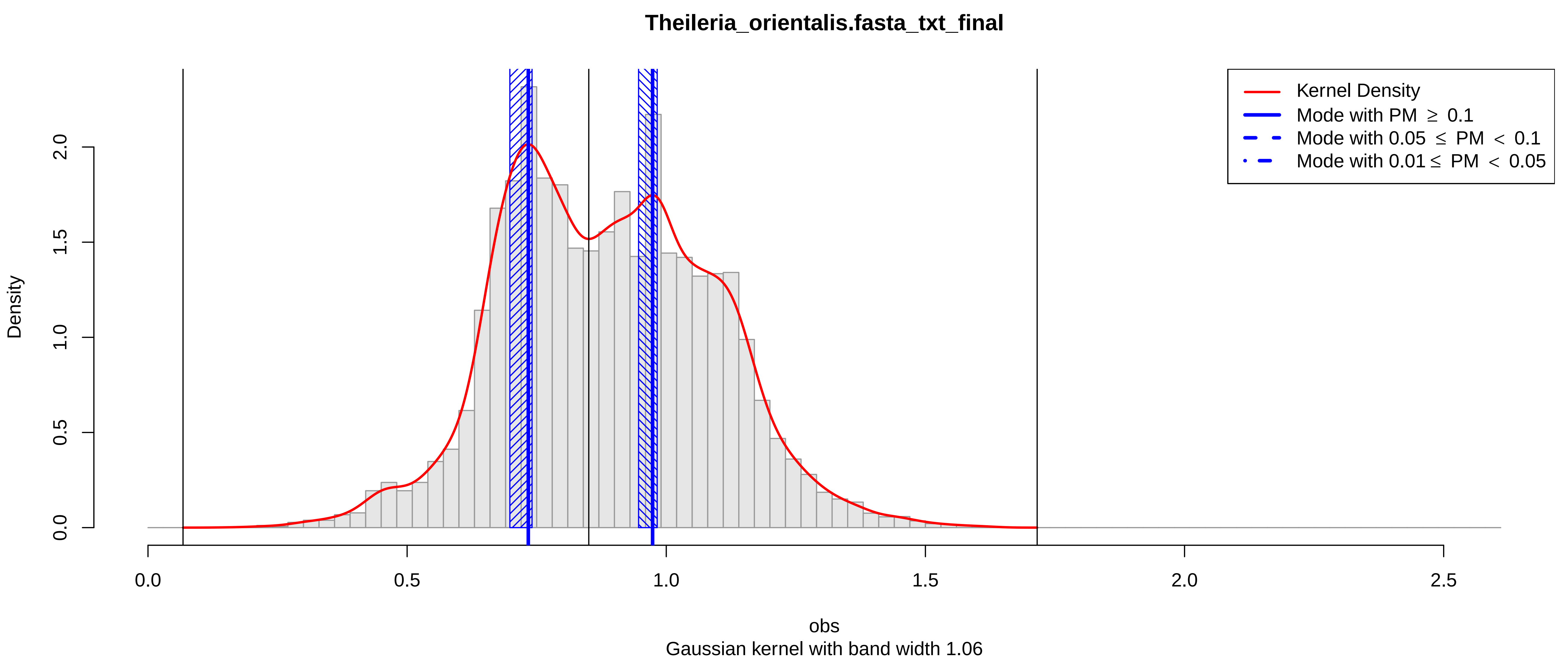

Theileria_orientalis.fasta_txt_final
Kernel Density
Mode with PM ≥ 0.1
Mode with 0.05 ≤ PM < 0.1
Mode with 0.01 ≤ PM < 0.05
Density
0.0
0.5
1.0
1.5
2.0
2.5
obs
Gaussian kernel with band width 1.06

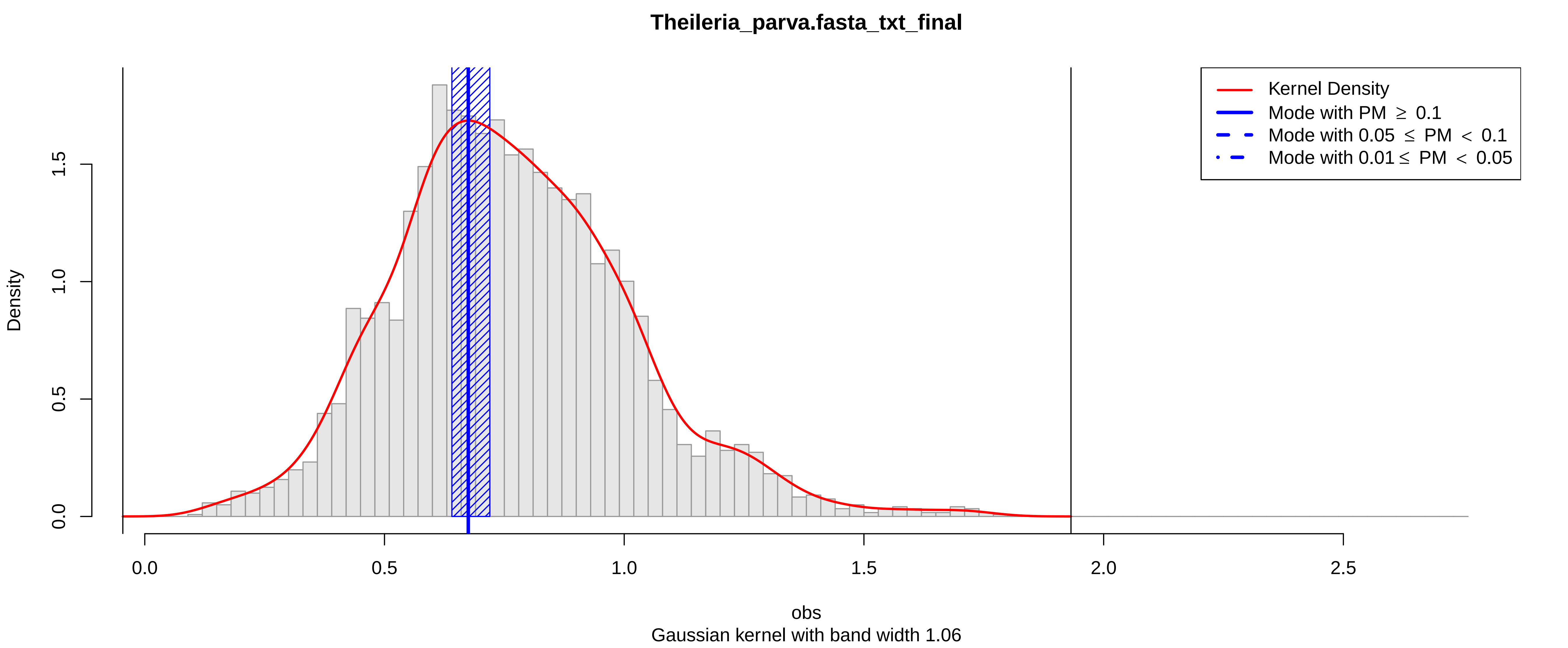

Theileria_parva.fasta_txt_final
Kernel Density
Mode with PM ≥ 0.1
Mode with 0.05 ≤ PM < 0.1
Mode with 0.01 ≤ PM < 0.05
Density
0.0
0.5
1.0
1.5
0.0
0.5
1.0
1.5
2.0
2.5
obs
Gaussian kernel with band width 1.06

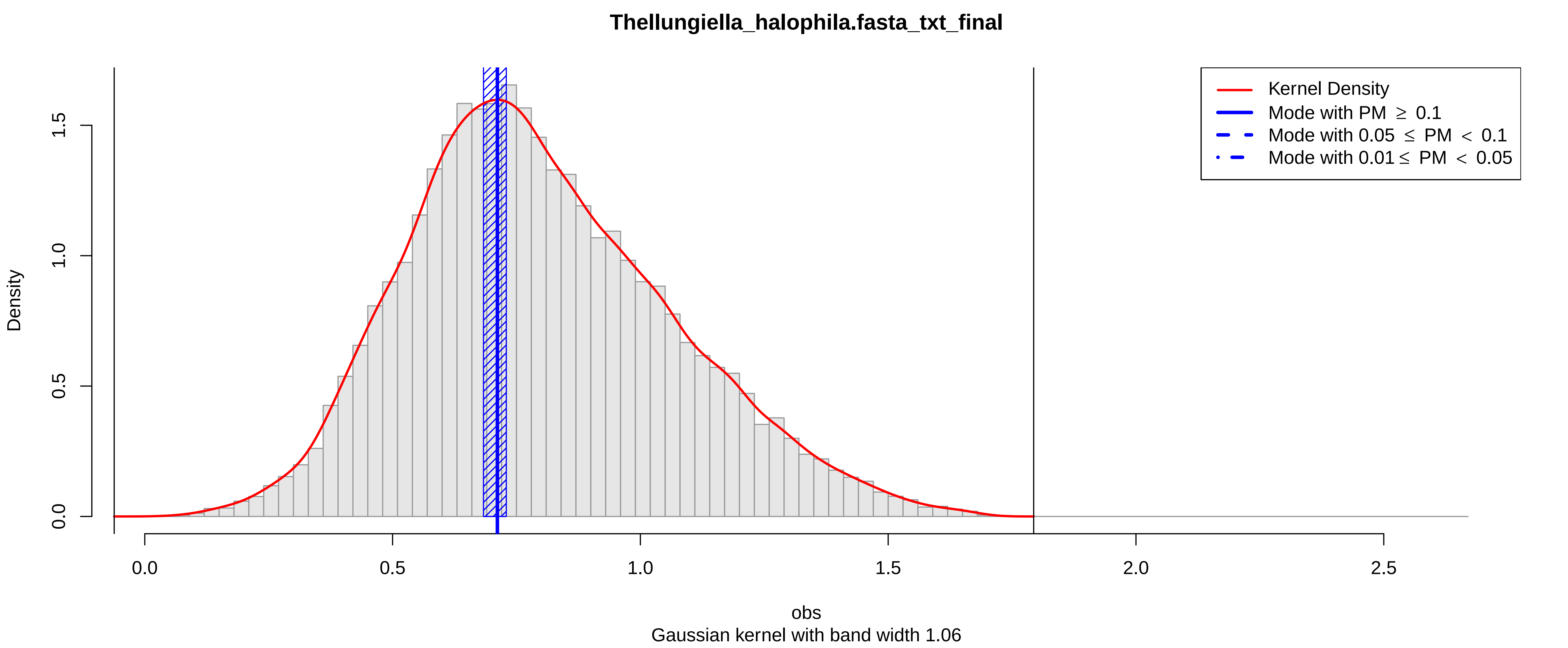
Thellungiella_halophila.fasta_txt_final
Kernel Density
Mode with PM ≥ 0.1
Mode with 0.05 ≤ PM < 0.1
Mode with 0.01 ≤ PM < 0.05
Density
0.0
0.5
1.0
1.5
0.0
0.5
1.0
1.5
2.0
2.5
obs
Gaussian kernel with band width 1.06

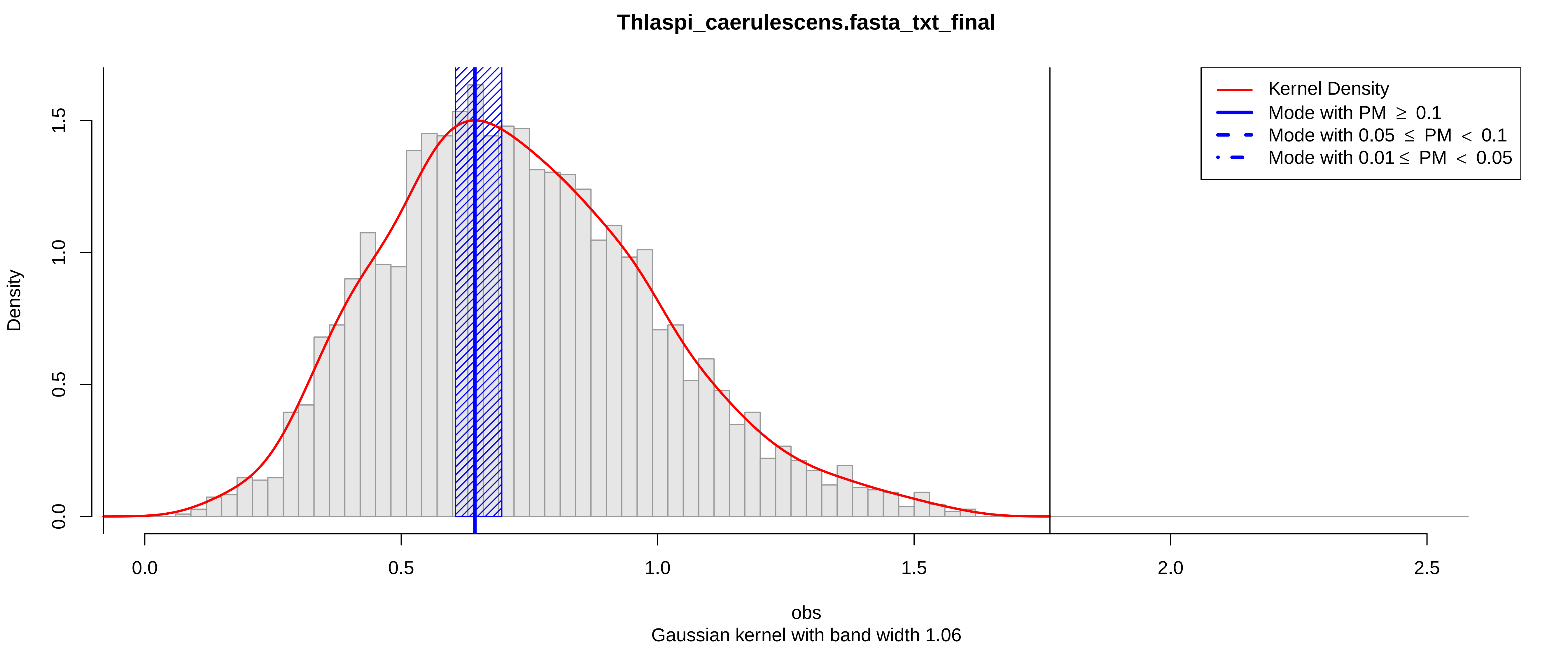
Thlaspi_caerulescens.fasta_txt_final
Kernel Density
Mode with PM ≥ 0.1
Mode with 0.05 ≤ PM < 0.1
Mode with 0.01 ≤ PM < 0.05
Density
0.0
0.5
1.0
1.5
0.0
0.5
1.0
1.5
2.0
2.5
obs
Gaussian kernel with band width 1.06

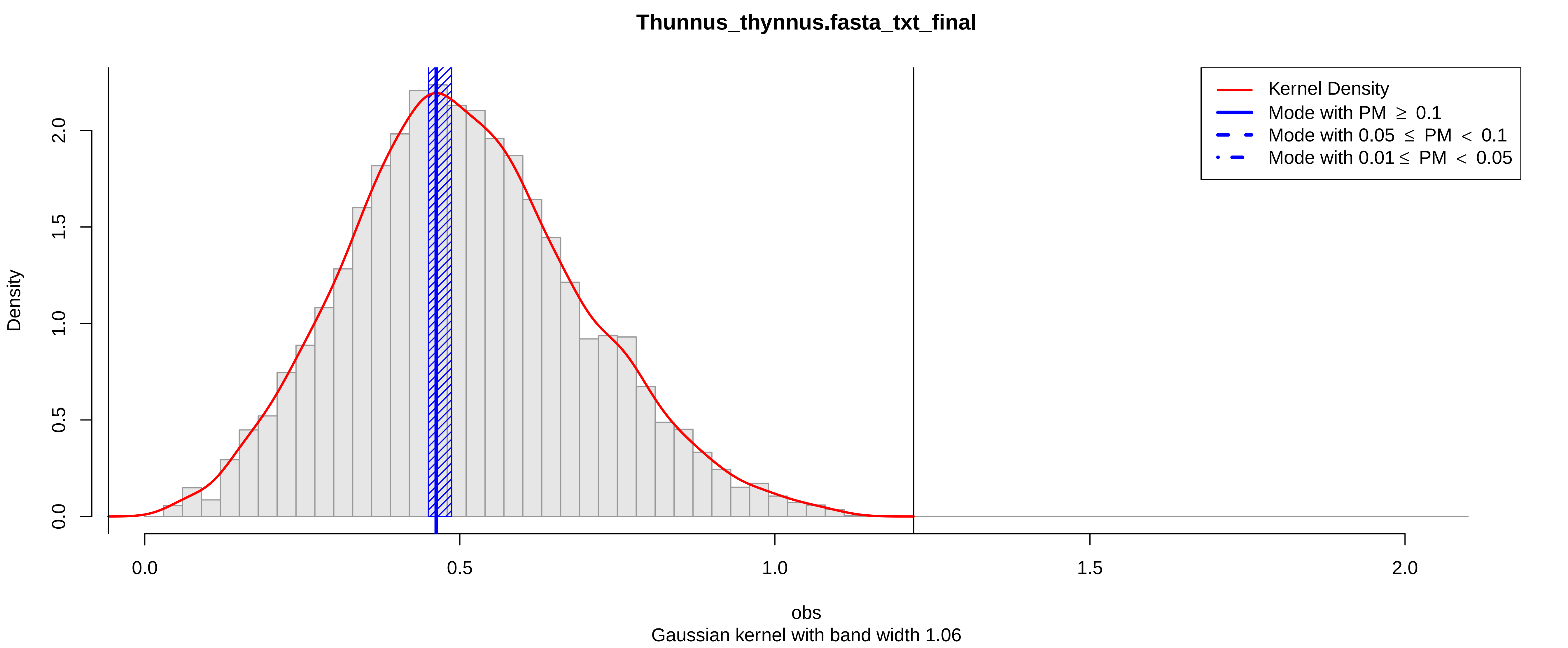

Thunnus_thynnus.fasta_txt_final
Kernel Density
Mode with PM ≥ 0.1
Mode with 0.05 ≤ PM < 0.1
Mode with 0.01 ≤ PM < 0.05
Density
0.0
0.5
1.0
1.5
2.0
0.0
0.5
1.0
1.5
2.0
obs
Gaussian kernel with band width 1.06

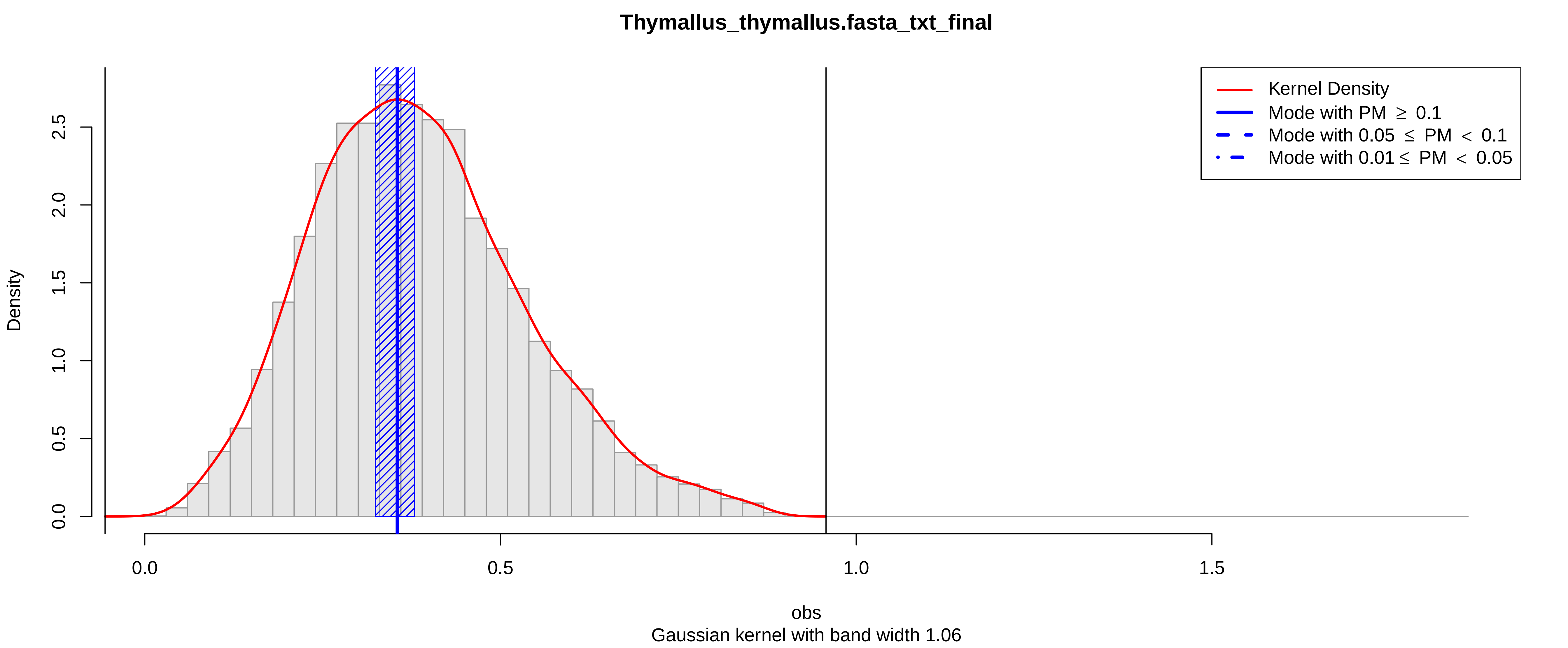
Thymallus_thymallus.fasta_txt_final
Kernel Density
Mode with PM ≥ 0.1
Mode with 0.05 ≤ PM < 0.1
Mode with 0.01 ≤ PM < 0.05
Density
0.0
0.5
1.0
1.5
2.0
2.5
0.0
0.5
1.0
1.5
obs
Gaussian kernel with band width 1.06

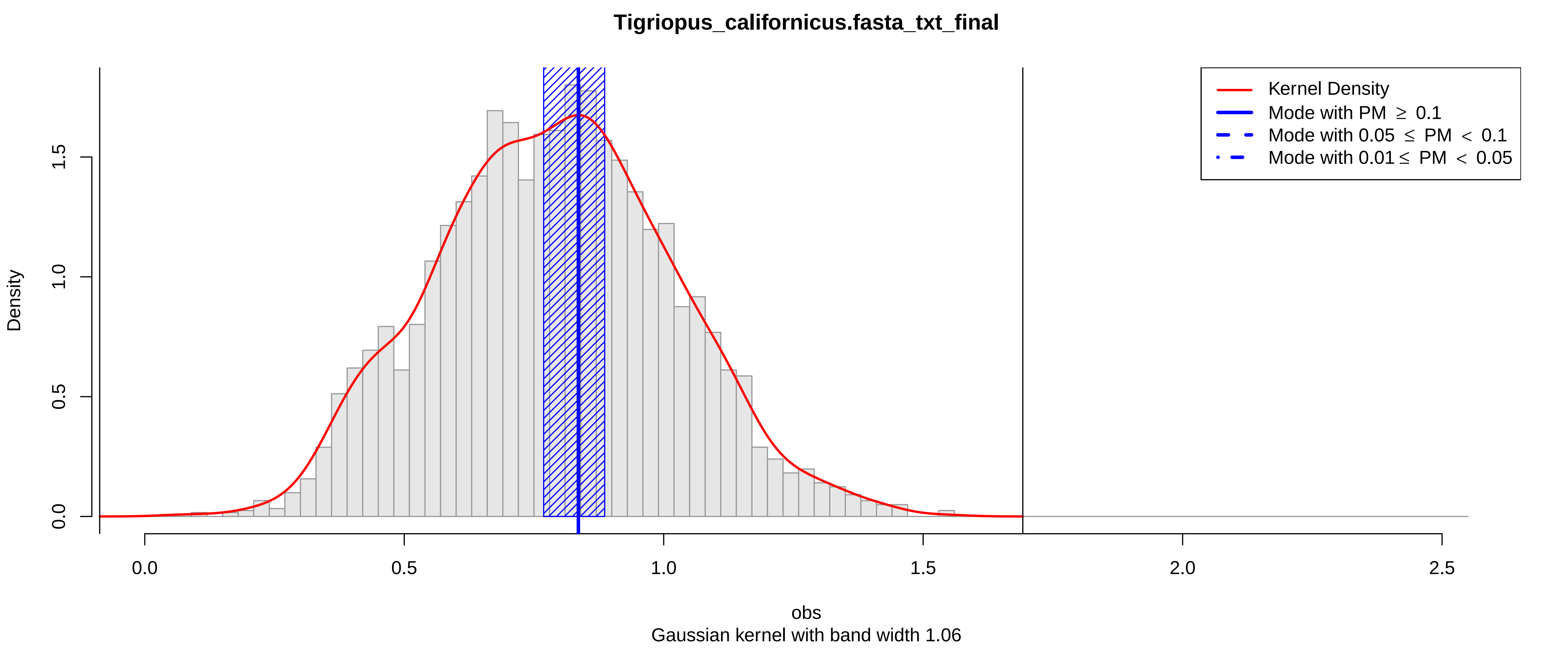
Tigriopus_californicus.fasta_txt_final
Kernel Density
Mode with PM ≥ 0.1
Mode with 0.05 ≤ PM < 0.1
Mode with 0.01 ≤ PM < 0.05
Density
0.0
0.5
1.0
1.5
0.0
0.5
1.0
1.5
2.0
2.5
obs
Gaussian kernel with band width 1.06

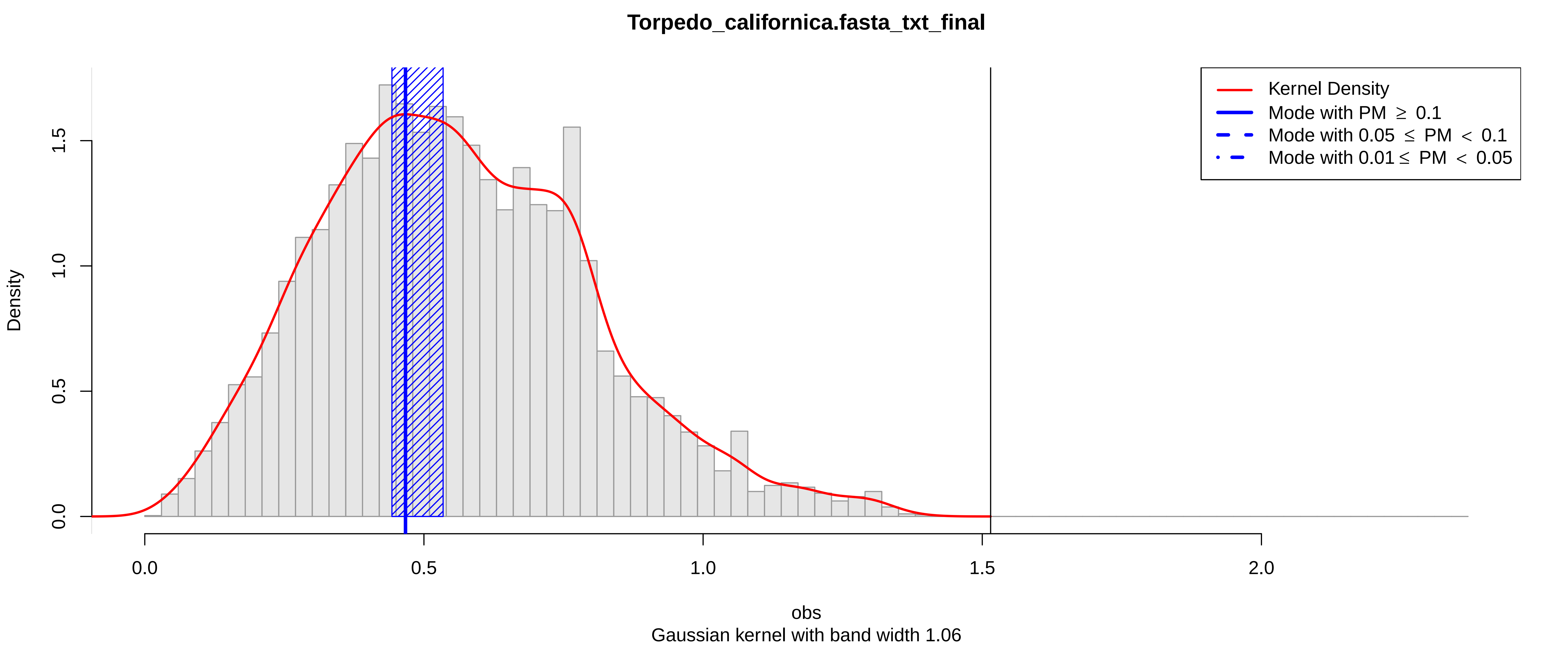
Torpedo_californica.fasta_txt_final
Kernel Density
Mode with PM ≥ 0.1
Mode with 0.05 ≤ PM < 0.1
Mode with 0.01 ≤ PM < 0.05
Density
0.0
0.5
1.0
1.5
0.0
0.5
1.0
1.5
2.0
obs
Gaussian kernel with band width 1.06

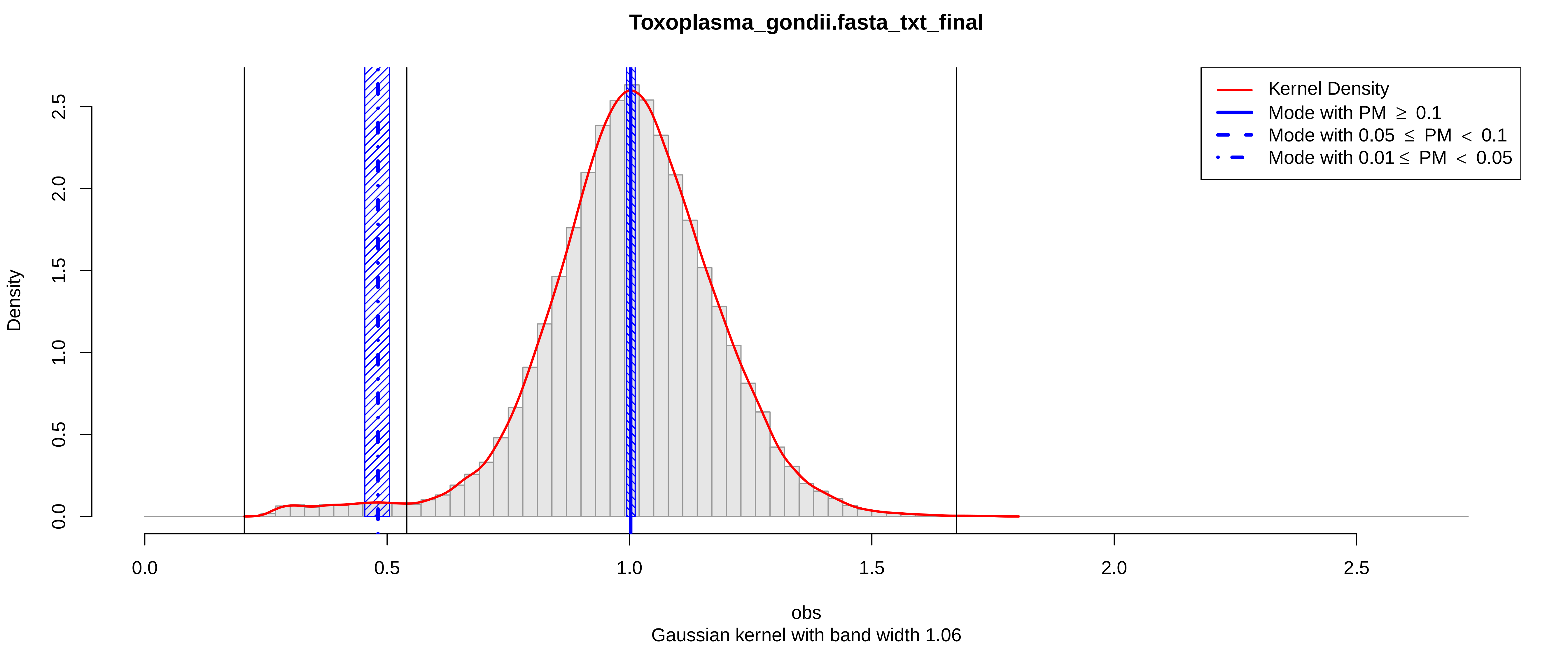

Toxoplasma_gondii.fasta_txt_final
Kernel Density
Mode with PM ≥ 0.1
Mode with 0.05 ≤ PM < 0.1
Mode with 0.01 ≤ PM < 0.05
Density
0.0
0.5
1.0
1.5
2.0
2.5
0.0
0.5
1.0
1.5
2.0
2.5
obs
Gaussian kernel with band width 1.06

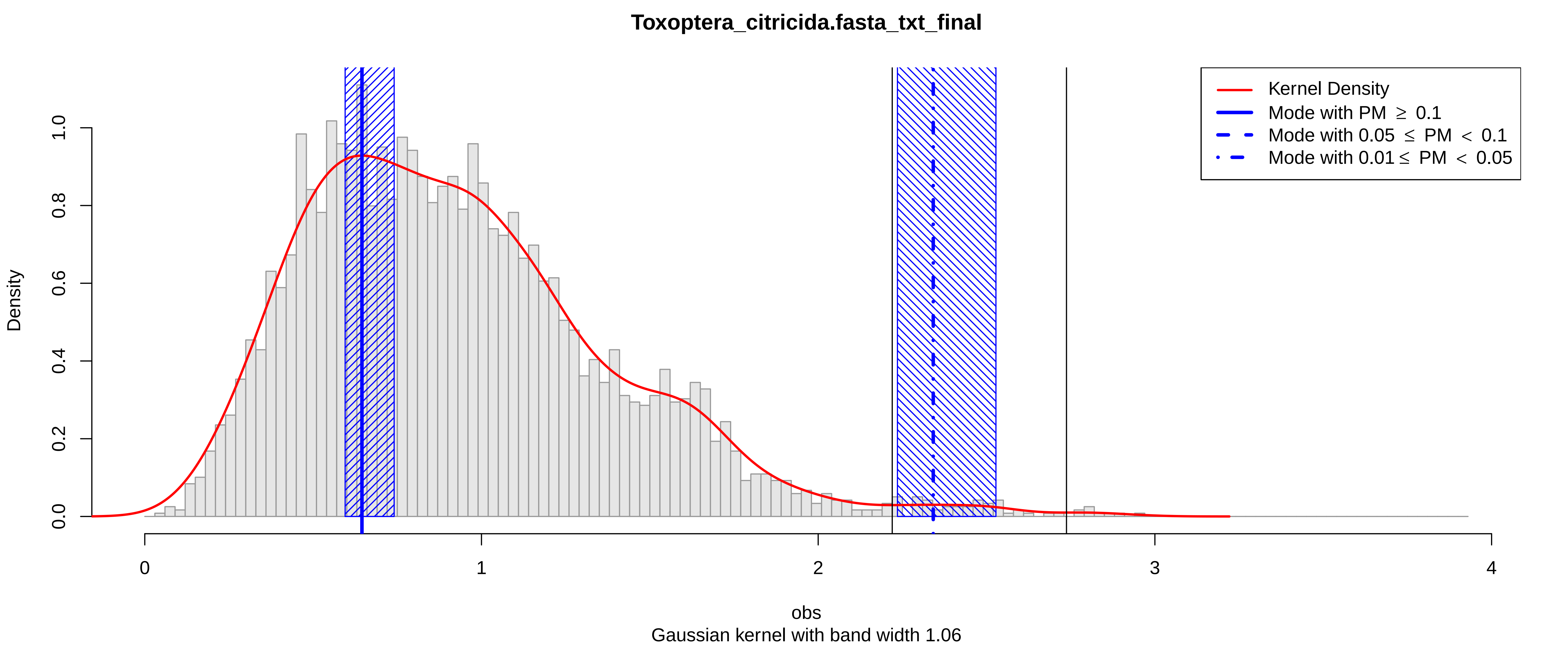
Toxoptera_citricida.fasta_txt_final
Kernel Density
Mode with PM ≥ 0.1
Mode with 0.05 ≤ PM < 0.1
Mode with 0.01 ≤ PM < 0.05
Density
0.0
0.2
0.4
0.6
0.8
1.0
0
1
2
3
4
obs
Gaussian kernel with band width 1.06

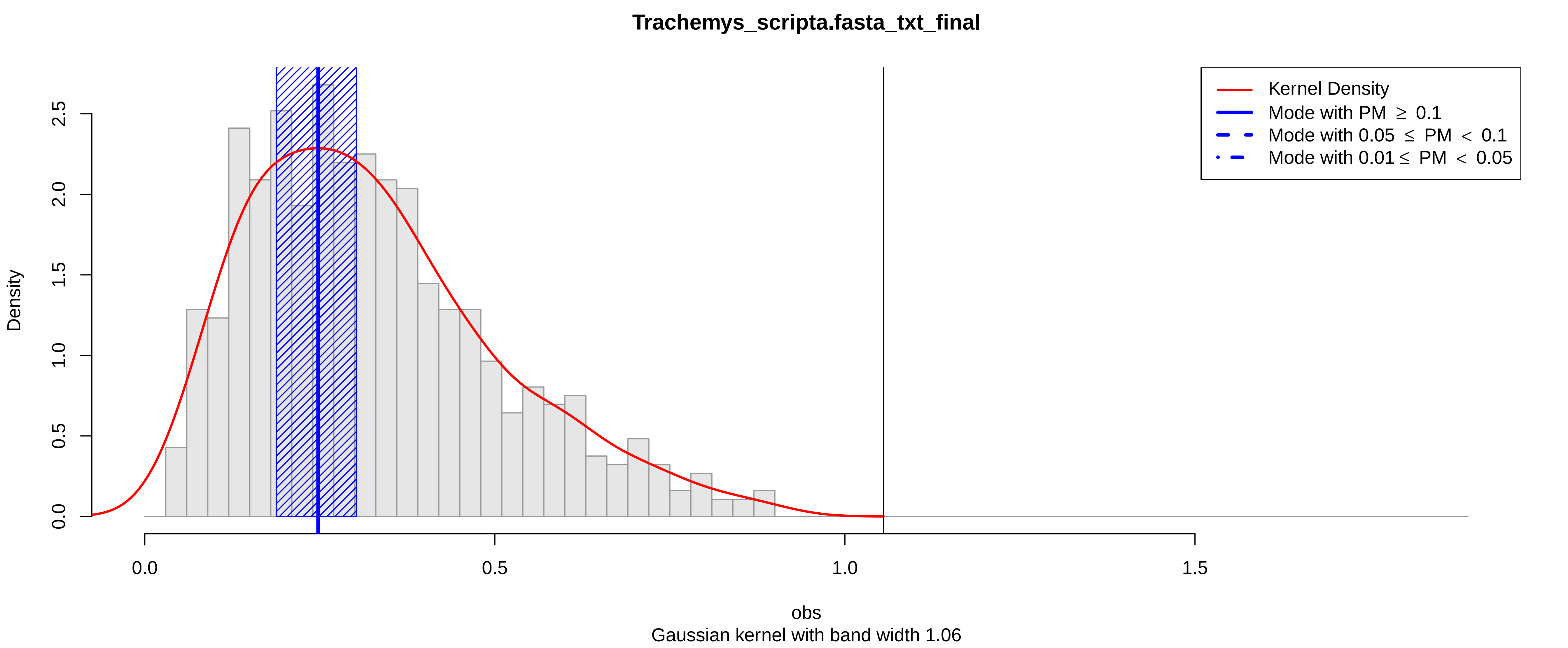
Trachemys_scripta.fasta_txt_final
Kernel Density
Mode with PM ≥ 0.1
Mode with 0.05 ≤ PM < 0.1
Mode with 0.01 ≤ PM < 0.05
Density
0.0
0.5
1.0
1.5
2.0
2.5
0.0
0.5
1.0
1.5
obs
Gaussian kernel with band width 1.06

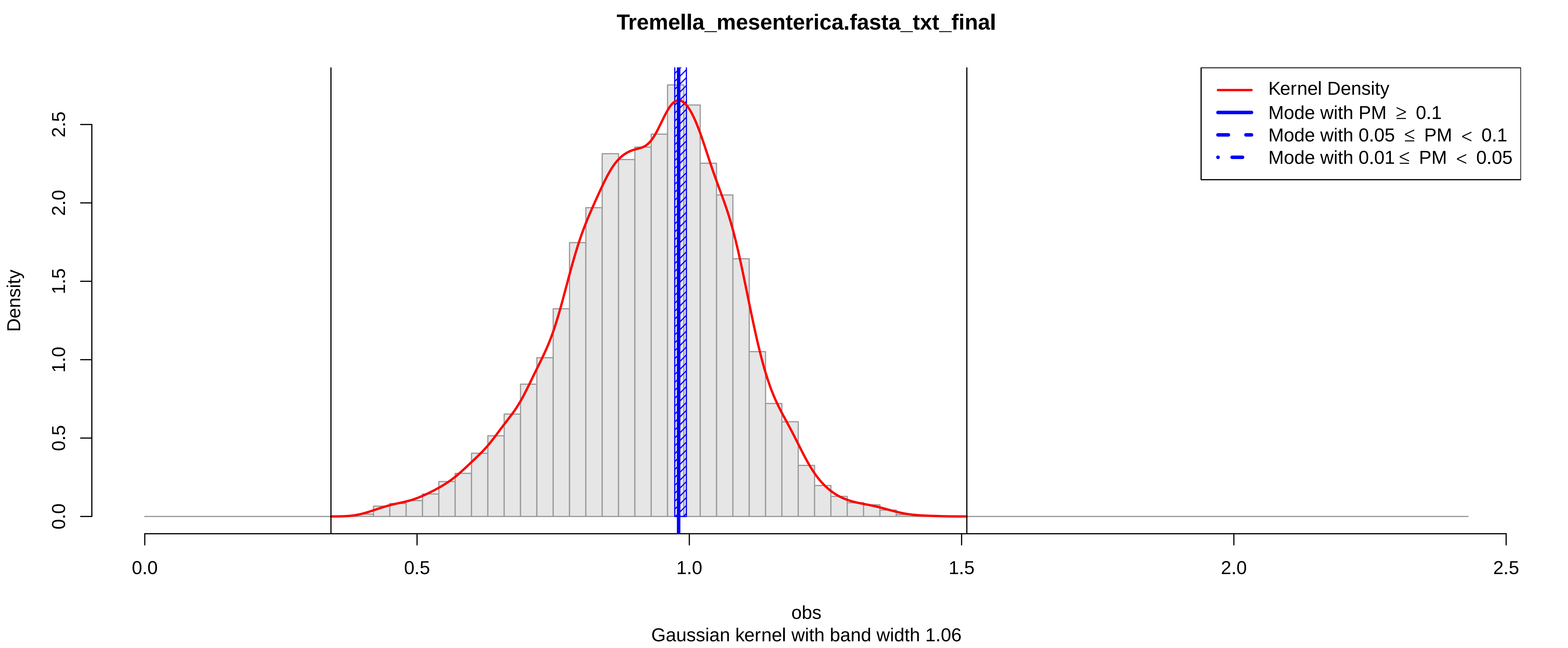
Tremella_mesenterica.fasta_txt_final
Kernel Density
Mode with PM ≥ 0.1
Mode with 0.05 ≤ PM < 0.1
Mode with 0.01 ≤ PM < 0.05
Density
0.0
0.5
1.0
1.5
2.0
2.5
obs
Gaussian kernel with band width 1.06

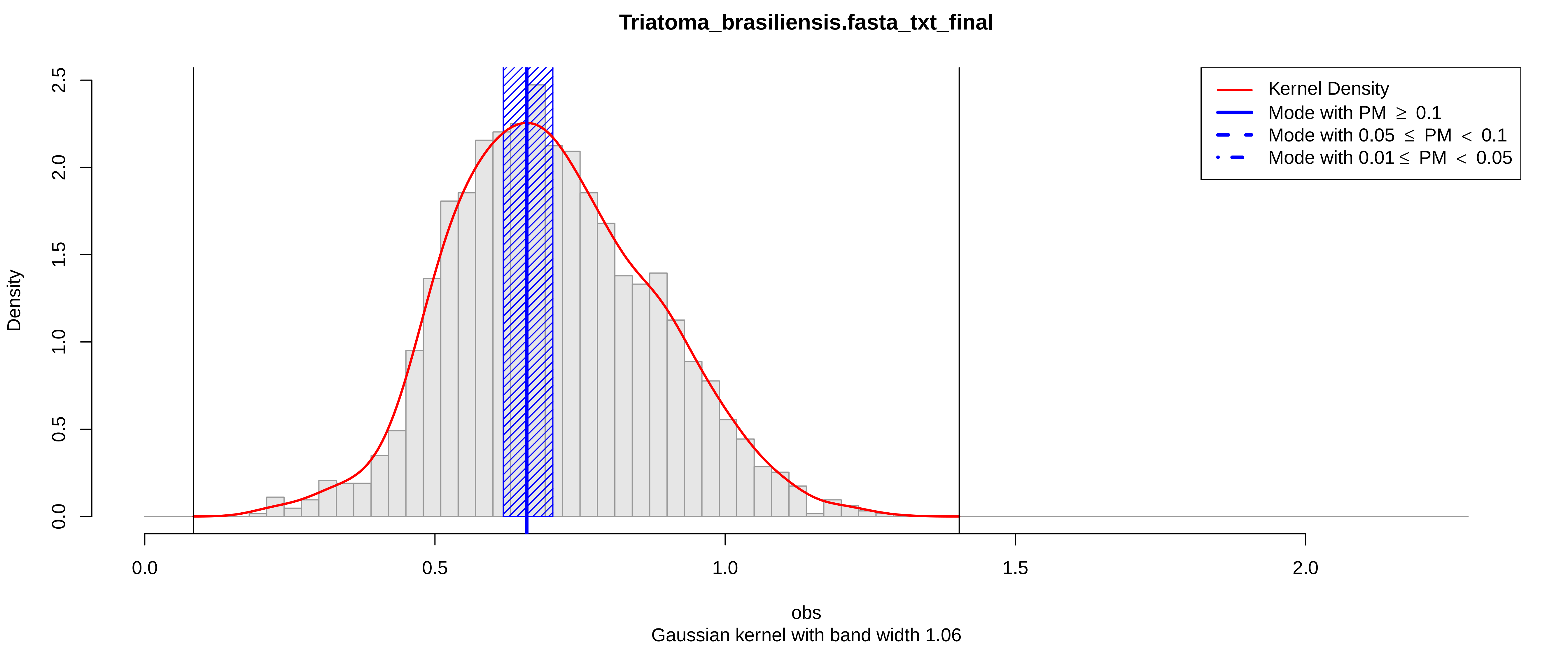
Triatoma_brasiliensis.fasta_txt_final
Kernel Density
Mode with PM ≥ 0.1
Mode with 0.05 ≤ PM < 0.1
Mode with 0.01 ≤ PM < 0.05
Density
0.0
0.5
1.0
1.5
2.0
2.5
0.0
0.5
1.0
1.5
2.0
obs
Gaussian kernel with band width 1.06

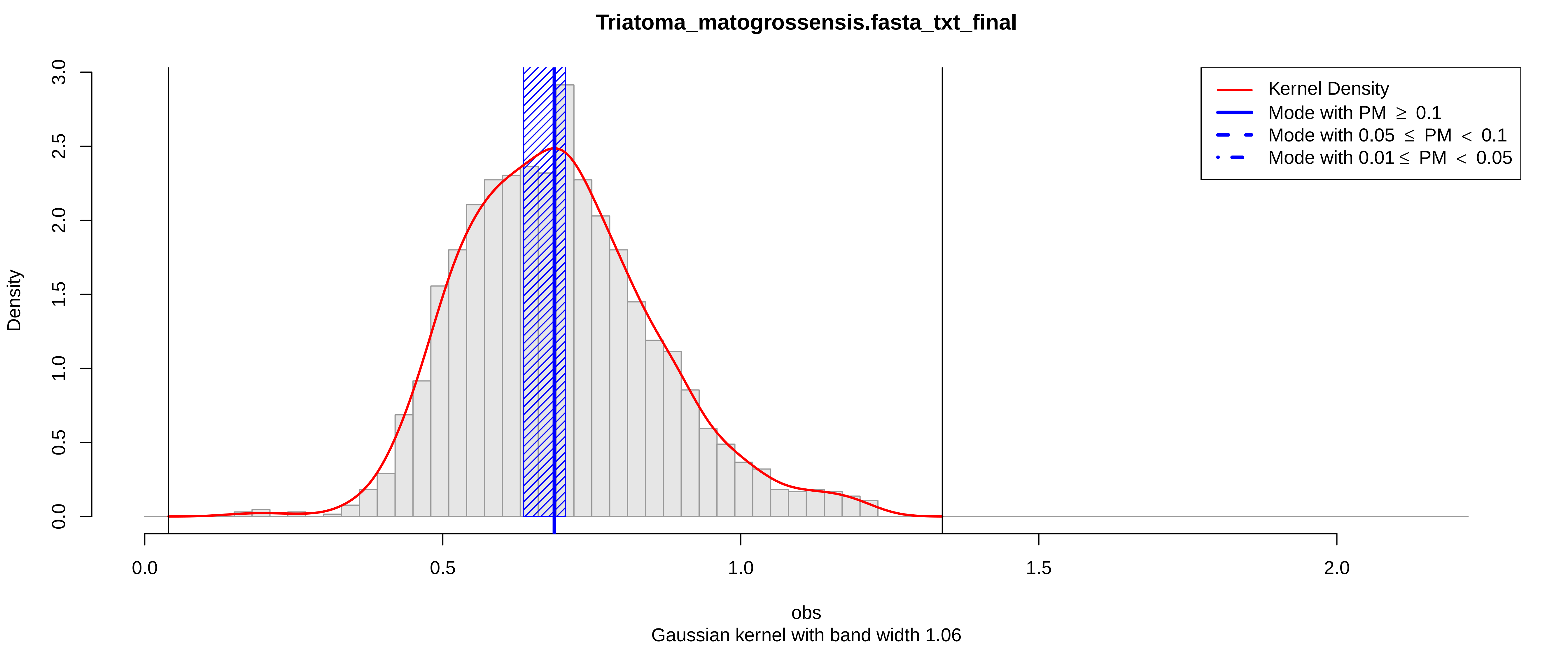
Triatoma_matogrossensis.fasta_txt_final
Kernel Density
Mode with PM ≥ 0.1
Mode with 0.05 ≤ PM < 0.1
Mode with 0.01 ≤ PM < 0.05
Density
0.0
0.5
1.0
1.5
2.0
2.5
3.0
obs
Gaussian kernel with band width 1.06

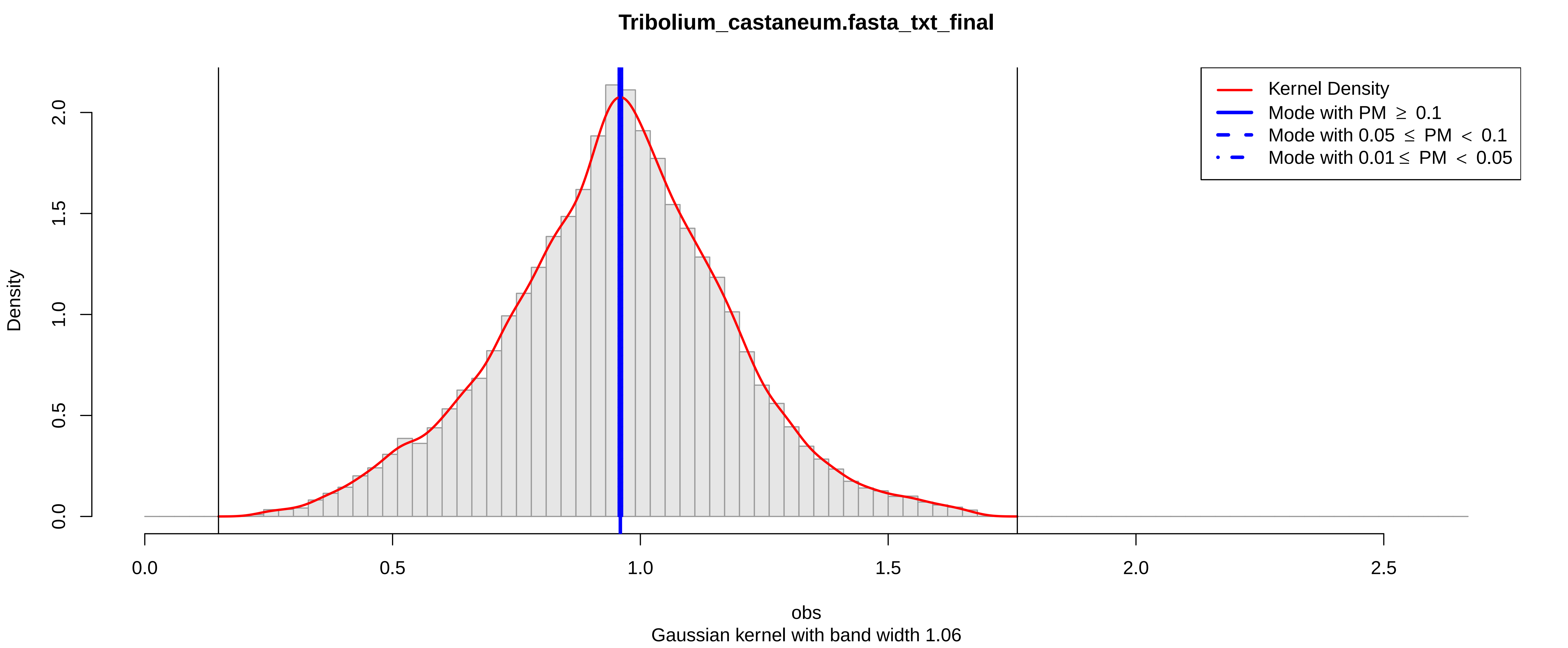
Tribolium_castaneum.fasta_txt_final
Kernel Density
Mode with PM ≥ 0.1
Mode with 0.05 ≤ PM < 0.1
Mode with 0.01 ≤ PM < 0.05
Density
0.0
0.5
1.0
1.5
2.0
0.0
0.5
1.0
1.5
2.0
2.5
obs
Gaussian kernel with band width 1.06

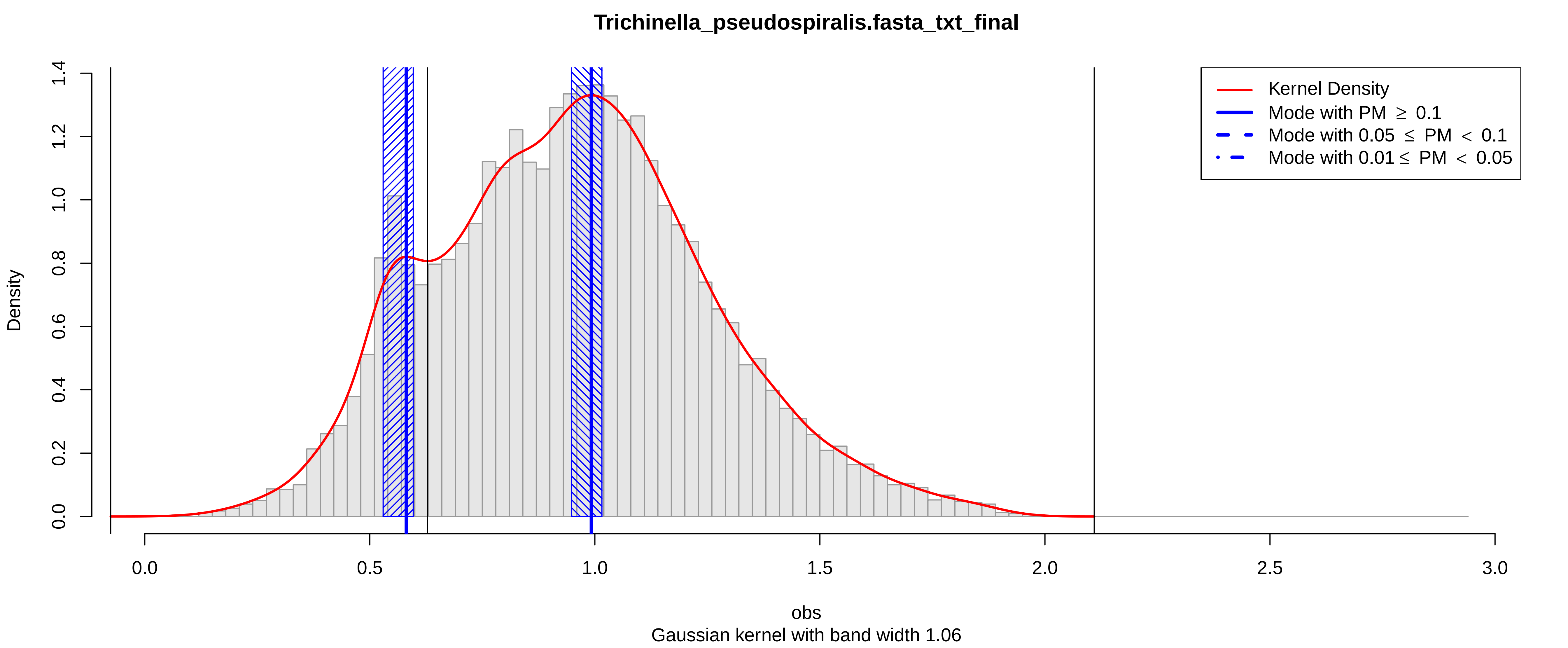
Trichinella_pseudospiralis.fasta_txt_final
Kernel Density
Mode with PM ≥ 0.1
Mode with 0.05 ≤ PM < 0.1
Mode with 0.01 ≤ PM < 0.05
Density
0.0
0.2
0.4
0.6
0.8
1.0
1.2
1.4
0.0
0.5
1.0
1.5
2.0
2.5
3.0
obs
Gaussian kernel with band width 1.06

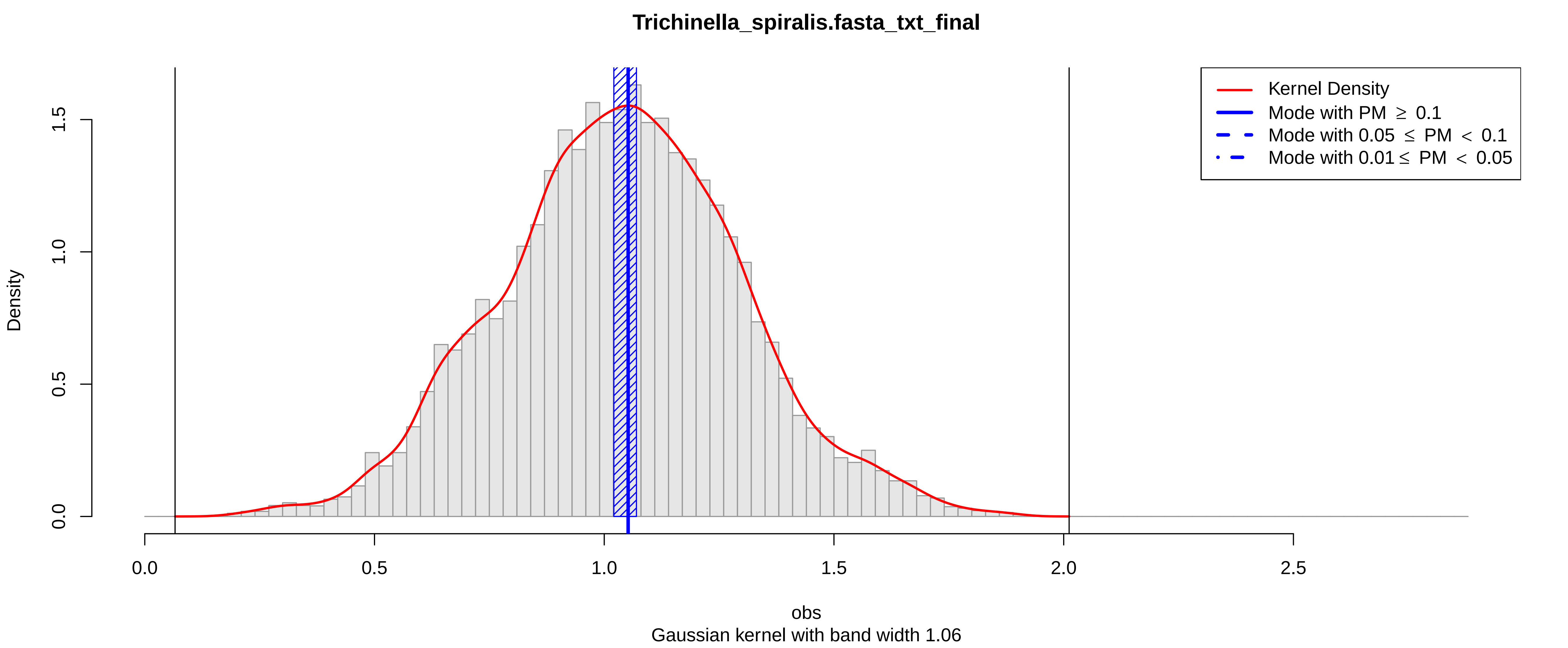
Trichinella_spiralis.fasta_txt_final
Kernel Density
Mode with PM ≥ 0.1
Mode with 0.05 ≤ PM < 0.1
Mode with 0.01 ≤ PM < 0.05
Density
0.0
0.5
1.0
1.5
0.0
0.5
1.0
1.5
2.0
2.5
obs
Gaussian kernel with band width 1.06

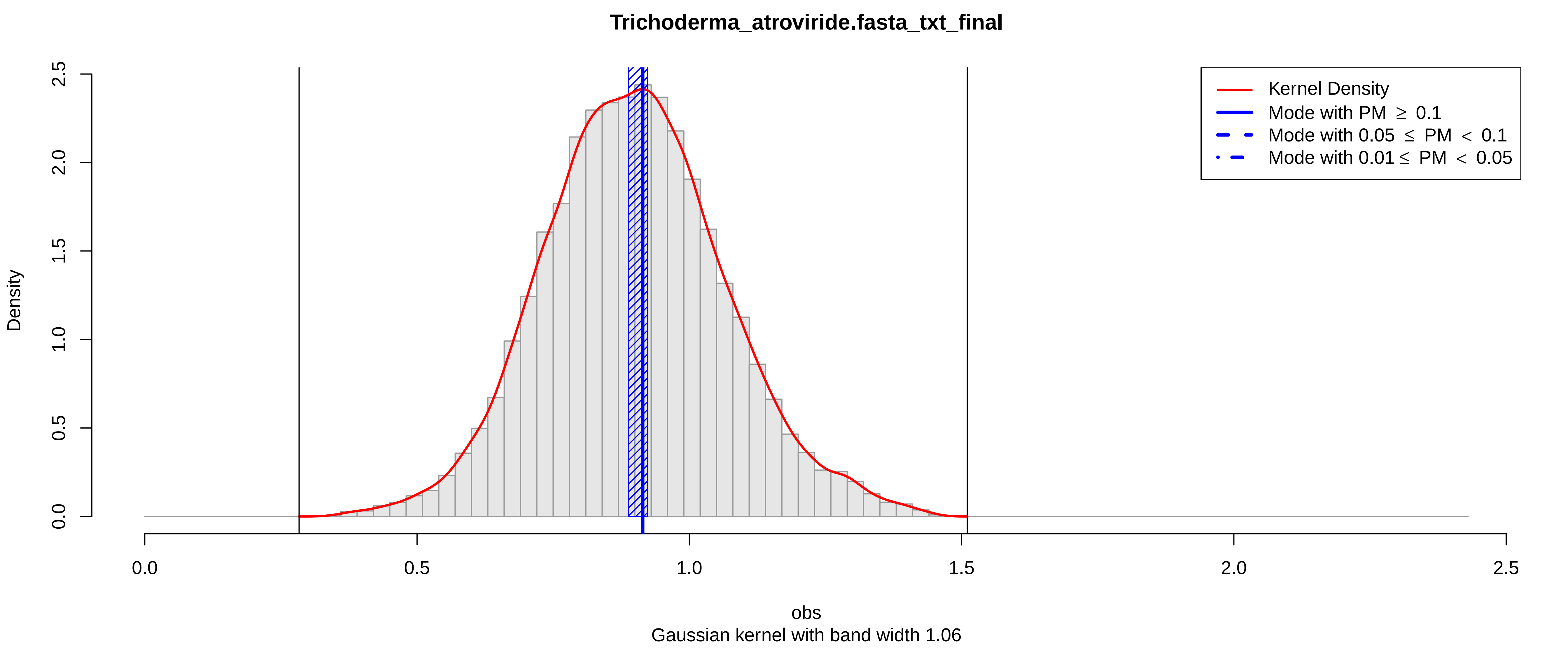
Trichoderma_atroviride.fasta_txt_final
Kernel Density
Mode with PM ≥ 0.1
Mode with 0.05 ≤ PM < 0.1
Mode with 0.01 ≤ PM < 0.05
Density
0.0
0.5
1.0
1.5
2.0
2.5
obs
Gaussian kernel with band width 1.06

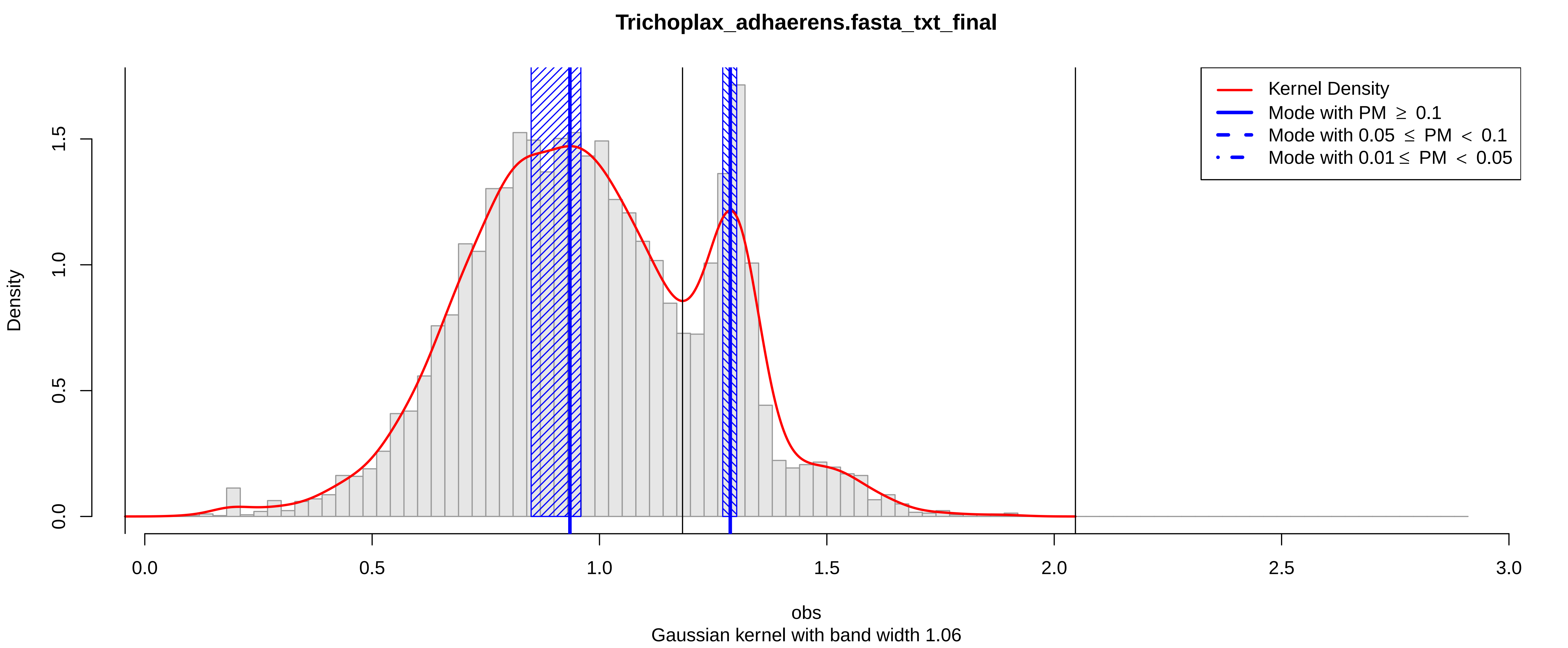

Trichoplax_adhaerens.fasta_txt_final
Kernel Density
Mode with PM ≥ 0.1
Mode with 0.05 ≤ PM < 0.1
Mode with 0.01 ≤ PM < 0.05
Density
0.0
0.5
1.0
1.5
0.0
0.5
1.0
1.5
2.0
2.5
3.0
obs
Gaussian kernel with band width 1.06

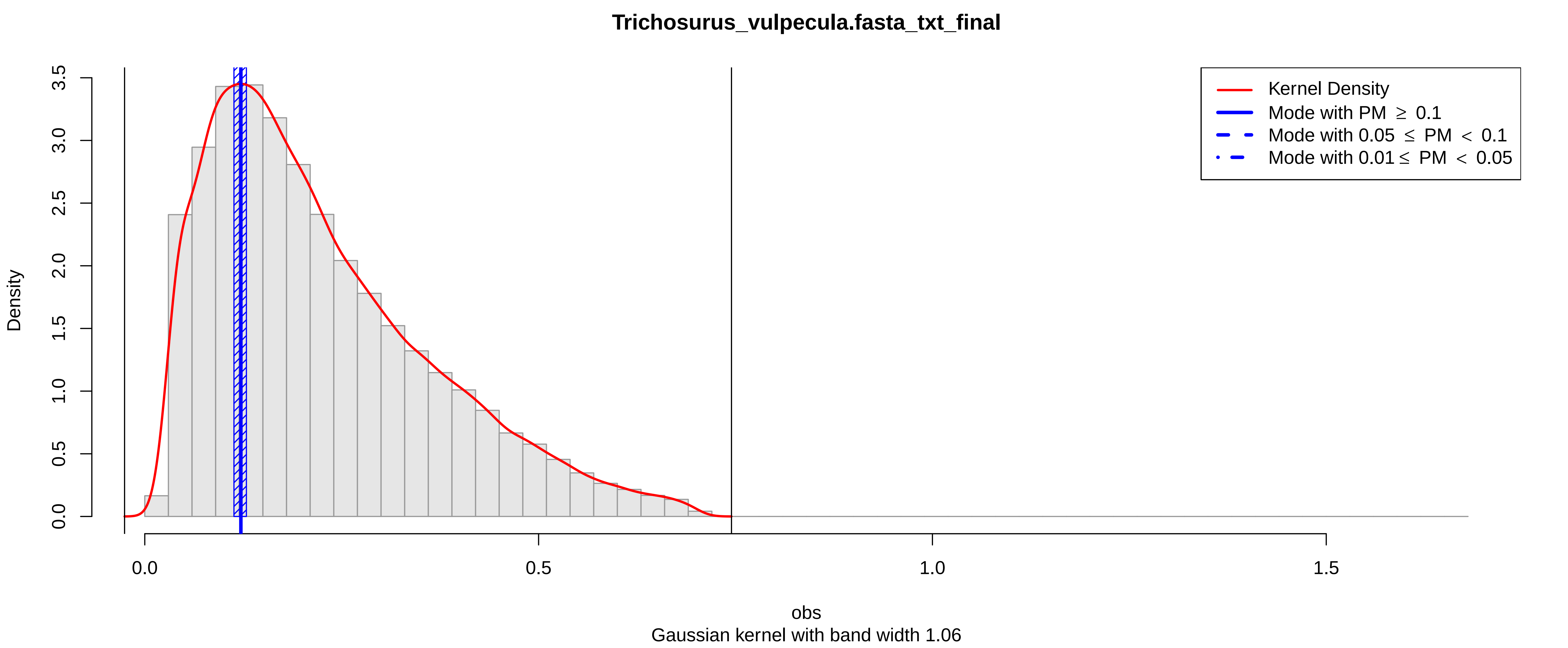
Trichosurus_vulpecula.fasta_txt_final
Kernel Density
Mode with PM ≥ 0.1
Mode with 0.05 ≤ PM < 0.1
Mode with 0.01 ≤ PM < 0.05
Density
0.0
0.5
1.0
1.5
2.0
2.5
3.0
3.5
0.0
0.5
1.0
1.5
obs
Gaussian kernel with band width 1.06

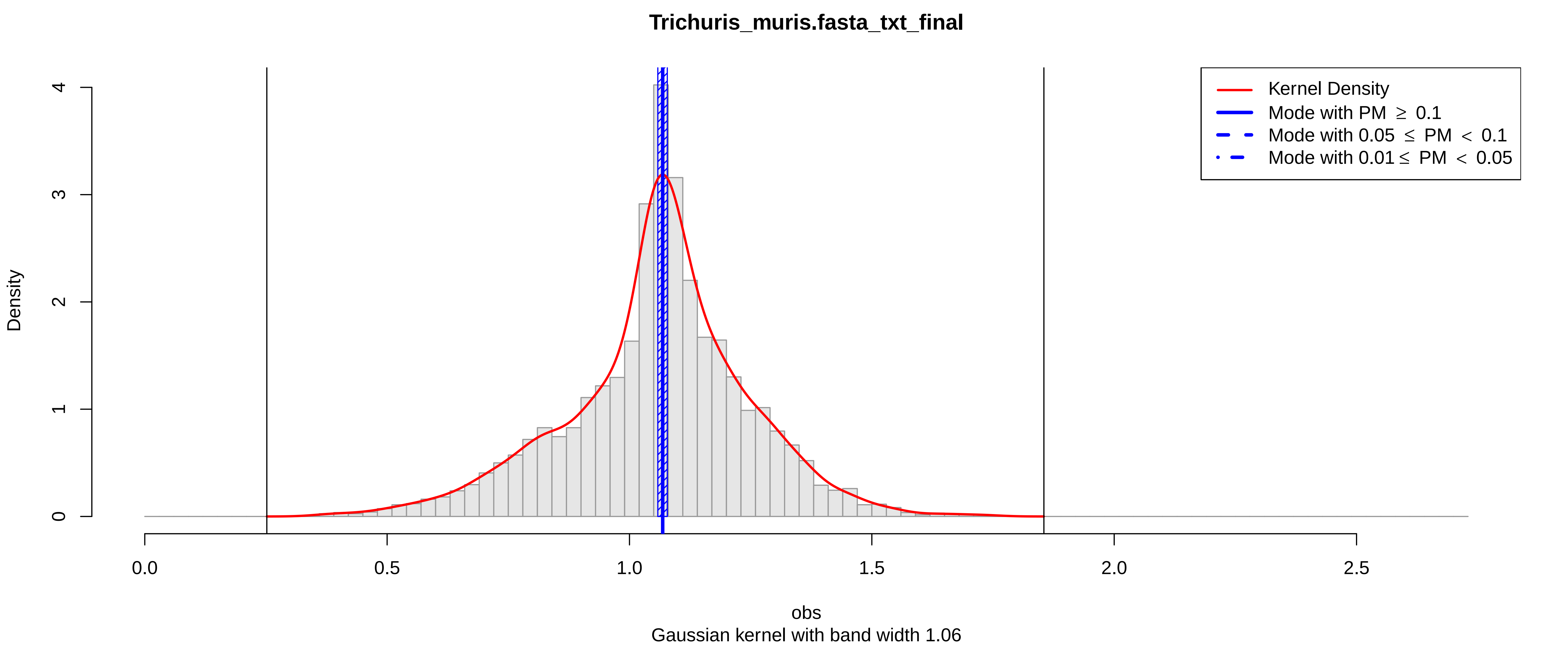
Trichuris_muris.fasta_txt_final
Kernel Density
Mode with PM ≥ 0.1
Mode with 0.05 ≤ PM < 0.1
Mode with 0.01 ≤ PM < 0.05
Density
0
1
2
3
4
0.0
0.5
1.0
1.5
2.0
2.5
obs
Gaussian kernel with band width 1.06

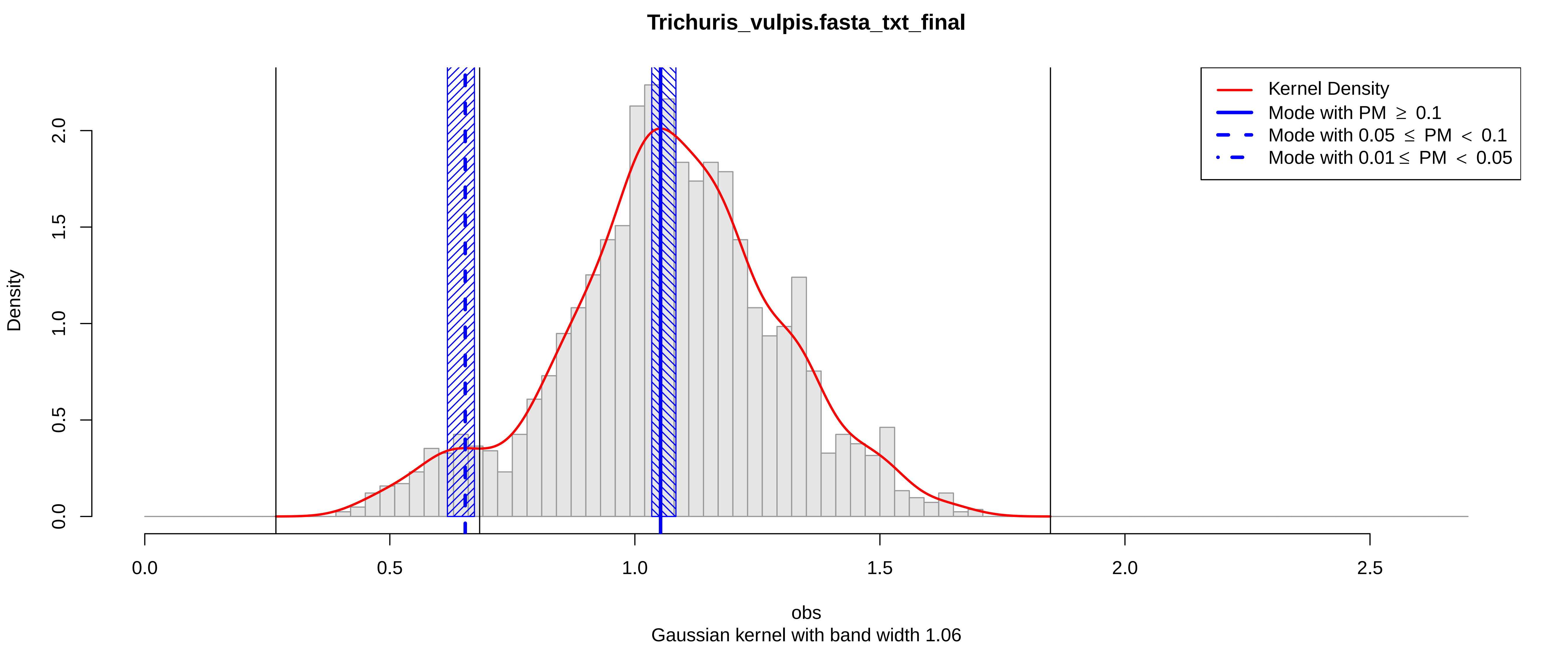

Trichuris_vulpis.fasta_txt_final
Kernel Density
Mode with PM ≥ 0.1
Mode with 0.05 ≤ PM < 0.1
Mode with 0.01 ≤ PM < 0.05
Density
0.0
0.5
1.0
1.5
2.0
0.0
0.5
1.0
1.5
2.0
2.5
obs
Gaussian kernel with band width 1.06

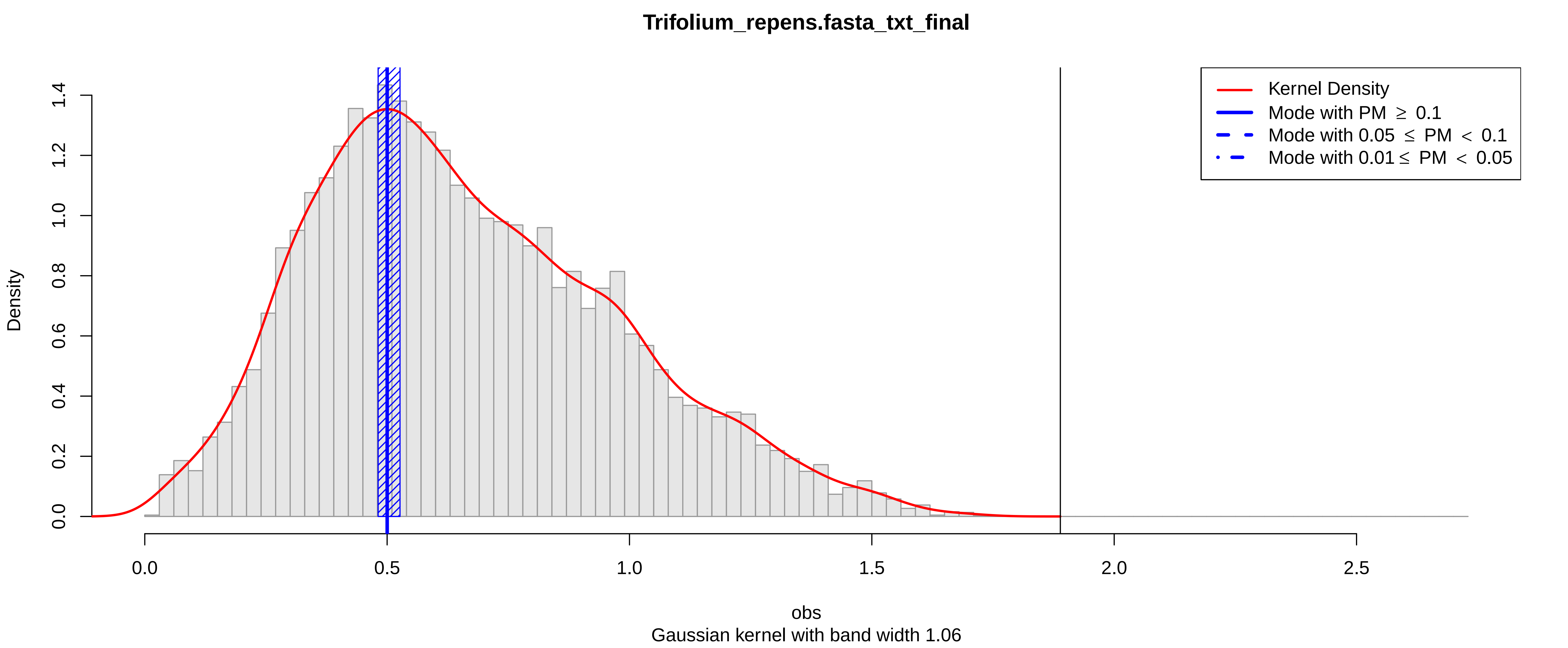
Trifolium_repens.fasta_txt_final
Kernel Density
Mode with PM ≥ 0.1
Mode with 0.05 ≤ PM < 0.1
Mode with 0.01 ≤ PM < 0.05
Density
0.0
0.2
0.4
0.6
0.8
1.0
1.2
1.4
0.0
0.5
1.0
1.5
2.0
2.5
obs
Gaussian kernel with band width 1.06

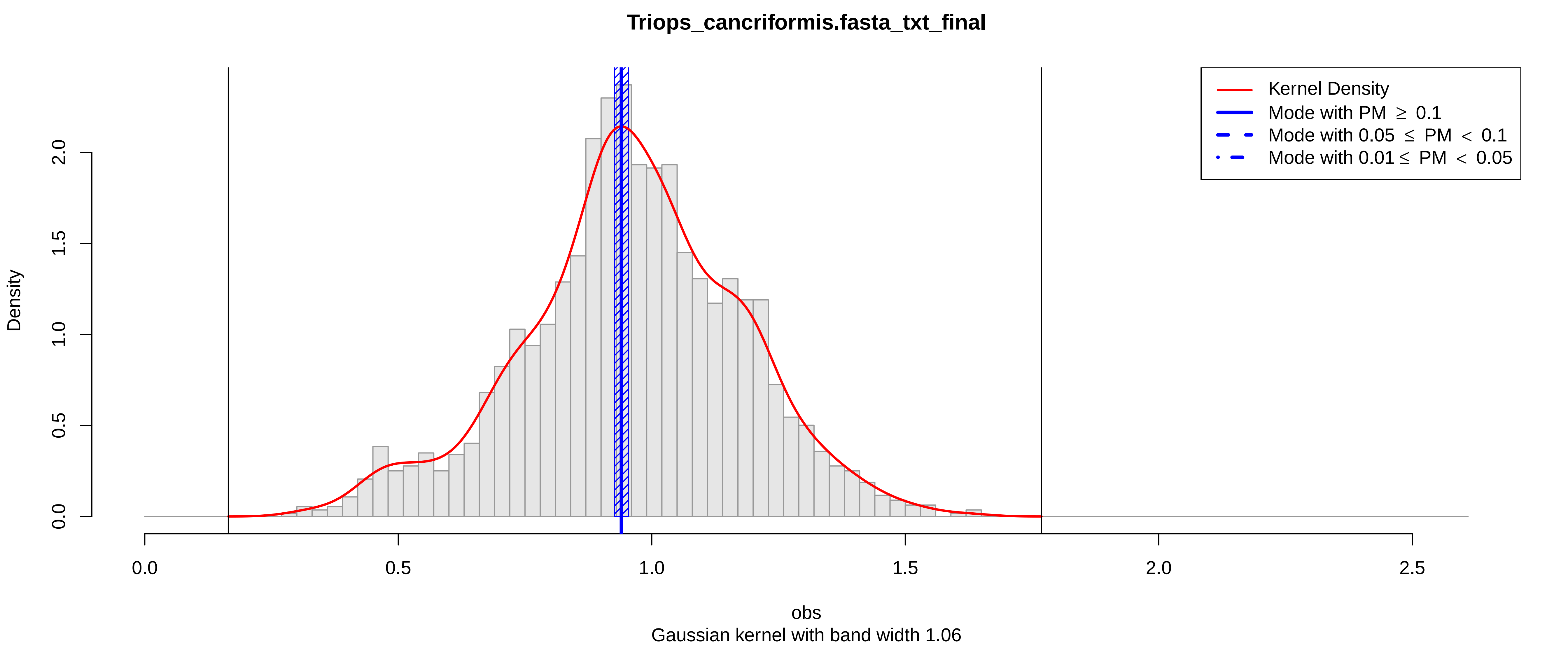

Triops_cancriformis.fasta_txt_final
Kernel Density
Mode with PM ≥ 0.1
Mode with 0.05 ≤ PM < 0.1
Mode with 0.01 ≤ PM < 0.05
Density
0.0
0.5
1.0
1.5
2.0
0.0
0.5
1.0
1.5
2.0
2.5
obs
Gaussian kernel with band width 1.06

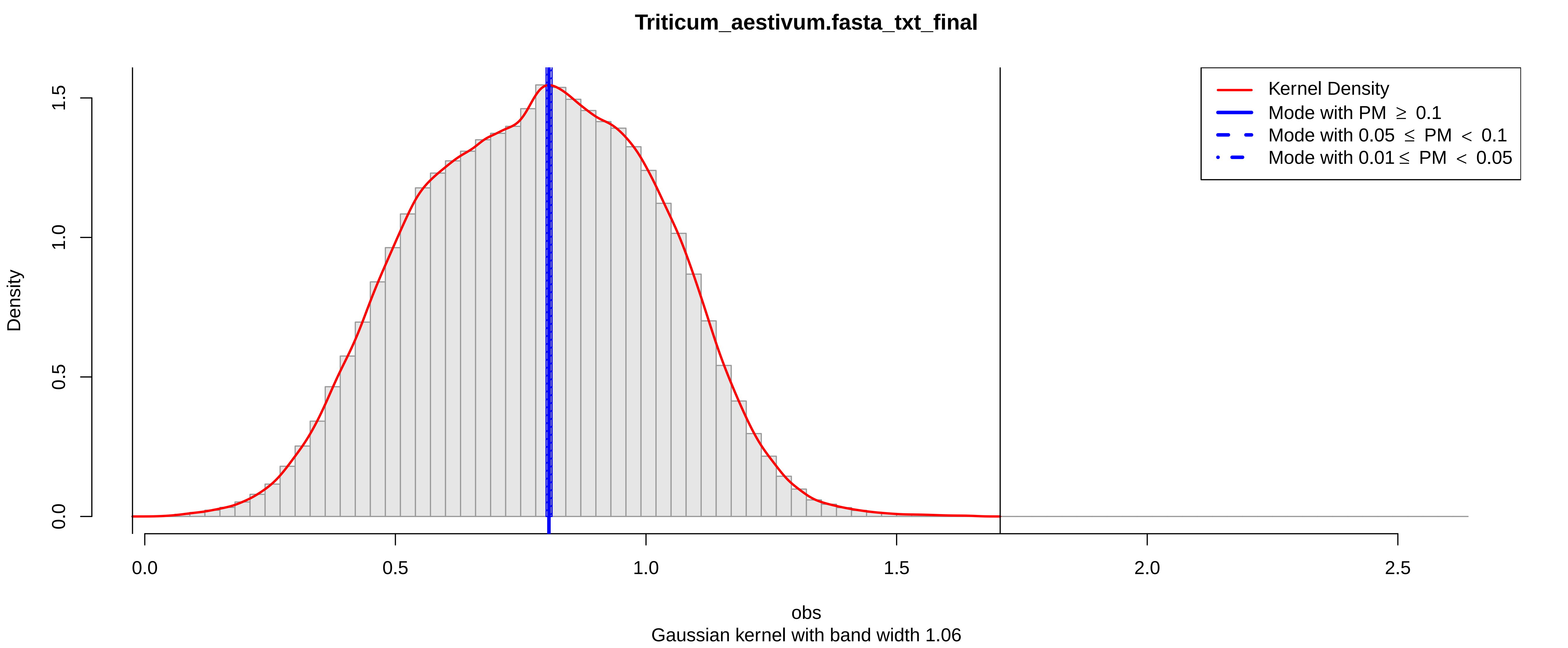
Triticum_aestivum.fasta_txt_final
Density
obs
Gaussian kernel with band width 1.06
Kernel Density
Mode with PM ≥ 0.1
Mode with 0.05 ≤ PM < 0.1
Mode with 0.01 ≤ PM < 0.05
0.0
0.5
1.0
1.5
2.0
2.5

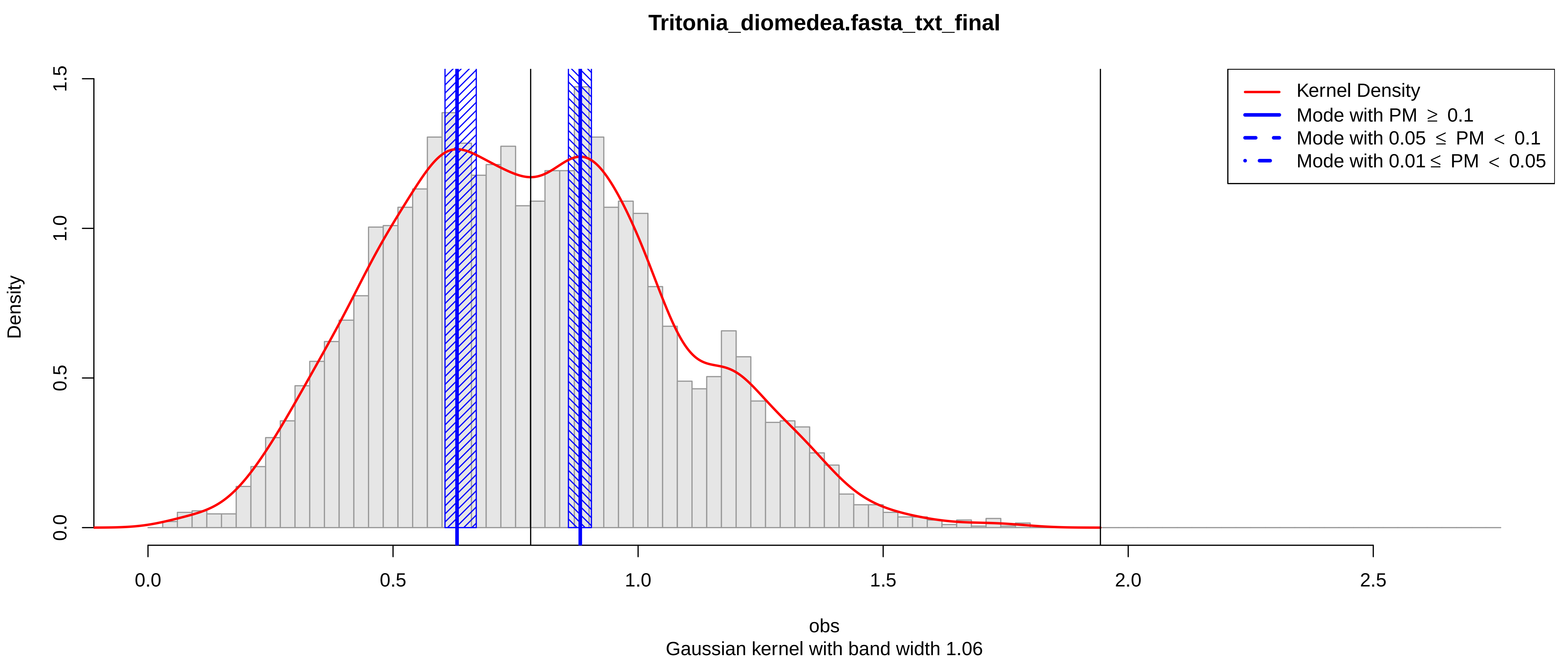
Tritonia_diomedea.fasta_txt_final
Kernel Density
Mode with PM ≥ 0.1
Mode with 0.05 ≤ PM < 0.1
Mode with 0.01 ≤ PM < 0.05
Density
0.0
0.5
1.0
1.5
0.0
0.5
1.0
1.5
2.0
2.5
obs
Gaussian kernel with band width 1.06

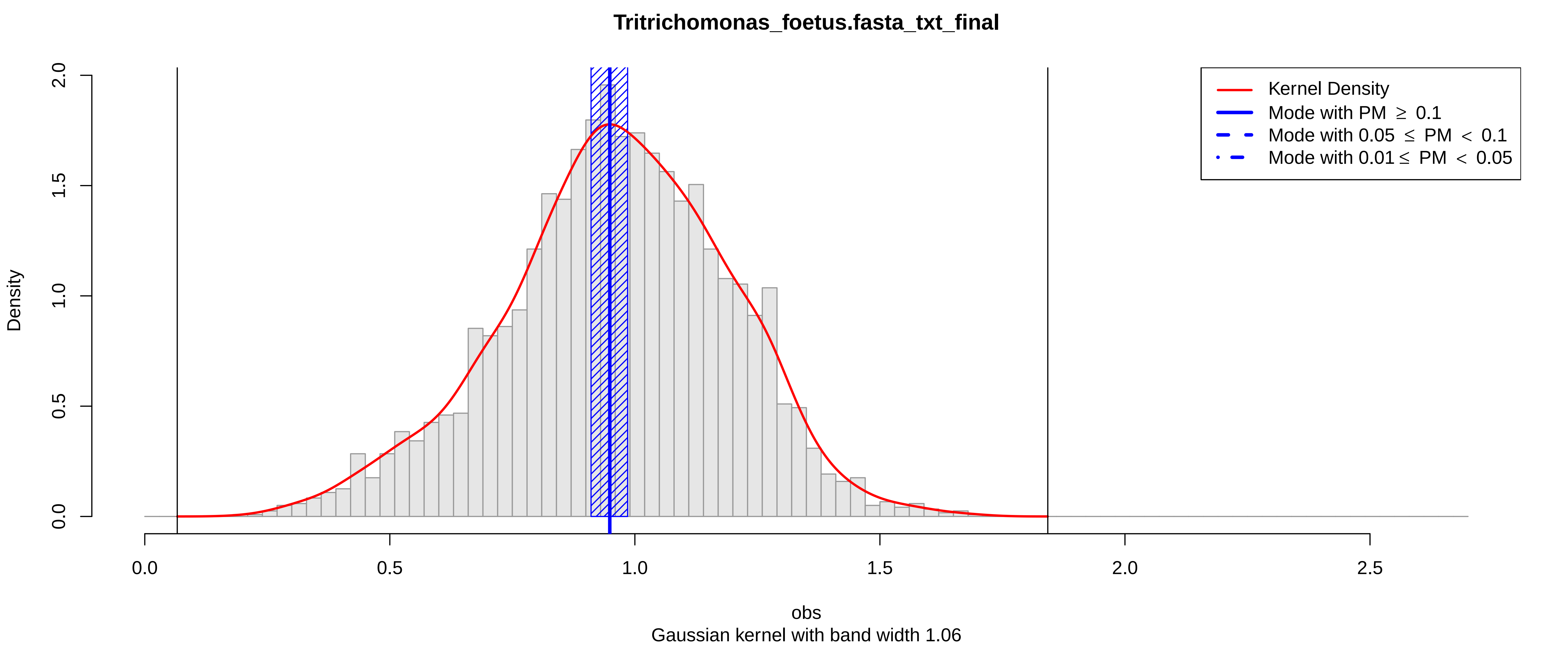

Tritrichomonas_foetus.fasta_txt_final
Density
obs
Gaussian kernel with band width 1.06
Kernel Density
Mode with PM ≥ 0.1
Mode with 0.05 ≤ PM < 0.1
Mode with 0.01 ≤ PM < 0.05
0.0
0.5
1.0
1.5
2.0
2.5

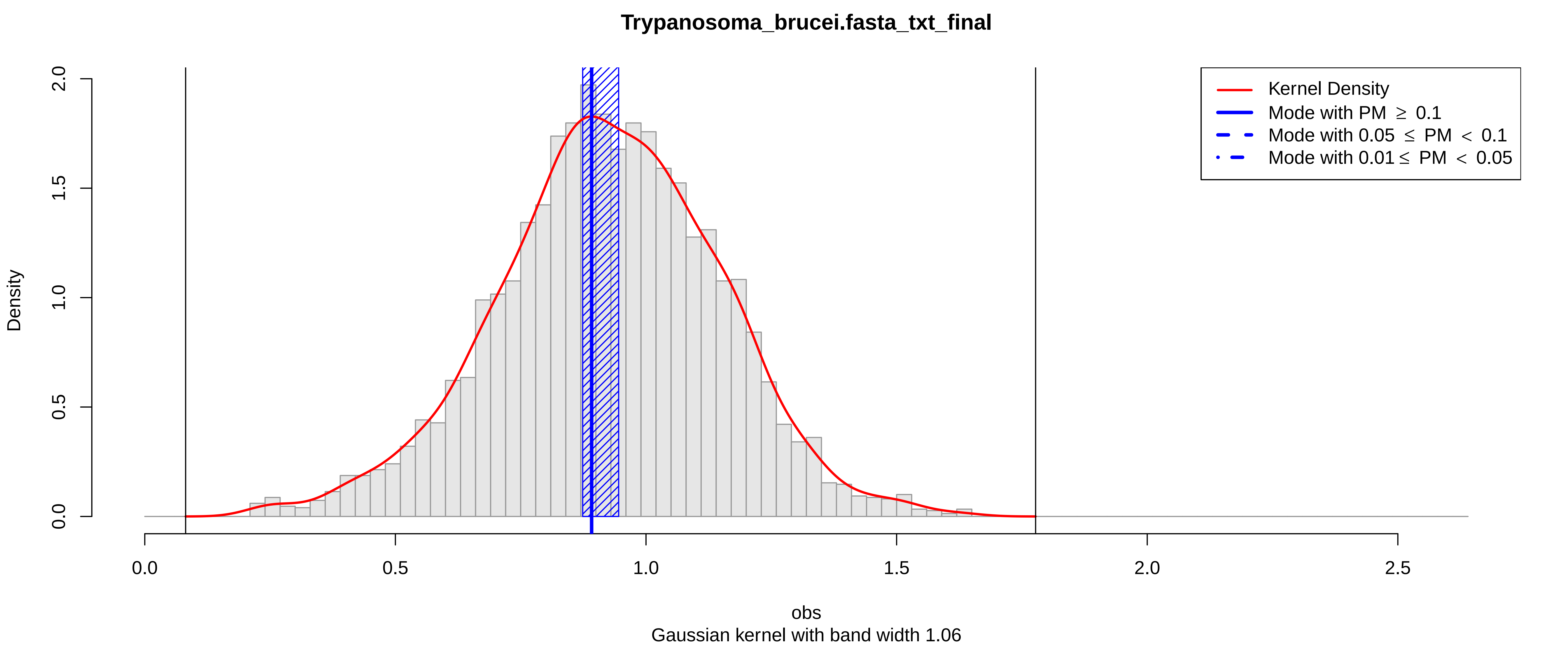

Trypanosoma_brucei.fasta_txt_final
Density
0.0
0.5
1.0
1.5
2.0
2.5
obs
Gaussian kernel with band width 1.06
Kernel Density
Mode with PM ≥ 0.1
Mode with 0.05 ≤ PM < 0.1
Mode with 0.01 ≤ PM < 0.05

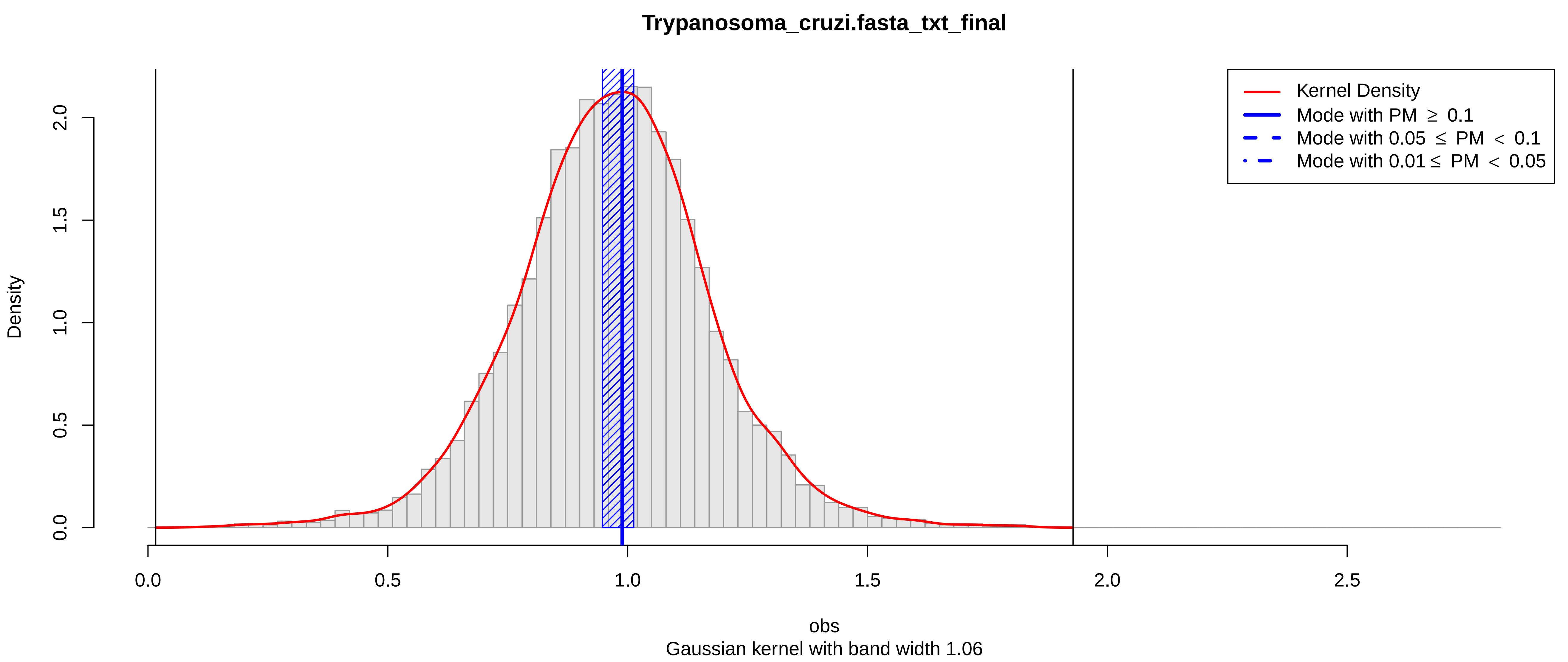

Trypanosoma_cruzi.fasta_txt_final
Kernel Density
Mode with PM ≥ 0.1
Mode with 0.05 ≤ PM < 0.1
Mode with 0.01 ≤ PM < 0.05
Density
0.0
0.5
1.0
1.5
2.0
2.5
obs
Gaussian kernel with band width 1.06

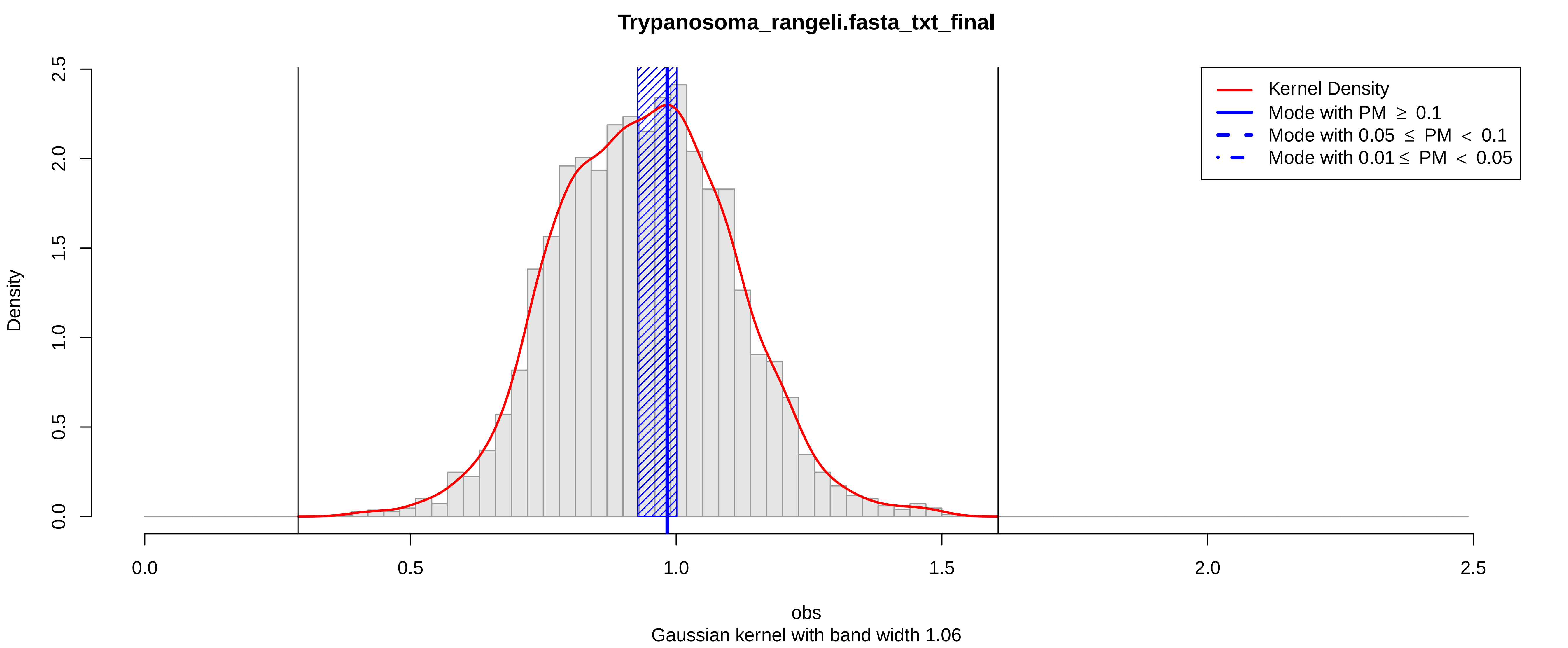

Trypanosoma_rangeli.fasta_txt_final
Kernel Density
Mode with PM ≥ 0.1
Mode with 0.05 ≤ PM < 0.1
Mode with 0.01 ≤ PM < 0.05
Density
0.0
0.5
1.0
1.5
2.0
2.5
obs
Gaussian kernel with band width 1.06

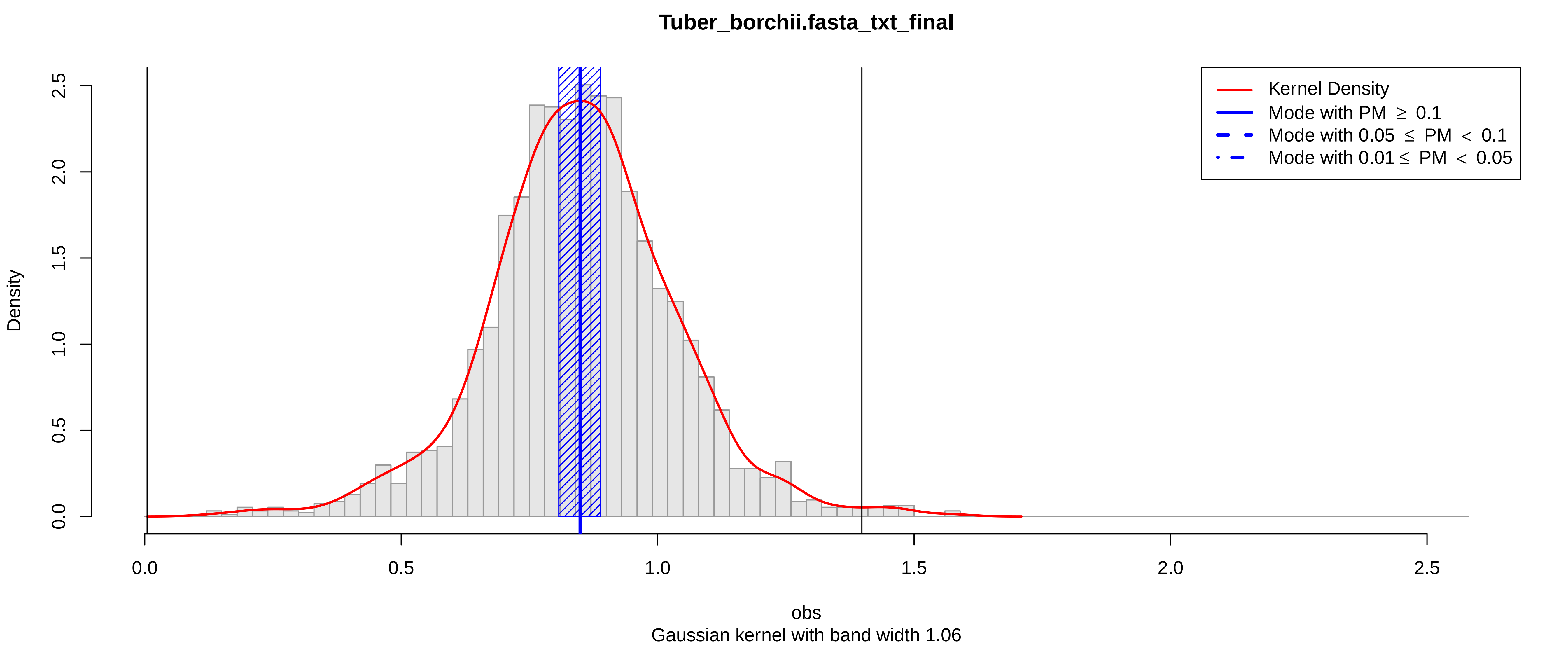
Tuber_borchii.fasta_txt_final
Density
obs
Gaussian kernel with band width 1.06
Kernel Density
Mode with PM ≥ 0.1
Mode with 0.05 ≤ PM < 0.1
Mode with 0.01 ≤ PM < 0.05
0.0
0.5
1.0
1.5
2.0
2.5

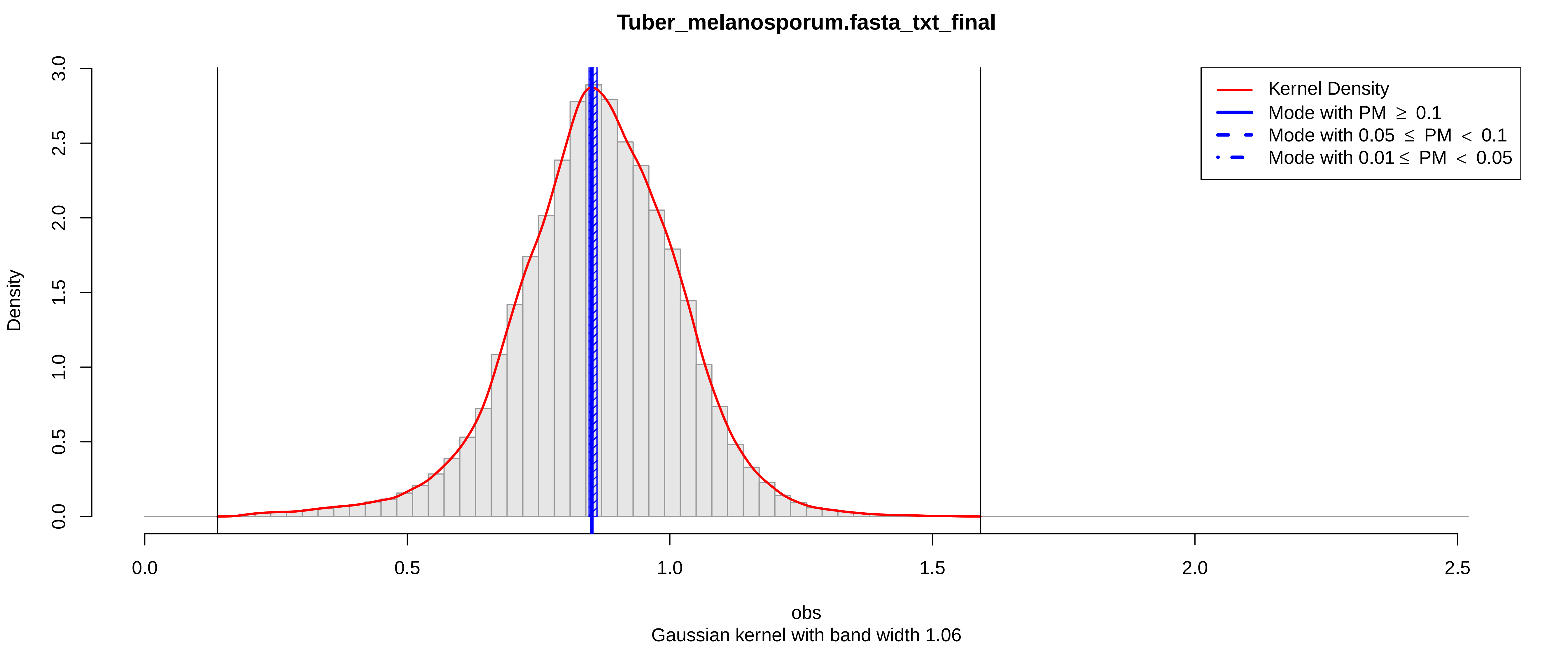

Tuber_melanosporum.fasta_txt_final
Density
0.0
0.5
1.0
1.5
2.0
2.5
3.0
Kernel Density
Mode with PM ≥ 0.1
Mode with 0.05 ≤ PM < 0.1
Mode with 0.01 ≤ PM < 0.05
0.0
0.5
1.0
1.5
2.0
2.5
obs
Gaussian kernel with band width 1.06

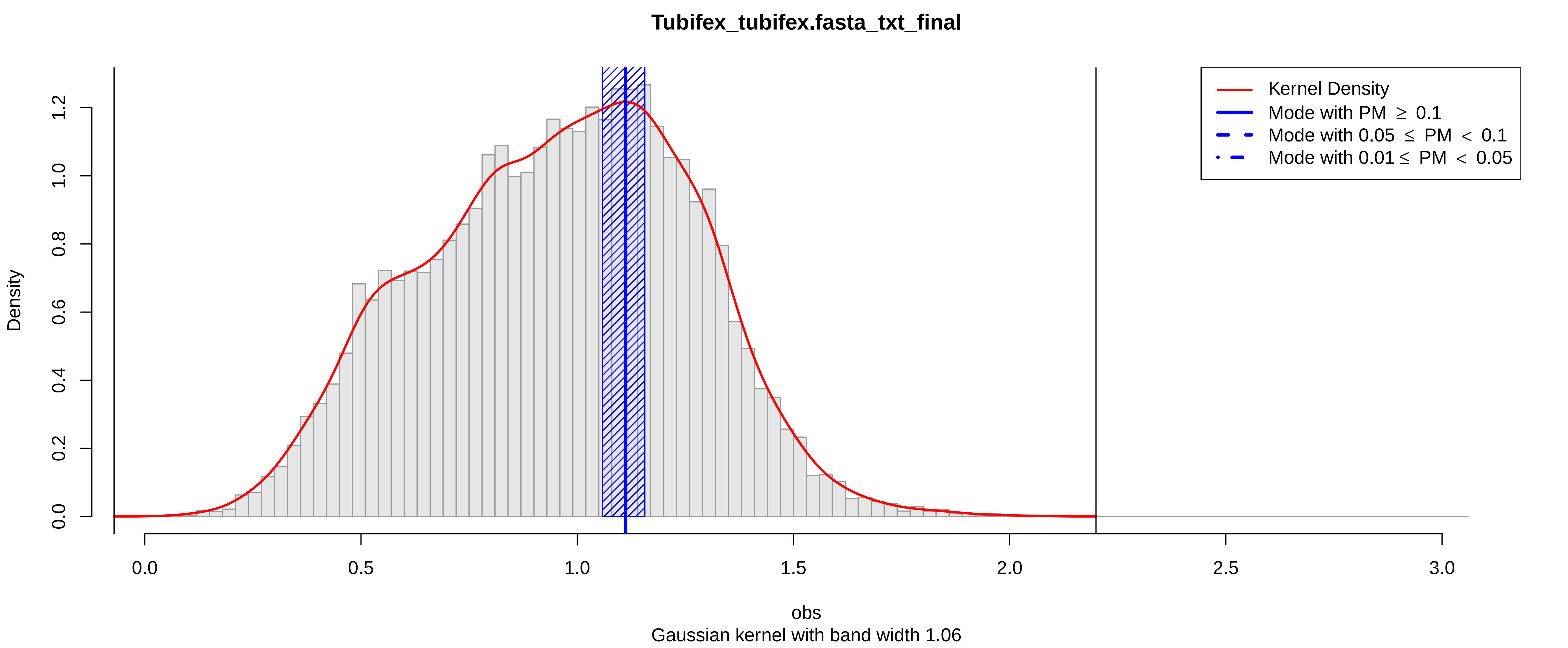

Tubifex_tubifex.fasta_txt_final
Kernel Density
Mode with PM ≥ 0.1
Mode with 0.05 ≤ PM < 0.1
Mode with 0.01 ≤ PM < 0.05
Density
0.0
0.2
0.4
0.6
0.8
1.0
1.2
0.0
0.5
1.0
1.5
2.0
2.5
3.0
obs
Gaussian kernel with band width 1.06

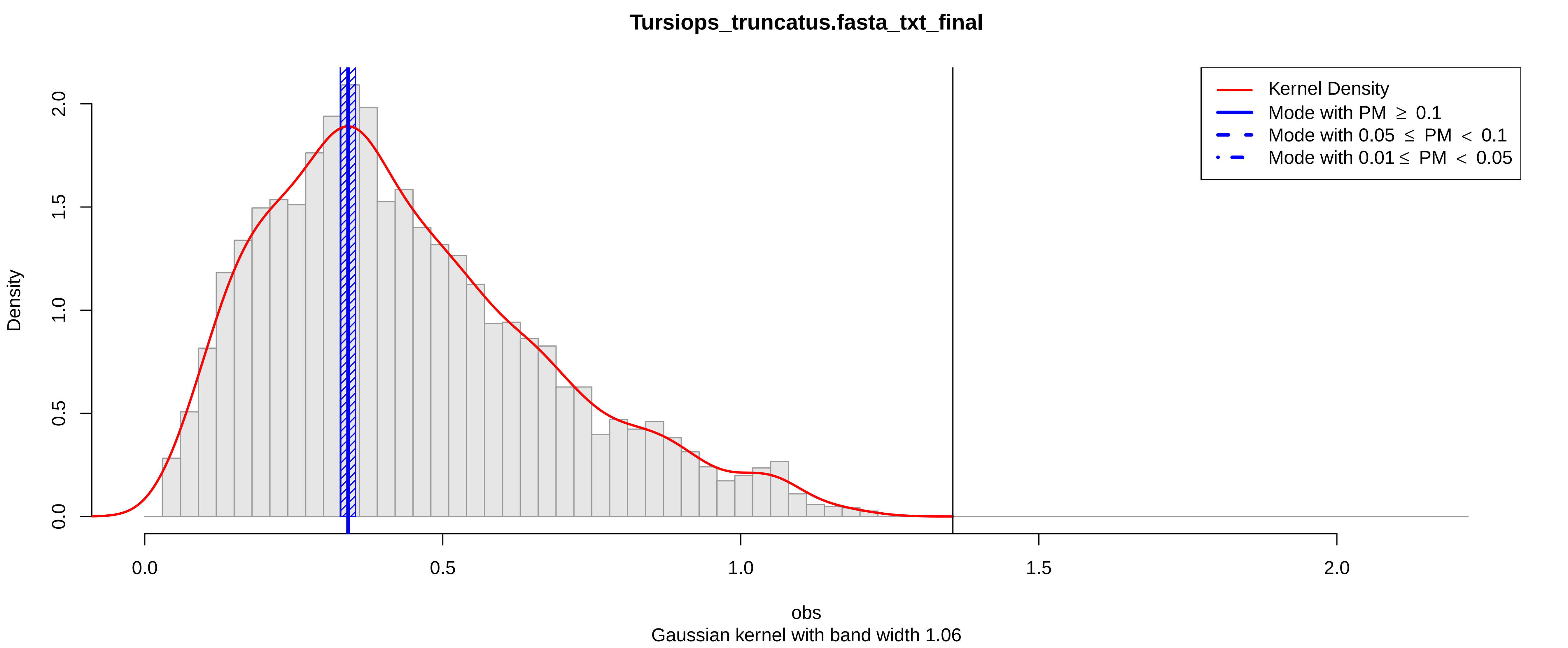

Tursiops_truncatus.fasta_txt_final
Kernel Density
Mode with PM ≥ 0.1
Mode with 0.05 ≤ PM < 0.1
Mode with 0.01 ≤ PM < 0.05
Density
0.0
0.5
1.0
1.5
2.0
obs
Gaussian kernel with band width 1.06

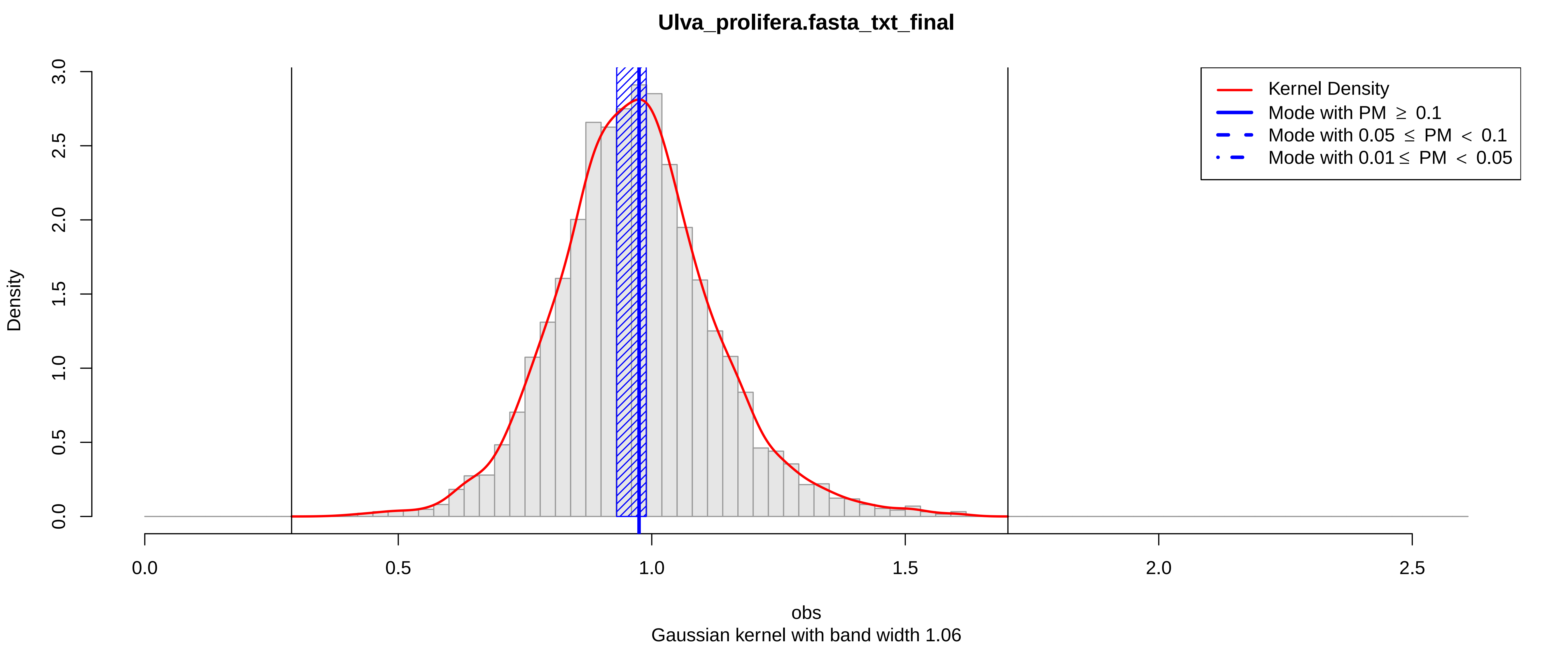

Ulva_prolifera.fasta_txt_final
Density
0.0
0.5
1.0
1.5
2.0
2.5
3.0
Kernel Density
Mode with PM ≥ 0.1
Mode with 0.05 ≤ PM < 0.1
Mode with 0.01 ≤ PM < 0.05
0.0
0.5
1.0
1.5
2.0
2.5
obs
Gaussian kernel with band width 1.06

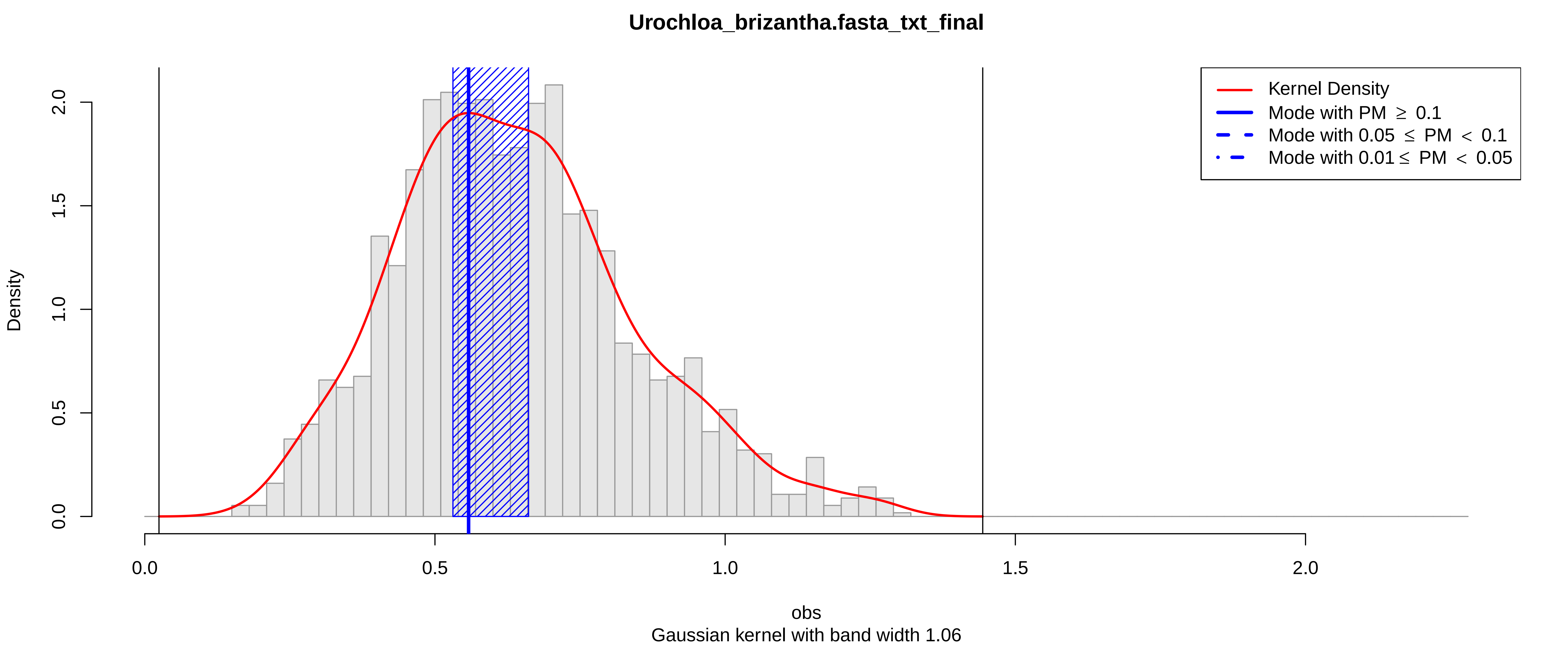
Urochloa_brizantha.fasta_txt_final
Kernel Density
Mode with PM ≥ 0.1
Mode with 0.05 ≤ PM < 0.1
Mode with 0.01 ≤ PM < 0.05
Density
0.0
0.5
1.0
1.5
2.0
obs
Gaussian kernel with band width 1.06

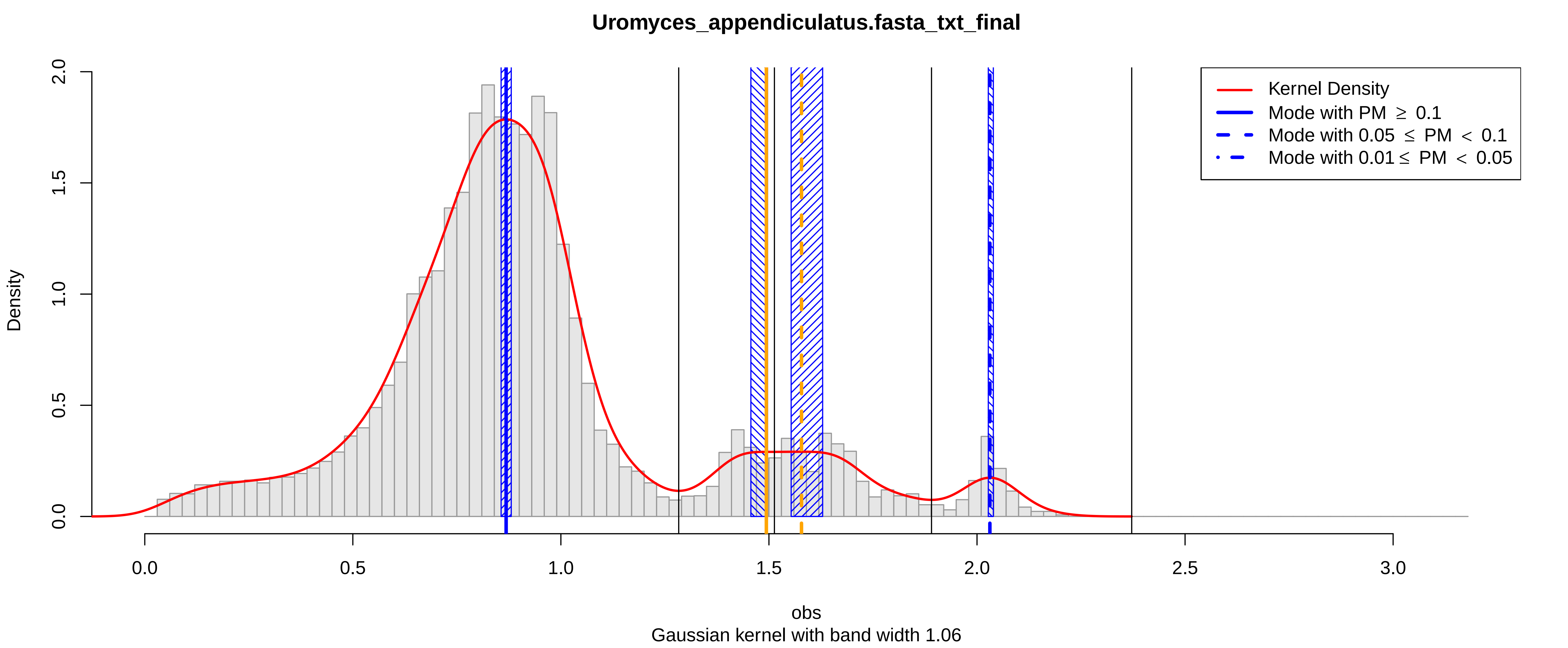
Uromyces_appendiculatus.fasta_txt_final
Density
obs
Gaussian kernel with band width 1.06
Kernel Density
Mode with PM ≥ 0.1
Mode with 0.05 ≤ PM < 0.1
Mode with 0.01 ≤ PM < 0.05
0.0
0.5
1.0
1.5
2.0
2.5
3.0

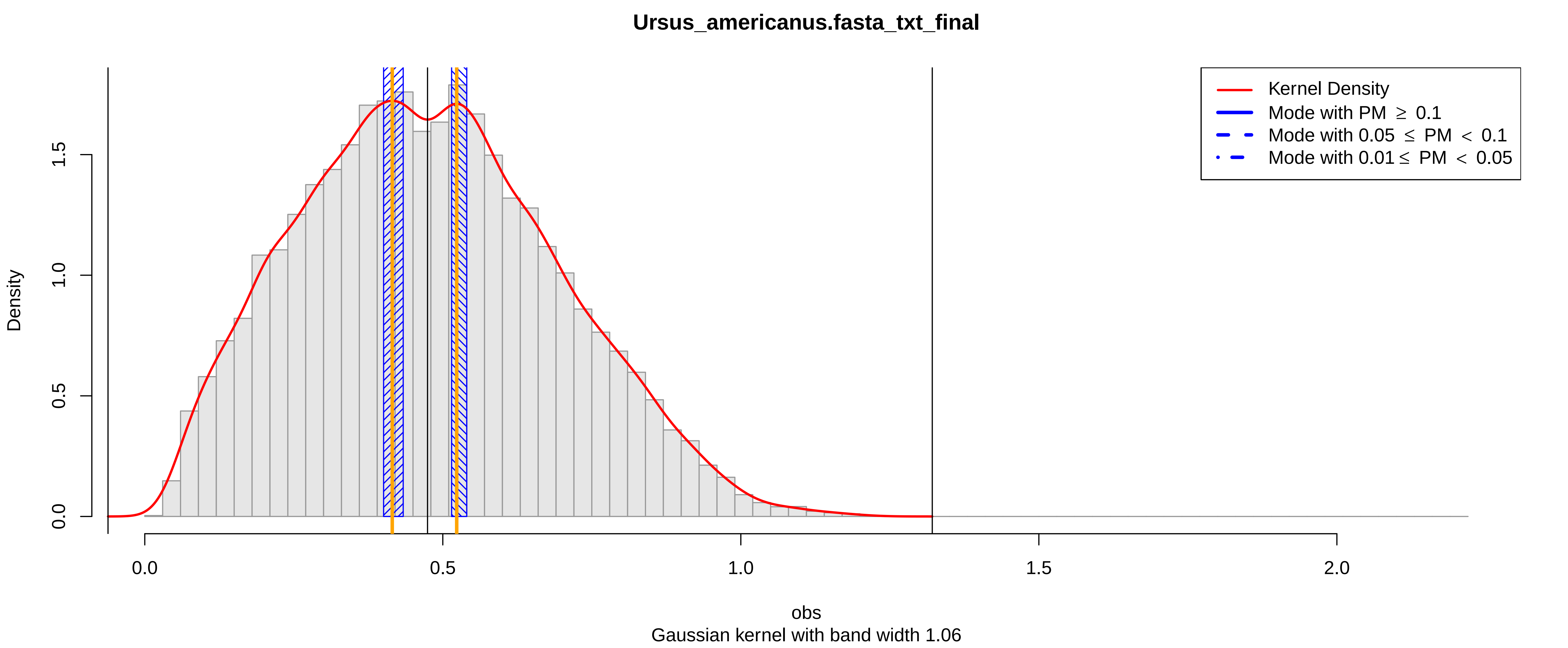

Ursus_americanus.fasta_txt_final
Kernel Density
Mode with PM ≥ 0.1
Mode with 0.05 ≤ PM < 0.1
Mode with 0.01 ≤ PM < 0.05
Density
0.0
0.5
1.0
1.5
0.0
0.5
1.0
1.5
2.0
obs
Gaussian kernel with band width 1.06

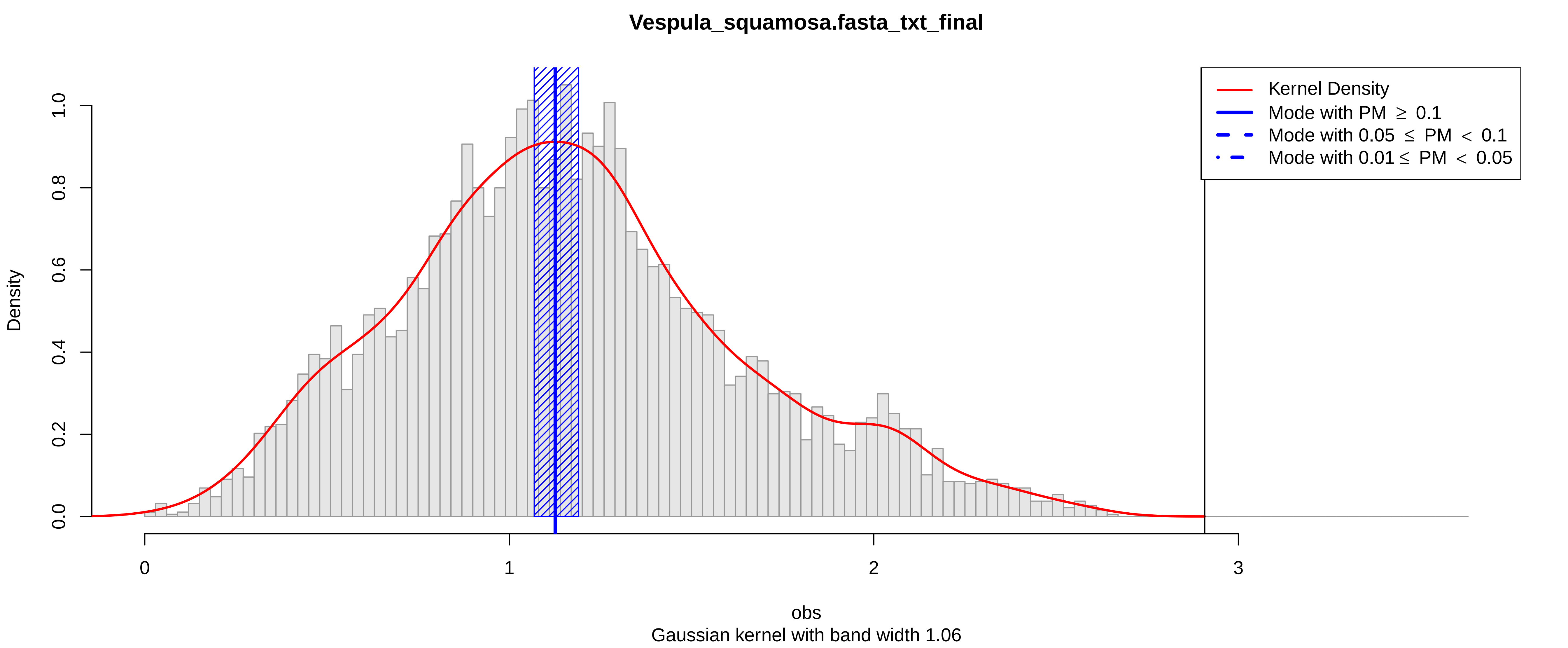

Vespula_squamosa.fasta_txt_final
Kernel Density
Mode with PM ≥ 0.1
Mode with 0.05 ≤ PM < 0.1
Mode with 0.01 ≤ PM < 0.05
Density
0.0
0.2
0.4
0.6
0.8
1.0
0
1
2
3
obs
Gaussian kernel with band width 1.06

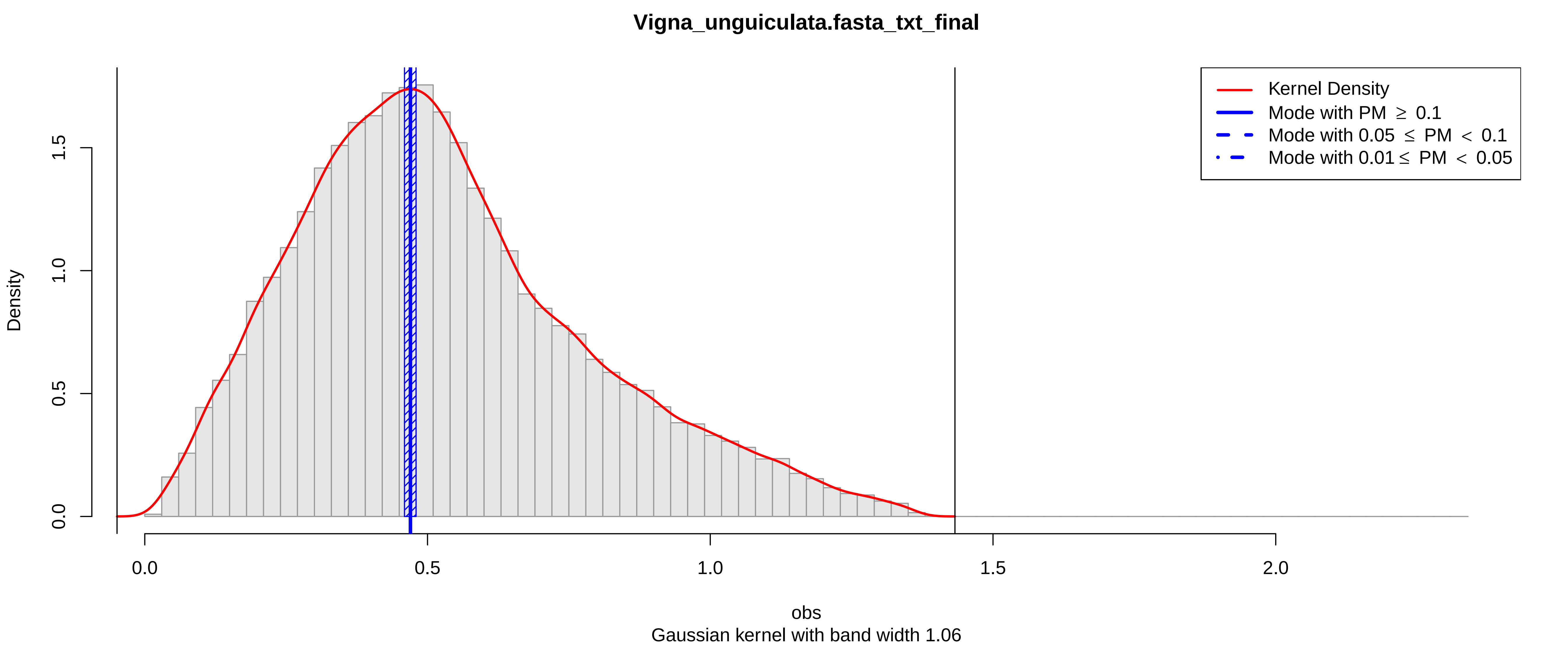

Vigna_unguiculata.fasta_txt_final
Kernel Density
Mode with PM ≥ 0.1
Mode with 0.05 ≤ PM < 0.1
Mode with 0.01 ≤ PM < 0.05
Density
0.0
0.5
1.0
1.5
0.0
0.5
1.0
1.5
2.0
obs
Gaussian kernel with band width 1.06

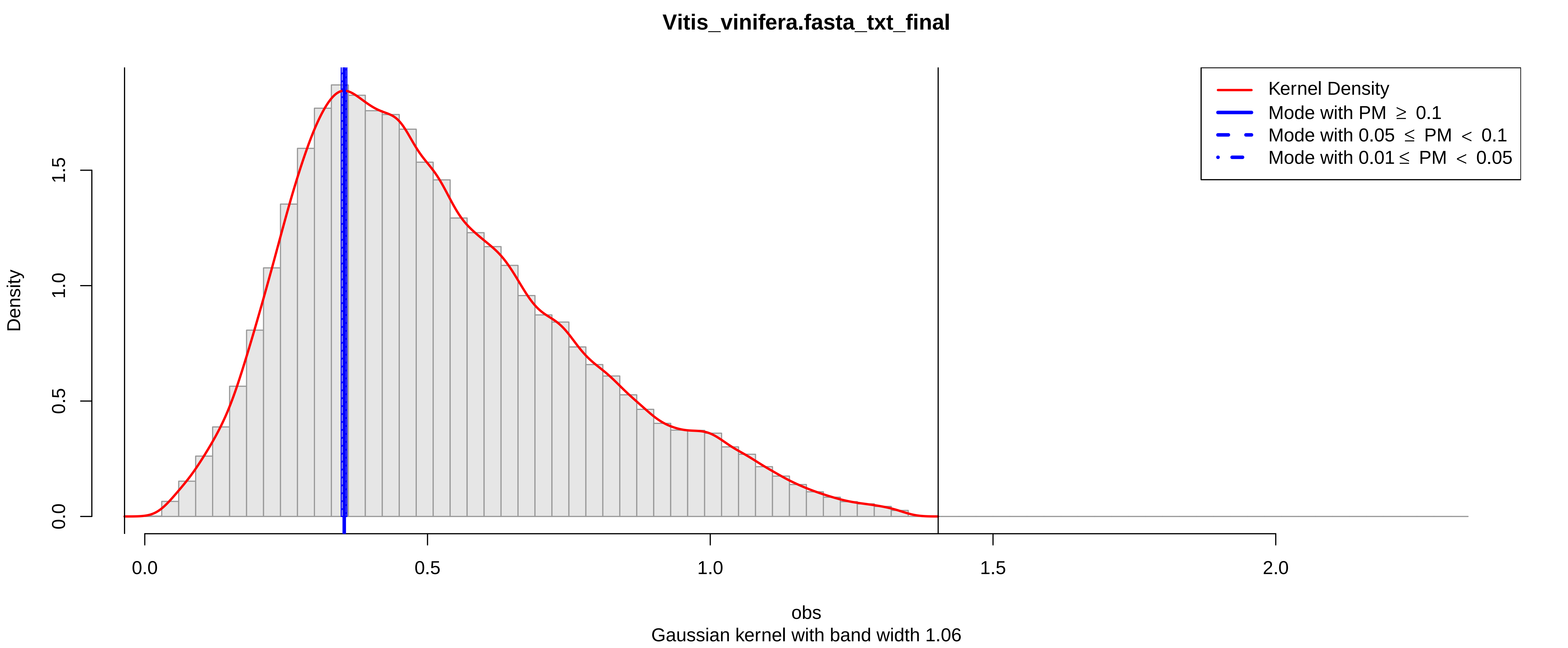

Vitis_vinifera.fasta_txt_final
Kernel Density
Mode with PM ≥ 0.1
Mode with 0.05 ≤ PM < 0.1
Mode with 0.01 ≤ PM < 0.05
Density
0.0
0.5
1.0
1.5
0.0
0.5
1.0
1.5
2.0
obs
Gaussian kernel with band width 1.06

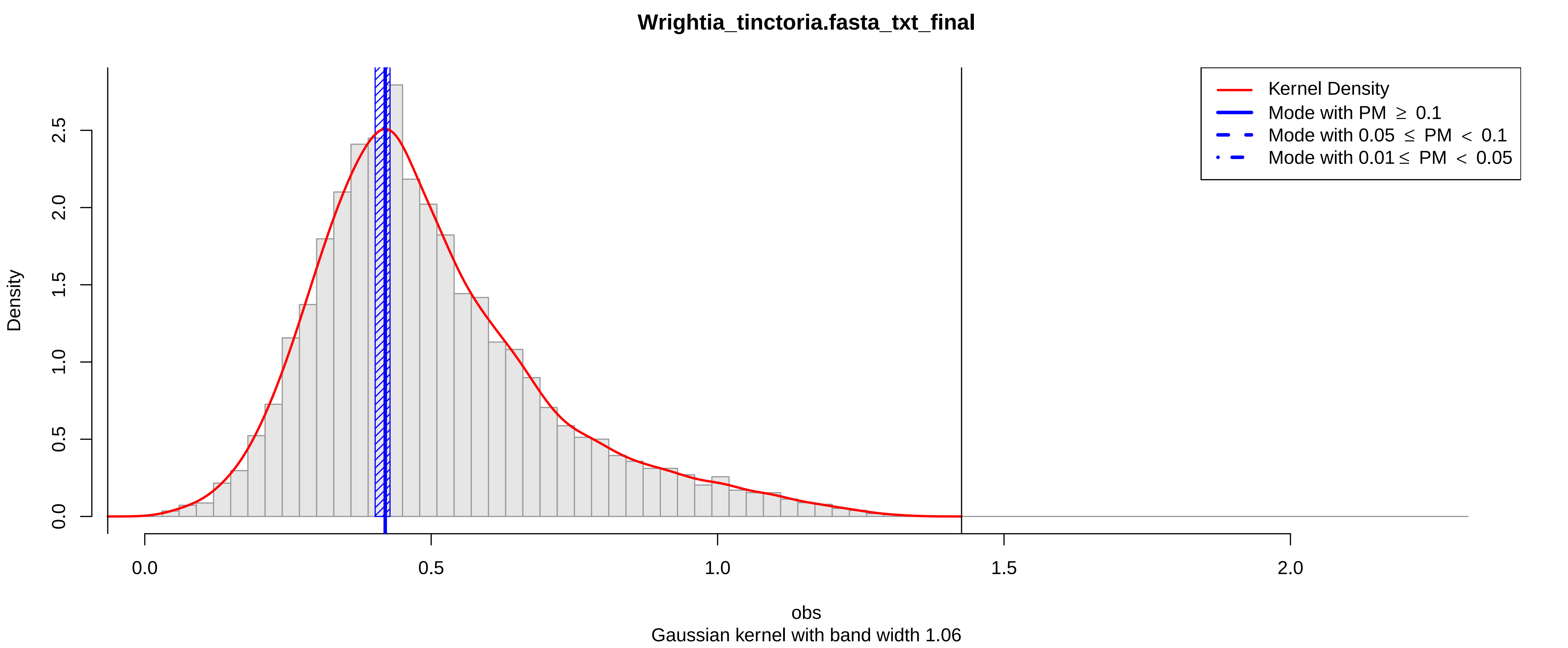

Wrightia_tinctoria.fasta_txt_final
Kernel Density
Mode with PM ≥ 0.1
Mode with 0.05 ≤ PM < 0.1
Mode with 0.01 ≤ PM < 0.05
Density
0.0
0.5
1.0
1.5
2.0
2.5
0.0
0.5
1.0
1.5
2.0
obs
Gaussian kernel with band width 1.06

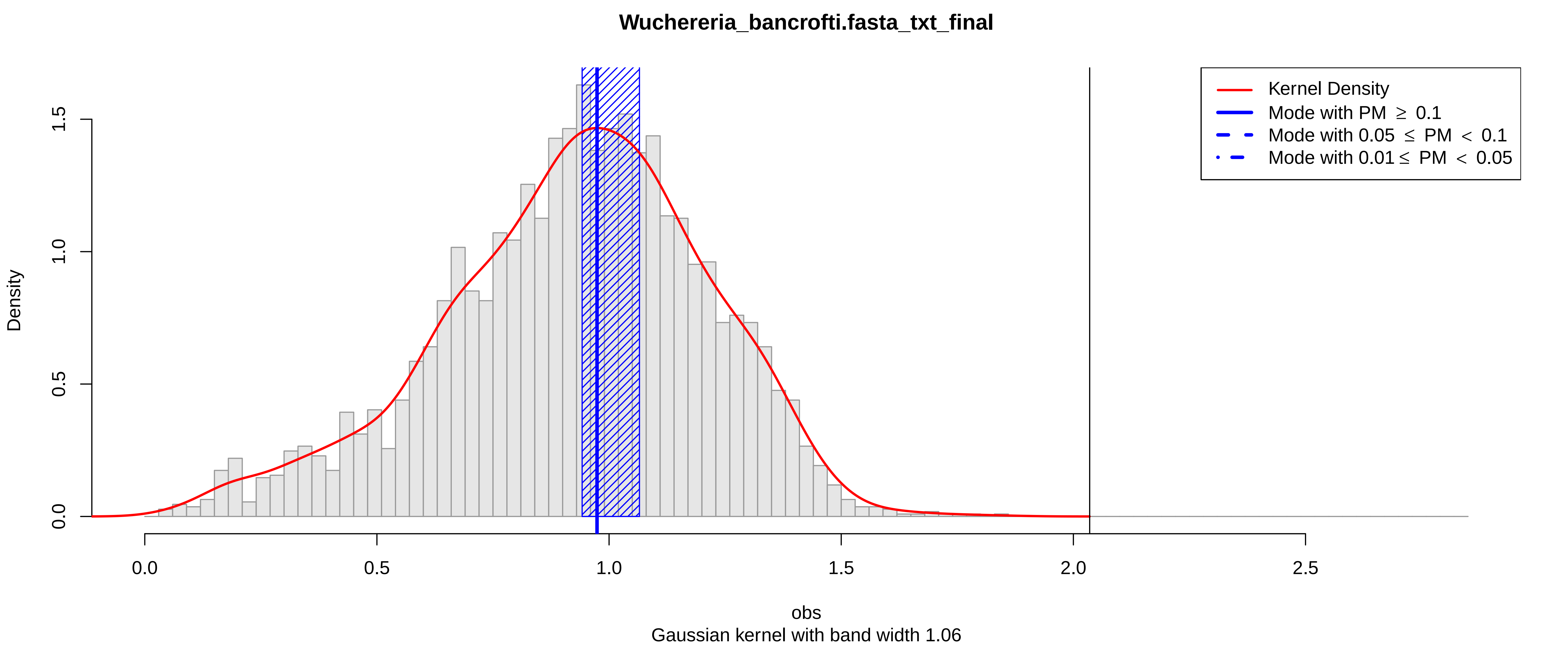

Wuchereria_bancrofti.fasta_txt_final
Kernel Density
Mode with PM ≥ 0.1
Mode with 0.05 ≤ PM < 0.1
Mode with 0.01 ≤ PM < 0.05
Density
0.0
0.5
1.0
1.5
0.0
0.5
1.0
1.5
2.0
2.5
obs
Gaussian kernel with band width 1.06

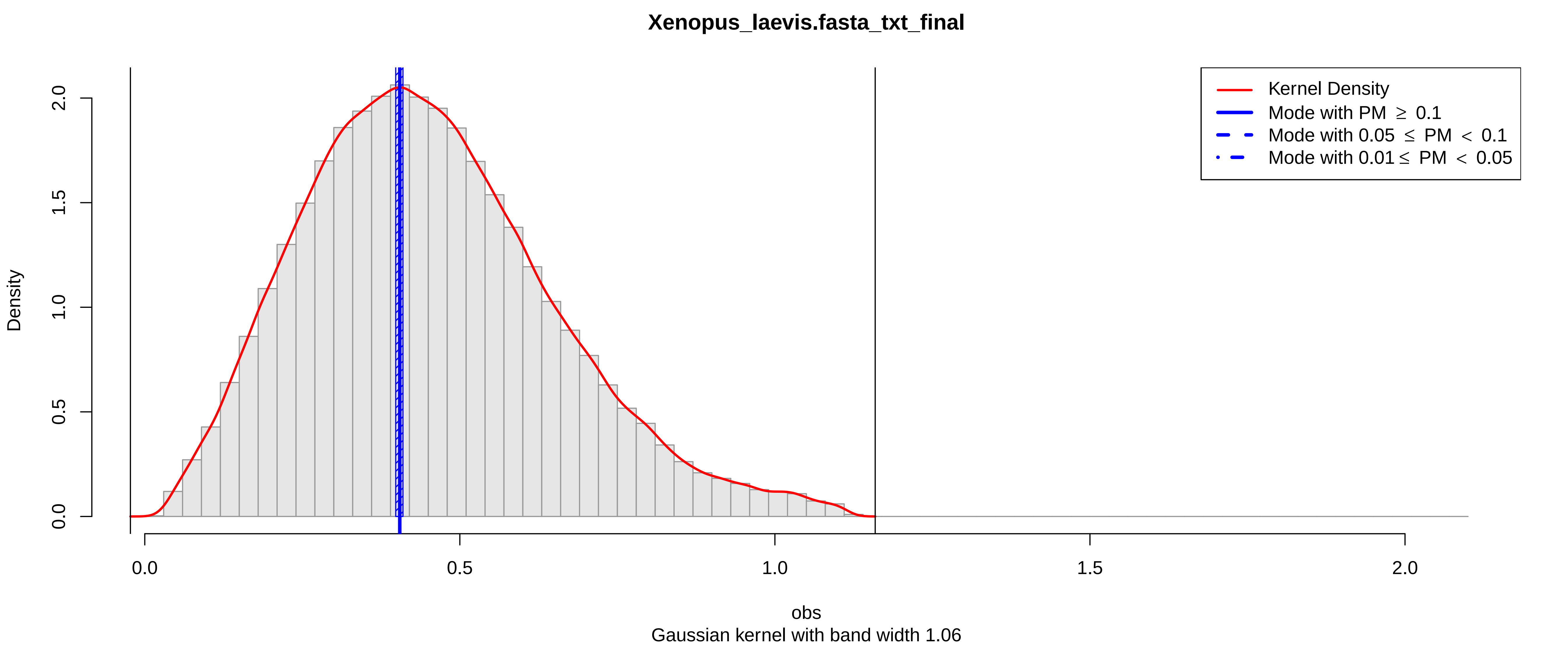

Xenopus_laevis.fasta_txt_final
Kernel Density
Mode with PM ≥ 0.1
Mode with 0.05 ≤ PM < 0.1
Mode with 0.01 ≤ PM < 0.05
Density
0.0
0.5
1.0
1.5
2.0
0.0
0.5
1.0
1.5
2.0
obs
Gaussian kernel with band width 1.06

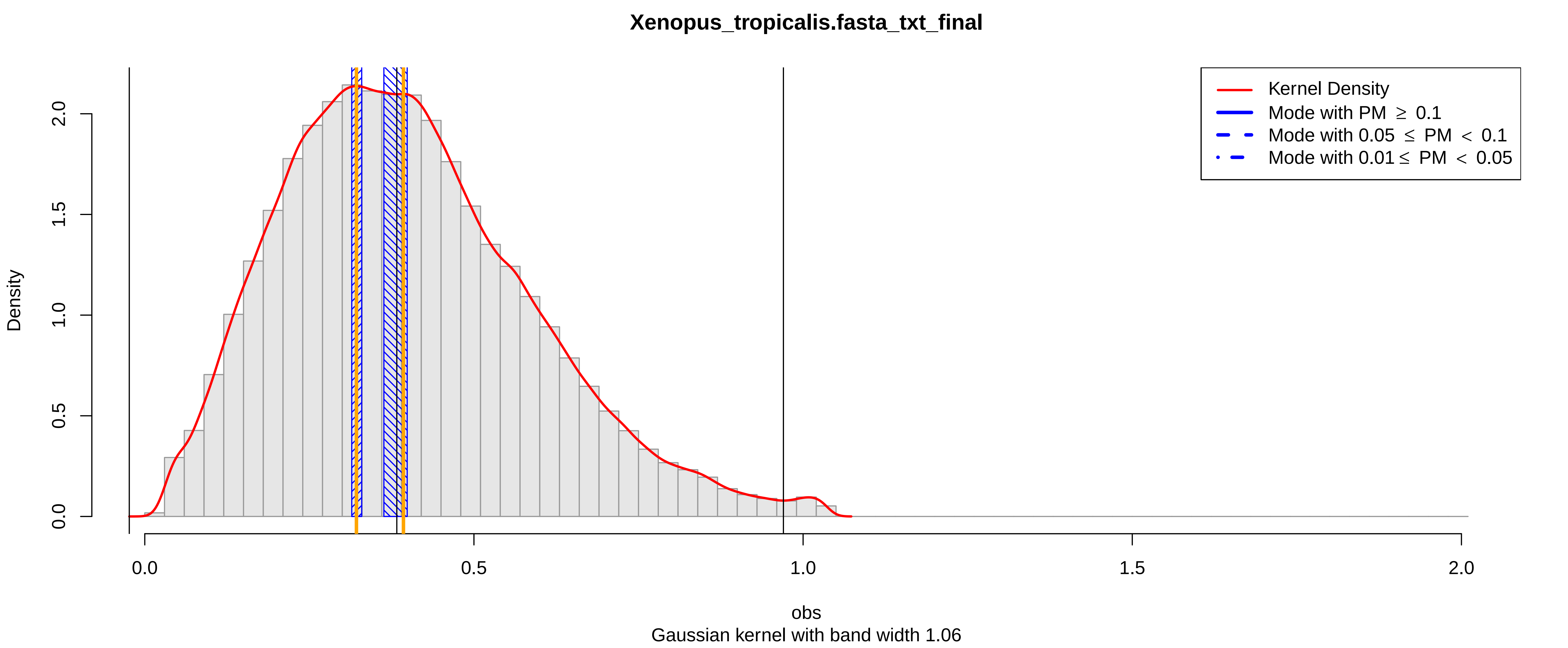

Xenopus_tropicalis.fasta_txt_final
Kernel Density
Mode with PM ≥ 0.1
Mode with 0.05 ≤ PM < 0.1
Mode with 0.01 ≤ PM < 0.05
Density
0.0
0.5
1.0
1.5
2.0
obs
Gaussian kernel with band width 1.06

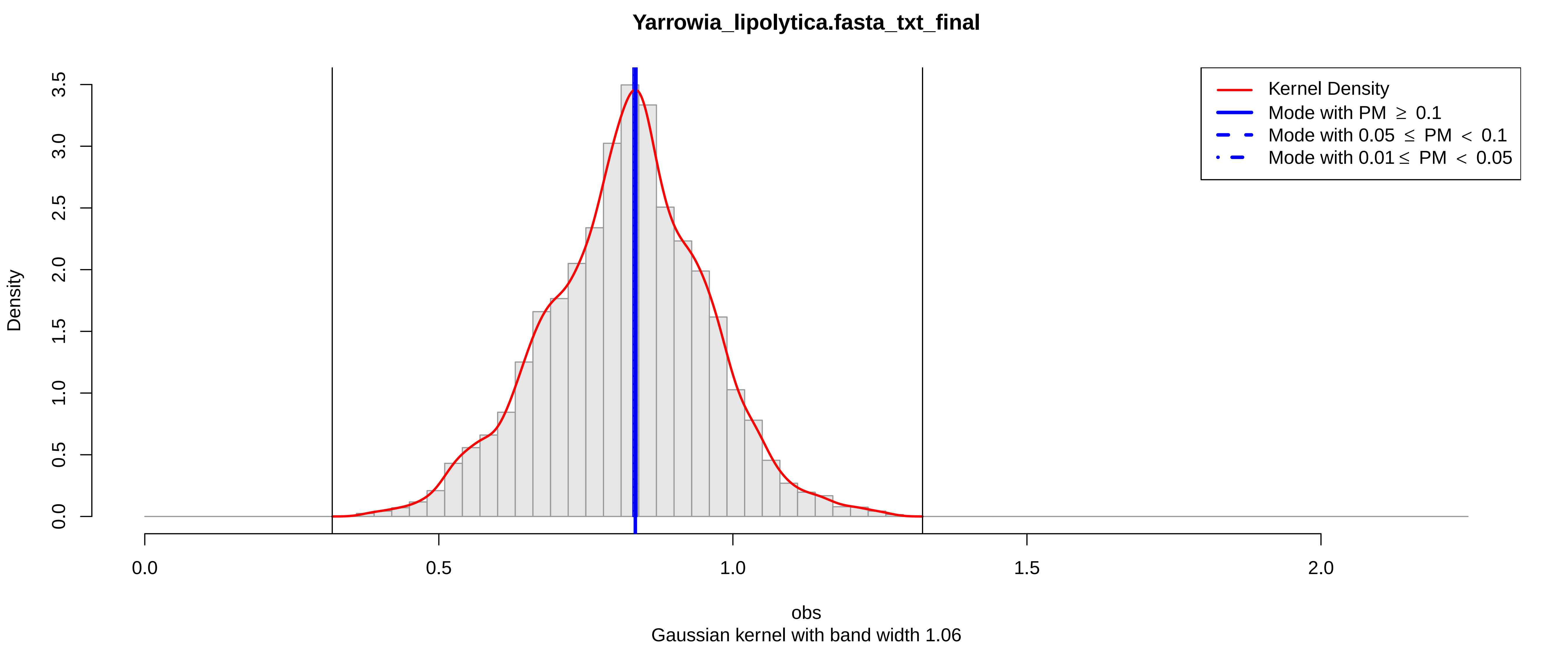
Yarrowia_lipolytica.fasta_txt_final
Kernel Density
Mode with PM ≥ 0.1
Mode with 0.05 ≤ PM < 0.1
Mode with 0.01 ≤ PM < 0.05
Density
0.0
0.5
1.0
1.5
2.0
2.5
3.0
3.5
0.0
0.5
1.0
1.5
2.0
obs
Gaussian kernel with band width 1.06

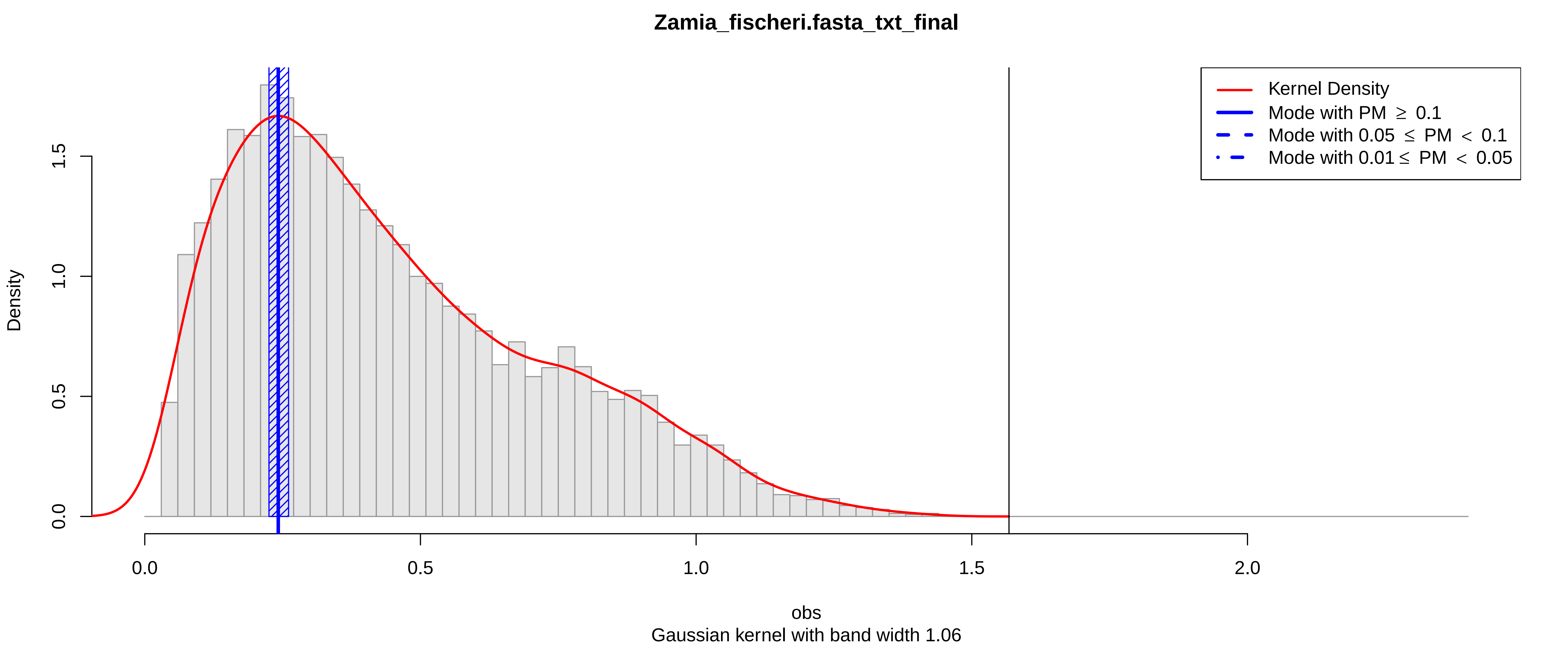
Zamia_fischeri.fasta_txt_final
Kernel Density
Mode with PM ≥ 0.1
Mode with 0.05 ≤ PM < 0.1
Mode with 0.01 ≤ PM < 0.05
Density
0.0
0.5
1.0
1.5
0.0
0.5
1.0
1.5
2.0
obs
Gaussian kernel with band width 1.06

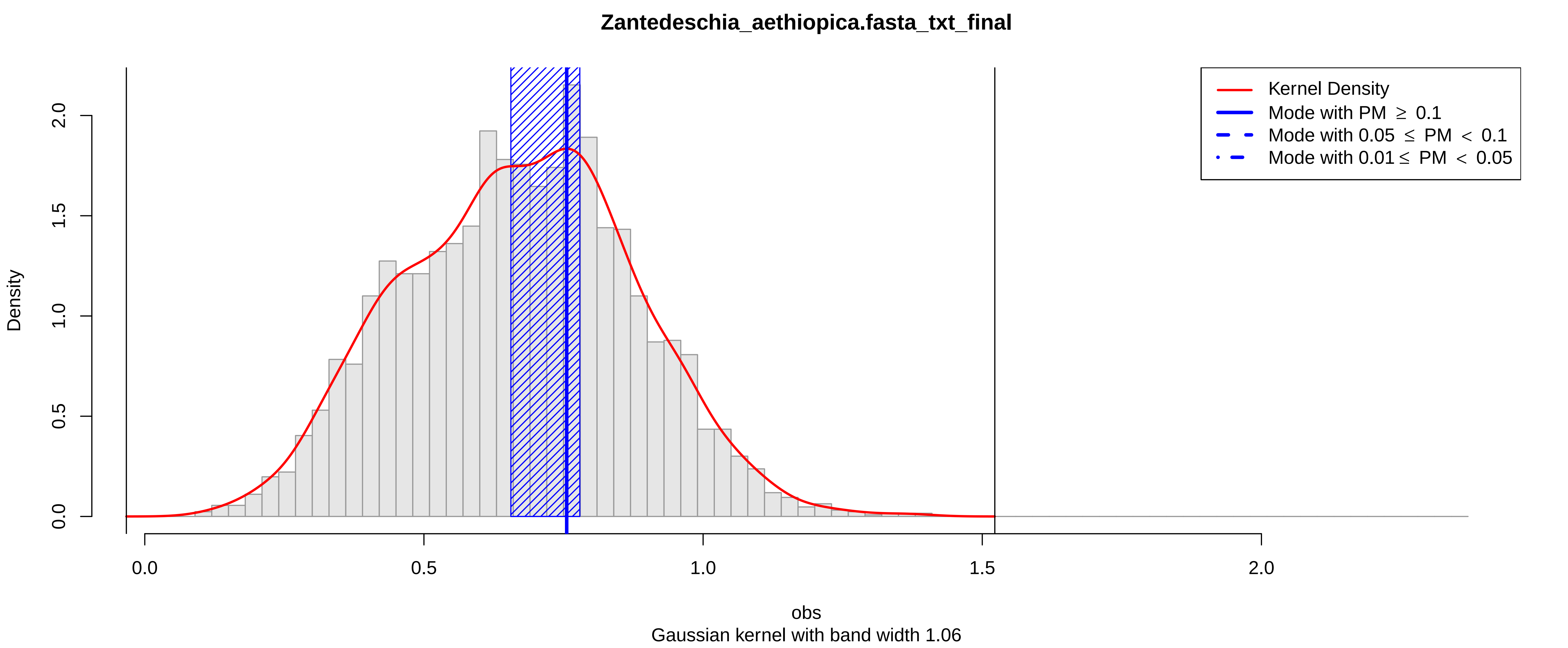

Zantedeschia_aethiopica.fasta_txt_final
Kernel Density
Mode with PM ≥ 0.1
Mode with 0.05 ≤ PM < 0.1
Mode with 0.01 ≤ PM < 0.05
Density
0.0
0.5
1.0
1.5
2.0
obs
Gaussian kernel with band width 1.06

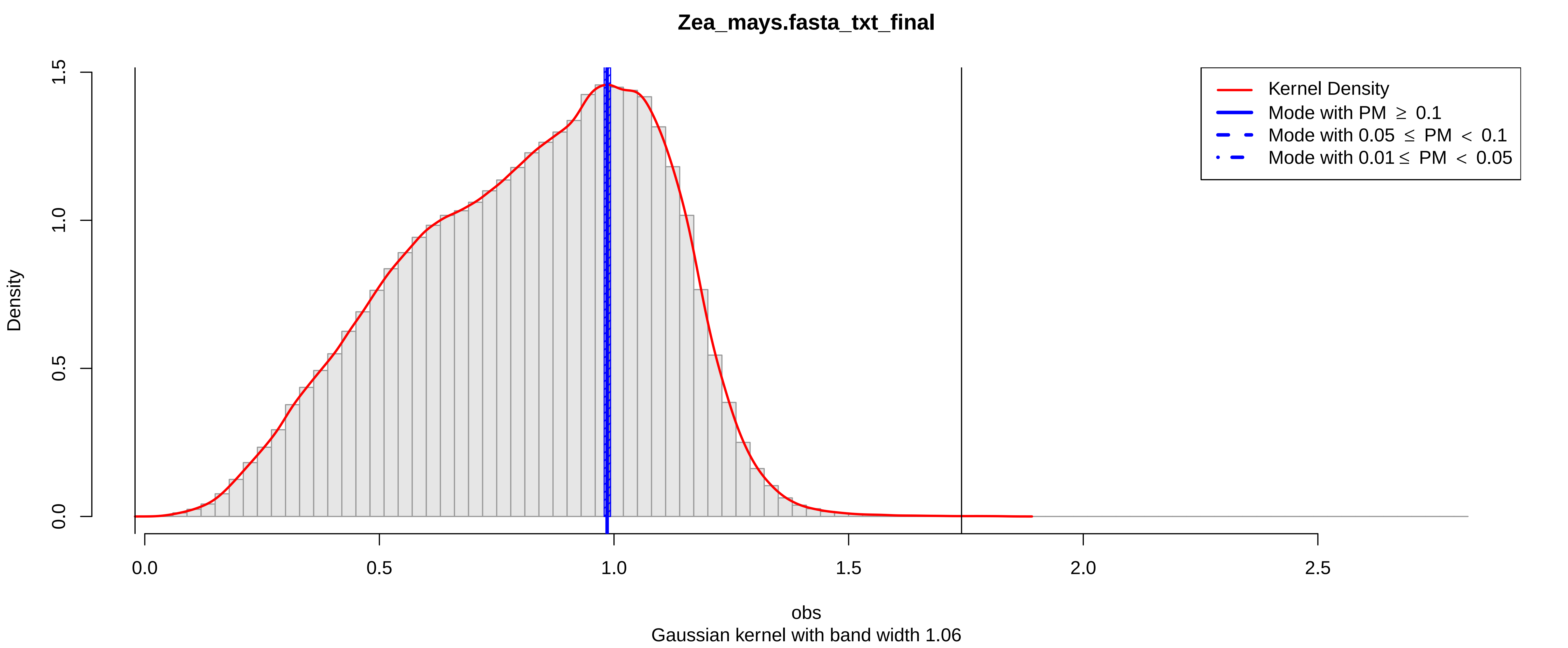
Zea_mays.fasta_txt_final
Density
obs
Gaussian kernel with band width 1.06
0.0
0.5
1.0
1.5
2.0
2.5
Kernel Density
Mode with PM ≥ 0.1
Mode with 0.05 ≤ PM < 0.1
Mode with 0.01 ≤ PM < 0.05

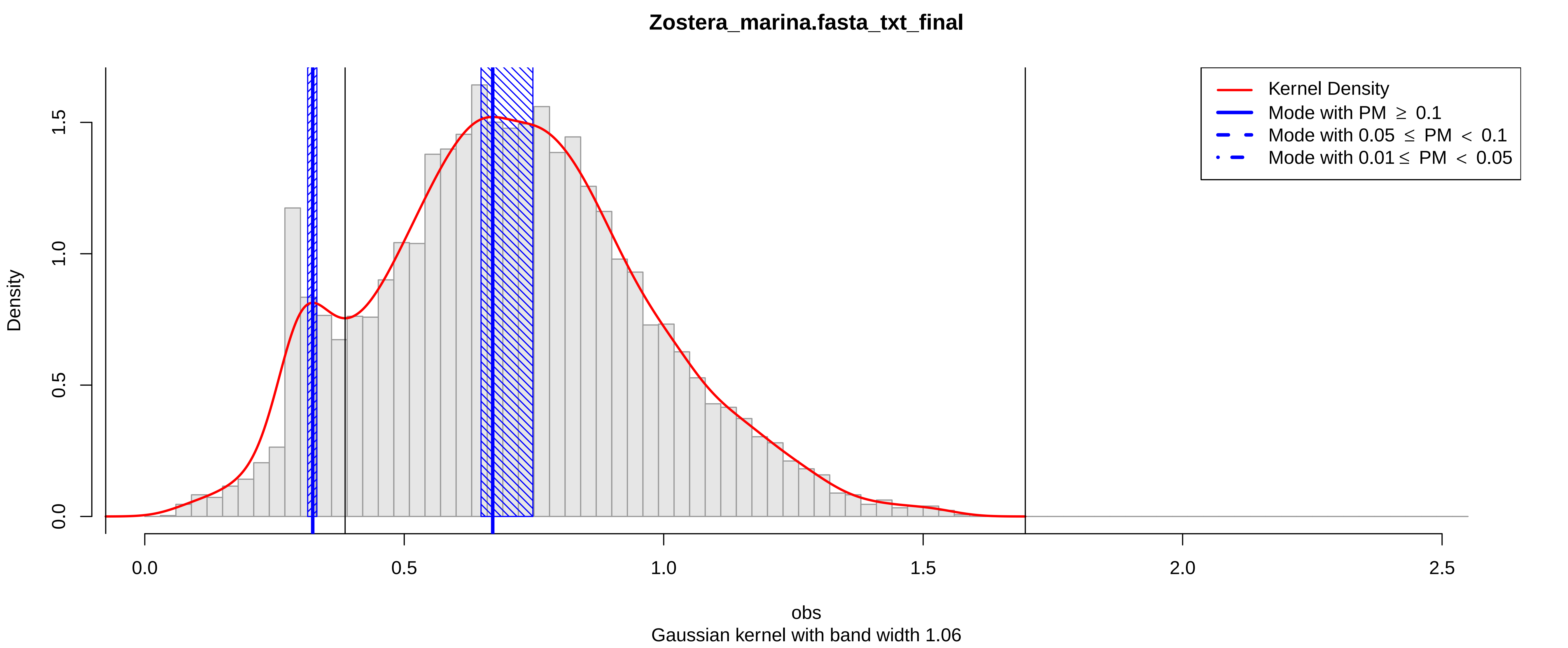
Zostera_marina.fasta_txt_final
Kernel Density
Mode with PM ≥ 0.1
Mode with 0.05 ≤ PM < 0.1
Mode with 0.01 ≤ PM < 0.05
Density
0.0
0.5
1.0
1.5
0.0
0.5
1.0
1.5
2.0
2.5
obs
Gaussian kernel with band width 1.06

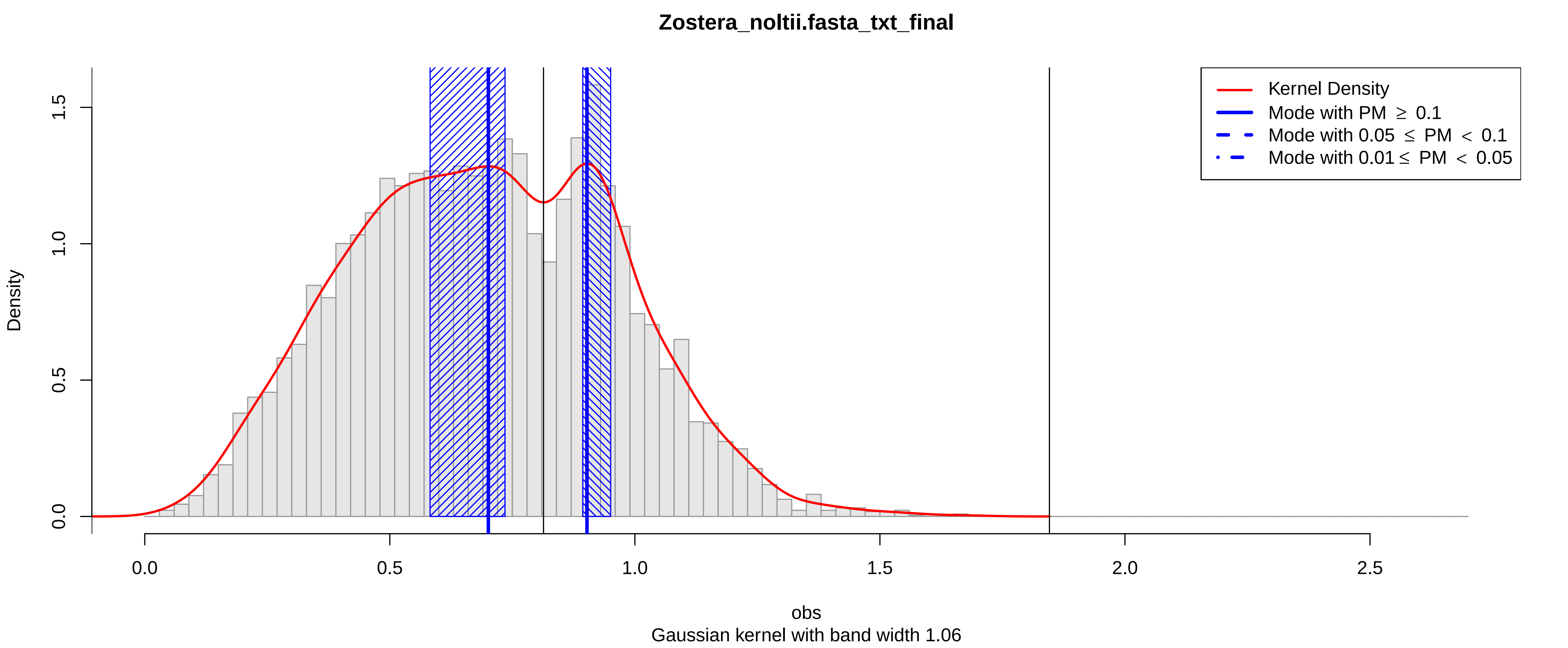

Zostera_noltii.fasta_txt_final
Kernel Density
Mode with PM ≥ 0.1
Mode with 0.05 ≤ PM < 0.1
Mode with 0.01 ≤ PM < 0.05
Density
0.0
0.5
1.0
1.5
0.0
0.5
1.0
1.5
2.0
2.5
obs
Gaussian kernel with band width 1.06
